# Systematic DFT Modeling van der Waals Heterostructures from a Complete Configurational Basis Applied to γ-PC/WS_2_

**DOI:** 10.1021/acs.jctc.3c00932

**Published:** 2024-03-06

**Authors:** Joran Celis, Wei Cao

**Affiliations:** Nano and Molecular Systems Research Unit, Faculty of Science, University of Oulu, FIN-90014 Oulu, Finland

## Abstract

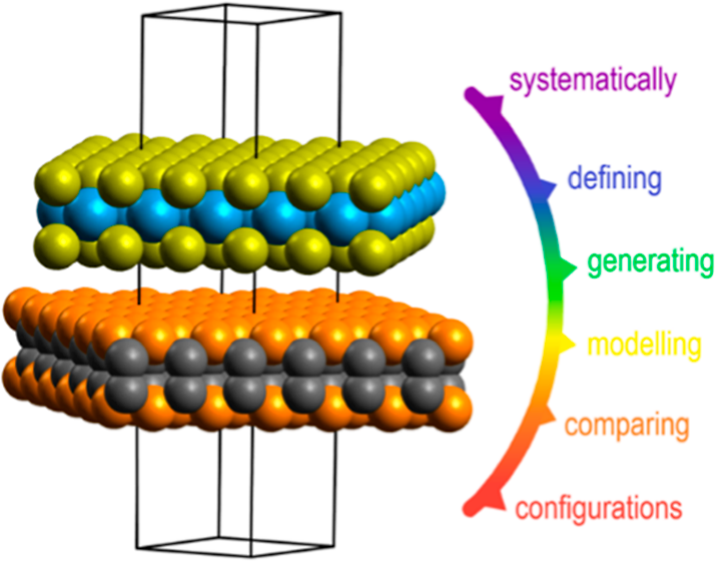

Periodic boundary conditions in density functional theory (DFT)-based modeling of bilayer van der Waals heterostructures introduce an artificial lock to a metastable configuration. Depending on the initial supercell, geometric optimization may reach local energy minima at a fixed twist-angle in a restricted strain-space. In this work, an algorithm was introduced for generating a complete scope of ways to combine two monolayer unit cells into a common supercell. In its application to γ-PC/WS_2_, 18,123 bilayer supercells were derived, for which the constituting monolayers possessed isotropic strains, anisotropic strains, or intralayer shear strains. Based on analysis, 45 isotropically strained configurations were carefully chosen for optimization by DFT. Geometric and energetic features and band structures were revealed and compared, following the variations at different strains and twist-angles. As such, this case study brought to resolution the impacts of supercell construction on DFT’s outcomes and the merits of in-depth screening of the different options. Repetitions and extensions to the demonstrated approach may be applied to characterize van der Waals heterostructures and derivatives in the future.

## 1 Introduction

Layered materials are those where sheets of atomic-scale thickness remain connected through van der Waals interactions. The most well-known example is graphite, a construct of two-dimensional (2D) graphene sheets. The isolation of individual 2D layers was (re)invented in 2004 by mechanical exfoliation of a single graphene layer.^1^ The class of van der Waals heterostructure (vdWH) materials emerged around 2010 with the fabrication of graphene on boron nitride devices.^2,3^ Ever since, intense research efforts have been made to explore the novel vdWH material space.^4,5^

The class of vdWHs is made up of layered materials that possess atomic layers of varying phases or compositions. As a representative type, bilayer (BL) vdWHs commonly possess a high surface area, remarkable optical and catalytic properties, and spin–orbit coupling.^4,5^ Moreover, BL vdWHs have been associated with great versatility. This is because the material space starts from a huge number of widely combinable and stable 2D monolayers (MLs). In each combination of two MLs, the layers mutually influence the other’s characteristics, possibly leading to unique quantum phenomena, which have been attributed to van der Waals interactions between the layers.^6^ This overall interaction has been described starting from local stacking configurations,^7,8^ and ordinarily, local stacking configurations in a BL (quasi)periodically modulate over a so-called Moiré superlattice.^9^ Then, on the other hand, the versatility of BL vdWHs is underpinned by their tunability through stacking and straining. Indeed, by varying either the relative orientation between the MLs (i.e., the twist-angle) or the strains present on the MLs, the interlayer interactions may be influenced and thus the BL material properties. As such, effects of straining and stacking have been reported for a very wide set of material properties, including optical, electronic, excitonic, and magnetic properties and intralayer atomistic diffusivity^6,10−13^ for various materials. However, it is worth mentioning that straining may affect the MLs alone, in part independent of the BL coupling.^14^

Theoretical predictions remain crucial in the material research of BL vdWHs. Although the tunability by stacking and straining may be revealed experimentally,^15−18^ even in the absence of uncontrolled distortions,^19^ only a minority of predicted stable MLs have been achieved at lab scale to date.^6,20,21^ Theoretically, density function theory (DFT) serves as one of the most important and frequently utilized tools in parallel with the experimental efforts in the field.^22,23^ However, aside from general imperfections of DFT as a description of reality and its empirical treatment of exchange and correlation of electrons in particular,^24^ additional drawbacks may be encountered while treating specifically the BL vdWHs.

A typical DFT study of crystals debuts from a simulation box, and periodic boundary conditions (PBC) are imposed over the system. This allows the representation of real infinite systems by a finite unit cell.^25^ However, when considering BL vdWHs, this also causes the following issues. Fitting two ML cells of varying size within a single simulation box requires modifications, e.g., squeezing and/or stretching of the MLs.^26,27^ A strain pair consisting of strains on the individual MLs is thereby defined. This strain pair does have a degree of variability since it may be altered with the lattice vectors ***a*** and ***b*** of the BL supercell.^28^ Still, the 2D strain-space thereby created remains non-all-inclusive. Second, the gradual rotation of one ML with respect to the other inescapably breaks the fulfillment of the PBC.^29^ Therefore, a twist-angle is also defined by setting up a BL supercell,^30^ which cannot alter during DFT-based geometric optimization. By these two root-causes, the apparent potential energy landscape becomes dependent on arbitrary supercell construction. DFT-based optimizations therefore generally lead merely to a metastable lowest energy configuration in which the strain pair and the twist-angle will be referred to as the “strain-twist-angle combination” (STAC) associated with the supercell. A true global minimum energy configuration of a BL vdWH may presumably still be retrieved but only via one of very many possible BL supercell constructions. Moreover, modeling it may not be feasible when Moiré superlattices are too large for practical DFT implementations,^31^ forcing alternative periodicity.

Although optimizations of multiple BL configurations are conventionally included in DFT-studies on BL vdWHs, the possibilities beyond the sets of probed systems generally remain overlooked. To an extent, it then remains up for guessing whether additional simulations at alternative strains, twist-angles, and/or periodicities would reveal improved or additional insights into the material. Marking an opportunity in this uncertainty, an in-depth BL supercell screening approach was demonstrated in this work, characterizing the previously unreported InSe-like phosphorus carbide^32−36^ (γ-PC) on tungsten sulfide^37,38^ (WS_2_) vdWH. About the constituting MLs, a wide 2.65 eV band gap and ultrahigh conductivity were predicted for γ-PC,^33,36^ suggesting plausible applicability in photocatalytic water splitting,^34^ as an anode material in lithium-ion batteries^32^ and for gas sensors.^35^ However, the material has not yet been achieved synthetically to date despite its predicted high stability, in contrast to various other PC allotropes.^39,40^ On the other hand, WS_2_ is a well-known commercially available 2D material. Through varied synthetic procedures, its nanomorphology has been established as nanoflowers, nanospheres, nanowires, and nanobelts.^37^ In general, WS_2_ materials have been reported as excellent UV–vis and NIR light absorbers and strong photoluminescent materials, possessing large exciton binding energy, good carrier mobility, large spin–orbit coupling, and good stability.^37,38^

The devoted starting point of the investigation was to bring a refined perspective on considerable configurational options for modeling BL vdWHs. Then, a “complete bilayer basis derivation (CBBD) algorithm” was introduced, allowing access to the proposed configurational space in practice. It covers any plausible lattice match while approximations are circumvented, all strain types are considered, and while not requiring the input unit cells to be hexagonal or to be of similar geometry. In this regard, we believe that the algorithm trumped over many of the existing encoded BL supercell construction methods,^41−46^ while matching the ARTEMIS program by Taylor *et al*.^47^ The CBBD algorithm further generates a set of systems, which we claim to consist of at least one BL supercell for each plausible supercell-dependent lowest energy configuration. In its application on ≈10% lattice mismatching γ-PC and WS_2_ unit cells, a total of 18,123 nonidentical BL supercells were generated. The patterns and characteristics therein were analyzed. This allowed a thoughtful selection of 45 isotropically strained γ-PC/WS_2_ configurations for further DFT-based geometric relaxation by the rev-vdW-DF2 functional.^48,49^ By execution, geometric and energetic material properties and the band structures were revealed, and in their comparison, isolated effects by twisting and straining were emphasized upon. In the future, other BL vdWHs may be exposed to the applied workflow and its modifications, integrating various other methodologies building forth from DFT.

## 2 Methods

### 2.1 Algorithm Conceptual Foundation

The CBBD algorithm established herein was designed with the objective of generating at least one BL supercell for each obtainable supercell-dependent lowest energy configuration. As a first concern, this objective was reformulated to be a computationally tractable one. Consider any imaginable configuration of an infinitely sized BL parallel to the *xy*-plane through which a PBC-fulfilling simulation box has been defined. It can be modified by translation of one ML over the *xy*-plane while both the cell boundary and the other ML remain fixed, such that (at least) one atom of the constituting MLs stacks on top of each other at identical *xy*-positions. In addition, the cell boundary may be displaced while the BL remains fixed such that the cell edge intersects the stacked atoms, which we then define as the origin (0, 0, *z*) position. It can be shown that PBC remains intact throughout these two operations, which means that the apparent potential energy landscape in DFT treatments remains unchanged. Therefore, the set of only those BL systems which possess at least one atom of both MLs stacked at the origin (0, 0, *z*) position intersected by the cell boundary may be considered a starting point from which all supercell-dependent lowest energy configurations, obtainable by DFT, can in principle be retrieved through geometric optimization. Realize that in this set of systems, the four cell edges must be intersecting the same atom type(s) at identical *z*-coordinate(s).

However, further considerations were needed because the set of systems described above remains infinitely large. A maximum lattice length and global strain were therefore imposed. The latter limited both regular strains over the MLs in the *x*- and *y*-directions and intralayer shear strains over the MLs in the *xy*-plane. Lastly, the strain operations performed by the algorithm were required to coincide with two selected target atoms (one of each ML) precisely at their averaged *xy*-position for the purpose of consistently collapsing the infinite 2D strain space of the supercells to a single point near the optimum. This is further clarified in understanding the algorithm’s computational procedure, explained in the next section. By these considerations, the number of plausibly constructed nonidentical supercells became finite and, hence, computationally tractable. Its derivation was achieved by the CBBD algorithm, thus delivering a complete configurational basis from which all supercell-dependent lowest energy configurations and their STACs (within threshold strain and lattice length) can in principle be found.

### 2.2 Algorithm Computational Procedure

**Figure 1:**
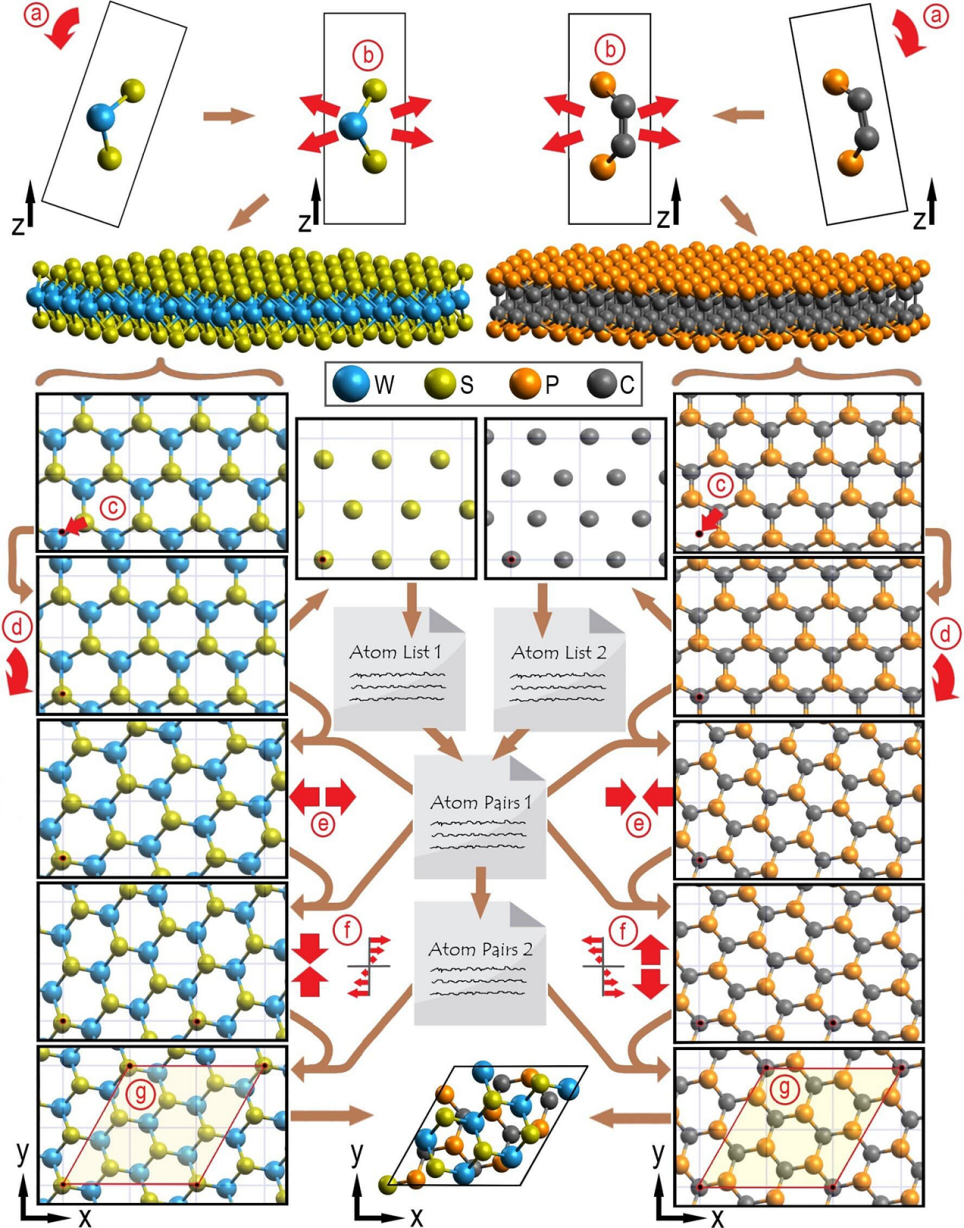
Scheme of the basic procedure for constructing a BL system. The illustrated strains were enlarged to better visualize the procedure. The performed steps are (a) alignment of the ML unit cell with the *z*-axis, (b) expansion of the ML unit cell into a large slab, (c) translation, moving a selected atom to the origin position, (d) rotation of the slab along the *z*-axis at the origin, (e) straining the slab along the *x*-direction, (f) straining the slab along the *y*-direction and providing shear strain, and (g) combining the slabs, carving out a BL supercell.

The key steps of the algorithm and the written code are explained in the following paragraphs and illustrated in Figure 1. As a first preparatory step, the input ML unit cells were perfectly aligned with the *xy*-plane and expanded into large slabs (Figure 1a,b), the details of which are given in Supporting Information 1. The heights of the atoms were adjusted such that the lowest atom of the lower slab was positioned at *z* = 10 Å and the lowest atom of the upper slab was positioned 3 Å above the highest atom of the lower slab. At a later stage, 10 Å of empty space was also built-in above the upper slab. The thereby created 3 Å-sized interlayer distance (*d*_IL_)^50^ and the 20 Å-sized vacuum layer^51−53^ were considered as fitting values for initiating DFT-optimization of the material.

Then, a series of in-plane translations were conducted, each one moving a different atom of the original ML unit cell to the origin (0, 0, *z*) position (Figure 1c). Thereby, *a* + *b* slabs were created, with *a* and *b* being the number of atoms in the ML unit cells. Subsequent code runs for all combinations thereof and thus for a total of *a* × *b* expanded slab pairs.

For each of these combinations, an atom list was created for both of the expanded slabs. All atoms of the slab of the same element at the same height as the atom that was previously moved to the (0, 0, *z*) origin were included. Added to these lists were the distances (*d*) between each listed atom and the origin atom and the angles (α) drawn between the *x*-axis and the line connecting the listed atom with the origin atom. By writing out all combinations of atoms from the atom lists (involving two atoms, each originating from a different slab), fulfilling the criteria given by eqs 1–4, a list of atom pairs was generated.
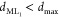
1
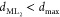
2
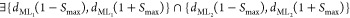
3
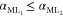
4Here, *d*_max_ is the chosen threshold lattice length of the supercell and *S*_max_ the threshold strain expressed as a fraction. For each entry on this list of atom pairs, the ML slabs were rotated around the origin by angles α_ML1_ and α_ML2_ such that the atoms making up the atom pair lined up on the *x*-axis (Figure 1d). These rotations were carried out via eqs 5 and 6.
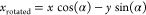
5
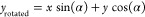
6

The twist-angles of the eventually generated BL systems were defined based on these performed rotations, more specifically via eq 7.
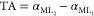
7

where TA is the twist-angle. The atom pair that dictated the slab rotations further dictated the *x*-directional strain on each slab (Figure 1e). Strain was achieved by the multiplication of the *x*-coordinates of all atoms in each slab by a specific factor, making the atoms of the atom pair coincide at their averaged position on the *x*-axis.

Next, a modified list of atom pairs was generated from the original one by additionally requiring the fulfillment of eq 8. Its justification is given in full in Supporting Information 2. Basically, it ensured a degree of proximity between the atoms of the enlisted atom pairs within the intermediate configuration of the BL created up until here.
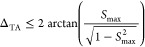
8where Δ_TA_ is the absolute difference between twist-angles, one associated with an atom pair and the other associated with the considered intermediate configuration. Besides, the atom pair that dictated rotation and *x*-directional strain itself was also excluded from the modified list of atom pairs.

In turn, additional strain was introduced for each entry on the modified list of atom pairs, involving *y*-directional strain and intralayer shear strain, the latter displacing the atoms in the *x*-direction proportional to the y-coordinate (Figure 1f). Analogously to before, the strains were built-in via multiplications on the atomic coordinates of the slab, stacking a target atom pair at their averaged *xy*-coordinate.

After the above procedures are followed, at least three positions of stacked atoms must exist on the *xy*-plane. It turned out that a fourth position could always be identified from there and that at least one PBC-fulfilling supercell could be drawn with the cell edges intersecting stacked atoms. In the next step, the BL supercells were generated by “carving them out” of the expanded, rotated, and doubly strained ML slabs (Figure 1g). The implemented carving procedure is detailed in Supporting Information 3.

In performing the repetitions as described above, all searched-for manners to construct a PBC-fulfilling BL supercell were indisputably considered. However, a huge number of duplicate solutions would also be created. Hence, strategies to prevent and filter out duplicates were implemented in the code, as explained in Supporting Information 4. In the end, only nonidentical BL supercells remained.

### 2.3 Coding Aspects and Parametrization

The algorithm was written in computer code compatible with the DFT framework. It required input ML unit cells in the POSCAR format and generated output BL supercells in the POSCAR format. This allowed their direct use in DFT calculations via the Vienna ab initio simulation package (VASP) while facile follow-up file conversion would allow integration with other DFT codes.

In addition to generating the BL supercells, an overview of defining characteristics was given by running the code. Included in this list were the strains, the twist-angles, the number of atoms and ML unit cells fitting in the BL system, the atom types at the (0, 0, *z*) origin, lattice lengths |*a*| and |*b*|, and lattice angle γ. As such, an estimate of all achievable STACs of the material was embedded in this list. Due to numerical instabilities in the code, however, the calculated strains and twist-angles were, in rare cases, adjusted by ±0.001% and ±0.001°. This was manually corrected prior to further analysis of the code outcomes presented in this work. For the γ-PC/WS_2_ system, the CBBD code was run starting from SCAN + rVV10-optimized unit cells and from rev-vdW-DF2-optimized unit cells. In the section “Analysis of the Generated BL Supercells”, the outcomes obtained via SCAN + rVV10-optimized unit cells were generally relied upon. The *d*_max_ parameter was set to 20 Å while allowing shear strains and to 33 Å while disallowing shear strains. Those values were considered large enough to cover most systems eligible for practical DFT implementations. The *S*_max_ parameter was set to 5.5% in all cases, ensuring that the commensurably stacked unit cells were included in the outcome.

Universal applicability of the algorithm to any pair of layered materials was implied by the circumvention of assumptions. The smooth functionality of the written computer code was, besides the γ-PC/WS_2_ system, also verified for graphene/h-BN, MoSe_2_/WSe_2_, and TiO_2_/SnS_2_. The CBBD code outcomes generated for those alternative systems are provided in Supporting Information 5 and briefly discussed in Supporting Information 6.

### 2.4 DFT Implementation

DFT modeling the 45 selected BL systems using the initially preferred SCAN-rVV10 functional^54^ turned out to be too computationally expensive. Hence, the cheaper rev-vdW-DF2 functional^48^ was relied upon. Projector-augmented wave (PAW) pseudopotentials from the .54 version of VASP’s PAW data set were used to treat the core electrons. The “_sv-version” was applied for the W atoms,^55^ while the standard versions were applied to treat the other elements. The cutoff energy was set to 520 eV to expand the plane-wave basis. Spin polarization was included. A conjugate-gradient algorithm was applied where convergence was assumed as the change in free energy of consecutive ionic and electronic relaxation steps became smaller than 10^–6^ eV. Afterward, the electronic structure was recalculated with the convergence criteria sharpened to 10^–8^ eV. Further, an *N* × *N* × 1 Γ-centered Monkhorst–Pack *k*-point grid was applied, where *N* was chosen as an arbitrary constant divided by the magnitude of the lattice vectors, |***a***| = |***b***|, rounded to the nearest integer number. Thereby, the grid point density in reciprocal space became similar among the differently sized supercells. The arbitrary constant was set to 60 during the initial geometric relaxation and increased to 100 during the follow-up electronic relaxation. During geometric relaxation, the shape of the supercell was free to adjust under a constant cell volume. The initially set vacuum layer of 20 Å thereby remained nearly constant, minimizing the self-interaction of the BL in the *z*-direction. The vacuum energy was chosen as the highest averaged local potential in the *z*-direction, excluding the contribution by exchange and correlation, as this appeared to introduce an unphysical variance. Lastly, even though all systems listed in Table 1 were optimized and effectively considered in the study of geometric and energetic features, BLs 9905, 9906, 9913, 9917, 12,829, and 14,455 were excluded from band structure calculation to preserve computational resources.

**Table 1:** Selected BL Configurations for Further DFT Study^a^

BL number	atoms	γ-PC unit cells	WS_2_ unit cells	γ-PC origin atom	WS_2_ origin atom	twist-angle (deg)	γ-PC isotropic strain (%)	WS_2_ isotropic strain (%)
1	7	1	1	C	S	0.00	4.84	–4.41
2	7	1	1	C	S	60.00	4.84	–4.41
3	7	1	1	C	W	0.00	4.84	–4.41
4	7	1	1	C	W	60.00	4.84	–4.41
5	7	1	1	P	S	0.00	4.84	–4.41
6	7	1	1	P	W	60.00	4.84	–4.41
7	25	4	3	C	S	30.00	–2.51	2.65
8	25	4	3	C	S	90.00	–2.51	2.65
9	25	4	3	C	W	30.00	–2.51	2.65
10	25	4	3	C	W	90.00	–2.51	2.65
103	57	9	7	C	S	19.11	–1.64	1.70
104	57	9	7	C	S	40.89	–1.64	1.70
105	57	9	7	C	S	79.11	–1.64	1.70
106	57	9	7	C	S	100.89	–1.64	1.70
107	57	9	7	P	S	19.11	–1.64	1.70
108	57	9	7	P	S	40.89	–1.64	1.70
109	57	9	7	P	S	79.11	–1.64	1.70
110	57	9	7	P	W	100.89	–1.64	1.70
917	103	16	13	C	S	13.90	–0.57	0.58
918	103	16	13	C	S	46.10	–0.57	0.58
919	103	16	13	C	S	73.90	–0.57	0.58
920	103	16	13	C	S	106.10	–0.57	0.58
921	103	16	13	C	W	13.90	–0.57	0.58
922	103	16	13	C	W	46.10	–0.57	0.58
923	103	16	13	C	W	73.90	–0.57	0.58
924	103	16	13	C	W	106.10	–0.57	0.58
925	103	16	13	P	S	46.10	–0.57	0.58
926	103	16	13	P	S	73.90	–0.57	0.58
927	103	16	13	P	W	13.90	–0.57	0.58
928	103	16	13	P	W	106.10	–0.57	0.58
1223	115	19	13	C	S	9.52	–4.64	5.12
1224	115	19	13	C	S	22.69	–4.64	5.12
1231	115	19	13	C	W	9.52	–4.64	5.12
1232	115	19	13	C	W	22.69	–4.64	5.12
1239	115	19	13	P	S	9.52	–4.64	5.12
1243	115	19	13	P	W	22.69	–4.64	5.12
9897	241	37	31	C	S	16.34	0.19	–0.19
9898	241	37	31	C	S	25.77	0.19	–0.19
9905	241	37	31	C	W	16.34	0.19	–0.19
9906	241	37	31	C	W	25.77	0.19	–0.19
9913	241	37	31	P	S	16.34	0.19	–0.19
9917	241	37	31	P	W	25.77	0.19	–0.19
12,825	280	43	36	C	S	7.59	0.17	–0.17
12,829	280	43	36	C	W	7.59	0.17	–0.17
14,455	313	49	39	C	S	5.69	–1.08	1.10

a In columns 3 and 4, the numbers of unit cells of γ-PC and WS_2_ that fit the BL supercell are shown. In columns 5 and 6, the types of the atom at the origin (0, 0, *z*) position are listed for both MLs. The given isotropic strains in columns 8 and 9 were derived using rev-vdW-DF2-optimized ML unit cells as input for the code.

### 2.5 Band Structure Calculation

The bands in the band structures were color coded by the relative occupancies (in %) of the states over the atoms in the γ-PC layer relative to the WS_2_ layer. The states were assigned to one layer or the other, depending on the relative occupancy falling above or below 50%. Based on that, the intralayer band edges were defined. To visualize the effects of the interlayer interaction, the band structures of the isolated MLs alone were plotted on top of the band structures of the BLs for the BL systems up to 57 atoms. For larger systems, this approach was not followed because the huge amounts of overlapping bands were considered to obscure the images.

### 2.6 Additional Computational Details

The binding energy (*E*_b_) as defined by eq 9 allowed a sensible comparison between the differently sized BL systems.^50^
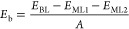
9Here, *E*_BL_, *E*_ML1_, and *E*_ML2_ are the total energies of the BL and its constituting MLs, and *A* is the cross-sectional area of the supercell. The deformation energies of the MLs in the BL supercells were calculated as the decrease in the total energy by geometrically reoptimizing the MLs of the relaxed BLs separately. The CM and *d*_IL_ values were obtained after first perfectly realigning the optimized BL supercells with the *z*-axis, as described in Supporting Information 1. Then, considering Cartesian coordinates, CM was defined as the largest difference in *z*-coordinates found among the upper half of all phosphorus atoms in the γ-PC layer (the lower layer) and for WS_2_ (the upper layer) as the largest difference in *z*-coordinates found among the lower half of all sulfur atoms. *d*_IL_ was calculated by subtracting the averages of these two sets of *z*-coordinates. As such, local out-of-plane corrugations were approximately corrected for in the definition of *d*_IL_.

## 3 Results and Discussion

### 3.1 Analysis of Generated BL Supercells

The list with defining characteristics of all 18,123 generated γ-PC/WS_2_ supercells can be accessed in Supporting Information 5. The entries therein were ordered by the number of atoms in the system, the atom types at the (0, 0, *z*) origin, and then the twist-angles. Afterward, a “BL number” was assigned, which served to label the BL supercells. It turned out that the largest retrieved BL system contained a total of 832 atoms. It also turned out that for each BL supercell, a small number (1–3) of other BL supercells could be identified, differing only by an in-plane translation of one ML relative to the other. Implied by it is that they correspond to the same STAC. As such, the total of 18,123 generated BL systems corresponded to 5728 STAC estimates. This finding rather conveniently suits subsequent DFT study because optimization starting from multiple qualitatively varying initial states may provide more conviction that the most relevant energetic minima and the STAC can be retrieved.

**Figure 2:**
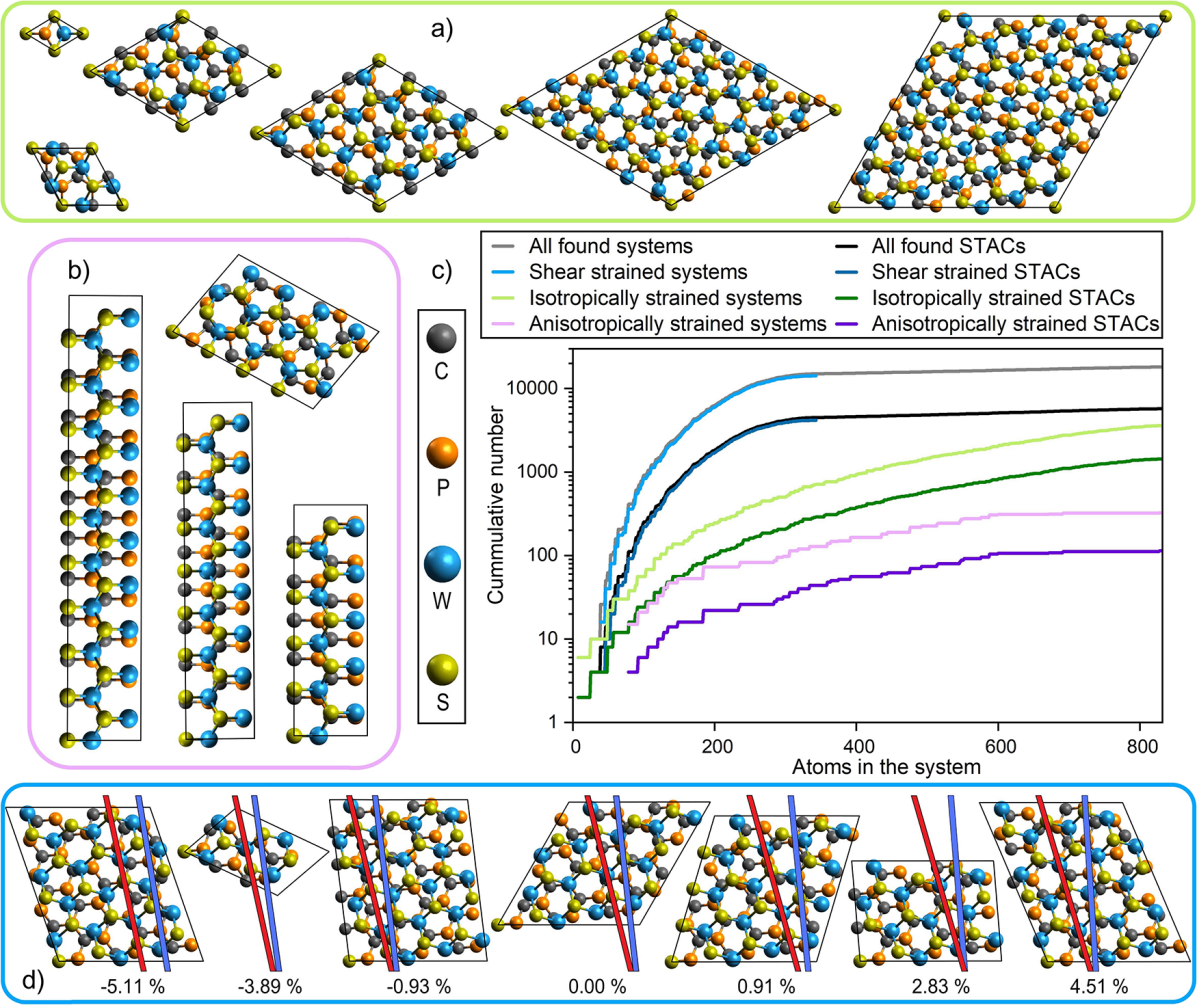
Illustrating (c) categorized cumulative number of systems and STACs against the number of atoms in the system and examples of generated (a) isotropically strained, (b) anisotropically strained, and (d) shear-strained systems. Regarding the latter, red and blue lines were drawn along the armchair direction of WS_2_ and the zigzag direction of γ-PC, respectively, to aid visualizing the shear strains. The percentages below refer to the intralayer shear strain introduced on the γ-PC layer.

The generated configurations were categorized based on the applied strains. In total, 3595 BL systems were isotropically strained along lattice vectors *a* and *b*, 325 were anisotropically strained along lattice vectors *a* and *b*, and 14,203 included intralayer shear strain. The cumulative number of strain-categorized systems and STACs is plotted against the number of atoms in the system in Figure 2c. Note that the curves for the shear-strained and anisotropically strained systems and STACs were diverted downward because the code relied on a maximum lattice length instead of a maximum atom amount. Still, exponential growth of generated BL supercells with an increasing number of atoms in the system may be noted for all types of strain.

**Figure 3:**
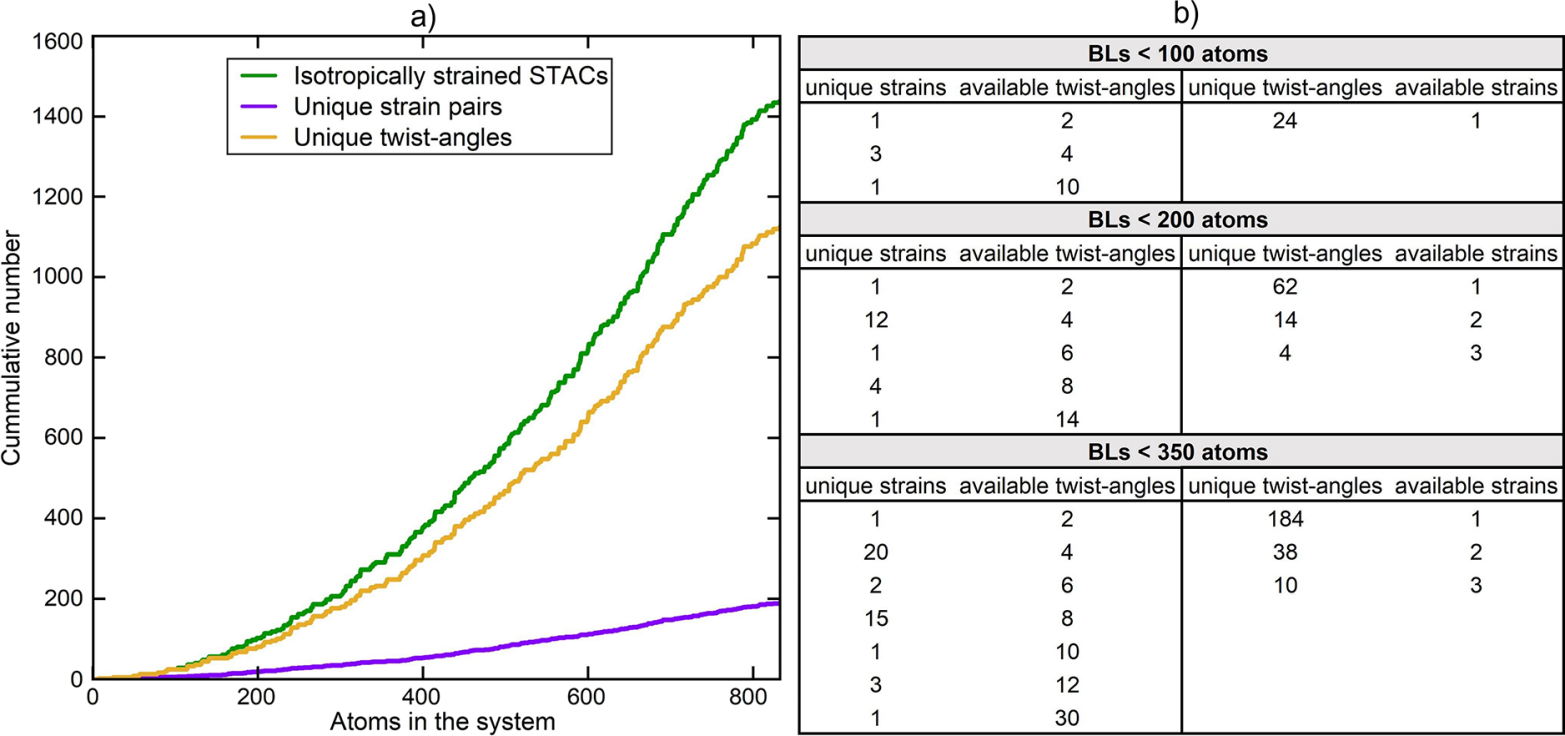
Showing (a) STACs, unique twist-angles, and unique strain pairs against the number of atoms in the system for the generated isotropically strained systems and listing (b) amounts of unique strain pairs per twist-angle and vice versa for isotropically strained systems containing less than 100, 200, and 350 atoms.

Patterns and characteristics of the 3595 isotropically strained BL systems were elaborated on first. As shown in Figure 3a, the number of unique twist-angles far exceeded the number of unique strain pairs among the found isotropically strained systems. To complement this result, the amounts of available strain pairs at a unique twist-angle and vice versa are given in Figure 3b for systems containing less than 100, 200, and 350 atoms. This was of particular interest because it provided initial prospects for the ability of DFT to capture isolated impacts by stacking and straining the γ-PC/WS_2_ material.

Contributing to these outcomes were independently reoccurring strains and twist-angles among differently sized systems. However, the more important observed pattern was the appearance of groups of typically 4, 6, 8, 12, 16, 24, 32, or 48 isotropically strained systems. Within these groups, the BLs were equally sized and identically strained but possessed varied twist-angles at 0 and 60, at 30 and 90°, or at a multiple of 4 different twist-angles conforming to eq 10. This relation emerged because of the honeycomb structure of both γ-PC and WS_2_.
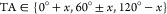
10Here, *x* took the same multiple of seemingly unrelated values between 0 and 30°. Equation 10 implies that for a certain value of *x*, the generated BL systems relate to each other as follows. They are enantiomeric, differ by a 60° rotation of one ML relative to the other, or they would become enantiomeric after a 60° rotation of one ML relative to the other. Alternatively, one of these relations emerges by in-plane translation of one ML. Regardless, similarities between the DFT-calculable properties can be suspected among these systems.

**Figure 4:**
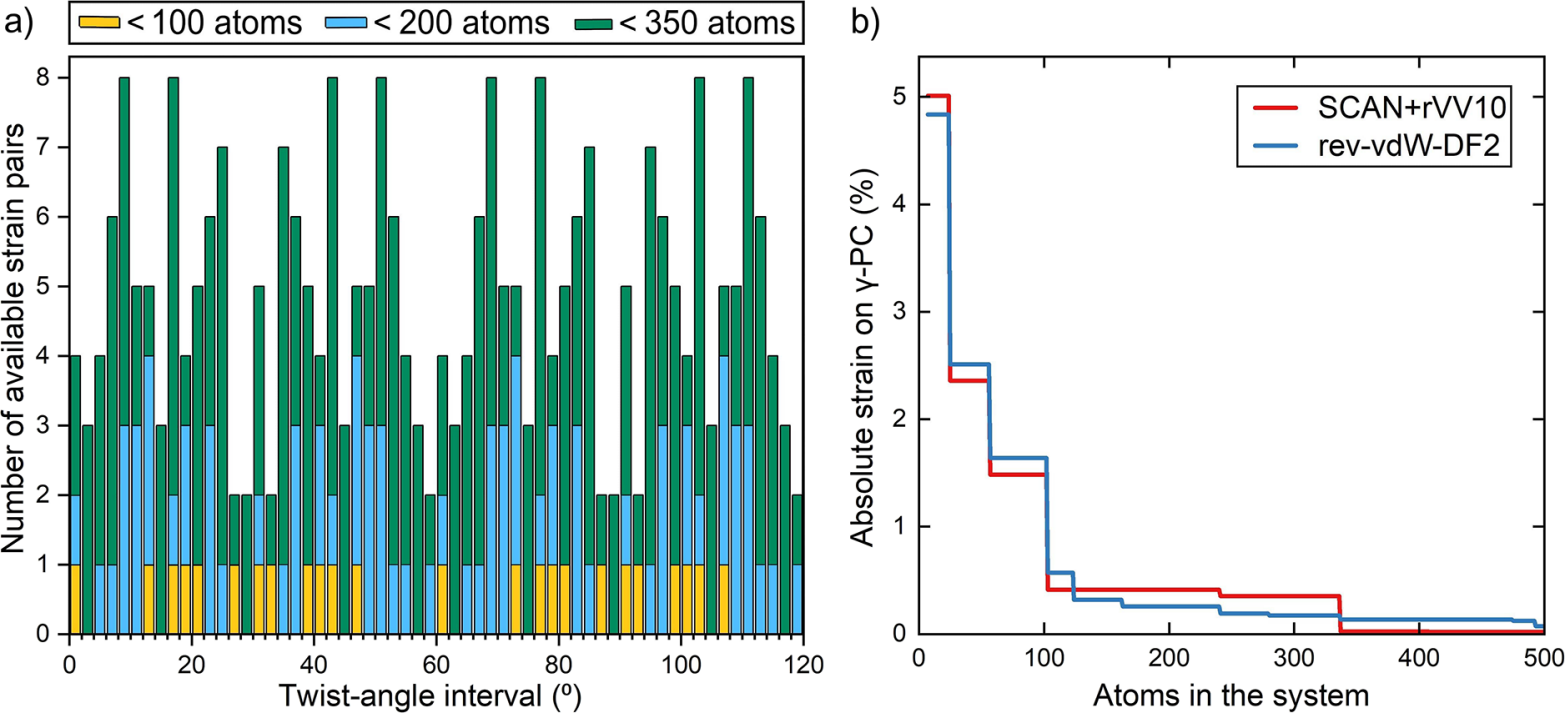
(a) Distribution of twist-angles within 2° intervals for systems with less than 100, 200, and 350 atoms and (b) lowest accessible absolute strain on γ-PC against the number of atoms in the system, where the input unit cell dimensions were obtained via DFT, applying either the SCAN + rVV10 or the rev-vdW-DF2 functional.

Further, the distribution of twist-angles over the interval of 0 to 120° is given in Figure 4a, showing wide twist-angle availability at system sizes, which are well-manageable in practical DFT. Also, the feasibility of modeling strainless γ-PC on WS_2_ configurations was concluded via Figure 4b. Here, the minimum absolute strain on the γ-PC ML is plotted against a threshold number of atoms in the system. Most notably, an absolute strain of merely 0.41% was obtained for a 103-atom-sized system, although this value was found to be sensitive to the method for determining the input unit cell dimensions. As the code was rerun from very slightly deformed unit cells (at lattice lengths of 2.89 and 3.17 Å instead of 2.87 and 3.16 Å), the outcome was retrieved at 0.57%.

Regarding the anisotropically strained systems, 292 supercells were noticed to possess a 90° lattice angle γ, while 33 supercells deviated. Their unequal strains along lattice vectors **a** and **b** were noticed to reoccur in isotropically strained systems along both lattice vectors. We mention the interesting case of 148-atom-sized BL 2614, also shown in Figure 2b. As it contains merely 0.01% of strain in one direction, it represents a unidirectionally strained BL. Simultaneously, a resemblance with a strip on the 784-atom-sized Moiré pattern given by BL 17,895 (Figure S4) can be noticed.

Next, the surprisingly huge amount of 14,203 generated shear-strained systems can be understood as follows. Consider the intermediate of expanded slabs having a stacked carbon and sulfur atom at the origin, −4.50% (γ-PC) and 4.94% (WS_2_) strain along the *x*-direction and a twist-angle of 22.69°. Starting from this intermediate, the further strain operations eventually produced a total of 19 different systems, all except for one possessing intralayer shear strains. The defining characteristics of these 19 systems are grouped together in Table S1, and some of these systems are illustrated in Figure 2d.

Large abundances of small-sized (shear-strained) BL supercells may be of interest, considering that correlations can be widely expected between characteristics over the configurational space. Tracking correlation over systems that are easily computed by DFT might be leveraged to mimic prediction of the more cumbersome via supervised machine learning-based approaches.^50,56,57^ Yet, standalone DFT modeling of shear-strained systems may be of interest as well. Physical manifestations of intralayer shear strain have recently been reported emerging spontaneously in BL graphene,^58^ MoS_2_, and MoS_2_/WS_2_.^59^

### 3.2 System Selection for DFT Treatments

**Figure 5:**
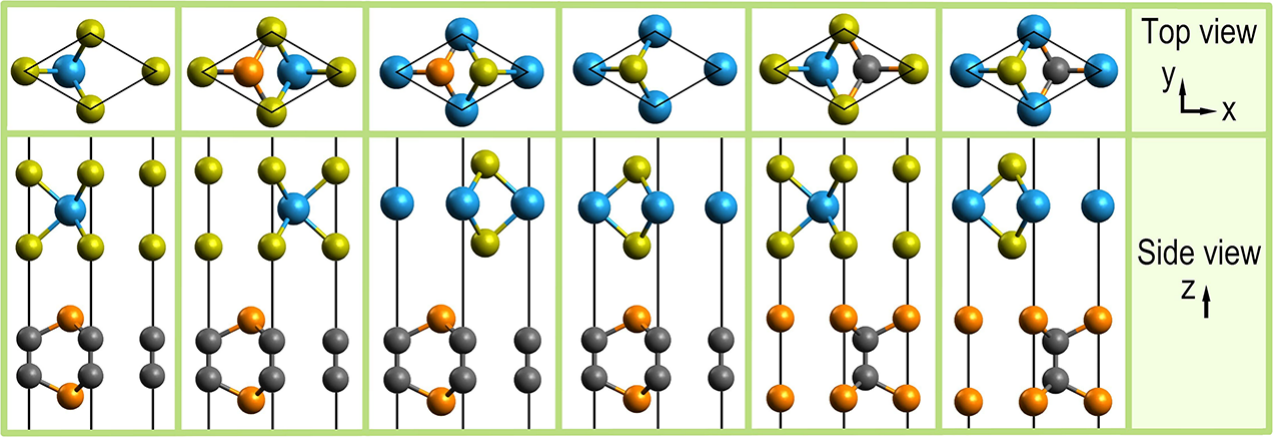
Illustrating BL 1–6 from left to right, the generated commensurably stacked unit cells. The atoms were color coded as in Figures 1 and 2.

A representative subset of systems was selected for DFT modeling from the generated BL supercells, which was restricted to isotropically strained systems only. The system selection started with the six smallest generated systems, containing the high-symmetry configurations of the commensurably stacked unit cells (Figure 5). Noteworthy is that precisely these six configurational types,^53^ or a subset thereof,^30^ have been used as the starting point in previous DFT-based investigations on BL vdWHs of analogous symmetry.

The first few larger systems contained different twist-angles adhering to the relation given by eq 10 and possessed a favorable trade-off between strain and system size, as illustrated by Figure 4b (BL 7–10, BL 103–110, and BL 917–928). Next, relatively small-sized BL supercells, of which the twist-angles breached eq 10 were considered (BL 1223, 1224, 1231, 1232, 1239, and 1243). Additionally, a group of systems containing 241 or 280 atoms, three different twist-angles breaching eq 10, and possessing negligible strains were probed (BL 9897, 9898, 9905, 9906, 9913, 9917, 12,825, and 12,829). Lastly, BL 14,455 was modeled by serendipity. All selected BL supercells have their characteristics summarized in Table 1, and some of them are illustrated in Figure 2a.

### 3.3 DFT-Based Geometric and Energetic Features

**Figure 6:**
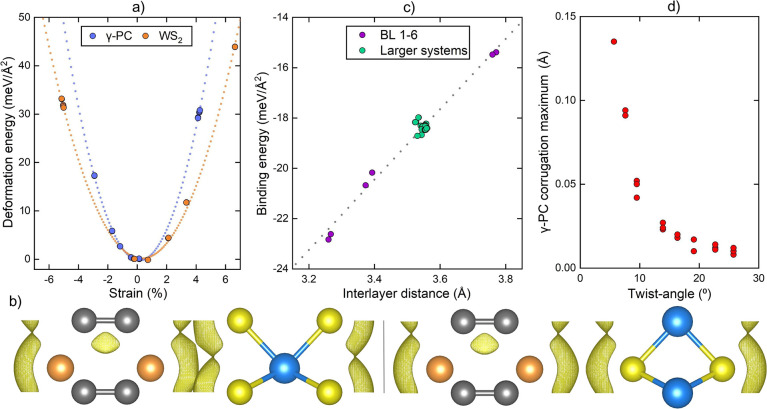
Showing (a) deformation energies per area as a function of strain, (b) electron density isosurfaces of BL 1 (left) and BL 4 (right) at 0.0078 e/Å^3^, (c) calculated values of *E*_b_ and *d*_IL_ correlating linearly, and (d) apparent relation between out-of-plane corrugations in the γ-PC layer and the twist-angle.

The as-generated initial geometries of the BL supercells were adjusted in a few ways through DFT-based optimization. The diameters of the supercells were altered until the MLs took on the least destabilizing strain pair. Thereby, the initially constructed γ-PC layers turned out to be overstrained by up to 60% in BLs 1223–1243, compared to the strain optima. The opposing WS_2_ layers turned out to be understrained by up to 23% in BLs 1223–1243. These outcomes correspond to the steeper profile of the deformation energy per area against the isotropic strain of γ-PC compared to that of WS_2_, shown in Figure 6a. Based on it, a correction to our estimate of the STACs and the generated BL supercell geometries could, in principle, be thought of.

Besides changing the supercell dimensions, DFT-based relaxation moved the positions of the MLs relative to each other. More specifically, the MLs moved along the *z*-axis until the equilibrium *d*_IL_ was reached, although in-plane translations of the MLs remained absent. The resulting *d*_IL_ values were calculated at 3.27 Å up to 3.77 Å for the commensurably stacked unit cells. BL 1 had its layers in the closest proximity. Here, the outer phosphorus atoms of γ-PC stacked on top of the inner tungsten atom of WS_2_ and the inner carbon atoms of γ-PC on top of the outer sulfur atoms of WS_2_. BL 4 had its layers the furthest apart, and the outer and inner atoms of both MLs were stacked on top of each other. Consequently, the electron density isosurfaces interlocked and eclipsed within these two extreme cases (Figure 6b). In turn, the binding energy (*E*_b_) is affected. *E*_b_ and *d*_IL_ appeared to correlate fairly linearly, with the more closely distanced systems expressing a stronger binding energy (Figure 6c). Quantitatively, *E*_b_ ranged between −22.8 and −15.4 meV/Å^2^. The mentioned magnitudes of *E*_b_ and *d*_IL_ can be considered ordinary for BL vdWH systems.^50^

In contrast, all BL systems larger than the commensurably stacked unit cells distanced fairly consistently, with *d*_IL_ adhering to a small interval between 3.52 and 3.56 Å and *E*_b_ taking on values between −18.7 and −18.2 meV/Å^2^. These fundamental similarities lead one to suspect other DFT-calculable characteristics to be analogously less reliant. In particular, the geometric and energetic properties among the groups of BL 7–10, BL 103–110, and BL 917–928 were found to be virtually identical.

In taking a further look, however, a correlation between the magnitude of locally emerging out-of-plane corrugations in the γ-PC layer and the twist-angle in the interval between 0 and 30° could be identified. This is illustrated in Figure 6d via a corrugation maximum (CM) as defined in the section “Additional Computational Details”. The corrugations were clearly expressed more strongly at the lower twist-angles, seemingly independent of strain. A stabilization of the system, thus an increase of *E*_b_, can be inferred to coincide, implying a degree of preference for standalone γ-PC/WS_2_ BL material to adopt a 0° twist-angle. However, an analogous trend, as in Figure 6d, between *E*_b_ and the twist-angle could not be drawn. This was likely because of a dependency of comparable magnitude of *E*_b_ on strain, as it affected the areal atomic densities in the BLs.

All geometric and energetic quantities of interest of the DFT-optimized BL systems are given in Table S2. In addition to the above, we mention that out-of-plane corrugations were very less pronounced in WS_2_, with the largest value of CM reaching only 0.03 Å. Further, in-plane corrugations were not apparent. The phenomenon where commensurably stacked zones appear in BL vdWHs separated by highly strained soliton boundaries^60,61^ was thus not encountered. This can be understood considering that the summed intralayer deformation energies of the MLs in commensurably stacked unit cells were calculated at 62 meV/Å^2^, an order of magnitude higher compared to the largest surplus of *E*_b_ by configurational variation, found at −7.4 meV/Å.

### 3.4 Band Structures

**Figure 7:**
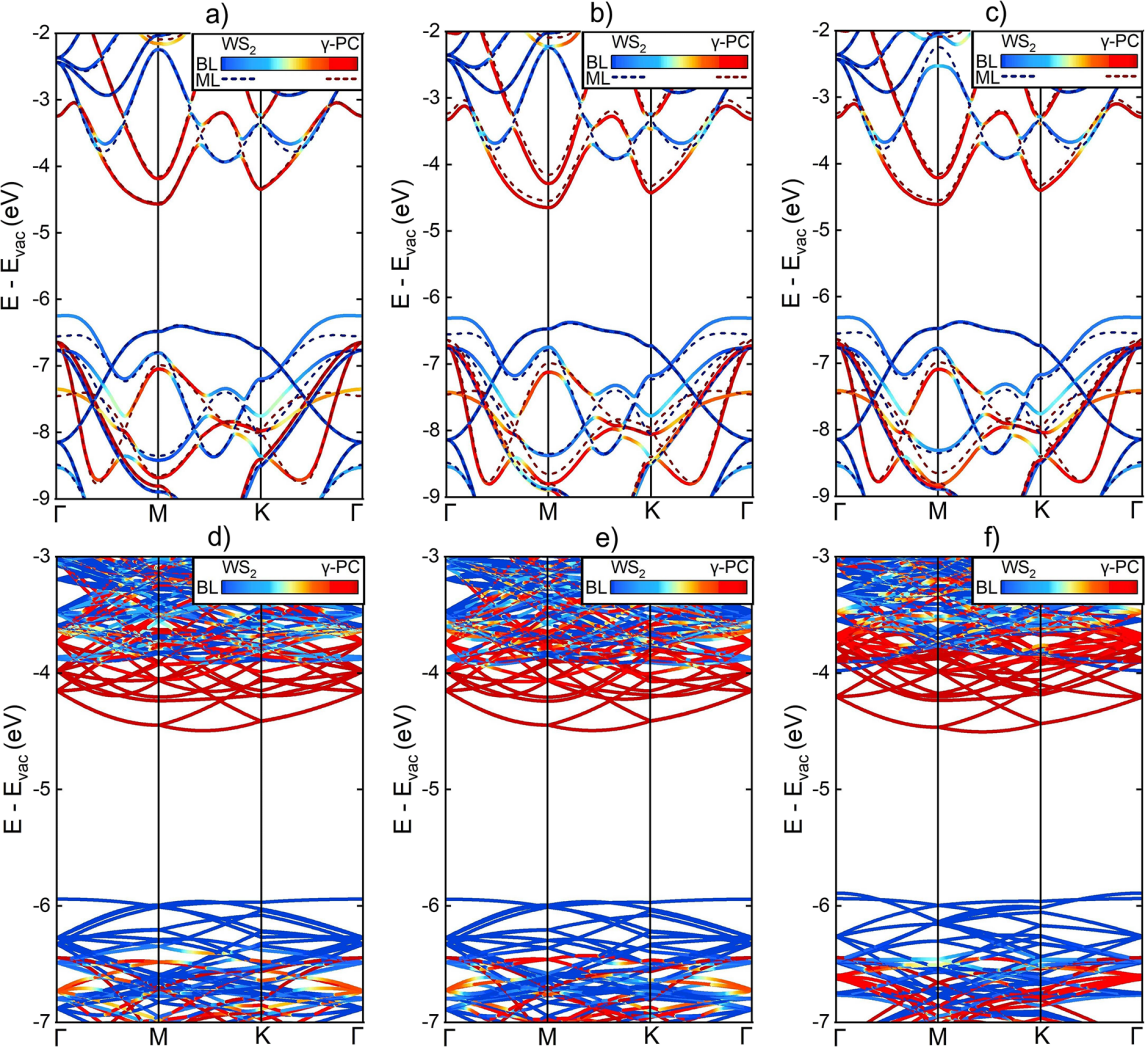
Illustrating from (a) to (f), respectively, the color-coded band structures of BLs 1, 2, 5, 9897, 9898, and 12,825.

The impacts of the interlayer interactions were assessed by comparing the band structures of the BLs to those of the constituting MLs alone. For the commensurably stacked unit cells (Figure 7a–c), an upward shift of the valence band energy of WS_2_ near the Γ-point was found, similar to what was previously reported for the class of transition-metal dichalcogenide heterostructures.^27^ The effect was more pronounced in BLs 1 and 6, the strongest interacting BLs, and came alongside an increased occupancy of the WS_2_ valence band states over the γ-PC layer. Additionally, a shift of the position of the valence band maximum (VBM) from between the M- and the *K*-point to the Γ-point occurred, except in BLs 4 and 5. The conduction band states of WS_2_ seemed unaffected by the interlayer interaction, making intralayer band gaps in WS_2_ range between 2.31 and 2.43 eV, depending on configuration. The energies of the γ-PC states, on the other hand, were shifted downward by the interlayer interaction, up to ≈−0.10 eV for BLs 2 and 3, and basically over the entire band structure. The intralayer band gaps of γ-PC hence remained unaffected compared to the constituting MLs alone and were calculated at 2.08 eV. The band structures of BLs 1–6 were all of type II. The interlayer band gaps varied between 1.66 and 1.74 eV.

Similar impacts of interlayer interactions were found among all larger BL systems. The VBMs of WS_2_ positioned at the Γ-point as well and were shifted upward by a variable amount between 0.01 and 0.10 eV, compared to the standalone MLs. The conduction band minimum (CBM) of WS_2_ remained unaffected in all cases. For γ-PC, the VBMs and CBMs shifted downward between −0.08 and −0.10 eV compared to the isolated MLs, with the exception of BL 9897 and BL 9898, where the VBMs were affected by −0.03 and −0.06 eV, respectively.

Thus, the changes to the band energies by the BL interactions were minor. Trends with the strains or twist-angles could not be convincingly identified. Furthermore, the band structures of BLs 1, 2, and 4 very strongly resembled the ones of BLs 6, 3, and 5, respectively. The band structures calculated for the groups of BLs 7–10, BLs 103–110, and BLs 917–928 seemed practically identical (Figure S5). This is in line with the similarities among their energetic and geometric features.

In a further analysis, the occupancies of the states in the band structures were carefully examined. The extent with which the state occupancies were shared across both γ-PC and WS_2_ layers appeared to vary at the crossings of the ML bands in the comparison between BLs 9897, 9898, and 12,825 and over the entire BL band structure (Figure 7d–f). Most notably, a relative occupancy of 50% over both layers was attributed to the VBM of γ-PC in BL 9897, while it remained (almost) fully localized on γ-PC in BLs 9898 and 12,825. This variation can be considered as dictated by the twist-angle, which took on values of 16.34, 25.77, and 7.59°, whereas the strains differed negligibly. The finding implies that the optical and excitonic behaviors of γ-PC/WS_2_ can be expected to vary with the twist-angle. Indeed, Fermi’s golden rule states the probability of electronic transition to be proportional to the spatial extent of the acceptor state at the energy of the donor state.^62^ Analogous findings on varied state occupancies were found while comparing the band structures of BLs 1223 and 1224, given in Figure S6.

**Figure 8:**
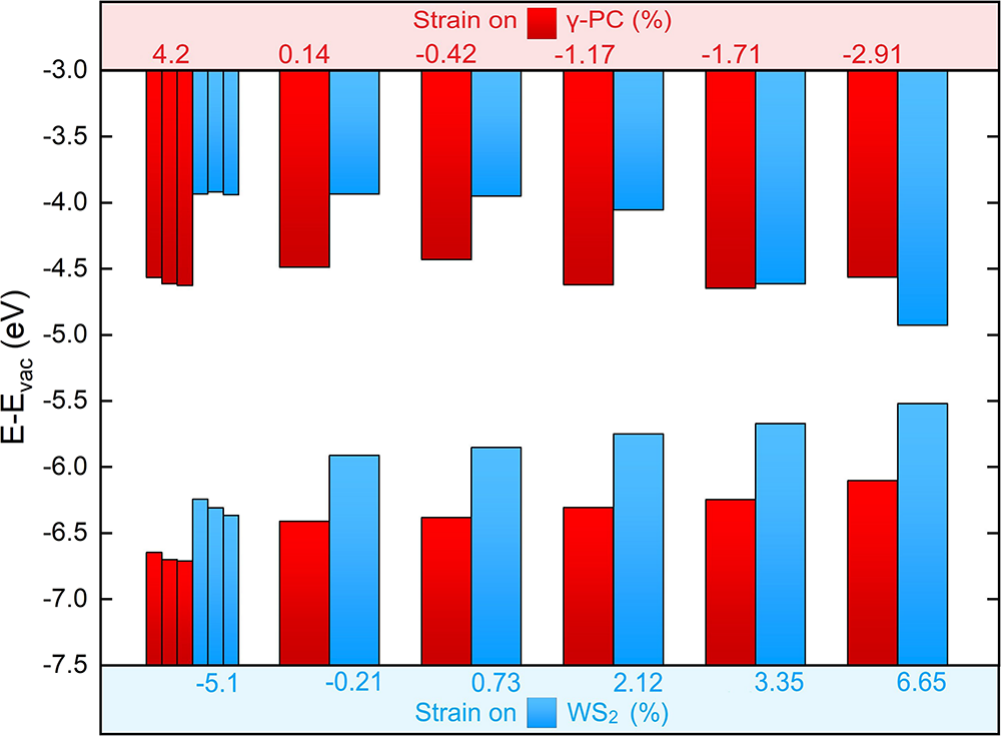
Band edges of the relaxed BLs at varied imposed heterostrains. BLs 1 and 6, BLs 2 and 3, and BLs 4 and 5 were shown together separately (from left to right) at 4.2% strain on γ-PC and −5.1% strain on WS_2_.

The positions of the intralayer band edges at the varied heterostrains over the BLs are given in Figure 8. Herein, the band edges were depicted as the averages of the different probed, equally strained BL systems. This is justified as the band energies were found to be very sensitive to the applied heterostrain compared to the interlayer interaction. Considering the states occupying the WS_2_ layer of the BLs, the VBMs increased from −6.3 to −5.53 eV, with increasing strains from −5.1 to 6.65%. The CBMs decreased from −3.94 to −4.94 eV with increasing strains from −0.21 to 6.65%, although they altered negligibly by compression. For the states occupying the γ-PC layer, the VBMs decreased from −6.12 to −6.7 eV with increasing strains from −2.91 to 4.2%, whereas no clear trend between the CBMs and the strain could be identified. As the band edges of WS_2_ were closing upon stretching the ML, the BL was noticed to transition from a type II to a type I heterostructure beyond 3.35% strain on the WS_2_ layer. All quantities of interest related to the band structures are given in Table S3.

## 4 Conclusions and Outlook

A perspective on configurational space in DFT-modeling BL vdWHs was explained, and the CBBD algorithm was proposed to access it. In application to γ-PC/WS_2_ BL, 3595 isotropically strained, 325 anisotropically strained, and 14,203 shear-strained systems were generated, of which 45 isotropically strained systems served to represent the material in the follow-up DFT study.

The highlights of this DFT study were that calculated *d*_IL_ and *E*_b_ parameters differed substantially among optimized commensurably stacked unit cell configurations, whereas they lied in close proximity for all larger systems. Still, a relationship between the twist-angle and local out-of-plane corrugations in the γ-PC layer could be identified. The band energies were minorly affected by the interlayer interactions and strongly affected by the applied heterostrains. Specific states in the band structures were noted for having a varying occupancy over the layers at a differing twist-angle between 0 and 30°. Overall, the fundamental material properties of γ-PC/WS_2_ and their variations over differently constructed BL supercells were described.

The success of the demonstrated workflow relied on understanding and responding to the PBC-imposed restrictions in the DFT-based study of BL vdWHs. The approach may be reused on other BL vdWHs and derivatives alongside integrations of various DFT-derived methodologies, possibly complemented by machine-learning-based techniques. In particular, we would like to encourage the search for systems where optimizations of varied BL supercell constructions lead to unusual variability in geometry, believing it to be vital in underpinning tunability by twisting and straining on different levels.

## Data Availability

The codes and underlying data for this study were made openly available in a Zenodo repository at https://doi.org/10.5281/zenodo.8304785.

## Supporting Information Available

Additional details on CBBD algorithm and coding, listings and illustrations of generated bilayer systems, quantities of interest, and band structures (PDF)
Characteristics of generated γ-PC/WS_2_ supercells (PDF)
Characteristics of generated graphene/h-BN supercells (PDF)
Characteristics of generated MoSe_2_/WSe_2_ supercells (PDF)
Characteristics of generated TiO_2_/SnS_2_ supercells (PDF)
