# Supplementary material for: Systematic DFT Modeling van der Waals Heterostructures from a Complete Configurational Basis Applied to γ-PC/WS2
Source: J Chem Theory Comput. 2024 Mar 6;20(6):2377–89. doi: 10.1021/acs.jctc.3c00932 (PMC10976641; doi:10.1021/acs.jctc.3c00932)
Supplement: Supplementary file 1 — ct3c00932_si_001.pdf [file ct3c00932_si_001.pdf]

Supplementary Information:

Systematic DFT Modeling van der Waals Heterostructures from a Complete Configurational Basis Applied to γ-PC/WS_2

Joran Celis[a*] *and Wei Cao*[a]

[a]Nano and Molecular Systems Research Unit, Faculty of Science, University of Oulu, FIN-90014, Oulu, Finland

[*]Email: joran.celis@oulu.fi

Table of Contents

Supplement 1: alignment and expansion of the ML unit cells 3

Supplement 2: derivation of Eq. 8. 4

Supplement 3: carving out the BL supercells from expanded slabs 6

Supplement 4: avoiding and filtering duplicate BL systems 7

Supplement 5: generated CBBD code outcomes 10

Supplement 6: discussion on graphene/h-BN, $MoSe_2/WSe_2$ and TiO_2/SnS_2 systems 11

Supplement 7: showing BL 148 resembling a strip on BL 17895 12

Supplement 8: exemplary list of 19 systems 13

Supplement 9: geometric and energetic quantities of interest 13

Supplement 10: band structures of BL 7, 9, 103, 104, 917 and 918 14

Supplement 11: band structures of BL 1223 and 1224 17

Supplement 12: band-structure-related quantities of interest 18

Supplement 1: alignment and expansion of the ML unit cells

It was recognized that geometric relaxation of a ML by DFT may slightly truncate the cell shape and that small misalignments between the input cells and the xy-plane may affect the performance of the encoded CBBD-Algorithm. Hence, the input cells were first tilted, perfectly aligning its upper plane with the xy-plane. This was achieved by first redefining the coordinate system as follows (**Fig. S1a**). The newly defined y'-axis was set perpendicular to the upper plane of the input cell. Then, the newly defined z'-axis was set perpendicular to the y'z-plane. Then, the newly defined x'-axis was set perpendicular to the y'z'-plane. Within the new coordinate system, tilting of the input cells was achieved via **Eq. 5** and **6**, with α being the angle between the y'-axis and the original z-axis. After tilting the input unit cell, the coordinate system was redefined as it was originally.

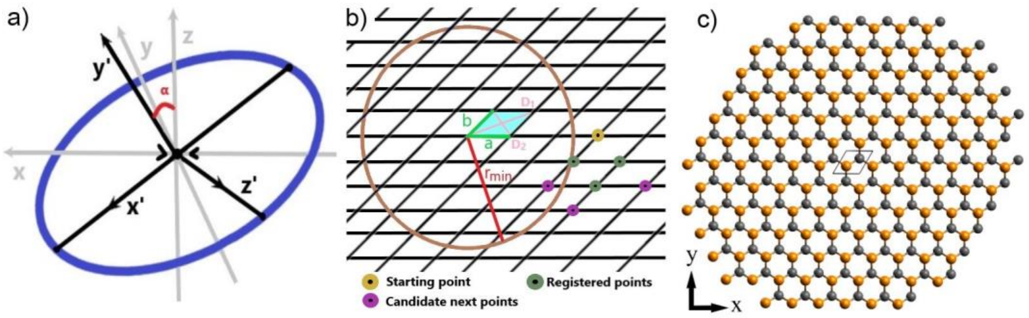

Figure S1. Illustrating **a)** the changes to the coordinate system during initial tilting of the input cell, with the blue circle representing the upper plane of the input unit cell, **b)** the method used to derive how to expand the starting unit cell to create a large enough slab, and **c)** an example of an expanded γ-PC slab, obtained with d_{max} parameter set to 4 Å.

Then, the unit cells were copied several times in both the directions of the lattice vectors **a** and **b** to create slabs which are large enough such that any derived BL supercell can be carved out from them, at the end of the procedure. A slab can be shown to become guaranteed to be large enough for this purpose if it fully spans the circle around the origin with radius r_{min}, when r_{min} is given by **Eq. S1**.

$$r_{min} = \frac{2 \, . \, d_{max}}{1 - S_{max}} + D \tag{S1}$$

Here, d_{max} is the threshold length of the supercell, S_{max} the strain threshold expressed as a fraction, and D the largest of the two diagonals which can be drawn over the cross-section of the unit cell in the xy-plane. The information on *how* to sensibly copy the unit cells to create a slab just fulfilling this requirement was extracted from a set of lattice points, which was derived as follows (**Fig. S1b**). The first lattice point was given by the smallest multiple of lattice vector **a** located outside of the r_{min}-sized circle. Its four neighbouring lattice points, then, formed the candidate second points of which the lattice point most closely residing to the origin, but outside of the r_{min}-sized circle, was chosen. All further lattice points were analogously derived but without considering previously registered lattice points as candidates. Then, the set of lattice points completes as the starting lattice point is reobtained. Then, the r_{min}-sized circle becomes encircled by the registered lattice points. Therefore, they indeed contain the information on how to sensibly copy the unit cells to create a large enough slab. An example of an expanded γ-PC slab was illustrated in **figure S1c**.

Supplement 2: derivation of Eq. 8.

Recall **Eq. 8**:

$$\Delta_{TA} \leq 2 \arctan\left(\frac{S_{max}}{\sqrt{1 - S_{max}^2}}\right) \tag{8}$$

Here, Δ_{TA} is the absolute difference between the twist-angle associated to an atom pair from the list of atom pairs, compared to the twist-angle given to the considered intermediate configuration.

To help derive this equation, a representative intermediate BL configuration was illustrated in **figure S2,** obtained after the rotations on both MLs were carried out. Here, 'atom pair 1' refers to one which dictated the rotations. 'Atom pair 2' could be any other atom pair from the list of atom pairs fulfilling

Eq. 1-4. Further, it was assumed that both atoms of atom pair 2 position at an equal distance d from the origin. Then, the displacement needed to coincide both atoms of atom pair 2 at the averaged xy-position is given by the length 'o', defined in **figure S2**. We say that length 'o' is not allowed to exceed the distance d multiplied by the set threshold strain S_{max}. Otherwise, the second strain operation would end up creating an unwanted overstrained BL system.

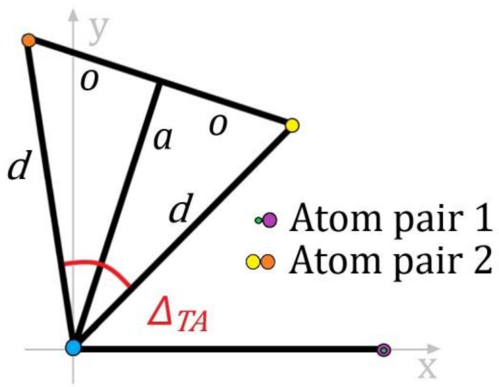

Figure S2. Representing an intermediate state of the BL after the first strain operation.

We can thus write,

$$o_{max} = d \,.\, S_{max} \tag{S2}$$

This can be brought in relation with a maximum allowed difference between the twist-angles associated to atom pair 1 and atom pair 2 ($\Delta_{TA_{max}}$) as follows:

$$\Leftrightarrow \frac{o_{max}}{a} = \frac{d.S_{max}}{a} \tag{S3}$$

$$\Leftrightarrow \frac{o_{max}}{a} = \frac{d.S_{max}}{\sqrt{d^2 - o^2}} \tag{S4}$$

$$\Leftrightarrow \frac{o_{max}}{a} = \frac{d.S_{max}}{\sqrt{d^2 - d^2.S_{max}^2}} \tag{S5}$$

$$\Leftrightarrow \frac{o_{max}}{a} = \frac{S_{max}}{\sqrt{1 - S_{max}^2}} \tag{S6}$$

$$\Leftrightarrow 2\ \arctan\left(\frac{o_{max}}{a}\right) = 2\ \arctan\left(\frac{S_{max}}{\sqrt{1-S_{max}^2}}\right) \quad \text{(S7)}$$

$$\Leftrightarrow \Delta_{TA_{max}} = 2\ \arctan\left(\frac{S_{max}}{\sqrt{1-S_{max}^2}}\right) \quad \text{(S8)}$$

Which implies **Eq. 8**. In turn, the validity of **Eq. 8** under the assumption of equal distances d for atom pair 2, serves as the starting point to confirm the validity of the equation when the assumption is not strictly true. It can be shown that $\Delta_{TA} > \Delta_{TA_{max}}$ will then still imply that the atom pair 2 must be unfit to dictate the second strain operation, because it would always lead to an overstrained BL. However, allowing unequal distances d within atom pair 2 does lead to the possibility that even when $\Delta_{TA} \leq \Delta_{TA_{max}}$, certain atom pairs will be dictating the generation of overstrained BL systems. This imperfection was not considered a difficult problem though. At a later stage of the code, all slightly overstrained BL systems were easily recognized and discarded at little computational expense.

Supplement 3: carving out the BL supercells from expanded slabs

In our DFT-calculations of γ-PC/WS_2, it was preferred to treat only the smallest possible repeating unit of a BL system. Additionally, when equally sized BL supercells with varying lattice angle (γ) could be drawn for a single system, the option where γ is closest to 90 ° was preferred. Presumably, the same preferences hold for most DFT-studies on BL vdWHs. Therefore, the carving procedure of the BL systems from the extended slabs was designed to target only those commonly preferred supercells.

The steps taken to carve out the BL supercell were decoupled from previous intermediate outcomes. The origin was considered as the first cell edge position. The stacked atoms alike those at the origin (0, 0, z) position with $x > 0$ and $y = 0$ defined the potential second cell edge positions. The stacked atoms alike those at the origin (0, 0, z) position, with $y > 0$ defined the potential third cell edge

positions. This leads to a set of potential triplets of cell edge positions. In turn, the three existing complementary fourth edge positions were identified for each given triplet. The resulting quadruplets were then considered to map the potential ways to carve out a PBC-fulfilling system.

Next, the quadruplets were sorted in descending size of the cross-sectional area of the supercell in the xy-plane and with descending deviation of the lattice angle γ with 90 degrees. Afterwards, the fulfilment of PBC was cross-checked for the quadruplets in descending order until a positive outcome was obtained. The cross-checking involved, first, a preliminary confirmation of the presence of the stacked atoms alike those at the origin, at the fourth cell edge position. Then, two copies of the considered BL supercell candidate were created and translated along lattice vectors **a** and **b** with lattice lengths |**a**| and |**b**|. Then, all atomic positions of the BL supercell copies were compared to the atomic positions of the extended slabs. It can be shown that the radius of the extended slabs, as defined by **Eq. S1**, will always be large enough to successfully perform this PBC-fulfilment test. After confirming PBC-fulfilment, a BL supercell is effectively 'carved out' from the slabs by building a POSCAR file containing the BL supercell.

We mention that this is essentially a flawed approach. The BL supercells with lattice parameters **a** and **b** smaller than $d_{max}/2$ failed to be carved out as desired. Instead of providing an algorithmic improvement, the code was simply rerun at varied settings for the d_{max} input parameter, and well-carved outcomes were combined.

Supplement 4: avoiding and filtering duplicate BL systems

In this section, applied performance improvements were explained. We mention three general performance improvements. Firstly, stacked atoms on the xy-plane of the individual ML unit cells were considered together. Therefore, as $a * b$ repetitions of the encoded procedure were carried out, a and b become the number of non-stacked atoms instead of the total number of atoms in the unit cells. For γ-PC/WS_2 material $a * b$ thus becomes $2 * 2$ instead of $3 * 4$.

Secondly, additional requirements beyond **Eq. 1-4** were set when listing the atom pairs. These were written in **Eq. S9** and **S10** and further prevent retrieving duplicate solutions. However, their validity required the assumption of centrosymmetry in the ML unit cells and therefore the equations may not be universally applicable. For the γ-PC/WS_2 system, nevertheless, it was safe to implement **Eq. S9** and **S10**.

$$\alpha_{ML_1} < 180\,° \tag{S9}$$

$$TA = \alpha_{ML_2} - \alpha_{ML_1} < 180\,° \tag{S10}$$

Thirdly, the option was built-in to disallow shear strains. Under this setting, larger systems may be searched for at far more efficient computational expense.

In addition, a performance improvement was realized through parallelizing the code. Thus, all repetitions of each operation generally finalise, prior to initiating the next operation. The advantage of this strategy is that duplicate intermediates can be tracked and filtered as they become apparent, omitting the computational expense needed to perform any further computations.

Two types of filters were designed for this purpose. The first type functioned based on overlapping atomic coordinates and was carried out after introducing x-directional strain. However, filtering in this manner failed to recognize identical BL intermediates which were rotated or translated as a whole. Hence, a more sophisticated method of filtering was needed. The second filter type functioned by drawing a path connecting 6 atomic positions. These 6 atomic positions were selected one after another, and according to specific selection rules:

- **i)** All atoms in the BL are projected onto the same xy-plane in determining the path.
- **ii)** The starting point of the path considers all atoms of the BL, and stacked atoms are always considered together.

iii) In determining the 2nd, 4th and 6th position, only the atoms of one ML in the BL are considered, and stacked atoms within this ML are always considered together.

iv) In determining the 3th and 5th position, only the atoms of the other ML are considered and stacked atoms within this ML are always considered together.

v) Generally, the atom which lies closest to the last selected atomic position of the path, defines the next atomic position of the path.

vi) The 1st, 2nd, 4th and 6th atomic position may never coincide. Also, the 1st, 3rd and 5th atomic position may never coincide.

vii) If multiple potentially selected atomic positions reside at equal distance from the last selected atomic position of the path, the counterclockwise angle between the second last, the last, and the next atomic position is calculated for the potential next atomic positions. Then, the one where this angle is closest to 180 ^{o} is selected. If the last and the second last atomic positions coincide, then the third last atomic position will be included in defining the angle. If two equally valid candidates then still exist (for example at angles 120 ^{o} and 240 ^{o}), then the atom making up the largest angle will define the next atomic position of the path.

Afterwards, a fingerprint of the drawn path was generated. It contained the 5 distances between the atomic positions, the counterclockwise angles between atomic positions 1-2-4, 1-2-5 and 1-2-6, and the elements and the z-coordinates of the atoms that dictated the atomic positions. Here, it was assumed that in choosing a common starting point, identical path fingerprints would be generated for identical systems and different path fingerprints for different systems. This appeared to be true in case of γ-PC/WS_2. However, ambiguity in the selection rules still occurred. Upon requiring an angle for determining the second atomic position (rule **vii**), a second last atomic position would not exist. In such cases, multiple path fingerprints were generated, one for each valid option.

Filtering by path fingerprints was carried out after x-directional strain was introduced. Then, the paths were chosen to debut from the origin (0, 0, z) position. Further filtering by path fingerprints was performed after y-directional strain and shear strains were introduced. Then, the paths were drawn starting from the origin (0, 0, z) position and starting from other positions on the xy-plane where atoms of the MLs coincided, for which the elements or z-coordinates differed compared to the stacked atoms at the (0, 0, z) origin.

We mention that a small minority of duplicate systems were found to remain unrecognized by the applied filters due to numerical instabilities. After manually altering few strains and twist-angles by ± 0.001 % and ± 0.001 ^{o} in the generated summary of BL supercell characteristics, the identical entries within were easily tracked. Based on it, few additional recognitions of duplicate BL supercells were made. In turn, only unique BL supercells were kept.

Supplement 5: generated CBBD code outcomes

Since the list of all 18123 derived γ-PC/WS_2 BL supercells was huge, it was provided in a separate file entitled 'Supplementary_Dataset1.pdf'. Similarly, the CBBD code outcomes for the alternative systems of graphene/h-BN, $MoSe_2$/WSe_2 and TiO_2/SnS_2, were given by 'Supplementary_Dataset2.pdf,' 'Supplementary_Dataset3.pdf,' and 'Supplementary_Dataset4.pdf,' respectively.

Supplement 6: discussion on graphene/h-BN, $MoSe_2/WSe_2$ and TiO_2/SnS_2 systems

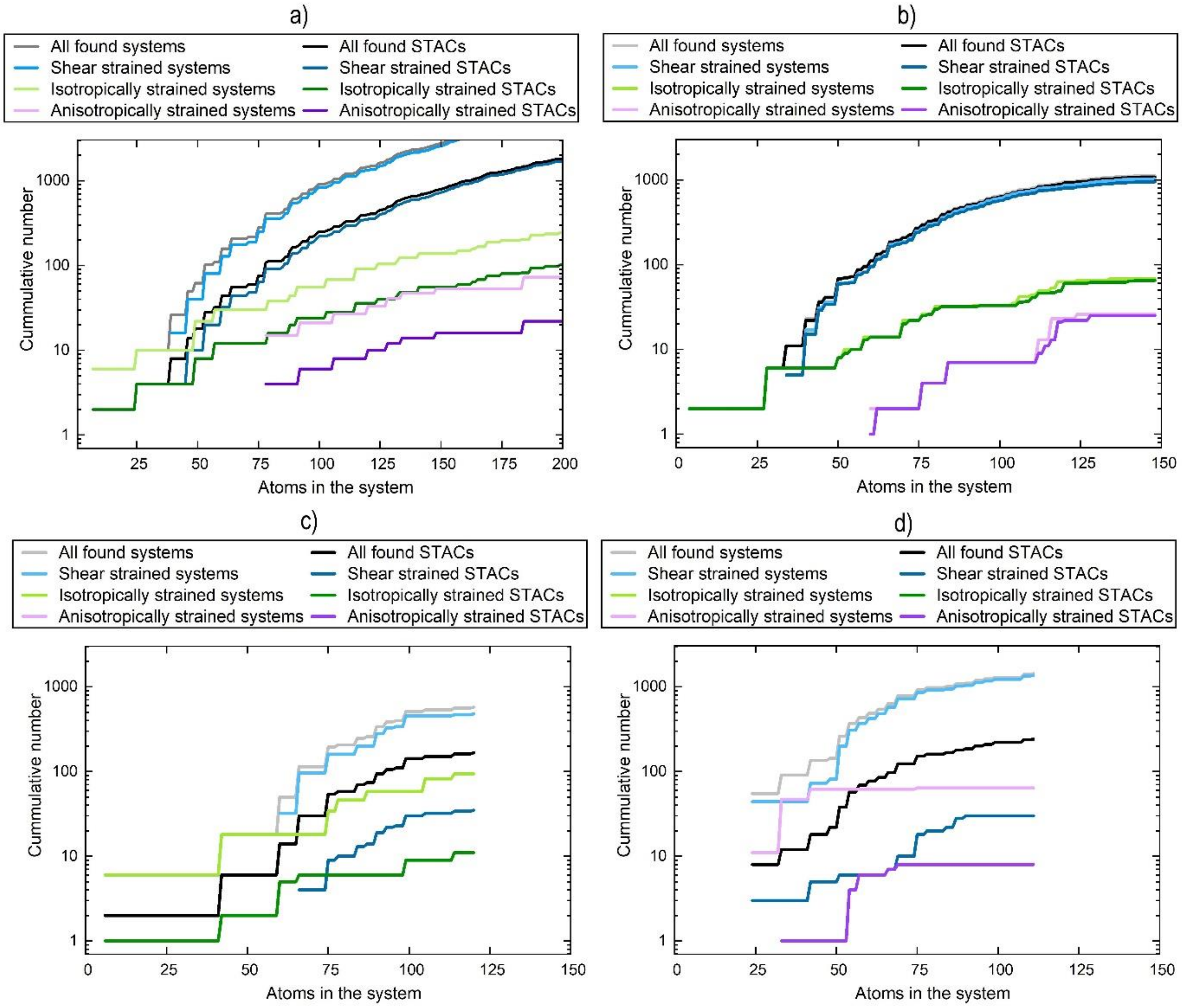

Figure S3. The cumulative number of strain-categorized outcomes plotted against the number of atoms in the system for **a)** γ-PC, **b)** graphene/h-BN, **c)** $MoSe_2/WSe_2$ and **d)** TiO_2/SnS_2 systems. The MAXLENGTH variable in the CBBD code was set to 33 Å excluding shear strains and to 20 Å including shear strains for the γ-PC/WS_2 system. For the alternative systems it was kept at 15 Å whilst shear strains were included. The MAXSTRAIN variable was set to 5.5 % in all cases.

It can be noticed for graphene/h-BN that the number of found systems is almost equal to the number of STACs, which of course stems from graphene consisting of only a single element. For the case of $MoSe_2/WSe_2$, the lack of anisotropically strained systems stems from the 0 % lattice mismatch of its unit cells. Finally, the lack of isotropically strained systems for TiO_2/SnS_2 results from combining a hexagonal with a square unit cell.

Supplement 7: showing BL 148 resembling a strip on BL 17895

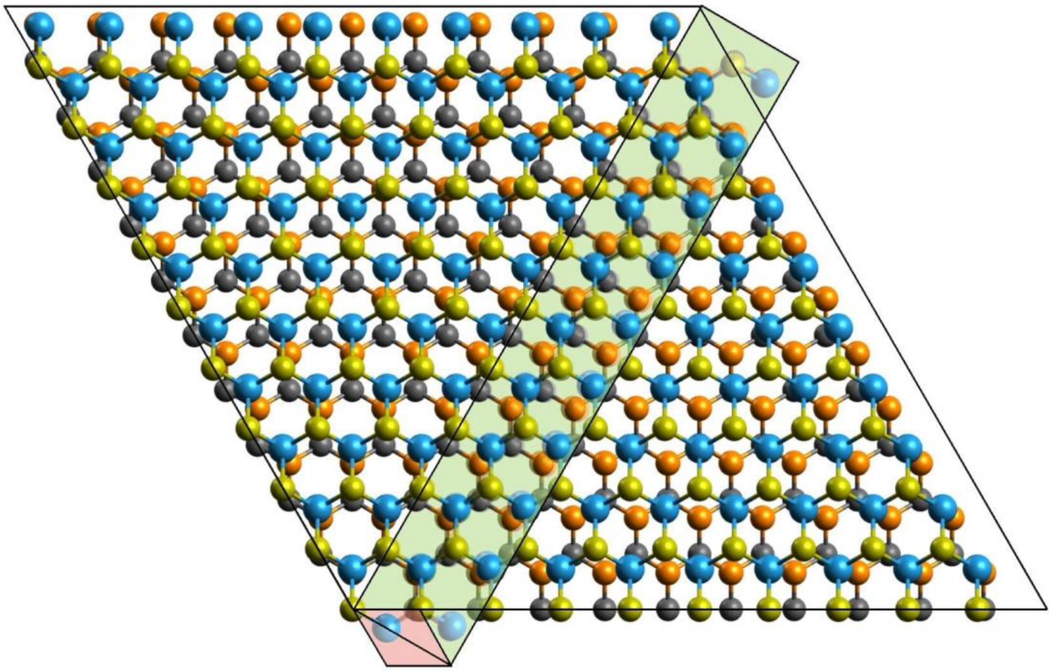

Figure S4. Showing BL 17895 in a white background, BL 148 in a green background, and BL 1 in an orange background.

Supplement 8: exemplary list of 19 systems

Table S1. Summarizing 19 systems generated from the same intermediate configuration of rotated and x-directionally strained expanded slabs.

BL number	Atoms	γ-PC unit cells	WS_2 unit cells	γ-PC origin atom	WS_2 origin atom	twist-angle (°)	γ-PC strain 1 (%)	γ-PC strain 2 (%)	γ-PC strain 3 (%)	WS_2 strain 1 (%)	WS_2 strain 2 (%)	WS_2 strain 3 (%)	γ (°)	$\lvert a \rvert$ (Å)	$\lvert b \rvert$ (Å)
11	39	6	5	C	S	22.69	-4.50	-3.89	5.42	4.94	3.51	-4.89	65.36	8.49	5.60
647	93	15	11	C	S	22.69	-4.50	2.83	-1.23	4.94	-2.90	1.26	85.25	11.96	8.49
1099	110	17	14	C	S	22.69	-4.50	3.62	4.77	4.94	-3.30	-4.35	72.99	11.96	10.63
1224	115	19	13	C	S	22.69	-4.50	0.00	-4.50	4.94	0.00	4.94	60.00	11.96	11.96
1983	132	21	16	C	S	22.69	-4.50	0.91	0.67	4.94	-0.90	-0.66	73.52	11.96	12.59
2318	139	22	17	C	S	22.69	-4.50	-5.11	1.39	4.94	4.97	-1.35	70.86	11.96	13.48
2568	147	24	17	C	S	22.69	-4.50	4.51	-2.89	4.94	-4.78	3.07	66.89	11.96	14.47
2692	149	23	19	C	S	22.69	-4.50	1.66	4.94	4.94	-1.51	-4.50	85.18	11.96	13.83
2972	154	25	18	C	S	22.69	-4.50	-0.93	-2.12	4.94	0.97	2.21	83.03	11.96	14.07
3582	164	26	20	C	S	22.69	-4.50	4.89	1.16	4.94	-4.78	-1.13	75.53	11.96	15.51
4155	169	28	19	C	S	22.69	-4.50	2.35	-4.87	4.94	-2.60	5.40	86.22	11.96	15.24
4277	171	27	21	C	S	22.69	-4.50	-0.16	1.73	4.94	0.15	-1.67	75.29	11.96	16.21
4476	176	29	20	C	S	22.69	-4.50	-2.27	-4.13	4.94	2.47	4.51	72.59	11.96	16.63
4552	178	28	22	C	S	22.69	-4.50	-4.85	2.25	4.94	4.64	-2.16	89.49	11.96	16.34
4763	181	28	23	C	S	22.69	-4.50	5.23	4.63	4.94	-4.78	-4.24	84.99	11.96	16.79
5332	188	29	24	C	S	22.69	-4.50	0.51	5.04	4.94	-0.47	-4.58	72.64	11.96	18.22
5709	193	31	23	C	S	22.69	-4.50	-1.51	-0.66	4.94	1.53	0.67	81.69	11.96	17.77
6455	203	32	25	C	S	22.69	-4.50	3.25	1.96	4.94	-3.13	-1.88	78.43	11.96	19.01
7042	210	33	26	C	S	22.69	-4.50	-0.84	2.40	4.94	0.80	-2.29	89.53	11.96	19.29

Supplement 9: geometric and energetic quantities of interest

Table S2. Summarizing the calculated geometric and energetic quantities of interest.

BL number	twist-angle (°)	γ-PC isotropic strain (%)	WS_2 isotropic strain (%)	d_{IL} (Å)	E_b (meV/Å^2)	areal atomic density (atoms/nm^2)	BL deformation energy (meV/Å^2)	e- density redistribution (e/nm^2)	$CM_{\gamma\text{-}PC}$ (Å)	CM_{WS2} (Å)
1	0	4.23	-5.03	3.26	-22.84	88.75	62.18	-0.149	0.000	0.000
2	60	4.22	-5.04	3.37	-20.67	88.76	62.21	-0.142	0.000	0.000
3	0	4.24	-5.02	3.39	-20.17	88.73	62.20	-0.134	0.000	0.000
4	60	4.14	-5.13	3.77	-15.39	88.92	62.39	-0.096	0.000	0.000
5	0	4.13	-5.14	3.76	-15.48	88.93	62.38	-0.094	0.000	0.000
6	60	4.26	-5.00	3.27	-22.62	88.70	62.15	-0.145	0.000	0.000
7	30	-1.71	3.35	3.56	-18.23	89.31	17.55	-0.078	0.008	0.008
8	90	-1.71	3.35	3.56	-18.23	89.31	17.55	-0.079	0.008	0.009
9	30	-1.70	3.35	3.56	-18.28	89.31	17.57	-0.087	0.004	0.000
10	90	-1.71	3.34	3.55	-18.28	89.32	17.57	-0.088	0.005	0.000

103	19.11	-1.17	2.12	3.55	-18.31	89.56	7.08	-0.092	0.010	0.005
104	40.89	-1.17	2.12	3.55	-18.31	89.56	7.08	-0.093	0.009	0.006
105	79.11	-1.17	2.11	3.54	-18.34	89.57	7.08	-0.093	0.009	0.007
106	100.89	-1.16	2.12	3.56	-18.33	89.55	7.07	-0.092	0.009	0.004
107	19.11	-1.18	2.11	3.54	-18.31	89.57	7.07	-0.101	0.017	0.007
108	40.89	-1.17	2.12	3.55	-18.34	89.56	7.07	-0.101	0.017	0.007
109	79.11	-1.17	2.12	3.55	-18.34	89.56	7.07	-0.101	0.017	0.007
110	100.89	-1.17	2.12	3.55	-18.33	89.55	7.07	-0.099	0.016	0.007
917	13.90	-0.42	0.73	3.55	-18.47	89.70	0.25	-0.095	0.023	0.008
918	46.10	-0.43	0.73	3.54	-18.45	89.71	0.25	-0.097	0.026	0.012
919	73.90	-0.42	0.73	3.56	-18.48	89.69	0.25	-0.095	0.026	0.010
920	106.10	-0.42	0.73	3.55	-18.47	89.70	0.25	-0.095	0.023	0.008
921	13.90	-0.42	0.73	3.55	-18.48	89.70	0.25	-0.097	0.027	0.011
922	46.10	-0.42	0.73	3.55	-18.47	89.70	0.25	-0.096	0.023	0.009
923	73.90	-0.42	0.73	3.55	-18.47	89.70	0.25	-0.096	0.023	0.009
924	106.10	-0.42	0.73	3.55	-18.48	89.70	0.25	-0.097	0.027	0.011
925	46.10	-0.43	0.72	3.54	-18.46	89.72	0.26	-0.098	0.030	0.012
926	73.90	-0.42	0.73	3.56	-18.48	89.70	0.25	-0.097	0.027	0.012
927	13.90	-0.42	0.73	3.55	-18.47	89.70	0.25	-0.096	0.024	0.010
928	106.10	-0.42	0.73	3.55	-18.47	89.70	0.25	-0.096	0.024	0.010
1223	9.52	-2.91	6.65	3.53	-18.16	88.57	61.20	-0.086	0.042	0.005
1224	22.69	-2.91	6.65	3.53	-17.98	88.56	61.14	-0.086	0.012	0.006
1231	9.52	-2.91	6.65	3.52	-18.17	88.57	61.21	-0.088	0.052	0.006
1232	22.69	-2.91	6.65	3.54	-17.98	88.56	61.14	-0.085	0.011	0.008
1239	22.69	-2.91	6.65	3.53	-17.98	88.56	61.14	-0.087	0.014	0.008
1243	9.52	-2.91	6.65	3.53	-18.17	88.56	61.21	-0.086	0.050	0.007
9897	16.34	0.14	-0.22	3.55	-18.47	89.76	0.14	-0.099	0.020	0.010
9898	25.77	0.14	-0.22	3.56	-18.41	89.76	0.12	-0.098	0.008	0.007
9905	16.34	0.14	-0.22	3.56	-18.47	89.75	0.14	-0.098	0.018	0.010
9906	25.77	0.14	-0.22	3.56	-18.41	89.76	0.12	-0.099	0.010	0.008
9913	16.34	0.14	-0.22	3.56	-18.47	89.75	0.14	-0.099	0.020	0.010
9917	25.77	0.14	-0.22	3.56	-18.41	89.76	0.12	-0.098	0.012	0.008
12825	7.59	0.13	-0.18	3.54	-18.68	89.75	0.19	-0.099	0.094	0.029
12829	7.59	0.13	-0.19	3.54	-18.67	89.75	0.19	-0.099	0.091	0.025
14455	5.69			3.53	-18.71	89.65		-0.097	0.135	0.013

Supplement 10: band structures of BL 7, 9, 103, 104, 917 and 918

The band structures of BL 7, 9, 103, 104, 917 and 918 were shown in **figure S4**. Note that two BLs for each of the studied group of systems adhering to **Eq. 10** were included. Within these groups, the bands were found near-identical from the Γ-point to the M-point and from the K-point to the Γ-point, although the states at the M -and K-point were found differently connected by the bands inbetween. This was expressed most clearly in BL 103 and BL 104. The observation does not signify the presence of a twist-angle dependency but rather a bias stemming from DFT calculation as the orientation of

the ML varies relative to the simulation box. This can be inferred, as the calculated bands of the isolated MLs were found to run alongside the BL bands.

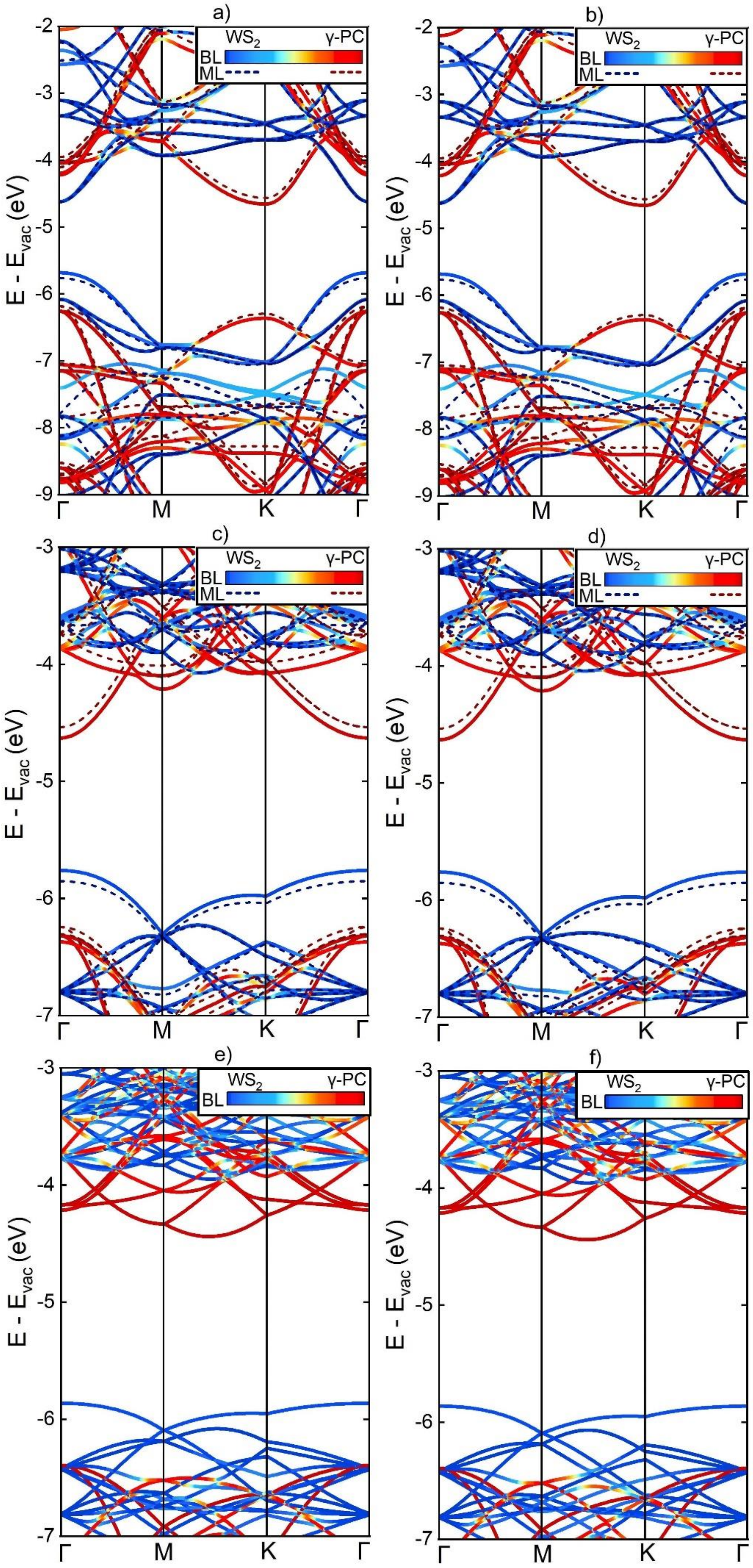
a)
b)
c)
d)
e)
f)
WS2
γ-PC
BL
ML
E - Evac (eV)
Γ
M
K
Γ
-2
-3
-4
-5
-6
-7
-8
-9

Figure S5. Illustrating from **a)** to **f)**, respectively, the colour-coded band structures of BL 7, 9, 103, 104, 917 and 918.

Supplement 11: band structures of BL 1223 and 1224

Despite the similarity of the shapes of the conduction bands, it was noticed that BL 1223 possessed shared states near the CBM of γ-PC, whilst BL 1224 did not.

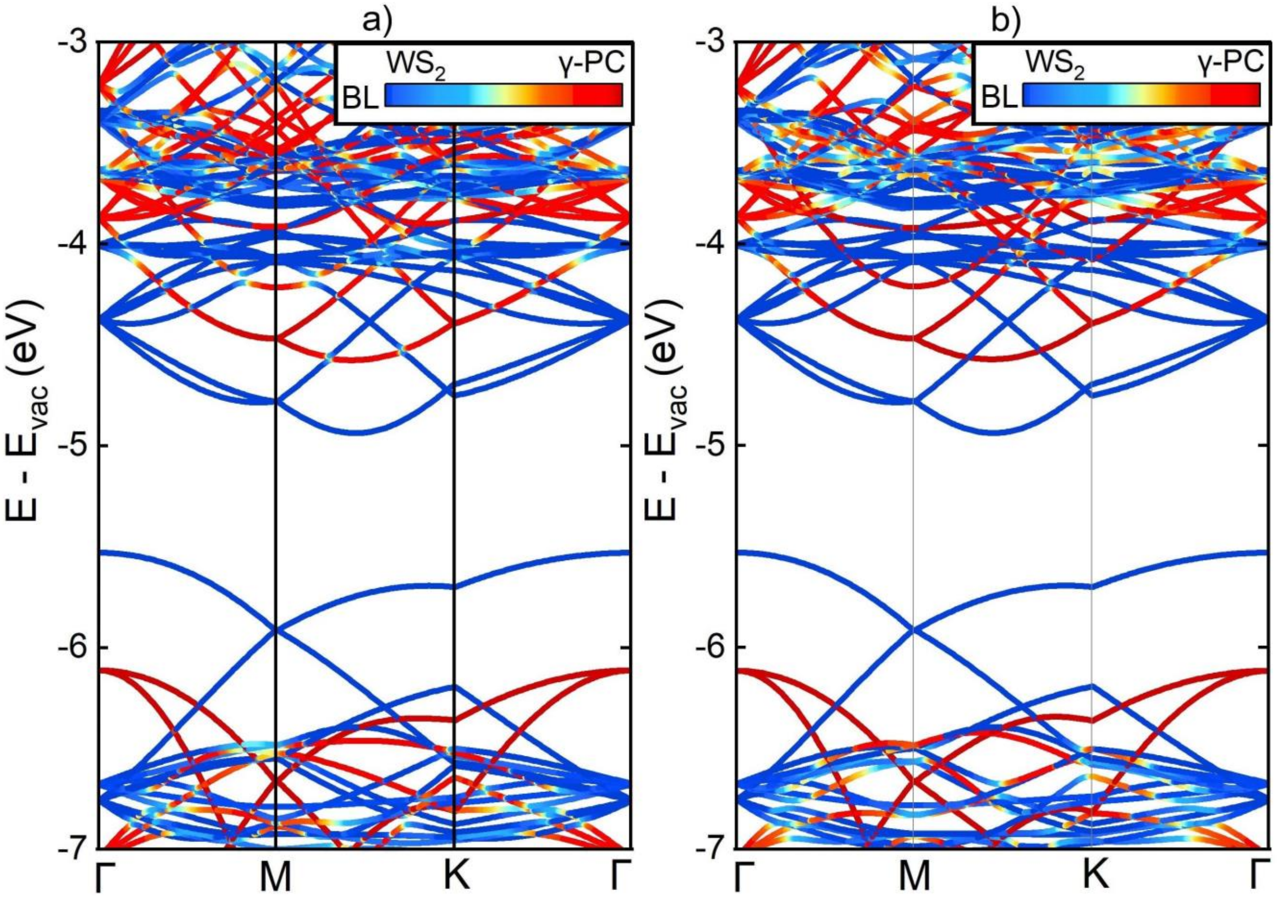

Figure S6. Showing colour-coded band structures of **a)** BL 1223 and **b)** BL 1224.

Supplement 12: band-structure-related quantities of interest

Table S3. Summarizing the calculated band-structure-related quantities of interest.

BL number	γ-PC BL VBM (eV)	γ-PC BL CBM (eV)	WS_2 BL VBM (eV)	WS_2 BL CBM (eV)	γ-PC ML VBM (eV)	γ-PC ML CBM (eV)	WS_2 ML VBM (eV)	WS_2 ML CBM (eV)	γ-PC Δ VBM (eV)	γ-PC Δ CBM (eV)	WS_2 Δ VBM (eV)	WS_2 Δ CBM (eV)	γ-PC BL BG (eV)	WS_2 BL BG (eV)	γ-PC ML BG (eV)	WS_2 ML BG (eV)	γ-PC Δ BG (eV)	WS_2 Δ BG (eV)	BL BG (eV)
1	-6.65	-4.57	-6.24	-3.93	-6.64	-4.55	-6.39	-3.94	-0.01	-0.02	0.14	0.00	2.08	2.31	2.09	2.45	-0.01	0.14	1.68
2	-6.72	-4.65	-6.31	-3.92	-6.64	-4.55	-6.39	-3.93	-0.09	-0.10	0.08	0.02	2.08	2.39	2.09	2.46	-0.01	0.06	1.66
3	-6.70	-4.61	-6.31	-3.92	-6.64	-4.55	-6.39	-3.93	-0.06	-0.06	0.08	0.02	2.09	2.39	2.09	2.46	0.00	0.07	1.70
4	-6.71	-4.63	-6.37	-3.94	-6.63	-4.54	-6.38	-3.94	-0.08	-0.09	0.02	0.00	2.09	2.43	2.09	2.45	-0.01	0.02	1.74
5	-6.71	-4.63	-6.37	-3.94	-6.63	-4.54	-6.38	-3.94	-0.08	-0.09	0.02	0.00	2.08	2.43	2.09	2.44	-0.01	0.02	1.74
6	-6.64	-4.54	-6.25	-3.94	-6.64	-4.55	-6.39	-3.93	0.01	0.01	0.14	-0.01	2.09	2.31	2.09	2.46	0.00	0.15	1.71
7	-6.26	-4.66	-5.68	-4.62	-6.18	-4.56	-5.76	-4.63	-0.08	-0.09	0.08	0.01	1.60	1.06	1.62	1.14	-0.02	0.08	1.03
8	-6.26	-4.66	-5.68	-4.62	-6.18	-4.56	-5.76	-4.63	-0.08	-0.09	0.08	0.01	1.60	1.06	1.62	1.14	-0.02	0.08	1.03
9	-6.26	-4.66	-5.68	-4.63	-6.18	-4.56	-5.76	-4.63	-0.08	-0.09	0.08	0.00	1.60	1.06	1.62	1.14	-0.02	0.08	1.03
10	-6.26	-4.66	-5.68	-4.62	-6.18	-4.56	-5.77	-4.63	-0.08	-0.09	0.08	0.00	1.60	1.06	1.62	1.14	-0.02	0.08	1.03
103	-6.32	-4.63	-5.76	-4.08	-6.24	-4.54	-5.85	-4.07	-0.08	-0.09	0.09	0.00	1.69	1.68	1.71	1.78	-0.02	0.10	1.13
104	-6.32	-4.63	-5.76	-4.05	-6.24	-4.54	-5.85	-4.04	-0.08	-0.09	0.09	-0.01	1.69	1.71	1.71	1.81	-0.02	0.10	1.13
105	-6.32	-4.63	-5.76	-4.08	-6.24	-4.54	-5.85	-4.07	-0.08	-0.09	0.09	0.00	1.69	1.68	1.71	1.78	-0.02	0.10	1.13
106	-6.32	-4.63	-5.76	-4.05	-6.24	-4.54	-5.85	-4.04	-0.08	-0.09	0.09	-0.01	1.69	1.71	1.71	1.81	-0.02	0.10	1.13
107	-6.32	-4.63	-5.76	-4.08	-6.24	-4.54	-5.85	-4.07	-0.08	-0.10	0.09	-0.01	1.69	1.68	1.71	1.78	-0.02	0.10	1.13
108	-6.32	-4.63	-5.76	-4.05	-6.24	-4.54	-5.85	-4.04	-0.08	-0.09	0.09	-0.01	1.69	1.71	1.71	1.81	-0.02	0.10	1.13
109	-6.32	-4.63	-5.76	-4.08	-6.24	-4.54	-5.85	-4.08	-0.08	-0.09	0.09	-0.01	1.69	1.68	1.71	1.78	-0.02	0.10	1.13
110	-6.32	-4.63	-5.76	-4.05	-6.25	-4.54	-5.85	-4.04	-0.08	-0.09	0.09	-0.01	1.69	1.71	1.71	1.81	-0.02	0.10	1.13
917	-6.39	-4.44	-5.86	-3.95	-6.32	-4.35	-5.97	-3.96	-0.08	-0.10	0.10	0.00	1.96	1.91	1.97	2.01	-0.02	0.10	1.42
918	-6.39	-4.44	-5.86	-3.96	-6.32	-4.35	-5.97	-3.96	-0.08	-0.10	0.10	0.00	1.95	1.90	1.97	2.00	-0.02	0.10	1.42
919	-6.39	-4.44	-5.86	-3.96	-6.32	-4.34	-5.97	-3.96	-0.08	-0.09	0.10	0.00	1.96	1.91	1.98	2.01	-0.02	0.10	1.43
920	-6.39	-4.44	-5.86	-3.96	-6.32	-4.34	-5.97	-3.96	-0.08	-0.10	0.10	0.00	1.96	1.90	1.97	2.00	-0.02	0.10	1.42
921	-6.39	-4.44	-5.86	-3.95	-6.32	-4.34	-5.97	-3.96	-0.08	-0.09	0.10	0.00	1.96	1.91	1.98	2.01	-0.02	0.10	1.42
922	-6.39	-4.44	-5.86	-3.96	-6.32	-4.35	-5.97	-3.96	-0.08	-0.09	0.10	0.00	1.96	1.90	1.97	2.00	-0.02	0.10	1.42
923	-6.39	-4.44	-5.86	-3.95	-6.32	-4.35	-5.97	-3.96	-0.08	-0.10	0.10	0.00	1.96	1.91	1.97	2.01	-0.02	0.10	1.42
924	-6.40	-4.44	-5.86	-3.96	-6.32	-4.35	-5.97	-3.96	-0.08	-0.10	0.10	0.00	1.96	1.90	1.98	2.00	-0.02	0.10	1.42
925	-6.39	-4.44	-5.86	-3.96	-6.32	-4.35	-5.97	-3.96	-0.08	-0.10	0.10	0.00	1.95	1.90	1.97	2.00	-0.02	0.10	1.42
926	-6.39	-4.44	-5.86	-3.95	-6.32	-4.35	-5.97	-3.96	-0.08	-0.09	0.10	0.00	1.96	1.91	1.97	2.01	-0.02	0.10	1.43
927	-6.40	-4.44	-5.86	-3.95	-6.32	-4.35	-5.97	-3.96	-0.08	-0.10	0.10	0.00	1.96	1.91	1.97	2.01	-0.02	0.10	1.42
928	-6.40	-4.44	-5.86	-3.96	-6.32	-4.35	-5.97	-3.96	-0.08	-0.10	0.10	0.00	1.96	1.90	1.97	2.00	-0.02	0.10	1.42
1223	-6.11	-4.58	-5.53	-4.94	-6.04	-4.48	-5.60	-4.94	-0.08	-0.10	0.06	0.01	1.54	0.59	1.56	0.65	-0.02	0.06	*Type I*
1224	-6.12	-4.57	-5.53	-4.94	-6.04	-4.48	-5.60	-4.94	-0.08	-0.09	0.06	0.01	1.54	0.59	1.56	0.65	-0.02	0.06	*Type I*
1231	-6.11	-4.58	-5.53	-4.94	-6.04	-4.48	-5.60	-4.94	-0.08	-0.10	0.06	0.01	1.54	0.59	1.56	0.65	-0.02	0.06	*Type I*
1232	-6.12	-4.57	-5.53	-4.94	-6.04	-4.48	-5.60	-4.94	-0.08	-0.09	0.06	0.01	1.54	0.59	1.56	0.65	-0.02	0.06	*Type I*
1239	-6.12	-4.57	-5.53	-4.94	-6.04	-4.48	-5.60	-4.94	-0.08	-0.09	0.06	0.01	1.54	0.59	1.56	0.65	-0.02	0.06	*Type I*
1243	-6.11	-4.58	-5.53	-4.94	-6.04	-4.48	-5.60	-4.94	-0.08	-0.10	0.06	0.01	1.54	0.59	1.56	0.65	-0.02	0.06	*Type I*
9897	-6.40	-4.50	-5.94	-3.92	-6.37	-4.40	-5.97	-3.92	-0.03	-0.09	0.03	0.01	1.90	2.03	1.97	2.05	-0.07	0.02	1.45
9898	-6.43	-4.49	-5.94	-3.94	-6.36	-4.40	-5.97	-3.92	-0.06	-0.09	0.03	-0.02	1.93	2.01	1.96	2.05	-0.03	0.04	1.45
12825	-6.44	-4.51	-5.89	-3.98	-6.36	-4.41	-5.90	-3.99	-0.09	-0.10	0.01	0.01	1.94	1.91	1.95	1.91	-0.01	0.00	1.39
