# Supplementary material for: Systematic DFT Modeling van der Waals Heterostructures from a Complete Configurational Basis Applied to γ-PC/WS2
Source: J Chem Theory Comput. 2024 Mar 6;20(6):2377–89. doi: 10.1021/acs.jctc.3c00932 (PMC10976641; doi:10.1021/acs.jctc.3c00932)
Supplement: Supplementary file 2 — ct3c00932_si_002.pdf [file ct3c00932_si_002.pdf]

BL number	Atoms	γ-PC unit cells	WS_2 unit cells	γ-PC origin atom	WS_2 origin atom	Twist-angle (°)	γ-PC strain 1 (%)	γ-PC strain 2 (%)	γ-PC strain 3 (%)	WS_2 strain 1 (%)	WS_2 strain 2 (%)	WS_2 strain 3 (%)	γ (°)	|**a**|(Å)	|**b**|(Å)
1	7	1	1	C	S	0.000	5.011	0.000	5.011	-4.554	0.000	-4.555	60.000	3.017	3.017
2	7	1	1	C	S	60.000	5.011	0.000	5.011	-4.554	0.000	-4.555	60.000	3.017	3.017
3	7	1	1	C	W	0.000	5.011	0.000	5.011	-4.554	0.000	-4.555	60.000	3.017	3.017
4	7	1	1	C	W	60.000	5.011	0.000	5.011	-4.554	0.000	-4.555	60.000	3.017	3.017
5	7	1	1	P	S	0.000	5.011	0.000	5.011	-4.554	0.000	-4.555	60.000	3.017	3.017
6	7	1	1	P	W	60.000	5.011	0.000	5.011	-4.554	0.000	-4.555	60.000	3.017	3.017
7	25	4	3	C	S	30.000	-2.359	0.000	-2.359	2.476	0.000	2.476	60.000	5.611	5.611
8	25	4	3	C	S	90.000	-2.359	0.000	-2.359	2.476	0.000	2.476	60.000	5.611	5.611
9	25	4	3	C	W	30.000	-2.359	0.000	-2.359	2.476	0.000	2.476	60.000	5.611	5.611
10	25	4	3	C	W	90.000	-2.359	0.000	-2.359	2.476	0.000	2.476	60.000	5.611	5.611
11	39	6	5	C	S	22.689	-4.497	-3.888	5.421	4.941	3.508	-4.891	65.355	8.493	5.595
12	39	6	5	C	S	37.311	-4.497	3.888	5.421	4.941	-3.508	-4.891	65.355	5.595	8.493
13	39	6	5	C	S	82.689	-4.497	-3.888	5.421	4.941	3.508	-4.891	65.355	8.493	5.595
14	39	6	5	C	S	97.311	-4.497	3.888	5.421	4.941	-3.508	-4.891	65.355	5.595	8.493
15	39	6	5	C	W	22.689	-4.497	-3.888	5.421	4.941	3.508	-4.891	65.355	8.493	5.595
16	39	6	5	C	W	37.311	-4.497	3.888	5.421	4.941	-3.508	-4.891	65.355	5.595	8.493
17	39	6	5	C	W	82.689	-4.497	-3.888	5.421	4.941	3.508	-4.891	65.355	8.493	5.595
18	39	6	5	C	W	97.311	-4.497	3.888	5.421	4.941	-3.508	-4.891	65.355	5.595	8.493
19	39	6	5	P	S	22.689	-4.497	-3.888	5.421	4.941	3.508	-4.891	65.355	8.493	5.595
20	39	6	5	P	S	37.311	-4.497	3.888	5.421	4.941	-3.508	-4.891	65.355	5.595	8.493
21	39	6	5	P	S	82.689	-4.497	-3.888	5.421	4.941	3.508	-4.891	65.355	8.493	5.595
22	39	6	5	P	S	97.311	-4.497	3.888	5.421	4.941	-3.508	-4.891	65.355	5.595	8.493
23	39	6	5	P	W	22.689	-4.497	-3.888	5.421	4.941	3.508	-4.891	65.355	8.493	5.595
24	39	6	5	P	W	37.311	-4.497	3.888	5.421	4.941	-3.508	-4.891	65.355	5.595	8.493
25	39	6	5	P	W	82.689	-4.497	-3.888	5.421	4.941	3.508	-4.891	65.355	8.493	5.595
26	39	6	5	P	W	97.311	-4.497	3.888	5.421	4.941	-3.508	-4.891	65.355	5.595	8.493
27	46	7	6	C	S	0.000	5.011	-4.331	-2.848	-4.554	4.592	3.020	87.448	3.017	16.939
28	46	7	6	C	S	4.715	-3.007	4.000	5.198	3.200	-3.623	-4.708	87.822	16.951	3.014
29	46	7	6	C	S	55.285	-3.007	-4.000	5.198	3.200	3.623	-4.708	87.822	16.951	3.014
30	46	7	6	C	S	60.000	5.011	-4.331	-2.848	-4.554	4.592	3.020	87.448	3.017	16.938
31	46	7	6	C	S	64.715	-3.007	4.000	5.198	3.200	-3.623	-4.708	87.823	16.951	3.014
32	46	7	6	C	S	115.285	-3.007	-4.000	5.198	3.200	3.623	-4.708	87.823	16.951	3.014
33	46	7	6	C	W	0.000	5.011	-4.331	-2.848	-4.554	4.592	3.020	87.448	3.017	16.939
34	46	7	6	C	W	4.715	-3.007	4.000	5.198	3.200	-3.623	-4.708	87.822	16.951	3.014
35	46	7	6	C	W	55.285	-3.007	-4.000	5.198	3.200	3.623	-4.708	87.822	16.951	3.014
36	46	7	6	C	W	60.000	5.011	-4.331	-2.848	-4.554	4.592	3.020	87.448	3.017	16.938
37	46	7	6	C	W	64.715	-3.007	4.000	5.198	3.200	-3.623	-4.708	87.823	16.951	3.014
38	46	7	6	C	W	115.285	-3.007	-4.000	5.198	3.200	3.623	-4.708	87.823	16.951	3.014
39	46	7	6	P	S	0.000	5.011	-4.331	-2.848	-4.554	4.592	3.020	87.448	3.017	16.939
40	46	7	6	P	S	4.715	-3.007	4.000	5.198	3.200	-3.623	-4.708	87.822	16.951	3.014
41	46	7	6	P	S	55.285	-3.007	-4.000	5.198	3.200	3.623	-4.708	87.822	16.951	3.014
42	46	7	6	P	S	60.000	5.011	-4.331	-2.848	-4.554	4.592	3.020	87.448	3.017	16.938
43	46	7	6	P	S	64.715	-3.007	4.000	5.198	3.200	-3.623	-4.708	87.823	16.951	3.014
44	46	7	6	P	S	115.285	-3.007	-4.000	5.198	3.200	3.623	-4.708	87.823	16.951	3.014
45	46	7	6	P	W	0.000	5.011	-4.331	-2.848	-4.554	4.592	3.020	87.448	3.017	16.939
46	46	7	6	P	W	4.715	-3.007	4.000	5.198	3.200	-3.623	-4.708	87.822	16.951	3.014
47	46	7	6	P	W	55.285	-3.007	-4.000	5.198	3.200	3.623	-4.708	87.822	16.951	3.014
48	46	7	6	P	W	60.000	5.011	-4.331	-2.848	-4.554	4.592	3.020	87.448	3.017	16.938
49	46	7	6	P	W	64.715	-3.007	4.000	5.198	3.200	-3.623	-4.708	87.823	16.951	3.014
50	46	7	6	P	W	115.285	-3.007	-4.000	5.198	3.200	3.623	-4.708	87.823	16.951	3.014
51	49	7	7	C	S	21.787	5.011	0.000	5.011	-4.554	0.000	-4.555	60.000	7.983	7.983

BL number	Atoms	γ-PC unit cells	WS_2 unit cells	γ-PC origin atom	WS_2 origin atom	Twist-angle (°)	γ-PC strain 1 (%)	γ-PC strain 2 (%)	γ-PC strain 3 (%)	WS_2 strain 1 (%)	WS_2 strain 2 (%)	WS_2 strain 3 (%)	γ (°)	|**a**|(Å)	|**b**|(Å)
52	49	7	7	C	S	38.213	5.011	0.000	5.011	-4.554	0.000	-4.555	60.000	7.983	7.983
53	49	7	7	C	S	81.787	5.011	0.000	5.011	-4.554	0.000	-4.555	60.000	7.983	7.983
54	49	7	7	C	S	98.213	5.011	0.000	5.011	-4.554	0.000	-4.555	60.000	7.983	7.983
55	49	7	7	C	W	21.787	5.011	0.000	5.011	-4.554	0.000	-4.555	60.000	7.983	7.983
56	49	7	7	C	W	38.213	5.011	0.000	5.011	-4.554	0.000	-4.555	60.000	7.983	7.983
57	49	7	7	C	W	81.787	5.011	0.000	5.011	-4.554	0.000	-4.555	60.000	7.983	7.983
58	49	7	7	C	W	98.213	5.011	0.000	5.011	-4.554	0.000	-4.555	60.000	7.983	7.983
59	49	7	7	P	S	38.213	5.011	0.000	5.011	-4.554	0.000	-4.555	60.000	7.983	7.983
60	49	7	7	P	S	81.787	5.011	0.000	5.011	-4.554	0.000	-4.555	60.000	7.983	7.983
61	49	7	7	P	W	21.787	5.011	0.000	5.011	-4.554	0.000	-4.555	60.000	7.983	7.983
62	49	7	7	P	W	98.213	5.011	0.000	5.011	-4.554	0.000	-4.555	60.000	7.983	7.983
63	53	8	7	C	S	0.000	5.011	-3.789	-1.865	-4.554	3.936	1.938	87.789	3.017	19.549
64	53	8	7	C	S	4.715	-1.702	4.602	4.825	1.762	-4.197	-4.400	87.487	19.567	3.015
65	53	8	7	C	S	27.796	5.011	-3.789	-1.865	-4.554	3.936	1.938	75.204	10.862	5.612
66	53	8	7	C	S	32.204	5.011	3.789	-1.865	-4.554	-3.936	1.938	75.204	5.612	10.862
67	53	8	7	C	S	55.285	-1.702	-4.601	4.825	1.762	4.197	-4.400	87.487	19.567	3.015
68	53	8	7	C	S	60.000	5.011	-3.789	-1.865	-4.554	3.936	1.938	87.789	3.017	19.549
69	53	8	7	C	S	64.715	-1.702	4.602	4.825	1.762	-4.197	-4.400	87.486	19.567	3.015
70	53	8	7	C	S	87.796	5.011	-3.789	-1.865	-4.554	3.936	1.938	75.204	10.862	5.612
71	53	8	7	C	S	92.204	5.011	3.789	-1.865	-4.554	-3.936	1.938	75.204	5.612	10.862
72	53	8	7	C	S	115.285	-1.702	-4.601	4.825	1.762	4.197	-4.400	87.487	19.567	3.015
73	53	8	7	C	W	0.000	5.011	-3.789	-1.865	-4.554	3.936	1.938	87.789	3.017	19.549
74	53	8	7	C	W	4.715	-1.702	4.602	4.825	1.762	-4.197	-4.400	87.487	19.567	3.015
75	53	8	7	C	W	27.796	5.011	-3.789	-1.865	-4.554	3.936	1.938	75.204	10.862	5.612
76	53	8	7	C	W	32.204	5.011	3.789	-1.865	-4.554	-3.936	1.938	75.204	5.612	10.862
77	53	8	7	C	W	55.285	-1.702	-4.601	4.825	1.762	4.197	-4.400	87.487	19.567	3.015
78	53	8	7	C	W	60.000	5.011	-3.789	-1.865	-4.554	3.936	1.938	87.789	3.017	19.549
79	53	8	7	C	W	64.715	-1.702	4.602	4.825	1.762	-4.197	-4.400	87.486	19.567	3.015
80	53	8	7	C	W	87.796	5.011	-3.789	-1.865	-4.554	3.936	1.938	75.204	10.862	5.612
81	53	8	7	C	W	92.204	5.011	3.789	-1.865	-4.554	-3.936	1.938	75.204	5.612	10.862
82	53	8	7	C	W	115.285	-1.702	-4.601	4.825	1.762	4.197	-4.400	87.487	19.567	3.015
83	53	8	7	P	S	0.000	5.011	-3.789	-1.865	-4.554	3.936	1.938	87.789	3.017	19.549
84	53	8	7	P	S	4.715	-1.702	4.602	4.825	1.762	-4.197	-4.400	87.487	19.567	3.015
85	53	8	7	P	S	27.796	5.011	-3.789	-1.865	-4.554	3.936	1.938	75.204	10.862	5.612
86	53	8	7	P	S	32.204	5.011	3.789	-1.865	-4.554	-3.936	1.938	75.204	5.612	10.862
87	53	8	7	P	S	55.285	-1.702	-4.601	4.825	1.762	4.197	-4.400	87.487	19.567	3.015
88	53	8	7	P	S	60.000	5.011	-3.789	-1.865	-4.554	3.936	1.938	87.789	3.017	19.549
89	53	8	7	P	S	64.715	-1.702	4.602	4.825	1.762	-4.197	-4.400	87.486	19.567	3.015
90	53	8	7	P	S	87.796	5.011	-3.789	-1.865	-4.554	3.936	1.938	75.204	10.862	5.612
91	53	8	7	P	S	92.204	5.011	3.789	-1.865	-4.554	-3.936	1.938	75.204	5.612	10.862
92	53	8	7	P	S	115.285	-1.702	-4.601	4.825	1.762	4.197	-4.400	87.487	19.567	3.015
93	53	8	7	P	W	0.000	5.011	-3.789	-1.865	-4.554	3.936	1.938	87.789	3.017	19.549
94	53	8	7	P	W	4.715	-1.702	4.602	4.825	1.762	-4.197	-4.400	87.487	19.567	3.015
95	53	8	7	P	W	27.796	5.011	-3.789	-1.865	-4.554	3.936	1.938	75.204	10.862	5.612
96	53	8	7	P	W	32.204	5.011	3.789	-1.865	-4.554	-3.936	1.938	75.204	5.612	10.862
97	53	8	7	P	W	55.285	-1.702	-4.601	4.825	1.762	4.197	-4.400	87.487	19.567	3.015
98	53	8	7	P	W	60.000	5.011	-3.789	-1.865	-4.554	3.936	1.938	87.789	3.017	19.549
99	53	8	7	P	W	64.715	-1.702	4.602	4.825	1.762	-4.197	-4.400	87.486	19.567	3.015
100	53	8	7	P	W	87.796	5.011	-3.789	-1.865	-4.554	3.936	1.938	75.204	10.862	5.612
101	53	8	7	P	W	92.204	5.011	3.789	-1.865	-4.554	-3.936	1.938	75.204	5.612	10.862
102	53	8	7	P	W	115.285	-1.702	-4.601	4.825	1.762	4.197	-4.400	87.487	19.567	3.015

BL number	Atoms	γ-PC unit cells	WS_2 unit cells	γ-PC origin atom	WS_2 origin atom	Twist-angle (°)	γ-PC strain 1 (%)	γ-PC strain 2 (%)	γ-PC strain 3 (%)	WS_2 strain 1 (%)	WS_2 strain 2 (%)	WS_2 strain 3 (%)	γ (°)	$\lvert\mathbf{a}\rvert$(Å)	$\lvert\mathbf{b}\rvert$(Å)
103	57	9	7	C	S	19.107	-1.485	0.000	-1.485	1.530	0.000	1.530	60.000	8.492	8.492
104	57	9	7	C	S	40.893	-1.485	0.000	-1.485	1.530	0.000	1.530	60.000	8.492	8.492
105	57	9	7	C	S	79.107	-1.485	0.000	-1.485	1.530	0.000	1.530	60.000	8.492	8.492
106	57	9	7	C	S	100.893	-1.485	0.000	-1.485	1.530	0.000	1.530	60.000	8.492	8.492
107	57	9	7	P	S	19.107	-1.485	0.000	-1.485	1.530	0.000	1.530	60.000	8.492	8.492
108	57	9	7	P	S	40.893	-1.485	0.000	-1.485	1.530	0.000	1.530	60.000	8.492	8.492
109	57	9	7	P	S	79.107	-1.485	0.000	-1.485	1.530	0.000	1.530	60.000	8.492	8.492
110	57	9	7	P	W	100.893	-1.485	0.000	-1.485	1.530	0.000	1.530	60.000	8.492	8.492
111	60	9	8	C	S	17.897	5.011	-3.368	-1.101	-4.554	3.444	1.126	51.841	7.972	10.660
112	60	9	8	C	S	19.107	-1.485	-1.354	5.446	1.530	1.221	-4.911	80.521	8.492	7.980
113	60	9	8	C	S	21.787	5.011	3.368	-1.101	-4.554	-3.444	1.126	80.336	7.983	8.491
114	60	9	8	C	S	38.213	5.011	-3.368	-1.101	-4.554	3.444	1.126	80.336	7.983	8.491
115	60	9	8	C	S	40.893	-1.485	1.354	5.446	1.530	-1.221	-4.911	80.522	8.492	7.980
116	60	9	8	C	S	42.103	5.011	3.368	-1.101	-4.554	-3.444	1.126	51.841	10.660	7.972
117	60	9	8	C	S	77.897	5.011	-3.368	-1.101	-4.554	3.444	1.126	51.841	7.972	10.660
118	60	9	8	C	S	79.107	-1.485	-1.354	5.446	1.530	1.221	-4.911	80.521	8.492	7.980
119	60	9	8	C	S	81.787	5.011	3.368	-1.101	-4.554	-3.444	1.126	80.336	7.983	8.491
120	60	9	8	C	S	98.213	5.011	-3.368	-1.101	-4.554	3.444	1.126	80.336	7.983	8.491
121	60	9	8	C	S	100.893	-1.485	1.354	5.446	1.530	-1.221	-4.911	80.521	8.492	7.980
122	60	9	8	C	S	102.104	5.011	3.368	-1.101	-4.554	-3.444	1.126	51.841	10.660	7.972
123	60	9	8	C	W	17.897	5.011	-3.368	-1.101	-4.554	3.444	1.126	51.841	7.972	10.660
124	60	9	8	C	W	19.107	-1.485	-1.354	5.446	1.530	1.221	-4.911	80.521	8.492	7.980
125	60	9	8	C	W	21.787	5.011	3.368	-1.101	-4.554	-3.444	1.126	80.336	7.983	8.491
126	60	9	8	C	W	38.213	5.011	-3.368	-1.101	-4.554	3.444	1.126	80.336	7.983	8.491
127	60	9	8	C	W	40.893	-1.485	1.354	5.446	1.530	-1.221	-4.911	80.522	8.492	7.980
128	60	9	8	C	W	42.103	5.011	3.368	-1.101	-4.554	-3.444	1.126	51.841	10.660	7.972
129	60	9	8	C	W	77.897	5.011	-3.368	-1.101	-4.554	3.444	1.126	51.841	7.972	10.660
130	60	9	8	C	W	79.107	-1.485	-1.354	5.446	1.530	1.221	-4.911	80.521	8.492	7.980
131	60	9	8	C	W	81.787	5.011	3.368	-1.101	-4.554	-3.444	1.126	80.336	7.983	8.491
132	60	9	8	C	W	98.213	5.011	-3.368	-1.101	-4.554	3.444	1.126	80.336	7.983	8.491
133	60	9	8	C	W	100.893	-1.485	1.354	5.446	1.530	-1.221	-4.911	80.521	8.492	7.980
134	60	9	8	C	W	102.104	5.011	3.368	-1.101	-4.554	-3.444	1.126	51.841	10.660	7.972
135	60	9	8	P	S	17.897	5.011	-3.368	-1.101	-4.554	3.444	1.126	51.841	7.972	10.660
136	60	9	8	P	S	19.107	-1.485	-1.354	5.446	1.530	1.221	-4.911	80.521	8.492	7.980
137	60	9	8	P	S	21.787	5.011	3.368	-1.101	-4.554	-3.444	1.126	80.336	7.983	8.491
138	60	9	8	P	S	38.213	5.011	-3.368	-1.101	-4.554	3.444	1.126	80.336	7.983	8.491
139	60	9	8	P	S	40.893	-1.485	1.354	5.446	1.530	-1.221	-4.911	80.522	8.492	7.980
140	60	9	8	P	S	42.103	5.011	3.368	-1.101	-4.554	-3.444	1.126	51.841	10.660	7.972
141	60	9	8	P	S	77.897	5.011	-3.368	-1.101	-4.554	3.444	1.126	51.841	7.972	10.660
142	60	9	8	P	S	79.107	-1.485	-1.354	5.446	1.530	1.221	-4.911	80.521	8.492	7.980
143	60	9	8	P	S	81.787	5.011	3.368	-1.101	-4.554	-3.444	1.126	80.336	7.983	8.491
144	60	9	8	P	S	98.213	5.011	-3.368	-1.101	-4.554	3.444	1.126	80.336	7.983	8.491
145	60	9	8	P	S	100.893	-1.485	1.354	5.446	1.530	-1.221	-4.911	80.521	8.492	7.980
146	60	9	8	P	S	102.104	5.011	3.368	-1.101	-4.554	-3.444	1.126	51.841	10.660	7.972
147	60	9	8	P	W	17.897	5.011	-3.368	-1.101	-4.554	3.444	1.126	51.841	7.972	10.660
148	60	9	8	P	W	19.107	-1.485	-1.354	5.446	1.530	1.221	-4.911	80.521	8.492	7.980
149	60	9	8	P	W	21.787	5.011	3.368	-1.101	-4.554	-3.444	1.126	80.336	7.983	8.491
150	60	9	8	P	W	38.213	5.011	-3.368	-1.101	-4.554	3.444	1.126	80.336	7.983	8.491
151	60	9	8	P	W	40.893	-1.485	1.354	5.446	1.530	-1.221	-4.911	80.522	8.492	7.980
152	60	9	8	P	W	42.103	5.011	3.368	-1.101	-4.554	-3.444	1.126	51.841	10.660	7.972
153	60	9	8	P	W	77.897	5.011	-3.368	-1.101	-4.554	3.444	1.126	51.841	7.972	10.660

BL number	Atoms	γ-PC unit cells	WS_2 unit cells	γ-PC origin atom	WS_2 origin atom	Twist-angle (°)	γ-PC strain 1 (%)	γ-PC strain 2 (%)	γ-PC strain 3 (%)	WS_2 strain 1 (%)	WS_2 strain 2 (%)	WS_2 strain 3 (%)	γ (°)	|**a**|(Å)	|**b**|(Å)
154	60	9	8	P	W	79.107	-1.485	-1.354	5.446	1.530	1.221	-4.911	80.521	8.492	7.980
155	60	9	8	P	W	81.787	5.011	3.368	-1.101	-4.554	-3.444	1.126	80.336	7.983	8.491
156	60	9	8	P	W	98.213	5.011	-3.368	-1.101	-4.554	3.444	1.126	80.336	7.983	8.491
157	60	9	8	P	W	100.893	-1.485	1.354	5.446	1.530	-1.221	-4.911	80.521	8.492	7.980
158	60	9	8	P	W	102.104	5.011	3.368	-1.101	-4.554	-3.444	1.126	51.841	10.660	7.972
159	64	10	8	C	S	24.791	-3.388	-3.586	1.939	3.635	3.452	-1.867	63.247	14.076	5.602
160	64	10	8	C	S	25.693	2.326	-1.866	-3.733	-2.223	2.016	4.034	86.642	5.606	12.584
161	64	10	8	C	S	28.055	2.281	2.109	-3.694	-2.182	-2.277	3.988	68.723	5.611	13.468
162	64	10	8	C	S	31.945	2.281	-2.109	-3.694	-2.182	2.277	3.988	68.723	13.468	5.611
163	64	10	8	C	S	34.307	2.326	1.866	-3.733	-2.223	-2.016	4.034	86.642	12.584	5.606
164	64	10	8	C	S	35.209	-3.388	3.586	1.939	3.635	-3.452	-1.867	63.247	5.602	14.076
165	64	10	8	C	S	84.791	-3.388	-3.586	1.939	3.635	3.452	-1.867	63.247	14.076	5.602
166	64	10	8	C	S	85.694	2.326	-1.866	-3.733	-2.223	2.016	4.034	86.642	5.606	12.584
167	64	10	8	C	S	88.055	2.281	2.109	-3.694	-2.182	-2.277	3.988	68.723	5.611	13.468
168	64	10	8	C	S	91.945	2.281	-2.109	-3.694	-2.182	2.277	3.988	68.723	13.468	5.611
169	64	10	8	C	S	94.307	2.326	1.866	-3.733	-2.223	-2.016	4.034	86.642	12.584	5.606
170	64	10	8	C	S	95.209	-3.388	3.586	1.939	3.635	-3.452	-1.867	63.247	5.602	14.076
171	64	10	8	C	W	24.791	-3.388	-3.586	1.939	3.635	3.452	-1.867	63.247	14.076	5.602
172	64	10	8	C	W	25.693	2.326	-1.866	-3.733	-2.223	2.016	4.034	86.642	5.606	12.584
173	64	10	8	C	W	28.055	2.281	2.109	-3.694	-2.182	-2.277	3.988	68.723	5.611	13.468
174	64	10	8	C	W	31.945	2.281	-2.109	-3.694	-2.182	2.277	3.988	68.723	13.468	5.611
175	64	10	8	C	W	34.307	2.326	1.866	-3.733	-2.223	-2.016	4.034	86.642	12.584	5.606
176	64	10	8	C	W	35.209	-3.388	3.586	1.939	3.635	-3.452	-1.867	63.247	5.602	14.076
177	64	10	8	C	W	84.791	-3.388	-3.586	1.939	3.635	3.452	-1.867	63.247	14.076	5.602
178	64	10	8	C	W	85.694	2.326	-1.866	-3.733	-2.223	2.016	4.034	86.642	5.606	12.584
179	64	10	8	C	W	88.055	2.281	2.109	-3.694	-2.182	-2.277	3.988	68.723	5.611	13.468
180	64	10	8	C	W	91.945	2.281	-2.109	-3.694	-2.182	2.277	3.988	68.723	13.468	5.611
181	64	10	8	C	W	94.307	2.326	1.866	-3.733	-2.223	-2.016	4.034	86.642	12.584	5.606
182	64	10	8	C	W	95.209	-3.388	3.586	1.939	3.635	-3.452	-1.867	63.247	5.602	14.076
183	64	10	8	P	S	24.791	-3.388	-3.586	1.939	3.635	3.452	-1.867	63.247	14.076	5.602
184	64	10	8	P	S	25.693	2.326	-1.866	-3.733	-2.223	2.016	4.034	86.642	5.606	12.584
185	64	10	8	P	S	28.055	2.281	2.109	-3.694	-2.182	-2.277	3.988	68.723	5.611	13.468
186	64	10	8	P	S	31.945	2.281	-2.109	-3.694	-2.182	2.277	3.988	68.723	13.468	5.611
187	64	10	8	P	S	34.307	2.326	1.866	-3.733	-2.223	-2.016	4.034	86.642	12.584	5.606
188	64	10	8	P	S	35.209	-3.388	3.586	1.939	3.635	-3.452	-1.867	63.247	5.602	14.076
189	64	10	8	P	S	84.791	-3.388	-3.586	1.939	3.635	3.452	-1.867	63.247	14.076	5.602
190	64	10	8	P	S	85.694	2.326	-1.866	-3.733	-2.223	2.016	4.034	86.642	5.606	12.584
191	64	10	8	P	S	88.055	2.281	2.109	-3.694	-2.182	-2.277	3.988	68.723	5.611	13.468
192	64	10	8	P	S	91.945	2.281	-2.109	-3.694	-2.182	2.277	3.988	68.723	13.468	5.611
193	64	10	8	P	S	94.307	2.326	1.866	-3.733	-2.223	-2.016	4.034	86.642	12.584	5.606
194	64	10	8	P	S	95.209	-3.388	3.586	1.939	3.635	-3.452	-1.867	63.247	5.602	14.076
195	64	10	8	P	W	24.791	-3.388	-3.586	1.939	3.635	3.452	-1.867	63.247	14.076	5.602
196	64	10	8	P	W	25.693	2.326	-1.866	-3.733	-2.223	2.016	4.034	86.642	5.606	12.584
197	64	10	8	P	W	28.055	2.281	2.109	-3.694	-2.182	-2.277	3.988	68.723	5.611	13.468
198	64	10	8	P	W	31.945	2.281	-2.109	-3.694	-2.182	2.277	3.988	68.723	13.468	5.611
199	64	10	8	P	W	34.307	2.326	1.866	-3.733	-2.223	-2.016	4.034	86.642	12.584	5.606
200	64	10	8	P	W	35.209	-3.388	3.586	1.939	3.635	-3.452	-1.867	63.247	5.602	14.076
201	64	10	8	P	W	84.791	-3.388	-3.586	1.939	3.635	3.452	-1.867	63.247	14.076	5.602
202	64	10	8	P	W	85.694	2.326	-1.866	-3.733	-2.223	2.016	4.034	86.642	5.606	12.584
203	64	10	8	P	W	88.055	2.281	2.109	-3.694	-2.182	-2.277	3.988	68.723	5.611	13.468
204	64	10	8	P	W	91.945	2.281	-2.109	-3.694	-2.182	2.277	3.988	68.723	13.468	5.611

BL number	Atoms	γ-PC unit cells	WS_2 unit cells	γ-PC origin atom	WS_2 origin atom	Twist-angle (°)	γ-PC strain 1 (%)	γ-PC strain 2 (%)	γ-PC strain 3 (%)	WS_2 strain 1 (%)	WS_2 strain 2 (%)	WS_2 strain 3 (%)	γ (°)	|**a**|(Å)	|**b**|(Å)
205	64	10	8	P	W	94.307	2.326	1.866	-3.733	-2.223	-2.016	4.034	86.642	12.584	5.606
206	64	10	8	P	W	95.209	-3.388	3.586	1.939	3.635	-3.452	-1.867	63.247	5.602	14.076
207	71	11	9	C	S	1.945	-4.723	3.572	4.685	5.216	-3.266	-4.284	80.305	15.225	5.227
208	71	11	9	C	S	58.055	-4.723	-3.572	4.685	5.216	3.266	-4.284	80.305	5.227	15.225
209	71	11	9	C	S	61.945	-4.723	3.572	4.685	5.216	-3.266	-4.284	80.305	15.225	5.227
210	71	11	9	C	S	118.055	-4.723	-3.572	4.685	5.216	3.266	-4.284	80.305	5.227	15.225
211	71	11	9	C	W	1.945	-4.723	3.572	4.685	5.216	-3.266	-4.284	80.305	15.225	5.227
212	71	11	9	C	W	58.055	-4.723	-3.572	4.685	5.216	3.266	-4.284	80.305	5.227	15.225
213	71	11	9	C	W	61.945	-4.723	3.572	4.685	5.216	-3.266	-4.284	80.305	15.225	5.227
214	71	11	9	C	W	118.055	-4.723	-3.572	4.685	5.216	3.266	-4.284	80.305	5.227	15.225
215	71	11	9	P	S	1.945	-4.723	3.572	4.685	5.216	-3.266	-4.284	80.305	15.225	5.227
216	71	11	9	P	S	118.055	-4.723	-3.572	4.685	5.216	3.266	-4.284	80.305	5.227	15.225
217	71	11	9	P	W	58.055	-4.723	-3.572	4.685	5.216	3.266	-4.284	80.305	5.227	15.225
218	71	11	9	P	W	61.945	-4.723	3.572	4.685	5.216	-3.266	-4.284	80.305	15.225	5.227
219	75	12	9	C	S	13.898	-4.228	-3.840	-0.414	4.619	3.872	0.417	76.555	9.921	8.479
220	75	12	9	C	S	16.337	0.353	0.389	-4.926	-0.351	-0.432	5.464	57.422	8.489	11.442
221	75	12	9	C	S	17.480	-4.685	2.414	0.086	5.169	-2.409	-0.085	76.403	9.911	8.496
222	75	12	9	C	S	19.107	-1.485	5.078	-3.218	1.530	-5.428	3.439	76.430	8.492	9.909
223	75	12	9	C	S	40.893	-1.485	-5.078	-3.218	1.530	5.427	3.439	76.430	8.492	9.909
224	75	12	9	C	S	42.520	-4.685	-2.414	0.086	5.169	2.409	-0.085	76.403	8.496	9.911
225	75	12	9	C	S	43.663	0.353	-0.389	-4.926	-0.351	0.432	5.464	57.422	11.442	8.489
226	75	12	9	C	S	46.102	-4.228	3.840	-0.414	4.619	-3.872	0.417	76.555	9.921	8.479
227	75	12	9	C	S	73.898	-0.414	-3.686	-4.228	0.417	4.026	4.619	76.465	8.483	9.921
228	75	12	9	C	S	76.337	0.353	0.389	-4.926	-0.351	-0.432	5.464	57.422	8.489	11.442
229	75	12	9	C	S	77.480	-4.685	2.414	0.086	5.169	-2.409	-0.085	76.403	9.911	8.496
230	75	12	9	C	S	79.107	-1.485	5.078	-3.218	1.530	-5.428	3.439	76.430	8.492	9.909
231	75	12	9	C	S	100.893	-1.485	-5.078	-3.218	1.530	5.427	3.439	76.430	8.492	9.909
232	75	12	9	C	S	102.520	-4.685	-2.414	0.086	5.169	2.409	-0.085	76.403	8.496	9.911
233	75	12	9	C	S	103.663	0.353	-0.389	-4.926	-0.351	0.432	5.464	57.422	11.442	8.489
234	75	12	9	C	S	106.102	-0.414	3.686	-4.228	0.417	-4.026	4.619	76.465	9.921	8.483
235	75	12	9	C	W	13.898	-4.228	-3.840	-0.414	4.619	3.872	0.417	76.555	9.921	8.479
236	75	12	9	C	W	16.337	0.353	0.389	-4.926	-0.351	-0.432	5.464	57.422	8.489	11.442
237	75	12	9	C	W	17.480	-4.685	2.414	0.086	5.169	-2.409	-0.085	76.403	9.911	8.496
238	75	12	9	C	W	19.107	-1.485	5.078	-3.218	1.530	-5.428	3.439	76.430	8.492	9.909
239	75	12	9	C	W	40.893	-1.485	-5.078	-3.218	1.530	5.427	3.439	76.430	8.492	9.909
240	75	12	9	C	W	42.520	-4.685	-2.414	0.086	5.169	2.409	-0.085	76.403	8.496	9.911
241	75	12	9	C	W	43.663	0.353	-0.389	-4.926	-0.351	0.432	5.464	57.422	11.442	8.489
242	75	12	9	C	W	46.102	-4.228	3.840	-0.414	4.619	-3.872	0.417	76.555	9.921	8.479
243	75	12	9	C	W	73.898	-0.414	-3.686	-4.228	0.417	4.026	4.619	76.465	8.483	9.921
244	75	12	9	C	W	76.337	0.353	0.389	-4.926	-0.351	-0.432	5.464	57.422	8.489	11.442
245	75	12	9	C	W	77.480	-4.685	2.414	0.086	5.169	-2.409	-0.085	76.403	9.911	8.496
246	75	12	9	C	W	79.107	-1.485	5.078	-3.218	1.530	-5.428	3.439	76.430	8.492	9.909
247	75	12	9	C	W	100.893	-1.485	-5.078	-3.218	1.530	5.427	3.439	76.430	8.492	9.909
248	75	12	9	C	W	102.520	-4.685	-2.414	0.086	5.169	2.409	-0.085	76.403	8.496	9.911
249	75	12	9	C	W	103.663	0.353	-0.389	-4.926	-0.351	0.432	5.464	57.422	11.442	8.489
250	75	12	9	C	W	106.102	-0.414	3.686	-4.228	0.417	-4.026	4.619	76.465	9.921	8.483
251	75	12	9	P	S	13.898	-4.228	-3.840	-0.414	4.619	3.872	0.417	76.555	9.921	8.479
252	75	12	9	P	S	16.337	0.353	0.389	-4.926	-0.351	-0.432	5.464	57.422	8.489	11.442
253	75	12	9	P	S	17.480	-4.685	2.414	0.086	5.169	-2.409	-0.085	76.403	9.911	8.496
254	75	12	9	P	S	19.107	-1.485	5.078	-3.218	1.530	-5.428	3.439	76.430	8.492	9.909
255	75	12	9	P	S	40.893	-1.485	-5.078	-3.218	1.530	5.427	3.439	76.430	8.492	9.909

BL number	Atoms	γ-PC unit cells	WS_2 unit cells	γ-PC origin atom	WS_2 origin atom	Twist-angle (°)	γ-PC strain 1 (%)	γ-PC strain 2 (%)	γ-PC strain 3 (%)	WS_2 strain 1 (%)	WS_2 strain 2 (%)	WS_2 strain 3 (%)	γ (°)	|**a**|(Å)	|**b**|(Å)
256	75	12	9	P	S	42.520	-4.685	-2.414	0.086	5.169	2.409	-0.085	76.403	8.496	9.911
257	75	12	9	P	S	43.663	0.353	-0.389	-4.926	-0.351	0.432	5.464	57.422	11.442	8.489
258	75	12	9	P	S	46.102	-4.228	3.840	-0.414	4.619	-3.872	0.417	76.555	9.921	8.479
259	75	12	9	P	S	73.898	-0.414	-3.686	-4.228	0.417	4.026	4.619	76.465	8.483	9.921
260	75	12	9	P	S	76.337	0.353	0.389	-4.926	-0.351	-0.432	5.464	57.422	8.489	11.442
261	75	12	9	P	S	77.480	-4.685	2.414	0.086	5.169	-2.409	-0.085	76.403	9.911	8.496
262	75	12	9	P	S	79.107	-1.485	5.078	-3.218	1.530	-5.428	3.439	76.430	8.492	9.909
263	75	12	9	P	S	100.893	-1.485	-5.078	-3.218	1.530	5.427	3.439	76.430	8.492	9.909
264	75	12	9	P	S	102.520	-4.685	-2.414	0.086	5.169	2.409	-0.085	76.403	8.496	9.911
265	75	12	9	P	S	103.663	0.353	-0.389	-4.926	-0.351	0.432	5.464	57.422	11.442	8.489
266	75	12	9	P	S	106.102	-0.414	3.686	-4.228	0.417	-4.026	4.619	76.465	9.921	8.483
267	75	12	9	P	W	13.898	-4.228	-3.840	-0.414	4.619	3.872	0.417	76.555	9.921	8.479
268	75	12	9	P	W	16.337	0.353	0.389	-4.926	-0.351	-0.432	5.464	57.422	8.489	11.442
269	75	12	9	P	W	17.480	-4.685	2.414	0.086	5.169	-2.409	-0.085	76.403	9.911	8.496
270	75	12	9	P	W	19.107	-1.485	5.078	-3.218	1.530	-5.428	3.439	76.430	8.492	9.909
271	75	12	9	P	W	40.893	-1.485	-5.078	-3.218	1.530	5.427	3.439	76.430	8.492	9.909
272	75	12	9	P	W	42.520	-4.685	-2.414	0.086	5.169	2.409	-0.085	76.403	8.496	9.911
273	75	12	9	P	W	43.663	0.353	-0.389	-4.926	-0.351	0.432	5.464	57.422	11.442	8.489
274	75	12	9	P	W	46.102	-4.228	3.840	-0.414	4.619	-3.872	0.417	76.555	9.921	8.479
275	75	12	9	P	W	73.898	-0.414	-3.686	-4.228	0.417	4.026	4.619	76.465	8.483	9.921
276	75	12	9	P	W	76.337	0.353	0.389	-4.926	-0.351	-0.432	5.464	57.422	8.489	11.442
277	75	12	9	P	W	77.480	-4.685	2.414	0.086	5.169	-2.409	-0.085	76.403	9.911	8.496
278	75	12	9	P	W	79.107	-1.485	5.078	-3.218	1.530	-5.428	3.439	76.430	8.492	9.909
279	75	12	9	P	W	100.893	-1.485	-5.078	-3.218	1.530	5.427	3.439	76.430	8.492	9.909
280	75	12	9	P	W	102.520	-4.685	-2.414	0.086	5.169	2.409	-0.085	76.403	8.496	9.911
281	75	12	9	P	W	103.663	0.353	-0.389	-4.926	-0.351	0.432	5.464	57.422	11.442	8.489
282	75	12	9	P	W	106.102	-0.414	3.686	-4.228	0.417	-4.026	4.619	76.465	9.921	8.483
283	78	12	10	C	S	0.000	5.011	0.000	-4.158	-4.554	0.000	4.535	90.000	5.226	16.522
284	78	12	10	C	S	30.000	2.934	0.000	-2.359	-2.772	0.000	2.476	90.000	15.368	5.611
285	78	12	10	C	S	60.000	5.011	0.000	-4.158	-4.554	0.000	4.535	90.000	3.017	28.618
286	78	12	10	C	S	90.000	2.934	0.000	-2.359	-2.772	0.000	2.476	90.000	15.368	5.611
287	78	12	10	C	W	0.000	5.011	0.000	-4.158	-4.554	0.000	4.535	90.000	5.226	16.522
288	78	12	10	C	W	30.000	2.934	0.000	-2.359	-2.772	0.000	2.476	90.000	15.368	5.611
289	78	12	10	C	W	60.000	5.011	0.000	-4.158	-4.554	0.000	4.535	90.000	5.226	16.522
290	78	12	10	C	W	90.000	2.934	0.000	-2.359	-2.772	0.000	2.476	90.000	15.368	5.611
291	78	12	10	P	S	0.000	5.011	0.000	-4.158	-4.554	0.000	4.535	90.000	5.226	16.522
292	78	12	10	P	S	30.000	2.934	0.000	-2.359	-2.772	0.000	2.476	90.000	15.368	5.611
293	78	12	10	P	S	60.000	5.011	0.000	-4.158	-4.554	0.000	4.535	90.000	3.017	28.618
294	78	12	10	P	S	90.000	-2.359	0.000	2.934	2.476	0.000	-2.772	90.000	5.611	15.368
295	78	12	10	P	W	30.000	2.934	0.000	-2.359	-2.772	0.000	2.476	90.000	15.368	5.611
296	78	12	10	P	W	60.000	5.011	0.000	-4.158	-4.554	0.000	4.535	90.000	5.226	16.522
297	78	12	10	P	W	90.000	-2.359	0.000	2.934	2.476	0.000	-2.772	90.000	5.611	15.368
298	78	12	10	C	S	3.004	-3.388	5.478	4.103	3.635	-5.063	-3.792	89.777	16.508	5.227
299	78	12	10	C	S	13.898	-0.414	-5.160	0.858	0.417	5.073	-0.843	72.462	10.654	8.482
300	78	12	10	C	S	15.608	-2.145	-2.186	2.697	2.241	2.074	-2.559	62.579	11.446	8.486
301	78	12	10	C	S	16.102	2.853	-1.237	-2.286	-2.699	1.297	2.395	72.437	10.655	8.488
302	78	12	10	C	S	19.107	1.980	3.925	-1.485	-1.905	-4.045	1.530	72.347	8.495	10.648
303	78	12	10	C	S	27.126	1.029	-4.992	-0.580	-1.008	5.051	0.587	69.859	16.359	5.610
304	78	12	10	C	S	28.055	2.281	-3.339	-1.764	-2.182	3.461	1.829	89.916	15.362	5.611
305	78	12	10	C	S	31.945	2.281	3.339	-1.764	-2.182	-3.461	1.829	89.916	5.611	15.362
306	78	12	10	C	S	32.874	1.029	4.992	-0.580	-1.008	-5.051	0.587	69.859	5.610	16.359

BL number	Atoms	γ-PC unit cells	WS_2 unit cells	γ-PC origin atom	WS_2 origin atom	Twist-angle (°)	γ-PC strain 1 (%)	γ-PC strain 2 (%)	γ-PC strain 3 (%)	WS_2 strain 1 (%)	WS_2 strain 2 (%)	WS_2 strain 3 (%)	γ (°)	$\lvert\mathbf{a}\rvert$(Å)	$\lvert\mathbf{b}\rvert$(Å)
307	78	12	10	C	S	40.893	1.980	-3.925	-1.485	-1.905	4.045	1.530	72.347	10.648	8.495
308	78	12	10	C	S	43.898	2.853	1.237	-2.286	-2.699	-1.297	2.395	72.437	10.655	8.488
309	78	12	10	C	S	44.392	-2.145	2.186	2.697	2.241	-2.074	-2.559	62.579	8.486	11.446
310	78	12	10	C	S	46.102	-0.414	5.160	0.858	0.417	-5.073	-0.843	72.462	8.482	10.654
311	78	12	10	C	S	56.996	-3.388	-5.478	4.103	3.635	5.063	-3.792	89.777	5.227	16.508
312	78	12	10	C	S	63.005	-3.388	5.478	4.103	3.635	-5.063	-3.792	89.777	16.508	5.227
313	78	12	10	C	S	73.898	-0.414	-5.160	0.858	0.417	5.073	-0.843	72.462	10.654	8.482
314	78	12	10	C	S	75.609	-2.145	-2.186	2.697	2.241	2.074	-2.559	62.579	11.446	8.486
315	78	12	10	C	S	76.102	2.853	-1.237	-2.286	-2.699	1.297	2.395	72.437	10.655	8.488
316	78	12	10	C	S	79.107	-1.485	4.063	1.980	1.530	-3.908	-1.905	72.323	8.492	10.653
317	78	12	10	C	S	87.126	1.029	-4.992	-0.580	-1.008	5.051	0.587	69.859	16.359	5.610
318	78	12	10	C	S	88.055	2.281	-3.339	-1.764	-2.182	3.461	1.829	89.916	15.362	5.611
319	78	12	10	C	S	91.945	2.281	3.339	-1.764	-2.182	-3.461	1.829	89.916	5.611	15.362
320	78	12	10	C	S	92.874	1.029	4.992	-0.580	-1.008	-5.051	0.587	69.859	5.610	16.359
321	78	12	10	C	S	100.893	-1.485	-4.063	1.980	1.530	3.908	-1.905	72.323	8.492	10.653
322	78	12	10	C	S	103.898	2.853	1.237	-2.286	-2.699	-1.297	2.395	72.437	10.655	8.488
323	78	12	10	C	S	104.392	-2.145	2.186	2.697	2.241	-2.074	-2.559	62.579	8.486	11.446
324	78	12	10	C	S	106.102	-0.414	5.160	0.858	0.417	-5.073	-0.843	72.462	8.482	10.654
325	78	12	10	C	S	116.996	-3.388	-5.478	4.103	3.635	5.063	-3.792	89.777	5.227	16.508
326	78	12	10	C	W	3.004	-3.388	5.478	4.103	3.635	-5.063	-3.792	89.777	16.508	5.227
327	78	12	10	C	W	13.898	-0.414	-5.160	0.858	0.417	5.073	-0.843	72.462	10.654	8.482
328	78	12	10	C	W	15.608	-2.145	-2.186	2.697	2.241	2.074	-2.559	62.579	11.446	8.486
329	78	12	10	C	W	16.102	2.853	-1.237	-2.286	-2.699	1.297	2.395	72.437	10.655	8.488
330	78	12	10	C	W	19.107	1.980	3.925	-1.485	-1.905	-4.045	1.530	72.347	8.495	10.648
331	78	12	10	C	W	27.126	1.029	-4.992	-0.580	-1.008	5.051	0.587	69.859	16.359	5.610
332	78	12	10	C	W	28.055	2.281	-3.339	-1.764	-2.182	3.461	1.829	89.916	15.362	5.611
333	78	12	10	C	W	31.945	2.281	3.339	-1.764	-2.182	-3.461	1.829	89.916	5.611	15.362
334	78	12	10	C	W	32.874	1.029	4.992	-0.580	-1.008	-5.051	0.587	69.859	5.610	16.359
335	78	12	10	C	W	40.893	1.980	-3.925	-1.485	-1.905	4.045	1.530	72.347	10.648	8.495
336	78	12	10	C	W	43.898	2.853	1.237	-2.286	-2.699	-1.297	2.395	72.437	10.655	8.488
337	78	12	10	C	W	44.392	-2.145	2.186	2.697	2.241	-2.074	-2.559	62.579	8.486	11.446
338	78	12	10	C	W	46.102	-0.414	5.160	0.858	0.417	-5.073	-0.843	72.462	8.482	10.654
339	78	12	10	C	W	56.996	-3.388	-5.478	4.103	3.635	5.063	-3.792	89.777	5.227	16.508
340	78	12	10	C	W	63.005	-3.388	5.478	4.103	3.635	-5.063	-3.792	89.777	16.508	5.227
341	78	12	10	C	W	73.898	-0.414	-5.160	0.858	0.417	5.073	-0.843	72.462	10.654	8.482
342	78	12	10	C	W	75.609	-2.145	-2.186	2.697	2.241	2.074	-2.559	62.579	11.446	8.486
343	78	12	10	C	W	76.102	2.853	-1.237	-2.286	-2.699	1.297	2.395	72.437	10.655	8.488
344	78	12	10	C	W	79.107	-1.485	4.063	1.980	1.530	-3.908	-1.905	72.323	8.492	10.653
345	78	12	10	C	W	87.126	1.029	-4.992	-0.580	-1.008	5.051	0.587	69.859	16.359	5.610
346	78	12	10	C	W	88.055	2.281	-3.339	-1.764	-2.182	3.461	1.829	89.916	15.362	5.611
347	78	12	10	C	W	91.945	2.281	3.339	-1.764	-2.182	-3.461	1.829	89.916	5.611	15.362
348	78	12	10	C	W	92.874	1.029	4.992	-0.580	-1.008	-5.051	0.587	69.859	5.610	16.359
349	78	12	10	C	W	100.893	-1.485	-4.063	1.980	1.530	3.908	-1.905	72.323	8.492	10.653
350	78	12	10	C	W	103.898	2.853	1.237	-2.286	-2.699	-1.297	2.395	72.437	10.655	8.488
351	78	12	10	C	W	104.392	-2.145	2.186	2.697	2.241	-2.074	-2.559	62.579	8.486	11.446
352	78	12	10	C	W	106.102	-0.414	5.160	0.858	0.417	-5.073	-0.843	72.462	8.482	10.654
353	78	12	10	C	W	116.996	-3.388	-5.478	4.103	3.635	5.063	-3.792	89.777	5.227	16.508
354	78	12	10	P	S	3.004	-3.388	5.478	4.103	3.635	-5.063	-3.792	89.777	16.508	5.227
355	78	12	10	P	S	13.898	-0.414	-5.160	0.858	0.417	5.073	-0.843	72.462	10.654	8.482
356	78	12	10	P	S	15.608	-2.145	-2.186	2.697	2.241	2.074	-2.559	62.579	11.446	8.486
357	78	12	10	P	S	16.102	2.853	-1.237	-2.286	-2.699	1.297	2.395	72.437	10.655	8.488

BL number	Atoms	γ-PC unit cells	WS_2 unit cells	γ-PC origin atom	WS_2 origin atom	Twist-angle (°)	γ-PC strain 1 (%)	γ-PC strain 2 (%)	γ-PC strain 3 (%)	WS_2 strain 1 (%)	WS_2 strain 2 (%)	WS_2 strain 3 (%)	γ (°)	|**a**|(Å)	|**b**|(Å)
358	78	12	10	P	S	19.107	1.980	3.925	-1.485	-1.905	-4.045	1.530	72.347	8.495	10.648
359	78	12	10	P	S	27.126	1.029	-4.992	-0.580	-1.008	5.051	0.587	69.859	16.359	5.610
360	78	12	10	P	S	28.055	2.281	-3.339	-1.764	-2.182	3.461	1.829	89.916	15.362	5.611
361	78	12	10	P	S	31.945	2.281	3.339	-1.764	-2.182	-3.461	1.829	89.916	5.611	15.362
362	78	12	10	P	S	32.874	1.029	4.992	-0.580	-1.008	-5.051	0.587	69.859	5.610	16.359
363	78	12	10	P	S	40.893	1.980	-3.925	-1.485	-1.905	4.045	1.530	72.347	10.648	8.495
364	78	12	10	P	S	43.898	2.853	1.237	-2.286	-2.699	-1.297	2.395	72.437	10.655	8.488
365	78	12	10	P	S	44.392	-2.145	2.186	2.697	2.241	-2.074	-2.559	62.579	8.486	11.446
366	78	12	10	P	S	46.102	-0.414	5.160	0.858	0.417	-5.073	-0.843	72.462	8.482	10.654
367	78	12	10	P	S	73.898	-0.414	-5.160	0.858	0.417	5.073	-0.843	72.462	10.654	8.482
368	78	12	10	P	S	75.609	-2.145	-2.186	2.697	2.241	2.074	-2.559	62.579	11.446	8.486
369	78	12	10	P	S	76.102	2.853	-1.237	-2.286	-2.699	1.297	2.395	72.437	10.655	8.488
370	78	12	10	P	S	79.107	-1.485	4.063	1.980	1.530	-3.908	-1.905	72.323	8.492	10.653
371	78	12	10	P	S	87.126	1.029	-4.992	-0.580	-1.008	5.051	0.587	69.859	16.359	5.610
372	78	12	10	P	S	88.055	2.281	-3.339	-1.764	-2.182	3.461	1.829	89.916	15.362	5.611
373	78	12	10	P	S	91.945	2.281	3.339	-1.764	-2.182	-3.461	1.829	89.916	5.611	15.362
374	78	12	10	P	S	92.874	1.029	4.992	-0.580	-1.008	-5.051	0.587	69.859	5.610	16.359
375	78	12	10	P	S	100.893	-1.485	-4.063	1.980	1.530	3.908	-1.905	72.323	8.492	10.653
376	78	12	10	P	S	103.898	2.853	1.237	-2.286	-2.699	-1.297	2.395	72.437	10.655	8.488
377	78	12	10	P	S	104.392	-2.145	2.186	2.697	2.241	-2.074	-2.559	62.579	8.486	11.446
378	78	12	10	P	S	106.102	-0.414	5.160	0.858	0.417	-5.073	-0.843	72.462	8.482	10.654
379	78	12	10	P	S	116.996	-3.388	-5.478	4.103	3.635	5.063	-3.792	89.777	5.227	16.508
380	78	12	10	P	W	13.898	-0.414	-5.160	0.858	0.417	5.073	-0.843	72.462	10.654	8.482
381	78	12	10	P	W	15.608	-2.145	-2.186	2.697	2.241	2.074	-2.559	62.579	11.446	8.486
382	78	12	10	P	W	16.102	2.853	-1.237	-2.286	-2.699	1.297	2.395	72.437	10.655	8.488
383	78	12	10	P	W	19.107	1.980	3.925	-1.485	-1.905	-4.045	1.530	72.347	8.495	10.648
384	78	12	10	P	W	27.126	1.029	-4.992	-0.580	-1.008	5.051	0.587	69.859	16.359	5.610
385	78	12	10	P	W	28.055	2.281	-3.339	-1.764	-2.182	3.461	1.829	89.916	15.362	5.611
386	78	12	10	P	W	31.945	2.281	3.339	-1.764	-2.182	-3.461	1.829	89.916	5.611	15.362
387	78	12	10	P	W	32.874	1.029	4.992	-0.580	-1.008	-5.051	0.587	69.859	5.610	16.359
388	78	12	10	P	W	40.893	1.980	-3.925	-1.485	-1.905	4.045	1.530	72.347	10.648	8.495
389	78	12	10	P	W	43.898	2.853	1.237	-2.286	-2.699	-1.297	2.395	72.437	10.655	8.488
390	78	12	10	P	W	44.392	-2.145	2.186	2.697	2.241	-2.074	-2.559	62.579	8.486	11.446
391	78	12	10	P	W	46.102	-0.414	5.160	0.858	0.417	-5.073	-0.843	72.462	8.482	10.654
392	78	12	10	P	W	56.996	-3.388	-5.478	4.103	3.635	5.063	-3.792	89.777	5.227	16.508
393	78	12	10	P	W	63.005	-3.388	5.478	4.103	3.635	-5.063	-3.792	89.777	16.508	5.227
394	78	12	10	P	W	73.898	-0.414	-5.160	0.858	0.417	5.073	-0.843	72.462	10.654	8.482
395	78	12	10	P	W	75.609	-2.145	-2.186	2.697	2.241	2.074	-2.559	62.579	11.446	8.486
396	78	12	10	P	W	76.102	2.853	-1.237	-2.286	-2.699	1.297	2.395	72.437	10.655	8.488
397	78	12	10	P	W	79.107	-1.485	4.063	1.980	1.530	-3.908	-1.905	72.323	8.492	10.653
398	78	12	10	P	W	87.126	1.029	-4.992	-0.580	-1.008	5.051	0.587	69.859	16.359	5.610
399	78	12	10	P	W	88.055	2.281	-3.339	-1.764	-2.182	3.461	1.829	89.916	15.362	5.611
400	78	12	10	P	W	91.945	2.281	3.339	-1.764	-2.182	-3.461	1.829	89.916	5.611	15.362
401	78	12	10	P	W	92.874	1.029	4.992	-0.580	-1.008	-5.051	0.587	69.859	5.610	16.359
402	78	12	10	P	W	100.893	-1.485	-4.063	1.980	1.530	3.908	-1.905	72.323	8.492	10.653
403	78	12	10	P	W	103.898	2.853	1.237	-2.286	-2.699	-1.297	2.395	72.437	10.655	8.488
404	78	12	10	P	W	104.392	-2.145	2.186	2.697	2.241	-2.074	-2.559	62.579	8.486	11.446
405	78	12	10	P	W	106.102	-0.414	5.160	0.858	0.417	-5.073	-0.843	72.462	8.482	10.654
406	79	13	9	C	S	13.898	-4.228	0.000	-4.228	4.619	0.000	4.619	60.000	9.921	9.921
407	79	13	9	C	S	46.102	-4.228	0.000	-4.228	4.619	0.000	4.619	60.000	9.921	9.921
408	79	13	9	C	S	73.898	-4.228	0.000	-4.228	4.619	0.000	4.619	60.000	9.921	9.921

BL number	Atoms	γ-PC unit cells	WS_2 unit cells	γ-PC origin atom	WS_2 origin atom	Twist-angle (°)	γ-PC strain 1 (%)	γ-PC strain 2 (%)	γ-PC strain 3 (%)	WS_2 strain 1 (%)	WS_2 strain 2 (%)	WS_2 strain 3 (%)	γ (°)	|**a**|(Å)	|**b**|(Å)
409	79	13	9	C	S	106.102	-4.228	0.000	-4.228	4.619	0.000	4.619	60.000	9.921	9.921
410	79	13	9	C	W	13.898	-4.228	0.000	-4.228	4.619	0.000	4.619	60.000	9.921	9.921
411	79	13	9	C	W	46.102	-4.228	0.000	-4.228	4.619	0.000	4.619	60.000	9.921	9.921
412	79	13	9	C	W	73.898	-4.228	0.000	-4.228	4.619	0.000	4.619	60.000	9.921	9.921
413	79	13	9	C	W	106.102	-4.228	0.000	-4.228	4.619	0.000	4.619	60.000	9.921	9.921
414	85	13	11	C	S	1.359	-3.292	2.494	4.822	3.523	-2.274	-4.398	81.852	18.211	5.226
415	85	13	11	C	S	58.641	-3.292	-2.494	4.822	3.523	2.274	-4.398	81.852	5.226	18.211
416	85	13	11	C	S	61.359	-3.292	2.494	4.822	3.523	-2.274	-4.398	81.852	18.211	5.226
417	85	13	11	C	S	118.641	-3.292	-2.494	4.822	3.523	2.274	-4.398	81.852	5.226	18.211
418	85	13	11	C	W	1.359	-3.292	2.494	4.822	3.523	-2.274	-4.398	81.852	18.211	5.226
419	85	13	11	C	W	58.641	-3.292	-2.494	4.822	3.523	2.274	-4.398	81.852	5.226	18.211
420	85	13	11	C	W	61.359	-3.292	2.494	4.822	3.523	-2.274	-4.398	81.852	18.211	5.226
421	85	13	11	C	W	118.641	-3.292	-2.494	4.822	3.523	2.274	-4.398	81.852	5.226	18.211
422	85	13	11	P	S	1.359	-3.292	2.494	4.822	3.523	-2.274	-4.398	81.852	18.211	5.226
423	85	13	11	P	S	118.641	-3.292	-2.494	4.822	3.523	2.274	-4.398	81.852	5.226	18.211
424	85	13	11	P	W	58.641	-3.292	-2.494	4.822	3.523	2.274	-4.398	81.852	5.226	18.211
425	85	13	11	P	W	61.359	-3.292	2.494	4.822	3.523	-2.274	-4.398	81.852	18.211	5.226
426	86	14	10	C	S	24.791	-3.388	-4.696	-3.626	3.635	5.063	3.909	87.672	5.608	16.632
427	86	14	10	C	S	25.285	-4.781	-3.927	-2.197	5.287	4.107	2.298	87.701	16.641	5.606
428	86	14	10	C	S	30.000	-2.359	-4.027	-4.628	2.476	4.437	5.100	87.582	5.611	16.626
429	86	14	10	C	S	34.715	-4.781	3.927	-2.197	5.287	-4.107	2.298	87.701	16.641	5.606
430	86	14	10	C	S	35.209	-3.388	4.696	-3.626	3.635	-5.063	3.909	87.672	16.632	5.608
431	86	14	10	C	S	84.791	-3.388	-4.696	-3.626	3.635	5.063	3.909	87.672	5.608	16.632
432	86	14	10	C	S	85.285	-4.781	-3.927	-2.197	5.287	4.107	2.298	87.701	16.641	5.606
433	86	14	10	C	S	90.000	-2.359	-4.027	-4.628	2.476	4.437	5.100	87.582	5.611	16.626
434	86	14	10	C	S	94.715	-4.781	3.927	-2.197	5.287	-4.107	2.298	87.701	16.641	5.606
435	86	14	10	C	S	95.209	-3.388	4.696	-3.626	3.635	-5.063	3.909	87.672	16.632	5.608
436	86	14	10	C	W	24.791	-3.388	-4.696	-3.626	3.635	5.063	3.909	87.672	5.608	16.632
437	86	14	10	C	W	25.285	-4.781	-3.927	-2.197	5.287	4.107	2.298	87.701	16.641	5.606
438	86	14	10	C	W	30.000	-2.359	-4.027	-4.628	2.476	4.437	5.100	87.582	5.611	16.626
439	86	14	10	C	W	34.715	-4.781	3.927	-2.197	5.287	-4.107	2.298	87.701	16.641	5.606
440	86	14	10	C	W	35.209	-3.388	4.696	-3.626	3.635	-5.063	3.909	87.672	16.632	5.608
441	86	14	10	C	W	84.791	-3.388	-4.696	-3.626	3.635	5.063	3.909	87.672	5.608	16.632
442	86	14	10	C	W	85.285	-4.781	-3.927	-2.197	5.287	4.107	2.298	87.701	16.641	5.606
443	86	14	10	C	W	90.000	-2.359	-4.027	-4.628	2.476	4.437	5.100	87.582	5.611	16.626
444	86	14	10	C	W	94.715	-4.781	3.927	-2.197	5.287	-4.107	2.298	87.701	16.641	5.606
445	86	14	10	C	W	95.209	-3.388	4.696	-3.626	3.635	-5.063	3.909	87.672	16.632	5.608
446	86	14	10	P	S	24.791	-3.388	-4.696	-3.626	3.635	5.063	3.909	87.672	5.608	16.632
447	86	14	10	P	S	25.285	-4.781	-3.927	-2.197	5.287	4.107	2.298	87.701	16.641	5.606
448	86	14	10	P	S	30.000	-2.359	-4.027	-4.628	2.476	4.437	5.100	87.582	5.611	16.626
449	86	14	10	P	S	34.715	-4.781	3.927	-2.197	5.287	-4.107	2.298	87.701	16.641	5.606
450	86	14	10	P	S	35.209	-3.388	4.696	-3.626	3.635	-5.063	3.909	87.672	16.632	5.608
451	86	14	10	P	S	84.791	-3.388	-4.696	-3.626	3.635	5.063	3.909	87.672	5.608	16.632
452	86	14	10	P	S	85.285	-4.781	-3.927	-2.197	5.287	4.107	2.298	87.701	16.641	5.606
453	86	14	10	P	S	90.000	-2.359	-4.027	-4.628	2.476	4.437	5.100	87.582	5.611	16.626
454	86	14	10	P	S	94.715	-4.781	3.927	-2.197	5.287	-4.107	2.298	87.701	16.641	5.606
455	86	14	10	P	S	95.209	-3.388	4.696	-3.626	3.635	-5.063	3.909	87.672	16.632	5.608
456	86	14	10	P	W	24.791	-3.388	-4.696	-3.626	3.635	5.063	3.909	87.672	5.608	16.632
457	86	14	10	P	W	25.285	-4.781	-3.927	-2.197	5.287	4.107	2.298	87.701	16.641	5.606
458	86	14	10	P	W	30.000	-2.359	-4.027	-4.628	2.476	4.437	5.100	87.582	5.611	16.626
459	86	14	10	P	W	34.715	-4.781	3.927	-2.197	5.287	-4.107	2.298	87.701	16.641	5.606

BL number	Atoms	γ-PC unit cells	WS_2 unit cells	γ-PC origin atom	WS_2 origin atom	Twist-angle (°)	γ-PC strain 1 (%)	γ-PC strain 2 (%)	γ-PC strain 3 (%)	WS_2 strain 1 (%)	WS_2 strain 2 (%)	WS_2 strain 3 (%)	γ (°)	$\lvert\mathbf{a}\rvert$(Å)	$\lvert\mathbf{b}\rvert$(Å)
460	86	14	10	P	W	35.209	-3.388	4.696	-3.626	3.635	-5.063	3.909	87.672	16.632	5.608
461	86	14	10	P	W	84.791	-3.388	-4.696	-3.626	3.635	5.063	3.909	87.672	5.608	16.632
462	86	14	10	P	W	85.285	-4.781	-3.927	-2.197	5.287	4.107	2.298	87.701	16.641	5.606
463	86	14	10	P	W	90.000	-2.359	-4.027	-4.628	2.476	4.437	5.100	87.582	5.611	16.626
464	86	14	10	P	W	94.715	-4.781	3.927	-2.197	5.287	-4.107	2.298	87.701	16.641	5.606
465	86	14	10	P	W	95.209	-3.388	4.696	-3.626	3.635	-5.063	3.909	87.672	16.632	5.608
466	88	13	12	C	S	43.898	2.853	0.000	2.853	-2.699	0.000	-2.699	60.000	10.655	10.655
467	88	13	12	C	S	76.102	2.853	0.000	2.853	-2.699	0.000	-2.699	60.000	10.655	10.655
468	88	13	12	C	S	103.898	2.853	0.000	2.853	-2.699	0.000	-2.699	60.000	10.655	10.655
469	88	13	12	C	W	16.102	2.853	0.000	2.853	-2.699	0.000	-2.699	60.000	10.655	10.655
470	88	13	12	C	W	43.898	2.853	0.000	2.853	-2.699	0.000	-2.699	60.000	10.655	10.655
471	88	13	12	C	W	103.898	2.853	0.000	2.853	-2.699	0.000	-2.699	60.000	10.655	10.655
472	88	13	12	C	S	21.052	1.339	-3.751	4.411	-1.304	3.447	-4.053	78.291	7.981	12.584
473	88	13	12	C	S	21.787	5.011	-2.332	0.779	-4.554	2.296	-0.767	78.237	7.983	12.586
474	88	13	12	C	S	23.413	0.481	0.558	5.335	-0.477	-0.504	-4.821	78.331	12.584	7.982
475	88	13	12	C	S	25.285	4.262	3.859	1.480	-3.928	-3.748	-1.437	66.198	13.480	7.973
476	88	13	12	C	S	25.693	2.326	4.664	3.385	-2.223	-4.368	-3.170	78.352	12.588	7.975
477	88	13	12	C	S	34.307	2.326	-4.664	3.385	-2.223	4.368	-3.170	78.352	7.975	12.588
478	88	13	12	C	S	34.715	4.262	-3.859	1.480	-3.928	3.748	-1.437	66.198	7.973	13.480
479	88	13	12	C	S	36.587	0.481	-0.558	5.335	-0.477	0.504	-4.821	78.331	12.584	7.982
480	88	13	12	C	S	38.213	5.011	2.332	0.779	-4.554	-2.296	-0.767	78.237	7.983	12.586
481	88	13	12	C	S	38.948	1.339	3.751	4.411	-1.304	-3.447	-4.053	78.291	12.584	7.981
482	88	13	12	C	S	81.052	1.339	-3.751	4.411	-1.304	3.447	-4.053	78.291	7.981	12.584
483	88	13	12	C	S	81.787	5.011	-2.332	0.779	-4.554	2.296	-0.767	78.237	7.983	12.586
484	88	13	12	C	S	83.413	0.481	0.558	5.335	-0.477	-0.504	-4.821	78.331	12.584	7.982
485	88	13	12	C	S	85.285	4.262	3.859	1.480	-3.928	-3.748	-1.437	66.198	13.480	7.973
486	88	13	12	C	S	85.694	2.326	4.664	3.385	-2.223	-4.368	-3.170	78.352	12.588	7.975
487	88	13	12	C	S	94.307	2.326	-4.664	3.385	-2.223	4.368	-3.170	78.352	7.975	12.588
488	88	13	12	C	S	94.715	4.262	-3.859	1.480	-3.928	3.748	-1.437	66.198	7.973	13.480
489	88	13	12	C	S	96.587	0.481	-0.558	5.335	-0.477	0.504	-4.821	78.331	12.584	7.982
490	88	13	12	C	S	98.213	5.011	2.332	0.779	-4.554	-2.296	-0.767	78.237	7.983	12.586
491	88	13	12	C	S	98.948	1.339	3.751	4.411	-1.304	-3.447	-4.053	78.291	12.584	7.981
492	88	13	12	C	W	21.052	1.339	-3.751	4.411	-1.304	3.447	-4.053	78.291	7.981	12.584
493	88	13	12	C	W	21.787	5.011	-2.332	0.779	-4.554	2.296	-0.767	78.237	7.983	12.586
494	88	13	12	C	W	23.413	0.481	0.558	5.335	-0.477	-0.504	-4.821	78.331	12.584	7.982
495	88	13	12	C	W	25.285	4.262	3.859	1.480	-3.928	-3.748	-1.437	66.198	13.480	7.973
496	88	13	12	C	W	25.693	2.326	4.664	3.385	-2.223	-4.368	-3.170	78.352	12.588	7.975
497	88	13	12	C	W	34.307	2.326	-4.664	3.385	-2.223	4.368	-3.170	78.352	7.975	12.588
498	88	13	12	C	W	34.715	4.262	-3.859	1.480	-3.928	3.748	-1.437	66.198	7.973	13.480
499	88	13	12	C	W	36.587	0.481	-0.558	5.335	-0.477	0.504	-4.821	78.331	12.584	7.982
500	88	13	12	C	W	38.213	5.011	2.332	0.779	-4.554	-2.296	-0.767	78.237	7.983	12.586
501	88	13	12	C	W	38.948	1.339	3.751	4.411	-1.304	-3.447	-4.053	78.291	12.584	7.981
502	88	13	12	C	W	81.052	1.339	-3.751	4.411	-1.304	3.447	-4.053	78.291	7.981	12.584
503	88	13	12	C	W	81.787	5.011	-2.332	0.779	-4.554	2.296	-0.767	78.237	7.983	12.586
504	88	13	12	C	W	83.413	0.481	0.558	5.335	-0.477	-0.504	-4.821	78.331	12.584	7.982
505	88	13	12	C	W	85.285	4.262	3.859	1.480	-3.928	-3.748	-1.437	66.198	13.480	7.973
506	88	13	12	C	W	85.694	2.326	4.664	3.385	-2.223	-4.368	-3.170	78.352	12.588	7.975
507	88	13	12	C	W	94.307	2.326	-4.664	3.385	-2.223	4.368	-3.170	78.352	7.975	12.588
508	88	13	12	C	W	94.715	4.262	-3.859	1.480	-3.928	3.748	-1.437	66.198	7.973	13.480
509	88	13	12	C	W	96.587	0.481	-0.558	5.335	-0.477	0.504	-4.821	78.331	12.584	7.982
510	88	13	12	C	W	98.213	5.011	2.332	0.779	-4.554	-2.296	-0.767	78.237	7.983	12.586

BL number	Atoms	γ-PC unit cells	WS_2 unit cells	γ-PC origin atom	WS_2 origin atom	Twist-angle (°)	γ-PC strain 1 (%)	γ-PC strain 2 (%)	γ-PC strain 3 (%)	WS_2 strain 1 (%)	WS_2 strain 2 (%)	WS_2 strain 3 (%)	γ (°)	|**a**|(Å)	|**b**|(Å)
511	88	13	12	C	W	98.948	1.339	3.751	4.411	-1.304	-3.447	-4.053	78.291	12.584	7.981
512	88	13	12	P	S	21.052	1.339	-3.751	4.411	-1.304	3.447	-4.053	78.291	7.981	12.584
513	88	13	12	P	S	21.787	5.011	-2.332	0.779	-4.554	2.296	-0.767	78.237	7.983	12.586
514	88	13	12	P	S	23.413	0.481	0.558	5.335	-0.477	-0.504	-4.821	78.331	12.584	7.982
515	88	13	12	P	S	25.285	4.262	3.859	1.480	-3.928	-3.748	-1.437	66.198	13.480	7.973
516	88	13	12	P	S	25.693	2.326	4.664	3.385	-2.223	-4.368	-3.170	78.352	12.588	7.975
517	88	13	12	P	S	34.307	2.326	-4.664	3.385	-2.223	4.368	-3.170	78.352	7.975	12.588
518	88	13	12	P	S	34.715	4.262	-3.859	1.480	-3.928	3.748	-1.437	66.198	7.973	13.480
519	88	13	12	P	S	36.587	0.481	-0.558	5.335	-0.477	0.504	-4.821	78.331	12.584	7.982
520	88	13	12	P	S	38.213	5.011	2.332	0.779	-4.554	-2.296	-0.767	78.237	7.983	12.586
521	88	13	12	P	S	38.948	1.339	3.751	4.411	-1.304	-3.447	-4.053	78.291	12.584	7.981
522	88	13	12	P	S	81.052	1.339	-3.751	4.411	-1.304	3.447	-4.053	78.291	7.981	12.584
523	88	13	12	P	S	81.787	5.011	-2.332	0.779	-4.554	2.296	-0.767	78.237	7.983	12.586
524	88	13	12	P	S	83.413	0.481	0.558	5.335	-0.477	-0.504	-4.821	78.331	12.584	7.982
525	88	13	12	P	S	85.285	4.262	3.859	1.480	-3.928	-3.748	-1.437	66.198	13.480	7.973
526	88	13	12	P	S	85.694	2.326	4.664	3.385	-2.223	-4.368	-3.170	78.352	12.588	7.975
527	88	13	12	P	S	94.307	2.326	-4.664	3.385	-2.223	4.368	-3.170	78.352	7.975	12.588
528	88	13	12	P	S	94.715	4.262	-3.859	1.480	-3.928	3.748	-1.437	66.198	7.973	13.480
529	88	13	12	P	S	96.587	0.481	-0.558	5.335	-0.477	0.504	-4.821	78.331	12.584	7.982
530	88	13	12	P	S	98.213	5.011	2.332	0.779	-4.554	-2.296	-0.767	78.237	7.983	12.586
531	88	13	12	P	S	98.948	1.339	3.751	4.411	-1.304	-3.447	-4.053	78.291	12.584	7.981
532	88	13	12	P	W	21.052	1.339	-3.751	4.411	-1.304	3.447	-4.053	78.291	7.981	12.584
533	88	13	12	P	W	21.787	5.011	-2.332	0.779	-4.554	2.296	-0.767	78.237	7.983	12.586
534	88	13	12	P	W	23.413	0.481	0.558	5.335	-0.477	-0.504	-4.821	78.331	12.584	7.982
535	88	13	12	P	W	25.285	4.262	3.859	1.480	-3.928	-3.748	-1.437	66.198	13.480	7.973
536	88	13	12	P	W	25.693	2.326	4.664	3.385	-2.223	-4.368	-3.170	78.352	12.588	7.975
537	88	13	12	P	W	34.307	2.326	-4.664	3.385	-2.223	4.368	-3.170	78.352	7.975	12.588
538	88	13	12	P	W	34.715	4.262	-3.859	1.480	-3.928	3.748	-1.437	66.198	7.973	13.480
539	88	13	12	P	W	36.587	0.481	-0.558	5.335	-0.477	0.504	-4.821	78.331	12.584	7.982
540	88	13	12	P	W	38.213	5.011	2.332	0.779	-4.554	-2.296	-0.767	78.237	7.983	12.586
541	88	13	12	P	W	38.948	1.339	3.751	4.411	-1.304	-3.447	-4.053	78.291	12.584	7.981
542	88	13	12	P	W	81.052	1.339	-3.751	4.411	-1.304	3.447	-4.053	78.291	7.981	12.584
543	88	13	12	P	W	81.787	5.011	-2.332	0.779	-4.554	2.296	-0.767	78.237	7.983	12.586
544	88	13	12	P	W	83.413	0.481	0.558	5.335	-0.477	-0.504	-4.821	78.331	12.584	7.982
545	88	13	12	P	W	85.285	4.262	3.859	1.480	-3.928	-3.748	-1.437	66.198	13.480	7.973
546	88	13	12	P	W	85.694	2.326	4.664	3.385	-2.223	-4.368	-3.170	78.352	12.588	7.975
547	88	13	12	P	W	94.307	2.326	-4.664	3.385	-2.223	4.368	-3.170	78.352	7.975	12.588
548	88	13	12	P	W	94.715	4.262	-3.859	1.480	-3.928	3.748	-1.437	66.198	7.973	13.480
549	88	13	12	P	W	96.587	0.481	-0.558	5.335	-0.477	0.504	-4.821	78.331	12.584	7.982
550	88	13	12	P	W	98.213	5.011	2.332	0.779	-4.554	-2.296	-0.767	78.237	7.983	12.586
551	88	13	12	P	W	98.948	1.339	3.751	4.411	-1.304	-3.447	-4.053	78.291	12.584	7.981
552	89	14	11	C	S	25.050	-0.955	-4.546	-1.520	0.973	4.688	1.567	83.207	17.541	5.605
553	89	14	11	C	S	25.767	0.353	-3.271	-2.779	-0.351	3.463	2.943	83.226	17.539	5.607
554	89	14	11	C	S	27.126	1.029	-0.957	-3.404	-1.008	1.027	3.653	83.205	5.609	17.538
555	89	14	11	C	S	30.000	-2.359	-4.027	-0.090	2.476	4.034	0.091	83.106	5.611	17.529
556	89	14	11	C	S	32.874	1.029	0.957	-3.404	-1.008	-1.027	3.653	83.205	17.538	5.609
557	89	14	11	C	S	34.233	0.353	3.271	-2.779	-0.351	-3.463	2.943	83.226	17.539	5.607
558	89	14	11	C	S	34.950	-0.955	4.546	-1.520	0.973	-4.688	1.567	83.207	5.605	17.541
559	89	14	11	C	S	85.050	-0.955	-4.546	-1.520	0.973	4.688	1.567	83.207	17.541	5.605
560	89	14	11	C	S	85.767	0.353	-3.271	-2.779	-0.351	3.463	2.943	83.226	17.539	5.607
561	89	14	11	C	S	87.126	1.029	-0.957	-3.404	-1.008	1.027	3.653	83.205	5.609	17.538

BL number	Atoms	γ-PC unit cells	WS_2 unit cells	γ-PC origin atom	WS_2 origin atom	Twist-angle (°)	γ-PC strain 1 (%)	γ-PC strain 2 (%)	γ-PC strain 3 (%)	WS_2 strain 1 (%)	WS_2 strain 2 (%)	WS_2 strain 3 (%)	γ (°)	|**a**|(Å)	|**b**|(Å)
562	89	14	11	C	S	90.000	-2.359	-4.027	-0.090	2.476	4.034	0.091	83.106	5.611	17.529
563	89	14	11	C	S	92.874	1.029	0.957	-3.404	-1.008	-1.027	3.653	83.205	17.538	5.609
564	89	14	11	C	S	94.233	0.353	3.271	-2.779	-0.351	-3.463	2.943	83.226	17.539	5.607
565	89	14	11	C	S	94.950	-0.955	4.546	-1.520	0.973	-4.688	1.567	83.207	5.605	17.541
566	89	14	11	C	W	25.050	-0.955	-4.546	-1.520	0.973	4.688	1.567	83.207	17.541	5.605
567	89	14	11	C	W	25.767	0.353	-3.271	-2.779	-0.351	3.463	2.943	83.226	17.539	5.607
568	89	14	11	C	W	27.126	1.029	-0.957	-3.404	-1.008	1.027	3.653	83.205	5.609	17.538
569	89	14	11	C	W	30.000	-2.359	-4.027	-0.090	2.476	4.034	0.091	83.106	5.611	17.529
570	89	14	11	C	W	32.874	1.029	0.957	-3.404	-1.008	-1.027	3.653	83.205	17.538	5.609
571	89	14	11	C	W	34.233	0.353	3.271	-2.779	-0.351	-3.463	2.943	83.226	17.539	5.607
572	89	14	11	C	W	34.950	-0.955	4.546	-1.520	0.973	-4.688	1.567	83.207	5.605	17.541
573	89	14	11	C	W	85.050	-0.955	-4.546	-1.520	0.973	4.688	1.567	83.207	17.541	5.605
574	89	14	11	C	W	85.767	0.353	-3.271	-2.779	-0.351	3.463	2.943	83.226	17.539	5.607
575	89	14	11	C	W	87.126	1.029	-0.957	-3.404	-1.008	1.027	3.653	83.205	5.609	17.538
576	89	14	11	C	W	90.000	-2.359	-4.027	-0.090	2.476	4.034	0.091	83.106	5.611	17.529
577	89	14	11	C	W	92.874	1.029	0.957	-3.404	-1.008	-1.027	3.653	83.205	17.538	5.609
578	89	14	11	C	W	94.233	0.353	3.271	-2.779	-0.351	-3.463	2.943	83.226	17.539	5.607
579	89	14	11	C	W	94.950	-0.955	4.546	-1.520	0.973	-4.688	1.567	83.207	5.605	17.541
580	89	14	11	P	S	25.050	-0.955	-4.546	-1.520	0.973	4.688	1.567	83.207	17.541	5.605
581	89	14	11	P	S	25.767	0.353	-3.271	-2.779	-0.351	3.463	2.943	83.226	17.539	5.607
582	89	14	11	P	S	27.126	1.029	-0.957	-3.404	-1.008	1.027	3.653	83.205	5.609	17.538
583	89	14	11	P	S	30.000	-2.359	-4.027	-0.090	2.476	4.034	0.091	83.106	5.611	17.529
584	89	14	11	P	S	32.874	1.029	0.957	-3.404	-1.008	-1.027	3.653	83.205	17.538	5.609
585	89	14	11	P	S	34.233	0.353	3.271	-2.779	-0.351	-3.463	2.943	83.226	17.539	5.607
586	89	14	11	P	S	34.950	-0.955	4.546	-1.520	0.973	-4.688	1.567	83.207	5.605	17.541
587	89	14	11	P	S	85.050	-0.955	-4.546	-1.520	0.973	4.688	1.567	83.207	17.541	5.605
588	89	14	11	P	S	85.767	0.353	-3.271	-2.779	-0.351	3.463	2.943	83.226	17.539	5.607
589	89	14	11	P	S	87.126	1.029	-0.957	-3.404	-1.008	1.027	3.653	83.205	5.609	17.538
590	89	14	11	P	S	90.000	-2.359	-4.027	-0.090	2.476	4.034	0.091	83.106	5.611	17.529
591	89	14	11	P	S	92.874	1.029	0.957	-3.404	-1.008	-1.027	3.653	83.205	17.538	5.609
592	89	14	11	P	S	94.233	0.353	3.271	-2.779	-0.351	-3.463	2.943	83.226	17.539	5.607
593	89	14	11	P	S	94.950	-0.955	4.546	-1.520	0.973	-4.688	1.567	83.207	5.605	17.541
594	89	14	11	P	W	25.050	-0.955	-4.546	-1.520	0.973	4.688	1.567	83.207	17.541	5.605
595	89	14	11	P	W	25.767	0.353	-3.271	-2.779	-0.351	3.463	2.943	83.226	17.539	5.607
596	89	14	11	P	W	27.126	1.029	-0.957	-3.404	-1.008	1.027	3.653	83.205	5.609	17.538
597	89	14	11	P	W	30.000	-2.359	-4.027	-0.090	2.476	4.034	0.091	83.106	5.611	17.529
598	89	14	11	P	W	32.874	1.029	0.957	-3.404	-1.008	-1.027	3.653	83.205	17.538	5.609
599	89	14	11	P	W	34.233	0.353	3.271	-2.779	-0.351	-3.463	2.943	83.226	17.539	5.607
600	89	14	11	P	W	34.950	-0.955	4.546	-1.520	0.973	-4.688	1.567	83.207	5.605	17.541
601	89	14	11	P	W	85.050	-0.955	-4.546	-1.520	0.973	4.688	1.567	83.207	17.541	5.605
602	89	14	11	P	W	85.767	0.353	-3.271	-2.779	-0.351	3.463	2.943	83.226	17.539	5.607
603	89	14	11	P	W	87.126	1.029	-0.957	-3.404	-1.008	1.027	3.653	83.205	5.609	17.538
604	89	14	11	P	W	90.000	-2.359	-4.027	-0.090	2.476	4.034	0.091	83.106	5.611	17.529
605	89	14	11	P	W	92.874	1.029	0.957	-3.404	-1.008	-1.027	3.653	83.205	17.538	5.609
606	89	14	11	P	W	94.233	0.353	3.271	-2.779	-0.351	-3.463	2.943	83.226	17.539	5.607
607	89	14	11	P	W	94.950	-0.955	4.546	-1.520	0.973	-4.688	1.567	83.207	5.605	17.541
608	91	13	13	C	S	27.796	5.011	0.000	5.011	-4.554	0.000	-4.555	60.000	10.878	10.878
609	91	13	13	C	S	32.204	5.011	0.000	5.011	-4.554	0.000	-4.555	60.000	10.878	10.878
610	91	13	13	C	S	87.796	5.011	0.000	5.011	-4.554	0.000	-4.555	60.000	10.878	10.878
611	91	13	13	C	S	92.204	5.011	0.000	5.011	-4.554	0.000	-4.555	60.000	10.878	10.878
612	91	13	13	C	W	27.796	5.011	0.000	5.011	-4.554	0.000	-4.555	60.000	10.878	10.878

BL number	Atoms	γ-PC unit cells	WS_2 unit cells	γ-PC origin atom	WS_2 origin atom	Twist-angle (°)	γ-PC strain 1 (%)	γ-PC strain 2 (%)	γ-PC strain 3 (%)	WS_2 strain 1 (%)	WS_2 strain 2 (%)	WS_2 strain 3 (%)	γ (°)	$\vert\mathbf{a}\vert$(Å)	$\vert\mathbf{b}\vert$(Å)
613	91	13	13	C	W	32.204	5.011	0.000	5.011	-4.554	0.000	-4.555	60.000	10.878	10.878
614	91	13	13	C	W	87.796	5.011	0.000	5.011	-4.554	0.000	-4.555	60.000	10.878	10.878
615	91	13	13	C	W	92.204	5.011	0.000	5.011	-4.554	0.000	-4.555	60.000	10.878	10.878
616	91	13	13	P	S	27.796	5.011	0.000	5.011	-4.554	0.000	-4.555	60.000	10.878	10.878
617	91	13	13	P	S	92.204	5.011	0.000	5.011	-4.554	0.000	-4.555	60.000	10.878	10.878
618	91	13	13	P	W	32.204	5.011	0.000	5.011	-4.554	0.000	-4.555	60.000	10.878	10.878
619	91	13	13	P	W	87.796	5.011	0.000	5.011	-4.554	0.000	-4.555	60.000	10.878	10.878
620	92	14	12	C	S	0.000	5.011	0.000	-2.848	-4.554	0.000	3.020	90.000	5.226	19.540
621	92	14	12	C	S	60.000	5.011	0.000	-2.848	-4.554	0.000	3.020	90.000	5.226	19.539
622	92	14	12	C	W	0.000	5.011	0.000	-2.848	-4.554	0.000	3.020	90.000	5.226	19.540
623	92	14	12	C	W	60.000	5.011	0.000	-2.848	-4.554	0.000	3.020	90.000	5.226	19.539
624	92	14	12	P	S	0.000	5.011	0.000	-2.848	-4.554	0.000	3.020	90.000	5.226	19.540
625	92	14	12	P	W	60.000	5.011	0.000	-2.848	-4.554	0.000	3.020	90.000	5.226	19.539
626	92	14	12	C	S	25.285	4.262	-4.300	-2.197	-3.928	4.497	2.298	87.483	18.222	5.606
627	92	14	12	C	S	30.000	-2.359	4.027	4.447	2.476	-3.698	-4.084	74.898	9.718	10.879
628	92	14	12	C	S	34.715	4.262	4.300	-2.197	-3.928	-4.497	2.298	87.483	18.222	5.606
629	92	14	12	C	S	85.285	4.262	-4.300	-2.197	-3.928	4.497	2.298	87.483	18.222	5.606
630	92	14	12	C	S	90.000	-2.359	-4.027	4.447	2.476	3.698	-4.084	87.792	5.611	18.206
631	92	14	12	C	S	94.715	4.262	4.300	-2.197	-3.928	-4.497	2.298	87.483	18.222	5.606
632	92	14	12	C	W	25.285	4.262	-4.300	-2.197	-3.928	4.497	2.298	87.483	18.222	5.606
633	92	14	12	C	W	30.000	-2.359	4.027	4.447	2.476	-3.698	-4.084	74.898	9.718	10.879
634	92	14	12	C	W	34.715	4.262	4.300	-2.197	-3.928	-4.497	2.298	87.483	18.222	5.606
635	92	14	12	C	W	85.285	4.262	-4.300	-2.197	-3.928	4.497	2.298	87.483	18.222	5.606
636	92	14	12	C	W	90.000	-2.359	-4.027	4.447	2.476	3.698	-4.084	87.792	5.611	18.206
637	92	14	12	C	W	94.715	4.262	4.300	-2.197	-3.928	-4.497	2.298	87.483	18.222	5.606
638	92	14	12	P	S	25.285	4.262	-4.300	-2.197	-3.928	4.497	2.298	87.483	18.222	5.606
639	92	14	12	P	S	30.000	-2.359	4.027	4.447	2.476	-3.698	-4.084	87.792	5.611	18.206
640	92	14	12	P	S	90.000	-2.359	-4.027	4.447	2.476	3.698	-4.084	87.792	5.611	18.206
641	92	14	12	P	S	94.715	4.262	4.300	-2.197	-3.928	-4.497	2.298	87.483	18.222	5.606
642	92	14	12	P	W	30.000	-2.359	4.027	4.447	2.476	-3.698	-4.084	74.898	9.718	10.879
643	92	14	12	P	W	34.715	4.262	4.300	-2.197	-3.928	-4.497	2.298	87.483	18.222	5.606
644	92	14	12	P	W	85.285	4.262	-4.300	-2.197	-3.928	4.497	2.298	87.483	18.222	5.606
645	92	14	12	P	W	90.000	-2.359	4.027	4.447	2.476	-3.698	-4.084	87.792	5.611	18.206
646	93	15	11	C	S	19.842	-4.723	-2.096	-0.986	5.216	2.138	1.006	85.114	8.496	11.951
647	93	15	11	C	S	22.689	-4.497	2.828	-1.230	4.941	-2.899	1.261	85.254	11.961	8.486
648	93	15	11	C	S	23.413	-2.043	3.969	-3.725	2.130	-4.288	4.025	85.206	11.956	8.489
649	93	15	11	C	S	36.587	-2.043	-3.969	-3.725	2.130	4.288	4.025	85.206	8.489	11.956
650	93	15	11	C	S	37.311	-4.497	-2.828	-1.230	4.941	2.899	1.261	85.254	11.961	8.486
651	93	15	11	C	S	40.158	-4.723	2.096	-0.986	5.216	-2.138	1.006	85.114	11.951	8.496
652	93	15	11	C	S	40.893	-1.485	3.250	-4.257	1.530	-3.553	4.653	85.149	8.492	11.954
653	93	15	11	C	S	79.107	-1.485	-3.250	-4.257	1.530	3.553	4.653	85.149	8.492	11.954
654	93	15	11	C	S	79.842	-4.723	-2.096	-0.986	5.216	2.138	1.006	85.114	8.496	11.951
655	93	15	11	C	S	82.689	-4.497	2.828	-1.230	4.941	-2.899	1.261	85.254	11.961	8.486
656	93	15	11	C	S	83.413	-2.043	3.969	-3.725	2.130	-4.288	4.025	85.206	11.956	8.489
657	93	15	11	C	S	96.587	-2.043	-3.969	-3.725	2.130	4.288	4.025	85.206	8.489	11.956
658	93	15	11	C	S	97.311	-4.497	-2.828	-1.230	4.941	2.899	1.261	85.254	11.961	8.486
659	93	15	11	C	S	100.158	-4.723	2.096	-0.986	5.216	-2.138	1.006	85.114	11.951	8.496
660	93	15	11	C	W	19.107	-1.485	-3.250	-4.257	1.530	3.553	4.653	85.149	8.492	11.954
661	93	15	11	C	W	19.842	-4.723	-2.096	-0.986	5.216	2.138	1.006	85.114	8.496	11.951
662	93	15	11	C	W	22.689	-4.497	2.828	-1.230	4.941	-2.899	1.261	85.254	11.961	8.486
663	93	15	11	C	W	23.413	-2.043	3.969	-3.725	2.130	-4.288	4.025	85.206	11.956	8.489

BL number	Atoms	γ-PC unit cells	WS_2 unit cells	γ-PC origin atom	WS_2 origin atom	Twist-angle (°)	γ-PC strain 1 (%)	γ-PC strain 2 (%)	γ-PC strain 3 (%)	WS_2 strain 1 (%)	WS_2 strain 2 (%)	WS_2 strain 3 (%)	γ (°)	|**a**|(Å)	|**b**|(Å)
664	93	15	11	C	W	36.587	-2.043	-3.969	-3.725	2.130	4.288	4.025	85.206	8.489	11.956
665	93	15	11	C	W	37.311	-4.497	-2.828	-1.230	4.941	2.899	1.261	85.254	11.961	8.486
666	93	15	11	C	W	40.158	-4.723	2.096	-0.986	5.216	-2.138	1.006	85.114	11.951	8.496
667	93	15	11	C	W	79.842	-4.723	-2.096	-0.986	5.216	2.138	1.006	85.114	8.496	11.951
668	93	15	11	C	W	82.689	-4.497	2.828	-1.230	4.941	-2.899	1.261	85.254	11.961	8.486
669	93	15	11	C	W	83.413	-2.043	3.969	-3.725	2.130	-4.288	4.025	85.206	11.956	8.489
670	93	15	11	C	W	96.587	-2.043	-3.969	-3.725	2.130	4.288	4.025	85.206	8.489	11.956
671	93	15	11	C	W	97.311	-4.497	-2.828	-1.230	4.941	2.899	1.261	85.254	11.961	8.486
672	93	15	11	C	W	100.158	-4.723	2.096	-0.986	5.216	-2.138	1.006	85.114	11.951	8.496
673	93	15	11	C	W	100.893	-1.485	3.250	-4.257	1.530	-3.553	4.653	85.149	8.492	11.954
674	93	15	11	P	S	19.107	-1.485	-3.250	-4.257	1.530	3.553	4.653	85.149	8.492	11.954
675	93	15	11	P	S	19.842	-4.723	-2.096	-0.986	5.216	2.138	1.006	85.114	8.496	11.951
676	93	15	11	P	S	22.689	-4.497	2.828	-1.230	4.941	-2.899	1.261	85.254	11.961	8.486
677	93	15	11	P	S	23.413	-2.043	3.969	-3.725	2.130	-4.288	4.025	85.206	11.956	8.489
678	93	15	11	P	S	36.587	-2.043	-3.969	-3.725	2.130	4.288	4.025	85.206	8.489	11.956
679	93	15	11	P	S	37.311	-4.497	-2.828	-1.230	4.941	2.899	1.261	85.254	11.961	8.486
680	93	15	11	P	S	40.158	-4.723	2.096	-0.986	5.216	-2.138	1.006	85.114	11.951	8.496
681	93	15	11	P	S	79.842	-4.723	-2.096	-0.986	5.216	2.138	1.006	85.114	8.496	11.951
682	93	15	11	P	S	82.689	-4.497	2.828	-1.230	4.941	-2.899	1.261	85.254	11.961	8.486
683	93	15	11	P	S	83.413	-2.043	3.969	-3.725	2.130	-4.288	4.025	85.206	11.956	8.489
684	93	15	11	P	S	96.587	-2.043	-3.969	-3.725	2.130	4.288	4.025	85.206	8.489	11.956
685	93	15	11	P	S	97.311	-4.497	-2.828	-1.230	4.941	2.899	1.261	85.254	11.961	8.486
686	93	15	11	P	S	100.158	-4.723	2.096	-0.986	5.216	-2.138	1.006	85.114	11.951	8.496
687	93	15	11	P	S	100.893	-1.485	3.250	-4.257	1.530	-3.553	4.653	85.149	8.492	11.954
688	93	15	11	P	W	19.842	-4.723	-2.096	-0.986	5.216	2.138	1.006	85.114	8.496	11.951
689	93	15	11	P	W	22.689	-4.497	2.828	-1.230	4.941	-2.899	1.261	85.254	11.961	8.486
690	93	15	11	P	W	23.413	-2.043	3.969	-3.725	2.130	-4.288	4.025	85.206	11.956	8.489
691	93	15	11	P	W	36.587	-2.043	-3.969	-3.725	2.130	4.288	4.025	85.206	8.489	11.956
692	93	15	11	P	W	37.311	-4.497	-2.828	-1.230	4.941	2.899	1.261	85.254	11.961	8.486
693	93	15	11	P	W	40.158	-4.723	2.096	-0.986	5.216	-2.138	1.006	85.114	11.951	8.496
694	93	15	11	P	W	40.893	-1.485	3.250	-4.257	1.530	-3.553	4.653	85.149	8.492	11.954
695	93	15	11	P	W	79.107	-1.485	-3.250	-4.257	1.530	3.553	4.653	85.149	8.492	11.954
696	93	15	11	P	W	79.842	-4.723	-2.096	-0.986	5.216	2.138	1.006	85.114	8.496	11.951
697	93	15	11	P	W	82.689	-4.497	2.828	-1.230	4.941	-2.899	1.261	85.254	11.961	8.486
698	93	15	11	P	W	83.413	-2.043	3.969	-3.725	2.130	-4.288	4.025	85.206	11.956	8.489
699	93	15	11	P	W	96.587	-2.043	-3.969	-3.725	2.130	4.288	4.025	85.206	8.489	11.956
700	93	15	11	P	W	97.311	-4.497	-2.828	-1.230	4.941	2.899	1.261	85.254	11.961	8.486
701	93	15	11	P	W	100.158	-4.723	2.096	-0.986	5.216	-2.138	1.006	85.114	11.951	8.496
702	96	15	12	C	S	12.520	2.326	-4.042	-3.733	-2.223	4.368	4.034	74.907	8.475	12.910
703	96	15	12	C	S	13.898	-4.228	-1.843	2.892	4.619	1.742	-2.734	74.944	12.905	8.479
704	96	15	12	C	S	16.102	2.853	1.979	-4.194	-2.699	-2.161	4.578	67.573	8.491	13.463
705	96	15	12	C	S	19.107	-1.485	-4.063	-0.099	1.530	4.071	0.099	81.215	8.492	12.576
706	96	15	12	C	S	21.052	1.339	-0.650	-2.844	-1.304	0.689	3.015	81.267	12.581	8.491
707	96	15	12	C	S	23.413	0.481	3.384	-2.042	-0.477	-3.528	2.130	81.302	12.584	8.485
708	96	15	12	C	S	36.587	0.481	-3.384	-2.042	-0.477	3.528	2.130	81.302	12.584	8.485
709	96	15	12	C	S	38.948	1.339	0.650	-2.844	-1.304	-0.689	3.015	81.267	8.491	12.581
710	96	15	12	C	S	40.893	-1.485	4.063	-0.099	1.530	-4.071	0.099	81.215	8.492	12.576
711	96	15	12	C	S	43.898	2.853	-1.979	-4.194	-2.699	2.161	4.578	67.573	13.463	8.491
712	96	15	12	C	S	46.102	-4.228	1.843	2.892	4.619	-1.742	-2.734	88.974	9.921	10.653
713	96	15	12	C	S	47.480	2.326	4.042	-3.733	-2.223	-4.368	4.034	74.907	12.910	8.475
714	96	15	12	C	S	72.520	2.326	-4.042	-3.733	-2.223	4.368	4.034	74.907	8.475	12.910

BL number	Atoms	γ-PC unit cells	WS_2 unit cells	γ-PC origin atom	WS_2 origin atom	Twist-angle (°)	γ-PC strain 1 (%)	γ-PC strain 2 (%)	γ-PC strain 3 (%)	WS_2 strain 1 (%)	WS_2 strain 2 (%)	WS_2 strain 3 (%)	γ (°)	$\lvert\mathbf{a}\rvert$(Å)	$\lvert\mathbf{b}\rvert$(Å)
715	96	15	12	C	S	73.898	-4.228	-1.843	2.892	4.619	1.742	-2.734	88.974	9.921	10.653
716	96	15	12	C	S	76.102	2.853	1.979	-4.194	-2.699	-2.161	4.578	67.573	8.491	13.463
717	96	15	12	C	S	79.107	-1.485	-4.063	-0.099	1.530	4.071	0.099	81.215	8.492	12.576
718	96	15	12	C	S	81.052	1.339	-0.650	-2.844	-1.304	0.689	3.015	81.267	12.581	8.491
719	96	15	12	C	S	83.413	-2.043	3.473	0.481	2.130	-3.440	-0.477	81.275	8.483	12.589
720	96	15	12	C	S	96.587	-2.043	-3.473	0.481	2.130	3.440	-0.477	81.275	12.589	8.483
721	96	15	12	C	S	98.948	1.339	0.650	-2.844	-1.304	-0.689	3.015	81.267	8.491	12.581
722	96	15	12	C	S	100.893	-1.485	4.063	-0.099	1.530	-4.071	0.099	81.215	8.492	12.576
723	96	15	12	C	S	103.898	2.853	-1.979	-4.194	-2.699	2.161	4.578	67.573	13.463	8.491
724	96	15	12	C	S	106.102	-4.228	1.843	2.892	4.619	-1.742	-2.734	74.944	8.479	12.905
725	96	15	12	C	S	107.480	2.326	4.042	-3.733	-2.223	-4.368	4.034	74.907	12.910	8.475
726	96	15	12	C	W	12.520	2.326	-4.042	-3.733	-2.223	4.368	4.034	74.907	8.475	12.910
727	96	15	12	C	W	13.898	-4.228	-1.843	2.892	4.619	1.742	-2.734	74.944	12.905	8.479
728	96	15	12	C	W	16.102	2.853	1.979	-4.194	-2.699	-2.161	4.578	88.816	10.655	9.920
729	96	15	12	C	W	19.107	-1.485	-4.063	-0.099	1.530	4.071	0.099	81.215	8.492	12.576
730	96	15	12	C	W	21.052	1.339	-0.650	-2.844	-1.304	0.689	3.015	81.267	12.581	8.491
731	96	15	12	C	W	23.413	0.481	3.384	-2.042	-0.477	-3.528	2.130	81.302	12.584	8.485
732	96	15	12	C	W	36.587	0.481	-3.384	-2.042	-0.477	3.528	2.130	81.302	12.584	8.485
733	96	15	12	C	W	38.948	1.339	0.650	-2.844	-1.304	-0.689	3.015	81.267	8.491	12.581
734	96	15	12	C	W	40.893	-1.485	4.063	-0.099	1.530	-4.071	0.099	81.215	8.492	12.576
735	96	15	12	C	W	43.898	2.853	-1.979	-4.194	-2.699	2.161	4.578	67.573	13.463	8.491
736	96	15	12	C	W	46.102	-4.228	1.843	2.892	4.619	-1.742	-2.734	74.944	8.479	12.905
737	96	15	12	C	W	47.480	2.326	4.042	-3.733	-2.223	-4.368	4.034	74.907	12.910	8.475
738	96	15	12	C	W	72.520	2.326	-4.042	-3.733	-2.223	4.368	4.034	74.907	8.475	12.910
739	96	15	12	C	W	73.898	-4.228	-1.843	2.892	4.619	1.742	-2.734	88.974	9.921	10.653
740	96	15	12	C	W	76.102	2.853	1.979	-4.194	-2.699	-2.161	4.578	67.573	8.491	13.463
741	96	15	12	C	W	79.107	-1.485	-4.063	-0.099	1.530	4.071	0.099	81.215	8.492	12.576
742	96	15	12	C	W	81.052	1.339	-0.650	-2.844	-1.304	0.689	3.015	81.267	12.581	8.491
743	96	15	12	C	W	83.413	-2.043	3.473	0.481	2.130	-3.440	-0.477	81.275	8.483	12.589
744	96	15	12	C	W	96.587	-2.043	-3.473	0.481	2.130	3.440	-0.477	81.275	12.589	8.483
745	96	15	12	C	W	98.948	1.339	0.650	-2.844	-1.304	-0.689	3.015	81.267	8.491	12.581
746	96	15	12	C	W	100.893	-1.485	4.063	-0.099	1.530	-4.071	0.099	81.215	8.492	12.576
747	96	15	12	C	W	103.898	2.853	-1.979	-4.194	-2.699	2.161	4.578	88.816	10.655	9.920
748	96	15	12	C	W	106.102	-4.228	1.843	2.892	4.619	-1.742	-2.734	74.944	8.479	12.905
749	96	15	12	C	W	107.480	2.326	4.042	-3.733	-2.223	-4.368	4.034	74.907	12.910	8.475
750	96	15	12	P	S	13.898	-4.228	-1.843	2.892	4.619	1.742	-2.734	88.974	9.921	10.653
751	96	15	12	P	S	19.107	-1.485	-4.063	-0.099	1.530	4.071	0.099	81.215	8.492	12.576
752	96	15	12	P	S	21.052	1.339	-0.650	-2.844	-1.304	0.689	3.015	81.267	12.581	8.491
753	96	15	12	P	S	23.413	0.481	3.384	-2.042	-0.477	-3.528	2.130	81.302	12.584	8.485
754	96	15	12	P	S	36.587	0.481	-3.384	-2.042	-0.477	3.528	2.130	81.302	12.584	8.485
755	96	15	12	P	S	38.948	1.339	0.650	-2.844	-1.304	-0.689	3.015	81.267	8.491	12.581
756	96	15	12	P	S	40.893	-1.485	4.063	-0.099	1.530	-4.071	0.099	81.215	8.492	12.576
757	96	15	12	P	S	43.898	2.853	-1.979	-4.194	-2.699	2.161	4.578	88.816	10.655	9.920
758	96	15	12	P	S	46.102	-4.228	1.843	2.892	4.619	-1.742	-2.734	88.974	9.921	10.653
759	96	15	12	P	S	73.898	-4.228	-1.843	2.892	4.619	1.742	-2.734	88.974	9.921	10.653
760	96	15	12	P	S	76.102	2.853	1.979	-4.194	-2.699	-2.161	4.578	88.816	10.655	9.920
761	96	15	12	P	S	79.107	-1.485	-4.063	-0.099	1.530	4.071	0.099	81.215	8.492	12.576
762	96	15	12	P	S	81.052	1.339	-0.650	-2.844	-1.304	0.689	3.015	81.267	12.581	8.491
763	96	15	12	P	S	83.413	-2.043	3.473	0.481	2.130	-3.440	-0.477	81.275	8.483	12.589
764	96	15	12	P	S	96.587	-2.043	-3.473	0.481	2.130	3.440	-0.477	81.275	12.589	8.483
765	96	15	12	P	S	98.948	1.339	0.650	-2.844	-1.304	-0.689	3.015	81.267	8.491	12.581

BL number	Atoms	γ-PC unit cells	WS_2 unit cells	γ-PC origin atom	WS_2 origin atom	Twist-angle (°)	γ-PC strain 1 (%)	γ-PC strain 2 (%)	γ-PC strain 3 (%)	WS_2 strain 1 (%)	WS_2 strain 2 (%)	WS_2 strain 3 (%)	γ (°)	|**a**|(Å)	|**b**|(Å)
766	96	15	12	P	S	100.893	-1.485	4.063	-0.099	1.530	-4.071	0.099	81.215	8.492	12.576
767	96	15	12	P	S	106.102	-4.228	1.843	2.892	4.619	-1.742	-2.734	88.974	9.921	10.653
768	96	15	12	P	W	13.898	-4.228	-1.843	2.892	4.619	1.742	-2.734	88.974	9.921	10.653
769	96	15	12	P	W	16.102	2.853	1.979	-4.194	-2.699	-2.161	4.578	88.816	10.655	9.920
770	96	15	12	P	W	19.107	-1.485	-4.063	-0.099	1.530	4.071	0.099	81.215	8.492	12.576
771	96	15	12	P	W	21.052	1.339	-0.650	-2.844	-1.304	0.689	3.015	81.267	12.581	8.491
772	96	15	12	P	W	23.413	0.481	3.384	-2.042	-0.477	-3.528	2.130	81.302	12.584	8.485
773	96	15	12	P	W	36.587	0.481	-3.384	-2.042	-0.477	3.528	2.130	81.302	12.584	8.485
774	96	15	12	P	W	38.948	1.339	0.650	-2.844	-1.304	-0.689	3.015	81.267	8.491	12.581
775	96	15	12	P	W	40.893	-1.485	4.063	-0.099	1.530	-4.071	0.099	81.215	8.492	12.576
776	96	15	12	P	W	79.107	-1.485	-4.063	-0.099	1.530	4.071	0.099	81.215	8.492	12.576
777	96	15	12	P	W	81.052	1.339	-0.650	-2.844	-1.304	0.689	3.015	81.267	12.581	8.491
778	96	15	12	P	W	83.413	-2.043	3.473	0.481	2.130	-3.440	-0.477	81.275	8.483	12.589
779	96	15	12	P	W	96.587	-2.043	-3.473	0.481	2.130	3.440	-0.477	81.275	12.589	8.483
780	96	15	12	P	W	98.948	1.339	0.650	-2.844	-1.304	-0.689	3.015	81.267	8.491	12.581
781	96	15	12	P	W	100.893	-1.485	4.063	-0.099	1.530	-4.071	0.099	81.215	8.492	12.576
782	96	15	12	P	W	103.898	2.853	-1.979	-4.194	-2.699	2.161	4.578	88.816	10.655	9.920
783	98	14	14	C	S	13.174	5.011	-4.331	5.011	-4.554	3.936	-4.555	76.161	10.662	10.662
784	98	14	14	C	S	17.897	5.011	4.331	5.011	-4.554	-3.936	-4.555	76.066	10.664	10.664
785	98	14	14	C	S	42.103	5.011	-4.331	5.011	-4.554	3.936	-4.555	76.066	10.664	10.664
786	98	14	14	C	S	46.827	5.011	4.331	5.011	-4.554	-3.936	-4.555	76.161	10.662	10.662
787	98	14	14	C	S	73.174	5.011	-4.331	5.011	-4.554	3.936	-4.555	76.161	10.662	10.662
788	98	14	14	C	S	77.897	5.011	4.331	5.011	-4.554	-3.936	-4.555	76.066	10.664	10.664
789	98	14	14	C	S	102.104	5.011	-4.331	5.011	-4.554	3.936	-4.555	76.066	10.664	10.664
790	98	14	14	C	S	106.827	5.011	4.331	5.011	-4.554	-3.936	-4.555	76.161	10.662	10.662
791	98	14	14	C	W	13.174	5.011	-4.331	5.011	-4.554	3.936	-4.555	76.161	10.662	10.662
792	98	14	14	C	W	17.897	5.011	4.331	5.011	-4.554	-3.936	-4.555	76.066	10.664	10.664
793	98	14	14	C	W	42.103	5.011	-4.331	5.011	-4.554	3.936	-4.555	76.066	10.664	10.664
794	98	14	14	C	W	46.827	5.011	4.331	5.011	-4.554	-3.936	-4.555	76.161	10.662	10.662
795	98	14	14	C	W	73.174	5.011	-4.331	5.011	-4.554	3.936	-4.555	76.161	10.662	10.662
796	98	14	14	C	W	77.897	5.011	4.331	5.011	-4.554	-3.936	-4.555	76.066	10.664	10.664
797	98	14	14	C	W	102.104	5.011	-4.331	5.011	-4.554	3.936	-4.555	76.066	10.664	10.664
798	98	14	14	C	W	106.827	5.011	4.331	5.011	-4.554	-3.936	-4.555	76.161	10.662	10.662
799	98	14	14	P	S	13.174	5.011	-4.331	5.011	-4.554	3.936	-4.555	76.161	10.662	10.662
800	98	14	14	P	S	17.897	5.011	4.331	5.011	-4.554	-3.936	-4.555	76.066	10.664	10.664
801	98	14	14	P	S	42.103	5.011	-4.331	5.011	-4.554	3.936	-4.555	76.066	10.664	10.664
802	98	14	14	P	S	46.827	5.011	4.331	5.011	-4.554	-3.936	-4.555	76.161	10.662	10.662
803	98	14	14	P	S	73.174	5.011	-4.331	5.011	-4.554	3.936	-4.555	76.161	10.662	10.662
804	98	14	14	P	S	77.897	5.011	4.331	5.011	-4.554	-3.936	-4.555	76.066	10.664	10.664
805	98	14	14	P	S	102.104	5.011	-4.331	5.011	-4.554	3.936	-4.555	76.066	10.664	10.664
806	98	14	14	P	S	106.827	5.011	4.331	5.011	-4.554	-3.936	-4.555	76.161	10.662	10.662
807	98	14	14	P	W	13.174	5.011	-4.331	5.011	-4.554	3.936	-4.555	76.161	10.662	10.662
808	98	14	14	P	W	17.897	5.011	4.331	5.011	-4.554	-3.936	-4.555	76.066	10.664	10.664
809	98	14	14	P	W	42.103	5.011	-4.331	5.011	-4.554	3.936	-4.555	76.066	10.664	10.664
810	98	14	14	P	W	46.827	5.011	4.331	5.011	-4.554	-3.936	-4.555	76.161	10.662	10.662
811	98	14	14	P	W	73.174	5.011	-4.331	5.011	-4.554	3.936	-4.555	76.161	10.662	10.662
812	98	14	14	P	W	77.897	5.011	4.331	5.011	-4.554	-3.936	-4.555	76.066	10.664	10.664
813	98	14	14	P	W	102.104	5.011	-4.331	5.011	-4.554	3.936	-4.555	76.066	10.664	10.664
814	98	14	14	P	W	106.827	5.011	4.331	5.011	-4.554	-3.936	-4.555	76.161	10.662	10.662
815	99	15	13	C	S	13.174	5.011	-4.042	-2.324	-4.554	4.239	2.437	80.601	13.151	8.477
816	99	15	13	C	S	17.897	5.011	4.042	-2.324	-4.554	-4.239	2.437	80.304	8.499	13.129

BL number	Atoms	γ-PC unit cells	WS_2 unit cells	γ-PC origin atom	WS_2 origin atom	Twist-angle (°)	γ-PC strain 1 (%)	γ-PC strain 2 (%)	γ-PC strain 3 (%)	WS_2 strain 1 (%)	WS_2 strain 2 (%)	WS_2 strain 3 (%)	γ (°)	|**a**|(Å)	|**b**|(Å)
817	99	15	13	C	S	19.107	-1.485	-4.875	4.060	1.530	4.509	-3.755	78.426	7.982	14.058
818	99	15	13	C	S	23.413	-2.043	2.977	4.688	2.130	-2.721	-4.286	78.222	14.072	7.983
819	99	15	13	C	S	36.587	-2.043	-2.976	4.688	2.130	2.721	-4.286	78.222	14.072	7.983
820	99	15	13	C	S	40.893	-1.485	4.875	4.060	1.530	-4.509	-3.755	78.426	14.058	7.982
821	99	15	13	C	S	42.103	5.011	-4.042	-2.324	-4.554	4.239	2.437	80.304	13.129	8.499
822	99	15	13	C	S	46.827	5.011	4.042	-2.324	-4.554	-4.239	2.437	80.602	13.151	8.477
823	99	15	13	C	S	73.174	5.011	-4.042	-2.324	-4.554	4.239	2.437	80.602	13.151	8.477
824	99	15	13	C	S	77.897	5.011	4.042	-2.324	-4.554	-4.239	2.437	80.304	8.499	13.129
825	99	15	13	C	S	79.107	-1.485	-4.875	4.060	1.530	4.509	-3.755	74.299	8.492	13.448
826	99	15	13	C	S	83.413	-2.043	2.977	4.688	2.130	-2.721	-4.286	78.222	14.072	7.983
827	99	15	13	C	S	96.587	-2.043	-2.976	4.688	2.130	2.721	-4.286	78.222	14.072	7.983
828	99	15	13	C	S	100.893	-1.485	4.875	4.060	1.530	-4.509	-3.755	74.299	8.492	13.448
829	99	15	13	C	S	102.104	5.011	-4.042	-2.324	-4.554	4.239	2.437	80.304	13.129	8.499
830	99	15	13	C	S	106.827	5.011	4.042	-2.324	-4.554	-4.239	2.437	80.602	13.151	8.477
831	99	15	13	C	W	13.174	5.011	-4.042	-2.324	-4.554	4.239	2.437	80.601	13.151	8.477
832	99	15	13	C	W	17.897	5.011	4.042	-2.324	-4.554	-4.239	2.437	80.304	8.499	13.129
833	99	15	13	C	W	19.107	-1.485	-4.875	4.060	1.530	4.509	-3.755	74.299	8.492	13.448
834	99	15	13	C	W	40.893	-1.485	4.875	4.060	1.530	-4.509	-3.755	74.299	8.492	13.448
835	99	15	13	C	W	42.103	5.011	-4.042	-2.324	-4.554	4.239	2.437	80.304	13.129	8.499
836	99	15	13	C	W	46.827	5.011	4.042	-2.324	-4.554	-4.239	2.437	80.602	13.151	8.477
837	99	15	13	C	W	73.174	5.011	-4.042	-2.324	-4.554	4.239	2.437	80.602	13.151	8.477
838	99	15	13	C	W	77.897	5.011	4.042	-2.324	-4.554	-4.239	2.437	80.304	8.499	13.129
839	99	15	13	C	W	79.107	-1.485	-4.875	4.060	1.530	4.509	-3.755	74.299	8.492	13.448
840	99	15	13	C	W	100.893	-1.485	4.875	4.060	1.530	-4.509	-3.755	74.299	8.492	13.448
841	99	15	13	C	W	102.104	5.011	-4.042	-2.324	-4.554	4.239	2.437	80.304	13.129	8.499
842	99	15	13	C	W	106.827	5.011	4.042	-2.324	-4.554	-4.239	2.437	80.602	13.151	8.477
843	99	15	13	P	S	13.174	5.011	-4.042	-2.324	-4.554	4.239	2.437	80.601	13.151	8.477
844	99	15	13	P	S	17.897	5.011	4.042	-2.324	-4.554	-4.239	2.437	80.304	8.499	13.129
845	99	15	13	P	S	19.107	-1.485	-4.875	4.060	1.530	4.509	-3.755	78.426	7.982	14.058
846	99	15	13	P	S	23.413	-2.043	2.977	4.688	2.130	-2.721	-4.286	78.222	14.072	7.983
847	99	15	13	P	S	36.587	-2.043	-2.976	4.688	2.130	2.721	-4.286	78.222	14.072	7.983
848	99	15	13	P	S	40.893	-1.485	4.875	4.060	1.530	-4.509	-3.755	78.426	14.058	7.982
849	99	15	13	P	S	42.103	5.011	-4.042	-2.324	-4.554	4.239	2.437	80.304	13.129	8.499
850	99	15	13	P	S	46.827	5.011	4.042	-2.324	-4.554	-4.239	2.437	80.602	13.151	8.477
851	99	15	13	P	S	73.174	5.011	-4.042	-2.324	-4.554	4.239	2.437	80.602	13.151	8.477
852	99	15	13	P	S	77.897	5.011	4.042	-2.324	-4.554	-4.239	2.437	80.304	8.499	13.129
853	99	15	13	P	S	79.107	-1.485	-4.875	4.060	1.530	4.509	-3.755	74.299	8.492	13.448
854	99	15	13	P	S	83.413	-2.043	2.977	4.688	2.130	-2.721	-4.286	78.222	14.072	7.983
855	99	15	13	P	S	96.587	-2.043	-2.976	4.688	2.130	2.721	-4.286	78.222	14.072	7.983
856	99	15	13	P	S	100.893	-1.485	4.875	4.060	1.530	-4.509	-3.755	78.426	14.058	7.982
857	99	15	13	P	S	102.104	5.011	-4.042	-2.324	-4.554	4.239	2.437	80.304	13.129	8.499
858	99	15	13	P	S	106.827	5.011	4.042	-2.324	-4.554	-4.239	2.437	80.602	13.151	8.477
859	99	15	13	P	W	13.174	5.011	-4.042	-2.324	-4.554	4.239	2.437	80.601	13.151	8.477
860	99	15	13	P	W	17.897	5.011	4.042	-2.324	-4.554	-4.239	2.437	80.304	8.499	13.129
861	99	15	13	P	W	19.107	-1.485	-4.875	4.060	1.530	4.509	-3.755	74.299	8.492	13.448
862	99	15	13	P	W	42.103	5.011	-4.042	-2.324	-4.554	4.239	2.437	80.304	13.129	8.499
863	99	15	13	P	W	46.827	5.011	4.042	-2.324	-4.554	-4.239	2.437	80.602	13.151	8.477
864	99	15	13	P	W	73.174	5.011	-4.042	-2.324	-4.554	4.239	2.437	80.602	13.151	8.477
865	99	15	13	P	W	77.897	5.011	4.042	-2.324	-4.554	-4.239	2.437	80.304	8.499	13.129
866	99	15	13	P	W	100.893	-1.485	4.875	4.060	1.530	-4.509	-3.755	74.299	8.492	13.448
867	99	15	13	P	W	102.104	5.011	-4.042	-2.324	-4.554	4.239	2.437	80.304	13.129	8.499

BL number	Atoms	γ-PC unit cells	WS_2 unit cells	γ-PC origin atom	WS_2 origin atom	Twist-angle (°)	γ-PC strain 1 (%)	γ-PC strain 2 (%)	γ-PC strain 3 (%)	WS_2 strain 1 (%)	WS_2 strain 2 (%)	WS_2 strain 3 (%)	γ (°)	$\lvert\mathbf{a}\rvert$(Å)	$\lvert\mathbf{b}\rvert$(Å)
868	99	15	13	P	W	106.827	5.011	4.042	-2.324	-4.554	-4.239	2.437	80.602	13.151	8.477
869	100	16	12	C	S	10.893	-1.983	-3.979	-2.733	2.064	4.209	2.891	73.949	11.446	9.914
870	100	16	12	C	S	13.898	-4.228	1.152	-0.414	4.619	-1.162	0.417	73.871	9.921	11.447
871	100	16	12	C	S	15.608	-2.145	4.035	-2.572	2.241	-4.254	2.712	73.869	9.916	11.449
872	100	16	12	C	S	44.392	-2.145	-4.035	-2.572	2.241	4.254	2.712	73.869	11.449	9.916
873	100	16	12	C	S	46.102	-4.228	-1.152	-0.414	4.619	1.162	0.417	73.871	9.921	11.447
874	100	16	12	C	S	49.107	-1.983	3.979	-2.733	2.064	-4.209	2.891	73.949	9.914	11.446
875	100	16	12	C	S	70.893	-1.983	-3.979	-2.733	2.064	4.209	2.891	73.949	11.446	9.914
876	100	16	12	C	S	73.898	-0.414	1.106	-4.228	0.417	-1.208	4.619	73.900	11.445	9.921
877	100	16	12	C	S	75.609	-2.145	4.035	-2.572	2.241	-4.254	2.712	73.869	9.916	11.449
878	100	16	12	C	S	104.392	-2.145	-4.035	-2.572	2.241	4.254	2.712	73.869	11.449	9.916
879	100	16	12	C	S	106.102	-4.228	-1.152	-0.414	4.619	1.162	0.417	73.871	9.921	11.447
880	100	16	12	C	S	109.107	-1.983	3.979	-2.733	2.064	-4.209	2.891	73.949	9.914	11.446
881	100	16	12	C	W	10.893	-1.983	-3.979	-2.733	2.064	4.209	2.891	73.949	11.446	9.914
882	100	16	12	C	W	13.898	-4.228	1.152	-0.414	4.619	-1.162	0.417	73.871	9.921	11.447
883	100	16	12	C	W	15.608	-2.145	4.035	-2.572	2.241	-4.254	2.712	73.869	9.916	11.449
884	100	16	12	C	W	44.392	-2.145	-4.035	-2.572	2.241	4.254	2.712	73.869	11.449	9.916
885	100	16	12	C	W	46.102	-4.228	-1.152	-0.414	4.619	1.162	0.417	73.871	9.921	11.447
886	100	16	12	C	W	49.107	-1.983	3.979	-2.733	2.064	-4.209	2.891	73.949	9.914	11.446
887	100	16	12	C	W	70.893	-1.983	-3.979	-2.733	2.064	4.209	2.891	73.949	11.446	9.914
888	100	16	12	C	W	73.898	-4.228	1.152	-0.414	4.619	-1.162	0.417	73.871	9.921	11.447
889	100	16	12	C	W	75.609	-2.145	4.035	-2.572	2.241	-4.254	2.712	73.869	9.916	11.449
890	100	16	12	C	W	104.392	-2.145	-4.035	-2.572	2.241	4.254	2.712	73.869	11.449	9.916
891	100	16	12	C	W	106.102	-0.414	-1.106	-4.228	0.417	1.208	4.619	73.900	11.445	9.921
892	100	16	12	C	W	109.107	-1.983	3.979	-2.733	2.064	-4.209	2.891	73.949	9.914	11.446
893	100	16	12	P	S	10.893	-1.983	-3.979	-2.733	2.064	4.209	2.891	73.949	11.446	9.914
894	100	16	12	P	S	13.898	-4.228	1.152	-0.414	4.619	-1.162	0.417	73.871	9.921	11.447
895	100	16	12	P	S	15.608	-2.145	4.035	-2.572	2.241	-4.254	2.712	73.869	9.916	11.449
896	100	16	12	P	S	44.392	-2.145	-4.035	-2.572	2.241	4.254	2.712	73.869	11.449	9.916
897	100	16	12	P	S	46.102	-4.228	-1.152	-0.414	4.619	1.162	0.417	73.871	9.921	11.447
898	100	16	12	P	S	49.107	-1.983	3.979	-2.733	2.064	-4.209	2.891	73.949	9.914	11.446
899	100	16	12	P	S	70.893	-1.983	-3.979	-2.733	2.064	4.209	2.891	73.949	11.446	9.914
900	100	16	12	P	S	73.898	-4.228	1.152	-0.414	4.619	-1.162	0.417	73.871	9.921	11.447
901	100	16	12	P	S	75.609	-2.145	4.035	-2.572	2.241	-4.254	2.712	73.869	9.916	11.449
902	100	16	12	P	S	104.392	-2.145	-4.035	-2.572	2.241	4.254	2.712	73.869	11.449	9.916
903	100	16	12	P	S	106.102	-0.414	-1.106	-4.228	0.417	1.208	4.619	73.900	11.445	9.921
904	100	16	12	P	S	109.107	-1.983	3.979	-2.733	2.064	-4.209	2.891	73.949	9.914	11.446
905	100	16	12	P	W	10.893	-1.983	-3.979	-2.733	2.064	4.209	2.891	73.949	11.446	9.914
906	100	16	12	P	W	13.898	-4.228	1.152	-0.414	4.619	-1.162	0.417	73.871	9.921	11.447
907	100	16	12	P	W	15.608	-2.145	4.035	-2.572	2.241	-4.254	2.712	73.869	9.916	11.449
908	100	16	12	P	W	44.392	-2.145	-4.035	-2.572	2.241	4.254	2.712	73.869	11.449	9.916
909	100	16	12	P	W	46.102	-4.228	-1.152	-0.414	4.619	1.162	0.417	73.871	9.921	11.447
910	100	16	12	P	W	49.107	-1.983	3.979	-2.733	2.064	-4.209	2.891	73.949	9.914	11.446
911	100	16	12	P	W	70.893	-1.983	-3.979	-2.733	2.064	4.209	2.891	73.949	11.446	9.914
912	100	16	12	P	W	73.898	-0.414	1.106	-4.228	0.417	-1.208	4.619	73.900	11.445	9.921
913	100	16	12	P	W	75.609	-2.145	4.035	-2.572	2.241	-4.254	2.712	73.869	9.916	11.449
914	100	16	12	P	W	104.392	-2.145	-4.035	-2.572	2.241	4.254	2.712	73.869	11.449	9.916
915	100	16	12	P	W	106.102	-4.228	-1.152	-0.414	4.619	1.162	0.417	73.871	9.921	11.447
916	100	16	12	P	W	109.107	-1.983	3.979	-2.733	2.064	-4.209	2.891	73.949	9.914	11.446
917	103	16	13	C	S	13.898	-0.414	0.000	-0.414	0.417	0.000	0.417	60.000	11.445	11.445
918	103	16	13	C	S	46.102	-0.414	0.000	-0.414	0.417	0.000	0.417	60.000	11.445	11.445

BL number	Atoms	γ-PC unit cells	WS_2 unit cells	γ-PC origin atom	WS_2 origin atom	Twist-angle (°)	γ-PC strain 1 (%)	γ-PC strain 2 (%)	γ-PC strain 3 (%)	WS_2 strain 1 (%)	WS_2 strain 2 (%)	WS_2 strain 3 (%)	γ (°)	|**a**|(Å)	|**b**|(Å)
919	103	16	13	C	S	73.898	-0.414	0.000	-0.414	0.417	0.000	0.417	60.000	11.445	11.445
920	103	16	13	C	S	106.102	-0.414	0.000	-0.414	0.417	0.000	0.417	60.000	11.445	11.445
921	103	16	13	C	W	13.898	-0.414	0.000	-0.414	0.417	0.000	0.417	60.000	11.445	11.445
922	103	16	13	C	W	46.102	-0.414	0.000	-0.414	0.417	0.000	0.417	60.000	11.445	11.445
923	103	16	13	C	W	73.898	-0.414	0.000	-0.414	0.417	0.000	0.417	60.000	11.445	11.445
924	103	16	13	C	W	106.102	-0.414	0.000	-0.414	0.417	0.000	0.417	60.000	11.445	11.445
925	103	16	13	P	S	46.102	-0.414	0.000	-0.414	0.417	0.000	0.417	60.000	11.445	11.445
926	103	16	13	P	S	73.898	-0.414	0.000	-0.414	0.417	0.000	0.417	60.000	11.445	11.445
927	103	16	13	P	W	13.898	-0.414	0.000	-0.414	0.417	0.000	0.417	60.000	11.445	11.445
928	103	16	13	P	W	106.102	-0.414	0.000	-0.414	0.417	0.000	0.417	60.000	11.445	11.445
929	104	17	12	C	S	9.515	-4.497	-3.618	-3.055	4.941	3.853	3.254	71.701	11.961	9.909
930	104	17	12	C	S	13.898	-4.228	3.795	-3.331	4.619	-4.065	3.568	71.693	9.921	11.946
931	104	17	12	C	S	46.102	-4.228	-3.795	-3.331	4.619	4.065	3.568	71.693	9.921	11.946
932	104	17	12	C	S	50.485	-4.497	3.618	-3.055	4.941	-3.853	3.254	71.701	11.961	9.909
933	104	17	12	C	S	69.515	-4.497	-3.618	-3.055	4.941	3.853	3.254	71.701	11.961	9.909
934	104	17	12	C	S	73.898	-4.228	3.795	-3.331	4.619	-4.065	3.568	71.693	9.921	11.946
935	104	17	12	C	S	106.102	-4.228	-3.795	-3.331	4.619	4.065	3.568	71.693	9.921	11.946
936	104	17	12	C	S	110.485	-4.497	3.618	-3.055	4.941	-3.853	3.254	71.701	11.961	9.909
937	104	17	12	C	W	9.515	-4.497	-3.618	-3.055	4.941	3.853	3.254	71.701	11.961	9.909
938	104	17	12	C	W	13.898	-4.228	3.795	-3.331	4.619	-4.065	3.568	71.693	9.921	11.946
939	104	17	12	C	W	46.102	-4.228	-3.795	-3.331	4.619	4.065	3.568	71.693	9.921	11.946
940	104	17	12	C	W	50.485	-4.497	3.618	-3.055	4.941	-3.853	3.254	71.701	11.961	9.909
941	104	17	12	C	W	69.515	-4.497	-3.618	-3.055	4.941	3.853	3.254	71.701	11.961	9.909
942	104	17	12	C	W	73.898	-4.228	3.795	-3.331	4.619	-4.065	3.568	71.693	9.921	11.946
943	104	17	12	C	W	106.102	-4.228	-3.795	-3.331	4.619	4.065	3.568	71.693	9.921	11.946
944	104	17	12	C	W	110.485	-4.497	3.618	-3.055	4.941	-3.853	3.254	71.701	11.961	9.909
945	104	17	12	P	S	9.515	-4.497	-3.618	-3.055	4.941	3.853	3.254	71.701	11.961	9.909
946	104	17	12	P	S	13.898	-4.228	3.795	-3.331	4.619	-4.065	3.568	71.693	9.921	11.946
947	104	17	12	P	S	46.102	-4.228	-3.795	-3.331	4.619	4.065	3.568	71.693	9.921	11.946
948	104	17	12	P	S	50.485	-4.497	3.618	-3.055	4.941	-3.853	3.254	71.701	11.961	9.909
949	104	17	12	P	S	69.515	-4.497	-3.618	-3.055	4.941	3.853	3.254	71.701	11.961	9.909
950	104	17	12	P	S	73.898	-4.228	3.795	-3.331	4.619	-4.065	3.568	71.693	9.921	11.946
951	104	17	12	P	S	106.102	-4.228	-3.795	-3.331	4.619	4.065	3.568	71.693	9.921	11.946
952	104	17	12	P	S	110.485	-4.497	3.618	-3.055	4.941	-3.853	3.254	71.701	11.961	9.909
953	104	17	12	P	W	9.515	-4.497	-3.618	-3.055	4.941	3.853	3.254	71.701	11.961	9.909
954	104	17	12	P	W	13.898	-4.228	3.795	-3.331	4.619	-4.065	3.568	71.693	9.921	11.946
955	104	17	12	P	W	46.102	-4.228	-3.795	-3.331	4.619	4.065	3.568	71.693	9.921	11.946
956	104	17	12	P	W	50.485	-4.497	3.618	-3.055	4.941	-3.853	3.254	71.701	11.961	9.909
957	104	17	12	P	W	69.515	-4.497	-3.618	-3.055	4.941	3.853	3.254	71.701	11.961	9.909
958	104	17	12	P	W	73.898	-4.228	3.795	-3.331	4.619	-4.065	3.568	71.693	9.921	11.946
959	104	17	12	P	W	106.102	-4.228	-3.795	-3.331	4.619	4.065	3.568	71.693	9.921	11.946
960	104	17	12	P	W	110.485	-4.497	3.618	-3.055	4.941	-3.853	3.254	71.701	11.961	9.909
961	105	15	15	C	S	27.796	5.011	-4.042	5.011	-4.554	3.674	-4.555	90.000	10.878	10.870
962	105	15	15	C	S	32.204	5.011	4.042	5.011	-4.554	-3.674	-4.555	90.000	10.878	10.870
963	105	15	15	C	S	87.796	5.011	-4.042	5.011	-4.554	3.674	-4.555	90.000	10.878	10.870
964	105	15	15	C	S	92.204	5.011	4.042	5.011	-4.554	-3.674	-4.555	90.000	10.878	10.870
965	105	15	15	C	W	27.796	5.011	-4.042	5.011	-4.554	3.674	-4.555	90.000	10.878	10.870
966	105	15	15	C	W	32.204	5.011	4.042	5.011	-4.554	-3.674	-4.555	90.000	10.878	10.870
967	105	15	15	C	W	87.796	5.011	-4.042	5.011	-4.554	3.674	-4.555	90.000	10.878	10.870
968	105	15	15	C	W	92.204	5.011	4.042	5.011	-4.554	-3.674	-4.555	90.000	10.878	10.870
969	105	15	15	P	S	27.796	5.011	-4.042	5.011	-4.554	3.674	-4.555	90.000	10.878	10.870

BL number	Atoms	γ-PC unit cells	WS_2 unit cells	γ-PC origin atom	WS_2 origin atom	Twist-angle (°)	γ-PC strain 1 (%)	γ-PC strain 2 (%)	γ-PC strain 3 (%)	WS_2 strain 1 (%)	WS_2 strain 2 (%)	WS_2 strain 3 (%)	γ (°)	|**a**|(Å)	|**b**|(Å)
970	105	15	15	P	S	32.204	5.011	4.042	5.011	-4.554	-3.674	-4.555	90.000	10.878	10.870
971	105	15	15	P	S	87.796	5.011	-4.042	5.011	-4.554	3.674	-4.555	90.000	10.878	10.870
972	105	15	15	P	S	92.204	5.011	4.042	5.011	-4.554	-3.674	-4.555	90.000	10.878	10.870
973	105	15	15	P	W	27.796	5.011	-4.042	5.011	-4.554	3.674	-4.555	90.000	10.878	10.870
974	105	15	15	P	W	32.204	5.011	4.042	5.011	-4.554	-3.674	-4.555	90.000	10.878	10.870
975	105	15	15	P	W	87.796	5.011	-4.042	5.011	-4.554	3.674	-4.555	90.000	10.878	10.870
976	105	15	15	P	W	92.204	5.011	4.042	5.011	-4.554	-3.674	-4.555	90.000	10.878	10.870
977	106	16	14	C	S	0.000	5.011	0.000	-1.865	-4.554	0.000	1.938	90.000	5.226	22.557
978	106	16	14	C	S	60.000	5.011	0.000	-1.865	-4.554	0.000	1.938	90.000	5.226	22.557
979	106	16	14	C	W	0.000	5.011	0.000	-1.865	-4.554	0.000	1.938	90.000	5.226	22.557
980	106	16	14	C	W	60.000	5.011	0.000	-1.865	-4.554	0.000	1.938	90.000	5.226	22.557
981	106	16	14	P	S	0.000	5.011	0.000	-1.865	-4.554	0.000	1.938	90.000	5.226	22.557
982	106	16	14	P	W	60.000	5.011	0.000	-1.865	-4.554	0.000	1.938	90.000	5.226	22.557
983	106	16	14	C	S	12.520	2.326	-3.498	0.605	-2.223	3.456	-0.597	75.048	10.646	11.449
984	106	16	14	C	S	13.898	-0.414	-1.106	3.401	0.417	1.035	-3.184	75.033	11.445	10.653
985	106	16	14	C	S	16.102	2.853	2.783	0.100	-2.699	-2.778	-0.100	74.954	10.655	11.445
986	106	16	14	C	S	16.337	0.353	3.271	2.587	-0.351	-3.110	-2.460	74.992	11.443	10.654
987	106	16	14	C	S	43.663	0.353	-3.271	2.587	-0.351	3.110	-2.460	74.992	10.654	11.443
988	106	16	14	C	S	43.898	2.853	-2.783	0.100	-2.699	2.778	-0.100	74.954	10.655	11.445
989	106	16	14	C	S	46.102	-0.414	1.106	3.401	0.417	-1.035	-3.184	75.033	11.445	10.653
990	106	16	14	C	S	47.480	2.326	3.498	0.605	-2.223	-3.456	-0.597	75.048	11.449	10.646
991	106	16	14	C	S	72.520	2.326	-3.498	0.605	-2.223	3.456	-0.597	75.048	10.646	11.449
992	106	16	14	C	S	73.898	-0.414	-1.106	3.401	0.417	1.035	-3.184	75.033	11.445	10.653
993	106	16	14	C	S	76.102	2.853	2.783	0.100	-2.699	-2.778	-0.100	74.954	10.655	11.445
994	106	16	14	C	S	76.337	0.353	3.271	2.587	-0.351	-3.110	-2.460	74.992	11.443	10.654
995	106	16	14	C	S	103.663	0.353	-3.271	2.587	-0.351	3.110	-2.460	74.992	10.654	11.443
996	106	16	14	C	S	103.898	2.853	-2.783	0.100	-2.699	2.778	-0.100	74.954	10.655	11.445
997	106	16	14	C	S	106.102	-0.414	1.106	3.401	0.417	-1.035	-3.184	75.033	11.445	10.653
998	106	16	14	C	S	107.480	2.326	3.498	0.605	-2.223	-3.456	-0.597	75.048	11.449	10.646
999	106	16	14	C	W	12.520	2.326	-3.498	0.605	-2.223	3.456	-0.597	75.048	10.646	11.449
1000	106	16	14	C	W	13.898	-0.414	-1.106	3.401	0.417	1.035	-3.184	75.033	11.445	10.653
1001	106	16	14	C	W	16.102	2.853	2.783	0.100	-2.699	-2.778	-0.100	74.954	10.655	11.445
1002	106	16	14	C	W	16.337	0.353	3.271	2.587	-0.351	-3.110	-2.460	74.992	11.443	10.654
1003	106	16	14	C	W	43.663	0.353	-3.271	2.587	-0.351	3.110	-2.460	74.992	10.654	11.443
1004	106	16	14	C	W	43.898	2.853	-2.783	0.100	-2.699	2.778	-0.100	74.954	10.655	11.445
1005	106	16	14	C	W	46.102	-0.414	1.106	3.401	0.417	-1.035	-3.184	75.033	11.445	10.653
1006	106	16	14	C	W	47.480	2.326	3.498	0.605	-2.223	-3.456	-0.597	75.048	11.449	10.646
1007	106	16	14	C	W	72.520	2.326	-3.498	0.605	-2.223	3.456	-0.597	75.048	10.646	11.449
1008	106	16	14	C	W	73.898	-0.414	-1.106	3.401	0.417	1.035	-3.184	75.033	11.445	10.653
1009	106	16	14	C	W	76.102	2.853	2.783	0.100	-2.699	-2.778	-0.100	74.954	10.655	11.445
1010	106	16	14	C	W	76.337	0.353	3.271	2.587	-0.351	-3.110	-2.460	74.992	11.443	10.654
1011	106	16	14	C	W	103.663	0.353	-3.271	2.587	-0.351	3.110	-2.460	74.992	10.654	11.443
1012	106	16	14	C	W	103.898	2.853	-2.783	0.100	-2.699	2.778	-0.100	74.954	10.655	11.445
1013	106	16	14	C	W	106.102	-0.414	1.106	3.401	0.417	-1.035	-3.184	75.033	11.445	10.653
1014	106	16	14	C	W	107.480	2.326	3.498	0.605	-2.223	-3.456	-0.597	75.048	11.449	10.646
1015	106	16	14	P	S	12.520	2.326	-3.498	0.605	-2.223	3.456	-0.597	75.048	10.646	11.449
1016	106	16	14	P	S	13.898	-0.414	-1.106	3.401	0.417	1.035	-3.184	75.033	11.445	10.653
1017	106	16	14	P	S	16.102	2.853	2.783	0.100	-2.699	-2.778	-0.100	74.954	10.655	11.445
1018	106	16	14	P	S	16.337	0.353	3.271	2.587	-0.351	-3.110	-2.460	74.992	11.443	10.654
1019	106	16	14	P	S	43.663	0.353	-3.271	2.587	-0.351	3.110	-2.460	74.992	10.654	11.443
1020	106	16	14	P	S	43.898	2.853	-2.783	0.100	-2.699	2.778	-0.100	74.954	10.655	11.445

BL number	Atoms	γ-PC unit cells	WS_2 unit cells	γ-PC origin atom	WS_2 origin atom	Twist-angle (°)	γ-PC strain 1 (%)	γ-PC strain 2 (%)	γ-PC strain 3 (%)	WS_2 strain 1 (%)	WS_2 strain 2 (%)	WS_2 strain 3 (%)	γ (°)	$\lvert\mathbf{a}\rvert$(Å)	$\lvert\mathbf{b}\rvert$(Å)
1021	106	16	14	P	S	46.102	-0.414	1.106	3.401	0.417	-1.035	-3.184	75.033	11.445	10.653
1022	106	16	14	P	S	47.480	2.326	3.498	0.605	-2.223	-3.456	-0.597	75.048	11.449	10.646
1023	106	16	14	P	S	72.520	2.326	-3.498	0.605	-2.223	3.456	-0.597	75.048	10.646	11.449
1024	106	16	14	P	S	73.898	-0.414	-1.106	3.401	0.417	1.035	-3.184	75.033	11.445	10.653
1025	106	16	14	P	S	76.102	2.853	2.783	0.100	-2.699	-2.778	-0.100	74.954	10.655	11.445
1026	106	16	14	P	S	76.337	0.353	3.271	2.587	-0.351	-3.110	-2.460	74.992	11.443	10.654
1027	106	16	14	P	S	103.663	0.353	-3.271	2.587	-0.351	3.110	-2.460	74.992	10.654	11.443
1028	106	16	14	P	S	103.898	2.853	-2.783	0.100	-2.699	2.778	-0.100	74.954	10.655	11.445
1029	106	16	14	P	S	106.102	-0.414	1.106	3.401	0.417	-1.035	-3.184	75.033	11.445	10.653
1030	106	16	14	P	S	107.480	2.326	3.498	0.605	-2.223	-3.456	-0.597	75.048	11.449	10.646
1031	106	16	14	P	W	12.520	2.326	-3.498	0.605	-2.223	3.456	-0.597	75.048	10.646	11.449
1032	106	16	14	P	W	13.898	-0.414	-1.106	3.401	0.417	1.035	-3.184	75.033	11.445	10.653
1033	106	16	14	P	W	16.102	2.853	2.783	0.100	-2.699	-2.778	-0.100	74.954	10.655	11.445
1034	106	16	14	P	W	16.337	0.353	3.271	2.587	-0.351	-3.110	-2.460	74.992	11.443	10.654
1035	106	16	14	P	W	43.663	0.353	-3.271	2.587	-0.351	3.110	-2.460	74.992	10.654	11.443
1036	106	16	14	P	W	43.898	2.853	-2.783	0.100	-2.699	2.778	-0.100	74.954	10.655	11.445
1037	106	16	14	P	W	46.102	-0.414	1.106	3.401	0.417	-1.035	-3.184	75.033	11.445	10.653
1038	106	16	14	P	W	47.480	2.326	3.498	0.605	-2.223	-3.456	-0.597	75.048	11.449	10.646
1039	106	16	14	P	W	72.520	2.326	-3.498	0.605	-2.223	3.456	-0.597	75.048	10.646	11.449
1040	106	16	14	P	W	73.898	-0.414	-1.106	3.401	0.417	1.035	-3.184	75.033	11.445	10.653
1041	106	16	14	P	W	76.102	2.853	2.783	0.100	-2.699	-2.778	-0.100	74.954	10.655	11.445
1042	106	16	14	P	W	76.337	0.353	3.271	2.587	-0.351	-3.110	-2.460	74.992	11.443	10.654
1043	106	16	14	P	W	103.663	0.353	-3.271	2.587	-0.351	3.110	-2.460	74.992	10.654	11.443
1044	106	16	14	P	W	103.898	2.853	-2.783	0.100	-2.699	2.778	-0.100	74.954	10.655	11.445
1045	106	16	14	P	W	106.102	-0.414	1.106	3.401	0.417	-1.035	-3.184	75.033	11.445	10.653
1046	106	16	14	P	W	107.480	2.326	3.498	0.605	-2.223	-3.456	-0.597	75.048	11.449	10.646
1047	109	16	15	C	S	18.613	3.582	-3.788	2.948	-3.342	3.577	-2.784	80.412	7.975	15.512
1048	109	16	15	C	S	21.052	1.339	0.609	5.261	-1.304	-0.551	-4.760	80.407	15.503	7.982
1049	109	16	15	C	S	21.787	5.011	1.895	1.573	-4.554	-1.837	-1.525	80.470	7.983	15.498
1050	109	16	15	C	S	38.213	5.011	-1.895	1.573	-4.554	1.837	-1.525	80.470	7.983	15.498
1051	109	16	15	C	S	40.893	1.980	2.944	4.580	-1.905	-2.697	-4.195	80.413	15.504	7.980
1052	109	16	15	C	S	41.387	3.582	3.788	2.948	-3.342	-3.577	-2.784	80.412	15.512	7.975
1053	109	16	15	C	S	78.613	3.582	-3.788	2.948	-3.342	3.577	-2.784	80.412	7.975	15.512
1054	109	16	15	C	S	81.052	1.339	0.609	5.261	-1.304	-0.551	-4.760	80.407	15.503	7.982
1055	109	16	15	C	S	81.787	5.011	1.895	1.573	-4.554	-1.837	-1.525	80.470	7.983	15.498
1056	109	16	15	C	S	98.213	5.011	-1.895	1.573	-4.554	1.837	-1.525	80.470	7.983	15.498
1057	109	16	15	C	S	100.893	1.980	2.944	4.580	-1.905	-2.697	-4.195	80.413	15.504	7.980
1058	109	16	15	C	S	101.387	3.582	3.788	2.948	-3.342	-3.577	-2.784	80.412	15.512	7.975
1059	109	16	15	C	W	18.613	3.582	-3.788	2.948	-3.342	3.577	-2.784	80.412	7.975	15.512
1060	109	16	15	C	W	21.052	1.339	0.609	5.261	-1.304	-0.551	-4.760	80.407	15.503	7.982
1061	109	16	15	C	W	21.787	5.011	1.895	1.573	-4.554	-1.837	-1.525	80.470	7.983	15.498
1062	109	16	15	C	W	38.213	5.011	-1.895	1.573	-4.554	1.837	-1.525	80.470	7.983	15.498
1063	109	16	15	C	W	40.893	1.980	2.944	4.580	-1.905	-2.697	-4.195	80.413	15.504	7.980
1064	109	16	15	C	W	41.387	3.582	3.788	2.948	-3.342	-3.577	-2.784	80.412	15.512	7.975
1065	109	16	15	C	W	78.613	3.582	-3.788	2.948	-3.342	3.577	-2.784	80.412	7.975	15.512
1066	109	16	15	C	W	81.052	1.339	0.609	5.261	-1.304	-0.551	-4.760	80.407	15.503	7.982
1067	109	16	15	C	W	81.787	5.011	1.895	1.573	-4.554	-1.837	-1.525	80.470	7.983	15.498
1068	109	16	15	C	W	98.213	5.011	-1.895	1.573	-4.554	1.837	-1.525	80.470	7.983	15.498
1069	109	16	15	C	W	100.893	1.980	2.944	4.580	-1.905	-2.697	-4.195	80.413	15.504	7.980
1070	109	16	15	C	W	101.387	3.582	3.788	2.948	-3.342	-3.577	-2.784	80.412	15.512	7.975
1071	109	16	15	P	S	18.613	3.582	-3.788	2.948	-3.342	3.577	-2.784	80.412	7.975	15.512

BL number	Atoms	γ-PC unit cells	WS_2 unit cells	γ-PC origin atom	WS_2 origin atom	Twist-angle (°)	γ-PC strain 1 (%)	γ-PC strain 2 (%)	γ-PC strain 3 (%)	WS_2 strain 1 (%)	WS_2 strain 2 (%)	WS_2 strain 3 (%)	γ (°)	$\lvert\mathbf{a}\rvert$(Å)	$\lvert\mathbf{b}\rvert$(Å)
1072	109	16	15	P	S	21.052	1.339	0.609	5.261	-1.304	-0.551	-4.760	80.407	15.503	7.982
1073	109	16	15	P	S	21.787	5.011	1.895	1.573	-4.554	-1.837	-1.525	80.470	7.983	15.498
1074	109	16	15	P	S	38.213	5.011	-1.895	1.573	-4.554	1.837	-1.525	80.470	7.983	15.498
1075	109	16	15	P	S	40.893	1.980	2.944	4.580	-1.905	-2.697	-4.195	80.413	15.504	7.980
1076	109	16	15	P	S	41.387	3.582	3.788	2.948	-3.342	-3.577	-2.784	80.412	15.512	7.975
1077	109	16	15	P	S	78.613	3.582	-3.788	2.948	-3.342	3.577	-2.784	80.412	7.975	15.512
1078	109	16	15	P	S	81.052	1.339	0.609	5.261	-1.304	-0.551	-4.760	80.407	15.503	7.982
1079	109	16	15	P	S	81.787	5.011	1.895	1.573	-4.554	-1.837	-1.525	80.470	7.983	15.498
1080	109	16	15	P	S	98.213	5.011	-1.895	1.573	-4.554	1.837	-1.525	80.470	7.983	15.498
1081	109	16	15	P	S	100.893	1.980	2.944	4.580	-1.905	-2.697	-4.195	80.413	15.504	7.980
1082	109	16	15	P	S	101.387	3.582	3.788	2.948	-3.342	-3.577	-2.784	80.412	15.512	7.975
1083	109	16	15	P	W	18.613	3.582	-3.788	2.948	-3.342	3.577	-2.784	80.412	7.975	15.512
1084	109	16	15	P	W	21.052	1.339	0.609	5.261	-1.304	-0.551	-4.760	80.407	15.503	7.982
1085	109	16	15	P	W	21.787	5.011	1.895	1.573	-4.554	-1.837	-1.525	80.470	7.983	15.498
1086	109	16	15	P	W	38.213	5.011	-1.895	1.573	-4.554	1.837	-1.525	80.470	7.983	15.498
1087	109	16	15	P	W	40.893	1.980	2.944	4.580	-1.905	-2.697	-4.195	80.413	15.504	7.980
1088	109	16	15	P	W	41.387	3.582	3.788	2.948	-3.342	-3.577	-2.784	80.412	15.512	7.975
1089	109	16	15	P	W	78.613	3.582	-3.788	2.948	-3.342	3.577	-2.784	80.412	7.975	15.512
1090	109	16	15	P	W	81.052	1.339	0.609	5.261	-1.304	-0.551	-4.760	80.407	15.503	7.982
1091	109	16	15	P	W	81.787	5.011	1.895	1.573	-4.554	-1.837	-1.525	80.470	7.983	15.498
1092	109	16	15	P	W	98.213	5.011	-1.895	1.573	-4.554	1.837	-1.525	80.470	7.983	15.498
1093	109	16	15	P	W	100.893	1.980	2.944	4.580	-1.905	-2.697	-4.195	80.413	15.504	7.980
1094	109	16	15	P	W	101.387	3.582	3.788	2.948	-3.342	-3.577	-2.784	80.412	15.512	7.975
1095	110	17	14	C	S	0.000	5.011	1.783	-4.697	-4.554	-1.968	5.184	88.928	7.983	15.240
1096	110	17	14	C	S	1.945	-4.723	1.618	5.043	5.216	-1.470	-4.581	89.118	15.242	7.981
1097	110	17	14	C	S	19.842	-4.723	-1.618	5.043	5.216	1.470	-4.581	89.118	15.242	7.981
1098	110	17	14	C	S	21.787	5.011	1.783	-4.697	-4.554	-1.968	5.184	88.928	7.983	15.240
1099	110	17	14	C	S	22.689	-4.497	3.618	4.769	4.941	-3.303	-4.354	72.985	11.961	10.632
1100	110	17	14	C	S	37.311	-4.497	-3.618	4.769	4.941	3.303	-4.354	72.985	11.961	10.632
1101	110	17	14	C	S	38.213	5.011	-1.783	-4.697	-4.554	1.968	5.184	88.928	7.983	15.240
1102	110	17	14	C	S	40.158	-4.723	1.618	5.043	5.216	-1.470	-4.581	89.118	15.242	7.981
1103	110	17	14	C	S	58.055	-4.723	-1.618	5.043	5.216	1.470	-4.581	89.118	15.242	7.981
1104	110	17	14	C	S	60.000	5.011	1.783	-4.697	-4.554	-1.968	5.184	88.928	7.983	15.240
1105	110	17	14	C	S	61.945	-4.723	1.618	5.043	5.216	-1.470	-4.581	89.118	15.242	7.981
1106	110	17	14	C	S	79.842	-4.723	-1.618	5.043	5.216	1.470	-4.581	89.118	15.242	7.981
1107	110	17	14	C	S	81.787	5.011	1.783	-4.697	-4.554	-1.968	5.184	88.928	7.983	15.240
1108	110	17	14	C	S	82.689	-4.497	3.618	4.769	4.941	-3.303	-4.354	72.985	11.961	10.632
1109	110	17	14	C	S	97.311	-4.497	-3.618	4.769	4.941	3.303	-4.354	72.985	11.961	10.632
1110	110	17	14	C	S	98.213	5.011	-1.783	-4.697	-4.554	1.968	5.184	88.928	7.983	15.240
1111	110	17	14	C	S	100.158	-4.723	1.618	5.043	5.216	-1.470	-4.581	89.118	15.242	7.981
1112	110	17	14	C	S	118.055	-4.723	-1.618	5.043	5.216	1.470	-4.581	89.118	15.242	7.981
1113	110	17	14	C	W	0.000	5.011	1.783	-4.697	-4.554	-1.968	5.184	88.928	7.983	15.240
1114	110	17	14	C	W	1.945	-4.723	1.618	5.043	5.216	-1.470	-4.581	89.118	15.242	7.981
1115	110	17	14	C	W	19.842	-4.723	-1.618	5.043	5.216	1.470	-4.581	89.118	15.242	7.981
1116	110	17	14	C	W	21.787	5.011	1.783	-4.697	-4.554	-1.968	5.184	88.928	7.983	15.240
1117	110	17	14	C	W	22.689	-4.497	3.618	4.769	4.941	-3.303	-4.354	72.985	11.961	10.632
1118	110	17	14	C	W	37.311	-4.497	-3.618	4.769	4.941	3.303	-4.354	72.985	11.961	10.632
1119	110	17	14	C	W	38.213	5.011	-1.783	-4.697	-4.554	1.968	5.184	88.928	7.983	15.240
1120	110	17	14	C	W	40.158	-4.723	1.618	5.043	5.216	-1.470	-4.581	89.118	15.242	7.981
1121	110	17	14	C	W	58.055	-4.723	-1.618	5.043	5.216	1.470	-4.581	89.118	15.242	7.981
1122	110	17	14	C	W	60.000	5.011	1.783	-4.697	-4.554	-1.968	5.184	88.928	7.983	15.240

BL number	Atoms	γ-PC unit cells	WS_2 unit cells	γ-PC origin atom	WS_2 origin atom	Twist-angle (°)	γ-PC strain 1 (%)	γ-PC strain 2 (%)	γ-PC strain 3 (%)	WS_2 strain 1 (%)	WS_2 strain 2 (%)	WS_2 strain 3 (%)	γ (°)	|**a**|(Å)	|**b**|(Å)
1123	110	17	14	C	W	61.945	-4.723	1.618	5.043	5.216	-1.470	-4.581	89.118	15.242	7.981
1124	110	17	14	C	W	79.842	-4.723	-1.618	5.043	5.216	1.470	-4.581	89.118	15.242	7.981
1125	110	17	14	C	W	81.787	5.011	1.783	-4.697	-4.554	-1.968	5.184	88.928	7.983	15.240
1126	110	17	14	C	W	82.689	-4.497	3.618	4.769	4.941	-3.303	-4.354	72.985	11.961	10.632
1127	110	17	14	C	W	97.311	-4.497	-3.618	4.769	4.941	3.303	-4.354	72.985	11.961	10.632
1128	110	17	14	C	W	98.213	5.011	-1.783	-4.697	-4.554	1.968	5.184	88.928	7.983	15.240
1129	110	17	14	C	W	100.158	-4.723	1.618	5.043	5.216	-1.470	-4.581	89.118	15.242	7.981
1130	110	17	14	C	W	118.055	-4.723	-1.618	5.043	5.216	1.470	-4.581	89.118	15.242	7.981
1131	110	17	14	P	S	0.000	5.011	1.783	-4.697	-4.554	-1.968	5.184	88.928	7.983	15.240
1132	110	17	14	P	S	1.945	-4.723	1.618	5.043	5.216	-1.470	-4.581	89.118	15.242	7.981
1133	110	17	14	P	S	19.842	-4.723	-1.618	5.043	5.216	1.470	-4.581	89.118	15.242	7.981
1134	110	17	14	P	S	21.787	5.011	1.783	-4.697	-4.554	-1.968	5.184	88.928	7.983	15.240
1135	110	17	14	P	S	22.689	-4.497	3.618	4.769	4.941	-3.303	-4.354	72.985	11.961	10.632
1136	110	17	14	P	S	37.311	-4.497	-3.618	4.769	4.941	3.303	-4.354	72.985	11.961	10.632
1137	110	17	14	P	S	38.213	5.011	-1.783	-4.697	-4.554	1.968	5.184	88.928	7.983	15.240
1138	110	17	14	P	S	40.158	-4.723	1.618	5.043	5.216	-1.470	-4.581	89.118	15.242	7.981
1139	110	17	14	P	S	58.055	-4.723	-1.618	5.043	5.216	1.470	-4.581	89.118	15.242	7.981
1140	110	17	14	P	S	60.000	5.011	1.783	-4.697	-4.554	-1.968	5.184	88.928	7.983	15.240
1141	110	17	14	P	S	61.945	-4.723	1.618	5.043	5.216	-1.470	-4.581	89.118	15.242	7.981
1142	110	17	14	P	S	79.842	-4.723	-1.618	5.043	5.216	1.470	-4.581	89.118	15.242	7.981
1143	110	17	14	P	S	81.787	5.011	1.783	-4.697	-4.554	-1.968	5.184	88.928	7.983	15.240
1144	110	17	14	P	S	82.689	-4.497	3.618	4.769	4.941	-3.303	-4.354	72.985	11.961	10.632
1145	110	17	14	P	S	97.311	-4.497	-3.618	4.769	4.941	3.303	-4.354	72.985	11.961	10.632
1146	110	17	14	P	S	98.213	5.011	-1.783	-4.697	-4.554	1.968	5.184	88.928	7.983	15.240
1147	110	17	14	P	S	100.158	-4.723	1.618	5.043	5.216	-1.470	-4.581	89.118	15.242	7.981
1148	110	17	14	P	S	118.055	-4.723	-1.618	5.043	5.216	1.470	-4.581	89.118	15.242	7.981
1149	110	17	14	P	W	0.000	5.011	1.783	-4.697	-4.554	-1.968	5.184	88.928	7.983	15.240
1150	110	17	14	P	W	1.945	-4.723	1.618	5.043	5.216	-1.470	-4.581	89.118	15.242	7.981
1151	110	17	14	P	W	19.842	-4.723	-1.618	5.043	5.216	1.470	-4.581	89.118	15.242	7.981
1152	110	17	14	P	W	21.787	5.011	1.783	-4.697	-4.554	-1.968	5.184	88.928	7.983	15.240
1153	110	17	14	P	W	22.689	-4.497	3.618	4.769	4.941	-3.303	-4.354	72.985	11.961	10.632
1154	110	17	14	P	W	37.311	-4.497	-3.618	4.769	4.941	3.303	-4.354	72.985	11.961	10.632
1155	110	17	14	P	W	38.213	5.011	-1.783	-4.697	-4.554	1.968	5.184	88.928	7.983	15.240
1156	110	17	14	P	W	40.158	-4.723	1.618	5.043	5.216	-1.470	-4.581	89.118	15.242	7.981
1157	110	17	14	P	W	58.055	-4.723	-1.618	5.043	5.216	1.470	-4.581	89.118	15.242	7.981
1158	110	17	14	P	W	60.000	5.011	1.783	-4.697	-4.554	-1.968	5.184	88.928	7.983	15.240
1159	110	17	14	P	W	61.945	-4.723	1.618	5.043	5.216	-1.470	-4.581	89.118	15.242	7.981
1160	110	17	14	P	W	79.842	-4.723	-1.618	5.043	5.216	1.470	-4.581	89.118	15.242	7.981
1161	110	17	14	P	W	81.787	5.011	1.783	-4.697	-4.554	-1.968	5.184	88.928	7.983	15.240
1162	110	17	14	P	W	82.689	-4.497	3.618	4.769	4.941	-3.303	-4.354	72.985	11.961	10.632
1163	110	17	14	P	W	97.311	-4.497	-3.618	4.769	4.941	3.303	-4.354	72.985	11.961	10.632
1164	110	17	14	P	W	98.213	5.011	-1.783	-4.697	-4.554	1.968	5.184	88.928	7.983	15.240
1165	110	17	14	P	W	100.158	-4.723	1.618	5.043	5.216	-1.470	-4.581	89.118	15.242	7.981
1166	110	17	14	P	W	118.055	-4.723	-1.618	5.043	5.216	1.470	-4.581	89.118	15.242	7.981
1167	111	18	13	C	S	16.537	-3.292	-3.652	-3.208	3.523	3.903	3.428	78.260	8.495	14.483
1168	111	18	13	C	S	17.480	-4.685	-2.092	-1.769	5.169	2.169	1.834	78.248	14.491	8.492
1169	111	18	13	C	S	19.107	-1.485	0.677	-4.950	1.530	-0.752	5.494	78.327	8.492	14.490
1170	111	18	13	C	S	19.842	-4.723	1.965	-1.728	5.216	-2.035	1.790	78.378	14.482	8.494
1171	111	18	13	C	S	40.158	-4.723	-1.965	-1.728	5.216	2.035	1.790	78.378	8.494	14.482
1172	111	18	13	C	S	42.520	-4.685	2.092	-1.769	5.169	-2.169	1.834	78.248	14.491	8.492
1173	111	18	13	C	S	43.463	-3.292	3.652	-3.208	3.523	-3.903	3.428	78.260	14.483	8.495

BL number	Atoms	γ-PC unit cells	WS_2 unit cells	γ-PC origin atom	WS_2 origin atom	Twist-angle (°)	γ-PC strain 1 (%)	γ-PC strain 2 (%)	γ-PC strain 3 (%)	WS_2 strain 1 (%)	WS_2 strain 2 (%)	WS_2 strain 3 (%)	γ (°)	|**a**|(Å)	|**b**|(Å)
1174	111	18	13	C	S	76.537	-3.292	-3.652	-3.208	3.523	3.903	3.428	78.260	8.495	14.483
1175	111	18	13	C	S	77.480	-4.685	-2.092	-1.769	5.169	2.169	1.834	78.248	14.491	8.492
1176	111	18	13	C	S	79.842	-4.723	1.965	-1.728	5.216	-2.035	1.790	78.378	14.482	8.494
1177	111	18	13	C	S	100.158	-4.723	-1.965	-1.728	5.216	2.035	1.790	78.378	8.494	14.482
1178	111	18	13	C	S	100.893	-1.485	-0.677	-4.950	1.530	0.752	5.494	78.327	8.492	14.490
1179	111	18	13	C	S	102.520	-4.685	2.092	-1.769	5.169	-2.169	1.834	78.248	14.491	8.492
1180	111	18	13	C	S	103.463	-3.292	3.652	-3.208	3.523	-3.903	3.428	78.260	14.483	8.495
1181	111	18	13	C	W	16.537	-3.292	-3.652	-3.208	3.523	3.903	3.428	78.260	8.495	14.483
1182	111	18	13	C	W	17.480	-4.685	-2.092	-1.769	5.169	2.169	1.834	78.248	14.491	8.492
1183	111	18	13	C	W	19.842	-4.723	1.965	-1.728	5.216	-2.035	1.790	78.378	14.482	8.494
1184	111	18	13	C	W	40.158	-4.723	-1.965	-1.728	5.216	2.035	1.790	78.378	8.494	14.482
1185	111	18	13	C	W	40.893	-1.485	-0.677	-4.950	1.530	0.752	5.494	78.328	8.492	14.490
1186	111	18	13	C	W	42.520	-4.685	2.092	-1.769	5.169	-2.169	1.834	78.248	14.491	8.492
1187	111	18	13	C	W	43.463	-3.292	3.652	-3.208	3.523	-3.903	3.428	78.260	14.483	8.495
1188	111	18	13	C	W	76.537	-3.292	-3.652	-3.208	3.523	3.903	3.428	78.260	8.495	14.483
1189	111	18	13	C	W	77.480	-4.685	-2.092	-1.769	5.169	2.169	1.834	78.248	14.491	8.492
1190	111	18	13	C	W	79.107	-1.485	0.677	-4.950	1.530	-0.752	5.494	78.327	8.492	14.490
1191	111	18	13	C	W	79.842	-4.723	1.965	-1.728	5.216	-2.035	1.790	78.378	14.482	8.494
1192	111	18	13	C	W	100.158	-4.723	-1.965	-1.728	5.216	2.035	1.790	78.378	8.494	14.482
1193	111	18	13	C	W	102.520	-4.685	2.092	-1.769	5.169	-2.169	1.834	78.248	14.491	8.492
1194	111	18	13	C	W	103.463	-3.292	3.652	-3.208	3.523	-3.903	3.428	78.260	14.483	8.495
1195	111	18	13	P	S	16.537	-3.292	-3.652	-3.208	3.523	3.903	3.428	78.260	8.495	14.483
1196	111	18	13	P	S	17.480	-4.685	-2.092	-1.769	5.169	2.169	1.834	78.248	14.491	8.492
1197	111	18	13	P	S	19.842	-4.723	1.965	-1.728	5.216	-2.035	1.790	78.378	14.482	8.494
1198	111	18	13	P	S	40.158	-4.723	-1.965	-1.728	5.216	2.035	1.790	78.378	8.494	14.482
1199	111	18	13	P	S	40.893	-1.485	-0.677	-4.950	1.530	0.752	5.494	78.328	8.492	14.490
1200	111	18	13	P	S	42.520	-4.685	2.092	-1.769	5.169	-2.169	1.834	78.248	14.491	8.492
1201	111	18	13	P	S	43.463	-3.292	3.652	-3.208	3.523	-3.903	3.428	78.260	14.483	8.495
1202	111	18	13	P	S	76.537	-3.292	-3.652	-3.208	3.523	3.903	3.428	78.260	8.495	14.483
1203	111	18	13	P	S	77.480	-4.685	-2.092	-1.769	5.169	2.169	1.834	78.248	14.491	8.492
1204	111	18	13	P	S	79.107	-1.485	0.677	-4.950	1.530	-0.752	5.494	78.327	8.492	14.490
1205	111	18	13	P	S	79.842	-4.723	1.965	-1.728	5.216	-2.035	1.790	78.378	14.482	8.494
1206	111	18	13	P	S	100.158	-4.723	-1.965	-1.728	5.216	2.035	1.790	78.378	8.494	14.482
1207	111	18	13	P	S	102.520	-4.685	2.092	-1.769	5.169	-2.169	1.834	78.248	14.491	8.492
1208	111	18	13	P	S	103.463	-3.292	3.652	-3.208	3.523	-3.903	3.428	78.260	14.483	8.495
1209	111	18	13	P	W	16.537	-3.292	-3.652	-3.208	3.523	3.903	3.428	78.260	8.495	14.483
1210	111	18	13	P	W	17.480	-4.685	-2.092	-1.769	5.169	2.169	1.834	78.248	14.491	8.492
1211	111	18	13	P	W	19.107	-1.485	0.677	-4.950	1.530	-0.752	5.494	78.327	8.492	14.490
1212	111	18	13	P	W	19.842	-4.723	1.965	-1.728	5.216	-2.035	1.790	78.378	14.482	8.494
1213	111	18	13	P	W	40.158	-4.723	-1.965	-1.728	5.216	2.035	1.790	78.378	8.494	14.482
1214	111	18	13	P	W	42.520	-4.685	2.092	-1.769	5.169	-2.169	1.834	78.248	14.491	8.492
1215	111	18	13	P	W	43.463	-3.292	3.652	-3.208	3.523	-3.903	3.428	78.260	14.483	8.495
1216	111	18	13	P	W	76.537	-3.292	-3.652	-3.208	3.523	3.903	3.428	78.260	8.495	14.483
1217	111	18	13	P	W	77.480	-4.685	-2.092	-1.769	5.169	2.169	1.834	78.248	14.491	8.492
1218	111	18	13	P	W	79.842	-4.723	1.965	-1.728	5.216	-2.035	1.790	78.378	14.482	8.494
1219	111	18	13	P	W	100.158	-4.723	-1.965	-1.728	5.216	2.035	1.790	78.378	8.494	14.482
1220	111	18	13	P	W	100.893	-1.485	-0.677	-4.950	1.530	0.752	5.494	78.327	8.492	14.490
1221	111	18	13	P	W	102.520	-4.685	2.092	-1.769	5.169	-2.169	1.834	78.248	14.491	8.492
1222	111	18	13	P	W	103.463	-3.292	3.652	-3.208	3.523	-3.903	3.428	78.260	14.483	8.495
1223	115	19	13	C	S	9.515	-4.497	0.000	-4.497	4.941	0.000	4.941	60.000	11.961	11.961
1224	115	19	13	C	S	22.689	-4.497	0.000	-4.497	4.941	0.000	4.941	60.000	11.961	11.961

BL number	Atoms	γ-PC unit cells	WS_2 unit cells	γ-PC origin atom	WS_2 origin atom	Twist-angle (°)	γ-PC strain 1 (%)	γ-PC strain 2 (%)	γ-PC strain 3 (%)	WS_2 strain 1 (%)	WS_2 strain 2 (%)	WS_2 strain 3 (%)	γ (°)	$\lvert\mathbf{a}\rvert$(Å)	$\lvert\mathbf{b}\rvert$(Å)
1225	115	19	13	C	S	37.311	-4.497	0.000	-4.497	4.941	0.000	4.941	60.000	11.961	11.961
1226	115	19	13	C	S	50.485	-4.497	0.000	-4.497	4.941	0.000	4.941	60.000	11.961	11.961
1227	115	19	13	C	S	69.515	-4.497	0.000	-4.497	4.941	0.000	4.941	60.000	11.961	11.961
1228	115	19	13	C	S	82.689	-4.497	0.000	-4.497	4.941	0.000	4.941	60.000	11.961	11.961
1229	115	19	13	C	S	97.311	-4.497	0.000	-4.497	4.941	0.000	4.941	60.000	11.961	11.961
1230	115	19	13	C	S	110.485	-4.497	0.000	-4.497	4.941	0.000	4.941	60.000	11.961	11.961
1231	115	19	13	C	W	9.515	-4.497	0.000	-4.497	4.941	0.000	4.941	60.000	11.961	11.961
1232	115	19	13	C	W	22.689	-4.497	0.000	-4.497	4.941	0.000	4.941	60.000	11.961	11.961
1233	115	19	13	C	W	37.311	-4.497	0.000	-4.497	4.941	0.000	4.941	60.000	11.961	11.961
1234	115	19	13	C	W	50.485	-4.497	0.000	-4.497	4.941	0.000	4.941	60.000	11.961	11.961
1235	115	19	13	C	W	69.515	-4.497	0.000	-4.497	4.941	0.000	4.941	60.000	11.961	11.961
1236	115	19	13	C	W	82.689	-4.497	0.000	-4.497	4.941	0.000	4.941	60.000	11.961	11.961
1237	115	19	13	C	W	97.311	-4.497	0.000	-4.497	4.941	0.000	4.941	60.000	11.961	11.961
1238	115	19	13	C	W	110.485	-4.497	0.000	-4.497	4.941	0.000	4.941	60.000	11.961	11.961
1239	115	19	13	P	S	22.689	-4.497	0.000	-4.497	4.941	0.000	4.941	60.000	11.961	11.961
1240	115	19	13	P	S	50.485	-4.497	0.000	-4.497	4.941	0.000	4.941	60.000	11.961	11.961
1241	115	19	13	P	S	69.515	-4.497	0.000	-4.497	4.941	0.000	4.941	60.000	11.961	11.961
1242	115	19	13	P	S	97.311	-4.497	0.000	-4.497	4.941	0.000	4.941	60.000	11.961	11.961
1243	115	19	13	P	W	9.515	-4.497	0.000	-4.497	4.941	0.000	4.941	60.000	11.961	11.961
1244	115	19	13	P	W	37.311	-4.497	0.000	-4.497	4.941	0.000	4.941	60.000	11.961	11.961
1245	115	19	13	P	W	82.689	-4.497	0.000	-4.497	4.941	0.000	4.941	60.000	11.961	11.961
1246	115	19	13	P	W	110.485	-4.497	0.000	-4.497	4.941	0.000	4.941	60.000	11.961	11.961
1247	116	17	16	C	S	12.520	2.326	-3.018	4.432	-2.223	2.772	-4.071	68.027	13.147	10.652
1248	116	17	16	C	S	13.174	5.011	-1.783	1.775	-4.554	1.722	-1.714	68.052	13.151	10.648
1249	116	17	16	C	S	21.787	5.011	-5.350	1.775	-4.554	5.166	-1.714	84.993	7.983	16.334
1250	116	17	16	C	S	27.796	5.011	5.350	1.775	-4.554	-5.166	-1.714	84.857	16.378	7.963
1251	116	17	16	C	S	32.204	5.011	-5.350	1.775	-4.554	5.166	-1.714	71.588	10.878	12.584
1252	116	17	16	C	S	38.213	5.011	5.350	1.775	-4.554	-5.166	-1.714	71.819	12.599	10.852
1253	116	17	16	C	S	43.898	2.853	-3.493	3.889	-2.699	3.241	-3.608	68.027	10.655	13.143
1254	116	17	16	C	S	46.827	5.011	1.783	1.775	-4.554	-1.722	-1.714	68.052	13.151	10.648
1255	116	17	16	C	S	47.480	2.326	3.018	4.432	-2.223	-2.772	-4.071	68.027	10.652	13.147
1256	116	17	16	C	S	72.520	2.326	-3.018	4.432	-2.223	2.772	-4.071	68.027	13.147	10.652
1257	116	17	16	C	S	73.174	5.011	-1.783	1.775	-4.554	1.722	-1.714	68.052	13.151	10.648
1258	116	17	16	C	S	76.102	2.853	3.493	3.889	-2.699	-3.241	-3.608	68.027	10.655	13.143
1259	116	17	16	C	S	81.787	5.011	-5.350	1.775	-4.554	5.166	-1.714	71.819	10.852	12.599
1260	116	17	16	C	S	87.796	5.011	5.350	1.775	-4.554	-5.166	-1.714	71.588	10.878	12.584
1261	116	17	16	C	S	92.204	5.011	-5.350	1.775	-4.554	5.166	-1.714	84.857	7.963	16.378
1262	116	17	16	C	S	98.213	5.011	5.350	1.775	-4.554	-5.166	-1.714	84.993	7.983	16.334
1263	116	17	16	C	S	103.898	2.853	-3.493	3.889	-2.699	3.241	-3.608	68.027	10.655	13.143
1264	116	17	16	C	S	106.827	5.011	1.783	1.775	-4.554	-1.722	-1.714	68.052	13.151	10.648
1265	116	17	16	C	W	12.520	2.326	-3.018	4.432	-2.223	2.772	-4.071	68.027	13.147	10.652
1266	116	17	16	C	W	13.174	5.011	-1.783	1.775	-4.554	1.722	-1.714	68.052	13.151	10.648
1267	116	17	16	C	W	16.102	2.853	3.493	3.889	-2.699	-3.241	-3.608	68.027	10.655	13.143
1268	116	17	16	C	W	21.787	5.011	-5.350	1.775	-4.554	5.166	-1.714	84.993	7.983	16.334
1269	116	17	16	C	W	27.796	5.011	5.350	1.775	-4.554	-5.166	-1.714	84.857	16.378	7.963
1270	116	17	16	C	W	32.204	5.011	-5.350	1.775	-4.554	5.166	-1.714	71.588	10.878	12.584
1271	116	17	16	C	W	38.213	5.011	5.350	1.775	-4.554	-5.166	-1.714	71.819	12.599	10.852
1272	116	17	16	C	W	43.898	2.853	-3.493	3.889	-2.699	3.241	-3.608	68.027	10.655	13.143
1273	116	17	16	C	W	46.827	5.011	1.783	1.775	-4.554	-1.722	-1.714	68.052	13.151	10.648
1274	116	17	16	C	W	72.520	2.326	-3.018	4.432	-2.223	2.772	-4.071	68.027	13.147	10.652
1275	116	17	16	C	W	73.174	5.011	-1.783	1.775	-4.554	1.722	-1.714	68.052	13.151	10.648

BL number	Atoms	γ-PC unit cells	WS_2 unit cells	γ-PC origin atom	WS_2 origin atom	Twist-angle (°)	γ-PC strain 1 (%)	γ-PC strain 2 (%)	γ-PC strain 3 (%)	WS_2 strain 1 (%)	WS_2 strain 2 (%)	WS_2 strain 3 (%)	γ (°)	|**a**|(Å)	|**b**|(Å)
1276	116	17	16	C	W	81.787	5.011	-5.350	1.775	-4.554	5.166	-1.714	71.819	10.852	12.599
1277	116	17	16	C	W	87.796	5.011	5.350	1.775	-4.554	-5.166	-1.714	71.588	10.878	12.584
1278	116	17	16	C	W	92.204	5.011	-5.350	1.775	-4.554	5.166	-1.714	84.857	7.963	16.378
1279	116	17	16	C	W	98.213	5.011	5.350	1.775	-4.554	-5.166	-1.714	84.993	7.983	16.334
1280	116	17	16	C	W	103.898	2.853	-3.493	3.889	-2.699	3.241	-3.608	68.027	10.655	13.143
1281	116	17	16	C	W	106.827	5.011	1.783	1.775	-4.554	-1.722	-1.714	68.052	13.151	10.648
1282	116	17	16	C	W	107.480	2.326	3.018	4.432	-2.223	-2.772	-4.071	68.028	10.652	13.147
1283	116	17	16	P	S	12.520	2.326	-3.018	4.432	-2.223	2.772	-4.071	68.027	13.147	10.652
1284	116	17	16	P	S	13.174	5.011	-1.783	1.775	-4.554	1.722	-1.714	68.052	13.151	10.648
1285	116	17	16	P	S	16.102	2.853	3.493	3.889	-2.699	-3.241	-3.608	68.027	10.655	13.143
1286	116	17	16	P	S	21.787	5.011	-5.350	1.775	-4.554	5.166	-1.714	84.993	7.983	16.334
1287	116	17	16	P	S	27.796	5.011	5.350	1.775	-4.554	-5.166	-1.714	84.857	16.378	7.963
1288	116	17	16	P	S	32.204	5.011	-5.350	1.775	-4.554	5.166	-1.714	71.588	10.878	12.584
1289	116	17	16	P	S	38.213	5.011	5.350	1.775	-4.554	-5.166	-1.714	71.819	12.599	10.852
1290	116	17	16	P	S	43.898	2.853	-3.493	3.889	-2.699	3.241	-3.608	68.027	10.655	13.143
1291	116	17	16	P	S	46.827	5.011	1.783	1.775	-4.554	-1.722	-1.714	68.052	13.151	10.648
1292	116	17	16	P	S	72.520	2.326	-3.018	4.432	-2.223	2.772	-4.071	68.027	13.147	10.652
1293	116	17	16	P	S	73.174	5.011	-1.783	1.775	-4.554	1.722	-1.714	68.052	13.151	10.648
1294	116	17	16	P	S	81.787	5.011	-5.350	1.775	-4.554	5.166	-1.714	71.819	10.852	12.599
1295	116	17	16	P	S	87.796	5.011	5.350	1.775	-4.554	-5.166	-1.714	71.588	10.878	12.584
1296	116	17	16	P	S	92.204	5.011	-5.350	1.775	-4.554	5.166	-1.714	84.857	7.963	16.378
1297	116	17	16	P	S	98.213	5.011	5.350	1.775	-4.554	-5.166	-1.714	84.993	7.983	16.334
1298	116	17	16	P	S	103.898	2.853	-3.493	3.889	-2.699	3.241	-3.608	68.027	10.655	13.143
1299	116	17	16	P	S	106.827	5.011	1.783	1.775	-4.554	-1.722	-1.714	68.052	13.151	10.648
1300	116	17	16	P	S	107.480	2.326	3.018	4.432	-2.223	-2.772	-4.071	68.028	10.652	13.147
1301	116	17	16	P	W	12.520	2.326	-3.018	4.432	-2.223	2.772	-4.071	68.027	13.147	10.652
1302	116	17	16	P	W	13.174	5.011	-1.783	1.775	-4.554	1.722	-1.714	68.052	13.151	10.648
1303	116	17	16	P	W	21.787	5.011	-5.350	1.775	-4.554	5.166	-1.714	71.819	10.852	12.599
1304	116	17	16	P	W	27.796	5.011	5.350	1.775	-4.554	-5.166	-1.714	71.588	10.878	12.584
1305	116	17	16	P	W	32.204	5.011	-5.350	1.775	-4.554	5.166	-1.714	84.857	7.963	16.378
1306	116	17	16	P	W	38.213	5.011	5.350	1.775	-4.554	-5.166	-1.714	84.993	7.983	16.334
1307	116	17	16	P	W	43.898	2.853	-3.493	3.889	-2.699	3.241	-3.608	68.027	10.655	13.143
1308	116	17	16	P	W	46.827	5.011	1.783	1.775	-4.554	-1.722	-1.714	68.052	13.151	10.648
1309	116	17	16	P	W	47.480	2.326	3.018	4.432	-2.223	-2.772	-4.071	68.027	10.652	13.147
1310	116	17	16	P	W	72.520	2.326	-3.018	4.432	-2.223	2.772	-4.071	68.027	13.147	10.652
1311	116	17	16	P	W	73.174	5.011	-1.783	1.775	-4.554	1.722	-1.714	68.052	13.151	10.648
1312	116	17	16	P	W	76.102	2.853	3.493	3.889	-2.699	-3.241	-3.608	68.027	10.655	13.143
1313	116	17	16	P	W	81.787	5.011	-5.350	1.775	-4.554	5.166	-1.714	84.993	7.983	16.334
1314	116	17	16	P	W	87.796	5.011	5.350	1.775	-4.554	-5.166	-1.714	84.857	16.378	7.963
1315	116	17	16	P	W	92.204	5.011	-5.350	1.775	-4.554	5.166	-1.714	71.588	10.878	12.584
1316	116	17	16	P	W	98.213	5.011	5.350	1.775	-4.554	-5.166	-1.714	71.819	12.599	10.852
1317	116	17	16	P	W	103.898	2.853	-3.493	3.889	-2.699	3.241	-3.608	68.027	10.655	13.143
1318	116	17	16	P	W	106.827	5.011	1.783	1.775	-4.554	-1.722	-1.714	68.052	13.151	10.648
1319	117	18	15	C	S	0.000	5.011	1.684	-4.158	-4.554	-1.837	4.535	79.060	7.983	16.525
1320	117	18	15	C	S	13.174	5.011	-1.684	-4.158	-4.554	1.837	4.535	82.988	13.151	9.922
1321	117	18	15	C	S	13.898	-4.228	-0.512	5.096	4.619	0.465	-4.625	83.056	9.921	13.152
1322	117	18	15	C	S	17.696	1.029	-3.109	-0.580	-1.008	3.146	0.587	79.074	15.501	8.492
1323	117	18	15	C	S	19.107	-1.485	-0.677	1.980	1.530	0.651	-1.905	79.101	8.492	15.505
1324	117	18	15	C	S	21.052	1.339	2.709	-0.879	-1.304	-2.757	0.895	79.153	8.491	15.500
1325	117	18	15	C	S	25.693	2.326	-4.405	-1.805	-2.223	4.570	1.873	81.151	13.473	9.713
1326	117	18	15	C	S	30.000	-2.359	-3.132	2.934	2.476	2.958	-2.772	81.350	9.718	13.462

BL number	Atoms	γ-PC unit cells	WS_2 unit cells	γ-PC origin atom	WS_2 origin atom	Twist-angle (°)	γ-PC strain 1 (%)	γ-PC strain 2 (%)	γ-PC strain 3 (%)	WS_2 strain 1 (%)	WS_2 strain 2 (%)	WS_2 strain 3 (%)	γ (°)	|**a**|(Å)	|**b**|(Å)
1327	117	18	15	C	S	34.307	2.326	4.405	-1.805	-2.223	-4.570	1.873	81.151	13.473	9.713
1328	117	18	15	C	S	38.948	1.339	-2.709	-0.879	-1.304	2.757	0.895	79.153	15.500	8.491
1329	117	18	15	C	S	40.893	1.980	0.654	-1.485	-1.905	-0.674	1.530	79.099	15.504	8.492
1330	117	18	15	C	S	42.304	1.029	3.109	-0.580	-1.008	-3.146	0.587	79.074	8.492	15.501
1331	117	18	15	C	S	46.102	-4.228	0.512	5.096	4.619	-0.465	-4.625	83.056	9.921	13.152
1332	117	18	15	C	S	46.827	5.011	1.684	-4.158	-4.554	-1.837	4.535	82.988	13.151	9.922
1333	117	18	15	C	S	60.000	-4.158	-1.844	5.011	4.534	1.676	-4.554	79.065	16.522	7.984
1334	117	18	15	C	S	73.174	5.011	-1.684	-4.158	-4.554	1.837	4.535	82.988	13.151	9.922
1335	117	18	15	C	S	73.898	-4.228	-0.512	5.096	4.619	0.465	-4.625	83.056	9.921	13.152
1336	117	18	15	C	S	77.696	1.029	-3.109	-0.580	-1.008	3.146	0.587	79.074	15.501	8.492
1337	117	18	15	C	S	79.107	1.980	-0.654	-1.485	-1.905	0.674	1.530	79.099	15.504	8.492
1338	117	18	15	C	S	81.052	1.339	2.709	-0.879	-1.304	-2.757	0.895	79.153	8.491	15.500
1339	117	18	15	C	S	85.694	2.326	-4.405	-1.805	-2.223	4.570	1.873	81.151	13.473	9.713
1340	117	18	15	C	S	90.000	-2.359	-3.132	2.934	2.476	2.958	-2.772	81.350	9.718	13.462
1341	117	18	15	C	S	94.307	2.326	4.405	-1.805	-2.223	-4.570	1.873	81.151	13.473	9.713
1342	117	18	15	C	S	98.948	1.339	-2.709	-0.879	-1.304	2.757	0.895	79.153	15.500	8.491
1343	117	18	15	C	S	100.893	1.980	0.654	-1.485	-1.905	-0.674	1.530	79.100	15.504	8.492
1344	117	18	15	C	S	106.102	-4.228	0.512	5.096	4.619	-0.465	-4.625	83.056	9.921	13.152
1345	117	18	15	C	S	106.827	5.011	1.684	-4.158	-4.554	-1.837	4.535	82.988	13.151	9.922
1346	117	18	15	C	W	0.000	5.011	1.684	-4.158	-4.554	-1.837	4.535	79.060	7.983	16.525
1347	117	18	15	C	W	13.174	5.011	-1.684	-4.158	-4.554	1.837	4.535	82.988	13.151	9.922
1348	117	18	15	C	W	13.898	-4.228	-0.512	5.096	4.619	0.465	-4.625	83.056	9.921	13.152
1349	117	18	15	C	W	17.696	1.029	-3.109	-0.580	-1.008	3.146	0.587	79.074	15.501	8.492
1350	117	18	15	C	W	19.107	1.980	-0.654	-1.485	-1.905	0.674	1.530	79.100	15.504	8.492
1351	117	18	15	C	W	21.052	1.339	2.709	-0.879	-1.304	-2.757	0.895	79.153	8.491	15.500
1352	117	18	15	C	W	25.693	2.326	-4.405	-1.805	-2.223	4.570	1.873	81.151	13.473	9.713
1353	117	18	15	C	W	30.000	-2.359	-3.132	2.934	2.476	2.958	-2.772	81.350	9.718	13.462
1354	117	18	15	C	W	34.307	2.326	4.405	-1.805	-2.223	-4.570	1.873	81.151	13.473	9.713
1355	117	18	15	C	W	38.948	1.339	-2.709	-0.879	-1.304	2.757	0.895	79.153	15.500	8.491
1356	117	18	15	C	W	40.893	1.980	0.654	-1.485	-1.905	-0.674	1.530	79.099	15.504	8.492
1357	117	18	15	C	W	46.102	-4.228	0.512	5.096	4.619	-0.465	-4.625	83.056	9.921	13.152
1358	117	18	15	C	W	46.827	5.011	1.684	-4.158	-4.554	-1.837	4.535	82.988	13.151	9.922
1359	117	18	15	C	W	60.000	-4.158	-1.844	5.011	4.534	1.676	-4.554	79.065	16.522	7.984
1360	117	18	15	C	W	73.174	5.011	-1.684	-4.158	-4.554	1.837	4.535	82.988	13.151	9.922
1361	117	18	15	C	W	73.898	-4.228	-0.512	5.096	4.619	0.465	-4.625	83.056	9.921	13.152
1362	117	18	15	C	W	77.696	1.029	-3.109	-0.580	-1.008	3.146	0.587	79.074	15.501	8.492
1363	117	18	15	C	W	81.052	1.339	2.709	-0.879	-1.304	-2.757	0.895	79.153	8.491	15.500
1364	117	18	15	C	W	85.694	2.326	-4.405	-1.805	-2.223	4.570	1.873	81.151	13.473	9.713
1365	117	18	15	C	W	90.000	-2.359	-3.132	2.934	2.476	2.958	-2.772	81.350	9.718	13.462
1366	117	18	15	C	W	94.307	2.326	4.405	-1.805	-2.223	-4.570	1.873	81.151	13.473	9.713
1367	117	18	15	C	W	98.948	1.339	-2.709	-0.879	-1.304	2.757	0.895	79.153	15.500	8.491
1368	117	18	15	C	W	100.893	1.980	0.654	-1.485	-1.905	-0.674	1.530	79.100	15.504	8.492
1369	117	18	15	C	W	102.304	1.029	3.109	-0.580	-1.008	-3.146	0.587	79.074	8.492	15.501
1370	117	18	15	C	W	106.102	-4.228	0.512	5.096	4.619	-0.465	-4.625	83.056	9.921	13.152
1371	117	18	15	C	W	106.827	5.011	1.684	-4.158	-4.554	-1.837	4.535	82.988	13.151	9.922
1372	117	18	15	P	S	0.000	5.011	1.684	-4.158	-4.554	-1.837	4.535	79.060	7.983	16.525
1373	117	18	15	P	S	13.174	5.011	-1.684	-4.158	-4.554	1.837	4.535	82.988	13.151	9.922
1374	117	18	15	P	S	13.898	-4.228	-0.512	5.096	4.619	0.465	-4.625	83.056	9.921	13.152
1375	117	18	15	P	S	17.696	1.029	-3.109	-0.580	-1.008	3.146	0.587	79.074	15.501	8.492
1376	117	18	15	P	S	19.107	1.980	-0.654	-1.485	-1.905	0.674	1.530	79.100	15.504	8.492
1377	117	18	15	P	S	21.052	1.339	2.709	-0.879	-1.304	-2.757	0.895	79.153	8.491	15.500

BL number	Atoms	γ-PC unit cells	WS_2 unit cells	γ-PC origin atom	WS_2 origin atom	Twist-angle (°)	γ-PC strain 1 (%)	γ-PC strain 2 (%)	γ-PC strain 3 (%)	WS_2 strain 1 (%)	WS_2 strain 2 (%)	WS_2 strain 3 (%)	γ (°)	$\lvert\mathbf{a}\rvert$(Å)	$\lvert\mathbf{b}\rvert$(Å)
1378	117	18	15	P	S	38.948	1.339	-2.709	-0.879	-1.304	2.757	0.895	79.153	15.500	8.491
1379	117	18	15	P	S	40.893	1.980	0.654	-1.485	-1.905	-0.674	1.530	79.099	15.504	8.492
1380	117	18	15	P	S	46.102	-4.228	0.512	5.096	4.619	-0.465	-4.625	83.056	9.921	13.152
1381	117	18	15	P	S	46.827	5.011	1.684	-4.158	-4.554	-1.837	4.535	82.988	13.151	9.922
1382	117	18	15	P	S	60.000	-4.158	-1.844	5.011	4.534	1.676	-4.554	79.065	16.522	7.984
1383	117	18	15	P	S	73.174	5.011	-1.684	-4.158	-4.554	1.837	4.535	82.988	13.151	9.922
1384	117	18	15	P	S	73.898	-4.228	-0.512	5.096	4.619	0.465	-4.625	83.056	9.921	13.152
1385	117	18	15	P	S	77.696	1.029	-3.109	-0.580	-1.008	3.146	0.587	79.074	15.501	8.492
1386	117	18	15	P	S	81.052	1.339	2.709	-0.879	-1.304	-2.757	0.895	79.153	8.491	15.500
1387	117	18	15	P	S	98.948	1.339	-2.709	-0.879	-1.304	2.757	0.895	79.153	15.500	8.491
1388	117	18	15	P	S	100.893	1.980	0.654	-1.485	-1.905	-0.674	1.530	79.100	15.504	8.492
1389	117	18	15	P	S	102.304	1.029	3.109	-0.580	-1.008	-3.146	0.587	79.074	8.492	15.501
1390	117	18	15	P	S	106.102	-4.228	0.512	5.096	4.619	-0.465	-4.625	83.056	9.921	13.152
1391	117	18	15	P	S	106.827	5.011	1.684	-4.158	-4.554	-1.837	4.535	82.988	13.151	9.922
1392	117	18	15	P	W	0.000	5.011	1.684	-4.158	-4.554	-1.837	4.535	79.060	7.983	16.525
1393	117	18	15	P	W	13.174	5.011	-1.684	-4.158	-4.554	1.837	4.535	82.988	13.151	9.922
1394	117	18	15	P	W	13.898	-4.228	-0.512	5.096	4.619	0.465	-4.625	83.056	9.921	13.152
1395	117	18	15	P	W	17.696	1.029	-3.109	-0.580	-1.008	3.146	0.587	79.074	15.501	8.492
1396	117	18	15	P	W	19.107	-1.485	-0.677	1.980	1.530	0.651	-1.905	79.101	8.492	15.505
1397	117	18	15	P	W	21.052	1.339	2.709	-0.879	-1.304	-2.757	0.895	79.153	8.491	15.500
1398	117	18	15	P	W	38.948	1.339	-2.709	-0.879	-1.304	2.757	0.895	79.153	15.500	8.491
1399	117	18	15	P	W	40.893	1.980	0.654	-1.485	-1.905	-0.674	1.530	79.099	15.504	8.492
1400	117	18	15	P	W	42.304	1.029	3.109	-0.580	-1.008	-3.146	0.587	79.074	8.492	15.501
1401	117	18	15	P	W	46.102	-4.228	0.512	5.096	4.619	-0.465	-4.625	83.056	9.921	13.152
1402	117	18	15	P	W	46.827	5.011	1.684	-4.158	-4.554	-1.837	4.535	82.988	13.151	9.922
1403	117	18	15	P	W	60.000	-4.158	-1.844	5.011	4.534	1.676	-4.554	79.065	16.522	7.984
1404	117	18	15	P	W	73.174	5.011	-1.684	-4.158	-4.554	1.837	4.535	82.988	13.151	9.922
1405	117	18	15	P	W	73.898	-4.228	-0.512	5.096	4.619	0.465	-4.625	83.056	9.921	13.152
1406	117	18	15	P	W	77.696	1.029	-3.109	-0.580	-1.008	3.146	0.587	79.074	15.501	8.492
1407	117	18	15	P	W	79.107	1.980	-0.654	-1.485	-1.905	0.674	1.530	79.099	15.504	8.492
1408	117	18	15	P	W	81.052	1.339	2.709	-0.879	-1.304	-2.757	0.895	79.153	8.491	15.500
1409	117	18	15	P	W	98.948	1.339	-2.709	-0.879	-1.304	2.757	0.895	79.153	15.500	8.491
1410	117	18	15	P	W	100.893	1.980	0.654	-1.485	-1.905	-0.674	1.530	79.100	15.504	8.492
1411	117	18	15	P	W	106.102	-4.228	0.512	5.096	4.619	-0.465	-4.625	83.056	9.921	13.152
1412	117	18	15	P	W	106.827	5.011	1.684	-4.158	-4.554	-1.837	4.535	82.988	13.151	9.922
1413	119	17	17	C	S	17.897	5.011	-3.566	5.011	-4.554	3.241	-4.555	90.000	16.799	7.978
1414	119	17	17	C	S	21.787	5.011	3.566	5.011	-4.554	-3.241	-4.555	90.000	7.983	16.789
1415	119	17	17	C	S	38.213	5.011	-3.566	5.011	-4.554	3.241	-4.555	90.000	7.983	16.789
1416	119	17	17	C	S	42.103	5.011	3.566	5.011	-4.554	-3.241	-4.555	90.000	16.799	7.978
1417	119	17	17	C	S	77.897	5.011	-3.566	5.011	-4.554	3.241	-4.555	90.000	16.799	7.978
1418	119	17	17	C	S	81.787	5.011	3.566	5.011	-4.554	-3.241	-4.555	90.000	7.983	16.789
1419	119	17	17	C	S	98.213	5.011	-3.566	5.011	-4.554	3.241	-4.555	90.000	7.983	16.789
1420	119	17	17	C	S	102.104	5.011	3.566	5.011	-4.554	-3.241	-4.555	90.000	16.799	7.978
1421	119	17	17	C	W	17.897	5.011	-3.566	5.011	-4.554	3.241	-4.555	90.000	16.799	7.978
1422	119	17	17	C	W	21.787	5.011	3.566	5.011	-4.554	-3.241	-4.555	90.000	7.983	16.789
1423	119	17	17	C	W	38.213	5.011	-3.566	5.011	-4.554	3.241	-4.555	90.000	7.983	16.789
1424	119	17	17	C	W	42.103	5.011	3.566	5.011	-4.554	-3.241	-4.555	90.000	16.799	7.978
1425	119	17	17	C	W	77.897	5.011	-3.566	5.011	-4.554	3.241	-4.555	90.000	16.799	7.978
1426	119	17	17	C	W	81.787	5.011	3.566	5.011	-4.554	-3.241	-4.555	90.000	7.983	16.789
1427	119	17	17	C	W	98.213	5.011	-3.566	5.011	-4.554	3.241	-4.555	90.000	7.983	16.789
1428	119	17	17	C	W	102.104	5.011	3.566	5.011	-4.554	-3.241	-4.555	90.000	16.799	7.978

BL number	Atoms	γ-PC unit cells	WS_2 unit cells	γ-PC origin atom	WS_2 origin atom	Twist-angle (°)	γ-PC strain 1 (%)	γ-PC strain 2 (%)	γ-PC strain 3 (%)	WS_2 strain 1 (%)	WS_2 strain 2 (%)	WS_2 strain 3 (%)	γ (°)	$\lvert\mathbf{a}\rvert$(Å)	$\lvert\mathbf{b}\rvert$(Å)
1429	119	17	17	P	S	17.897	5.011	-3.566	5.011	-4.554	3.241	-4.555	90.000	16.799	7.978
1430	119	17	17	P	S	21.787	5.011	3.566	5.011	-4.554	-3.241	-4.555	90.000	7.983	16.789
1431	119	17	17	P	S	38.213	5.011	-3.566	5.011	-4.554	3.241	-4.555	90.000	7.983	16.789
1432	119	17	17	P	S	42.103	5.011	3.566	5.011	-4.554	-3.241	-4.555	90.000	16.799	7.978
1433	119	17	17	P	S	77.897	5.011	-3.566	5.011	-4.554	3.241	-4.555	90.000	16.799	7.978
1434	119	17	17	P	S	81.787	5.011	3.566	5.011	-4.554	-3.241	-4.555	90.000	7.983	16.789
1435	119	17	17	P	S	98.213	5.011	-3.566	5.011	-4.554	3.241	-4.555	90.000	7.983	16.789
1436	119	17	17	P	S	102.104	5.011	3.566	5.011	-4.554	-3.241	-4.555	90.000	16.799	7.978
1437	119	17	17	P	W	17.897	5.011	-3.566	5.011	-4.554	3.241	-4.555	90.000	16.799	7.978
1438	119	17	17	P	W	21.787	5.011	3.566	5.011	-4.554	-3.241	-4.555	90.000	7.983	16.789
1439	119	17	17	P	W	38.213	5.011	-3.566	5.011	-4.554	3.241	-4.555	90.000	7.983	16.789
1440	119	17	17	P	W	42.103	5.011	3.566	5.011	-4.554	-3.241	-4.555	90.000	16.799	7.978
1441	119	17	17	P	W	77.897	5.011	-3.566	5.011	-4.554	3.241	-4.555	90.000	16.799	7.978
1442	119	17	17	P	W	81.787	5.011	3.566	5.011	-4.554	-3.241	-4.555	90.000	7.983	16.789
1443	119	17	17	P	W	98.213	5.011	-3.566	5.011	-4.554	3.241	-4.555	90.000	7.983	16.789
1444	119	17	17	P	W	102.104	5.011	3.566	5.011	-4.554	-3.241	-4.555	90.000	16.799	7.978
1445	120	18	16	C	S	0.000	5.011	0.000	-1.101	-4.554	0.000	1.126	90.000	5.226	25.574
1446	120	18	16	C	S	60.000	5.011	0.000	-1.101	-4.554	0.000	1.126	90.000	5.226	25.574
1447	120	18	16	C	W	0.000	5.011	0.000	-1.101	-4.554	0.000	1.126	90.000	5.226	25.574
1448	120	18	16	C	W	60.000	5.011	0.000	-1.101	-4.554	0.000	1.126	90.000	5.226	25.574
1449	120	18	16	P	S	0.000	5.011	0.000	-1.101	-4.554	0.000	1.126	90.000	5.226	25.574
1450	120	18	16	P	W	60.000	5.011	0.000	-1.101	-4.554	0.000	1.126	90.000	5.226	25.574
1451	121	19	15	C	S	12.520	2.326	-0.491	-4.342	-2.223	0.538	4.755	84.139	13.473	9.921
1452	121	19	15	C	S	13.898	-4.228	1.940	2.196	4.619	-1.859	-2.104	84.039	9.921	13.473
1453	121	19	15	C	S	46.102	-4.228	-1.940	2.196	4.619	1.859	-2.104	84.039	9.921	13.473
1454	121	19	15	C	S	47.480	2.326	0.491	-4.342	-2.223	-0.538	4.755	84.139	13.473	9.921
1455	121	19	15	C	S	72.520	2.326	-0.491	-4.342	-2.223	0.538	4.755	84.139	13.473	9.921
1456	121	19	15	C	S	73.898	-4.228	1.940	2.196	4.619	-1.859	-2.104	84.039	9.921	13.473
1457	121	19	15	C	S	106.102	-4.228	-1.940	2.196	4.619	1.859	-2.104	84.039	9.921	13.473
1458	121	19	15	C	S	107.480	2.326	0.491	-4.342	-2.223	-0.538	4.755	84.139	13.473	9.921
1459	121	19	15	C	W	12.520	2.326	-0.491	-4.342	-2.223	0.538	4.755	84.139	13.473	9.921
1460	121	19	15	C	W	13.898	-4.228	1.940	2.196	4.619	-1.859	-2.104	84.039	9.921	13.473
1461	121	19	15	C	W	46.102	-4.228	-1.940	2.196	4.619	1.859	-2.104	84.039	9.921	13.473
1462	121	19	15	C	W	47.480	2.326	0.491	-4.342	-2.223	-0.538	4.755	84.139	13.473	9.921
1463	121	19	15	C	W	72.520	2.326	-0.491	-4.342	-2.223	0.538	4.755	84.139	13.473	9.921
1464	121	19	15	C	W	73.898	-4.228	1.940	2.196	4.619	-1.859	-2.104	84.039	9.921	13.473
1465	121	19	15	C	W	106.102	-4.228	-1.940	2.196	4.619	1.859	-2.104	84.039	9.921	13.473
1466	121	19	15	C	W	107.480	2.326	0.491	-4.342	-2.223	-0.538	4.755	84.139	13.473	9.921
1467	121	19	15	P	S	12.520	2.326	-0.491	-4.342	-2.223	0.538	4.755	84.139	13.473	9.921
1468	121	19	15	P	S	13.898	-4.228	1.940	2.196	4.619	-1.859	-2.104	84.039	9.921	13.473
1469	121	19	15	P	S	46.102	-4.228	-1.940	2.196	4.619	1.859	-2.104	84.039	9.921	13.473
1470	121	19	15	P	S	47.480	2.326	0.491	-4.342	-2.223	-0.538	4.755	84.139	13.473	9.921
1471	121	19	15	P	S	72.520	2.326	-0.491	-4.342	-2.223	0.538	4.755	84.139	13.473	9.921
1472	121	19	15	P	S	73.898	-4.228	1.940	2.196	4.619	-1.859	-2.104	84.039	9.921	13.473
1473	121	19	15	P	S	106.102	-4.228	-1.940	2.196	4.619	1.859	-2.104	84.039	9.921	13.473
1474	121	19	15	P	S	107.480	2.326	0.491	-4.342	-2.223	-0.538	4.755	84.139	13.473	9.921
1475	121	19	15	P	W	12.520	2.326	-0.491	-4.342	-2.223	0.538	4.755	84.139	13.473	9.921
1476	121	19	15	P	W	13.898	-4.228	1.940	2.196	4.619	-1.859	-2.104	84.039	9.921	13.473
1477	121	19	15	P	W	46.102	-4.228	-1.940	2.196	4.619	1.859	-2.104	84.039	9.921	13.473
1478	121	19	15	P	W	47.480	2.326	0.491	-4.342	-2.223	-0.538	4.755	84.139	13.473	9.921
1479	121	19	15	P	W	72.520	2.326	-0.491	-4.342	-2.223	0.538	4.755	84.139	13.473	9.921

BL number	Atoms	γ-PC unit cells	WS_2 unit cells	γ-PC origin atom	WS_2 origin atom	Twist-angle (°)	γ-PC strain 1 (%)	γ-PC strain 2 (%)	γ-PC strain 3 (%)	WS_2 strain 1 (%)	WS_2 strain 2 (%)	WS_2 strain 3 (%)	γ (°)	|**a**|(Å)	|**b**|(Å)
1480	121	19	15	P	W	73.898	-4.228	1.940	2.196	4.619	-1.859	-2.104	84.039	9.921	13.473
1481	121	19	15	P	W	106.102	-4.228	-1.940	2.196	4.619	1.859	-2.104	84.039	9.921	13.473
1482	121	19	15	P	W	107.480	2.326	0.491	-4.342	-2.223	-0.538	4.755	84.139	13.473	9.921
1483	124	19	16	C	S	23.413	0.481	0.000	0.481	-0.477	0.000	-0.477	60.000	12.584	12.584
1484	124	19	16	C	S	36.587	0.481	0.000	0.481	-0.477	0.000	-0.477	60.000	12.584	12.584
1485	124	19	16	C	S	83.413	0.481	0.000	0.481	-0.477	0.000	-0.477	60.000	12.584	12.584
1486	124	19	16	C	S	96.587	0.481	0.000	0.481	-0.477	0.000	-0.477	60.000	12.584	12.584
1487	124	19	16	C	W	23.413	0.481	0.000	0.481	-0.477	0.000	-0.477	60.000	12.584	12.584
1488	124	19	16	C	W	36.587	0.481	0.000	0.481	-0.477	0.000	-0.477	60.000	12.584	12.584
1489	124	19	16	C	W	83.413	0.481	0.000	0.481	-0.477	0.000	-0.477	60.000	12.584	12.584
1490	124	19	16	C	W	96.587	0.481	0.000	0.481	-0.477	0.000	-0.477	60.000	12.584	12.584
1491	124	19	16	P	S	23.413	0.481	0.000	0.481	-0.477	0.000	-0.477	60.000	12.584	12.584
1492	124	19	16	P	S	96.587	0.481	0.000	0.481	-0.477	0.000	-0.477	60.000	12.584	12.584
1493	124	19	16	P	W	36.587	0.481	0.000	0.481	-0.477	0.000	-0.477	60.000	12.584	12.584
1494	124	19	16	P	W	83.413	0.481	0.000	0.481	-0.477	0.000	-0.477	60.000	12.584	12.584
1495	124	19	16	C	S	0.000	5.011	1.595	-3.675	-4.554	-1.722	3.967	83.387	7.983	17.328
1496	124	19	16	C	S	1.359	-3.292	4.218	4.559	3.523	-3.866	-4.178	83.453	17.313	7.985
1497	124	19	16	C	S	3.004	-3.388	3.774	4.673	3.635	-3.452	-4.273	83.146	17.335	7.981
1498	124	19	16	C	S	10.893	-1.983	-4.468	3.072	2.064	4.209	-2.894	87.518	12.906	10.644
1499	124	19	16	C	S	15.608	-2.145	4.035	3.252	2.241	-3.789	-3.054	87.346	12.889	10.659
1500	124	19	16	C	S	16.102	2.853	4.688	-1.783	-2.699	-4.861	1.849	87.267	10.655	12.893
1501	124	19	16	C	S	43.898	2.853	-4.688	-1.783	-2.699	4.861	1.849	87.267	10.655	12.893
1502	124	19	16	C	S	44.392	-2.145	-4.035	3.252	2.241	3.789	-3.054	87.346	10.659	12.889
1503	124	19	16	C	S	49.107	-1.983	4.468	3.072	2.064	-4.209	-2.894	87.518	12.906	10.644
1504	124	19	16	C	S	56.996	-3.388	-3.774	4.672	3.635	3.452	-4.273	83.146	17.335	7.981
1505	124	19	16	C	S	58.641	-3.292	-4.218	4.559	3.523	3.866	-4.178	83.453	7.985	17.313
1506	124	19	16	C	S	60.000	5.011	1.595	-3.675	-4.554	-1.722	3.967	83.386	7.983	17.328
1507	124	19	16	C	S	61.359	-3.292	4.218	4.559	3.523	-3.866	-4.178	83.453	17.313	7.985
1508	124	19	16	C	S	63.005	-3.388	3.774	4.673	3.635	-3.452	-4.273	83.146	17.335	7.981
1509	124	19	16	C	S	70.893	-1.983	-4.468	3.072	2.064	4.209	-2.894	87.518	12.906	10.644
1510	124	19	16	C	S	75.609	-2.145	4.035	3.252	2.241	-3.789	-3.054	48.839	17.102	10.659
1511	124	19	16	C	S	76.102	2.853	4.688	-1.783	-2.699	-4.861	1.849	87.267	10.655	12.893
1512	124	19	16	C	S	103.898	2.853	-4.688	-1.783	-2.699	4.861	1.849	87.267	10.655	12.893
1513	124	19	16	C	S	104.392	-2.145	-4.035	3.252	2.241	3.789	-3.054	87.346	10.659	12.889
1514	124	19	16	C	S	109.107	-1.983	4.468	3.072	2.064	-4.209	-2.894	87.518	12.906	10.644
1515	124	19	16	C	S	116.996	-3.388	-3.774	4.672	3.635	3.452	-4.273	83.146	17.335	7.981
1516	124	19	16	C	S	118.641	-3.292	-4.218	4.559	3.523	3.866	-4.178	83.453	7.985	17.313
1517	124	19	16	C	W	0.000	5.011	1.595	-3.675	-4.554	-1.722	3.967	83.387	7.983	17.328
1518	124	19	16	C	W	1.359	-3.292	4.218	4.559	3.523	-3.866	-4.178	83.453	17.313	7.985
1519	124	19	16	C	W	3.004	-3.388	3.774	4.673	3.635	-3.452	-4.273	83.146	17.335	7.981
1520	124	19	16	C	W	10.893	-1.983	-4.468	3.072	2.064	4.209	-2.894	87.518	12.906	10.644
1521	124	19	16	C	W	15.608	-2.145	4.035	3.252	2.241	-3.789	-3.054	87.346	12.889	10.659
1522	124	19	16	C	W	16.102	2.853	4.688	-1.783	-2.699	-4.861	1.849	87.267	10.655	12.893
1523	124	19	16	C	W	43.898	2.853	-4.688	-1.783	-2.699	4.861	1.849	87.267	10.655	12.893
1524	124	19	16	C	W	44.392	-2.145	-4.035	3.252	2.241	3.789	-3.054	87.346	10.659	12.889
1525	124	19	16	C	W	49.107	-1.983	4.468	3.072	2.064	-4.209	-2.894	87.518	12.906	10.644
1526	124	19	16	C	W	56.996	-3.388	-3.774	4.672	3.635	3.452	-4.273	83.146	17.335	7.981
1527	124	19	16	C	W	58.641	-3.292	-4.218	4.559	3.523	3.866	-4.178	83.453	7.985	17.313
1528	124	19	16	C	W	60.000	5.011	1.595	-3.675	-4.554	-1.722	3.967	83.386	7.983	17.328
1529	124	19	16	C	W	61.359	-3.292	4.218	4.559	3.523	-3.866	-4.178	83.453	17.313	7.985
1530	124	19	16	C	W	63.005	-3.388	3.774	4.673	3.635	-3.452	-4.273	83.146	17.335	7.981

BL number	Atoms	γ-PC unit cells	WS_2 unit cells	γ-PC origin atom	WS_2 origin atom	Twist-angle (°)	γ-PC strain 1 (%)	γ-PC strain 2 (%)	γ-PC strain 3 (%)	WS_2 strain 1 (%)	WS_2 strain 2 (%)	WS_2 strain 3 (%)	γ (°)	**|a|**(Å)	**|b|**(Å)
1531	124	19	16	C	W	70.893	-1.983	-4.468	3.072	2.064	4.209	-2.894	87.518	12.906	10.644
1532	124	19	16	C	W	75.609	-2.145	4.035	3.252	2.241	-3.789	-3.054	87.346	12.889	10.659
1533	124	19	16	C	W	76.102	2.853	4.688	-1.783	-2.699	-4.861	1.849	87.267	10.655	12.893
1534	124	19	16	C	W	103.898	2.853	-4.688	-1.783	-2.699	4.861	1.849	87.267	10.655	12.893
1535	124	19	16	C	W	104.392	-2.145	-4.035	3.252	2.241	3.789	-3.054	87.346	10.659	12.889
1536	124	19	16	C	W	109.107	-1.983	4.468	3.072	2.064	-4.209	-2.894	87.518	12.906	10.644
1537	124	19	16	C	W	116.996	-3.388	-3.774	4.672	3.635	3.452	-4.273	83.146	17.335	7.981
1538	124	19	16	C	W	118.641	-3.292	-4.218	4.559	3.523	3.866	-4.178	83.453	7.985	17.313
1539	124	19	16	P	S	0.000	5.011	1.595	-3.675	-4.554	-1.722	3.967	83.387	7.983	17.328
1540	124	19	16	P	S	1.359	-3.292	4.218	4.559	3.523	-3.866	-4.178	83.453	17.313	7.985
1541	124	19	16	P	S	3.004	-3.388	3.774	4.673	3.635	-3.452	-4.273	83.146	17.335	7.981
1542	124	19	16	P	S	10.893	-1.983	-4.468	3.072	2.064	4.209	-2.894	87.518	12.906	10.644
1543	124	19	16	P	S	15.608	-2.145	4.035	3.252	2.241	-3.789	-3.054	87.346	12.889	10.659
1544	124	19	16	P	S	16.102	2.853	4.688	-1.783	-2.699	-4.861	1.849	87.267	10.655	12.893
1545	124	19	16	P	S	43.898	2.853	-4.688	-1.783	-2.699	4.861	1.849	87.267	10.655	12.893
1546	124	19	16	P	S	44.392	-2.145	-4.035	3.252	2.241	3.789	-3.054	87.346	10.659	12.889
1547	124	19	16	P	S	49.107	-1.983	4.468	3.072	2.064	-4.209	-2.894	87.518	12.906	10.644
1548	124	19	16	P	S	56.996	-3.388	-3.774	4.672	3.635	3.452	-4.273	83.146	17.335	7.981
1549	124	19	16	P	S	58.641	-3.292	-4.218	4.559	3.523	3.866	-4.178	83.453	7.985	17.313
1550	124	19	16	P	S	60.000	5.011	1.595	-3.675	-4.554	-1.722	3.967	83.386	7.983	17.328
1551	124	19	16	P	S	61.359	-3.292	4.218	4.559	3.523	-3.866	-4.178	83.453	17.313	7.985
1552	124	19	16	P	S	63.005	-3.388	3.774	4.673	3.635	-3.452	-4.273	83.146	17.335	7.981
1553	124	19	16	P	S	70.893	-1.983	-4.468	3.072	2.064	4.209	-2.894	87.518	12.906	10.644
1554	124	19	16	P	S	75.609	-2.145	4.035	3.252	2.241	-3.789	-3.054	48.839	17.102	10.659
1555	124	19	16	P	S	76.102	2.853	4.688	-1.783	-2.699	-4.861	1.849	87.267	10.655	12.893
1556	124	19	16	P	S	103.898	2.853	-4.688	-1.783	-2.699	4.861	1.849	87.267	10.655	12.893
1557	124	19	16	P	S	104.392	-2.145	-4.035	3.252	2.241	3.789	-3.054	87.346	10.659	12.889
1558	124	19	16	P	S	109.107	-1.983	4.468	3.072	2.064	-4.209	-2.894	87.518	12.906	10.644
1559	124	19	16	P	S	116.996	-3.388	-3.774	4.672	3.635	3.452	-4.273	83.146	17.335	7.981
1560	124	19	16	P	S	118.641	-3.292	-4.218	4.559	3.523	3.866	-4.178	83.453	7.985	17.313
1561	124	19	16	P	W	0.000	5.011	1.595	-3.675	-4.554	-1.722	3.967	83.387	7.983	17.328
1562	124	19	16	P	W	1.359	-3.292	4.218	4.559	3.523	-3.866	-4.178	83.453	17.313	7.985
1563	124	19	16	P	W	3.004	-3.388	3.774	4.673	3.635	-3.452	-4.273	83.146	17.335	7.981
1564	124	19	16	P	W	10.893	-1.983	-4.468	3.072	2.064	4.209	-2.894	87.518	12.906	10.644
1565	124	19	16	P	W	15.608	-2.145	4.035	3.252	2.241	-3.789	-3.054	87.346	12.889	10.659
1566	124	19	16	P	W	16.102	2.853	4.688	-1.783	-2.699	-4.861	1.849	87.267	10.655	12.893
1567	124	19	16	P	W	43.898	2.853	-4.688	-1.783	-2.699	4.861	1.849	87.267	10.655	12.893
1568	124	19	16	P	W	44.392	-2.145	-4.035	3.252	2.241	3.789	-3.054	87.346	10.659	12.889
1569	124	19	16	P	W	49.107	-1.983	4.468	3.072	2.064	-4.209	-2.894	87.518	12.906	10.644
1570	124	19	16	P	W	56.996	-3.388	-3.774	4.672	3.635	3.452	-4.273	83.146	17.335	7.981
1571	124	19	16	P	W	58.641	-3.292	-4.218	4.559	3.523	3.866	-4.178	83.453	7.985	17.313
1572	124	19	16	P	W	60.000	5.011	1.595	-3.675	-4.554	-1.722	3.967	83.386	7.983	17.328
1573	124	19	16	P	W	61.359	-3.292	4.218	4.559	3.523	-3.866	-4.178	83.453	17.313	7.985
1574	124	19	16	P	W	63.005	-3.388	3.774	4.673	3.635	-3.452	-4.273	83.146	17.335	7.981
1575	124	19	16	P	W	70.893	-1.983	-4.468	3.072	2.064	4.209	-2.894	87.518	12.906	10.644
1576	124	19	16	P	W	75.609	-2.145	4.035	3.252	2.241	-3.789	-3.054	87.346	12.889	10.659
1577	124	19	16	P	W	76.102	2.853	4.688	-1.783	-2.699	-4.861	1.849	87.267	10.655	12.893
1578	124	19	16	P	W	103.898	2.853	-4.688	-1.783	-2.699	4.861	1.849	87.267	10.655	12.893
1579	124	19	16	P	W	104.392	-2.145	-4.035	3.252	2.241	3.789	-3.054	87.346	10.659	12.889
1580	124	19	16	P	W	109.107	-1.983	4.468	3.072	2.064	-4.209	-2.894	87.518	12.906	10.644
1581	124	19	16	P	W	116.996	-3.388	-3.774	4.672	3.635	3.452	-4.273	83.146	17.335	7.981

BL number	Atoms	γ-PC unit cells	WS_2 unit cells	γ-PC origin atom	WS_2 origin atom	Twist-angle (°)	γ-PC strain 1 (%)	γ-PC strain 2 (%)	γ-PC strain 3 (%)	WS_2 strain 1 (%)	WS_2 strain 2 (%)	WS_2 strain 3 (%)	γ (°)	|**a**|(Å)	|**b**|(Å)
1582	124	19	16	P	W	118.641	-3.292	-4.218	4.559	3.523	3.866	-4.178	83.453	7.985	17.313
1583	125	20	15	C	S	8.948	-0.599	-4.304	-4.057	0.606	4.684	4.415	85.608	11.436	11.959
1584	125	20	15	C	S	9.515	-4.497	-3.499	-0.121	4.941	3.508	0.121	85.689	11.961	11.435
1585	125	20	15	C	S	13.898	-4.228	4.147	-0.414	4.619	-4.182	0.417	85.497	11.941	11.456
1586	125	20	15	C	S	25.285	-4.781	-2.474	0.193	5.287	2.464	-0.192	86.756	14.079	9.705
1587	125	20	15	C	S	25.767	0.353	-1.542	-4.926	-0.351	1.710	5.464	86.784	9.711	14.070
1588	125	20	15	C	S	34.233	0.353	1.542	-4.926	-0.351	-1.710	5.464	86.784	14.070	9.711
1589	125	20	15	C	S	34.715	-4.781	2.474	0.193	5.287	-2.464	-0.192	86.756	9.705	14.079
1590	125	20	15	C	S	46.102	-4.228	-4.147	-0.414	4.619	4.182	0.417	72.294	9.921	14.429
1591	125	20	15	C	S	50.485	-4.497	3.499	-0.121	4.941	-3.508	0.121	85.689	11.961	11.435
1592	125	20	15	C	S	51.052	-0.599	4.304	-4.057	0.606	-4.684	4.415	85.608	11.959	11.436
1593	125	20	15	C	S	68.948	-0.599	-4.304	-4.057	0.606	4.684	4.415	85.608	11.436	11.959
1594	125	20	15	C	S	69.515	-4.497	-3.499	-0.121	4.941	3.508	0.121	85.689	11.961	11.435
1595	125	20	15	C	S	73.898	-0.414	3.981	-4.228	0.417	-4.348	4.619	85.511	11.445	11.952
1596	125	20	15	C	S	85.285	-4.781	-2.474	0.193	5.287	2.464	-0.192	86.757	14.079	9.705
1597	125	20	15	C	S	85.767	0.353	-1.542	-4.926	-0.351	1.710	5.464	86.783	9.711	14.070
1598	125	20	15	C	S	94.233	0.353	1.542	-4.926	-0.351	-1.710	5.464	86.783	14.070	9.711
1599	125	20	15	C	S	94.715	-4.781	2.474	0.193	5.287	-2.464	-0.192	86.757	9.705	14.079
1600	125	20	15	C	S	106.102	-0.414	-3.981	-4.228	0.417	4.348	4.619	85.511	11.445	11.952
1601	125	20	15	C	S	110.485	-4.497	3.499	-0.121	4.941	-3.508	0.121	85.689	11.961	11.435
1602	125	20	15	C	S	111.052	-0.599	4.304	-4.057	0.606	-4.684	4.415	85.608	11.959	11.436
1603	125	20	15	C	W	8.948	-0.599	-4.304	-4.057	0.606	4.684	4.415	85.608	11.436	11.959
1604	125	20	15	C	W	9.515	-4.497	-3.499	-0.121	4.941	3.508	0.121	85.689	11.961	11.435
1605	125	20	15	C	W	13.898	-4.228	4.147	-0.414	4.619	-4.182	0.417	85.497	11.941	11.456
1606	125	20	15	C	W	25.285	-4.781	-2.474	0.193	5.287	2.464	-0.192	86.756	14.079	9.705
1607	125	20	15	C	W	25.767	0.353	-1.542	-4.926	-0.351	1.710	5.464	86.784	9.711	14.070
1608	125	20	15	C	W	34.233	0.353	1.542	-4.926	-0.351	-1.710	5.464	86.784	14.070	9.711
1609	125	20	15	C	W	34.715	-4.781	2.474	0.193	5.287	-2.464	-0.192	86.756	9.705	14.079
1610	125	20	15	C	W	46.102	-4.228	-4.147	-0.414	4.619	4.182	0.417	72.294	9.921	14.429
1611	125	20	15	C	W	50.485	-4.497	3.499	-0.121	4.941	-3.508	0.121	85.689	11.961	11.435
1612	125	20	15	C	W	51.052	-0.599	4.304	-4.057	0.606	-4.684	4.415	85.608	11.959	11.436
1613	125	20	15	C	W	68.948	-0.599	-4.304	-4.057	0.606	4.684	4.415	85.608	11.436	11.959
1614	125	20	15	C	W	69.515	-4.497	-3.499	-0.121	4.941	3.508	0.121	85.689	11.961	11.435
1615	125	20	15	C	W	73.898	-0.414	3.981	-4.228	0.417	-4.348	4.619	85.511	11.445	11.952
1616	125	20	15	C	W	85.285	-4.781	-2.474	0.193	5.287	2.464	-0.192	86.757	14.079	9.705
1617	125	20	15	C	W	85.767	0.353	-1.542	-4.926	-0.351	1.710	5.464	86.783	9.711	14.070
1618	125	20	15	C	W	94.233	0.353	1.542	-4.926	-0.351	-1.710	5.464	86.783	14.070	9.711
1619	125	20	15	C	W	94.715	-4.781	2.474	0.193	5.287	-2.464	-0.192	86.757	9.705	14.079
1620	125	20	15	C	W	106.102	-0.414	-3.981	-4.228	0.417	4.348	4.619	85.511	11.445	11.952
1621	125	20	15	C	W	110.485	-4.497	3.499	-0.121	4.941	-3.508	0.121	85.689	11.961	11.435
1622	125	20	15	C	W	111.052	-0.599	4.304	-4.057	0.606	-4.684	4.415	85.608	11.959	11.436
1623	125	20	15	P	S	46.102	-4.228	-4.147	-0.414	4.619	4.182	0.417	72.294	9.921	14.429
1624	125	20	15	P	S	68.948	-0.599	-4.304	-4.057	0.606	4.684	4.415	85.608	11.436	11.959
1625	125	20	15	P	S	73.898	-4.228	4.147	-0.414	4.619	-4.182	0.417	72.294	9.921	14.429
1626	125	20	15	P	W	8.948	-0.599	-4.304	-4.057	0.606	4.684	4.415	85.608	11.436	11.959
1627	125	20	15	P	W	13.898	-4.228	4.147	-0.414	4.619	-4.182	0.417	85.497	11.941	11.456
1628	125	20	15	P	W	106.102	-4.228	-4.147	-0.414	4.619	4.182	0.417	85.497	11.456	11.941
1629	126	18	18	C	S	21.787	5.011	-3.368	5.011	-4.554	3.061	-4.555	77.346	7.983	18.219
1630	126	18	18	C	S	38.213	5.011	3.368	5.011	-4.554	-3.061	-4.555	77.346	7.983	18.219
1631	126	18	18	C	S	81.787	5.011	-3.368	5.011	-4.554	3.061	-4.555	77.345	7.983	18.219
1632	126	18	18	C	S	98.213	5.011	3.368	5.011	-4.554	-3.061	-4.555	77.346	7.983	18.219

BL number	Atoms	γ-PC unit cells	WS_2 unit cells	γ-PC origin atom	WS_2 origin atom	Twist-angle (°)	γ-PC strain 1 (%)	γ-PC strain 2 (%)	γ-PC strain 3 (%)	WS_2 strain 1 (%)	WS_2 strain 2 (%)	WS_2 strain 3 (%)	γ (°)	$\lvert\mathbf{a}\rvert$(Å)	$\lvert\mathbf{b}\rvert$(Å)
1633	126	18	18	C	W	21.787	5.011	-3.368	5.011	-4.554	3.061	-4.555	77.346	7.983	18.219
1634	126	18	18	C	W	38.213	5.011	3.368	5.011	-4.554	-3.061	-4.555	77.346	7.983	18.219
1635	126	18	18	C	W	81.787	5.011	-3.368	5.011	-4.554	3.061	-4.555	77.345	7.983	18.219
1636	126	18	18	C	W	98.213	5.011	3.368	5.011	-4.554	-3.061	-4.555	77.346	7.983	18.219
1637	126	18	18	P	S	21.787	5.011	-3.368	5.011	-4.554	3.061	-4.555	77.346	7.983	18.219
1638	126	18	18	P	S	38.213	5.011	3.368	5.011	-4.554	-3.061	-4.555	77.346	7.983	18.219
1639	126	18	18	P	S	81.787	5.011	-3.368	5.011	-4.554	3.061	-4.555	77.345	7.983	18.219
1640	126	18	18	P	S	98.213	5.011	3.368	5.011	-4.554	-3.061	-4.555	77.345	7.983	18.219
1641	126	18	18	P	W	21.787	5.011	-3.368	5.011	-4.554	3.061	-4.555	77.346	7.983	18.219
1642	126	18	18	P	W	38.213	5.011	3.368	5.011	-4.554	-3.061	-4.555	77.346	7.983	18.219
1643	126	18	18	P	W	81.787	5.011	-3.368	5.011	-4.554	3.061	-4.555	77.345	7.983	18.219
1644	126	18	18	P	W	98.213	5.011	3.368	5.011	-4.554	-3.061	-4.555	77.346	7.983	18.219
1645	127	19	17	C	S	21.787	5.011	-3.191	-0.780	-4.554	3.241	0.792	86.320	7.983	17.766
1646	127	19	17	C	S	25.050	-0.955	2.621	5.207	0.973	-2.374	-4.716	86.515	17.772	7.979
1647	127	19	17	C	S	34.950	-0.955	-2.621	5.207	0.973	2.374	-4.716	86.515	17.772	7.979
1648	127	19	17	C	S	38.213	5.011	3.191	-0.780	-4.554	-3.242	0.792	86.320	7.983	17.766
1649	127	19	17	C	S	81.787	5.011	-3.191	-0.780	-4.554	3.241	0.792	86.320	7.983	17.766
1650	127	19	17	C	S	85.050	-0.955	2.621	5.207	0.973	-2.374	-4.716	86.515	17.772	7.979
1651	127	19	17	C	S	94.950	-0.955	-2.621	5.207	0.973	2.374	-4.716	86.515	17.772	7.979
1652	127	19	17	C	S	98.213	5.011	3.191	-0.780	-4.554	-3.242	0.792	86.320	7.983	17.766
1653	127	19	17	C	W	21.787	5.011	-3.191	-0.780	-4.554	3.241	0.792	86.320	7.983	17.766
1654	127	19	17	C	W	25.050	-0.955	2.621	5.207	0.973	-2.374	-4.716	86.515	17.772	7.979
1655	127	19	17	C	W	34.950	-0.955	-2.621	5.207	0.973	2.374	-4.716	86.515	17.772	7.979
1656	127	19	17	C	W	38.213	5.011	3.191	-0.780	-4.554	-3.242	0.792	86.320	7.983	17.766
1657	127	19	17	C	W	81.787	5.011	-3.191	-0.780	-4.554	3.241	0.792	86.320	7.983	17.766
1658	127	19	17	C	W	85.050	-0.955	2.621	5.207	0.973	-2.374	-4.716	86.515	17.772	7.979
1659	127	19	17	C	W	94.950	-0.955	-2.621	5.207	0.973	2.374	-4.716	86.515	17.772	7.979
1660	127	19	17	C	W	98.213	5.011	3.191	-0.780	-4.554	-3.242	0.792	86.320	7.983	17.766
1661	127	19	17	P	S	21.787	5.011	-3.191	-0.780	-4.554	3.241	0.792	86.320	7.983	17.766
1662	127	19	17	P	S	25.050	-0.955	2.621	5.207	0.973	-2.374	-4.716	86.515	17.772	7.979
1663	127	19	17	P	S	34.950	-0.955	-2.621	5.207	0.973	2.374	-4.716	86.515	17.772	7.979
1664	127	19	17	P	S	38.213	5.011	3.191	-0.780	-4.554	-3.242	0.792	86.320	7.983	17.766
1665	127	19	17	P	S	81.787	5.011	-3.191	-0.780	-4.554	3.241	0.792	86.320	7.983	17.766
1666	127	19	17	P	S	85.050	-0.955	2.621	5.207	0.973	-2.374	-4.716	86.515	17.772	7.979
1667	127	19	17	P	S	94.950	-0.955	-2.621	5.207	0.973	2.374	-4.716	86.515	17.772	7.979
1668	127	19	17	P	S	98.213	5.011	3.191	-0.780	-4.554	-3.242	0.792	86.320	7.983	17.766
1669	127	19	17	P	W	21.787	5.011	-3.191	-0.780	-4.554	3.241	0.792	86.320	7.983	17.766
1670	127	19	17	P	W	25.050	-0.955	2.621	5.207	0.973	-2.374	-4.716	86.515	17.772	7.979
1671	127	19	17	P	W	34.950	-0.955	-2.621	5.207	0.973	2.374	-4.716	86.515	17.772	7.979
1672	127	19	17	P	W	38.213	5.011	3.191	-0.780	-4.554	-3.242	0.792	86.320	7.983	17.766
1673	127	19	17	P	W	81.787	5.011	-3.191	-0.780	-4.554	3.241	0.792	86.320	7.983	17.766
1674	127	19	17	P	W	85.050	-0.955	2.621	5.207	0.973	-2.374	-4.716	86.515	17.772	7.979
1675	127	19	17	P	W	94.950	-0.955	-2.621	5.207	0.973	2.374	-4.716	86.515	17.772	7.979
1676	127	19	17	P	W	98.213	5.011	3.191	-0.780	-4.554	-3.242	0.792	86.320	7.983	17.766
1677	128	20	16	C	S	30.000	0.817	0.000	-2.359	-0.804	0.000	2.476	90.000	25.086	5.611
1678	128	20	16	C	S	90.000	0.817	0.000	-2.359	-0.804	0.000	2.476	90.000	25.086	5.611
1679	128	20	16	C	W	30.000	0.817	0.000	-2.359	-0.804	0.000	2.476	90.000	25.086	5.611
1680	128	20	16	C	W	90.000	0.817	0.000	-2.359	-0.804	0.000	2.476	90.000	25.086	5.611
1681	128	20	16	P	S	30.000	0.817	0.000	-2.359	-0.804	0.000	2.476	90.000	25.086	5.611
1682	128	20	16	P	S	90.000	0.817	0.000	-2.359	-0.804	0.000	2.476	90.000	25.086	5.611
1683	128	20	16	P	W	30.000	0.817	0.000	-2.359	-0.804	0.000	2.476	90.000	25.086	5.611

BL number	Atoms	γ-PC unit cells	WS_2 unit cells	γ-PC origin atom	WS_2 origin atom	Twist-angle (°)	γ-PC strain 1 (%)	γ-PC strain 2 (%)	γ-PC strain 3 (%)	WS_2 strain 1 (%)	WS_2 strain 2 (%)	WS_2 strain 3 (%)	γ (°)	|**a**|(Å)	|**b**|(Å)
1684	128	20	16	P	W	90.000	0.817	0.000	-2.359	-0.804	0.000	2.476	90.000	25.086	5.611
1685	128	20	16	C	S	10.893	-1.983	-2.122	0.418	2.064	2.105	-0.415	72.412	12.906	11.440
1686	128	20	16	C	S	13.898	-0.414	3.096	-1.177	0.417	-3.171	1.205	72.372	11.445	12.901
1687	128	20	16	C	S	16.102	2.853	-4.454	-4.194	-2.699	4.861	4.578	70.384	10.655	14.038
1688	128	20	16	C	S	17.480	-4.685	-2.317	3.425	5.169	2.169	-3.205	70.307	10.659	14.046
1689	128	20	16	C	S	18.613	3.582	-0.243	-4.818	-3.342	0.268	5.331	78.654	12.573	11.436
1690	128	20	16	C	S	19.842	-4.723	1.965	3.470	5.216	-1.837	-3.245	78.653	11.425	12.584
1691	128	20	16	C	S	40.158	-4.723	-1.965	3.470	5.216	1.837	-3.245	78.653	12.584	11.425
1692	128	20	16	C	S	41.387	3.582	0.243	-4.818	-3.342	-0.268	5.331	78.654	11.436	12.573
1693	128	20	16	C	S	42.520	-4.685	2.317	3.425	5.169	-2.169	-3.205	70.307	14.046	10.659
1694	128	20	16	C	S	43.898	2.853	4.454	-4.194	-2.699	-4.861	4.578	70.384	10.655	14.038
1695	128	20	16	C	S	46.102	-0.414	-3.096	-1.177	0.417	3.170	1.205	72.372	11.445	12.901
1696	128	20	16	C	S	49.107	-1.983	2.122	0.418	2.064	-2.105	-0.415	72.412	12.906	11.440
1697	128	20	16	C	S	70.893	0.418	-2.071	-1.983	-0.415	2.156	2.064	72.389	11.441	12.906
1698	128	20	16	C	S	73.898	-0.414	3.096	-1.177	0.417	-3.171	1.205	72.372	11.445	12.901
1699	128	20	16	C	S	76.102	2.853	-4.454	-4.194	-2.699	4.861	4.578	70.384	10.655	14.038
1700	128	20	16	C	S	77.480	-4.685	-2.317	3.425	5.169	2.169	-3.205	70.307	10.659	14.046
1701	128	20	16	C	S	78.613	3.582	-0.243	-4.818	-3.342	0.268	5.331	78.654	12.573	11.436
1702	128	20	16	C	S	79.842	-4.723	1.965	3.470	5.216	-1.837	-3.245	78.653	11.425	12.584
1703	128	20	16	C	S	100.158	-4.723	-1.965	3.470	5.216	1.837	-3.245	78.653	12.584	11.425
1704	128	20	16	C	S	101.387	3.582	0.243	-4.818	-3.342	-0.268	5.331	78.653	11.436	12.573
1705	128	20	16	C	S	102.520	-4.685	2.317	3.425	5.169	-2.169	-3.205	70.307	14.046	10.659
1706	128	20	16	C	S	103.898	2.853	4.454	-4.194	-2.699	-4.861	4.578	70.384	10.655	14.038
1707	128	20	16	C	S	106.102	-0.414	-3.096	-1.177	0.417	3.170	1.205	72.372	11.445	12.901
1708	128	20	16	C	S	109.107	0.418	2.071	-1.983	-0.415	-2.156	2.064	72.389	12.906	11.441
1709	128	20	16	C	W	10.893	-1.983	-2.122	0.418	2.064	2.105	-0.415	72.412	12.906	11.440
1710	128	20	16	C	W	13.898	-0.414	3.096	-1.177	0.417	-3.171	1.205	72.372	11.445	12.901
1711	128	20	16	C	W	16.102	2.853	-4.454	-4.194	-2.699	4.861	4.578	70.384	10.655	14.038
1712	128	20	16	C	W	17.480	-4.685	-2.317	3.425	5.169	2.169	-3.205	70.307	10.659	14.046
1713	128	20	16	C	W	18.613	3.582	-0.243	-4.818	-3.342	0.268	5.331	78.654	12.573	11.436
1714	128	20	16	C	W	19.842	-4.723	1.965	3.470	5.216	-1.837	-3.245	78.653	11.425	12.584
1715	128	20	16	C	W	40.158	-4.723	-1.965	3.470	5.216	1.837	-3.245	78.653	12.584	11.425
1716	128	20	16	C	W	41.387	3.582	0.243	-4.818	-3.342	-0.268	5.331	78.654	11.436	12.573
1717	128	20	16	C	W	42.520	-4.685	2.317	3.425	5.169	-2.169	-3.205	70.307	14.046	10.659
1718	128	20	16	C	W	43.898	2.853	4.454	-4.194	-2.699	-4.861	4.578	70.384	10.655	14.038
1719	128	20	16	C	W	46.102	-0.414	-3.096	-1.177	0.417	3.170	1.205	72.372	11.445	12.901
1720	128	20	16	C	W	49.107	-1.983	2.122	0.418	2.064	-2.105	-0.415	72.412	12.906	11.440
1721	128	20	16	C	W	70.893	0.418	-2.071	-1.983	-0.415	2.156	2.064	72.389	11.441	12.906
1722	128	20	16	C	W	73.898	-0.414	3.096	-1.177	0.417	-3.171	1.205	72.372	11.445	12.901
1723	128	20	16	C	W	76.102	2.853	-4.454	-4.194	-2.699	4.861	4.578	70.384	10.655	14.038
1724	128	20	16	C	W	77.480	-4.685	-2.317	3.425	5.169	2.169	-3.205	70.307	10.659	14.046
1725	128	20	16	C	W	78.613	3.582	-0.243	-4.818	-3.342	0.268	5.331	78.654	12.573	11.436
1726	128	20	16	C	W	79.842	-4.723	1.965	3.470	5.216	-1.837	-3.245	78.653	11.425	12.584
1727	128	20	16	C	W	100.158	-4.723	-1.965	3.470	5.216	1.837	-3.245	78.653	12.584	11.425
1728	128	20	16	C	W	101.387	3.582	0.243	-4.818	-3.342	-0.268	5.331	78.653	11.436	12.573
1729	128	20	16	C	W	102.520	-4.685	2.317	3.425	5.169	-2.169	-3.205	70.307	14.046	10.659
1730	128	20	16	C	W	103.898	2.853	4.454	-4.194	-2.699	-4.861	4.578	70.384	10.655	14.038
1731	128	20	16	C	W	106.102	-0.414	-3.096	-1.177	0.417	3.170	1.205	72.372	11.445	12.901
1732	128	20	16	C	W	109.107	0.418	2.071	-1.983	-0.415	-2.156	2.064	72.389	12.906	11.441
1733	128	20	16	P	S	10.893	-1.983	-2.122	0.418	2.064	2.105	-0.415	72.412	12.906	11.440
1734	128	20	16	P	S	13.898	-0.414	3.096	-1.177	0.417	-3.171	1.205	72.372	11.445	12.901

BL number	Atoms	γ-PC unit cells	WS_2 unit cells	γ-PC origin atom	WS_2 origin atom	Twist-angle (°)	γ-PC strain 1 (%)	γ-PC strain 2 (%)	γ-PC strain 3 (%)	WS_2 strain 1 (%)	WS_2 strain 2 (%)	WS_2 strain 3 (%)	γ (°)	|**a**|(Å)	|**b**|(Å)
1735	128	20	16	P	S	16.102	2.853	-4.454	-4.194	-2.699	4.861	4.578	70.384	10.655	14.038
1736	128	20	16	P	S	17.480	-4.685	-2.317	3.425	5.169	2.169	-3.205	70.307	10.659	14.046
1737	128	20	16	P	S	18.613	3.582	-0.243	-4.818	-3.342	0.268	5.331	78.654	12.573	11.436
1738	128	20	16	P	S	19.842	-4.723	1.965	3.470	5.216	-1.837	-3.245	78.653	11.425	12.584
1739	128	20	16	P	S	40.158	-4.723	-1.965	3.470	5.216	1.837	-3.245	78.653	12.584	11.425
1740	128	20	16	P	S	41.387	3.582	0.243	-4.818	-3.342	-0.268	5.331	78.654	11.436	12.573
1741	128	20	16	P	S	42.520	-4.685	2.317	3.425	5.169	-2.169	-3.205	70.307	14.046	10.659
1742	128	20	16	P	S	43.898	2.853	4.454	-4.194	-2.699	-4.861	4.578	70.384	10.655	14.038
1743	128	20	16	P	S	46.102	-0.414	-3.096	-1.177	0.417	3.170	1.205	72.372	11.445	12.901
1744	128	20	16	P	S	49.107	-1.983	2.122	0.418	2.064	-2.105	-0.415	72.412	12.906	11.440
1745	128	20	16	P	S	70.893	0.418	-2.071	-1.983	-0.415	2.156	2.064	72.389	11.441	12.906
1746	128	20	16	P	S	73.898	-0.414	3.096	-1.177	0.417	-3.171	1.205	72.372	11.445	12.901
1747	128	20	16	P	S	76.102	2.853	-4.454	-4.194	-2.699	4.861	4.578	70.384	10.655	14.038
1748	128	20	16	P	S	77.480	-4.685	-2.317	3.425	5.169	2.169	-3.205	70.307	10.659	14.046
1749	128	20	16	P	S	78.613	3.582	-0.243	-4.818	-3.342	0.268	5.331	78.654	12.573	11.436
1750	128	20	16	P	S	79.842	-4.723	1.965	3.470	5.216	-1.837	-3.245	78.653	11.425	12.584
1751	128	20	16	P	S	100.158	-4.723	-1.965	3.470	5.216	1.837	-3.245	78.653	12.584	11.425
1752	128	20	16	P	S	101.387	3.582	0.243	-4.818	-3.342	-0.268	5.331	78.653	11.436	12.573
1753	128	20	16	P	S	102.520	-4.685	2.317	3.425	5.169	-2.169	-3.205	70.307	14.046	10.659
1754	128	20	16	P	S	103.898	2.853	4.454	-4.194	-2.699	-4.861	4.578	70.384	10.655	14.038
1755	128	20	16	P	S	106.102	-0.414	-3.096	-1.177	0.417	3.170	1.205	72.372	11.445	12.901
1756	128	20	16	P	S	109.107	0.418	2.071	-1.983	-0.415	-2.156	2.064	72.389	12.906	11.441
1757	128	20	16	P	W	10.893	-1.983	-2.122	0.418	2.064	2.105	-0.415	72.412	12.906	11.440
1758	128	20	16	P	W	13.898	-0.414	3.096	-1.177	0.417	-3.171	1.205	72.372	11.445	12.901
1759	128	20	16	P	W	16.102	2.853	-4.454	-4.194	-2.699	4.861	4.578	70.384	10.655	14.038
1760	128	20	16	P	W	17.480	-4.685	-2.317	3.425	5.169	2.169	-3.205	70.307	10.659	14.046
1761	128	20	16	P	W	18.613	3.582	-0.243	-4.818	-3.342	0.268	5.331	78.654	12.573	11.436
1762	128	20	16	P	W	19.842	-4.723	1.965	3.470	5.216	-1.837	-3.245	78.653	11.425	12.584
1763	128	20	16	P	W	40.158	-4.723	-1.965	3.470	5.216	1.837	-3.245	78.653	12.584	11.425
1764	128	20	16	P	W	41.387	3.582	0.243	-4.818	-3.342	-0.268	5.331	78.654	11.436	12.573
1765	128	20	16	P	W	42.520	-4.685	2.317	3.425	5.169	-2.169	-3.205	70.307	14.046	10.659
1766	128	20	16	P	W	43.898	2.853	4.454	-4.194	-2.699	-4.861	4.578	70.384	10.655	14.038
1767	128	20	16	P	W	46.102	-0.414	-3.096	-1.177	0.417	3.170	1.205	72.372	11.445	12.901
1768	128	20	16	P	W	49.107	-1.983	2.122	0.418	2.064	-2.105	-0.415	72.412	12.906	11.440
1769	128	20	16	P	W	70.893	0.418	-2.071	-1.983	-0.415	2.156	2.064	72.389	11.441	12.906
1770	128	20	16	P	W	73.898	-0.414	3.096	-1.177	0.417	-3.171	1.205	72.372	11.445	12.901
1771	128	20	16	P	W	76.102	2.853	-4.454	-4.194	-2.699	4.861	4.578	70.384	10.655	14.038
1772	128	20	16	P	W	77.480	-4.685	-2.317	3.425	5.169	2.169	-3.205	70.307	10.659	14.046
1773	128	20	16	P	W	78.613	3.582	-0.243	-4.818	-3.342	0.268	5.331	78.654	12.573	11.436
1774	128	20	16	P	W	79.842	-4.723	1.965	3.470	5.216	-1.837	-3.245	78.653	11.425	12.584
1775	128	20	16	P	W	100.158	-4.723	-1.965	3.470	5.216	1.837	-3.245	78.653	12.584	11.425
1776	128	20	16	P	W	101.387	3.582	0.243	-4.818	-3.342	-0.268	5.331	78.653	11.436	12.573
1777	128	20	16	P	W	102.520	-4.685	2.317	3.425	5.169	-2.169	-3.205	70.307	14.046	10.659
1778	128	20	16	P	W	103.898	2.853	4.454	-4.194	-2.699	-4.861	4.578	70.384	10.655	14.038
1779	128	20	16	P	W	106.102	-0.414	-3.096	-1.177	0.417	3.170	1.205	72.372	11.445	12.901
1780	128	20	16	P	W	109.107	0.418	2.071	-1.983	-0.415	-2.156	2.064	72.389	12.906	11.441
1781	129	21	15	C	S	19.842	-4.723	4.865	-2.259	5.216	-5.095	2.366	73.557	9.900	14.725
1782	129	21	15	C	S	40.158	-4.723	-4.865	-2.259	5.216	5.095	2.366	73.557	14.725	9.900
1783	129	21	15	C	S	100.158	-4.723	-4.865	-2.259	5.216	5.095	2.366	73.557	14.725	9.900
1784	129	21	15	C	W	79.842	-4.723	4.865	-2.259	5.216	-5.095	2.366	73.557	9.900	14.725
1785	129	21	15	P	S	19.842	-4.723	4.865	-2.259	5.216	-5.095	2.366	73.557	9.900	14.725

BL number	Atoms	γ-PC unit cells	WS_2 unit cells	γ-PC origin atom	WS_2 origin atom	Twist-angle (°)	γ-PC strain 1 (%)	γ-PC strain 2 (%)	γ-PC strain 3 (%)	WS_2 strain 1 (%)	WS_2 strain 2 (%)	WS_2 strain 3 (%)	γ (°)	$\lvert\mathbf{a}\rvert$(Å)	$\lvert\mathbf{b}\rvert$(Å)
1786	129	21	15	P	S	40.158	-4.723	-4.865	-2.259	5.216	5.095	2.366	73.557	14.725	9.900
1787	129	21	15	P	S	100.158	-4.723	-4.865	-2.259	5.216	5.095	2.366	73.557	14.725	9.900
1788	129	21	15	P	W	79.842	-4.723	4.865	-2.259	5.216	-5.095	2.366	73.557	9.900	14.725
1789	130	19	18	C	S	16.102	2.853	-5.470	4.244	-2.699	5.042	-3.912	81.895	10.655	13.807
1790	130	19	18	C	S	21.787	5.011	4.786	2.116	-4.554	-4.592	-2.030	81.996	7.983	18.426
1791	130	19	18	C	S	25.285	4.262	-2.904	2.834	-3.928	2.748	-2.683	83.697	10.870	13.480
1792	130	19	18	C	S	25.693	2.326	-2.209	4.790	-2.223	2.016	-4.371	83.717	13.473	10.876
1793	130	19	18	C	S	27.796	5.011	1.596	2.116	-4.554	-1.531	-2.030	83.758	10.878	13.469
1794	130	19	18	C	S	31.945	2.281	-2.109	4.836	-2.182	1.923	-4.410	83.713	10.876	13.473
1795	130	19	18	C	S	32.204	5.011	-1.596	2.116	-4.554	1.531	-2.030	83.758	10.878	13.469
1796	130	19	18	C	S	34.715	4.262	2.904	2.834	-3.928	-2.748	-2.683	83.697	13.480	10.870
1797	130	19	18	C	S	38.213	5.011	-4.786	2.116	-4.554	4.592	-2.030	81.996	13.826	10.639
1798	130	19	18	C	S	43.898	2.853	5.469	4.244	-2.699	-5.042	-3.912	81.895	18.455	7.971
1799	130	19	18	C	S	76.102	2.853	-5.470	4.244	-2.699	5.042	-3.912	81.895	18.455	7.971
1800	130	19	18	C	S	81.787	5.011	4.786	2.116	-4.554	-4.592	-2.030	81.996	13.826	10.639
1801	130	19	18	C	S	85.285	4.262	-2.904	2.834	-3.928	2.748	-2.683	83.697	10.870	13.480
1802	130	19	18	C	S	85.694	2.326	-2.209	4.790	-2.223	2.016	-4.371	83.717	13.473	10.876
1803	130	19	18	C	S	87.796	5.011	1.596	2.116	-4.554	-1.531	-2.030	83.758	10.878	13.469
1804	130	19	18	C	S	91.945	2.281	-2.109	4.836	-2.182	1.923	-4.410	83.713	10.876	13.473
1805	130	19	18	C	S	92.204	5.011	-1.596	2.116	-4.554	1.531	-2.030	83.758	10.878	13.469
1806	130	19	18	C	S	94.715	4.262	2.904	2.834	-3.928	-2.748	-2.683	83.697	13.480	10.870
1807	130	19	18	C	S	98.213	5.011	-4.786	2.116	-4.554	4.592	-2.030	81.996	7.983	18.426
1808	130	19	18	C	S	103.898	2.853	5.469	4.244	-2.699	-5.042	-3.912	81.895	10.655	13.807
1809	130	19	18	C	W	16.102	2.853	-5.470	4.244	-2.699	5.042	-3.912	81.895	10.655	13.807
1810	130	19	18	C	W	21.787	5.011	4.786	2.116	-4.554	-4.592	-2.030	81.996	7.983	18.426
1811	130	19	18	C	W	25.285	4.262	-2.904	2.834	-3.928	2.748	-2.683	83.697	10.870	13.480
1812	130	19	18	C	W	25.693	2.326	-2.209	4.790	-2.223	2.016	-4.371	83.717	13.473	10.876
1813	130	19	18	C	W	27.796	5.011	1.596	2.116	-4.554	-1.531	-2.030	83.758	10.878	13.469
1814	130	19	18	C	W	31.945	2.281	-2.109	4.836	-2.182	1.923	-4.410	83.713	10.876	13.473
1815	130	19	18	C	W	32.204	5.011	-1.596	2.116	-4.554	1.531	-2.030	83.758	10.878	13.469
1816	130	19	18	C	W	34.715	4.262	2.904	2.834	-3.928	-2.748	-2.683	83.697	13.480	10.870
1817	130	19	18	C	W	38.213	5.011	-4.786	2.116	-4.554	4.592	-2.030	81.996	13.826	10.639
1818	130	19	18	C	W	43.898	2.853	5.469	4.244	-2.699	-5.042	-3.912	81.895	18.455	7.971
1819	130	19	18	C	W	76.102	2.853	-5.470	4.244	-2.699	5.042	-3.912	81.895	18.455	7.971
1820	130	19	18	C	W	81.787	5.011	4.786	2.116	-4.554	-4.592	-2.030	81.996	13.826	10.639
1821	130	19	18	C	W	85.285	4.262	-2.904	2.834	-3.928	2.748	-2.683	83.697	10.870	13.480
1822	130	19	18	C	W	85.694	2.326	-2.209	4.790	-2.223	2.016	-4.371	83.717	13.473	10.876
1823	130	19	18	C	W	87.796	5.011	1.596	2.116	-4.554	-1.531	-2.030	83.758	10.878	13.469
1824	130	19	18	C	W	91.945	2.281	-2.109	4.836	-2.182	1.923	-4.410	83.713	10.876	13.473
1825	130	19	18	C	W	92.204	5.011	-1.596	2.116	-4.554	1.531	-2.030	83.758	10.878	13.469
1826	130	19	18	C	W	94.715	4.262	2.904	2.834	-3.928	-2.748	-2.683	83.697	13.480	10.870
1827	130	19	18	C	W	98.213	5.011	-4.786	2.116	-4.554	4.592	-2.030	81.996	7.983	18.426
1828	130	19	18	C	W	103.898	2.853	5.469	4.244	-2.699	-5.042	-3.912	81.895	10.655	13.807
1829	130	19	18	P	S	25.285	4.262	-2.904	2.834	-3.928	2.748	-2.683	83.697	10.870	13.480
1830	130	19	18	P	S	25.693	2.326	-2.209	4.790	-2.223	2.016	-4.371	83.717	13.473	10.876
1831	130	19	18	P	S	27.796	5.011	1.596	2.116	-4.554	-1.531	-2.030	83.758	10.878	13.469
1832	130	19	18	P	S	31.945	2.281	-2.109	4.836	-2.182	1.923	-4.410	83.713	10.876	13.473
1833	130	19	18	P	S	32.204	5.011	-1.596	2.116	-4.554	1.531	-2.030	83.758	10.878	13.469
1834	130	19	18	P	S	34.715	4.262	2.904	2.834	-3.928	-2.748	-2.683	83.697	13.480	10.870
1835	130	19	18	P	S	38.213	5.011	-4.786	2.116	-4.554	4.592	-2.030	81.996	13.826	10.639
1836	130	19	18	P	S	43.898	2.853	5.469	4.244	-2.699	-5.042	-3.912	81.895	10.655	13.807

BL number	Atoms	γ-PC unit cells	WS_2 unit cells	γ-PC origin atom	WS_2 origin atom	Twist-angle (°)	γ-PC strain 1 (%)	γ-PC strain 2 (%)	γ-PC strain 3 (%)	WS_2 strain 1 (%)	WS_2 strain 2 (%)	WS_2 strain 3 (%)	γ (°)	$\lvert\mathbf{a}\rvert$(Å)	$\lvert\mathbf{b}\rvert$(Å)
1837	130	19	18	P	S	76.102	2.853	-5.470	4.244	-2.699	5.042	-3.912	81.895	10.655	13.807
1838	130	19	18	P	S	81.787	5.011	4.786	2.116	-4.554	-4.592	-2.030	81.996	13.826	10.639
1839	130	19	18	P	S	85.285	4.262	-2.904	2.834	-3.928	2.748	-2.683	83.697	10.870	13.480
1840	130	19	18	P	S	85.694	2.326	-2.209	4.790	-2.223	2.016	-4.371	83.717	13.473	10.876
1841	130	19	18	P	S	87.796	5.011	1.596	2.116	-4.554	-1.531	-2.030	83.758	10.878	13.469
1842	130	19	18	P	S	91.945	2.281	-2.109	4.836	-2.182	1.923	-4.410	83.713	10.876	13.473
1843	130	19	18	P	S	92.204	5.011	-1.596	2.116	-4.554	1.531	-2.030	83.758	10.878	13.469
1844	130	19	18	P	S	94.715	4.262	2.904	2.834	-3.928	-2.748	-2.683	83.697	13.480	10.870
1845	130	19	18	P	W	16.102	2.853	-5.470	4.244	-2.699	5.042	-3.912	81.895	10.655	13.807
1846	130	19	18	P	W	21.787	5.011	4.786	2.116	-4.554	-4.592	-2.030	81.996	13.826	10.639
1847	130	19	18	P	W	25.285	4.262	-2.904	2.834	-3.928	2.748	-2.683	83.697	10.870	13.480
1848	130	19	18	P	W	25.693	2.326	-2.209	4.790	-2.223	2.016	-4.371	83.717	13.473	10.876
1849	130	19	18	P	W	27.796	5.011	1.596	2.116	-4.554	-1.531	-2.030	83.758	10.878	13.469
1850	130	19	18	P	W	31.945	2.281	-2.109	4.836	-2.182	1.923	-4.410	83.713	10.876	13.473
1851	130	19	18	P	W	32.204	5.011	-1.596	2.116	-4.554	1.531	-2.030	83.758	10.878	13.469
1852	130	19	18	P	W	34.715	4.262	2.904	2.834	-3.928	-2.748	-2.683	83.697	13.480	10.870
1853	130	19	18	P	W	85.285	4.262	-2.904	2.834	-3.928	2.748	-2.683	83.697	10.870	13.480
1854	130	19	18	P	W	85.694	2.326	-2.209	4.790	-2.223	2.016	-4.371	83.717	13.473	10.876
1855	130	19	18	P	W	87.796	5.011	1.596	2.116	-4.554	-1.531	-2.030	83.758	10.878	13.469
1856	130	19	18	P	W	91.945	2.281	-2.109	4.836	-2.182	1.923	-4.410	83.713	10.876	13.473
1857	130	19	18	P	W	92.204	5.011	-1.596	2.116	-4.554	1.531	-2.030	83.758	10.878	13.469
1858	130	19	18	P	W	94.715	4.262	2.904	2.834	-3.928	-2.748	-2.683	83.697	13.480	10.870
1859	130	19	18	P	W	98.213	5.011	-4.786	2.116	-4.554	4.592	-2.030	81.996	13.826	10.639
1860	130	19	18	P	W	103.898	2.853	5.469	4.244	-2.699	-5.042	-3.912	81.895	10.655	13.807
1861	131	20	17	C	S	0.000	5.011	-1.516	-3.241	-4.554	1.621	3.465	87.309	7.983	18.220
1862	131	20	17	C	S	1.359	-3.292	0.855	5.071	3.523	-0.777	-4.604	87.423	18.221	7.982
1863	131	20	17	C	S	10.893	0.418	-3.106	1.019	-0.415	3.044	-0.998	70.231	13.471	11.442
1864	131	20	17	C	S	12.520	2.326	-0.233	-0.841	-2.223	0.237	0.856	70.210	13.473	11.444
1865	131	20	17	C	S	13.898	-0.414	2.211	1.875	0.417	-2.132	-1.807	70.184	11.445	13.472
1866	131	20	17	C	S	16.537	-3.292	-4.728	5.071	3.523	4.292	-4.604	89.533	18.221	7.974
1867	131	20	17	C	S	21.787	5.011	4.547	-3.241	-4.554	-4.862	3.465	89.103	7.983	18.202
1868	131	20	17	C	S	27.796	5.011	1.516	-3.241	-4.554	-1.621	3.465	71.791	10.878	14.059
1869	131	20	17	C	S	32.204	5.011	-1.516	-3.241	-4.554	1.621	3.465	71.791	10.878	14.059
1870	131	20	17	C	S	38.213	5.011	-4.547	-3.241	-4.554	4.862	3.465	67.074	13.826	11.409
1871	131	20	17	C	S	43.463	-3.292	4.728	5.071	3.523	-4.293	-4.604	89.533	18.221	7.974
1872	131	20	17	C	S	46.102	-0.414	-2.211	1.875	0.417	2.131	-1.807	70.184	11.445	13.472
1873	131	20	17	C	S	47.480	2.326	0.233	-0.841	-2.223	-0.237	0.856	70.210	13.473	11.444
1874	131	20	17	C	S	49.107	0.418	3.106	1.018	-0.415	-3.044	-0.998	70.231	11.442	13.471
1875	131	20	17	C	S	58.641	-3.292	-0.855	5.071	3.523	0.777	-4.604	87.424	18.221	7.982
1876	131	20	17	C	S	60.000	5.011	1.516	-3.241	-4.554	-1.621	3.465	87.309	7.983	18.220
1877	131	20	17	C	S	61.359	-3.292	0.855	5.071	3.523	-0.777	-4.604	87.424	18.221	7.982
1878	131	20	17	C	S	70.893	0.418	-3.106	1.019	-0.415	3.044	-0.998	70.231	13.471	11.442
1879	131	20	17	C	S	72.520	2.326	-0.233	-0.841	-2.223	0.237	0.856	70.210	13.473	11.444
1880	131	20	17	C	S	73.898	-0.414	2.211	1.875	0.417	-2.132	-1.807	70.184	11.445	13.472
1881	131	20	17	C	S	76.537	-3.292	-4.728	5.071	3.523	4.292	-4.604	89.533	18.221	7.974
1882	131	20	17	C	S	81.787	5.011	4.547	-3.241	-4.554	-4.862	3.465	67.074	13.826	11.409
1883	131	20	17	C	S	87.796	5.011	1.516	-3.241	-4.554	-1.621	3.465	71.791	10.878	14.059
1884	131	20	17	C	S	92.204	5.011	-1.516	-3.241	-4.554	1.621	3.465	44.110	14.059	14.846
1885	131	20	17	C	S	98.213	5.011	-4.547	-3.241	-4.554	4.862	3.465	89.103	7.983	18.202
1886	131	20	17	C	S	103.463	-3.292	4.728	5.071	3.523	-4.293	-4.604	89.534	18.221	7.974
1887	131	20	17	C	S	106.102	-0.414	-2.211	1.875	0.417	2.131	-1.807	70.184	11.445	13.472

BL number	Atoms	γ-PC unit cells	WS_2 unit cells	γ-PC origin atom	WS_2 origin atom	Twist-angle (°)	γ-PC strain 1 (%)	γ-PC strain 2 (%)	γ-PC strain 3 (%)	WS_2 strain 1 (%)	WS_2 strain 2 (%)	WS_2 strain 3 (%)	γ (°)	|**a**|(Å)	|**b**|(Å)
1888	131	20	17	C	S	107.480	2.326	0.233	-0.841	-2.223	-0.237	0.856	70.210	13.473	11.444
1889	131	20	17	C	S	109.107	0.418	3.106	1.018	-0.415	-3.044	-0.998	70.231	11.442	13.471
1890	131	20	17	C	S	118.641	-3.292	-0.855	5.071	3.523	0.777	-4.604	87.424	18.221	7.982
1891	131	20	17	C	W	0.000	5.011	-1.516	-3.241	-4.554	1.621	3.465	87.309	7.983	18.220
1892	131	20	17	C	W	1.359	-3.292	0.855	5.071	3.523	-0.777	-4.604	87.423	18.221	7.982
1893	131	20	17	C	W	10.893	0.418	-3.106	1.019	-0.415	3.044	-0.998	70.231	13.471	11.442
1894	131	20	17	C	W	12.520	2.326	-0.233	-0.841	-2.223	0.237	0.856	70.210	13.473	11.444
1895	131	20	17	C	W	13.898	-0.414	2.211	1.875	0.417	-2.132	-1.807	70.184	11.445	13.472
1896	131	20	17	C	W	16.537	-3.292	-4.728	5.071	3.523	4.292	-4.604	89.533	18.221	7.974
1897	131	20	17	C	W	21.787	5.011	4.547	-3.241	-4.554	-4.862	3.465	89.103	7.983	18.202
1898	131	20	17	C	W	27.796	5.011	1.516	-3.241	-4.554	-1.621	3.465	71.791	10.878	14.059
1899	131	20	17	C	W	32.204	5.011	-1.516	-3.241	-4.554	1.621	3.465	71.791	10.878	14.059
1900	131	20	17	C	W	38.213	5.011	-4.547	-3.241	-4.554	4.862	3.465	67.074	13.826	11.409
1901	131	20	17	C	W	43.463	-3.292	4.728	5.071	3.523	-4.293	-4.604	89.533	18.221	7.974
1902	131	20	17	C	W	46.102	-0.414	-2.211	1.875	0.417	2.131	-1.807	70.184	11.445	13.472
1903	131	20	17	C	W	47.480	2.326	0.233	-0.841	-2.223	-0.237	0.856	70.210	13.473	11.444
1904	131	20	17	C	W	49.107	0.418	3.106	1.018	-0.415	-3.044	-0.998	70.231	11.442	13.471
1905	131	20	17	C	W	58.641	-3.292	-0.855	5.071	3.523	0.777	-4.604	87.424	18.221	7.982
1906	131	20	17	C	W	60.000	5.011	1.516	-3.241	-4.554	-1.621	3.465	87.309	7.983	18.220
1907	131	20	17	C	W	61.359	-3.292	0.855	5.071	3.523	-0.777	-4.604	87.424	18.221	7.982
1908	131	20	17	C	W	70.893	0.418	-3.106	1.019	-0.415	3.044	-0.998	70.231	13.471	11.442
1909	131	20	17	C	W	72.520	2.326	-0.233	-0.841	-2.223	0.237	0.856	70.210	13.473	11.444
1910	131	20	17	C	W	73.898	-0.414	2.211	1.875	0.417	-2.132	-1.807	70.184	11.445	13.472
1911	131	20	17	C	W	76.537	-3.292	-4.728	5.071	3.523	4.292	-4.604	89.533	18.221	7.974
1912	131	20	17	C	W	81.787	5.011	4.547	-3.241	-4.554	-4.862	3.465	67.074	13.826	11.409
1913	131	20	17	C	W	87.796	5.011	1.516	-3.241	-4.554	-1.621	3.465	71.791	10.878	14.059
1914	131	20	17	C	W	92.204	5.011	-1.516	-3.241	-4.554	1.621	3.465	71.791	10.878	14.059
1915	131	20	17	C	W	98.213	5.011	-4.547	-3.241	-4.554	4.862	3.465	89.103	7.983	18.202
1916	131	20	17	C	W	103.463	-3.292	4.728	5.071	3.523	-4.293	-4.604	89.534	18.221	7.974
1917	131	20	17	C	W	106.102	-0.414	-2.211	1.875	0.417	2.131	-1.807	70.184	11.445	13.472
1918	131	20	17	C	W	107.480	2.326	0.233	-0.841	-2.223	-0.237	0.856	70.210	13.473	11.444
1919	131	20	17	C	W	109.107	0.418	3.106	1.018	-0.415	-3.044	-0.998	70.231	11.442	13.471
1920	131	20	17	C	W	118.641	-3.292	-0.855	5.071	3.523	0.777	-4.604	87.424	18.221	7.982
1921	131	20	17	P	S	0.000	5.011	-1.516	-3.241	-4.554	1.621	3.465	87.309	7.983	18.220
1922	131	20	17	P	S	1.359	-3.292	0.855	5.071	3.523	-0.777	-4.604	87.423	18.221	7.982
1923	131	20	17	P	S	10.893	0.418	-3.106	1.019	-0.415	3.044	-0.998	70.231	13.471	11.442
1924	131	20	17	P	S	12.520	2.326	-0.233	-0.841	-2.223	0.237	0.856	70.210	13.473	11.444
1925	131	20	17	P	S	13.898	-0.414	2.211	1.875	0.417	-2.132	-1.807	70.184	11.445	13.472
1926	131	20	17	P	S	27.796	5.011	1.516	-3.241	-4.554	-1.621	3.465	71.791	10.878	14.059
1927	131	20	17	P	S	32.204	5.011	-1.516	-3.241	-4.554	1.621	3.465	71.791	10.878	14.059
1928	131	20	17	P	S	38.213	5.011	-4.547	-3.241	-4.554	4.862	3.465	67.074	13.826	11.409
1929	131	20	17	P	S	43.463	-3.292	4.728	5.071	3.523	-4.293	-4.604	89.533	18.221	7.974
1930	131	20	17	P	S	46.102	-0.414	-2.211	1.875	0.417	2.131	-1.807	70.184	11.445	13.472
1931	131	20	17	P	S	47.480	2.326	0.233	-0.841	-2.223	-0.237	0.856	70.210	13.473	11.444
1932	131	20	17	P	S	49.107	0.418	3.106	1.018	-0.415	-3.044	-0.998	70.231	11.442	13.471
1933	131	20	17	P	S	58.641	-3.292	-0.855	5.071	3.523	0.777	-4.604	87.424	18.221	7.982
1934	131	20	17	P	S	60.000	5.011	1.516	-3.241	-4.554	-1.621	3.465	87.309	7.983	18.220
1935	131	20	17	P	S	61.359	-3.292	0.855	5.071	3.523	-0.777	-4.604	87.424	18.221	7.982
1936	131	20	17	P	S	70.893	0.418	-3.106	1.019	-0.415	3.044	-0.998	70.231	13.471	11.442
1937	131	20	17	P	S	72.520	2.326	-0.233	-0.841	-2.223	0.237	0.856	70.210	13.473	11.444
1938	131	20	17	P	S	73.898	-0.414	2.211	1.875	0.417	-2.132	-1.807	70.184	11.445	13.472

BL number	Atoms	γ-PC unit cells	WS_2 unit cells	γ-PC origin atom	WS_2 origin atom	Twist-angle (°)	γ-PC strain 1 (%)	γ-PC strain 2 (%)	γ-PC strain 3 (%)	WS_2 strain 1 (%)	WS_2 strain 2 (%)	WS_2 strain 3 (%)	γ (°)	$\lvert\mathbf{a}\rvert$(Å)	$\lvert\mathbf{b}\rvert$(Å)
1939	131	20	17	P	S	76.537	-3.292	-4.728	5.071	3.523	4.292	-4.604	89.533	18.221	7.974
1940	131	20	17	P	S	81.787	5.011	4.547	-3.241	-4.554	-4.862	3.465	67.074	13.826	11.409
1941	131	20	17	P	S	87.796	5.011	1.516	-3.241	-4.554	-1.621	3.465	71.791	10.878	14.059
1942	131	20	17	P	S	92.204	5.011	-1.516	-3.241	-4.554	1.621	3.465	44.110	14.059	14.846
1943	131	20	17	P	S	106.102	-0.414	-2.211	1.875	0.417	2.131	-1.807	70.184	11.445	13.472
1944	131	20	17	P	S	107.480	2.326	0.233	-0.841	-2.223	-0.237	0.856	70.210	13.473	11.444
1945	131	20	17	P	S	109.107	0.418	3.106	1.018	-0.415	-3.044	-0.998	70.231	11.442	13.471
1946	131	20	17	P	S	118.641	-3.292	-0.855	5.071	3.523	0.777	-4.604	87.424	18.221	7.982
1947	131	20	17	P	W	0.000	5.011	-1.516	-3.241	-4.554	1.621	3.465	87.309	7.983	18.220
1948	131	20	17	P	W	1.359	-3.292	0.855	5.071	3.523	-0.777	-4.604	87.423	18.221	7.982
1949	131	20	17	P	W	10.893	0.418	-3.106	1.019	-0.415	3.044	-0.998	70.231	13.471	11.442
1950	131	20	17	P	W	12.520	2.326	-0.233	-0.841	-2.223	0.237	0.856	70.210	13.473	11.444
1951	131	20	17	P	W	13.898	-0.414	2.211	1.875	0.417	-2.132	-1.807	70.184	11.445	13.472
1952	131	20	17	P	W	16.537	-3.292	-4.728	5.071	3.523	4.292	-4.604	89.533	18.221	7.974
1953	131	20	17	P	W	21.787	5.011	4.547	-3.241	-4.554	-4.862	3.465	89.103	7.983	18.202
1954	131	20	17	P	W	27.796	5.011	1.516	-3.241	-4.554	-1.621	3.465	71.791	10.878	14.059
1955	131	20	17	P	W	32.204	5.011	-1.516	-3.241	-4.554	1.621	3.465	71.791	10.878	14.059
1956	131	20	17	P	W	46.102	-0.414	-2.211	1.875	0.417	2.131	-1.807	70.184	11.445	13.472
1957	131	20	17	P	W	47.480	2.326	0.233	-0.841	-2.223	-0.237	0.856	70.210	13.473	11.444
1958	131	20	17	P	W	49.107	0.418	3.106	1.018	-0.415	-3.044	-0.998	70.231	11.442	13.471
1959	131	20	17	P	W	58.641	-3.292	-0.855	5.071	3.523	0.777	-4.604	87.424	18.221	7.982
1960	131	20	17	P	W	60.000	5.011	1.516	-3.241	-4.554	-1.621	3.465	87.309	7.983	18.220
1961	131	20	17	P	W	61.359	-3.292	0.855	5.071	3.523	-0.777	-4.604	87.424	18.221	7.982
1962	131	20	17	P	W	70.893	0.418	-3.106	1.019	-0.415	3.044	-0.998	70.231	13.471	11.442
1963	131	20	17	P	W	72.520	2.326	-0.233	-0.841	-2.223	0.237	0.856	70.210	13.473	11.444
1964	131	20	17	P	W	73.898	-0.414	2.211	1.875	0.417	-2.132	-1.807	70.184	11.445	13.472
1965	131	20	17	P	W	87.796	5.011	1.516	-3.241	-4.554	-1.621	3.465	71.791	10.878	14.059
1966	131	20	17	P	W	92.204	5.011	-1.516	-3.241	-4.554	1.621	3.465	71.791	10.878	14.059
1967	131	20	17	P	W	98.213	5.011	-4.547	-3.241	-4.554	4.862	3.465	89.103	7.983	18.202
1968	131	20	17	P	W	103.463	-3.292	4.728	5.071	3.523	-4.293	-4.604	89.534	18.221	7.974
1969	131	20	17	P	W	106.102	-0.414	-2.211	1.875	0.417	2.131	-1.807	70.184	11.445	13.472
1970	131	20	17	P	W	107.480	2.326	0.233	-0.841	-2.223	-0.237	0.856	70.210	13.473	11.444
1971	131	20	17	P	W	109.107	0.418	3.106	1.018	-0.415	-3.044	-0.998	70.231	11.442	13.471
1972	131	20	17	P	W	118.641	-3.292	-0.855	5.071	3.523	0.777	-4.604	87.424	18.221	7.982
1973	132	21	16	C	S	10.893	-1.983	0.000	-1.982	2.064	0.000	2.064	60.000	12.906	12.906
1974	132	21	16	C	S	49.107	-1.983	0.000	-1.982	2.064	0.000	2.064	60.000	12.906	12.906
1975	132	21	16	C	S	70.893	-1.983	0.000	-1.982	2.064	0.000	2.064	60.000	12.906	12.906
1976	132	21	16	C	S	109.107	-1.983	0.000	-1.982	2.064	0.000	2.064	60.000	12.906	12.906
1977	132	21	16	P	S	10.893	-1.983	0.000	-1.982	2.064	0.000	2.064	60.000	12.906	12.906
1978	132	21	16	P	S	49.107	-1.983	0.000	-1.982	2.064	0.000	2.064	60.000	12.906	12.906
1979	132	21	16	P	S	70.893	-1.983	0.000	-1.982	2.064	0.000	2.064	60.000	12.906	12.906
1980	132	21	16	P	S	109.107	-1.983	0.000	-1.982	2.064	0.000	2.064	60.000	12.906	12.906
1981	132	21	16	C	S	15.608	-2.145	-3.075	-1.819	2.241	3.191	1.888	83.523	17.102	8.488
1982	132	21	16	C	S	19.107	-1.485	-5.223	-2.475	1.530	5.495	2.604	73.627	12.580	11.950
1983	132	21	16	C	S	22.689	-4.497	0.909	0.671	4.941	-0.897	-0.662	73.515	11.961	12.585
1984	132	21	16	C	S	23.413	0.481	2.072	-4.326	-0.477	-2.268	4.736	73.513	12.584	11.961
1985	132	21	16	C	S	24.791	-3.388	4.553	-0.534	3.635	-4.603	0.540	78.990	17.335	8.479
1986	132	21	16	C	S	25.050	-0.955	4.875	-2.989	0.973	-5.185	3.179	78.923	17.331	8.481
1987	132	21	16	C	S	34.950	-0.955	-4.875	-2.989	0.973	5.185	3.179	78.923	8.481	17.332
1988	132	21	16	C	S	35.209	-3.388	-4.553	-0.534	3.635	4.603	0.540	78.990	17.335	8.479
1989	132	21	16	C	S	36.587	0.481	-2.072	-4.326	-0.477	2.268	4.736	73.513	12.584	11.961

BL number	Atoms	γ-PC unit cells	WS_2 unit cells	γ-PC origin atom	WS_2 origin atom	Twist-angle (°)	γ-PC strain 1 (%)	γ-PC strain 2 (%)	γ-PC strain 3 (%)	WS_2 strain 1 (%)	WS_2 strain 2 (%)	WS_2 strain 3 (%)	γ (°)	|**a**|(Å)	|**b**|(Å)
1990	132	21	16	C	S	37.311	-4.497	-0.909	0.671	4.941	0.897	-0.662	73.515	11.961	12.585
1991	132	21	16	C	S	40.893	-1.485	5.223	-2.475	1.530	-5.495	2.604	73.627	11.950	12.580
1992	132	21	16	C	S	43.463	-3.292	1.501	-0.637	3.523	-1.520	0.645	83.546	8.488	17.105
1993	132	21	16	C	S	75.609	-2.145	-3.075	-1.819	2.241	3.191	1.888	83.523	17.102	8.488
1994	132	21	16	C	S	79.107	-1.485	-5.223	-2.475	1.530	5.495	2.604	78.889	8.492	17.311
1995	132	21	16	C	S	82.689	-4.497	0.909	0.671	4.941	-0.897	-0.662	73.515	11.961	12.585
1996	132	21	16	C	S	83.413	0.481	2.072	-4.326	-0.477	-2.268	4.736	73.513	12.584	11.961
1997	132	21	16	C	S	84.791	-3.388	4.553	-0.534	3.635	-4.603	0.540	78.990	17.335	8.479
1998	132	21	16	C	S	85.050	-0.955	4.875	-2.989	0.973	-5.185	3.179	78.923	17.331	8.481
1999	132	21	16	C	S	94.950	-0.955	-4.875	-2.989	0.973	5.185	3.179	78.923	8.481	17.332
2000	132	21	16	C	S	95.209	-3.388	-4.553	-0.534	3.635	4.603	0.540	78.990	17.335	8.479
2001	132	21	16	C	S	96.587	0.481	-2.072	-4.326	-0.477	2.268	4.736	73.513	12.584	11.961
2002	132	21	16	C	S	97.311	-4.497	-0.909	0.671	4.941	0.897	-0.662	73.515	11.961	12.585
2003	132	21	16	C	S	100.893	-1.485	-2.902	-2.475	1.530	3.053	2.604	83.549	8.492	17.095
2004	132	21	16	C	S	103.463	-3.292	1.501	-0.637	3.523	-1.520	0.645	83.546	8.488	17.105
2005	132	21	16	C	W	15.608	-2.145	-3.075	-1.819	2.241	3.191	1.888	83.523	17.102	8.488
2006	132	21	16	C	W	19.107	-1.485	-5.223	-2.475	1.530	5.495	2.604	78.889	8.492	17.311
2007	132	21	16	C	W	24.791	-3.388	4.553	-0.534	3.635	-4.603	0.540	78.990	17.335	8.479
2008	132	21	16	C	W	25.050	-0.955	4.875	-2.989	0.973	-5.185	3.179	78.923	17.331	8.481
2009	132	21	16	C	W	34.950	-0.955	-4.875	-2.989	0.973	5.185	3.179	78.923	8.481	17.332
2010	132	21	16	C	W	35.209	-3.388	-4.553	-0.534	3.635	4.603	0.540	78.990	17.335	8.479
2011	132	21	16	C	W	40.893	-1.485	-2.902	-2.475	1.530	3.053	2.604	83.549	8.492	17.095
2012	132	21	16	C	W	43.463	-3.292	1.501	-0.637	3.523	-1.520	0.645	83.546	8.488	17.105
2013	132	21	16	C	W	75.609	-2.145	-3.075	-1.819	2.241	3.191	1.888	83.523	17.102	8.488
2014	132	21	16	C	W	79.107	-1.485	-5.223	-2.475	1.530	5.495	2.604	78.889	8.492	17.311
2015	132	21	16	C	W	84.791	-3.388	4.553	-0.534	3.635	-4.603	0.540	78.990	17.335	8.479
2016	132	21	16	C	W	85.050	-0.955	4.875	-2.989	0.973	-5.185	3.179	78.923	17.331	8.481
2017	132	21	16	C	W	94.950	-0.955	-4.875	-2.989	0.973	5.185	3.179	78.923	8.481	17.332
2018	132	21	16	C	W	95.209	-3.388	-4.553	-0.534	3.635	4.603	0.540	78.990	17.335	8.479
2019	132	21	16	C	W	100.893	-1.485	-2.902	-2.475	1.530	3.053	2.604	83.549	8.492	17.095
2020	132	21	16	C	W	103.463	-3.292	1.501	-0.637	3.523	-1.520	0.645	83.546	8.488	17.105
2021	132	21	16	C	W	104.392	-2.145	3.075	-1.819	2.241	-3.191	1.888	83.523	17.102	8.488
2022	132	21	16	P	S	15.608	-2.145	-3.075	-1.819	2.241	3.191	1.888	83.523	17.102	8.488
2023	132	21	16	P	S	19.107	-1.485	-5.223	-2.475	1.530	5.495	2.604	73.627	12.580	11.950
2024	132	21	16	P	S	22.689	-4.497	0.909	0.671	4.941	-0.897	-0.662	73.515	11.961	12.585
2025	132	21	16	P	S	23.413	0.481	2.072	-4.326	-0.477	-2.268	4.736	73.513	12.584	11.961
2026	132	21	16	P	S	24.791	-3.388	4.553	-0.534	3.635	-4.603	0.540	78.990	17.335	8.479
2027	132	21	16	P	S	25.050	-0.955	4.875	-2.989	0.973	-5.185	3.179	78.923	17.331	8.481
2028	132	21	16	P	S	36.587	0.481	-2.072	-4.326	-0.477	2.268	4.736	73.513	12.584	11.961
2029	132	21	16	P	S	37.311	-4.497	-0.909	0.671	4.941	0.897	-0.662	73.515	11.961	12.585
2030	132	21	16	P	S	40.893	-1.485	5.223	-2.475	1.530	-5.495	2.604	73.627	11.950	12.580
2031	132	21	16	P	S	43.463	-3.292	1.501	-0.637	3.523	-1.520	0.645	83.546	8.488	17.105
2032	132	21	16	P	S	75.609	-2.145	-3.075	-1.819	2.241	3.191	1.888	83.523	17.102	8.488
2033	132	21	16	P	S	79.107	-1.485	2.902	-2.475	1.530	-3.053	2.604	83.549	8.492	17.095
2034	132	21	16	P	S	82.689	-4.497	0.909	0.671	4.941	-0.897	-0.662	73.515	11.961	12.585
2035	132	21	16	P	S	83.413	0.481	2.072	-4.326	-0.477	-2.268	4.736	73.513	12.584	11.961
2036	132	21	16	P	S	94.950	-0.955	-4.875	-2.989	0.973	5.185	3.179	78.923	8.481	17.332
2037	132	21	16	P	S	95.209	-3.388	-4.553	-0.534	3.635	4.603	0.540	78.990	17.335	8.479
2038	132	21	16	P	S	96.587	0.481	-2.072	-4.326	-0.477	2.268	4.736	73.513	12.584	11.961
2039	132	21	16	P	S	97.311	-4.497	-0.909	0.671	4.941	0.897	-0.662	73.515	11.961	12.585
2040	132	21	16	P	S	100.893	-1.485	-2.902	-2.475	1.530	3.053	2.604	83.549	8.492	17.095

BL number	Atoms	γ-PC unit cells	WS_2 unit cells	γ-PC origin atom	WS_2 origin atom	Twist-angle (°)	γ-PC strain 1 (%)	γ-PC strain 2 (%)	γ-PC strain 3 (%)	WS_2 strain 1 (%)	WS_2 strain 2 (%)	WS_2 strain 3 (%)	γ (°)	$\lvert\mathbf{a}\rvert$(Å)	$\lvert\mathbf{b}\rvert$(Å)
2041	132	21	16	P	S	103.463	-3.292	1.501	-0.637	3.523	-1.520	0.645	83.546	8.488	17.105
2042	132	21	16	P	S	104.392	-2.145	3.075	-1.819	2.241	-3.191	1.888	83.523	17.102	8.488
2043	132	21	16	P	W	15.608	-2.145	-3.075	-1.819	2.241	3.191	1.888	83.523	17.102	8.488
2044	132	21	16	P	W	19.107	-1.485	2.902	-2.475	1.530	-3.053	2.604	83.549	8.492	17.095
2045	132	21	16	P	W	34.950	-0.955	-4.875	-2.989	0.973	5.185	3.179	78.923	8.481	17.332
2046	132	21	16	P	W	35.209	-3.388	-4.553	-0.534	3.635	4.603	0.540	78.990	17.335	8.479
2047	132	21	16	P	W	40.893	-1.485	-2.902	-2.475	1.530	3.053	2.604	83.549	8.492	17.095
2048	132	21	16	P	W	43.463	-3.292	1.501	-0.637	3.523	-1.520	0.645	83.546	8.488	17.105
2049	132	21	16	P	W	75.609	-2.145	-3.075	-1.819	2.241	3.191	1.888	83.523	17.102	8.488
2050	132	21	16	P	W	79.107	-1.485	-5.223	-2.475	1.530	5.495	2.604	78.889	8.492	17.311
2051	132	21	16	P	W	84.791	-3.388	4.553	-0.534	3.635	-4.603	0.540	78.990	17.335	8.479
2052	132	21	16	P	W	85.050	-0.955	4.875	-2.989	0.973	-5.185	3.179	78.923	17.331	8.481
2053	132	21	16	P	W	100.893	-1.485	-2.902	-2.475	1.530	3.053	2.604	83.549	8.492	17.095
2054	132	21	16	P	W	103.463	-3.292	1.501	-0.637	3.523	-1.520	0.645	83.546	8.488	17.105
2055	133	19	19	C	S	13.174	5.011	0.000	5.011	-4.554	0.000	-4.555	60.000	13.151	13.151
2056	133	19	19	C	S	46.827	5.011	0.000	5.011	-4.554	0.000	-4.555	60.000	13.151	13.151
2057	133	19	19	C	S	73.174	5.011	0.000	5.011	-4.554	0.000	-4.555	60.000	13.151	13.151
2058	133	19	19	C	S	106.827	5.011	0.000	5.011	-4.554	0.000	-4.555	60.000	13.151	13.151
2059	133	19	19	C	W	13.174	5.011	0.000	5.011	-4.554	0.000	-4.555	60.000	13.151	13.151
2060	133	19	19	C	W	46.827	5.011	0.000	5.011	-4.554	0.000	-4.555	60.000	13.151	13.151
2061	133	19	19	C	W	73.174	5.011	0.000	5.011	-4.554	0.000	-4.555	60.000	13.151	13.151
2062	133	19	19	C	W	106.827	5.011	0.000	5.011	-4.554	0.000	-4.555	60.000	13.151	13.151
2063	133	19	19	P	S	46.827	5.011	0.000	5.011	-4.554	0.000	-4.555	60.000	13.151	13.151
2064	133	19	19	P	S	73.174	5.011	0.000	5.011	-4.554	0.000	-4.555	60.000	13.151	13.151
2065	133	19	19	P	W	13.174	5.011	0.000	5.011	-4.554	0.000	-4.555	60.000	13.151	13.151
2066	133	19	19	P	W	106.827	5.011	0.000	5.011	-4.554	0.000	-4.555	60.000	13.151	13.151
2067	133	22	15	C	S	13.898	-4.228	-2.932	-4.922	4.619	3.252	5.459	85.215	9.921	14.486
2068	133	22	15	C	S	17.480	-4.685	3.028	-4.468	5.169	-3.325	4.906	85.195	14.491	9.918
2069	133	22	15	C	S	46.102	-4.228	2.932	-4.922	4.619	-3.252	5.459	85.215	9.921	14.486
2070	133	22	15	C	S	77.480	-4.685	3.028	-4.468	5.169	-3.325	4.906	85.195	14.491	9.918
2071	133	22	15	C	S	102.520	-4.685	-3.028	-4.468	5.169	3.325	4.906	85.195	14.491	9.918
2072	133	22	15	C	S	106.102	-4.228	2.932	-4.922	4.619	-3.252	5.459	85.215	9.921	14.486
2073	133	22	15	C	W	17.480	-4.685	3.028	-4.468	5.169	-3.325	4.906	85.195	14.491	9.918
2074	133	22	15	C	W	42.520	-4.685	-3.028	-4.468	5.169	3.325	4.906	85.195	14.491	9.918
2075	133	22	15	C	W	46.102	-4.228	2.932	-4.922	4.619	-3.252	5.459	85.215	9.921	14.486
2076	133	22	15	C	W	73.898	-4.228	-2.932	-4.922	4.619	3.252	5.459	85.215	9.921	14.486
2077	133	22	15	C	W	77.480	-4.685	3.028	-4.468	5.169	-3.325	4.906	85.195	14.491	9.918
2078	133	22	15	C	W	106.102	-4.228	2.932	-4.922	4.619	-3.252	5.459	85.215	9.921	14.486
2079	133	22	15	P	S	17.480	-4.685	3.028	-4.468	5.169	-3.325	4.906	85.195	14.491	9.918
2080	133	22	15	P	S	42.520	-4.685	-3.028	-4.468	5.169	3.325	4.906	85.195	14.491	9.918
2081	133	22	15	P	S	46.102	-4.228	2.932	-4.922	4.619	-3.252	5.459	85.215	9.921	14.486
2082	133	22	15	P	S	73.898	-4.228	-2.932	-4.922	4.619	3.252	5.459	85.215	9.921	14.486
2083	133	22	15	P	S	77.480	-4.685	3.028	-4.468	5.169	-3.325	4.906	85.195	14.491	9.918
2084	133	22	15	P	S	106.102	-4.228	2.932	-4.922	4.619	-3.252	5.459	85.215	9.921	14.486
2085	133	22	15	P	W	13.898	-4.228	-2.932	-4.922	4.619	3.252	5.459	85.215	9.921	14.486
2086	133	22	15	P	W	17.480	-4.685	3.028	-4.468	5.169	-3.325	4.906	85.195	14.491	9.918
2087	133	22	15	P	W	46.102	-4.228	2.932	-4.922	4.619	-3.252	5.459	85.215	9.921	14.486
2088	133	22	15	P	W	77.480	-4.685	3.028	-4.468	5.169	-3.325	4.906	85.195	14.491	9.918
2089	133	22	15	P	W	102.520	-4.685	-3.028	-4.468	5.169	3.325	4.906	85.195	14.491	9.918
2090	133	22	15	P	W	106.102	-4.228	2.932	-4.922	4.619	-3.252	5.459	85.215	9.921	14.486
2091	134	20	18	C	S	0.000	5.011	0.000	-0.490	-4.554	0.000	0.495	90.000	5.226	28.591

BL number	Atoms	γ-PC unit cells	WS_2 unit cells	γ-PC origin atom	WS_2 origin atom	Twist-angle (°)	γ-PC strain 1 (%)	γ-PC strain 2 (%)	γ-PC strain 3 (%)	WS_2 strain 1 (%)	WS_2 strain 2 (%)	WS_2 strain 3 (%)	γ (°)	|**a**|(Å)	|**b**|(Å)
2092	134	20	18	C	S	60.000	5.011	0.000	-0.490	-4.554	0.000	0.495	90.000	5.226	28.591
2093	134	20	18	C	W	0.000	5.011	0.000	-0.490	-4.554	0.000	0.495	90.000	5.226	28.591
2094	134	20	18	C	W	60.000	5.011	0.000	-0.490	-4.554	0.000	0.495	90.000	5.226	28.591
2095	134	20	18	P	S	0.000	5.011	0.000	-0.490	-4.554	0.000	0.495	90.000	5.226	28.591
2096	134	20	18	P	W	60.000	5.011	0.000	-0.490	-4.554	0.000	0.495	90.000	5.226	28.591
2097	134	20	18	C	S	10.158	2.281	-5.378	2.095	-2.182	5.162	-2.011	83.141	13.139	11.446
2098	134	20	18	C	S	10.893	4.020	-4.004	0.418	-3.721	3.971	-0.415	76.543	10.637	14.437
2099	134	20	18	C	S	13.898	-0.414	1.327	4.926	0.417	-1.208	-4.484	76.538	14.421	10.653
2100	134	20	18	C	S	16.102	2.853	5.196	1.532	-2.699	-5.041	-1.486	76.436	10.655	14.416
2101	134	20	18	C	S	43.898	2.853	-5.196	1.532	-2.699	5.042	-1.486	82.967	13.138	11.451
2102	134	20	18	C	S	46.102	-0.414	-1.327	4.926	0.417	1.208	-4.484	76.538	10.653	14.421
2103	134	20	18	C	S	49.107	0.418	4.141	4.020	-0.415	-3.833	-3.721	76.566	14.426	10.644
2104	134	20	18	C	S	49.842	2.281	5.378	2.095	-2.182	-5.162	-2.010	83.141	11.446	13.139
2105	134	20	18	C	S	70.158	2.281	-5.378	2.095	-2.182	5.162	-2.011	83.141	13.139	11.446
2106	134	20	18	C	S	70.893	0.418	-4.141	4.020	-0.415	3.833	-3.721	76.566	14.426	10.644
2107	134	20	18	C	S	73.898	-0.414	1.327	4.926	0.417	-1.208	-4.484	76.538	14.421	10.653
2108	134	20	18	C	S	76.102	2.853	5.196	1.532	-2.699	-5.041	-1.486	82.967	11.451	13.138
2109	134	20	18	C	S	103.898	2.853	-5.196	1.532	-2.699	5.042	-1.486	76.436	10.655	14.416
2110	134	20	18	C	S	106.102	-0.414	-1.327	4.926	0.417	1.208	-4.484	83.031	11.445	13.151
2111	134	20	18	C	S	109.107	0.418	4.141	4.020	-0.415	-3.833	-3.721	76.566	14.426	10.644
2112	134	20	18	C	S	109.842	2.281	5.378	2.095	-2.182	-5.162	-2.010	83.141	11.446	13.139
2113	134	20	18	C	W	10.158	2.281	-5.378	2.095	-2.182	5.162	-2.011	83.141	13.139	11.446
2114	134	20	18	C	W	10.893	4.020	-4.004	0.418	-3.721	3.971	-0.415	76.543	10.637	14.437
2115	134	20	18	C	W	13.898	-0.414	1.327	4.926	0.417	-1.208	-4.484	76.538	14.421	10.653
2116	134	20	18	C	W	16.102	2.853	5.196	1.532	-2.699	-5.041	-1.486	76.436	10.655	14.416
2117	134	20	18	C	W	43.898	2.853	-5.196	1.532	-2.699	5.042	-1.486	82.967	13.138	11.451
2118	134	20	18	C	W	46.102	-0.414	-1.327	4.926	0.417	1.208	-4.484	76.538	10.653	14.421
2119	134	20	18	C	W	49.107	0.418	4.141	4.020	-0.415	-3.833	-3.721	76.566	14.426	10.644
2120	134	20	18	C	W	49.842	2.281	5.378	2.095	-2.182	-5.162	-2.010	83.141	11.446	13.139
2121	134	20	18	C	W	70.158	2.281	-5.378	2.095	-2.182	5.162	-2.011	83.141	13.139	11.446
2122	134	20	18	C	W	70.893	0.418	-4.141	4.020	-0.415	3.833	-3.721	76.566	14.426	10.644
2123	134	20	18	C	W	73.898	-0.414	1.327	4.926	0.417	-1.208	-4.484	83.031	11.445	13.151
2124	134	20	18	C	W	76.102	2.853	5.196	1.532	-2.699	-5.041	-1.486	82.967	11.451	13.138
2125	134	20	18	C	W	103.898	2.853	-5.196	1.532	-2.699	5.042	-1.486	76.436	10.655	14.416
2126	134	20	18	C	W	106.102	-0.414	-1.327	4.926	0.417	1.208	-4.484	76.538	10.653	14.421
2127	134	20	18	C	W	109.107	0.418	4.141	4.020	-0.415	-3.833	-3.721	76.566	14.426	10.644
2128	134	20	18	C	W	109.842	2.281	5.378	2.095	-2.182	-5.162	-2.010	83.141	11.446	13.139
2129	134	20	18	P	S	43.898	2.853	-5.196	1.532	-2.699	5.042	-1.486	76.436	10.655	14.416
2130	134	20	18	P	S	46.102	-0.414	-1.327	4.926	0.417	1.208	-4.484	76.538	10.653	14.421
2131	134	20	18	P	S	49.107	0.418	4.141	4.020	-0.415	-3.833	-3.721	76.566	14.426	10.644
2132	134	20	18	P	S	70.893	0.418	-4.141	4.020	-0.415	3.833	-3.721	76.566	14.426	10.644
2133	134	20	18	P	S	73.898	-0.414	1.327	4.926	0.417	-1.208	-4.484	83.031	11.445	13.151
2134	134	20	18	P	S	76.102	2.853	5.196	1.532	-2.699	-5.041	-1.486	76.436	10.655	14.416
2135	134	20	18	P	W	10.893	4.020	-4.004	0.418	-3.721	3.971	-0.415	76.543	10.637	14.437
2136	134	20	18	P	W	13.898	-0.414	1.327	4.926	0.417	-1.208	-4.484	76.538	14.421	10.653
2137	134	20	18	P	W	16.102	2.853	5.196	1.532	-2.699	-5.041	-1.486	76.436	10.655	14.416
2138	134	20	18	P	W	76.102	2.853	5.196	1.532	-2.699	-5.041	-1.486	82.967	11.451	13.138
2139	134	20	18	P	W	103.898	2.853	-5.196	1.532	-2.699	5.042	-1.486	76.436	10.655	14.416
2140	134	20	18	P	W	106.102	-0.414	-1.327	4.926	0.417	1.208	-4.484	83.031	11.445	13.151
2141	134	20	18	P	W	109.107	0.418	4.141	4.020	-0.415	-3.833	-3.721	76.566	14.426	10.644
2142	135	21	17	C	S	16.337	0.353	-2.492	-1.348	-0.351	2.561	1.386	86.644	17.539	8.489

BL number	Atoms	γ-PC unit cells	WS_2 unit cells	γ-PC origin atom	WS_2 origin atom	Twist-angle (°)	γ-PC strain 1 (%)	γ-PC strain 2 (%)	γ-PC strain 3 (%)	WS_2 strain 1 (%)	WS_2 strain 2 (%)	WS_2 strain 3 (%)	γ (°)	$\lvert\mathbf{a}\rvert$(Å)	$\lvert\mathbf{b}\rvert$(Å)
2143	135	21	17	C	S	17.696	1.029	-0.150	-1.992	-1.008	0.156	2.075	86.661	8.491	17.537
2144	135	21	17	C	S	19.107	-1.485	2.322	0.495	1.530	-2.299	-0.490	86.695	8.492	17.533
2145	135	21	17	C	S	23.691	2.390	2.166	-3.239	-2.281	-2.315	3.464	88.565	17.539	8.483
2146	135	21	17	C	S	36.309	2.390	-2.165	-3.239	-2.281	2.315	3.464	88.565	8.483	17.539
2147	135	21	17	C	S	42.304	1.029	0.150	-1.992	-1.008	-0.156	2.075	86.661	17.537	8.491
2148	135	21	17	C	S	43.663	0.353	2.492	-1.348	-0.351	-2.561	1.386	86.644	17.539	8.489
2149	135	21	17	C	S	76.337	0.353	-2.492	-1.348	-0.351	2.561	1.386	86.644	17.539	8.489
2150	135	21	17	C	S	77.696	1.029	-0.150	-1.992	-1.008	0.156	2.075	86.661	8.491	17.537
2151	135	21	17	C	S	83.691	2.390	2.166	-3.239	-2.281	-2.315	3.464	88.565	17.539	8.483
2152	135	21	17	C	S	96.309	2.390	-2.165	-3.239	-2.281	2.315	3.464	88.565	8.483	17.539
2153	135	21	17	C	S	100.893	-1.485	-2.322	0.495	1.530	2.299	-0.490	86.695	8.492	17.533
2154	135	21	17	C	S	102.304	1.029	0.150	-1.992	-1.008	-0.156	2.075	86.661	17.537	8.491
2155	135	21	17	C	S	103.663	0.353	2.492	-1.348	-0.351	-2.561	1.386	86.644	17.539	8.489
2156	135	21	17	C	W	16.337	0.353	-2.492	-1.348	-0.351	2.561	1.386	86.644	17.539	8.489
2157	135	21	17	C	W	17.696	1.029	-0.150	-1.992	-1.008	0.156	2.075	86.661	8.491	17.537
2158	135	21	17	C	W	23.691	2.390	2.166	-3.239	-2.281	-2.315	3.464	88.565	17.539	8.483
2159	135	21	17	C	W	36.309	2.390	-2.165	-3.239	-2.281	2.315	3.464	88.565	8.483	17.539
2160	135	21	17	C	W	40.893	-1.485	-2.322	0.495	1.530	2.299	-0.490	86.695	8.492	17.533
2161	135	21	17	C	W	42.304	1.029	0.150	-1.992	-1.008	-0.156	2.075	86.661	17.537	8.491
2162	135	21	17	C	W	43.663	0.353	2.492	-1.348	-0.351	-2.561	1.386	86.644	17.539	8.489
2163	135	21	17	C	W	76.337	0.353	-2.492	-1.348	-0.351	2.561	1.386	86.644	17.539	8.489
2164	135	21	17	C	W	77.696	1.029	-0.150	-1.992	-1.008	0.156	2.075	86.661	8.491	17.537
2165	135	21	17	C	W	79.107	-1.485	2.322	0.495	1.530	-2.299	-0.490	86.695	8.492	17.533
2166	135	21	17	C	W	83.691	2.390	2.166	-3.239	-2.281	-2.315	3.464	88.565	17.539	8.483
2167	135	21	17	C	W	96.309	2.390	-2.165	-3.239	-2.281	2.315	3.464	88.565	8.483	17.539
2168	135	21	17	C	W	102.304	1.029	0.150	-1.992	-1.008	-0.156	2.075	86.661	17.537	8.491
2169	135	21	17	C	W	103.663	0.353	2.492	-1.348	-0.351	-2.561	1.386	86.644	17.539	8.489
2170	135	21	17	P	S	16.337	0.353	-2.492	-1.348	-0.351	2.561	1.386	86.644	17.539	8.489
2171	135	21	17	P	S	17.696	1.029	-0.150	-1.992	-1.008	0.156	2.075	86.661	8.491	17.537
2172	135	21	17	P	S	23.691	2.390	2.166	-3.239	-2.281	-2.315	3.464	88.565	17.539	8.483
2173	135	21	17	P	S	36.309	2.390	-2.165	-3.239	-2.281	2.315	3.464	88.565	8.483	17.539
2174	135	21	17	P	S	40.893	-1.485	-2.322	0.495	1.530	2.299	-0.490	86.695	8.492	17.533
2175	135	21	17	P	S	42.304	1.029	0.150	-1.992	-1.008	-0.156	2.075	86.661	17.537	8.491
2176	135	21	17	P	S	43.663	0.353	2.492	-1.348	-0.351	-2.561	1.386	86.644	17.539	8.489
2177	135	21	17	P	S	76.337	0.353	-2.492	-1.348	-0.351	2.561	1.386	86.644	17.539	8.489
2178	135	21	17	P	S	77.696	1.029	-0.150	-1.992	-1.008	0.156	2.075	86.661	8.491	17.537
2179	135	21	17	P	S	79.107	-1.485	2.322	0.495	1.530	-2.299	-0.490	86.695	8.492	17.533
2180	135	21	17	P	S	83.691	2.390	2.166	-3.239	-2.281	-2.315	3.464	88.565	17.539	8.483
2181	135	21	17	P	S	96.309	2.390	-2.165	-3.239	-2.281	2.315	3.464	88.565	8.483	17.539
2182	135	21	17	P	S	102.304	1.029	0.150	-1.992	-1.008	-0.156	2.075	86.661	17.537	8.491
2183	135	21	17	P	S	103.663	0.353	2.492	-1.348	-0.351	-2.561	1.386	86.644	17.539	8.489
2184	135	21	17	P	W	16.337	0.353	-2.492	-1.348	-0.351	2.561	1.386	86.644	17.539	8.489
2185	135	21	17	P	W	17.696	1.029	-0.150	-1.992	-1.008	0.156	2.075	86.661	8.491	17.537
2186	135	21	17	P	W	19.107	-1.485	2.322	0.495	1.530	-2.299	-0.490	86.695	8.492	17.533
2187	135	21	17	P	W	23.691	2.390	2.166	-3.239	-2.281	-2.315	3.464	88.565	17.539	8.483
2188	135	21	17	P	W	36.309	2.390	-2.165	-3.239	-2.281	2.315	3.464	88.565	8.483	17.539
2189	135	21	17	P	W	42.304	1.029	0.150	-1.992	-1.008	-0.156	2.075	86.661	17.537	8.491
2190	135	21	17	P	W	43.663	0.353	2.492	-1.348	-0.351	-2.561	1.386	86.644	17.539	8.489
2191	135	21	17	P	W	76.337	0.353	-2.492	-1.348	-0.351	2.561	1.386	86.644	17.539	8.489
2192	135	21	17	P	W	77.696	1.029	-0.150	-1.992	-1.008	0.156	2.075	86.661	8.491	17.537
2193	135	21	17	P	W	83.691	2.390	2.166	-3.239	-2.281	-2.315	3.464	88.565	17.539	8.483

BL number	Atoms	γ-PC unit cells	WS_2 unit cells	γ-PC origin atom	WS_2 origin atom	Twist-angle (°)	γ-PC strain 1 (%)	γ-PC strain 2 (%)	γ-PC strain 3 (%)	WS_2 strain 1 (%)	WS_2 strain 2 (%)	WS_2 strain 3 (%)	γ (°)	|**a**|(Å)	|**b**|(Å)
2194	135	21	17	P	W	96.309	2.390	-2.165	-3.239	-2.281	2.315	3.464	88.565	8.483	17.539
2195	135	21	17	P	W	100.893	-1.485	-2.322	0.495	1.530	2.299	-0.490	86.695	8.492	17.533
2196	135	21	17	P	W	102.304	1.029	0.150	-1.992	-1.008	-0.156	2.075	86.661	17.537	8.491
2197	135	21	17	P	W	103.663	0.353	2.492	-1.348	-0.351	-2.561	1.386	86.644	17.539	8.489
2198	136	22	16	C	S	8.213	-2.359	-2.562	-3.803	2.476	2.773	4.116	73.209	12.906	11.956
2199	136	22	16	C	S	9.515	-4.497	-0.386	-1.633	4.941	0.399	1.688	73.205	11.961	12.904
2200	136	22	16	C	S	10.893	-1.983	1.929	-4.165	2.064	-2.105	4.544	73.198	12.906	11.959
2201	136	22	16	C	S	49.107	-1.983	-1.929	-4.165	2.064	2.105	4.544	73.198	12.906	11.959
2202	136	22	16	C	S	69.515	-4.497	-0.386	-1.633	4.941	0.399	1.688	73.205	11.961	12.904
2203	136	22	16	C	S	109.107	-1.983	-1.929	-4.165	2.064	2.105	4.544	73.198	12.906	11.959
2204	136	22	16	C	S	110.485	-4.497	0.386	-1.633	4.941	-0.399	1.688	73.205	11.961	12.904
2205	136	22	16	C	S	111.787	-2.359	2.562	-3.803	2.476	-2.773	4.116	73.209	11.956	12.906
2206	136	22	16	C	W	9.515	-4.497	-0.386	-1.633	4.941	0.399	1.688	73.205	11.961	12.904
2207	136	22	16	C	W	49.107	-1.983	-1.929	-4.165	2.064	2.105	4.544	73.198	12.906	11.959
2208	136	22	16	C	W	50.485	-4.497	0.386	-1.633	4.941	-0.399	1.688	73.205	11.961	12.904
2209	136	22	16	C	W	51.787	-2.359	2.562	-3.803	2.476	-2.773	4.116	73.209	11.956	12.906
2210	136	22	16	C	W	68.213	-2.359	-2.562	-3.803	2.476	2.773	4.116	73.209	12.906	11.956
2211	136	22	16	C	W	69.515	-4.497	-0.386	-1.633	4.941	0.399	1.688	73.205	11.961	12.904
2212	136	22	16	C	W	70.893	-1.983	1.929	-4.165	2.064	-2.105	4.544	73.198	12.906	11.959
2213	136	22	16	C	W	109.107	-1.983	-1.929	-4.165	2.064	2.105	4.544	73.198	12.906	11.959
2214	136	22	16	P	S	9.515	-4.497	-0.386	-1.633	4.941	0.399	1.688	73.205	11.961	12.904
2215	136	22	16	P	S	49.107	-1.983	-1.929	-4.165	2.064	2.105	4.544	73.198	12.906	11.959
2216	136	22	16	P	S	50.485	-4.497	0.386	-1.633	4.941	-0.399	1.688	73.205	11.961	12.904
2217	136	22	16	P	S	51.787	-2.359	2.562	-3.803	2.476	-2.773	4.116	73.209	11.956	12.906
2218	136	22	16	P	S	68.213	-2.359	-2.562	-3.803	2.476	2.773	4.116	73.209	12.906	11.956
2219	136	22	16	P	S	69.515	-4.497	-0.386	-1.633	4.941	0.399	1.688	73.205	11.961	12.904
2220	136	22	16	P	S	70.893	-1.983	1.929	-4.165	2.064	-2.105	4.544	73.198	12.906	11.959
2221	136	22	16	P	S	109.107	-1.983	-1.929	-4.165	2.064	2.105	4.544	73.198	12.906	11.959
2222	136	22	16	P	W	8.213	-2.359	-2.562	-3.803	2.476	2.773	4.116	73.209	12.906	11.956
2223	136	22	16	P	W	9.515	-4.497	-0.386	-1.633	4.941	0.399	1.688	73.205	11.961	12.904
2224	136	22	16	P	W	10.893	-1.983	1.929	-4.165	2.064	-2.105	4.544	73.198	12.906	11.959
2225	136	22	16	P	W	49.107	-1.983	-1.929	-4.165	2.064	2.105	4.544	73.198	12.906	11.959
2226	136	22	16	P	W	69.515	-4.497	-0.386	-1.633	4.941	0.399	1.688	73.205	11.961	12.904
2227	136	22	16	P	W	109.107	-1.983	-1.929	-4.165	2.064	2.105	4.544	73.198	12.906	11.959
2228	136	22	16	P	W	110.485	-4.497	0.386	-1.633	4.941	-0.399	1.688	73.205	11.961	12.904
2229	136	22	16	P	W	111.787	-2.359	2.562	-3.803	2.476	-2.773	4.116	73.209	11.956	12.906
2230	137	20	19	C	S	21.787	5.011	-1.516	2.260	-4.554	1.450	-2.163	85.762	7.983	19.287
2231	137	20	19	C	S	23.691	2.390	1.933	4.875	-2.281	-1.761	-4.442	85.723	19.291	7.981
2232	137	20	19	C	S	36.309	2.390	-1.933	4.875	-2.281	1.761	-4.442	85.723	19.291	7.981
2233	137	20	19	C	S	38.213	5.011	1.516	2.260	-4.554	-1.450	-2.163	85.762	7.983	19.287
2234	137	20	19	C	S	81.787	5.011	-1.516	2.260	-4.554	1.450	-2.163	85.762	7.983	19.287
2235	137	20	19	C	S	83.691	2.390	1.933	4.875	-2.281	-1.761	-4.442	85.723	19.291	7.981
2236	137	20	19	C	S	96.309	2.390	-1.933	4.875	-2.281	1.761	-4.442	85.723	19.291	7.981
2237	137	20	19	C	S	98.213	5.011	1.516	2.260	-4.554	-1.450	-2.163	85.762	7.983	19.287
2238	137	20	19	C	W	21.787	5.011	-1.516	2.260	-4.554	1.450	-2.163	85.762	7.983	19.287
2239	137	20	19	C	W	23.691	2.390	1.933	4.875	-2.281	-1.761	-4.442	85.723	19.291	7.981
2240	137	20	19	C	W	36.309	2.390	-1.933	4.875	-2.281	1.761	-4.442	85.723	19.291	7.981
2241	137	20	19	C	W	38.213	5.011	1.516	2.260	-4.554	-1.450	-2.163	85.762	7.983	19.287
2242	137	20	19	C	W	81.787	5.011	-1.516	2.260	-4.554	1.450	-2.163	85.762	7.983	19.287
2243	137	20	19	C	W	83.691	2.390	1.933	4.875	-2.281	-1.761	-4.442	85.723	19.291	7.981
2244	137	20	19	C	W	96.309	2.390	-1.933	4.875	-2.281	1.761	-4.442	85.723	19.291	7.981

BL number	Atoms	γ-PC unit cells	WS_2 unit cells	γ-PC origin atom	WS_2 origin atom	Twist-angle (°)	γ-PC strain 1 (%)	γ-PC strain 2 (%)	γ-PC strain 3 (%)	WS_2 strain 1 (%)	WS_2 strain 2 (%)	WS_2 strain 3 (%)	γ (°)	$\lvert\mathbf{a}\rvert$(Å)	$\lvert\mathbf{b}\rvert$(Å)
2245	137	20	19	C	W	98.213	5.011	1.516	2.260	-4.554	-1.450	-2.163	85.762	7.983	19.287
2246	137	20	19	P	S	21.787	5.011	-1.516	2.260	-4.554	1.450	-2.163	85.762	7.983	19.287
2247	137	20	19	P	S	23.691	2.390	1.933	4.875	-2.281	-1.761	-4.442	85.723	19.291	7.981
2248	137	20	19	P	S	36.309	2.390	-1.933	4.875	-2.281	1.761	-4.442	85.723	19.291	7.981
2249	137	20	19	P	S	38.213	5.011	1.516	2.260	-4.554	-1.450	-2.163	85.762	7.983	19.287
2250	137	20	19	P	S	81.787	5.011	-1.516	2.260	-4.554	1.450	-2.163	85.762	7.983	19.287
2251	137	20	19	P	S	83.691	2.390	1.933	4.875	-2.281	-1.761	-4.442	85.723	19.291	7.981
2252	137	20	19	P	S	96.309	2.390	-1.933	4.875	-2.281	1.761	-4.442	85.723	19.291	7.981
2253	137	20	19	P	S	98.213	5.011	1.516	2.260	-4.554	-1.450	-2.163	85.762	7.983	19.287
2254	137	20	19	P	W	21.787	5.011	-1.516	2.260	-4.554	1.450	-2.163	85.762	7.983	19.287
2255	137	20	19	P	W	23.691	2.390	1.933	4.875	-2.281	-1.761	-4.442	85.723	19.291	7.981
2256	137	20	19	P	W	36.309	2.390	-1.933	4.875	-2.281	1.761	-4.442	85.723	19.291	7.981
2257	137	20	19	P	W	38.213	5.011	1.516	2.260	-4.554	-1.450	-2.163	85.762	7.983	19.287
2258	137	20	19	P	W	81.787	5.011	-1.516	2.260	-4.554	1.450	-2.163	85.762	7.983	19.287
2259	137	20	19	P	W	83.691	2.390	1.933	4.875	-2.281	-1.761	-4.442	85.723	19.291	7.981
2260	137	20	19	P	W	96.309	2.390	-1.933	4.875	-2.281	1.761	-4.442	85.723	19.291	7.981
2261	137	20	19	P	W	98.213	5.011	1.516	2.260	-4.554	-1.450	-2.163	85.762	7.983	19.287
2262	138	21	18	C	S	16.102	2.853	-3.535	-0.922	-2.699	3.601	0.940	77.528	18.455	8.490
2263	138	21	18	C	S	18.613	3.582	0.808	-1.590	-3.342	-0.835	1.642	77.647	8.492	18.448
2264	138	21	18	C	S	19.107	-1.485	1.741	3.466	1.530	-1.628	-3.241	77.660	14.708	10.651
2265	138	21	18	C	S	25.285	4.262	4.539	-2.197	-3.928	-4.747	2.298	82.349	18.222	8.477
2266	138	21	18	C	S	34.715	4.262	-4.539	-2.197	-3.928	4.747	2.298	82.349	18.222	8.477
2267	138	21	18	C	S	38.948	1.339	-5.108	0.524	-1.304	5.055	-0.519	77.673	10.642	14.711
2268	138	21	18	C	S	40.893	-1.485	-1.741	3.466	1.530	1.628	-3.241	77.660	14.708	10.651
2269	138	21	18	C	S	41.387	3.582	-0.808	-1.590	-3.342	0.835	1.642	77.647	18.448	8.492
2270	138	21	18	C	S	43.898	2.853	3.535	-0.922	-2.699	-3.601	0.940	77.528	18.455	8.490
2271	138	21	18	C	S	76.102	2.853	-3.535	-0.922	-2.699	3.601	0.940	77.528	18.455	8.490
2272	138	21	18	C	S	78.613	3.582	0.808	-1.590	-3.342	-0.835	1.642	77.647	8.492	18.448
2273	138	21	18	C	S	79.107	-1.485	1.741	3.466	1.530	-1.628	-3.241	77.660	8.492	18.448
2274	138	21	18	C	S	81.052	1.339	5.108	0.524	-1.304	-5.055	-0.519	77.673	14.711	10.642
2275	138	21	18	C	S	85.285	4.262	4.539	-2.197	-3.928	-4.747	2.298	82.349	18.222	8.477
2276	138	21	18	C	S	94.715	4.262	-4.539	-2.197	-3.928	4.747	2.298	82.349	18.222	8.477
2277	138	21	18	C	S	100.893	-1.485	-1.741	3.466	1.530	1.628	-3.241	77.660	8.492	18.448
2278	138	21	18	C	S	101.387	3.582	-0.808	-1.590	-3.342	0.835	1.642	77.647	18.448	8.492
2279	138	21	18	C	S	103.898	2.853	3.535	-0.922	-2.699	-3.601	0.940	77.528	18.455	8.490
2280	138	21	18	C	W	16.102	2.853	-3.535	-0.922	-2.699	3.601	0.940	77.528	10.655	14.706
2281	138	21	18	C	W	18.613	3.582	0.808	-1.590	-3.342	-0.835	1.642	77.647	8.492	18.448
2282	138	21	18	C	W	19.107	-1.485	1.741	3.466	1.530	-1.628	-3.241	77.660	8.492	18.448
2283	138	21	18	C	W	21.052	1.339	5.108	0.524	-1.304	-5.055	-0.519	77.673	14.711	10.642
2284	138	21	18	C	W	25.285	4.262	4.539	-2.197	-3.928	-4.747	2.298	82.349	18.222	8.477
2285	138	21	18	C	W	34.715	4.262	-4.539	-2.197	-3.928	4.747	2.298	82.349	18.222	8.477
2286	138	21	18	C	W	40.893	-1.485	-1.741	3.466	1.530	1.628	-3.241	77.660	8.492	18.448
2287	138	21	18	C	W	41.387	3.582	-0.808	-1.590	-3.342	0.835	1.642	77.647	18.448	8.492
2288	138	21	18	C	W	43.898	2.853	3.535	-0.922	-2.699	-3.601	0.940	77.528	18.455	8.490
2289	138	21	18	C	W	76.102	2.853	-3.535	-0.922	-2.699	3.601	0.940	77.528	18.455	8.490
2290	138	21	18	C	W	78.613	3.582	0.808	-1.590	-3.342	-0.835	1.642	77.647	8.492	18.448
2291	138	21	18	C	W	79.107	-1.485	1.741	3.466	1.530	-1.628	-3.241	77.660	8.492	18.448
2292	138	21	18	C	W	85.285	4.262	4.539	-2.197	-3.928	-4.747	2.298	82.349	18.222	8.477
2293	138	21	18	C	W	94.715	4.262	-4.539	-2.197	-3.928	4.747	2.298	82.349	18.222	8.477
2294	138	21	18	C	W	98.948	1.339	-5.108	0.524	-1.304	5.055	-0.519	77.673	10.642	14.710
2295	138	21	18	C	W	100.893	-1.485	-1.741	3.466	1.530	1.628	-3.241	77.660	8.492	18.448

BL number	Atoms	γ-PC unit cells	WS_2 unit cells	γ-PC origin atom	WS_2 origin atom	Twist-angle (°)	γ-PC strain 1 (%)	γ-PC strain 2 (%)	γ-PC strain 3 (%)	WS_2 strain 1 (%)	WS_2 strain 2 (%)	WS_2 strain 3 (%)	γ (°)	|**a**|(Å)	|**b**|(Å)
2296	138	21	18	C	W	101.387	3.582	-0.808	-1.590	-3.342	0.835	1.642	77.647	18.448	8.492
2297	138	21	18	C	W	103.898	2.853	3.535	-0.922	-2.699	-3.601	0.940	77.528	10.655	14.706
2298	138	21	18	P	S	19.107	-1.485	1.741	3.466	1.530	-1.628	-3.241	77.660	14.708	10.651
2299	138	21	18	P	S	25.285	4.262	4.539	-2.197	-3.928	-4.747	2.298	82.349	18.222	8.477
2300	138	21	18	P	S	34.715	4.262	-4.539	-2.197	-3.928	4.747	2.298	82.349	18.222	8.477
2301	138	21	18	P	S	38.948	1.339	-5.108	0.524	-1.304	5.055	-0.519	77.673	10.642	14.711
2302	138	21	18	P	S	40.893	-1.485	-1.741	3.466	1.530	1.628	-3.241	77.660	14.708	10.651
2303	138	21	18	P	S	43.898	2.853	3.535	-0.922	-2.699	-3.601	0.940	77.528	10.655	14.706
2304	138	21	18	P	S	76.102	2.853	-3.535	-0.922	-2.699	3.601	0.940	77.528	10.655	14.706
2305	138	21	18	P	S	79.107	-1.485	1.741	3.466	1.530	-1.628	-3.241	77.660	14.708	10.651
2306	138	21	18	P	S	81.052	1.339	5.108	0.524	-1.304	-5.055	-0.519	77.673	14.711	10.642
2307	138	21	18	P	S	85.285	4.262	4.539	-2.197	-3.928	-4.747	2.298	82.349	18.222	8.477
2308	138	21	18	P	S	94.715	4.262	-4.539	-2.197	-3.928	4.747	2.298	82.349	18.222	8.477
2309	138	21	18	P	S	100.893	-1.485	-1.741	3.466	1.530	1.628	-3.241	77.660	14.708	10.651
2310	138	21	18	P	W	16.102	2.853	-3.535	-0.922	-2.699	3.601	0.940	77.528	10.655	14.706
2311	138	21	18	P	W	21.052	1.339	5.108	0.524	-1.304	-5.055	-0.519	77.673	14.711	10.642
2312	138	21	18	P	W	25.285	4.262	4.539	-2.197	-3.928	-4.747	2.298	82.349	18.222	8.477
2313	138	21	18	P	W	34.715	4.262	-4.539	-2.197	-3.928	4.747	2.298	82.349	18.222	8.477
2314	138	21	18	P	W	85.285	4.262	4.539	-2.197	-3.928	-4.747	2.298	82.349	18.222	8.477
2315	138	21	18	P	W	94.715	4.262	-4.539	-2.197	-3.928	4.747	2.298	82.349	18.222	8.477
2316	138	21	18	P	W	98.948	1.339	-5.108	0.524	-1.304	5.055	-0.519	77.673	10.642	14.710
2317	138	21	18	P	W	103.898	2.853	3.535	-0.922	-2.699	-3.601	0.940	77.528	10.655	14.706
2318	139	22	17	C	S	22.689	-4.497	-5.109	1.390	4.941	4.971	-1.353	70.862	11.961	13.478
2319	139	22	17	C	S	37.311	-4.497	5.109	1.390	4.941	-4.971	-1.353	70.862	11.961	13.478
2320	139	22	17	C	S	82.689	-4.497	-5.109	1.390	4.941	4.971	-1.353	70.862	11.961	13.478
2321	139	22	17	C	S	97.311	-4.497	5.109	1.390	4.941	-4.971	-1.353	70.862	11.961	13.478
2322	139	22	17	C	W	22.689	-4.497	-5.109	1.390	4.941	4.971	-1.353	70.862	11.961	13.478
2323	139	22	17	C	W	37.311	-4.497	5.109	1.390	4.941	-4.971	-1.353	70.862	11.961	13.478
2324	139	22	17	C	W	82.689	-4.497	-5.109	1.390	4.941	4.971	-1.353	70.862	11.961	13.478
2325	139	22	17	C	W	97.311	-4.497	5.109	1.390	4.941	-4.971	-1.353	70.862	11.961	13.478
2326	139	22	17	P	S	22.689	-4.497	-5.109	1.390	4.941	4.971	-1.353	70.862	11.961	13.478
2327	139	22	17	P	S	37.311	-4.497	5.109	1.390	4.941	-4.971	-1.353	70.862	11.961	13.478
2328	139	22	17	P	S	82.689	-4.497	-5.109	1.390	4.941	4.971	-1.353	70.862	11.961	13.478
2329	139	22	17	P	S	97.311	-4.497	5.109	1.390	4.941	-4.971	-1.353	70.862	11.961	13.478
2330	139	22	17	P	W	22.689	-4.497	-5.109	1.390	4.941	4.971	-1.353	70.862	11.961	13.478
2331	139	22	17	P	W	37.311	-4.497	5.109	1.390	4.941	-4.971	-1.353	70.862	11.961	13.478
2332	139	22	17	P	W	82.689	-4.497	-5.109	1.390	4.941	4.971	-1.353	70.862	11.961	13.478
2333	139	22	17	P	W	97.311	-4.497	5.109	1.390	4.941	-4.971	-1.353	70.862	11.961	13.478
2334	141	21	19	C	S	12.520	2.326	0.000	2.326	-2.223	0.000	-2.223	60.000	13.473	13.473
2335	141	21	19	C	S	34.307	2.326	0.000	2.326	-2.223	0.000	-2.223	60.000	13.473	13.473
2336	141	21	19	C	S	47.480	2.326	0.000	2.326	-2.223	0.000	-2.223	60.000	13.473	13.473
2337	141	21	19	C	S	72.520	2.326	0.000	2.326	-2.223	0.000	-2.223	60.000	13.473	13.473
2338	141	21	19	C	S	94.307	2.326	0.000	2.326	-2.223	0.000	-2.223	60.000	13.473	13.473
2339	141	21	19	C	S	107.480	2.326	0.000	2.326	-2.223	0.000	-2.223	60.000	13.473	13.473
2340	141	21	19	P	S	12.520	2.326	0.000	2.326	-2.223	0.000	-2.223	60.000	13.473	13.473
2341	141	21	19	P	S	25.693	2.326	0.000	2.326	-2.223	0.000	-2.223	60.000	13.473	13.473
2342	141	21	19	P	S	34.307	2.326	0.000	2.326	-2.223	0.000	-2.223	60.000	13.473	13.473
2343	141	21	19	P	S	47.480	2.326	0.000	2.326	-2.223	0.000	-2.223	60.000	13.473	13.473
2344	141	21	19	P	S	72.520	2.326	0.000	2.326	-2.223	0.000	-2.223	60.000	13.473	13.473
2345	141	21	19	P	S	85.694	2.326	0.000	2.326	-2.223	0.000	-2.223	60.000	13.473	13.473
2346	141	21	19	P	S	94.307	2.326	0.000	2.326	-2.223	0.000	-2.223	60.000	13.473	13.473

BL number	Atoms	γ-PC unit cells	WS_2 unit cells	γ-PC origin atom	WS_2 origin atom	Twist-angle (°)	γ-PC strain 1 (%)	γ-PC strain 2 (%)	γ-PC strain 3 (%)	WS_2 strain 1 (%)	WS_2 strain 2 (%)	WS_2 strain 3 (%)	γ (°)	$\lvert\mathbf{a}\rvert$(Å)	$\lvert\mathbf{b}\rvert$(Å)
2347	141	21	19	P	S	107.480	2.326	0.000	2.326	-2.223	0.000	-2.223	60.000	13.473	13.473
2348	142	22	18	C	S	4.307	-4.685	4.871	4.639	5.169	-4.457	-4.245	84.610	14.491	10.873
2349	142	22	18	C	S	10.893	4.020	-2.730	-4.165	-3.721	2.978	4.544	88.368	15.815	9.918
2350	142	22	18	C	S	13.898	-4.228	2.513	4.094	4.619	-2.323	-3.784	83.111	14.819	10.658
2351	142	22	18	C	S	25.285	4.262	-0.684	-4.370	-3.928	0.749	4.789	85.488	9.709	16.202
2352	142	22	18	C	S	25.767	0.353	-4.842	-0.828	-0.351	4.924	0.842	80.161	16.358	9.712
2353	142	22	18	C	S	28.055	2.281	-0.863	-2.641	-2.182	0.911	2.789	80.114	16.362	9.716
2354	142	22	18	C	S	30.000	-2.359	-2.562	1.972	2.476	2.465	-1.897	80.024	9.718	16.361
2355	142	22	18	C	S	31.945	2.281	0.863	-2.641	-2.182	-0.911	2.789	80.114	16.362	9.716
2356	142	22	18	C	S	34.233	0.353	4.843	-0.828	-0.351	-4.924	0.842	80.161	9.712	16.358
2357	142	22	18	C	S	34.715	4.262	0.684	-4.370	-3.928	-0.750	4.789	85.488	16.202	9.709
2358	142	22	18	C	S	46.102	-4.228	-2.513	4.094	4.619	2.323	-3.784	88.617	9.921	15.809
2359	142	22	18	C	S	49.107	4.020	2.730	-4.165	-3.721	-2.978	4.544	88.368	15.815	9.918
2360	142	22	18	C	S	55.693	-4.685	-4.871	4.639	5.169	4.457	-4.245	84.609	14.491	10.873
2361	142	22	18	C	S	64.307	-4.685	4.871	4.639	5.169	-4.457	-4.245	84.609	14.491	10.873
2362	142	22	18	C	S	70.893	4.020	-2.730	-4.165	-3.721	2.978	4.544	88.368	15.815	9.918
2363	142	22	18	C	S	73.898	-4.228	2.513	4.094	4.619	-2.323	-3.784	88.617	9.921	15.809
2364	142	22	18	C	S	85.285	4.262	-0.684	-4.370	-3.928	0.749	4.789	85.488	9.709	16.202
2365	142	22	18	C	S	85.767	0.353	-4.842	-0.828	-0.351	4.924	0.842	80.161	16.358	9.712
2366	142	22	18	C	S	88.055	2.281	-0.863	-2.641	-2.182	0.911	2.789	80.114	16.362	9.716
2367	142	22	18	C	S	90.000	-2.359	-2.562	1.972	2.476	2.465	-1.897	80.024	9.718	16.361
2368	142	22	18	C	S	91.945	2.281	0.863	-2.641	-2.182	-0.911	2.789	80.114	16.362	9.716
2369	142	22	18	C	S	94.233	0.353	4.843	-0.828	-0.351	-4.924	0.842	80.161	9.712	16.358
2370	142	22	18	C	S	94.715	4.262	0.684	-4.370	-3.928	-0.750	4.789	85.488	16.202	9.709
2371	142	22	18	C	S	106.102	-4.228	-2.513	4.094	4.619	2.323	-3.784	83.111	10.658	14.819
2372	142	22	18	C	S	109.107	4.020	2.730	-4.165	-3.721	-2.978	4.544	88.368	15.815	9.918
2373	142	22	18	C	S	115.693	-4.685	-4.871	4.639	5.169	4.457	-4.245	84.609	14.491	10.873
2374	142	22	18	C	W	4.307	-4.685	4.871	4.639	5.169	-4.457	-4.245	84.610	14.491	10.873
2375	142	22	18	C	W	10.893	4.020	-2.730	-4.165	-3.721	2.978	4.544	88.368	15.815	9.918
2376	142	22	18	C	W	13.898	-4.228	2.513	4.094	4.619	-2.323	-3.784	83.111	14.819	10.658
2377	142	22	18	C	W	25.285	4.262	-0.684	-4.370	-3.928	0.749	4.789	85.488	9.709	16.202
2378	142	22	18	C	W	25.767	0.353	-4.842	-0.828	-0.351	4.924	0.842	80.161	16.358	9.712
2379	142	22	18	C	W	28.055	2.281	-0.863	-2.641	-2.182	0.911	2.789	80.114	16.362	9.716
2380	142	22	18	C	W	30.000	-2.359	-2.562	1.972	2.476	2.465	-1.897	80.024	9.718	16.361
2381	142	22	18	C	W	31.945	2.281	0.863	-2.641	-2.182	-0.911	2.789	80.114	16.362	9.716
2382	142	22	18	C	W	34.233	0.353	4.843	-0.828	-0.351	-4.924	0.842	80.161	9.712	16.358
2383	142	22	18	C	W	34.715	4.262	0.684	-4.370	-3.928	-0.750	4.789	85.488	16.202	9.709
2384	142	22	18	C	W	46.102	-4.228	-2.513	4.094	4.619	2.323	-3.784	88.617	9.921	15.809
2385	142	22	18	C	W	49.107	4.020	2.730	-4.165	-3.721	-2.978	4.544	88.368	15.815	9.918
2386	142	22	18	C	W	55.693	-4.685	-4.871	4.639	5.169	4.457	-4.245	84.609	14.491	10.873
2387	142	22	18	C	W	64.307	-4.685	4.871	4.639	5.169	-4.457	-4.245	84.609	14.491	10.873
2388	142	22	18	C	W	70.893	4.020	-2.730	-4.165	-3.721	2.978	4.544	88.368	15.815	9.918
2389	142	22	18	C	W	73.898	-4.228	2.513	4.094	4.619	-2.323	-3.784	88.617	9.921	15.809
2390	142	22	18	C	W	85.285	4.262	-0.684	-4.370	-3.928	0.749	4.789	85.488	9.709	16.202
2391	142	22	18	C	W	85.767	0.353	-4.842	-0.828	-0.351	4.924	0.842	80.161	16.358	9.712
2392	142	22	18	C	W	88.055	2.281	-0.863	-2.641	-2.182	0.911	2.789	80.114	16.362	9.716
2393	142	22	18	C	W	90.000	-2.359	-2.562	1.972	2.476	2.465	-1.897	80.024	9.718	16.361
2394	142	22	18	C	W	91.945	2.281	0.863	-2.641	-2.182	-0.911	2.789	80.114	16.362	9.716
2395	142	22	18	C	W	94.233	0.353	4.843	-0.828	-0.351	-4.924	0.842	80.161	9.712	16.358
2396	142	22	18	C	W	94.715	4.262	0.684	-4.370	-3.928	-0.750	4.789	85.488	16.202	9.709
2397	142	22	18	C	W	106.102	-4.228	-2.513	4.094	4.619	2.323	-3.784	83.111	10.658	14.819

BL number	Atoms	γ-PC unit cells	WS_2 unit cells	γ-PC origin atom	WS_2 origin atom	Twist-angle (°)	γ-PC strain 1 (%)	γ-PC strain 2 (%)	γ-PC strain 3 (%)	WS_2 strain 1 (%)	WS_2 strain 2 (%)	WS_2 strain 3 (%)	γ (°)	|**a**|(Å)	|**b**|(Å)
2398	142	22	18	C	W	109.107	4.020	2.730	-4.165	-3.721	-2.978	4.544	88.368	15.815	9.918
2399	142	22	18	C	W	115.693	-4.685	-4.871	4.639	5.169	4.457	-4.245	84.609	14.491	10.873
2400	142	22	18	P	S	4.307	-4.685	4.871	4.639	5.169	-4.457	-4.245	84.610	14.491	10.873
2401	142	22	18	P	S	10.893	4.020	-2.730	-4.165	-3.721	2.978	4.544	88.368	15.815	9.918
2402	142	22	18	P	S	46.102	-4.228	-2.513	4.094	4.619	2.323	-3.784	88.617	9.921	15.809
2403	142	22	18	P	S	55.693	-4.685	-4.871	4.639	5.169	4.457	-4.245	84.609	14.491	10.873
2404	142	22	18	P	S	64.307	-4.685	4.871	4.639	5.169	-4.457	-4.245	84.609	14.491	10.873
2405	142	22	18	P	S	73.898	-4.228	2.513	4.094	4.619	-2.323	-3.784	88.617	9.921	15.809
2406	142	22	18	P	S	94.233	0.353	4.843	-0.828	-0.351	-4.924	0.842	80.161	9.712	16.358
2407	142	22	18	P	S	115.693	-4.685	-4.871	4.639	5.169	4.457	-4.245	84.609	14.491	10.873
2408	142	22	18	P	W	4.307	-4.685	4.871	4.639	5.169	-4.457	-4.245	84.610	14.491	10.873
2409	142	22	18	P	W	13.898	-4.228	2.513	4.094	4.619	-2.323	-3.784	88.617	9.921	15.809
2410	142	22	18	P	W	34.233	0.353	4.843	-0.828	-0.351	-4.924	0.842	80.161	9.712	16.358
2411	142	22	18	P	W	55.693	-4.685	-4.871	4.639	5.169	4.457	-4.245	84.609	14.491	10.873
2412	142	22	18	P	W	64.307	-4.685	4.871	4.639	5.169	-4.457	-4.245	84.609	14.491	10.873
2413	142	22	18	P	W	115.693	-4.685	-4.871	4.639	5.169	4.457	-4.245	84.609	14.491	10.873
2414	144	21	20	C	S	10.158	2.281	-5.222	5.127	-2.182	4.737	-4.650	73.466	15.819	10.645
2415	144	21	20	C	S	27.796	5.011	-1.443	2.391	-4.554	1.378	-2.282	75.005	10.878	15.362
2416	144	21	20	C	S	28.055	2.281	-1.004	5.127	-2.182	0.911	-4.650	74.961	10.878	15.367
2417	144	21	20	C	S	30.000	2.934	-2.547	4.447	-2.772	2.339	-4.084	74.985	15.368	10.875
2418	144	21	20	C	S	31.945	2.281	1.004	5.127	-2.182	-0.911	-4.650	74.961	15.367	10.878
2419	144	21	20	C	S	32.204	5.011	1.443	2.391	-4.554	-1.378	-2.282	75.005	10.878	15.362
2420	144	21	20	C	S	49.842	2.281	5.222	5.127	-2.182	-4.737	-4.650	73.466	10.645	15.819
2421	144	21	20	C	S	70.158	2.281	-5.222	5.127	-2.182	4.737	-4.650	73.466	15.819	10.645
2422	144	21	20	C	S	87.796	5.011	-1.443	2.391	-4.554	1.378	-2.282	75.005	10.878	15.362
2423	144	21	20	C	S	88.055	2.281	-1.004	5.127	-2.182	0.911	-4.650	74.961	10.878	15.367
2424	144	21	20	C	S	90.000	2.934	-2.547	4.447	-2.772	2.339	-4.084	74.985	15.368	10.875
2425	144	21	20	C	S	91.945	2.281	1.004	5.127	-2.182	-0.911	-4.650	74.961	15.367	10.878
2426	144	21	20	C	S	92.204	5.011	1.443	2.391	-4.554	-1.378	-2.282	75.005	10.878	15.362
2427	144	21	20	C	S	109.842	2.281	5.222	5.127	-2.182	-4.737	-4.650	73.466	10.645	15.819
2428	144	21	20	C	W	10.158	2.281	-5.222	5.127	-2.182	4.737	-4.650	73.466	15.819	10.645
2429	144	21	20	C	W	27.796	5.011	-1.443	2.391	-4.554	1.378	-2.282	75.005	10.878	15.362
2430	144	21	20	C	W	28.055	2.281	-1.004	5.127	-2.182	0.911	-4.650	74.961	10.878	15.367
2431	144	21	20	C	W	30.000	2.934	-2.547	4.447	-2.772	2.339	-4.084	74.985	15.368	10.875
2432	144	21	20	C	W	31.945	2.281	1.004	5.127	-2.182	-0.911	-4.650	74.961	15.367	10.878
2433	144	21	20	C	W	32.204	5.011	1.443	2.391	-4.554	-1.378	-2.282	75.005	10.878	15.362
2434	144	21	20	C	W	49.842	2.281	5.222	5.127	-2.182	-4.737	-4.650	73.466	10.645	15.819
2435	144	21	20	C	W	70.158	2.281	-5.222	5.127	-2.182	4.737	-4.650	73.466	15.819	10.645
2436	144	21	20	C	W	87.796	5.011	-1.443	2.391	-4.554	1.378	-2.282	75.005	10.878	15.362
2437	144	21	20	C	W	88.055	2.281	-1.004	5.127	-2.182	0.911	-4.650	74.961	10.878	15.367
2438	144	21	20	C	W	90.000	2.934	-2.547	4.447	-2.772	2.339	-4.084	74.985	15.368	10.875
2439	144	21	20	C	W	91.945	2.281	1.004	5.127	-2.182	-0.911	-4.650	74.961	15.367	10.878
2440	144	21	20	C	W	92.204	5.011	1.443	2.391	-4.554	-1.378	-2.282	75.005	10.878	15.362
2441	144	21	20	C	W	109.842	2.281	5.222	5.127	-2.182	-4.737	-4.650	73.466	10.645	15.819
2442	144	21	20	P	S	10.158	2.281	-5.222	5.127	-2.182	4.737	-4.650	73.466	15.819	10.645
2443	144	21	20	P	S	27.796	5.011	-1.443	2.391	-4.554	1.378	-2.282	75.005	10.878	15.362
2444	144	21	20	P	S	28.055	2.281	-1.004	5.127	-2.182	0.911	-4.650	74.961	10.878	15.367
2445	144	21	20	P	S	30.000	2.934	-2.547	4.447	-2.772	2.339	-4.084	74.985	15.368	10.875
2446	144	21	20	P	S	31.945	2.281	1.004	5.127	-2.182	-0.911	-4.650	74.961	15.367	10.878
2447	144	21	20	P	S	32.204	5.011	1.443	2.391	-4.554	-1.378	-2.282	75.005	10.878	15.362
2448	144	21	20	P	S	49.842	2.281	5.222	5.127	-2.182	-4.737	-4.650	73.466	10.645	15.819

BL number	Atoms	γ-PC unit cells	WS_2 unit cells	γ-PC origin atom	WS_2 origin atom	Twist-angle (°)	γ-PC strain 1 (%)	γ-PC strain 2 (%)	γ-PC strain 3 (%)	WS_2 strain 1 (%)	WS_2 strain 2 (%)	WS_2 strain 3 (%)	γ (°)	$\lvert\mathbf{a}\rvert$(Å)	$\lvert\mathbf{b}\rvert$(Å)
2449	144	21	20	P	S	70.158	2.281	-5.222	5.127	-2.182	4.737	-4.650	73.466	15.819	10.645
2450	144	21	20	P	S	87.796	5.011	-1.443	2.391	-4.554	1.378	-2.282	75.005	10.878	15.362
2451	144	21	20	P	S	88.055	2.281	-1.004	5.127	-2.182	0.911	-4.650	74.961	10.878	15.367
2452	144	21	20	P	S	90.000	2.934	-2.547	4.447	-2.772	2.339	-4.084	74.985	15.368	10.875
2453	144	21	20	P	S	91.945	2.281	1.004	5.127	-2.182	-0.911	-4.650	74.961	15.367	10.878
2454	144	21	20	P	S	92.204	5.011	1.443	2.391	-4.554	-1.378	-2.282	75.005	10.878	15.362
2455	144	21	20	P	S	109.842	2.281	5.222	5.127	-2.182	-4.737	-4.650	73.466	10.645	15.819
2456	144	21	20	P	W	10.158	2.281	-5.222	5.127	-2.182	4.737	-4.650	73.466	15.819	10.645
2457	144	21	20	P	W	27.796	5.011	-1.443	2.391	-4.554	1.378	-2.282	75.005	10.878	15.362
2458	144	21	20	P	W	28.055	2.281	-1.004	5.127	-2.182	0.911	-4.650	74.961	10.878	15.367
2459	144	21	20	P	W	30.000	2.934	-2.547	4.447	-2.772	2.339	-4.084	74.985	15.368	10.875
2460	144	21	20	P	W	31.945	2.281	1.004	5.127	-2.182	-0.911	-4.650	74.961	15.367	10.878
2461	144	21	20	P	W	32.204	5.011	1.443	2.391	-4.554	-1.378	-2.282	75.005	10.878	15.362
2462	144	21	20	P	W	49.842	2.281	5.222	5.127	-2.182	-4.737	-4.650	73.466	10.645	15.819
2463	144	21	20	P	W	70.158	2.281	-5.222	5.127	-2.182	4.737	-4.650	73.466	15.819	10.645
2464	144	21	20	P	W	87.796	5.011	-1.443	2.391	-4.554	1.378	-2.282	75.005	10.878	15.362
2465	144	21	20	P	W	88.055	2.281	-1.004	5.127	-2.182	0.911	-4.650	74.961	10.878	15.367
2466	144	21	20	P	W	90.000	2.934	-2.547	4.447	-2.772	2.339	-4.084	74.985	15.368	10.875
2467	144	21	20	P	W	91.945	2.281	1.004	5.127	-2.182	-0.911	-4.650	74.961	15.367	10.878
2468	144	21	20	P	W	92.204	5.011	1.443	2.391	-4.554	-1.378	-2.282	75.005	10.878	15.362
2469	144	21	20	P	W	109.842	2.281	5.222	5.127	-2.182	-4.737	-4.650	73.466	10.645	15.819
2470	145	22	19	C	S	27.796	5.011	-1.378	-2.491	-4.554	1.450	2.621	85.959	10.878	14.841
2471	145	22	19	C	S	30.000	-2.359	2.562	4.859	2.476	-2.335	-4.429	85.807	14.845	10.877
2472	145	22	19	C	S	32.204	5.011	1.378	-2.491	-4.554	-1.450	2.621	85.959	10.878	14.841
2473	145	22	19	C	S	87.796	5.011	-1.378	-2.491	-4.554	1.450	2.621	85.959	10.878	14.841
2474	145	22	19	C	S	90.000	-2.359	-2.562	4.859	2.476	2.335	-4.429	85.807	14.845	10.877
2475	145	22	19	C	S	92.204	5.011	1.378	-2.491	-4.554	-1.450	2.621	85.958	10.878	14.841
2476	145	22	19	C	W	27.796	5.011	-1.378	-2.491	-4.554	1.450	2.621	85.959	10.878	14.841
2477	145	22	19	C	W	30.000	-2.359	2.562	4.859	2.476	-2.335	-4.429	85.807	14.845	10.877
2478	145	22	19	C	W	32.204	5.011	1.378	-2.491	-4.554	-1.450	2.621	85.959	10.878	14.841
2479	145	22	19	C	W	87.796	5.011	-1.378	-2.491	-4.554	1.450	2.621	85.959	10.878	14.841
2480	145	22	19	C	W	90.000	-2.359	-2.562	4.859	2.476	2.335	-4.429	85.807	14.845	10.877
2481	145	22	19	C	W	92.204	5.011	1.378	-2.491	-4.554	-1.450	2.621	85.958	10.878	14.841
2482	145	22	19	P	S	27.796	5.011	-1.378	-2.491	-4.554	1.450	2.621	85.959	10.878	14.841
2483	145	22	19	P	S	30.000	-2.359	2.562	4.859	2.476	-2.335	-4.429	85.807	14.845	10.877
2484	145	22	19	P	S	32.204	5.011	1.378	-2.491	-4.554	-1.450	2.621	85.959	10.878	14.841
2485	145	22	19	P	S	87.796	5.011	-1.378	-2.491	-4.554	1.450	2.621	85.959	10.878	14.841
2486	145	22	19	P	S	90.000	-2.359	-2.562	4.859	2.476	2.335	-4.429	85.807	14.845	10.877
2487	145	22	19	P	S	92.204	5.011	1.378	-2.491	-4.554	-1.450	2.621	85.958	10.878	14.841
2488	145	22	19	P	W	27.796	5.011	-1.378	-2.491	-4.554	1.450	2.621	85.959	10.878	14.841
2489	145	22	19	P	W	30.000	-2.359	2.562	4.859	2.476	-2.335	-4.429	85.807	14.845	10.877
2490	145	22	19	P	W	32.204	5.011	1.378	-2.491	-4.554	-1.450	2.621	85.959	10.878	14.841
2491	145	22	19	P	W	87.796	5.011	-1.378	-2.491	-4.554	1.450	2.621	85.959	10.878	14.841
2492	145	22	19	P	W	90.000	-2.359	-2.562	4.859	2.476	2.335	-4.429	85.807	14.845	10.877
2493	145	22	19	P	W	92.204	5.011	1.378	-2.491	-4.554	-1.450	2.621	85.958	10.878	14.841
2494	146	23	18	C	S	9.515	-4.497	-3.412	2.047	4.941	3.277	-1.967	84.068	11.961	13.470
2495	146	23	18	C	S	10.158	2.281	-2.109	-4.700	-2.182	2.328	5.188	81.086	16.362	9.915
2496	146	23	18	C	S	12.520	2.326	1.825	-4.739	-2.223	-2.016	5.235	84.287	13.473	11.956
2497	146	23	18	C	S	13.898	-4.228	4.408	1.742	4.619	-4.259	-1.683	80.828	9.921	16.358
2498	146	23	18	C	S	19.842	-4.723	4.612	2.308	5.216	-4.409	-2.206	80.800	15.242	10.653
2499	146	23	18	C	S	40.158	-4.723	-4.612	2.308	5.216	4.409	-2.206	80.800	15.242	10.653

BL number	Atoms	γ-PC unit cells	WS_2 unit cells	γ-PC origin atom	WS_2 origin atom	Twist-angle (°)	γ-PC strain 1 (%)	γ-PC strain 2 (%)	γ-PC strain 3 (%)	WS_2 strain 1 (%)	WS_2 strain 2 (%)	WS_2 strain 3 (%)	γ (°)	$\lvert\mathbf{a}\rvert$(Å)	$\lvert\mathbf{b}\rvert$(Å)
2500	146	23	18	C	S	46.102	-4.228	-4.407	1.742	4.619	4.259	-1.683	80.828	9.921	16.358
2501	146	23	18	C	S	47.480	2.326	-1.825	-4.739	-2.223	2.016	5.235	84.287	13.473	11.956
2502	146	23	18	C	S	49.842	2.281	2.109	-4.700	-2.182	-2.328	5.188	81.087	16.362	9.915
2503	146	23	18	C	S	50.485	-4.497	3.412	2.047	4.941	-3.277	-1.967	84.068	11.961	13.470
2504	146	23	18	C	S	69.515	-4.497	-3.412	2.047	4.941	3.277	-1.967	84.068	11.961	13.470
2505	146	23	18	C	S	70.158	2.281	-2.109	-4.700	-2.182	2.328	5.188	81.087	16.362	9.915
2506	146	23	18	C	S	72.520	2.326	1.825	-4.739	-2.223	-2.016	5.235	84.287	13.473	11.956
2507	146	23	18	C	S	73.898	-4.228	4.408	1.742	4.619	-4.259	-1.683	80.828	9.921	16.358
2508	146	23	18	C	S	79.842	-4.723	4.612	2.308	5.216	-4.409	-2.206	80.800	15.242	10.653
2509	146	23	18	C	S	100.158	-4.723	-4.612	2.308	5.216	4.409	-2.206	80.800	15.242	10.653
2510	146	23	18	C	S	106.102	-4.228	-4.407	1.742	4.619	4.259	-1.683	80.828	9.921	16.358
2511	146	23	18	C	S	107.480	2.326	-1.825	-4.739	-2.223	2.016	5.235	84.287	13.473	11.956
2512	146	23	18	C	S	109.842	2.281	2.109	-4.700	-2.182	-2.328	5.188	81.087	16.362	9.915
2513	146	23	18	C	S	110.485	-4.497	3.412	2.047	4.941	-3.277	-1.967	84.068	11.961	13.470
2514	146	23	18	C	W	9.515	-4.497	-3.412	2.047	4.941	3.277	-1.967	84.068	11.961	13.470
2515	146	23	18	C	W	10.158	2.281	-2.109	-4.700	-2.182	2.328	5.188	81.086	16.362	9.915
2516	146	23	18	C	W	12.520	2.326	1.825	-4.739	-2.223	-2.016	5.235	84.287	13.473	11.956
2517	146	23	18	C	W	13.898	-4.228	4.408	1.742	4.619	-4.259	-1.683	80.828	9.921	16.358
2518	146	23	18	C	W	19.842	-4.723	4.612	2.308	5.216	-4.409	-2.206	80.800	15.242	10.653
2519	146	23	18	C	W	40.158	-4.723	-4.612	2.308	5.216	4.409	-2.206	80.800	15.242	10.653
2520	146	23	18	C	W	46.102	-4.228	-4.407	1.742	4.619	4.259	-1.683	80.828	9.921	16.358
2521	146	23	18	C	W	47.480	2.326	-1.825	-4.739	-2.223	2.016	5.235	84.287	13.473	11.956
2522	146	23	18	C	W	49.842	2.281	2.109	-4.700	-2.182	-2.328	5.188	81.087	16.362	9.915
2523	146	23	18	C	W	50.485	-4.497	3.412	2.047	4.941	-3.277	-1.967	84.068	11.961	13.470
2524	146	23	18	C	W	69.515	-4.497	-3.412	2.047	4.941	3.277	-1.967	84.068	11.961	13.470
2525	146	23	18	C	W	70.158	2.281	-2.109	-4.700	-2.182	2.328	5.188	81.087	16.362	9.915
2526	146	23	18	C	W	72.520	2.326	1.825	-4.739	-2.223	-2.016	5.235	84.287	13.473	11.956
2527	146	23	18	C	W	73.898	-4.228	4.408	1.742	4.619	-4.259	-1.683	80.828	9.921	16.358
2528	146	23	18	C	W	79.842	-4.723	4.612	2.308	5.216	-4.409	-2.206	80.800	15.242	10.653
2529	146	23	18	C	W	100.158	-4.723	-4.612	2.308	5.216	4.409	-2.206	80.800	15.242	10.653
2530	146	23	18	C	W	106.102	-4.228	-4.407	1.742	4.619	4.259	-1.683	80.828	9.921	16.358
2531	146	23	18	C	W	107.480	2.326	-1.825	-4.739	-2.223	2.016	5.235	84.287	13.473	11.956
2532	146	23	18	C	W	109.842	2.281	2.109	-4.700	-2.182	-2.328	5.188	81.087	16.362	9.915
2533	146	23	18	C	W	110.485	-4.497	3.412	2.047	4.941	-3.277	-1.967	84.068	11.961	13.470
2534	146	23	18	P	S	9.515	-4.497	-3.412	2.047	4.941	3.277	-1.967	84.068	11.961	13.470
2535	146	23	18	P	S	10.158	2.281	-2.109	-4.700	-2.182	2.328	5.188	81.086	16.362	9.915
2536	146	23	18	P	S	12.520	2.326	1.825	-4.739	-2.223	-2.016	5.235	84.287	13.473	11.956
2537	146	23	18	P	S	13.898	-4.228	4.408	1.742	4.619	-4.259	-1.683	80.828	9.921	16.358
2538	146	23	18	P	S	46.102	-4.228	-4.407	1.742	4.619	4.259	-1.683	80.828	9.921	16.358
2539	146	23	18	P	S	47.480	2.326	-1.825	-4.739	-2.223	2.016	5.235	84.287	13.473	11.956
2540	146	23	18	P	S	49.842	2.281	2.109	-4.700	-2.182	-2.328	5.188	81.087	16.362	9.915
2541	146	23	18	P	S	50.485	-4.497	3.412	2.047	4.941	-3.277	-1.967	84.068	11.961	13.470
2542	146	23	18	P	S	69.515	-4.497	-3.412	2.047	4.941	3.277	-1.967	84.068	11.961	13.470
2543	146	23	18	P	S	70.158	2.281	-2.109	-4.700	-2.182	2.328	5.188	81.087	16.362	9.915
2544	146	23	18	P	S	72.520	2.326	1.825	-4.739	-2.223	-2.016	5.235	84.287	13.473	11.956
2545	146	23	18	P	S	73.898	-4.228	4.408	1.742	4.619	-4.259	-1.683	80.828	9.921	16.358
2546	146	23	18	P	S	106.102	-4.228	-4.407	1.742	4.619	4.259	-1.683	80.828	9.921	16.358
2547	146	23	18	P	S	107.480	2.326	-1.825	-4.739	-2.223	2.016	5.235	84.287	13.473	11.956
2548	146	23	18	P	S	109.842	2.281	2.109	-4.700	-2.182	-2.328	5.188	81.087	16.362	9.915
2549	146	23	18	P	S	110.485	-4.497	3.412	2.047	4.941	-3.277	-1.967	84.068	11.961	13.470
2550	146	23	18	P	W	9.515	-4.497	-3.412	2.047	4.941	3.277	-1.967	84.068	11.961	13.470

BL number	Atoms	γ-PC unit cells	WS_2 unit cells	γ-PC origin atom	WS_2 origin atom	Twist-angle (°)	γ-PC strain 1 (%)	γ-PC strain 2 (%)	γ-PC strain 3 (%)	WS_2 strain 1 (%)	WS_2 strain 2 (%)	WS_2 strain 3 (%)	γ (°)	|**a**|(Å)	|**b**|(Å)
2551	146	23	18	P	W	10.158	2.281	-2.109	-4.700	-2.182	2.328	5.188	81.086	16.362	9.915
2552	146	23	18	P	W	12.520	2.326	1.825	-4.739	-2.223	-2.016	5.235	84.287	13.473	11.956
2553	146	23	18	P	W	13.898	-4.228	4.408	1.742	4.619	-4.259	-1.683	80.828	9.921	16.358
2554	146	23	18	P	W	46.102	-4.228	-4.407	1.742	4.619	4.259	-1.683	80.828	9.921	16.358
2555	146	23	18	P	W	47.480	2.326	-1.825	-4.739	-2.223	2.016	5.235	84.287	13.473	11.956
2556	146	23	18	P	W	49.842	2.281	2.109	-4.700	-2.182	-2.328	5.188	81.087	16.362	9.915
2557	146	23	18	P	W	50.485	-4.497	3.412	2.047	4.941	-3.277	-1.967	84.068	11.961	13.470
2558	146	23	18	P	W	69.515	-4.497	-3.412	2.047	4.941	3.277	-1.967	84.068	11.961	13.470
2559	146	23	18	P	W	70.158	2.281	-2.109	-4.700	-2.182	2.328	5.188	81.087	16.362	9.915
2560	146	23	18	P	W	72.520	2.326	1.825	-4.739	-2.223	-2.016	5.235	84.287	13.473	11.956
2561	146	23	18	P	W	73.898	-4.228	4.408	1.742	4.619	-4.259	-1.683	80.828	9.921	16.358
2562	146	23	18	P	W	106.102	-4.228	-4.407	1.742	4.619	4.259	-1.683	80.828	9.921	16.358
2563	146	23	18	P	W	107.480	2.326	-1.825	-4.739	-2.223	2.016	5.235	84.287	13.473	11.956
2564	146	23	18	P	W	109.842	2.281	2.109	-4.700	-2.182	-2.328	5.188	81.087	16.362	9.915
2565	146	23	18	P	W	110.485	-4.497	3.412	2.047	4.941	-3.277	-1.967	84.068	11.961	13.470
2566	147	24	17	C	S	6.587	-3.853	-2.739	-3.549	4.175	2.948	3.821	68.038	14.354	11.952
2567	147	24	17	C	S	9.515	-4.497	2.209	-2.892	4.941	-2.345	3.070	68.045	11.961	14.344
2568	147	24	17	C	S	22.689	-4.497	4.507	-2.892	4.941	-4.783	3.070	66.889	11.961	14.465
2569	147	24	17	C	S	37.311	-4.497	-4.507	-2.892	4.941	4.783	3.070	66.889	11.961	14.465
2570	147	24	17	C	S	42.520	-4.685	4.345	-2.697	5.169	-4.592	2.851	66.895	14.491	11.939
2571	147	24	17	C	S	50.485	-4.497	-2.209	-2.892	4.941	2.345	3.070	68.045	11.961	14.344
2572	147	24	17	C	S	53.413	-3.853	2.739	-3.549	4.175	-2.948	3.821	68.038	14.354	11.952
2573	147	24	17	C	S	66.587	-3.853	-2.739	-3.549	4.175	2.948	3.821	68.038	14.354	11.952
2574	147	24	17	C	S	69.515	-4.497	2.209	-2.892	4.941	-2.345	3.070	68.045	11.961	14.344
2575	147	24	17	C	S	77.480	-4.685	-4.344	-2.697	5.169	4.592	2.851	66.895	14.491	11.939
2576	147	24	17	C	S	82.689	-4.497	4.507	-2.892	4.941	-4.783	3.070	66.889	11.961	14.465
2577	147	24	17	C	S	97.311	-4.497	-4.507	-2.892	4.941	4.783	3.070	66.889	11.961	14.465
2578	147	24	17	C	S	110.485	-4.497	-2.209	-2.892	4.941	2.345	3.070	68.045	11.961	14.344
2579	147	24	17	C	S	113.413	-3.853	2.739	-3.549	4.175	-2.948	3.821	68.038	14.354	11.952
2580	147	24	17	C	W	6.587	-3.853	-2.739	-3.549	4.175	2.948	3.821	68.038	14.354	11.952
2581	147	24	17	C	W	9.515	-4.497	2.209	-2.892	4.941	-2.345	3.070	68.045	11.961	14.344
2582	147	24	17	C	W	17.480	-4.685	-4.344	-2.697	5.169	4.592	2.851	66.895	14.491	11.939
2583	147	24	17	C	W	50.485	-4.497	-2.209	-2.892	4.941	2.345	3.070	68.045	11.961	14.344
2584	147	24	17	C	W	53.413	-3.853	2.739	-3.549	4.175	-2.948	3.821	68.038	14.354	11.952
2585	147	24	17	C	W	66.587	-3.853	-2.739	-3.549	4.175	2.948	3.821	68.038	14.354	11.952
2586	147	24	17	C	W	69.515	-4.497	2.209	-2.892	4.941	-2.345	3.070	68.045	11.961	14.344
2587	147	24	17	C	W	102.520	-4.685	4.345	-2.697	5.169	-4.592	2.851	66.895	14.491	11.939
2588	147	24	17	C	W	110.485	-4.497	-2.209	-2.892	4.941	2.345	3.070	68.045	11.961	14.344
2589	147	24	17	C	W	113.413	-3.853	2.739	-3.549	4.175	-2.948	3.821	68.038	14.354	11.952
2590	147	24	17	P	S	6.587	-3.853	-2.739	-3.549	4.175	2.948	3.821	68.038	14.354	11.952
2591	147	24	17	P	S	9.515	-4.497	2.209	-2.892	4.941	-2.345	3.070	68.045	11.961	14.344
2592	147	24	17	P	S	22.689	-4.497	4.507	-2.892	4.941	-4.783	3.070	66.889	11.961	14.465
2593	147	24	17	P	S	37.311	-4.497	-4.507	-2.892	4.941	4.783	3.070	66.889	11.961	14.465
2594	147	24	17	P	S	42.520	-4.685	4.345	-2.697	5.169	-4.592	2.851	66.895	14.491	11.939
2595	147	24	17	P	S	50.485	-4.497	-2.209	-2.892	4.941	2.345	3.070	68.045	11.961	14.344
2596	147	24	17	P	S	53.413	-3.853	2.739	-3.549	4.175	-2.948	3.821	68.038	14.354	11.952
2597	147	24	17	P	S	66.587	-3.853	-2.739	-3.549	4.175	2.948	3.821	68.038	14.354	11.952
2598	147	24	17	P	S	69.515	-4.497	2.209	-2.892	4.941	-2.345	3.070	68.045	11.961	14.344
2599	147	24	17	P	S	77.480	-4.685	-4.344	-2.697	5.169	4.592	2.851	66.895	14.491	11.939
2600	147	24	17	P	S	82.689	-4.497	4.507	-2.892	4.941	-4.783	3.070	66.889	11.961	14.465
2601	147	24	17	P	S	97.311	-4.497	-4.507	-2.892	4.941	4.783	3.070	66.889	11.961	14.465

BL number	Atoms	γ-PC unit cells	WS_2 unit cells	γ-PC origin atom	WS_2 origin atom	Twist-angle (°)	γ-PC strain 1 (%)	γ-PC strain 2 (%)	γ-PC strain 3 (%)	WS_2 strain 1 (%)	WS_2 strain 2 (%)	WS_2 strain 3 (%)	γ (°)	$\lvert\mathbf{a}\rvert$(Å)	$\lvert\mathbf{b}\rvert$(Å)
2602	147	24	17	P	S	110.485	-4.497	-2.209	-2.892	4.941	2.345	3.070	68.045	11.961	14.344
2603	147	24	17	P	S	113.413	-3.853	2.739	-3.549	4.175	-2.948	3.821	68.038	14.354	11.952
2604	147	24	17	P	W	6.587	-3.853	-2.739	-3.549	4.175	2.948	3.821	68.038	14.354	11.952
2605	147	24	17	P	W	9.515	-4.497	2.209	-2.892	4.941	-2.345	3.070	68.045	11.961	14.344
2606	147	24	17	P	W	17.480	-4.685	-4.344	-2.697	5.169	4.592	2.851	66.895	14.491	11.939
2607	147	24	17	P	W	50.485	-4.497	-2.209	-2.892	4.941	2.345	3.070	68.045	11.961	14.344
2608	147	24	17	P	W	53.413	-3.853	2.739	-3.549	4.175	-2.948	3.821	68.038	14.354	11.952
2609	147	24	17	P	W	66.587	-3.853	-2.739	-3.549	4.175	2.948	3.821	68.038	14.354	11.952
2610	147	24	17	P	W	69.515	-4.497	2.209	-2.892	4.941	-2.345	3.070	68.045	11.961	14.344
2611	147	24	17	P	W	102.520	-4.685	4.345	-2.697	5.169	-4.592	2.851	66.895	14.491	11.939
2612	147	24	17	P	W	110.485	-4.497	-2.209	-2.892	4.941	2.345	3.070	68.045	11.961	14.344
2613	147	24	17	P	W	113.413	-3.853	2.739	-3.549	4.175	-2.948	3.821	68.038	14.354	11.952
2614	148	22	20	C	S	0.000	5.011	0.000	0.010	-4.554	0.000	-0.010	90.000	5.226	31.608
2615	148	22	20	C	S	60.000	5.011	0.000	0.010	-4.554	0.000	-0.010	90.000	5.226	31.608
2616	148	22	20	C	W	0.000	5.011	0.000	0.010	-4.554	0.000	-0.010	90.000	5.226	31.608
2617	148	22	20	C	W	60.000	5.011	0.000	0.010	-4.554	0.000	-0.010	90.000	5.226	31.608
2618	148	22	20	P	S	0.000	5.011	0.000	0.010	-4.554	0.000	-0.010	90.000	5.226	31.608
2619	148	22	20	P	W	60.000	5.011	0.000	0.010	-4.554	0.000	-0.010	90.000	5.226	31.608
2620	148	22	20	C	S	16.102	2.853	-2.699	2.052	-2.699	2.593	-1.971	88.485	10.655	15.499
2621	148	22	20	C	S	17.696	1.029	0.143	3.913	-1.008	-0.133	-3.629	88.533	10.654	15.503
2622	148	22	20	C	S	18.613	3.582	1.763	1.344	-3.342	-1.717	-1.309	88.525	15.509	10.649
2623	148	22	20	C	S	19.107	1.980	-4.817	2.926	-1.905	4.551	-2.764	71.767	13.816	12.580
2624	148	22	20	C	S	23.413	0.481	2.967	4.497	-0.477	-2.722	-4.126	71.675	12.584	13.824
2625	148	22	20	C	S	36.587	0.481	-2.967	4.497	-0.477	2.722	-4.126	71.675	12.584	13.824
2626	148	22	20	C	S	40.893	1.980	-2.676	2.926	-1.905	2.528	-2.764	88.511	15.504	10.651
2627	148	22	20	C	S	41.387	3.582	-1.763	1.344	-3.342	1.717	-1.309	88.525	10.649	15.509
2628	148	22	20	C	S	43.898	2.853	2.699	2.052	-2.699	-2.593	-1.971	88.485	10.655	15.499
2629	148	22	20	C	S	76.102	2.853	-2.699	2.052	-2.699	2.593	-1.971	88.485	10.655	15.499
2630	148	22	20	C	S	77.696	1.029	0.143	3.913	-1.008	-0.133	-3.629	88.533	10.654	15.503
2631	148	22	20	C	S	78.613	3.582	1.763	1.344	-3.342	-1.717	-1.309	88.525	15.509	10.649
2632	148	22	20	C	S	79.107	1.980	-4.817	2.926	-1.905	4.551	-2.764	71.767	13.816	12.580
2633	148	22	20	C	S	83.413	0.481	2.967	4.497	-0.477	-2.722	-4.126	71.675	12.584	13.824
2634	148	22	20	C	S	96.587	0.481	-2.967	4.497	-0.477	2.722	-4.126	71.675	12.584	13.824
2635	148	22	20	C	S	100.893	1.980	-2.676	2.926	-1.905	2.528	-2.764	88.511	15.504	10.651
2636	148	22	20	C	S	101.387	3.582	-1.763	1.344	-3.342	1.717	-1.309	88.525	10.649	15.509
2637	148	22	20	C	S	102.304	1.029	-0.143	3.912	-1.008	0.133	-3.628	88.533	15.503	10.654
2638	148	22	20	C	S	103.898	2.853	2.699	2.052	-2.699	-2.593	-1.971	88.485	10.655	15.499
2639	148	22	20	C	W	16.102	2.853	-2.699	2.052	-2.699	2.593	-1.971	88.485	10.655	15.499
2640	148	22	20	C	W	17.696	1.029	0.143	3.913	-1.008	-0.133	-3.629	88.533	10.654	15.503
2641	148	22	20	C	W	18.613	3.582	1.763	1.344	-3.342	-1.717	-1.309	88.525	15.509	10.649
2642	148	22	20	C	W	19.107	1.980	-4.817	2.926	-1.905	4.551	-2.764	71.767	13.816	12.580
2643	148	22	20	C	W	23.413	0.481	2.967	4.497	-0.477	-2.722	-4.126	71.675	12.584	13.824
2644	148	22	20	C	W	36.587	0.481	-2.967	4.497	-0.477	2.722	-4.126	71.675	12.584	13.824
2645	148	22	20	C	W	40.893	1.980	-2.676	2.926	-1.905	2.528	-2.764	88.511	15.504	10.651
2646	148	22	20	C	W	41.387	3.582	-1.763	1.344	-3.342	1.717	-1.309	88.525	10.649	15.509
2647	148	22	20	C	W	42.304	1.029	-0.143	3.912	-1.008	0.133	-3.628	88.533	15.503	10.654
2648	148	22	20	C	W	43.898	2.853	2.699	2.052	-2.699	-2.593	-1.971	88.485	10.655	15.499
2649	148	22	20	C	W	76.102	2.853	-2.699	2.052	-2.699	2.593	-1.971	88.485	10.655	15.499
2650	148	22	20	C	W	77.696	1.029	0.143	3.913	-1.008	-0.133	-3.629	88.533	10.654	15.503
2651	148	22	20	C	W	78.613	3.582	1.763	1.344	-3.342	-1.717	-1.309	88.525	15.509	10.649
2652	148	22	20	C	W	79.107	1.980	-4.817	2.926	-1.905	4.551	-2.764	71.767	13.816	12.580

BL number	Atoms	γ-PC unit cells	WS_2 unit cells	γ-PC origin atom	WS_2 origin atom	Twist-angle (°)	γ-PC strain 1 (%)	γ-PC strain 2 (%)	γ-PC strain 3 (%)	WS_2 strain 1 (%)	WS_2 strain 2 (%)	WS_2 strain 3 (%)	γ (°)	|**a**|(Å)	|**b**|(Å)
2653	148	22	20	C	W	83.413	0.481	2.967	4.497	-0.477	-2.722	-4.126	71.675	12.584	13.824
2654	148	22	20	C	W	96.587	0.481	-2.967	4.497	-0.477	2.722	-4.126	71.675	12.584	13.824
2655	148	22	20	C	W	100.893	1.980	-2.676	2.926	-1.905	2.528	-2.764	88.511	15.504	10.651
2656	148	22	20	C	W	101.387	3.582	-1.763	1.344	-3.342	1.717	-1.309	88.525	10.649	15.509
2657	148	22	20	C	W	103.898	2.853	2.699	2.052	-2.699	-2.593	-1.971	88.485	10.655	15.499
2658	148	22	20	P	S	16.102	2.853	-2.699	2.052	-2.699	2.593	-1.971	88.485	10.655	15.499
2659	148	22	20	P	S	17.696	1.029	0.143	3.913	-1.008	-0.133	-3.629	88.533	10.654	15.503
2660	148	22	20	P	S	18.613	3.582	1.763	1.344	-3.342	-1.717	-1.309	88.525	15.509	10.649
2661	148	22	20	P	S	36.587	0.481	-2.967	4.497	-0.477	2.722	-4.126	71.675	12.584	13.824
2662	148	22	20	P	S	40.893	1.980	-2.676	2.926	-1.905	2.528	-2.764	88.511	15.504	10.651
2663	148	22	20	P	S	41.387	3.582	-1.763	1.344	-3.342	1.717	-1.309	88.525	10.649	15.509
2664	148	22	20	P	S	42.304	1.029	-0.143	3.912	-1.008	0.133	-3.628	88.533	15.503	10.654
2665	148	22	20	P	S	43.898	2.853	2.699	2.052	-2.699	-2.593	-1.971	88.485	10.655	15.499
2666	148	22	20	P	S	76.102	2.853	-2.699	2.052	-2.699	2.593	-1.971	88.485	10.655	15.499
2667	148	22	20	P	S	77.696	1.029	0.143	3.913	-1.008	-0.133	-3.629	88.533	10.654	15.503
2668	148	22	20	P	S	78.613	3.582	1.763	1.344	-3.342	-1.717	-1.309	88.525	15.509	10.649
2669	148	22	20	P	S	79.107	1.980	-4.817	2.926	-1.905	4.551	-2.764	71.767	13.816	12.580
2670	148	22	20	P	S	83.413	0.481	2.967	4.497	-0.477	-2.722	-4.126	71.675	12.584	13.824
2671	148	22	20	P	S	100.893	1.980	-2.676	2.926	-1.905	2.528	-2.764	88.511	15.504	10.651
2672	148	22	20	P	S	101.387	3.582	-1.763	1.344	-3.342	1.717	-1.309	88.525	10.649	15.509
2673	148	22	20	P	S	103.898	2.853	2.699	2.052	-2.699	-2.593	-1.971	88.485	10.655	15.499
2674	148	22	20	P	W	16.102	2.853	-2.699	2.052	-2.699	2.593	-1.971	88.485	10.655	15.499
2675	148	22	20	P	W	17.696	1.029	0.143	3.913	-1.008	-0.133	-3.629	88.533	10.654	15.503
2676	148	22	20	P	W	18.613	3.582	1.763	1.344	-3.342	-1.717	-1.309	88.525	15.509	10.649
2677	148	22	20	P	W	19.107	1.980	-4.817	2.926	-1.905	4.551	-2.764	71.767	13.816	12.580
2678	148	22	20	P	W	23.413	0.481	2.967	4.497	-0.477	-2.722	-4.126	71.675	12.584	13.824
2679	148	22	20	P	W	40.893	1.980	-2.676	2.926	-1.905	2.528	-2.764	88.511	15.504	10.651
2680	148	22	20	P	W	41.387	3.582	-1.763	1.344	-3.342	1.717	-1.309	88.525	10.649	15.509
2681	148	22	20	P	W	43.898	2.853	2.699	2.052	-2.699	-2.593	-1.971	88.485	10.655	15.499
2682	148	22	20	P	W	76.102	2.853	-2.699	2.052	-2.699	2.593	-1.971	88.485	10.655	15.499
2683	148	22	20	P	W	77.696	1.029	0.143	3.913	-1.008	-0.133	-3.629	88.533	10.654	15.503
2684	148	22	20	P	W	78.613	3.582	1.763	1.344	-3.342	-1.717	-1.309	88.525	15.509	10.649
2685	148	22	20	P	W	96.587	0.481	-2.967	4.497	-0.477	2.722	-4.126	71.675	12.584	13.824
2686	148	22	20	P	W	100.893	1.980	-2.676	2.926	-1.905	2.528	-2.764	88.511	15.504	10.651
2687	148	22	20	P	W	101.387	3.582	-1.763	1.344	-3.342	1.717	-1.309	88.525	10.649	15.509
2688	148	22	20	P	W	102.304	1.029	-0.143	3.912	-1.008	0.133	-3.628	88.533	15.503	10.654
2689	148	22	20	P	W	103.898	2.853	2.699	2.052	-2.699	-2.593	-1.971	88.485	10.655	15.499
2690	149	23	19	C	S	0.000	5.011	-2.636	-4.556	-4.554	2.900	5.013	83.696	10.878	15.242
2691	149	23	19	C	S	1.945	-4.723	0.683	5.214	5.216	-0.619	-4.721	83.883	15.242	10.877
2692	149	23	19	C	S	22.689	-4.497	1.660	4.939	4.941	-1.511	-4.495	85.179	11.961	13.827
2693	149	23	19	C	S	37.311	-4.497	-1.660	4.939	4.941	1.510	-4.495	85.178	11.961	13.827
2694	149	23	19	C	S	58.055	-4.723	-0.683	5.214	5.216	0.619	-4.721	83.883	15.242	10.877
2695	149	23	19	C	S	60.000	5.011	2.636	-4.556	-4.554	-2.900	5.013	83.696	10.878	15.242
2696	149	23	19	C	S	61.945	-4.723	0.683	5.214	5.216	-0.619	-4.721	83.883	15.242	10.877
2697	149	23	19	C	S	82.689	-4.497	1.660	4.939	4.941	-1.511	-4.495	85.178	11.961	13.827
2698	149	23	19	C	S	97.311	-4.497	-1.660	4.939	4.941	1.510	-4.495	85.178	11.961	13.827
2699	149	23	19	C	S	118.055	-4.723	-0.683	5.214	5.216	0.619	-4.721	83.883	15.242	10.877
2700	149	23	19	C	W	0.000	5.011	-2.636	-4.556	-4.554	2.900	5.013	83.696	10.878	15.242
2701	149	23	19	C	W	1.945	-4.723	0.683	5.214	5.216	-0.619	-4.721	83.883	15.242	10.877
2702	149	23	19	C	W	22.689	-4.497	1.660	4.939	4.941	-1.511	-4.495	85.179	11.961	13.827
2703	149	23	19	C	W	37.311	-4.497	-1.660	4.939	4.941	1.510	-4.495	85.178	11.961	13.827

BL number	Atoms	γ-PC unit cells	WS_2 unit cells	γ-PC origin atom	WS_2 origin atom	Twist-angle (°)	γ-PC strain 1 (%)	γ-PC strain 2 (%)	γ-PC strain 3 (%)	WS_2 strain 1 (%)	WS_2 strain 2 (%)	WS_2 strain 3 (%)	γ (°)	|**a**|(Å)	|**b**|(Å)
2704	149	23	19	C	W	58.055	-4.723	-0.683	5.214	5.216	0.619	-4.721	83.883	15.242	10.877
2705	149	23	19	C	W	60.000	5.011	2.636	-4.556	-4.554	-2.900	5.013	83.696	10.878	15.242
2706	149	23	19	C	W	61.945	-4.723	0.683	5.214	5.216	-0.619	-4.721	83.883	15.242	10.877
2707	149	23	19	C	W	82.689	-4.497	1.660	4.939	4.941	-1.511	-4.495	85.178	11.961	13.827
2708	149	23	19	C	W	97.311	-4.497	-1.660	4.939	4.941	1.510	-4.495	85.178	11.961	13.827
2709	149	23	19	C	W	118.055	-4.723	-0.683	5.214	5.216	0.619	-4.721	83.883	15.242	10.877
2710	149	23	19	P	S	0.000	5.011	-2.636	-4.556	-4.554	2.900	5.013	83.696	10.878	15.242
2711	149	23	19	P	S	1.945	-4.723	0.683	5.214	5.216	-0.619	-4.721	83.883	15.242	10.877
2712	149	23	19	P	S	37.311	-4.497	-1.660	4.939	4.941	1.510	-4.495	85.178	11.961	13.827
2713	149	23	19	P	S	58.055	-4.723	-0.683	5.214	5.216	0.619	-4.721	83.883	15.242	10.877
2714	149	23	19	P	S	60.000	5.011	2.636	-4.556	-4.554	-2.900	5.013	83.696	10.878	15.242
2715	149	23	19	P	S	61.945	-4.723	0.683	5.214	5.216	-0.619	-4.721	83.883	15.242	10.877
2716	149	23	19	P	S	82.689	-4.497	1.660	4.939	4.941	-1.511	-4.495	85.178	11.961	13.827
2717	149	23	19	P	S	118.055	-4.723	-0.683	5.214	5.216	0.619	-4.721	83.883	15.242	10.877
2718	149	23	19	P	W	0.000	5.011	-2.636	-4.556	-4.554	2.900	5.013	83.696	10.878	15.242
2719	149	23	19	P	W	1.945	-4.723	0.683	5.214	5.216	-0.619	-4.721	83.883	15.242	10.877
2720	149	23	19	P	W	22.689	-4.497	1.660	4.939	4.941	-1.511	-4.495	85.179	11.961	13.827
2721	149	23	19	P	W	58.055	-4.723	-0.683	5.214	5.216	0.619	-4.721	83.883	15.242	10.877
2722	149	23	19	P	W	60.000	5.011	2.636	-4.556	-4.554	-2.900	5.013	83.696	10.878	15.242
2723	149	23	19	P	W	61.945	-4.723	0.683	5.214	5.216	-0.619	-4.721	83.883	15.242	10.877
2724	149	23	19	P	W	97.311	-4.497	-1.660	4.939	4.941	1.510	-4.495	85.178	11.961	13.827
2725	149	23	19	P	W	118.055	-4.723	-0.683	5.214	5.216	0.619	-4.721	83.883	15.242	10.877
2726	150	24	18	C	S	7.589	0.334	-4.626	-4.909	-0.332	5.130	5.443	86.380	11.427	14.355
2727	150	24	18	C	S	24.791	-3.388	-4.233	-1.307	3.635	4.347	1.342	76.521	17.335	9.705
2728	150	24	18	C	S	25.050	-0.955	-3.689	-3.723	0.973	3.986	4.023	76.451	9.710	17.332
2729	150	24	18	C	S	30.000	-2.359	-4.698	-2.359	2.476	4.930	2.476	76.474	9.718	17.313
2730	150	24	18	C	S	34.950	-0.955	3.689	-3.723	0.973	-3.986	4.023	76.451	17.332	9.710
2731	150	24	18	C	S	35.209	-3.388	4.233	-1.307	3.635	-4.347	1.342	76.521	17.335	9.705
2732	150	24	18	C	S	52.411	0.334	4.626	-4.909	-0.332	-5.130	5.443	86.380	14.355	11.427
2733	150	24	18	C	S	67.589	0.334	-4.626	-4.909	-0.332	5.130	5.443	86.380	11.427	14.355
2734	150	24	18	C	S	84.791	-3.388	-4.233	-1.307	3.635	4.347	1.342	76.521	17.335	9.705
2735	150	24	18	C	S	85.050	-0.955	-3.689	-3.723	0.973	3.986	4.023	76.451	9.710	17.332
2736	150	24	18	C	S	90.000	-2.359	-4.698	-2.359	2.476	4.930	2.476	76.474	9.718	17.313
2737	150	24	18	C	S	94.950	-0.955	3.689	-3.723	0.973	-3.986	4.023	76.451	17.332	9.710
2738	150	24	18	C	S	95.209	-3.388	4.233	-1.307	3.635	-4.347	1.342	76.521	17.335	9.705
2739	150	24	18	C	S	112.411	0.334	4.626	-4.909	-0.332	-5.130	5.443	86.380	14.355	11.427
2740	150	24	18	C	W	7.589	0.334	-4.626	-4.909	-0.332	5.130	5.443	86.380	11.427	14.355
2741	150	24	18	C	W	24.791	-3.388	-4.233	-1.307	3.635	4.347	1.342	76.521	17.335	9.705
2742	150	24	18	C	W	25.050	-0.955	-3.689	-3.723	0.973	3.986	4.023	76.451	9.710	17.332
2743	150	24	18	C	W	30.000	-2.359	-4.698	-2.359	2.476	4.930	2.476	76.474	9.718	17.313
2744	150	24	18	C	W	34.950	-0.955	3.689	-3.723	0.973	-3.986	4.023	76.451	17.332	9.710
2745	150	24	18	C	W	35.209	-3.388	4.233	-1.307	3.635	-4.347	1.342	76.521	17.335	9.705
2746	150	24	18	C	W	52.411	0.334	4.626	-4.909	-0.332	-5.130	5.443	86.380	14.355	11.427
2747	150	24	18	C	W	67.589	0.334	-4.626	-4.909	-0.332	5.130	5.443	86.380	11.427	14.355
2748	150	24	18	C	W	84.791	-3.388	-4.233	-1.307	3.635	4.347	1.342	76.521	17.335	9.705
2749	150	24	18	C	W	85.050	-0.955	-3.689	-3.723	0.973	3.986	4.023	76.451	9.710	17.332
2750	150	24	18	C	W	90.000	-2.359	-4.698	-2.359	2.476	4.930	2.476	76.474	9.718	17.313
2751	150	24	18	C	W	94.950	-0.955	3.689	-3.723	0.973	-3.986	4.023	76.451	17.332	9.710
2752	150	24	18	C	W	95.209	-3.388	4.233	-1.307	3.635	-4.347	1.342	76.521	17.335	9.705
2753	150	24	18	C	W	112.411	0.334	4.626	-4.909	-0.332	-5.130	5.443	86.380	14.355	11.427
2754	150	24	18	P	S	7.589	0.334	-4.626	-4.909	-0.332	5.130	5.443	86.380	11.427	14.355

BL number	Atoms	γ-PC unit cells	WS_2 unit cells	γ-PC origin atom	WS_2 origin atom	Twist-angle (°)	γ-PC strain 1 (%)	γ-PC strain 2 (%)	γ-PC strain 3 (%)	WS_2 strain 1 (%)	WS_2 strain 2 (%)	WS_2 strain 3 (%)	γ (°)	**|a|**(Å)	**|b|**(Å)
2755	150	24	18	P	S	52.411	0.334	4.626	-4.909	-0.332	-5.130	5.443	86.380	14.355	11.427
2756	150	24	18	P	S	67.589	0.334	-4.626	-4.909	-0.332	5.130	5.443	86.380	11.427	14.355
2757	150	24	18	P	S	112.411	0.334	4.626	-4.909	-0.332	-5.130	5.443	86.380	14.355	11.427
2758	150	24	18	P	W	7.589	0.334	-4.626	-4.909	-0.332	5.130	5.443	86.380	11.427	14.355
2759	150	24	18	P	W	52.411	0.334	4.626	-4.909	-0.332	-5.130	5.443	86.380	14.355	11.427
2760	150	24	18	P	W	67.589	0.334	-4.626	-4.909	-0.332	5.130	5.443	86.380	11.427	14.355
2761	150	24	18	P	W	112.411	0.334	4.626	-4.909	-0.332	-5.130	5.443	86.380	14.355	11.427
2762	151	22	21	C	S	10.893	4.020	-2.730	3.474	-3.721	2.552	-3.248	72.881	13.143	13.478
2763	151	22	21	C	S	12.520	2.326	0.212	5.205	-2.223	-0.192	-4.714	72.865	13.473	13.151
2764	151	22	21	C	S	13.174	5.011	1.378	2.510	-4.554	-1.312	-2.390	72.821	13.151	13.475
2765	151	22	21	C	S	46.827	5.011	-1.378	2.510	-4.554	1.312	-2.390	72.821	13.151	13.475
2766	151	22	21	C	S	47.480	2.326	-0.212	5.205	-2.223	0.192	-4.714	72.865	13.473	13.151
2767	151	22	21	C	S	49.107	4.020	2.730	3.474	-3.721	-2.553	-3.248	72.881	13.478	13.143
2768	151	22	21	C	S	70.893	4.020	-2.730	3.474	-3.721	2.552	-3.248	72.881	13.143	13.478
2769	151	22	21	C	S	72.520	2.326	0.212	5.205	-2.223	-0.192	-4.714	72.865	13.473	13.151
2770	151	22	21	C	S	73.174	5.011	1.378	2.510	-4.554	-1.312	-2.390	72.821	13.151	13.475
2771	151	22	21	C	S	106.827	5.011	-1.378	2.510	-4.554	1.312	-2.390	72.821	13.151	13.475
2772	151	22	21	C	S	107.480	2.326	-0.212	5.205	-2.223	0.192	-4.714	72.865	13.473	13.151
2773	151	22	21	C	S	109.107	4.020	2.730	3.474	-3.721	-2.553	-3.248	72.881	13.478	13.143
2774	151	22	21	C	W	10.893	4.020	-2.730	3.474	-3.721	2.552	-3.248	72.881	13.143	13.478
2775	151	22	21	C	W	12.520	2.326	0.212	5.205	-2.223	-0.192	-4.714	72.865	13.473	13.151
2776	151	22	21	C	W	13.174	5.011	1.378	2.510	-4.554	-1.312	-2.390	72.821	13.151	13.475
2777	151	22	21	C	W	46.827	5.011	-1.378	2.510	-4.554	1.312	-2.390	72.821	13.151	13.475
2778	151	22	21	C	W	47.480	2.326	-0.212	5.205	-2.223	0.192	-4.714	72.865	13.473	13.151
2779	151	22	21	C	W	49.107	4.020	2.730	3.474	-3.721	-2.553	-3.248	72.881	13.478	13.143
2780	151	22	21	C	W	70.893	4.020	-2.730	3.474	-3.721	2.552	-3.248	72.881	13.143	13.478
2781	151	22	21	C	W	72.520	2.326	0.212	5.205	-2.223	-0.192	-4.714	72.865	13.473	13.151
2782	151	22	21	C	W	73.174	5.011	1.378	2.510	-4.554	-1.312	-2.390	72.821	13.151	13.475
2783	151	22	21	C	W	106.827	5.011	-1.378	2.510	-4.554	1.312	-2.390	72.821	13.151	13.475
2784	151	22	21	C	W	107.480	2.326	-0.212	5.205	-2.223	0.192	-4.714	72.865	13.473	13.151
2785	151	22	21	C	W	109.107	4.020	2.730	3.474	-3.721	-2.553	-3.248	72.881	13.478	13.143
2786	151	22	21	P	S	10.893	4.020	-2.730	3.474	-3.721	2.552	-3.248	72.881	13.143	13.478
2787	151	22	21	P	S	12.520	2.326	0.212	5.205	-2.223	-0.192	-4.714	72.865	13.473	13.151
2788	151	22	21	P	S	13.174	5.011	1.378	2.510	-4.554	-1.312	-2.390	72.821	13.151	13.475
2789	151	22	21	P	S	46.827	5.011	-1.378	2.510	-4.554	1.312	-2.390	72.821	13.151	13.475
2790	151	22	21	P	S	47.480	2.326	-0.212	5.205	-2.223	0.192	-4.714	72.865	13.473	13.151
2791	151	22	21	P	S	49.107	4.020	2.730	3.474	-3.721	-2.553	-3.248	72.881	13.478	13.143
2792	151	22	21	P	S	70.893	4.020	-2.730	3.474	-3.721	2.552	-3.248	72.881	13.143	13.478
2793	151	22	21	P	S	72.520	2.326	0.212	5.205	-2.223	-0.192	-4.714	72.865	13.473	13.151
2794	151	22	21	P	S	73.174	5.011	1.378	2.510	-4.554	-1.312	-2.390	72.821	13.151	13.475
2795	151	22	21	P	S	106.827	5.011	-1.378	2.510	-4.554	1.312	-2.390	72.821	13.151	13.475
2796	151	22	21	P	S	107.480	2.326	-0.212	5.205	-2.223	0.192	-4.714	72.865	13.473	13.151
2797	151	22	21	P	S	109.107	4.020	2.730	3.474	-3.721	-2.553	-3.248	72.881	13.478	13.143
2798	151	22	21	P	W	10.893	4.020	-2.730	3.474	-3.721	2.552	-3.248	72.881	13.143	13.478
2799	151	22	21	P	W	12.520	2.326	0.212	5.205	-2.223	-0.192	-4.714	72.865	13.473	13.151
2800	151	22	21	P	W	13.174	5.011	1.378	2.510	-4.554	-1.312	-2.390	72.821	13.151	13.475
2801	151	22	21	P	W	46.827	5.011	-1.378	2.510	-4.554	1.312	-2.390	72.821	13.151	13.475
2802	151	22	21	P	W	47.480	2.326	-0.212	5.205	-2.223	0.192	-4.714	72.865	13.473	13.151
2803	151	22	21	P	W	49.107	4.020	2.730	3.474	-3.721	-2.553	-3.248	72.881	13.478	13.143
2804	151	22	21	P	W	70.893	4.020	-2.730	3.474	-3.721	2.552	-3.248	72.881	13.143	13.478
2805	151	22	21	P	W	72.520	2.326	0.212	5.205	-2.223	-0.192	-4.714	72.865	13.473	13.151

BL number	Atoms	γ-PC unit cells	WS_2 unit cells	γ-PC origin atom	WS_2 origin atom	Twist-angle (°)	γ-PC strain 1 (%)	γ-PC strain 2 (%)	γ-PC strain 3 (%)	WS_2 strain 1 (%)	WS_2 strain 2 (%)	WS_2 strain 3 (%)	γ (°)	|**a**|(Å)	|**b**|(Å)
2806	151	22	21	P	W	73.174	5.011	1.378	2.510	-4.554	-1.312	-2.390	72.821	13.151	13.475
2807	151	22	21	P	W	106.827	5.011	-1.378	2.510	-4.554	1.312	-2.390	72.821	13.151	13.475
2808	151	22	21	P	W	107.480	2.326	-0.212	5.205	-2.223	0.192	-4.714	72.865	13.473	13.151
2809	151	22	21	P	W	109.107	4.020	2.730	3.474	-3.721	-2.553	-3.248	72.881	13.478	13.143
2810	152	23	20	C	S	10.893	-1.983	-2.768	4.803	2.064	2.525	-4.382	84.469	12.906	13.150
2811	152	23	20	C	S	13.174	5.011	1.318	-2.164	-4.554	-1.378	2.262	84.613	13.151	12.902
2812	152	23	20	C	S	23.413	0.481	-2.207	2.128	-0.477	2.117	-2.041	84.533	12.584	13.470
2813	152	23	20	C	S	23.691	2.390	-1.681	0.229	-2.281	1.673	-0.228	84.529	13.468	12.587
2814	152	23	20	C	S	25.693	2.326	1.825	0.290	-2.223	-1.815	-0.289	84.571	13.473	12.581
2815	152	23	20	C	S	34.233	0.353	-1.991	2.260	-0.351	1.905	-2.162	84.549	13.472	12.582
2816	152	23	20	C	S	36.309	2.390	1.681	0.229	-2.281	-1.673	-0.228	84.529	12.587	13.468
2817	152	23	20	C	S	36.587	0.481	2.207	2.128	-0.477	-2.117	-2.041	84.533	12.584	13.470
2818	152	23	20	C	S	46.827	5.011	-1.318	-2.164	-4.554	1.378	2.262	84.613	13.151	12.902
2819	152	23	20	C	S	49.107	-1.983	2.768	4.803	2.064	-2.525	-4.382	84.469	12.906	13.150
2820	152	23	20	C	S	70.893	-1.983	-2.768	4.803	2.064	2.525	-4.382	84.469	12.906	13.150
2821	152	23	20	C	S	73.174	5.011	1.318	-2.164	-4.554	-1.378	2.262	84.613	13.151	12.902
2822	152	23	20	C	S	83.413	0.481	-2.207	2.128	-0.477	2.117	-2.041	84.533	12.584	13.470
2823	152	23	20	C	S	83.691	2.390	-1.681	0.229	-2.281	1.673	-0.228	84.529	13.468	12.587
2824	152	23	20	C	S	85.694	2.326	1.825	0.290	-2.223	-1.815	-0.289	84.571	13.473	12.581
2825	152	23	20	C	S	94.233	0.353	-1.991	2.260	-0.351	1.905	-2.162	84.549	13.472	12.582
2826	152	23	20	C	S	96.309	2.390	1.681	0.229	-2.281	-1.673	-0.228	84.529	12.587	13.468
2827	152	23	20	C	S	96.587	0.481	2.207	2.128	-0.477	-2.117	-2.041	84.533	12.584	13.470
2828	152	23	20	C	S	106.827	5.011	-1.318	-2.164	-4.554	1.378	2.262	84.613	13.151	12.902
2829	152	23	20	C	S	109.107	-1.983	2.768	4.803	2.064	-2.525	-4.382	84.469	12.906	13.150
2830	152	23	20	C	W	10.893	-1.983	-2.768	4.803	2.064	2.525	-4.382	84.469	12.906	13.150
2831	152	23	20	C	W	13.174	5.011	1.318	-2.164	-4.554	-1.378	2.262	84.613	13.151	12.902
2832	152	23	20	C	W	23.413	0.481	-2.207	2.128	-0.477	2.117	-2.041	84.533	12.584	13.470
2833	152	23	20	C	W	23.691	2.390	-1.681	0.229	-2.281	1.673	-0.228	84.529	13.468	12.587
2834	152	23	20	C	W	25.693	2.326	1.825	0.290	-2.223	-1.815	-0.289	84.571	13.473	12.581
2835	152	23	20	C	W	34.233	0.353	-1.991	2.260	-0.351	1.905	-2.162	84.549	13.472	12.582
2836	152	23	20	C	W	36.309	2.390	1.681	0.229	-2.281	-1.673	-0.228	84.529	12.587	13.468
2837	152	23	20	C	W	36.587	0.481	2.207	2.128	-0.477	-2.117	-2.041	84.533	12.584	13.470
2838	152	23	20	C	W	46.827	5.011	-1.318	-2.164	-4.554	1.378	2.262	84.613	13.151	12.902
2839	152	23	20	C	W	49.107	-1.983	2.768	4.803	2.064	-2.525	-4.382	84.469	12.906	13.150
2840	152	23	20	C	W	70.893	-1.983	-2.768	4.803	2.064	2.525	-4.382	84.469	12.906	13.150
2841	152	23	20	C	W	73.174	5.011	1.318	-2.164	-4.554	-1.378	2.262	84.613	13.151	12.902
2842	152	23	20	C	W	83.413	0.481	-2.207	2.128	-0.477	2.117	-2.041	84.533	12.584	13.470
2843	152	23	20	C	W	83.691	2.390	-1.681	0.229	-2.281	1.673	-0.228	84.529	13.468	12.587
2844	152	23	20	C	W	85.694	2.326	1.825	0.290	-2.223	-1.815	-0.289	84.571	13.473	12.581
2845	152	23	20	C	W	94.233	0.353	-1.991	2.260	-0.351	1.905	-2.162	84.549	13.472	12.582
2846	152	23	20	C	W	96.309	2.390	1.681	0.229	-2.281	-1.673	-0.228	84.529	12.587	13.468
2847	152	23	20	C	W	96.587	0.481	2.207	2.128	-0.477	-2.117	-2.041	84.533	12.584	13.470
2848	152	23	20	C	W	106.827	5.011	-1.318	-2.164	-4.554	1.378	2.262	84.613	13.151	12.902
2849	152	23	20	C	W	109.107	-1.983	2.768	4.803	2.064	-2.525	-4.382	84.469	12.906	13.150
2850	152	23	20	P	S	10.893	-1.983	-2.768	4.803	2.064	2.525	-4.382	84.469	12.906	13.150
2851	152	23	20	P	S	13.174	5.011	1.318	-2.164	-4.554	-1.378	2.262	84.613	13.151	12.902
2852	152	23	20	P	S	23.413	0.481	-2.207	2.128	-0.477	2.117	-2.041	84.533	12.584	13.470
2853	152	23	20	P	S	23.691	2.390	-1.681	0.229	-2.281	1.673	-0.228	84.529	13.468	12.587
2854	152	23	20	P	S	25.693	2.326	1.825	0.290	-2.223	-1.815	-0.289	84.571	13.473	12.581
2855	152	23	20	P	S	34.233	0.353	-1.991	2.260	-0.351	1.905	-2.162	84.549	13.472	12.582
2856	152	23	20	P	S	36.309	2.390	1.681	0.229	-2.281	-1.673	-0.228	84.529	12.587	13.468

BL number	Atoms	γ-PC unit cells	WS_2 unit cells	γ-PC origin atom	WS_2 origin atom	Twist-angle (°)	γ-PC strain 1 (%)	γ-PC strain 2 (%)	γ-PC strain 3 (%)	WS_2 strain 1 (%)	WS_2 strain 2 (%)	WS_2 strain 3 (%)	γ (°)	$\lvert\mathbf{a}\rvert$(Å)	$\lvert\mathbf{b}\rvert$(Å)
2857	152	23	20	P	S	36.587	0.481	2.207	2.128	-0.477	-2.117	-2.041	84.533	12.584	13.470
2858	152	23	20	P	S	46.827	5.011	-1.318	-2.164	-4.554	1.378	2.262	84.613	13.151	12.902
2859	152	23	20	P	S	49.107	-1.983	2.768	4.803	2.064	-2.525	-4.382	84.469	12.906	13.150
2860	152	23	20	P	S	70.893	-1.983	-2.768	4.803	2.064	2.525	-4.382	84.469	12.906	13.150
2861	152	23	20	P	S	73.174	5.011	1.318	-2.164	-4.554	-1.378	2.262	84.613	13.151	12.902
2862	152	23	20	P	S	83.413	0.481	-2.207	2.128	-0.477	2.117	-2.041	84.533	12.584	13.470
2863	152	23	20	P	S	83.691	2.390	-1.681	0.229	-2.281	1.673	-0.228	84.529	13.468	12.587
2864	152	23	20	P	S	85.694	2.326	1.825	0.290	-2.223	-1.815	-0.289	84.571	13.473	12.581
2865	152	23	20	P	S	94.233	0.353	-1.991	2.260	-0.351	1.905	-2.162	84.549	13.472	12.582
2866	152	23	20	P	S	96.309	2.390	1.681	0.229	-2.281	-1.673	-0.228	84.529	12.587	13.468
2867	152	23	20	P	S	96.587	0.481	2.207	2.128	-0.477	-2.117	-2.041	84.533	12.584	13.470
2868	152	23	20	P	S	106.827	5.011	-1.318	-2.164	-4.554	1.378	2.262	84.613	13.151	12.902
2869	152	23	20	P	S	109.107	-1.983	2.768	4.803	2.064	-2.525	-4.382	84.469	12.906	13.150
2870	152	23	20	P	W	10.893	-1.983	-2.768	4.803	2.064	2.525	-4.382	84.469	12.906	13.150
2871	152	23	20	P	W	13.174	5.011	1.318	-2.164	-4.554	-1.378	2.262	84.613	13.151	12.902
2872	152	23	20	P	W	23.413	0.481	-2.207	2.128	-0.477	2.117	-2.041	84.533	12.584	13.470
2873	152	23	20	P	W	23.691	2.390	-1.681	0.229	-2.281	1.673	-0.228	84.529	13.468	12.587
2874	152	23	20	P	W	25.693	2.326	1.825	0.290	-2.223	-1.815	-0.289	84.571	13.473	12.581
2875	152	23	20	P	W	34.233	0.353	-1.991	2.260	-0.351	1.905	-2.162	84.549	13.472	12.582
2876	152	23	20	P	W	36.309	2.390	1.681	0.229	-2.281	-1.673	-0.228	84.529	12.587	13.468
2877	152	23	20	P	W	36.587	0.481	2.207	2.128	-0.477	-2.117	-2.041	84.533	12.584	13.470
2878	152	23	20	P	W	46.827	5.011	-1.318	-2.164	-4.554	1.378	2.262	84.613	13.151	12.902
2879	152	23	20	P	W	49.107	-1.983	2.768	4.803	2.064	-2.525	-4.382	84.469	12.906	13.150
2880	152	23	20	P	W	70.893	-1.983	-2.768	4.803	2.064	2.525	-4.382	84.469	12.906	13.150
2881	152	23	20	P	W	73.174	5.011	1.318	-2.164	-4.554	-1.378	2.262	84.613	13.151	12.902
2882	152	23	20	P	W	83.413	0.481	-2.207	2.128	-0.477	2.117	-2.041	84.533	12.584	13.470
2883	152	23	20	P	W	83.691	2.390	-1.681	0.229	-2.281	1.673	-0.228	84.529	13.468	12.587
2884	152	23	20	P	W	85.694	2.326	1.825	0.290	-2.223	-1.815	-0.289	84.571	13.473	12.581
2885	152	23	20	P	W	94.233	0.353	-1.991	2.260	-0.351	1.905	-2.162	84.549	13.472	12.582
2886	152	23	20	P	W	96.309	2.390	1.681	0.229	-2.281	-1.673	-0.228	84.529	12.587	13.468
2887	152	23	20	P	W	96.587	0.481	2.207	2.128	-0.477	-2.117	-2.041	84.533	12.584	13.470
2888	152	23	20	P	W	106.827	5.011	-1.318	-2.164	-4.554	1.378	2.262	84.613	13.151	12.902
2889	152	23	20	P	W	109.107	-1.983	2.768	4.803	2.064	-2.525	-4.382	84.469	12.906	13.150
2890	153	24	19	C	S	8.213	-2.359	-4.698	0.288	2.476	4.671	-0.286	82.001	14.845	11.429
2891	153	24	19	C	S	8.948	1.048	-3.293	-3.069	-1.026	3.509	3.269	81.924	11.433	14.847
2892	153	24	19	C	S	13.898	-0.414	-4.423	-1.685	0.417	4.577	1.744	87.424	19.824	8.483
2893	153	24	19	C	S	16.537	-3.292	0.113	1.291	3.523	-0.110	-1.259	87.420	11.442	14.704
2894	153	24	19	C	S	19.107	-1.485	4.571	-0.619	1.530	-4.628	0.626	87.367	14.708	11.434
2895	153	24	19	C	S	40.893	-1.485	-4.571	-0.619	1.530	4.628	0.626	87.367	14.708	11.434
2896	153	24	19	C	S	43.463	-3.292	-0.113	1.291	3.523	0.110	-1.259	87.420	14.704	11.442
2897	153	24	19	C	S	46.102	-0.414	4.423	-1.685	0.417	-4.577	1.744	87.424	19.824	8.483
2898	153	24	19	C	S	51.052	1.048	3.293	-3.069	-1.026	-3.509	3.270	81.924	14.847	11.433
2899	153	24	19	C	S	51.787	-2.359	4.698	0.288	2.476	-4.671	-0.286	82.001	14.845	11.429
2900	153	24	19	C	S	68.213	-2.359	-4.698	0.288	2.476	4.671	-0.286	82.001	14.845	11.429
2901	153	24	19	C	S	68.948	1.048	-3.293	-3.069	-1.026	3.509	3.269	81.924	11.433	14.847
2902	153	24	19	C	S	73.898	-0.414	-4.423	-1.685	0.417	4.577	1.744	87.424	11.445	14.693
2903	153	24	19	C	S	77.696	1.029	2.069	-3.051	-1.008	-2.204	3.249	87.417	14.708	11.438
2904	153	24	19	C	S	79.107	-1.485	4.571	-0.619	1.530	-4.628	0.626	87.367	8.492	19.804
2905	153	24	19	C	S	100.893	-1.485	-4.571	-0.619	1.530	4.628	0.626	87.367	8.492	19.804
2906	153	24	19	C	S	102.304	1.029	-2.069	-3.051	-1.008	2.204	3.250	87.417	11.438	14.708
2907	153	24	19	C	S	103.463	-3.292	-0.113	1.291	3.523	0.110	-1.259	87.420	14.704	11.442

BL number	Atoms	γ-PC unit cells	WS_2 unit cells	γ-PC origin atom	WS_2 origin atom	Twist-angle (°)	γ-PC strain 1 (%)	γ-PC strain 2 (%)	γ-PC strain 3 (%)	WS_2 strain 1 (%)	WS_2 strain 2 (%)	WS_2 strain 3 (%)	γ (°)	|**a**|(Å)	|**b**|(Å)
2908	153	24	19	C	S	106.102	-0.414	-5.160	-1.685	0.417	5.340	1.744	81.893	11.445	14.826
2909	153	24	19	C	S	111.052	1.048	3.293	-3.069	-1.026	-3.509	3.270	81.924	14.847	11.433
2910	153	24	19	C	S	111.787	-2.359	4.698	0.288	2.476	-4.671	-0.286	82.001	14.845	11.429
2911	153	24	19	C	W	8.213	-2.359	-4.698	0.288	2.476	4.671	-0.286	82.001	14.845	11.429
2912	153	24	19	C	W	8.948	1.048	-3.293	-3.069	-1.026	3.509	3.269	81.924	11.433	14.847
2913	153	24	19	C	W	13.898	-0.414	-4.423	-1.685	0.417	4.577	1.744	87.424	19.824	8.483
2914	153	24	19	C	W	17.696	1.029	2.069	-3.051	-1.008	-2.204	3.249	87.417	14.708	11.438
2915	153	24	19	C	W	19.107	-1.485	4.571	-0.619	1.530	-4.628	0.626	87.367	8.492	19.804
2916	153	24	19	C	W	40.893	-1.485	-4.571	-0.619	1.530	4.628	0.626	87.367	8.492	19.804
2917	153	24	19	C	W	42.304	1.029	-2.069	-3.051	-1.008	2.204	3.250	87.417	11.438	14.708
2918	153	24	19	C	W	46.102	-0.414	4.423	-1.685	0.417	-4.577	1.744	87.424	19.824	8.483
2919	153	24	19	C	W	51.052	1.048	3.293	-3.069	-1.026	-3.509	3.270	81.924	14.847	11.433
2920	153	24	19	C	W	51.787	-2.359	4.698	0.288	2.476	-4.671	-0.286	82.001	14.845	11.429
2921	153	24	19	C	W	68.213	-2.359	-4.698	0.288	2.476	4.671	-0.286	82.001	14.845	11.429
2922	153	24	19	C	W	68.948	1.048	-3.293	-3.069	-1.026	3.509	3.269	81.924	11.433	14.847
2923	153	24	19	C	W	73.898	-0.414	5.160	-1.685	0.417	-5.340	1.744	81.893	11.445	14.826
2924	153	24	19	C	W	76.537	-3.292	0.113	1.291	3.523	-0.110	-1.259	87.420	11.442	14.704
2925	153	24	19	C	W	79.107	-1.485	4.571	-0.619	1.530	-4.628	0.626	87.367	8.492	19.804
2926	153	24	19	C	W	100.893	-1.485	-4.571	-0.619	1.530	4.628	0.626	87.367	8.492	19.804
2927	153	24	19	C	W	106.102	-0.414	-5.160	-1.685	0.417	5.340	1.744	81.893	11.445	14.826
2928	153	24	19	C	W	111.052	1.048	3.293	-3.069	-1.026	-3.509	3.270	81.924	14.847	11.433
2929	153	24	19	C	W	111.787	-2.359	4.698	0.288	2.476	-4.671	-0.286	82.001	14.845	11.429
2930	153	24	19	P	S	8.213	-2.359	-4.698	0.288	2.476	4.671	-0.286	82.001	14.845	11.429
2931	153	24	19	P	S	8.948	1.048	-3.293	-3.069	-1.026	3.509	3.269	81.924	11.433	14.847
2932	153	24	19	P	S	13.898	-0.414	-4.423	-1.685	0.417	4.577	1.744	87.424	11.445	14.693
2933	153	24	19	P	S	19.107	-1.485	4.571	-0.619	1.530	-4.628	0.626	87.367	14.708	11.434
2934	153	24	19	P	S	40.893	-1.485	-4.571	-0.619	1.530	4.628	0.626	87.367	14.708	11.434
2935	153	24	19	P	S	42.304	1.029	-2.069	-3.051	-1.008	2.204	3.250	87.417	11.438	14.708
2936	153	24	19	P	S	43.463	-3.292	-0.113	1.291	3.523	0.110	-1.259	87.420	14.704	11.442
2937	153	24	19	P	S	46.102	-0.414	4.423	-1.685	0.417	-4.577	1.744	87.424	19.824	8.483
2938	153	24	19	P	S	51.052	1.048	3.293	-3.069	-1.026	-3.509	3.270	81.924	14.847	11.433
2939	153	24	19	P	S	51.787	-2.359	4.698	0.288	2.476	-4.671	-0.286	82.001	14.845	11.429
2940	153	24	19	P	S	68.213	-2.359	-4.698	0.288	2.476	4.671	-0.286	82.001	14.845	11.429
2941	153	24	19	P	S	68.948	1.048	-3.293	-3.069	-1.026	3.509	3.269	81.924	11.433	14.847
2942	153	24	19	P	S	73.898	-0.414	-4.423	-1.685	0.417	4.577	1.744	87.424	11.445	14.693
2943	153	24	19	P	S	76.537	-3.292	0.113	1.291	3.523	-0.110	-1.259	87.420	11.442	14.704
2944	153	24	19	P	S	77.696	1.029	2.069	-3.051	-1.008	-2.204	3.249	87.417	14.708	11.438
2945	153	24	19	P	S	79.107	-1.485	4.571	-0.619	1.530	-4.628	0.626	87.367	8.492	19.804
2946	153	24	19	P	S	100.893	-1.485	-4.571	-0.619	1.530	4.628	0.626	87.367	14.708	11.434
2947	153	24	19	P	S	103.463	-3.292	-0.113	1.291	3.523	0.110	-1.259	87.420	14.704	11.442
2948	153	24	19	P	S	106.102	-0.414	-5.160	-1.685	0.417	5.340	1.744	81.893	11.445	14.826
2949	153	24	19	P	S	111.052	1.048	3.293	-3.069	-1.026	-3.509	3.270	81.924	14.847	11.433
2950	153	24	19	P	S	111.787	-2.359	4.698	0.288	2.476	-4.671	-0.286	82.001	14.845	11.429
2951	153	24	19	P	W	8.213	-2.359	-4.698	0.288	2.476	4.671	-0.286	82.001	14.845	11.429
2952	153	24	19	P	W	8.948	1.048	-3.293	-3.069	-1.026	3.509	3.269	81.924	11.433	14.847
2953	153	24	19	P	W	13.898	-0.414	-4.423	-1.685	0.417	4.577	1.744	87.424	19.824	8.483
2954	153	24	19	P	W	16.537	-3.292	0.113	1.291	3.523	-0.110	-1.259	87.420	11.442	14.704
2955	153	24	19	P	W	17.696	1.029	2.069	-3.051	-1.008	-2.204	3.249	87.417	14.708	11.438
2956	153	24	19	P	W	19.107	-1.485	4.571	-0.619	1.530	-4.628	0.626	87.367	8.492	19.804
2957	153	24	19	P	W	46.102	-0.414	-5.160	-1.685	0.417	5.340	1.744	81.892	11.445	14.826
2958	153	24	19	P	W	51.052	1.048	3.293	-3.069	-1.026	-3.509	3.270	81.924	14.847	11.433

BL number	Atoms	γ-PC unit cells	WS_2 unit cells	γ-PC origin atom	WS_2 origin atom	Twist-angle (°)	γ-PC strain 1 (%)	γ-PC strain 2 (%)	γ-PC strain 3 (%)	WS_2 strain 1 (%)	WS_2 strain 2 (%)	WS_2 strain 3 (%)	γ (°)	$\lvert\mathbf{a}\rvert$(Å)	$\lvert\mathbf{b}\rvert$(Å)
2959	153	24	19	P	W	51.787	-2.359	4.698	0.288	2.476	-4.671	-0.286	82.001	14.845	11.429
2960	153	24	19	P	W	68.213	-2.359	-4.698	0.288	2.476	4.671	-0.286	82.001	14.845	11.429
2961	153	24	19	P	W	68.948	1.048	-3.293	-3.069	-1.026	3.509	3.269	81.924	11.433	14.847
2962	153	24	19	P	W	73.898	-0.414	5.160	-1.685	0.417	-5.340	1.744	81.893	11.445	14.826
2963	153	24	19	P	W	79.107	-1.485	4.571	-0.619	1.530	-4.628	0.626	87.367	14.708	11.434
2964	153	24	19	P	W	100.893	-1.485	-4.571	-0.619	1.530	4.628	0.626	87.367	8.492	19.804
2965	153	24	19	P	W	102.304	1.029	-2.069	-3.051	-1.008	2.204	3.250	87.417	11.438	14.708
2966	153	24	19	P	W	106.102	-0.414	-5.160	-1.685	0.417	5.340	1.744	81.893	11.445	14.826
2967	153	24	19	P	W	111.052	1.048	3.293	-3.069	-1.026	-3.509	3.270	81.924	14.847	11.433
2968	153	24	19	P	W	111.787	-2.359	4.698	0.288	2.476	-4.671	-0.286	82.001	14.845	11.429
2969	154	25	18	C	S	13.898	-4.228	-1.843	-2.397	4.619	1.936	2.518	79.934	9.921	17.103
2970	154	25	18	C	S	15.608	-2.145	1.049	-4.469	2.241	-1.152	4.908	79.986	17.102	9.921
2971	154	25	18	C	S	16.537	-3.292	2.630	-3.352	3.523	-2.819	3.593	79.980	9.916	17.109
2972	154	25	18	C	S	22.689	-4.497	-0.933	-2.116	4.941	0.974	2.210	83.033	11.961	14.073
2973	154	25	18	C	S	23.413	-2.043	0.298	-4.567	2.130	-0.328	5.026	83.064	14.072	11.961
2974	154	25	18	C	S	24.791	-3.388	2.629	-3.255	3.635	-2.813	3.482	83.076	11.953	14.078
2975	154	25	18	C	S	35.209	-3.388	-2.629	-3.255	3.635	2.813	3.482	83.076	14.078	11.953
2976	154	25	18	C	S	36.587	-2.043	-0.298	-4.567	2.130	0.328	5.026	83.064	14.072	11.961
2977	154	25	18	C	S	37.311	-4.497	0.933	-2.116	4.941	-0.974	2.210	83.033	11.961	14.073
2978	154	25	18	C	S	43.463	-3.292	-2.630	-3.352	3.523	2.819	3.593	79.980	17.109	9.916
2979	154	25	18	C	S	44.392	-2.145	-1.049	-4.469	2.241	1.152	4.908	79.986	17.102	9.921
2980	154	25	18	C	S	46.102	-4.228	1.843	-2.397	4.619	-1.936	2.518	79.934	9.921	17.103
2981	154	25	18	C	S	73.898	-4.228	-1.843	-2.397	4.619	1.936	2.518	79.934	9.921	17.103
2982	154	25	18	C	S	75.609	-2.145	1.049	-4.469	2.241	-1.152	4.908	79.986	17.102	9.921
2983	154	25	18	C	S	76.537	-3.292	2.630	-3.352	3.523	-2.819	3.593	79.980	9.916	17.109
2984	154	25	18	C	S	82.689	-4.497	-0.933	-2.116	4.941	0.974	2.210	83.033	11.961	14.073
2985	154	25	18	C	S	83.413	-2.043	0.298	-4.567	2.130	-0.328	5.026	83.064	14.072	11.961
2986	154	25	18	C	S	84.791	-3.388	2.629	-3.255	3.635	-2.813	3.482	83.076	11.953	14.078
2987	154	25	18	C	S	95.209	-3.388	-2.629	-3.255	3.635	2.813	3.482	83.076	14.078	11.953
2988	154	25	18	C	S	96.587	-2.043	-0.298	-4.567	2.130	0.328	5.026	83.064	14.072	11.961
2989	154	25	18	C	S	97.311	-4.497	0.933	-2.116	4.941	-0.974	2.210	83.033	11.961	14.073
2990	154	25	18	C	S	103.463	-3.292	-2.630	-3.352	3.523	2.819	3.593	79.980	17.109	9.916
2991	154	25	18	C	S	104.392	-2.145	-1.049	-4.469	2.241	1.152	4.908	79.986	17.102	9.921
2992	154	25	18	C	S	106.102	-4.228	1.843	-2.397	4.619	-1.936	2.518	79.934	9.921	17.103
2993	154	25	18	C	W	13.898	-4.228	-1.843	-2.397	4.619	1.936	2.518	79.934	9.921	17.103
2994	154	25	18	C	W	15.608	-2.145	1.049	-4.469	2.241	-1.152	4.908	79.986	17.102	9.921
2995	154	25	18	C	W	16.537	-3.292	2.630	-3.352	3.523	-2.819	3.593	79.980	9.916	17.109
2996	154	25	18	C	W	22.689	-4.497	-0.933	-2.116	4.941	0.974	2.210	83.033	11.961	14.073
2997	154	25	18	C	W	23.413	-2.043	0.298	-4.567	2.130	-0.328	5.026	83.064	14.072	11.961
2998	154	25	18	C	W	24.791	-3.388	2.629	-3.255	3.635	-2.813	3.482	83.076	11.953	14.078
2999	154	25	18	C	W	35.209	-3.388	-2.629	-3.255	3.635	2.813	3.482	83.076	14.078	11.953
3000	154	25	18	C	W	36.587	-2.043	-0.298	-4.567	2.130	0.328	5.026	83.064	14.072	11.961
3001	154	25	18	C	W	37.311	-4.497	0.933	-2.116	4.941	-0.974	2.210	83.033	11.961	14.073
3002	154	25	18	C	W	43.463	-3.292	-2.630	-3.352	3.523	2.819	3.593	79.980	17.109	9.916
3003	154	25	18	C	W	44.392	-2.145	-1.049	-4.469	2.241	1.152	4.908	79.986	17.102	9.921
3004	154	25	18	C	W	46.102	-4.228	1.843	-2.397	4.619	-1.936	2.518	79.934	9.921	17.103
3005	154	25	18	C	W	73.898	-4.228	-1.843	-2.397	4.619	1.936	2.518	79.934	9.921	17.103
3006	154	25	18	C	W	75.609	-2.145	1.049	-4.469	2.241	-1.152	4.908	79.986	17.102	9.921
3007	154	25	18	C	W	76.537	-3.292	2.630	-3.352	3.523	-2.819	3.593	79.980	9.916	17.109
3008	154	25	18	C	W	82.689	-4.497	-0.933	-2.116	4.941	0.974	2.210	83.033	11.961	14.073
3009	154	25	18	C	W	83.413	-2.043	0.298	-4.567	2.130	-0.328	5.026	83.064	14.072	11.961

BL number	Atoms	γ-PC unit cells	WS_2 unit cells	γ-PC origin atom	WS_2 origin atom	Twist-angle (°)	γ-PC strain 1 (%)	γ-PC strain 2 (%)	γ-PC strain 3 (%)	WS_2 strain 1 (%)	WS_2 strain 2 (%)	WS_2 strain 3 (%)	γ (°)	|**a**|(Å)	|**b**|(Å)
3010	154	25	18	C	W	84.791	-3.388	2.629	-3.255	3.635	-2.813	3.482	83.076	11.953	14.078
3011	154	25	18	C	W	95.209	-3.388	-2.629	-3.255	3.635	2.813	3.482	83.076	14.078	11.953
3012	154	25	18	C	W	96.587	-2.043	-0.298	-4.567	2.130	0.328	5.026	83.064	14.072	11.961
3013	154	25	18	C	W	97.311	-4.497	0.933	-2.116	4.941	-0.974	2.210	83.033	11.961	14.073
3014	154	25	18	C	W	103.463	-3.292	-2.630	-3.352	3.523	2.819	3.593	79.980	17.109	9.916
3015	154	25	18	C	W	104.392	-2.145	-1.049	-4.469	2.241	1.152	4.908	79.986	17.102	9.921
3016	154	25	18	C	W	106.102	-4.228	1.843	-2.397	4.619	-1.936	2.518	79.934	9.921	17.103
3017	154	25	18	P	S	13.898	-4.228	-1.843	-2.397	4.619	1.936	2.518	79.934	9.921	17.103
3018	154	25	18	P	S	15.608	-2.145	1.049	-4.469	2.241	-1.152	4.908	79.986	17.102	9.921
3019	154	25	18	P	S	16.537	-3.292	2.630	-3.352	3.523	-2.819	3.593	79.980	9.916	17.109
3020	154	25	18	P	S	22.689	-4.497	-0.933	-2.116	4.941	0.974	2.210	83.033	11.961	14.073
3021	154	25	18	P	S	23.413	-2.043	0.298	-4.567	2.130	-0.328	5.026	83.064	14.072	11.961
3022	154	25	18	P	S	24.791	-3.388	2.629	-3.255	3.635	-2.813	3.482	83.076	11.953	14.078
3023	154	25	18	P	S	35.209	-3.388	-2.629	-3.255	3.635	2.813	3.482	83.076	14.078	11.953
3024	154	25	18	P	S	36.587	-2.043	-0.298	-4.567	2.130	0.328	5.026	83.064	14.072	11.961
3025	154	25	18	P	S	37.311	-4.497	0.933	-2.116	4.941	-0.974	2.210	83.033	11.961	14.073
3026	154	25	18	P	S	43.463	-3.292	-2.630	-3.352	3.523	2.819	3.593	79.980	17.109	9.916
3027	154	25	18	P	S	44.392	-2.145	-1.049	-4.469	2.241	1.152	4.908	79.986	17.102	9.921
3028	154	25	18	P	S	46.102	-4.228	1.843	-2.397	4.619	-1.936	2.518	79.934	9.921	17.103
3029	154	25	18	P	S	73.898	-4.228	-1.843	-2.397	4.619	1.936	2.518	79.934	9.921	17.103
3030	154	25	18	P	S	75.609	-2.145	1.049	-4.469	2.241	-1.152	4.908	79.986	17.102	9.921
3031	154	25	18	P	S	76.537	-3.292	2.630	-3.352	3.523	-2.819	3.593	79.980	9.916	17.109
3032	154	25	18	P	S	82.689	-4.497	-0.933	-2.116	4.941	0.974	2.210	83.033	11.961	14.073
3033	154	25	18	P	S	83.413	-2.043	0.298	-4.567	2.130	-0.328	5.026	83.064	14.072	11.961
3034	154	25	18	P	S	84.791	-3.388	2.629	-3.255	3.635	-2.813	3.482	83.076	11.953	14.078
3035	154	25	18	P	S	95.209	-3.388	-2.629	-3.255	3.635	2.813	3.482	83.076	14.078	11.953
3036	154	25	18	P	S	96.587	-2.043	-0.298	-4.567	2.130	0.328	5.026	83.064	14.072	11.961
3037	154	25	18	P	S	97.311	-4.497	0.933	-2.116	4.941	-0.974	2.210	83.033	11.961	14.073
3038	154	25	18	P	S	103.463	-3.292	-2.630	-3.352	3.523	2.819	3.593	79.980	17.109	9.916
3039	154	25	18	P	S	104.392	-2.145	-1.049	-4.469	2.241	1.152	4.908	79.986	17.102	9.921
3040	154	25	18	P	S	106.102	-4.228	1.843	-2.397	4.619	-1.936	2.518	79.934	9.921	17.103
3041	154	25	18	P	W	13.898	-4.228	-1.843	-2.397	4.619	1.936	2.518	79.934	9.921	17.103
3042	154	25	18	P	W	15.608	-2.145	1.049	-4.469	2.241	-1.152	4.908	79.986	17.102	9.921
3043	154	25	18	P	W	16.537	-3.292	2.630	-3.352	3.523	-2.819	3.593	79.980	9.916	17.109
3044	154	25	18	P	W	22.689	-4.497	-0.933	-2.116	4.941	0.974	2.210	83.033	11.961	14.073
3045	154	25	18	P	W	23.413	-2.043	0.298	-4.567	2.130	-0.328	5.026	83.064	14.072	11.961
3046	154	25	18	P	W	24.791	-3.388	2.629	-3.255	3.635	-2.813	3.482	83.076	11.953	14.078
3047	154	25	18	P	W	35.209	-3.388	-2.629	-3.255	3.635	2.813	3.482	83.076	14.078	11.953
3048	154	25	18	P	W	36.587	-2.043	-0.298	-4.567	2.130	0.328	5.026	83.064	14.072	11.961
3049	154	25	18	P	W	37.311	-4.497	0.933	-2.116	4.941	-0.974	2.210	83.033	11.961	14.073
3050	154	25	18	P	W	43.463	-3.292	-2.630	-3.352	3.523	2.819	3.593	79.980	17.109	9.916
3051	154	25	18	P	W	44.392	-2.145	-1.049	-4.469	2.241	1.152	4.908	79.986	17.102	9.921
3052	154	25	18	P	W	46.102	-4.228	1.843	-2.397	4.619	-1.936	2.518	79.934	9.921	17.103
3053	154	25	18	P	W	73.898	-4.228	-1.843	-2.397	4.619	1.936	2.518	79.934	9.921	17.103
3054	154	25	18	P	W	75.609	-2.145	1.049	-4.469	2.241	-1.152	4.908	79.986	17.102	9.921
3055	154	25	18	P	W	76.537	-3.292	2.630	-3.352	3.523	-2.819	3.593	79.980	9.916	17.109
3056	154	25	18	P	W	82.689	-4.497	-0.933	-2.116	4.941	0.974	2.210	83.033	11.961	14.073
3057	154	25	18	P	W	83.413	-2.043	0.298	-4.567	2.130	-0.328	5.026	83.064	14.072	11.961
3058	154	25	18	P	W	84.791	-3.388	2.629	-3.255	3.635	-2.813	3.482	83.076	11.953	14.078
3059	154	25	18	P	W	95.209	-3.388	-2.629	-3.255	3.635	2.813	3.482	83.076	14.078	11.953
3060	154	25	18	P	W	96.587	-2.043	-0.298	-4.567	2.130	0.328	5.026	83.064	14.072	11.961

BL number	Atoms	γ-PC unit cells	WS_2 unit cells	γ-PC origin atom	WS_2 origin atom	Twist-angle (°)	γ-PC strain 1 (%)	γ-PC strain 2 (%)	γ-PC strain 3 (%)	WS_2 strain 1 (%)	WS_2 strain 2 (%)	WS_2 strain 3 (%)	γ (°)	|**a**|(Å)	|**b**|(Å)
3061	154	25	18	P	W	97.311	-4.497	0.933	-2.116	4.941	-0.974	2.210	83.033	11.961	14.073
3062	154	25	18	P	W	103.463	-3.292	-2.630	-3.352	3.523	2.819	3.593	79.980	17.109	9.916
3063	154	25	18	P	W	104.392	-2.145	-1.049	-4.469	2.241	1.152	4.908	79.986	17.102	9.921
3064	154	25	18	P	W	106.102	-4.228	1.843	-2.397	4.619	-1.936	2.518	79.934	9.921	17.103
3065	155	23	21	C	S	27.796	5.011	5.272	0.227	-4.554	-5.248	-0.226	79.569	10.878	16.176
3066	155	23	21	C	S	32.204	5.011	-5.272	0.227	-4.554	5.248	-0.226	79.569	10.878	16.176
3067	155	23	21	C	S	87.796	5.011	5.272	0.227	-4.554	-5.248	-0.226	79.569	10.878	16.176
3068	155	23	21	C	S	92.204	5.011	-5.272	0.227	-4.554	5.248	-0.226	79.569	10.878	16.176
3069	155	23	21	C	W	27.796	5.011	5.272	0.227	-4.554	-5.248	-0.226	79.569	10.878	16.176
3070	155	23	21	C	W	32.204	5.011	-5.272	0.227	-4.554	5.248	-0.226	79.569	10.878	16.176
3071	155	23	21	C	W	87.796	5.011	5.272	0.227	-4.554	-5.248	-0.226	79.569	10.878	16.176
3072	155	23	21	C	W	92.204	5.011	-5.272	0.227	-4.554	5.248	-0.226	79.569	10.878	16.176
3073	155	23	21	P	S	27.796	5.011	5.272	0.227	-4.554	-5.248	-0.226	79.569	10.878	16.176
3074	155	23	21	P	S	32.204	5.011	-5.272	0.227	-4.554	5.248	-0.226	79.569	10.878	16.176
3075	155	23	21	P	S	87.796	5.011	5.272	0.227	-4.554	-5.248	-0.226	79.569	10.878	16.176
3076	155	23	21	P	S	92.204	5.011	-5.272	0.227	-4.554	5.248	-0.226	79.569	10.878	16.176
3077	155	23	21	P	W	27.796	5.011	5.272	0.227	-4.554	-5.248	-0.226	79.569	10.878	16.176
3078	155	23	21	P	W	32.204	5.011	-5.272	0.227	-4.554	5.248	-0.226	79.569	10.878	16.176
3079	155	23	21	P	W	87.796	5.011	5.272	0.227	-4.554	-5.248	-0.226	79.569	10.878	16.176
3080	155	23	21	P	W	92.204	5.011	-5.272	0.227	-4.554	5.248	-0.226	79.569	10.878	16.176
3081	156	24	20	C	S	0.000	5.011	2.526	-4.158	-4.554	-2.755	4.535	73.831	10.878	16.528
3082	156	24	20	C	S	8.948	1.048	-4.234	-0.599	-1.026	4.286	0.606	82.661	13.463	12.906
3083	156	24	20	C	S	10.893	-1.983	-0.884	2.519	2.064	0.842	-2.398	82.628	12.906	13.471
3084	156	24	20	C	S	12.520	2.326	1.943	-1.805	-2.223	-2.016	1.873	82.558	13.473	12.905
3085	156	24	20	C	S	13.898	-0.414	4.423	0.858	0.417	-4.348	-0.843	82.554	12.900	13.473
3086	156	24	20	C	S	19.107	-1.485	-3.047	1.980	1.530	2.931	-1.905	68.773	14.708	12.573
3087	156	24	20	C	S	23.413	0.481	4.532	-0.044	-0.477	-4.536	0.044	68.697	12.584	14.698
3088	156	24	20	C	S	30.000	-2.359	-4.698	2.934	2.476	4.437	-2.772	67.670	14.845	12.559
3089	156	24	20	C	S	36.587	0.481	-4.532	-0.044	-0.477	4.536	0.044	68.697	12.584	14.698
3090	156	24	20	C	S	40.893	1.980	2.944	-1.485	-1.905	-3.034	1.530	68.743	14.712	12.572
3091	156	24	20	C	S	46.102	-0.414	-4.423	0.858	0.417	4.348	-0.843	82.554	13.473	12.900
3092	156	24	20	C	S	47.480	2.326	-1.943	-1.805	-2.223	2.016	1.873	82.558	13.473	12.905
3093	156	24	20	C	S	49.107	-1.983	0.884	2.519	2.064	-0.842	-2.398	82.628	12.906	13.471
3094	156	24	20	C	S	49.842	2.281	2.109	-1.764	-2.182	-2.186	1.829	71.400	15.904	11.437
3095	156	24	20	C	S	51.052	-0.599	4.304	1.048	0.606	-4.216	-1.026	71.426	15.901	11.434
3096	156	24	20	C	S	60.000	5.011	2.526	-4.158	-4.554	-2.755	4.535	73.831	10.878	16.528
3097	156	24	20	C	S	68.948	1.048	-4.234	-0.599	-1.026	4.286	0.606	82.661	13.463	12.906
3098	156	24	20	C	S	70.158	2.281	-2.109	-1.764	-2.182	2.186	1.829	71.400	11.437	15.904
3099	156	24	20	C	S	70.893	-1.983	-0.884	2.519	2.064	0.842	-2.398	82.628	12.906	13.471
3100	156	24	20	C	S	72.520	2.326	1.943	-1.805	-2.223	-2.016	1.873	82.558	13.473	12.905
3101	156	24	20	C	S	73.898	-0.414	4.423	0.858	0.417	-4.348	-0.843	71.418	11.445	15.886
3102	156	24	20	C	S	79.107	1.980	-2.944	-1.485	-1.905	3.034	1.530	68.743	12.572	14.712
3103	156	24	20	C	S	83.413	0.481	4.532	-0.044	-0.477	-4.536	0.044	68.697	12.584	14.698
3104	156	24	20	C	S	90.000	-2.359	4.698	2.934	2.476	-4.437	-2.772	67.670	14.845	12.559
3105	156	24	20	C	S	96.587	0.481	-4.532	-0.044	-0.477	4.536	0.044	68.697	12.584	14.698
3106	156	24	20	C	S	100.893	-1.485	3.047	1.980	1.530	-2.931	-1.905	68.773	14.708	12.573
3107	156	24	20	C	S	106.102	-0.414	-4.423	0.858	0.417	4.348	-0.843	71.418	11.445	15.886
3108	156	24	20	C	S	107.480	2.326	-1.943	-1.805	-2.223	2.016	1.873	82.558	13.473	12.905
3109	156	24	20	C	S	109.107	-1.983	0.884	2.519	2.064	-0.842	-2.398	82.628	12.906	13.471
3110	156	24	20	C	S	111.052	1.048	4.234	-0.599	-1.027	-4.286	0.606	82.661	12.906	13.463
3111	156	24	20	C	W	0.000	5.011	2.526	-4.158	-4.554	-2.755	4.535	73.831	10.878	16.528

BL number	Atoms	γ-PC unit cells	WS_2 unit cells	γ-PC origin atom	WS_2 origin atom	Twist-angle (°)	γ-PC strain 1 (%)	γ-PC strain 2 (%)	γ-PC strain 3 (%)	WS_2 strain 1 (%)	WS_2 strain 2 (%)	WS_2 strain 3 (%)	γ (°)	|**a**|(Å)	|**b**|(Å)
3112	156	24	20	C	W	8.948	-0.599	-4.304	1.048	0.606	4.216	-1.026	71.426	15.901	11.434
3113	156	24	20	C	W	10.158	2.281	-2.109	-1.764	-2.182	2.186	1.829	71.400	11.437	15.904
3114	156	24	20	C	W	10.893	-1.983	-0.884	2.519	2.064	0.842	-2.398	82.628	12.906	13.471
3115	156	24	20	C	W	12.520	2.326	1.943	-1.805	-2.223	-2.016	1.873	82.558	13.473	12.905
3116	156	24	20	C	W	13.898	-0.414	4.423	0.858	0.417	-4.348	-0.843	82.554	12.900	13.473
3117	156	24	20	C	W	30.000	-2.359	-4.698	2.934	2.476	4.437	-2.772	67.670	14.845	12.559
3118	156	24	20	C	W	46.102	-0.414	-4.423	0.858	0.417	4.348	-0.843	82.554	13.473	12.900
3119	156	24	20	C	W	47.480	2.326	-1.943	-1.805	-2.223	2.016	1.873	82.558	13.473	12.905
3120	156	24	20	C	W	49.107	-1.983	0.884	2.519	2.064	-0.842	-2.398	82.628	12.906	13.471
3121	156	24	20	C	W	51.052	1.048	4.234	-0.599	-1.027	-4.286	0.606	82.661	12.906	13.463
3122	156	24	20	C	W	60.000	5.011	2.526	-4.158	-4.554	-2.755	4.535	73.831	10.878	16.528
3123	156	24	20	C	W	68.948	1.048	-4.234	-0.599	-1.026	4.286	0.606	82.661	13.463	12.906
3124	156	24	20	C	W	70.893	-1.983	-0.884	2.519	2.064	0.842	-2.398	82.628	12.906	13.471
3125	156	24	20	C	W	72.520	2.326	1.943	-1.805	-2.223	-2.016	1.873	82.558	13.473	12.905
3126	156	24	20	C	W	73.898	-0.414	4.423	0.858	0.417	-4.348	-0.843	82.554	12.900	13.473
3127	156	24	20	C	W	90.000	-2.359	4.698	2.934	2.476	-4.437	-2.772	67.670	14.845	12.559
3128	156	24	20	C	W	100.893	1.980	2.944	-1.485	-1.905	-3.034	1.530	68.743	14.712	12.572
3129	156	24	20	C	W	106.102	-0.414	-4.423	0.858	0.417	4.348	-0.843	82.554	13.473	12.900
3130	156	24	20	C	W	107.480	2.326	-1.943	-1.805	-2.223	2.016	1.873	82.558	13.473	12.905
3131	156	24	20	C	W	109.107	-1.983	0.884	2.519	2.064	-0.842	-2.398	82.628	12.906	13.471
3132	156	24	20	C	W	109.842	2.281	2.109	-1.764	-2.182	-2.186	1.829	71.400	15.904	11.437
3133	156	24	20	C	W	111.052	1.048	4.234	-0.599	-1.027	-4.286	0.606	82.661	12.906	13.463
3134	156	24	20	P	S	0.000	5.011	2.526	-4.158	-4.554	-2.755	4.535	73.831	10.878	16.528
3135	156	24	20	P	S	8.948	-0.599	-4.304	1.048	0.606	4.216	-1.026	71.426	15.901	11.434
3136	156	24	20	P	S	13.898	-0.414	4.423	0.858	0.417	-4.348	-0.843	71.418	11.445	15.886
3137	156	24	20	P	S	19.107	-1.485	-3.047	1.980	1.530	2.931	-1.905	68.773	14.708	12.573
3138	156	24	20	P	S	23.413	0.481	4.532	-0.044	-0.477	-4.536	0.044	68.697	12.584	14.698
3139	156	24	20	P	S	30.000	-2.359	-4.698	2.934	2.476	4.437	-2.772	67.670	14.845	12.559
3140	156	24	20	P	S	36.587	0.481	-4.532	-0.044	-0.477	4.536	0.044	68.697	12.584	14.698
3141	156	24	20	P	S	40.893	1.980	2.944	-1.485	-1.905	-3.034	1.530	68.743	14.712	12.572
3142	156	24	20	P	S	46.102	-0.414	-4.423	0.858	0.417	4.348	-0.843	82.554	13.473	12.900
3143	156	24	20	P	S	47.480	2.326	-1.943	-1.805	-2.223	2.016	1.873	82.558	13.473	12.905
3144	156	24	20	P	S	49.107	-1.983	0.884	2.519	2.064	-0.842	-2.398	82.628	12.906	13.471
3145	156	24	20	P	S	51.052	-0.599	4.304	1.048	0.606	-4.216	-1.026	71.426	15.901	11.434
3146	156	24	20	P	S	60.000	5.011	2.526	-4.158	-4.554	-2.755	4.535	73.831	10.878	16.528
3147	156	24	20	P	S	68.948	1.048	-4.234	-0.599	-1.026	4.286	0.606	82.661	13.463	12.906
3148	156	24	20	P	S	70.158	2.281	-2.109	-1.764	-2.182	2.186	1.829	71.400	11.437	15.904
3149	156	24	20	P	S	70.893	-1.983	-0.884	2.519	2.064	0.842	-2.398	82.628	12.906	13.471
3150	156	24	20	P	S	72.520	2.326	1.943	-1.805	-2.223	-2.016	1.873	82.558	13.473	12.905
3151	156	24	20	P	S	73.898	-0.414	4.423	0.858	0.417	-4.348	-0.843	71.418	11.445	15.886
3152	156	24	20	P	S	79.107	1.980	-2.944	-1.485	-1.905	3.034	1.530	68.743	12.572	14.712
3153	156	24	20	P	S	83.413	0.481	4.532	-0.044	-0.477	-4.536	0.044	68.697	12.584	14.698
3154	156	24	20	P	S	90.000	-2.359	4.698	2.934	2.476	-4.437	-2.772	67.670	14.845	12.559
3155	156	24	20	P	S	96.587	0.481	-4.532	-0.044	-0.477	4.536	0.044	68.697	12.584	14.698
3156	156	24	20	P	S	100.893	-1.485	3.047	1.980	1.530	-2.931	-1.905	68.773	14.708	12.573
3157	156	24	20	P	S	106.102	-0.414	-4.423	0.858	0.417	4.348	-0.843	71.418	11.445	15.886
3158	156	24	20	P	S	109.842	2.281	2.109	-1.764	-2.182	-2.186	1.829	71.400	15.904	11.437
3159	156	24	20	P	S	111.052	-0.599	4.304	1.048	0.606	-4.216	-1.026	71.426	15.901	11.434
3160	156	24	20	P	W	0.000	5.011	2.526	-4.158	-4.554	-2.755	4.535	73.831	10.878	16.528
3161	156	24	20	P	W	8.948	1.048	-4.234	-0.599	-1.026	4.286	0.606	82.661	13.463	12.906
3162	156	24	20	P	W	10.158	2.281	-2.109	-1.764	-2.182	2.186	1.829	71.400	11.437	15.904

BL number	Atoms	γ-PC unit cells	WS_2 unit cells	γ-PC origin atom	WS_2 origin atom	Twist-angle (°)	γ-PC strain 1 (%)	γ-PC strain 2 (%)	γ-PC strain 3 (%)	WS_2 strain 1 (%)	WS_2 strain 2 (%)	WS_2 strain 3 (%)	γ (°)	|**a**|(Å)	|**b**|(Å)
3163	156	24	20	P	W	10.893	-1.983	-0.884	2.519	2.064	0.842	-2.398	82.628	12.906	13.471
3164	156	24	20	P	W	12.520	2.326	1.943	-1.805	-2.223	-2.016	1.873	82.558	13.473	12.905
3165	156	24	20	P	W	13.898	-0.414	4.423	0.858	0.417	-4.348	-0.843	82.554	12.900	13.473
3166	156	24	20	P	W	30.000	-2.359	-4.698	2.934	2.476	4.437	-2.772	67.670	14.845	12.559
3167	156	24	20	P	W	49.842	2.281	2.109	-1.764	-2.182	-2.186	1.829	71.400	15.904	11.437
3168	156	24	20	P	W	60.000	5.011	2.526	-4.158	-4.554	-2.755	4.535	73.831	10.878	16.528
3169	156	24	20	P	W	68.948	-0.599	-4.304	1.048	0.606	4.216	-1.026	71.426	15.901	11.434
3170	156	24	20	P	W	79.107	1.980	-2.944	-1.485	-1.905	3.034	1.530	68.743	12.572	14.712
3171	156	24	20	P	W	90.000	-2.359	4.698	2.934	2.476	-4.437	-2.772	67.670	14.845	12.559
3172	156	24	20	P	W	106.102	-0.414	-4.423	0.858	0.417	4.348	-0.843	82.554	13.473	12.900
3173	156	24	20	P	W	107.480	2.326	-1.943	-1.805	-2.223	2.016	1.873	82.558	13.473	12.905
3174	156	24	20	P	W	109.107	-1.983	0.884	2.519	2.064	-0.842	-2.398	82.628	12.906	13.471
3175	156	24	20	P	W	111.052	1.048	4.234	-0.599	-1.027	-4.286	0.606	82.661	12.906	13.463
3176	157	25	19	C	S	23.413	-2.043	0.000	-2.042	2.130	0.000	2.130	60.000	14.072	14.072
3177	157	25	19	C	S	36.587	-2.043	0.000	-2.042	2.130	0.000	2.130	60.000	14.072	14.072
3178	157	25	19	C	S	83.413	-2.043	0.000	-2.042	2.130	0.000	2.130	60.000	14.072	14.072
3179	157	25	19	C	S	96.587	-2.043	0.000	-2.042	2.130	0.000	2.130	60.000	14.072	14.072
3180	157	25	19	C	W	23.413	-2.043	0.000	-2.042	2.130	0.000	2.130	60.000	14.072	14.072
3181	157	25	19	C	W	36.587	-2.043	0.000	-2.042	2.130	0.000	2.130	60.000	14.072	14.072
3182	157	25	19	C	W	83.413	-2.043	0.000	-2.042	2.130	0.000	2.130	60.000	14.072	14.072
3183	157	25	19	C	W	96.587	-2.043	0.000	-2.042	2.130	0.000	2.130	60.000	14.072	14.072
3184	157	25	19	P	S	36.587	-2.043	0.000	-2.042	2.130	0.000	2.130	60.000	14.072	14.072
3185	157	25	19	P	S	83.413	-2.043	0.000	-2.042	2.130	0.000	2.130	60.000	14.072	14.072
3186	157	25	19	P	W	23.413	-2.043	0.000	-2.042	2.130	0.000	2.130	60.000	14.072	14.072
3187	157	25	19	P	W	96.587	-2.043	0.000	-2.042	2.130	0.000	2.130	60.000	14.072	14.072
3188	157	25	19	C	S	7.154	-0.955	-4.648	-3.106	0.973	4.956	3.312	84.160	14.422	11.956
3189	157	25	19	C	S	9.515	-4.497	-0.679	0.544	4.941	0.671	-0.538	84.123	11.961	14.424
3190	157	25	19	C	S	10.893	0.418	1.656	-4.383	-0.415	-1.816	4.805	84.066	14.426	11.960
3191	157	25	19	C	S	49.107	0.418	-1.656	-4.383	-0.415	1.816	4.805	84.066	14.426	11.960
3192	157	25	19	C	S	50.485	-4.497	0.679	0.544	4.941	-0.671	-0.538	84.123	11.961	14.424
3193	157	25	19	C	S	52.846	-0.955	4.648	-3.106	0.973	-4.956	3.312	84.160	11.956	14.422
3194	157	25	19	C	S	67.154	-0.955	-4.648	-3.106	0.973	4.956	3.312	84.160	14.422	11.956
3195	157	25	19	C	S	69.515	-4.497	-0.679	0.544	4.941	0.671	-0.538	84.123	11.961	14.424
3196	157	25	19	C	S	70.893	0.418	1.656	-4.383	-0.415	-1.816	4.805	84.066	14.426	11.960
3197	157	25	19	C	S	109.107	0.418	-1.656	-4.383	-0.415	1.816	4.805	84.066	14.426	11.960
3198	157	25	19	C	S	110.485	-4.497	0.679	0.544	4.941	-0.671	-0.538	84.123	11.961	14.424
3199	157	25	19	C	S	112.846	-0.955	4.648	-3.106	0.973	-4.956	3.312	84.160	11.956	14.422
3200	157	25	19	C	W	7.154	-0.955	-4.648	-3.106	0.973	4.956	3.312	84.160	14.422	11.956
3201	157	25	19	C	W	9.515	-4.497	-0.679	0.544	4.941	0.671	-0.538	84.123	11.961	14.424
3202	157	25	19	C	W	10.893	0.418	1.656	-4.383	-0.415	-1.816	4.805	84.066	14.426	11.960
3203	157	25	19	C	W	49.107	0.418	-1.656	-4.383	-0.415	1.816	4.805	84.066	14.426	11.960
3204	157	25	19	C	W	50.485	-4.497	0.679	0.544	4.941	-0.671	-0.538	84.123	11.961	14.424
3205	157	25	19	C	W	52.846	-0.955	4.648	-3.106	0.973	-4.956	3.312	84.160	11.956	14.422
3206	157	25	19	C	W	67.154	-0.955	-4.648	-3.106	0.973	4.956	3.312	84.160	14.422	11.956
3207	157	25	19	C	W	69.515	-4.497	-0.679	0.544	4.941	0.671	-0.538	84.123	11.961	14.424
3208	157	25	19	C	W	70.893	0.418	1.656	-4.383	-0.415	-1.816	4.805	84.066	14.426	11.960
3209	157	25	19	C	W	109.107	0.418	-1.656	-4.383	-0.415	1.816	4.805	84.066	14.426	11.960
3210	157	25	19	C	W	110.485	-4.497	0.679	0.544	4.941	-0.671	-0.538	84.123	11.961	14.424
3211	157	25	19	C	W	112.846	-0.955	4.648	-3.106	0.973	-4.956	3.312	84.160	11.956	14.422
3212	157	25	19	P	S	7.154	-0.955	-4.648	-3.106	0.973	4.956	3.312	84.160	14.422	11.956
3213	157	25	19	P	S	9.515	-4.497	-0.679	0.544	4.941	0.671	-0.538	84.123	11.961	14.424

BL number	Atoms	γ-PC unit cells	WS_2 unit cells	γ-PC origin atom	WS_2 origin atom	Twist-angle (°)	γ-PC strain 1 (%)	γ-PC strain 2 (%)	γ-PC strain 3 (%)	WS_2 strain 1 (%)	WS_2 strain 2 (%)	WS_2 strain 3 (%)	γ (°)	|**a**|(Å)	|**b**|(Å)
3214	157	25	19	P	S	10.893	0.418	1.656	-4.383	-0.415	-1.816	4.805	84.066	14.426	11.960
3215	157	25	19	P	S	49.107	0.418	-1.656	-4.383	-0.415	1.816	4.805	84.066	14.426	11.960
3216	157	25	19	P	S	50.485	-4.497	0.679	0.544	4.941	-0.671	-0.538	84.123	11.961	14.424
3217	157	25	19	P	S	52.846	-0.955	4.648	-3.106	0.973	-4.956	3.312	84.160	11.956	14.422
3218	157	25	19	P	S	67.154	-0.955	-4.648	-3.106	0.973	4.956	3.312	84.160	14.422	11.956
3219	157	25	19	P	S	69.515	-4.497	-0.679	0.544	4.941	0.671	-0.538	84.123	11.961	14.424
3220	157	25	19	P	S	70.893	0.418	1.656	-4.383	-0.415	-1.816	4.805	84.066	14.426	11.960
3221	157	25	19	P	S	109.107	0.418	-1.656	-4.383	-0.415	1.816	4.805	84.066	14.426	11.960
3222	157	25	19	P	S	110.485	-4.497	0.679	0.544	4.941	-0.671	-0.538	84.123	11.961	14.424
3223	157	25	19	P	S	112.846	-0.955	4.648	-3.106	0.973	-4.956	3.312	84.160	11.956	14.422
3224	157	25	19	P	W	7.154	-0.955	-4.648	-3.106	0.973	4.956	3.312	84.160	14.422	11.956
3225	157	25	19	P	W	9.515	-4.497	-0.679	0.544	4.941	0.671	-0.538	84.123	11.961	14.424
3226	157	25	19	P	W	10.893	0.418	1.656	-4.383	-0.415	-1.816	4.805	84.066	14.426	11.960
3227	157	25	19	P	W	49.107	0.418	-1.656	-4.383	-0.415	1.816	4.805	84.066	14.426	11.960
3228	157	25	19	P	W	50.485	-4.497	0.679	0.544	4.941	-0.671	-0.538	84.123	11.961	14.424
3229	157	25	19	P	W	52.846	-0.955	4.648	-3.106	0.973	-4.956	3.312	84.160	11.956	14.422
3230	157	25	19	P	W	67.154	-0.955	-4.648	-3.106	0.973	4.956	3.312	84.160	14.422	11.956
3231	157	25	19	P	W	69.515	-4.497	-0.679	0.544	4.941	0.671	-0.538	84.123	11.961	14.424
3232	157	25	19	P	W	70.893	0.418	1.656	-4.383	-0.415	-1.816	4.805	84.066	14.426	11.960
3233	157	25	19	P	W	109.107	0.418	-1.656	-4.383	-0.415	1.816	4.805	84.066	14.426	11.960
3234	157	25	19	P	W	110.485	-4.497	0.679	0.544	4.941	-0.671	-0.538	84.123	11.961	14.424
3235	157	25	19	P	W	112.846	-0.955	4.648	-3.106	0.973	-4.956	3.312	84.160	11.956	14.422
3236	158	23	22	C	S	16.102	2.853	-1.936	4.768	-2.699	1.768	-4.353	81.935	10.655	16.796
3237	158	23	22	C	S	18.613	3.582	2.635	4.023	-3.342	-2.439	-3.723	81.955	10.656	16.792
3238	158	23	22	C	S	27.796	5.011	-3.954	2.619	-4.554	3.757	-2.489	84.979	10.862	16.376
3239	158	23	22	C	S	28.055	2.281	-3.576	5.366	-2.182	3.229	-4.846	84.977	16.362	10.872
3240	158	23	22	C	S	31.945	2.281	3.576	5.366	-2.182	-3.229	-4.846	84.977	16.362	10.872
3241	158	23	22	C	S	32.204	5.011	3.954	2.619	-4.554	-3.757	-2.489	84.863	10.878	16.354
3242	158	23	22	C	S	41.387	3.582	-2.635	4.023	-3.342	2.439	-3.723	81.955	16.792	10.656
3243	158	23	22	C	S	42.103	5.011	-1.318	2.619	-4.554	1.252	-2.489	81.958	16.799	10.653
3244	158	23	22	C	S	43.898	2.853	1.937	4.768	-2.699	-1.768	-4.353	81.935	10.655	16.796
3245	158	23	22	C	S	76.102	2.853	-1.936	4.768	-2.699	1.768	-4.353	81.935	10.655	16.796
3246	158	23	22	C	S	77.897	5.011	1.318	2.619	-4.555	-1.252	-2.489	81.958	16.799	10.653
3247	158	23	22	C	S	78.613	3.582	2.635	4.023	-3.342	-2.439	-3.723	81.955	10.656	16.792
3248	158	23	22	C	S	87.796	5.011	-3.954	2.619	-4.554	3.757	-2.489	84.863	10.878	16.354
3249	158	23	22	C	S	88.055	2.281	-3.576	5.366	-2.182	3.229	-4.846	84.977	16.362	10.872
3250	158	23	22	C	S	91.945	2.281	3.576	5.366	-2.182	-3.229	-4.846	84.977	16.362	10.872
3251	158	23	22	C	S	92.204	5.011	3.954	2.619	-4.554	-3.757	-2.489	84.979	16.376	10.862
3252	158	23	22	C	S	101.387	3.582	-2.635	4.023	-3.342	2.439	-3.723	81.955	16.792	10.656
3253	158	23	22	C	S	102.104	5.011	-1.318	2.619	-4.554	1.252	-2.489	81.959	16.799	10.653
3254	158	23	22	C	W	16.102	2.853	-1.936	4.768	-2.699	1.768	-4.353	81.935	10.655	16.796
3255	158	23	22	C	W	17.897	5.011	1.318	2.619	-4.555	-1.252	-2.489	81.959	16.799	10.653
3256	158	23	22	C	W	18.613	3.582	2.635	4.023	-3.342	-2.439	-3.723	81.955	10.656	16.792
3257	158	23	22	C	W	27.796	5.011	-3.954	2.619	-4.554	3.757	-2.489	84.979	10.862	16.376
3258	158	23	22	C	W	28.055	2.281	-3.576	5.366	-2.182	3.229	-4.846	84.977	16.362	10.872
3259	158	23	22	C	W	31.945	2.281	3.576	5.366	-2.182	-3.229	-4.846	84.977	16.362	10.872
3260	158	23	22	C	W	32.204	5.011	3.954	2.619	-4.554	-3.757	-2.489	84.863	10.878	16.354
3261	158	23	22	C	W	41.387	3.582	-2.635	4.023	-3.342	2.439	-3.723	81.955	16.792	10.656
3262	158	23	22	C	W	42.103	5.011	-1.318	2.619	-4.554	1.252	-2.489	81.958	16.799	10.653
3263	158	23	22	C	W	76.102	2.853	-1.936	4.768	-2.699	1.768	-4.353	81.935	10.655	16.796
3264	158	23	22	C	W	78.613	3.582	2.635	4.023	-3.342	-2.439	-3.723	81.955	10.656	16.792

BL number	Atoms	γ-PC unit cells	WS_2 unit cells	γ-PC origin atom	WS_2 origin atom	Twist-angle (°)	γ-PC strain 1 (%)	γ-PC strain 2 (%)	γ-PC strain 3 (%)	WS_2 strain 1 (%)	WS_2 strain 2 (%)	WS_2 strain 3 (%)	γ (°)	|**a**|(Å)	|**b**|(Å)
3265	158	23	22	C	W	87.796	5.011	-3.954	2.619	-4.554	3.757	-2.489	84.863	10.878	16.354
3266	158	23	22	C	W	88.055	2.281	-3.576	5.366	-2.182	3.229	-4.846	84.977	16.362	10.872
3267	158	23	22	C	W	91.945	2.281	3.576	5.366	-2.182	-3.229	-4.846	84.977	16.362	10.872
3268	158	23	22	C	W	92.204	5.011	3.954	2.619	-4.554	-3.757	-2.489	84.979	16.376	10.862
3269	158	23	22	C	W	101.387	3.582	-2.635	4.023	-3.342	2.439	-3.723	81.955	16.792	10.656
3270	158	23	22	C	W	102.104	5.011	-1.318	2.619	-4.554	1.252	-2.489	81.959	16.799	10.653
3271	158	23	22	C	W	103.898	2.853	1.937	4.768	-2.699	-1.768	-4.353	81.935	10.655	16.796
3272	158	23	22	P	S	16.102	2.853	-1.936	4.768	-2.699	1.768	-4.353	81.935	10.655	16.796
3273	158	23	22	P	S	17.897	5.011	1.318	2.619	-4.555	-1.252	-2.489	81.959	16.799	10.653
3274	158	23	22	P	S	18.613	3.582	2.635	4.023	-3.342	-2.439	-3.723	81.955	10.656	16.792
3275	158	23	22	P	S	27.796	5.011	-3.954	2.619	-4.554	3.757	-2.489	84.979	10.862	16.376
3276	158	23	22	P	S	28.055	2.281	-3.576	5.366	-2.182	3.229	-4.846	84.977	16.362	10.872
3277	158	23	22	P	S	32.204	5.011	3.954	2.619	-4.554	-3.757	-2.489	84.863	10.878	16.354
3278	158	23	22	P	S	41.387	3.582	-2.635	4.023	-3.342	2.439	-3.723	81.955	16.792	10.656
3279	158	23	22	P	S	42.103	5.011	-1.318	2.619	-4.554	1.252	-2.489	81.958	16.799	10.653
3280	158	23	22	P	S	76.102	2.853	-1.936	4.768	-2.699	1.768	-4.353	81.935	10.655	16.796
3281	158	23	22	P	S	78.613	3.582	2.635	4.023	-3.342	-2.439	-3.723	81.955	10.656	16.792
3282	158	23	22	P	S	87.796	5.011	-3.954	2.619	-4.554	3.757	-2.489	84.863	10.878	16.354
3283	158	23	22	P	S	91.945	2.281	3.576	5.366	-2.182	-3.229	-4.846	84.977	16.362	10.872
3284	158	23	22	P	S	92.204	5.011	3.954	2.619	-4.554	-3.757	-2.489	84.979	16.376	10.862
3285	158	23	22	P	S	101.387	3.582	-2.635	4.023	-3.342	2.439	-3.723	81.955	16.792	10.656
3286	158	23	22	P	S	102.104	5.011	-1.318	2.619	-4.554	1.252	-2.489	81.959	16.799	10.653
3287	158	23	22	P	S	103.898	2.853	1.937	4.768	-2.699	-1.768	-4.353	81.935	10.655	16.796
3288	158	23	22	P	W	16.102	2.853	-1.936	4.768	-2.699	1.768	-4.353	81.935	10.655	16.796
3289	158	23	22	P	W	18.613	3.582	2.635	4.023	-3.342	-2.439	-3.723	81.955	10.656	16.792
3290	158	23	22	P	W	27.796	5.011	-3.954	2.619	-4.554	3.757	-2.489	84.863	10.878	16.354
3291	158	23	22	P	W	31.945	2.281	3.576	5.366	-2.182	-3.229	-4.846	84.977	16.362	10.872
3292	158	23	22	P	W	32.204	5.011	3.954	2.619	-4.554	-3.757	-2.489	84.979	16.376	10.862
3293	158	23	22	P	W	41.387	3.582	-2.635	4.023	-3.342	2.439	-3.723	81.955	16.792	10.656
3294	158	23	22	P	W	42.103	5.011	-1.318	2.619	-4.554	1.252	-2.489	81.958	16.799	10.653
3295	158	23	22	P	W	43.898	2.853	1.937	4.768	-2.699	-1.768	-4.353	81.935	10.655	16.796
3296	158	23	22	P	W	76.102	2.853	-1.936	4.768	-2.699	1.768	-4.353	81.935	10.655	16.796
3297	158	23	22	P	W	77.897	5.011	1.318	2.619	-4.555	-1.252	-2.489	81.958	16.799	10.653
3298	158	23	22	P	W	78.613	3.582	2.635	4.023	-3.342	-2.439	-3.723	81.955	10.656	16.792
3299	158	23	22	P	W	87.796	5.011	-3.954	2.619	-4.554	3.757	-2.489	84.863	10.878	16.354
3300	158	23	22	P	W	88.055	2.281	-3.576	5.366	-2.182	3.229	-4.846	84.977	16.362	10.872
3301	158	23	22	P	W	92.204	5.011	3.954	2.619	-4.554	-3.757	-2.489	84.863	10.878	16.354
3302	158	23	22	P	W	101.387	3.582	-2.635	4.023	-3.342	2.439	-3.723	81.955	16.792	10.656
3303	158	23	22	P	W	102.104	5.011	-1.318	2.619	-4.554	1.252	-2.489	81.959	16.799	10.653
3304	158	26	18	C	S	4.307	-4.685	-4.345	-3.767	5.169	4.699	4.074	80.085	14.491	11.944
3305	158	26	18	C	S	9.515	-4.497	4.405	-3.958	4.941	-4.783	4.298	80.076	11.961	14.471
3306	158	26	18	C	S	50.485	-4.497	-4.405	-3.958	4.941	4.783	4.298	80.076	11.961	14.471
3307	158	26	18	C	S	55.693	-4.685	4.344	-3.767	5.169	-4.698	4.074	80.085	14.491	11.944
3308	158	26	18	C	S	64.307	-4.685	-4.345	-3.767	5.169	4.699	4.074	80.085	14.491	11.944
3309	158	26	18	C	S	69.515	-4.497	4.405	-3.958	4.941	-4.783	4.298	80.077	11.961	14.471
3310	158	26	18	C	S	110.485	-4.497	-4.405	-3.958	4.941	4.783	4.298	80.076	11.961	14.471
3311	158	26	18	C	S	115.693	-4.685	4.344	-3.767	5.169	-4.698	4.074	80.085	14.491	11.944
3312	158	26	18	C	W	4.307	-4.685	-4.345	-3.767	5.169	4.699	4.074	80.085	14.491	11.944
3313	158	26	18	C	W	9.515	-4.497	4.405	-3.958	4.941	-4.783	4.298	80.076	11.961	14.471
3314	158	26	18	C	W	50.485	-4.497	-4.405	-3.958	4.941	4.783	4.298	80.076	11.961	14.471
3315	158	26	18	C	W	55.693	-4.685	4.344	-3.767	5.169	-4.698	4.074	80.085	14.491	11.944

BL number	Atoms	γ-PC unit cells	WS_2 unit cells	γ-PC origin atom	WS_2 origin atom	Twist-angle (°)	γ-PC strain 1 (%)	γ-PC strain 2 (%)	γ-PC strain 3 (%)	WS_2 strain 1 (%)	WS_2 strain 2 (%)	WS_2 strain 3 (%)	γ (°)	|**a**|(Å)	|**b**|(Å)
3316	158	26	18	C	W	64.307	-4.685	-4.345	-3.767	5.169	4.699	4.074	80.085	14.491	11.944
3317	158	26	18	C	W	69.515	-4.497	4.405	-3.958	4.941	-4.783	4.298	80.077	11.961	14.471
3318	158	26	18	C	W	110.485	-4.497	-4.405	-3.958	4.941	4.783	4.298	80.076	11.961	14.471
3319	158	26	18	C	W	115.693	-4.685	4.344	-3.767	5.169	-4.698	4.074	80.085	14.491	11.944
3320	158	26	18	P	S	4.307	-4.685	-4.345	-3.767	5.169	4.699	4.074	80.085	14.491	11.944
3321	158	26	18	P	S	115.693	-4.685	4.344	-3.767	5.169	-4.698	4.074	80.085	14.491	11.944
3322	158	26	18	P	W	55.693	-4.685	4.344	-3.767	5.169	-4.698	4.074	80.085	14.491	11.944
3323	159	24	21	C	S	8.948	1.048	-5.175	1.871	-1.026	4.989	-1.804	77.663	15.808	11.437
3324	159	24	21	C	S	10.893	4.020	-1.668	-0.982	-3.721	1.702	1.002	77.646	15.815	11.440
3325	159	24	21	C	S	13.898	-0.414	3.686	3.401	0.417	-3.451	-3.184	77.538	11.445	15.810
3326	159	24	21	C	S	17.897	5.011	1.263	-1.865	-4.554	-1.312	1.938	72.523	16.210	11.436
3327	159	24	21	C	S	27.796	5.011	-3.789	-1.865	-4.554	3.936	1.938	75.204	18.813	9.721
3328	159	24	21	C	S	32.204	5.011	3.789	-1.865	-4.554	-3.936	1.938	75.204	9.721	18.813
3329	159	24	21	C	S	42.103	5.011	-1.263	-1.865	-4.554	1.312	1.938	72.523	11.436	16.210
3330	159	24	21	C	S	46.102	-0.414	-3.685	3.401	0.417	3.451	-3.184	77.538	11.445	15.810
3331	159	24	21	C	S	49.107	4.020	1.668	-0.982	-3.721	-1.702	1.002	77.646	15.815	11.440
3332	159	24	21	C	S	51.052	1.048	5.175	1.871	-1.026	-4.989	-1.804	77.663	11.437	15.808
3333	159	24	21	C	S	68.948	1.048	-5.175	1.871	-1.026	4.989	-1.804	77.664	15.808	11.437
3334	159	24	21	C	S	70.893	4.020	-1.668	-0.982	-3.721	1.702	1.002	77.646	15.815	11.440
3335	159	24	21	C	S	73.898	-0.414	3.686	3.401	0.417	-3.451	-3.184	77.538	11.445	15.810
3336	159	24	21	C	S	77.897	5.011	1.263	-1.865	-4.554	-1.312	1.938	72.523	16.210	11.436
3337	159	24	21	C	S	87.796	5.011	-3.789	-1.865	-4.554	3.936	1.938	75.204	18.813	9.721
3338	159	24	21	C	S	102.104	5.011	-1.263	-1.865	-4.554	1.312	1.938	72.523	11.436	16.210
3339	159	24	21	C	S	106.102	-0.414	-3.685	3.401	0.417	3.451	-3.184	77.538	11.445	15.810
3340	159	24	21	C	S	109.107	4.020	1.668	-0.982	-3.721	-1.702	1.002	77.646	15.815	11.440
3341	159	24	21	C	S	111.052	1.048	5.175	1.871	-1.026	-4.989	-1.804	77.664	11.437	15.808
3342	159	24	21	C	W	8.948	1.048	-5.175	1.871	-1.026	4.989	-1.804	77.663	15.808	11.437
3343	159	24	21	C	W	10.893	4.020	-1.668	-0.982	-3.721	1.702	1.002	77.646	15.815	11.440
3344	159	24	21	C	W	13.898	-0.414	3.686	3.401	0.417	-3.451	-3.184	77.538	11.445	15.810
3345	159	24	21	C	W	17.897	5.011	1.263	-1.865	-4.554	-1.312	1.938	72.523	16.210	11.436
3346	159	24	21	C	W	42.103	5.011	-1.263	-1.865	-4.554	1.312	1.938	72.523	11.436	16.210
3347	159	24	21	C	W	46.102	-0.414	-3.685	3.401	0.417	3.451	-3.184	77.538	11.445	15.810
3348	159	24	21	C	W	49.107	4.020	1.668	-0.982	-3.721	-1.702	1.002	77.646	15.815	11.440
3349	159	24	21	C	W	51.052	1.048	5.175	1.871	-1.026	-4.989	-1.804	77.663	11.437	15.808
3350	159	24	21	C	W	68.948	1.048	-5.175	1.871	-1.026	4.989	-1.804	77.664	15.808	11.437
3351	159	24	21	C	W	70.893	4.020	-1.668	-0.982	-3.721	1.702	1.002	77.646	15.815	11.440
3352	159	24	21	C	W	73.898	-0.414	3.686	3.401	0.417	-3.451	-3.184	77.538	11.445	15.810
3353	159	24	21	C	W	77.897	5.011	1.263	-1.865	-4.554	-1.312	1.938	72.523	16.210	11.436
3354	159	24	21	C	W	92.204	5.011	3.789	-1.865	-4.554	-3.936	1.938	75.204	9.721	18.813
3355	159	24	21	C	W	102.104	5.011	-1.263	-1.865	-4.554	1.312	1.938	72.523	11.436	16.210
3356	159	24	21	C	W	106.102	-0.414	-3.685	3.401	0.417	3.451	-3.184	77.538	11.445	15.810
3357	159	24	21	C	W	109.107	4.020	1.668	-0.982	-3.721	-1.702	1.002	77.646	15.815	11.440
3358	159	24	21	C	W	111.052	1.048	5.175	1.871	-1.026	-4.989	-1.804	77.664	11.437	15.808
3359	159	24	21	P	S	8.948	1.048	-5.175	1.871	-1.026	4.989	-1.804	77.663	15.808	11.437
3360	159	24	21	P	S	10.893	4.020	-1.668	-0.982	-3.721	1.702	1.002	77.646	15.815	11.440
3361	159	24	21	P	S	13.898	-0.414	3.686	3.401	0.417	-3.451	-3.184	77.538	11.445	15.810
3362	159	24	21	P	S	17.897	5.011	1.263	-1.865	-4.554	-1.312	1.938	72.523	16.210	11.436
3363	159	24	21	P	S	42.103	5.011	-1.263	-1.865	-4.554	1.312	1.938	72.523	11.436	16.210
3364	159	24	21	P	S	46.102	-0.414	-3.685	3.401	0.417	3.451	-3.184	77.538	11.445	15.810
3365	159	24	21	P	S	49.107	4.020	1.668	-0.982	-3.721	-1.702	1.002	77.646	15.815	11.440
3366	159	24	21	P	S	51.052	1.048	5.175	1.871	-1.026	-4.989	-1.804	77.663	11.437	15.808

BL number	Atoms	γ-PC unit cells	WS_2 unit cells	γ-PC origin atom	WS_2 origin atom	Twist-angle (°)	γ-PC strain 1 (%)	γ-PC strain 2 (%)	γ-PC strain 3 (%)	WS_2 strain 1 (%)	WS_2 strain 2 (%)	WS_2 strain 3 (%)	γ (°)	$\lvert\mathbf{a}\rvert$(Å)	$\lvert\mathbf{b}\rvert$(Å)
3367	159	24	21	P	S	68.948	1.048	-5.175	1.871	-1.026	4.989	-1.804	77.664	15.808	11.437
3368	159	24	21	P	S	70.893	4.020	-1.668	-0.982	-3.721	1.702	1.002	77.646	15.815	11.440
3369	159	24	21	P	S	73.898	-0.414	3.686	3.401	0.417	-3.451	-3.184	77.538	11.445	15.810
3370	159	24	21	P	S	77.897	5.011	1.263	-1.865	-4.554	-1.312	1.938	72.523	16.210	11.436
3371	159	24	21	P	S	102.104	5.011	-1.263	-1.865	-4.554	1.312	1.938	72.523	11.436	16.210
3372	159	24	21	P	S	106.102	-0.414	-3.685	3.401	0.417	3.451	-3.184	77.538	11.445	15.810
3373	159	24	21	P	S	109.107	4.020	1.668	-0.982	-3.721	-1.702	1.002	77.646	15.815	11.440
3374	159	24	21	P	S	111.052	1.048	5.175	1.871	-1.026	-4.989	-1.804	77.664	11.437	15.808
3375	159	24	21	P	W	8.948	1.048	-5.175	1.871	-1.026	4.989	-1.804	77.663	15.808	11.437
3376	159	24	21	P	W	10.893	4.020	-1.668	-0.982	-3.721	1.702	1.002	77.646	15.815	11.440
3377	159	24	21	P	W	13.898	-0.414	3.686	3.401	0.417	-3.451	-3.184	77.538	11.445	15.810
3378	159	24	21	P	W	17.897	5.011	1.263	-1.865	-4.554	-1.312	1.938	72.523	16.210	11.436
3379	159	24	21	P	W	42.103	5.011	-1.263	-1.865	-4.554	1.312	1.938	72.523	11.436	16.210
3380	159	24	21	P	W	46.102	-0.414	-3.685	3.401	0.417	3.451	-3.184	77.538	11.445	15.810
3381	159	24	21	P	W	49.107	4.020	1.668	-0.982	-3.721	-1.702	1.002	77.646	15.815	11.440
3382	159	24	21	P	W	51.052	1.048	5.175	1.871	-1.026	-4.989	-1.804	77.663	11.437	15.808
3383	159	24	21	P	W	68.948	1.048	-5.175	1.871	-1.026	4.989	-1.804	77.664	15.808	11.437
3384	159	24	21	P	W	70.893	4.020	-1.668	-0.982	-3.721	1.702	1.002	77.646	15.815	11.440
3385	159	24	21	P	W	73.898	-0.414	3.686	3.401	0.417	-3.451	-3.184	77.538	11.445	15.810
3386	159	24	21	P	W	77.897	5.011	1.263	-1.865	-4.554	-1.312	1.938	72.523	16.210	11.436
3387	159	24	21	P	W	102.104	5.011	-1.263	-1.865	-4.554	1.312	1.938	72.523	11.436	16.210
3388	159	24	21	P	W	106.102	-0.414	-3.685	3.401	0.417	3.451	-3.184	77.538	11.445	15.810
3389	159	24	21	P	W	109.107	4.020	1.668	-0.982	-3.721	-1.702	1.002	77.646	15.815	11.440
3390	159	24	21	P	W	111.052	1.048	5.175	1.871	-1.026	-4.989	-1.804	77.664	11.437	15.808
3391	160	25	20	C	S	8.948	-0.599	-2.525	-0.994	0.606	2.576	1.014	70.916	14.425	12.903
3392	160	25	20	C	S	10.893	-1.983	0.849	0.418	2.064	-0.842	-0.415	70.885	12.906	14.427
3393	160	25	20	C	S	23.413	0.481	-0.290	-2.042	-0.477	0.302	2.130	83.412	12.584	14.072
3394	160	25	20	C	S	34.950	-0.955	-2.546	-0.638	0.973	2.578	0.647	83.436	12.584	14.069
3395	160	25	20	C	S	36.587	0.481	0.290	-2.042	-0.477	-0.302	2.130	83.412	12.584	14.072
3396	160	25	20	C	S	49.107	-1.983	-0.849	0.418	2.064	0.842	-0.415	70.885	12.906	14.427
3397	160	25	20	C	S	51.052	-0.599	2.525	-0.994	0.606	-2.576	1.014	70.916	12.903	14.425
3398	160	25	20	C	S	68.948	-0.599	-2.525	-0.994	0.606	2.576	1.014	70.916	14.425	12.903
3399	160	25	20	C	S	70.893	-1.983	0.849	0.418	2.064	-0.842	-0.415	70.885	12.906	14.427
3400	160	25	20	C	S	83.413	-2.043	-0.298	0.481	2.130	0.295	-0.477	83.410	14.072	12.584
3401	160	25	20	C	S	85.050	-0.955	2.546	-0.638	0.973	-2.578	0.647	83.436	14.069	12.584
3402	160	25	20	C	S	94.950	-0.955	-2.546	-0.638	0.973	2.578	0.647	83.436	12.584	14.069
3403	160	25	20	C	S	96.587	-2.043	0.298	0.481	2.130	-0.295	-0.477	83.410	14.072	12.584
3404	160	25	20	C	S	109.107	-1.983	-0.849	0.418	2.064	0.842	-0.415	70.885	12.906	14.427
3405	160	25	20	C	S	111.052	-0.599	2.525	-0.994	0.606	-2.576	1.014	70.916	12.903	14.425
3406	160	25	20	C	W	8.948	-0.599	-2.525	-0.994	0.606	2.576	1.014	70.916	14.425	12.903
3407	160	25	20	C	W	10.893	-1.983	0.849	0.418	2.064	-0.842	-0.415	70.885	12.906	14.427
3408	160	25	20	C	W	23.413	0.481	-0.290	-2.042	-0.477	0.302	2.130	83.412	12.584	14.072
3409	160	25	20	C	W	25.050	-0.955	2.546	-0.638	0.973	-2.578	0.647	83.436	14.069	12.584
3410	160	25	20	C	W	34.950	-0.955	-2.546	-0.638	0.973	2.578	0.647	83.436	12.584	14.069
3411	160	25	20	C	W	36.587	-2.043	0.298	0.481	2.130	-0.295	-0.477	83.410	14.072	12.584
3412	160	25	20	C	W	49.107	-1.983	-0.849	0.418	2.064	0.842	-0.415	70.885	12.906	14.427
3413	160	25	20	C	W	51.052	-0.599	2.525	-0.994	0.606	-2.576	1.014	70.916	12.903	14.425
3414	160	25	20	C	W	68.948	-0.599	-2.525	-0.994	0.606	2.576	1.014	70.916	14.425	12.903
3415	160	25	20	C	W	70.893	-1.983	0.849	0.418	2.064	-0.842	-0.415	70.885	12.906	14.427
3416	160	25	20	C	W	83.413	-2.043	-0.298	0.481	2.130	0.295	-0.477	83.410	14.072	12.584
3417	160	25	20	C	W	94.950	-0.955	-2.546	-0.638	0.973	2.578	0.647	83.436	12.584	14.069

BL number	Atoms	γ-PC unit cells	WS_2 unit cells	γ-PC origin atom	WS_2 origin atom	Twist-angle (°)	γ-PC strain 1 (%)	γ-PC strain 2 (%)	γ-PC strain 3 (%)	WS_2 strain 1 (%)	WS_2 strain 2 (%)	WS_2 strain 3 (%)	γ (°)	|**a**|(Å)	|**b**|(Å)
3418	160	25	20	C	W	96.587	-2.043	0.298	0.481	2.130	-0.295	-0.477	83.410	14.072	12.584
3419	160	25	20	C	W	109.107	-1.983	-0.849	0.418	2.064	0.842	-0.415	70.885	12.906	14.427
3420	160	25	20	C	W	111.052	-0.599	2.525	-0.994	0.606	-2.576	1.014	70.916	12.903	14.425
3421	160	25	20	P	S	8.948	-0.599	-2.525	-0.994	0.606	2.576	1.014	70.916	14.425	12.903
3422	160	25	20	P	S	10.893	-1.983	0.849	0.418	2.064	-0.842	-0.415	70.885	12.906	14.427
3423	160	25	20	P	S	23.413	0.481	-0.290	-2.042	-0.477	0.302	2.130	83.412	12.584	14.072
3424	160	25	20	P	S	25.050	-0.955	2.546	-0.638	0.973	-2.578	0.647	83.436	14.069	12.584
3425	160	25	20	P	S	34.950	-0.955	-2.546	-0.638	0.973	2.578	0.647	83.436	12.584	14.069
3426	160	25	20	P	S	36.587	-2.043	0.298	0.481	2.130	-0.295	-0.477	83.410	14.072	12.584
3427	160	25	20	P	S	49.107	-1.983	-0.849	0.418	2.064	0.842	-0.415	70.885	12.906	14.427
3428	160	25	20	P	S	51.052	-0.599	2.525	-0.994	0.606	-2.576	1.014	70.916	12.903	14.425
3429	160	25	20	P	S	68.948	-0.599	-2.525	-0.994	0.606	2.576	1.014	70.916	14.425	12.903
3430	160	25	20	P	S	70.893	-1.983	0.849	0.418	2.064	-0.842	-0.415	70.885	12.906	14.427
3431	160	25	20	P	S	83.413	-2.043	-0.298	0.481	2.130	0.295	-0.477	83.410	14.072	12.584
3432	160	25	20	P	S	94.950	-0.955	-2.546	-0.638	0.973	2.578	0.647	83.436	12.584	14.069
3433	160	25	20	P	S	96.587	-2.043	0.298	0.481	2.130	-0.295	-0.477	83.410	14.072	12.584
3434	160	25	20	P	S	109.107	-1.983	-0.849	0.418	2.064	0.842	-0.415	70.885	12.906	14.427
3435	160	25	20	P	S	111.052	-0.599	2.525	-0.994	0.606	-2.576	1.014	70.916	12.903	14.425
3436	160	25	20	P	W	8.948	-0.599	-2.525	-0.994	0.606	2.576	1.014	70.916	14.425	12.903
3437	160	25	20	P	W	10.893	-1.983	0.849	0.418	2.064	-0.842	-0.415	70.885	12.906	14.427
3438	160	25	20	P	W	23.413	0.481	-0.290	-2.042	-0.477	0.302	2.130	83.412	12.584	14.072
3439	160	25	20	P	W	34.950	-0.955	-2.546	-0.638	0.973	2.578	0.647	83.436	12.584	14.069
3440	160	25	20	P	W	36.587	0.481	0.290	-2.042	-0.477	-0.302	2.130	83.412	12.584	14.072
3441	160	25	20	P	W	49.107	-1.983	-0.849	0.418	2.064	0.842	-0.415	70.885	12.906	14.427
3442	160	25	20	P	W	51.052	-0.599	2.525	-0.994	0.606	-2.576	1.014	70.916	12.903	14.425
3443	160	25	20	P	W	68.948	-0.599	-2.525	-0.994	0.606	2.576	1.014	70.916	14.425	12.903
3444	160	25	20	P	W	70.893	-1.983	0.849	0.418	2.064	-0.842	-0.415	70.885	12.906	14.427
3445	160	25	20	P	W	83.413	-2.043	-0.298	0.481	2.130	0.295	-0.477	83.410	14.072	12.584
3446	160	25	20	P	W	85.050	-0.955	2.546	-0.638	0.973	-2.578	0.647	83.436	14.069	12.584
3447	160	25	20	P	W	94.950	-0.955	-2.546	-0.638	0.973	2.578	0.647	83.436	12.584	14.069
3448	160	25	20	P	W	96.587	-2.043	0.298	0.481	2.130	-0.295	-0.477	83.410	14.072	12.584
3449	160	25	20	P	W	109.107	-1.983	-0.849	0.418	2.064	0.842	-0.415	70.885	12.906	14.427
3450	160	25	20	P	W	111.052	-0.599	2.525	-0.994	0.606	-2.576	1.014	70.916	12.903	14.425
3451	161	23	23	C	S	9.430	5.011	-5.272	5.011	-4.554	4.792	-4.555	85.771	13.484	13.484
3452	161	23	23	C	S	50.570	5.011	5.272	5.011	-4.554	-4.792	-4.555	85.771	13.484	13.484
3453	161	23	23	C	S	69.430	5.011	-5.272	5.011	-4.554	4.792	-4.555	85.771	13.484	13.484
3454	161	23	23	C	S	110.570	5.011	5.272	5.011	-4.554	-4.792	-4.555	85.771	13.484	13.484
3455	161	23	23	C	W	9.430	5.011	-5.272	5.011	-4.554	4.792	-4.555	85.771	13.484	13.484
3456	161	23	23	C	W	50.570	5.011	5.272	5.011	-4.554	-4.792	-4.555	85.771	13.484	13.484
3457	161	23	23	C	W	69.430	5.011	-5.272	5.011	-4.554	4.792	-4.555	85.771	13.484	13.484
3458	161	23	23	C	W	110.570	5.011	5.272	5.011	-4.554	-4.792	-4.555	85.771	13.484	13.484
3459	161	23	23	P	S	9.430	5.011	-5.272	5.011	-4.554	4.792	-4.555	85.771	13.484	13.484
3460	161	23	23	P	S	50.570	5.011	5.272	5.011	-4.554	-4.792	-4.555	85.771	13.484	13.484
3461	161	23	23	P	S	69.430	5.011	-5.272	5.011	-4.554	4.792	-4.555	85.771	13.484	13.484
3462	161	23	23	P	S	110.570	5.011	5.272	5.011	-4.554	-4.792	-4.555	85.771	13.484	13.484
3463	161	23	23	P	W	9.430	5.011	-5.272	5.011	-4.554	4.792	-4.555	85.771	13.484	13.484
3464	161	23	23	P	W	50.570	5.011	5.272	5.011	-4.554	-4.792	-4.555	85.771	13.484	13.484
3465	161	23	23	P	W	69.430	5.011	-5.272	5.011	-4.554	4.792	-4.555	85.771	13.484	13.484
3466	161	23	23	P	W	110.570	5.011	5.272	5.011	-4.554	-4.792	-4.555	85.771	13.484	13.484
3467	162	24	22	C	S	9.430	5.011	-5.052	0.427	-4.554	5.010	-0.423	77.496	10.628	17.439
3468	162	24	22	C	S	50.570	5.011	5.052	0.427	-4.554	-5.010	-0.423	77.496	17.439	10.628

BL number	Atoms	γ-PC unit cells	WS_2 unit cells	γ-PC origin atom	WS_2 origin atom	Twist-angle (°)	γ-PC strain 1 (%)	γ-PC strain 2 (%)	γ-PC strain 3 (%)	WS_2 strain 1 (%)	WS_2 strain 2 (%)	WS_2 strain 3 (%)	γ (°)	|**a**|(Å)	|**b**|(Å)
3469	162	24	22	C	S	69.430	5.011	-5.052	0.427	-4.554	5.010	-0.423	77.496	10.628	17.439
3470	162	24	22	C	S	110.570	5.011	5.052	0.427	-4.554	-5.010	-0.423	77.496	17.439	10.628
3471	162	24	22	C	W	9.430	5.011	-5.052	0.427	-4.554	5.010	-0.423	77.496	10.628	17.439
3472	162	24	22	C	W	50.570	5.011	5.052	0.427	-4.554	-5.010	-0.423	77.496	17.439	10.628
3473	162	24	22	C	W	69.430	5.011	-5.052	0.427	-4.554	5.010	-0.423	77.496	10.628	17.439
3474	162	24	22	C	W	110.570	5.011	5.052	0.427	-4.554	-5.010	-0.423	77.496	17.439	10.628
3475	162	24	22	P	S	9.430	5.011	-5.052	0.427	-4.554	5.010	-0.423	77.496	10.628	17.439
3476	162	24	22	P	S	50.570	5.011	5.052	0.427	-4.554	-5.010	-0.423	77.496	17.439	10.628
3477	162	24	22	P	S	69.430	5.011	-5.052	0.427	-4.554	5.010	-0.423	77.496	10.628	17.439
3478	162	24	22	P	S	110.570	5.011	5.052	0.427	-4.554	-5.010	-0.423	77.496	17.439	10.628
3479	162	24	22	P	W	9.430	5.011	-5.052	0.427	-4.554	5.010	-0.423	77.496	10.628	17.439
3480	162	24	22	P	W	50.570	5.011	5.052	0.427	-4.554	-5.010	-0.423	77.496	17.439	10.628
3481	162	24	22	P	W	69.430	5.011	-5.052	0.427	-4.554	5.010	-0.423	77.496	10.628	17.439
3482	162	24	22	P	W	110.570	5.011	5.052	0.427	-4.554	-5.010	-0.423	77.496	17.439	10.628
3483	163	25	21	C	S	10.893	0.418	0.000	0.418	-0.415	0.000	-0.415	60.000	14.426	14.426
3484	163	25	21	C	S	49.107	0.418	0.000	0.418	-0.415	0.000	-0.415	60.000	14.426	14.426
3485	163	25	21	C	S	70.893	0.418	0.000	0.418	-0.415	0.000	-0.415	60.000	14.426	14.426
3486	163	25	21	C	S	109.107	0.418	0.000	0.418	-0.415	0.000	-0.415	60.000	14.426	14.426
3487	163	25	21	C	W	10.893	0.418	0.000	0.418	-0.415	0.000	-0.415	60.000	14.426	14.426
3488	163	25	21	C	W	49.107	0.418	0.000	0.418	-0.415	0.000	-0.415	60.000	14.426	14.426
3489	163	25	21	C	W	70.893	0.418	0.000	0.418	-0.415	0.000	-0.415	60.000	14.426	14.426
3490	163	25	21	C	W	109.107	0.418	0.000	0.418	-0.415	0.000	-0.415	60.000	14.426	14.426
3491	163	25	21	C	S	0.000	5.011	2.425	-3.791	-4.554	-2.624	4.102	78.599	10.878	16.933
3492	163	25	21	C	S	1.359	-3.292	5.115	4.423	3.523	-4.699	-4.063	78.633	16.914	10.885
3493	163	25	21	C	S	9.430	5.011	-4.850	-3.791	-4.554	5.248	4.102	82.817	18.353	9.917
3494	163	25	21	C	S	21.052	1.339	-4.681	-0.486	-1.304	4.727	0.491	72.109	13.458	14.074
3495	163	25	21	C	S	23.413	-2.043	-0.595	3.006	2.130	0.562	-2.835	72.066	14.072	13.470
3496	163	25	21	C	S	25.693	2.326	3.358	-1.420	-2.223	-3.456	1.461	71.970	13.473	14.073
3497	163	25	21	C	S	34.307	2.326	-3.358	-1.420	-2.223	3.456	1.461	71.970	13.473	14.073
3498	163	25	21	C	S	36.587	-2.043	0.595	3.006	2.130	-0.562	-2.835	72.066	14.072	13.470
3499	163	25	21	C	S	38.948	1.339	4.681	-0.486	-1.304	-4.727	0.491	72.109	14.074	13.458
3500	163	25	21	C	S	50.570	5.011	4.850	-3.791	-4.554	-5.248	4.102	82.817	18.353	9.917
3501	163	25	21	C	S	58.641	-3.292	-5.115	4.423	3.523	4.699	-4.063	78.633	10.885	16.914
3502	163	25	21	C	S	60.000	5.011	2.425	-3.791	-4.554	-2.624	4.102	78.599	10.878	16.933
3503	163	25	21	C	S	61.359	-3.292	5.115	4.423	3.523	-4.699	-4.063	78.633	16.914	10.885
3504	163	25	21	C	S	69.430	5.011	-4.850	-3.791	-4.554	5.248	4.102	82.817	18.353	9.917
3505	163	25	21	C	S	81.052	1.339	-4.681	-0.486	-1.304	4.727	0.491	72.109	13.458	14.074
3506	163	25	21	C	S	83.413	-2.043	-0.595	3.006	2.130	0.562	-2.835	72.066	14.072	13.470
3507	163	25	21	C	S	85.694	2.326	3.358	-1.420	-2.223	-3.456	1.461	71.970	13.473	14.073
3508	163	25	21	C	S	94.307	2.326	-3.358	-1.420	-2.223	3.456	1.461	71.970	13.473	14.073
3509	163	25	21	C	S	96.587	-2.043	0.595	3.006	2.130	-0.562	-2.835	72.066	14.072	13.470
3510	163	25	21	C	S	98.948	1.339	4.681	-0.486	-1.304	-4.727	0.491	72.109	14.074	13.458
3511	163	25	21	C	S	110.570	5.011	4.850	-3.791	-4.554	-5.248	4.102	82.817	18.353	9.917
3512	163	25	21	C	S	118.641	-3.292	-5.115	4.423	3.523	4.699	-4.063	78.633	10.885	16.914
3513	163	25	21	C	W	0.000	5.011	2.425	-3.791	-4.554	-2.624	4.102	78.599	10.878	16.933
3514	163	25	21	C	W	1.359	-3.292	5.115	4.423	3.523	-4.699	-4.063	78.633	16.914	10.885
3515	163	25	21	C	W	9.430	5.011	-4.850	-3.791	-4.554	5.248	4.102	82.817	18.353	9.917
3516	163	25	21	C	W	21.052	1.339	-4.681	-0.486	-1.304	4.727	0.491	72.109	13.458	14.074
3517	163	25	21	C	W	23.413	-2.043	-0.595	3.006	2.130	0.562	-2.835	72.066	14.072	13.470
3518	163	25	21	C	W	25.693	2.326	3.358	-1.420	-2.223	-3.456	1.461	71.970	13.473	14.073
3519	163	25	21	C	W	34.307	2.326	-3.358	-1.420	-2.223	3.456	1.461	71.970	13.473	14.073

BL number	Atoms	γ-PC unit cells	WS_2 unit cells	γ-PC origin atom	WS_2 origin atom	Twist-angle (°)	γ-PC strain 1 (%)	γ-PC strain 2 (%)	γ-PC strain 3 (%)	WS_2 strain 1 (%)	WS_2 strain 2 (%)	WS_2 strain 3 (%)	γ (°)	|**a**|(Å)	|**b**|(Å)
3520	163	25	21	C	W	36.587	-2.043	0.595	3.006	2.130	-0.562	-2.835	72.066	14.072	13.470
3521	163	25	21	C	W	38.948	1.339	4.681	-0.486	-1.304	-4.727	0.491	72.109	14.074	13.458
3522	163	25	21	C	W	50.570	5.011	4.850	-3.791	-4.554	-5.248	4.102	82.817	18.353	9.917
3523	163	25	21	C	W	58.641	-3.292	-5.115	4.423	3.523	4.699	-4.063	78.633	10.885	16.914
3524	163	25	21	C	W	60.000	5.011	2.425	-3.791	-4.554	-2.624	4.102	78.599	10.878	16.933
3525	163	25	21	C	W	61.359	-3.292	5.115	4.423	3.523	-4.699	-4.063	78.633	16.914	10.885
3526	163	25	21	C	W	69.430	5.011	-4.850	-3.791	-4.554	5.248	4.102	82.817	18.353	9.917
3527	163	25	21	C	W	81.052	1.339	-4.681	-0.486	-1.304	4.727	0.491	72.109	13.458	14.074
3528	163	25	21	C	W	83.413	-2.043	-0.595	3.006	2.130	0.562	-2.835	72.066	14.072	13.470
3529	163	25	21	C	W	85.694	2.326	3.358	-1.420	-2.223	-3.456	1.461	71.970	13.473	14.073
3530	163	25	21	C	W	94.307	2.326	-3.358	-1.420	-2.223	3.456	1.461	71.970	13.473	14.073
3531	163	25	21	C	W	96.587	-2.043	0.595	3.006	2.130	-0.562	-2.835	72.066	14.072	13.470
3532	163	25	21	C	W	98.948	1.339	4.681	-0.486	-1.304	-4.727	0.491	72.109	14.074	13.458
3533	163	25	21	C	W	110.570	5.011	4.850	-3.791	-4.554	-5.248	4.102	82.817	18.353	9.917
3534	163	25	21	C	W	118.641	-3.292	-5.115	4.423	3.523	4.699	-4.063	78.633	10.885	16.914
3535	163	25	21	P	S	0.000	5.011	2.425	-3.791	-4.554	-2.624	4.102	78.599	10.878	16.933
3536	163	25	21	P	S	1.359	-3.292	5.115	4.423	3.523	-4.699	-4.063	78.633	16.914	10.885
3537	163	25	21	P	S	9.430	5.011	-4.850	-3.791	-4.554	5.248	4.102	82.817	18.353	9.917
3538	163	25	21	P	S	21.052	1.339	-4.681	-0.486	-1.304	4.727	0.491	72.109	13.458	14.074
3539	163	25	21	P	S	23.413	-2.043	-0.595	3.006	2.130	0.562	-2.835	72.066	14.072	13.470
3540	163	25	21	P	S	25.693	2.326	3.358	-1.420	-2.223	-3.456	1.461	71.970	13.473	14.073
3541	163	25	21	P	S	34.307	2.326	-3.358	-1.420	-2.223	3.456	1.461	71.970	13.473	14.073
3542	163	25	21	P	S	36.587	-2.043	0.595	3.006	2.130	-0.562	-2.835	72.066	14.072	13.470
3543	163	25	21	P	S	38.948	1.339	4.681	-0.486	-1.304	-4.727	0.491	72.109	14.074	13.458
3544	163	25	21	P	S	50.570	5.011	4.850	-3.791	-4.554	-5.248	4.102	82.817	18.353	9.917
3545	163	25	21	P	S	58.641	-3.292	-5.115	4.423	3.523	4.699	-4.063	78.633	10.885	16.914
3546	163	25	21	P	S	60.000	5.011	2.425	-3.791	-4.554	-2.624	4.102	78.599	10.878	16.933
3547	163	25	21	P	S	61.359	-3.292	5.115	4.423	3.523	-4.699	-4.063	78.633	16.914	10.885
3548	163	25	21	P	S	69.430	5.011	-4.850	-3.791	-4.554	5.248	4.102	82.817	18.353	9.917
3549	163	25	21	P	S	81.052	1.339	-4.681	-0.486	-1.304	4.727	0.491	72.109	13.458	14.074
3550	163	25	21	P	S	83.413	-2.043	-0.595	3.006	2.130	0.562	-2.835	72.066	14.072	13.470
3551	163	25	21	P	S	85.694	2.326	3.358	-1.420	-2.223	-3.456	1.461	71.970	13.473	14.073
3552	163	25	21	P	S	94.307	2.326	-3.358	-1.420	-2.223	3.456	1.461	71.970	13.473	14.073
3553	163	25	21	P	S	96.587	-2.043	0.595	3.006	2.130	-0.562	-2.835	72.066	14.072	13.470
3554	163	25	21	P	S	98.948	1.339	4.681	-0.486	-1.304	-4.727	0.491	72.109	14.074	13.458
3555	163	25	21	P	S	110.570	5.011	4.850	-3.791	-4.554	-5.248	4.102	82.817	18.353	9.917
3556	163	25	21	P	S	118.641	-3.292	-5.115	4.423	3.523	4.699	-4.063	78.633	10.885	16.914
3557	163	25	21	P	W	0.000	5.011	2.425	-3.791	-4.554	-2.624	4.102	78.599	10.878	16.933
3558	163	25	21	P	W	1.359	-3.292	5.115	4.423	3.523	-4.699	-4.063	78.633	16.914	10.885
3559	163	25	21	P	W	9.430	5.011	-4.850	-3.791	-4.554	5.248	4.102	82.817	18.353	9.917
3560	163	25	21	P	W	21.052	1.339	-4.681	-0.486	-1.304	4.727	0.491	72.109	13.458	14.074
3561	163	25	21	P	W	23.413	-2.043	-0.595	3.006	2.130	0.562	-2.835	72.066	14.072	13.470
3562	163	25	21	P	W	25.693	2.326	3.358	-1.420	-2.223	-3.456	1.461	71.970	13.473	14.073
3563	163	25	21	P	W	34.307	2.326	-3.358	-1.420	-2.223	3.456	1.461	71.970	13.473	14.073
3564	163	25	21	P	W	36.587	-2.043	0.595	3.006	2.130	-0.562	-2.835	72.066	14.072	13.470
3565	163	25	21	P	W	38.948	1.339	4.681	-0.486	-1.304	-4.727	0.491	72.109	14.074	13.458
3566	163	25	21	P	W	50.570	5.011	4.850	-3.791	-4.554	-5.248	4.102	82.817	18.353	9.917
3567	163	25	21	P	W	58.641	-3.292	-5.115	4.423	3.523	4.699	-4.063	78.633	10.885	16.914
3568	163	25	21	P	W	60.000	5.011	2.425	-3.791	-4.554	-2.624	4.102	78.599	10.878	16.933
3569	163	25	21	P	W	61.359	-3.292	5.115	4.423	3.523	-4.699	-4.063	78.633	16.914	10.885
3570	163	25	21	P	W	69.430	5.011	-4.850	-3.791	-4.554	5.248	4.102	82.817	18.353	9.917

BL number	Atoms	γ-PC unit cells	WS_2 unit cells	γ-PC origin atom	WS_2 origin atom	Twist-angle (°)	γ-PC strain 1 (%)	γ-PC strain 2 (%)	γ-PC strain 3 (%)	WS_2 strain 1 (%)	WS_2 strain 2 (%)	WS_2 strain 3 (%)	γ (°)	|**a**|(Å)	|**b**|(Å)
3571	163	25	21	P	W	81.052	1.339	-4.681	-0.486	-1.304	4.727	0.491	72.109	13.458	14.074
3572	163	25	21	P	W	83.413	-2.043	-0.595	3.006	2.130	0.562	-2.835	72.066	14.072	13.470
3573	163	25	21	P	W	85.694	2.326	3.358	-1.420	-2.223	-3.456	1.461	71.970	13.473	14.073
3574	163	25	21	P	W	94.307	2.326	-3.358	-1.420	-2.223	3.456	1.461	71.970	13.473	14.073
3575	163	25	21	P	W	96.587	-2.043	0.595	3.006	2.130	-0.562	-2.835	72.066	14.072	13.470
3576	163	25	21	P	W	98.948	1.339	4.681	-0.486	-1.304	-4.727	0.491	72.109	14.074	13.458
3577	163	25	21	P	W	110.570	5.011	4.850	-3.791	-4.554	-5.248	4.102	82.817	18.353	9.917
3578	163	25	21	P	W	118.641	-3.292	-5.115	4.423	3.523	4.699	-4.063	78.633	10.885	16.914
3579	164	26	20	C	S	8.213	-2.359	-2.168	-1.137	2.476	2.219	1.164	69.553	14.845	12.900
3580	164	26	20	C	S	10.893	-1.983	2.449	-1.521	2.064	-2.525	1.569	69.546	12.906	14.838
3581	164	26	20	C	S	17.480	-4.685	-4.345	1.370	5.169	4.229	-1.333	80.578	14.491	12.563
3582	164	26	20	C	S	22.689	-4.497	4.894	1.158	4.941	-4.783	-1.132	75.532	11.961	15.505
3583	164	26	20	C	S	23.413	0.481	-4.741	-3.887	-0.477	5.141	4.215	74.279	12.584	14.819
3584	164	26	20	C	S	25.285	-4.781	-1.691	1.480	5.287	1.643	-1.437	74.249	12.587	14.826
3585	164	26	20	C	S	34.715	-4.781	1.691	1.480	5.287	-1.643	-1.437	74.249	14.826	12.587
3586	164	26	20	C	S	36.587	0.481	4.741	-3.887	-0.477	-5.141	4.215	74.279	12.584	14.819
3587	164	26	20	C	S	37.311	-4.497	-4.894	1.158	4.941	4.783	-1.132	75.532	11.961	15.505
3588	164	26	20	C	S	42.520	-4.685	4.345	1.370	5.169	-4.229	-1.333	80.578	14.491	12.563
3589	164	26	20	C	S	49.107	-1.983	-2.449	-1.521	2.064	2.525	1.569	69.546	12.906	14.838
3590	164	26	20	C	S	51.052	-0.599	0.883	-2.879	0.606	-0.937	3.055	69.525	14.844	12.904
3591	164	26	20	C	S	51.787	-2.359	2.168	-1.137	2.476	-2.219	1.164	69.553	14.845	12.900
3592	164	26	20	C	S	68.213	-2.359	-2.168	-1.137	2.476	2.219	1.164	69.553	14.845	12.900
3593	164	26	20	C	S	68.948	-0.599	-0.883	-2.879	0.606	0.937	3.055	69.525	12.904	14.844
3594	164	26	20	C	S	70.893	-1.983	2.449	-1.521	2.064	-2.525	1.569	69.546	12.906	14.838
3595	164	26	20	C	S	77.480	-4.685	-4.345	1.370	5.169	4.229	-1.333	80.578	14.491	12.563
3596	164	26	20	C	S	82.689	-4.497	4.894	1.158	4.941	-4.783	-1.132	75.532	11.961	15.505
3597	164	26	20	C	S	83.413	0.481	-4.741	-3.887	-0.477	5.141	4.215	74.279	12.584	14.819
3598	164	26	20	C	S	85.285	-4.781	-1.691	1.480	5.287	1.643	-1.437	74.249	12.587	14.826
3599	164	26	20	C	S	94.715	-4.781	1.691	1.480	5.287	-1.643	-1.437	74.249	14.826	12.587
3600	164	26	20	C	S	96.587	0.481	4.741	-3.887	-0.477	-5.141	4.215	74.279	12.584	14.819
3601	164	26	20	C	S	97.311	-4.497	-4.894	1.158	4.941	4.783	-1.132	75.532	11.961	15.505
3602	164	26	20	C	S	102.520	-4.685	4.345	1.370	5.169	-4.229	-1.333	80.578	14.491	12.563
3603	164	26	20	C	S	111.052	-0.599	0.883	-2.879	0.606	-0.937	3.055	69.525	14.844	12.904
3604	164	26	20	C	S	111.787	-2.359	2.168	-1.137	2.476	-2.219	1.164	69.553	14.845	12.900
3605	164	26	20	C	W	8.213	-2.359	-2.168	-1.137	2.476	2.219	1.164	69.553	14.845	12.900
3606	164	26	20	C	W	8.948	-0.599	-0.883	-2.879	0.606	0.937	3.055	69.525	12.904	14.844
3607	164	26	20	C	W	10.893	-1.983	2.449	-1.521	2.064	-2.525	1.569	69.546	12.906	14.838
3608	164	26	20	C	W	17.480	-4.685	-4.345	1.370	5.169	4.229	-1.333	80.578	14.491	12.563
3609	164	26	20	C	W	22.689	-4.497	4.894	1.158	4.941	-4.783	-1.132	75.532	11.961	15.505
3610	164	26	20	C	W	23.413	0.481	-4.741	-3.887	-0.477	5.141	4.215	74.279	12.584	14.819
3611	164	26	20	C	W	25.285	-4.781	-1.691	1.480	5.287	1.643	-1.437	74.249	12.587	14.826
3612	164	26	20	C	W	34.715	-4.781	1.691	1.480	5.287	-1.643	-1.437	74.249	14.826	12.587
3613	164	26	20	C	W	36.587	0.481	4.741	-3.887	-0.477	-5.141	4.215	74.279	12.584	14.819
3614	164	26	20	C	W	37.311	-4.497	-4.894	1.158	4.941	4.783	-1.132	75.532	11.961	15.505
3615	164	26	20	C	W	42.520	-4.685	4.345	1.370	5.169	-4.229	-1.333	80.578	14.491	12.563
3616	164	26	20	C	W	51.052	-0.599	0.883	-2.879	0.606	-0.937	3.055	69.525	14.844	12.904
3617	164	26	20	C	W	51.787	-2.359	2.168	-1.137	2.476	-2.219	1.164	69.553	14.845	12.900
3618	164	26	20	C	W	68.213	-2.359	-2.168	-1.137	2.476	2.219	1.164	69.553	14.845	12.900
3619	164	26	20	C	W	70.893	-1.983	2.449	-1.521	2.064	-2.525	1.569	69.546	12.906	14.838
3620	164	26	20	C	W	77.480	-4.685	-4.345	1.370	5.169	4.229	-1.333	80.578	14.491	12.563
3621	164	26	20	C	W	82.689	-4.497	4.894	1.158	4.941	-4.783	-1.132	75.532	11.961	15.505

BL number	Atoms	γ-PC unit cells	WS_2 unit cells	γ-PC origin atom	WS_2 origin atom	Twist-angle (°)	γ-PC strain 1 (%)	γ-PC strain 2 (%)	γ-PC strain 3 (%)	WS_2 strain 1 (%)	WS_2 strain 2 (%)	WS_2 strain 3 (%)	γ (°)	|**a**|(Å)	|**b**|(Å)
3622	164	26	20	C	W	83.413	0.481	-4.741	-3.887	-0.477	5.141	4.215	74.279	12.584	14.819
3623	164	26	20	C	W	85.285	-4.781	-1.691	1.480	5.287	1.643	-1.437	74.249	12.587	14.826
3624	164	26	20	C	W	94.715	-4.781	1.691	1.480	5.287	-1.643	-1.437	74.249	14.826	12.587
3625	164	26	20	C	W	96.587	0.481	4.741	-3.887	-0.477	-5.141	4.215	74.279	12.584	14.819
3626	164	26	20	C	W	97.311	-4.497	-4.894	1.158	4.941	4.783	-1.132	75.532	11.961	15.505
3627	164	26	20	C	W	102.520	-4.685	4.345	1.370	5.169	-4.229	-1.333	80.578	14.491	12.563
3628	164	26	20	C	W	109.107	-1.983	-2.449	-1.521	2.064	2.525	1.569	69.546	12.906	14.838
3629	164	26	20	C	W	111.052	-0.599	0.883	-2.879	0.606	-0.937	3.055	69.525	14.844	12.904
3630	164	26	20	C	W	111.787	-2.359	2.168	-1.137	2.476	-2.219	1.164	69.553	14.845	12.900
3631	164	26	20	P	S	8.213	-2.359	-2.168	-1.137	2.476	2.219	1.164	69.553	14.845	12.900
3632	164	26	20	P	S	8.948	-0.599	-0.883	-2.879	0.606	0.937	3.055	69.525	12.904	14.844
3633	164	26	20	P	S	10.893	-1.983	2.449	-1.521	2.064	-2.525	1.569	69.546	12.906	14.838
3634	164	26	20	P	S	17.480	-4.685	-4.345	1.370	5.169	4.229	-1.333	80.578	14.491	12.563
3635	164	26	20	P	S	22.689	-4.497	4.894	1.158	4.941	-4.783	-1.132	75.532	11.961	15.505
3636	164	26	20	P	S	23.413	0.481	-4.741	-3.887	-0.477	5.141	4.215	74.279	12.584	14.819
3637	164	26	20	P	S	25.285	-4.781	-1.691	1.480	5.287	1.643	-1.437	74.249	12.587	14.826
3638	164	26	20	P	S	34.715	-4.781	1.691	1.480	5.287	-1.643	-1.437	74.249	14.826	12.587
3639	164	26	20	P	S	36.587	0.481	4.741	-3.887	-0.477	-5.141	4.215	74.279	12.584	14.819
3640	164	26	20	P	S	51.052	-0.599	0.883	-2.879	0.606	-0.937	3.055	69.525	14.844	12.904
3641	164	26	20	P	S	51.787	-2.359	2.168	-1.137	2.476	-2.219	1.164	69.553	14.845	12.900
3642	164	26	20	P	S	68.213	-2.359	-2.168	-1.137	2.476	2.219	1.164	69.553	14.845	12.900
3643	164	26	20	P	S	70.893	-1.983	2.449	-1.521	2.064	-2.525	1.569	69.546	12.906	14.838
3644	164	26	20	P	S	83.413	0.481	-4.741	-3.887	-0.477	5.141	4.215	74.279	12.584	14.819
3645	164	26	20	P	S	85.285	-4.781	-1.691	1.480	5.287	1.643	-1.437	74.249	12.587	14.826
3646	164	26	20	P	S	94.715	-4.781	1.691	1.480	5.287	-1.643	-1.437	74.249	14.826	12.587
3647	164	26	20	P	S	96.587	0.481	4.741	-3.887	-0.477	-5.141	4.215	74.279	12.584	14.819
3648	164	26	20	P	S	97.311	-4.497	-4.894	1.158	4.941	4.783	-1.132	75.532	11.961	15.505
3649	164	26	20	P	S	102.520	-4.685	4.345	1.370	5.169	-4.229	-1.333	80.578	14.491	12.563
3650	164	26	20	P	S	109.107	-1.983	-2.449	-1.521	2.064	2.525	1.569	69.546	12.906	14.838
3651	164	26	20	P	S	111.052	-0.599	0.883	-2.879	0.606	-0.937	3.055	69.525	14.844	12.904
3652	164	26	20	P	S	111.787	-2.359	2.168	-1.137	2.476	-2.219	1.164	69.553	14.845	12.900
3653	164	26	20	P	W	8.213	-2.359	-2.168	-1.137	2.476	2.219	1.164	69.553	14.845	12.900
3654	164	26	20	P	W	10.893	-1.983	2.449	-1.521	2.064	-2.525	1.569	69.546	12.906	14.838
3655	164	26	20	P	W	23.413	0.481	-4.741	-3.887	-0.477	5.141	4.215	74.279	12.584	14.819
3656	164	26	20	P	W	25.285	-4.781	-1.691	1.480	5.287	1.643	-1.437	74.249	12.587	14.826
3657	164	26	20	P	W	34.715	-4.781	1.691	1.480	5.287	-1.643	-1.437	74.249	14.826	12.587
3658	164	26	20	P	W	36.587	0.481	4.741	-3.887	-0.477	-5.141	4.215	74.279	12.584	14.819
3659	164	26	20	P	W	37.311	-4.497	-4.894	1.158	4.941	4.783	-1.132	75.532	11.961	15.505
3660	164	26	20	P	W	42.520	-4.685	4.345	1.370	5.169	-4.229	-1.333	80.578	14.491	12.563
3661	164	26	20	P	W	49.107	-1.983	-2.449	-1.521	2.064	2.525	1.569	69.546	12.906	14.838
3662	164	26	20	P	W	51.052	-0.599	0.883	-2.879	0.606	-0.937	3.055	69.525	14.844	12.904
3663	164	26	20	P	W	51.787	-2.359	2.168	-1.137	2.476	-2.219	1.164	69.553	14.845	12.900
3664	164	26	20	P	W	68.213	-2.359	-2.168	-1.137	2.476	2.219	1.164	69.553	14.845	12.900
3665	164	26	20	P	W	68.948	-0.599	-0.883	-2.879	0.606	0.937	3.055	69.525	12.904	14.844
3666	164	26	20	P	W	70.893	-1.983	2.449	-1.521	2.064	-2.525	1.569	69.546	12.906	14.838
3667	164	26	20	P	W	77.480	-4.685	-4.345	1.370	5.169	4.229	-1.333	80.578	14.491	12.563
3668	164	26	20	P	W	82.689	-4.497	4.894	1.158	4.941	-4.783	-1.132	75.532	11.961	15.505
3669	164	26	20	P	W	83.413	0.481	-4.741	-3.887	-0.477	5.141	4.215	74.279	12.584	14.819
3670	164	26	20	P	W	85.285	-4.781	-1.691	1.480	5.287	1.643	-1.437	74.249	12.587	14.826
3671	164	26	20	P	W	94.715	-4.781	1.691	1.480	5.287	-1.643	-1.437	74.249	14.826	12.587
3672	164	26	20	P	W	96.587	0.481	4.741	-3.887	-0.477	-5.141	4.215	74.279	12.584	14.819

BL number	Atoms	γ-PC unit cells	WS_2 unit cells	γ-PC origin atom	WS_2 origin atom	Twist-angle (°)	γ-PC strain 1 (%)	γ-PC strain 2 (%)	γ-PC strain 3 (%)	WS_2 strain 1 (%)	WS_2 strain 2 (%)	WS_2 strain 3 (%)	γ (°)	**a**(Å)	**b**(Å)
3673	164	26	20	P	W	111.052	-0.599	0.883	-2.879	0.606	-0.937	3.055	69.525	14.844	12.904
3674	164	26	20	P	W	111.787	-2.359	2.168	-1.137	2.476	-2.219	1.164	69.553	14.845	12.900
3675	165	24	23	C	S	21.787	5.011	-3.789	2.719	-4.554	3.594	-2.579	83.685	13.826	13.468
3676	165	24	23	C	S	25.693	2.326	3.304	5.424	-2.223	-2.980	-4.893	83.793	13.473	13.819
3677	165	24	23	C	S	34.307	2.326	-3.304	5.424	-2.223	2.980	-4.893	83.793	13.473	13.819
3678	165	24	23	C	S	38.213	5.011	3.789	2.719	-4.554	-3.594	-2.579	83.685	13.826	13.468
3679	165	24	23	C	S	81.787	5.011	-3.789	2.719	-4.554	3.594	-2.579	83.685	13.826	13.468
3680	165	24	23	C	S	85.694	2.326	3.304	5.424	-2.223	-2.980	-4.893	83.793	13.473	13.819
3681	165	24	23	C	S	94.307	2.326	-3.304	5.424	-2.223	2.980	-4.893	83.793	13.473	13.819
3682	165	24	23	C	S	98.213	5.011	3.789	2.719	-4.554	-3.594	-2.579	83.685	13.826	13.468
3683	165	24	23	C	W	21.787	5.011	-3.789	2.719	-4.554	3.594	-2.579	83.685	13.826	13.468
3684	165	24	23	C	W	25.693	2.326	3.304	5.424	-2.223	-2.980	-4.893	83.793	13.473	13.819
3685	165	24	23	C	W	34.307	2.326	-3.304	5.424	-2.223	2.980	-4.893	83.793	13.473	13.819
3686	165	24	23	C	W	38.213	5.011	3.789	2.719	-4.554	-3.594	-2.579	83.685	13.826	13.468
3687	165	24	23	C	W	81.787	5.011	-3.789	2.719	-4.554	3.594	-2.579	83.685	13.826	13.468
3688	165	24	23	C	W	85.694	2.326	3.304	5.424	-2.223	-2.980	-4.893	83.793	13.473	13.819
3689	165	24	23	C	W	94.307	2.326	-3.304	5.424	-2.223	2.980	-4.893	83.793	13.473	13.819
3690	165	24	23	C	W	98.213	5.011	3.789	2.719	-4.554	-3.594	-2.579	83.685	13.826	13.468
3691	165	24	23	P	S	21.787	5.011	-3.789	2.719	-4.554	3.594	-2.579	83.685	13.826	13.468
3692	165	24	23	P	S	34.307	2.326	-3.304	5.424	-2.223	2.980	-4.893	83.793	13.473	13.819
3693	165	24	23	P	S	38.213	5.011	3.789	2.719	-4.554	-3.594	-2.579	83.685	13.826	13.468
3694	165	24	23	P	S	81.787	5.011	-3.789	2.719	-4.554	3.594	-2.579	83.685	13.826	13.468
3695	165	24	23	P	S	85.694	2.326	3.304	5.424	-2.223	-2.980	-4.893	83.793	13.473	13.819
3696	165	24	23	P	S	98.213	5.011	3.789	2.719	-4.554	-3.594	-2.579	83.685	13.826	13.468
3697	165	24	23	P	W	21.787	5.011	-3.789	2.719	-4.554	3.594	-2.579	83.685	13.826	13.468
3698	165	24	23	P	W	25.693	2.326	3.304	5.424	-2.223	-2.980	-4.893	83.793	13.473	13.819
3699	165	24	23	P	W	38.213	5.011	3.789	2.719	-4.554	-3.594	-2.579	83.685	13.826	13.468
3700	165	24	23	P	W	94.307	2.326	-3.304	5.424	-2.223	2.980	-4.893	83.793	13.473	13.819
3701	165	24	23	P	W	98.213	5.011	3.789	2.719	-4.554	-3.594	-2.579	83.685	13.826	13.468
3702	165	27	19	C	S	6.587	-3.853	0.000	-3.853	4.175	0.000	4.175	60.000	14.354	14.354
3703	165	27	19	C	S	53.413	-3.853	0.000	-3.853	4.175	0.000	4.175	60.000	14.354	14.354
3704	165	27	19	C	S	66.587	-3.853	0.000	-3.853	4.175	0.000	4.175	60.000	14.354	14.354
3705	165	27	19	C	S	113.413	-3.853	0.000	-3.853	4.175	0.000	4.175	60.000	14.354	14.354
3706	165	27	19	P	S	6.587	-3.853	0.000	-3.853	4.175	0.000	4.175	60.000	14.354	14.354
3707	165	27	19	P	S	53.413	-3.853	0.000	-3.853	4.175	0.000	4.175	60.000	14.354	14.354
3708	165	27	19	P	S	66.587	-3.853	0.000	-3.853	4.175	0.000	4.175	60.000	14.354	14.354
3709	165	27	19	P	S	113.413	-3.853	0.000	-3.853	4.175	0.000	4.175	60.000	14.354	14.354
3710	166	25	22	C	S	10.158	2.281	-2.109	0.937	-2.182	2.070	-0.920	71.729	13.467	14.429
3711	166	25	22	C	S	10.893	0.418	-0.828	2.819	-0.415	0.784	-2.669	71.723	14.426	13.472
3712	166	25	22	C	S	12.520	2.326	2.052	0.894	-2.223	-2.016	-0.878	71.686	13.473	14.427
3713	166	25	22	C	S	16.102	2.853	-0.594	0.386	-2.699	0.589	-0.383	80.926	10.655	17.539
3714	166	25	22	C	S	17.696	1.029	2.238	2.187	-1.008	-2.145	-2.096	80.947	17.539	10.653
3715	166	25	22	C	S	21.787	5.011	-3.638	-1.590	-4.554	3.757	1.643	71.599	13.826	14.078
3716	166	25	22	C	S	38.213	5.011	3.638	-1.590	-4.554	-3.757	1.643	71.599	13.826	14.078
3717	166	25	22	C	S	42.304	1.029	-2.238	2.187	-1.008	2.145	-2.096	80.947	10.653	17.539
3718	166	25	22	C	S	43.663	0.353	0.187	2.887	-0.351	-0.177	-2.730	80.938	17.539	10.655
3719	166	25	22	C	S	47.480	2.326	-2.052	0.894	-2.223	2.016	-0.878	71.686	13.473	14.427
3720	166	25	22	C	S	49.107	0.418	0.828	2.819	-0.415	-0.784	-2.669	71.723	14.426	13.472
3721	166	25	22	C	S	49.842	2.281	2.109	0.937	-2.182	-2.070	-0.920	71.729	14.429	13.467
3722	166	25	22	C	S	70.158	2.281	-2.109	0.937	-2.182	2.070	-0.920	71.729	13.467	14.429
3723	166	25	22	C	S	70.893	0.418	-0.828	2.819	-0.415	0.784	-2.669	71.723	14.426	13.472

BL number	Atoms	γ-PC unit cells	WS_2 unit cells	γ-PC origin atom	WS_2 origin atom	Twist-angle (°)	γ-PC strain 1 (%)	γ-PC strain 2 (%)	γ-PC strain 3 (%)	WS_2 strain 1 (%)	WS_2 strain 2 (%)	WS_2 strain 3 (%)	γ (°)	|**a**|(Å)	|**b**|(Å)
3724	166	25	22	C	S	72.520	2.326	2.052	0.894	-2.223	-2.016	-0.878	71.686	13.473	14.427
3725	166	25	22	C	S	76.102	2.853	-0.594	0.386	-2.699	0.589	-0.383	80.926	10.655	17.539
3726	166	25	22	C	S	77.696	1.029	2.238	2.187	-1.008	-2.145	-2.096	80.947	17.539	10.653
3727	166	25	22	C	S	81.787	5.011	-3.638	-1.590	-4.554	3.757	1.643	71.599	13.826	14.078
3728	166	25	22	C	S	98.213	5.011	3.638	-1.590	-4.554	-3.757	1.643	71.599	13.826	14.078
3729	166	25	22	C	S	103.663	0.353	0.187	2.887	-0.351	-0.177	-2.730	80.938	17.539	10.655
3730	166	25	22	C	S	103.898	2.853	0.594	0.386	-2.699	-0.589	-0.383	80.926	10.655	17.539
3731	166	25	22	C	S	107.480	2.326	-2.052	0.894	-2.223	2.016	-0.878	71.686	13.473	14.427
3732	166	25	22	C	S	109.107	0.418	0.828	2.819	-0.415	-0.784	-2.669	71.723	14.426	13.472
3733	166	25	22	C	S	109.842	2.281	2.109	0.937	-2.182	-2.070	-0.920	71.729	14.429	13.467
3734	166	25	22	C	W	10.158	2.281	-2.109	0.937	-2.182	2.070	-0.920	71.729	13.467	14.429
3735	166	25	22	C	W	10.893	0.418	-0.828	2.819	-0.415	0.784	-2.669	71.723	14.426	13.472
3736	166	25	22	C	W	12.520	2.326	2.052	0.894	-2.223	-2.016	-0.878	71.686	13.473	14.427
3737	166	25	22	C	W	16.102	2.853	-0.594	0.386	-2.699	0.589	-0.383	80.926	10.655	17.539
3738	166	25	22	C	W	17.696	1.029	2.238	2.187	-1.008	-2.145	-2.096	80.947	17.539	10.653
3739	166	25	22	C	W	21.787	5.011	-3.638	-1.590	-4.554	3.757	1.643	71.599	13.826	14.078
3740	166	25	22	C	W	38.213	5.011	3.638	-1.590	-4.554	-3.757	1.643	71.599	13.826	14.078
3741	166	25	22	C	W	43.663	0.353	0.187	2.887	-0.351	-0.177	-2.730	80.938	17.539	10.655
3742	166	25	22	C	W	43.898	2.853	0.594	0.386	-2.699	-0.589	-0.383	80.926	10.655	17.539
3743	166	25	22	C	W	47.480	2.326	-2.052	0.894	-2.223	2.016	-0.878	71.686	13.473	14.427
3744	166	25	22	C	W	49.107	0.418	0.828	2.819	-0.415	-0.784	-2.669	71.723	14.426	13.472
3745	166	25	22	C	W	49.842	2.281	2.109	0.937	-2.182	-2.070	-0.920	71.729	14.429	13.467
3746	166	25	22	C	W	70.158	2.281	-2.109	0.937	-2.182	2.070	-0.920	71.729	13.467	14.429
3747	166	25	22	C	W	70.893	0.418	-0.828	2.819	-0.415	0.784	-2.669	71.723	14.426	13.472
3748	166	25	22	C	W	72.520	2.326	2.052	0.894	-2.223	-2.016	-0.878	71.686	13.473	14.427
3749	166	25	22	C	W	76.102	2.853	-0.594	0.386	-2.699	0.589	-0.383	80.926	10.655	17.539
3750	166	25	22	C	W	77.696	1.029	2.238	2.187	-1.008	-2.145	-2.096	80.947	17.539	10.653
3751	166	25	22	C	W	81.787	5.011	-3.638	-1.590	-4.554	3.757	1.643	71.599	13.826	14.078
3752	166	25	22	C	W	98.213	5.011	3.638	-1.590	-4.554	-3.757	1.643	71.599	13.826	14.078
3753	166	25	22	C	W	102.304	1.029	-2.238	2.187	-1.008	2.145	-2.096	80.947	10.653	17.539
3754	166	25	22	C	W	103.663	0.353	0.187	2.887	-0.351	-0.177	-2.730	80.938	17.539	10.655
3755	166	25	22	C	W	107.480	2.326	-2.052	0.894	-2.223	2.016	-0.878	71.686	13.473	14.427
3756	166	25	22	C	W	109.107	0.418	0.828	2.819	-0.415	-0.784	-2.669	71.723	14.426	13.472
3757	166	25	22	C	W	109.842	2.281	2.109	0.937	-2.182	-2.070	-0.920	71.729	14.429	13.467
3758	166	25	22	P	S	10.158	2.281	-2.109	0.937	-2.182	2.070	-0.920	71.729	13.467	14.429
3759	166	25	22	P	S	10.893	0.418	-0.828	2.819	-0.415	0.784	-2.669	71.723	14.426	13.472
3760	166	25	22	P	S	12.520	2.326	2.052	0.894	-2.223	-2.016	-0.878	71.686	13.473	14.427
3761	166	25	22	P	S	16.102	2.853	-0.594	0.386	-2.699	0.589	-0.383	80.926	10.655	17.539
3762	166	25	22	P	S	17.696	1.029	2.238	2.187	-1.008	-2.145	-2.096	80.947	17.539	10.653
3763	166	25	22	P	S	38.213	5.011	3.638	-1.590	-4.554	-3.757	1.643	71.599	13.826	14.078
3764	166	25	22	P	S	43.663	0.353	0.187	2.887	-0.351	-0.177	-2.730	80.938	17.539	10.655
3765	166	25	22	P	S	43.898	2.853	0.594	0.386	-2.699	-0.589	-0.383	80.926	10.655	17.539
3766	166	25	22	P	S	47.480	2.326	-2.052	0.894	-2.223	2.016	-0.878	71.686	13.473	14.427
3767	166	25	22	P	S	49.107	0.418	0.828	2.819	-0.415	-0.784	-2.669	71.723	14.426	13.472
3768	166	25	22	P	S	49.842	2.281	2.109	0.937	-2.182	-2.070	-0.920	71.729	14.429	13.467
3769	166	25	22	P	S	70.158	2.281	-2.109	0.937	-2.182	2.070	-0.920	71.729	13.467	14.429
3770	166	25	22	P	S	70.893	0.418	-0.828	2.819	-0.415	0.784	-2.669	71.723	14.426	13.472
3771	166	25	22	P	S	72.520	2.326	2.052	0.894	-2.223	-2.016	-0.878	71.686	13.473	14.427
3772	166	25	22	P	S	76.102	2.853	-0.594	0.386	-2.699	0.589	-0.383	80.926	10.655	17.539
3773	166	25	22	P	S	77.696	1.029	2.238	2.187	-1.008	-2.145	-2.096	80.947	17.539	10.653
3774	166	25	22	P	S	81.787	5.011	-3.638	-1.590	-4.554	3.757	1.643	71.599	13.826	14.078

BL number	Atoms	γ-PC unit cells	WS_2 unit cells	γ-PC origin atom	WS_2 origin atom	Twist-angle (°)	γ-PC strain 1 (%)	γ-PC strain 2 (%)	γ-PC strain 3 (%)	WS_2 strain 1 (%)	WS_2 strain 2 (%)	WS_2 strain 3 (%)	γ (°)	|**a**|(Å)	|**b**|(Å)
3775	166	25	22	P	S	102.304	1.029	-2.238	2.187	-1.008	2.145	-2.096	80.947	10.653	17.539
3776	166	25	22	P	S	103.663	0.353	0.187	2.887	-0.351	-0.177	-2.730	80.938	17.539	10.655
3777	166	25	22	P	S	107.480	2.326	-2.052	0.894	-2.223	2.016	-0.878	71.686	13.473	14.427
3778	166	25	22	P	S	109.107	0.418	0.828	2.819	-0.415	-0.784	-2.669	71.723	14.426	13.472
3779	166	25	22	P	S	109.842	2.281	2.109	0.937	-2.182	-2.070	-0.920	71.729	14.429	13.467
3780	166	25	22	P	W	10.158	2.281	-2.109	0.937	-2.182	2.070	-0.920	71.729	13.467	14.429
3781	166	25	22	P	W	10.893	0.418	-0.828	2.819	-0.415	0.784	-2.669	71.723	14.426	13.472
3782	166	25	22	P	W	12.520	2.326	2.052	0.894	-2.223	-2.016	-0.878	71.686	13.473	14.427
3783	166	25	22	P	W	16.102	2.853	-0.594	0.386	-2.699	0.589	-0.383	80.926	10.655	17.539
3784	166	25	22	P	W	17.696	1.029	2.238	2.187	-1.008	-2.145	-2.096	80.947	17.539	10.653
3785	166	25	22	P	W	21.787	5.011	-3.638	-1.590	-4.554	3.757	1.643	71.599	13.826	14.078
3786	166	25	22	P	W	42.304	1.029	-2.238	2.187	-1.008	2.145	-2.096	80.947	10.653	17.539
3787	166	25	22	P	W	43.663	0.353	0.187	2.887	-0.351	-0.177	-2.730	80.938	17.539	10.655
3788	166	25	22	P	W	47.480	2.326	-2.052	0.894	-2.223	2.016	-0.878	71.686	13.473	14.427
3789	166	25	22	P	W	49.107	0.418	0.828	2.819	-0.415	-0.784	-2.669	71.723	14.426	13.472
3790	166	25	22	P	W	49.842	2.281	2.109	0.937	-2.182	-2.070	-0.920	71.729	14.429	13.467
3791	166	25	22	P	W	70.158	2.281	-2.109	0.937	-2.182	2.070	-0.920	71.729	13.467	14.429
3792	166	25	22	P	W	70.893	0.418	-0.828	2.819	-0.415	0.784	-2.669	71.723	14.426	13.472
3793	166	25	22	P	W	72.520	2.326	2.052	0.894	-2.223	-2.016	-0.878	71.686	13.473	14.427
3794	166	25	22	P	W	76.102	2.853	-0.594	0.386	-2.699	0.589	-0.383	80.926	10.655	17.539
3795	166	25	22	P	W	77.696	1.029	2.238	2.187	-1.008	-2.145	-2.096	80.947	17.539	10.653
3796	166	25	22	P	W	98.213	5.011	3.638	-1.590	-4.554	-3.757	1.643	71.599	13.826	14.078
3797	166	25	22	P	W	103.663	0.353	0.187	2.887	-0.351	-0.177	-2.730	80.938	17.539	10.655
3798	166	25	22	P	W	103.898	2.853	0.594	0.386	-2.699	-0.589	-0.383	80.926	10.655	17.539
3799	166	25	22	P	W	107.480	2.326	-2.052	0.894	-2.223	2.016	-0.878	71.686	13.473	14.427
3800	166	25	22	P	W	109.107	0.418	0.828	2.819	-0.415	-0.784	-2.669	71.723	14.426	13.472
3801	166	25	22	P	W	109.842	2.281	2.109	0.937	-2.182	-2.070	-0.920	71.729	14.429	13.467
3802	167	26	21	C	S	9.183	3.582	-3.637	-4.383	-3.342	3.986	4.804	87.822	18.586	9.912
3803	167	26	21	C	S	9.515	-4.497	-3.344	3.716	4.941	3.113	-3.459	76.682	11.961	15.819
3804	167	26	21	C	S	10.893	4.020	-0.770	-4.753	-3.721	0.851	5.252	76.807	15.815	11.961
3805	167	26	21	C	S	13.898	-4.228	-4.608	3.401	4.619	4.314	-3.184	87.448	9.921	18.572
3806	167	26	21	C	S	17.480	-4.685	1.894	3.938	5.169	-1.755	-3.651	70.796	14.491	13.456
3807	167	26	21	C	S	18.613	3.582	3.637	-4.383	-3.342	-3.986	4.804	87.822	18.586	9.912
3808	167	26	21	C	S	27.126	1.029	-2.820	-2.101	-1.008	2.943	2.193	83.797	19.034	9.715
3809	167	26	21	C	S	30.000	-2.359	-2.168	1.306	2.476	2.113	-1.272	83.892	9.718	19.028
3810	167	26	21	C	S	32.874	1.029	2.820	-2.101	-1.008	-2.943	2.193	83.797	19.034	9.715
3811	167	26	21	C	S	41.387	3.582	-3.637	-4.383	-3.342	3.986	4.804	87.822	18.586	9.913
3812	167	26	21	C	S	42.520	-4.685	-1.894	3.938	5.169	1.755	-3.651	70.796	14.491	13.456
3813	167	26	21	C	S	46.102	-4.228	-4.608	3.401	4.619	4.314	-3.184	87.448	9.921	18.572
3814	167	26	21	C	S	49.107	4.020	0.770	-4.753	-3.721	-0.851	5.252	76.807	15.815	11.961
3815	167	26	21	C	S	50.485	-4.497	3.344	3.716	4.941	-3.113	-3.459	76.682	11.961	15.819
3816	167	26	21	C	S	50.817	3.582	3.637	-4.383	-3.342	-3.986	4.804	87.822	18.586	9.912
3817	167	26	21	C	S	69.183	3.582	-3.637	-4.383	-3.342	3.986	4.804	87.822	18.586	9.912
3818	167	26	21	C	S	69.515	-4.497	-3.344	3.716	4.941	3.113	-3.459	76.682	11.961	15.819
3819	167	26	21	C	S	70.893	4.020	-0.770	-4.753	-3.721	0.851	5.252	76.807	15.815	11.961
3820	167	26	21	C	S	73.898	-4.228	-4.608	3.401	4.619	4.314	-3.184	87.448	9.921	18.572
3821	167	26	21	C	S	77.480	-4.685	1.894	3.938	5.169	-1.755	-3.651	70.796	14.491	13.456
3822	167	26	21	C	S	78.613	3.582	3.637	-4.383	-3.342	-3.986	4.804	87.822	18.586	9.912
3823	167	26	21	C	S	87.126	1.029	-2.820	-2.101	-1.008	2.943	2.193	83.797	19.034	9.715
3824	167	26	21	C	S	90.000	-2.359	-2.168	1.306	2.476	2.113	-1.272	83.892	9.718	19.027
3825	167	26	21	C	S	92.874	1.029	2.820	-2.101	-1.008	-2.943	2.193	83.797	19.034	9.715

BL number	Atoms	γ-PC unit cells	WS_2 unit cells	γ-PC origin atom	WS_2 origin atom	Twist-angle (°)	γ-PC strain 1 (%)	γ-PC strain 2 (%)	γ-PC strain 3 (%)	WS_2 strain 1 (%)	WS_2 strain 2 (%)	WS_2 strain 3 (%)	γ (°)	$\vert\mathbf{a}\vert$(Å)	$\vert\mathbf{b}\vert$(Å)
3826	167	26	21	C	S	101.387	3.582	-3.637	-4.383	-3.342	3.986	4.804	87.822	18.586	9.912
3827	167	26	21	C	S	102.520	-4.685	-1.894	3.938	5.169	1.755	-3.651	70.796	14.491	13.456
3828	167	26	21	C	S	106.102	-4.228	-4.608	3.401	4.619	4.314	-3.184	87.448	9.921	18.572
3829	167	26	21	C	S	109.107	4.020	0.770	-4.753	-3.721	-0.851	5.252	76.807	15.815	11.961
3830	167	26	21	C	S	110.485	-4.497	3.344	3.716	4.941	-3.113	-3.459	76.682	11.961	15.819
3831	167	26	21	C	S	110.817	3.582	3.637	-4.383	-3.342	-3.986	4.804	87.822	18.586	9.912
3832	167	26	21	C	W	9.183	3.582	-3.637	-4.383	-3.342	3.986	4.804	87.822	18.586	9.912
3833	167	26	21	C	W	9.515	-4.497	-3.344	3.716	4.941	3.113	-3.459	76.682	11.961	15.819
3834	167	26	21	C	W	10.893	4.020	-0.770	-4.753	-3.721	0.851	5.252	76.807	15.815	11.961
3835	167	26	21	C	W	13.898	-4.228	-4.608	3.401	4.619	4.314	-3.184	87.448	9.921	18.572
3836	167	26	21	C	W	17.480	-4.685	1.894	3.938	5.169	-1.755	-3.651	70.796	14.491	13.456
3837	167	26	21	C	W	18.613	3.582	3.637	-4.383	-3.342	-3.986	4.804	87.822	18.586	9.912
3838	167	26	21	C	W	27.126	1.029	-2.820	-2.101	-1.008	2.943	2.193	83.797	19.034	9.715
3839	167	26	21	C	W	30.000	-2.359	-2.168	1.306	2.476	2.113	-1.272	83.892	9.718	19.028
3840	167	26	21	C	W	32.874	1.029	2.820	-2.101	-1.008	-2.943	2.193	83.797	19.034	9.715
3841	167	26	21	C	W	41.387	3.582	-3.637	-4.383	-3.342	3.986	4.804	87.822	18.586	9.913
3842	167	26	21	C	W	42.520	-4.685	-1.894	3.938	5.169	1.755	-3.651	70.796	14.491	13.456
3843	167	26	21	C	W	46.102	-4.228	-4.608	3.401	4.619	4.314	-3.184	87.448	9.921	18.572
3844	167	26	21	C	W	49.107	4.020	0.770	-4.753	-3.721	-0.851	5.252	76.807	15.815	11.961
3845	167	26	21	C	W	50.485	-4.497	3.344	3.716	4.941	-3.113	-3.459	76.682	11.961	15.819
3846	167	26	21	C	W	50.817	3.582	3.637	-4.383	-3.342	-3.986	4.804	87.822	18.586	9.912
3847	167	26	21	C	W	69.183	3.582	-3.637	-4.383	-3.342	3.986	4.804	87.822	18.586	9.912
3848	167	26	21	C	W	69.515	-4.497	-3.344	3.716	4.941	3.113	-3.459	76.682	11.961	15.819
3849	167	26	21	C	W	70.893	4.020	-0.770	-4.753	-3.721	0.851	5.252	76.807	15.815	11.961
3850	167	26	21	C	W	73.898	-4.228	-4.608	3.401	4.619	4.314	-3.184	87.448	9.921	18.572
3851	167	26	21	C	W	77.480	-4.685	1.894	3.938	5.169	-1.755	-3.651	70.796	14.491	13.456
3852	167	26	21	C	W	78.613	3.582	3.637	-4.383	-3.342	-3.986	4.804	87.822	18.586	9.912
3853	167	26	21	C	W	87.126	1.029	-2.820	-2.101	-1.008	2.943	2.193	83.797	19.034	9.715
3854	167	26	21	C	W	90.000	-2.359	-2.168	1.306	2.476	2.113	-1.272	83.892	9.718	19.027
3855	167	26	21	C	W	92.874	1.029	2.820	-2.101	-1.008	-2.943	2.193	83.797	19.034	9.715
3856	167	26	21	C	W	101.387	3.582	-3.637	-4.383	-3.342	3.986	4.804	87.822	18.586	9.912
3857	167	26	21	C	W	102.520	-4.685	-1.894	3.938	5.169	1.755	-3.651	70.796	14.491	13.456
3858	167	26	21	C	W	106.102	-4.228	-4.608	3.401	4.619	4.314	-3.184	87.448	9.921	18.572
3859	167	26	21	C	W	109.107	4.020	0.770	-4.753	-3.721	-0.851	5.252	76.807	15.815	11.961
3860	167	26	21	C	W	110.485	-4.497	3.344	3.716	4.941	-3.113	-3.459	76.682	11.961	15.819
3861	167	26	21	C	W	110.817	3.582	3.637	-4.383	-3.342	-3.986	4.804	87.822	18.586	9.912
3862	167	26	21	P	S	9.183	3.582	-3.637	-4.383	-3.342	3.986	4.804	87.822	18.586	9.912
3863	167	26	21	P	S	9.515	-4.497	-3.344	3.716	4.941	3.113	-3.459	76.682	11.961	15.819
3864	167	26	21	P	S	10.893	4.020	-0.770	-4.753	-3.721	0.851	5.252	76.807	15.815	11.961
3865	167	26	21	P	S	13.898	-4.228	-4.608	3.401	4.619	4.314	-3.184	87.448	9.921	18.572
3866	167	26	21	P	S	17.480	-4.685	1.894	3.938	5.169	-1.755	-3.651	70.796	14.491	13.456
3867	167	26	21	P	S	18.613	3.582	3.637	-4.383	-3.342	-3.986	4.804	87.822	18.586	9.912
3868	167	26	21	P	S	41.387	3.582	-3.637	-4.383	-3.342	3.986	4.804	87.822	18.586	9.913
3869	167	26	21	P	S	42.520	-4.685	-1.894	3.938	5.169	1.755	-3.651	70.796	14.491	13.456
3870	167	26	21	P	S	46.102	-4.228	-4.608	3.401	4.619	4.314	-3.184	87.448	9.921	18.572
3871	167	26	21	P	S	49.107	4.020	0.770	-4.753	-3.721	-0.851	5.252	76.807	15.815	11.961
3872	167	26	21	P	S	50.485	-4.497	3.344	3.716	4.941	-3.113	-3.459	76.682	11.961	15.819
3873	167	26	21	P	S	50.817	3.582	3.637	-4.383	-3.342	-3.986	4.804	87.822	18.586	9.912
3874	167	26	21	P	S	69.183	3.582	-3.637	-4.383	-3.342	3.986	4.804	87.822	18.586	9.912
3875	167	26	21	P	S	69.515	-4.497	-3.344	3.716	4.941	3.113	-3.459	76.682	11.961	15.819
3876	167	26	21	P	S	70.893	4.020	-0.770	-4.753	-3.721	0.851	5.252	76.807	15.815	11.961

BL number	Atoms	γ-PC unit cells	WS_2 unit cells	γ-PC origin atom	WS_2 origin atom	Twist-angle (°)	γ-PC strain 1 (%)	γ-PC strain 2 (%)	γ-PC strain 3 (%)	WS_2 strain 1 (%)	WS_2 strain 2 (%)	WS_2 strain 3 (%)	γ (°)	|**a**|(Å)	|**b**|(Å)
3877	167	26	21	P	S	73.898	-4.228	-4.608	3.401	4.619	4.314	-3.184	87.448	9.921	18.572
3878	167	26	21	P	S	77.480	-4.685	1.894	3.938	5.169	-1.755	-3.651	70.796	14.491	13.456
3879	167	26	21	P	S	78.613	3.582	3.637	-4.383	-3.342	-3.986	4.804	87.822	18.586	9.912
3880	167	26	21	P	S	101.387	3.582	-3.637	-4.383	-3.342	3.986	4.804	87.822	18.586	9.912
3881	167	26	21	P	S	102.520	-4.685	-1.894	3.938	5.169	1.755	-3.651	70.796	14.491	13.456
3882	167	26	21	P	S	106.102	-4.228	-4.608	3.401	4.619	4.314	-3.184	87.448	9.921	18.572
3883	167	26	21	P	S	109.107	4.020	0.770	-4.753	-3.721	-0.851	5.252	76.807	15.815	11.961
3884	167	26	21	P	S	110.485	-4.497	3.344	3.716	4.941	-3.113	-3.459	76.682	11.961	15.819
3885	167	26	21	P	S	110.817	3.582	3.637	-4.383	-3.342	-3.986	4.804	87.822	18.586	9.912
3886	167	26	21	P	W	9.183	3.582	-3.637	-4.383	-3.342	3.986	4.804	87.822	18.586	9.912
3887	167	26	21	P	W	9.515	-4.497	-3.344	3.716	4.941	3.113	-3.459	76.682	11.961	15.819
3888	167	26	21	P	W	10.893	4.020	-0.770	-4.753	-3.721	0.851	5.252	76.807	15.815	11.961
3889	167	26	21	P	W	13.898	-4.228	-4.608	3.401	4.619	4.314	-3.184	87.448	9.921	18.572
3890	167	26	21	P	W	17.480	-4.685	1.894	3.938	5.169	-1.755	-3.651	70.796	14.491	13.456
3891	167	26	21	P	W	18.613	3.582	3.637	-4.383	-3.342	-3.986	4.804	87.822	18.586	9.912
3892	167	26	21	P	W	41.387	3.582	-3.637	-4.383	-3.342	3.986	4.804	87.822	18.586	9.913
3893	167	26	21	P	W	42.520	-4.685	-1.894	3.938	5.169	1.755	-3.651	70.796	14.491	13.456
3894	167	26	21	P	W	46.102	-4.228	-4.608	3.401	4.619	4.314	-3.184	87.448	9.921	18.572
3895	167	26	21	P	W	49.107	4.020	0.770	-4.753	-3.721	-0.851	5.252	76.807	15.815	11.961
3896	167	26	21	P	W	50.485	-4.497	3.344	3.716	4.941	-3.113	-3.459	76.682	11.961	15.819
3897	167	26	21	P	W	50.817	3.582	3.637	-4.383	-3.342	-3.986	4.804	87.822	18.586	9.912
3898	167	26	21	P	W	69.183	3.582	-3.637	-4.383	-3.342	3.986	4.804	87.822	18.586	9.912
3899	167	26	21	P	W	69.515	-4.497	-3.344	3.716	4.941	3.113	-3.459	76.682	11.961	15.819
3900	167	26	21	P	W	70.893	4.020	-0.770	-4.753	-3.721	0.851	5.252	76.807	15.815	11.961
3901	167	26	21	P	W	73.898	-4.228	-4.608	3.401	4.619	4.314	-3.184	87.448	9.921	18.572
3902	167	26	21	P	W	77.480	-4.685	1.894	3.938	5.169	-1.755	-3.651	70.796	14.491	13.456
3903	167	26	21	P	W	78.613	3.582	3.637	-4.383	-3.342	-3.986	4.804	87.822	18.586	9.912
3904	167	26	21	P	W	101.387	3.582	-3.637	-4.383	-3.342	3.986	4.804	87.822	18.586	9.912
3905	167	26	21	P	W	102.520	-4.685	-1.894	3.938	5.169	1.755	-3.651	70.796	14.491	13.456
3906	167	26	21	P	W	106.102	-4.228	-4.608	3.401	4.619	4.314	-3.184	87.448	9.921	18.572
3907	167	26	21	P	W	109.107	4.020	0.770	-4.753	-3.721	-0.851	5.252	76.807	15.815	11.961
3908	167	26	21	P	W	110.485	-4.497	3.344	3.716	4.941	-3.113	-3.459	76.682	11.961	15.819
3909	167	26	21	P	W	110.817	3.582	3.637	-4.383	-3.342	-3.986	4.804	87.822	18.586	9.912
3910	168	24	24	C	S	13.174	5.011	2.526	5.011	-4.554	-2.296	-4.555	65.436	13.151	15.818
3911	168	24	24	C	S	27.796	5.011	2.526	5.011	-4.554	-2.296	-4.555	72.634	10.878	18.223
3912	168	24	24	C	S	32.204	5.011	-2.526	5.011	-4.554	2.296	-4.555	72.634	10.878	18.223
3913	168	24	24	C	S	46.827	5.011	-2.526	5.011	-4.554	2.296	-4.555	65.436	13.151	15.818
3914	168	24	24	C	S	73.174	5.011	2.526	5.011	-4.554	-2.296	-4.555	65.436	13.151	15.818
3915	168	24	24	C	S	87.796	5.011	2.526	5.011	-4.554	-2.296	-4.555	72.634	10.878	18.223
3916	168	24	24	C	S	92.204	5.011	-2.526	5.011	-4.554	2.296	-4.555	72.634	10.878	18.223
3917	168	24	24	C	S	106.827	5.011	-2.526	5.011	-4.554	2.296	-4.555	65.436	13.151	15.818
3918	168	24	24	C	W	13.174	5.011	2.526	5.011	-4.554	-2.296	-4.555	65.436	13.151	15.818
3919	168	24	24	C	W	27.796	5.011	2.526	5.011	-4.554	-2.296	-4.555	72.634	10.878	18.223
3920	168	24	24	C	W	32.204	5.011	-2.526	5.011	-4.554	2.296	-4.555	72.634	10.878	18.223
3921	168	24	24	C	W	46.827	5.011	-2.526	5.011	-4.554	2.296	-4.555	65.436	13.151	15.818
3922	168	24	24	C	W	73.174	5.011	2.526	5.011	-4.554	-2.296	-4.555	65.436	13.151	15.818
3923	168	24	24	C	W	87.796	5.011	2.526	5.011	-4.554	-2.296	-4.555	72.634	10.878	18.223
3924	168	24	24	C	W	92.204	5.011	-2.526	5.011	-4.554	2.296	-4.555	72.634	10.878	18.223
3925	168	24	24	C	W	106.827	5.011	-2.526	5.011	-4.554	2.296	-4.555	65.436	13.151	15.818
3926	168	24	24	P	S	13.174	5.011	2.526	5.011	-4.554	-2.296	-4.555	65.436	13.151	15.818
3927	168	24	24	P	S	27.796	5.011	2.526	5.011	-4.554	-2.296	-4.555	72.634	10.878	18.223

BL number	Atoms	γ-PC unit cells	WS_2 unit cells	γ-PC origin atom	WS_2 origin atom	Twist-angle (°)	γ-PC strain 1 (%)	γ-PC strain 2 (%)	γ-PC strain 3 (%)	WS_2 strain 1 (%)	WS_2 strain 2 (%)	WS_2 strain 3 (%)	γ (°)	|**a**|(Å)	|**b**|(Å)
3928	168	24	24	P	S	32.204	5.011	-2.526	5.011	-4.554	2.296	-4.555	72.634	10.878	18.223
3929	168	24	24	P	S	46.827	5.011	-2.526	5.011	-4.554	2.296	-4.555	65.436	13.151	15.818
3930	168	24	24	P	S	73.174	5.011	2.526	5.011	-4.554	-2.296	-4.555	65.436	13.151	15.818
3931	168	24	24	P	S	87.796	5.011	2.526	5.011	-4.554	-2.296	-4.555	72.634	10.878	18.223
3932	168	24	24	P	S	92.204	5.011	-2.526	5.011	-4.554	2.296	-4.555	72.634	10.878	18.223
3933	168	24	24	P	S	106.827	5.011	-2.526	5.011	-4.554	2.296	-4.555	65.436	13.151	15.818
3934	168	24	24	P	W	13.174	5.011	2.526	5.011	-4.554	-2.296	-4.555	65.436	13.151	15.818
3935	168	24	24	P	W	27.796	5.011	2.526	5.011	-4.554	-2.296	-4.555	72.634	10.878	18.223
3936	168	24	24	P	W	32.204	5.011	-2.526	5.011	-4.554	2.296	-4.555	72.634	10.878	18.223
3937	168	24	24	P	W	46.827	5.011	-2.526	5.011	-4.554	2.296	-4.555	65.436	13.151	15.818
3938	168	24	24	P	W	73.174	5.011	2.526	5.011	-4.554	-2.296	-4.555	65.436	13.151	15.818
3939	168	24	24	P	W	87.796	5.011	2.526	5.011	-4.554	-2.296	-4.555	72.634	10.878	18.223
3940	168	24	24	P	W	92.204	5.011	-2.526	5.011	-4.554	2.296	-4.555	72.634	10.878	18.223
3941	168	24	24	P	W	106.827	5.011	-2.526	5.011	-4.554	2.296	-4.555	65.436	13.151	15.818
3942	168	27	20	C	S	6.587	-3.853	-3.409	-1.424	4.175	3.508	1.466	81.294	14.354	12.894
3943	168	27	20	C	S	7.154	-0.955	-2.357	-4.295	0.973	2.578	4.698	81.212	12.900	14.352
3944	168	27	20	C	S	8.948	-0.599	0.638	-4.624	0.606	-0.703	5.095	74.197	15.901	11.960
3945	168	27	20	C	S	9.515	-4.497	1.649	-0.737	4.941	-1.674	0.748	74.142	11.961	15.904
3946	168	27	20	C	S	10.893	-1.983	3.930	-3.316	2.064	-4.209	3.552	81.220	12.906	14.342
3947	168	27	20	C	S	19.842	-4.723	-4.802	-0.491	5.216	4.850	0.496	72.546	15.242	12.587
3948	168	27	20	C	S	25.285	-4.781	4.683	-0.427	5.287	-4.723	0.430	72.851	15.207	12.595
3949	168	27	20	C	S	34.715	-4.781	-4.683	-0.427	5.287	4.723	0.430	72.851	12.595	15.207
3950	168	27	20	C	S	40.158	-4.723	4.802	-0.491	5.216	-4.850	0.496	72.546	15.242	12.587
3951	168	27	20	C	S	49.107	-1.983	-3.930	-3.316	2.064	4.209	3.552	81.220	12.906	14.342
3952	168	27	20	C	S	50.485	-4.497	-1.649	-0.737	4.941	1.674	0.748	74.142	11.961	15.904
3953	168	27	20	C	S	51.052	-0.599	-0.638	-4.624	0.606	0.703	5.095	74.197	15.901	11.960
3954	168	27	20	C	S	52.846	-0.955	2.357	-4.295	0.973	-2.579	4.698	81.212	14.352	12.900
3955	168	27	20	C	S	53.413	-3.853	3.409	-1.424	4.175	-3.508	1.466	81.294	14.354	12.894
3956	168	27	20	C	S	53.822	-2.145	4.036	-3.158	2.241	-4.308	3.371	74.233	11.951	15.904
3957	168	27	20	C	S	66.178	-2.145	-4.035	-3.158	2.241	4.307	3.371	74.233	15.904	11.951
3958	168	27	20	C	S	66.587	-3.853	-3.409	-1.424	4.175	3.508	1.466	81.294	14.354	12.894
3959	168	27	20	C	S	67.154	-0.955	-2.357	-4.295	0.973	2.578	4.698	81.212	12.900	14.352
3960	168	27	20	C	S	68.948	-0.599	0.638	-4.624	0.606	-0.703	5.095	74.197	15.901	11.960
3961	168	27	20	C	S	69.515	-4.497	1.649	-0.737	4.941	-1.674	0.748	74.142	11.961	15.904
3962	168	27	20	C	S	70.893	-1.983	3.930	-3.316	2.064	-4.209	3.552	81.220	12.906	14.342
3963	168	27	20	C	S	79.842	-4.723	-4.802	-0.491	5.216	4.850	0.496	72.546	15.242	12.587
3964	168	27	20	C	S	85.285	-4.781	4.683	-0.427	5.287	-4.723	0.430	72.851	15.207	12.595
3965	168	27	20	C	S	94.715	-4.781	-4.683	-0.427	5.287	4.723	0.430	72.851	12.595	15.207
3966	168	27	20	C	S	100.158	-4.723	4.802	-0.491	5.216	-4.850	0.496	72.546	15.242	12.587
3967	168	27	20	C	S	109.107	-1.983	-3.930	-3.316	2.064	4.209	3.552	81.220	12.906	14.342
3968	168	27	20	C	S	110.485	-4.497	-1.649	-0.737	4.941	1.674	0.748	74.142	11.961	15.904
3969	168	27	20	C	S	111.052	-0.599	-0.638	-4.624	0.606	0.703	5.095	74.197	15.901	11.960
3970	168	27	20	C	S	112.846	-0.955	2.357	-4.295	0.973	-2.579	4.698	81.212	14.352	12.900
3971	168	27	20	C	S	113.413	-3.853	3.409	-1.424	4.175	-3.508	1.466	81.294	14.354	12.894
3972	168	27	20	C	S	113.822	-2.145	4.036	-3.158	2.241	-4.308	3.371	74.233	11.951	15.904
3973	168	27	20	C	W	6.178	-2.145	-4.035	-3.158	2.241	4.307	3.371	74.233	15.904	11.951
3974	168	27	20	C	W	6.587	-3.853	-3.409	-1.424	4.175	3.508	1.466	81.294	14.354	12.894
3975	168	27	20	C	W	7.154	-0.955	-2.357	-4.295	0.973	2.578	4.698	81.212	12.900	14.352
3976	168	27	20	C	W	10.893	-1.983	3.930	-3.316	2.064	-4.209	3.552	81.220	12.906	14.342
3977	168	27	20	C	W	19.842	-4.723	-4.802	-0.491	5.216	4.850	0.496	72.546	15.242	12.587
3978	168	27	20	C	W	25.285	-4.781	4.683	-0.427	5.287	-4.723	0.430	72.851	15.207	12.595

BL number	Atoms	γ-PC unit cells	WS_2 unit cells	γ-PC origin atom	WS_2 origin atom	Twist-angle (°)	γ-PC strain 1 (%)	γ-PC strain 2 (%)	γ-PC strain 3 (%)	WS_2 strain 1 (%)	WS_2 strain 2 (%)	WS_2 strain 3 (%)	γ (°)	|**a**|(Å)	|**b**|(Å)
3979	168	27	20	C	W	34.715	-4.781	-4.683	-0.427	5.287	4.723	0.430	72.851	12.595	15.207
3980	168	27	20	C	W	40.158	-4.723	4.802	-0.491	5.216	-4.850	0.496	72.546	15.242	12.587
3981	168	27	20	C	W	49.107	-1.983	-3.930	-3.316	2.064	4.209	3.552	81.220	12.906	14.342
3982	168	27	20	C	W	52.846	-0.955	2.357	-4.295	0.973	-2.579	4.698	81.212	14.352	12.900
3983	168	27	20	C	W	53.413	-3.853	3.409	-1.424	4.175	-3.508	1.466	81.294	14.354	12.894
3984	168	27	20	C	W	66.587	-3.853	-3.409	-1.424	4.175	3.508	1.466	81.294	14.354	12.894
3985	168	27	20	C	W	67.154	-0.955	-2.357	-4.295	0.973	2.578	4.698	81.212	12.900	14.352
3986	168	27	20	C	W	70.893	-1.983	3.930	-3.316	2.064	-4.209	3.552	81.220	12.906	14.342
3987	168	27	20	C	W	79.842	-4.723	-4.802	-0.491	5.216	4.850	0.496	72.546	15.242	12.587
3988	168	27	20	C	W	85.285	-4.781	4.683	-0.427	5.287	-4.723	0.430	72.851	15.207	12.595
3989	168	27	20	C	W	94.715	-4.781	-4.683	-0.427	5.287	4.723	0.430	72.851	12.595	15.207
3990	168	27	20	C	W	100.158	-4.723	4.802	-0.491	5.216	-4.850	0.496	72.546	15.242	12.587
3991	168	27	20	C	W	109.107	-1.983	-3.930	-3.316	2.064	4.209	3.552	81.220	12.906	14.342
3992	168	27	20	C	W	112.846	-0.955	2.357	-4.295	0.973	-2.579	4.698	81.212	14.352	12.900
3993	168	27	20	C	W	113.413	-3.853	3.409	-1.424	4.175	-3.508	1.466	81.294	14.354	12.894
3994	168	27	20	P	S	8.948	-0.599	0.638	-4.624	0.606	-0.703	5.095	74.197	15.901	11.960
3995	168	27	20	P	S	9.515	-4.497	1.649	-0.737	4.941	-1.674	0.748	74.142	11.961	15.904
3996	168	27	20	P	S	19.842	-4.723	-4.802	-0.491	5.216	4.850	0.496	72.546	15.242	12.587
3997	168	27	20	P	S	25.285	-4.781	4.683	-0.427	5.287	-4.723	0.430	72.851	15.207	12.595
3998	168	27	20	P	S	34.715	-4.781	-4.683	-0.427	5.287	4.723	0.430	72.851	12.595	15.207
3999	168	27	20	P	S	40.158	-4.723	4.802	-0.491	5.216	-4.850	0.496	72.546	15.242	12.587
4000	168	27	20	P	S	49.107	-1.983	-3.930	-3.316	2.064	4.209	3.552	81.220	12.906	14.342
4001	168	27	20	P	S	50.485	-4.497	-1.649	-0.737	4.941	1.674	0.748	74.142	11.961	15.904
4002	168	27	20	P	S	51.052	-0.599	-0.638	-4.624	0.606	0.703	5.095	74.197	15.901	11.960
4003	168	27	20	P	S	52.846	-0.955	2.357	-4.295	0.973	-2.579	4.698	81.212	14.352	12.900
4004	168	27	20	P	S	53.413	-3.853	3.409	-1.424	4.175	-3.508	1.466	81.294	14.354	12.894
4005	168	27	20	P	S	53.822	-2.145	4.036	-3.158	2.241	-4.308	3.371	74.233	11.951	15.904
4006	168	27	20	P	S	66.178	-2.145	-4.035	-3.158	2.241	4.307	3.371	74.233	15.904	11.951
4007	168	27	20	P	S	66.587	-3.853	-3.409	-1.424	4.175	3.508	1.466	81.294	14.354	12.894
4008	168	27	20	P	S	67.154	-0.955	-2.357	-4.295	0.973	2.578	4.698	81.212	12.900	14.352
4009	168	27	20	P	S	68.948	-0.599	0.638	-4.624	0.606	-0.703	5.095	74.197	15.901	11.960
4010	168	27	20	P	S	69.515	-4.497	1.649	-0.737	4.941	-1.674	0.748	74.142	11.961	15.904
4011	168	27	20	P	S	70.893	-1.983	3.930	-3.316	2.064	-4.209	3.552	81.220	12.906	14.342
4012	168	27	20	P	S	79.842	-4.723	-4.802	-0.491	5.216	4.850	0.496	72.546	15.242	12.587
4013	168	27	20	P	S	85.285	-4.781	4.683	-0.427	5.287	-4.723	0.430	72.851	15.207	12.595
4014	168	27	20	P	S	94.715	-4.781	-4.683	-0.427	5.287	4.723	0.430	72.851	12.595	15.207
4015	168	27	20	P	S	100.158	-4.723	4.802	-0.491	5.216	-4.850	0.496	72.546	15.242	12.587
4016	168	27	20	P	S	110.485	-4.497	-1.649	-0.737	4.941	1.674	0.748	74.142	11.961	15.904
4017	168	27	20	P	S	111.052	-0.599	-0.638	-4.624	0.606	0.703	5.095	74.197	15.901	11.960
4018	168	27	20	P	S	113.822	-2.145	4.036	-3.158	2.241	-4.308	3.371	74.233	11.951	15.904
4019	168	27	20	P	W	6.178	-2.145	-4.035	-3.158	2.241	4.307	3.371	74.233	15.904	11.951
4020	168	27	20	P	W	6.587	-3.853	-3.409	-1.424	4.175	3.508	1.466	81.294	14.354	12.894
4021	168	27	20	P	W	7.154	-0.955	-2.357	-4.295	0.973	2.578	4.698	81.212	12.900	14.352
4022	168	27	20	P	W	10.893	-1.983	3.930	-3.316	2.064	-4.209	3.552	81.220	12.906	14.342
4023	168	27	20	P	W	19.842	-4.723	-4.802	-0.491	5.216	4.850	0.496	72.546	15.242	12.587
4024	168	27	20	P	W	25.285	-4.781	4.683	-0.427	5.287	-4.723	0.430	72.851	15.207	12.595
4025	168	27	20	P	W	34.715	-4.781	-4.683	-0.427	5.287	4.723	0.430	72.851	12.595	15.207
4026	168	27	20	P	W	40.158	-4.723	4.802	-0.491	5.216	-4.850	0.496	72.546	15.242	12.587
4027	168	27	20	P	W	79.842	-4.723	-4.802	-0.491	5.216	4.850	0.496	72.546	15.242	12.587
4028	168	27	20	P	W	85.285	-4.781	4.683	-0.427	5.287	-4.723	0.430	72.851	15.207	12.595
4029	168	27	20	P	W	94.715	-4.781	-4.683	-0.427	5.287	4.723	0.430	72.851	12.595	15.207

BL number	Atoms	γ-PC unit cells	WS_2 unit cells	γ-PC origin atom	WS_2 origin atom	Twist-angle (°)	γ-PC strain 1 (%)	γ-PC strain 2 (%)	γ-PC strain 3 (%)	WS_2 strain 1 (%)	WS_2 strain 2 (%)	WS_2 strain 3 (%)	γ (°)	|**a**|(Å)	|**b**|(Å)
4030	168	27	20	P	W	100.158	-4.723	4.802	-0.491	5.216	-4.850	0.496	72.546	15.242	12.587
4031	168	27	20	P	W	109.107	-1.983	-3.930	-3.316	2.064	4.209	3.552	81.220	12.906	14.342
4032	168	27	20	P	W	112.846	-0.955	2.357	-4.295	0.973	-2.579	4.698	81.212	14.352	12.900
4033	168	27	20	P	W	113.413	-3.853	3.409	-1.424	4.175	-3.508	1.466	81.294	14.354	12.894
4034	169	25	23	C	S	9.183	3.582	-4.655	1.960	-3.342	4.479	-1.886	84.636	13.133	14.436
4035	169	25	23	C	S	10.893	0.418	-1.656	5.220	-0.415	1.500	-4.727	84.604	14.426	13.149
4036	169	25	23	C	S	13.174	5.011	2.425	0.610	-4.554	-2.396	-0.603	84.493	13.151	14.425
4037	169	25	23	C	S	23.691	2.390	-4.911	3.142	-2.281	4.621	-2.956	81.894	10.867	17.545
4038	169	25	23	C	S	25.767	0.353	-1.196	5.291	-0.351	1.082	-4.785	81.878	17.539	10.877
4039	169	25	23	C	S	27.796	5.011	2.425	0.610	-4.554	-2.396	-0.603	81.771	10.878	17.539
4040	169	25	23	C	S	32.204	5.011	-2.425	0.610	-4.554	2.396	-0.603	81.771	10.878	17.539
4041	169	25	23	C	S	34.233	0.353	1.196	5.291	-0.351	-1.082	-4.785	81.878	17.539	10.877
4042	169	25	23	C	S	36.309	2.390	4.911	3.142	-2.281	-4.621	-2.956	81.894	17.545	10.867
4043	169	25	23	C	S	46.827	5.011	-2.425	0.610	-4.554	2.396	-0.603	84.493	13.151	14.425
4044	169	25	23	C	S	49.107	0.418	1.657	5.220	-0.415	-1.500	-4.727	84.604	14.426	13.149
4045	169	25	23	C	S	50.817	3.582	4.655	1.960	-3.342	-4.479	-1.886	84.636	14.436	13.133
4046	169	25	23	C	S	69.183	3.582	-4.655	1.960	-3.342	4.479	-1.886	84.636	13.133	14.436
4047	169	25	23	C	S	70.893	0.418	-1.656	5.220	-0.415	1.500	-4.727	84.604	14.426	13.149
4048	169	25	23	C	S	73.174	5.011	2.425	0.610	-4.554	-2.396	-0.603	84.493	13.151	14.425
4049	169	25	23	C	S	83.691	2.390	-4.911	3.142	-2.281	4.621	-2.956	81.894	10.867	17.545
4050	169	25	23	C	S	85.767	0.353	-1.196	5.291	-0.351	1.082	-4.785	81.878	17.539	10.877
4051	169	25	23	C	S	87.796	5.011	2.425	0.610	-4.554	-2.396	-0.603	81.771	10.878	17.539
4052	169	25	23	C	S	92.204	5.011	-2.425	0.610	-4.554	2.396	-0.603	81.771	10.878	17.539
4053	169	25	23	C	S	94.233	0.353	1.196	5.291	-0.351	-1.082	-4.785	81.878	17.539	10.877
4054	169	25	23	C	S	96.309	2.390	4.911	3.142	-2.281	-4.621	-2.956	81.894	17.545	10.867
4055	169	25	23	C	S	106.827	5.011	-2.425	0.610	-4.554	2.396	-0.603	84.493	13.151	14.425
4056	169	25	23	C	S	109.107	0.418	1.657	5.220	-0.415	-1.500	-4.727	84.604	14.426	13.149
4057	169	25	23	C	S	110.817	3.582	4.655	1.960	-3.342	-4.479	-1.886	84.636	14.436	13.133
4058	169	25	23	C	W	9.183	3.582	-4.655	1.960	-3.342	4.479	-1.886	84.636	13.133	14.436
4059	169	25	23	C	W	10.893	0.418	-1.656	5.220	-0.415	1.500	-4.727	84.604	14.426	13.149
4060	169	25	23	C	W	13.174	5.011	2.425	0.610	-4.554	-2.396	-0.603	84.493	13.151	14.425
4061	169	25	23	C	W	23.691	2.390	-4.911	3.142	-2.281	4.621	-2.956	81.894	10.867	17.545
4062	169	25	23	C	W	25.767	0.353	-1.196	5.291	-0.351	1.082	-4.785	81.878	17.539	10.877
4063	169	25	23	C	W	27.796	5.011	2.425	0.610	-4.554	-2.396	-0.603	81.771	10.878	17.539
4064	169	25	23	C	W	32.204	5.011	-2.425	0.610	-4.554	2.396	-0.603	81.771	10.878	17.539
4065	169	25	23	C	W	34.233	0.353	1.196	5.291	-0.351	-1.082	-4.785	81.878	17.539	10.877
4066	169	25	23	C	W	36.309	2.390	4.911	3.142	-2.281	-4.621	-2.956	81.894	17.545	10.867
4067	169	25	23	C	W	46.827	5.011	-2.425	0.610	-4.554	2.396	-0.603	84.493	13.151	14.425
4068	169	25	23	C	W	49.107	0.418	1.657	5.220	-0.415	-1.500	-4.727	84.604	14.426	13.149
4069	169	25	23	C	W	50.817	3.582	4.655	1.960	-3.342	-4.479	-1.886	84.636	14.436	13.133
4070	169	25	23	C	W	69.183	3.582	-4.655	1.960	-3.342	4.479	-1.886	84.636	13.133	14.436
4071	169	25	23	C	W	70.893	0.418	-1.656	5.220	-0.415	1.500	-4.727	84.604	14.426	13.149
4072	169	25	23	C	W	73.174	5.011	2.425	0.610	-4.554	-2.396	-0.603	84.493	13.151	14.425
4073	169	25	23	C	W	83.691	2.390	-4.911	3.142	-2.281	4.621	-2.956	81.894	10.867	17.545
4074	169	25	23	C	W	85.767	0.353	-1.196	5.291	-0.351	1.082	-4.785	81.878	17.539	10.877
4075	169	25	23	C	W	87.796	5.011	2.425	0.610	-4.554	-2.396	-0.603	81.771	10.878	17.539
4076	169	25	23	C	W	92.204	5.011	-2.425	0.610	-4.554	2.396	-0.603	81.771	10.878	17.539
4077	169	25	23	C	W	94.233	0.353	1.196	5.291	-0.351	-1.082	-4.785	81.878	17.539	10.877
4078	169	25	23	C	W	96.309	2.390	4.911	3.142	-2.281	-4.621	-2.956	81.894	17.545	10.867
4079	169	25	23	C	W	106.827	5.011	-2.425	0.610	-4.554	2.396	-0.603	84.493	13.151	14.425
4080	169	25	23	C	W	109.107	0.418	1.657	5.220	-0.415	-1.500	-4.727	84.604	14.426	13.149

BL number	Atoms	γ-PC unit cells	WS_2 unit cells	γ-PC origin atom	WS_2 origin atom	Twist-angle (°)	γ-PC strain 1 (%)	γ-PC strain 2 (%)	γ-PC strain 3 (%)	WS_2 strain 1 (%)	WS_2 strain 2 (%)	WS_2 strain 3 (%)	γ (°)	|**a**|(Å)	|**b**|(Å)
4081	169	25	23	C	W	110.817	3.582	4.655	1.960	-3.342	-4.479	-1.886	84.636	14.436	13.133
4082	169	25	23	P	S	9.183	3.582	-4.655	1.960	-3.342	4.479	-1.886	84.636	13.133	14.436
4083	169	25	23	P	S	10.893	0.418	-1.656	5.220	-0.415	1.500	-4.727	84.604	14.426	13.149
4084	169	25	23	P	S	13.174	5.011	2.425	0.610	-4.554	-2.396	-0.603	84.493	13.151	14.425
4085	169	25	23	P	S	23.691	2.390	-4.911	3.142	-2.281	4.621	-2.956	81.894	10.867	17.545
4086	169	25	23	P	S	25.767	0.353	-1.196	5.291	-0.351	1.082	-4.785	81.878	17.539	10.877
4087	169	25	23	P	S	27.796	5.011	2.425	0.610	-4.554	-2.396	-0.603	81.771	10.878	17.539
4088	169	25	23	P	S	32.204	5.011	-2.425	0.610	-4.554	2.396	-0.603	81.771	10.878	17.539
4089	169	25	23	P	S	34.233	0.353	1.196	5.291	-0.351	-1.082	-4.785	81.878	17.539	10.877
4090	169	25	23	P	S	36.309	2.390	4.911	3.142	-2.281	-4.621	-2.956	81.894	17.545	10.867
4091	169	25	23	P	S	46.827	5.011	-2.425	0.610	-4.554	2.396	-0.603	84.493	13.151	14.425
4092	169	25	23	P	S	49.107	0.418	1.657	5.220	-0.415	-1.500	-4.727	84.604	14.426	13.149
4093	169	25	23	P	S	50.817	3.582	4.655	1.960	-3.342	-4.479	-1.886	84.636	14.436	13.133
4094	169	25	23	P	S	69.183	3.582	-4.655	1.960	-3.342	4.479	-1.886	84.636	13.133	14.436
4095	169	25	23	P	S	70.893	0.418	-1.656	5.220	-0.415	1.500	-4.727	84.604	14.426	13.149
4096	169	25	23	P	S	73.174	5.011	2.425	0.610	-4.554	-2.396	-0.603	84.493	13.151	14.425
4097	169	25	23	P	S	83.691	2.390	-4.911	3.142	-2.281	4.621	-2.956	81.894	10.867	17.545
4098	169	25	23	P	S	85.767	0.353	-1.196	5.291	-0.351	1.082	-4.785	81.878	17.539	10.877
4099	169	25	23	P	S	87.796	5.011	2.425	0.610	-4.554	-2.396	-0.603	81.771	10.878	17.539
4100	169	25	23	P	S	92.204	5.011	-2.425	0.610	-4.554	2.396	-0.603	81.771	10.878	17.539
4101	169	25	23	P	S	94.233	0.353	1.196	5.291	-0.351	-1.082	-4.785	81.878	17.539	10.877
4102	169	25	23	P	S	96.309	2.390	4.911	3.142	-2.281	-4.621	-2.956	81.894	17.545	10.867
4103	169	25	23	P	S	106.827	5.011	-2.425	0.610	-4.554	2.396	-0.603	84.493	13.151	14.425
4104	169	25	23	P	S	109.107	0.418	1.657	5.220	-0.415	-1.500	-4.727	84.604	14.426	13.149
4105	169	25	23	P	S	110.817	3.582	4.655	1.960	-3.342	-4.479	-1.886	84.636	14.436	13.133
4106	169	25	23	P	W	9.183	3.582	-4.655	1.960	-3.342	4.479	-1.886	84.636	13.133	14.436
4107	169	25	23	P	W	10.893	0.418	-1.656	5.220	-0.415	1.500	-4.727	84.604	14.426	13.149
4108	169	25	23	P	W	13.174	5.011	2.425	0.610	-4.554	-2.396	-0.603	84.493	13.151	14.425
4109	169	25	23	P	W	23.691	2.390	-4.911	3.142	-2.281	4.621	-2.956	81.894	10.867	17.545
4110	169	25	23	P	W	25.767	0.353	-1.196	5.291	-0.351	1.082	-4.785	81.878	17.539	10.877
4111	169	25	23	P	W	27.796	5.011	2.425	0.610	-4.554	-2.396	-0.603	81.771	10.878	17.539
4112	169	25	23	P	W	32.204	5.011	-2.425	0.610	-4.554	2.396	-0.603	81.771	10.878	17.539
4113	169	25	23	P	W	34.233	0.353	1.196	5.291	-0.351	-1.082	-4.785	81.878	17.539	10.877
4114	169	25	23	P	W	36.309	2.390	4.911	3.142	-2.281	-4.621	-2.956	81.894	17.545	10.867
4115	169	25	23	P	W	46.827	5.011	-2.425	0.610	-4.554	2.396	-0.603	84.493	13.151	14.425
4116	169	25	23	P	W	49.107	0.418	1.657	5.220	-0.415	-1.500	-4.727	84.604	14.426	13.149
4117	169	25	23	P	W	50.817	3.582	4.655	1.960	-3.342	-4.479	-1.886	84.636	14.436	13.133
4118	169	25	23	P	W	69.183	3.582	-4.655	1.960	-3.342	4.479	-1.886	84.636	13.133	14.436
4119	169	25	23	P	W	70.893	0.418	-1.656	5.220	-0.415	1.500	-4.727	84.604	14.426	13.149
4120	169	25	23	P	W	73.174	5.011	2.425	0.610	-4.554	-2.396	-0.603	84.493	13.151	14.425
4121	169	25	23	P	W	83.691	2.390	-4.911	3.142	-2.281	4.621	-2.956	81.894	10.867	17.545
4122	169	25	23	P	W	85.767	0.353	-1.196	5.291	-0.351	1.082	-4.785	81.878	17.539	10.877
4123	169	25	23	P	W	87.796	5.011	2.425	0.610	-4.554	-2.396	-0.603	81.771	10.878	17.539
4124	169	25	23	P	W	92.204	5.011	-2.425	0.610	-4.554	2.396	-0.603	81.771	10.878	17.539
4125	169	25	23	P	W	94.233	0.353	1.196	5.291	-0.351	-1.082	-4.785	81.878	17.539	10.877
4126	169	25	23	P	W	96.309	2.390	4.911	3.142	-2.281	-4.621	-2.956	81.894	17.545	10.867
4127	169	25	23	P	W	106.827	5.011	-2.425	0.610	-4.554	2.396	-0.603	84.493	13.151	14.425
4128	169	25	23	P	W	109.107	0.418	1.657	5.220	-0.415	-1.500	-4.727	84.604	14.426	13.149
4129	169	25	23	P	W	110.817	3.582	4.655	1.960	-3.342	-4.479	-1.886	84.636	14.436	13.133
4130	169	28	19	C	S	4.307	-4.685	0.000	-4.684	5.169	0.000	5.169	60.000	14.491	14.491
4131	169	28	19	C	S	17.480	-4.685	0.000	-4.684	5.169	0.000	5.169	60.000	14.491	14.491

BL number	Atoms	γ-PC unit cells	WS_2 unit cells	γ-PC origin atom	WS_2 origin atom	Twist-angle (°)	γ-PC strain 1 (%)	γ-PC strain 2 (%)	γ-PC strain 3 (%)	WS_2 strain 1 (%)	WS_2 strain 2 (%)	WS_2 strain 3 (%)	γ (°)	|**a**|(Å)	|**b**|(Å)
4132	169	28	19	C	S	42.520	-4.685	0.000	-4.684	5.169	0.000	5.169	60.000	14.491	14.491
4133	169	28	19	C	S	55.693	-4.685	0.000	-4.684	5.169	0.000	5.169	60.000	14.491	14.491
4134	169	28	19	C	S	64.307	-4.685	0.000	-4.684	5.169	0.000	5.169	60.000	14.491	14.491
4135	169	28	19	C	S	77.480	-4.685	0.000	-4.684	5.169	0.000	5.169	60.000	14.491	14.491
4136	169	28	19	C	S	102.520	-4.685	0.000	-4.684	5.169	0.000	5.169	60.000	14.491	14.491
4137	169	28	19	C	S	115.693	-4.685	0.000	-4.684	5.169	0.000	5.169	60.000	14.491	14.491
4138	169	28	19	C	W	4.307	-4.685	0.000	-4.684	5.169	0.000	5.169	60.000	14.491	14.491
4139	169	28	19	C	W	17.480	-4.685	0.000	-4.684	5.169	0.000	5.169	60.000	14.491	14.491
4140	169	28	19	C	W	42.520	-4.685	0.000	-4.684	5.169	0.000	5.169	60.000	14.491	14.491
4141	169	28	19	C	W	55.693	-4.685	0.000	-4.684	5.169	0.000	5.169	60.000	14.491	14.491
4142	169	28	19	C	W	64.307	-4.685	0.000	-4.684	5.169	0.000	5.169	60.000	14.491	14.491
4143	169	28	19	C	W	77.480	-4.685	0.000	-4.684	5.169	0.000	5.169	60.000	14.491	14.491
4144	169	28	19	C	W	102.520	-4.685	0.000	-4.684	5.169	0.000	5.169	60.000	14.491	14.491
4145	169	28	19	C	W	115.693	-4.685	0.000	-4.684	5.169	0.000	5.169	60.000	14.491	14.491
4146	169	28	19	P	S	17.480	-4.685	0.000	-4.684	5.169	0.000	5.169	60.000	14.491	14.491
4147	169	28	19	P	S	55.693	-4.685	0.000	-4.684	5.169	0.000	5.169	60.000	14.491	14.491
4148	169	28	19	P	S	64.307	-4.685	0.000	-4.684	5.169	0.000	5.169	60.000	14.491	14.491
4149	169	28	19	P	S	102.520	-4.685	0.000	-4.684	5.169	0.000	5.169	60.000	14.491	14.491
4150	169	28	19	P	W	4.307	-4.685	0.000	-4.684	5.169	0.000	5.169	60.000	14.491	14.491
4151	169	28	19	P	W	42.520	-4.685	0.000	-4.684	5.169	0.000	5.169	60.000	14.491	14.491
4152	169	28	19	P	W	77.480	-4.685	0.000	-4.684	5.169	0.000	5.169	60.000	14.491	14.491
4153	169	28	19	P	W	115.693	-4.685	0.000	-4.684	5.169	0.000	5.169	60.000	14.491	14.491
4154	169	28	19	C	S	19.842	-4.723	-2.386	-4.646	5.216	2.630	5.122	86.211	15.242	11.958
4155	169	28	19	C	S	22.689	-4.497	2.348	-4.872	4.941	-2.601	5.398	86.219	11.961	15.238
4156	169	28	19	C	S	40.158	-4.723	2.386	-4.646	5.216	-2.630	5.122	86.211	15.242	11.958
4157	169	28	19	C	S	82.689	-4.497	2.348	-4.872	4.941	-2.601	5.398	86.219	11.961	15.238
4158	169	28	19	C	S	97.311	-4.497	-2.348	-4.872	4.941	2.601	5.398	86.219	11.961	15.238
4159	169	28	19	C	S	100.158	-4.723	2.386	-4.646	5.216	-2.630	5.122	86.211	15.242	11.958
4160	169	28	19	C	W	22.689	-4.497	2.348	-4.872	4.941	-2.601	5.398	86.219	11.961	15.238
4161	169	28	19	C	W	37.311	-4.497	-2.348	-4.872	4.941	2.601	5.398	86.219	11.961	15.238
4162	169	28	19	C	W	40.158	-4.723	2.386	-4.646	5.216	-2.630	5.122	86.211	15.242	11.958
4163	169	28	19	C	W	79.842	-4.723	-2.386	-4.646	5.216	2.630	5.122	86.211	15.242	11.958
4164	169	28	19	C	W	82.689	-4.497	2.348	-4.872	4.941	-2.601	5.398	86.219	11.961	15.238
4165	169	28	19	C	W	100.158	-4.723	2.386	-4.646	5.216	-2.630	5.122	86.211	15.242	11.958
4166	169	28	19	P	S	22.689	-4.497	2.348	-4.872	4.941	-2.601	5.398	86.219	11.961	15.238
4167	169	28	19	P	S	37.311	-4.497	-2.348	-4.872	4.941	2.601	5.398	86.219	11.961	15.238
4168	169	28	19	P	S	40.158	-4.723	2.386	-4.646	5.216	-2.630	5.122	86.211	15.242	11.958
4169	169	28	19	P	S	79.842	-4.723	-2.386	-4.646	5.216	2.630	5.122	86.211	15.242	11.958
4170	169	28	19	P	S	82.689	-4.497	2.348	-4.872	4.941	-2.601	5.398	86.219	11.961	15.238
4171	169	28	19	P	S	100.158	-4.723	2.386	-4.646	5.216	-2.630	5.122	86.211	15.242	11.958
4172	169	28	19	P	W	19.842	-4.723	-2.386	-4.646	5.216	2.630	5.122	86.211	15.242	11.958
4173	169	28	19	P	W	22.689	-4.497	2.348	-4.872	4.941	-2.601	5.398	86.219	11.961	15.238
4174	169	28	19	P	W	40.158	-4.723	2.386	-4.646	5.216	-2.630	5.122	86.211	15.242	11.958
4175	169	28	19	P	W	82.689	-4.497	2.348	-4.872	4.941	-2.601	5.398	86.219	11.961	15.238
4176	169	28	19	P	W	97.311	-4.497	-2.348	-4.872	4.941	2.601	5.398	86.219	11.961	15.238
4177	169	28	19	P	W	100.158	-4.723	2.386	-4.646	5.216	-2.630	5.122	86.211	15.242	11.958
4178	170	26	22	C	S	0.000	5.011	-2.332	-3.452	-4.554	2.505	3.708	88.616	10.878	17.329
4179	170	26	22	C	S	3.004	-3.388	2.988	4.935	3.635	-2.720	-4.492	88.369	17.335	10.875
4180	170	26	22	C	S	13.174	5.011	2.332	-3.452	-4.554	-2.505	3.708	75.122	13.151	14.827
4181	170	26	22	C	S	24.791	-3.388	-2.988	4.935	3.635	2.720	-4.492	88.369	17.335	10.875
4182	170	26	22	C	S	25.693	2.326	-5.382	-1.064	-2.223	5.499	1.087	70.252	13.473	14.840

BL number	Atoms	γ-PC unit cells	WS_2 unit cells	γ-PC origin atom	WS_2 origin atom	Twist-angle (°)	γ-PC strain 1 (%)	γ-PC strain 2 (%)	γ-PC strain 3 (%)	WS_2 strain 1 (%)	WS_2 strain 2 (%)	WS_2 strain 3 (%)	γ (°)	|**a**|(Å)	|**b**|(Å)
4183	170	26	22	C	S	27.796	5.011	2.332	-3.452	-4.554	-2.505	3.708	88.616	10.878	17.329
4184	170	26	22	C	S	30.000	-2.359	2.168	3.749	2.476	-2.017	-3.487	70.441	14.845	13.461
4185	170	26	22	C	S	32.204	5.011	-2.332	-3.452	-4.554	2.505	3.708	88.616	10.878	17.329
4186	170	26	22	C	S	34.307	2.326	5.382	-1.064	-2.223	-5.499	1.087	70.252	13.473	14.840
4187	170	26	22	C	S	35.209	-3.388	2.988	4.935	3.635	-2.720	-4.492	88.369	17.335	10.875
4188	170	26	22	C	S	46.827	5.011	-2.332	-3.452	-4.554	2.505	3.708	75.122	13.151	14.827
4189	170	26	22	C	S	56.996	-3.388	-2.988	4.935	3.635	2.720	-4.492	88.369	17.335	10.875
4190	170	26	22	C	S	60.000	5.011	2.332	-3.452	-4.554	-2.505	3.708	88.616	10.878	17.329
4191	170	26	22	C	S	63.005	-3.388	2.988	4.935	3.635	-2.720	-4.492	88.369	17.335	10.875
4192	170	26	22	C	S	73.174	5.011	2.332	-3.452	-4.554	-2.505	3.708	75.122	13.151	14.827
4193	170	26	22	C	S	84.791	-3.388	-2.988	4.935	3.635	2.720	-4.492	88.369	17.335	10.875
4194	170	26	22	C	S	85.694	2.326	-5.382	-1.064	-2.223	5.499	1.087	70.252	13.473	14.840
4195	170	26	22	C	S	87.796	5.011	2.332	-3.452	-4.554	-2.505	3.708	88.616	10.878	17.329
4196	170	26	22	C	S	90.000	-2.359	-2.168	3.749	2.476	2.017	-3.487	70.441	14.845	13.461
4197	170	26	22	C	S	92.204	5.011	-2.332	-3.452	-4.554	2.505	3.708	88.617	10.878	17.329
4198	170	26	22	C	S	94.307	2.326	5.382	-1.064	-2.223	-5.499	1.087	70.252	13.473	14.840
4199	170	26	22	C	S	95.209	-3.388	2.988	4.935	3.635	-2.720	-4.492	88.369	17.335	10.875
4200	170	26	22	C	S	106.827	5.011	-2.332	-3.452	-4.554	2.505	3.708	75.122	13.151	14.827
4201	170	26	22	C	S	116.996	-3.388	-2.988	4.935	3.635	2.720	-4.492	88.369	17.335	10.875
4202	170	26	22	C	W	0.000	5.011	-2.332	-3.452	-4.554	2.505	3.708	88.616	10.878	17.329
4203	170	26	22	C	W	3.004	-3.388	2.988	4.935	3.635	-2.720	-4.492	88.369	17.335	10.875
4204	170	26	22	C	W	13.174	5.011	2.332	-3.452	-4.554	-2.505	3.708	75.122	13.151	14.827
4205	170	26	22	C	W	24.791	-3.388	-2.988	4.935	3.635	2.720	-4.492	88.369	17.335	10.875
4206	170	26	22	C	W	25.693	2.326	-5.382	-1.064	-2.223	5.499	1.087	70.252	13.473	14.840
4207	170	26	22	C	W	27.796	5.011	2.332	-3.452	-4.554	-2.505	3.708	88.616	10.878	17.329
4208	170	26	22	C	W	30.000	-2.359	2.168	3.749	2.476	-2.017	-3.487	70.441	14.845	13.461
4209	170	26	22	C	W	32.204	5.011	-2.332	-3.452	-4.554	2.505	3.708	88.616	10.878	17.329
4210	170	26	22	C	W	34.307	2.326	5.382	-1.064	-2.223	-5.499	1.087	70.252	13.473	14.840
4211	170	26	22	C	W	35.209	-3.388	2.988	4.935	3.635	-2.720	-4.492	88.369	17.335	10.875
4212	170	26	22	C	W	46.827	5.011	-2.332	-3.452	-4.554	2.505	3.708	75.122	13.151	14.827
4213	170	26	22	C	W	56.996	-3.388	-2.988	4.935	3.635	2.720	-4.492	88.369	17.335	10.875
4214	170	26	22	C	W	60.000	5.011	2.332	-3.452	-4.554	-2.505	3.708	88.616	10.878	17.329
4215	170	26	22	C	W	63.005	-3.388	2.988	4.935	3.635	-2.720	-4.492	88.369	17.335	10.875
4216	170	26	22	C	W	73.174	5.011	2.332	-3.452	-4.554	-2.505	3.708	75.122	13.151	14.827
4217	170	26	22	C	W	84.791	-3.388	-2.988	4.935	3.635	2.720	-4.492	88.369	17.335	10.875
4218	170	26	22	C	W	85.694	2.326	-5.382	-1.064	-2.223	5.499	1.087	70.252	13.473	14.840
4219	170	26	22	C	W	87.796	5.011	2.332	-3.452	-4.554	-2.505	3.708	88.616	10.878	17.329
4220	170	26	22	C	W	90.000	-2.359	-2.168	3.749	2.476	2.017	-3.487	70.441	14.845	13.461
4221	170	26	22	C	W	92.204	5.011	-2.332	-3.452	-4.554	2.505	3.708	88.617	10.878	17.329
4222	170	26	22	C	W	94.307	2.326	5.382	-1.064	-2.223	-5.499	1.087	70.252	13.473	14.840
4223	170	26	22	C	W	95.209	-3.388	2.988	4.935	3.635	-2.720	-4.492	88.369	17.335	10.875
4224	170	26	22	C	W	106.827	5.011	-2.332	-3.452	-4.554	2.505	3.708	75.122	13.151	14.827
4225	170	26	22	C	W	116.996	-3.388	-2.988	4.935	3.635	2.720	-4.492	88.369	17.335	10.875
4226	170	26	22	P	S	0.000	5.011	-2.332	-3.452	-4.554	2.505	3.708	88.616	10.878	17.329
4227	170	26	22	P	S	3.004	-3.388	2.988	4.935	3.635	-2.720	-4.492	88.369	17.335	10.875
4228	170	26	22	P	S	13.174	5.011	2.332	-3.452	-4.554	-2.505	3.708	75.122	13.151	14.827
4229	170	26	22	P	S	24.791	-3.388	-2.988	4.935	3.635	2.720	-4.492	88.369	17.335	10.875
4230	170	26	22	P	S	25.693	2.326	-5.382	-1.064	-2.223	5.499	1.087	70.252	13.473	14.840
4231	170	26	22	P	S	27.796	5.011	2.332	-3.452	-4.554	-2.505	3.708	88.616	10.878	17.329
4232	170	26	22	P	S	30.000	-2.359	2.168	3.749	2.476	-2.017	-3.487	70.441	14.845	13.461
4233	170	26	22	P	S	32.204	5.011	-2.332	-3.452	-4.554	2.505	3.708	88.616	10.878	17.329

BL number	Atoms	γ-PC unit cells	WS_2 unit cells	γ-PC origin atom	WS_2 origin atom	Twist-angle (°)	γ-PC strain 1 (%)	γ-PC strain 2 (%)	γ-PC strain 3 (%)	WS_2 strain 1 (%)	WS_2 strain 2 (%)	WS_2 strain 3 (%)	γ (°)	|**a**|(Å)	|**b**|(Å)
4234	170	26	22	P	S	34.307	2.326	5.382	-1.064	-2.223	-5.499	1.087	70.252	13.473	14.840
4235	170	26	22	P	S	35.209	-3.388	2.988	4.935	3.635	-2.720	-4.492	88.369	17.335	10.875
4236	170	26	22	P	S	46.827	5.011	-2.332	-3.452	-4.554	2.505	3.708	75.122	13.151	14.827
4237	170	26	22	P	S	56.996	-3.388	-2.988	4.935	3.635	2.720	-4.492	88.369	17.335	10.875
4238	170	26	22	P	S	60.000	5.011	2.332	-3.452	-4.554	-2.505	3.708	88.616	10.878	17.329
4239	170	26	22	P	S	63.005	-3.388	2.988	4.935	3.635	-2.720	-4.492	88.369	17.335	10.875
4240	170	26	22	P	S	73.174	5.011	2.332	-3.452	-4.554	-2.505	3.708	75.122	13.151	14.827
4241	170	26	22	P	S	84.791	-3.388	-2.988	4.935	3.635	2.720	-4.492	88.369	17.335	10.875
4242	170	26	22	P	S	85.694	2.326	-5.382	-1.064	-2.223	5.499	1.087	70.252	13.473	14.840
4243	170	26	22	P	S	87.796	5.011	2.332	-3.452	-4.554	-2.505	3.708	88.616	10.878	17.329
4244	170	26	22	P	S	90.000	-2.359	-2.168	3.749	2.476	2.017	-3.487	70.441	14.845	13.461
4245	170	26	22	P	S	92.204	5.011	-2.332	-3.452	-4.554	2.505	3.708	88.617	10.878	17.329
4246	170	26	22	P	S	94.307	2.326	5.382	-1.064	-2.223	-5.499	1.087	70.252	13.473	14.840
4247	170	26	22	P	S	95.209	-3.388	2.988	4.935	3.635	-2.720	-4.492	88.369	17.335	10.875
4248	170	26	22	P	S	106.827	5.011	-2.332	-3.452	-4.554	2.505	3.708	75.122	13.151	14.827
4249	170	26	22	P	S	116.996	-3.388	-2.988	4.935	3.635	2.720	-4.492	88.369	17.335	10.875
4250	170	26	22	P	W	0.000	5.011	-2.332	-3.452	-4.554	2.505	3.708	88.616	10.878	17.329
4251	170	26	22	P	W	3.004	-3.388	2.988	4.935	3.635	-2.720	-4.492	88.369	17.335	10.875
4252	170	26	22	P	W	13.174	5.011	2.332	-3.452	-4.554	-2.505	3.708	75.122	13.151	14.827
4253	170	26	22	P	W	24.791	-3.388	-2.988	4.935	3.635	2.720	-4.492	88.369	17.335	10.875
4254	170	26	22	P	W	25.693	2.326	-5.382	-1.064	-2.223	5.499	1.087	70.252	13.473	14.840
4255	170	26	22	P	W	27.796	5.011	2.332	-3.452	-4.554	-2.505	3.708	88.616	10.878	17.329
4256	170	26	22	P	W	30.000	-2.359	2.168	3.749	2.476	-2.017	-3.487	70.441	14.845	13.461
4257	170	26	22	P	W	32.204	5.011	-2.332	-3.452	-4.554	2.505	3.708	88.616	10.878	17.329
4258	170	26	22	P	W	34.307	2.326	5.382	-1.064	-2.223	-5.499	1.087	70.252	13.473	14.840
4259	170	26	22	P	W	35.209	-3.388	2.988	4.935	3.635	-2.720	-4.492	88.369	17.335	10.875
4260	170	26	22	P	W	46.827	5.011	-2.332	-3.452	-4.554	2.505	3.708	75.122	13.151	14.827
4261	170	26	22	P	W	56.996	-3.388	-2.988	4.935	3.635	2.720	-4.492	88.369	17.335	10.875
4262	170	26	22	P	W	60.000	5.011	2.332	-3.452	-4.554	-2.505	3.708	88.616	10.878	17.329
4263	170	26	22	P	W	63.005	-3.388	2.988	4.935	3.635	-2.720	-4.492	88.369	17.335	10.875
4264	170	26	22	P	W	73.174	5.011	2.332	-3.452	-4.554	-2.505	3.708	75.122	13.151	14.827
4265	170	26	22	P	W	84.791	-3.388	-2.988	4.935	3.635	2.720	-4.492	88.369	17.335	10.875
4266	170	26	22	P	W	85.694	2.326	-5.382	-1.064	-2.223	5.499	1.087	70.252	13.473	14.840
4267	170	26	22	P	W	87.796	5.011	2.332	-3.452	-4.554	-2.505	3.708	88.616	10.878	17.329
4268	170	26	22	P	W	90.000	-2.359	-2.168	3.749	2.476	2.017	-3.487	70.441	14.845	13.461
4269	170	26	22	P	W	92.204	5.011	-2.332	-3.452	-4.554	2.505	3.708	88.617	10.878	17.329
4270	170	26	22	P	W	94.307	2.326	5.382	-1.064	-2.223	-5.499	1.087	70.252	13.473	14.840
4271	170	26	22	P	W	95.209	-3.388	2.988	4.935	3.635	-2.720	-4.492	88.369	17.335	10.875
4272	170	26	22	P	W	106.827	5.011	-2.332	-3.452	-4.554	2.505	3.708	75.122	13.151	14.827
4273	170	26	22	P	W	116.996	-3.388	-2.988	4.935	3.635	2.720	-4.492	88.369	17.335	10.875
4274	171	27	21	C	S	13.898	-4.228	-2.730	1.423	4.619	2.655	-1.383	83.473	9.921	19.022
4275	171	27	21	C	S	17.696	1.029	3.795	-3.875	-1.008	-4.114	4.200	83.279	19.034	9.916
4276	171	27	21	C	S	21.052	1.339	-2.889	-4.154	-1.304	3.151	4.530	75.224	16.211	11.961
4277	171	27	21	C	S	22.689	-4.497	-0.157	1.726	4.941	0.152	-1.668	75.290	11.961	16.210
4278	171	27	21	C	S	25.767	0.353	5.053	-3.256	-0.351	-5.405	3.483	75.391	11.957	16.196
4279	171	27	21	C	S	34.233	0.353	-5.053	-3.256	-0.351	5.405	3.483	75.391	16.196	11.957
4280	171	27	21	C	S	37.311	-4.497	0.157	1.726	4.941	-0.152	-1.668	75.290	11.961	16.210
4281	171	27	21	C	S	38.948	1.339	2.889	-4.154	-1.304	-3.151	4.530	75.224	16.211	11.961
4282	171	27	21	C	S	42.304	1.029	-3.795	-3.875	-1.008	4.114	4.200	83.279	19.034	9.916
4283	171	27	21	C	S	46.102	-4.228	2.731	1.423	4.619	-2.655	-1.383	83.473	9.921	19.022
4284	171	27	21	C	S	73.898	-4.228	-2.730	1.423	4.619	2.655	-1.383	83.472	9.921	19.021

BL number	Atoms	γ-PC unit cells	WS_2 unit cells	γ-PC origin atom	WS_2 origin atom	Twist-angle (°)	γ-PC strain 1 (%)	γ-PC strain 2 (%)	γ-PC strain 3 (%)	WS_2 strain 1 (%)	WS_2 strain 2 (%)	WS_2 strain 3 (%)	γ (°)	|**a**|(Å)	|**b**|(Å)
4285	171	27	21	C	S	77.696	1.029	3.795	-3.875	-1.008	-4.114	4.200	83.279	19.034	9.916
4286	171	27	21	C	S	81.052	1.339	-2.889	-4.154	-1.304	3.151	4.530	75.224	16.211	11.961
4287	171	27	21	C	S	82.689	-4.497	-0.157	1.726	4.941	0.152	-1.668	75.290	11.961	16.210
4288	171	27	21	C	S	85.767	0.353	5.053	-3.256	-0.351	-5.405	3.483	75.391	11.957	16.196
4289	171	27	21	C	S	94.233	0.353	-5.053	-3.256	-0.351	5.405	3.483	75.391	16.196	11.957
4290	171	27	21	C	S	97.311	-4.497	0.157	1.726	4.941	-0.152	-1.668	75.290	11.961	16.210
4291	171	27	21	C	S	98.948	1.339	2.889	-4.154	-1.304	-3.151	4.530	75.224	16.211	11.961
4292	171	27	21	C	S	102.304	1.029	-3.795	-3.875	-1.008	4.114	4.200	83.279	19.034	9.916
4293	171	27	21	C	S	106.102	-4.228	2.731	1.423	4.619	-2.655	-1.383	83.473	9.921	19.021
4294	171	27	21	C	W	13.898	-4.228	-2.730	1.423	4.619	2.655	-1.383	83.473	9.921	19.022
4295	171	27	21	C	W	17.696	1.029	3.795	-3.875	-1.008	-4.114	4.200	83.279	19.034	9.916
4296	171	27	21	C	W	21.052	1.339	-2.889	-4.154	-1.304	3.151	4.530	75.224	16.211	11.961
4297	171	27	21	C	W	22.689	-4.497	-0.157	1.726	4.941	0.152	-1.668	75.290	11.961	16.210
4298	171	27	21	C	W	25.767	0.353	5.053	-3.256	-0.351	-5.405	3.483	75.391	11.957	16.196
4299	171	27	21	C	W	34.233	0.353	-5.053	-3.256	-0.351	5.405	3.483	75.391	16.196	11.957
4300	171	27	21	C	W	37.311	-4.497	0.157	1.726	4.941	-0.152	-1.668	75.290	11.961	16.210
4301	171	27	21	C	W	38.948	1.339	2.889	-4.154	-1.304	-3.151	4.530	75.224	16.211	11.961
4302	171	27	21	C	W	42.304	1.029	-3.795	-3.875	-1.008	4.114	4.200	83.279	19.034	9.916
4303	171	27	21	C	W	46.102	-4.228	2.731	1.423	4.619	-2.655	-1.383	83.473	9.921	19.022
4304	171	27	21	C	W	73.898	-4.228	-2.730	1.423	4.619	2.655	-1.383	83.472	9.921	19.021
4305	171	27	21	C	W	77.696	1.029	3.795	-3.875	-1.008	-4.114	4.200	83.279	19.034	9.916
4306	171	27	21	C	W	81.052	1.339	-2.889	-4.154	-1.304	3.151	4.530	75.224	16.211	11.961
4307	171	27	21	C	W	82.689	-4.497	-0.157	1.726	4.941	0.152	-1.668	75.290	11.961	16.210
4308	171	27	21	C	W	85.767	0.353	5.053	-3.256	-0.351	-5.405	3.483	75.391	11.957	16.196
4309	171	27	21	C	W	94.233	0.353	-5.053	-3.256	-0.351	5.405	3.483	75.391	16.196	11.957
4310	171	27	21	C	W	97.311	-4.497	0.157	1.726	4.941	-0.152	-1.668	75.290	11.961	16.210
4311	171	27	21	C	W	98.948	1.339	2.889	-4.154	-1.304	-3.151	4.530	75.224	16.211	11.961
4312	171	27	21	C	W	102.304	1.029	-3.795	-3.875	-1.008	4.114	4.200	83.279	19.034	9.916
4313	171	27	21	C	W	106.102	-4.228	2.731	1.423	4.619	-2.655	-1.383	83.473	9.921	19.021
4314	171	27	21	P	S	13.898	-4.228	-2.730	1.423	4.619	2.655	-1.383	83.473	9.921	19.022
4315	171	27	21	P	S	17.696	1.029	3.795	-3.875	-1.008	-4.114	4.200	83.279	19.034	9.916
4316	171	27	21	P	S	21.052	1.339	-2.889	-4.154	-1.304	3.151	4.530	75.224	16.211	11.961
4317	171	27	21	P	S	22.689	-4.497	-0.157	1.726	4.941	0.152	-1.668	75.290	11.961	16.210
4318	171	27	21	P	S	25.767	0.353	5.053	-3.256	-0.351	-5.405	3.483	75.391	11.957	16.196
4319	171	27	21	P	S	34.233	0.353	-5.053	-3.256	-0.351	5.405	3.483	75.391	16.196	11.957
4320	171	27	21	P	S	37.311	-4.497	0.157	1.726	4.941	-0.152	-1.668	75.290	11.961	16.210
4321	171	27	21	P	S	38.948	1.339	2.889	-4.154	-1.304	-3.151	4.530	75.224	16.211	11.961
4322	171	27	21	P	S	42.304	1.029	-3.795	-3.875	-1.008	4.114	4.200	83.279	19.034	9.916
4323	171	27	21	P	S	46.102	-4.228	2.731	1.423	4.619	-2.655	-1.383	83.473	9.921	19.022
4324	171	27	21	P	S	73.898	-4.228	-2.730	1.423	4.619	2.655	-1.383	83.472	9.921	19.021
4325	171	27	21	P	S	77.696	1.029	3.795	-3.875	-1.008	-4.114	4.200	83.279	19.034	9.916
4326	171	27	21	P	S	81.052	1.339	-2.889	-4.154	-1.304	3.151	4.530	75.224	16.211	11.961
4327	171	27	21	P	S	82.689	-4.497	-0.157	1.726	4.941	0.152	-1.668	75.290	11.961	16.210
4328	171	27	21	P	S	85.767	0.353	5.053	-3.256	-0.351	-5.405	3.483	75.391	11.957	16.196
4329	171	27	21	P	S	94.233	0.353	-5.053	-3.256	-0.351	5.405	3.483	75.391	16.196	11.957
4330	171	27	21	P	S	97.311	-4.497	0.157	1.726	4.941	-0.152	-1.668	75.290	11.961	16.210
4331	171	27	21	P	S	98.948	1.339	2.889	-4.154	-1.304	-3.151	4.530	75.224	16.211	11.961
4332	171	27	21	P	S	102.304	1.029	-3.795	-3.875	-1.008	4.114	4.200	83.279	19.034	9.916
4333	171	27	21	P	S	106.102	-4.228	2.731	1.423	4.619	-2.655	-1.383	83.473	9.921	19.021
4334	171	27	21	P	W	13.898	-4.228	-2.730	1.423	4.619	2.655	-1.383	83.473	9.921	19.022
4335	171	27	21	P	W	17.696	1.029	3.795	-3.875	-1.008	-4.114	4.200	83.279	19.034	9.916

BL number	Atoms	γ-PC unit cells	WS_2 unit cells	γ-PC origin atom	WS_2 origin atom	Twist-angle (°)	γ-PC strain 1 (%)	γ-PC strain 2 (%)	γ-PC strain 3 (%)	WS_2 strain 1 (%)	WS_2 strain 2 (%)	WS_2 strain 3 (%)	γ (°)	|**a**|(Å)	|**b**|(Å)
4336	171	27	21	P	W	21.052	1.339	-2.889	-4.154	-1.304	3.151	4.530	75.224	16.211	11.961
4337	171	27	21	P	W	22.689	-4.497	-0.157	1.726	4.941	0.152	-1.668	75.290	11.961	16.210
4338	171	27	21	P	W	25.767	0.353	5.053	-3.256	-0.351	-5.405	3.483	75.391	11.957	16.196
4339	171	27	21	P	W	34.233	0.353	-5.053	-3.256	-0.351	5.405	3.483	75.391	16.196	11.957
4340	171	27	21	P	W	37.311	-4.497	0.157	1.726	4.941	-0.152	-1.668	75.290	11.961	16.210
4341	171	27	21	P	W	38.948	1.339	2.889	-4.154	-1.304	-3.151	4.530	75.224	16.211	11.961
4342	171	27	21	P	W	42.304	1.029	-3.795	-3.875	-1.008	4.114	4.200	83.279	19.034	9.916
4343	171	27	21	P	W	46.102	-4.228	2.731	1.423	4.619	-2.655	-1.383	83.473	9.921	19.022
4344	171	27	21	P	W	73.898	-4.228	-2.730	1.423	4.619	2.655	-1.383	83.472	9.921	19.021
4345	171	27	21	P	W	77.696	1.029	3.795	-3.875	-1.008	-4.114	4.200	83.279	19.034	9.916
4346	171	27	21	P	W	81.052	1.339	-2.889	-4.154	-1.304	3.151	4.530	75.224	16.211	11.961
4347	171	27	21	P	W	82.689	-4.497	-0.157	1.726	4.941	0.152	-1.668	75.290	11.961	16.210
4348	171	27	21	P	W	85.767	0.353	5.053	-3.256	-0.351	-5.405	3.483	75.391	11.957	16.196
4349	171	27	21	P	W	94.233	0.353	-5.053	-3.256	-0.351	5.405	3.483	75.391	16.196	11.957
4350	171	27	21	P	W	97.311	-4.497	0.157	1.726	4.941	-0.152	-1.668	75.290	11.961	16.210
4351	171	27	21	P	W	98.948	1.339	2.889	-4.154	-1.304	-3.151	4.530	75.224	16.211	11.961
4352	171	27	21	P	W	102.304	1.029	-3.795	-3.875	-1.008	4.114	4.200	83.279	19.034	9.916
4353	171	27	21	P	W	106.102	-4.228	2.731	1.423	4.619	-2.655	-1.383	83.473	9.921	19.021
4354	174	27	22	C	S	1.945	-4.723	4.802	4.460	5.216	-4.409	-4.095	73.326	15.242	13.158
4355	174	27	22	C	S	16.102	2.853	0.550	-3.346	-2.699	-0.589	3.586	81.268	10.655	18.221
4356	174	27	22	C	S	16.537	-3.292	1.368	2.791	3.523	-1.295	-2.644	81.238	18.221	10.656
4357	174	27	22	C	S	24.791	-3.388	3.320	2.901	3.635	-3.138	-2.742	75.853	14.681	13.479
4358	174	27	22	C	S	34.307	2.326	-4.664	-2.876	-2.223	4.949	3.052	75.922	13.473	14.680
4359	174	27	22	C	S	35.209	-3.388	-3.320	2.901	3.635	3.138	-2.742	75.853	13.480	14.681
4360	174	27	22	C	S	43.463	-3.292	-1.367	2.791	3.523	1.295	-2.644	81.238	18.221	10.656
4361	174	27	22	C	S	58.055	-4.723	-4.802	4.460	5.216	4.409	-4.095	73.326	15.242	13.158
4362	174	27	22	C	S	61.945	-4.723	4.802	4.460	5.216	-4.409	-4.095	73.326	15.242	13.158
4363	174	27	22	C	S	76.102	2.853	0.550	-3.346	-2.699	-0.589	3.586	81.268	10.655	18.221
4364	174	27	22	C	S	76.537	-3.292	1.368	2.791	3.523	-1.295	-2.644	81.238	18.221	10.656
4365	174	27	22	C	S	84.791	-3.388	3.320	2.901	3.635	-3.138	-2.742	75.853	14.681	13.480
4366	174	27	22	C	S	85.694	2.326	4.664	-2.876	-2.223	-4.949	3.052	75.922	13.473	14.680
4367	174	27	22	C	S	103.463	-3.292	-1.367	2.791	3.523	1.295	-2.644	81.238	18.221	10.656
4368	174	27	22	C	S	103.898	2.853	-0.550	-3.346	-2.699	0.589	3.586	81.268	10.655	18.221
4369	174	27	22	C	S	118.055	-4.723	-4.802	4.460	5.216	4.409	-4.095	73.326	15.242	13.158
4370	174	27	22	C	W	1.945	-4.723	4.802	4.460	5.216	-4.409	-4.095	73.326	15.242	13.158
4371	174	27	22	C	W	16.102	2.853	0.550	-3.346	-2.699	-0.589	3.586	81.268	10.655	18.221
4372	174	27	22	C	W	25.693	2.326	4.664	-2.876	-2.223	-4.949	3.052	75.922	13.473	14.680
4373	174	27	22	C	W	43.463	-3.292	-1.367	2.791	3.523	1.295	-2.644	81.238	18.221	10.656
4374	174	27	22	C	W	43.898	2.853	-0.550	-3.346	-2.699	0.589	3.586	81.268	10.655	18.221
4375	174	27	22	C	W	58.055	-4.723	-4.802	4.460	5.216	4.409	-4.095	73.326	15.242	13.158
4376	174	27	22	C	W	61.945	-4.723	4.802	4.460	5.216	-4.409	-4.095	73.326	15.242	13.158
4377	174	27	22	C	W	76.102	2.853	0.550	-3.346	-2.699	-0.589	3.586	81.268	10.655	18.221
4378	174	27	22	C	W	76.537	-3.292	1.368	2.791	3.523	-1.295	-2.644	81.238	18.221	10.656
4379	174	27	22	C	W	94.307	2.326	-4.664	-2.876	-2.223	4.949	3.052	75.922	13.473	14.680
4380	174	27	22	C	W	95.209	-3.388	-3.320	2.901	3.635	3.138	-2.742	75.853	13.479	14.681
4381	174	27	22	C	W	103.463	-3.292	-1.367	2.791	3.523	1.295	-2.644	81.238	18.221	10.656
4382	174	27	22	C	W	118.055	-4.723	-4.802	4.460	5.216	4.409	-4.095	73.326	15.242	13.158
4383	174	27	22	P	S	1.945	-4.723	4.802	4.460	5.216	-4.409	-4.095	73.326	15.242	13.158
4384	174	27	22	P	S	16.102	2.853	0.550	-3.346	-2.699	-0.589	3.586	81.268	10.655	18.221
4385	174	27	22	P	S	24.791	-3.388	3.320	2.901	3.635	-3.138	-2.742	75.853	14.681	13.479
4386	174	27	22	P	S	34.307	2.326	-4.664	-2.876	-2.223	4.949	3.052	75.922	13.473	14.680

BL number	Atoms	γ-PC unit cells	WS_2 unit cells	γ-PC origin atom	WS_2 origin atom	Twist-angle (°)	γ-PC strain 1 (%)	γ-PC strain 2 (%)	γ-PC strain 3 (%)	WS_2 strain 1 (%)	WS_2 strain 2 (%)	WS_2 strain 3 (%)	γ (°)	$\lvert\mathbf{a}\rvert$(Å)	$\lvert\mathbf{b}\rvert$(Å)
4387	174	27	22	P	S	35.209	-3.388	-3.320	2.901	3.635	3.138	-2.742	75.853	13.480	14.681
4388	174	27	22	P	S	43.463	-3.292	-1.367	2.791	3.523	1.295	-2.644	81.238	18.221	10.656
4389	174	27	22	P	S	43.898	2.853	-0.550	-3.346	-2.699	0.589	3.586	81.268	10.655	18.221
4390	174	27	22	P	S	58.055	-4.723	-4.802	4.460	5.216	4.409	-4.095	73.326	15.242	13.158
4391	174	27	22	P	S	61.945	-4.723	4.802	4.460	5.216	-4.409	-4.095	73.326	15.242	13.158
4392	174	27	22	P	S	76.102	2.853	0.550	-3.346	-2.699	-0.589	3.586	81.268	10.655	18.221
4393	174	27	22	P	S	76.537	-3.292	1.368	2.791	3.523	-1.295	-2.644	81.238	18.221	10.656
4394	174	27	22	P	S	84.791	-3.388	3.320	2.901	3.635	-3.138	-2.742	75.853	14.681	13.480
4395	174	27	22	P	S	85.694	2.326	4.664	-2.876	-2.223	-4.949	3.052	75.922	13.473	14.680
4396	174	27	22	P	S	103.463	-3.292	-1.367	2.791	3.523	1.295	-2.644	81.238	18.221	10.656
4397	174	27	22	P	S	118.055	-4.723	-4.802	4.460	5.216	4.409	-4.095	73.326	15.242	13.158
4398	174	27	22	P	W	1.945	-4.723	4.802	4.460	5.216	-4.409	-4.095	73.326	15.242	13.158
4399	174	27	22	P	W	16.102	2.853	0.550	-3.346	-2.699	-0.589	3.586	81.268	10.655	18.221
4400	174	27	22	P	W	16.537	-3.292	1.368	2.791	3.523	-1.295	-2.644	81.238	18.221	10.656
4401	174	27	22	P	W	25.693	2.326	4.664	-2.876	-2.223	-4.949	3.052	75.922	13.473	14.680
4402	174	27	22	P	W	43.463	-3.292	-1.367	2.791	3.523	1.295	-2.644	81.238	18.221	10.656
4403	174	27	22	P	W	58.055	-4.723	-4.802	4.460	5.216	4.409	-4.095	73.326	15.242	13.158
4404	174	27	22	P	W	61.945	-4.723	4.802	4.460	5.216	-4.409	-4.095	73.326	15.242	13.158
4405	174	27	22	P	W	76.102	2.853	0.550	-3.346	-2.699	-0.589	3.586	81.268	10.655	18.221
4406	174	27	22	P	W	76.537	-3.292	1.368	2.791	3.523	-1.295	-2.644	81.238	18.221	10.656
4407	174	27	22	P	W	94.307	2.326	-4.664	-2.876	-2.223	4.949	3.052	75.922	13.473	14.680
4408	174	27	22	P	W	95.209	-3.388	-3.320	2.901	3.635	3.138	-2.742	75.853	13.479	14.681
4409	174	27	22	P	W	103.463	-3.292	-1.367	2.791	3.523	1.295	-2.644	81.238	18.221	10.656
4410	174	27	22	P	W	103.898	2.853	-0.550	-3.346	-2.699	0.589	3.586	81.268	10.655	18.221
4411	174	27	22	P	W	118.055	-4.723	-4.802	4.460	5.216	4.409	-4.095	73.326	15.242	13.158
4412	175	28	21	C	S	8.213	-2.359	0.000	-2.359	2.476	0.000	2.476	60.000	14.845	14.845
4413	175	28	21	C	S	51.787	-2.359	0.000	-2.359	2.476	0.000	2.476	60.000	14.845	14.845
4414	175	28	21	C	S	68.213	-2.359	0.000	-2.359	2.476	0.000	2.476	60.000	14.845	14.845
4415	175	28	21	C	S	111.787	-2.359	0.000	-2.359	2.476	0.000	2.476	60.000	14.845	14.845
4416	175	28	21	C	W	8.213	-2.359	0.000	-2.359	2.476	0.000	2.476	60.000	14.845	14.845
4417	175	28	21	C	W	51.787	-2.359	0.000	-2.359	2.476	0.000	2.476	60.000	14.845	14.845
4418	175	28	21	C	W	68.213	-2.359	0.000	-2.359	2.476	0.000	2.476	60.000	14.845	14.845
4419	175	28	21	C	W	111.787	-2.359	0.000	-2.359	2.476	0.000	2.476	60.000	14.845	14.845
4420	175	28	21	C	S	13.898	-4.228	-0.988	-0.414	4.619	0.996	0.417	76.078	17.184	11.446
4421	175	28	21	C	S	16.537	-3.292	3.570	-1.408	3.523	-3.674	1.449	76.188	17.170	11.447
4422	175	28	21	C	S	43.463	-3.292	-3.570	-1.408	3.523	3.674	1.449	76.188	11.447	17.170
4423	175	28	21	C	S	46.102	-4.228	0.987	-0.414	4.619	-0.996	0.417	76.078	9.921	19.826
4424	175	28	21	C	S	73.898	-0.414	-0.948	-4.228	0.417	1.035	4.619	76.104	11.445	17.184
4425	175	28	21	C	S	76.537	-3.292	3.570	-1.408	3.523	-3.674	1.449	76.188	17.170	11.447
4426	175	28	21	C	S	103.463	-3.292	-3.570	-1.408	3.523	3.674	1.449	76.188	11.447	17.170
4427	175	28	21	C	S	106.102	-0.414	0.948	-4.228	0.417	-1.035	4.619	76.104	11.445	17.184
4428	175	28	21	C	W	13.898	-4.228	-0.988	-0.414	4.619	0.996	0.417	76.078	17.184	11.446
4429	175	28	21	C	W	16.537	-3.292	3.570	-1.408	3.523	-3.674	1.449	76.188	17.170	11.447
4430	175	28	21	C	W	43.463	-3.292	-3.570	-1.408	3.523	3.674	1.449	76.188	11.447	17.170
4431	175	28	21	C	W	46.102	-4.228	0.987	-0.414	4.619	-0.996	0.417	76.078	9.921	19.826
4432	175	28	21	C	W	73.898	-0.414	-0.948	-4.228	0.417	1.035	4.619	76.104	11.445	17.184
4433	175	28	21	C	W	76.537	-3.292	3.570	-1.408	3.523	-3.674	1.449	76.188	17.170	11.447
4434	175	28	21	C	W	103.463	-3.292	-3.570	-1.408	3.523	3.674	1.449	76.188	11.447	17.170
4435	175	28	21	C	W	106.102	-0.414	0.948	-4.228	0.417	-1.035	4.619	76.104	11.445	17.184
4436	175	28	21	P	S	13.898	-0.414	-0.948	-4.228	0.417	1.035	4.619	76.104	11.445	17.184
4437	175	28	21	P	S	46.102	-4.228	0.987	-0.414	4.619	-0.996	0.417	76.078	9.921	19.826

BL number	Atoms	γ-PC unit cells	WS_2 unit cells	γ-PC origin atom	WS_2 origin atom	Twist-angle (°)	γ-PC strain 1 (%)	γ-PC strain 2 (%)	γ-PC strain 3 (%)	WS_2 strain 1 (%)	WS_2 strain 2 (%)	WS_2 strain 3 (%)	γ (°)	**|a|**(Å)	**|b|**(Å)
4438	175	28	21	P	S	73.898	-4.228	-0.988	-0.414	4.619	0.996	0.417	76.079	9.921	19.826
4439	175	28	21	P	S	106.102	-0.414	0.948	-4.228	0.417	-1.035	4.619	76.104	19.824	9.921
4440	175	28	21	P	W	13.898	-0.414	-0.948	-4.228	0.417	1.035	4.619	76.104	19.824	9.921
4441	175	28	21	P	W	46.102	-0.414	0.948	-4.228	0.417	-1.035	4.619	76.104	19.824	9.921
4442	175	28	21	P	W	73.898	-0.414	-0.948	-4.228	0.417	1.035	4.619	76.104	19.824	9.921
4443	175	28	21	P	W	106.102	-0.414	0.948	-4.228	0.417	-1.035	4.619	76.104	19.824	9.921
4444	176	26	24	C	S	10.158	2.281	-2.109	3.430	-2.182	1.974	-3.210	67.393	15.811	13.472
4445	176	26	24	C	S	10.893	4.020	-0.770	1.711	-3.721	0.745	-1.655	67.396	15.815	13.470
4446	176	26	24	C	S	47.480	2.326	-2.152	3.385	-2.223	2.016	-3.170	67.392	13.473	15.810
4447	176	26	24	C	S	49.107	4.020	0.770	1.711	-3.721	-0.745	-1.655	67.396	15.815	13.470
4448	176	26	24	C	S	70.158	2.281	-2.109	3.430	-2.182	1.974	-3.210	67.393	15.811	13.472
4449	176	26	24	C	S	70.893	4.020	-0.770	1.711	-3.721	0.745	-1.655	67.396	15.815	13.470
4450	176	26	24	C	S	107.480	2.326	-2.152	3.385	-2.223	2.016	-3.170	67.392	13.473	15.810
4451	176	26	24	C	S	109.107	4.020	0.770	1.711	-3.721	-0.745	-1.655	67.396	15.815	13.470
4452	176	26	24	C	W	10.158	2.281	-2.109	3.430	-2.182	1.974	-3.210	67.393	15.811	13.472
4453	176	26	24	C	W	10.893	4.020	-0.770	1.711	-3.721	0.745	-1.655	67.396	15.815	13.470
4454	176	26	24	C	W	47.480	2.326	-2.152	3.385	-2.223	2.016	-3.170	67.392	13.473	15.810
4455	176	26	24	C	W	49.107	4.020	0.770	1.711	-3.721	-0.745	-1.655	67.396	15.815	13.470
4456	176	26	24	C	W	70.158	2.281	-2.109	3.430	-2.182	1.974	-3.210	67.393	15.811	13.472
4457	176	26	24	C	W	70.893	4.020	-0.770	1.711	-3.721	0.745	-1.655	67.396	15.815	13.470
4458	176	26	24	C	W	107.480	2.326	-2.152	3.385	-2.223	2.016	-3.170	67.392	13.473	15.810
4459	176	26	24	C	W	109.107	4.020	0.770	1.711	-3.721	-0.745	-1.655	67.396	15.815	13.470
4460	176	26	24	P	S	10.158	2.281	-2.109	3.430	-2.182	1.974	-3.210	67.393	15.811	13.472
4461	176	26	24	P	S	10.893	4.020	-0.770	1.711	-3.721	0.745	-1.655	67.396	15.815	13.470
4462	176	26	24	P	S	47.480	2.326	-2.152	3.385	-2.223	2.016	-3.170	67.392	13.473	15.810
4463	176	26	24	P	S	49.107	4.020	0.770	1.711	-3.721	-0.745	-1.655	67.396	15.815	13.470
4464	176	26	24	P	S	70.158	2.281	-2.109	3.430	-2.182	1.974	-3.210	67.393	15.811	13.472
4465	176	26	24	P	S	70.893	4.020	-0.770	1.711	-3.721	0.745	-1.655	67.396	15.815	13.470
4466	176	26	24	P	S	107.480	2.326	-2.152	3.385	-2.223	2.016	-3.170	67.392	13.473	15.810
4467	176	26	24	P	S	109.107	4.020	0.770	1.711	-3.721	-0.745	-1.655	67.396	15.815	13.470
4468	176	26	24	P	W	10.158	2.281	-2.109	3.430	-2.182	1.974	-3.210	67.393	15.811	13.472
4469	176	26	24	P	W	10.893	4.020	-0.770	1.711	-3.721	0.745	-1.655	67.396	15.815	13.470
4470	176	26	24	P	W	47.480	2.326	-2.152	3.385	-2.223	2.016	-3.170	67.392	13.473	15.810
4471	176	26	24	P	W	49.107	4.020	0.770	1.711	-3.721	-0.745	-1.655	67.396	15.815	13.470
4472	176	26	24	P	W	70.158	2.281	-2.109	3.430	-2.182	1.974	-3.210	67.393	15.811	13.472
4473	176	26	24	P	W	70.893	4.020	-0.770	1.711	-3.721	0.745	-1.655	67.396	15.815	13.470
4474	176	26	24	P	W	107.480	2.326	-2.152	3.385	-2.223	2.016	-3.170	67.392	13.473	15.810
4475	176	26	24	P	W	109.107	4.020	0.770	1.711	-3.721	-0.745	-1.655	67.396	15.815	13.470
4476	176	29	20	C	S	22.689	-4.497	-2.267	-4.134	4.941	2.471	4.507	72.593	11.961	16.632
4477	176	29	20	C	S	25.285	-4.781	2.085	-3.846	5.287	-2.259	4.166	72.596	16.641	11.954
4478	176	29	20	C	S	34.715	-4.781	-2.085	-3.846	5.287	2.259	4.166	72.596	16.641	11.954
4479	176	29	20	C	S	37.311	-4.497	2.267	-4.134	4.941	-2.471	4.507	72.593	11.961	16.632
4480	176	29	20	C	S	82.689	-4.497	-2.267	-4.134	4.941	2.471	4.507	72.593	11.961	16.632
4481	176	29	20	C	S	85.285	-4.781	2.085	-3.846	5.287	-2.259	4.166	72.596	16.641	11.954
4482	176	29	20	C	S	94.715	-4.781	-2.085	-3.846	5.287	2.259	4.166	72.596	16.641	11.954
4483	176	29	20	C	S	97.311	-4.497	2.267	-4.134	4.941	-2.471	4.507	72.593	11.961	16.632
4484	176	29	20	C	W	22.689	-4.497	-2.267	-4.134	4.941	2.471	4.507	72.593	11.961	16.632
4485	176	29	20	C	W	25.285	-4.781	2.085	-3.846	5.287	-2.259	4.166	72.596	16.641	11.954
4486	176	29	20	C	W	34.715	-4.781	-2.085	-3.846	5.287	2.259	4.166	72.596	16.641	11.954
4487	176	29	20	C	W	37.311	-4.497	2.267	-4.134	4.941	-2.471	4.507	72.593	11.961	16.632
4488	176	29	20	C	W	82.689	-4.497	-2.267	-4.134	4.941	2.471	4.507	72.593	11.961	16.632

BL number	Atoms	γ-PC unit cells	WS_2 unit cells	γ-PC origin atom	WS_2 origin atom	Twist-angle (°)	γ-PC strain 1 (%)	γ-PC strain 2 (%)	γ-PC strain 3 (%)	WS_2 strain 1 (%)	WS_2 strain 2 (%)	WS_2 strain 3 (%)	γ (°)	|**a**|(Å)	|**b**|(Å)
4489	176	29	20	C	W	85.285	-4.781	2.085	-3.846	5.287	-2.259	4.166	72.596	16.641	11.954
4490	176	29	20	C	W	94.715	-4.781	-2.085	-3.846	5.287	2.259	4.166	72.596	16.641	11.954
4491	176	29	20	C	W	97.311	-4.497	2.267	-4.134	4.941	-2.471	4.507	72.593	11.961	16.632
4492	176	29	20	P	S	22.689	-4.497	-2.267	-4.134	4.941	2.471	4.507	72.593	11.961	16.632
4493	176	29	20	P	S	25.285	-4.781	2.085	-3.846	5.287	-2.259	4.166	72.596	16.641	11.954
4494	176	29	20	P	S	34.715	-4.781	-2.085	-3.846	5.287	2.259	4.166	72.596	16.641	11.954
4495	176	29	20	P	S	37.311	-4.497	2.267	-4.134	4.941	-2.471	4.507	72.593	11.961	16.632
4496	176	29	20	P	S	82.689	-4.497	-2.267	-4.134	4.941	2.471	4.507	72.593	11.961	16.632
4497	176	29	20	P	S	85.285	-4.781	2.085	-3.846	5.287	-2.259	4.166	72.596	16.641	11.954
4498	176	29	20	P	S	94.715	-4.781	-2.085	-3.846	5.287	2.259	4.166	72.596	16.641	11.954
4499	176	29	20	P	S	97.311	-4.497	2.267	-4.134	4.941	-2.471	4.507	72.593	11.961	16.632
4500	176	29	20	P	W	22.689	-4.497	-2.267	-4.134	4.941	2.471	4.507	72.593	11.961	16.632
4501	176	29	20	P	W	25.285	-4.781	2.085	-3.846	5.287	-2.259	4.166	72.596	16.641	11.954
4502	176	29	20	P	W	34.715	-4.781	-2.085	-3.846	5.287	2.259	4.166	72.596	16.641	11.954
4503	176	29	20	P	W	37.311	-4.497	2.267	-4.134	4.941	-2.471	4.507	72.593	11.961	16.632
4504	176	29	20	P	W	82.689	-4.497	-2.267	-4.134	4.941	2.471	4.507	72.593	11.961	16.632
4505	176	29	20	P	W	85.285	-4.781	2.085	-3.846	5.287	-2.259	4.166	72.596	16.641	11.954
4506	176	29	20	P	W	94.715	-4.781	-2.085	-3.846	5.287	2.259	4.166	72.596	16.641	11.954
4507	176	29	20	P	W	97.311	-4.497	2.267	-4.134	4.941	-2.471	4.507	72.593	11.961	16.632
4508	177	27	23	C	S	0.000	5.011	-2.245	-3.139	-4.554	2.396	3.349	82.081	10.878	18.222
4509	177	27	23	C	S	1.359	-3.292	0.067	5.191	3.523	-0.060	-4.703	82.200	18.221	10.878
4510	177	27	23	C	S	19.107	-1.485	5.417	3.136	1.530	-5.097	-2.951	82.519	14.708	13.449
4511	177	27	23	C	S	40.893	-1.485	-5.417	3.136	1.530	5.097	-2.951	82.520	14.708	13.449
4512	177	27	23	C	S	41.387	3.582	-4.310	-1.889	-3.342	4.479	1.963	82.457	13.444	14.719
4513	177	27	23	C	S	58.641	-3.292	-0.067	5.191	3.523	0.060	-4.702	82.200	18.221	10.878
4514	177	27	23	C	S	60.000	5.011	2.246	-3.139	-4.554	-2.396	3.349	82.081	10.878	18.222
4515	177	27	23	C	S	61.359	-3.292	0.067	5.191	3.523	-0.060	-4.703	82.200	18.221	10.878
4516	177	27	23	C	S	78.613	3.582	4.310	-1.889	-3.342	-4.479	1.963	82.457	14.719	13.444
4517	177	27	23	C	S	79.107	-1.485	5.417	3.136	1.530	-5.097	-2.951	82.520	14.708	13.449
4518	177	27	23	C	S	100.893	-1.485	-5.417	3.136	1.530	5.097	-2.951	82.520	14.708	13.449
4519	177	27	23	C	S	118.641	-3.292	-0.067	5.191	3.523	0.060	-4.702	82.200	18.221	10.878
4520	177	27	23	C	W	0.000	5.011	-2.245	-3.139	-4.554	2.396	3.349	82.081	10.878	18.222
4521	177	27	23	C	W	1.359	-3.292	0.067	5.191	3.523	-0.060	-4.703	82.200	18.221	10.878
4522	177	27	23	C	W	18.613	3.582	4.310	-1.889	-3.342	-4.479	1.963	82.457	14.719	13.444
4523	177	27	23	C	W	58.641	-3.292	-0.067	5.191	3.523	0.060	-4.702	82.200	18.221	10.878
4524	177	27	23	C	W	60.000	5.011	2.246	-3.139	-4.554	-2.396	3.349	82.081	10.878	18.222
4525	177	27	23	C	W	61.359	-3.292	0.067	5.191	3.523	-0.060	-4.703	82.200	18.221	10.878
4526	177	27	23	C	W	101.387	3.582	-4.310	-1.889	-3.342	4.479	1.963	82.457	13.444	14.719
4527	177	27	23	C	W	118.641	-3.292	-0.067	5.191	3.523	0.060	-4.702	82.200	18.221	10.878
4528	177	27	23	P	S	0.000	5.011	-2.245	-3.139	-4.554	2.396	3.349	82.081	10.878	18.222
4529	177	27	23	P	S	1.359	-3.292	0.067	5.191	3.523	-0.060	-4.703	82.200	18.221	10.878
4530	177	27	23	P	S	19.107	-1.485	5.417	3.136	1.530	-5.097	-2.951	82.519	14.708	13.449
4531	177	27	23	P	S	40.893	-1.485	-5.417	3.136	1.530	5.097	-2.951	82.520	14.708	13.449
4532	177	27	23	P	S	41.387	3.582	-4.310	-1.889	-3.342	4.479	1.963	82.457	13.444	14.719
4533	177	27	23	P	S	58.641	-3.292	-0.067	5.191	3.523	0.060	-4.702	82.200	18.221	10.878
4534	177	27	23	P	S	60.000	5.011	2.246	-3.139	-4.554	-2.396	3.349	82.081	10.878	18.222
4535	177	27	23	P	S	61.359	-3.292	0.067	5.191	3.523	-0.060	-4.703	82.200	18.221	10.878
4536	177	27	23	P	S	78.613	3.582	4.310	-1.889	-3.342	-4.479	1.963	82.457	14.719	13.444
4537	177	27	23	P	S	79.107	-1.485	5.417	3.136	1.530	-5.097	-2.951	82.520	14.708	13.449
4538	177	27	23	P	S	100.893	-1.485	-5.417	3.136	1.530	5.097	-2.951	82.520	14.708	13.449
4539	177	27	23	P	S	118.641	-3.292	-0.067	5.191	3.523	0.060	-4.702	82.200	18.221	10.878

BL number	Atoms	γ-PC unit cells	WS_2 unit cells	γ-PC origin atom	WS_2 origin atom	Twist-angle (°)	γ-PC strain 1 (%)	γ-PC strain 2 (%)	γ-PC strain 3 (%)	WS_2 strain 1 (%)	WS_2 strain 2 (%)	WS_2 strain 3 (%)	γ (°)	|**a**|(Å)	|**b**|(Å)
4540	177	27	23	P	W	0.000	5.011	-2.245	-3.139	-4.554	2.396	3.349	82.081	10.878	18.222
4541	177	27	23	P	W	1.359	-3.292	0.067	5.191	3.523	-0.060	-4.703	82.200	18.221	10.878
4542	177	27	23	P	W	18.613	3.582	4.310	-1.889	-3.342	-4.479	1.963	82.457	14.719	13.444
4543	177	27	23	P	W	58.641	-3.292	-0.067	5.191	3.523	0.060	-4.702	82.200	18.221	10.878
4544	177	27	23	P	W	60.000	5.011	2.246	-3.139	-4.554	-2.396	3.349	82.081	10.878	18.222
4545	177	27	23	P	W	61.359	-3.292	0.067	5.191	3.523	-0.060	-4.703	82.200	18.221	10.878
4546	177	27	23	P	W	101.387	3.582	-4.310	-1.889	-3.342	4.479	1.963	82.457	13.444	14.719
4547	177	27	23	P	W	118.641	-3.292	-0.067	5.191	3.523	0.060	-4.702	82.200	18.221	10.878
4548	178	28	22	C	S	9.515	-4.497	-0.909	2.254	4.941	0.870	-2.157	87.286	11.961	16.362
4549	178	28	22	C	S	10.158	2.281	0.226	-4.521	-2.182	-0.248	4.970	87.335	16.362	11.961
4550	178	28	22	C	S	13.898	-0.414	-1.580	-2.048	0.417	1.647	2.136	86.125	11.445	17.100
4551	178	28	22	C	S	15.608	-2.145	1.369	-0.314	2.241	-1.378	0.316	86.154	17.102	11.444
4552	178	28	22	C	S	22.689	-4.497	-4.847	2.254	4.941	4.638	-2.157	89.491	11.961	16.344
4553	178	28	22	C	S	28.055	2.281	4.444	-4.521	-2.182	-4.886	4.970	89.864	16.362	11.948
4554	178	28	22	C	S	31.945	2.281	-4.444	-4.521	-2.182	4.886	4.970	89.864	16.362	11.948
4555	178	28	22	C	S	37.311	-4.497	4.847	2.254	4.941	-4.638	-2.157	89.491	11.961	16.344
4556	178	28	22	C	S	44.392	-2.145	-1.369	-0.314	2.241	1.378	0.316	86.154	17.102	11.444
4557	178	28	22	C	S	46.102	-0.414	1.580	-2.049	0.417	-1.647	2.136	86.125	11.445	17.100
4558	178	28	22	C	S	49.842	2.281	-0.226	-4.521	-2.182	0.248	4.970	87.335	16.362	11.961
4559	178	28	22	C	S	50.485	-4.497	0.909	2.254	4.941	-0.870	-2.157	87.286	11.961	16.362
4560	178	28	22	C	S	69.515	-4.497	-0.909	2.254	4.941	0.870	-2.157	87.286	11.961	16.362
4561	178	28	22	C	S	70.158	2.281	0.226	-4.521	-2.182	-0.248	4.970	87.335	16.362	11.961
4562	178	28	22	C	S	75.609	-2.145	1.369	-0.314	2.241	-1.378	0.316	86.154	17.102	11.444
4563	178	28	22	C	S	82.689	-4.497	-4.847	2.254	4.941	4.638	-2.157	89.491	11.961	16.344
4564	178	28	22	C	S	88.055	2.281	4.444	-4.521	-2.182	-4.886	4.970	89.864	16.362	11.948
4565	178	28	22	C	S	91.945	2.281	-4.444	-4.521	-2.182	4.886	4.970	89.864	16.362	11.948
4566	178	28	22	C	S	97.311	-4.497	4.847	2.254	4.941	-4.638	-2.157	89.491	11.961	16.344
4567	178	28	22	C	S	104.392	-2.145	-1.369	-0.314	2.241	1.378	0.316	86.154	17.102	11.444
4568	178	28	22	C	S	106.102	-0.414	1.580	-2.049	0.417	-1.647	2.136	86.125	11.445	17.100
4569	178	28	22	C	S	109.842	2.281	-0.226	-4.521	-2.182	0.248	4.970	87.335	16.362	11.961
4570	178	28	22	C	S	110.485	-4.497	0.909	2.254	4.941	-0.870	-2.157	87.286	11.961	16.362
4571	178	28	22	C	W	9.515	-4.497	-0.909	2.254	4.941	0.870	-2.157	87.286	11.961	16.362
4572	178	28	22	C	W	10.158	2.281	0.226	-4.521	-2.182	-0.248	4.970	87.335	16.362	11.961
4573	178	28	22	C	W	15.608	-2.145	1.369	-0.314	2.241	-1.378	0.316	86.154	17.102	11.444
4574	178	28	22	C	W	22.689	-4.497	-4.847	2.254	4.941	4.638	-2.157	89.491	11.961	16.344
4575	178	28	22	C	W	28.055	2.281	4.444	-4.521	-2.182	-4.886	4.970	89.864	16.362	11.948
4576	178	28	22	C	W	31.945	2.281	-4.444	-4.521	-2.182	4.886	4.970	89.864	16.362	11.948
4577	178	28	22	C	W	37.311	-4.497	4.847	2.254	4.941	-4.638	-2.157	89.491	11.961	16.344
4578	178	28	22	C	W	44.392	-2.145	-1.369	-0.314	2.241	1.378	0.316	86.154	17.102	11.444
4579	178	28	22	C	W	46.102	-0.414	1.580	-2.049	0.417	-1.647	2.136	86.125	11.445	17.100
4580	178	28	22	C	W	49.842	2.281	-0.226	-4.521	-2.182	0.248	4.970	87.335	16.362	11.961
4581	178	28	22	C	W	50.485	-4.497	0.909	2.254	4.941	-0.870	-2.157	87.286	11.961	16.362
4582	178	28	22	C	W	69.515	-4.497	-0.909	2.254	4.941	0.870	-2.157	87.286	11.961	16.362
4583	178	28	22	C	W	70.158	2.281	0.226	-4.521	-2.182	-0.248	4.970	87.335	16.362	11.961
4584	178	28	22	C	W	73.898	-0.414	-1.580	-2.048	0.417	1.647	2.136	86.125	11.445	17.100
4585	178	28	22	C	W	75.609	-2.145	1.369	-0.314	2.241	-1.378	0.316	86.154	17.102	11.444
4586	178	28	22	C	W	82.689	-4.497	-4.847	2.254	4.941	4.638	-2.157	89.491	11.961	16.344
4587	178	28	22	C	W	88.055	2.281	4.444	-4.521	-2.182	-4.886	4.970	89.864	16.362	11.948
4588	178	28	22	C	W	91.945	2.281	-4.444	-4.521	-2.182	4.886	4.970	89.864	16.362	11.948
4589	178	28	22	C	W	97.311	-4.497	4.847	2.254	4.941	-4.638	-2.157	89.491	11.961	16.344
4590	178	28	22	C	W	104.392	-2.145	-1.369	-0.314	2.241	1.378	0.316	86.154	17.102	11.444

BL number	Atoms	γ-PC unit cells	WS_2 unit cells	γ-PC origin atom	WS_2 origin atom	Twist-angle (°)	γ-PC strain 1 (%)	γ-PC strain 2 (%)	γ-PC strain 3 (%)	WS_2 strain 1 (%)	WS_2 strain 2 (%)	WS_2 strain 3 (%)	γ (°)	$\lvert\mathbf{a}\rvert$(Å)	$\lvert\mathbf{b}\rvert$(Å)
4591	178	28	22	C	W	109.842	2.281	-0.226	-4.521	-2.182	0.248	4.970	87.335	16.362	11.961
4592	178	28	22	C	W	110.485	-4.497	0.909	2.254	4.941	-0.870	-2.157	87.286	11.961	16.362
4593	178	28	22	P	S	15.608	-2.145	1.369	-0.314	2.241	-1.378	0.316	86.154	17.102	11.444
4594	178	28	22	P	S	22.689	-4.497	-4.847	2.254	4.941	4.638	-2.157	89.491	11.961	16.344
4595	178	28	22	P	S	28.055	2.281	4.444	-4.521	-2.182	-4.886	4.970	89.864	16.362	11.948
4596	178	28	22	P	S	31.945	2.281	-4.444	-4.521	-2.182	4.886	4.970	89.864	16.362	11.948
4597	178	28	22	P	S	37.311	-4.497	4.847	2.254	4.941	-4.638	-2.157	89.491	11.961	16.344
4598	178	28	22	P	S	44.392	-2.145	-1.369	-0.314	2.241	1.378	0.316	86.154	17.102	11.444
4599	178	28	22	P	S	46.102	-0.414	1.580	-2.049	0.417	-1.647	2.136	86.125	11.445	17.100
4600	178	28	22	P	S	49.842	2.281	-0.226	-4.521	-2.182	0.248	4.970	87.335	16.362	11.961
4601	178	28	22	P	S	50.485	-4.497	0.909	2.254	4.941	-0.870	-2.157	87.286	11.961	16.362
4602	178	28	22	P	S	69.515	-4.497	-0.909	2.254	4.941	0.870	-2.157	87.286	11.961	16.362
4603	178	28	22	P	S	70.158	2.281	0.226	-4.521	-2.182	-0.248	4.970	87.335	16.362	11.961
4604	178	28	22	P	S	73.898	-0.414	-1.580	-2.048	0.417	1.647	2.136	86.125	11.445	17.100
4605	178	28	22	P	S	75.609	-2.145	1.369	-0.314	2.241	-1.378	0.316	86.154	17.102	11.444
4606	178	28	22	P	S	82.689	-4.497	-4.847	2.254	4.941	4.638	-2.157	89.491	11.961	16.344
4607	178	28	22	P	S	88.055	2.281	4.444	-4.521	-2.182	-4.886	4.970	89.864	16.362	11.948
4608	178	28	22	P	S	91.945	2.281	-4.444	-4.521	-2.182	4.886	4.970	89.864	16.362	11.948
4609	178	28	22	P	S	97.311	-4.497	4.847	2.254	4.941	-4.638	-2.157	89.491	11.961	16.344
4610	178	28	22	P	S	104.392	-2.145	-1.369	-0.314	2.241	1.378	0.316	86.154	17.102	11.444
4611	178	28	22	P	S	109.842	2.281	-0.226	-4.521	-2.182	0.248	4.970	87.335	16.362	11.961
4612	178	28	22	P	W	9.515	-4.497	-0.909	2.254	4.941	0.870	-2.157	87.286	11.961	16.362
4613	178	28	22	P	W	10.158	2.281	0.226	-4.521	-2.182	-0.248	4.970	87.335	16.362	11.961
4614	178	28	22	P	W	13.898	-0.414	-1.580	-2.048	0.417	1.647	2.136	86.125	11.445	17.100
4615	178	28	22	P	W	15.608	-2.145	1.369	-0.314	2.241	-1.378	0.316	86.154	17.102	11.444
4616	178	28	22	P	W	22.689	-4.497	-4.847	2.254	4.941	4.638	-2.157	89.491	11.961	16.344
4617	178	28	22	P	W	28.055	2.281	4.444	-4.521	-2.182	-4.886	4.970	89.864	16.362	11.948
4618	178	28	22	P	W	31.945	2.281	-4.444	-4.521	-2.182	4.886	4.970	89.864	16.362	11.948
4619	178	28	22	P	W	37.311	-4.497	4.847	2.254	4.941	-4.638	-2.157	89.491	11.961	16.344
4620	178	28	22	P	W	44.392	-2.145	-1.369	-0.314	2.241	1.378	0.316	86.154	17.102	11.444
4621	178	28	22	P	W	46.102	-0.414	1.580	-2.049	0.417	-1.647	2.136	86.125	11.445	17.100
4622	178	28	22	P	W	49.842	2.281	-0.226	-4.521	-2.182	0.248	4.970	87.335	16.362	11.961
4623	178	28	22	P	W	75.609	-2.145	1.369	-0.314	2.241	-1.378	0.316	86.154	17.102	11.444
4624	178	28	22	P	W	82.689	-4.497	-4.847	2.254	4.941	4.638	-2.157	89.491	11.961	16.344
4625	178	28	22	P	W	88.055	2.281	4.444	-4.521	-2.182	-4.886	4.970	89.864	16.362	11.948
4626	178	28	22	P	W	91.945	2.281	-4.444	-4.521	-2.182	4.886	4.970	89.864	16.362	11.948
4627	178	28	22	P	W	97.311	-4.497	4.847	2.254	4.941	-4.638	-2.157	89.491	11.961	16.344
4628	178	28	22	P	W	104.392	-2.145	-1.369	-0.314	2.241	1.378	0.316	86.154	17.102	11.444
4629	178	28	22	P	W	106.102	-0.414	1.580	-2.049	0.417	-1.647	2.136	86.125	11.445	17.100
4630	178	28	22	P	W	109.842	2.281	-0.226	-4.521	-2.182	0.248	4.970	87.335	16.362	11.961
4631	178	28	22	P	W	110.485	-4.497	0.909	2.254	4.941	-0.870	-2.157	87.286	11.961	16.362
4632	179	26	25	C	S	17.897	5.011	-3.498	2.895	-4.554	3.306	-2.737	69.620	13.809	15.516
4633	179	26	25	C	S	21.787	5.011	3.498	2.895	-4.554	-3.306	-2.737	69.498	13.826	15.509
4634	179	26	25	C	S	38.213	5.011	-3.498	2.895	-4.554	3.306	-2.737	69.498	13.826	15.509
4635	179	26	25	C	S	42.103	5.011	3.498	2.895	-4.554	-3.306	-2.737	69.620	15.516	13.809
4636	179	26	25	C	S	77.897	5.011	-3.498	2.895	-4.554	3.306	-2.737	69.620	13.809	15.516
4637	179	26	25	C	S	81.787	5.011	3.498	2.895	-4.554	-3.306	-2.737	69.498	13.826	15.509
4638	179	26	25	C	S	98.213	5.011	-3.498	2.895	-4.554	3.306	-2.737	69.498	13.826	15.509
4639	179	26	25	C	S	102.104	5.011	3.498	2.895	-4.554	-3.306	-2.737	69.620	15.516	13.809
4640	179	26	25	C	W	17.897	5.011	-3.498	2.895	-4.554	3.306	-2.737	69.620	13.809	15.516
4641	179	26	25	C	W	21.787	5.011	3.498	2.895	-4.554	-3.306	-2.737	69.498	13.826	15.509

BL number	Atoms	γ-PC unit cells	WS_2 unit cells	γ-PC origin atom	WS_2 origin atom	Twist-angle (°)	γ-PC strain 1 (%)	γ-PC strain 2 (%)	γ-PC strain 3 (%)	WS_2 strain 1 (%)	WS_2 strain 2 (%)	WS_2 strain 3 (%)	γ (°)	|**a**|(Å)	|**b**|(Å)
4642	179	26	25	C	W	38.213	5.011	-3.498	2.895	-4.554	3.306	-2.737	69.498	13.826	15.509
4643	179	26	25	C	W	42.103	5.011	3.498	2.895	-4.554	-3.306	-2.737	69.620	15.516	13.809
4644	179	26	25	C	W	77.897	5.011	-3.498	2.895	-4.554	3.306	-2.737	69.620	13.809	15.516
4645	179	26	25	C	W	81.787	5.011	3.498	2.895	-4.554	-3.306	-2.737	69.498	13.826	15.509
4646	179	26	25	C	W	98.213	5.011	-3.498	2.895	-4.554	3.306	-2.737	69.498	13.826	15.509
4647	179	26	25	C	W	102.104	5.011	3.498	2.895	-4.554	-3.306	-2.737	69.620	15.516	13.809
4648	179	26	25	P	S	38.213	5.011	-3.498	2.895	-4.554	3.306	-2.737	69.498	13.826	15.509
4649	179	26	25	P	S	42.103	5.011	3.498	2.895	-4.554	-3.306	-2.737	69.620	15.516	13.809
4650	179	26	25	P	S	77.897	5.011	-3.498	2.895	-4.554	3.306	-2.737	69.620	13.809	15.516
4651	179	26	25	P	S	81.787	5.011	3.498	2.895	-4.554	-3.306	-2.737	69.498	13.826	15.509
4652	179	26	25	P	W	17.897	5.011	-3.498	2.895	-4.554	3.306	-2.737	69.620	13.809	15.516
4653	179	26	25	P	W	21.787	5.011	3.498	2.895	-4.554	-3.306	-2.737	69.498	13.826	15.509
4654	179	26	25	P	W	98.213	5.011	-3.498	2.895	-4.554	3.306	-2.737	69.498	13.826	15.509
4655	179	26	25	P	W	102.104	5.011	3.498	2.895	-4.554	-3.306	-2.737	69.620	15.516	13.809
4656	179	29	21	C	S	4.715	-3.007	-4.506	-3.367	3.200	4.831	3.611	73.665	16.951	11.946
4657	179	29	21	C	S	9.515	-4.497	3.656	-1.841	4.941	-3.796	1.912	73.720	11.961	16.928
4658	179	29	21	C	S	50.485	-4.497	-3.656	-1.841	4.941	3.796	1.912	73.720	11.961	16.928
4659	179	29	21	C	S	55.285	-3.007	4.506	-3.367	3.200	-4.831	3.611	73.665	16.951	11.946
4660	179	29	21	C	S	64.715	-3.007	-4.506	-3.367	3.200	4.831	3.611	73.665	16.951	11.946
4661	179	29	21	C	S	69.515	-4.497	3.656	-1.841	4.941	-3.796	1.912	73.720	11.961	16.928
4662	179	29	21	C	S	110.485	-4.497	-3.656	-1.841	4.941	3.796	1.912	73.720	11.961	16.928
4663	179	29	21	C	S	115.285	-3.007	4.506	-3.367	3.200	-4.831	3.611	73.665	16.951	11.946
4664	179	29	21	C	W	4.715	-3.007	-4.506	-3.367	3.200	4.831	3.611	73.665	16.951	11.946
4665	179	29	21	C	W	9.515	-4.497	3.656	-1.841	4.941	-3.796	1.912	73.720	11.961	16.928
4666	179	29	21	C	W	50.485	-4.497	-3.656	-1.841	4.941	3.796	1.912	73.720	11.961	16.928
4667	179	29	21	C	W	55.285	-3.007	4.506	-3.367	3.200	-4.831	3.611	73.665	16.951	11.946
4668	179	29	21	C	W	64.715	-3.007	-4.506	-3.367	3.200	4.831	3.611	73.665	16.951	11.946
4669	179	29	21	C	W	69.515	-4.497	3.656	-1.841	4.941	-3.796	1.912	73.720	11.961	16.928
4670	179	29	21	C	W	110.485	-4.497	-3.656	-1.841	4.941	3.796	1.912	73.720	11.961	16.928
4671	179	29	21	C	W	115.285	-3.007	4.506	-3.367	3.200	-4.831	3.611	73.665	16.951	11.946
4672	179	29	21	P	S	4.715	-3.007	-4.506	-3.367	3.200	4.831	3.611	73.665	16.951	11.946
4673	179	29	21	P	S	9.515	-4.497	3.656	-1.841	4.941	-3.796	1.912	73.720	11.961	16.928
4674	179	29	21	P	S	50.485	-4.497	-3.656	-1.841	4.941	3.796	1.912	73.720	11.961	16.928
4675	179	29	21	P	S	55.285	-3.007	4.506	-3.367	3.200	-4.831	3.611	73.665	16.951	11.946
4676	179	29	21	P	S	64.715	-3.007	-4.506	-3.367	3.200	4.831	3.611	73.665	16.951	11.946
4677	179	29	21	P	S	69.515	-4.497	3.656	-1.841	4.941	-3.796	1.912	73.720	11.961	16.928
4678	179	29	21	P	S	110.485	-4.497	-3.656	-1.841	4.941	3.796	1.912	73.720	11.961	16.928
4679	179	29	21	P	S	115.285	-3.007	4.506	-3.367	3.200	-4.831	3.611	73.665	16.951	11.946
4680	179	29	21	P	W	4.715	-3.007	-4.506	-3.367	3.200	4.831	3.611	73.665	16.951	11.946
4681	179	29	21	P	W	9.515	-4.497	3.656	-1.841	4.941	-3.796	1.912	73.720	11.961	16.928
4682	179	29	21	P	W	50.485	-4.497	-3.656	-1.841	4.941	3.796	1.912	73.720	11.961	16.928
4683	179	29	21	P	W	55.285	-3.007	4.506	-3.367	3.200	-4.831	3.611	73.665	16.951	11.946
4684	179	29	21	P	W	64.715	-3.007	-4.506	-3.367	3.200	4.831	3.611	73.665	16.951	11.946
4685	179	29	21	P	W	69.515	-4.497	3.656	-1.841	4.941	-3.796	1.912	73.720	11.961	16.928
4686	179	29	21	P	W	110.485	-4.497	-3.656	-1.841	4.941	3.796	1.912	73.720	11.961	16.928
4687	179	29	21	P	W	115.285	-3.007	4.506	-3.367	3.200	-4.831	3.611	73.665	16.951	11.946
4688	180	27	24	C	S	19.107	-1.485	-1.354	5.446	1.530	1.221	-4.911	80.522	14.708	13.822
4689	180	27	24	C	S	21.787	5.011	3.368	-1.101	-4.554	-3.444	1.126	80.336	13.826	14.708
4690	180	27	24	C	S	23.413	0.481	-3.760	3.286	-0.477	3.528	-3.083	76.828	12.584	16.349
4691	180	27	24	C	S	25.285	4.262	-0.372	-0.427	-3.928	0.375	0.430	76.824	16.356	12.583
4692	180	27	24	C	S	28.055	2.281	4.530	1.452	-2.182	-4.403	-1.411	76.743	16.362	12.577

BL number	Atoms	γ-PC unit cells	WS_2 unit cells	γ-PC origin atom	WS_2 origin atom	Twist-angle (°)	γ-PC strain 1 (%)	γ-PC strain 2 (%)	γ-PC strain 3 (%)	WS_2 strain 1 (%)	WS_2 strain 2 (%)	WS_2 strain 3 (%)	γ (°)	|**a**|(Å)	|**b**|(Å)
4693	180	27	24	C	S	31.945	2.281	-4.530	1.452	-2.182	4.403	-1.411	76.743	16.362	12.577
4694	180	27	24	C	S	34.715	4.262	0.372	-0.427	-3.928	-0.375	0.430	76.824	12.583	16.356
4695	180	27	24	C	S	36.587	0.481	3.760	3.286	-0.477	-3.528	-3.083	76.828	12.584	16.349
4696	180	27	24	C	S	38.213	5.011	-3.368	-1.101	-4.554	3.444	1.126	80.336	13.826	14.708
4697	180	27	24	C	S	40.893	-1.485	1.354	5.446	1.530	-1.221	-4.911	80.521	14.708	13.822
4698	180	27	24	C	S	79.107	-1.485	-1.354	5.446	1.530	1.221	-4.911	80.521	14.708	13.822
4699	180	27	24	C	S	83.413	0.481	-3.760	3.286	-0.477	3.528	-3.083	76.828	12.584	16.349
4700	180	27	24	C	S	85.285	4.262	-0.372	-0.427	-3.928	0.375	0.430	76.824	16.356	12.583
4701	180	27	24	C	S	88.055	2.281	4.530	1.452	-2.182	-4.403	-1.411	76.743	16.362	12.577
4702	180	27	24	C	S	91.945	2.281	-4.530	1.452	-2.182	4.403	-1.411	76.743	16.362	12.577
4703	180	27	24	C	S	94.715	4.262	0.372	-0.427	-3.928	-0.375	0.430	76.824	12.583	16.356
4704	180	27	24	C	S	96.587	0.481	3.760	3.286	-0.477	-3.528	-3.083	76.828	12.584	16.349
4705	180	27	24	C	S	98.213	5.011	-3.368	-1.101	-4.554	3.444	1.126	80.336	13.826	14.708
4706	180	27	24	C	S	100.893	-1.485	1.354	5.446	1.530	-1.221	-4.911	80.521	14.708	13.822
4707	180	27	24	C	W	23.413	0.481	-3.760	3.286	-0.477	3.528	-3.083	76.828	12.584	16.349
4708	180	27	24	C	W	25.285	4.262	-0.372	-0.427	-3.928	0.375	0.430	76.824	16.356	12.583
4709	180	27	24	C	W	28.055	2.281	4.530	1.452	-2.182	-4.403	-1.411	76.743	16.362	12.577
4710	180	27	24	C	W	31.945	2.281	-4.530	1.452	-2.182	4.403	-1.411	76.743	16.362	12.577
4711	180	27	24	C	W	34.715	4.262	0.372	-0.427	-3.928	-0.375	0.430	76.824	12.583	16.356
4712	180	27	24	C	W	36.587	0.481	3.760	3.286	-0.477	-3.528	-3.083	76.828	12.584	16.349
4713	180	27	24	C	W	81.787	5.011	3.368	-1.101	-4.554	-3.444	1.126	80.336	13.826	14.708
4714	180	27	24	C	W	83.413	0.481	-3.760	3.286	-0.477	3.528	-3.083	76.828	12.584	16.349
4715	180	27	24	C	W	85.285	4.262	-0.372	-0.427	-3.928	0.375	0.430	76.824	16.356	12.583
4716	180	27	24	C	W	88.055	2.281	4.530	1.452	-2.182	-4.403	-1.411	76.743	16.362	12.577
4717	180	27	24	C	W	91.945	2.281	-4.530	1.452	-2.182	4.403	-1.411	76.743	16.362	12.577
4718	180	27	24	C	W	94.715	4.262	0.372	-0.427	-3.928	-0.375	0.430	76.824	12.583	16.356
4719	180	27	24	C	W	96.587	0.481	3.760	3.286	-0.477	-3.528	-3.083	76.828	12.584	16.349
4720	180	27	24	P	S	19.107	-1.485	-1.354	5.446	1.530	1.221	-4.911	80.522	14.708	13.822
4721	180	27	24	P	S	21.787	5.011	3.368	-1.101	-4.554	-3.444	1.126	80.336	13.826	14.708
4722	180	27	24	P	S	23.413	0.481	-3.760	3.286	-0.477	3.528	-3.083	76.828	12.584	16.349
4723	180	27	24	P	S	25.285	4.262	-0.372	-0.427	-3.928	0.375	0.430	76.824	16.356	12.583
4724	180	27	24	P	S	28.055	2.281	4.530	1.452	-2.182	-4.403	-1.411	76.743	16.362	12.577
4725	180	27	24	P	S	31.945	2.281	-4.530	1.452	-2.182	4.403	-1.411	76.743	16.362	12.577
4726	180	27	24	P	S	34.715	4.262	0.372	-0.427	-3.928	-0.375	0.430	76.824	12.583	16.356
4727	180	27	24	P	S	36.587	0.481	3.760	3.286	-0.477	-3.528	-3.083	76.828	12.584	16.349
4728	180	27	24	P	S	38.213	5.011	-3.368	-1.101	-4.554	3.444	1.126	80.336	13.826	14.708
4729	180	27	24	P	S	40.893	-1.485	1.354	5.446	1.530	-1.221	-4.911	80.521	14.708	13.822
4730	180	27	24	P	S	79.107	-1.485	-1.354	5.446	1.530	1.221	-4.911	80.521	14.708	13.822
4731	180	27	24	P	S	83.413	0.481	-3.760	3.286	-0.477	3.528	-3.083	76.828	12.584	16.349
4732	180	27	24	P	S	85.285	4.262	-0.372	-0.427	-3.928	0.375	0.430	76.824	16.356	12.583
4733	180	27	24	P	S	88.055	2.281	4.530	1.452	-2.182	-4.403	-1.411	76.743	16.362	12.577
4734	180	27	24	P	S	91.945	2.281	-4.530	1.452	-2.182	4.403	-1.411	76.743	16.362	12.577
4735	180	27	24	P	S	94.715	4.262	0.372	-0.427	-3.928	-0.375	0.430	76.824	12.583	16.356
4736	180	27	24	P	S	96.587	0.481	3.760	3.286	-0.477	-3.528	-3.083	76.828	12.584	16.349
4737	180	27	24	P	S	98.213	5.011	-3.368	-1.101	-4.554	3.444	1.126	80.336	13.826	14.708
4738	180	27	24	P	S	100.893	-1.485	1.354	5.446	1.530	-1.221	-4.911	80.521	14.708	13.822
4739	180	27	24	P	W	23.413	0.481	-3.760	3.286	-0.477	3.528	-3.083	76.828	12.584	16.349
4740	180	27	24	P	W	25.285	4.262	-0.372	-0.427	-3.928	0.375	0.430	76.824	16.356	12.583
4741	180	27	24	P	W	28.055	2.281	4.530	1.452	-2.182	-4.403	-1.411	76.743	16.362	12.577
4742	180	27	24	P	W	31.945	2.281	-4.530	1.452	-2.182	4.403	-1.411	76.743	16.362	12.577
4743	180	27	24	P	W	34.715	4.262	0.372	-0.427	-3.928	-0.375	0.430	76.824	12.583	16.356

BL number	Atoms	γ-PC unit cells	WS_2 unit cells	γ-PC origin atom	WS_2 origin atom	Twist-angle (°)	γ-PC strain 1 (%)	γ-PC strain 2 (%)	γ-PC strain 3 (%)	WS_2 strain 1 (%)	WS_2 strain 2 (%)	WS_2 strain 3 (%)	γ (°)	$\lvert\mathbf{a}\rvert$(Å)	$\lvert\mathbf{b}\rvert$(Å)
4744	180	27	24	P	W	36.587	0.481	3.760	3.286	-0.477	-3.528	-3.083	76.828	12.584	16.349
4745	180	27	24	P	W	81.787	5.011	3.368	-1.101	-4.554	-3.444	1.126	80.336	13.826	14.708
4746	180	27	24	P	W	83.413	0.481	-3.760	3.286	-0.477	3.528	-3.083	76.828	12.584	16.349
4747	180	27	24	P	W	85.285	4.262	-0.372	-0.427	-3.928	0.375	0.430	76.824	16.356	12.583
4748	180	27	24	P	W	88.055	2.281	4.530	1.452	-2.182	-4.403	-1.411	76.743	16.362	12.577
4749	180	27	24	P	W	91.945	2.281	-4.530	1.452	-2.182	4.403	-1.411	76.743	16.362	12.577
4750	180	27	24	P	W	94.715	4.262	0.372	-0.427	-3.928	-0.375	0.430	76.824	12.583	16.356
4751	180	27	24	P	W	96.587	0.481	3.760	3.286	-0.477	-3.528	-3.083	76.828	12.584	16.349
4752	181	28	23	C	S	0.000	5.011	1.083	-4.812	-4.554	-1.198	5.325	86.745	13.151	15.239
4753	181	28	23	C	S	1.945	-4.723	2.386	4.902	5.216	-2.173	-4.464	86.555	15.242	13.150
4754	181	28	23	C	S	4.307	-4.685	4.344	4.856	5.169	-3.960	-4.426	87.628	14.491	13.818
4755	181	28	23	C	S	8.213	-2.359	-4.027	2.178	2.476	3.859	-2.087	87.743	14.845	13.464
4756	181	28	23	C	S	8.513	2.390	-3.329	-2.552	-2.281	3.508	2.689	79.434	11.429	17.777
4757	181	28	23	C	S	12.520	2.326	3.498	-2.494	-2.223	-3.682	2.625	87.945	13.473	14.834
4758	181	28	23	C	S	13.898	-0.414	-2.211	0.131	0.417	2.206	-0.131	84.056	11.445	17.535
4759	181	28	23	C	S	16.337	0.353	2.036	-0.633	-0.351	-2.062	0.641	84.075	17.539	11.442
4760	181	28	23	C	S	17.480	-4.685	-4.345	4.856	5.169	3.960	-4.426	87.627	14.491	13.818
4761	181	28	23	C	S	17.897	5.011	-3.248	-4.812	-4.554	3.594	5.325	85.448	16.799	11.949
4762	181	28	23	C	S	21.787	5.011	3.248	-4.812	-4.554	-3.594	5.325	88.046	13.826	14.480
4763	181	28	23	C	S	22.689	-4.497	5.226	4.629	4.941	-4.783	-4.237	84.994	11.961	16.788
4764	181	28	23	C	S	25.693	2.326	-3.498	-2.494	-2.223	3.682	2.625	87.945	13.473	14.834
4765	181	28	23	C	S	30.000	-2.359	-4.027	2.178	2.476	3.859	-2.087	87.743	14.845	13.464
4766	181	28	23	C	S	34.307	2.326	3.498	-2.494	-2.223	-3.682	2.625	87.945	13.473	14.834
4767	181	28	23	C	S	37.311	-4.497	-5.226	4.629	4.941	4.783	-4.237	84.994	11.961	16.788
4768	181	28	23	C	S	38.213	5.011	-3.248	-4.812	-4.554	3.594	5.325	88.046	13.826	14.480
4769	181	28	23	C	S	42.103	5.011	3.248	-4.812	-4.554	-3.594	5.325	85.448	16.799	11.949
4770	181	28	23	C	S	42.520	-4.685	4.344	4.856	5.169	-3.960	-4.426	87.627	14.491	13.818
4771	181	28	23	C	S	43.663	0.353	-2.036	-0.633	-0.351	2.062	0.641	84.075	17.539	11.442
4772	181	28	23	C	S	46.102	-0.414	2.211	0.131	0.417	-2.206	-0.131	84.056	11.445	17.535
4773	181	28	23	C	S	47.480	2.326	-3.498	-2.494	-2.223	3.682	2.625	87.945	13.473	14.834
4774	181	28	23	C	S	51.487	2.390	3.329	-2.552	-2.281	-3.508	2.689	79.434	17.777	11.429
4775	181	28	23	C	S	51.787	-2.359	4.027	2.178	2.476	-3.859	-2.087	87.743	14.845	13.464
4776	181	28	23	C	S	55.693	-4.685	-4.345	4.856	5.169	3.960	-4.426	87.627	14.491	13.818
4777	181	28	23	C	S	58.055	-4.723	-2.385	4.902	5.216	2.172	-4.464	86.555	15.242	13.150
4778	181	28	23	C	S	60.000	5.011	3.248	-4.812	-4.554	-3.594	5.325	88.046	13.826	14.480
4779	181	28	23	C	S	61.945	-4.723	2.386	4.902	5.216	-2.173	-4.464	86.555	15.242	13.150
4780	181	28	23	C	S	64.307	-4.685	4.344	4.856	5.169	-3.960	-4.426	87.627	14.491	13.818
4781	181	28	23	C	S	68.213	-2.359	-4.027	2.178	2.476	3.859	-2.087	87.743	14.845	13.464
4782	181	28	23	C	S	68.513	2.390	-3.329	-2.552	-2.281	3.508	2.689	79.434	11.429	17.777
4783	181	28	23	C	S	72.520	2.326	3.498	-2.494	-2.223	-3.682	2.625	87.945	13.473	14.834
4784	181	28	23	C	S	73.898	-0.414	-2.211	0.131	0.417	2.206	-0.131	84.056	11.445	17.535
4785	181	28	23	C	S	76.337	0.353	2.036	-0.633	-0.351	-2.062	0.641	84.075	17.539	11.442
4786	181	28	23	C	S	77.480	-4.685	-4.345	4.856	5.169	3.960	-4.426	87.627	14.491	13.818
4787	181	28	23	C	S	77.897	5.011	-3.248	-4.812	-4.554	3.594	5.325	85.448	16.799	11.949
4788	181	28	23	C	S	81.787	5.011	3.248	-4.812	-4.554	-3.594	5.325	88.046	13.826	14.480
4789	181	28	23	C	S	82.689	-4.497	5.226	4.629	4.941	-4.783	-4.237	84.994	11.961	16.788
4790	181	28	23	C	S	85.694	2.326	-3.498	-2.494	-2.223	3.682	2.625	87.945	13.473	14.834
4791	181	28	23	C	S	90.000	-2.359	4.027	2.178	2.476	-3.859	-2.087	87.743	14.845	13.464
4792	181	28	23	C	S	94.307	2.326	3.498	-2.494	-2.223	-3.682	2.625	87.945	13.473	14.834
4793	181	28	23	C	S	97.311	-4.497	-5.226	4.629	4.941	4.783	-4.237	84.994	11.961	16.788
4794	181	28	23	C	S	98.213	5.011	-3.248	-4.812	-4.554	3.594	5.325	88.046	13.826	14.480

BL number	Atoms	γ-PC unit cells	WS_2 unit cells	γ-PC origin atom	WS_2 origin atom	Twist-angle (°)	γ-PC strain 1 (%)	γ-PC strain 2 (%)	γ-PC strain 3 (%)	WS_2 strain 1 (%)	WS_2 strain 2 (%)	WS_2 strain 3 (%)	γ (°)	$\lvert\mathbf{a}\rvert$(Å)	$\lvert\mathbf{b}\rvert$(Å)
4795	181	28	23	C	S	102.104	5.011	3.248	-4.812	-4.554	-3.594	5.325	85.448	16.799	11.949
4796	181	28	23	C	S	102.520	-4.685	4.344	4.856	5.169	-3.960	-4.426	87.627	14.491	13.818
4797	181	28	23	C	S	103.663	0.353	-2.036	-0.633	-0.351	2.062	0.641	84.075	17.539	11.442
4798	181	28	23	C	S	106.102	-0.414	2.211	0.131	0.417	-2.206	-0.131	84.056	11.445	17.535
4799	181	28	23	C	S	107.480	2.326	-3.498	-2.494	-2.223	3.682	2.625	87.945	13.473	14.834
4800	181	28	23	C	S	111.487	2.390	3.329	-2.552	-2.281	-3.508	2.689	79.434	17.777	11.429
4801	181	28	23	C	S	111.787	-2.359	4.027	2.178	2.476	-3.859	-2.087	87.743	14.845	13.464
4802	181	28	23	C	S	115.693	-4.685	-4.345	4.856	5.169	3.960	-4.426	87.627	14.491	13.818
4803	181	28	23	C	S	118.055	-4.723	-2.385	4.902	5.216	2.172	-4.464	86.555	15.242	13.150
4804	181	28	23	C	W	0.000	5.011	1.083	-4.812	-4.554	-1.198	5.325	86.745	13.151	15.239
4805	181	28	23	C	W	1.945	-4.723	2.386	4.902	5.216	-2.173	-4.464	86.555	15.242	13.150
4806	181	28	23	C	W	4.307	-4.685	4.344	4.856	5.169	-3.960	-4.426	87.628	14.491	13.818
4807	181	28	23	C	W	8.213	-2.359	-4.027	2.178	2.476	3.859	-2.087	87.743	14.845	13.464
4808	181	28	23	C	W	8.513	2.390	-3.329	-2.552	-2.281	3.508	2.689	79.434	11.429	17.777
4809	181	28	23	C	W	12.520	2.326	3.498	-2.494	-2.223	-3.682	2.625	87.945	13.473	14.834
4810	181	28	23	C	W	13.898	-0.414	-2.211	0.131	0.417	2.206	-0.131	84.056	11.445	17.535
4811	181	28	23	C	W	16.337	0.353	2.036	-0.633	-0.351	-2.062	0.641	84.075	17.539	11.442
4812	181	28	23	C	W	17.480	-4.685	-4.345	4.856	5.169	3.960	-4.426	87.627	14.491	13.818
4813	181	28	23	C	W	17.897	5.011	-3.248	-4.812	-4.554	3.594	5.325	85.448	16.799	11.949
4814	181	28	23	C	W	21.787	5.011	3.248	-4.812	-4.554	-3.594	5.325	88.046	13.826	14.480
4815	181	28	23	C	W	22.689	-4.497	5.226	4.629	4.941	-4.783	-4.237	84.994	11.961	16.788
4816	181	28	23	C	W	25.693	2.326	-3.498	-2.494	-2.223	3.682	2.625	87.945	13.473	14.834
4817	181	28	23	C	W	30.000	-2.359	-4.027	2.178	2.476	3.859	-2.087	87.743	14.845	13.464
4818	181	28	23	C	W	34.307	2.326	3.498	-2.494	-2.223	-3.682	2.625	87.945	13.473	14.834
4819	181	28	23	C	W	37.311	-4.497	-5.226	4.629	4.941	4.783	-4.237	84.994	11.961	16.788
4820	181	28	23	C	W	38.213	5.011	-3.248	-4.812	-4.554	3.594	5.325	88.046	13.826	14.480
4821	181	28	23	C	W	42.103	5.011	3.248	-4.812	-4.554	-3.594	5.325	85.448	16.799	11.949
4822	181	28	23	C	W	42.520	-4.685	4.344	4.856	5.169	-3.960	-4.426	87.627	14.491	13.818
4823	181	28	23	C	W	43.663	0.353	-2.036	-0.633	-0.351	2.062	0.641	84.075	17.539	11.442
4824	181	28	23	C	W	46.102	-0.414	2.211	0.131	0.417	-2.206	-0.131	84.056	11.445	17.535
4825	181	28	23	C	W	47.480	2.326	-3.498	-2.494	-2.223	3.682	2.625	87.945	13.473	14.834
4826	181	28	23	C	W	51.487	2.390	3.329	-2.552	-2.281	-3.508	2.689	79.434	17.777	11.429
4827	181	28	23	C	W	51.787	-2.359	4.027	2.178	2.476	-3.859	-2.087	87.743	14.845	13.464
4828	181	28	23	C	W	55.693	-4.685	-4.345	4.856	5.169	3.960	-4.426	87.627	14.491	13.818
4829	181	28	23	C	W	58.055	-4.723	-2.385	4.902	5.216	2.172	-4.464	86.555	15.242	13.150
4830	181	28	23	C	W	60.000	5.011	3.248	-4.812	-4.554	-3.594	5.325	88.046	13.826	14.480
4831	181	28	23	C	W	61.945	-4.723	2.386	4.902	5.216	-2.173	-4.464	86.555	15.242	13.150
4832	181	28	23	C	W	64.307	-4.685	4.344	4.856	5.169	-3.960	-4.426	87.627	14.491	13.818
4833	181	28	23	C	W	68.213	-2.359	-4.027	2.178	2.476	3.859	-2.087	87.743	14.845	13.464
4834	181	28	23	C	W	68.513	2.390	-3.329	-2.552	-2.281	3.508	2.689	79.434	11.429	17.777
4835	181	28	23	C	W	72.520	2.326	3.498	-2.494	-2.223	-3.682	2.625	87.945	13.473	14.834
4836	181	28	23	C	W	73.898	-0.414	-2.211	0.131	0.417	2.206	-0.131	84.056	11.445	17.535
4837	181	28	23	C	W	76.337	0.353	2.036	-0.633	-0.351	-2.062	0.641	84.075	17.539	11.442
4838	181	28	23	C	W	77.480	-4.685	-4.345	4.856	5.169	3.960	-4.426	87.627	14.491	13.818
4839	181	28	23	C	W	77.897	5.011	-3.248	-4.812	-4.554	3.594	5.325	85.448	16.799	11.949
4840	181	28	23	C	W	81.787	5.011	3.248	-4.812	-4.554	-3.594	5.325	88.046	13.826	14.480
4841	181	28	23	C	W	82.689	-4.497	5.226	4.629	4.941	-4.783	-4.237	84.994	11.961	16.788
4842	181	28	23	C	W	85.694	2.326	-3.498	-2.494	-2.223	3.682	2.625	87.945	13.473	14.834
4843	181	28	23	C	W	90.000	-2.359	4.027	2.178	2.476	-3.859	-2.087	87.743	14.845	13.464
4844	181	28	23	C	W	94.307	2.326	3.498	-2.494	-2.223	-3.682	2.625	87.945	13.473	14.834
4845	181	28	23	C	W	97.311	-4.497	-5.226	4.629	4.941	4.783	-4.237	84.994	11.961	16.788

BL number	Atoms	γ-PC unit cells	WS_2 unit cells	γ-PC origin atom	WS_2 origin atom	Twist-angle (°)	γ-PC strain 1 (%)	γ-PC strain 2 (%)	γ-PC strain 3 (%)	WS_2 strain 1 (%)	WS_2 strain 2 (%)	WS_2 strain 3 (%)	γ (°)	|**a**|(Å)	|**b**|(Å)
4846	181	28	23	C	W	98.213	5.011	-3.248	-4.812	-4.554	3.594	5.325	88.046	13.826	14.480
4847	181	28	23	C	W	102.104	5.011	3.248	-4.812	-4.554	-3.594	5.325	85.448	16.799	11.949
4848	181	28	23	C	W	102.520	-4.685	4.344	4.856	5.169	-3.960	-4.426	87.627	14.491	13.818
4849	181	28	23	C	W	103.663	0.353	-2.036	-0.633	-0.351	2.062	0.641	84.075	17.539	11.442
4850	181	28	23	C	W	106.102	-0.414	2.211	0.131	0.417	-2.206	-0.131	84.056	11.445	17.535
4851	181	28	23	C	W	107.480	2.326	-3.498	-2.494	-2.223	3.682	2.625	87.945	13.473	14.834
4852	181	28	23	C	W	111.487	2.390	3.329	-2.552	-2.281	-3.508	2.689	79.434	17.777	11.429
4853	181	28	23	C	W	111.787	-2.359	4.027	2.178	2.476	-3.859	-2.087	87.743	14.845	13.464
4854	181	28	23	C	W	115.693	-4.685	-4.345	4.856	5.169	3.960	-4.426	87.627	14.491	13.818
4855	181	28	23	C	W	118.055	-4.723	-2.385	4.902	5.216	2.172	-4.464	86.555	15.242	13.150
4856	181	28	23	P	S	0.000	5.011	1.083	-4.812	-4.554	-1.198	5.325	86.745	13.151	15.239
4857	181	28	23	P	S	1.945	-4.723	2.386	4.902	5.216	-2.173	-4.464	86.555	15.242	13.150
4858	181	28	23	P	S	4.307	-4.685	4.344	4.856	5.169	-3.960	-4.426	87.628	14.491	13.818
4859	181	28	23	P	S	8.213	-2.359	-4.027	2.178	2.476	3.859	-2.087	87.743	14.845	13.464
4860	181	28	23	P	S	8.513	2.390	-3.329	-2.552	-2.281	3.508	2.689	79.434	11.429	17.777
4861	181	28	23	P	S	12.520	2.326	3.498	-2.494	-2.223	-3.682	2.625	87.945	13.473	14.834
4862	181	28	23	P	S	13.898	-0.414	-2.211	0.131	0.417	2.206	-0.131	84.056	11.445	17.535
4863	181	28	23	P	S	16.337	0.353	2.036	-0.633	-0.351	-2.062	0.641	84.075	17.539	11.442
4864	181	28	23	P	S	25.693	2.326	-3.498	-2.494	-2.223	3.682	2.625	87.945	13.473	14.834
4865	181	28	23	P	S	30.000	-2.359	-4.027	2.178	2.476	3.859	-2.087	87.743	14.845	13.464
4866	181	28	23	P	S	34.307	2.326	3.498	-2.494	-2.223	-3.682	2.625	87.945	13.473	14.834
4867	181	28	23	P	S	37.311	-4.497	-5.226	4.629	4.941	4.783	-4.237	84.994	11.961	16.788
4868	181	28	23	P	S	38.213	5.011	-3.248	-4.812	-4.554	3.594	5.325	88.046	13.826	14.480
4869	181	28	23	P	S	42.103	5.011	3.248	-4.812	-4.554	-3.594	5.325	85.448	16.799	11.949
4870	181	28	23	P	S	42.520	-4.685	4.344	4.856	5.169	-3.960	-4.426	87.627	14.491	13.818
4871	181	28	23	P	S	43.663	0.353	-2.036	-0.633	-0.351	2.062	0.641	84.075	17.539	11.442
4872	181	28	23	P	S	46.102	-0.414	2.211	0.131	0.417	-2.206	-0.131	84.056	11.445	17.535
4873	181	28	23	P	S	47.480	2.326	-3.498	-2.494	-2.223	3.682	2.625	87.945	13.473	14.834
4874	181	28	23	P	S	51.487	2.390	3.329	-2.552	-2.281	-3.508	2.689	79.434	17.777	11.429
4875	181	28	23	P	S	51.787	-2.359	4.027	2.178	2.476	-3.859	-2.087	87.743	14.845	13.464
4876	181	28	23	P	S	58.055	-4.723	-2.385	4.902	5.216	2.172	-4.464	86.555	15.242	13.150
4877	181	28	23	P	S	60.000	5.011	1.083	-4.812	-4.554	-1.198	5.325	86.745	13.151	15.239
4878	181	28	23	P	S	61.945	-4.723	2.386	4.902	5.216	-2.173	-4.464	86.555	15.242	13.150
4879	181	28	23	P	S	68.213	-2.359	-4.027	2.178	2.476	3.859	-2.087	87.743	14.845	13.464
4880	181	28	23	P	S	68.513	2.390	-3.329	-2.552	-2.281	3.508	2.689	79.434	11.429	17.777
4881	181	28	23	P	S	72.520	2.326	3.498	-2.494	-2.223	-3.682	2.625	87.945	13.473	14.834
4882	181	28	23	P	S	73.898	-0.414	-2.211	0.131	0.417	2.206	-0.131	84.056	11.445	17.535
4883	181	28	23	P	S	76.337	0.353	2.036	-0.633	-0.351	-2.062	0.641	84.075	17.539	11.442
4884	181	28	23	P	S	77.480	-4.685	-4.345	4.856	5.169	3.960	-4.426	87.627	14.491	13.818
4885	181	28	23	P	S	77.897	5.011	-3.248	-4.812	-4.554	3.594	5.325	85.448	16.799	11.949
4886	181	28	23	P	S	81.787	5.011	3.248	-4.812	-4.554	-3.594	5.325	88.046	13.826	14.480
4887	181	28	23	P	S	82.689	-4.497	5.226	4.629	4.941	-4.783	-4.237	84.994	11.961	16.788
4888	181	28	23	P	S	85.694	2.326	-3.498	-2.494	-2.223	3.682	2.625	87.945	13.473	14.834
4889	181	28	23	P	S	90.000	-2.359	4.027	2.178	2.476	-3.859	-2.087	87.743	14.845	13.464
4890	181	28	23	P	S	94.307	2.326	3.498	-2.494	-2.223	-3.682	2.625	87.945	13.473	14.834
4891	181	28	23	P	S	103.663	0.353	-2.036	-0.633	-0.351	2.062	0.641	84.075	17.539	11.442
4892	181	28	23	P	S	106.102	-0.414	2.211	0.131	0.417	-2.206	-0.131	84.056	11.445	17.535
4893	181	28	23	P	S	107.480	2.326	-3.498	-2.494	-2.223	3.682	2.625	87.945	13.473	14.834
4894	181	28	23	P	S	111.487	2.390	3.329	-2.552	-2.281	-3.508	2.689	79.434	17.777	11.429
4895	181	28	23	P	S	111.787	-2.359	4.027	2.178	2.476	-3.859	-2.087	87.743	14.845	13.464
4896	181	28	23	P	S	115.693	-4.685	-4.345	4.856	5.169	3.960	-4.426	87.627	14.491	13.818

BL number	Atoms	γ-PC unit cells	WS_2 unit cells	γ-PC origin atom	WS_2 origin atom	Twist-angle (°)	γ-PC strain 1 (%)	γ-PC strain 2 (%)	γ-PC strain 3 (%)	WS_2 strain 1 (%)	WS_2 strain 2 (%)	WS_2 strain 3 (%)	γ (°)	|**a**|(Å)	|**b**|(Å)
4897	181	28	23	P	S	118.055	-4.723	-2.385	4.902	5.216	2.172	-4.464	86.555	15.242	13.150
4898	181	28	23	P	W	0.000	5.011	1.083	-4.812	-4.554	-1.198	5.325	86.745	13.151	15.239
4899	181	28	23	P	W	1.945	-4.723	2.386	4.902	5.216	-2.173	-4.464	86.555	15.242	13.150
4900	181	28	23	P	W	8.213	-2.359	-4.027	2.178	2.476	3.859	-2.087	87.743	14.845	13.464
4901	181	28	23	P	W	8.513	2.390	-3.329	-2.552	-2.281	3.508	2.689	79.434	11.429	17.777
4902	181	28	23	P	W	12.520	2.326	3.498	-2.494	-2.223	-3.682	2.625	87.945	13.473	14.834
4903	181	28	23	P	W	13.898	-0.414	-2.211	0.131	0.417	2.206	-0.131	84.056	11.445	17.535
4904	181	28	23	P	W	16.337	0.353	2.036	-0.633	-0.351	-2.062	0.641	84.075	17.539	11.442
4905	181	28	23	P	W	17.480	-4.685	-4.345	4.856	5.169	3.960	-4.426	87.627	14.491	13.818
4906	181	28	23	P	W	17.897	5.011	-3.248	-4.812	-4.554	3.594	5.325	85.448	16.799	11.949
4907	181	28	23	P	W	21.787	5.011	3.248	-4.812	-4.554	-3.594	5.325	88.046	13.826	14.480
4908	181	28	23	P	W	22.689	-4.497	5.226	4.629	4.941	-4.783	-4.237	84.994	11.961	16.788
4909	181	28	23	P	W	25.693	2.326	-3.498	-2.494	-2.223	3.682	2.625	87.945	13.473	14.834
4910	181	28	23	P	W	30.000	-2.359	-4.027	2.178	2.476	3.859	-2.087	87.743	14.845	13.464
4911	181	28	23	P	W	34.307	2.326	3.498	-2.494	-2.223	-3.682	2.625	87.945	13.473	14.834
4912	181	28	23	P	W	43.663	0.353	-2.036	-0.633	-0.351	2.062	0.641	84.075	17.539	11.442
4913	181	28	23	P	W	46.102	-0.414	2.211	0.131	0.417	-2.206	-0.131	84.056	11.445	17.535
4914	181	28	23	P	W	47.480	2.326	-3.498	-2.494	-2.223	3.682	2.625	87.945	13.473	14.834
4915	181	28	23	P	W	51.487	2.390	3.329	-2.552	-2.281	-3.508	2.689	79.434	17.777	11.429
4916	181	28	23	P	W	51.787	-2.359	4.027	2.178	2.476	-3.859	-2.087	87.743	14.845	13.464
4917	181	28	23	P	W	55.693	-4.685	-4.345	4.856	5.169	3.960	-4.426	87.627	14.491	13.818
4918	181	28	23	P	W	58.055	-4.723	-2.385	4.902	5.216	2.172	-4.464	86.555	15.242	13.150
4919	181	28	23	P	W	60.000	5.011	3.248	-4.812	-4.554	-3.594	5.325	88.046	13.826	14.480
4920	181	28	23	P	W	61.945	-4.723	2.386	4.902	5.216	-2.173	-4.464	86.555	15.242	13.150
4921	181	28	23	P	W	64.307	-4.685	4.344	4.856	5.169	-3.960	-4.426	87.627	14.491	13.818
4922	181	28	23	P	W	68.213	-2.359	-4.027	2.178	2.476	3.859	-2.087	87.743	14.845	13.464
4923	181	28	23	P	W	68.513	2.390	-3.329	-2.552	-2.281	3.508	2.689	79.434	11.429	17.777
4924	181	28	23	P	W	72.520	2.326	3.498	-2.494	-2.223	-3.682	2.625	87.945	13.473	14.834
4925	181	28	23	P	W	73.898	-0.414	-2.211	0.131	0.417	2.206	-0.131	84.056	11.445	17.535
4926	181	28	23	P	W	76.337	0.353	2.036	-0.633	-0.351	-2.062	0.641	84.075	17.539	11.442
4927	181	28	23	P	W	85.694	2.326	-3.498	-2.494	-2.223	3.682	2.625	87.945	13.473	14.834
4928	181	28	23	P	W	90.000	-2.359	4.027	2.178	2.476	-3.859	-2.087	87.743	14.845	13.464
4929	181	28	23	P	W	94.307	2.326	3.498	-2.494	-2.223	-3.682	2.625	87.945	13.473	14.834
4930	181	28	23	P	W	97.311	-4.497	-5.226	4.629	4.941	4.783	-4.237	84.994	11.961	16.788
4931	181	28	23	P	W	98.213	5.011	-3.248	-4.812	-4.554	3.594	5.325	88.046	13.826	14.480
4932	181	28	23	P	W	102.104	5.011	3.248	-4.812	-4.554	-3.594	5.325	85.448	16.799	11.949
4933	181	28	23	P	W	102.520	-4.685	4.344	4.856	5.169	-3.960	-4.426	87.627	14.491	13.818
4934	181	28	23	P	W	103.663	0.353	-2.036	-0.633	-0.351	2.062	0.641	84.075	17.539	11.442
4935	181	28	23	P	W	106.102	-0.414	2.211	0.131	0.417	-2.206	-0.131	84.056	11.445	17.535
4936	181	28	23	P	W	107.480	2.326	-3.498	-2.494	-2.223	3.682	2.625	87.945	13.473	14.834
4937	181	28	23	P	W	111.487	2.390	3.329	-2.552	-2.281	-3.508	2.689	79.434	17.777	11.429
4938	181	28	23	P	W	111.787	-2.359	4.027	2.178	2.476	-3.859	-2.087	87.743	14.845	13.464
4939	181	28	23	P	W	118.055	-4.723	-2.385	4.902	5.216	2.172	-4.464	86.555	15.242	13.150
4940	183	27	25	C	S	30.000	2.934	0.000	2.934	-2.772	0.000	-2.772	60.000	15.368	15.368
4941	183	27	25	C	S	90.000	2.934	0.000	2.934	-2.772	0.000	-2.772	60.000	15.368	15.368
4942	183	27	25	P	S	30.000	2.934	0.000	2.934	-2.772	0.000	-2.772	60.000	15.368	15.368
4943	183	27	25	P	S	90.000	2.934	0.000	2.934	-2.772	0.000	-2.772	60.000	15.368	15.368
4944	183	27	25	C	S	25.693	2.326	-3.800	3.550	-2.223	3.549	-3.315	81.275	13.473	15.359
4945	183	27	25	C	S	30.000	2.934	-3.962	2.934	-2.772	3.742	-2.772	81.247	15.368	13.465
4946	183	27	25	C	S	34.307	2.326	3.800	3.550	-2.223	-3.549	-3.315	81.275	13.473	15.359
4947	183	27	25	C	S	85.694	2.326	-3.800	3.550	-2.223	3.549	-3.315	81.275	13.473	15.359

BL number	Atoms	γ-PC unit cells	WS_2 unit cells	γ-PC origin atom	WS_2 origin atom	Twist-angle (°)	γ-PC strain 1 (%)	γ-PC strain 2 (%)	γ-PC strain 3 (%)	WS_2 strain 1 (%)	WS_2 strain 2 (%)	WS_2 strain 3 (%)	γ (°)	|**a**|(Å)	|**b**|(Å)
4948	183	27	25	C	S	87.126	1.029	-1.226	4.911	-1.008	1.116	-4.472	81.143	19.034	10.878
4949	183	27	25	C	S	90.000	2.934	-3.962	2.934	-2.772	3.742	-2.772	81.247	15.368	13.465
4950	183	27	25	C	S	94.307	2.326	3.800	3.550	-2.223	-3.549	-3.315	81.275	13.473	15.359
4951	183	27	25	C	W	25.693	2.326	-3.800	3.550	-2.223	3.549	-3.315	81.275	13.473	15.359
4952	183	27	25	C	W	27.126	1.029	-1.226	4.911	-1.008	1.116	-4.472	81.143	19.034	10.878
4953	183	27	25	C	W	30.000	2.934	-3.962	2.934	-2.772	3.742	-2.772	81.247	15.368	13.465
4954	183	27	25	C	W	34.307	2.326	3.800	3.550	-2.223	-3.549	-3.315	81.275	13.473	15.359
4955	183	27	25	C	W	85.694	2.326	-3.800	3.550	-2.223	3.549	-3.315	81.275	13.473	15.359
4956	183	27	25	C	W	90.000	2.934	-3.962	2.934	-2.772	3.742	-2.772	81.247	15.368	13.465
4957	183	27	25	C	W	94.307	2.326	3.800	3.550	-2.223	-3.549	-3.315	81.275	13.473	15.359
4958	183	27	25	P	S	25.693	2.326	-3.800	3.550	-2.223	3.549	-3.315	81.275	13.473	15.359
4959	183	27	25	P	S	30.000	2.934	3.962	2.934	-2.772	-3.742	-2.772	81.247	15.368	13.465
4960	183	27	25	P	S	87.126	1.029	-1.226	4.911	-1.008	1.116	-4.472	81.143	19.034	10.878
4961	183	27	25	P	S	90.000	2.934	-3.962	2.934	-2.772	3.742	-2.772	81.247	15.368	13.465
4962	183	27	25	P	S	94.307	2.326	3.800	3.550	-2.223	-3.549	-3.315	81.275	13.473	15.359
4963	183	27	25	P	W	27.126	1.029	-1.226	4.911	-1.008	1.116	-4.472	81.143	19.034	10.878
4964	183	27	25	P	W	30.000	2.934	-3.962	2.934	-2.772	3.742	-2.772	81.247	15.368	13.465
4965	183	27	25	P	W	34.307	2.326	3.800	3.550	-2.223	-3.549	-3.315	81.275	13.473	15.359
4966	183	27	25	P	W	85.694	2.326	-3.800	3.550	-2.223	3.549	-3.315	81.275	13.473	15.359
4967	183	27	25	P	W	90.000	2.934	3.962	2.934	-2.772	-3.742	-2.772	81.248	15.368	13.465
4968	183	30	21	C	S	4.307	-4.685	-1.641	-3.254	5.169	1.756	3.480	72.031	14.491	14.348
4969	183	30	21	C	S	4.715	-3.007	-0.933	-4.922	3.200	1.035	5.459	71.986	14.354	14.489
4970	183	30	21	C	S	6.587	-3.853	2.191	-4.096	4.175	-2.387	4.461	72.025	14.354	14.484
4971	183	30	21	C	S	53.413	-3.853	-2.191	-4.096	4.175	2.387	4.461	72.025	14.354	14.484
4972	183	30	21	C	S	55.285	-3.007	0.933	-4.922	3.200	-1.035	5.459	71.986	14.489	14.354
4973	183	30	21	C	S	55.693	-4.685	1.641	-3.254	5.169	-1.756	3.480	72.031	14.491	14.348
4974	183	30	21	C	S	64.307	-4.685	-1.641	-3.254	5.169	1.756	3.480	72.031	14.491	14.348
4975	183	30	21	C	S	64.715	-3.007	-0.933	-4.922	3.200	1.035	5.459	71.986	14.354	14.489
4976	183	30	21	C	S	66.587	-3.853	2.191	-4.096	4.175	-2.387	4.461	72.025	14.354	14.484
4977	183	30	21	C	S	113.413	-3.853	-2.191	-4.096	4.175	2.387	4.461	72.025	14.354	14.484
4978	183	30	21	C	S	115.285	-3.007	0.933	-4.922	3.200	-1.035	5.459	71.986	14.489	14.354
4979	183	30	21	C	S	115.693	-4.685	1.641	-3.254	5.169	-1.756	3.480	72.031	14.491	14.348
4980	183	30	21	C	W	4.307	-4.685	-1.641	-3.254	5.169	1.756	3.480	72.031	14.491	14.348
4981	183	30	21	C	W	4.715	-3.007	-0.933	-4.922	3.200	1.035	5.459	71.986	14.354	14.489
4982	183	30	21	C	W	6.587	-3.853	2.191	-4.096	4.175	-2.387	4.461	72.025	14.354	14.484
4983	183	30	21	C	W	53.413	-3.853	-2.191	-4.096	4.175	2.387	4.461	72.025	14.354	14.484
4984	183	30	21	C	W	55.285	-3.007	0.933	-4.922	3.200	-1.035	5.459	71.986	14.489	14.354
4985	183	30	21	C	W	55.693	-4.685	1.641	-3.254	5.169	-1.756	3.480	72.031	14.491	14.348
4986	183	30	21	C	W	64.307	-4.685	-1.641	-3.254	5.169	1.756	3.480	72.031	14.491	14.348
4987	183	30	21	C	W	64.715	-3.007	-0.933	-4.922	3.200	1.035	5.459	71.986	14.354	14.489
4988	183	30	21	C	W	66.587	-3.853	2.191	-4.096	4.175	-2.387	4.461	72.025	14.354	14.484
4989	183	30	21	C	W	113.413	-3.853	-2.191	-4.096	4.175	2.387	4.461	72.025	14.354	14.484
4990	183	30	21	C	W	115.285	-3.007	0.933	-4.922	3.200	-1.035	5.459	71.986	14.489	14.354
4991	183	30	21	C	W	115.693	-4.685	1.641	-3.254	5.169	-1.756	3.480	72.031	14.491	14.348
4992	183	30	21	P	S	4.307	-4.685	-1.641	-3.254	5.169	1.756	3.480	72.031	14.491	14.348
4993	183	30	21	P	S	4.715	-3.007	-0.933	-4.922	3.200	1.035	5.459	71.986	14.354	14.489
4994	183	30	21	P	S	6.587	-3.853	2.191	-4.096	4.175	-2.387	4.461	72.025	14.354	14.484
4995	183	30	21	P	S	53.413	-3.853	-2.191	-4.096	4.175	2.387	4.461	72.025	14.354	14.484
4996	183	30	21	P	S	55.285	-3.007	0.933	-4.922	3.200	-1.035	5.459	71.986	14.489	14.354
4997	183	30	21	P	S	55.693	-4.685	1.641	-3.254	5.169	-1.756	3.480	72.031	14.491	14.348
4998	183	30	21	P	S	64.307	-4.685	-1.641	-3.254	5.169	1.756	3.480	72.031	14.491	14.348

BL number	Atoms	γ-PC unit cells	WS_2 unit cells	γ-PC origin atom	WS_2 origin atom	Twist-angle (°)	γ-PC strain 1 (%)	γ-PC strain 2 (%)	γ-PC strain 3 (%)	WS_2 strain 1 (%)	WS_2 strain 2 (%)	WS_2 strain 3 (%)	γ (°)	$\lvert\mathbf{a}\rvert$(Å)	$\lvert\mathbf{b}\rvert$(Å)
4999	183	30	21	P	S	64.715	-3.007	-0.933	-4.922	3.200	1.035	5.459	71.986	14.354	14.489
5000	183	30	21	P	S	66.587	-3.853	2.191	-4.096	4.175	-2.387	4.461	72.025	14.354	14.484
5001	183	30	21	P	S	113.413	-3.853	-2.191	-4.096	4.175	2.387	4.461	72.025	14.354	14.484
5002	183	30	21	P	S	115.285	-3.007	0.933	-4.922	3.200	-1.035	5.459	71.986	14.489	14.354
5003	183	30	21	P	S	115.693	-4.685	1.641	-3.254	5.169	-1.756	3.480	72.031	14.491	14.348
5004	183	30	21	P	W	4.307	-4.685	-1.641	-3.254	5.169	1.756	3.480	72.031	14.491	14.348
5005	183	30	21	P	W	4.715	-3.007	-0.933	-4.922	3.200	1.035	5.459	71.986	14.354	14.489
5006	183	30	21	P	W	6.587	-3.853	2.191	-4.096	4.175	-2.387	4.461	72.025	14.354	14.484
5007	183	30	21	P	W	53.413	-3.853	-2.191	-4.096	4.175	2.387	4.461	72.025	14.354	14.484
5008	183	30	21	P	W	55.285	-3.007	0.933	-4.922	3.200	-1.035	5.459	71.986	14.489	14.354
5009	183	30	21	P	W	55.693	-4.685	1.641	-3.254	5.169	-1.756	3.480	72.031	14.491	14.348
5010	183	30	21	P	W	64.307	-4.685	-1.641	-3.254	5.169	1.756	3.480	72.031	14.491	14.348
5011	183	30	21	P	W	64.715	-3.007	-0.933	-4.922	3.200	1.035	5.459	71.986	14.354	14.489
5012	183	30	21	P	W	66.587	-3.853	2.191	-4.096	4.175	-2.387	4.461	72.025	14.354	14.484
5013	183	30	21	P	W	113.413	-3.853	-2.191	-4.096	4.175	2.387	4.461	72.025	14.354	14.484
5014	183	30	21	P	W	115.285	-3.007	0.933	-4.922	3.200	-1.035	5.459	71.986	14.489	14.354
5015	183	30	21	P	W	115.693	-4.685	1.641	-3.254	5.169	-1.756	3.480	72.031	14.491	14.348
5016	184	28	24	C	S	10.893	4.020	0.000	-1.982	-3.721	0.000	2.064	90.000	15.815	12.906
5017	184	28	24	C	S	30.000	-2.359	0.000	4.447	2.476	0.000	-4.084	90.000	9.718	21.007
5018	184	28	24	C	S	49.107	-1.983	0.000	4.020	2.064	0.000	-3.721	90.000	12.906	15.815
5019	184	28	24	C	S	70.893	4.020	0.000	-1.982	-3.721	0.000	2.064	90.000	15.815	12.906
5020	184	28	24	C	S	90.000	-2.359	0.000	4.447	2.476	0.000	-4.084	90.000	9.718	21.007
5021	184	28	24	C	S	109.107	-1.983	0.000	4.020	2.064	0.000	-3.721	90.000	12.906	15.815
5022	184	28	24	C	W	10.893	4.020	0.000	-1.982	-3.721	0.000	2.064	90.000	15.815	12.906
5023	184	28	24	C	W	30.000	-2.359	0.000	4.447	2.476	0.000	-4.084	90.000	9.718	21.007
5024	184	28	24	C	W	49.107	-1.983	0.000	4.020	2.064	0.000	-3.721	90.000	12.906	15.815
5025	184	28	24	C	W	70.893	4.020	0.000	-1.982	-3.721	0.000	2.064	90.000	15.815	12.906
5026	184	28	24	C	W	90.000	-2.359	0.000	4.447	2.476	0.000	-4.084	90.000	9.718	21.007
5027	184	28	24	C	W	109.107	-1.983	0.000	4.020	2.064	0.000	-3.721	90.000	12.906	15.815
5028	184	28	24	P	S	10.893	4.020	0.000	-1.982	-3.721	0.000	2.064	90.000	15.815	12.906
5029	184	28	24	P	S	49.107	-1.983	0.000	4.020	2.064	0.000	-3.721	90.000	12.906	15.815
5030	184	28	24	P	S	70.893	4.020	0.000	-1.982	-3.721	0.000	2.064	90.000	15.815	12.906
5031	184	28	24	P	S	109.107	-1.983	0.000	4.020	2.064	0.000	-3.721	90.000	12.906	15.815
5032	184	28	24	P	W	10.893	4.020	0.000	-1.982	-3.721	0.000	2.064	90.000	15.815	12.906
5033	184	28	24	P	W	49.107	-1.983	0.000	4.020	2.064	0.000	-3.721	90.000	12.906	15.815
5034	184	28	24	P	W	70.893	4.020	0.000	-1.982	-3.721	0.000	2.064	90.000	15.815	12.906
5035	184	28	24	P	W	109.107	-1.983	0.000	4.020	2.064	0.000	-3.721	90.000	12.906	15.815
5036	184	28	24	C	S	0.000	5.011	-2.165	-2.848	-4.554	2.296	3.020	73.842	10.878	19.545
5037	184	28	24	C	S	9.183	3.582	-2.944	-1.590	-3.342	3.041	1.642	73.809	18.586	11.432
5038	184	28	24	C	S	13.898	-0.414	-2.843	2.311	0.417	2.718	-2.209	74.963	19.824	10.653
5039	184	28	24	C	S	16.102	2.853	1.060	-0.922	-2.699	-1.080	0.940	75.021	10.655	19.818
5040	184	28	24	C	S	17.696	1.029	3.885	0.832	-1.008	-3.821	-0.818	75.042	11.440	18.450
5041	184	28	24	C	S	21.052	1.339	-2.090	0.524	-1.304	2.068	-0.519	88.809	16.211	12.582
5042	184	28	24	C	S	23.413	0.481	2.072	1.383	-0.477	-2.016	-1.346	88.829	12.584	16.208
5043	184	28	24	C	S	36.587	0.481	-2.072	1.383	-0.477	2.016	-1.346	88.829	12.584	16.208
5044	184	28	24	C	S	38.948	1.339	2.090	0.524	-1.304	-2.068	-0.519	88.809	16.211	12.582
5045	184	28	24	C	S	42.304	1.029	-3.885	0.832	-1.008	3.821	-0.818	75.042	18.450	11.440
5046	184	28	24	C	S	43.898	2.853	-1.060	-0.922	-2.699	1.080	0.940	75.020	18.455	11.442
5047	184	28	24	C	S	46.102	-0.414	2.843	2.311	0.417	-2.718	-2.209	74.963	19.824	10.653
5048	184	28	24	C	S	50.817	3.582	2.944	-1.590	-3.342	-3.041	1.642	73.809	18.586	11.432
5049	184	28	24	C	S	60.000	5.011	2.165	-2.848	-4.554	-2.296	3.020	73.842	10.878	19.545

BL number	Atoms	γ-PC unit cells	WS_2 unit cells	γ-PC origin atom	WS_2 origin atom	Twist-angle (°)	γ-PC strain 1 (%)	γ-PC strain 2 (%)	γ-PC strain 3 (%)	WS_2 strain 1 (%)	WS_2 strain 2 (%)	WS_2 strain 3 (%)	γ (°)	|**a**|(Å)	|**b**|(Å)
5050	184	28	24	C	S	69.183	3.582	-2.944	-1.590	-3.342	3.041	1.642	73.809	18.586	11.432
5051	184	28	24	C	S	73.898	-0.414	-2.843	2.311	0.417	2.718	-2.209	74.963	11.445	18.452
5052	184	28	24	C	S	76.102	2.853	1.060	-0.922	-2.699	-1.080	0.940	75.020	18.455	11.442
5053	184	28	24	C	S	77.696	1.029	3.885	0.832	-1.008	-3.821	-0.818	75.042	11.440	18.450
5054	184	28	24	C	S	81.052	1.339	-2.090	0.524	-1.304	2.068	-0.519	88.809	16.211	12.582
5055	184	28	24	C	S	83.413	0.481	2.072	1.383	-0.477	-2.016	-1.346	88.829	12.584	16.208
5056	184	28	24	C	S	96.587	0.481	-2.072	1.383	-0.477	2.016	-1.346	88.829	12.584	16.208
5057	184	28	24	C	S	98.948	1.339	2.090	0.524	-1.304	-2.068	-0.519	88.809	16.211	12.582
5058	184	28	24	C	S	102.304	1.029	-3.885	0.832	-1.008	3.821	-0.818	75.042	18.450	11.440
5059	184	28	24	C	S	103.898	2.853	-1.060	-0.922	-2.699	1.080	0.940	75.020	10.655	19.818
5060	184	28	24	C	S	106.102	-0.414	-5.371	2.311	0.417	5.133	-2.209	73.652	11.445	18.571
5061	184	28	24	C	S	110.817	3.582	2.944	-1.590	-3.342	-3.041	1.642	73.809	18.586	11.432
5062	184	28	24	C	W	0.000	5.011	-2.165	-2.848	-4.554	2.296	3.020	73.842	10.878	19.545
5063	184	28	24	C	W	9.183	3.582	-2.944	-1.590	-3.342	3.041	1.642	73.809	18.586	11.432
5064	184	28	24	C	W	13.898	-0.414	-2.843	2.311	0.417	2.718	-2.209	74.963	19.824	10.653
5065	184	28	24	C	W	16.102	2.853	1.060	-0.922	-2.699	-1.080	0.940	75.021	10.655	19.818
5066	184	28	24	C	W	17.696	1.029	3.885	0.832	-1.008	-3.821	-0.818	75.042	11.440	18.450
5067	184	28	24	C	W	21.052	1.339	-2.090	0.524	-1.304	2.068	-0.519	88.809	16.211	12.582
5068	184	28	24	C	W	23.413	0.481	2.072	1.383	-0.477	-2.016	-1.346	88.829	12.584	16.208
5069	184	28	24	C	W	36.587	0.481	-2.072	1.383	-0.477	2.016	-1.346	88.829	12.584	16.208
5070	184	28	24	C	W	38.948	1.339	2.090	0.524	-1.304	-2.068	-0.519	88.809	16.211	12.582
5071	184	28	24	C	W	42.304	1.029	-3.885	0.832	-1.008	3.821	-0.818	75.042	18.450	11.440
5072	184	28	24	C	W	43.898	2.853	-1.060	-0.922	-2.699	1.080	0.940	75.020	18.455	11.442
5073	184	28	24	C	W	46.102	-0.414	2.843	2.311	0.417	-2.718	-2.209	74.963	19.824	10.653
5074	184	28	24	C	W	50.817	3.582	2.944	-1.590	-3.342	-3.041	1.642	73.809	18.586	11.432
5075	184	28	24	C	W	60.000	5.011	2.165	-2.848	-4.554	-2.296	3.020	73.842	10.878	19.545
5076	184	28	24	C	W	69.183	3.582	-2.944	-1.590	-3.342	3.041	1.642	73.809	18.586	11.432
5077	184	28	24	C	W	73.898	-0.414	-2.843	2.311	0.417	2.718	-2.209	74.963	11.445	18.452
5078	184	28	24	C	W	76.102	2.853	1.060	-0.922	-2.699	-1.080	0.940	75.020	18.455	11.442
5079	184	28	24	C	W	77.696	1.029	3.885	0.832	-1.008	-3.821	-0.818	75.042	11.440	18.450
5080	184	28	24	C	W	81.052	1.339	-2.090	0.524	-1.304	2.068	-0.519	88.809	16.211	12.582
5081	184	28	24	C	W	83.413	0.481	2.072	1.383	-0.477	-2.016	-1.346	88.829	12.584	16.208
5082	184	28	24	C	W	96.587	0.481	-2.072	1.383	-0.477	2.016	-1.346	88.829	12.584	16.208
5083	184	28	24	C	W	98.948	1.339	2.090	0.524	-1.304	-2.068	-0.519	88.809	16.211	12.582
5084	184	28	24	C	W	102.304	1.029	-3.885	0.832	-1.008	3.821	-0.818	75.042	18.450	11.440
5085	184	28	24	C	W	103.898	2.853	-1.060	-0.922	-2.699	1.080	0.940	75.020	10.655	19.818
5086	184	28	24	C	W	106.102	-0.414	-5.371	2.311	0.417	5.133	-2.209	73.652	11.445	18.571
5087	184	28	24	C	W	110.817	3.582	2.944	-1.590	-3.342	-3.041	1.642	73.809	18.586	11.432
5088	184	28	24	P	S	0.000	5.011	-2.165	-2.848	-4.554	2.296	3.020	73.842	10.878	19.545
5089	184	28	24	P	S	9.183	3.582	-2.944	-1.590	-3.342	3.041	1.642	73.809	18.586	11.432
5090	184	28	24	P	S	13.898	-0.414	-2.843	2.311	0.417	2.718	-2.209	74.963	11.445	18.452
5091	184	28	24	P	S	21.052	1.339	-2.090	0.524	-1.304	2.068	-0.519	88.809	16.211	12.582
5092	184	28	24	P	S	23.413	0.481	2.072	1.383	-0.477	-2.016	-1.346	88.829	12.584	16.208
5093	184	28	24	P	S	36.587	0.481	-2.072	1.383	-0.477	2.016	-1.346	88.829	12.584	16.208
5094	184	28	24	P	S	38.948	1.339	2.090	0.524	-1.304	-2.068	-0.519	88.809	16.211	12.582
5095	184	28	24	P	S	43.898	2.853	-1.060	-0.922	-2.699	1.080	0.940	75.021	10.655	19.818
5096	184	28	24	P	S	46.102	-0.414	2.843	2.311	0.417	-2.718	-2.209	74.963	19.824	10.653
5097	184	28	24	P	S	50.817	3.582	2.944	-1.590	-3.342	-3.041	1.642	73.809	18.586	11.432
5098	184	28	24	P	S	60.000	5.011	2.165	-2.848	-4.554	-2.296	3.020	73.842	10.878	19.545
5099	184	28	24	P	S	69.183	3.582	-2.944	-1.590	-3.342	3.041	1.642	73.809	18.586	11.432
5100	184	28	24	P	S	73.898	-0.414	5.371	2.311	0.417	-5.133	-2.209	73.651	11.445	18.571

BL number	Atoms	γ-PC unit cells	WS_2 unit cells	γ-PC origin atom	WS_2 origin atom	Twist-angle (°)	γ-PC strain 1 (%)	γ-PC strain 2 (%)	γ-PC strain 3 (%)	WS_2 strain 1 (%)	WS_2 strain 2 (%)	WS_2 strain 3 (%)	γ (°)	$\lvert\mathbf{a}\rvert$(Å)	$\lvert\mathbf{b}\rvert$(Å)
5101	184	28	24	P	S	76.102	2.853	1.060	-0.922	-2.699	-1.080	0.940	75.020	10.655	19.818
5102	184	28	24	P	S	81.052	1.339	-2.090	0.524	-1.304	2.068	-0.519	88.809	16.211	12.582
5103	184	28	24	P	S	83.413	0.481	2.072	1.383	-0.477	-2.016	-1.346	88.829	12.584	16.208
5104	184	28	24	P	S	96.587	0.481	-2.072	1.383	-0.477	2.016	-1.346	88.829	12.584	16.208
5105	184	28	24	P	S	98.948	1.339	2.090	0.524	-1.304	-2.068	-0.519	88.809	16.211	12.582
5106	184	28	24	P	S	106.102	-0.414	-5.371	2.311	0.417	5.133	-2.209	73.652	11.445	18.571
5107	184	28	24	P	S	110.817	3.582	2.944	-1.590	-3.342	-3.041	1.642	73.809	18.586	11.432
5108	184	28	24	P	W	0.000	5.011	-2.165	-2.848	-4.554	2.296	3.020	73.842	10.878	19.545
5109	184	28	24	P	W	9.183	3.582	-2.944	-1.590	-3.342	3.041	1.642	73.809	18.586	11.432
5110	184	28	24	P	W	13.898	-0.414	-2.843	2.311	0.417	2.718	-2.209	74.963	19.824	10.653
5111	184	28	24	P	W	16.102	2.853	1.060	-0.922	-2.699	-1.080	0.940	75.021	10.655	19.818
5112	184	28	24	P	W	21.052	1.339	-2.090	0.524	-1.304	2.068	-0.519	88.809	16.211	12.582
5113	184	28	24	P	W	23.413	0.481	2.072	1.383	-0.477	-2.016	-1.346	88.829	12.584	16.208
5114	184	28	24	P	W	36.587	0.481	-2.072	1.383	-0.477	2.016	-1.346	88.829	12.584	16.208
5115	184	28	24	P	W	38.948	1.339	2.090	0.524	-1.304	-2.068	-0.519	88.809	16.211	12.582
5116	184	28	24	P	W	46.102	-0.414	2.843	2.311	0.417	-2.718	-2.209	74.963	11.445	18.452
5117	184	28	24	P	W	50.817	3.582	2.944	-1.590	-3.342	-3.041	1.642	73.809	18.586	11.432
5118	184	28	24	P	W	60.000	5.011	2.165	-2.848	-4.554	-2.296	3.020	73.842	10.878	19.545
5119	184	28	24	P	W	69.183	3.582	-2.944	-1.590	-3.342	3.041	1.642	73.809	18.586	11.432
5120	184	28	24	P	W	73.898	-0.414	-2.843	2.311	0.417	2.718	-2.209	74.963	11.445	18.452
5121	184	28	24	P	W	81.052	1.339	-2.090	0.524	-1.304	2.068	-0.519	88.809	16.211	12.582
5122	184	28	24	P	W	83.413	0.481	2.072	1.383	-0.477	-2.016	-1.346	88.829	12.584	16.208
5123	184	28	24	P	W	96.587	0.481	-2.072	1.383	-0.477	2.016	-1.346	88.829	12.584	16.208
5124	184	28	24	P	W	98.948	1.339	2.090	0.524	-1.304	-2.068	-0.519	88.809	16.211	12.582
5125	184	28	24	P	W	103.898	2.853	-1.060	-0.922	-2.699	1.080	0.940	75.020	10.655	19.818
5126	184	28	24	P	W	106.102	-0.414	-5.371	2.311	0.417	5.133	-2.209	73.652	11.445	18.571
5127	184	28	24	P	W	110.817	3.582	2.944	-1.590	-3.342	-3.041	1.642	73.809	18.586	11.432
5128	185	29	23	C	S	19.842	-4.723	-4.336	3.009	5.216	4.090	-2.838	82.952	15.242	13.452
5129	185	29	23	C	S	40.158	-4.723	4.336	3.009	5.216	-4.090	-2.838	82.952	15.242	13.452
5130	185	29	23	C	S	79.842	-4.723	-4.336	3.009	5.216	4.090	-2.838	82.952	15.242	13.452
5131	185	29	23	C	S	100.158	-4.723	4.336	3.009	5.216	-4.090	-2.838	82.952	15.242	13.452
5132	185	29	23	C	W	19.842	-4.723	-4.336	3.009	5.216	4.090	-2.838	82.952	15.242	13.452
5133	185	29	23	C	W	40.158	-4.723	4.336	3.009	5.216	-4.090	-2.838	82.952	15.242	13.452
5134	185	29	23	C	W	79.842	-4.723	-4.336	3.009	5.216	4.090	-2.838	82.952	15.242	13.452
5135	185	29	23	C	W	100.158	-4.723	4.336	3.009	5.216	-4.090	-2.838	82.952	15.242	13.452
5136	185	29	23	P	S	19.842	-4.723	-4.336	3.009	5.216	4.090	-2.838	82.952	15.242	13.452
5137	185	29	23	P	S	40.158	-4.723	4.336	3.009	5.216	-4.090	-2.838	82.952	15.242	13.452
5138	185	29	23	P	S	79.842	-4.723	-4.336	3.009	5.216	4.090	-2.838	82.952	15.242	13.452
5139	185	29	23	P	S	100.158	-4.723	4.336	3.009	5.216	-4.090	-2.838	82.952	15.242	13.452
5140	185	29	23	P	W	19.842	-4.723	-4.336	3.009	5.216	4.090	-2.838	82.952	15.242	13.452
5141	185	29	23	P	W	40.158	-4.723	4.336	3.009	5.216	-4.090	-2.838	82.952	15.242	13.452
5142	185	29	23	P	W	79.842	-4.723	-4.336	3.009	5.216	4.090	-2.838	82.952	15.242	13.452
5143	185	29	23	P	W	100.158	-4.723	4.336	3.009	5.216	-4.090	-2.838	82.952	15.242	13.452
5144	186	27	26	C	S	9.183	3.582	-3.861	4.386	-3.342	3.550	-4.033	78.318	15.816	13.475
5145	186	27	26	C	S	46.827	5.011	-3.368	2.974	-4.554	3.179	-2.807	75.922	13.151	16.362
5146	186	27	26	C	S	50.570	5.011	3.368	2.974	-4.554	-3.179	-2.807	76.030	16.375	13.136
5147	186	27	26	C	S	50.817	3.582	3.861	4.386	-3.342	-3.550	-4.033	78.318	13.475	15.816
5148	186	27	26	C	S	69.183	3.582	-3.861	4.386	-3.342	3.550	-4.033	78.318	15.816	13.475
5149	186	27	26	C	S	69.430	5.011	-3.368	2.974	-4.554	3.179	-2.807	76.030	13.136	16.375
5150	186	27	26	C	S	73.174	5.011	3.368	2.974	-4.554	-3.179	-2.807	75.922	13.151	16.362
5151	186	27	26	C	S	110.817	3.582	3.861	4.386	-3.342	-3.550	-4.033	78.318	13.475	15.816

BL number	Atoms	γ-PC unit cells	WS_2 unit cells	γ-PC origin atom	WS_2 origin atom	Twist-angle (°)	γ-PC strain 1 (%)	γ-PC strain 2 (%)	γ-PC strain 3 (%)	WS_2 strain 1 (%)	WS_2 strain 2 (%)	WS_2 strain 3 (%)	γ (°)	|**a**|(Å)	|**b**|(Å)
5152	186	27	26	C	W	9.183	3.582	-3.861	4.386	-3.342	3.550	-4.033	78.318	15.816	13.475
5153	186	27	26	C	W	9.430	5.011	-3.368	2.974	-4.554	3.179	-2.807	76.030	13.136	16.375
5154	186	27	26	C	W	13.174	5.011	3.368	2.974	-4.554	-3.179	-2.807	75.922	13.151	16.362
5155	186	27	26	C	W	50.817	3.582	3.861	4.386	-3.342	-3.550	-4.033	78.318	13.475	15.816
5156	186	27	26	C	W	69.183	3.582	-3.861	4.386	-3.342	3.550	-4.033	78.318	15.816	13.475
5157	186	27	26	C	W	106.827	5.011	-3.368	2.974	-4.554	3.179	-2.807	75.922	13.151	16.362
5158	186	27	26	C	W	110.570	5.011	3.368	2.974	-4.554	-3.179	-2.807	76.030	16.375	13.136
5159	186	27	26	C	W	110.817	3.582	3.861	4.386	-3.342	-3.550	-4.033	78.318	13.475	15.816
5160	186	27	26	P	S	46.827	5.011	-3.368	2.974	-4.554	3.179	-2.807	75.922	13.151	16.362
5161	186	27	26	P	S	50.570	5.011	3.368	2.974	-4.554	-3.179	-2.807	76.030	16.375	13.136
5162	186	27	26	P	S	50.817	3.582	3.861	4.386	-3.342	-3.550	-4.033	78.318	13.475	15.816
5163	186	27	26	P	S	69.183	3.582	-3.861	4.386	-3.342	3.550	-4.033	78.318	15.816	13.475
5164	186	27	26	P	S	69.430	5.011	-3.368	2.974	-4.554	3.179	-2.807	76.030	13.136	16.375
5165	186	27	26	P	S	73.174	5.011	3.368	2.974	-4.554	-3.179	-2.807	75.922	13.151	16.362
5166	186	27	26	P	W	9.183	3.582	-3.861	4.386	-3.342	3.550	-4.033	78.318	15.816	13.475
5167	186	27	26	P	W	9.430	5.011	-3.368	2.974	-4.554	3.179	-2.807	76.030	13.136	16.375
5168	186	27	26	P	W	13.174	5.011	3.368	2.974	-4.554	-3.179	-2.807	75.922	13.151	16.362
5169	186	27	26	P	W	106.827	5.011	-3.368	2.974	-4.554	3.179	-2.807	75.922	13.151	16.362
5170	186	27	26	P	W	110.570	5.011	3.368	2.974	-4.554	-3.179	-2.807	76.030	16.375	13.136
5171	186	27	26	P	W	110.817	3.582	3.861	4.386	-3.342	-3.550	-4.033	78.318	13.475	15.816
5172	186	30	22	C	S	6.178	-2.145	-1.547	-3.626	2.241	1.668	3.910	71.689	14.845	14.352
5173	186	30	22	C	S	6.587	-3.853	-0.877	-1.910	4.175	0.911	1.986	71.706	14.354	14.842
5174	186	30	22	C	S	19.107	-1.485	2.438	-4.257	1.530	-2.664	4.653	71.686	14.708	14.488
5175	186	30	22	C	S	40.893	-1.485	-2.438	-4.257	1.530	2.664	4.653	71.686	14.708	14.488
5176	186	30	22	C	S	42.520	-4.685	0.290	-1.027	5.169	-0.296	1.049	71.702	14.491	14.706
5177	186	30	22	C	S	44.392	-2.145	3.430	-3.626	2.241	-3.698	3.910	71.725	14.484	14.706
5178	186	30	22	C	S	51.787	-2.359	-1.879	-3.418	2.476	2.017	3.669	71.698	14.845	14.351
5179	186	30	22	C	S	66.587	-3.853	-0.877	-1.910	4.175	0.911	1.986	71.705	14.354	14.842
5180	186	30	22	C	S	75.609	-2.145	-3.430	-3.626	2.241	3.698	3.910	71.725	14.706	14.484
5181	186	30	22	C	S	77.480	-4.685	-0.290	-1.027	5.169	0.296	1.049	71.702	14.491	14.706
5182	186	30	22	C	S	79.107	-1.485	2.438	-4.257	1.530	-2.664	4.653	71.686	14.708	14.488
5183	186	30	22	C	S	100.893	-1.485	-2.438	-4.257	1.530	2.664	4.653	71.686	14.708	14.488
5184	186	30	22	C	S	104.392	-2.145	3.430	-3.626	2.241	-3.698	3.910	71.725	14.484	14.706
5185	186	30	22	C	S	111.787	-2.359	-1.879	-3.418	2.476	2.017	3.669	71.698	14.845	14.351
5186	186	30	22	C	S	113.413	-3.853	0.877	-1.910	4.174	-0.911	1.986	71.705	14.354	14.842
5187	186	30	22	C	W	6.587	-3.853	-0.877	-1.910	4.175	0.911	1.986	71.706	14.354	14.842
5188	186	30	22	C	W	15.608	-2.145	-3.430	-3.626	2.241	3.698	3.910	71.725	14.706	14.484
5189	186	30	22	C	W	17.480	-4.685	-0.290	-1.027	5.169	0.296	1.049	71.702	14.491	14.706
5190	186	30	22	C	W	51.787	-2.359	-1.879	-3.418	2.476	2.017	3.669	71.698	14.845	14.351
5191	186	30	22	C	W	53.413	-3.853	0.877	-1.910	4.174	-0.911	1.986	71.706	14.354	14.842
5192	186	30	22	C	W	66.178	-2.145	-1.547	-3.626	2.241	1.668	3.910	71.689	14.845	14.352
5193	186	30	22	C	W	66.587	-3.853	-0.877	-1.910	4.175	0.911	1.986	71.705	14.354	14.842
5194	186	30	22	C	W	102.520	-4.685	0.290	-1.027	5.169	-0.296	1.049	71.702	14.491	14.706
5195	186	30	22	C	W	111.787	-2.359	-1.879	-3.418	2.476	2.017	3.669	71.698	14.845	14.351
5196	186	30	22	P	S	6.587	-3.853	-0.877	-1.910	4.175	0.911	1.986	71.706	14.354	14.842
5197	186	30	22	P	S	19.107	-1.485	2.438	-4.257	1.530	-2.664	4.653	71.686	14.708	14.488
5198	186	30	22	P	S	40.893	-1.485	-2.438	-4.257	1.530	2.664	4.653	71.686	14.708	14.488
5199	186	30	22	P	S	42.520	-4.685	0.290	-1.027	5.169	-0.296	1.049	71.702	14.491	14.706
5200	186	30	22	P	S	44.392	-2.145	3.430	-3.626	2.241	-3.698	3.910	71.725	14.484	14.706
5201	186	30	22	P	S	51.787	-2.359	-1.879	-3.418	2.476	2.017	3.669	71.698	14.845	14.351
5202	186	30	22	P	S	53.413	-3.853	0.877	-1.910	4.174	-0.911	1.986	71.706	14.354	14.842

BL number	Atoms	γ-PC unit cells	WS_2 unit cells	γ-PC origin atom	WS_2 origin atom	Twist-angle (°)	γ-PC strain 1 (%)	γ-PC strain 2 (%)	γ-PC strain 3 (%)	WS_2 strain 1 (%)	WS_2 strain 2 (%)	WS_2 strain 3 (%)	γ (°)	$\lvert\mathbf{a}\rvert$(Å)	$\lvert\mathbf{b}\rvert$(Å)
5203	186	30	22	P	S	66.178	-2.145	-1.547	-3.626	2.241	1.668	3.910	71.689	14.845	14.352
5204	186	30	22	P	S	66.587	-3.853	-0.877	-1.910	4.175	0.911	1.986	71.705	14.354	14.842
5205	186	30	22	P	S	75.609	-2.145	-3.430	-3.626	2.241	3.698	3.910	71.725	14.706	14.484
5206	186	30	22	P	S	77.480	-4.685	-0.290	-1.027	5.169	0.296	1.049	71.702	14.491	14.706
5207	186	30	22	P	S	79.107	-1.485	2.438	-4.257	1.530	-2.664	4.653	71.686	14.708	14.488
5208	186	30	22	P	S	100.893	-1.485	-2.438	-4.257	1.530	2.664	4.653	71.686	14.708	14.488
5209	186	30	22	P	S	104.392	-2.145	3.430	-3.626	2.241	-3.698	3.910	71.725	14.484	14.706
5210	186	30	22	P	S	111.787	-2.359	-1.879	-3.418	2.476	2.017	3.669	71.698	14.845	14.351
5211	186	30	22	P	W	6.178	-2.145	-1.547	-3.626	2.241	1.668	3.910	71.689	14.845	14.352
5212	186	30	22	P	W	6.587	-3.853	-0.877	-1.910	4.175	0.911	1.986	71.706	14.354	14.842
5213	186	30	22	P	W	15.608	-2.145	-3.430	-3.626	2.241	3.698	3.910	71.725	14.706	14.484
5214	186	30	22	P	W	17.480	-4.685	-0.290	-1.027	5.169	0.296	1.049	71.702	14.491	14.706
5215	186	30	22	P	W	51.787	-2.359	-1.879	-3.418	2.476	2.017	3.669	71.698	14.845	14.351
5216	186	30	22	P	W	66.587	-3.853	-0.877	-1.910	4.175	0.911	1.986	71.705	14.354	14.842
5217	186	30	22	P	W	102.520	-4.685	0.290	-1.027	5.169	-0.296	1.049	71.702	14.491	14.706
5218	186	30	22	P	W	111.787	-2.359	-1.879	-3.418	2.476	2.017	3.669	71.698	14.845	14.351
5219	186	30	22	P	W	113.413	-3.853	0.877	-1.910	4.174	-0.911	1.986	71.705	14.354	14.842
5220	187	28	25	C	S	19.107	1.980	0.000	1.980	-1.905	0.000	-1.905	60.000	15.505	15.504
5221	187	28	25	C	S	79.107	1.980	0.000	1.980	-1.905	0.000	-1.905	60.000	15.505	15.505
5222	187	28	25	C	W	19.107	1.980	0.000	1.980	-1.905	0.000	-1.905	60.000	15.505	15.505
5223	187	28	25	C	W	40.893	1.980	0.000	1.980	-1.905	0.000	-1.905	60.000	15.504	15.505
5224	187	28	25	C	W	79.107	1.980	0.000	1.980	-1.905	0.000	-1.905	60.000	15.505	15.505
5225	187	28	25	P	S	19.107	1.980	0.000	1.980	-1.905	0.000	-1.905	60.000	15.505	15.505
5226	187	28	25	P	S	100.893	1.980	0.000	1.980	-1.905	0.000	-1.905	60.000	15.504	15.505
5227	187	28	25	P	W	19.107	1.980	0.000	1.980	-1.905	0.000	-1.905	60.000	15.505	15.504
5228	187	28	25	P	W	40.893	1.980	0.000	1.980	-1.905	0.000	-1.905	60.000	15.504	15.505
5229	187	28	25	P	W	79.107	1.980	0.000	1.980	-1.905	0.000	-1.905	60.000	15.505	15.505
5230	187	28	25	C	S	8.948	-0.599	-4.304	4.694	0.606	3.935	-4.291	85.411	15.901	13.143
5231	187	28	25	C	S	9.430	5.011	-3.248	-0.883	-4.554	3.306	0.899	83.149	18.353	11.434
5232	187	28	25	C	S	13.174	5.011	3.248	-0.883	-4.554	-3.306	0.899	85.628	13.151	15.889
5233	187	28	25	C	S	13.898	-0.414	4.739	4.490	0.417	-4.348	-4.120	85.560	15.885	13.152
5234	187	28	25	C	S	18.613	3.582	4.935	0.427	-3.342	-4.894	-0.423	78.356	18.586	11.439
5235	187	28	25	C	S	41.387	3.582	-4.935	0.427	-3.342	4.894	-0.423	78.356	18.586	11.439
5236	187	28	25	C	S	46.102	-0.414	-4.739	4.490	0.417	4.348	-4.120	85.560	13.152	15.885
5237	187	28	25	C	S	46.827	5.011	-3.248	-0.883	-4.554	3.306	0.899	85.628	13.151	15.889
5238	187	28	25	C	S	50.570	5.011	3.248	-0.883	-4.554	-3.306	0.899	83.149	18.353	11.434
5239	187	28	25	C	S	51.052	-0.599	4.304	4.694	0.606	-3.935	-4.291	85.411	15.901	13.143
5240	187	28	25	C	S	68.948	-0.599	-4.304	4.694	0.606	3.935	-4.291	85.411	15.901	13.143
5241	187	28	25	C	S	69.430	5.011	-3.248	-0.883	-4.554	3.306	0.899	83.149	18.353	11.434
5242	187	28	25	C	S	73.174	5.011	3.248	-0.883	-4.554	-3.306	0.899	85.628	13.151	15.889
5243	187	28	25	C	S	73.898	-0.414	-3.475	4.490	0.417	3.189	-4.120	78.549	11.445	18.569
5244	187	28	25	C	S	78.613	3.582	4.935	0.427	-3.342	-4.894	-0.423	78.356	18.586	11.439
5245	187	28	25	C	S	101.387	3.582	-4.935	0.427	-3.342	4.894	-0.423	78.356	18.586	11.439
5246	187	28	25	C	S	106.102	-0.414	-4.739	4.490	0.417	4.348	-4.120	82.934	11.445	18.339
5247	187	28	25	C	S	106.827	5.011	-3.248	-0.883	-4.554	3.306	0.899	85.628	13.151	15.889
5248	187	28	25	C	S	110.570	5.011	3.248	-0.883	-4.554	-3.306	0.899	83.149	18.353	11.434
5249	187	28	25	C	S	111.052	-0.599	4.304	4.694	0.606	-3.935	-4.291	85.411	15.901	13.143
5250	187	28	25	C	W	8.948	-0.599	-4.304	4.694	0.606	3.935	-4.291	85.411	15.901	13.143
5251	187	28	25	C	W	9.430	5.011	-3.248	-0.883	-4.554	3.306	0.899	83.149	18.353	11.434
5252	187	28	25	C	W	13.174	5.011	3.248	-0.883	-4.554	-3.306	0.899	85.628	13.151	15.889
5253	187	28	25	C	W	13.898	-0.414	4.739	4.490	0.417	-4.348	-4.120	85.560	15.885	13.152

BL number	Atoms	γ-PC unit cells	WS_2 unit cells	γ-PC origin atom	WS_2 origin atom	Twist-angle (°)	γ-PC strain 1 (%)	γ-PC strain 2 (%)	γ-PC strain 3 (%)	WS_2 strain 1 (%)	WS_2 strain 2 (%)	WS_2 strain 3 (%)	γ (°)	|**a**|(Å)	|**b**|(Å)
5254	187	28	25	C	W	18.613	3.582	4.935	0.427	-3.342	-4.894	-0.423	78.356	18.586	11.439
5255	187	28	25	C	W	41.387	3.582	-4.935	0.427	-3.342	4.894	-0.423	78.356	18.586	11.439
5256	187	28	25	C	W	46.102	-0.414	-4.739	4.490	0.417	4.348	-4.120	85.560	13.152	15.885
5257	187	28	25	C	W	46.827	5.011	-3.248	-0.883	-4.554	3.306	0.899	85.628	13.151	15.889
5258	187	28	25	C	W	50.570	5.011	3.248	-0.883	-4.554	-3.306	0.899	83.149	18.353	11.434
5259	187	28	25	C	W	51.052	-0.599	4.304	4.694	0.606	-3.935	-4.291	85.411	15.901	13.143
5260	187	28	25	C	W	68.948	-0.599	-4.304	4.694	0.606	3.935	-4.291	85.411	15.901	13.143
5261	187	28	25	C	W	69.430	5.011	-3.248	-0.883	-4.554	3.306	0.899	83.149	18.353	11.434
5262	187	28	25	C	W	73.174	5.011	3.248	-0.883	-4.554	-3.306	0.899	85.628	13.151	15.889
5263	187	28	25	C	W	73.898	-0.414	-3.475	4.490	0.417	3.189	-4.120	78.549	11.445	18.569
5264	187	28	25	C	W	78.613	3.582	4.935	0.427	-3.342	-4.894	-0.423	78.356	18.586	11.439
5265	187	28	25	C	W	101.387	3.582	-4.935	0.427	-3.342	4.894	-0.423	78.356	18.586	11.439
5266	187	28	25	C	W	106.102	-0.414	-4.739	4.490	0.417	4.348	-4.120	82.934	11.445	18.339
5267	187	28	25	C	W	106.827	5.011	-3.248	-0.883	-4.554	3.306	0.899	85.628	13.151	15.889
5268	187	28	25	C	W	110.570	5.011	3.248	-0.883	-4.554	-3.306	0.899	83.149	18.353	11.434
5269	187	28	25	C	W	111.052	-0.599	4.304	4.694	0.606	-3.935	-4.291	85.411	15.901	13.143
5270	187	28	25	P	S	13.898	-0.414	-3.475	4.490	0.417	3.189	-4.120	78.549	11.445	18.569
5271	187	28	25	P	S	18.613	3.582	4.935	0.427	-3.342	-4.894	-0.423	78.356	18.586	11.439
5272	187	28	25	P	S	41.387	3.582	-4.935	0.427	-3.342	4.894	-0.423	78.356	18.586	11.439
5273	187	28	25	P	S	46.102	-0.414	-4.739	4.490	0.417	4.348	-4.120	85.560	13.152	15.885
5274	187	28	25	P	S	46.827	5.011	-3.248	-0.883	-4.554	3.306	0.899	85.628	13.151	15.889
5275	187	28	25	P	S	50.570	5.011	3.248	-0.883	-4.554	-3.306	0.899	83.149	18.353	11.434
5276	187	28	25	P	S	51.052	-0.599	4.304	4.694	0.606	-3.935	-4.291	85.411	15.901	13.143
5277	187	28	25	P	S	68.948	-0.599	-4.304	4.694	0.606	3.935	-4.291	85.411	15.901	13.143
5278	187	28	25	P	S	69.430	5.011	-3.248	-0.883	-4.554	3.306	0.899	83.149	18.353	11.434
5279	187	28	25	P	S	73.174	5.011	3.248	-0.883	-4.554	-3.306	0.899	85.628	13.151	15.889
5280	187	28	25	P	S	73.898	-0.414	-3.475	4.490	0.417	3.189	-4.120	78.549	11.445	18.569
5281	187	28	25	P	S	78.613	3.582	4.935	0.427	-3.342	-4.894	-0.423	78.356	18.586	11.439
5282	187	28	25	P	S	101.387	3.582	-4.935	0.427	-3.342	4.894	-0.423	78.356	18.586	11.439
5283	187	28	25	P	S	106.102	-0.414	3.475	4.490	0.417	-3.189	-4.120	78.549	11.445	18.569
5284	187	28	25	P	W	8.948	-0.599	-4.304	4.694	0.606	3.935	-4.291	85.411	15.901	13.143
5285	187	28	25	P	W	9.430	5.011	-3.248	-0.883	-4.554	3.306	0.899	83.149	18.353	11.434
5286	187	28	25	P	W	13.174	5.011	3.248	-0.883	-4.554	-3.306	0.899	85.628	13.151	15.889
5287	187	28	25	P	W	13.898	-0.414	4.739	4.490	0.417	-4.348	-4.120	85.560	15.885	13.152
5288	187	28	25	P	W	18.613	3.582	4.935	0.427	-3.342	-4.894	-0.423	78.356	18.586	11.439
5289	187	28	25	P	W	41.387	3.582	-4.935	0.427	-3.342	4.894	-0.423	78.356	18.586	11.439
5290	187	28	25	P	W	46.102	-0.414	3.475	4.490	0.417	-3.189	-4.120	78.549	11.445	18.569
5291	187	28	25	P	W	73.898	-0.414	-3.475	4.490	0.417	3.189	-4.120	78.549	11.445	18.569
5292	187	28	25	P	W	78.613	3.582	4.935	0.427	-3.342	-4.894	-0.423	78.356	18.586	11.439
5293	187	28	25	P	W	101.387	3.582	-4.935	0.427	-3.342	4.894	-0.423	78.356	18.586	11.439
5294	187	28	25	P	W	106.102	-0.414	-4.739	4.490	0.417	4.348	-4.120	82.934	11.445	18.339
5295	187	28	25	P	W	106.827	5.011	-3.248	-0.883	-4.554	3.306	0.899	85.628	13.151	15.889
5296	187	28	25	P	W	110.570	5.011	3.248	-0.883	-4.554	-3.306	0.899	83.149	18.353	11.434
5297	187	28	25	P	W	111.052	-0.599	4.304	4.694	0.606	-3.935	-4.291	85.411	15.901	13.143
5298	187	31	21	C	S	1.945	-4.723	0.000	-4.723	5.216	0.000	5.216	60.000	15.242	15.242
5299	187	31	21	C	S	19.842	-4.723	0.000	-4.723	5.216	0.000	5.216	60.000	15.242	15.242
5300	187	31	21	C	S	40.158	-4.723	0.000	-4.723	5.216	0.000	5.216	60.000	15.242	15.242
5301	187	31	21	C	S	58.055	-4.723	0.000	-4.723	5.216	0.000	5.216	60.000	15.242	15.242
5302	187	31	21	C	S	61.945	-4.723	0.000	-4.723	5.216	0.000	5.216	60.000	15.242	15.242
5303	187	31	21	C	S	79.842	-4.723	0.000	-4.723	5.216	0.000	5.216	60.000	15.242	15.242
5304	187	31	21	C	S	100.158	-4.723	0.000	-4.723	5.216	0.000	5.216	60.000	15.242	15.242

BL number	Atoms	γ-PC unit cells	WS_2 unit cells	γ-PC origin atom	WS_2 origin atom	Twist-angle (°)	γ-PC strain 1 (%)	γ-PC strain 2 (%)	γ-PC strain 3 (%)	WS_2 strain 1 (%)	WS_2 strain 2 (%)	WS_2 strain 3 (%)	γ (°)	|**a**|(Å)	|**b**|(Å)
5305	187	31	21	C	S	118.055	-4.723	0.000	-4.723	5.216	0.000	5.216	60.000	15.242	15.242
5306	187	31	21	C	W	1.945	-4.723	0.000	-4.723	5.216	0.000	5.216	60.000	15.242	15.242
5307	187	31	21	C	W	19.842	-4.723	0.000	-4.723	5.216	0.000	5.216	60.000	15.242	15.242
5308	187	31	21	C	W	40.158	-4.723	0.000	-4.723	5.216	0.000	5.216	60.000	15.242	15.242
5309	187	31	21	C	W	58.055	-4.723	0.000	-4.723	5.216	0.000	5.216	60.000	15.242	15.242
5310	187	31	21	C	W	61.945	-4.723	0.000	-4.723	5.216	0.000	5.216	60.000	15.242	15.242
5311	187	31	21	C	W	79.842	-4.723	0.000	-4.723	5.216	0.000	5.216	60.000	15.242	15.242
5312	187	31	21	C	W	100.158	-4.723	0.000	-4.723	5.216	0.000	5.216	60.000	15.242	15.242
5313	187	31	21	C	W	118.055	-4.723	0.000	-4.723	5.216	0.000	5.216	60.000	15.242	15.242
5314	187	31	21	C	S	9.515	-4.497	-3.694	-4.948	4.941	4.100	5.492	78.290	11.961	17.178
5315	187	31	21	C	S	50.485	-4.497	3.694	-4.948	4.941	-4.100	5.492	78.290	11.961	17.178
5316	187	31	21	C	S	69.515	-4.497	-3.694	-4.948	4.941	4.100	5.492	78.290	11.961	17.178
5317	187	31	21	C	S	110.485	-4.497	3.694	-4.948	4.941	-4.100	5.492	78.290	11.961	17.178
5318	187	31	21	C	W	9.515	-4.497	-3.694	-4.948	4.941	4.100	5.492	78.290	11.961	17.178
5319	187	31	21	C	W	50.485	-4.497	3.694	-4.948	4.941	-4.100	5.492	78.290	11.961	17.178
5320	187	31	21	C	W	69.515	-4.497	-3.694	-4.948	4.941	4.100	5.492	78.290	11.961	17.178
5321	187	31	21	C	W	110.485	-4.497	3.694	-4.948	4.941	-4.100	5.492	78.290	11.961	17.178
5322	188	29	24	C	S	0.000	5.011	1.045	-4.474	-4.554	-1.148	4.913	69.044	13.151	16.934
5323	188	29	24	C	S	1.945	-4.723	0.135	5.314	5.216	-0.122	-4.803	80.864	15.242	13.824
5324	188	29	24	C	S	7.589	0.334	-4.328	-0.244	-0.332	4.349	0.245	79.538	16.357	12.901
5325	188	29	24	C	S	9.430	5.011	-3.136	-4.474	-4.554	3.444	4.913	71.262	18.353	11.966
5326	188	29	24	C	S	9.515	-4.497	-3.291	5.039	4.941	2.990	-4.577	71.271	11.961	18.361
5327	188	29	24	C	S	10.158	2.281	0.145	-2.097	-2.182	-0.152	2.189	79.480	16.362	12.905
5328	188	29	24	C	S	10.893	-1.983	1.464	2.157	2.064	-1.403	-2.068	79.450	12.906	16.363
5329	188	29	24	C	S	13.174	5.011	3.136	-4.474	-4.554	-3.444	4.913	67.958	13.151	17.061
5330	188	29	24	C	S	13.898	-4.228	4.767	4.716	4.619	-4.356	-4.309	67.946	17.046	13.161
5331	188	29	24	C	S	16.102	2.853	-5.119	-2.615	-2.699	5.402	2.759	75.185	12.551	17.114
5332	188	29	24	C	S	22.689	-4.497	0.512	5.039	4.941	-0.465	-4.577	72.641	11.961	18.218
5333	188	29	24	C	S	23.413	0.481	-2.000	-0.389	-0.477	2.016	0.392	70.126	12.584	17.534
5334	188	29	24	C	S	25.285	4.262	4.843	-3.846	-3.928	-5.247	4.166	72.431	18.222	11.965
5335	188	29	24	C	S	25.767	0.353	2.094	-0.263	-0.351	-2.106	0.264	70.123	17.539	12.581
5336	188	29	24	C	S	34.715	4.262	-4.843	-3.846	-3.928	5.247	4.166	72.431	18.222	11.965
5337	188	29	24	C	S	34.950	-0.955	-0.859	1.064	0.973	0.841	-1.042	70.118	17.539	12.583
5338	188	29	24	C	S	36.587	0.481	2.000	-0.389	-0.477	-2.016	0.392	70.126	12.584	17.534
5339	188	29	24	C	S	37.311	-4.497	-0.512	5.039	4.941	0.465	-4.577	72.641	11.961	18.218
5340	188	29	24	C	S	43.898	2.853	5.119	-2.615	-2.699	-5.402	2.759	75.185	17.114	12.551
5341	188	29	24	C	S	46.102	-4.228	-4.767	4.716	4.619	4.356	-4.309	67.946	13.161	17.046
5342	188	29	24	C	S	46.827	5.011	-3.136	-4.474	-4.554	3.444	4.913	67.958	13.151	17.061
5343	188	29	24	C	S	49.107	-1.983	-1.464	2.157	2.064	1.403	-2.068	79.450	12.906	16.363
5344	188	29	24	C	S	49.842	2.281	-0.145	-2.097	-2.182	0.152	2.189	79.480	16.362	12.905
5345	188	29	24	C	S	50.485	-4.497	3.291	5.039	4.941	-2.990	-4.577	71.271	11.961	18.361
5346	188	29	24	C	S	50.570	5.011	3.136	-4.474	-4.554	-3.444	4.913	71.262	18.353	11.966
5347	188	29	24	C	S	52.411	0.334	4.328	-0.244	-0.332	-4.349	0.245	79.538	12.901	16.357
5348	188	29	24	C	S	58.055	-4.723	-0.135	5.314	5.216	0.122	-4.803	80.864	15.242	13.824
5349	188	29	24	C	S	60.000	5.011	3.136	-4.474	-4.554	-3.444	4.913	80.679	13.826	15.243
5350	188	29	24	C	S	61.945	-4.723	0.135	5.314	5.216	-0.122	-4.803	80.864	15.242	13.824
5351	188	29	24	C	S	67.589	0.334	-4.328	-0.244	-0.332	4.349	0.245	79.538	16.357	12.901
5352	188	29	24	C	S	69.430	5.011	-3.136	-4.474	-4.554	3.444	4.913	71.262	18.353	11.966
5353	188	29	24	C	S	69.515	-4.497	-3.291	5.039	4.941	2.990	-4.577	71.271	11.961	18.361
5354	188	29	24	C	S	70.158	2.281	0.145	-2.097	-2.182	-0.152	2.189	79.480	16.362	12.905
5355	188	29	24	C	S	70.893	-1.983	1.464	2.157	2.064	-1.403	-2.068	79.450	12.906	16.363

BL number	Atoms	γ-PC unit cells	WS_2 unit cells	γ-PC origin atom	WS_2 origin atom	Twist-angle (°)	γ-PC strain 1 (%)	γ-PC strain 2 (%)	γ-PC strain 3 (%)	WS_2 strain 1 (%)	WS_2 strain 2 (%)	WS_2 strain 3 (%)	γ (°)	|**a**|(Å)	|**b**|(Å)
5356	188	29	24	C	S	73.174	5.011	3.136	-4.474	-4.554	-3.444	4.913	67.958	13.151	17.061
5357	188	29	24	C	S	73.898	-4.228	4.767	4.716	4.619	-4.356	-4.309	67.946	17.046	13.161
5358	188	29	24	C	S	76.102	2.853	-5.119	-2.615	-2.699	5.402	2.759	75.185	12.551	17.114
5359	188	29	24	C	S	82.689	-4.497	0.512	5.039	4.941	-0.465	-4.577	72.641	11.961	18.218
5360	188	29	24	C	S	83.413	0.481	-2.000	-0.389	-0.477	2.016	0.392	70.126	12.584	17.534
5361	188	29	24	C	S	85.285	4.262	4.843	-3.846	-3.928	-5.247	4.166	72.431	18.222	11.965
5362	188	29	24	C	S	85.767	0.353	2.094	-0.263	-0.351	-2.106	0.264	70.123	17.539	12.581
5363	188	29	24	C	S	94.715	4.262	-4.843	-3.846	-3.928	5.247	4.166	72.431	18.222	11.965
5364	188	29	24	C	S	94.950	-0.955	-0.859	1.064	0.973	0.841	-1.042	70.118	17.539	12.583
5365	188	29	24	C	S	96.587	0.481	2.000	-0.389	-0.477	-2.016	0.392	70.126	12.584	17.534
5366	188	29	24	C	S	97.311	-4.497	-0.512	5.039	4.941	0.465	-4.577	72.641	11.961	18.218
5367	188	29	24	C	S	103.898	2.853	5.119	-2.615	-2.699	-5.402	2.759	75.185	17.114	12.551
5368	188	29	24	C	S	106.102	-4.228	-4.767	4.716	4.619	4.356	-4.309	67.946	13.161	17.046
5369	188	29	24	C	S	106.827	5.011	-3.136	-4.474	-4.554	3.444	4.913	67.958	13.151	17.061
5370	188	29	24	C	S	109.107	-1.983	-1.464	2.157	2.064	1.403	-2.068	79.450	12.906	16.363
5371	188	29	24	C	S	109.842	2.281	-0.145	-2.097	-2.182	0.152	2.189	79.480	16.362	12.905
5372	188	29	24	C	S	110.485	-4.497	3.291	5.039	4.941	-2.990	-4.577	71.271	11.961	18.361
5373	188	29	24	C	S	110.570	5.011	3.136	-4.474	-4.554	-3.444	4.913	71.262	18.353	11.966
5374	188	29	24	C	S	112.411	0.334	4.328	-0.244	-0.332	-4.349	0.245	79.538	12.901	16.357
5375	188	29	24	C	S	118.055	-4.723	-0.135	5.314	5.216	0.122	-4.803	80.864	15.242	13.824
5376	188	29	24	C	W	0.000	5.011	1.045	-4.474	-4.554	-1.148	4.913	69.044	13.151	16.934
5377	188	29	24	C	W	1.945	-4.723	0.135	5.314	5.216	-0.122	-4.803	80.864	15.242	13.824
5378	188	29	24	C	W	7.589	0.334	-4.328	-0.244	-0.332	4.349	0.245	79.538	16.357	12.901
5379	188	29	24	C	W	9.430	5.011	-3.136	-4.474	-4.554	3.444	4.913	71.262	18.353	11.966
5380	188	29	24	C	W	9.515	-4.497	-3.291	5.039	4.941	2.990	-4.577	71.271	11.961	18.361
5381	188	29	24	C	W	10.158	2.281	0.145	-2.097	-2.182	-0.152	2.189	79.480	16.362	12.905
5382	188	29	24	C	W	10.893	-1.983	1.464	2.157	2.064	-1.403	-2.068	79.450	12.906	16.363
5383	188	29	24	C	W	13.174	5.011	3.136	-4.474	-4.554	-3.444	4.913	67.958	13.151	17.061
5384	188	29	24	C	W	13.898	-4.228	4.767	4.716	4.619	-4.356	-4.309	67.946	17.046	13.161
5385	188	29	24	C	W	16.102	2.853	-5.119	-2.615	-2.699	5.402	2.759	75.185	12.551	17.114
5386	188	29	24	C	W	22.689	-4.497	0.512	5.039	4.941	-0.465	-4.577	72.641	11.961	18.218
5387	188	29	24	C	W	23.413	0.481	-2.000	-0.389	-0.477	2.016	0.392	70.126	12.584	17.534
5388	188	29	24	C	W	25.285	4.262	4.843	-3.846	-3.928	-5.247	4.166	72.431	18.222	11.965
5389	188	29	24	C	W	25.767	0.353	2.094	-0.263	-0.351	-2.106	0.264	70.123	17.539	12.581
5390	188	29	24	C	W	34.715	4.262	-4.843	-3.846	-3.928	5.247	4.166	72.431	18.222	11.965
5391	188	29	24	C	W	34.950	-0.955	-0.859	1.064	0.973	0.841	-1.042	70.118	17.539	12.583
5392	188	29	24	C	W	36.587	0.481	2.000	-0.389	-0.477	-2.016	0.392	70.126	12.584	17.534
5393	188	29	24	C	W	37.311	-4.497	-0.512	5.039	4.941	0.465	-4.577	72.641	11.961	18.218
5394	188	29	24	C	W	43.898	2.853	5.119	-2.615	-2.699	-5.402	2.759	75.185	17.114	12.551
5395	188	29	24	C	W	46.102	-4.228	-4.767	4.716	4.619	4.356	-4.309	67.946	13.161	17.046
5396	188	29	24	C	W	46.827	5.011	-3.136	-4.474	-4.554	3.444	4.913	67.958	13.151	17.061
5397	188	29	24	C	W	49.107	-1.983	-1.464	2.157	2.064	1.403	-2.068	79.450	12.906	16.363
5398	188	29	24	C	W	49.842	2.281	-0.145	-2.097	-2.182	0.152	2.189	79.480	16.362	12.905
5399	188	29	24	C	W	50.485	-4.497	3.291	5.039	4.941	-2.990	-4.577	71.271	11.961	18.361
5400	188	29	24	C	W	50.570	5.011	3.136	-4.474	-4.554	-3.444	4.913	71.262	18.353	11.966
5401	188	29	24	C	W	52.411	0.334	4.328	-0.244	-0.332	-4.349	0.245	79.538	12.901	16.357
5402	188	29	24	C	W	58.055	-4.723	-0.135	5.314	5.216	0.122	-4.803	80.864	15.242	13.824
5403	188	29	24	C	W	60.000	5.011	3.136	-4.474	-4.554	-3.444	4.913	80.679	13.826	15.243
5404	188	29	24	C	W	61.945	-4.723	0.135	5.314	5.216	-0.122	-4.803	80.864	15.242	13.824
5405	188	29	24	C	W	67.589	0.334	-4.328	-0.244	-0.332	4.349	0.245	79.538	16.357	12.901
5406	188	29	24	C	W	69.430	5.011	-3.136	-4.474	-4.554	3.444	4.913	71.262	18.353	11.966

BL number	Atoms	γ-PC unit cells	WS_2 unit cells	γ-PC origin atom	WS_2 origin atom	Twist-angle (°)	γ-PC strain 1 (%)	γ-PC strain 2 (%)	γ-PC strain 3 (%)	WS_2 strain 1 (%)	WS_2 strain 2 (%)	WS_2 strain 3 (%)	γ (°)	|**a**|(Å)	|**b**|(Å)
5407	188	29	24	C	W	69.515	-4.497	-3.291	5.039	4.941	2.990	-4.577	71.271	11.961	18.361
5408	188	29	24	C	W	70.158	2.281	0.145	-2.097	-2.182	-0.152	2.189	79.480	16.362	12.905
5409	188	29	24	C	W	70.893	-1.983	1.464	2.157	2.064	-1.403	-2.068	79.450	12.906	16.363
5410	188	29	24	C	W	73.174	5.011	3.136	-4.474	-4.554	-3.444	4.913	67.958	13.151	17.061
5411	188	29	24	C	W	73.898	-4.228	4.767	4.716	4.619	-4.356	-4.309	67.946	17.046	13.161
5412	188	29	24	C	W	76.102	2.853	-5.119	-2.615	-2.699	5.402	2.759	75.185	12.551	17.114
5413	188	29	24	C	W	82.689	-4.497	0.512	5.039	4.941	-0.465	-4.577	72.641	11.961	18.218
5414	188	29	24	C	W	83.413	0.481	-2.000	-0.389	-0.477	2.016	0.392	70.126	12.584	17.534
5415	188	29	24	C	W	85.285	4.262	4.843	-3.846	-3.928	-5.247	4.166	72.431	18.222	11.965
5416	188	29	24	C	W	85.767	0.353	2.094	-0.263	-0.351	-2.106	0.264	70.123	17.539	12.581
5417	188	29	24	C	W	94.715	4.262	-4.843	-3.846	-3.928	5.247	4.166	72.431	18.222	11.965
5418	188	29	24	C	W	94.950	-0.955	-0.859	1.064	0.973	0.841	-1.042	70.118	17.539	12.583
5419	188	29	24	C	W	96.587	0.481	2.000	-0.389	-0.477	-2.016	0.392	70.126	12.584	17.534
5420	188	29	24	C	W	97.311	-4.497	-0.512	5.039	4.941	0.465	-4.577	72.641	11.961	18.218
5421	188	29	24	C	W	103.898	2.853	5.119	-2.615	-2.699	-5.402	2.759	75.185	17.114	12.551
5422	188	29	24	C	W	106.102	-4.228	-4.767	4.716	4.619	4.356	-4.309	67.946	13.161	17.046
5423	188	29	24	C	W	106.827	5.011	-3.136	-4.474	-4.554	3.444	4.913	67.958	13.151	17.061
5424	188	29	24	C	W	109.107	-1.983	-1.464	2.157	2.064	1.403	-2.068	79.450	12.906	16.363
5425	188	29	24	C	W	109.842	2.281	-0.145	-2.097	-2.182	0.152	2.189	79.480	16.362	12.905
5426	188	29	24	C	W	110.485	-4.497	3.291	5.039	4.941	-2.990	-4.577	71.271	11.961	18.361
5427	188	29	24	C	W	110.570	5.011	3.136	-4.474	-4.554	-3.444	4.913	71.262	18.353	11.966
5428	188	29	24	C	W	112.411	0.334	4.328	-0.244	-0.332	-4.349	0.245	79.538	12.901	16.357
5429	188	29	24	C	W	118.055	-4.723	-0.135	5.314	5.216	0.122	-4.803	80.864	15.242	13.824
5430	188	29	24	P	S	0.000	5.011	1.045	-4.474	-4.554	-1.148	4.913	69.044	13.151	16.934
5431	188	29	24	P	S	1.945	-4.723	0.135	5.314	5.216	-0.122	-4.803	80.864	15.242	13.824
5432	188	29	24	P	S	7.589	0.334	-4.328	-0.244	-0.332	4.349	0.245	79.538	16.357	12.901
5433	188	29	24	P	S	10.158	2.281	0.145	-2.097	-2.182	-0.152	2.189	79.480	16.362	12.905
5434	188	29	24	P	S	10.893	-1.983	1.464	2.157	2.064	-1.403	-2.068	79.450	12.906	16.363
5435	188	29	24	P	S	22.689	-4.497	0.512	5.039	4.941	-0.465	-4.577	72.641	11.961	18.218
5436	188	29	24	P	S	23.413	0.481	-2.000	-0.389	-0.477	2.016	0.392	70.126	12.584	17.534
5437	188	29	24	P	S	25.285	4.262	4.843	-3.846	-3.928	-5.247	4.166	72.431	18.222	11.965
5438	188	29	24	P	S	25.767	0.353	2.094	-0.263	-0.351	-2.106	0.264	70.123	17.539	12.581
5439	188	29	24	P	S	34.715	4.262	-4.843	-3.846	-3.928	5.247	4.166	72.431	18.222	11.965
5440	188	29	24	P	S	34.950	-0.955	-0.859	1.064	0.973	0.841	-1.042	70.118	17.539	12.583
5441	188	29	24	P	S	36.587	0.481	2.000	-0.389	-0.477	-2.016	0.392	70.126	12.584	17.534
5442	188	29	24	P	S	37.311	-4.497	-0.512	5.039	4.941	0.465	-4.577	72.641	11.961	18.218
5443	188	29	24	P	S	49.107	-1.983	-1.464	2.157	2.064	1.403	-2.068	79.450	12.906	16.363
5444	188	29	24	P	S	49.842	2.281	-0.145	-2.097	-2.182	0.152	2.189	79.480	16.362	12.905
5445	188	29	24	P	S	52.411	0.334	4.328	-0.244	-0.332	-4.349	0.245	79.538	12.901	16.357
5446	188	29	24	P	S	60.000	5.011	1.045	-4.474	-4.554	-1.148	4.913	69.044	13.151	16.934
5447	188	29	24	P	S	67.589	0.334	-4.328	-0.244	-0.332	4.349	0.245	79.538	16.357	12.901
5448	188	29	24	P	S	70.158	2.281	0.145	-2.097	-2.182	-0.152	2.189	79.480	16.362	12.905
5449	188	29	24	P	S	70.893	-1.983	1.464	2.157	2.064	-1.403	-2.068	79.450	12.906	16.363
5450	188	29	24	P	S	82.689	-4.497	0.512	5.039	4.941	-0.465	-4.577	72.641	11.961	18.218
5451	188	29	24	P	S	83.413	0.481	-2.000	-0.389	-0.477	2.016	0.392	70.126	12.584	17.534
5452	188	29	24	P	S	85.285	4.262	4.843	-3.846	-3.928	-5.247	4.166	72.431	18.222	11.965
5453	188	29	24	P	S	85.767	0.353	2.094	-0.263	-0.351	-2.106	0.264	70.123	17.539	12.581
5454	188	29	24	P	S	94.715	4.262	-4.843	-3.846	-3.928	5.247	4.166	72.431	18.222	11.965
5455	188	29	24	P	S	94.950	-0.955	-0.859	1.064	0.973	0.841	-1.042	70.118	17.539	12.583
5456	188	29	24	P	S	96.587	0.481	2.000	-0.389	-0.477	-2.016	0.392	70.126	12.584	17.534
5457	188	29	24	P	S	97.311	-4.497	-0.512	5.039	4.941	0.465	-4.577	72.641	11.961	18.218

BL number	Atoms	γ-PC unit cells	WS_2 unit cells	γ-PC origin atom	WS_2 origin atom	Twist-angle (°)	γ-PC strain 1 (%)	γ-PC strain 2 (%)	γ-PC strain 3 (%)	WS_2 strain 1 (%)	WS_2 strain 2 (%)	WS_2 strain 3 (%)	γ (°)	$\lvert\mathbf{a}\rvert$(Å)	$\lvert\mathbf{b}\rvert$(Å)
5458	188	29	24	P	S	109.107	-1.983	-1.464	2.157	2.064	1.403	-2.068	79.450	12.906	16.363
5459	188	29	24	P	S	109.842	2.281	-0.145	-2.097	-2.182	0.152	2.189	79.480	16.362	12.905
5460	188	29	24	P	S	112.411	0.334	4.328	-0.244	-0.332	-4.349	0.245	79.538	12.901	16.357
5461	188	29	24	P	S	118.055	-4.723	-0.135	5.314	5.216	0.122	-4.803	80.864	15.242	13.824
5462	188	29	24	P	W	0.000	5.011	1.045	-4.474	-4.554	-1.148	4.913	69.044	13.151	16.934
5463	188	29	24	P	W	7.589	0.334	-4.328	-0.244	-0.332	4.349	0.245	79.538	16.357	12.901
5464	188	29	24	P	W	10.158	2.281	0.145	-2.097	-2.182	-0.152	2.189	79.480	16.362	12.905
5465	188	29	24	P	W	10.893	-1.983	1.464	2.157	2.064	-1.403	-2.068	79.450	12.906	16.363
5466	188	29	24	P	W	22.689	-4.497	0.512	5.039	4.941	-0.465	-4.577	72.641	11.961	18.218
5467	188	29	24	P	W	23.413	0.481	-2.000	-0.389	-0.477	2.016	0.392	70.126	12.584	17.534
5468	188	29	24	P	W	25.285	4.262	4.843	-3.846	-3.928	-5.247	4.166	72.431	18.222	11.965
5469	188	29	24	P	W	25.767	0.353	2.094	-0.263	-0.351	-2.106	0.264	70.123	17.539	12.581
5470	188	29	24	P	W	34.715	4.262	-4.843	-3.846	-3.928	5.247	4.166	72.431	18.222	11.965
5471	188	29	24	P	W	34.950	-0.955	-0.859	1.064	0.973	0.841	-1.042	70.118	17.539	12.583
5472	188	29	24	P	W	36.587	0.481	2.000	-0.389	-0.477	-2.016	0.392	70.126	12.584	17.534
5473	188	29	24	P	W	37.311	-4.497	-0.512	5.039	4.941	0.465	-4.577	72.641	11.961	18.218
5474	188	29	24	P	W	49.107	-1.983	-1.464	2.157	2.064	1.403	-2.068	79.450	12.906	16.363
5475	188	29	24	P	W	49.842	2.281	-0.145	-2.097	-2.182	0.152	2.189	79.480	16.362	12.905
5476	188	29	24	P	W	52.411	0.334	4.328	-0.244	-0.332	-4.349	0.245	79.538	12.901	16.357
5477	188	29	24	P	W	58.055	-4.723	-0.135	5.314	5.216	0.122	-4.803	80.864	15.242	13.824
5478	188	29	24	P	W	60.000	5.011	3.136	-4.474	-4.554	-3.444	4.913	80.679	13.826	15.243
5479	188	29	24	P	W	61.945	-4.723	0.135	5.314	5.216	-0.122	-4.803	80.864	15.242	13.824
5480	188	29	24	P	W	67.589	0.334	-4.328	-0.244	-0.332	4.349	0.245	79.538	16.357	12.901
5481	188	29	24	P	W	70.158	2.281	0.145	-2.097	-2.182	-0.152	2.189	79.480	16.362	12.905
5482	188	29	24	P	W	70.893	-1.983	1.464	2.157	2.064	-1.403	-2.068	79.450	12.906	16.363
5483	188	29	24	P	W	82.689	-4.497	0.512	5.039	4.941	-0.465	-4.577	72.641	11.961	18.218
5484	188	29	24	P	W	83.413	0.481	-2.000	-0.389	-0.477	2.016	0.392	70.126	12.584	17.534
5485	188	29	24	P	W	85.285	4.262	4.843	-3.846	-3.928	-5.247	4.166	72.431	18.222	11.965
5486	188	29	24	P	W	85.767	0.353	2.094	-0.263	-0.351	-2.106	0.264	70.123	17.539	12.581
5487	188	29	24	P	W	94.715	4.262	-4.843	-3.846	-3.928	5.247	4.166	72.431	18.222	11.965
5488	188	29	24	P	W	94.950	-0.955	-0.859	1.064	0.973	0.841	-1.042	70.118	17.539	12.583
5489	188	29	24	P	W	96.587	0.481	2.000	-0.389	-0.477	-2.016	0.392	70.126	12.584	17.534
5490	188	29	24	P	W	97.311	-4.497	-0.512	5.039	4.941	0.465	-4.577	72.641	11.961	18.218
5491	188	29	24	P	W	109.107	-1.983	-1.464	2.157	2.064	1.403	-2.068	79.450	12.906	16.363
5492	188	29	24	P	W	109.842	2.281	-0.145	-2.097	-2.182	0.152	2.189	79.480	16.362	12.905
5493	188	29	24	P	W	112.411	0.334	4.328	-0.244	-0.332	-4.349	0.245	79.538	12.901	16.357
5494	189	30	23	C	S	6.587	-3.853	-3.944	0.276	4.175	3.923	-0.275	87.747	14.354	14.417
5495	189	30	23	C	S	8.948	1.048	0.188	-4.551	-1.026	-0.207	5.006	83.142	17.420	11.961
5496	189	30	23	C	S	9.515	-4.497	1.202	0.987	4.941	-1.178	-0.968	83.095	11.961	17.421
5497	189	30	23	C	S	10.893	0.418	3.451	-3.983	-0.415	-3.750	4.328	87.942	14.426	14.344
5498	189	30	23	C	S	17.480	-4.685	5.117	1.199	5.169	-4.997	-1.171	82.554	14.491	14.398
5499	189	30	23	C	S	19.107	-1.485	-3.656	-2.178	1.530	3.823	2.277	87.859	14.708	14.063
5500	189	30	23	C	S	23.413	-2.043	3.721	-1.622	2.130	-3.846	1.676	87.834	14.072	14.698
5501	189	30	23	C	S	27.126	1.029	0.657	-4.534	-1.008	-0.722	4.986	82.326	14.841	14.065
5502	189	30	23	C	S	32.874	1.029	-0.657	-4.534	-1.008	0.722	4.986	82.326	14.065	14.841
5503	189	30	23	C	S	36.587	-2.043	-3.721	-1.622	2.130	3.845	1.676	87.834	14.072	14.698
5504	189	30	23	C	S	40.893	-1.485	3.657	-2.178	1.530	-3.823	2.277	87.859	14.708	14.063
5505	189	30	23	C	S	42.520	-4.685	-5.117	1.199	5.169	4.997	-1.171	82.554	14.491	14.398
5506	189	30	23	C	S	49.107	0.418	-3.451	-3.983	-0.415	3.750	4.328	87.942	14.426	14.344
5507	189	30	23	C	S	50.485	-4.497	-1.202	0.987	4.941	1.178	-0.968	83.095	11.961	17.421
5508	189	30	23	C	S	51.052	1.048	-0.188	-4.551	-1.026	0.207	5.006	83.142	17.420	11.961

BL number	Atoms	γ-PC unit cells	WS_2 unit cells	γ-PC origin atom	WS_2 origin atom	Twist-angle (°)	γ-PC strain 1 (%)	γ-PC strain 2 (%)	γ-PC strain 3 (%)	WS_2 strain 1 (%)	WS_2 strain 2 (%)	WS_2 strain 3 (%)	γ (°)	|**a**|(Å)	|**b**|(Å)
5509	189	30	23	C	S	53.413	-3.853	3.944	0.276	4.175	-3.923	-0.275	87.747	14.354	14.417
5510	189	30	23	C	S	66.587	-3.853	-3.944	0.276	4.175	3.923	-0.275	87.748	14.354	14.417
5511	189	30	23	C	S	68.948	1.048	0.188	-4.551	-1.026	-0.207	5.006	83.142	17.420	11.961
5512	189	30	23	C	S	69.515	-4.497	1.202	0.987	4.941	-1.178	-0.968	83.095	11.961	17.421
5513	189	30	23	C	S	70.893	0.418	3.451	-3.983	-0.415	-3.750	4.328	87.942	14.426	14.344
5514	189	30	23	C	S	77.480	-4.685	5.117	1.199	5.169	-4.997	-1.171	82.554	14.491	14.398
5515	189	30	23	C	S	79.107	-1.485	-3.656	-2.178	1.530	3.823	2.277	87.859	14.708	14.063
5516	189	30	23	C	S	83.413	-2.043	3.721	-1.622	2.130	-3.846	1.676	87.834	14.072	14.698
5517	189	30	23	C	S	87.126	1.029	0.657	-4.534	-1.008	-0.722	4.986	82.326	14.841	14.065
5518	189	30	23	C	S	92.874	1.029	-0.657	-4.534	-1.008	0.722	4.986	82.326	14.065	14.841
5519	189	30	23	C	S	96.587	-2.043	-3.721	-1.622	2.130	3.845	1.676	87.834	14.072	14.698
5520	189	30	23	C	S	100.893	-1.485	3.657	-2.178	1.530	-3.823	2.277	87.859	14.708	14.063
5521	189	30	23	C	S	102.520	-4.685	-5.117	1.199	5.169	4.997	-1.171	82.554	14.491	14.398
5522	189	30	23	C	S	109.107	0.418	-3.451	-3.983	-0.415	3.750	4.328	87.942	14.426	14.344
5523	189	30	23	C	S	110.485	-4.497	-1.202	0.987	4.941	1.178	-0.968	83.095	11.961	17.421
5524	189	30	23	C	S	111.052	1.048	-0.188	-4.551	-1.026	0.207	5.006	83.142	17.420	11.961
5525	189	30	23	C	S	113.413	-3.853	3.944	0.276	4.175	-3.923	-0.275	87.748	14.354	14.417
5526	189	30	23	C	W	6.587	-3.853	-3.944	0.276	4.175	3.923	-0.275	87.747	14.354	14.417
5527	189	30	23	C	W	8.948	1.048	0.188	-4.551	-1.026	-0.207	5.006	83.142	17.420	11.961
5528	189	30	23	C	W	9.515	-4.497	1.202	0.987	4.941	-1.178	-0.968	83.095	11.961	17.421
5529	189	30	23	C	W	10.893	0.418	3.451	-3.983	-0.415	-3.750	4.328	87.942	14.426	14.344
5530	189	30	23	C	W	17.480	-4.685	5.117	1.199	5.169	-4.997	-1.171	82.554	14.491	14.398
5531	189	30	23	C	W	27.126	1.029	0.657	-4.534	-1.008	-0.722	4.986	82.326	14.841	14.065
5532	189	30	23	C	W	32.874	1.029	-0.657	-4.534	-1.008	0.722	4.986	82.326	14.065	14.841
5533	189	30	23	C	W	42.520	-4.685	-5.117	1.199	5.169	4.997	-1.171	82.554	14.491	14.398
5534	189	30	23	C	W	49.107	0.418	-3.451	-3.983	-0.415	3.750	4.328	87.942	14.426	14.344
5535	189	30	23	C	W	50.485	-4.497	-1.202	0.987	4.941	1.178	-0.968	83.095	11.961	17.421
5536	189	30	23	C	W	51.052	1.048	-0.188	-4.551	-1.026	0.207	5.006	83.142	17.420	11.961
5537	189	30	23	C	W	53.413	-3.853	3.944	0.276	4.175	-3.923	-0.275	87.747	14.354	14.417
5538	189	30	23	C	W	66.587	-3.853	-3.944	0.276	4.175	3.923	-0.275	87.748	14.354	14.417
5539	189	30	23	C	W	68.948	1.048	0.188	-4.551	-1.026	-0.207	5.006	83.142	17.420	11.961
5540	189	30	23	C	W	69.515	-4.497	1.202	0.987	4.941	-1.178	-0.968	83.095	11.961	17.421
5541	189	30	23	C	W	70.893	0.418	3.451	-3.983	-0.415	-3.750	4.328	87.942	14.426	14.344
5542	189	30	23	C	W	77.480	-4.685	5.117	1.199	5.169	-4.997	-1.171	82.554	14.491	14.398
5543	189	30	23	C	W	87.126	1.029	0.657	-4.534	-1.008	-0.722	4.986	82.326	14.841	14.065
5544	189	30	23	C	W	92.874	1.029	-0.657	-4.534	-1.008	0.722	4.986	82.326	14.065	14.841
5545	189	30	23	C	W	102.520	-4.685	-5.117	1.199	5.169	4.997	-1.171	82.554	14.491	14.398
5546	189	30	23	C	W	109.107	0.418	-3.451	-3.983	-0.415	3.750	4.328	87.942	14.426	14.344
5547	189	30	23	C	W	110.485	-4.497	-1.202	0.987	4.941	1.178	-0.968	83.095	11.961	17.421
5548	189	30	23	C	W	111.052	1.048	-0.188	-4.551	-1.026	0.207	5.006	83.142	17.420	11.961
5549	189	30	23	C	W	113.413	-3.853	3.944	0.276	4.175	-3.923	-0.275	87.748	14.354	14.417
5550	189	30	23	P	S	6.587	-3.853	-3.944	0.276	4.175	3.923	-0.275	87.747	14.354	14.417
5551	189	30	23	P	S	8.948	1.048	0.188	-4.551	-1.026	-0.207	5.006	83.142	17.420	11.961
5552	189	30	23	P	S	9.515	-4.497	1.202	0.987	4.941	-1.178	-0.968	83.095	11.961	17.421
5553	189	30	23	P	S	10.893	0.418	3.451	-3.983	-0.415	-3.750	4.328	87.942	14.426	14.344
5554	189	30	23	P	S	17.480	-4.685	5.117	1.199	5.169	-4.997	-1.171	82.554	14.491	14.398
5555	189	30	23	P	S	19.107	-1.485	-3.656	-2.178	1.530	3.823	2.277	87.859	14.708	14.063
5556	189	30	23	P	S	23.413	-2.043	3.721	-1.622	2.130	-3.846	1.676	87.834	14.072	14.698
5557	189	30	23	P	S	27.126	1.029	0.657	-4.534	-1.008	-0.722	4.986	82.326	14.841	14.065
5558	189	30	23	P	S	32.874	1.029	-0.657	-4.534	-1.008	0.722	4.986	82.326	14.065	14.841
5559	189	30	23	P	S	36.587	-2.043	-3.721	-1.622	2.130	3.845	1.676	87.834	14.072	14.698

BL number	Atoms	γ-PC unit cells	WS_2 unit cells	γ-PC origin atom	WS_2 origin atom	Twist-angle (°)	γ-PC strain 1 (%)	γ-PC strain 2 (%)	γ-PC strain 3 (%)	WS_2 strain 1 (%)	WS_2 strain 2 (%)	WS_2 strain 3 (%)	γ (°)	|**a**|(Å)	|**b**|(Å)
5560	189	30	23	P	S	40.893	-1.485	3.657	-2.178	1.530	-3.823	2.277	87.859	14.708	14.063
5561	189	30	23	P	S	42.520	-4.685	-5.117	1.199	5.169	4.997	-1.171	82.554	14.491	14.398
5562	189	30	23	P	S	49.107	0.418	-3.451	-3.983	-0.415	3.750	4.328	87.942	14.426	14.344
5563	189	30	23	P	S	50.485	-4.497	-1.202	0.987	4.941	1.178	-0.968	83.095	11.961	17.421
5564	189	30	23	P	S	51.052	1.048	-0.188	-4.551	-1.026	0.207	5.006	83.142	17.420	11.961
5565	189	30	23	P	S	53.413	-3.853	3.944	0.276	4.175	-3.923	-0.275	87.747	14.354	14.417
5566	189	30	23	P	S	66.587	-3.853	-3.944	0.276	4.175	3.923	-0.275	87.748	14.354	14.417
5567	189	30	23	P	S	68.948	1.048	0.188	-4.551	-1.026	-0.207	5.006	83.142	17.420	11.961
5568	189	30	23	P	S	69.515	-4.497	1.202	0.987	4.941	-1.178	-0.968	83.095	11.961	17.421
5569	189	30	23	P	S	70.893	0.418	3.451	-3.983	-0.415	-3.750	4.328	87.942	14.426	14.344
5570	189	30	23	P	S	77.480	-4.685	5.117	1.199	5.169	-4.997	-1.171	82.554	14.491	14.398
5571	189	30	23	P	S	79.107	-1.485	-3.656	-2.178	1.530	3.823	2.277	87.859	14.708	14.063
5572	189	30	23	P	S	83.413	-2.043	3.721	-1.622	2.130	-3.846	1.676	87.834	14.072	14.698
5573	189	30	23	P	S	87.126	1.029	0.657	-4.534	-1.008	-0.722	4.986	82.326	14.841	14.065
5574	189	30	23	P	S	92.874	1.029	-0.657	-4.534	-1.008	0.722	4.986	82.326	14.065	14.841
5575	189	30	23	P	S	96.587	-2.043	-3.721	-1.622	2.130	3.845	1.676	87.834	14.072	14.698
5576	189	30	23	P	S	100.893	-1.485	3.657	-2.178	1.530	-3.823	2.277	87.859	14.708	14.063
5577	189	30	23	P	S	102.520	-4.685	-5.117	1.199	5.169	4.997	-1.171	82.554	14.491	14.398
5578	189	30	23	P	S	109.107	0.418	-3.451	-3.983	-0.415	3.750	4.328	87.942	14.426	14.344
5579	189	30	23	P	S	110.485	-4.497	-1.202	0.987	4.941	1.178	-0.968	83.095	11.961	17.421
5580	189	30	23	P	S	111.052	1.048	-0.188	-4.551	-1.026	0.207	5.006	83.142	17.420	11.961
5581	189	30	23	P	S	113.413	-3.853	3.944	0.276	4.175	-3.923	-0.275	87.748	14.354	14.417
5582	189	30	23	P	W	6.587	-3.853	-3.944	0.276	4.175	3.923	-0.275	87.747	14.354	14.417
5583	189	30	23	P	W	8.948	1.048	0.188	-4.551	-1.026	-0.207	5.006	83.142	17.420	11.961
5584	189	30	23	P	W	9.515	-4.497	1.202	0.987	4.941	-1.178	-0.968	83.095	11.961	17.421
5585	189	30	23	P	W	10.893	0.418	3.451	-3.983	-0.415	-3.750	4.328	87.942	14.426	14.344
5586	189	30	23	P	W	17.480	-4.685	5.117	1.199	5.169	-4.997	-1.171	82.554	14.491	14.398
5587	189	30	23	P	W	27.126	1.029	0.657	-4.534	-1.008	-0.722	4.986	82.326	14.841	14.065
5588	189	30	23	P	W	32.874	1.029	-0.657	-4.534	-1.008	0.722	4.986	82.326	14.065	14.841
5589	189	30	23	P	W	42.520	-4.685	-5.117	1.199	5.169	4.997	-1.171	82.554	14.491	14.398
5590	189	30	23	P	W	49.107	0.418	-3.451	-3.983	-0.415	3.750	4.328	87.942	14.426	14.344
5591	189	30	23	P	W	50.485	-4.497	-1.202	0.987	4.941	1.178	-0.968	83.095	11.961	17.421
5592	189	30	23	P	W	51.052	1.048	-0.188	-4.551	-1.026	0.207	5.006	83.142	17.420	11.961
5593	189	30	23	P	W	53.413	-3.853	3.944	0.276	4.175	-3.923	-0.275	87.747	14.354	14.417
5594	189	30	23	P	W	66.587	-3.853	-3.944	0.276	4.175	3.923	-0.275	87.748	14.354	14.417
5595	189	30	23	P	W	68.948	1.048	0.188	-4.551	-1.026	-0.207	5.006	83.142	17.420	11.961
5596	189	30	23	P	W	69.515	-4.497	1.202	0.987	4.941	-1.178	-0.968	83.095	11.961	17.421
5597	189	30	23	P	W	70.893	0.418	3.451	-3.983	-0.415	-3.750	4.328	87.942	14.426	14.344
5598	189	30	23	P	W	77.480	-4.685	5.117	1.199	5.169	-4.997	-1.171	82.554	14.491	14.398
5599	189	30	23	P	W	87.126	1.029	0.657	-4.534	-1.008	-0.722	4.986	82.326	14.841	14.065
5600	189	30	23	P	W	92.874	1.029	-0.657	-4.534	-1.008	0.722	4.986	82.326	14.065	14.841
5601	189	30	23	P	W	102.520	-4.685	-5.117	1.199	5.169	4.997	-1.171	82.554	14.491	14.398
5602	189	30	23	P	W	109.107	0.418	-3.451	-3.983	-0.415	3.750	4.328	87.942	14.426	14.344
5603	189	30	23	P	W	110.485	-4.497	-1.202	0.987	4.941	1.178	-0.968	83.095	11.961	17.421
5604	189	30	23	P	W	111.052	1.048	-0.188	-4.551	-1.026	0.207	5.006	83.142	17.420	11.961
5605	189	30	23	P	W	113.413	-3.853	3.944	0.276	4.175	-3.923	-0.275	87.748	14.354	14.417
5606	191	29	25	C	S	8.513	2.390	-3.450	-0.204	-2.281	3.464	0.205	81.666	13.459	15.908
5607	191	29	25	C	S	8.948	-0.599	-2.771	2.808	0.606	2.623	-2.659	81.677	15.901	13.466
5608	191	29	25	C	S	12.520	2.326	3.538	-0.143	-2.223	-3.548	0.144	81.571	13.473	15.896
5609	191	29	25	C	S	47.480	2.326	-3.538	-0.143	-2.223	3.548	0.144	81.571	13.473	15.896
5610	191	29	25	C	S	51.052	-0.599	2.771	2.808	0.606	-2.623	-2.659	81.677	15.901	13.466

BL number	Atoms	γ-PC unit cells	WS_2 unit cells	γ-PC origin atom	WS_2 origin atom	Twist-angle (°)	γ-PC strain 1 (%)	γ-PC strain 2 (%)	γ-PC strain 3 (%)	WS_2 strain 1 (%)	WS_2 strain 2 (%)	WS_2 strain 3 (%)	γ (°)	$\lvert\mathbf{a}\rvert$(Å)	$\lvert\mathbf{b}\rvert$(Å)
5611	191	29	25	C	S	51.487	2.390	3.450	-0.204	-2.281	-3.464	0.205	81.666	15.908	13.459
5612	191	29	25	C	S	68.513	2.390	-3.450	-0.204	-2.281	3.464	0.205	81.665	13.459	15.908
5613	191	29	25	C	S	68.948	-0.599	-2.771	2.808	0.606	2.623	-2.659	81.677	15.901	13.466
5614	191	29	25	C	S	72.520	2.326	3.538	-0.143	-2.223	-3.548	0.144	81.572	13.473	15.896
5615	191	29	25	C	S	107.480	2.326	-3.538	-0.143	-2.223	3.548	0.144	81.572	13.473	15.896
5616	191	29	25	C	S	111.052	-0.599	2.771	2.808	0.606	-2.623	-2.659	81.677	15.901	13.466
5617	191	29	25	C	S	111.487	2.390	3.450	-0.204	-2.281	-3.464	0.205	81.665	15.908	13.459
5618	191	29	25	C	W	8.513	2.390	-3.450	-0.204	-2.281	3.464	0.205	81.666	13.459	15.908
5619	191	29	25	C	W	8.948	-0.599	-2.771	2.808	0.606	2.623	-2.659	81.677	15.901	13.466
5620	191	29	25	C	W	12.520	2.326	3.538	-0.143	-2.223	-3.548	0.144	81.571	13.473	15.896
5621	191	29	25	C	W	47.480	2.326	-3.538	-0.143	-2.223	3.548	0.144	81.571	13.473	15.896
5622	191	29	25	C	W	51.052	-0.599	2.771	2.808	0.606	-2.623	-2.659	81.677	15.901	13.466
5623	191	29	25	C	W	51.487	2.390	3.450	-0.204	-2.281	-3.464	0.205	81.666	15.908	13.459
5624	191	29	25	C	W	68.513	2.390	-3.450	-0.204	-2.281	3.464	0.205	81.665	13.459	15.908
5625	191	29	25	C	W	68.948	-0.599	-2.771	2.808	0.606	2.623	-2.659	81.677	15.901	13.466
5626	191	29	25	C	W	72.520	2.326	3.538	-0.143	-2.223	-3.548	0.144	81.572	13.473	15.896
5627	191	29	25	C	W	107.480	2.326	-3.538	-0.143	-2.223	3.548	0.144	81.572	13.473	15.896
5628	191	29	25	C	W	111.052	-0.599	2.771	2.808	0.606	-2.623	-2.659	81.677	15.901	13.466
5629	191	29	25	C	W	111.487	2.390	3.450	-0.204	-2.281	-3.464	0.205	81.665	15.908	13.459
5630	191	29	25	P	S	8.513	2.390	-3.450	-0.204	-2.281	3.464	0.205	81.666	13.459	15.908
5631	191	29	25	P	S	8.948	-0.599	-2.771	2.808	0.606	2.623	-2.659	81.677	15.901	13.466
5632	191	29	25	P	S	12.520	2.326	3.538	-0.143	-2.223	-3.548	0.144	81.571	13.473	15.896
5633	191	29	25	P	S	47.480	2.326	-3.538	-0.143	-2.223	3.548	0.144	81.571	13.473	15.896
5634	191	29	25	P	S	51.052	-0.599	2.771	2.808	0.606	-2.623	-2.659	81.677	15.901	13.466
5635	191	29	25	P	S	51.487	2.390	3.450	-0.204	-2.281	-3.464	0.205	81.666	15.908	13.459
5636	191	29	25	P	S	68.513	2.390	-3.450	-0.204	-2.281	3.464	0.205	81.665	13.459	15.908
5637	191	29	25	P	S	68.948	-0.599	-2.771	2.808	0.606	2.623	-2.659	81.677	15.901	13.466
5638	191	29	25	P	S	72.520	2.326	3.538	-0.143	-2.223	-3.548	0.144	81.572	13.473	15.896
5639	191	29	25	P	S	107.480	2.326	-3.538	-0.143	-2.223	3.548	0.144	81.572	13.473	15.896
5640	191	29	25	P	S	111.052	-0.599	2.771	2.808	0.606	-2.623	-2.659	81.677	15.901	13.466
5641	191	29	25	P	S	111.487	2.390	3.450	-0.204	-2.281	-3.464	0.205	81.665	15.908	13.459
5642	191	29	25	P	W	8.513	2.390	-3.450	-0.204	-2.281	3.464	0.205	81.666	13.459	15.908
5643	191	29	25	P	W	8.948	-0.599	-2.771	2.808	0.606	2.623	-2.659	81.677	15.901	13.466
5644	191	29	25	P	W	12.520	2.326	3.538	-0.143	-2.223	-3.548	0.144	81.571	13.473	15.896
5645	191	29	25	P	W	47.480	2.326	-3.538	-0.143	-2.223	3.548	0.144	81.571	13.473	15.896
5646	191	29	25	P	W	51.052	-0.599	2.771	2.808	0.606	-2.623	-2.659	81.677	15.901	13.466
5647	191	29	25	P	W	51.487	2.390	3.450	-0.204	-2.281	-3.464	0.205	81.666	15.908	13.459
5648	191	29	25	P	W	68.513	2.390	-3.450	-0.204	-2.281	3.464	0.205	81.665	13.459	15.908
5649	191	29	25	P	W	68.948	-0.599	-2.771	2.808	0.606	2.623	-2.659	81.677	15.901	13.466
5650	191	29	25	P	W	72.520	2.326	3.538	-0.143	-2.223	-3.548	0.144	81.572	13.473	15.896
5651	191	29	25	P	W	107.480	2.326	-3.538	-0.143	-2.223	3.548	0.144	81.572	13.473	15.896
5652	191	29	25	P	W	111.052	-0.599	2.771	2.808	0.606	-2.623	-2.659	81.677	15.901	13.466
5653	191	29	25	P	W	111.487	2.390	3.450	-0.204	-2.281	-3.464	0.205	81.665	15.908	13.459
5654	192	30	24	C	S	7.154	-0.955	-3.689	-0.638	0.973	3.737	0.647	69.960	17.421	12.897
5655	192	30	24	C	S	7.589	0.334	-2.896	-1.903	-0.332	3.011	1.978	80.461	14.419	14.845
5656	192	30	24	C	S	8.213	-2.359	-1.879	0.817	2.476	1.849	-0.804	80.477	14.845	14.421
5657	192	30	24	C	S	8.948	1.048	-0.565	-2.575	-1.026	0.595	2.714	69.944	17.420	12.903
5658	192	30	24	C	S	10.893	-1.983	2.830	0.418	2.064	-2.806	-0.415	69.882	12.906	17.420
5659	192	30	24	C	S	49.107	-1.983	-2.830	0.418	2.064	2.806	-0.415	69.882	12.906	17.420
5660	192	30	24	C	S	51.052	1.048	0.565	-2.575	-1.026	-0.595	2.715	69.944	17.420	12.903
5661	192	30	24	C	S	51.787	-2.359	1.879	0.817	2.476	-1.849	-0.804	80.477	14.845	14.421

BL number	Atoms	γ-PC unit cells	WS_2 unit cells	γ-PC origin atom	WS_2 origin atom	Twist-angle (°)	γ-PC strain 1 (%)	γ-PC strain 2 (%)	γ-PC strain 3 (%)	WS_2 strain 1 (%)	WS_2 strain 2 (%)	WS_2 strain 3 (%)	γ (°)	$\lvert\mathbf{a}\rvert$(Å)	$\lvert\mathbf{b}\rvert$(Å)
5662	192	30	24	C	S	52.411	0.334	2.896	-1.903	-0.332	-3.011	1.978	80.461	14.845	14.419
5663	192	30	24	C	S	52.846	-0.955	3.689	-0.638	0.973	-3.737	0.647	69.960	12.897	17.421
5664	192	30	24	C	S	67.154	-0.955	-3.689	-0.638	0.973	3.737	0.647	69.960	17.421	12.897
5665	192	30	24	C	S	67.589	0.334	-2.896	-1.903	-0.332	3.011	1.978	80.461	14.419	14.845
5666	192	30	24	C	S	68.213	-2.359	-1.879	0.817	2.476	1.849	-0.804	80.477	14.845	14.421
5667	192	30	24	C	S	68.948	1.048	-0.565	-2.575	-1.026	0.595	2.714	69.944	17.420	12.903
5668	192	30	24	C	S	70.893	0.418	2.761	-1.983	-0.415	-2.875	2.064	80.408	14.426	14.841
5669	192	30	24	C	S	109.107	0.418	-2.761	-1.983	-0.415	2.875	2.064	80.408	14.426	14.841
5670	192	30	24	C	S	111.052	1.048	0.565	-2.575	-1.026	-0.595	2.715	69.944	17.420	12.903
5671	192	30	24	C	S	111.787	-2.359	1.879	0.817	2.476	-1.849	-0.804	80.477	14.845	14.421
5672	192	30	24	C	S	112.411	0.334	2.896	-1.903	-0.332	-3.011	1.978	80.461	14.845	14.419
5673	192	30	24	C	S	112.846	-0.955	3.689	-0.638	0.973	-3.737	0.647	69.960	12.897	17.421
5674	192	30	24	C	W	7.154	-0.955	-3.689	-0.638	0.973	3.737	0.647	69.960	17.421	12.897
5675	192	30	24	C	W	8.948	1.048	-0.565	-2.575	-1.026	0.595	2.714	69.944	17.420	12.903
5676	192	30	24	C	W	10.893	-1.983	2.830	0.418	2.064	-2.806	-0.415	69.882	12.906	17.420
5677	192	30	24	C	W	49.107	-1.983	-2.830	0.418	2.064	2.806	-0.415	69.882	12.906	17.420
5678	192	30	24	C	W	51.052	1.048	0.565	-2.575	-1.026	-0.595	2.715	69.944	17.420	12.903
5679	192	30	24	C	W	52.846	-0.955	3.689	-0.638	0.973	-3.737	0.647	69.960	12.897	17.421
5680	192	30	24	C	W	67.154	-0.955	-3.689	-0.638	0.973	3.737	0.647	69.960	17.421	12.897
5681	192	30	24	C	W	68.948	1.048	-0.565	-2.575	-1.026	0.595	2.714	69.944	17.420	12.903
5682	192	30	24	C	W	70.893	-1.983	2.830	0.418	2.064	-2.806	-0.415	69.882	12.906	17.420
5683	192	30	24	C	W	109.107	-1.983	-2.830	0.418	2.064	2.806	-0.415	69.882	12.906	17.420
5684	192	30	24	C	W	111.052	1.048	0.565	-2.575	-1.026	-0.595	2.715	69.944	17.420	12.903
5685	192	30	24	C	W	112.846	-0.955	3.689	-0.638	0.973	-3.737	0.647	69.960	12.897	17.421
5686	192	30	24	P	S	7.589	0.334	-2.896	-1.903	-0.332	3.011	1.978	80.461	14.419	14.845
5687	192	30	24	P	S	8.213	-2.359	-1.879	0.817	2.476	1.849	-0.804	80.477	14.845	14.421
5688	192	30	24	P	S	10.893	0.418	2.761	-1.983	-0.415	-2.875	2.064	80.408	14.426	14.841
5689	192	30	24	P	S	49.107	0.418	-2.761	-1.983	-0.415	2.875	2.064	80.408	14.426	14.841
5690	192	30	24	P	S	51.787	-2.359	1.879	0.817	2.476	-1.849	-0.804	80.477	14.845	14.421
5691	192	30	24	P	S	52.411	0.334	2.896	-1.903	-0.332	-3.011	1.978	80.461	14.845	14.419
5692	192	30	24	P	S	67.589	0.334	-2.896	-1.903	-0.332	3.011	1.978	80.461	14.419	14.845
5693	192	30	24	P	S	68.213	-2.359	-1.879	0.817	2.476	1.849	-0.804	80.477	14.845	14.421
5694	192	30	24	P	S	70.893	0.418	2.761	-1.983	-0.415	-2.875	2.064	80.408	14.426	14.841
5695	192	30	24	P	S	109.107	0.418	-2.761	-1.983	-0.415	2.875	2.064	80.408	14.426	14.841
5696	192	30	24	P	S	111.052	1.048	0.565	-2.575	-1.026	-0.595	2.715	69.944	17.420	12.903
5697	192	30	24	P	S	111.787	-2.359	1.879	0.817	2.476	-1.849	-0.804	80.477	14.845	14.421
5698	192	30	24	P	S	112.411	0.334	2.896	-1.903	-0.332	-3.011	1.978	80.461	14.845	14.419
5699	192	30	24	P	W	10.893	-1.983	2.830	0.418	2.064	-2.806	-0.415	69.882	12.906	17.420
5700	192	30	24	P	W	68.948	1.048	-0.565	-2.575	-1.026	0.595	2.714	69.944	17.420	12.903
5701	193	28	27	C	S	10.893	4.020	0.000	4.020	-3.721	0.000	-3.721	60.000	15.815	15.815
5702	193	28	27	C	S	49.107	4.020	0.000	4.020	-3.721	0.000	-3.721	60.000	15.815	15.815
5703	193	28	27	C	S	70.893	4.020	0.000	4.020	-3.721	0.000	-3.721	60.000	15.815	15.815
5704	193	28	27	C	S	109.107	4.020	0.000	4.020	-3.721	0.000	-3.721	60.000	15.815	15.815
5705	193	28	27	C	W	10.893	4.020	0.000	4.020	-3.721	0.000	-3.721	60.000	15.815	15.815
5706	193	28	27	C	W	49.107	4.020	0.000	4.020	-3.721	0.000	-3.721	60.000	15.815	15.815
5707	193	28	27	C	W	70.893	4.020	0.000	4.020	-3.721	0.000	-3.721	60.000	15.815	15.815
5708	193	28	27	C	W	109.107	4.020	0.000	4.020	-3.721	0.000	-3.721	60.000	15.815	15.815
5709	193	31	23	C	S	22.689	-4.497	-1.505	-0.658	4.941	1.525	0.666	81.694	11.961	17.766
5710	193	31	23	C	S	25.050	-0.955	2.499	-4.221	0.973	-2.730	4.610	81.624	17.772	11.958
5711	193	31	23	C	S	34.950	-0.955	-2.499	-4.221	0.973	2.730	4.610	81.624	17.772	11.958
5712	193	31	23	C	S	37.311	-4.497	1.505	-0.658	4.941	-1.525	0.666	81.694	11.961	17.766

BL number	Atoms	γ-PC unit cells	WS_2 unit cells	γ-PC origin atom	WS_2 origin atom	Twist-angle (°)	γ-PC strain 1 (%)	γ-PC strain 2 (%)	γ-PC strain 3 (%)	WS_2 strain 1 (%)	WS_2 strain 2 (%)	WS_2 strain 3 (%)	γ (°)	|**a**|(Å)	|**b**|(Å)
5713	193	31	23	C	S	82.689	-4.497	-1.505	-0.658	4.941	1.525	0.666	81.694	11.961	17.766
5714	193	31	23	C	S	85.050	-0.955	2.499	-4.221	0.973	-2.730	4.610	81.624	17.772	11.958
5715	193	31	23	C	S	94.950	-0.955	-2.499	-4.221	0.973	2.730	4.610	81.624	17.772	11.958
5716	193	31	23	C	S	97.311	-4.497	1.505	-0.658	4.941	-1.525	0.666	81.694	11.961	17.766
5717	193	31	23	C	W	22.689	-4.497	-1.505	-0.658	4.941	1.525	0.666	81.694	11.961	17.766
5718	193	31	23	C	W	25.050	-0.955	2.499	-4.221	0.973	-2.730	4.610	81.624	17.772	11.958
5719	193	31	23	C	W	34.950	-0.955	-2.499	-4.221	0.973	2.730	4.610	81.624	17.772	11.958
5720	193	31	23	C	W	37.311	-4.497	1.505	-0.658	4.941	-1.525	0.666	81.694	11.961	17.766
5721	193	31	23	C	W	82.689	-4.497	-1.505	-0.658	4.941	1.525	0.666	81.694	11.961	17.766
5722	193	31	23	C	W	85.050	-0.955	2.499	-4.221	0.973	-2.730	4.610	81.624	17.772	11.958
5723	193	31	23	C	W	94.950	-0.955	-2.499	-4.221	0.973	2.730	4.610	81.624	17.772	11.958
5724	193	31	23	C	W	97.311	-4.497	1.505	-0.658	4.941	-1.525	0.666	81.694	11.961	17.766
5725	193	31	23	P	S	22.689	-4.497	-1.505	-0.658	4.941	1.525	0.666	81.694	11.961	17.766
5726	193	31	23	P	S	25.050	-0.955	2.499	-4.221	0.973	-2.730	4.610	81.624	17.772	11.958
5727	193	31	23	P	S	34.950	-0.955	-2.499	-4.221	0.973	2.730	4.610	81.624	17.772	11.958
5728	193	31	23	P	S	37.311	-4.497	1.505	-0.658	4.941	-1.525	0.666	81.694	11.961	17.766
5729	193	31	23	P	S	82.689	-4.497	-1.505	-0.658	4.941	1.525	0.666	81.694	11.961	17.766
5730	193	31	23	P	S	85.050	-0.955	2.499	-4.221	0.973	-2.730	4.610	81.624	17.772	11.958
5731	193	31	23	P	S	94.950	-0.955	-2.499	-4.221	0.973	2.730	4.610	81.624	17.772	11.958
5732	193	31	23	P	S	97.311	-4.497	1.505	-0.658	4.941	-1.525	0.666	81.694	11.961	17.766
5733	193	31	23	P	W	22.689	-4.497	-1.505	-0.658	4.941	1.525	0.666	81.694	11.961	17.766
5734	193	31	23	P	W	25.050	-0.955	2.499	-4.221	0.973	-2.730	4.610	81.624	17.772	11.958
5735	193	31	23	P	W	34.950	-0.955	-2.499	-4.221	0.973	2.730	4.610	81.624	17.772	11.958
5736	193	31	23	P	W	37.311	-4.497	1.505	-0.658	4.941	-1.525	0.666	81.694	11.961	17.766
5737	193	31	23	P	W	82.689	-4.497	-1.505	-0.658	4.941	1.525	0.666	81.694	11.961	17.766
5738	193	31	23	P	W	85.050	-0.955	2.499	-4.221	0.973	-2.730	4.610	81.624	17.772	11.958
5739	193	31	23	P	W	94.950	-0.955	-2.499	-4.221	0.973	2.730	4.610	81.624	17.772	11.958
5740	193	31	23	P	W	97.311	-4.497	1.505	-0.658	4.941	-1.525	0.666	81.694	11.961	17.766
5741	194	29	26	C	S	25.693	2.326	-2.091	1.851	-2.223	2.016	-1.785	78.660	13.473	16.357
5742	194	29	26	C	S	27.126	1.029	0.462	3.169	-1.008	-0.434	-2.980	78.635	13.472	16.361
5743	194	29	26	C	S	28.055	2.281	2.109	1.895	-2.182	-2.032	-1.826	78.660	16.362	13.469
5744	194	29	26	C	S	31.945	2.281	-2.109	1.895	-2.182	2.032	-1.826	78.659	16.362	13.469
5745	194	29	26	C	S	32.874	1.029	-0.462	3.169	-1.008	0.434	-2.980	78.635	16.361	13.472
5746	194	29	26	C	S	34.307	2.326	2.091	1.851	-2.223	-2.016	-1.785	78.660	13.473	16.357
5747	194	29	26	C	S	85.694	2.326	-2.091	1.851	-2.223	2.016	-1.785	78.660	13.473	16.357
5748	194	29	26	C	S	87.126	1.029	0.462	3.169	-1.008	-0.434	-2.980	78.635	13.472	16.361
5749	194	29	26	C	S	88.055	2.281	2.109	1.895	-2.182	-2.032	-1.826	78.660	16.362	13.469
5750	194	29	26	C	S	91.945	2.281	-2.109	1.895	-2.182	2.032	-1.826	78.660	16.362	13.469
5751	194	29	26	C	S	92.874	1.029	-0.462	3.169	-1.008	0.434	-2.980	78.635	16.361	13.472
5752	194	29	26	C	S	94.307	2.326	2.091	1.851	-2.223	-2.016	-1.785	78.660	13.473	16.357
5753	194	29	26	C	W	25.693	2.326	-2.091	1.851	-2.223	2.016	-1.785	78.660	13.473	16.357
5754	194	29	26	C	W	27.126	1.029	0.462	3.169	-1.008	-0.434	-2.980	78.635	13.472	16.361
5755	194	29	26	C	W	28.055	2.281	2.109	1.895	-2.182	-2.032	-1.826	78.660	16.362	13.469
5756	194	29	26	C	W	31.945	2.281	-2.109	1.895	-2.182	2.032	-1.826	78.659	16.362	13.469
5757	194	29	26	C	W	32.874	1.029	-0.462	3.169	-1.008	0.434	-2.980	78.635	16.361	13.472
5758	194	29	26	C	W	34.307	2.326	2.091	1.851	-2.223	-2.016	-1.785	78.660	13.473	16.357
5759	194	29	26	C	W	85.694	2.326	-2.091	1.851	-2.223	2.016	-1.785	78.660	13.473	16.357
5760	194	29	26	C	W	87.126	1.029	0.462	3.169	-1.008	-0.434	-2.980	78.635	13.472	16.361
5761	194	29	26	C	W	88.055	2.281	2.109	1.895	-2.182	-2.032	-1.826	78.660	16.362	13.469
5762	194	29	26	C	W	91.945	2.281	-2.109	1.895	-2.182	2.032	-1.826	78.660	16.362	13.469
5763	194	29	26	C	W	92.874	1.029	-0.462	3.169	-1.008	0.434	-2.980	78.635	16.361	13.472

BL number	Atoms	γ-PC unit cells	WS_2 unit cells	γ-PC origin atom	WS_2 origin atom	Twist-angle (°)	γ-PC strain 1 (%)	γ-PC strain 2 (%)	γ-PC strain 3 (%)	WS_2 strain 1 (%)	WS_2 strain 2 (%)	WS_2 strain 3 (%)	γ (°)	|**a**|(Å)	|**b**|(Å)
5764	194	29	26	C	W	94.307	2.326	2.091	1.851	-2.223	-2.016	-1.785	78.660	13.473	16.357
5765	194	29	26	P	S	25.693	2.326	-2.091	1.851	-2.223	2.016	-1.785	78.660	13.473	16.357
5766	194	29	26	P	S	27.126	1.029	0.462	3.169	-1.008	-0.434	-2.980	78.635	13.472	16.361
5767	194	29	26	P	S	28.055	2.281	2.109	1.895	-2.182	-2.032	-1.826	78.660	16.362	13.469
5768	194	29	26	P	S	31.945	2.281	-2.109	1.895	-2.182	2.032	-1.826	78.659	16.362	13.469
5769	194	29	26	P	S	32.874	1.029	-0.462	3.169	-1.008	0.434	-2.980	78.635	16.361	13.472
5770	194	29	26	P	S	34.307	2.326	2.091	1.851	-2.223	-2.016	-1.785	78.660	13.473	16.357
5771	194	29	26	P	S	85.694	2.326	-2.091	1.851	-2.223	2.016	-1.785	78.660	13.473	16.357
5772	194	29	26	P	S	87.126	1.029	0.462	3.169	-1.008	-0.434	-2.980	78.635	13.472	16.361
5773	194	29	26	P	S	88.055	2.281	2.109	1.895	-2.182	-2.032	-1.826	78.660	16.362	13.469
5774	194	29	26	P	S	91.945	2.281	-2.109	1.895	-2.182	2.032	-1.826	78.660	16.362	13.469
5775	194	29	26	P	S	92.874	1.029	-0.462	3.169	-1.008	0.434	-2.980	78.635	16.361	13.472
5776	194	29	26	P	S	94.307	2.326	2.091	1.851	-2.223	-2.016	-1.785	78.660	13.473	16.357
5777	194	29	26	P	W	25.693	2.326	-2.091	1.851	-2.223	2.016	-1.785	78.660	13.473	16.357
5778	194	29	26	P	W	27.126	1.029	0.462	3.169	-1.008	-0.434	-2.980	78.635	13.472	16.361
5779	194	29	26	P	W	28.055	2.281	2.109	1.895	-2.182	-2.032	-1.826	78.660	16.362	13.469
5780	194	29	26	P	W	31.945	2.281	-2.109	1.895	-2.182	2.032	-1.826	78.659	16.362	13.469
5781	194	29	26	P	W	32.874	1.029	-0.462	3.169	-1.008	0.434	-2.980	78.635	16.361	13.472
5782	194	29	26	P	W	34.307	2.326	2.091	1.851	-2.223	-2.016	-1.785	78.660	13.473	16.357
5783	194	29	26	P	W	85.694	2.326	-2.091	1.851	-2.223	2.016	-1.785	78.660	13.473	16.357
5784	194	29	26	P	W	87.126	1.029	0.462	3.169	-1.008	-0.434	-2.980	78.635	13.472	16.361
5785	194	29	26	P	W	88.055	2.281	2.109	1.895	-2.182	-2.032	-1.826	78.660	16.362	13.469
5786	194	29	26	P	W	91.945	2.281	-2.109	1.895	-2.182	2.032	-1.826	78.660	16.362	13.469
5787	194	29	26	P	W	92.874	1.029	-0.462	3.169	-1.008	0.434	-2.980	78.635	16.361	13.472
5788	194	29	26	P	W	94.307	2.326	2.091	1.851	-2.223	-2.016	-1.785	78.660	13.473	16.357
5789	194	32	22	C	S	1.945	-4.723	-1.965	-4.049	5.216	2.138	4.406	71.333	15.242	14.484
5790	194	32	22	C	S	4.307	-4.685	1.991	-4.088	5.169	-2.168	4.452	71.333	14.491	15.234
5791	194	32	22	C	S	55.693	-4.685	-1.991	-4.088	5.169	2.168	4.452	71.333	14.491	15.234
5792	194	32	22	C	S	58.055	-4.723	1.965	-4.049	5.216	-2.138	4.406	71.333	15.242	14.484
5793	194	32	22	C	S	61.945	-4.723	-1.965	-4.049	5.216	2.138	4.406	71.333	15.242	14.484
5794	194	32	22	C	S	64.307	-4.685	1.991	-4.088	5.169	-2.168	4.452	71.333	14.491	15.234
5795	194	32	22	C	S	115.693	-4.685	-1.991	-4.088	5.169	2.168	4.452	71.333	14.491	15.234
5796	194	32	22	C	S	118.055	-4.723	1.965	-4.049	5.216	-2.138	4.406	71.333	15.242	14.484
5797	194	32	22	C	W	1.945	-4.723	-1.965	-4.049	5.216	2.138	4.406	71.333	15.242	14.484
5798	194	32	22	C	W	4.307	-4.685	1.991	-4.088	5.169	-2.168	4.452	71.333	14.491	15.234
5799	194	32	22	C	W	55.693	-4.685	-1.991	-4.088	5.169	2.168	4.452	71.333	14.491	15.234
5800	194	32	22	C	W	58.055	-4.723	1.965	-4.049	5.216	-2.138	4.406	71.333	15.242	14.484
5801	194	32	22	C	W	61.945	-4.723	-1.965	-4.049	5.216	2.138	4.406	71.333	15.242	14.484
5802	194	32	22	C	W	64.307	-4.685	1.991	-4.088	5.169	-2.168	4.452	71.333	14.491	15.234
5803	194	32	22	C	W	115.693	-4.685	-1.991	-4.088	5.169	2.168	4.452	71.333	14.491	15.234
5804	194	32	22	C	W	118.055	-4.723	1.965	-4.049	5.216	-2.138	4.406	71.333	15.242	14.484
5805	194	32	22	P	S	1.945	-4.723	-1.965	-4.049	5.216	2.138	4.406	71.333	15.242	14.484
5806	194	32	22	P	S	4.307	-4.685	1.991	-4.088	5.169	-2.168	4.452	71.333	14.491	15.234
5807	194	32	22	P	S	55.693	-4.685	-1.991	-4.088	5.169	2.168	4.452	71.333	14.491	15.234
5808	194	32	22	P	S	58.055	-4.723	1.965	-4.049	5.216	-2.138	4.406	71.333	15.242	14.484
5809	194	32	22	P	S	61.945	-4.723	-1.965	-4.049	5.216	2.138	4.406	71.333	15.242	14.484
5810	194	32	22	P	S	64.307	-4.685	1.991	-4.088	5.169	-2.168	4.452	71.333	14.491	15.234
5811	194	32	22	P	S	115.693	-4.685	-1.991	-4.088	5.169	2.168	4.452	71.333	14.491	15.234
5812	194	32	22	P	S	118.055	-4.723	1.965	-4.049	5.216	-2.138	4.406	71.333	15.242	14.484
5813	194	32	22	P	W	1.945	-4.723	-1.965	-4.049	5.216	2.138	4.406	71.333	15.242	14.484
5814	194	32	22	P	W	4.307	-4.685	1.991	-4.088	5.169	-2.168	4.452	71.333	14.491	15.234

BL number	Atoms	γ-PC unit cells	WS_2 unit cells	γ-PC origin atom	WS_2 origin atom	Twist-angle (°)	γ-PC strain 1 (%)	γ-PC strain 2 (%)	γ-PC strain 3 (%)	WS_2 strain 1 (%)	WS_2 strain 2 (%)	WS_2 strain 3 (%)	γ (°)	$\lvert\mathbf{a}\rvert$(Å)	$\lvert\mathbf{b}\rvert$(Å)
5815	194	32	22	P	W	55.693	-4.685	-1.991	-4.088	5.169	2.168	4.452	71.333	14.491	15.234
5816	194	32	22	P	W	58.055	-4.723	1.965	-4.049	5.216	-2.138	4.406	71.333	15.242	14.484
5817	194	32	22	P	W	61.945	-4.723	-1.965	-4.049	5.216	2.138	4.406	71.333	15.242	14.484
5818	194	32	22	P	W	64.307	-4.685	1.991	-4.088	5.169	-2.168	4.452	71.333	14.491	15.234
5819	194	32	22	P	W	115.693	-4.685	-1.991	-4.088	5.169	2.168	4.452	71.333	14.491	15.234
5820	194	32	22	P	W	118.055	-4.723	1.965	-4.049	5.216	-2.138	4.406	71.333	15.242	14.484
5821	195	30	25	C	S	0.000	5.011	3.031	-4.158	-4.554	-3.306	4.535	70.816	13.826	16.530
5822	195	30	25	C	S	8.948	-0.599	-1.339	1.048	0.606	1.312	-1.026	69.929	15.901	14.424
5823	195	30	25	C	S	10.893	0.418	2.071	0.018	-0.415	-2.070	-0.018	69.911	14.426	15.900
5824	195	30	25	C	S	16.337	0.353	-3.645	0.083	-0.351	3.639	-0.083	70.942	15.499	14.704
5825	195	30	25	C	S	16.537	-3.292	2.371	3.991	3.523	-2.196	-3.696	74.959	14.403	15.507
5826	195	30	25	C	S	19.107	-1.485	1.219	1.980	1.530	-1.172	-1.905	70.884	14.708	15.505
5827	195	30	25	C	S	23.413	-2.043	3.225	2.585	2.130	-3.066	-2.458	81.338	14.072	15.492
5828	195	30	25	C	S	23.691	2.390	3.562	-1.864	-2.281	-3.700	1.936	81.313	14.078	15.486
5829	195	30	25	C	S	30.000	-2.359	1.879	2.934	2.476	-1.775	-2.772	70.871	14.845	15.370
5830	195	30	25	C	S	36.309	2.390	-3.562	-1.864	-2.281	3.700	1.936	81.313	15.486	14.078
5831	195	30	25	C	S	36.587	-2.043	-3.225	2.585	2.130	3.066	-2.458	81.338	14.072	15.492
5832	195	30	25	C	S	40.893	1.980	4.318	-1.485	-1.905	-4.450	1.530	81.167	15.504	14.064
5833	195	30	25	C	S	43.463	-3.292	-2.371	3.991	3.523	2.196	-3.696	74.959	15.507	14.403
5834	195	30	25	C	S	43.663	0.353	3.645	0.083	-0.351	-3.639	-0.083	70.942	14.704	15.499
5835	195	30	25	C	S	49.107	0.418	-2.071	0.018	-0.415	2.070	-0.018	69.911	14.426	15.900
5836	195	30	25	C	S	51.052	-0.599	1.339	1.048	0.606	-1.312	-1.026	69.929	15.901	14.424
5837	195	30	25	C	S	60.000	5.011	3.031	-4.158	-4.554	-3.306	4.535	70.816	13.826	16.530
5838	195	30	25	C	S	68.948	-0.599	-1.339	1.048	0.606	1.312	-1.026	69.929	15.901	14.424
5839	195	30	25	C	S	70.893	0.418	2.071	0.018	-0.415	-2.070	-0.018	69.911	14.426	15.900
5840	195	30	25	C	S	76.337	0.353	-3.645	0.083	-0.351	3.639	-0.083	70.942	15.499	14.704
5841	195	30	25	C	S	76.537	-3.292	2.371	3.991	3.523	-2.196	-3.696	74.959	14.403	15.507
5842	195	30	25	C	S	79.107	1.980	-4.318	-1.485	-1.905	4.450	1.530	81.167	15.504	14.064
5843	195	30	25	C	S	83.413	-2.043	3.225	2.585	2.130	-3.066	-2.458	81.338	14.072	15.492
5844	195	30	25	C	S	83.691	2.390	3.562	-1.864	-2.281	-3.700	1.936	81.313	14.078	15.486
5845	195	30	25	C	S	90.000	2.934	-1.783	-2.359	-2.772	1.871	2.476	70.867	15.368	14.847
5846	195	30	25	C	S	96.309	2.390	-3.562	-1.864	-2.281	3.700	1.936	81.313	15.486	14.078
5847	195	30	25	C	S	96.587	-2.043	-3.225	2.585	2.130	3.066	-2.458	81.339	14.072	15.492
5848	195	30	25	C	S	100.893	1.980	-1.178	-1.485	-1.905	1.213	1.530	70.882	15.504	14.709
5849	195	30	25	C	S	103.463	-3.292	-2.371	3.991	3.523	2.196	-3.696	74.959	15.507	14.403
5850	195	30	25	C	S	103.663	0.353	3.645	0.083	-0.351	-3.639	-0.083	70.942	14.704	15.499
5851	195	30	25	C	S	109.107	0.418	-2.071	0.018	-0.415	2.070	-0.018	69.911	14.426	15.900
5852	195	30	25	C	S	111.052	-0.599	1.339	1.048	0.606	-1.312	-1.026	69.929	15.901	14.424
5853	195	30	25	C	W	0.000	5.011	3.031	-4.158	-4.554	-3.306	4.535	70.816	13.826	16.530
5854	195	30	25	C	W	8.948	-0.599	-1.339	1.048	0.606	1.312	-1.026	69.929	15.901	14.424
5855	195	30	25	C	W	10.893	0.418	2.071	0.018	-0.415	-2.070	-0.018	69.911	14.426	15.900
5856	195	30	25	C	W	16.537	-3.292	2.371	3.991	3.523	-2.196	-3.696	74.959	14.403	15.507
5857	195	30	25	C	W	19.107	1.980	-4.318	-1.485	-1.905	4.450	1.530	81.167	15.504	14.064
5858	195	30	25	C	W	23.413	-2.043	3.225	2.585	2.130	-3.066	-2.458	81.338	14.072	15.492
5859	195	30	25	C	W	23.691	2.390	3.562	-1.864	-2.281	-3.700	1.936	81.313	14.078	15.486
5860	195	30	25	C	W	30.000	-2.359	1.879	2.934	2.476	-1.775	-2.772	70.871	14.845	15.370
5861	195	30	25	C	W	36.309	2.390	-3.562	-1.864	-2.281	3.700	1.936	81.313	15.486	14.078
5862	195	30	25	C	W	36.587	-2.043	-3.225	2.585	2.130	3.066	-2.458	81.338	14.072	15.492
5863	195	30	25	C	W	40.893	1.980	4.318	-1.485	-1.905	-4.450	1.530	81.167	15.504	14.064
5864	195	30	25	C	W	43.463	-3.292	-2.371	3.991	3.523	2.196	-3.696	74.959	15.507	14.403
5865	195	30	25	C	W	49.107	0.418	-2.071	0.018	-0.415	2.070	-0.018	69.911	14.426	15.900

BL number	Atoms	γ-PC unit cells	WS_2 unit cells	γ-PC origin atom	WS_2 origin atom	Twist-angle (°)	γ-PC strain 1 (%)	γ-PC strain 2 (%)	γ-PC strain 3 (%)	WS_2 strain 1 (%)	WS_2 strain 2 (%)	WS_2 strain 3 (%)	γ (°)	|**a**|(Å)	|**b**|(Å)
5866	195	30	25	C	W	51.052	-0.599	1.339	1.048	0.606	-1.312	-1.026	69.929	15.901	14.424
5867	195	30	25	C	W	60.000	5.011	3.031	-4.158	-4.554	-3.306	4.535	70.816	13.826	16.530
5868	195	30	25	C	W	68.948	-0.599	-1.339	1.048	0.606	1.312	-1.026	69.929	15.901	14.424
5869	195	30	25	C	W	70.893	0.418	2.071	0.018	-0.415	-2.070	-0.018	69.911	14.426	15.900
5870	195	30	25	C	W	76.537	-3.292	2.371	3.991	3.523	-2.196	-3.696	74.959	14.403	15.507
5871	195	30	25	C	W	79.107	1.980	-4.318	-1.485	-1.905	4.450	1.530	81.167	15.504	14.064
5872	195	30	25	C	W	83.413	-2.043	3.225	2.585	2.130	-3.066	-2.458	81.338	14.072	15.492
5873	195	30	25	C	W	83.691	2.390	3.562	-1.864	-2.281	-3.700	1.936	81.313	14.078	15.486
5874	195	30	25	C	W	90.000	2.934	-1.783	-2.359	-2.772	1.871	2.476	70.867	15.368	14.847
5875	195	30	25	C	W	96.309	2.390	-3.562	-1.864	-2.281	3.700	1.936	81.313	15.486	14.078
5876	195	30	25	C	W	96.587	-2.043	-3.225	2.585	2.130	3.066	-2.458	81.339	14.072	15.492
5877	195	30	25	C	W	100.893	1.980	4.318	-1.485	-1.905	-4.450	1.530	81.167	15.504	14.064
5878	195	30	25	C	W	103.463	-3.292	-2.371	3.991	3.523	2.196	-3.696	74.959	15.507	14.403
5879	195	30	25	C	W	109.107	0.418	-2.071	0.018	-0.415	2.070	-0.018	69.911	14.426	15.900
5880	195	30	25	C	W	111.052	-0.599	1.339	1.048	0.606	-1.312	-1.026	69.929	15.901	14.424
5881	195	30	25	P	S	0.000	5.011	3.031	-4.158	-4.554	-3.306	4.535	70.816	13.826	16.530
5882	195	30	25	P	S	8.948	-0.599	-1.339	1.048	0.606	1.312	-1.026	69.929	15.901	14.424
5883	195	30	25	P	S	10.893	0.418	2.071	0.018	-0.415	-2.070	-0.018	69.911	14.426	15.900
5884	195	30	25	P	S	16.337	0.353	-3.645	0.083	-0.351	3.639	-0.083	70.942	15.499	14.704
5885	195	30	25	P	S	16.537	-3.292	2.371	3.991	3.523	-2.196	-3.696	74.959	14.403	15.507
5886	195	30	25	P	S	19.107	-1.485	1.219	1.980	1.530	-1.172	-1.905	70.884	14.708	15.505
5887	195	30	25	P	S	23.413	-2.043	3.225	2.585	2.130	-3.066	-2.458	81.338	14.072	15.492
5888	195	30	25	P	S	23.691	2.390	3.562	-1.864	-2.281	-3.700	1.936	81.313	14.078	15.486
5889	195	30	25	P	S	30.000	-2.359	1.879	2.934	2.476	-1.775	-2.772	70.871	14.845	15.370
5890	195	30	25	P	S	36.309	2.390	-3.562	-1.864	-2.281	3.700	1.936	81.313	15.486	14.078
5891	195	30	25	P	S	36.587	-2.043	-3.225	2.585	2.130	3.066	-2.458	81.338	14.072	15.492
5892	195	30	25	P	S	40.893	1.980	4.318	-1.485	-1.905	-4.450	1.530	81.167	15.504	14.064
5893	195	30	25	P	S	43.463	-3.292	-2.371	3.991	3.523	2.196	-3.696	74.959	15.507	14.403
5894	195	30	25	P	S	43.663	0.353	3.645	0.083	-0.351	-3.639	-0.083	70.942	14.704	15.499
5895	195	30	25	P	S	49.107	0.418	-2.071	0.018	-0.415	2.070	-0.018	69.911	14.426	15.900
5896	195	30	25	P	S	51.052	-0.599	1.339	1.048	0.606	-1.312	-1.026	69.929	15.901	14.424
5897	195	30	25	P	S	60.000	-4.158	-1.107	5.011	4.534	1.006	-4.554	83.387	16.522	13.152
5898	195	30	25	P	S	68.948	-0.599	-1.339	1.048	0.606	1.312	-1.026	69.929	15.901	14.424
5899	195	30	25	P	S	70.893	0.418	2.071	0.018	-0.415	-2.070	-0.018	69.911	14.426	15.900
5900	195	30	25	P	S	76.337	0.353	-3.645	0.083	-0.351	3.639	-0.083	70.942	15.499	14.704
5901	195	30	25	P	S	76.537	-3.292	2.371	3.991	3.523	-2.196	-3.696	74.959	14.403	15.507
5902	195	30	25	P	S	79.107	1.980	-4.318	-1.485	-1.905	4.450	1.530	81.167	15.504	14.064
5903	195	30	25	P	S	83.413	-2.043	3.225	2.585	2.130	-3.066	-2.458	81.338	14.072	15.492
5904	195	30	25	P	S	83.691	2.390	3.562	-1.864	-2.281	-3.700	1.936	81.313	14.078	15.486
5905	195	30	25	P	S	90.000	2.934	-1.783	-2.359	-2.772	1.871	2.476	70.867	15.368	14.847
5906	195	30	25	P	S	96.309	2.390	-3.562	-1.864	-2.281	3.700	1.936	81.313	15.486	14.078
5907	195	30	25	P	S	96.587	-2.043	-3.225	2.585	2.130	3.066	-2.458	81.339	14.072	15.492
5908	195	30	25	P	S	100.893	1.980	-1.178	-1.485	-1.905	1.213	1.530	70.882	15.504	14.709
5909	195	30	25	P	S	103.463	-3.292	-2.371	3.991	3.523	2.196	-3.696	74.959	15.507	14.403
5910	195	30	25	P	S	103.663	0.353	3.645	0.083	-0.351	-3.639	-0.083	70.942	14.704	15.499
5911	195	30	25	P	S	109.107	0.418	-2.071	0.018	-0.415	2.070	-0.018	69.911	14.426	15.900
5912	195	30	25	P	S	111.052	-0.599	1.339	1.048	0.606	-1.312	-1.026	69.929	15.901	14.424
5913	195	30	25	P	W	0.000	5.011	3.031	-4.158	-4.554	-3.306	4.535	70.816	13.826	16.530
5914	195	30	25	P	W	8.948	-0.599	-1.339	1.048	0.606	1.312	-1.026	69.929	15.901	14.424
5915	195	30	25	P	W	10.893	0.418	2.071	0.018	-0.415	-2.070	-0.018	69.911	14.426	15.900
5916	195	30	25	P	W	16.537	-3.292	2.371	3.991	3.523	-2.196	-3.696	74.959	14.403	15.507

BL number	Atoms	γ-PC unit cells	WS_2 unit cells	γ-PC origin atom	WS_2 origin atom	Twist-angle (°)	γ-PC strain 1 (%)	γ-PC strain 2 (%)	γ-PC strain 3 (%)	WS_2 strain 1 (%)	WS_2 strain 2 (%)	WS_2 strain 3 (%)	γ (°)	$\lvert\mathbf{a}\rvert$(Å)	$\lvert\mathbf{b}\rvert$(Å)
5917	195	30	25	P	W	19.107	1.980	-4.318	-1.485	-1.905	4.450	1.530	81.167	15.504	14.064
5918	195	30	25	P	W	23.413	-2.043	3.225	2.585	2.130	-3.066	-2.458	81.338	14.072	15.492
5919	195	30	25	P	W	23.691	2.390	3.562	-1.864	-2.281	-3.700	1.936	81.313	14.078	15.486
5920	195	30	25	P	W	30.000	-2.359	1.879	2.934	2.476	-1.775	-2.772	70.871	14.845	15.370
5921	195	30	25	P	W	36.309	2.390	-3.562	-1.864	-2.281	3.700	1.936	81.313	15.486	14.078
5922	195	30	25	P	W	36.587	-2.043	-3.225	2.585	2.130	3.066	-2.458	81.338	14.072	15.492
5923	195	30	25	P	W	40.893	1.980	4.318	-1.485	-1.905	-4.450	1.530	81.167	15.504	14.064
5924	195	30	25	P	W	43.463	-3.292	-2.371	3.991	3.523	2.196	-3.696	74.959	15.507	14.403
5925	195	30	25	P	W	49.107	0.418	-2.071	0.018	-0.415	2.070	-0.018	69.911	14.426	15.900
5926	195	30	25	P	W	51.052	-0.599	1.339	1.048	0.606	-1.312	-1.026	69.929	15.901	14.424
5927	195	30	25	P	W	60.000	5.011	3.031	-4.158	-4.554	-3.306	4.535	70.816	13.826	16.530
5928	195	30	25	P	W	68.948	-0.599	-1.339	1.048	0.606	1.312	-1.026	69.929	15.901	14.424
5929	195	30	25	P	W	70.893	0.418	2.071	0.018	-0.415	-2.070	-0.018	69.911	14.426	15.900
5930	195	30	25	P	W	76.537	-3.292	2.371	3.991	3.523	-2.196	-3.696	74.959	14.403	15.507
5931	195	30	25	P	W	79.107	1.980	-4.318	-1.485	-1.905	4.450	1.530	81.167	15.504	14.064
5932	195	30	25	P	W	83.413	-2.043	3.225	2.585	2.130	-3.066	-2.458	81.338	14.072	15.492
5933	195	30	25	P	W	83.691	2.390	3.562	-1.864	-2.281	-3.700	1.936	81.313	14.078	15.486
5934	195	30	25	P	W	90.000	2.934	-1.783	-2.359	-2.772	1.871	2.476	70.867	15.368	14.847
5935	195	30	25	P	W	96.309	2.390	-3.562	-1.864	-2.281	3.700	1.936	81.313	15.486	14.078
5936	195	30	25	P	W	96.587	-2.043	-3.225	2.585	2.130	3.066	-2.458	81.339	14.072	15.492
5937	195	30	25	P	W	100.893	1.980	4.318	-1.485	-1.905	-4.450	1.530	81.167	15.504	14.064
5938	195	30	25	P	W	103.463	-3.292	-2.371	3.991	3.523	2.196	-3.696	74.959	15.507	14.403
5939	195	30	25	P	W	109.107	0.418	-2.071	0.018	-0.415	2.070	-0.018	69.911	14.426	15.900
5940	195	30	25	P	W	111.052	-0.599	1.339	1.048	0.606	-1.312	-1.026	69.929	15.901	14.424
5941	196	31	24	C	S	6.178	-2.145	-4.036	-1.042	2.241	4.121	1.064	76.750	17.102	12.892
5942	196	31	24	C	S	7.589	0.334	-1.557	-3.454	-0.332	1.673	3.710	76.699	12.900	17.101
5943	196	31	24	C	S	10.893	-1.983	4.107	-1.208	2.064	-4.209	1.238	76.743	12.906	17.084
5944	196	31	24	C	S	16.337	0.353	3.828	-3.472	-0.351	-4.114	3.730	71.732	17.539	12.890
5945	196	31	24	C	S	16.537	-3.292	4.328	0.159	3.523	-4.315	-0.159	71.695	12.883	17.551
5946	196	31	24	C	S	23.413	0.481	-0.468	-3.590	-0.477	0.504	3.867	79.821	12.584	17.334
5947	196	31	24	C	S	24.791	-3.388	1.928	0.263	3.635	-1.918	-0.262	79.755	17.335	12.585
5948	196	31	24	C	S	35.209	-3.388	-1.928	0.263	3.635	1.918	-0.262	79.755	17.335	12.585
5949	196	31	24	C	S	36.587	0.481	0.468	-3.590	-0.477	-0.504	3.867	79.821	12.584	17.334
5950	196	31	24	C	S	43.463	-3.292	-4.328	0.159	3.523	4.315	-0.159	71.695	17.551	12.883
5951	196	31	24	C	S	43.663	0.353	-3.828	-3.472	-0.351	4.114	3.730	71.732	17.539	12.890
5952	196	31	24	C	S	49.107	-1.983	-4.107	-1.208	2.064	4.209	1.238	76.743	12.906	17.084
5953	196	31	24	C	S	52.411	0.334	1.557	-3.454	-0.332	-1.673	3.710	76.699	17.101	12.900
5954	196	31	24	C	S	53.822	-2.145	4.036	-1.042	2.241	-4.122	1.064	76.750	17.102	12.892
5955	196	31	24	C	S	66.178	-2.145	-4.036	-1.042	2.241	4.121	1.064	76.750	17.102	12.892
5956	196	31	24	C	S	67.589	0.334	-1.557	-3.454	-0.332	1.673	3.710	76.699	12.900	17.101
5957	196	31	24	C	S	70.893	-1.983	4.107	-1.208	2.064	-4.209	1.238	76.743	12.906	17.084
5958	196	31	24	C	S	76.337	0.353	3.828	-3.472	-0.351	-4.114	3.730	71.732	17.539	12.890
5959	196	31	24	C	S	76.537	-3.292	4.328	0.159	3.523	-4.315	-0.159	71.695	12.883	17.551
5960	196	31	24	C	S	83.413	0.481	-0.468	-3.590	-0.477	0.504	3.867	79.821	12.584	17.334
5961	196	31	24	C	S	84.791	-3.388	1.928	0.263	3.635	-1.918	-0.262	79.755	17.335	12.585
5962	196	31	24	C	S	95.209	-3.388	-1.928	0.263	3.635	1.918	-0.262	79.755	17.335	12.585
5963	196	31	24	C	S	96.587	0.481	0.468	-3.590	-0.477	-0.504	3.867	79.821	12.584	17.334
5964	196	31	24	C	S	103.463	-3.292	-4.328	0.159	3.523	4.315	-0.159	71.695	17.551	12.883
5965	196	31	24	C	S	103.663	0.353	-3.828	-3.472	-0.351	4.114	3.730	71.732	17.539	12.890
5966	196	31	24	C	S	109.107	-1.983	-4.107	-1.208	2.064	4.209	1.238	76.743	12.906	17.084
5967	196	31	24	C	S	112.411	0.334	1.557	-3.454	-0.332	-1.673	3.710	76.699	17.101	12.900

BL number	Atoms	γ-PC unit cells	WS_2 unit cells	γ-PC origin atom	WS_2 origin atom	Twist-angle (°)	γ-PC strain 1 (%)	γ-PC strain 2 (%)	γ-PC strain 3 (%)	WS_2 strain 1 (%)	WS_2 strain 2 (%)	WS_2 strain 3 (%)	γ (°)	|**a**|(Å)	|**b**|(Å)
5968	196	31	24	C	S	113.822	-2.145	4.036	-1.042	2.241	-4.122	1.064	76.750	17.102	12.892
5969	196	31	24	C	W	6.178	-2.145	-4.036	-1.042	2.241	4.121	1.064	76.750	17.102	12.892
5970	196	31	24	C	W	7.589	0.334	-1.557	-3.454	-0.332	1.673	3.710	76.699	12.900	17.101
5971	196	31	24	C	W	10.893	-1.983	4.107	-1.208	2.064	-4.209	1.238	76.743	12.906	17.084
5972	196	31	24	C	W	16.337	0.353	3.828	-3.472	-0.351	-4.114	3.730	71.732	17.539	12.890
5973	196	31	24	C	W	16.537	-3.292	4.328	0.159	3.523	-4.315	-0.159	71.695	12.883	17.551
5974	196	31	24	C	W	23.413	0.481	-0.468	-3.590	-0.477	0.504	3.867	79.821	12.584	17.334
5975	196	31	24	C	W	24.791	-3.388	1.928	0.263	3.635	-1.918	-0.262	79.755	17.335	12.585
5976	196	31	24	C	W	35.209	-3.388	-1.928	0.263	3.635	1.918	-0.262	79.755	17.335	12.585
5977	196	31	24	C	W	36.587	0.481	0.468	-3.590	-0.477	-0.504	3.867	79.821	12.584	17.334
5978	196	31	24	C	W	43.463	-3.292	-4.328	0.159	3.523	4.315	-0.159	71.695	17.551	12.883
5979	196	31	24	C	W	43.663	0.353	-3.828	-3.472	-0.351	4.114	3.730	71.732	17.539	12.890
5980	196	31	24	C	W	49.107	-1.983	-4.107	-1.208	2.064	4.209	1.238	76.743	12.906	17.084
5981	196	31	24	C	W	52.411	0.334	1.557	-3.454	-0.332	-1.673	3.710	76.699	17.101	12.900
5982	196	31	24	C	W	53.822	-2.145	4.036	-1.042	2.241	-4.122	1.064	76.750	17.102	12.892
5983	196	31	24	C	W	66.178	-2.145	-4.036	-1.042	2.241	4.121	1.064	76.750	17.102	12.892
5984	196	31	24	C	W	67.589	0.334	-1.557	-3.454	-0.332	1.673	3.710	76.699	12.900	17.101
5985	196	31	24	C	W	70.893	-1.983	4.107	-1.208	2.064	-4.209	1.238	76.743	12.906	17.084
5986	196	31	24	C	W	76.337	0.353	3.828	-3.472	-0.351	-4.114	3.730	71.732	17.539	12.890
5987	196	31	24	C	W	76.537	-3.292	4.328	0.159	3.523	-4.315	-0.159	71.695	12.883	17.551
5988	196	31	24	C	W	83.413	0.481	-0.468	-3.590	-0.477	0.504	3.867	79.821	12.584	17.334
5989	196	31	24	C	W	84.791	-3.388	1.928	0.263	3.635	-1.918	-0.262	79.755	17.335	12.585
5990	196	31	24	C	W	95.209	-3.388	-1.928	0.263	3.635	1.918	-0.262	79.755	17.335	12.585
5991	196	31	24	C	W	96.587	0.481	0.468	-3.590	-0.477	-0.504	3.867	79.821	12.584	17.334
5992	196	31	24	C	W	103.463	-3.292	-4.328	0.159	3.523	4.315	-0.159	71.695	17.551	12.883
5993	196	31	24	C	W	103.663	0.353	-3.828	-3.472	-0.351	4.114	3.730	71.732	17.539	12.890
5994	196	31	24	C	W	109.107	-1.983	-4.107	-1.208	2.064	4.209	1.238	76.743	12.906	17.084
5995	196	31	24	C	W	112.411	0.334	1.557	-3.454	-0.332	-1.673	3.710	76.699	17.101	12.900
5996	196	31	24	C	W	113.822	-2.145	4.036	-1.042	2.241	-4.122	1.064	76.750	17.102	12.892
5997	196	31	24	P	S	6.178	-2.145	-4.036	-1.042	2.241	4.121	1.064	76.750	17.102	12.892
5998	196	31	24	P	S	7.589	0.334	-1.557	-3.454	-0.332	1.673	3.710	76.699	12.900	17.101
5999	196	31	24	P	S	10.893	-1.983	4.107	-1.208	2.064	-4.209	1.238	76.743	12.906	17.084
6000	196	31	24	P	S	16.337	0.353	3.828	-3.472	-0.351	-4.114	3.730	71.732	17.539	12.890
6001	196	31	24	P	S	16.537	-3.292	4.328	0.159	3.523	-4.315	-0.159	71.695	12.883	17.551
6002	196	31	24	P	S	23.413	0.481	-0.468	-3.590	-0.477	0.504	3.867	79.821	12.584	17.334
6003	196	31	24	P	S	24.791	-3.388	1.928	0.263	3.635	-1.918	-0.262	79.755	17.335	12.585
6004	196	31	24	P	S	35.209	-3.388	-1.928	0.263	3.635	1.918	-0.262	79.755	17.335	12.585
6005	196	31	24	P	S	36.587	0.481	0.468	-3.590	-0.477	-0.504	3.867	79.821	12.584	17.334
6006	196	31	24	P	S	43.463	-3.292	-4.328	0.159	3.523	4.315	-0.159	71.695	17.551	12.883
6007	196	31	24	P	S	43.663	0.353	-3.828	-3.472	-0.351	4.114	3.730	71.732	17.539	12.890
6008	196	31	24	P	S	49.107	-1.983	-4.107	-1.208	2.064	4.209	1.238	76.743	12.906	17.084
6009	196	31	24	P	S	52.411	0.334	1.557	-3.454	-0.332	-1.673	3.710	76.699	17.101	12.900
6010	196	31	24	P	S	53.822	-2.145	4.036	-1.042	2.241	-4.122	1.064	76.750	17.102	12.892
6011	196	31	24	P	S	66.178	-2.145	-4.036	-1.042	2.241	4.121	1.064	76.750	17.102	12.892
6012	196	31	24	P	S	67.589	0.334	-1.557	-3.454	-0.332	1.673	3.710	76.699	12.900	17.101
6013	196	31	24	P	S	70.893	-1.983	4.107	-1.208	2.064	-4.209	1.238	76.743	12.906	17.084
6014	196	31	24	P	S	76.337	0.353	3.828	-3.472	-0.351	-4.114	3.730	71.732	17.539	12.890
6015	196	31	24	P	S	76.537	-3.292	4.328	0.159	3.523	-4.315	-0.159	71.695	12.883	17.551
6016	196	31	24	P	S	83.413	0.481	-0.468	-3.590	-0.477	0.504	3.867	79.821	12.584	17.334
6017	196	31	24	P	S	84.791	-3.388	1.928	0.263	3.635	-1.918	-0.262	79.755	17.335	12.585
6018	196	31	24	P	S	95.209	-3.388	-1.928	0.263	3.635	1.918	-0.262	79.755	17.335	12.585

BL number	Atoms	γ-PC unit cells	WS_2 unit cells	γ-PC origin atom	WS_2 origin atom	Twist-angle (°)	γ-PC strain 1 (%)	γ-PC strain 2 (%)	γ-PC strain 3 (%)	WS_2 strain 1 (%)	WS_2 strain 2 (%)	WS_2 strain 3 (%)	γ (°)	|**a**|(Å)	|**b**|(Å)
6019	196	31	24	P	S	96.587	0.481	0.468	-3.590	-0.477	-0.504	3.867	79.821	12.584	17.334
6020	196	31	24	P	S	103.463	-3.292	-4.328	0.159	3.523	4.315	-0.159	71.695	17.551	12.883
6021	196	31	24	P	S	103.663	0.353	-3.828	-3.472	-0.351	4.114	3.730	71.732	17.539	12.890
6022	196	31	24	P	S	109.107	-1.983	-4.107	-1.208	2.064	4.209	1.238	76.743	12.906	17.084
6023	196	31	24	P	S	112.411	0.334	1.557	-3.454	-0.332	-1.673	3.710	76.699	17.101	12.900
6024	196	31	24	P	S	113.822	-2.145	4.036	-1.042	2.241	-4.122	1.064	76.750	17.102	12.892
6025	196	31	24	P	W	6.178	-2.145	-4.036	-1.042	2.241	4.121	1.064	76.750	17.102	12.892
6026	196	31	24	P	W	7.589	0.334	-1.557	-3.454	-0.332	1.673	3.710	76.699	12.900	17.101
6027	196	31	24	P	W	10.893	-1.983	4.107	-1.208	2.064	-4.209	1.238	76.743	12.906	17.084
6028	196	31	24	P	W	16.337	0.353	3.828	-3.472	-0.351	-4.114	3.730	71.732	17.539	12.890
6029	196	31	24	P	W	16.537	-3.292	4.328	0.159	3.523	-4.315	-0.159	71.695	12.883	17.551
6030	196	31	24	P	W	23.413	0.481	-0.468	-3.590	-0.477	0.504	3.867	79.821	12.584	17.334
6031	196	31	24	P	W	24.791	-3.388	1.928	0.263	3.635	-1.918	-0.262	79.755	17.335	12.585
6032	196	31	24	P	W	35.209	-3.388	-1.928	0.263	3.635	1.918	-0.262	79.755	17.335	12.585
6033	196	31	24	P	W	36.587	0.481	0.468	-3.590	-0.477	-0.504	3.867	79.821	12.584	17.334
6034	196	31	24	P	W	43.463	-3.292	-4.328	0.159	3.523	4.315	-0.159	71.695	17.551	12.883
6035	196	31	24	P	W	43.663	0.353	-3.828	-3.472	-0.351	4.114	3.730	71.732	17.539	12.890
6036	196	31	24	P	W	49.107	-1.983	-4.107	-1.208	2.064	4.209	1.238	76.743	12.906	17.084
6037	196	31	24	P	W	52.411	0.334	1.557	-3.454	-0.332	-1.673	3.710	76.699	17.101	12.900
6038	196	31	24	P	W	53.822	-2.145	4.036	-1.042	2.241	-4.122	1.064	76.750	17.102	12.892
6039	196	31	24	P	W	66.178	-2.145	-4.036	-1.042	2.241	4.121	1.064	76.750	17.102	12.892
6040	196	31	24	P	W	67.589	0.334	-1.557	-3.454	-0.332	1.673	3.710	76.699	12.900	17.101
6041	196	31	24	P	W	70.893	-1.983	4.107	-1.208	2.064	-4.209	1.238	76.743	12.906	17.084
6042	196	31	24	P	W	76.337	0.353	3.828	-3.472	-0.351	-4.114	3.730	71.732	17.539	12.890
6043	196	31	24	P	W	76.537	-3.292	4.328	0.159	3.523	-4.315	-0.159	71.695	12.883	17.551
6044	196	31	24	P	W	83.413	0.481	-0.468	-3.590	-0.477	0.504	3.867	79.821	12.584	17.334
6045	196	31	24	P	W	84.791	-3.388	1.928	0.263	3.635	-1.918	-0.262	79.755	17.335	12.585
6046	196	31	24	P	W	95.209	-3.388	-1.928	0.263	3.635	1.918	-0.262	79.755	17.335	12.585
6047	196	31	24	P	W	96.587	0.481	0.468	-3.590	-0.477	-0.504	3.867	79.821	12.584	17.334
6048	196	31	24	P	W	103.463	-3.292	-4.328	0.159	3.523	4.315	-0.159	71.695	17.551	12.883
6049	196	31	24	P	W	103.663	0.353	-3.828	-3.472	-0.351	4.114	3.730	71.732	17.539	12.890
6050	196	31	24	P	W	109.107	-1.983	-4.107	-1.208	2.064	4.209	1.238	76.743	12.906	17.084
6051	196	31	24	P	W	112.411	0.334	1.557	-3.454	-0.332	-1.673	3.710	76.699	17.101	12.900
6052	196	31	24	P	W	113.822	-2.145	4.036	-1.042	2.241	-4.122	1.064	76.750	17.102	12.892
6053	197	29	27	C	S	12.520	2.326	-5.468	3.845	-2.223	5.078	-3.571	77.090	13.473	16.776
6054	197	29	27	C	S	13.174	5.011	-4.181	1.217	-4.554	4.082	-1.188	72.786	13.151	17.542
6055	197	29	27	C	S	17.696	1.029	3.968	5.214	-1.008	-3.594	-4.722	77.095	13.461	16.796
6056	197	29	27	C	S	17.897	5.011	4.181	1.217	-4.554	-4.082	-1.188	77.200	16.799	13.452
6057	197	29	27	C	S	21.052	1.339	-1.345	4.880	-1.304	1.226	-4.446	79.466	16.211	13.826
6058	197	29	27	C	S	23.691	2.390	3.450	3.780	-2.281	-3.207	-3.514	79.494	16.213	13.819
6059	197	29	27	C	S	36.309	2.390	-3.450	3.780	-2.281	3.207	-3.514	79.494	13.819	16.213
6060	197	29	27	C	S	42.103	5.011	-4.181	1.217	-4.554	4.082	-1.188	77.199	16.799	13.452
6061	197	29	27	C	S	42.304	1.029	-3.968	5.214	-1.008	3.594	-4.722	77.095	16.796	13.461
6062	197	29	27	C	S	46.827	5.011	4.181	1.217	-4.554	-4.082	-1.188	72.786	13.151	17.542
6063	197	29	27	C	S	47.480	2.326	5.468	3.845	-2.223	-5.078	-3.571	77.090	13.473	16.776
6064	197	29	27	C	S	72.520	2.326	-5.468	3.845	-2.223	5.078	-3.571	77.090	13.473	16.776
6065	197	29	27	C	S	73.174	5.011	-4.181	1.217	-4.554	4.082	-1.188	72.786	13.151	17.542
6066	197	29	27	C	S	77.696	1.029	3.968	5.214	-1.008	-3.594	-4.722	77.095	13.461	16.796
6067	197	29	27	C	S	77.897	5.011	4.181	1.217	-4.554	-4.082	-1.188	77.200	16.799	13.452
6068	197	29	27	C	S	81.052	1.339	-1.345	4.880	-1.304	1.226	-4.446	79.466	16.211	13.826
6069	197	29	27	C	S	83.691	2.390	3.450	3.780	-2.281	-3.207	-3.514	79.494	16.213	13.819

BL number	Atoms	γ-PC unit cells	WS_2 unit cells	γ-PC origin atom	WS_2 origin atom	Twist-angle (°)	γ-PC strain 1 (%)	γ-PC strain 2 (%)	γ-PC strain 3 (%)	WS_2 strain 1 (%)	WS_2 strain 2 (%)	WS_2 strain 3 (%)	γ (°)	|**a**|(Å)	|**b**|(Å)
6070	197	29	27	C	S	96.309	2.390	-3.450	3.780	-2.281	3.207	-3.514	79.494	13.819	16.213
6071	197	29	27	C	S	102.104	5.011	-4.181	1.217	-4.554	4.082	-1.188	77.200	16.799	13.452
6072	197	29	27	C	S	102.304	1.029	-3.968	5.214	-1.008	3.594	-4.722	77.095	16.796	13.461
6073	197	29	27	C	S	106.827	5.011	4.181	1.217	-4.554	-4.082	-1.188	72.786	13.151	17.542
6074	197	29	27	C	S	107.480	2.326	5.468	3.845	-2.223	-5.078	-3.571	77.090	13.473	16.776
6075	197	29	27	C	W	12.520	2.326	-5.468	3.845	-2.223	5.078	-3.571	77.090	13.473	16.776
6076	197	29	27	C	W	13.174	5.011	-4.181	1.217	-4.554	4.082	-1.188	72.786	13.151	17.542
6077	197	29	27	C	W	17.696	1.029	3.968	5.214	-1.008	-3.594	-4.722	77.095	13.461	16.796
6078	197	29	27	C	W	17.897	5.011	4.181	1.217	-4.554	-4.082	-1.188	77.200	16.799	13.452
6079	197	29	27	C	W	21.052	1.339	-1.345	4.880	-1.304	1.226	-4.446	79.466	16.211	13.826
6080	197	29	27	C	W	23.691	2.390	3.450	3.780	-2.281	-3.207	-3.514	79.494	16.213	13.819
6081	197	29	27	C	W	36.309	2.390	-3.450	3.780	-2.281	3.207	-3.514	79.494	13.819	16.213
6082	197	29	27	C	W	42.103	5.011	-4.181	1.217	-4.554	4.082	-1.188	77.199	16.799	13.452
6083	197	29	27	C	W	42.304	1.029	-3.968	5.214	-1.008	3.594	-4.722	77.095	16.796	13.461
6084	197	29	27	C	W	46.827	5.011	4.181	1.217	-4.554	-4.082	-1.188	72.786	13.151	17.542
6085	197	29	27	C	W	47.480	2.326	5.468	3.845	-2.223	-5.078	-3.571	77.090	13.473	16.776
6086	197	29	27	C	W	72.520	2.326	-5.468	3.845	-2.223	5.078	-3.571	77.090	13.473	16.776
6087	197	29	27	C	W	73.174	5.011	-4.181	1.217	-4.554	4.082	-1.188	72.786	13.151	17.542
6088	197	29	27	C	W	77.696	1.029	3.968	5.214	-1.008	-3.594	-4.722	77.095	13.461	16.796
6089	197	29	27	C	W	77.897	5.011	4.181	1.217	-4.554	-4.082	-1.188	77.200	16.799	13.452
6090	197	29	27	C	W	81.052	1.339	-1.345	4.880	-1.304	1.226	-4.446	79.466	16.211	13.826
6091	197	29	27	C	W	83.691	2.390	3.450	3.780	-2.281	-3.207	-3.514	79.494	16.213	13.819
6092	197	29	27	C	W	96.309	2.390	-3.450	3.780	-2.281	3.207	-3.514	79.494	13.819	16.213
6093	197	29	27	C	W	102.104	5.011	-4.181	1.217	-4.554	4.082	-1.188	77.200	16.799	13.452
6094	197	29	27	C	W	102.304	1.029	-3.968	5.214	-1.008	3.594	-4.722	77.095	16.796	13.461
6095	197	29	27	C	W	106.827	5.011	4.181	1.217	-4.554	-4.082	-1.188	72.786	13.151	17.542
6096	197	29	27	C	W	107.480	2.326	5.468	3.845	-2.223	-5.078	-3.571	77.090	13.473	16.776
6097	197	29	27	P	S	12.520	2.326	-5.468	3.845	-2.223	5.078	-3.571	77.090	13.473	16.776
6098	197	29	27	P	S	13.174	5.011	-4.181	1.217	-4.554	4.082	-1.188	72.786	13.151	17.542
6099	197	29	27	P	S	17.696	1.029	3.968	5.214	-1.008	-3.594	-4.722	77.095	13.461	16.796
6100	197	29	27	P	S	17.897	5.011	4.181	1.217	-4.554	-4.082	-1.188	77.200	16.799	13.452
6101	197	29	27	P	S	36.309	2.390	-3.450	3.780	-2.281	3.207	-3.514	79.494	13.819	16.213
6102	197	29	27	P	S	42.103	5.011	-4.181	1.217	-4.554	4.082	-1.188	77.199	16.799	13.452
6103	197	29	27	P	S	42.304	1.029	-3.968	5.214	-1.008	3.594	-4.722	77.095	16.796	13.461
6104	197	29	27	P	S	46.827	5.011	4.181	1.217	-4.554	-4.082	-1.188	72.786	13.151	17.542
6105	197	29	27	P	S	47.480	2.326	5.468	3.845	-2.223	-5.078	-3.571	77.090	13.473	16.776
6106	197	29	27	P	S	72.520	2.326	-5.468	3.845	-2.223	5.078	-3.571	77.090	13.473	16.776
6107	197	29	27	P	S	73.174	5.011	-4.181	1.217	-4.554	4.082	-1.188	72.786	13.151	17.542
6108	197	29	27	P	S	77.696	1.029	3.968	5.214	-1.008	-3.594	-4.722	77.095	13.461	16.796
6109	197	29	27	P	S	77.897	5.011	4.181	1.217	-4.554	-4.082	-1.188	77.200	16.799	13.452
6110	197	29	27	P	S	81.052	1.339	-1.345	4.880	-1.304	1.226	-4.446	79.466	16.211	13.826
6111	197	29	27	P	S	83.691	2.390	3.450	3.780	-2.281	-3.207	-3.514	79.494	16.213	13.819
6112	197	29	27	P	S	102.104	5.011	-4.181	1.217	-4.554	4.082	-1.188	77.200	16.799	13.452
6113	197	29	27	P	S	102.304	1.029	-3.968	5.214	-1.008	3.594	-4.722	77.095	16.796	13.461
6114	197	29	27	P	S	106.827	5.011	4.181	1.217	-4.554	-4.082	-1.188	72.786	13.151	17.542
6115	197	29	27	P	S	107.480	2.326	5.468	3.845	-2.223	-5.078	-3.571	77.090	13.473	16.776
6116	197	29	27	P	W	12.520	2.326	-5.468	3.845	-2.223	5.078	-3.571	77.090	13.473	16.776
6117	197	29	27	P	W	13.174	5.011	-4.181	1.217	-4.554	4.082	-1.188	72.786	13.151	17.542
6118	197	29	27	P	W	17.696	1.029	3.968	5.214	-1.008	-3.594	-4.722	77.095	13.461	16.796
6119	197	29	27	P	W	17.897	5.011	4.181	1.217	-4.554	-4.082	-1.188	77.200	16.799	13.452
6120	197	29	27	P	W	21.052	1.339	-1.345	4.880	-1.304	1.226	-4.446	79.466	16.211	13.826

BL number	Atoms	γ-PC unit cells	WS_2 unit cells	γ-PC origin atom	WS_2 origin atom	Twist-angle (°)	γ-PC strain 1 (%)	γ-PC strain 2 (%)	γ-PC strain 3 (%)	WS_2 strain 1 (%)	WS_2 strain 2 (%)	WS_2 strain 3 (%)	γ (°)	|**a**|(Å)	|**b**|(Å)
6121	197	29	27	P	W	23.691	2.390	3.450	3.780	-2.281	-3.207	-3.514	79.494	16.213	13.819
6122	197	29	27	P	W	42.103	5.011	-4.181	1.217	-4.554	4.082	-1.188	77.199	16.799	13.452
6123	197	29	27	P	W	42.304	1.029	-3.968	5.214	-1.008	3.594	-4.722	77.095	16.796	13.461
6124	197	29	27	P	W	46.827	5.011	4.181	1.217	-4.554	-4.082	-1.188	72.786	13.151	17.542
6125	197	29	27	P	W	47.480	2.326	5.468	3.845	-2.223	-5.078	-3.571	77.090	13.473	16.776
6126	197	29	27	P	W	72.520	2.326	-5.468	3.845	-2.223	5.078	-3.571	77.090	13.473	16.776
6127	197	29	27	P	W	73.174	5.011	-4.181	1.217	-4.554	4.082	-1.188	72.786	13.151	17.542
6128	197	29	27	P	W	77.696	1.029	3.968	5.214	-1.008	-3.594	-4.722	77.095	13.461	16.796
6129	197	29	27	P	W	77.897	5.011	4.181	1.217	-4.554	-4.082	-1.188	77.200	16.799	13.452
6130	197	29	27	P	W	96.309	2.390	-3.450	3.780	-2.281	3.207	-3.514	79.494	13.819	16.213
6131	197	29	27	P	W	102.104	5.011	-4.181	1.217	-4.554	4.082	-1.188	77.200	16.799	13.452
6132	197	29	27	P	W	102.304	1.029	-3.968	5.214	-1.008	3.594	-4.722	77.095	16.796	13.461
6133	197	29	27	P	W	106.827	5.011	4.181	1.217	-4.554	-4.082	-1.188	72.786	13.151	17.542
6134	197	29	27	P	W	107.480	2.326	5.468	3.845	-2.223	-5.078	-3.571	77.090	13.473	16.776
6135	197	32	23	C	S	4.307	-4.685	-3.077	-2.001	5.169	3.206	2.085	83.780	14.491	14.834
6136	197	32	23	C	S	8.213	-2.359	3.523	-4.344	2.476	-3.859	4.757	83.694	14.845	14.481
6137	197	32	23	C	S	51.787	-2.359	-3.523	-4.344	2.476	3.859	4.757	83.694	14.845	14.481
6138	197	32	23	C	S	55.693	-4.685	3.077	-2.001	5.169	-3.206	2.085	83.780	14.491	14.834
6139	197	32	23	C	S	64.307	-4.685	-3.077	-2.001	5.169	3.206	2.085	83.780	14.491	14.834
6140	197	32	23	C	S	68.213	-2.359	3.523	-4.344	2.476	-3.859	4.757	83.694	14.845	14.481
6141	197	32	23	C	S	111.787	-2.359	-3.523	-4.344	2.476	3.859	4.757	83.694	14.845	14.481
6142	197	32	23	C	S	115.693	-4.685	3.077	-2.001	5.169	-3.206	2.085	83.780	14.491	14.834
6143	197	32	23	C	W	4.307	-4.685	-3.077	-2.001	5.169	3.206	2.085	83.780	14.491	14.834
6144	197	32	23	C	W	8.213	-2.359	3.523	-4.344	2.476	-3.859	4.757	83.694	14.845	14.481
6145	197	32	23	C	W	51.787	-2.359	-3.523	-4.344	2.476	3.859	4.757	83.694	14.845	14.481
6146	197	32	23	C	W	55.693	-4.685	3.077	-2.001	5.169	-3.206	2.085	83.780	14.491	14.834
6147	197	32	23	C	W	64.307	-4.685	-3.077	-2.001	5.169	3.206	2.085	83.780	14.491	14.834
6148	197	32	23	C	W	68.213	-2.359	3.523	-4.344	2.476	-3.859	4.757	83.694	14.845	14.481
6149	197	32	23	C	W	111.787	-2.359	-3.523	-4.344	2.476	3.859	4.757	83.694	14.845	14.481
6150	197	32	23	C	W	115.693	-4.685	3.077	-2.001	5.169	-3.206	2.085	83.780	14.491	14.834
6151	197	32	23	P	S	4.307	-4.685	-3.077	-2.001	5.169	3.206	2.085	83.780	14.491	14.834
6152	197	32	23	P	S	8.213	-2.359	3.523	-4.344	2.476	-3.859	4.757	83.694	14.845	14.481
6153	197	32	23	P	S	51.787	-2.359	-3.523	-4.344	2.476	3.859	4.757	83.694	14.845	14.481
6154	197	32	23	P	S	55.693	-4.685	3.077	-2.001	5.169	-3.206	2.085	83.780	14.491	14.834
6155	197	32	23	P	S	64.307	-4.685	-3.077	-2.001	5.169	3.206	2.085	83.780	14.491	14.834
6156	197	32	23	P	S	68.213	-2.359	3.523	-4.344	2.476	-3.859	4.757	83.694	14.845	14.481
6157	197	32	23	P	S	111.787	-2.359	-3.523	-4.344	2.476	3.859	4.757	83.694	14.845	14.481
6158	197	32	23	P	S	115.693	-4.685	3.077	-2.001	5.169	-3.206	2.085	83.780	14.491	14.834
6159	197	32	23	P	W	4.307	-4.685	-3.077	-2.001	5.169	3.206	2.085	83.780	14.491	14.834
6160	197	32	23	P	W	8.213	-2.359	3.523	-4.344	2.476	-3.859	4.757	83.694	14.845	14.481
6161	197	32	23	P	W	51.787	-2.359	-3.523	-4.344	2.476	3.859	4.757	83.694	14.845	14.481
6162	197	32	23	P	W	55.693	-4.685	3.077	-2.001	5.169	-3.206	2.085	83.780	14.491	14.834
6163	197	32	23	P	W	64.307	-4.685	-3.077	-2.001	5.169	3.206	2.085	83.780	14.491	14.834
6164	197	32	23	P	W	68.213	-2.359	3.523	-4.344	2.476	-3.859	4.757	83.694	14.845	14.481
6165	197	32	23	P	W	111.787	-2.359	-3.523	-4.344	2.476	3.859	4.757	83.694	14.845	14.481
6166	197	32	23	P	W	115.693	-4.685	3.077	-2.001	5.169	-3.206	2.085	83.780	14.491	14.834
6167	198	30	26	C	S	8.948	1.048	-2.070	1.377	-1.026	2.015	-1.340	68.592	16.360	14.425
6168	198	30	26	C	S	10.158	2.281	0.070	0.165	-2.182	-0.070	-0.165	68.578	16.362	14.426
6169	198	30	26	C	S	49.842	2.281	-0.070	0.165	-2.182	0.070	-0.165	68.578	16.362	14.426
6170	198	30	26	C	S	51.052	1.048	2.070	1.377	-1.026	-2.015	-1.340	68.592	14.425	16.360
6171	198	30	26	C	S	68.948	1.048	-2.070	1.377	-1.026	2.015	-1.340	68.592	16.360	14.425

BL number	Atoms	γ-PC unit cells	WS_2 unit cells	γ-PC origin atom	WS_2 origin atom	Twist-angle (°)	γ-PC strain 1 (%)	γ-PC strain 2 (%)	γ-PC strain 3 (%)	WS_2 strain 1 (%)	WS_2 strain 2 (%)	WS_2 strain 3 (%)	γ (°)	|**a**|(Å)	|**b**|(Å)
6172	198	30	26	C	S	70.158	2.281	0.070	0.165	-2.182	-0.070	-0.165	68.579	16.362	14.426
6173	198	30	26	C	S	109.842	2.281	-0.070	0.165	-2.182	0.070	-0.165	68.579	16.362	14.426
6174	198	30	26	C	S	111.052	1.048	2.070	1.377	-1.026	-2.015	-1.340	68.592	14.425	16.360
6175	198	30	26	C	W	8.948	1.048	-2.070	1.377	-1.026	2.015	-1.340	68.592	16.360	14.425
6176	198	30	26	C	W	10.158	2.281	0.070	0.165	-2.182	-0.070	-0.165	68.578	16.362	14.426
6177	198	30	26	C	W	49.842	2.281	-0.070	0.165	-2.182	0.070	-0.165	68.578	16.362	14.426
6178	198	30	26	C	W	51.052	1.048	2.070	1.377	-1.026	-2.015	-1.340	68.592	14.425	16.360
6179	198	30	26	C	W	68.948	1.048	-2.070	1.377	-1.026	2.015	-1.340	68.592	16.360	14.425
6180	198	30	26	C	W	70.158	2.281	0.070	0.165	-2.182	-0.070	-0.165	68.579	16.362	14.426
6181	198	30	26	C	W	109.842	2.281	-0.070	0.165	-2.182	0.070	-0.165	68.579	16.362	14.426
6182	198	30	26	C	W	111.052	1.048	2.070	1.377	-1.026	-2.015	-1.340	68.592	14.425	16.360
6183	198	30	26	P	S	8.948	1.048	-2.070	1.377	-1.026	2.015	-1.340	68.592	16.360	14.425
6184	198	30	26	P	S	10.158	2.281	0.070	0.165	-2.182	-0.070	-0.165	68.578	16.362	14.426
6185	198	30	26	P	S	49.842	2.281	-0.070	0.165	-2.182	0.070	-0.165	68.578	16.362	14.426
6186	198	30	26	P	S	51.052	1.048	2.070	1.377	-1.026	-2.015	-1.340	68.592	14.425	16.360
6187	198	30	26	P	S	68.948	1.048	-2.070	1.377	-1.026	2.015	-1.340	68.592	16.360	14.425
6188	198	30	26	P	S	70.158	2.281	0.070	0.165	-2.182	-0.070	-0.165	68.579	16.362	14.426
6189	198	30	26	P	S	109.842	2.281	-0.070	0.165	-2.182	0.070	-0.165	68.579	16.362	14.426
6190	198	30	26	P	S	111.052	1.048	2.070	1.377	-1.026	-2.015	-1.340	68.592	14.425	16.360
6191	198	30	26	P	W	8.948	1.048	-2.070	1.377	-1.026	2.015	-1.340	68.592	16.360	14.425
6192	198	30	26	P	W	10.158	2.281	0.070	0.165	-2.182	-0.070	-0.165	68.578	16.362	14.426
6193	198	30	26	P	W	49.842	2.281	-0.070	0.165	-2.182	0.070	-0.165	68.578	16.362	14.426
6194	198	30	26	P	W	51.052	1.048	2.070	1.377	-1.026	-2.015	-1.340	68.592	14.425	16.360
6195	198	30	26	P	W	68.948	1.048	-2.070	1.377	-1.026	2.015	-1.340	68.592	16.360	14.425
6196	198	30	26	P	W	70.158	2.281	0.070	0.165	-2.182	-0.070	-0.165	68.579	16.362	14.426
6197	198	30	26	P	W	109.842	2.281	-0.070	0.165	-2.182	0.070	-0.165	68.579	16.362	14.426
6198	198	30	26	P	W	111.052	1.048	2.070	1.377	-1.026	-2.015	-1.340	68.592	14.425	16.360
6199	199	31	25	C	S	51.052	-0.599	0.000	-0.599	0.606	0.000	0.606	60.000	15.901	15.901
6200	199	31	25	C	S	68.948	-0.599	0.000	-0.599	0.606	0.000	0.606	60.000	15.901	15.901
6201	199	31	25	C	S	111.052	-0.599	0.000	-0.599	0.606	0.000	0.606	60.000	15.901	15.901
6202	199	31	25	C	W	8.948	-0.599	0.000	-0.599	0.606	0.000	0.606	60.000	15.901	15.901
6203	199	31	25	C	W	51.052	-0.599	0.000	-0.599	0.606	0.000	0.606	60.000	15.901	15.901
6204	199	31	25	C	W	111.052	-0.599	0.000	-0.599	0.606	0.000	0.606	60.000	15.901	15.901
6205	199	31	25	P	S	51.052	-0.599	0.000	-0.599	0.606	0.000	0.606	60.000	15.901	15.901
6206	199	31	25	P	S	68.948	-0.599	0.000	-0.599	0.606	0.000	0.606	60.000	15.901	15.901
6207	199	31	25	P	W	8.948	-0.599	0.000	-0.599	0.606	0.000	0.606	60.000	15.901	15.901
6208	199	31	25	P	W	111.052	-0.599	0.000	-0.599	0.606	0.000	0.606	60.000	15.901	15.901
6209	199	31	25	C	S	9.183	3.582	-1.564	-4.453	-3.342	1.717	4.888	80.612	18.586	11.962
6210	199	31	25	C	S	50.485	-4.497	1.095	3.633	4.941	-1.021	-3.387	80.629	11.961	18.587
6211	199	31	25	C	S	50.817	3.582	1.564	-4.453	-3.342	-1.717	4.888	80.612	18.586	11.962
6212	199	31	25	C	S	69.183	3.582	-1.564	-4.453	-3.342	1.717	4.888	80.612	18.586	11.962
6213	199	31	25	C	S	69.515	-4.497	-1.094	3.633	4.941	1.020	-3.387	80.629	11.961	18.587
6214	199	31	25	C	S	110.485	-4.497	1.095	3.633	4.941	-1.021	-3.387	80.629	11.961	18.587
6215	199	31	25	C	S	110.817	3.582	1.564	-4.453	-3.342	-1.717	4.888	80.611	18.586	11.962
6216	199	31	25	C	W	9.183	3.582	-1.564	-4.453	-3.342	1.717	4.888	80.612	18.586	11.962
6217	199	31	25	C	W	9.515	-4.497	-1.094	3.633	4.941	1.020	-3.387	80.629	11.961	18.587
6218	199	31	25	C	W	50.485	-4.497	1.095	3.633	4.941	-1.021	-3.387	80.629	11.961	18.587
6219	199	31	25	C	W	50.817	3.582	1.564	-4.453	-3.342	-1.717	4.888	80.612	18.586	11.962
6220	199	31	25	C	W	69.183	3.582	-1.564	-4.453	-3.342	1.717	4.888	80.612	18.586	11.962
6221	199	31	25	C	W	110.485	-4.497	1.095	3.633	4.941	-1.021	-3.387	80.629	11.961	18.587
6222	199	31	25	C	W	110.817	3.582	1.564	-4.453	-3.342	-1.717	4.888	80.611	18.586	11.962

BL number	Atoms	γ-PC unit cells	WS_2 unit cells	γ-PC origin atom	WS_2 origin atom	Twist-angle (°)	γ-PC strain 1 (%)	γ-PC strain 2 (%)	γ-PC strain 3 (%)	WS_2 strain 1 (%)	WS_2 strain 2 (%)	WS_2 strain 3 (%)	γ (°)	|**a**|(Å)	|**b**|(Å)
6223	199	31	25	P	S	9.183	3.582	-1.564	-4.453	-3.342	1.717	4.888	80.612	18.586	11.962
6224	199	31	25	P	S	9.515	-4.497	-1.094	3.633	4.941	1.020	-3.387	80.629	11.961	18.587
6225	199	31	25	P	S	50.485	-4.497	1.095	3.633	4.941	-1.021	-3.387	80.629	11.961	18.587
6226	199	31	25	P	S	50.817	3.582	1.564	-4.453	-3.342	-1.717	4.888	80.612	18.586	11.962
6227	199	31	25	P	S	69.183	3.582	-1.564	-4.453	-3.342	1.717	4.888	80.612	18.586	11.962
6228	199	31	25	P	S	110.485	-4.497	1.095	3.633	4.941	-1.021	-3.387	80.629	11.961	18.587
6229	199	31	25	P	S	110.817	3.582	1.564	-4.453	-3.342	-1.717	4.888	80.611	18.586	11.962
6230	199	31	25	P	W	9.183	3.582	-1.564	-4.453	-3.342	1.717	4.888	80.612	18.586	11.962
6231	199	31	25	P	W	50.485	-4.497	1.095	3.633	4.941	-1.021	-3.387	80.629	11.961	18.587
6232	199	31	25	P	W	50.817	3.582	1.564	-4.453	-3.342	-1.717	4.888	80.612	18.586	11.962
6233	199	31	25	P	W	69.183	3.582	-1.564	-4.453	-3.342	1.717	4.888	80.612	18.586	11.962
6234	199	31	25	P	W	69.515	-4.497	-1.094	3.633	4.941	1.020	-3.387	80.629	11.961	18.587
6235	199	31	25	P	W	110.485	-4.497	1.095	3.633	4.941	-1.021	-3.387	80.629	11.961	18.587
6236	199	31	25	P	W	110.817	3.582	1.564	-4.453	-3.342	-1.717	4.888	80.611	18.586	11.962
6237	200	32	24	C	S	4.715	-3.007	-5.250	-1.702	3.200	5.435	1.762	86.943	16.951	12.886
6238	200	32	24	C	S	7.589	0.334	-0.302	-4.909	-0.332	0.334	5.443	74.907	18.904	11.959
6239	200	32	24	C	S	9.515	-4.497	3.049	-0.121	4.941	-3.056	0.121	74.781	11.961	18.908
6240	200	32	24	C	S	50.485	-4.497	-3.049	-0.121	4.941	3.056	0.121	74.781	11.961	18.908
6241	200	32	24	C	S	52.411	0.334	0.302	-4.909	-0.332	-0.335	5.443	74.907	18.904	11.959
6242	200	32	24	C	S	55.285	-3.007	5.250	-1.702	3.200	-5.435	1.762	86.943	16.951	12.886
6243	200	32	24	C	S	64.715	-3.007	-5.250	-1.702	3.200	5.435	1.762	86.943	16.951	12.886
6244	200	32	24	C	S	67.589	0.334	-0.302	-4.909	-0.332	0.334	5.443	74.907	18.904	11.959
6245	200	32	24	C	S	69.515	-4.497	3.049	-0.121	4.941	-3.056	0.121	74.781	11.961	18.908
6246	200	32	24	C	S	110.485	-4.497	-3.049	-0.121	4.941	3.056	0.121	74.781	11.961	18.908
6247	200	32	24	C	S	112.411	0.334	0.302	-4.909	-0.332	-0.335	5.443	74.907	18.904	11.959
6248	200	32	24	C	S	115.285	-3.007	5.250	-1.702	3.200	-5.435	1.762	86.943	16.951	12.886
6249	200	32	24	C	W	4.715	-3.007	-5.250	-1.702	3.200	5.435	1.762	86.943	16.951	12.886
6250	200	32	24	C	W	7.589	0.334	-0.302	-4.909	-0.332	0.334	5.443	74.907	18.904	11.959
6251	200	32	24	C	W	9.515	-4.497	3.049	-0.121	4.941	-3.056	0.121	74.781	11.961	18.908
6252	200	32	24	C	W	50.485	-4.497	-3.049	-0.121	4.941	3.056	0.121	74.781	11.961	18.908
6253	200	32	24	C	W	52.411	0.334	0.302	-4.909	-0.332	-0.335	5.443	74.907	18.904	11.959
6254	200	32	24	C	W	55.285	-3.007	5.250	-1.702	3.200	-5.435	1.762	86.943	16.951	12.886
6255	200	32	24	C	W	64.715	-3.007	-5.250	-1.702	3.200	5.435	1.762	86.943	16.951	12.886
6256	200	32	24	C	W	67.589	0.334	-0.302	-4.909	-0.332	0.334	5.443	74.907	18.904	11.959
6257	200	32	24	C	W	69.515	-4.497	3.049	-0.121	4.941	-3.056	0.121	74.781	11.961	18.908
6258	200	32	24	C	W	110.485	-4.497	-3.049	-0.121	4.941	3.056	0.121	74.781	11.961	18.908
6259	200	32	24	C	W	112.411	0.334	0.302	-4.909	-0.332	-0.335	5.443	74.907	18.904	11.959
6260	200	32	24	C	W	115.285	-3.007	5.250	-1.702	3.200	-5.435	1.762	86.943	16.951	12.886
6261	200	32	24	P	S	4.715	-3.007	-5.250	-1.702	3.200	5.435	1.762	86.943	16.951	12.886
6262	200	32	24	P	S	7.589	0.334	-0.302	-4.909	-0.332	0.334	5.443	74.907	18.904	11.959
6263	200	32	24	P	S	9.515	-4.497	3.049	-0.121	4.941	-3.056	0.121	74.781	11.961	18.908
6264	200	32	24	P	S	50.485	-4.497	-3.049	-0.121	4.941	3.056	0.121	74.781	11.961	18.908
6265	200	32	24	P	S	52.411	0.334	0.302	-4.909	-0.332	-0.335	5.443	74.907	18.904	11.959
6266	200	32	24	P	S	55.285	-3.007	5.250	-1.702	3.200	-5.435	1.762	86.943	16.951	12.886
6267	200	32	24	P	S	64.715	-3.007	-5.250	-1.702	3.200	5.435	1.762	86.943	16.951	12.886
6268	200	32	24	P	S	67.589	0.334	-0.302	-4.909	-0.332	0.334	5.443	74.907	18.904	11.959
6269	200	32	24	P	S	69.515	-4.497	3.049	-0.121	4.941	-3.056	0.121	74.781	11.961	18.908
6270	200	32	24	P	S	110.485	-4.497	-3.049	-0.121	4.941	3.056	0.121	74.781	11.961	18.908
6271	200	32	24	P	S	112.411	0.334	0.302	-4.909	-0.332	-0.335	5.443	74.907	18.904	11.959
6272	200	32	24	P	S	115.285	-3.007	5.250	-1.702	3.200	-5.435	1.762	86.943	16.951	12.886
6273	200	32	24	P	W	4.715	-3.007	-5.250	-1.702	3.200	5.435	1.762	86.943	16.951	12.886

BL number	Atoms	γ-PC unit cells	WS_2 unit cells	γ-PC origin atom	WS_2 origin atom	Twist-angle (°)	γ-PC strain 1 (%)	γ-PC strain 2 (%)	γ-PC strain 3 (%)	WS_2 strain 1 (%)	WS_2 strain 2 (%)	WS_2 strain 3 (%)	γ (°)	$\lvert\mathbf{a}\rvert$(Å)	$\lvert\mathbf{b}\rvert$(Å)
6274	200	32	24	P	W	7.589	0.334	-0.302	-4.909	-0.332	0.334	5.443	74.907	18.904	11.959
6275	200	32	24	P	W	9.515	-4.497	3.049	-0.121	4.941	-3.056	0.121	74.781	11.961	18.908
6276	200	32	24	P	W	50.485	-4.497	-3.049	-0.121	4.941	3.056	0.121	74.781	11.961	18.908
6277	200	32	24	P	W	52.411	0.334	0.302	-4.909	-0.332	-0.335	5.443	74.907	18.904	11.959
6278	200	32	24	P	W	55.285	-3.007	5.250	-1.702	3.200	-5.435	1.762	86.943	16.951	12.886
6279	200	32	24	P	W	64.715	-3.007	-5.250	-1.702	3.200	5.435	1.762	86.943	16.951	12.886
6280	200	32	24	P	W	67.589	0.334	-0.302	-4.909	-0.332	0.334	5.443	74.907	18.904	11.959
6281	200	32	24	P	W	69.515	-4.497	3.049	-0.121	4.941	-3.056	0.121	74.781	11.961	18.908
6282	200	32	24	P	W	110.485	-4.497	-3.049	-0.121	4.941	3.056	0.121	74.781	11.961	18.908
6283	200	32	24	P	W	112.411	0.334	0.302	-4.909	-0.332	-0.335	5.443	74.907	18.904	11.959
6284	200	32	24	P	W	115.285	-3.007	5.250	-1.702	3.200	-5.435	1.762	86.943	16.951	12.886
6285	201	30	27	C	S	8.948	1.048	-2.823	3.353	-1.026	2.645	-3.143	72.705	17.420	13.467
6286	201	30	27	C	S	9.183	3.582	-2.344	0.830	-3.342	2.305	-0.817	72.703	13.463	17.427
6287	201	30	27	C	S	10.893	0.418	0.690	4.020	-0.415	-0.639	-3.721	79.108	14.426	15.814
6288	201	30	27	C	S	12.520	2.326	3.576	2.050	-2.223	-3.435	-1.970	72.656	13.473	17.415
6289	201	30	27	C	S	47.480	2.326	-3.576	2.050	-2.223	3.435	-1.970	72.656	13.473	17.415
6290	201	30	27	C	S	49.107	0.418	-0.690	4.020	-0.415	0.639	-3.721	79.108	14.426	15.814
6291	201	30	27	C	S	50.817	3.582	2.344	0.830	-3.342	-2.305	-0.817	72.703	17.427	13.463
6292	201	30	27	C	S	51.052	1.048	2.823	3.353	-1.026	-2.646	-3.143	72.705	17.420	13.467
6293	201	30	27	C	S	51.487	2.390	3.562	1.987	-2.281	-3.426	-1.911	79.153	14.428	15.805
6294	201	30	27	C	S	68.513	2.390	-3.562	1.987	-2.281	3.426	-1.911	79.153	15.805	14.428
6295	201	30	27	C	S	68.948	1.048	-2.823	3.353	-1.026	2.645	-3.143	72.705	17.420	13.467
6296	201	30	27	C	S	69.183	3.582	-2.344	0.830	-3.342	2.305	-0.817	72.703	13.463	17.427
6297	201	30	27	C	S	70.893	0.418	0.690	4.020	-0.415	-0.639	-3.721	79.108	14.426	15.814
6298	201	30	27	C	S	72.520	2.326	3.576	2.050	-2.223	-3.435	-1.970	72.656	13.473	17.415
6299	201	30	27	C	S	107.480	2.326	-3.576	2.050	-2.223	3.435	-1.970	72.656	13.473	17.415
6300	201	30	27	C	S	109.107	0.418	-0.690	4.020	-0.415	0.639	-3.721	79.108	14.426	15.814
6301	201	30	27	C	S	110.817	3.582	2.344	0.830	-3.342	-2.305	-0.817	72.703	17.427	13.463
6302	201	30	27	C	S	111.052	1.048	2.823	3.353	-1.026	-2.646	-3.143	72.705	17.420	13.467
6303	201	30	27	C	S	111.487	2.390	3.562	1.987	-2.281	-3.426	-1.911	79.153	14.428	15.805
6304	201	30	27	C	W	8.513	2.390	-3.562	1.987	-2.281	3.426	-1.911	79.153	15.805	14.428
6305	201	30	27	C	W	8.948	1.048	-2.823	3.353	-1.026	2.645	-3.143	72.705	17.420	13.467
6306	201	30	27	C	W	9.183	3.582	-2.344	0.830	-3.342	2.305	-0.817	72.703	13.463	17.427
6307	201	30	27	C	W	10.893	4.020	0.667	0.418	-3.721	-0.662	-0.415	79.092	15.815	14.427
6308	201	30	27	C	W	12.520	2.326	3.576	2.050	-2.223	-3.435	-1.970	72.656	13.473	17.415
6309	201	30	27	C	W	47.480	2.326	-3.576	2.050	-2.223	3.435	-1.970	72.656	13.473	17.415
6310	201	30	27	C	W	50.817	3.582	2.344	0.830	-3.342	-2.305	-0.817	72.703	17.427	13.463
6311	201	30	27	C	W	51.052	1.048	2.823	3.353	-1.026	-2.646	-3.143	72.705	17.420	13.467
6312	201	30	27	C	W	68.948	1.048	-2.823	3.353	-1.026	2.645	-3.143	72.705	17.420	13.467
6313	201	30	27	C	W	69.183	3.582	-2.344	0.830	-3.342	2.305	-0.817	72.703	13.463	17.427
6314	201	30	27	C	W	72.520	2.326	3.576	2.050	-2.223	-3.435	-1.970	72.656	13.473	17.415
6315	201	30	27	C	W	107.480	2.326	-3.576	2.050	-2.223	3.435	-1.970	72.656	13.473	17.415
6316	201	30	27	C	W	109.107	4.020	-0.667	0.418	-3.721	0.662	-0.415	79.092	15.815	14.427
6317	201	30	27	C	W	110.817	3.582	2.344	0.830	-3.342	-2.305	-0.817	72.703	17.427	13.463
6318	201	30	27	C	W	111.052	1.048	2.823	3.353	-1.026	-2.646	-3.143	72.705	17.420	13.467
6319	201	30	27	P	S	8.948	1.048	-2.823	3.353	-1.026	2.645	-3.143	72.705	17.420	13.467
6320	201	30	27	P	S	10.893	0.418	0.690	4.020	-0.415	-0.639	-3.721	79.108	14.426	15.814
6321	201	30	27	P	S	49.107	0.418	-0.690	4.020	-0.415	0.639	-3.721	79.108	14.426	15.814
6322	201	30	27	P	S	51.487	2.390	3.562	1.987	-2.281	-3.426	-1.911	79.153	14.428	15.805
6323	201	30	27	P	S	68.513	2.390	-3.562	1.987	-2.281	3.426	-1.911	79.153	15.805	14.428
6324	201	30	27	P	S	70.893	0.418	0.690	4.020	-0.415	-0.639	-3.721	79.108	14.426	15.814

BL number	Atoms	γ-PC unit cells	WS_2 unit cells	γ-PC origin atom	WS_2 origin atom	Twist-angle (°)	γ-PC strain 1 (%)	γ-PC strain 2 (%)	γ-PC strain 3 (%)	WS_2 strain 1 (%)	WS_2 strain 2 (%)	WS_2 strain 3 (%)	γ (°)	$\lvert\mathbf{a}\rvert$(Å)	$\lvert\mathbf{b}\rvert$(Å)
6325	201	30	27	P	S	109.107	0.418	-0.690	4.020	-0.415	0.639	-3.721	79.108	14.426	15.814
6326	201	30	27	P	S	111.052	1.048	2.823	3.353	-1.026	-2.646	-3.143	72.705	17.420	13.467
6327	201	30	27	P	S	111.487	2.390	3.562	1.987	-2.281	-3.426	-1.911	79.153	14.428	15.805
6328	201	30	27	P	W	8.513	2.390	-3.562	1.987	-2.281	3.426	-1.911	79.153	15.805	14.428
6329	201	30	27	P	W	10.893	4.020	0.667	0.418	-3.721	-0.662	-0.415	79.092	15.815	14.427
6330	201	30	27	P	W	51.052	1.048	2.823	3.353	-1.026	-2.646	-3.143	72.705	17.420	13.467
6331	201	30	27	P	W	68.948	1.048	-2.823	3.353	-1.026	2.645	-3.143	72.705	17.420	13.467
6332	201	30	27	P	W	109.107	4.020	-0.667	0.418	-3.721	0.662	-0.415	79.092	15.815	14.427
6333	201	33	23	C	S	1.945	-4.723	-3.810	-3.416	5.216	4.090	3.667	83.390	15.242	14.339
6334	201	33	23	C	S	6.587	-3.853	3.984	-4.294	4.175	-4.358	4.698	83.352	14.354	15.226
6335	201	33	23	C	S	53.413	-3.853	-3.984	-4.294	4.175	4.358	4.698	83.352	14.354	15.226
6336	201	33	23	C	S	58.055	-4.723	3.810	-3.416	5.216	-4.090	3.667	83.390	15.242	14.339
6337	201	33	23	C	S	61.945	-4.723	-3.810	-3.416	5.216	4.090	3.667	83.390	15.242	14.339
6338	201	33	23	C	S	66.587	-3.853	3.984	-4.294	4.175	-4.358	4.698	83.352	14.354	15.226
6339	201	33	23	C	S	113.413	-3.853	-3.984	-4.294	4.175	4.358	4.698	83.352	14.354	15.226
6340	201	33	23	C	S	118.055	-4.723	3.810	-3.416	5.216	-4.090	3.667	83.390	15.242	14.339
6341	201	33	23	C	W	1.945	-4.723	-3.810	-3.416	5.216	4.090	3.667	83.390	15.242	14.339
6342	201	33	23	C	W	6.587	-3.853	3.984	-4.294	4.175	-4.358	4.698	83.352	14.354	15.226
6343	201	33	23	C	W	53.413	-3.853	-3.984	-4.294	4.175	4.358	4.698	83.352	14.354	15.226
6344	201	33	23	C	W	58.055	-4.723	3.810	-3.416	5.216	-4.090	3.667	83.390	15.242	14.339
6345	201	33	23	C	W	61.945	-4.723	-3.810	-3.416	5.216	4.090	3.667	83.390	15.242	14.339
6346	201	33	23	C	W	66.587	-3.853	3.984	-4.294	4.175	-4.358	4.698	83.352	14.354	15.226
6347	201	33	23	C	W	113.413	-3.853	-3.984	-4.294	4.175	4.358	4.698	83.352	14.354	15.226
6348	201	33	23	C	W	118.055	-4.723	3.810	-3.416	5.216	-4.090	3.667	83.390	15.242	14.339
6349	201	33	23	P	S	1.945	-4.723	-3.810	-3.416	5.216	4.090	3.667	83.390	15.242	14.339
6350	201	33	23	P	S	6.587	-3.853	3.984	-4.294	4.175	-4.358	4.698	83.352	14.354	15.226
6351	201	33	23	P	S	53.413	-3.853	-3.984	-4.294	4.175	4.358	4.698	83.352	14.354	15.226
6352	201	33	23	P	S	58.055	-4.723	3.810	-3.416	5.216	-4.090	3.667	83.390	15.242	14.339
6353	201	33	23	P	S	61.945	-4.723	-3.810	-3.416	5.216	4.090	3.667	83.390	15.242	14.339
6354	201	33	23	P	S	66.587	-3.853	3.984	-4.294	4.175	-4.358	4.698	83.352	14.354	15.226
6355	201	33	23	P	S	113.413	-3.853	-3.984	-4.294	4.175	4.358	4.698	83.352	14.354	15.226
6356	201	33	23	P	S	118.055	-4.723	3.810	-3.416	5.216	-4.090	3.667	83.390	15.242	14.339
6357	201	33	23	P	W	1.945	-4.723	-3.810	-3.416	5.216	4.090	3.667	83.390	15.242	14.339
6358	201	33	23	P	W	6.587	-3.853	3.984	-4.294	4.175	-4.358	4.698	83.352	14.354	15.226
6359	201	33	23	P	W	53.413	-3.853	-3.984	-4.294	4.175	4.358	4.698	83.352	14.354	15.226
6360	201	33	23	P	W	58.055	-4.723	3.810	-3.416	5.216	-4.090	3.667	83.390	15.242	14.339
6361	201	33	23	P	W	61.945	-4.723	-3.810	-3.416	5.216	4.090	3.667	83.390	15.242	14.339
6362	201	33	23	P	W	66.587	-3.853	3.984	-4.294	4.175	-4.358	4.698	83.352	14.354	15.226
6363	201	33	23	P	W	113.413	-3.853	-3.984	-4.294	4.175	4.358	4.698	83.352	14.354	15.226
6364	201	33	23	P	W	118.055	-4.723	3.810	-3.416	5.216	-4.090	3.667	83.390	15.242	14.339
6365	202	31	26	C	S	0.000	5.011	2.933	-3.862	-4.554	-3.179	4.185	71.445	13.826	17.070
6366	202	31	26	C	S	3.004	-3.388	4.434	4.452	3.635	-4.071	-4.088	78.829	17.335	13.151
6367	202	31	26	C	S	12.520	2.326	-3.761	-1.494	-2.223	3.877	1.540	75.855	13.473	17.099
6368	202	31	26	C	S	15.608	-2.145	1.627	3.037	2.241	-1.534	-2.864	75.990	17.102	13.467
6369	202	31	26	C	S	17.696	1.029	5.212	-0.261	-1.008	-5.240	0.263	76.009	17.104	13.456
6370	202	31	26	C	S	42.304	1.029	-5.212	-0.261	-1.008	5.240	0.263	76.009	13.456	17.104
6371	202	31	26	C	S	44.392	-2.145	-1.627	3.037	2.241	1.534	-2.864	75.990	17.102	13.467
6372	202	31	26	C	S	47.480	2.326	3.761	-1.494	-2.223	-3.877	1.540	75.855	13.473	17.099
6373	202	31	26	C	S	56.996	-3.388	-4.434	4.452	3.635	4.071	-4.088	78.829	17.335	13.151
6374	202	31	26	C	S	60.000	5.011	2.933	-3.862	-4.554	-3.179	4.185	71.445	13.826	17.070
6375	202	31	26	C	S	63.005	-3.388	4.434	4.452	3.635	-4.071	-4.088	78.829	17.335	13.151

BL number	Atoms	γ-PC unit cells	WS_2 unit cells	γ-PC origin atom	WS_2 origin atom	Twist-angle (°)	γ-PC strain 1 (%)	γ-PC strain 2 (%)	γ-PC strain 3 (%)	WS_2 strain 1 (%)	WS_2 strain 2 (%)	WS_2 strain 3 (%)	γ (°)	|**a**|(Å)	|**b**|(Å)
6376	202	31	26	C	S	72.520	2.326	-3.761	-1.494	-2.223	3.877	1.540	75.855	13.473	17.099
6377	202	31	26	C	S	75.609	-2.145	1.627	3.037	2.241	-1.534	-2.864	75.990	17.102	13.467
6378	202	31	26	C	S	77.696	1.029	5.212	-0.261	-1.008	-5.240	0.263	76.009	17.104	13.456
6379	202	31	26	C	S	102.304	1.029	-5.212	-0.261	-1.008	5.240	0.263	76.009	13.456	17.104
6380	202	31	26	C	S	104.392	-2.145	-1.627	3.037	2.241	1.534	-2.864	75.990	17.102	13.467
6381	202	31	26	C	S	107.480	2.326	3.761	-1.494	-2.223	-3.877	1.540	75.855	13.473	17.099
6382	202	31	26	C	S	116.996	-3.388	-4.434	4.452	3.635	4.071	-4.088	78.829	17.335	13.151
6383	202	31	26	C	W	0.000	5.011	2.933	-3.862	-4.554	-3.179	4.185	71.445	13.826	17.070
6384	202	31	26	C	W	3.004	-3.388	4.434	4.452	3.635	-4.071	-4.088	78.829	17.335	13.151
6385	202	31	26	C	W	12.520	2.326	-3.761	-1.494	-2.223	3.877	1.540	75.855	13.473	17.099
6386	202	31	26	C	W	15.608	-2.145	1.627	3.037	2.241	-1.534	-2.864	75.990	17.102	13.467
6387	202	31	26	C	W	17.696	1.029	5.212	-0.261	-1.008	-5.240	0.263	76.009	17.104	13.456
6388	202	31	26	C	W	42.304	1.029	-5.212	-0.261	-1.008	5.240	0.263	76.009	13.456	17.104
6389	202	31	26	C	W	44.392	-2.145	-1.627	3.037	2.241	1.534	-2.864	75.990	17.102	13.467
6390	202	31	26	C	W	47.480	2.326	3.761	-1.494	-2.223	-3.877	1.540	75.855	13.473	17.099
6391	202	31	26	C	W	56.996	-3.388	-4.434	4.452	3.635	4.071	-4.088	78.829	17.335	13.151
6392	202	31	26	C	W	60.000	5.011	2.933	-3.862	-4.554	-3.179	4.185	71.445	13.826	17.070
6393	202	31	26	C	W	63.005	-3.388	4.434	4.452	3.635	-4.071	-4.088	78.829	17.335	13.151
6394	202	31	26	C	W	72.520	2.326	-3.761	-1.494	-2.223	3.877	1.540	75.855	13.473	17.099
6395	202	31	26	C	W	75.609	-2.145	1.627	3.037	2.241	-1.534	-2.864	75.990	17.102	13.467
6396	202	31	26	C	W	77.696	1.029	5.212	-0.261	-1.008	-5.240	0.263	76.009	17.104	13.456
6397	202	31	26	C	W	102.304	1.029	-5.212	-0.261	-1.008	5.240	0.263	76.009	13.456	17.104
6398	202	31	26	C	W	104.392	-2.145	-1.627	3.037	2.241	1.534	-2.864	75.990	17.102	13.467
6399	202	31	26	C	W	107.480	2.326	3.761	-1.494	-2.223	-3.877	1.540	75.855	13.473	17.099
6400	202	31	26	C	W	116.996	-3.388	-4.434	4.452	3.635	4.071	-4.088	78.829	17.335	13.151
6401	202	31	26	P	S	0.000	5.011	2.933	-3.862	-4.554	-3.179	4.185	71.445	13.826	17.070
6402	202	31	26	P	S	3.004	-3.388	4.434	4.452	3.635	-4.071	-4.088	78.829	17.335	13.151
6403	202	31	26	P	S	12.520	2.326	-3.761	-1.494	-2.223	3.877	1.540	75.855	13.473	17.099
6404	202	31	26	P	S	15.608	-2.145	1.627	3.037	2.241	-1.534	-2.864	75.990	17.102	13.467
6405	202	31	26	P	S	17.696	1.029	5.212	-0.261	-1.008	-5.240	0.263	76.009	17.104	13.456
6406	202	31	26	P	S	42.304	1.029	-5.212	-0.261	-1.008	5.240	0.263	76.009	13.456	17.104
6407	202	31	26	P	S	44.392	-2.145	-1.627	3.037	2.241	1.534	-2.864	75.990	17.102	13.467
6408	202	31	26	P	S	47.480	2.326	3.761	-1.494	-2.223	-3.877	1.540	75.855	13.473	17.099
6409	202	31	26	P	S	56.996	-3.388	-4.434	4.452	3.635	4.071	-4.088	78.829	17.335	13.151
6410	202	31	26	P	S	60.000	5.011	0.978	-3.862	-4.554	-1.060	4.185	79.062	13.151	17.328
6411	202	31	26	P	S	63.005	-3.388	4.434	4.452	3.635	-4.071	-4.088	78.829	17.335	13.151
6412	202	31	26	P	S	72.520	2.326	-3.761	-1.494	-2.223	3.877	1.540	75.855	13.473	17.099
6413	202	31	26	P	S	75.609	-2.145	1.627	3.037	2.241	-1.534	-2.864	75.990	17.102	13.467
6414	202	31	26	P	S	77.696	1.029	5.212	-0.261	-1.008	-5.240	0.263	76.009	17.104	13.456
6415	202	31	26	P	S	102.304	1.029	-5.212	-0.261	-1.008	5.240	0.263	76.009	13.456	17.104
6416	202	31	26	P	S	104.392	-2.145	-1.627	3.037	2.241	1.534	-2.864	75.990	17.102	13.467
6417	202	31	26	P	S	107.480	2.326	3.761	-1.494	-2.223	-3.877	1.540	75.855	13.473	17.099
6418	202	31	26	P	S	116.996	-3.388	-4.434	4.452	3.635	4.071	-4.088	78.829	17.335	13.151
6419	202	31	26	P	W	0.000	5.011	-0.978	-3.862	-4.554	1.060	4.185	79.062	13.151	17.328
6420	202	31	26	P	W	3.004	-3.388	4.434	4.452	3.635	-4.071	-4.088	78.829	17.335	13.151
6421	202	31	26	P	W	12.520	2.326	-3.761	-1.494	-2.223	3.877	1.540	75.855	13.473	17.099
6422	202	31	26	P	W	15.608	-2.145	1.627	3.037	2.241	-1.534	-2.864	75.990	17.102	13.467
6423	202	31	26	P	W	17.696	1.029	5.212	-0.261	-1.008	-5.240	0.263	76.009	17.104	13.456
6424	202	31	26	P	W	42.304	1.029	-5.212	-0.261	-1.008	5.240	0.263	76.009	13.456	17.104
6425	202	31	26	P	W	44.392	-2.145	-1.627	3.037	2.241	1.534	-2.864	75.990	17.102	13.467
6426	202	31	26	P	W	47.480	2.326	3.761	-1.494	-2.223	-3.877	1.540	75.855	13.473	17.099

BL number	Atoms	γ-PC unit cells	WS_2 unit cells	γ-PC origin atom	WS_2 origin atom	Twist-angle (°)	γ-PC strain 1 (%)	γ-PC strain 2 (%)	γ-PC strain 3 (%)	WS_2 strain 1 (%)	WS_2 strain 2 (%)	WS_2 strain 3 (%)	γ (°)	|**a**|(Å)	|**b**|(Å)
6427	202	31	26	P	W	56.996	-3.388	-4.434	4.452	3.635	4.071	-4.088	78.829	17.335	13.151
6428	202	31	26	P	W	60.000	5.011	2.933	-3.862	-4.554	-3.179	4.185	71.445	13.826	17.070
6429	202	31	26	P	W	63.005	-3.388	4.434	4.452	3.635	-4.071	-4.088	78.829	17.335	13.151
6430	202	31	26	P	W	72.520	2.326	-3.761	-1.494	-2.223	3.877	1.540	75.855	13.473	17.099
6431	202	31	26	P	W	75.609	-2.145	1.627	3.037	2.241	-1.534	-2.864	75.990	17.102	13.467
6432	202	31	26	P	W	77.696	1.029	5.212	-0.261	-1.008	-5.240	0.263	76.009	17.104	13.456
6433	202	31	26	P	W	102.304	1.029	-5.212	-0.261	-1.008	5.240	0.263	76.009	13.456	17.104
6434	202	31	26	P	W	104.392	-2.145	-1.627	3.037	2.241	1.534	-2.864	75.990	17.102	13.467
6435	202	31	26	P	W	107.480	2.326	3.761	-1.494	-2.223	-3.877	1.540	75.855	13.473	17.099
6436	202	31	26	P	W	116.996	-3.388	-4.434	4.452	3.635	4.071	-4.088	78.829	17.335	13.151
6437	203	29	29	C	S	13.174	5.011	4.181	5.011	-4.554	-3.800	-4.555	69.280	13.151	18.586
6438	203	29	29	C	S	46.827	5.011	-4.181	5.011	-4.554	3.800	-4.555	69.280	13.151	18.586
6439	203	29	29	C	S	73.174	5.011	4.181	5.011	-4.554	-3.800	-4.555	69.280	13.151	18.586
6440	203	29	29	C	S	106.827	5.011	-4.181	5.011	-4.554	3.800	-4.555	69.280	13.151	18.586
6441	203	29	29	C	W	13.174	5.011	4.181	5.011	-4.554	-3.800	-4.555	69.280	13.151	18.586
6442	203	29	29	C	W	46.827	5.011	-4.181	5.011	-4.554	3.800	-4.555	69.280	13.151	18.586
6443	203	29	29	C	W	73.174	5.011	4.181	5.011	-4.554	-3.800	-4.555	69.280	13.151	18.586
6444	203	29	29	C	W	106.827	5.011	-4.181	5.011	-4.554	3.800	-4.555	69.280	13.151	18.586
6445	203	29	29	P	S	13.174	5.011	4.181	5.011	-4.554	-3.800	-4.555	69.280	13.151	18.586
6446	203	29	29	P	S	46.827	5.011	-4.181	5.011	-4.554	3.800	-4.555	69.280	13.151	18.586
6447	203	29	29	P	S	73.174	5.011	4.181	5.011	-4.554	-3.800	-4.555	69.280	13.151	18.586
6448	203	29	29	P	S	106.827	5.011	-4.181	5.011	-4.554	3.800	-4.555	69.280	13.151	18.586
6449	203	29	29	P	W	13.174	5.011	4.181	5.011	-4.554	-3.800	-4.555	69.280	13.151	18.586
6450	203	29	29	P	W	46.827	5.011	-4.181	5.011	-4.554	3.800	-4.555	69.280	13.151	18.586
6451	203	29	29	P	W	73.174	5.011	4.181	5.011	-4.554	-3.800	-4.555	69.280	13.151	18.586
6452	203	29	29	P	W	106.827	5.011	-4.181	5.011	-4.554	3.800	-4.555	69.280	13.151	18.586
6453	203	32	25	C	S	17.480	-4.685	-0.543	2.173	5.169	0.521	-2.082	82.631	14.491	15.502
6454	203	32	25	C	S	19.107	1.980	-2.944	-4.517	-1.905	3.236	4.966	70.477	15.504	15.244
6455	203	32	25	C	S	22.689	-4.497	3.247	1.957	4.941	-3.125	-1.883	78.428	11.961	19.011
6456	203	32	25	C	S	25.285	-4.781	-1.202	2.284	5.287	1.150	-2.184	83.579	16.641	13.474
6457	203	32	25	C	S	25.693	2.326	-0.437	-4.817	-2.223	0.484	5.331	83.622	13.473	16.641
6458	203	32	25	C	S	34.307	2.326	0.437	-4.817	-2.223	-0.484	5.331	83.622	13.473	16.641
6459	203	32	25	C	S	34.715	-4.781	1.203	2.284	5.287	-1.150	-2.184	83.579	16.641	13.474
6460	203	32	25	C	S	37.311	-4.497	-3.247	1.957	4.941	3.125	-1.883	78.429	11.961	19.011
6461	203	32	25	C	S	40.158	-4.723	1.842	2.217	5.216	-1.764	-2.123	70.488	15.242	15.509
6462	203	32	25	C	S	40.893	1.980	-2.208	-4.517	-1.905	2.427	4.966	82.535	15.504	14.491
6463	203	32	25	C	S	42.520	-4.685	0.543	2.173	5.169	-0.521	-2.082	82.631	14.491	15.502
6464	203	32	25	C	S	51.052	-0.599	-1.255	-2.142	0.606	1.312	2.238	70.521	15.901	14.844
6465	203	32	25	C	S	77.480	-4.685	-0.543	2.173	5.169	0.521	-2.082	82.630	14.491	15.502
6466	203	32	25	C	S	79.107	1.980	-2.944	-4.517	-1.905	3.236	4.966	70.477	15.504	15.244
6467	203	32	25	C	S	79.842	-4.723	-1.842	2.217	5.216	1.764	-2.123	70.488	15.242	15.509
6468	203	32	25	C	S	82.689	-4.497	3.247	1.957	4.941	-3.125	-1.883	78.429	11.961	19.011
6469	203	32	25	C	S	85.285	-4.781	-1.202	2.284	5.287	1.150	-2.184	83.579	16.641	13.474
6470	203	32	25	C	S	85.694	2.326	-0.437	-4.817	-2.223	0.484	5.331	83.622	13.473	16.641
6471	203	32	25	C	S	94.307	2.326	0.437	-4.817	-2.223	-0.484	5.331	83.622	13.473	16.641
6472	203	32	25	C	S	94.715	-4.781	1.203	2.284	5.287	-1.150	-2.184	83.579	16.641	13.474
6473	203	32	25	C	S	97.311	-4.497	-3.247	1.957	4.941	3.125	-1.883	78.428	11.961	19.011
6474	203	32	25	C	S	100.158	-4.723	1.842	2.217	5.216	-1.764	-2.123	70.488	15.242	15.509
6475	203	32	25	C	S	100.893	1.980	-2.208	-4.517	-1.905	2.427	4.966	82.535	15.504	14.491
6476	203	32	25	C	S	102.520	-4.685	0.543	2.173	5.169	-0.521	-2.082	82.631	14.491	15.502
6477	203	32	25	C	S	111.052	-0.599	-1.255	-2.142	0.606	1.312	2.238	70.521	15.901	14.844

BL number	Atoms	γ-PC unit cells	WS_2 unit cells	γ-PC origin atom	WS_2 origin atom	Twist-angle (°)	γ-PC strain 1 (%)	γ-PC strain 2 (%)	γ-PC strain 3 (%)	WS_2 strain 1 (%)	WS_2 strain 2 (%)	WS_2 strain 3 (%)	γ (°)	|**a**|(Å)	|**b**|(Å)
6478	203	32	25	C	W	17.480	-4.685	-0.543	2.173	5.169	0.521	-2.082	82.631	14.491	15.502
6479	203	32	25	C	W	19.107	1.980	-2.944	-4.517	-1.905	3.236	4.966	70.477	15.504	15.244
6480	203	32	25	C	W	19.842	-4.723	-1.842	2.217	5.216	1.764	-2.123	70.488	15.242	15.509
6481	203	32	25	C	W	22.689	-4.497	3.247	1.957	4.941	-3.125	-1.883	78.428	11.961	19.011
6482	203	32	25	C	W	25.285	-4.781	-1.202	2.284	5.287	1.150	-2.184	83.579	16.641	13.474
6483	203	32	25	C	W	25.693	2.326	-0.437	-4.817	-2.223	0.484	5.331	83.622	13.473	16.641
6484	203	32	25	C	W	34.307	2.326	0.437	-4.817	-2.223	-0.484	5.331	83.622	13.473	16.641
6485	203	32	25	C	W	34.715	-4.781	1.203	2.284	5.287	-1.150	-2.184	83.579	16.641	13.474
6486	203	32	25	C	W	37.311	-4.497	-3.247	1.957	4.941	3.125	-1.883	78.429	11.961	19.011
6487	203	32	25	C	W	40.158	-4.723	1.842	2.217	5.216	-1.764	-2.123	70.488	15.242	15.509
6488	203	32	25	C	W	40.893	1.980	-2.208	-4.517	-1.905	2.427	4.966	82.535	15.504	14.491
6489	203	32	25	C	W	42.520	-4.685	0.543	2.173	5.169	-0.521	-2.082	82.631	14.491	15.502
6490	203	32	25	C	W	51.052	-0.599	-1.255	-2.142	0.606	1.312	2.238	70.521	15.901	14.844
6491	203	32	25	C	W	77.480	-4.685	-0.543	2.173	5.169	0.521	-2.082	82.630	14.491	15.502
6492	203	32	25	C	W	79.107	1.980	-2.944	-4.517	-1.905	3.236	4.966	70.477	15.504	15.244
6493	203	32	25	C	W	82.689	-4.497	3.247	1.957	4.941	-3.125	-1.883	78.429	11.961	19.011
6494	203	32	25	C	W	85.285	-4.781	-1.202	2.284	5.287	1.150	-2.184	83.579	16.641	13.474
6495	203	32	25	C	W	85.694	2.326	-0.437	-4.817	-2.223	0.484	5.331	83.622	13.473	16.641
6496	203	32	25	C	W	94.307	2.326	0.437	-4.817	-2.223	-0.484	5.331	83.622	13.473	16.641
6497	203	32	25	C	W	94.715	-4.781	1.203	2.284	5.287	-1.150	-2.184	83.579	16.641	13.474
6498	203	32	25	C	W	97.311	-4.497	-3.247	1.957	4.941	3.125	-1.883	78.428	11.961	19.011
6499	203	32	25	C	W	100.158	-4.723	1.842	2.217	5.216	-1.764	-2.123	70.488	15.242	15.509
6500	203	32	25	C	W	100.893	1.980	-2.208	-4.517	-1.905	2.427	4.966	82.535	15.504	14.491
6501	203	32	25	C	W	102.520	-4.685	0.543	2.173	5.169	-0.521	-2.082	82.631	14.491	15.502
6502	203	32	25	C	W	111.052	-0.599	-1.255	-2.142	0.606	1.312	2.238	70.521	15.901	14.844
6503	203	32	25	P	S	17.480	-4.685	-0.543	2.173	5.169	0.521	-2.082	82.631	14.491	15.502
6504	203	32	25	P	S	19.107	1.980	-2.944	-4.517	-1.905	3.236	4.966	70.477	15.504	15.244
6505	203	32	25	P	S	19.842	-4.723	-1.842	2.217	5.216	1.764	-2.123	70.488	15.242	15.509
6506	203	32	25	P	S	22.689	-4.497	3.247	1.957	4.941	-3.125	-1.883	78.428	11.961	19.011
6507	203	32	25	P	S	25.285	-4.781	-1.202	2.284	5.287	1.150	-2.184	83.579	16.641	13.474
6508	203	32	25	P	S	25.693	2.326	-0.437	-4.817	-2.223	0.484	5.331	83.622	13.473	16.641
6509	203	32	25	P	S	34.307	2.326	0.437	-4.817	-2.223	-0.484	5.331	83.622	13.473	16.641
6510	203	32	25	P	S	34.715	-4.781	1.203	2.284	5.287	-1.150	-2.184	83.579	16.641	13.474
6511	203	32	25	P	S	37.311	-4.497	-3.247	1.957	4.941	3.125	-1.883	78.429	11.961	19.011
6512	203	32	25	P	S	40.158	-4.723	1.842	2.217	5.216	-1.764	-2.123	70.488	15.242	15.509
6513	203	32	25	P	S	40.893	1.980	-2.208	-4.517	-1.905	2.427	4.966	82.535	15.504	14.491
6514	203	32	25	P	S	42.520	-4.685	0.543	2.173	5.169	-0.521	-2.082	82.631	14.491	15.502
6515	203	32	25	P	S	51.052	-0.599	-1.255	-2.142	0.606	1.312	2.238	70.521	15.901	14.844
6516	203	32	25	P	S	77.480	-4.685	-0.543	2.173	5.169	0.521	-2.082	82.630	14.491	15.502
6517	203	32	25	P	S	79.107	1.980	-2.944	-4.517	-1.905	3.236	4.966	70.477	15.504	15.244
6518	203	32	25	P	S	82.689	-4.497	3.247	1.957	4.941	-3.125	-1.883	78.429	11.961	19.011
6519	203	32	25	P	S	85.285	-4.781	-1.202	2.284	5.287	1.150	-2.184	83.579	16.641	13.474
6520	203	32	25	P	S	85.694	2.326	-0.437	-4.817	-2.223	0.484	5.331	83.622	13.473	16.641
6521	203	32	25	P	S	94.307	2.326	0.437	-4.817	-2.223	-0.484	5.331	83.622	13.473	16.641
6522	203	32	25	P	S	94.715	-4.781	1.203	2.284	5.287	-1.150	-2.184	83.579	16.641	13.474
6523	203	32	25	P	S	97.311	-4.497	-3.247	1.957	4.941	3.125	-1.883	78.428	11.961	19.011
6524	203	32	25	P	S	100.158	-4.723	1.842	2.217	5.216	-1.764	-2.123	70.488	15.242	15.509
6525	203	32	25	P	S	100.893	1.980	2.944	-4.517	-1.905	-3.236	4.966	70.477	15.504	15.244
6526	203	32	25	P	S	111.052	-0.599	-1.255	-2.142	0.606	1.312	2.238	70.521	15.901	14.844
6527	203	32	25	P	W	17.480	-4.685	-0.543	2.173	5.169	0.521	-2.082	82.631	14.491	15.502
6528	203	32	25	P	W	19.107	1.980	-2.944	-4.517	-1.905	3.236	4.966	70.477	15.504	15.244

BL number	Atoms	γ-PC unit cells	WS_2 unit cells	γ-PC origin atom	WS_2 origin atom	Twist-angle (°)	γ-PC strain 1 (%)	γ-PC strain 2 (%)	γ-PC strain 3 (%)	WS_2 strain 1 (%)	WS_2 strain 2 (%)	WS_2 strain 3 (%)	γ (°)	|**a**|(Å)	|**b**|(Å)
6529	203	32	25	P	W	22.689	-4.497	3.247	1.957	4.941	-3.125	-1.883	78.428	11.961	19.011
6530	203	32	25	P	W	25.285	-4.781	-1.202	2.284	5.287	1.150	-2.184	83.579	16.641	13.474
6531	203	32	25	P	W	25.693	2.326	-0.437	-4.817	-2.223	0.484	5.331	83.622	13.473	16.641
6532	203	32	25	P	W	34.307	2.326	0.437	-4.817	-2.223	-0.484	5.331	83.622	13.473	16.641
6533	203	32	25	P	W	34.715	-4.781	1.203	2.284	5.287	-1.150	-2.184	83.579	16.641	13.474
6534	203	32	25	P	W	37.311	-4.497	-3.247	1.957	4.941	3.125	-1.883	78.429	11.961	19.011
6535	203	32	25	P	W	40.158	-4.723	1.842	2.217	5.216	-1.764	-2.123	70.488	15.242	15.509
6536	203	32	25	P	W	40.893	1.980	2.944	-4.517	-1.905	-3.236	4.966	70.477	15.504	15.244
6537	203	32	25	P	W	51.052	-0.599	-1.255	-2.142	0.606	1.312	2.238	70.521	15.901	14.844
6538	203	32	25	P	W	77.480	-4.685	-0.543	2.173	5.169	0.521	-2.082	82.630	14.491	15.502
6539	203	32	25	P	W	79.107	1.980	-2.944	-4.517	-1.905	3.236	4.966	70.477	15.504	15.244
6540	203	32	25	P	W	79.842	-4.723	-1.842	2.217	5.216	1.764	-2.123	70.488	15.242	15.509
6541	203	32	25	P	W	82.689	-4.497	3.247	1.957	4.941	-3.125	-1.883	78.429	11.961	19.011
6542	203	32	25	P	W	85.285	-4.781	-1.202	2.284	5.287	1.150	-2.184	83.579	16.641	13.474
6543	203	32	25	P	W	85.694	2.326	-0.437	-4.817	-2.223	0.484	5.331	83.622	13.473	16.641
6544	203	32	25	P	W	94.307	2.326	0.437	-4.817	-2.223	-0.484	5.331	83.622	13.473	16.641
6545	203	32	25	P	W	94.715	-4.781	1.203	2.284	5.287	-1.150	-2.184	83.579	16.641	13.474
6546	203	32	25	P	W	97.311	-4.497	-3.247	1.957	4.941	3.125	-1.883	78.428	11.961	19.011
6547	203	32	25	P	W	100.158	-4.723	1.842	2.217	5.216	-1.764	-2.123	70.488	15.242	15.509
6548	203	32	25	P	W	100.893	1.980	-2.208	-4.517	-1.905	2.427	4.966	82.535	15.504	14.491
6549	203	32	25	P	W	102.520	-4.685	0.543	2.173	5.169	-0.521	-2.082	82.631	14.491	15.502
6550	203	32	25	P	W	111.052	-0.599	-1.255	-2.142	0.606	1.312	2.238	70.521	15.901	14.844
6551	204	30	28	C	S	8.948	1.048	-3.576	5.330	-1.026	3.231	-4.816	85.607	17.420	13.143
6552	204	30	28	C	S	13.174	5.011	4.042	1.344	-4.554	-3.936	-1.308	85.439	13.151	17.410
6553	204	30	28	C	S	46.827	5.011	-4.042	1.344	-4.554	3.936	-1.308	85.439	13.151	17.410
6554	204	30	28	C	S	51.052	1.048	3.576	5.329	-1.026	-3.231	-4.816	85.607	17.420	13.143
6555	204	30	28	C	S	68.948	1.048	-3.576	5.330	-1.026	3.231	-4.816	85.607	17.420	13.143
6556	204	30	28	C	S	73.174	5.011	4.042	1.344	-4.554	-3.936	-1.308	85.439	13.151	17.410
6557	204	30	28	C	S	106.827	5.011	-4.042	1.344	-4.554	3.936	-1.308	85.439	13.151	17.410
6558	204	30	28	C	S	111.052	1.048	3.576	5.329	-1.026	-3.231	-4.816	85.607	17.420	13.143
6559	204	30	28	C	W	8.948	1.048	-3.576	5.330	-1.026	3.231	-4.816	85.607	17.420	13.143
6560	204	30	28	C	W	13.174	5.011	4.042	1.344	-4.554	-3.936	-1.308	85.439	13.151	17.410
6561	204	30	28	C	W	46.827	5.011	-4.042	1.344	-4.554	3.936	-1.308	85.439	13.151	17.410
6562	204	30	28	C	W	51.052	1.048	3.576	5.329	-1.026	-3.231	-4.816	85.607	17.420	13.143
6563	204	30	28	C	W	68.948	1.048	-3.576	5.330	-1.026	3.231	-4.816	85.607	17.420	13.143
6564	204	30	28	C	W	73.174	5.011	4.042	1.344	-4.554	-3.936	-1.308	85.439	13.151	17.410
6565	204	30	28	C	W	106.827	5.011	-4.042	1.344	-4.554	3.936	-1.308	85.439	13.151	17.410
6566	204	30	28	C	W	111.052	1.048	3.576	5.329	-1.026	-3.231	-4.816	85.607	17.420	13.143
6567	204	30	28	P	S	8.948	1.048	-3.576	5.330	-1.026	3.231	-4.816	85.607	17.420	13.143
6568	204	30	28	P	S	13.174	5.011	4.042	1.344	-4.554	-3.936	-1.308	85.439	13.151	17.410
6569	204	30	28	P	S	46.827	5.011	-4.042	1.344	-4.554	3.936	-1.308	85.439	13.151	17.410
6570	204	30	28	P	S	51.052	1.048	3.576	5.329	-1.026	-3.231	-4.816	85.607	17.420	13.143
6571	204	30	28	P	S	68.948	1.048	-3.576	5.330	-1.026	3.231	-4.816	85.607	17.420	13.143
6572	204	30	28	P	S	73.174	5.011	4.042	1.344	-4.554	-3.936	-1.308	85.439	13.151	17.410
6573	204	30	28	P	S	106.827	5.011	-4.042	1.344	-4.554	3.936	-1.308	85.439	13.151	17.410
6574	204	30	28	P	S	111.052	1.048	3.576	5.329	-1.026	-3.231	-4.816	85.607	17.420	13.143
6575	204	30	28	P	W	8.948	1.048	-3.576	5.330	-1.026	3.231	-4.816	85.607	17.420	13.143
6576	204	30	28	P	W	13.174	5.011	4.042	1.344	-4.554	-3.936	-1.308	85.439	13.151	17.410
6577	204	30	28	P	W	46.827	5.011	-4.042	1.344	-4.554	3.936	-1.308	85.439	13.151	17.410
6578	204	30	28	P	W	51.052	1.048	3.576	5.329	-1.026	-3.231	-4.816	85.607	17.420	13.143
6579	204	30	28	P	W	68.948	1.048	-3.576	5.330	-1.026	3.231	-4.816	85.607	17.420	13.143

BL number	Atoms	γ-PC unit cells	WS_2 unit cells	γ-PC origin atom	WS_2 origin atom	Twist-angle (°)	γ-PC strain 1 (%)	γ-PC strain 2 (%)	γ-PC strain 3 (%)	WS_2 strain 1 (%)	WS_2 strain 2 (%)	WS_2 strain 3 (%)	γ (°)	|**a**|(Å)	|**b**|(Å)
6580	204	30	28	P	W	73.174	5.011	4.042	1.344	-4.554	-3.936	-1.308	85.439	13.151	17.410
6581	204	30	28	P	W	106.827	5.011	-4.042	1.344	-4.554	3.936	-1.308	85.439	13.151	17.410
6582	204	30	28	P	W	111.052	1.048	3.576	5.329	-1.026	-3.231	-4.816	85.607	17.420	13.143
6583	204	33	24	C	S	4.715	-3.007	-1.980	-3.166	3.200	2.114	3.379	65.644	16.951	14.349
6584	204	33	24	C	S	6.587	-3.853	1.195	-2.307	4.175	-1.253	2.419	65.645	14.354	16.946
6585	204	33	24	C	S	10.893	-1.983	-2.572	-4.165	2.064	2.806	4.544	88.463	12.906	17.178
6586	204	33	24	C	S	13.898	-4.228	2.513	-1.916	4.619	-2.613	1.993	88.532	17.184	12.901
6587	204	33	24	C	S	19.107	-1.485	-1.108	-4.635	1.530	1.221	5.109	81.411	14.708	15.241
6588	204	33	24	C	S	40.158	-4.723	-0.119	-1.391	5.216	0.122	1.431	81.417	15.242	14.707
6589	204	33	24	C	S	46.102	-4.228	-2.513	-1.916	4.619	2.613	1.993	88.532	17.184	12.901
6590	204	33	24	C	S	49.107	-1.983	2.572	-4.165	2.064	-2.806	4.544	88.462	12.906	17.178
6591	204	33	24	C	S	53.413	-3.853	-1.195	-2.307	4.175	1.253	2.419	65.645	14.354	16.946
6592	204	33	24	C	S	53.822	-2.145	-0.489	-4.009	2.241	0.532	4.359	65.618	16.951	14.354
6593	204	33	24	C	S	55.285	-3.007	1.980	-3.166	3.200	-2.114	3.379	65.644	16.951	14.349
6594	204	33	24	C	S	64.715	-3.007	-1.980	-3.166	3.200	2.114	3.379	65.644	16.951	14.349
6595	204	33	24	C	S	66.178	-2.145	0.489	-4.009	2.241	-0.532	4.359	65.618	14.354	16.951
6596	204	33	24	C	S	66.587	-3.853	1.195	-2.307	4.175	-1.253	2.419	65.645	14.354	16.946
6597	204	33	24	C	S	70.893	-1.983	-2.572	-4.165	2.064	2.806	4.544	88.462	12.906	17.178
6598	204	33	24	C	S	73.898	-4.228	2.513	-1.916	4.619	-2.613	1.993	88.532	17.184	12.901
6599	204	33	24	C	S	79.107	-1.485	-1.108	-4.635	1.530	1.221	5.109	81.411	14.708	15.241
6600	204	33	24	C	S	100.158	-4.723	-0.119	-1.391	5.216	0.122	1.431	81.417	15.242	14.707
6601	204	33	24	C	S	100.893	-1.485	1.108	-4.635	1.530	-1.221	5.109	81.411	14.708	15.241
6602	204	33	24	C	S	106.102	-4.228	-2.513	-1.916	4.619	2.613	1.993	88.532	17.184	12.901
6603	204	33	24	C	S	109.107	-1.983	2.572	-4.165	2.064	-2.806	4.544	88.463	12.906	17.178
6604	204	33	24	C	S	113.822	-2.145	-0.489	-4.009	2.241	0.532	4.359	65.619	16.951	14.354
6605	204	33	24	C	S	115.285	-3.007	1.980	-3.166	3.200	-2.114	3.379	65.644	16.951	14.349
6606	204	33	24	C	W	4.715	-3.007	-1.980	-3.166	3.200	2.114	3.379	65.644	16.951	14.349
6607	204	33	24	C	W	6.178	-2.145	0.489	-4.009	2.241	-0.532	4.359	65.618	14.354	16.951
6608	204	33	24	C	W	6.587	-3.853	1.195	-2.307	4.175	-1.253	2.419	65.645	14.354	16.946
6609	204	33	24	C	W	10.893	-1.983	-2.572	-4.165	2.064	2.806	4.544	88.463	12.906	17.178
6610	204	33	24	C	W	13.898	-4.228	2.513	-1.916	4.619	-2.613	1.993	88.532	17.184	12.901
6611	204	33	24	C	W	40.893	-1.485	1.108	-4.635	1.530	-1.221	5.109	81.411	14.708	15.241
6612	204	33	24	C	W	46.102	-4.228	-2.513	-1.916	4.619	2.613	1.993	88.532	17.184	12.901
6613	204	33	24	C	W	49.107	-1.983	2.572	-4.165	2.064	-2.806	4.544	88.462	12.906	17.178
6614	204	33	24	C	W	53.822	-2.145	-0.489	-4.009	2.241	0.532	4.359	65.618	16.951	14.354
6615	204	33	24	C	W	55.285	-3.007	1.980	-3.166	3.200	-2.114	3.379	65.644	16.951	14.349
6616	204	33	24	C	W	64.715	-3.007	-1.980	-3.166	3.200	2.114	3.379	65.644	16.951	14.349
6617	204	33	24	C	W	66.587	-3.853	1.195	-2.307	4.175	-1.253	2.419	65.645	14.354	16.946
6618	204	33	24	C	W	70.893	-1.983	-2.572	-4.165	2.064	2.806	4.544	88.462	12.906	17.178
6619	204	33	24	C	W	73.898	-4.228	2.513	-1.916	4.619	-2.613	1.993	88.532	17.184	12.901
6620	204	33	24	C	W	79.107	-1.485	-1.108	-4.635	1.530	1.221	5.109	81.411	14.708	15.241
6621	204	33	24	C	W	106.102	-4.228	-2.513	-1.916	4.619	2.613	1.993	88.532	17.184	12.901
6622	204	33	24	C	W	109.107	-1.983	2.572	-4.165	2.064	-2.806	4.544	88.463	12.906	17.178
6623	204	33	24	C	W	113.413	-3.853	-1.195	-2.307	4.175	1.253	2.419	65.645	14.354	16.946
6624	204	33	24	C	W	113.822	-2.145	-0.489	-4.009	2.241	0.532	4.359	65.619	16.951	14.354
6625	204	33	24	C	W	115.285	-3.007	1.980	-3.166	3.200	-2.114	3.379	65.644	16.951	14.349
6626	204	33	24	P	S	4.715	-3.007	-1.980	-3.166	3.200	2.114	3.379	65.644	16.951	14.349
6627	204	33	24	P	S	6.178	-2.145	0.489	-4.009	2.241	-0.532	4.359	65.618	14.354	16.951
6628	204	33	24	P	S	6.587	-3.853	1.195	-2.307	4.175	-1.253	2.419	65.645	14.354	16.946
6629	204	33	24	P	S	19.107	-1.485	-1.108	-4.635	1.530	1.221	5.109	81.411	14.708	15.241
6630	204	33	24	P	S	40.158	-4.723	-0.119	-1.391	5.216	0.122	1.431	81.417	15.242	14.707

BL number	Atoms	γ-PC unit cells	WS_2 unit cells	γ-PC origin atom	WS_2 origin atom	Twist-angle (°)	γ-PC strain 1 (%)	γ-PC strain 2 (%)	γ-PC strain 3 (%)	WS_2 strain 1 (%)	WS_2 strain 2 (%)	WS_2 strain 3 (%)	γ (°)	$\lvert\mathbf{a}\rvert$(Å)	$\lvert\mathbf{b}\rvert$(Å)
6631	204	33	24	P	S	40.893	-1.485	1.108	-4.635	1.530	-1.221	5.109	81.411	14.708	15.241
6632	204	33	24	P	S	53.822	-2.145	-0.489	-4.009	2.241	0.532	4.359	65.618	16.951	14.354
6633	204	33	24	P	S	55.285	-3.007	1.980	-3.166	3.200	-2.114	3.379	65.644	16.951	14.349
6634	204	33	24	P	S	64.715	-3.007	-1.980	-3.166	3.200	2.114	3.379	65.644	16.951	14.349
6635	204	33	24	P	S	66.587	-3.853	1.195	-2.307	4.175	-1.253	2.419	65.645	14.354	16.946
6636	204	33	24	P	S	79.107	-1.485	-1.108	-4.635	1.530	1.221	5.109	81.411	14.708	15.241
6637	204	33	24	P	S	100.158	-4.723	-0.119	-1.391	5.216	0.122	1.431	81.417	15.242	14.707
6638	204	33	24	P	S	113.413	-3.853	-1.195	-2.307	4.175	1.253	2.419	65.645	14.354	16.946
6639	204	33	24	P	S	113.822	-2.145	-0.489	-4.009	2.241	0.532	4.359	65.619	16.951	14.354
6640	204	33	24	P	S	115.285	-3.007	1.980	-3.166	3.200	-2.114	3.379	65.644	16.951	14.349
6641	204	33	24	P	W	4.715	-3.007	-1.980	-3.166	3.200	2.114	3.379	65.644	16.951	14.349
6642	204	33	24	P	W	6.587	-3.853	1.195	-2.307	4.175	-1.253	2.419	65.645	14.354	16.946
6643	204	33	24	P	W	19.107	-1.485	-1.108	-4.635	1.530	1.221	5.109	81.411	14.708	15.241
6644	204	33	24	P	W	53.413	-3.853	-1.195	-2.307	4.175	1.253	2.419	65.645	14.354	16.946
6645	204	33	24	P	W	53.822	-2.145	-0.489	-4.009	2.241	0.532	4.359	65.618	16.951	14.354
6646	204	33	24	P	W	55.285	-3.007	1.980	-3.166	3.200	-2.114	3.379	65.644	16.951	14.349
6647	204	33	24	P	W	64.715	-3.007	-1.980	-3.166	3.200	2.114	3.379	65.644	16.951	14.349
6648	204	33	24	P	W	66.178	-2.145	0.489	-4.009	2.241	-0.532	4.359	65.618	14.354	16.951
6649	204	33	24	P	W	66.587	-3.853	1.195	-2.307	4.175	-1.253	2.419	65.645	14.354	16.946
6650	204	33	24	P	W	100.893	-1.485	1.108	-4.635	1.530	-1.221	5.109	81.411	14.708	15.241
6651	204	33	24	P	W	109.107	-1.983	2.572	-4.165	2.064	-2.806	4.544	88.463	12.906	17.178
6652	204	33	24	P	W	113.822	-2.145	-0.489	-4.009	2.241	0.532	4.359	65.619	16.951	14.354
6653	204	33	24	P	W	115.285	-3.007	1.980	-3.166	3.200	-2.114	3.379	65.644	16.951	14.349
6654	205	31	27	C	S	21.052	1.339	0.000	1.339	-1.304	0.000	-1.304	60.000	16.211	16.211
6655	205	31	27	C	S	38.948	1.339	0.000	1.339	-1.304	0.000	-1.304	60.000	16.211	16.211
6656	205	31	27	C	S	81.052	1.339	0.000	1.339	-1.304	0.000	-1.304	60.000	16.211	16.211
6657	205	31	27	C	S	98.948	1.339	0.000	1.339	-1.304	0.000	-1.304	60.000	16.211	16.211
6658	205	31	27	C	W	21.052	1.339	0.000	1.339	-1.304	0.000	-1.304	60.000	16.211	16.211
6659	205	31	27	C	W	38.948	1.339	0.000	1.339	-1.304	0.000	-1.304	60.000	16.211	16.211
6660	205	31	27	C	W	81.052	1.339	0.000	1.339	-1.304	0.000	-1.304	60.000	16.211	16.211
6661	205	31	27	C	W	98.948	1.339	0.000	1.339	-1.304	0.000	-1.304	60.000	16.211	16.211
6662	205	31	27	C	S	13.174	5.011	3.911	-2.087	-4.554	-4.082	2.178	77.608	13.151	17.740
6663	205	31	27	C	S	27.126	1.029	1.932	1.652	-1.008	-1.871	-1.599	74.438	17.539	13.471
6664	205	31	27	C	S	34.233	0.353	0.482	2.345	-0.351	-0.461	-2.240	74.430	17.539	13.473
6665	205	31	27	C	S	34.307	2.326	0.602	0.371	-2.223	-0.598	-0.369	74.421	13.473	17.539
6666	205	31	27	C	S	46.827	5.011	-3.911	-2.087	-4.554	4.082	2.178	77.608	13.151	17.740
6667	205	31	27	C	S	73.174	5.011	3.911	-2.087	-4.554	-4.082	2.178	77.608	13.151	17.740
6668	205	31	27	C	S	85.694	2.326	-0.602	0.371	-2.223	0.597	-0.369	74.421	13.473	17.539
6669	205	31	27	C	S	87.126	1.029	1.932	1.652	-1.008	-1.871	-1.599	74.439	17.539	13.471
6670	205	31	27	C	S	94.233	0.353	0.482	2.345	-0.351	-0.461	-2.240	74.430	17.539	13.473
6671	205	31	27	C	S	106.827	5.011	-3.911	-2.087	-4.554	4.082	2.178	77.608	13.151	17.740
6672	205	31	27	C	W	13.174	5.011	3.911	-2.087	-4.554	-4.082	2.178	77.608	13.151	17.740
6673	205	31	27	C	W	25.693	2.326	-0.602	0.371	-2.223	0.597	-0.369	74.421	13.473	17.539
6674	205	31	27	C	W	27.126	1.029	1.932	1.652	-1.008	-1.871	-1.599	74.438	17.539	13.471
6675	205	31	27	C	W	34.233	0.353	0.482	2.345	-0.351	-0.461	-2.240	74.430	17.539	13.473
6676	205	31	27	C	W	46.827	5.011	-3.911	-2.087	-4.554	4.082	2.178	77.608	13.151	17.740
6677	205	31	27	C	W	73.174	5.011	3.911	-2.087	-4.554	-4.082	2.178	77.608	13.151	17.740
6678	205	31	27	C	W	87.126	1.029	1.932	1.652	-1.008	-1.871	-1.599	74.439	17.539	13.471
6679	205	31	27	C	W	94.233	0.353	0.482	2.345	-0.351	-0.461	-2.240	74.430	17.539	13.473
6680	205	31	27	C	W	94.307	2.326	0.602	0.371	-2.223	-0.598	-0.369	74.421	13.473	17.539
6681	205	31	27	C	W	106.827	5.011	-3.911	-2.087	-4.554	4.082	2.178	77.608	13.151	17.740

BL number	Atoms	γ-PC unit cells	WS_2 unit cells	γ-PC origin atom	WS_2 origin atom	Twist-angle (°)	γ-PC strain 1 (%)	γ-PC strain 2 (%)	γ-PC strain 3 (%)	WS_2 strain 1 (%)	WS_2 strain 2 (%)	WS_2 strain 3 (%)	γ (°)	|**a**|(Å)	|**b**|(Å)
6682	205	31	27	P	S	13.174	5.011	3.911	-2.087	-4.554	-4.082	2.178	77.608	13.151	17.740
6683	205	31	27	P	S	25.693	2.326	-0.602	0.371	-2.223	0.597	-0.369	74.421	13.473	17.539
6684	205	31	27	P	S	27.126	1.029	1.932	1.652	-1.008	-1.871	-1.599	74.438	17.539	13.471
6685	205	31	27	P	S	34.233	0.353	0.482	2.345	-0.351	-0.461	-2.240	74.430	17.539	13.473
6686	205	31	27	P	S	46.827	5.011	-3.911	-2.087	-4.554	4.082	2.178	77.608	13.151	17.740
6687	205	31	27	P	S	73.174	5.011	3.911	-2.087	-4.554	-4.082	2.178	77.608	13.151	17.740
6688	205	31	27	P	S	87.126	1.029	1.932	1.652	-1.008	-1.871	-1.599	74.439	17.539	13.471
6689	205	31	27	P	S	94.233	0.353	0.482	2.345	-0.351	-0.461	-2.240	74.430	17.539	13.473
6690	205	31	27	P	S	94.307	2.326	0.602	0.371	-2.223	-0.598	-0.369	74.421	13.473	17.539
6691	205	31	27	P	S	106.827	5.011	-3.911	-2.087	-4.554	4.082	2.178	77.608	13.151	17.740
6692	205	31	27	P	W	13.174	5.011	3.911	-2.087	-4.554	-4.082	2.178	77.608	13.151	17.740
6693	205	31	27	P	W	27.126	1.029	1.932	1.652	-1.008	-1.871	-1.599	74.438	17.539	13.471
6694	205	31	27	P	W	34.233	0.353	0.482	2.345	-0.351	-0.461	-2.240	74.430	17.539	13.473
6695	205	31	27	P	W	34.307	2.326	0.602	0.371	-2.223	-0.598	-0.369	74.421	13.473	17.539
6696	205	31	27	P	W	46.827	5.011	-3.911	-2.087	-4.554	4.082	2.178	77.608	13.151	17.740
6697	205	31	27	P	W	73.174	5.011	3.911	-2.087	-4.554	-4.082	2.178	77.608	13.151	17.740
6698	205	31	27	P	W	85.694	2.326	-0.602	0.371	-2.223	0.597	-0.369	74.421	13.473	17.539
6699	205	31	27	P	W	87.126	1.029	1.932	1.652	-1.008	-1.871	-1.599	74.439	17.539	13.471
6700	205	31	27	P	W	94.233	0.353	0.482	2.345	-0.351	-0.461	-2.240	74.430	17.539	13.473
6701	205	31	27	P	W	106.827	5.011	-3.911	-2.087	-4.554	4.082	2.178	77.608	13.151	17.740
6702	205	34	23	C	S	17.480	-4.685	-1.959	-4.825	5.169	2.169	5.340	86.875	14.491	15.239
6703	205	34	23	C	S	19.842	-4.723	1.965	-4.786	5.216	-2.173	5.293	86.874	15.242	14.489
6704	205	34	23	C	S	40.158	-4.723	-1.965	-4.786	5.216	2.173	5.293	86.874	15.242	14.489
6705	205	34	23	C	S	42.520	-4.685	1.959	-4.825	5.169	-2.169	5.340	86.875	14.491	15.239
6706	205	34	23	C	S	77.480	-4.685	-1.959	-4.825	5.169	2.169	5.340	86.875	14.491	15.239
6707	205	34	23	C	S	79.842	-4.723	1.965	-4.786	5.216	-2.173	5.293	86.874	15.242	14.489
6708	205	34	23	C	S	100.158	-4.723	-1.965	-4.786	5.216	2.173	5.293	86.874	15.242	14.489
6709	205	34	23	C	S	102.520	-4.685	1.959	-4.825	5.169	-2.169	5.340	86.875	14.491	15.239
6710	205	34	23	C	W	17.480	-4.685	-1.959	-4.825	5.169	2.169	5.340	86.875	14.491	15.239
6711	205	34	23	C	W	19.842	-4.723	1.965	-4.786	5.216	-2.173	5.293	86.874	15.242	14.489
6712	205	34	23	C	W	40.158	-4.723	-1.965	-4.786	5.216	2.173	5.293	86.874	15.242	14.489
6713	205	34	23	C	W	42.520	-4.685	1.959	-4.825	5.169	-2.169	5.340	86.875	14.491	15.239
6714	205	34	23	C	W	77.480	-4.685	-1.959	-4.825	5.169	2.169	5.340	86.875	14.491	15.239
6715	205	34	23	C	W	79.842	-4.723	1.965	-4.786	5.216	-2.173	5.293	86.874	15.242	14.489
6716	205	34	23	C	W	100.158	-4.723	-1.965	-4.786	5.216	2.173	5.293	86.874	15.242	14.489
6717	205	34	23	C	W	102.520	-4.685	1.959	-4.825	5.169	-2.169	5.340	86.875	14.491	15.239
6718	205	34	23	P	S	17.480	-4.685	-1.959	-4.825	5.169	2.169	5.340	86.875	14.491	15.239
6719	205	34	23	P	S	19.842	-4.723	1.965	-4.786	5.216	-2.173	5.293	86.874	15.242	14.489
6720	205	34	23	P	S	40.158	-4.723	-1.965	-4.786	5.216	2.173	5.293	86.874	15.242	14.489
6721	205	34	23	P	S	42.520	-4.685	1.959	-4.825	5.169	-2.169	5.340	86.875	14.491	15.239
6722	205	34	23	P	S	77.480	-4.685	-1.959	-4.825	5.169	2.169	5.340	86.875	14.491	15.239
6723	205	34	23	P	S	79.842	-4.723	1.965	-4.786	5.216	-2.173	5.293	86.874	15.242	14.489
6724	205	34	23	P	S	100.158	-4.723	-1.965	-4.786	5.216	2.173	5.293	86.874	15.242	14.489
6725	205	34	23	P	S	102.520	-4.685	1.959	-4.825	5.169	-2.169	5.340	86.875	14.491	15.239
6726	205	34	23	P	W	17.480	-4.685	-1.959	-4.825	5.169	2.169	5.340	86.875	14.491	15.239
6727	205	34	23	P	W	19.842	-4.723	1.965	-4.786	5.216	-2.173	5.293	86.874	15.242	14.489
6728	205	34	23	P	W	40.158	-4.723	-1.965	-4.786	5.216	2.173	5.293	86.874	15.242	14.489
6729	205	34	23	P	W	42.520	-4.685	1.959	-4.825	5.169	-2.169	5.340	86.875	14.491	15.239
6730	205	34	23	P	W	77.480	-4.685	-1.959	-4.825	5.169	2.169	5.340	86.875	14.491	15.239
6731	205	34	23	P	W	79.842	-4.723	1.965	-4.786	5.216	-2.173	5.293	86.874	15.242	14.489
6732	205	34	23	P	W	100.158	-4.723	-1.965	-4.786	5.216	2.173	5.293	86.874	15.242	14.489

BL number	Atoms	γ-PC unit cells	WS_2 unit cells	γ-PC origin atom	WS_2 origin atom	Twist-angle (°)	γ-PC strain 1 (%)	γ-PC strain 2 (%)	γ-PC strain 3 (%)	WS_2 strain 1 (%)	WS_2 strain 2 (%)	WS_2 strain 3 (%)	γ (°)	|**a**|(Å)	|**b**|(Å)
6733	205	34	23	P	W	102.520	-4.685	1.959	-4.825	5.169	-2.169	5.340	86.875	14.491	15.239
6734	206	32	26	C	S	17.480	-4.685	4.525	4.259	5.169	-4.170	-3.925	74.014	14.491	16.320
6735	206	32	26	C	S	25.050	-0.955	-5.188	0.133	0.973	5.174	-0.133	69.275	16.360	14.828
6736	206	32	26	C	S	28.055	2.281	0.066	-2.970	-2.182	-0.070	3.158	69.209	16.362	14.843
6737	206	32	26	C	S	30.000	-2.359	-3.523	1.611	2.476	3.413	-1.561	69.122	14.845	16.364
6738	206	32	26	C	S	31.945	2.281	-0.066	-2.970	-2.182	0.070	3.158	69.209	16.362	14.843
6739	206	32	26	C	S	34.950	-0.955	5.188	0.133	0.973	-5.174	-0.133	69.275	14.828	16.360
6740	206	32	26	C	S	42.520	-4.685	-4.525	4.259	5.169	4.170	-3.925	74.014	14.491	16.320
6741	206	32	26	C	S	77.480	-4.685	4.525	4.259	5.169	-4.170	-3.925	74.014	14.491	16.320
6742	206	32	26	C	S	85.050	-0.955	-5.188	0.133	0.973	5.174	-0.133	69.275	16.360	14.828
6743	206	32	26	C	S	88.055	2.281	0.066	-2.970	-2.182	-0.070	3.158	69.209	16.362	14.843
6744	206	32	26	C	S	90.000	-2.359	3.523	1.611	2.476	-3.413	-1.561	69.122	14.845	16.364
6745	206	32	26	C	S	91.945	2.281	-0.066	-2.970	-2.182	0.070	3.158	69.209	16.362	14.843
6746	206	32	26	C	S	94.950	-0.955	5.188	0.133	0.973	-5.174	-0.133	69.275	14.828	16.360
6747	206	32	26	C	S	102.520	-4.685	-4.525	4.259	5.169	4.170	-3.925	74.014	14.491	16.320
6748	206	32	26	C	W	17.480	-4.685	4.525	4.259	5.169	-4.170	-3.925	74.014	14.491	16.320
6749	206	32	26	C	W	25.050	-0.955	-5.188	0.133	0.973	5.174	-0.133	69.275	16.360	14.828
6750	206	32	26	C	W	28.055	2.281	0.066	-2.970	-2.182	-0.070	3.158	69.209	16.362	14.843
6751	206	32	26	C	W	30.000	-2.359	-3.523	1.611	2.476	3.413	-1.561	69.122	14.845	16.364
6752	206	32	26	C	W	31.945	2.281	-0.066	-2.970	-2.182	0.070	3.158	69.209	16.362	14.843
6753	206	32	26	C	W	34.950	-0.955	5.188	0.133	0.973	-5.174	-0.133	69.275	14.828	16.360
6754	206	32	26	C	W	42.520	-4.685	-4.525	4.259	5.169	4.170	-3.925	74.014	14.491	16.320
6755	206	32	26	C	W	77.480	-4.685	4.525	4.259	5.169	-4.170	-3.925	74.014	14.491	16.320
6756	206	32	26	C	W	85.050	-0.955	-5.188	0.133	0.973	5.174	-0.133	69.275	16.360	14.828
6757	206	32	26	C	W	88.055	2.281	0.066	-2.970	-2.182	-0.070	3.158	69.209	16.362	14.843
6758	206	32	26	C	W	90.000	-2.359	3.523	1.611	2.476	-3.413	-1.561	69.122	14.845	16.364
6759	206	32	26	C	W	91.945	2.281	-0.066	-2.970	-2.182	0.070	3.158	69.209	16.362	14.843
6760	206	32	26	C	W	94.950	-0.955	5.188	0.133	0.973	-5.174	-0.133	69.275	14.828	16.360
6761	206	32	26	C	W	102.520	-4.685	-4.525	4.259	5.169	4.170	-3.925	74.014	14.491	16.320
6762	206	32	26	P	S	17.480	-4.685	4.525	4.259	5.169	-4.170	-3.925	74.014	14.491	16.320
6763	206	32	26	P	S	25.050	-0.955	-5.188	0.133	0.973	5.174	-0.133	69.275	16.360	14.828
6764	206	32	26	P	S	28.055	2.281	0.066	-2.970	-2.182	-0.070	3.158	69.209	16.362	14.843
6765	206	32	26	P	S	30.000	-2.359	-3.523	1.611	2.476	3.413	-1.561	69.122	14.845	16.364
6766	206	32	26	P	S	31.945	2.281	-0.066	-2.970	-2.182	0.070	3.158	69.209	16.362	14.843
6767	206	32	26	P	S	34.950	-0.955	5.188	0.133	0.973	-5.174	-0.133	69.275	14.828	16.360
6768	206	32	26	P	S	42.520	-4.685	-4.525	4.259	5.169	4.170	-3.925	74.014	14.491	16.320
6769	206	32	26	P	S	77.480	-4.685	4.525	4.259	5.169	-4.170	-3.925	74.014	14.491	16.320
6770	206	32	26	P	S	85.050	-0.955	-5.188	0.133	0.973	5.174	-0.133	69.275	16.360	14.828
6771	206	32	26	P	S	88.055	2.281	0.066	-2.970	-2.182	-0.070	3.158	69.209	16.362	14.843
6772	206	32	26	P	S	90.000	-2.359	3.523	1.611	2.476	-3.413	-1.561	69.122	14.845	16.364
6773	206	32	26	P	S	91.945	2.281	-0.066	-2.970	-2.182	0.070	3.158	69.209	16.362	14.843
6774	206	32	26	P	S	94.950	-0.955	5.188	0.133	0.973	-5.174	-0.133	69.275	14.828	16.360
6775	206	32	26	P	S	102.520	-4.685	-4.525	4.259	5.169	4.170	-3.925	74.014	14.491	16.320
6776	206	32	26	P	W	17.480	-4.685	4.525	4.259	5.169	-4.170	-3.925	74.014	14.491	16.320
6777	206	32	26	P	W	25.050	-0.955	-5.188	0.133	0.973	5.174	-0.133	69.275	16.360	14.828
6778	206	32	26	P	W	28.055	2.281	0.066	-2.970	-2.182	-0.070	3.158	69.209	16.362	14.843
6779	206	32	26	P	W	30.000	-2.359	-3.523	1.611	2.476	3.413	-1.561	69.122	14.845	16.364
6780	206	32	26	P	W	31.945	2.281	-0.066	-2.970	-2.182	0.070	3.158	69.209	16.362	14.843
6781	206	32	26	P	W	34.950	-0.955	5.188	0.133	0.973	-5.174	-0.133	69.275	14.828	16.360
6782	206	32	26	P	W	42.520	-4.685	-4.525	4.259	5.169	4.170	-3.925	74.014	14.491	16.320
6783	206	32	26	P	W	77.480	-4.685	4.525	4.259	5.169	-4.170	-3.925	74.014	14.491	16.320

BL number	Atoms	γ-PC unit cells	WS_2 unit cells	γ-PC origin atom	WS_2 origin atom	Twist-angle (°)	γ-PC strain 1 (%)	γ-PC strain 2 (%)	γ-PC strain 3 (%)	WS_2 strain 1 (%)	WS_2 strain 2 (%)	WS_2 strain 3 (%)	γ (°)	|**a**|(Å)	|**b**|(Å)
6784	206	32	26	P	W	85.050	-0.955	-5.188	0.133	0.973	5.174	-0.133	69.275	16.360	14.828
6785	206	32	26	P	W	88.055	2.281	0.066	-2.970	-2.182	-0.070	3.158	69.209	16.362	14.843
6786	206	32	26	P	W	90.000	-2.359	3.523	1.611	2.476	-3.413	-1.561	69.122	14.845	16.364
6787	206	32	26	P	W	91.945	2.281	-0.066	-2.970	-2.182	0.070	3.158	69.209	16.362	14.843
6788	206	32	26	P	W	94.950	-0.955	5.188	0.133	0.973	-5.174	-0.133	69.275	14.828	16.360
6789	206	32	26	P	W	102.520	-4.685	-4.525	4.259	5.169	4.170	-3.925	74.014	14.491	16.320
6790	207	30	29	C	S	27.796	5.011	3.031	3.177	-4.554	-2.850	-2.987	71.173	13.477	18.217
6791	207	30	29	C	S	32.204	5.011	-3.031	3.177	-4.554	2.850	-2.987	71.173	18.217	13.477
6792	207	30	29	C	S	87.796	5.011	3.031	3.177	-4.554	-2.850	-2.987	71.173	13.477	18.217
6793	207	30	29	C	S	92.204	5.011	-3.031	3.177	-4.554	2.850	-2.987	71.173	18.217	13.477
6794	207	30	29	C	W	27.796	5.011	3.031	3.177	-4.554	-2.850	-2.987	71.173	13.477	18.217
6795	207	30	29	C	W	32.204	5.011	-3.031	3.177	-4.554	2.850	-2.987	71.173	18.217	13.477
6796	207	30	29	C	W	87.796	5.011	3.031	3.177	-4.554	-2.850	-2.987	71.173	13.477	18.217
6797	207	30	29	C	W	92.204	5.011	-3.031	3.177	-4.554	2.850	-2.987	71.173	18.217	13.477
6798	207	30	29	P	S	27.796	5.011	3.031	3.177	-4.554	-2.850	-2.987	71.173	13.477	18.217
6799	207	30	29	P	S	92.204	5.011	-3.031	3.177	-4.554	2.850	-2.987	71.173	18.217	13.477
6800	207	30	29	P	W	32.204	5.011	-3.031	3.177	-4.554	2.850	-2.987	71.173	18.217	13.477
6801	207	30	29	P	W	87.796	5.011	3.031	3.177	-4.554	-2.850	-2.987	71.173	13.477	18.217
6802	207	33	25	C	S	6.587	-3.853	-1.594	-0.320	4.175	1.604	0.322	82.261	14.354	15.897
6803	207	33	25	C	S	8.948	-0.599	2.435	-3.593	0.606	-2.623	3.871	82.194	15.901	14.351
6804	207	33	25	C	S	25.285	-4.781	3.998	0.700	5.287	-3.943	-0.690	67.856	16.641	14.676
6805	207	33	25	C	S	25.767	0.353	4.587	-4.470	-0.351	-5.038	4.909	67.764	16.642	14.682
6806	207	33	25	C	S	34.233	0.353	-4.587	-4.470	-0.351	5.038	4.909	67.764	14.682	16.642
6807	207	33	25	C	S	34.715	-4.781	-3.998	0.700	5.287	3.943	-0.690	67.856	16.641	14.676
6808	207	33	25	C	S	51.052	-0.599	-2.435	-3.593	0.606	2.623	3.871	82.194	15.901	14.351
6809	207	33	25	C	S	53.413	-3.853	1.594	-0.320	4.175	-1.604	0.322	82.261	14.354	15.897
6810	207	33	25	C	S	66.587	-3.853	-1.594	-0.320	4.175	1.604	0.322	82.261	14.354	15.897
6811	207	33	25	C	S	68.948	-0.599	2.435	-3.593	0.606	-2.623	3.871	82.194	15.901	14.351
6812	207	33	25	C	S	85.285	-4.781	3.998	0.700	5.287	-3.943	-0.690	67.856	16.641	14.676
6813	207	33	25	C	S	85.767	0.353	4.587	-4.470	-0.351	-5.038	4.909	67.764	16.642	14.682
6814	207	33	25	C	S	94.233	0.353	-4.587	-4.470	-0.351	5.038	4.909	67.764	14.682	16.642
6815	207	33	25	C	S	94.715	-4.781	-3.998	0.700	5.287	3.943	-0.690	67.856	16.641	14.676
6816	207	33	25	C	S	111.052	-0.599	-2.435	-3.593	0.606	2.623	3.871	82.194	15.901	14.351
6817	207	33	25	C	S	113.413	-3.853	1.594	-0.320	4.175	-1.604	0.322	82.261	14.354	15.897
6818	207	33	25	C	W	6.587	-3.853	-1.594	-0.320	4.175	1.604	0.322	82.261	14.354	15.897
6819	207	33	25	C	W	8.948	-0.599	2.435	-3.593	0.606	-2.623	3.871	82.194	15.901	14.351
6820	207	33	25	C	W	51.052	-0.599	-2.435	-3.593	0.606	2.623	3.871	82.194	15.901	14.351
6821	207	33	25	C	W	53.413	-3.853	1.594	-0.320	4.175	-1.604	0.322	82.261	14.354	15.897
6822	207	33	25	C	W	66.587	-3.853	-1.594	-0.320	4.175	1.604	0.322	82.261	14.354	15.897
6823	207	33	25	C	W	68.948	-0.599	2.435	-3.593	0.606	-2.623	3.871	82.194	15.901	14.351
6824	207	33	25	C	W	111.052	-0.599	-2.435	-3.593	0.606	2.623	3.871	82.194	15.901	14.351
6825	207	33	25	C	W	113.413	-3.853	1.594	-0.320	4.175	-1.604	0.322	82.261	14.354	15.897
6826	207	33	25	P	S	6.587	-3.853	-1.594	-0.320	4.175	1.604	0.322	82.261	14.354	15.897
6827	207	33	25	P	S	8.948	-0.599	2.435	-3.593	0.606	-2.623	3.871	82.194	15.901	14.351
6828	207	33	25	P	S	25.285	-4.781	3.998	0.700	5.287	-3.943	-0.690	67.856	16.641	14.676
6829	207	33	25	P	S	25.767	0.353	4.587	-4.470	-0.351	-5.038	4.909	67.764	16.642	14.682
6830	207	33	25	P	S	34.233	0.353	-4.587	-4.470	-0.351	5.038	4.909	67.764	14.682	16.642
6831	207	33	25	P	S	34.715	-4.781	-3.998	0.700	5.287	3.943	-0.690	67.856	16.641	14.676
6832	207	33	25	P	S	51.052	-0.599	-2.435	-3.593	0.606	2.623	3.871	82.194	15.901	14.351
6833	207	33	25	P	S	53.413	-3.853	1.594	-0.320	4.175	-1.604	0.322	82.261	14.354	15.897
6834	207	33	25	P	S	66.587	-3.853	-1.594	-0.320	4.175	1.604	0.322	82.261	14.354	15.897

BL number	Atoms	γ-PC unit cells	WS_2 unit cells	γ-PC origin atom	WS_2 origin atom	Twist-angle (°)	γ-PC strain 1 (%)	γ-PC strain 2 (%)	γ-PC strain 3 (%)	WS_2 strain 1 (%)	WS_2 strain 2 (%)	WS_2 strain 3 (%)	γ (°)	$\lvert\mathbf{a}\rvert$(Å)	$\lvert\mathbf{b}\rvert$(Å)
6835	207	33	25	P	S	68.948	-0.599	2.435	-3.593	0.606	-2.623	3.871	82.194	15.901	14.351
6836	207	33	25	P	S	85.285	-4.781	3.998	0.700	5.287	-3.943	-0.690	67.856	16.641	14.676
6837	207	33	25	P	S	85.767	0.353	4.587	-4.470	-0.351	-5.038	4.909	67.764	16.642	14.682
6838	207	33	25	P	S	94.233	0.353	-4.587	-4.470	-0.351	5.038	4.909	67.764	14.682	16.642
6839	207	33	25	P	S	94.715	-4.781	-3.998	0.700	5.287	3.943	-0.690	67.856	16.641	14.676
6840	207	33	25	P	S	111.052	-0.599	-2.435	-3.593	0.606	2.623	3.871	82.194	15.901	14.351
6841	207	33	25	P	S	113.413	-3.853	1.594	-0.320	4.175	-1.604	0.322	82.261	14.354	15.897
6842	207	33	25	P	W	6.587	-3.853	-1.594	-0.320	4.175	1.604	0.322	82.261	14.354	15.897
6843	207	33	25	P	W	8.948	-0.599	2.435	-3.593	0.606	-2.623	3.871	82.194	15.901	14.351
6844	207	33	25	P	W	51.052	-0.599	-2.435	-3.593	0.606	2.623	3.871	82.194	15.901	14.351
6845	207	33	25	P	W	53.413	-3.853	1.594	-0.320	4.175	-1.604	0.322	82.261	14.354	15.897
6846	207	33	25	P	W	66.587	-3.853	-1.594	-0.320	4.175	1.604	0.322	82.261	14.354	15.897
6847	207	33	25	P	W	68.948	-0.599	2.435	-3.593	0.606	-2.623	3.871	82.194	15.901	14.351
6848	207	33	25	P	W	111.052	-0.599	-2.435	-3.593	0.606	2.623	3.871	82.194	15.901	14.351
6849	207	33	25	P	W	113.413	-3.853	1.594	-0.320	4.175	-1.604	0.322	82.261	14.354	15.897
6850	208	31	28	C	S	28.055	2.281	0.000	2.281	-2.182	0.000	-2.182	60.000	16.362	16.362
6851	208	31	28	C	S	31.945	2.281	0.000	2.281	-2.182	0.000	-2.182	60.000	16.362	16.362
6852	208	31	28	C	S	49.842	2.281	0.000	2.281	-2.182	0.000	-2.182	60.000	16.362	16.362
6853	208	31	28	C	S	70.158	2.281	0.000	2.281	-2.182	0.000	-2.182	60.000	16.362	16.362
6854	208	31	28	C	S	88.055	2.281	0.000	2.281	-2.182	0.000	-2.182	60.000	16.362	16.362
6855	208	31	28	C	S	91.945	2.281	0.000	2.281	-2.182	0.000	-2.182	60.000	16.362	16.362
6856	208	31	28	C	W	10.158	2.281	0.000	2.281	-2.182	0.000	-2.182	60.000	16.362	16.362
6857	208	31	28	C	W	28.055	2.281	0.000	2.281	-2.182	0.000	-2.182	60.000	16.362	16.362
6858	208	31	28	C	W	31.945	2.281	0.000	2.281	-2.182	0.000	-2.182	60.000	16.362	16.362
6859	208	31	28	C	W	88.055	2.281	0.000	2.281	-2.182	0.000	-2.182	60.000	16.362	16.362
6860	208	31	28	C	W	91.945	2.281	0.000	2.281	-2.182	0.000	-2.182	60.000	16.362	16.362
6861	208	31	28	C	W	109.842	2.281	0.000	2.281	-2.182	0.000	-2.182	60.000	16.362	16.362
6862	208	31	28	P	S	31.945	2.281	0.000	2.281	-2.182	0.000	-2.182	60.000	16.362	16.362
6863	208	31	28	P	S	88.055	2.281	0.000	2.281	-2.182	0.000	-2.182	60.000	16.362	16.362
6864	208	31	28	P	W	28.055	2.281	0.000	2.281	-2.182	0.000	-2.182	60.000	16.362	16.362
6865	208	31	28	P	W	91.945	2.281	0.000	2.281	-2.182	0.000	-2.182	60.000	16.362	16.362
6866	208	31	28	P	W	109.842	2.281	0.000	2.281	-2.182	0.000	-2.182	60.000	16.362	16.362
6867	208	31	28	C	S	18.613	3.582	-4.614	1.013	-3.342	4.523	-0.992	82.807	18.586	12.576
6868	208	31	28	C	S	23.413	0.481	-4.912	4.145	-0.477	4.536	-3.828	78.201	12.584	18.828
6869	208	31	28	C	S	27.796	5.011	2.934	-0.313	-4.554	-2.952	0.315	78.360	18.842	12.571
6870	208	31	28	C	S	32.204	5.011	-2.933	-0.313	-4.554	2.952	0.315	78.360	18.842	12.571
6871	208	31	28	C	S	36.587	0.481	-3.977	4.145	-0.477	3.672	-3.828	82.958	12.584	18.570
6872	208	31	28	C	S	41.387	3.582	4.614	1.013	-3.342	-4.523	-0.992	82.807	18.586	12.576
6873	208	31	28	C	S	78.613	3.582	-4.614	1.013	-3.342	4.523	-0.992	82.807	18.586	12.576
6874	208	31	28	C	S	83.413	0.481	-4.912	4.145	-0.477	4.536	-3.828	78.201	12.584	18.827
6875	208	31	28	C	S	87.796	5.011	2.934	-0.313	-4.554	-2.952	0.315	78.360	18.842	12.571
6876	208	31	28	C	S	92.204	5.011	-2.933	-0.313	-4.554	2.952	0.315	78.360	18.842	12.571
6877	208	31	28	C	S	96.587	0.481	-3.977	4.145	-0.477	3.672	-3.828	82.958	12.584	18.570
6878	208	31	28	C	S	101.387	3.582	4.614	1.013	-3.342	-4.523	-0.992	82.807	18.586	12.576
6879	208	31	28	C	W	18.613	3.582	-4.614	1.013	-3.342	4.523	-0.992	82.807	18.586	12.576
6880	208	31	28	C	W	23.413	0.481	-4.912	4.145	-0.477	4.536	-3.828	78.201	12.584	18.828
6881	208	31	28	C	W	27.796	5.011	2.934	-0.313	-4.554	-2.952	0.315	78.360	18.842	12.571
6882	208	31	28	C	W	32.204	5.011	-2.933	-0.313	-4.554	2.952	0.315	78.360	18.842	12.571
6883	208	31	28	C	W	36.587	0.481	-3.977	4.145	-0.477	3.672	-3.828	82.958	12.584	18.570
6884	208	31	28	C	W	41.387	3.582	4.614	1.013	-3.342	-4.523	-0.992	82.807	18.586	12.576
6885	208	31	28	C	W	78.613	3.582	-4.614	1.013	-3.342	4.523	-0.992	82.807	18.586	12.576

BL number	Atoms	γ-PC unit cells	WS_2 unit cells	γ-PC origin atom	WS_2 origin atom	Twist-angle (°)	γ-PC strain 1 (%)	γ-PC strain 2 (%)	γ-PC strain 3 (%)	WS_2 strain 1 (%)	WS_2 strain 2 (%)	WS_2 strain 3 (%)	γ (°)	$\vert\mathbf{a}\vert$(Å)	$\vert\mathbf{b}\vert$(Å)
6886	208	31	28	C	W	83.413	0.481	-4.912	4.145	-0.477	4.536	-3.828	78.201	12.584	18.827
6887	208	31	28	C	W	87.796	5.011	2.934	-0.313	-4.554	-2.952	0.315	78.360	18.842	12.571
6888	208	31	28	C	W	92.204	5.011	-2.933	-0.313	-4.554	2.952	0.315	78.360	18.842	12.571
6889	208	31	28	C	W	96.587	0.481	-3.977	4.145	-0.477	3.672	-3.828	82.958	12.584	18.570
6890	208	31	28	C	W	101.387	3.582	4.614	1.013	-3.342	-4.523	-0.992	82.807	18.586	12.576
6891	208	31	28	P	S	18.613	3.582	-4.614	1.013	-3.342	4.523	-0.992	82.807	18.586	12.576
6892	208	31	28	P	S	23.413	0.481	-4.912	4.145	-0.477	4.536	-3.828	78.201	12.584	18.828
6893	208	31	28	P	S	27.796	5.011	2.934	-0.313	-4.554	-2.952	0.315	78.360	18.842	12.571
6894	208	31	28	P	S	36.587	0.481	-3.977	4.145	-0.477	3.672	-3.828	82.958	12.584	18.570
6895	208	31	28	P	S	41.387	3.582	4.614	1.013	-3.342	-4.523	-0.992	82.807	18.586	12.576
6896	208	31	28	P	S	78.613	3.582	-4.614	1.013	-3.342	4.523	-0.992	82.807	18.586	12.576
6897	208	31	28	P	S	83.413	0.481	3.977	4.145	-0.477	-3.672	-3.828	82.958	12.584	18.570
6898	208	31	28	P	S	92.204	5.011	-2.933	-0.313	-4.554	2.952	0.315	78.360	18.842	12.571
6899	208	31	28	P	S	96.587	0.481	-3.977	4.145	-0.477	3.672	-3.828	82.958	12.584	18.570
6900	208	31	28	P	S	101.387	3.582	4.614	1.013	-3.342	-4.523	-0.992	82.807	18.586	12.576
6901	208	31	28	P	W	18.613	3.582	-4.614	1.013	-3.342	4.523	-0.992	82.807	18.586	12.576
6902	208	31	28	P	W	23.413	0.481	3.977	4.145	-0.477	-3.672	-3.828	82.958	12.584	18.570
6903	208	31	28	P	W	27.796	5.011	2.934	-0.313	-4.554	-2.952	0.315	78.360	18.842	12.571
6904	208	31	28	P	W	32.204	5.011	-2.933	-0.313	-4.554	2.952	0.315	78.360	18.842	12.571
6905	208	31	28	P	W	36.587	0.481	-3.977	4.145	-0.477	3.672	-3.828	82.958	12.584	18.570
6906	208	31	28	P	W	41.387	3.582	4.614	1.013	-3.342	-4.523	-0.992	82.807	18.586	12.576
6907	208	31	28	P	W	78.613	3.582	-4.614	1.013	-3.342	4.523	-0.992	82.807	18.586	12.576
6908	208	31	28	P	W	83.413	0.481	-4.912	4.145	-0.477	4.536	-3.828	78.201	12.584	18.827
6909	208	31	28	P	W	87.796	5.011	2.934	-0.313	-4.554	-2.952	0.315	78.360	18.842	12.571
6910	208	31	28	P	W	96.587	0.481	-3.977	4.145	-0.477	3.672	-3.828	82.958	12.584	18.570
6911	208	31	28	P	W	101.387	3.582	4.614	1.013	-3.342	-4.523	-0.992	82.807	18.586	12.576
6912	208	34	24	C	S	3.004	-3.388	-1.758	-4.172	3.635	1.918	4.551	66.380	16.955	14.487
6913	208	34	24	C	S	4.307	-4.685	0.426	-2.861	5.169	-0.452	3.034	66.366	14.491	16.952
6914	208	34	24	C	S	13.898	-4.228	-3.253	-3.331	4.619	3.485	3.568	65.292	17.113	14.476
6915	208	34	24	C	S	17.480	-4.685	2.811	-2.861	5.169	-2.982	3.034	65.196	14.491	17.109
6916	208	34	24	C	S	42.520	-4.685	-2.811	-2.861	5.169	2.982	3.034	65.196	14.491	17.109
6917	208	34	24	C	S	46.102	-4.228	3.253	-3.331	4.619	-3.485	3.568	65.292	14.476	17.113
6918	208	34	24	C	S	55.285	-3.007	-1.098	-4.543	3.200	1.208	4.997	66.390	16.951	14.489
6919	208	34	24	C	S	55.693	-4.685	-0.426	-2.861	5.169	0.452	3.034	66.366	14.491	16.952
6920	208	34	24	C	S	63.005	-3.388	-1.758	-4.172	3.635	1.918	4.551	66.380	16.955	14.487
6921	208	34	24	C	S	64.715	-3.007	1.098	-4.543	3.200	-1.208	4.997	66.390	16.951	14.489
6922	208	34	24	C	S	73.898	-4.228	-3.253	-3.331	4.619	3.485	3.568	65.292	17.113	14.476
6923	208	34	24	C	S	77.480	-4.685	2.811	-2.861	5.169	-2.982	3.034	65.196	14.491	17.109
6924	208	34	24	C	S	102.520	-4.685	-2.811	-2.861	5.169	2.982	3.034	65.196	14.491	17.109
6925	208	34	24	C	S	106.102	-4.228	3.253	-3.331	4.619	-3.485	3.568	65.292	14.476	17.113
6926	208	34	24	C	S	115.285	-3.007	-1.098	-4.543	3.200	1.208	4.997	66.390	16.951	14.489
6927	208	34	24	C	S	116.996	-3.388	1.758	-4.172	3.634	-1.918	4.551	66.380	14.487	16.955
6928	208	34	24	C	W	3.004	-3.388	-1.758	-4.172	3.635	1.918	4.551	66.380	16.955	14.487
6929	208	34	24	C	W	4.715	-3.007	1.098	-4.543	3.200	-1.208	4.997	66.390	16.951	14.489
6930	208	34	24	C	W	13.898	-4.228	-3.253	-3.331	4.619	3.485	3.568	65.292	17.113	14.476
6931	208	34	24	C	W	17.480	-4.685	2.811	-2.861	5.169	-2.982	3.034	65.196	14.491	17.109
6932	208	34	24	C	W	42.520	-4.685	-2.811	-2.861	5.169	2.982	3.034	65.196	14.491	17.109
6933	208	34	24	C	W	46.102	-4.228	3.253	-3.331	4.619	-3.485	3.568	65.292	14.476	17.113
6934	208	34	24	C	W	55.285	-3.007	-1.098	-4.543	3.200	1.208	4.997	66.390	16.951	14.489
6935	208	34	24	C	W	56.996	-3.388	1.758	-4.172	3.634	-1.918	4.551	66.380	14.487	16.955
6936	208	34	24	C	W	63.005	-3.388	-1.758	-4.172	3.635	1.918	4.551	66.380	16.955	14.487

BL number	Atoms	γ-PC unit cells	WS_2 unit cells	γ-PC origin atom	WS_2 origin atom	Twist-angle (°)	γ-PC strain 1 (%)	γ-PC strain 2 (%)	γ-PC strain 3 (%)	WS_2 strain 1 (%)	WS_2 strain 2 (%)	WS_2 strain 3 (%)	γ (°)	$\lvert\mathbf{a}\rvert$(Å)	$\lvert\mathbf{b}\rvert$(Å)
6937	208	34	24	C	W	64.307	-4.685	0.426	-2.861	5.169	-0.452	3.034	66.366	14.491	16.953
6938	208	34	24	C	W	73.898	-4.228	-3.253	-3.331	4.619	3.485	3.568	65.292	17.113	14.476
6939	208	34	24	C	W	77.480	-4.685	2.811	-2.861	5.169	-2.982	3.034	65.196	14.491	17.109
6940	208	34	24	C	W	102.520	-4.685	-2.811	-2.861	5.169	2.982	3.034	65.196	14.491	17.109
6941	208	34	24	C	W	106.102	-4.228	3.253	-3.331	4.619	-3.485	3.568	65.292	14.476	17.113
6942	208	34	24	C	W	115.285	-3.007	-1.098	-4.543	3.200	1.208	4.997	66.390	16.951	14.489
6943	208	34	24	C	W	115.693	-4.685	-0.426	-2.861	5.169	0.452	3.034	66.366	14.491	16.952
6944	208	34	24	P	S	3.004	-3.388	-1.758	-4.172	3.635	1.918	4.551	66.380	16.955	14.487
6945	208	34	24	P	S	4.715	-3.007	1.098	-4.543	3.200	-1.208	4.997	66.390	16.951	14.489
6946	208	34	24	P	S	55.285	-3.007	-1.098	-4.543	3.200	1.208	4.997	66.390	16.951	14.489
6947	208	34	24	P	S	56.996	-3.388	1.758	-4.172	3.634	-1.918	4.551	66.380	14.487	16.955
6948	208	34	24	P	S	63.005	-3.388	-1.758	-4.172	3.635	1.918	4.551	66.380	16.955	14.487
6949	208	34	24	P	S	64.307	-4.685	0.426	-2.861	5.169	-0.452	3.034	66.366	14.491	16.953
6950	208	34	24	P	S	115.285	-3.007	-1.098	-4.543	3.200	1.208	4.997	66.390	16.951	14.489
6951	208	34	24	P	S	115.693	-4.685	-0.426	-2.861	5.169	0.452	3.034	66.366	14.491	16.952
6952	208	34	24	P	W	3.004	-3.388	-1.758	-4.172	3.635	1.918	4.551	66.380	16.955	14.487
6953	208	34	24	P	W	4.307	-4.685	0.426	-2.861	5.169	-0.452	3.034	66.366	14.491	16.952
6954	208	34	24	P	W	55.285	-3.007	-1.098	-4.543	3.200	1.208	4.997	66.390	16.951	14.489
6955	208	34	24	P	W	55.693	-4.685	-0.426	-2.861	5.169	0.452	3.034	66.366	14.491	16.952
6956	208	34	24	P	W	63.005	-3.388	-1.758	-4.172	3.635	1.918	4.551	66.380	16.955	14.487
6957	208	34	24	P	W	64.715	-3.007	1.098	-4.543	3.200	-1.208	4.997	66.390	16.951	14.489
6958	208	34	24	P	W	115.285	-3.007	-1.098	-4.543	3.200	1.208	4.997	66.390	16.951	14.489
6959	208	34	24	P	W	116.996	-3.388	1.758	-4.172	3.634	-1.918	4.551	66.380	14.487	16.955
6960	209	32	27	C	S	0.000	5.011	0.947	-3.585	-4.554	-1.020	3.861	75.143	13.151	18.221
6961	209	32	27	C	S	1.359	-3.292	3.518	4.666	3.523	-3.218	-4.268	75.051	18.221	13.154
6962	209	32	27	C	S	8.213	-2.359	-3.523	3.596	2.476	3.287	-3.355	80.349	14.845	15.812
6963	209	32	27	C	S	10.893	4.020	1.251	-2.733	-3.721	-1.323	2.891	80.507	15.815	14.839
6964	209	32	27	C	S	27.796	5.011	2.842	-3.585	-4.554	-3.061	3.861	74.659	14.852	16.172
6965	209	32	27	C	S	32.204	5.011	-2.842	-3.585	-4.554	3.061	3.861	74.659	16.172	14.852
6966	209	32	27	C	S	49.107	4.020	-1.251	-2.733	-3.721	1.323	2.891	80.507	15.815	14.839
6967	209	32	27	C	S	51.787	-2.359	3.523	3.596	2.476	-3.287	-3.355	80.349	14.845	15.812
6968	209	32	27	C	S	58.641	-3.292	-3.518	4.666	3.523	3.217	-4.268	75.051	18.221	13.154
6969	209	32	27	C	S	60.000	5.011	2.842	-3.585	-4.554	-3.061	3.861	81.616	13.826	16.933
6970	209	32	27	C	S	61.359	-3.292	3.518	4.666	3.523	-3.218	-4.268	75.051	18.221	13.154
6971	209	32	27	C	S	68.213	-2.359	-3.523	3.596	2.476	3.287	-3.355	80.349	14.845	15.812
6972	209	32	27	C	S	70.893	4.020	1.251	-2.733	-3.721	-1.323	2.891	80.507	15.815	14.840
6973	209	32	27	C	S	87.796	5.011	2.842	-3.585	-4.554	-3.061	3.861	74.659	14.852	16.172
6974	209	32	27	C	S	92.204	5.011	-2.842	-3.585	-4.554	3.061	3.861	74.659	16.172	14.852
6975	209	32	27	C	S	109.107	4.020	-1.251	-2.733	-3.721	1.323	2.891	80.507	15.815	14.839
6976	209	32	27	C	S	111.787	-2.359	3.523	3.596	2.476	-3.287	-3.355	80.349	14.845	15.812
6977	209	32	27	C	S	118.641	-3.292	-3.518	4.666	3.523	3.217	-4.268	75.051	18.221	13.154
6978	209	32	27	C	W	0.000	5.011	0.947	-3.585	-4.554	-1.020	3.861	75.143	13.151	18.221
6979	209	32	27	C	W	1.359	-3.292	3.518	4.666	3.523	-3.218	-4.268	75.051	18.221	13.154
6980	209	32	27	C	W	8.213	-2.359	-3.523	3.596	2.476	3.287	-3.355	80.349	14.845	15.812
6981	209	32	27	C	W	10.893	4.020	1.251	-2.733	-3.721	-1.323	2.891	80.507	15.815	14.839
6982	209	32	27	C	W	27.796	5.011	2.842	-3.585	-4.554	-3.061	3.861	74.659	14.852	16.172
6983	209	32	27	C	W	32.204	5.011	-2.842	-3.585	-4.554	3.061	3.861	74.659	16.172	14.852
6984	209	32	27	C	W	49.107	4.020	-1.251	-2.733	-3.721	1.323	2.891	80.507	15.815	14.839
6985	209	32	27	C	W	51.787	-2.359	3.523	3.596	2.476	-3.287	-3.355	80.349	14.845	15.812
6986	209	32	27	C	W	58.641	-3.292	-3.518	4.666	3.523	3.217	-4.268	75.051	18.221	13.154
6987	209	32	27	C	W	60.000	5.011	2.842	-3.585	-4.554	-3.061	3.861	81.616	13.826	16.933

BL number	Atoms	γ-PC unit cells	WS_2 unit cells	γ-PC origin atom	WS_2 origin atom	Twist-angle (°)	γ-PC strain 1 (%)	γ-PC strain 2 (%)	γ-PC strain 3 (%)	WS_2 strain 1 (%)	WS_2 strain 2 (%)	WS_2 strain 3 (%)	γ (°)	$\lvert\mathbf{a}\rvert$(Å)	$\lvert\mathbf{b}\rvert$(Å)
6988	209	32	27	C	W	61.359	-3.292	3.518	4.666	3.523	-3.218	-4.268	75.051	18.221	13.154
6989	209	32	27	C	W	68.213	-2.359	-3.523	3.596	2.476	3.287	-3.355	80.349	14.845	15.812
6990	209	32	27	C	W	70.893	4.020	1.251	-2.733	-3.721	-1.323	2.891	80.507	15.815	14.840
6991	209	32	27	C	W	87.796	5.011	2.842	-3.585	-4.554	-3.061	3.861	74.659	14.852	16.172
6992	209	32	27	C	W	92.204	5.011	-2.842	-3.585	-4.554	3.061	3.861	74.659	16.172	14.852
6993	209	32	27	C	W	109.107	4.020	-1.251	-2.733	-3.721	1.323	2.891	80.507	15.815	14.839
6994	209	32	27	C	W	111.787	-2.359	3.523	3.596	2.476	-3.287	-3.355	80.349	14.845	15.812
6995	209	32	27	C	W	118.641	-3.292	-3.518	4.666	3.523	3.217	-4.268	75.051	18.221	13.154
6996	209	32	27	P	S	0.000	5.011	0.947	-3.585	-4.554	-1.020	3.861	75.143	13.151	18.221
6997	209	32	27	P	S	1.359	-3.292	3.518	4.666	3.523	-3.218	-4.268	75.051	18.221	13.154
6998	209	32	27	P	S	27.796	5.011	2.842	-3.585	-4.554	-3.061	3.861	74.659	14.852	16.172
6999	209	32	27	P	S	49.107	4.020	-1.251	-2.733	-3.721	1.323	2.891	80.507	15.815	14.839
7000	209	32	27	P	S	51.787	-2.359	3.523	3.596	2.476	-3.287	-3.355	80.349	14.845	15.812
7001	209	32	27	P	S	58.641	-3.292	-3.518	4.666	3.523	3.217	-4.268	75.051	18.221	13.154
7002	209	32	27	P	S	60.000	5.011	0.947	-3.585	-4.554	-1.020	3.861	75.143	13.151	18.221
7003	209	32	27	P	S	61.359	-3.292	3.518	4.666	3.523	-3.218	-4.268	75.051	18.221	13.154
7004	209	32	27	P	S	68.213	-2.359	-3.523	3.596	2.476	3.287	-3.355	80.349	14.845	15.812
7005	209	32	27	P	S	70.893	4.020	1.251	-2.733	-3.721	-1.323	2.891	80.507	15.815	14.840
7006	209	32	27	P	S	92.204	5.011	-2.842	-3.585	-4.554	3.061	3.861	74.659	16.172	14.852
7007	209	32	27	P	S	118.641	-3.292	-3.518	4.666	3.523	3.217	-4.268	75.051	18.221	13.154
7008	209	32	27	P	W	0.000	5.011	0.947	-3.585	-4.554	-1.020	3.861	75.143	13.151	18.221
7009	209	32	27	P	W	1.359	-3.292	3.518	4.666	3.523	-3.218	-4.268	75.051	18.221	13.154
7010	209	32	27	P	W	8.213	-2.359	-3.523	3.596	2.476	3.287	-3.355	80.349	14.845	15.812
7011	209	32	27	P	W	10.893	4.020	1.251	-2.733	-3.721	-1.323	2.891	80.507	15.815	14.839
7012	209	32	27	P	W	32.204	5.011	-2.842	-3.585	-4.554	3.061	3.861	74.659	16.172	14.852
7013	209	32	27	P	W	58.641	-3.292	-3.518	4.666	3.523	3.217	-4.268	75.051	18.221	13.154
7014	209	32	27	P	W	60.000	5.011	2.842	-3.585	-4.554	-3.061	3.861	81.616	13.826	16.933
7015	209	32	27	P	W	61.359	-3.292	3.518	4.666	3.523	-3.218	-4.268	75.051	18.221	13.154
7016	209	32	27	P	W	87.796	5.011	2.842	-3.585	-4.554	-3.061	3.861	74.659	14.852	16.172
7017	209	32	27	P	W	109.107	4.020	-1.251	-2.733	-3.721	1.323	2.891	80.507	15.815	14.839
7018	209	32	27	P	W	111.787	-2.359	3.523	3.596	2.476	-3.287	-3.355	80.349	14.845	15.812
7019	209	32	27	P	W	118.641	-3.292	-3.518	4.666	3.523	3.217	-4.268	75.051	18.221	13.154
7020	210	30	30	C	S	9.430	5.011	-2.021	5.011	-4.554	1.837	-4.555	70.907	15.820	15.820
7021	210	30	30	C	S	50.570	5.011	2.021	5.011	-4.554	-1.837	-4.555	70.907	15.820	15.820
7022	210	30	30	C	S	69.430	5.011	-2.021	5.011	-4.554	1.837	-4.555	70.907	15.820	15.820
7023	210	30	30	C	S	110.570	5.011	2.021	5.011	-4.554	-1.837	-4.555	70.907	15.820	15.820
7024	210	30	30	C	W	9.430	5.011	-2.021	5.011	-4.554	1.837	-4.555	70.907	15.820	15.820
7025	210	30	30	C	W	50.570	5.011	2.021	5.011	-4.554	-1.837	-4.555	70.907	15.820	15.820
7026	210	30	30	C	W	69.430	5.011	-2.021	5.011	-4.554	1.837	-4.555	70.907	15.820	15.820
7027	210	30	30	C	W	110.570	5.011	2.021	5.011	-4.554	-1.837	-4.555	70.907	15.820	15.820
7028	210	30	30	P	S	9.430	5.011	-2.021	5.011	-4.554	1.837	-4.555	70.907	15.820	15.820
7029	210	30	30	P	S	50.570	5.011	2.021	5.011	-4.554	-1.837	-4.555	70.907	15.820	15.820
7030	210	30	30	P	S	69.430	5.011	-2.021	5.011	-4.554	1.837	-4.555	70.907	15.820	15.820
7031	210	30	30	P	S	110.570	5.011	2.021	5.011	-4.554	-1.837	-4.555	70.907	15.820	15.820
7032	210	30	30	P	W	9.430	5.011	-2.021	5.011	-4.554	1.837	-4.555	70.907	15.820	15.820
7033	210	30	30	P	W	50.570	5.011	2.021	5.011	-4.554	-1.837	-4.555	70.907	15.820	15.820
7034	210	30	30	P	W	69.430	5.011	-2.021	5.011	-4.554	1.837	-4.555	70.907	15.820	15.820
7035	210	30	30	P	W	110.570	5.011	2.021	5.011	-4.554	-1.837	-4.555	70.907	15.820	15.820
7036	210	33	26	C	S	6.587	-3.853	-4.383	1.667	4.175	4.241	-1.613	79.131	14.354	16.359
7037	210	33	26	C	S	8.513	2.390	-0.896	-4.490	-2.281	0.984	4.933	89.463	19.291	11.960
7038	210	33	26	C	S	9.515	-4.497	0.835	2.398	4.941	-0.797	-2.288	89.533	11.961	19.290

BL number	Atoms	γ-PC unit cells	WS_2 unit cells	γ-PC origin atom	WS_2 origin atom	Twist-angle (°)	γ-PC strain 1 (%)	γ-PC strain 2 (%)	γ-PC strain 3 (%)	WS_2 strain 1 (%)	WS_2 strain 2 (%)	WS_2 strain 3 (%)	γ (°)	$\lvert\mathbf{a}\rvert$(Å)	$\lvert\mathbf{b}\rvert$(Å)
7039	210	33	26	C	S	10.158	2.281	1.853	-4.395	-2.182	-2.032	4.819	79.362	16.362	14.346
7040	210	33	26	C	S	16.337	0.353	-1.444	-2.649	-0.351	1.525	2.797	66.435	14.703	17.102
7041	210	33	26	C	S	19.107	-1.485	3.324	-0.855	1.530	-3.382	0.870	66.450	14.708	17.091
7042	210	33	26	C	S	22.689	-4.497	-0.835	2.398	4.941	0.797	-2.288	89.533	11.961	19.290
7043	210	33	26	C	S	23.691	2.390	0.896	-4.490	-2.281	-0.984	4.933	89.463	19.291	11.960
7044	210	33	26	C	S	36.309	2.390	-0.896	-4.490	-2.281	0.984	4.933	89.463	19.291	11.960
7045	210	33	26	C	S	37.311	-4.497	0.835	2.398	4.941	-0.797	-2.288	89.533	11.961	19.290
7046	210	33	26	C	S	40.893	-1.485	-3.324	-0.855	1.530	3.382	0.870	66.450	14.708	17.091
7047	210	33	26	C	S	43.663	0.353	1.444	-2.649	-0.351	-1.525	2.797	66.435	17.102	14.703
7048	210	33	26	C	S	44.392	-2.145	2.752	-0.177	2.241	-2.761	0.178	66.467	17.102	14.698
7049	210	33	26	C	S	49.842	2.281	-1.853	-4.395	-2.182	2.032	4.819	79.362	16.362	14.346
7050	210	33	26	C	S	50.485	-4.497	-0.835	2.398	4.941	0.797	-2.288	89.533	11.961	19.290
7051	210	33	26	C	S	51.487	2.390	0.896	-4.490	-2.281	-0.984	4.933	89.463	19.291	11.960
7052	210	33	26	C	S	53.413	-3.853	4.383	1.667	4.175	-4.241	-1.613	79.131	14.354	16.359
7053	210	33	26	C	S	66.587	-3.853	-4.383	1.667	4.175	4.241	-1.613	79.131	14.354	16.359
7054	210	33	26	C	S	68.513	2.390	-0.896	-4.490	-2.281	0.984	4.933	89.463	19.291	11.960
7055	210	33	26	C	S	69.515	-4.497	0.835	2.398	4.941	-0.797	-2.288	89.533	11.961	19.290
7056	210	33	26	C	S	70.158	2.281	1.853	-4.395	-2.182	-2.032	4.819	79.361	16.362	14.346
7057	210	33	26	C	S	75.609	-2.145	-2.751	-0.177	2.241	2.761	0.178	66.467	17.102	14.698
7058	210	33	26	C	S	76.337	0.353	-1.444	-2.649	-0.351	1.525	2.797	66.435	14.703	17.102
7059	210	33	26	C	S	79.107	-1.485	3.324	-0.855	1.530	-3.382	0.870	66.450	14.708	17.091
7060	210	33	26	C	S	82.689	-4.497	-0.835	2.398	4.941	0.797	-2.288	89.533	11.961	19.290
7061	210	33	26	C	S	83.691	2.390	0.896	-4.490	-2.281	-0.984	4.933	89.463	19.291	11.960
7062	210	33	26	C	S	96.309	2.390	-0.896	-4.490	-2.281	0.984	4.933	89.463	19.291	11.960
7063	210	33	26	C	S	97.311	-4.497	0.835	2.398	4.941	-0.797	-2.288	89.533	11.961	19.290
7064	210	33	26	C	S	100.893	-1.485	-3.324	-0.855	1.530	3.382	0.870	66.450	14.708	17.091
7065	210	33	26	C	S	103.663	0.353	1.444	-2.649	-0.351	-1.525	2.797	66.435	17.102	14.703
7066	210	33	26	C	S	104.392	-2.145	2.752	-0.177	2.241	-2.761	0.178	66.467	17.102	14.698
7067	210	33	26	C	S	109.842	2.281	-1.853	-4.395	-2.182	2.032	4.819	79.361	16.362	14.346
7068	210	33	26	C	S	110.485	-4.497	-0.835	2.398	4.941	0.797	-2.288	89.533	11.961	19.290
7069	210	33	26	C	S	111.487	2.390	0.896	-4.490	-2.281	-0.984	4.933	89.463	19.291	11.960
7070	210	33	26	C	S	113.413	-3.853	4.383	1.667	4.175	-4.241	-1.613	79.131	14.354	16.359
7071	210	33	26	C	W	6.587	-3.853	-4.383	1.667	4.175	4.241	-1.613	79.131	14.354	16.359
7072	210	33	26	C	W	8.513	2.390	-0.896	-4.490	-2.281	0.984	4.933	89.463	19.291	11.960
7073	210	33	26	C	W	9.515	-4.497	0.835	2.398	4.941	-0.797	-2.288	89.533	11.961	19.290
7074	210	33	26	C	W	10.158	2.281	1.853	-4.395	-2.182	-2.032	4.819	79.362	16.362	14.346
7075	210	33	26	C	W	15.608	-2.145	-2.751	-0.177	2.241	2.761	0.178	66.467	17.102	14.698
7076	210	33	26	C	W	22.689	-4.497	-0.835	2.398	4.941	0.797	-2.288	89.533	11.961	19.290
7077	210	33	26	C	W	23.691	2.390	0.896	-4.490	-2.281	-0.984	4.933	89.463	19.291	11.960
7078	210	33	26	C	W	36.309	2.390	-0.896	-4.490	-2.281	0.984	4.933	89.463	19.291	11.960
7079	210	33	26	C	W	37.311	-4.497	0.835	2.398	4.941	-0.797	-2.288	89.533	11.961	19.290
7080	210	33	26	C	W	49.842	2.281	-1.853	-4.395	-2.182	2.032	4.819	79.362	16.362	14.346
7081	210	33	26	C	W	50.485	-4.497	-0.835	2.398	4.941	0.797	-2.288	89.533	11.961	19.290
7082	210	33	26	C	W	51.487	2.390	0.896	-4.490	-2.281	-0.984	4.933	89.463	19.291	11.960
7083	210	33	26	C	W	53.413	-3.853	4.383	1.667	4.175	-4.241	-1.613	79.131	14.354	16.359
7084	210	33	26	C	W	66.587	-3.853	-4.383	1.667	4.175	4.241	-1.613	79.131	14.354	16.359
7085	210	33	26	C	W	68.513	2.390	-0.896	-4.490	-2.281	0.984	4.933	89.463	19.291	11.960
7086	210	33	26	C	W	69.515	-4.497	0.835	2.398	4.941	-0.797	-2.288	89.533	11.961	19.290
7087	210	33	26	C	W	70.158	2.281	1.853	-4.395	-2.182	-2.032	4.819	79.361	16.362	14.346
7088	210	33	26	C	W	82.689	-4.497	-0.835	2.398	4.941	0.797	-2.288	89.533	11.961	19.290
7089	210	33	26	C	W	83.691	2.390	0.896	-4.490	-2.281	-0.984	4.933	89.463	19.291	11.960

BL number	Atoms	γ-PC unit cells	WS_2 unit cells	γ-PC origin atom	WS_2 origin atom	Twist-angle (°)	γ-PC strain 1 (%)	γ-PC strain 2 (%)	γ-PC strain 3 (%)	WS_2 strain 1 (%)	WS_2 strain 2 (%)	WS_2 strain 3 (%)	γ (°)	|**a**|(Å)	|**b**|(Å)
7090	210	33	26	C	W	96.309	2.390	-0.896	-4.490	-2.281	0.984	4.933	89.463	19.291	11.960
7091	210	33	26	C	W	97.311	-4.497	0.835	2.398	4.941	-0.797	-2.288	89.533	11.961	19.290
7092	210	33	26	C	W	100.893	-1.485	-3.324	-0.855	1.530	3.382	0.870	66.450	14.708	17.091
7093	210	33	26	C	W	109.842	2.281	-1.853	-4.395	-2.182	2.032	4.819	79.361	16.362	14.346
7094	210	33	26	C	W	110.485	-4.497	-0.835	2.398	4.941	0.797	-2.288	89.533	11.961	19.290
7095	210	33	26	C	W	111.487	2.390	0.896	-4.490	-2.281	-0.984	4.933	89.463	19.291	11.960
7096	210	33	26	C	W	113.413	-3.853	4.383	1.667	4.175	-4.241	-1.613	79.131	14.354	16.359
7097	210	33	26	P	S	6.587	-3.853	-4.383	1.667	4.175	4.241	-1.613	79.131	14.354	16.359
7098	210	33	26	P	S	8.513	2.390	-0.896	-4.490	-2.281	0.984	4.933	89.463	19.291	11.960
7099	210	33	26	P	S	9.515	-4.497	0.835	2.398	4.941	-0.797	-2.288	89.533	11.961	19.290
7100	210	33	26	P	S	10.158	2.281	1.853	-4.395	-2.182	-2.032	4.819	79.362	16.362	14.346
7101	210	33	26	P	S	16.337	0.353	-1.444	-2.649	-0.351	1.525	2.797	66.435	14.703	17.102
7102	210	33	26	P	S	19.107	-1.485	3.324	-0.855	1.530	-3.382	0.870	66.450	14.708	17.091
7103	210	33	26	P	S	22.689	-4.497	-0.835	2.398	4.941	0.797	-2.288	89.533	11.961	19.290
7104	210	33	26	P	S	23.691	2.390	0.896	-4.490	-2.281	-0.984	4.933	89.463	19.291	11.960
7105	210	33	26	P	S	36.309	2.390	-0.896	-4.490	-2.281	0.984	4.933	89.463	19.291	11.960
7106	210	33	26	P	S	37.311	-4.497	0.835	2.398	4.941	-0.797	-2.288	89.533	11.961	19.290
7107	210	33	26	P	S	40.893	-1.485	-3.324	-0.855	1.530	3.382	0.870	66.450	14.708	17.091
7108	210	33	26	P	S	43.663	0.353	1.444	-2.649	-0.351	-1.525	2.797	66.435	17.102	14.703
7109	210	33	26	P	S	44.392	-2.145	2.752	-0.177	2.241	-2.761	0.178	66.467	17.102	14.698
7110	210	33	26	P	S	49.842	2.281	-1.853	-4.395	-2.182	2.032	4.819	79.362	16.362	14.346
7111	210	33	26	P	S	50.485	-4.497	-0.835	2.398	4.941	0.797	-2.288	89.533	11.961	19.290
7112	210	33	26	P	S	51.487	2.390	0.896	-4.490	-2.281	-0.984	4.933	89.463	19.291	11.960
7113	210	33	26	P	S	53.413	-3.853	4.383	1.667	4.175	-4.241	-1.613	79.131	14.354	16.359
7114	210	33	26	P	S	66.587	-3.853	-4.383	1.667	4.175	4.241	-1.613	79.131	14.354	16.359
7115	210	33	26	P	S	68.513	2.390	-0.896	-4.490	-2.281	0.984	4.933	89.463	19.291	11.960
7116	210	33	26	P	S	69.515	-4.497	0.835	2.398	4.941	-0.797	-2.288	89.533	11.961	19.290
7117	210	33	26	P	S	70.158	2.281	1.853	-4.395	-2.182	-2.032	4.819	79.361	16.362	14.346
7118	210	33	26	P	S	75.609	-2.145	-2.751	-0.177	2.241	2.761	0.178	66.467	17.102	14.698
7119	210	33	26	P	S	76.337	0.353	-1.444	-2.649	-0.351	1.525	2.797	66.435	14.703	17.102
7120	210	33	26	P	S	79.107	-1.485	3.324	-0.855	1.530	-3.382	0.870	66.450	14.708	17.091
7121	210	33	26	P	S	82.689	-4.497	-0.835	2.398	4.941	0.797	-2.288	89.533	11.961	19.290
7122	210	33	26	P	S	83.691	2.390	0.896	-4.490	-2.281	-0.984	4.933	89.463	19.291	11.960
7123	210	33	26	P	S	96.309	2.390	-0.896	-4.490	-2.281	0.984	4.933	89.463	19.291	11.960
7124	210	33	26	P	S	97.311	-4.497	0.835	2.398	4.941	-0.797	-2.288	89.533	11.961	19.290
7125	210	33	26	P	S	100.893	-1.485	-3.324	-0.855	1.530	3.382	0.870	66.450	14.708	17.091
7126	210	33	26	P	S	103.663	0.353	1.444	-2.649	-0.351	-1.525	2.797	66.435	17.102	14.703
7127	210	33	26	P	S	104.392	-2.145	2.752	-0.177	2.241	-2.761	0.178	66.467	17.102	14.698
7128	210	33	26	P	S	109.842	2.281	-1.853	-4.395	-2.182	2.032	4.819	79.361	16.362	14.346
7129	210	33	26	P	S	110.485	-4.497	-0.835	2.398	4.941	0.797	-2.288	89.533	11.961	19.290
7130	210	33	26	P	S	111.487	2.390	0.896	-4.490	-2.281	-0.984	4.933	89.463	19.291	11.960
7131	210	33	26	P	S	113.413	-3.853	4.383	1.667	4.175	-4.241	-1.613	79.131	14.354	16.359
7132	210	33	26	P	W	6.587	-3.853	-4.383	1.667	4.175	4.241	-1.613	79.131	14.354	16.359
7133	210	33	26	P	W	8.513	2.390	-0.896	-4.490	-2.281	0.984	4.933	89.463	19.291	11.960
7134	210	33	26	P	W	9.515	-4.497	0.835	2.398	4.941	-0.797	-2.288	89.533	11.961	19.290
7135	210	33	26	P	W	10.158	2.281	1.853	-4.395	-2.182	-2.032	4.819	79.362	16.362	14.346
7136	210	33	26	P	W	15.608	-2.145	-2.751	-0.177	2.241	2.761	0.178	66.467	17.102	14.698
7137	210	33	26	P	W	22.689	-4.497	-0.835	2.398	4.941	0.797	-2.288	89.533	11.961	19.290
7138	210	33	26	P	W	23.691	2.390	0.896	-4.490	-2.281	-0.984	4.933	89.463	19.291	11.960
7139	210	33	26	P	W	36.309	2.390	-0.896	-4.490	-2.281	0.984	4.933	89.463	19.291	11.960
7140	210	33	26	P	W	37.311	-4.497	0.835	2.398	4.941	-0.797	-2.288	89.533	11.961	19.290

BL number	Atoms	γ-PC unit cells	WS_2 unit cells	γ-PC origin atom	WS_2 origin atom	Twist-angle (°)	γ-PC strain 1 (%)	γ-PC strain 2 (%)	γ-PC strain 3 (%)	WS_2 strain 1 (%)	WS_2 strain 2 (%)	WS_2 strain 3 (%)	γ (°)	**|a|**(Å)	**|b|**(Å)
7141	210	33	26	P	W	49.842	2.281	-1.853	-4.395	-2.182	2.032	4.819	79.362	16.362	14.346
7142	210	33	26	P	W	50.485	-4.497	-0.835	2.398	4.941	0.797	-2.288	89.533	11.961	19.290
7143	210	33	26	P	W	51.487	2.390	0.896	-4.490	-2.281	-0.984	4.933	89.463	19.291	11.960
7144	210	33	26	P	W	53.413	-3.853	4.383	1.667	4.175	-4.241	-1.613	79.131	14.354	16.359
7145	210	33	26	P	W	66.587	-3.853	-4.383	1.667	4.175	4.241	-1.613	79.131	14.354	16.359
7146	210	33	26	P	W	68.513	2.390	-0.896	-4.490	-2.281	0.984	4.933	89.463	19.291	11.960
7147	210	33	26	P	W	69.515	-4.497	0.835	2.398	4.941	-0.797	-2.288	89.533	11.961	19.290
7148	210	33	26	P	W	70.158	2.281	1.853	-4.395	-2.182	-2.032	4.819	79.361	16.362	14.346
7149	210	33	26	P	W	82.689	-4.497	-0.835	2.398	4.941	0.797	-2.288	89.533	11.961	19.290
7150	210	33	26	P	W	83.691	2.390	0.896	-4.490	-2.281	-0.984	4.933	89.463	19.291	11.960
7151	210	33	26	P	W	96.309	2.390	-0.896	-4.490	-2.281	0.984	4.933	89.463	19.291	11.960
7152	210	33	26	P	W	97.311	-4.497	0.835	2.398	4.941	-0.797	-2.288	89.533	11.961	19.290
7153	210	33	26	P	W	109.842	2.281	-1.853	-4.395	-2.182	2.032	4.819	79.361	16.362	14.346
7154	210	33	26	P	W	110.485	-4.497	-0.835	2.398	4.941	0.797	-2.288	89.533	11.961	19.290
7155	210	33	26	P	W	111.487	2.390	0.896	-4.490	-2.281	-0.984	4.933	89.463	19.291	11.960
7156	210	33	26	P	W	113.413	-3.853	4.383	1.667	4.175	-4.241	-1.613	79.131	14.354	16.359
7157	211	31	29	C	S	8.513	2.390	-3.667	4.036	-2.281	3.393	-3.735	72.841	18.345	13.469
7158	211	31	29	C	S	9.430	5.011	-1.956	1.462	-4.554	1.900	-1.420	72.860	18.353	13.464
7159	211	31	29	C	S	12.520	2.326	3.611	4.103	-2.223	-3.337	-3.791	72.814	13.473	18.342
7160	211	31	29	C	S	47.480	2.326	-3.611	4.103	-2.223	3.337	-3.791	72.814	13.473	18.342
7161	211	31	29	C	S	50.570	5.011	1.956	1.462	-4.554	-1.900	-1.420	72.860	18.353	13.464
7162	211	31	29	C	S	51.487	2.390	3.667	4.036	-2.281	-3.393	-3.735	72.841	13.469	18.345
7163	211	31	29	C	S	68.513	2.390	-3.667	4.036	-2.281	3.393	-3.735	72.841	18.345	13.469
7164	211	31	29	C	S	69.430	5.011	-1.956	1.462	-4.554	1.900	-1.420	72.860	18.353	13.464
7165	211	31	29	C	S	72.520	2.326	3.611	4.103	-2.223	-3.337	-3.791	72.814	13.473	18.342
7166	211	31	29	C	S	107.480	2.326	-3.611	4.103	-2.223	3.337	-3.791	72.814	13.473	18.342
7167	211	31	29	C	S	110.570	5.011	1.956	1.462	-4.554	-1.900	-1.420	72.860	18.353	13.464
7168	211	31	29	C	S	111.487	2.390	3.667	4.036	-2.281	-3.393	-3.735	72.841	13.469	18.344
7169	211	31	29	C	W	8.513	2.390	-3.667	4.036	-2.281	3.393	-3.735	72.841	18.345	13.469
7170	211	31	29	C	W	9.430	5.011	-1.956	1.462	-4.554	1.900	-1.420	72.860	18.353	13.464
7171	211	31	29	C	W	12.520	2.326	3.611	4.103	-2.223	-3.337	-3.791	72.814	13.473	18.342
7172	211	31	29	C	W	47.480	2.326	-3.611	4.103	-2.223	3.337	-3.791	72.814	13.473	18.342
7173	211	31	29	C	W	50.570	5.011	1.956	1.462	-4.554	-1.900	-1.420	72.860	18.353	13.464
7174	211	31	29	C	W	51.487	2.390	3.667	4.036	-2.281	-3.393	-3.735	72.841	13.469	18.345
7175	211	31	29	C	W	68.513	2.390	-3.667	4.036	-2.281	3.393	-3.735	72.841	18.345	13.469
7176	211	31	29	C	W	69.430	5.011	-1.956	1.462	-4.554	1.900	-1.420	72.860	18.353	13.464
7177	211	31	29	C	W	72.520	2.326	3.611	4.103	-2.223	-3.337	-3.791	72.814	13.473	18.342
7178	211	31	29	C	W	107.480	2.326	-3.611	4.103	-2.223	3.337	-3.791	72.814	13.473	18.342
7179	211	31	29	C	W	110.570	5.011	1.956	1.462	-4.554	-1.900	-1.420	72.860	18.353	13.464
7180	211	31	29	C	W	111.487	2.390	3.667	4.036	-2.281	-3.393	-3.735	72.841	13.469	18.344
7181	211	31	29	P	S	8.513	2.390	-3.667	4.036	-2.281	3.393	-3.735	72.841	18.345	13.469
7182	211	31	29	P	S	9.430	5.011	-1.956	1.462	-4.554	1.900	-1.420	72.860	18.353	13.464
7183	211	31	29	P	S	12.520	2.326	3.611	4.103	-2.223	-3.337	-3.791	72.814	13.473	18.342
7184	211	31	29	P	S	47.480	2.326	-3.611	4.103	-2.223	3.337	-3.791	72.814	13.473	18.342
7185	211	31	29	P	S	50.570	5.011	1.956	1.462	-4.554	-1.900	-1.420	72.860	18.353	13.464
7186	211	31	29	P	S	51.487	2.390	3.667	4.036	-2.281	-3.393	-3.735	72.841	13.469	18.345
7187	211	31	29	P	S	68.513	2.390	-3.667	4.036	-2.281	3.393	-3.735	72.841	18.345	13.469
7188	211	31	29	P	S	69.430	5.011	-1.956	1.462	-4.554	1.900	-1.420	72.860	18.353	13.464
7189	211	31	29	P	S	72.520	2.326	3.611	4.103	-2.223	-3.337	-3.791	72.814	13.473	18.342
7190	211	31	29	P	S	107.480	2.326	-3.611	4.103	-2.223	3.337	-3.791	72.814	13.473	18.342
7191	211	31	29	P	S	110.570	5.011	1.956	1.462	-4.554	-1.900	-1.420	72.860	18.353	13.464

BL number	Atoms	γ-PC unit cells	WS_2 unit cells	γ-PC origin atom	WS_2 origin atom	Twist-angle (°)	γ-PC strain 1 (%)	γ-PC strain 2 (%)	γ-PC strain 3 (%)	WS_2 strain 1 (%)	WS_2 strain 2 (%)	WS_2 strain 3 (%)	γ (°)	|**a**|(Å)	|**b**|(Å)
7192	211	31	29	P	S	111.487	2.390	3.667	4.036	-2.281	-3.393	-3.735	72.841	13.469	18.344
7193	211	31	29	P	W	8.513	2.390	-3.667	4.036	-2.281	3.393	-3.735	72.841	18.345	13.469
7194	211	31	29	P	W	9.430	5.011	-1.956	1.462	-4.554	1.900	-1.420	72.860	18.353	13.464
7195	211	31	29	P	W	12.520	2.326	3.611	4.103	-2.223	-3.337	-3.791	72.814	13.473	18.342
7196	211	31	29	P	W	47.480	2.326	-3.611	4.103	-2.223	3.337	-3.791	72.814	13.473	18.342
7197	211	31	29	P	W	50.570	5.011	1.956	1.462	-4.554	-1.900	-1.420	72.860	18.353	13.464
7198	211	31	29	P	W	51.487	2.390	3.667	4.036	-2.281	-3.393	-3.735	72.841	13.469	18.345
7199	211	31	29	P	W	68.513	2.390	-3.667	4.036	-2.281	3.393	-3.735	72.841	18.345	13.469
7200	211	31	29	P	W	69.430	5.011	-1.956	1.462	-4.554	1.900	-1.420	72.860	18.353	13.464
7201	211	31	29	P	W	72.520	2.326	3.611	4.103	-2.223	-3.337	-3.791	72.814	13.473	18.342
7202	211	31	29	P	W	107.480	2.326	-3.611	4.103	-2.223	3.337	-3.791	72.814	13.473	18.342
7203	211	31	29	P	W	110.570	5.011	1.956	1.462	-4.554	-1.900	-1.420	72.860	18.353	13.464
7204	211	31	29	P	W	111.487	2.390	3.667	4.036	-2.281	-3.393	-3.735	72.841	13.469	18.344
7205	211	34	25	C	S	4.307	-4.685	-4.345	-0.897	5.169	4.424	0.913	85.625	14.491	15.891
7206	211	34	25	C	S	25.050	-0.955	-3.337	-4.631	0.973	3.677	5.103	68.485	14.833	16.638
7207	211	34	25	C	S	25.285	-4.781	-3.072	-0.791	5.287	3.121	0.804	68.556	16.641	14.824
7208	211	34	25	C	S	30.000	-2.359	-4.974	-3.293	2.476	5.325	3.525	68.479	14.845	16.620
7209	211	34	25	C	S	34.715	-4.781	3.072	-0.791	5.287	-3.121	0.804	68.556	16.641	14.824
7210	211	34	25	C	S	34.950	-0.955	3.337	-4.631	0.973	-3.677	5.103	68.485	16.638	14.833
7211	211	34	25	C	S	55.693	-4.685	4.345	-0.897	5.169	-4.424	0.913	85.625	14.491	15.891
7212	211	34	25	C	S	64.307	-4.685	-4.345	-0.897	5.169	4.424	0.913	85.625	14.491	15.891
7213	211	34	25	C	S	85.050	-0.955	-3.337	-4.631	0.973	3.677	5.103	68.485	14.833	16.638
7214	211	34	25	C	S	85.285	-4.781	-3.072	-0.791	5.287	3.121	0.804	68.556	16.641	14.824
7215	211	34	25	C	S	90.000	-2.359	4.974	-3.293	2.476	-5.325	3.526	68.479	14.845	16.620
7216	211	34	25	C	S	94.715	-4.781	3.072	-0.791	5.287	-3.121	0.804	68.556	16.641	14.824
7217	211	34	25	C	S	94.950	-0.955	3.337	-4.631	0.973	-3.677	5.103	68.485	16.638	14.833
7218	211	34	25	C	S	115.693	-4.685	4.345	-0.897	5.169	-4.424	0.913	85.625	14.491	15.891
7219	211	34	25	C	W	4.307	-4.685	-4.345	-0.897	5.169	4.424	0.913	85.625	14.491	15.891
7220	211	34	25	C	W	25.050	-0.955	-3.337	-4.631	0.973	3.677	5.103	68.485	14.833	16.638
7221	211	34	25	C	W	25.285	-4.781	-3.072	-0.791	5.287	3.121	0.804	68.556	16.641	14.824
7222	211	34	25	C	W	30.000	-2.359	-4.974	-3.293	2.476	5.325	3.525	68.479	14.845	16.620
7223	211	34	25	C	W	34.715	-4.781	3.072	-0.791	5.287	-3.121	0.804	68.556	16.641	14.824
7224	211	34	25	C	W	34.950	-0.955	3.337	-4.631	0.973	-3.677	5.103	68.485	16.638	14.833
7225	211	34	25	C	W	55.693	-4.685	4.345	-0.897	5.169	-4.424	0.913	85.625	14.491	15.891
7226	211	34	25	C	W	64.307	-4.685	-4.345	-0.897	5.169	4.424	0.913	85.625	14.491	15.891
7227	211	34	25	C	W	85.050	-0.955	-3.337	-4.631	0.973	3.677	5.103	68.485	14.833	16.638
7228	211	34	25	C	W	85.285	-4.781	-3.072	-0.791	5.287	3.121	0.804	68.556	16.641	14.824
7229	211	34	25	C	W	90.000	-2.359	4.974	-3.293	2.476	-5.325	3.526	68.479	14.845	16.620
7230	211	34	25	C	W	94.715	-4.781	3.072	-0.791	5.287	-3.121	0.804	68.556	16.641	14.824
7231	211	34	25	C	W	94.950	-0.955	3.337	-4.631	0.973	-3.677	5.103	68.485	16.638	14.833
7232	211	34	25	C	W	115.693	-4.685	4.345	-0.897	5.169	-4.424	0.913	85.625	14.491	15.891
7233	211	34	25	P	S	25.050	-0.955	-3.337	-4.631	0.973	3.677	5.103	68.485	14.833	16.638
7234	211	34	25	P	S	25.285	-4.781	-3.072	-0.791	5.287	3.121	0.804	68.556	16.641	14.824
7235	211	34	25	P	S	30.000	-2.359	-4.974	-3.293	2.476	5.325	3.525	68.479	14.845	16.620
7236	211	34	25	P	S	34.715	-4.781	3.072	-0.791	5.287	-3.121	0.804	68.556	16.641	14.824
7237	211	34	25	P	S	34.950	-0.955	3.337	-4.631	0.973	-3.677	5.103	68.485	16.638	14.833
7238	211	34	25	P	S	55.693	-4.685	4.345	-0.897	5.169	-4.424	0.913	85.625	14.491	15.891
7239	211	34	25	P	S	64.307	-4.685	-4.345	-0.897	5.169	4.424	0.913	85.625	14.491	15.891
7240	211	34	25	P	S	85.050	-0.955	-3.337	-4.631	0.973	3.677	5.103	68.485	14.833	16.638
7241	211	34	25	P	S	85.285	-4.781	-3.072	-0.791	5.287	3.121	0.804	68.556	16.641	14.824
7242	211	34	25	P	S	90.000	-2.359	4.974	-3.293	2.476	-5.325	3.526	68.479	14.845	16.620

BL number	Atoms	γ-PC unit cells	WS_2 unit cells	γ-PC origin atom	WS_2 origin atom	Twist-angle (°)	γ-PC strain 1 (%)	γ-PC strain 2 (%)	γ-PC strain 3 (%)	WS_2 strain 1 (%)	WS_2 strain 2 (%)	WS_2 strain 3 (%)	γ (°)	|**a**|(Å)	|**b**|(Å)
7243	211	34	25	P	S	94.715	-4.781	3.072	-0.791	5.287	-3.121	0.804	68.556	16.641	14.824
7244	211	34	25	P	S	94.950	-0.955	3.337	-4.631	0.973	-3.677	5.103	68.485	16.638	14.833
7245	211	34	25	P	W	4.307	-4.685	-4.345	-0.897	5.169	4.424	0.913	85.625	14.491	15.891
7246	211	34	25	P	W	25.050	-0.955	-3.337	-4.631	0.973	3.677	5.103	68.485	14.833	16.638
7247	211	34	25	P	W	25.285	-4.781	-3.072	-0.791	5.287	3.121	0.804	68.556	16.641	14.824
7248	211	34	25	P	W	30.000	-2.359	-4.974	-3.293	2.476	5.325	3.525	68.479	14.845	16.620
7249	211	34	25	P	W	34.715	-4.781	3.072	-0.791	5.287	-3.121	0.804	68.556	16.641	14.824
7250	211	34	25	P	W	34.950	-0.955	3.337	-4.631	0.973	-3.677	5.103	68.485	16.638	14.833
7251	211	34	25	P	W	85.050	-0.955	-3.337	-4.631	0.973	3.677	5.103	68.485	14.833	16.638
7252	211	34	25	P	W	85.285	-4.781	-3.072	-0.791	5.287	3.121	0.804	68.556	16.641	14.824
7253	211	34	25	P	W	90.000	-2.359	4.974	-3.293	2.476	-5.325	3.526	68.479	14.845	16.620
7254	211	34	25	P	W	94.715	-4.781	3.072	-0.791	5.287	-3.121	0.804	68.556	16.641	14.824
7255	211	34	25	P	W	94.950	-0.955	3.337	-4.631	0.973	-3.677	5.103	68.485	16.638	14.833
7256	211	34	25	P	W	115.693	-4.685	4.345	-0.897	5.169	-4.424	0.913	85.625	14.491	15.891
7257	212	32	28	C	S	6.178	-2.145	5.296	5.332	2.241	-4.786	-4.818	87.121	17.102	13.806
7258	212	32	28	C	S	9.430	5.011	-1.895	-1.865	-4.554	1.968	1.938	84.486	18.353	12.906
7259	212	32	28	C	S	10.893	-1.983	0.663	5.145	2.064	-0.601	-4.665	84.595	12.906	18.351
7260	212	32	28	C	S	15.608	-2.145	-5.297	5.332	2.241	4.786	-4.818	87.121	17.102	13.806
7261	212	32	28	C	S	23.413	0.481	0.227	2.454	-0.477	-0.216	-2.339	75.967	12.584	19.291
7262	212	32	28	C	S	36.309	2.390	-0.711	0.543	-2.281	0.703	-0.537	75.955	19.291	12.585
7263	212	32	28	C	S	36.587	0.481	-0.227	2.454	-0.477	0.216	-2.339	75.967	12.584	19.291
7264	212	32	28	C	S	44.392	-2.145	5.296	5.332	2.241	-4.786	-4.818	87.121	17.102	13.806
7265	212	32	28	C	S	49.107	-1.983	-0.663	5.145	2.064	0.601	-4.665	84.595	12.906	18.351
7266	212	32	28	C	S	50.570	5.011	1.895	-1.865	-4.554	-1.968	1.938	84.486	18.353	12.906
7267	212	32	28	C	S	53.822	-2.145	-5.297	5.332	2.241	4.786	-4.818	87.121	17.102	13.806
7268	212	32	28	C	S	66.178	-2.145	5.296	5.332	2.241	-4.786	-4.818	87.121	17.102	13.805
7269	212	32	28	C	S	69.430	5.011	-1.895	-1.865	-4.554	1.968	1.938	84.486	18.353	12.906
7270	212	32	28	C	S	70.893	-1.983	0.663	5.145	2.064	-0.601	-4.665	84.596	12.906	18.351
7271	212	32	28	C	S	75.609	-2.145	-5.297	5.332	2.241	4.786	-4.818	87.121	17.102	13.806
7272	212	32	28	C	S	83.413	0.481	0.227	2.454	-0.477	-0.216	-2.339	75.967	12.584	19.291
7273	212	32	28	C	S	83.691	2.390	0.711	0.543	-2.281	-0.703	-0.537	75.955	19.291	12.585
7274	212	32	28	C	S	96.309	2.390	-0.711	0.543	-2.281	0.703	-0.537	75.955	19.291	12.585
7275	212	32	28	C	S	104.392	-2.145	5.296	5.332	2.241	-4.786	-4.818	87.121	17.102	13.805
7276	212	32	28	C	S	109.107	-1.983	-0.663	5.145	2.064	0.601	-4.665	84.595	12.906	18.351
7277	212	32	28	C	S	110.570	5.011	1.895	-1.865	-4.554	-1.968	1.938	84.486	18.353	12.906
7278	212	32	28	C	S	113.822	-2.145	-5.297	5.332	2.241	4.786	-4.818	87.121	17.102	13.805
7279	212	32	28	C	W	6.178	-2.145	5.296	5.332	2.241	-4.786	-4.818	87.121	17.102	13.806
7280	212	32	28	C	W	9.430	5.011	-1.895	-1.865	-4.554	1.968	1.938	84.486	18.353	12.906
7281	212	32	28	C	W	10.893	-1.983	0.663	5.145	2.064	-0.601	-4.665	84.595	12.906	18.351
7282	212	32	28	C	W	15.608	-2.145	-5.297	5.332	2.241	4.786	-4.818	87.121	17.102	13.806
7283	212	32	28	C	W	23.413	0.481	0.227	2.454	-0.477	-0.216	-2.339	75.967	12.584	19.291
7284	212	32	28	C	W	23.691	2.390	0.711	0.543	-2.281	-0.703	-0.537	75.955	19.291	12.585
7285	212	32	28	C	W	36.309	2.390	-0.711	0.543	-2.281	0.703	-0.537	75.955	19.291	12.585
7286	212	32	28	C	W	44.392	-2.145	5.296	5.332	2.241	-4.786	-4.818	87.121	17.102	13.806
7287	212	32	28	C	W	49.107	-1.983	-0.663	5.145	2.064	0.601	-4.665	84.595	12.906	18.351
7288	212	32	28	C	W	50.570	5.011	1.895	-1.865	-4.554	-1.968	1.938	84.486	18.353	12.906
7289	212	32	28	C	W	53.822	-2.145	-5.297	5.332	2.241	4.786	-4.818	87.121	17.102	13.806
7290	212	32	28	C	W	66.178	-2.145	5.296	5.332	2.241	-4.786	-4.818	87.121	17.102	13.805
7291	212	32	28	C	W	69.430	5.011	-1.895	-1.865	-4.554	1.968	1.938	84.486	18.353	12.906
7292	212	32	28	C	W	70.893	-1.983	0.663	5.145	2.064	-0.601	-4.665	84.596	12.906	18.351
7293	212	32	28	C	W	75.609	-2.145	-5.297	5.332	2.241	4.786	-4.818	87.121	17.102	13.806

BL number	Atoms	γ-PC unit cells	WS_2 unit cells	γ-PC origin atom	WS_2 origin atom	Twist-angle (°)	γ-PC strain 1 (%)	γ-PC strain 2 (%)	γ-PC strain 3 (%)	WS_2 strain 1 (%)	WS_2 strain 2 (%)	WS_2 strain 3 (%)	γ (°)	|**a**|(Å)	|**b**|(Å)
7294	212	32	28	C	W	83.413	0.481	0.227	2.454	-0.477	-0.216	-2.339	75.967	12.584	19.291
7295	212	32	28	C	W	96.309	2.390	-0.711	0.543	-2.281	0.703	-0.537	75.955	19.291	12.585
7296	212	32	28	C	W	96.587	0.481	-0.227	2.454	-0.477	0.216	-2.339	75.967	12.584	19.291
7297	212	32	28	C	W	104.392	-2.145	5.296	5.332	2.241	-4.786	-4.818	87.121	17.102	13.805
7298	212	32	28	C	W	109.107	-1.983	-0.663	5.145	2.064	0.601	-4.665	84.595	12.906	18.351
7299	212	32	28	C	W	110.570	5.011	1.895	-1.865	-4.554	-1.968	1.938	84.486	18.353	12.906
7300	212	32	28	C	W	113.822	-2.145	-5.297	5.332	2.241	4.786	-4.818	87.121	17.102	13.805
7301	212	32	28	P	S	6.178	-2.145	5.296	5.332	2.241	-4.786	-4.818	87.121	17.102	13.806
7302	212	32	28	P	S	9.430	5.011	-1.895	-1.865	-4.554	1.968	1.938	84.486	18.353	12.906
7303	212	32	28	P	S	10.893	-1.983	0.663	5.145	2.064	-0.601	-4.665	84.595	12.906	18.351
7304	212	32	28	P	S	23.413	0.481	0.227	2.454	-0.477	-0.216	-2.339	75.967	12.584	19.291
7305	212	32	28	P	S	23.691	2.390	0.711	0.543	-2.281	-0.703	-0.537	75.955	19.291	12.585
7306	212	32	28	P	S	36.309	2.390	-0.711	0.543	-2.281	0.703	-0.537	75.955	19.291	12.585
7307	212	32	28	P	S	44.392	-2.145	5.296	5.332	2.241	-4.786	-4.818	87.121	17.102	13.806
7308	212	32	28	P	S	49.107	-1.983	-0.663	5.145	2.064	0.601	-4.665	84.595	12.906	18.351
7309	212	32	28	P	S	50.570	5.011	1.895	-1.865	-4.554	-1.968	1.938	84.486	18.353	12.906
7310	212	32	28	P	S	53.822	-2.145	-5.297	5.332	2.241	4.786	-4.818	87.121	17.102	13.806
7311	212	32	28	P	S	69.430	5.011	-1.895	-1.865	-4.554	1.968	1.938	84.486	18.353	12.906
7312	212	32	28	P	S	70.893	-1.983	0.663	5.145	2.064	-0.601	-4.665	84.596	12.906	18.351
7313	212	32	28	P	S	75.609	-2.145	-5.297	5.332	2.241	4.786	-4.818	87.121	17.102	13.806
7314	212	32	28	P	S	83.413	0.481	0.227	2.454	-0.477	-0.216	-2.339	75.967	12.584	19.291
7315	212	32	28	P	S	96.309	2.390	-0.711	0.543	-2.281	0.703	-0.537	75.955	19.291	12.585
7316	212	32	28	P	S	96.587	0.481	-0.227	2.454	-0.477	0.216	-2.339	75.967	12.584	19.291
7317	212	32	28	P	S	109.107	-1.983	-0.663	5.145	2.064	0.601	-4.665	84.595	12.906	18.351
7318	212	32	28	P	S	110.570	5.011	1.895	-1.865	-4.554	-1.968	1.938	84.486	18.353	12.906
7319	212	32	28	P	S	113.822	-2.145	-5.297	5.332	2.241	4.786	-4.818	87.121	17.102	13.805
7320	212	32	28	P	W	9.430	5.011	-1.895	-1.865	-4.554	1.968	1.938	84.486	18.353	12.906
7321	212	32	28	P	W	10.893	-1.983	0.663	5.145	2.064	-0.601	-4.665	84.595	12.906	18.351
7322	212	32	28	P	W	15.608	-2.145	-5.297	5.332	2.241	4.786	-4.818	87.121	17.102	13.806
7323	212	32	28	P	W	23.413	0.481	0.227	2.454	-0.477	-0.216	-2.339	75.967	12.584	19.291
7324	212	32	28	P	W	36.309	2.390	-0.711	0.543	-2.281	0.703	-0.537	75.955	19.291	12.585
7325	212	32	28	P	W	36.587	0.481	-0.227	2.454	-0.477	0.216	-2.339	75.967	12.584	19.291
7326	212	32	28	P	W	49.107	-1.983	-0.663	5.145	2.064	0.601	-4.665	84.595	12.906	18.351
7327	212	32	28	P	W	50.570	5.011	1.895	-1.865	-4.554	-1.968	1.938	84.486	18.353	12.906
7328	212	32	28	P	W	53.822	-2.145	-5.297	5.332	2.241	4.786	-4.818	87.121	17.102	13.806
7329	212	32	28	P	W	66.178	-2.145	5.296	5.332	2.241	-4.786	-4.818	87.121	17.102	13.805
7330	212	32	28	P	W	69.430	5.011	-1.895	-1.865	-4.554	1.968	1.938	84.486	18.353	12.906
7331	212	32	28	P	W	70.893	-1.983	0.663	5.145	2.064	-0.601	-4.665	84.596	12.906	18.351
7332	212	32	28	P	W	83.413	0.481	0.227	2.454	-0.477	-0.216	-2.339	75.967	12.584	19.291
7333	212	32	28	P	W	83.691	2.390	0.711	0.543	-2.281	-0.703	-0.537	75.955	19.291	12.585
7334	212	32	28	P	W	96.309	2.390	-0.711	0.543	-2.281	0.703	-0.537	75.955	19.291	12.585
7335	212	32	28	P	W	104.392	-2.145	5.296	5.332	2.241	-4.786	-4.818	87.121	17.102	13.805
7336	212	32	28	P	W	109.107	-1.983	-0.663	5.145	2.064	0.601	-4.665	84.595	12.906	18.351
7337	212	32	28	P	W	110.570	5.011	1.895	-1.865	-4.554	-1.968	1.938	84.486	18.353	12.906
7338	212	32	28	P	W	113.822	-2.145	-5.297	5.332	2.241	4.786	-4.818	87.121	17.102	13.805
7339	212	35	24	C	S	58.055	-4.723	-3.256	-4.169	5.216	3.552	4.548	79.839	15.242	15.228
7340	212	35	24	C	S	61.945	-4.723	3.256	-4.168	5.216	-3.552	4.548	79.839	15.242	15.228
7341	212	35	24	C	S	118.055	-4.723	-3.256	-4.169	5.216	3.552	4.548	79.839	15.242	15.228
7342	212	35	24	C	W	58.055	-4.723	-3.256	-4.169	5.216	3.552	4.548	79.839	15.242	15.228
7343	212	35	24	C	W	61.945	-4.723	3.256	-4.168	5.216	-3.552	4.548	79.839	15.242	15.228
7344	212	35	24	C	W	118.055	-4.723	-3.256	-4.169	5.216	3.552	4.548	79.839	15.242	15.228

BL number	Atoms	γ-PC unit cells	WS_2 unit cells	γ-PC origin atom	WS_2 origin atom	Twist-angle (°)	γ-PC strain 1 (%)	γ-PC strain 2 (%)	γ-PC strain 3 (%)	WS_2 strain 1 (%)	WS_2 strain 2 (%)	WS_2 strain 3 (%)	γ (°)	|**a**|(Å)	|**b**|(Å)
7345	212	35	24	P	S	58.055	-4.723	-3.256	-4.169	5.216	3.552	4.548	79.839	15.242	15.228
7346	212	35	24	P	S	61.945	-4.723	3.256	-4.168	5.216	-3.552	4.548	79.839	15.242	15.228
7347	212	35	24	P	W	118.055	-4.723	-3.256	-4.169	5.216	3.552	4.548	79.839	15.242	15.228
7348	213	33	27	C	S	19.107	-1.485	-2.216	1.035	1.530	2.171	-1.014	80.048	14.708	16.210
7349	213	33	27	C	S	38.948	1.339	-1.182	-1.772	-1.304	1.225	1.837	80.103	16.211	14.705
7350	213	33	27	C	S	40.893	-1.485	2.216	1.035	1.530	-2.171	-1.014	80.048	14.708	16.210
7351	213	33	27	C	S	79.107	-1.485	-2.216	1.035	1.530	2.171	-1.014	80.048	14.708	16.210
7352	213	33	27	C	S	81.052	1.339	1.182	-1.772	-1.304	-1.226	1.837	80.103	16.211	14.705
7353	213	33	27	C	S	100.893	-1.485	2.216	1.035	1.530	-2.171	-1.014	80.048	14.708	16.210
7354	213	33	27	C	W	21.052	1.339	1.182	-1.772	-1.304	-1.226	1.837	80.103	16.211	14.705
7355	213	33	27	C	W	98.948	1.339	-1.182	-1.772	-1.304	1.225	1.837	80.103	16.211	14.705
7356	213	33	27	P	S	19.107	-1.485	-2.216	1.035	1.530	2.171	-1.014	80.048	14.708	16.210
7357	213	33	27	P	S	38.948	1.339	-1.182	-1.772	-1.304	1.225	1.837	80.103	16.211	14.705
7358	213	33	27	P	S	40.893	-1.485	2.216	1.035	1.530	-2.171	-1.014	80.048	14.708	16.210
7359	213	33	27	P	S	79.107	-1.485	-2.216	1.035	1.530	2.171	-1.014	80.048	14.708	16.210
7360	213	33	27	P	S	81.052	1.339	1.182	-1.772	-1.304	-1.226	1.837	80.103	16.211	14.705
7361	213	33	27	P	S	100.893	-1.485	2.216	1.035	1.530	-2.171	-1.014	80.048	14.708	16.210
7362	213	33	27	P	W	19.107	-1.485	-2.216	1.035	1.530	2.171	-1.014	80.048	14.708	16.210
7363	213	33	27	P	W	21.052	1.339	1.182	-1.772	-1.304	-1.226	1.837	80.103	16.211	14.705
7364	213	33	27	P	W	98.948	1.339	-1.182	-1.772	-1.304	1.225	1.837	80.103	16.211	14.705
7365	214	31	30	C	S	13.174	5.011	-2.934	3.236	-4.554	2.755	-3.040	81.913	13.151	18.452
7366	214	31	30	C	S	16.102	2.853	2.395	5.410	-2.699	-2.161	-4.882	81.983	18.455	13.148
7367	214	31	30	C	S	21.787	5.011	-2.934	3.236	-4.554	2.755	-3.040	72.642	13.826	18.206
7368	214	31	30	C	S	25.285	4.262	3.398	3.971	-3.928	-3.148	-3.679	72.629	18.222	13.815
7369	214	31	30	C	S	34.715	4.262	-3.398	3.971	-3.928	3.148	-3.679	72.629	18.222	13.815
7370	214	31	30	C	S	38.213	5.011	2.934	3.236	-4.554	-2.755	-3.040	72.642	13.826	18.206
7371	214	31	30	C	S	43.898	2.853	-2.394	5.410	-2.699	2.161	-4.882	81.983	18.455	13.148
7372	214	31	30	C	S	46.827	5.011	2.934	3.236	-4.554	-2.755	-3.040	81.913	13.151	18.452
7373	214	31	30	C	S	73.174	5.011	-2.934	3.236	-4.554	2.755	-3.040	81.913	13.151	18.452
7374	214	31	30	C	S	76.102	2.853	2.395	5.410	-2.699	-2.161	-4.882	81.983	18.455	13.148
7375	214	31	30	C	S	81.787	5.011	-2.934	3.236	-4.554	2.755	-3.040	72.642	13.826	18.206
7376	214	31	30	C	S	85.285	4.262	3.398	3.971	-3.928	-3.148	-3.679	72.629	18.222	13.815
7377	214	31	30	C	S	94.715	4.262	-3.398	3.971	-3.928	3.148	-3.679	72.629	18.222	13.815
7378	214	31	30	C	S	98.213	5.011	2.934	3.236	-4.554	-2.755	-3.040	72.642	13.826	18.206
7379	214	31	30	C	S	103.898	2.853	-2.394	5.410	-2.699	2.161	-4.882	81.983	18.455	13.148
7380	214	31	30	C	S	106.827	5.011	2.934	3.236	-4.554	-2.755	-3.040	81.913	13.151	18.452
7381	214	31	30	C	W	13.174	5.011	-2.934	3.236	-4.554	2.755	-3.040	81.913	13.151	18.452
7382	214	31	30	C	W	16.102	2.853	2.395	5.410	-2.699	-2.161	-4.882	81.983	18.455	13.148
7383	214	31	30	C	W	21.787	5.011	-2.934	3.236	-4.554	2.755	-3.040	72.642	13.826	18.206
7384	214	31	30	C	W	25.285	4.262	3.398	3.971	-3.928	-3.148	-3.679	72.629	18.222	13.815
7385	214	31	30	C	W	34.715	4.262	-3.398	3.971	-3.928	3.148	-3.679	72.629	18.222	13.815
7386	214	31	30	C	W	38.213	5.011	2.934	3.236	-4.554	-2.755	-3.040	72.642	13.826	18.206
7387	214	31	30	C	W	43.898	2.853	-2.394	5.410	-2.699	2.161	-4.882	81.983	18.455	13.148
7388	214	31	30	C	W	46.827	5.011	2.934	3.236	-4.554	-2.755	-3.040	81.913	13.151	18.452
7389	214	31	30	C	W	73.174	5.011	-2.934	3.236	-4.554	2.755	-3.040	81.913	13.151	18.452
7390	214	31	30	C	W	76.102	2.853	2.395	5.410	-2.699	-2.161	-4.882	81.983	18.455	13.148
7391	214	31	30	C	W	81.787	5.011	-2.934	3.236	-4.554	2.755	-3.040	72.642	13.826	18.206
7392	214	31	30	C	W	85.285	4.262	3.398	3.971	-3.928	-3.148	-3.679	72.629	18.222	13.815
7393	214	31	30	C	W	94.715	4.262	-3.398	3.971	-3.928	3.148	-3.679	72.629	18.222	13.815
7394	214	31	30	C	W	98.213	5.011	2.934	3.236	-4.554	-2.755	-3.040	72.642	13.826	18.206
7395	214	31	30	C	W	103.898	2.853	-2.394	5.410	-2.699	2.161	-4.882	81.983	18.455	13.148

BL number	Atoms	γ-PC unit cells	WS_2 unit cells	γ-PC origin atom	WS_2 origin atom	Twist-angle (°)	γ-PC strain 1 (%)	γ-PC strain 2 (%)	γ-PC strain 3 (%)	WS_2 strain 1 (%)	WS_2 strain 2 (%)	WS_2 strain 3 (%)	γ (°)	$\lvert\mathbf{a}\rvert$(Å)	$\lvert\mathbf{b}\rvert$(Å)
7396	214	31	30	C	W	106.827	5.011	2.934	3.236	-4.554	-2.755	-3.040	81.913	13.151	18.452
7397	214	31	30	P	S	34.715	4.262	-3.398	3.971	-3.928	3.148	-3.679	72.629	18.222	13.815
7398	214	31	30	P	S	38.213	5.011	2.934	3.236	-4.554	-2.755	-3.040	72.642	13.826	18.206
7399	214	31	30	P	S	81.787	5.011	-2.934	3.236	-4.554	2.755	-3.040	72.642	13.826	18.206
7400	214	31	30	P	S	85.285	4.262	3.398	3.971	-3.928	-3.148	-3.679	72.629	18.222	13.815
7401	214	31	30	P	W	21.787	5.011	-2.934	3.236	-4.554	2.755	-3.040	72.642	13.826	18.206
7402	214	31	30	P	W	25.285	4.262	3.398	3.971	-3.928	-3.148	-3.679	72.629	18.222	13.815
7403	214	31	30	P	W	94.715	4.262	-3.398	3.971	-3.928	3.148	-3.679	72.629	18.222	13.815
7404	214	31	30	P	W	98.213	5.011	2.934	3.236	-4.554	-2.755	-3.040	72.642	13.826	18.206
7405	214	34	26	C	S	6.178	-2.145	-1.840	-1.642	2.241	1.902	1.698	67.191	17.102	14.840
7406	214	34	26	C	S	8.213	-2.359	1.658	-1.425	2.476	-1.707	1.467	67.193	14.845	17.097
7407	214	34	26	C	S	24.791	-3.388	-4.043	-0.352	3.635	4.072	0.355	65.614	17.335	14.821
7408	214	34	26	C	S	25.767	0.353	-2.254	-4.042	-0.351	2.452	4.397	65.539	14.834	17.335
7409	214	34	26	C	S	30.000	-2.359	4.974	-1.425	2.476	-5.120	1.467	65.575	14.845	17.309
7410	214	34	26	C	S	34.233	0.353	2.254	-4.042	-0.351	-2.452	4.397	65.539	17.335	14.834
7411	214	34	26	C	S	35.209	-3.388	4.043	-0.353	3.635	-4.072	0.355	65.614	17.335	14.821
7412	214	34	26	C	S	52.846	-0.955	0.163	-2.816	0.973	-0.172	2.984	67.172	17.101	14.844
7413	214	34	26	C	S	66.178	-2.145	-1.840	-1.642	2.241	1.902	1.698	67.191	17.102	14.840
7414	214	34	26	C	S	84.791	-3.388	-4.043	-0.352	3.635	4.072	0.355	65.613	17.335	14.821
7415	214	34	26	C	S	85.767	0.353	-2.254	-4.042	-0.351	2.452	4.397	65.540	14.834	17.335
7416	214	34	26	C	S	90.000	-2.359	-4.974	-1.425	2.476	5.120	1.467	65.575	14.845	17.309
7417	214	34	26	C	S	94.233	0.353	2.254	-4.042	-0.351	-2.452	4.397	65.539	17.335	14.834
7418	214	34	26	C	S	95.209	-3.388	4.043	-0.353	3.635	-4.072	0.355	65.613	17.335	14.821
7419	214	34	26	C	S	112.846	-0.955	0.163	-2.816	0.973	-0.172	2.984	67.172	17.101	14.844
7420	214	34	26	C	S	113.822	-2.145	1.840	-1.642	2.241	-1.902	1.698	67.191	17.102	14.840
7421	214	34	26	C	W	6.178	-2.145	-1.840	-1.642	2.241	1.902	1.698	67.191	17.102	14.840
7422	214	34	26	C	W	24.791	-3.388	-4.043	-0.352	3.635	4.072	0.355	65.614	17.335	14.821
7423	214	34	26	C	W	25.767	0.353	-2.254	-4.042	-0.351	2.452	4.397	65.539	14.834	17.335
7424	214	34	26	C	W	30.000	-2.359	4.974	-1.425	2.476	-5.120	1.467	65.575	14.845	17.309
7425	214	34	26	C	W	34.233	0.353	2.254	-4.042	-0.351	-2.452	4.397	65.539	17.335	14.834
7426	214	34	26	C	W	35.209	-3.388	4.043	-0.353	3.635	-4.072	0.355	65.614	17.335	14.821
7427	214	34	26	C	W	52.846	-0.955	0.163	-2.816	0.973	-0.172	2.984	67.172	17.101	14.844
7428	214	34	26	C	W	53.822	-2.145	1.840	-1.642	2.241	-1.902	1.698	67.191	17.102	14.840
7429	214	34	26	C	W	66.178	-2.145	-1.840	-1.642	2.241	1.902	1.698	67.191	17.102	14.840
7430	214	34	26	C	W	68.213	-2.359	1.658	-1.425	2.476	-1.707	1.467	67.193	14.845	17.097
7431	214	34	26	C	W	84.791	-3.388	-4.043	-0.352	3.635	4.072	0.355	65.613	17.335	14.821
7432	214	34	26	C	W	85.767	0.353	-2.254	-4.042	-0.351	2.452	4.397	65.540	14.834	17.335
7433	214	34	26	C	W	90.000	-2.359	-4.974	-1.425	2.476	5.120	1.467	65.575	14.845	17.309
7434	214	34	26	C	W	94.233	0.353	2.254	-4.042	-0.351	-2.452	4.397	65.539	17.335	14.834
7435	214	34	26	C	W	95.209	-3.388	4.043	-0.353	3.635	-4.072	0.355	65.613	17.335	14.821
7436	214	34	26	C	W	112.846	-0.955	0.163	-2.816	0.973	-0.172	2.984	67.172	17.101	14.844
7437	214	34	26	P	S	6.178	-2.145	-1.840	-1.642	2.241	1.902	1.698	67.191	17.102	14.840
7438	214	34	26	P	S	24.791	-3.388	-4.043	-0.352	3.635	4.072	0.355	65.614	17.335	14.821
7439	214	34	26	P	S	25.767	0.353	-2.254	-4.042	-0.351	2.452	4.397	65.539	14.834	17.335
7440	214	34	26	P	S	30.000	-2.359	4.974	-1.425	2.476	-5.120	1.467	65.575	14.845	17.309
7441	214	34	26	P	S	34.233	0.353	2.254	-4.042	-0.351	-2.452	4.397	65.539	17.335	14.834
7442	214	34	26	P	S	35.209	-3.388	4.043	-0.353	3.635	-4.072	0.355	65.614	17.335	14.821
7443	214	34	26	P	S	52.846	-0.955	0.163	-2.816	0.973	-0.172	2.984	67.172	17.101	14.844
7444	214	34	26	P	S	53.822	-2.145	1.840	-1.642	2.241	-1.902	1.698	67.191	17.102	14.840
7445	214	34	26	P	S	66.178	-2.145	-1.840	-1.642	2.241	1.902	1.698	67.191	17.102	14.840
7446	214	34	26	P	S	68.213	-2.359	1.658	-1.425	2.476	-1.707	1.467	67.193	14.845	17.097

BL number	Atoms	γ-PC unit cells	WS_2 unit cells	γ-PC origin atom	WS_2 origin atom	Twist-angle (°)	γ-PC strain 1 (%)	γ-PC strain 2 (%)	γ-PC strain 3 (%)	WS_2 strain 1 (%)	WS_2 strain 2 (%)	WS_2 strain 3 (%)	γ (°)	|**a**|(Å)	|**b**|(Å)
7447	214	34	26	P	S	84.791	-3.388	-4.043	-0.352	3.635	4.072	0.355	65.613	17.335	14.821
7448	214	34	26	P	S	85.767	0.353	-2.254	-4.042	-0.351	2.452	4.397	65.540	14.834	17.335
7449	214	34	26	P	S	90.000	-2.359	-4.974	-1.425	2.476	5.120	1.467	65.575	14.845	17.309
7450	214	34	26	P	S	94.233	0.353	2.254	-4.042	-0.351	-2.452	4.397	65.539	17.335	14.834
7451	214	34	26	P	S	95.209	-3.388	4.043	-0.353	3.635	-4.072	0.355	65.613	17.335	14.821
7452	214	34	26	P	S	112.846	-0.955	0.163	-2.816	0.973	-0.172	2.984	67.172	17.101	14.844
7453	214	34	26	P	W	6.178	-2.145	-1.840	-1.642	2.241	1.902	1.698	67.191	17.102	14.840
7454	214	34	26	P	W	8.213	-2.359	1.658	-1.425	2.476	-1.707	1.467	67.193	14.845	17.097
7455	214	34	26	P	W	24.791	-3.388	-4.043	-0.352	3.635	4.072	0.355	65.614	17.335	14.821
7456	214	34	26	P	W	25.767	0.353	-2.254	-4.042	-0.351	2.452	4.397	65.539	14.834	17.335
7457	214	34	26	P	W	30.000	-2.359	4.974	-1.425	2.476	-5.120	1.467	65.575	14.845	17.309
7458	214	34	26	P	W	34.233	0.353	2.254	-4.042	-0.351	-2.452	4.397	65.539	17.335	14.834
7459	214	34	26	P	W	35.209	-3.388	4.043	-0.353	3.635	-4.072	0.355	65.614	17.335	14.821
7460	214	34	26	P	W	52.846	-0.955	0.163	-2.816	0.973	-0.172	2.984	67.172	17.101	14.844
7461	214	34	26	P	W	66.178	-2.145	-1.840	-1.642	2.241	1.902	1.698	67.191	17.102	14.840
7462	214	34	26	P	W	84.791	-3.388	-4.043	-0.352	3.635	4.072	0.355	65.613	17.335	14.821
7463	214	34	26	P	W	85.767	0.353	-2.254	-4.042	-0.351	2.452	4.397	65.540	14.834	17.335
7464	214	34	26	P	W	90.000	-2.359	-4.974	-1.425	2.476	5.120	1.467	65.575	14.845	17.309
7465	214	34	26	P	W	94.233	0.353	2.254	-4.042	-0.351	-2.452	4.397	65.539	17.335	14.834
7466	214	34	26	P	W	95.209	-3.388	4.043	-0.353	3.635	-4.072	0.355	65.613	17.335	14.821
7467	214	34	26	P	W	112.846	-0.955	0.163	-2.816	0.973	-0.172	2.984	67.172	17.101	14.844
7468	214	34	26	P	W	113.822	-2.145	1.840	-1.642	2.241	-1.902	1.698	67.191	17.102	14.840
7469	215	32	29	C	S	13.174	5.011	-2.842	-0.146	-4.554	2.850	0.147	73.611	13.151	19.013
7470	215	32	29	C	S	13.898	-0.414	-1.659	5.308	0.417	1.499	-4.798	73.528	13.152	19.023
7471	215	32	29	C	S	17.696	1.029	5.246	3.744	-1.008	-4.881	-3.483	73.482	19.034	13.139
7472	215	32	29	C	S	21.787	5.011	-2.842	-0.146	-4.554	2.850	0.147	81.901	13.826	17.525
7473	215	32	29	C	S	25.767	0.353	4.293	4.465	-0.351	-3.941	-4.099	81.745	17.539	13.818
7474	215	32	29	C	S	34.233	0.353	-4.293	4.465	-0.351	3.941	-4.099	81.745	17.539	13.818
7475	215	32	29	C	S	38.213	5.011	2.842	-0.146	-4.554	-2.850	0.147	81.901	13.826	17.525
7476	215	32	29	C	S	42.304	1.029	-5.246	3.744	-1.008	4.881	-3.483	73.482	19.034	13.139
7477	215	32	29	C	S	46.102	-0.414	1.659	5.308	0.417	-1.499	-4.798	73.528	19.023	13.152
7478	215	32	29	C	S	46.827	5.011	2.842	-0.146	-4.554	-2.850	0.147	73.612	13.151	19.013
7479	215	32	29	C	S	73.174	5.011	-2.842	-0.146	-4.554	2.850	0.147	73.612	13.151	19.013
7480	215	32	29	C	S	73.898	-0.414	-1.659	5.308	0.417	1.499	-4.798	73.528	13.151	19.023
7481	215	32	29	C	S	77.696	1.029	5.246	3.744	-1.008	-4.881	-3.483	73.482	19.034	13.139
7482	215	32	29	C	S	81.787	5.011	-2.842	-0.146	-4.554	2.850	0.147	81.901	13.826	17.525
7483	215	32	29	C	S	85.767	0.353	4.293	4.465	-0.351	-3.941	-4.099	81.745	17.539	13.818
7484	215	32	29	C	S	94.233	0.353	-4.293	4.465	-0.351	3.941	-4.099	81.745	17.539	13.818
7485	215	32	29	C	S	98.213	5.011	2.842	-0.146	-4.554	-2.850	0.147	81.901	13.826	17.525
7486	215	32	29	C	S	102.304	1.029	-5.246	3.744	-1.008	4.881	-3.483	73.482	19.034	13.139
7487	215	32	29	C	S	106.102	-0.414	1.659	5.308	0.417	-1.499	-4.798	73.528	19.023	13.151
7488	215	32	29	C	S	106.827	5.011	2.842	-0.146	-4.554	-2.850	0.147	73.612	13.151	19.013
7489	215	32	29	C	W	13.174	5.011	-2.842	-0.146	-4.554	2.850	0.147	73.611	13.151	19.013
7490	215	32	29	C	W	13.898	-0.414	-1.659	5.308	0.417	1.499	-4.798	73.528	13.152	19.023
7491	215	32	29	C	W	17.696	1.029	5.246	3.744	-1.008	-4.881	-3.483	73.482	19.034	13.139
7492	215	32	29	C	W	21.787	5.011	-2.842	-0.146	-4.554	2.850	0.147	81.901	13.826	17.525
7493	215	32	29	C	W	25.767	0.353	4.293	4.465	-0.351	-3.941	-4.099	81.745	17.539	13.818
7494	215	32	29	C	W	34.233	0.353	-4.293	4.465	-0.351	3.941	-4.099	81.745	17.539	13.818
7495	215	32	29	C	W	38.213	5.011	2.842	-0.146	-4.554	-2.850	0.147	81.901	13.826	17.525
7496	215	32	29	C	W	42.304	1.029	-5.246	3.744	-1.008	4.881	-3.483	73.482	19.034	13.139
7497	215	32	29	C	W	46.102	-0.414	1.659	5.308	0.417	-1.499	-4.798	73.528	19.023	13.152

BL number	Atoms	γ-PC unit cells	WS_2 unit cells	γ-PC origin atom	WS_2 origin atom	Twist-angle (°)	γ-PC strain 1 (%)	γ-PC strain 2 (%)	γ-PC strain 3 (%)	WS_2 strain 1 (%)	WS_2 strain 2 (%)	WS_2 strain 3 (%)	γ (°)	$\lvert\mathbf{a}\rvert$(Å)	$\lvert\mathbf{b}\rvert$(Å)
7498	215	32	29	C	W	46.827	5.011	2.842	-0.146	-4.554	-2.850	0.147	73.612	13.151	19.013
7499	215	32	29	C	W	73.174	5.011	-2.842	-0.146	-4.554	2.850	0.147	73.612	13.151	19.013
7500	215	32	29	C	W	73.898	-0.414	-1.659	5.308	0.417	1.499	-4.798	73.528	13.151	19.023
7501	215	32	29	C	W	77.696	1.029	5.246	3.744	-1.008	-4.881	-3.483	73.482	19.034	13.139
7502	215	32	29	C	W	81.787	5.011	-2.842	-0.146	-4.554	2.850	0.147	81.901	13.826	17.525
7503	215	32	29	C	W	85.767	0.353	4.293	4.465	-0.351	-3.941	-4.099	81.745	17.539	13.818
7504	215	32	29	C	W	94.233	0.353	-4.293	4.465	-0.351	3.941	-4.099	81.745	17.539	13.818
7505	215	32	29	C	W	98.213	5.011	2.842	-0.146	-4.554	-2.850	0.147	81.901	13.826	17.525
7506	215	32	29	C	W	102.304	1.029	-5.246	3.744	-1.008	4.881	-3.483	73.482	19.034	13.139
7507	215	32	29	C	W	106.102	-0.414	1.659	5.308	0.417	-1.499	-4.798	73.528	19.023	13.151
7508	215	32	29	C	W	106.827	5.011	2.842	-0.146	-4.554	-2.850	0.147	73.612	13.151	19.013
7509	215	32	29	P	S	13.174	5.011	-2.842	-0.146	-4.554	2.850	0.147	73.611	13.151	19.013
7510	215	32	29	P	S	17.696	1.029	5.246	3.744	-1.008	-4.881	-3.483	73.482	19.034	13.139
7511	215	32	29	P	S	21.787	5.011	-2.842	-0.146	-4.554	2.850	0.147	81.901	13.826	17.525
7512	215	32	29	P	S	34.233	0.353	-4.293	4.465	-0.351	3.941	-4.099	81.745	17.539	13.818
7513	215	32	29	P	S	38.213	5.011	2.842	-0.146	-4.554	-2.850	0.147	81.901	13.826	17.525
7514	215	32	29	P	S	42.304	1.029	-5.246	3.744	-1.008	4.881	-3.483	73.482	19.034	13.139
7515	215	32	29	P	S	46.102	-0.414	1.659	5.308	0.417	-1.499	-4.798	73.528	19.023	13.152
7516	215	32	29	P	S	46.827	5.011	2.842	-0.146	-4.554	-2.850	0.147	73.612	13.151	19.013
7517	215	32	29	P	S	73.174	5.011	-2.842	-0.146	-4.554	2.850	0.147	73.612	13.151	19.013
7518	215	32	29	P	S	73.898	-0.414	-1.659	5.308	0.417	1.499	-4.798	73.528	13.151	19.023
7519	215	32	29	P	S	77.696	1.029	5.246	3.744	-1.008	-4.881	-3.483	73.482	19.034	13.139
7520	215	32	29	P	S	81.787	5.011	-2.842	-0.146	-4.554	2.850	0.147	81.901	13.826	17.525
7521	215	32	29	P	S	85.767	0.353	4.293	4.465	-0.351	-3.941	-4.099	81.745	17.539	13.818
7522	215	32	29	P	S	94.233	0.353	-4.293	4.465	-0.351	3.941	-4.099	81.745	17.539	13.818
7523	215	32	29	P	S	102.304	1.029	-5.246	3.744	-1.008	4.881	-3.483	73.482	19.034	13.139
7524	215	32	29	P	W	13.174	5.011	-2.842	-0.146	-4.554	2.850	0.147	73.611	13.151	19.013
7525	215	32	29	P	W	13.898	-0.414	-1.659	5.308	0.417	1.499	-4.798	73.528	13.152	19.023
7526	215	32	29	P	W	17.696	1.029	5.246	3.744	-1.008	-4.881	-3.483	73.482	19.034	13.139
7527	215	32	29	P	W	21.787	5.011	-2.842	-0.146	-4.554	2.850	0.147	81.901	13.826	17.525
7528	215	32	29	P	W	25.767	0.353	4.293	4.465	-0.351	-3.941	-4.099	81.745	17.539	13.818
7529	215	32	29	P	W	34.233	0.353	-4.293	4.465	-0.351	3.941	-4.099	81.745	17.539	13.818
7530	215	32	29	P	W	77.696	1.029	5.246	3.744	-1.008	-4.881	-3.483	73.482	19.034	13.139
7531	215	32	29	P	W	81.787	5.011	-2.842	-0.146	-4.554	2.850	0.147	81.901	13.826	17.525
7532	215	32	29	P	W	94.233	0.353	-4.293	4.465	-0.351	3.941	-4.099	81.745	17.539	13.818
7533	215	32	29	P	W	98.213	5.011	2.842	-0.146	-4.554	-2.850	0.147	81.901	13.826	17.525
7534	215	32	29	P	W	102.304	1.029	-5.246	3.744	-1.008	4.881	-3.483	73.482	19.034	13.139
7535	215	32	29	P	W	106.102	-0.414	1.659	5.308	0.417	-1.499	-4.798	73.528	19.023	13.151
7536	215	32	29	P	W	106.827	5.011	2.842	-0.146	-4.554	-2.850	0.147	73.612	13.151	19.013
7537	215	35	25	C	S	23.413	-2.043	-1.276	-4.927	2.130	1.415	5.466	84.380	14.072	16.639
7538	215	35	25	C	S	25.285	-4.781	1.885	-2.197	5.287	-1.971	2.298	84.314	16.641	14.072
7539	215	35	25	C	S	34.715	-4.781	-1.885	-2.197	5.287	1.971	2.298	84.314	16.641	14.072
7540	215	35	25	C	S	36.587	-2.043	1.276	-4.927	2.130	-1.415	5.466	84.380	14.072	16.639
7541	215	35	25	C	S	83.413	-2.043	-1.276	-4.927	2.130	1.415	5.466	84.380	14.072	16.639
7542	215	35	25	C	S	85.285	-4.781	1.885	-2.197	5.287	-1.971	2.298	84.314	16.641	14.072
7543	215	35	25	C	S	94.715	-4.781	-1.885	-2.197	5.287	1.971	2.298	84.314	16.641	14.072
7544	215	35	25	C	S	96.587	-2.043	1.276	-4.927	2.130	-1.415	5.466	84.380	14.072	16.639
7545	215	35	25	C	W	23.413	-2.043	-1.276	-4.927	2.130	1.415	5.466	84.380	14.072	16.639
7546	215	35	25	C	W	25.285	-4.781	1.885	-2.197	5.287	-1.971	2.298	84.314	16.641	14.072
7547	215	35	25	C	W	34.715	-4.781	-1.885	-2.197	5.287	1.971	2.298	84.314	16.641	14.072
7548	215	35	25	C	W	36.587	-2.043	1.276	-4.927	2.130	-1.415	5.466	84.380	14.072	16.639

BL number	Atoms	γ-PC unit cells	WS_2 unit cells	γ-PC origin atom	WS_2 origin atom	Twist-angle (°)	γ-PC strain 1 (%)	γ-PC strain 2 (%)	γ-PC strain 3 (%)	WS_2 strain 1 (%)	WS_2 strain 2 (%)	WS_2 strain 3 (%)	γ (°)	**a**(Å)	**b**(Å)
7549	215	35	25	C	W	83.413	-2.043	-1.276	-4.927	2.130	1.415	5.466	84.380	14.072	16.639
7550	215	35	25	C	W	85.285	-4.781	1.885	-2.197	5.287	-1.971	2.298	84.314	16.641	14.072
7551	215	35	25	C	W	94.715	-4.781	-1.885	-2.197	5.287	1.971	2.298	84.314	16.641	14.072
7552	215	35	25	C	W	96.587	-2.043	1.276	-4.927	2.130	-1.415	5.466	84.380	14.072	16.639
7553	215	35	25	P	S	23.413	-2.043	-1.276	-4.927	2.130	1.415	5.466	84.380	14.072	16.639
7554	215	35	25	P	S	25.285	-4.781	1.885	-2.197	5.287	-1.971	2.298	84.314	16.641	14.072
7555	215	35	25	P	S	94.715	-4.781	-1.885	-2.197	5.287	1.971	2.298	84.314	16.641	14.072
7556	215	35	25	P	S	96.587	-2.043	1.276	-4.927	2.130	-1.415	5.466	84.380	14.072	16.639
7557	215	35	25	P	W	34.715	-4.781	-1.885	-2.197	5.287	1.971	2.298	84.314	16.641	14.072
7558	215	35	25	P	W	36.587	-2.043	1.276	-4.927	2.130	-1.415	5.466	84.380	14.072	16.639
7559	215	35	25	P	W	83.413	-2.043	-1.276	-4.927	2.130	1.415	5.466	84.380	14.072	16.639
7560	215	35	25	P	W	85.285	-4.781	1.885	-2.197	5.287	-1.971	2.298	84.314	16.641	14.072
7561	216	33	28	C	S	0.000	5.011	-0.919	-3.324	-4.554	0.984	3.561	88.367	13.151	18.219
7562	216	33	28	C	S	1.359	-3.292	1.501	4.973	3.523	-1.365	-4.523	88.258	18.221	13.151
7563	216	33	28	C	S	3.004	-3.388	2.535	5.087	3.635	-2.301	-4.617	88.618	17.335	13.821
7564	216	33	28	C	S	7.154	-0.955	-4.779	2.353	0.973	4.564	-2.248	89.765	17.772	13.458
7565	216	33	28	C	S	10.893	-1.983	1.929	3.474	2.064	-1.804	-3.248	86.089	12.906	18.584
7566	216	33	28	C	S	16.537	-3.292	3.193	4.973	3.523	-2.904	-4.523	89.181	18.221	13.146
7567	216	33	28	C	S	17.696	1.029	-4.992	0.318	-1.008	4.961	-0.316	88.159	19.034	12.568
7568	216	33	28	C	S	21.787	5.011	-2.756	-3.324	-4.554	2.952	3.561	88.367	13.826	17.330
7569	216	33	28	C	S	23.413	0.481	-3.296	0.864	-0.477	3.240	-0.849	87.131	12.584	19.024
7570	216	33	28	C	S	24.791	-3.388	2.535	5.087	3.635	-2.301	-4.617	88.618	17.335	13.821
7571	216	33	28	C	S	25.693	2.326	0.707	-0.929	-2.223	-0.720	0.946	87.305	13.473	17.772
7572	216	33	28	C	S	32.874	1.029	-3.225	0.318	-1.008	3.204	-0.316	87.151	19.034	12.577
7573	216	33	28	C	S	34.950	-0.955	0.419	2.353	0.973	-0.400	-2.248	87.327	17.772	13.473
7574	216	33	28	C	S	35.209	-3.388	-2.535	5.087	3.635	2.301	-4.617	88.618	17.335	13.821
7575	216	33	28	C	S	36.587	0.481	-5.054	0.864	-0.477	4.968	-0.849	88.128	12.584	19.011
7576	216	33	28	C	S	38.213	5.011	2.756	-3.324	-4.554	-2.952	3.561	88.367	13.826	17.330
7577	216	33	28	C	S	42.304	1.029	4.992	0.318	-1.008	-4.961	-0.316	88.159	19.034	12.568
7578	216	33	28	C	S	43.463	-3.292	-3.193	4.973	3.523	2.904	-4.523	89.181	18.221	13.146
7579	216	33	28	C	S	46.827	5.011	2.756	-3.324	-4.554	-2.952	3.561	89.456	13.151	18.212
7580	216	33	28	C	S	47.480	2.326	-4.664	-0.929	-2.223	4.752	0.946	89.591	13.473	17.752
7581	216	33	28	C	S	49.107	-1.983	-1.929	3.474	2.064	1.804	-3.248	86.089	12.906	18.584
7582	216	33	28	C	S	50.817	3.582	1.102	-2.079	-3.342	-1.150	2.169	86.180	18.586	12.904
7583	216	33	28	C	S	52.846	-0.955	4.779	2.353	0.973	-4.564	-2.248	89.765	17.772	13.458
7584	216	33	28	C	S	56.996	-3.388	-2.535	5.087	3.635	2.301	-4.617	88.618	17.335	13.821
7585	216	33	28	C	S	58.641	-3.292	-1.501	4.973	3.523	1.365	-4.523	88.258	18.221	13.151
7586	216	33	28	C	S	60.000	5.011	2.756	-3.324	-4.554	-2.952	3.561	88.367	13.826	17.330
7587	216	33	28	C	S	61.359	-3.292	1.501	4.973	3.523	-1.365	-4.523	88.258	18.221	13.151
7588	216	33	28	C	S	63.005	-3.388	2.535	5.087	3.635	-2.301	-4.617	88.618	17.335	13.821
7589	216	33	28	C	S	67.154	-0.955	-4.779	2.353	0.973	4.564	-2.248	89.765	17.772	13.458
7590	216	33	28	C	S	69.183	3.582	-1.102	-2.079	-3.342	1.150	2.169	86.180	18.586	12.904
7591	216	33	28	C	S	70.893	-1.983	1.929	3.474	2.064	-1.804	-3.248	86.089	12.906	18.584
7592	216	33	28	C	S	72.520	2.326	4.664	-0.929	-2.223	-4.752	0.946	89.591	13.473	17.752
7593	216	33	28	C	S	73.174	5.011	-2.756	-3.324	-4.554	2.952	3.561	89.456	13.151	18.212
7594	216	33	28	C	S	77.696	1.029	-4.992	0.318	-1.008	4.961	-0.316	88.159	19.034	12.568
7595	216	33	28	C	S	81.787	5.011	-2.756	-3.324	-4.554	2.952	3.561	88.367	13.826	17.330
7596	216	33	28	C	S	83.413	0.481	-3.296	0.864	-0.477	3.240	-0.849	87.131	12.584	19.024
7597	216	33	28	C	S	84.791	-3.388	2.535	5.087	3.635	-2.301	-4.617	88.618	17.335	13.821
7598	216	33	28	C	S	85.694	2.326	0.707	-0.929	-2.223	-0.720	0.946	87.305	13.473	17.772
7599	216	33	28	C	S	87.126	1.029	3.225	0.318	-1.008	-3.204	-0.316	87.151	19.034	12.577

BL number	Atoms	γ-PC unit cells	WS_2 unit cells	γ-PC origin atom	WS_2 origin atom	Twist-angle (°)	γ-PC strain 1 (%)	γ-PC strain 2 (%)	γ-PC strain 3 (%)	WS_2 strain 1 (%)	WS_2 strain 2 (%)	WS_2 strain 3 (%)	γ (°)	$\lvert\mathbf{a}\rvert$(Å)	$\lvert\mathbf{b}\rvert$(Å)
7600	216	33	28	C	S	94.950	-0.955	0.419	2.353	0.973	-0.400	-2.248	87.327	17.772	13.473
7601	216	33	28	C	S	95.209	-3.388	-2.535	5.087	3.635	2.301	-4.617	88.618	17.335	13.821
7602	216	33	28	C	S	96.587	0.481	-5.054	0.864	-0.477	4.968	-0.849	88.128	12.584	19.011
7603	216	33	28	C	S	98.213	5.011	2.756	-3.324	-4.554	-2.952	3.561	88.367	13.826	17.330
7604	216	33	28	C	S	102.304	1.029	4.992	0.318	-1.008	-4.961	-0.316	88.159	19.034	12.568
7605	216	33	28	C	S	103.463	-3.292	-3.193	4.973	3.523	2.904	-4.523	89.181	18.221	13.146
7606	216	33	28	C	S	109.107	-1.983	-1.929	3.474	2.064	1.804	-3.248	86.089	12.906	18.584
7607	216	33	28	C	S	112.846	-0.955	4.779	2.353	0.973	-4.564	-2.248	89.765	17.772	13.458
7608	216	33	28	C	S	116.996	-3.388	-2.535	5.087	3.635	2.301	-4.617	88.618	17.335	13.821
7609	216	33	28	C	S	118.641	-3.292	-1.501	4.973	3.523	1.365	-4.523	88.258	18.221	13.151
7610	216	33	28	C	W	0.000	5.011	-0.919	-3.324	-4.554	0.984	3.561	88.367	13.151	18.219
7611	216	33	28	C	W	1.359	-3.292	1.501	4.973	3.523	-1.365	-4.523	88.258	18.221	13.151
7612	216	33	28	C	W	3.004	-3.388	2.535	5.087	3.635	-2.301	-4.617	88.618	17.335	13.821
7613	216	33	28	C	W	9.183	3.582	-1.102	-2.079	-3.342	1.150	2.169	86.180	18.586	12.904
7614	216	33	28	C	W	12.520	2.326	4.664	-0.929	-2.223	-4.752	0.946	89.591	13.473	17.752
7615	216	33	28	C	W	13.174	5.011	-2.756	-3.324	-4.554	2.952	3.561	89.456	13.151	18.212
7616	216	33	28	C	W	17.696	1.029	-4.992	0.318	-1.008	4.961	-0.316	88.159	19.034	12.568
7617	216	33	28	C	W	21.787	5.011	-2.756	-3.324	-4.554	2.952	3.561	88.367	13.826	17.330
7618	216	33	28	C	W	23.413	0.481	5.054	0.864	-0.477	-4.968	-0.849	88.128	12.584	19.011
7619	216	33	28	C	W	24.791	-3.388	2.535	5.087	3.635	-2.301	-4.617	88.618	17.335	13.821
7620	216	33	28	C	W	25.693	2.326	0.707	-0.929	-2.223	-0.720	0.946	87.305	13.473	17.772
7621	216	33	28	C	W	27.126	1.029	3.225	0.318	-1.008	-3.204	-0.316	87.151	19.034	12.577
7622	216	33	28	C	W	34.950	-0.955	0.419	2.353	0.973	-0.400	-2.248	87.327	17.772	13.473
7623	216	33	28	C	W	35.209	-3.388	-2.535	5.087	3.635	2.301	-4.617	88.618	17.335	13.821
7624	216	33	28	C	W	36.587	0.481	-5.054	0.864	-0.477	4.968	-0.849	88.128	12.584	19.011
7625	216	33	28	C	W	38.213	5.011	2.756	-3.324	-4.554	-2.952	3.561	88.367	13.826	17.330
7626	216	33	28	C	W	42.304	1.029	4.992	0.318	-1.008	-4.961	-0.316	88.159	19.034	12.568
7627	216	33	28	C	W	56.996	-3.388	-2.535	5.087	3.635	2.301	-4.617	88.618	17.335	13.821
7628	216	33	28	C	W	58.641	-3.292	-1.501	4.973	3.523	1.365	-4.523	88.258	18.221	13.151
7629	216	33	28	C	W	60.000	5.011	2.756	-3.324	-4.554	-2.952	3.561	88.367	13.826	17.330
7630	216	33	28	C	W	61.359	-3.292	1.501	4.973	3.523	-1.365	-4.523	88.258	18.221	13.151
7631	216	33	28	C	W	63.005	-3.388	2.535	5.087	3.635	-2.301	-4.617	88.618	17.335	13.821
7632	216	33	28	C	W	76.537	-3.292	3.193	4.973	3.523	-2.904	-4.523	89.181	18.221	13.146
7633	216	33	28	C	W	77.696	1.029	-4.992	0.318	-1.008	4.961	-0.316	88.159	19.034	12.568
7634	216	33	28	C	W	81.787	5.011	-2.756	-3.324	-4.554	2.952	3.561	88.367	13.826	17.330
7635	216	33	28	C	W	83.413	0.481	5.054	0.864	-0.477	-4.968	-0.849	88.128	12.584	19.011
7636	216	33	28	C	W	84.791	-3.388	2.535	5.087	3.635	-2.301	-4.617	88.618	17.335	13.821
7637	216	33	28	C	W	85.694	2.326	0.707	-0.929	-2.223	-0.720	0.946	87.305	13.473	17.772
7638	216	33	28	C	W	92.874	1.029	-3.225	0.318	-1.008	3.204	-0.316	87.151	19.034	12.577
7639	216	33	28	C	W	94.950	-0.955	0.419	2.353	0.973	-0.400	-2.248	87.327	17.772	13.473
7640	216	33	28	C	W	95.209	-3.388	-2.535	5.087	3.635	2.301	-4.617	88.618	17.335	13.821
7641	216	33	28	C	W	96.587	0.481	-5.054	0.864	-0.477	4.968	-0.849	88.128	12.584	19.011
7642	216	33	28	C	W	98.213	5.011	2.756	-3.324	-4.554	-2.952	3.561	88.367	13.826	17.330
7643	216	33	28	C	W	102.304	1.029	4.992	0.318	-1.008	-4.961	-0.316	88.159	19.034	12.568
7644	216	33	28	C	W	106.827	5.011	2.756	-3.324	-4.554	-2.952	3.561	89.456	13.151	18.212
7645	216	33	28	C	W	107.480	2.326	-4.664	-0.929	-2.223	4.752	0.946	89.591	13.473	17.752
7646	216	33	28	C	W	110.817	3.582	1.102	-2.079	-3.342	-1.150	2.169	86.180	18.586	12.904
7647	216	33	28	C	W	116.996	-3.388	-2.535	5.087	3.635	2.301	-4.617	88.618	17.335	13.821
7648	216	33	28	C	W	118.641	-3.292	-1.501	4.973	3.523	1.365	-4.523	88.258	18.221	13.151
7649	216	33	28	P	S	0.000	5.011	-0.919	-3.324	-4.554	0.984	3.561	88.367	13.151	18.219
7650	216	33	28	P	S	1.359	-3.292	1.501	4.973	3.523	-1.365	-4.523	88.258	18.221	13.151

BL number	Atoms	γ-PC unit cells	WS_2 unit cells	γ-PC origin atom	WS_2 origin atom	Twist-angle (°)	γ-PC strain 1 (%)	γ-PC strain 2 (%)	γ-PC strain 3 (%)	WS_2 strain 1 (%)	WS_2 strain 2 (%)	WS_2 strain 3 (%)	γ (°)	|**a**|(Å)	|**b**|(Å)
7651	216	33	28	P	S	3.004	-3.388	2.535	5.087	3.635	-2.301	-4.617	88.618	17.335	13.821
7652	216	33	28	P	S	7.154	-0.955	-4.779	2.353	0.973	4.564	-2.248	89.765	17.772	13.458
7653	216	33	28	P	S	10.893	-1.983	1.929	3.474	2.064	-1.804	-3.248	86.089	12.906	18.584
7654	216	33	28	P	S	16.537	-3.292	3.193	4.973	3.523	-2.904	-4.523	89.181	18.221	13.146
7655	216	33	28	P	S	17.696	1.029	-4.992	0.318	-1.008	4.961	-0.316	88.159	19.034	12.568
7656	216	33	28	P	S	23.413	0.481	-3.296	0.864	-0.477	3.240	-0.849	87.131	12.584	19.024
7657	216	33	28	P	S	25.693	2.326	0.707	-0.929	-2.223	-0.720	0.946	87.305	13.473	17.772
7658	216	33	28	P	S	32.874	1.029	-3.225	0.318	-1.008	3.204	-0.316	87.151	19.034	12.577
7659	216	33	28	P	S	35.209	-3.388	-2.535	5.087	3.635	2.301	-4.617	88.618	17.335	13.821
7660	216	33	28	P	S	36.587	0.481	-5.054	0.864	-0.477	4.968	-0.849	88.128	12.584	19.011
7661	216	33	28	P	S	38.213	5.011	2.756	-3.324	-4.554	-2.952	3.561	88.367	13.826	17.330
7662	216	33	28	P	S	42.304	1.029	4.992	0.318	-1.008	-4.961	-0.316	88.159	19.034	12.568
7663	216	33	28	P	S	43.463	-3.292	-3.193	4.973	3.523	2.904	-4.523	89.181	18.221	13.146
7664	216	33	28	P	S	46.827	5.011	2.756	-3.324	-4.554	-2.952	3.561	89.456	13.151	18.212
7665	216	33	28	P	S	47.480	2.326	-4.664	-0.929	-2.223	4.752	0.946	89.591	13.473	17.752
7666	216	33	28	P	S	49.107	-1.983	-1.929	3.474	2.064	1.804	-3.248	86.089	12.906	18.584
7667	216	33	28	P	S	50.817	3.582	1.102	-2.079	-3.342	-1.150	2.169	86.180	18.586	12.904
7668	216	33	28	P	S	52.846	-0.955	4.779	2.353	0.973	-4.564	-2.248	89.765	17.772	13.458
7669	216	33	28	P	S	58.641	-3.292	-1.501	4.973	3.523	1.365	-4.523	88.258	18.221	13.151
7670	216	33	28	P	S	60.000	5.011	0.919	-3.324	-4.554	-0.984	3.561	88.367	13.151	18.219
7671	216	33	28	P	S	61.359	-3.292	1.501	4.973	3.523	-1.365	-4.523	88.258	18.221	13.151
7672	216	33	28	P	S	67.154	-0.955	-4.779	2.353	0.973	4.564	-2.248	89.765	17.772	13.458
7673	216	33	28	P	S	69.183	3.582	-1.102	-2.079	-3.342	1.150	2.169	86.180	18.586	12.904
7674	216	33	28	P	S	70.893	-1.983	1.929	3.474	2.064	-1.804	-3.248	86.089	12.906	18.584
7675	216	33	28	P	S	72.520	2.326	4.664	-0.929	-2.223	-4.752	0.946	89.591	13.473	17.752
7676	216	33	28	P	S	73.174	5.011	-2.756	-3.324	-4.554	2.952	3.561	89.456	13.151	18.212
7677	216	33	28	P	S	76.537	-3.292	3.193	4.973	3.523	-2.904	-4.523	89.181	18.221	13.146
7678	216	33	28	P	S	77.696	1.029	-4.992	0.318	-1.008	4.961	-0.316	88.159	19.034	12.568
7679	216	33	28	P	S	81.787	5.011	-2.756	-3.324	-4.554	2.952	3.561	88.367	13.826	17.330
7680	216	33	28	P	S	83.413	0.481	-3.296	0.864	-0.477	3.240	-0.849	87.131	12.584	19.024
7681	216	33	28	P	S	84.791	-3.388	2.535	5.087	3.635	-2.301	-4.617	88.618	17.335	13.821
7682	216	33	28	P	S	87.126	1.029	3.225	0.318	-1.008	-3.204	-0.316	87.151	19.034	12.577
7683	216	33	28	P	S	94.950	-0.955	0.419	2.353	0.973	-0.400	-2.248	87.327	17.772	13.473
7684	216	33	28	P	S	96.587	0.481	-5.054	0.864	-0.477	4.968	-0.849	88.128	12.584	19.011
7685	216	33	28	P	S	102.304	1.029	4.992	0.318	-1.008	-4.961	-0.316	88.159	19.034	12.568
7686	216	33	28	P	S	103.463	-3.292	-3.193	4.973	3.523	2.904	-4.523	89.181	18.221	13.146
7687	216	33	28	P	S	109.107	-1.983	-1.929	3.474	2.064	1.804	-3.248	86.089	12.906	18.584
7688	216	33	28	P	S	112.846	-0.955	4.779	2.353	0.973	-4.564	-2.248	89.765	17.772	13.458
7689	216	33	28	P	S	116.996	-3.388	-2.535	5.087	3.635	2.301	-4.617	88.618	17.335	13.821
7690	216	33	28	P	S	118.641	-3.292	-1.501	4.973	3.523	1.365	-4.523	88.258	18.221	13.151
7691	216	33	28	P	W	0.000	5.011	-0.919	-3.324	-4.554	0.984	3.561	88.367	13.151	18.219
7692	216	33	28	P	W	1.359	-3.292	1.501	4.973	3.523	-1.365	-4.523	88.258	18.221	13.151
7693	216	33	28	P	W	9.183	3.582	-1.102	-2.079	-3.342	1.150	2.169	86.180	18.586	12.904
7694	216	33	28	P	W	12.520	2.326	4.664	-0.929	-2.223	-4.752	0.946	89.591	13.473	17.752
7695	216	33	28	P	W	13.174	5.011	-2.756	-3.324	-4.554	2.952	3.561	89.456	13.151	18.212
7696	216	33	28	P	W	17.696	1.029	-4.992	0.318	-1.008	4.961	-0.316	88.159	19.034	12.568
7697	216	33	28	P	W	21.787	5.011	-2.756	-3.324	-4.554	2.952	3.561	88.367	13.826	17.330
7698	216	33	28	P	W	23.413	0.481	5.054	0.864	-0.477	-4.968	-0.849	88.128	12.584	19.011
7699	216	33	28	P	W	24.791	-3.388	2.535	5.087	3.635	-2.301	-4.617	88.618	17.335	13.821
7700	216	33	28	P	W	27.126	1.029	3.225	0.318	-1.008	-3.204	-0.316	87.151	19.034	12.577
7701	216	33	28	P	W	34.950	-0.955	0.419	2.353	0.973	-0.400	-2.248	87.327	17.772	13.473

BL number	Atoms	γ-PC unit cells	WS_2 unit cells	γ-PC origin atom	WS_2 origin atom	Twist-angle (°)	γ-PC strain 1 (%)	γ-PC strain 2 (%)	γ-PC strain 3 (%)	WS_2 strain 1 (%)	WS_2 strain 2 (%)	WS_2 strain 3 (%)	γ (°)	|**a**|(Å)	|**b**|(Å)
7702	216	33	28	P	W	36.587	0.481	-5.054	0.864	-0.477	4.968	-0.849	88.128	12.584	19.011
7703	216	33	28	P	W	42.304	1.029	4.992	0.318	-1.008	-4.961	-0.316	88.159	19.034	12.568
7704	216	33	28	P	W	56.996	-3.388	-2.535	5.087	3.635	2.301	-4.617	88.618	17.335	13.821
7705	216	33	28	P	W	58.641	-3.292	-1.501	4.973	3.523	1.365	-4.523	88.258	18.221	13.151
7706	216	33	28	P	W	60.000	5.011	2.756	-3.324	-4.554	-2.952	3.561	88.367	13.826	17.330
7707	216	33	28	P	W	61.359	-3.292	1.501	4.973	3.523	-1.365	-4.523	88.258	18.221	13.151
7708	216	33	28	P	W	63.005	-3.388	2.535	5.087	3.635	-2.301	-4.617	88.618	17.335	13.821
7709	216	33	28	P	W	76.537	-3.292	3.193	4.973	3.523	-2.904	-4.523	89.181	18.221	13.146
7710	216	33	28	P	W	77.696	1.029	-4.992	0.318	-1.008	4.961	-0.316	88.159	19.034	12.568
7711	216	33	28	P	W	83.413	0.481	5.054	0.864	-0.477	-4.968	-0.849	88.128	12.584	19.011
7712	216	33	28	P	W	85.694	2.326	0.707	-0.929	-2.223	-0.720	0.946	87.305	13.473	17.772
7713	216	33	28	P	W	92.874	1.029	-3.225	0.318	-1.008	3.204	-0.316	87.151	19.034	12.577
7714	216	33	28	P	W	95.209	-3.388	-2.535	5.087	3.635	2.301	-4.617	88.618	17.335	13.821
7715	216	33	28	P	W	96.587	0.481	-5.054	0.864	-0.477	4.968	-0.849	88.128	12.584	19.011
7716	216	33	28	P	W	98.213	5.011	2.756	-3.324	-4.554	-2.952	3.561	88.367	13.826	17.330
7717	216	33	28	P	W	102.304	1.029	4.992	0.318	-1.008	-4.961	-0.316	88.159	19.034	12.568
7718	216	33	28	P	W	106.827	5.011	2.756	-3.324	-4.554	-2.952	3.561	89.456	13.151	18.212
7719	216	33	28	P	W	107.480	2.326	-4.664	-0.929	-2.223	4.752	0.946	89.591	13.473	17.752
7720	216	33	28	P	W	110.817	3.582	1.102	-2.079	-3.342	-1.150	2.169	86.180	18.586	12.904
7721	216	33	28	P	W	118.641	-3.292	-1.501	4.973	3.523	1.365	-4.523	88.258	18.221	13.151
7722	217	31	31	C	S	17.897	5.011	0.000	5.011	-4.554	0.000	-4.555	60.000	16.799	16.799
7723	217	31	31	C	S	42.103	5.011	0.000	5.011	-4.554	0.000	-4.555	60.000	16.799	16.799
7724	217	31	31	C	S	77.897	5.011	0.000	5.011	-4.554	0.000	-4.555	60.000	16.799	16.799
7725	217	31	31	C	S	102.104	5.011	0.000	5.011	-4.554	0.000	-4.555	60.000	16.799	16.799
7726	217	31	31	C	W	17.897	5.011	0.000	5.011	-4.554	0.000	-4.555	60.000	16.799	16.799
7727	217	31	31	C	W	42.103	5.011	0.000	5.011	-4.554	0.000	-4.555	60.000	16.799	16.799
7728	217	31	31	C	W	77.897	5.011	0.000	5.011	-4.554	0.000	-4.555	60.000	16.799	16.799
7729	217	31	31	C	W	102.104	5.011	0.000	5.011	-4.554	0.000	-4.555	60.000	16.799	16.799
7730	217	31	31	P	S	17.897	5.011	0.000	5.011	-4.554	0.000	-4.555	60.000	16.799	16.799
7731	217	31	31	P	S	102.104	5.011	0.000	5.011	-4.554	0.000	-4.555	60.000	16.799	16.799
7732	217	31	31	P	W	42.103	5.011	0.000	5.011	-4.554	0.000	-4.555	60.000	16.799	16.799
7733	217	31	31	P	W	77.897	5.011	0.000	5.011	-4.554	0.000	-4.555	60.000	16.799	16.799
7734	217	34	27	C	S	12.520	2.326	-2.058	-4.073	-2.223	2.240	4.434	76.569	13.473	18.207
7735	217	34	27	C	S	16.537	-3.292	4.953	1.450	3.523	-4.813	-1.409	76.338	18.221	13.470
7736	217	34	27	C	S	43.463	-3.292	-4.953	1.450	3.523	4.813	-1.409	76.338	18.221	13.470
7737	217	34	27	C	S	47.480	2.326	2.058	-4.073	-2.223	-2.240	4.434	76.569	13.473	18.207
7738	217	34	27	C	S	72.520	2.326	-2.058	-4.073	-2.223	2.240	4.434	76.569	13.473	18.207
7739	217	34	27	C	S	76.537	-3.292	4.953	1.450	3.523	-4.813	-1.409	76.338	18.221	13.470
7740	217	34	27	C	S	103.463	-3.292	-4.953	1.450	3.523	4.813	-1.409	76.338	18.221	13.470
7741	217	34	27	C	S	107.480	2.326	2.058	-4.073	-2.223	-2.240	4.434	76.569	13.473	18.207
7742	217	34	27	C	W	12.520	2.326	-2.058	-4.073	-2.223	2.240	4.434	76.569	13.473	18.207
7743	217	34	27	C	W	16.537	-3.292	4.953	1.450	3.523	-4.813	-1.409	76.338	18.221	13.470
7744	217	34	27	C	W	43.463	-3.292	-4.953	1.450	3.523	4.813	-1.409	76.338	18.221	13.470
7745	217	34	27	C	W	47.480	2.326	2.058	-4.073	-2.223	-2.240	4.434	76.569	13.473	18.207
7746	217	34	27	C	W	72.520	2.326	-2.058	-4.073	-2.223	2.240	4.434	76.569	13.473	18.207
7747	217	34	27	C	W	76.537	-3.292	4.953	1.450	3.523	-4.813	-1.409	76.338	18.221	13.470
7748	217	34	27	C	W	103.463	-3.292	-4.953	1.450	3.523	4.813	-1.409	76.338	18.221	13.470
7749	217	34	27	C	W	107.480	2.326	2.058	-4.073	-2.223	-2.240	4.434	76.569	13.473	18.207
7750	217	34	27	P	S	12.520	2.326	-2.058	-4.073	-2.223	2.240	4.434	76.569	13.473	18.207
7751	217	34	27	P	S	16.537	-3.292	4.953	1.450	3.523	-4.813	-1.409	76.338	18.221	13.470
7752	217	34	27	P	S	43.463	-3.292	-4.953	1.450	3.523	4.813	-1.409	76.338	18.221	13.470

BL number	Atoms	γ-PC unit cells	WS_2 unit cells	γ-PC origin atom	WS_2 origin atom	Twist-angle (°)	γ-PC strain 1 (%)	γ-PC strain 2 (%)	γ-PC strain 3 (%)	WS_2 strain 1 (%)	WS_2 strain 2 (%)	WS_2 strain 3 (%)	γ (°)	|**a**|(Å)	|**b**|(Å)
7753	217	34	27	P	S	47.480	2.326	2.058	-4.073	-2.223	-2.240	4.434	76.569	13.473	18.207
7754	217	34	27	P	S	72.520	2.326	-2.058	-4.073	-2.223	2.240	4.434	76.569	13.473	18.207
7755	217	34	27	P	S	76.537	-3.292	4.953	1.450	3.523	-4.813	-1.409	76.338	18.221	13.470
7756	217	34	27	P	S	103.463	-3.292	-4.953	1.450	3.523	4.813	-1.409	76.338	18.221	13.470
7757	217	34	27	P	S	107.480	2.326	2.058	-4.073	-2.223	-2.240	4.434	76.569	13.473	18.207
7758	217	34	27	P	W	12.520	2.326	-2.058	-4.073	-2.223	2.240	4.434	76.569	13.473	18.207
7759	217	34	27	P	W	16.537	-3.292	4.953	1.450	3.523	-4.813	-1.409	76.338	18.221	13.470
7760	217	34	27	P	W	43.463	-3.292	-4.953	1.450	3.523	4.813	-1.409	76.338	18.221	13.470
7761	217	34	27	P	W	47.480	2.326	2.058	-4.073	-2.223	-2.240	4.434	76.569	13.473	18.207
7762	217	34	27	P	W	72.520	2.326	-2.058	-4.073	-2.223	2.240	4.434	76.569	13.473	18.207
7763	217	34	27	P	W	76.537	-3.292	4.953	1.450	3.523	-4.813	-1.409	76.338	18.221	13.470
7764	217	34	27	P	W	103.463	-3.292	-4.953	1.450	3.523	4.813	-1.409	76.338	18.221	13.470
7765	217	34	27	P	W	107.480	2.326	2.058	-4.073	-2.223	-2.240	4.434	76.569	13.473	18.207
7766	218	32	30	C	S	9.183	3.582	-1.818	2.948	-3.342	1.717	-2.784	70.546	15.808	16.366
7767	218	32	30	C	S	10.158	2.281	-0.066	4.265	-2.182	0.061	-3.930	70.539	16.362	15.814
7768	218	32	30	C	S	10.893	4.020	1.251	2.519	-3.721	-1.191	-2.398	70.510	15.815	16.364
7769	218	32	30	C	S	16.102	2.853	-3.247	3.679	-2.699	3.025	-3.426	69.572	16.790	15.505
7770	218	32	30	C	S	19.107	1.980	2.208	4.580	-1.905	-2.023	-4.195	69.545	15.504	16.796
7771	218	32	30	C	S	25.285	4.262	-0.157	2.284	-3.928	0.150	-2.184	83.616	18.222	13.473
7772	218	32	30	C	S	25.693	2.326	0.583	4.219	-2.223	-0.538	-3.891	83.616	13.473	18.221
7773	218	32	30	C	S	34.715	4.262	0.157	2.284	-3.928	-0.150	-2.184	83.616	18.222	13.473
7774	218	32	30	C	S	40.893	1.980	-2.208	4.580	-1.905	2.023	-4.195	69.545	15.504	16.796
7775	218	32	30	C	S	43.898	2.853	3.247	3.679	-2.699	-3.025	-3.427	69.572	15.505	16.790
7776	218	32	30	C	S	49.107	4.020	-1.251	2.519	-3.721	1.191	-2.398	70.510	15.815	16.364
7777	218	32	30	C	S	49.842	2.281	0.066	4.265	-2.182	-0.061	-3.930	70.539	16.362	15.814
7778	218	32	30	C	S	50.817	3.582	1.818	2.948	-3.342	-1.717	-2.784	70.546	16.366	15.808
7779	218	32	30	C	S	69.183	3.582	-1.818	2.948	-3.342	1.717	-2.784	70.546	15.808	16.366
7780	218	32	30	C	S	70.158	2.281	-0.066	4.265	-2.182	0.061	-3.930	70.539	16.362	15.814
7781	218	32	30	C	S	70.893	4.020	1.251	2.519	-3.721	-1.191	-2.398	70.510	15.815	16.364
7782	218	32	30	C	S	76.102	2.853	-3.247	3.679	-2.699	3.025	-3.426	69.572	16.790	15.505
7783	218	32	30	C	S	79.107	1.980	2.208	4.580	-1.905	-2.023	-4.195	69.545	15.504	16.796
7784	218	32	30	C	S	85.694	2.326	0.583	4.219	-2.223	-0.538	-3.891	83.616	13.473	18.221
7785	218	32	30	C	S	94.307	2.326	-0.583	4.219	-2.223	0.538	-3.891	83.616	13.473	18.221
7786	218	32	30	C	S	94.715	4.262	0.157	2.284	-3.928	-0.150	-2.184	83.616	18.222	13.473
7787	218	32	30	C	S	100.893	1.980	-2.208	4.580	-1.905	2.023	-4.195	69.545	15.504	16.796
7788	218	32	30	C	S	103.898	2.853	3.247	3.679	-2.699	-3.025	-3.427	69.572	15.505	16.790
7789	218	32	30	C	S	109.107	4.020	-1.251	2.519	-3.721	1.191	-2.398	70.510	15.815	16.364
7790	218	32	30	C	S	109.842	2.281	0.066	4.265	-2.182	-0.061	-3.930	70.539	16.362	15.814
7791	218	32	30	C	S	110.817	3.582	1.818	2.948	-3.342	-1.717	-2.784	70.546	16.366	15.808
7792	218	32	30	C	W	9.183	3.582	-1.818	2.948	-3.342	1.717	-2.784	70.546	15.808	16.366
7793	218	32	30	C	W	10.158	2.281	-0.066	4.265	-2.182	0.061	-3.930	70.539	16.362	15.814
7794	218	32	30	C	W	10.893	4.020	1.251	2.519	-3.721	-1.191	-2.398	70.510	15.815	16.364
7795	218	32	30	C	W	16.102	2.853	-3.247	3.679	-2.699	3.025	-3.426	69.572	16.790	15.505
7796	218	32	30	C	W	19.107	1.980	2.208	4.580	-1.905	-2.023	-4.195	69.545	15.504	16.796
7797	218	32	30	C	W	25.693	2.326	0.583	4.219	-2.223	-0.538	-3.891	83.616	13.473	18.221
7798	218	32	30	C	W	34.307	2.326	-0.583	4.219	-2.223	0.538	-3.891	83.616	13.473	18.221
7799	218	32	30	C	W	34.715	4.262	0.157	2.284	-3.928	-0.150	-2.184	83.616	18.222	13.473
7800	218	32	30	C	W	40.893	1.980	-2.208	4.580	-1.905	2.023	-4.195	69.545	15.504	16.796
7801	218	32	30	C	W	43.898	2.853	3.247	3.679	-2.699	-3.025	-3.427	69.572	15.505	16.790
7802	218	32	30	C	W	49.107	4.020	-1.251	2.519	-3.721	1.191	-2.398	70.510	15.815	16.364
7803	218	32	30	C	W	49.842	2.281	0.066	4.265	-2.182	-0.061	-3.930	70.539	16.362	15.814

BL number	Atoms	γ-PC unit cells	WS_2 unit cells	γ-PC origin atom	WS_2 origin atom	Twist-angle (°)	γ-PC strain 1 (%)	γ-PC strain 2 (%)	γ-PC strain 3 (%)	WS_2 strain 1 (%)	WS_2 strain 2 (%)	WS_2 strain 3 (%)	γ (°)	|**a**|(Å)	|**b**|(Å)
7804	218	32	30	C	W	50.817	3.582	1.818	2.948	-3.342	-1.717	-2.784	70.546	16.366	15.808
7805	218	32	30	C	W	69.183	3.582	-1.818	2.948	-3.342	1.717	-2.784	70.546	15.808	16.366
7806	218	32	30	C	W	70.158	2.281	-0.066	4.265	-2.182	0.061	-3.930	70.539	16.362	15.814
7807	218	32	30	C	W	70.893	4.020	1.251	2.519	-3.721	-1.191	-2.398	70.510	15.815	16.364
7808	218	32	30	C	W	76.102	2.853	-3.247	3.679	-2.699	3.025	-3.426	69.572	16.790	15.505
7809	218	32	30	C	W	79.107	1.980	2.208	4.580	-1.905	-2.023	-4.195	69.545	15.504	16.796
7810	218	32	30	C	W	85.285	4.262	-0.157	2.284	-3.928	0.150	-2.184	83.616	18.222	13.473
7811	218	32	30	C	W	85.694	2.326	0.583	4.219	-2.223	-0.538	-3.891	83.616	13.473	18.221
7812	218	32	30	C	W	94.715	4.262	0.157	2.284	-3.928	-0.150	-2.184	83.616	18.222	13.473
7813	218	32	30	C	W	100.893	1.980	-2.208	4.580	-1.905	2.023	-4.195	69.545	15.504	16.796
7814	218	32	30	C	W	103.898	2.853	3.247	3.679	-2.699	-3.025	-3.427	69.572	15.505	16.790
7815	218	32	30	C	W	109.107	4.020	-1.251	2.519	-3.721	1.191	-2.398	70.510	15.815	16.364
7816	218	32	30	C	W	109.842	2.281	0.066	4.265	-2.182	-0.061	-3.930	70.539	16.362	15.814
7817	218	32	30	C	W	110.817	3.582	1.818	2.948	-3.342	-1.717	-2.784	70.546	16.366	15.808
7818	218	32	30	P	S	9.183	3.582	-1.818	2.948	-3.342	1.717	-2.784	70.546	15.808	16.366
7819	218	32	30	P	S	10.158	2.281	-0.066	4.265	-2.182	0.061	-3.930	70.539	16.362	15.814
7820	218	32	30	P	S	10.893	4.020	1.251	2.519	-3.721	-1.191	-2.398	70.510	15.815	16.364
7821	218	32	30	P	S	25.693	2.326	0.583	4.219	-2.223	-0.538	-3.891	83.616	13.473	18.221
7822	218	32	30	P	S	34.307	2.326	-0.583	4.219	-2.223	0.538	-3.891	83.616	13.473	18.221
7823	218	32	30	P	S	34.715	4.262	0.157	2.284	-3.928	-0.150	-2.184	83.616	18.222	13.473
7824	218	32	30	P	S	49.107	4.020	-1.251	2.519	-3.721	1.191	-2.398	70.510	15.815	16.364
7825	218	32	30	P	S	49.842	2.281	0.066	4.265	-2.182	-0.061	-3.930	70.539	16.362	15.814
7826	218	32	30	P	S	50.817	3.582	1.818	2.948	-3.342	-1.717	-2.784	70.546	16.366	15.808
7827	218	32	30	P	S	69.183	3.582	-1.818	2.948	-3.342	1.717	-2.784	70.546	15.808	16.366
7828	218	32	30	P	S	70.158	2.281	-0.066	4.265	-2.182	0.061	-3.930	70.539	16.362	15.814
7829	218	32	30	P	S	70.893	4.020	1.251	2.519	-3.721	-1.191	-2.398	70.510	15.815	16.364
7830	218	32	30	P	S	85.285	4.262	-0.157	2.284	-3.928	0.150	-2.184	83.616	18.222	13.473
7831	218	32	30	P	S	85.694	2.326	0.583	4.219	-2.223	-0.538	-3.891	83.616	13.473	18.221
7832	218	32	30	P	S	94.715	4.262	0.157	2.284	-3.928	-0.150	-2.184	83.616	18.222	13.473
7833	218	32	30	P	S	109.107	4.020	-1.251	2.519	-3.721	1.191	-2.398	70.510	15.815	16.364
7834	218	32	30	P	S	109.842	2.281	0.066	4.265	-2.182	-0.061	-3.930	70.539	16.362	15.814
7835	218	32	30	P	S	110.817	3.582	1.818	2.948	-3.342	-1.717	-2.784	70.546	16.366	15.808
7836	218	32	30	P	W	9.183	3.582	-1.818	2.948	-3.342	1.717	-2.784	70.546	15.808	16.366
7837	218	32	30	P	W	10.158	2.281	-0.066	4.265	-2.182	0.061	-3.930	70.539	16.362	15.814
7838	218	32	30	P	W	10.893	4.020	1.251	2.519	-3.721	-1.191	-2.398	70.510	15.815	16.364
7839	218	32	30	P	W	25.285	4.262	-0.157	2.284	-3.928	0.150	-2.184	83.616	18.222	13.473
7840	218	32	30	P	W	25.693	2.326	0.583	4.219	-2.223	-0.538	-3.891	83.616	13.473	18.221
7841	218	32	30	P	W	34.715	4.262	0.157	2.284	-3.928	-0.150	-2.184	83.616	18.222	13.473
7842	218	32	30	P	W	49.107	4.020	-1.251	2.519	-3.721	1.191	-2.398	70.510	15.815	16.364
7843	218	32	30	P	W	49.842	2.281	0.066	4.265	-2.182	-0.061	-3.930	70.539	16.362	15.814
7844	218	32	30	P	W	50.817	3.582	1.818	2.948	-3.342	-1.717	-2.784	70.546	16.366	15.808
7845	218	32	30	P	W	69.183	3.582	-1.818	2.948	-3.342	1.717	-2.784	70.546	15.808	16.366
7846	218	32	30	P	W	70.158	2.281	-0.066	4.265	-2.182	0.061	-3.930	70.539	16.362	15.814
7847	218	32	30	P	W	70.893	4.020	1.251	2.519	-3.721	-1.191	-2.398	70.510	15.815	16.364
7848	218	32	30	P	W	85.694	2.326	0.583	4.219	-2.223	-0.538	-3.891	83.616	13.473	18.221
7849	218	32	30	P	W	94.307	2.326	-0.583	4.219	-2.223	0.538	-3.891	83.616	13.473	18.221
7850	218	32	30	P	W	94.715	4.262	0.157	2.284	-3.928	-0.150	-2.184	83.616	18.222	13.473
7851	218	32	30	P	W	109.107	4.020	-1.251	2.519	-3.721	1.191	-2.398	70.510	15.815	16.364
7852	218	32	30	P	W	109.842	2.281	0.066	4.265	-2.182	-0.061	-3.930	70.539	16.362	15.814
7853	218	32	30	P	W	110.817	3.582	1.818	2.948	-3.342	-1.717	-2.784	70.546	16.366	15.808
7854	218	35	26	C	S	24.791	-3.388	0.854	-1.771	3.635	-0.885	1.836	76.793	17.335	14.070

BL number	Atoms	γ-PC unit cells	WS_2 unit cells	γ-PC origin atom	WS_2 origin atom	Twist-angle (°)	γ-PC strain 1 (%)	γ-PC strain 2 (%)	γ-PC strain 3 (%)	WS_2 strain 1 (%)	WS_2 strain 2 (%)	WS_2 strain 3 (%)	γ (°)	|**a**|(Å)	|**b**|(Å)
7855	218	35	26	C	S	35.209	-3.388	-0.854	-1.771	3.635	0.885	1.836	76.793	17.335	14.070
7856	218	35	26	C	S	84.791	-3.388	0.854	-1.771	3.635	-0.885	1.836	76.793	17.335	14.070
7857	218	35	26	C	S	95.209	-3.388	-0.854	-1.771	3.635	0.885	1.836	76.793	17.335	14.070
7858	218	35	26	C	W	24.791	-3.388	0.854	-1.771	3.635	-0.885	1.836	76.793	17.335	14.070
7859	218	35	26	C	W	35.209	-3.388	-0.854	-1.771	3.635	0.885	1.836	76.793	17.335	14.070
7860	218	35	26	C	W	84.791	-3.388	0.854	-1.771	3.635	-0.885	1.836	76.793	17.335	14.070
7861	218	35	26	C	W	95.209	-3.388	-0.854	-1.771	3.635	0.885	1.836	76.793	17.335	14.070
7862	218	35	26	P	S	24.791	-3.388	0.854	-1.771	3.635	-0.885	1.836	76.793	17.335	14.070
7863	218	35	26	P	S	35.209	-3.388	-0.854	-1.771	3.635	0.885	1.836	76.793	17.335	14.070
7864	218	35	26	P	S	84.791	-3.388	0.854	-1.771	3.635	-0.885	1.836	76.793	17.335	14.070
7865	218	35	26	P	S	95.209	-3.388	-0.854	-1.771	3.635	0.885	1.836	76.793	17.335	14.070
7866	218	35	26	P	W	24.791	-3.388	0.854	-1.771	3.635	-0.885	1.836	76.793	17.335	14.070
7867	218	35	26	P	W	35.209	-3.388	-0.854	-1.771	3.635	0.885	1.836	76.793	17.335	14.070
7868	218	35	26	P	W	84.791	-3.388	0.854	-1.771	3.635	-0.885	1.836	76.793	17.335	14.070
7869	218	35	26	P	W	95.209	-3.388	-0.854	-1.771	3.635	0.885	1.836	76.793	17.335	14.070
7870	219	33	29	C	S	19.107	-1.485	2.216	4.816	1.530	-2.021	-4.393	80.400	14.708	16.799
7871	219	33	29	C	S	40.893	-1.485	-2.216	4.816	1.530	2.021	-4.393	80.400	14.708	16.799
7872	219	33	29	C	S	79.107	-1.485	2.216	4.816	1.530	-2.021	-4.393	80.400	14.708	16.799
7873	219	33	29	C	S	100.893	-1.485	-2.216	4.816	1.530	2.021	-4.393	80.400	14.708	16.799
7874	219	33	29	P	S	19.107	-1.485	2.216	4.816	1.530	-2.021	-4.393	80.400	14.708	16.799
7875	219	33	29	P	S	40.893	-1.485	-2.216	4.816	1.530	2.021	-4.393	80.400	14.708	16.799
7876	219	33	29	P	S	79.107	-1.485	2.216	4.816	1.530	-2.021	-4.393	80.400	14.708	16.799
7877	219	33	29	P	S	100.893	-1.485	-2.216	4.816	1.530	2.021	-4.393	80.400	14.708	16.799
7878	219	36	25	C	S	0.000	-4.158	-3.689	-4.158	4.535	4.023	4.535	81.248	16.522	14.477
7879	219	36	25	C	S	1.945	-4.723	1.419	-3.585	5.216	-1.528	3.862	69.915	15.242	16.516
7880	219	36	25	C	S	4.307	-4.685	3.540	-3.624	5.169	-3.817	3.908	81.267	14.491	16.506
7881	219	36	25	C	S	25.285	-4.781	-4.734	-3.525	5.287	5.093	3.793	73.178	16.641	14.842
7882	219	36	25	C	S	34.715	-4.781	4.734	-3.525	5.287	-5.093	3.793	73.179	16.641	14.842
7883	219	36	25	C	S	55.693	-4.685	-3.540	-3.624	5.169	3.816	3.908	81.267	14.491	16.506
7884	219	36	25	C	S	58.055	-4.723	-1.419	-3.585	5.216	1.528	3.862	69.916	15.242	16.516
7885	219	36	25	C	S	60.000	-4.158	-3.689	-4.158	4.535	4.023	4.535	81.247	16.522	14.477
7886	219	36	25	C	S	61.945	-4.723	1.419	-3.585	5.216	-1.528	3.862	69.916	15.242	16.516
7887	219	36	25	C	S	64.307	-4.685	3.540	-3.624	5.169	-3.817	3.908	81.268	14.491	16.506
7888	219	36	25	C	S	85.285	-4.781	-4.734	-3.525	5.287	5.093	3.793	73.179	16.641	14.842
7889	219	36	25	C	S	94.715	-4.781	4.734	-3.525	5.287	-5.093	3.793	73.178	16.641	14.842
7890	219	36	25	C	S	115.693	-4.685	-3.540	-3.624	5.169	3.816	3.908	81.268	14.491	16.506
7891	219	36	25	C	S	118.055	-4.723	-1.419	-3.585	5.216	1.528	3.862	69.916	15.242	16.516
7892	219	36	25	C	W	0.000	-4.158	-3.689	-4.158	4.535	4.023	4.535	81.248	16.522	14.477
7893	219	36	25	C	W	1.945	-4.723	1.419	-3.585	5.216	-1.528	3.862	69.915	15.242	16.516
7894	219	36	25	C	W	4.307	-4.685	3.540	-3.624	5.169	-3.817	3.908	81.267	14.491	16.506
7895	219	36	25	C	W	25.285	-4.781	-4.734	-3.525	5.287	5.093	3.793	73.178	16.641	14.842
7896	219	36	25	C	W	34.715	-4.781	4.734	-3.525	5.287	-5.093	3.793	73.179	16.641	14.842
7897	219	36	25	C	W	55.693	-4.685	-3.540	-3.624	5.169	3.816	3.908	81.267	14.491	16.506
7898	219	36	25	C	W	58.055	-4.723	-1.419	-3.585	5.216	1.528	3.862	69.916	15.242	16.516
7899	219	36	25	C	W	60.000	-4.158	-3.689	-4.158	4.535	4.023	4.535	81.247	16.522	14.477
7900	219	36	25	C	W	61.945	-4.723	1.419	-3.585	5.216	-1.528	3.862	69.916	15.242	16.516
7901	219	36	25	C	W	64.307	-4.685	3.540	-3.624	5.169	-3.817	3.908	81.268	14.491	16.506
7902	219	36	25	C	W	85.285	-4.781	-4.734	-3.525	5.287	5.093	3.793	73.179	16.641	14.842
7903	219	36	25	C	W	94.715	-4.781	4.734	-3.525	5.287	-5.093	3.793	73.178	16.641	14.842
7904	219	36	25	C	W	115.693	-4.685	-3.540	-3.624	5.169	3.816	3.908	81.268	14.491	16.506
7905	219	36	25	C	W	118.055	-4.723	-1.419	-3.585	5.216	1.528	3.862	69.916	15.242	16.516

BL number	Atoms	γ-PC unit cells	WS_2 unit cells	γ-PC origin atom	WS_2 origin atom	Twist-angle (°)	γ-PC strain 1 (%)	γ-PC strain 2 (%)	γ-PC strain 3 (%)	WS_2 strain 1 (%)	WS_2 strain 2 (%)	WS_2 strain 3 (%)	γ (°)	|**a**|(Å)	|**b**|(Å)
7906	219	36	25	P	S	0.000	-4.158	-3.689	-4.158	4.535	4.023	4.535	81.248	16.522	14.477
7907	219	36	25	P	S	1.945	-4.723	1.419	-3.585	5.216	-1.528	3.862	69.915	15.242	16.516
7908	219	36	25	P	S	4.307	-4.685	3.540	-3.624	5.169	-3.817	3.908	81.267	14.491	16.506
7909	219	36	25	P	S	25.285	-4.781	-4.734	-3.525	5.287	5.093	3.793	73.178	16.641	14.842
7910	219	36	25	P	S	34.715	-4.781	4.734	-3.525	5.287	-5.093	3.793	73.179	16.641	14.842
7911	219	36	25	P	S	55.693	-4.685	-3.540	-3.624	5.169	3.816	3.908	81.267	14.491	16.506
7912	219	36	25	P	S	58.055	-4.723	-1.419	-3.585	5.216	1.528	3.862	69.916	15.242	16.516
7913	219	36	25	P	S	60.000	-4.158	-3.689	-4.158	4.535	4.023	4.535	81.247	16.522	14.477
7914	219	36	25	P	S	61.945	-4.723	1.419	-3.585	5.216	-1.528	3.862	69.916	15.242	16.516
7915	219	36	25	P	S	64.307	-4.685	3.540	-3.624	5.169	-3.817	3.908	81.268	14.491	16.506
7916	219	36	25	P	S	85.285	-4.781	-4.734	-3.525	5.287	5.093	3.793	73.179	16.641	14.842
7917	219	36	25	P	S	94.715	-4.781	4.734	-3.525	5.287	-5.093	3.793	73.178	16.641	14.842
7918	219	36	25	P	S	115.693	-4.685	-3.540	-3.624	5.169	3.816	3.908	81.268	14.491	16.506
7919	219	36	25	P	S	118.055	-4.723	-1.419	-3.585	5.216	1.528	3.862	69.916	15.242	16.516
7920	219	36	25	P	W	0.000	-4.158	-3.689	-4.158	4.535	4.023	4.535	81.248	16.522	14.477
7921	219	36	25	P	W	1.945	-4.723	1.419	-3.585	5.216	-1.528	3.862	69.915	15.242	16.516
7922	219	36	25	P	W	4.307	-4.685	3.540	-3.624	5.169	-3.817	3.908	81.267	14.491	16.506
7923	219	36	25	P	W	25.285	-4.781	-4.734	-3.525	5.287	5.093	3.793	73.178	16.641	14.842
7924	219	36	25	P	W	34.715	-4.781	4.734	-3.525	5.287	-5.093	3.793	73.179	16.641	14.842
7925	219	36	25	P	W	55.693	-4.685	-3.540	-3.624	5.169	3.816	3.908	81.267	14.491	16.506
7926	219	36	25	P	W	58.055	-4.723	-1.419	-3.585	5.216	1.528	3.862	69.916	15.242	16.516
7927	219	36	25	P	W	60.000	-4.158	-3.689	-4.158	4.535	4.023	4.535	81.247	16.522	14.477
7928	219	36	25	P	W	61.945	-4.723	1.419	-3.585	5.216	-1.528	3.862	69.916	15.242	16.516
7929	219	36	25	P	W	64.307	-4.685	3.540	-3.624	5.169	-3.817	3.908	81.268	14.491	16.506
7930	219	36	25	P	W	85.285	-4.781	-4.734	-3.525	5.287	5.093	3.793	73.179	16.641	14.842
7931	219	36	25	P	W	94.715	-4.781	4.734	-3.525	5.287	-5.093	3.793	73.178	16.641	14.842
7932	219	36	25	P	W	115.693	-4.685	-3.540	-3.624	5.169	3.816	3.908	81.268	14.491	16.506
7933	219	36	25	P	W	118.055	-4.723	-1.419	-3.585	5.216	1.528	3.862	69.916	15.242	16.516
7934	220	34	28	C	S	0.000	5.011	-3.566	-4.697	-4.554	3.936	5.184	90.000	16.799	14.481
7935	220	34	28	C	S	4.307	-4.685	4.004	4.996	5.169	-3.640	-4.542	89.582	14.491	16.787
7936	220	34	28	C	S	8.213	-2.359	-1.658	2.312	2.476	1.585	-2.209	89.072	14.845	16.360
7937	220	34	28	C	S	8.513	2.390	-1.070	-2.430	-2.281	1.125	2.555	77.330	19.291	12.902
7938	220	34	28	C	S	10.158	2.281	1.737	-2.332	-2.182	-1.822	2.446	88.981	16.362	14.843
7939	220	34	28	C	S	10.893	-1.983	3.121	1.901	2.064	-3.007	-1.832	77.231	12.906	19.289
7940	220	34	28	C	S	17.480	-4.685	-0.767	4.996	5.169	0.697	-4.542	87.816	14.491	16.799
7941	220	34	28	C	S	28.055	2.281	-1.737	-2.332	-2.182	1.822	2.446	88.981	16.362	14.843
7942	220	34	28	C	S	30.000	-2.359	1.658	2.312	2.476	-1.585	-2.209	89.072	14.845	16.360
7943	220	34	28	C	S	31.945	2.281	1.737	-2.332	-2.182	-1.822	2.446	88.981	16.362	14.843
7944	220	34	28	C	S	42.520	-4.685	0.767	4.996	5.169	-0.697	-4.542	87.816	14.491	16.799
7945	220	34	28	C	S	49.107	-1.983	-3.121	1.901	2.064	3.006	-1.832	77.231	12.906	19.289
7946	220	34	28	C	S	49.842	2.281	-1.737	-2.332	-2.182	1.822	2.446	88.981	16.362	14.843
7947	220	34	28	C	S	51.487	2.390	1.070	-2.430	-2.281	-1.125	2.555	77.330	19.291	12.902
7948	220	34	28	C	S	51.787	-2.359	1.658	2.312	2.476	-1.585	-2.209	89.072	14.845	16.360
7949	220	34	28	C	S	55.693	-4.685	-4.004	4.996	5.169	3.640	-4.542	89.582	14.491	16.787
7950	220	34	28	C	S	60.000	5.011	3.566	-4.697	-4.554	-3.936	5.184	90.000	16.799	14.481
7951	220	34	28	C	S	64.307	-4.685	4.004	4.996	5.169	-3.640	-4.542	89.582	14.491	16.787
7952	220	34	28	C	S	68.213	-2.359	-1.658	2.312	2.476	1.585	-2.209	89.072	14.845	16.360
7953	220	34	28	C	S	68.513	2.390	-1.070	-2.430	-2.281	1.125	2.555	77.330	19.291	12.902
7954	220	34	28	C	S	70.158	2.281	1.737	-2.332	-2.182	-1.822	2.446	88.981	16.362	14.843
7955	220	34	28	C	S	70.893	-1.983	3.121	1.901	2.064	-3.007	-1.832	77.231	12.906	19.289
7956	220	34	28	C	S	77.480	-4.685	-0.767	4.996	5.169	0.697	-4.542	87.816	14.491	16.799

BL number	Atoms	γ-PC unit cells	WS_2 unit cells	γ-PC origin atom	WS_2 origin atom	Twist-angle (°)	γ-PC strain 1 (%)	γ-PC strain 2 (%)	γ-PC strain 3 (%)	WS_2 strain 1 (%)	WS_2 strain 2 (%)	WS_2 strain 3 (%)	γ (°)	|**a**|(Å)	|**b**|(Å)
7957	220	34	28	C	S	88.055	2.281	-1.737	-2.332	-2.182	1.822	2.446	88.981	16.362	14.843
7958	220	34	28	C	S	90.000	-2.359	-1.658	2.312	2.476	1.585	-2.209	89.072	14.845	16.360
7959	220	34	28	C	S	91.945	2.281	1.737	-2.332	-2.182	-1.822	2.446	88.981	16.362	14.843
7960	220	34	28	C	S	102.520	-4.685	0.767	4.996	5.169	-0.697	-4.542	87.816	14.491	16.799
7961	220	34	28	C	S	109.107	-1.983	-3.121	1.901	2.064	3.006	-1.832	77.231	12.906	19.289
7962	220	34	28	C	S	109.842	2.281	-1.737	-2.332	-2.182	1.822	2.446	88.981	16.362	14.843
7963	220	34	28	C	S	111.487	2.390	1.070	-2.430	-2.281	-1.125	2.555	77.330	19.291	12.902
7964	220	34	28	C	S	111.787	-2.359	1.658	2.312	2.476	-1.585	-2.209	89.072	14.845	16.360
7965	220	34	28	C	S	115.693	-4.685	-4.004	4.996	5.169	3.640	-4.542	89.582	14.491	16.787
7966	220	34	28	C	W	0.000	5.011	-3.566	-4.697	-4.554	3.936	5.184	90.000	16.799	14.481
7967	220	34	28	C	W	4.307	-4.685	4.004	4.996	5.169	-3.640	-4.542	89.582	14.491	16.787
7968	220	34	28	C	W	8.213	-2.359	-1.658	2.312	2.476	1.585	-2.209	89.072	14.845	16.360
7969	220	34	28	C	W	8.513	2.390	-1.070	-2.430	-2.281	1.125	2.555	77.330	19.291	12.902
7970	220	34	28	C	W	10.158	2.281	1.737	-2.332	-2.182	-1.822	2.446	88.981	16.362	14.843
7971	220	34	28	C	W	10.893	-1.983	3.121	1.901	2.064	-3.007	-1.832	77.231	12.906	19.289
7972	220	34	28	C	W	17.480	-4.685	-0.767	4.996	5.169	0.697	-4.542	87.816	14.491	16.799
7973	220	34	28	C	W	28.055	2.281	-1.737	-2.332	-2.182	1.822	2.446	88.981	16.362	14.843
7974	220	34	28	C	W	30.000	-2.359	1.658	2.312	2.476	-1.585	-2.209	89.072	14.845	16.360
7975	220	34	28	C	W	31.945	2.281	1.737	-2.332	-2.182	-1.822	2.446	88.981	16.362	14.843
7976	220	34	28	C	W	42.520	-4.685	0.767	4.996	5.169	-0.697	-4.542	87.816	14.491	16.799
7977	220	34	28	C	W	49.107	-1.983	-3.121	1.901	2.064	3.006	-1.832	77.231	12.906	19.289
7978	220	34	28	C	W	49.842	2.281	-1.737	-2.332	-2.182	1.822	2.446	88.981	16.362	14.843
7979	220	34	28	C	W	51.487	2.390	1.070	-2.430	-2.281	-1.125	2.555	77.330	19.291	12.902
7980	220	34	28	C	W	51.787	-2.359	1.658	2.312	2.476	-1.585	-2.209	89.072	14.845	16.360
7981	220	34	28	C	W	55.693	-4.685	-4.004	4.996	5.169	3.640	-4.542	89.582	14.491	16.787
7982	220	34	28	C	W	60.000	5.011	3.566	-4.697	-4.554	-3.936	5.184	90.000	16.799	14.481
7983	220	34	28	C	W	64.307	-4.685	4.004	4.996	5.169	-3.640	-4.542	89.582	14.491	16.787
7984	220	34	28	C	W	68.213	-2.359	-1.658	2.312	2.476	1.585	-2.209	89.072	14.845	16.360
7985	220	34	28	C	W	68.513	2.390	-1.070	-2.430	-2.281	1.125	2.555	77.330	19.291	12.902
7986	220	34	28	C	W	70.158	2.281	1.737	-2.332	-2.182	-1.822	2.446	88.981	16.362	14.843
7987	220	34	28	C	W	70.893	-1.983	3.121	1.901	2.064	-3.007	-1.832	77.231	12.906	19.289
7988	220	34	28	C	W	77.480	-4.685	-0.767	4.996	5.169	0.697	-4.542	87.816	14.491	16.799
7989	220	34	28	C	W	88.055	2.281	-1.737	-2.332	-2.182	1.822	2.446	88.981	16.362	14.843
7990	220	34	28	C	W	90.000	-2.359	-1.658	2.312	2.476	1.585	-2.209	89.072	14.845	16.360
7991	220	34	28	C	W	91.945	2.281	1.737	-2.332	-2.182	-1.822	2.446	88.981	16.362	14.843
7992	220	34	28	C	W	102.520	-4.685	0.767	4.996	5.169	-0.697	-4.542	87.816	14.491	16.799
7993	220	34	28	C	W	109.107	-1.983	-3.121	1.901	2.064	3.006	-1.832	77.231	12.906	19.289
7994	220	34	28	C	W	109.842	2.281	-1.737	-2.332	-2.182	1.822	2.446	88.981	16.362	14.843
7995	220	34	28	C	W	111.487	2.390	1.070	-2.430	-2.281	-1.125	2.555	77.330	19.291	12.902
7996	220	34	28	C	W	111.787	-2.359	1.658	2.312	2.476	-1.585	-2.209	89.072	14.845	16.360
7997	220	34	28	C	W	115.693	-4.685	-4.004	4.996	5.169	3.640	-4.542	89.582	14.491	16.787
7998	220	34	28	P	S	0.000	5.011	-3.566	-4.697	-4.554	3.936	5.184	90.000	16.799	14.481
7999	220	34	28	P	S	4.307	-4.685	4.004	4.996	5.169	-3.640	-4.542	89.582	14.491	16.787
8000	220	34	28	P	S	8.213	-2.359	-1.658	2.312	2.476	1.585	-2.209	89.072	14.845	16.360
8001	220	34	28	P	S	8.513	2.390	-1.070	-2.430	-2.281	1.125	2.555	77.330	19.291	12.902
8002	220	34	28	P	S	10.158	2.281	1.737	-2.332	-2.182	-1.822	2.446	88.981	16.362	14.843
8003	220	34	28	P	S	10.893	-1.983	3.121	1.901	2.064	-3.007	-1.832	77.231	12.906	19.289
8004	220	34	28	P	S	17.480	-4.685	-0.767	4.996	5.169	0.697	-4.542	87.816	14.491	16.799
8005	220	34	28	P	S	28.055	2.281	-1.737	-2.332	-2.182	1.822	2.446	88.981	16.362	14.843
8006	220	34	28	P	S	30.000	-2.359	1.658	2.312	2.476	-1.585	-2.209	89.072	14.845	16.360
8007	220	34	28	P	S	31.945	2.281	1.737	-2.332	-2.182	-1.822	2.446	88.981	16.362	14.843

BL number	Atoms	γ-PC unit cells	WS_2 unit cells	γ-PC origin atom	WS_2 origin atom	Twist-angle (°)	γ-PC strain 1 (%)	γ-PC strain 2 (%)	γ-PC strain 3 (%)	WS_2 strain 1 (%)	WS_2 strain 2 (%)	WS_2 strain 3 (%)	γ (°)	|**a**|(Å)	|**b**|(Å)
8008	220	34	28	P	S	49.107	-1.983	-3.121	1.901	2.064	3.006	-1.832	77.231	12.906	19.289
8009	220	34	28	P	S	49.842	2.281	-1.737	-2.332	-2.182	1.822	2.446	88.981	16.362	14.843
8010	220	34	28	P	S	51.487	2.390	1.070	-2.430	-2.281	-1.125	2.555	77.330	19.291	12.902
8011	220	34	28	P	S	51.787	-2.359	1.658	2.312	2.476	-1.585	-2.209	89.072	14.845	16.360
8012	220	34	28	P	S	55.693	-4.685	-4.004	4.996	5.169	3.640	-4.542	89.582	14.491	16.787
8013	220	34	28	P	S	60.000	5.011	3.566	-4.697	-4.554	-3.936	5.184	90.000	16.799	14.481
8014	220	34	28	P	S	64.307	-4.685	4.004	4.996	5.169	-3.640	-4.542	89.582	14.491	16.787
8015	220	34	28	P	S	68.213	-2.359	-1.658	2.312	2.476	1.585	-2.209	89.072	14.845	16.360
8016	220	34	28	P	S	68.513	2.390	-1.070	-2.430	-2.281	1.125	2.555	77.330	19.291	12.902
8017	220	34	28	P	S	70.158	2.281	1.737	-2.332	-2.182	-1.822	2.446	88.981	16.362	14.843
8018	220	34	28	P	S	70.893	-1.983	3.121	1.901	2.064	-3.007	-1.832	77.231	12.906	19.289
8019	220	34	28	P	S	77.480	-4.685	-0.767	4.996	5.169	0.697	-4.542	87.816	14.491	16.799
8020	220	34	28	P	S	88.055	2.281	-1.737	-2.332	-2.182	1.822	2.446	88.981	16.362	14.843
8021	220	34	28	P	S	90.000	-2.359	-1.658	2.312	2.476	1.585	-2.209	89.072	14.845	16.360
8022	220	34	28	P	S	91.945	2.281	1.737	-2.332	-2.182	-1.822	2.446	88.981	16.362	14.843
8023	220	34	28	P	S	102.520	-4.685	0.767	4.996	5.169	-0.697	-4.542	87.816	14.491	16.799
8024	220	34	28	P	S	109.107	-1.983	-3.121	1.901	2.064	3.006	-1.832	77.231	12.906	19.289
8025	220	34	28	P	S	109.842	2.281	-1.737	-2.332	-2.182	1.822	2.446	88.981	16.362	14.843
8026	220	34	28	P	S	111.487	2.390	1.070	-2.430	-2.281	-1.125	2.555	77.330	19.291	12.902
8027	220	34	28	P	S	111.787	-2.359	1.658	2.312	2.476	-1.585	-2.209	89.072	14.845	16.360
8028	220	34	28	P	S	115.693	-4.685	-4.004	4.996	5.169	3.640	-4.542	89.582	14.491	16.787
8029	220	34	28	P	W	0.000	5.011	-3.566	-4.697	-4.554	3.936	5.184	90.000	16.799	14.481
8030	220	34	28	P	W	4.307	-4.685	4.004	4.996	5.169	-3.640	-4.542	89.582	14.491	16.787
8031	220	34	28	P	W	8.213	-2.359	-1.658	2.312	2.476	1.585	-2.209	89.072	14.845	16.360
8032	220	34	28	P	W	8.513	2.390	-1.070	-2.430	-2.281	1.125	2.555	77.330	19.291	12.902
8033	220	34	28	P	W	10.158	2.281	1.737	-2.332	-2.182	-1.822	2.446	88.981	16.362	14.843
8034	220	34	28	P	W	10.893	-1.983	3.121	1.901	2.064	-3.007	-1.832	77.231	12.906	19.289
8035	220	34	28	P	W	17.480	-4.685	-0.767	4.996	5.169	0.697	-4.542	87.816	14.491	16.799
8036	220	34	28	P	W	28.055	2.281	-1.737	-2.332	-2.182	1.822	2.446	88.981	16.362	14.843
8037	220	34	28	P	W	30.000	-2.359	1.658	2.312	2.476	-1.585	-2.209	89.072	14.845	16.360
8038	220	34	28	P	W	31.945	2.281	1.737	-2.332	-2.182	-1.822	2.446	88.981	16.362	14.843
8039	220	34	28	P	W	42.520	-4.685	0.767	4.996	5.169	-0.697	-4.542	87.816	14.491	16.799
8040	220	34	28	P	W	49.107	-1.983	-3.121	1.901	2.064	3.006	-1.832	77.231	12.906	19.289
8041	220	34	28	P	W	49.842	2.281	-1.737	-2.332	-2.182	1.822	2.446	88.981	16.362	14.843
8042	220	34	28	P	W	51.487	2.390	1.070	-2.430	-2.281	-1.125	2.555	77.330	19.291	12.902
8043	220	34	28	P	W	51.787	-2.359	1.658	2.312	2.476	-1.585	-2.209	89.072	14.845	16.360
8044	220	34	28	P	W	55.693	-4.685	-4.004	4.996	5.169	3.640	-4.542	89.582	14.491	16.787
8045	220	34	28	P	W	60.000	5.011	3.566	-4.697	-4.554	-3.936	5.184	90.000	16.799	14.481
8046	220	34	28	P	W	64.307	-4.685	4.004	4.996	5.169	-3.640	-4.542	89.582	14.491	16.787
8047	220	34	28	P	W	68.213	-2.359	-1.658	2.312	2.476	1.585	-2.209	89.072	14.845	16.360
8048	220	34	28	P	W	68.513	2.390	-1.070	-2.430	-2.281	1.125	2.555	77.330	19.291	12.902
8049	220	34	28	P	W	70.158	2.281	1.737	-2.332	-2.182	-1.822	2.446	88.981	16.362	14.843
8050	220	34	28	P	W	70.893	-1.983	3.121	1.901	2.064	-3.007	-1.832	77.231	12.906	19.289
8051	220	34	28	P	W	77.480	-4.685	-0.767	4.996	5.169	0.697	-4.542	87.816	14.491	16.799
8052	220	34	28	P	W	88.055	2.281	-1.737	-2.332	-2.182	1.822	2.446	88.981	16.362	14.843
8053	220	34	28	P	W	90.000	-2.359	-1.658	2.312	2.476	1.585	-2.209	89.072	14.845	16.360
8054	220	34	28	P	W	91.945	2.281	1.737	-2.332	-2.182	-1.822	2.446	88.981	16.362	14.843
8055	220	34	28	P	W	102.520	-4.685	0.767	4.996	5.169	-0.697	-4.542	87.816	14.491	16.799
8056	220	34	28	P	W	109.107	-1.983	-3.121	1.901	2.064	3.006	-1.832	77.231	12.906	19.289
8057	220	34	28	P	W	109.842	2.281	-1.737	-2.332	-2.182	1.822	2.446	88.981	16.362	14.843
8058	220	34	28	P	W	111.487	2.390	1.070	-2.430	-2.281	-1.125	2.555	77.330	19.291	12.902

BL number	Atoms	γ-PC unit cells	WS_2 unit cells	γ-PC origin atom	WS_2 origin atom	Twist-angle (°)	γ-PC strain 1 (%)	γ-PC strain 2 (%)	γ-PC strain 3 (%)	WS_2 strain 1 (%)	WS_2 strain 2 (%)	WS_2 strain 3 (%)	γ (°)	|**a**|(Å)	|**b**|(Å)
8059	220	34	28	P	W	111.787	-2.359	1.658	2.312	2.476	-1.585	-2.209	89.072	14.845	16.360
8060	220	34	28	P	W	115.693	-4.685	-4.004	4.996	5.169	3.640	-4.542	89.582	14.491	16.787
8061	221	32	31	C	S	13.174	5.011	4.736	3.292	-4.554	-4.444	-3.088	78.089	13.151	19.283
8062	221	32	31	C	S	46.827	5.011	-4.736	3.292	-4.554	4.444	-3.088	78.089	13.151	19.283
8063	221	32	31	C	S	73.174	5.011	4.736	3.292	-4.554	-4.444	-3.088	78.089	13.151	19.283
8064	221	32	31	C	S	106.827	5.011	-4.736	3.292	-4.554	4.444	-3.088	78.089	13.151	19.283
8065	221	32	31	C	W	13.174	5.011	4.736	3.292	-4.554	-4.444	-3.088	78.089	13.151	19.283
8066	221	32	31	C	W	46.827	5.011	-4.736	3.292	-4.554	4.444	-3.088	78.089	13.151	19.283
8067	221	32	31	C	W	73.174	5.011	4.736	3.292	-4.554	-4.444	-3.088	78.089	13.151	19.283
8068	221	32	31	C	W	106.827	5.011	-4.736	3.292	-4.554	4.444	-3.088	78.089	13.151	19.283
8069	221	32	31	P	S	13.174	5.011	4.736	3.292	-4.554	-4.444	-3.088	78.089	13.151	19.283
8070	221	32	31	P	S	46.827	5.011	-4.736	3.292	-4.554	4.444	-3.088	78.089	13.151	19.283
8071	221	32	31	P	S	73.174	5.011	4.736	3.292	-4.554	-4.444	-3.088	78.089	13.151	19.283
8072	221	32	31	P	S	106.827	5.011	-4.736	3.292	-4.554	4.444	-3.088	78.089	13.151	19.283
8073	221	32	31	P	W	13.174	5.011	4.736	3.292	-4.554	-4.444	-3.088	78.089	13.151	19.283
8074	221	32	31	P	W	46.827	5.011	-4.736	3.292	-4.554	4.444	-3.088	78.089	13.151	19.283
8075	221	32	31	P	W	73.174	5.011	4.736	3.292	-4.554	-4.444	-3.088	78.089	13.151	19.283
8076	221	32	31	P	W	106.827	5.011	-4.736	3.292	-4.554	4.444	-3.088	78.089	13.151	19.283
8077	221	35	27	C	S	10.893	0.418	-3.549	-3.697	-0.415	3.833	3.993	77.530	14.426	17.179
8078	221	35	27	C	S	13.898	-4.228	1.580	1.003	4.619	-1.549	-0.983	77.648	17.184	14.419
8079	221	35	27	C	S	17.696	1.029	-3.378	-4.251	-1.008	3.692	4.646	70.941	14.053	18.223
8080	221	35	27	C	S	19.842	-4.723	0.225	1.560	5.216	-0.218	-1.513	78.504	15.242	16.211
8081	221	35	27	C	S	21.052	1.339	2.229	-4.528	-1.304	-2.451	4.979	78.455	16.211	15.242
8082	221	35	27	C	S	25.050	-0.955	1.107	-2.401	0.973	-1.163	2.522	75.295	17.772	14.071
8083	221	35	27	C	S	34.950	-0.955	-1.107	-2.401	0.973	1.163	2.522	75.295	17.772	14.071
8084	221	35	27	C	S	36.587	-2.043	1.701	-1.321	2.130	-1.747	1.357	75.272	14.072	17.771
8085	221	35	27	C	S	38.948	1.339	-2.229	-4.528	-1.304	2.451	4.979	78.455	16.211	15.242
8086	221	35	27	C	S	40.158	-4.723	-0.225	1.560	5.216	0.218	-1.513	78.504	15.242	16.211
8087	221	35	27	C	S	42.304	1.029	3.378	-4.251	-1.008	-3.692	4.646	70.941	18.223	14.053
8088	221	35	27	C	S	46.102	-4.228	-1.580	1.003	4.619	1.549	-0.983	77.648	17.184	14.419
8089	221	35	27	C	S	49.107	0.418	-4.733	-3.698	-0.415	5.111	3.993	82.303	14.426	16.926
8090	221	35	27	C	S	70.893	0.418	-3.549	-3.697	-0.415	3.833	3.993	77.530	14.426	17.179
8091	221	35	27	C	S	73.898	-4.228	1.580	1.003	4.619	-1.549	-0.983	77.648	17.184	14.419
8092	221	35	27	C	S	77.696	1.029	-3.378	-4.251	-1.008	3.692	4.646	70.941	14.053	18.223
8093	221	35	27	C	S	79.842	-4.723	0.225	1.560	5.216	-0.218	-1.513	78.504	15.242	16.211
8094	221	35	27	C	S	81.052	1.339	2.229	-4.528	-1.304	-2.451	4.979	78.455	16.211	15.242
8095	221	35	27	C	S	83.413	-2.043	-1.701	-1.321	2.130	1.747	1.357	75.272	14.072	17.771
8096	221	35	27	C	S	85.050	-0.955	1.107	-2.401	0.973	-1.163	2.522	75.295	17.772	14.071
8097	221	35	27	C	S	94.950	-0.955	-1.107	-2.401	0.973	1.163	2.522	75.295	17.772	14.071
8098	221	35	27	C	S	98.948	1.339	-2.229	-4.528	-1.304	2.451	4.979	78.455	16.211	15.242
8099	221	35	27	C	S	100.158	-4.723	-0.225	1.560	5.216	0.218	-1.513	78.504	15.242	16.211
8100	221	35	27	C	S	102.304	1.029	3.378	-4.251	-1.008	-3.692	4.646	70.941	18.223	14.053
8101	221	35	27	C	S	106.102	-4.228	-1.580	1.003	4.619	1.549	-0.983	77.648	17.184	14.419
8102	221	35	27	C	S	109.107	0.418	-4.733	-3.698	-0.415	5.111	3.993	82.303	14.426	16.926
8103	221	35	27	C	W	10.893	0.418	-3.549	-3.697	-0.415	3.833	3.993	77.530	14.426	17.179
8104	221	35	27	C	W	13.898	-4.228	1.580	1.003	4.619	-1.549	-0.983	77.648	17.184	14.419
8105	221	35	27	C	W	17.696	1.029	-3.378	-4.251	-1.008	3.692	4.646	70.941	14.053	18.223
8106	221	35	27	C	W	19.842	-4.723	0.225	1.560	5.216	-0.218	-1.513	78.504	15.242	16.211
8107	221	35	27	C	W	21.052	1.339	2.229	-4.528	-1.304	-2.451	4.979	78.455	16.211	15.242
8108	221	35	27	C	W	23.413	-2.043	-1.701	-1.321	2.130	1.747	1.357	75.272	14.072	17.771
8109	221	35	27	C	W	25.050	-0.955	1.107	-2.401	0.973	-1.163	2.522	75.295	17.772	14.071

BL number	Atoms	γ-PC unit cells	WS_2 unit cells	γ-PC origin atom	WS_2 origin atom	Twist-angle (°)	γ-PC strain 1 (%)	γ-PC strain 2 (%)	γ-PC strain 3 (%)	WS_2 strain 1 (%)	WS_2 strain 2 (%)	WS_2 strain 3 (%)	γ (°)	$\lvert\mathbf{a}\rvert$(Å)	$\lvert\mathbf{b}\rvert$(Å)
8110	221	35	27	C	W	34.950	-0.955	-1.107	-2.401	0.973	1.163	2.522	75.295	17.772	14.071
8111	221	35	27	C	W	38.948	1.339	-2.229	-4.528	-1.304	2.451	4.979	78.455	16.211	15.242
8112	221	35	27	C	W	40.158	-4.723	-0.225	1.560	5.216	0.218	-1.513	78.504	15.242	16.211
8113	221	35	27	C	W	42.304	1.029	3.378	-4.251	-1.008	-3.692	4.646	70.941	18.223	14.053
8114	221	35	27	C	W	46.102	-4.228	-1.580	1.003	4.619	1.549	-0.983	77.648	17.184	14.419
8115	221	35	27	C	W	49.107	0.418	-4.733	-3.698	-0.415	5.111	3.993	82.303	14.426	16.926
8116	221	35	27	C	W	70.893	0.418	-3.549	-3.697	-0.415	3.833	3.993	77.530	14.426	17.179
8117	221	35	27	C	W	73.898	-4.228	1.580	1.003	4.619	-1.549	-0.983	77.648	17.184	14.419
8118	221	35	27	C	W	77.696	1.029	-3.378	-4.251	-1.008	3.692	4.646	70.941	14.053	18.223
8119	221	35	27	C	W	79.842	-4.723	0.225	1.560	5.216	-0.218	-1.513	78.504	15.242	16.211
8120	221	35	27	C	W	81.052	1.339	2.229	-4.528	-1.304	-2.451	4.979	78.455	16.211	15.242
8121	221	35	27	C	W	85.050	-0.955	1.107	-2.401	0.973	-1.163	2.522	75.295	17.772	14.071
8122	221	35	27	C	W	94.950	-0.955	-1.107	-2.401	0.973	1.163	2.522	75.295	17.772	14.071
8123	221	35	27	C	W	96.587	-2.043	1.701	-1.321	2.130	-1.747	1.357	75.272	14.072	17.771
8124	221	35	27	C	W	98.948	1.339	-2.229	-4.528	-1.304	2.451	4.979	78.455	16.211	15.242
8125	221	35	27	C	W	100.158	-4.723	-0.225	1.560	5.216	0.218	-1.513	78.504	15.242	16.211
8126	221	35	27	C	W	102.304	1.029	3.378	-4.251	-1.008	-3.692	4.646	70.941	18.223	14.053
8127	221	35	27	C	W	106.102	-4.228	-1.580	1.003	4.619	1.549	-0.983	77.648	17.184	14.419
8128	221	35	27	C	W	109.107	0.418	-4.733	-3.698	-0.415	5.111	3.993	82.303	14.426	16.926
8129	221	35	27	P	S	23.413	-2.043	-1.701	-1.321	2.130	1.747	1.357	75.272	14.072	17.771
8130	221	35	27	P	S	25.050	-0.955	1.107	-2.401	0.973	-1.163	2.522	75.295	17.772	14.071
8131	221	35	27	P	S	34.950	-0.955	-1.107	-2.401	0.973	1.163	2.522	75.295	17.772	14.071
8132	221	35	27	P	S	38.948	1.339	-2.229	-4.528	-1.304	2.451	4.979	78.455	16.211	15.242
8133	221	35	27	P	S	40.158	-4.723	-0.225	1.560	5.216	0.218	-1.513	78.504	15.242	16.211
8134	221	35	27	P	S	49.107	0.418	-4.733	-3.698	-0.415	5.111	3.993	82.303	14.426	16.926
8135	221	35	27	P	S	70.893	0.418	4.733	-3.698	-0.415	-5.111	3.993	82.303	14.426	16.926
8136	221	35	27	P	S	79.842	-4.723	0.225	1.560	5.216	-0.218	-1.513	78.504	15.242	16.211
8137	221	35	27	P	S	81.052	1.339	2.229	-4.528	-1.304	-2.451	4.979	78.455	16.211	15.242
8138	221	35	27	P	S	85.050	-0.955	1.107	-2.401	0.973	-1.163	2.522	75.295	17.772	14.071
8139	221	35	27	P	S	94.950	-0.955	-1.107	-2.401	0.973	1.163	2.522	75.295	17.772	14.071
8140	221	35	27	P	S	96.587	-2.043	1.701	-1.321	2.130	-1.747	1.357	75.272	14.072	17.771
8141	221	35	27	P	W	10.893	0.418	4.733	-3.698	-0.415	-5.111	3.993	82.303	14.426	16.926
8142	221	35	27	P	W	19.842	-4.723	0.225	1.560	5.216	-0.218	-1.513	78.504	15.242	16.211
8143	221	35	27	P	W	21.052	1.339	2.229	-4.528	-1.304	-2.451	4.979	78.455	16.211	15.242
8144	221	35	27	P	W	25.050	-0.955	1.107	-2.401	0.973	-1.163	2.522	75.295	17.772	14.071
8145	221	35	27	P	W	34.950	-0.955	-1.107	-2.401	0.973	1.163	2.522	75.295	17.772	14.071
8146	221	35	27	P	W	36.587	-2.043	1.701	-1.321	2.130	-1.747	1.357	75.272	14.072	17.771
8147	221	35	27	P	W	83.413	-2.043	-1.701	-1.321	2.130	1.747	1.357	75.272	14.072	17.771
8148	221	35	27	P	W	85.050	-0.955	1.107	-2.401	0.973	-1.163	2.522	75.295	17.772	14.071
8149	221	35	27	P	W	94.950	-0.955	-1.107	-2.401	0.973	1.163	2.522	75.295	17.772	14.071
8150	221	35	27	P	W	98.948	1.339	-2.229	-4.528	-1.304	2.451	4.979	78.455	16.211	15.242
8151	221	35	27	P	W	100.158	-4.723	-0.225	1.560	5.216	0.218	-1.513	78.504	15.242	16.211
8152	221	35	27	P	W	109.107	0.418	-4.733	-3.698	-0.415	5.111	3.993	82.303	14.426	16.926
8153	222	33	30	C	S	7.589	0.334	-5.412	4.656	-0.332	4.951	-4.260	86.082	18.904	13.136
8154	222	33	30	C	S	12.520	2.326	-3.251	2.576	-2.223	3.092	-2.450	85.196	13.473	18.445
8155	222	33	30	C	S	13.174	5.011	4.593	0.010	-4.554	-4.592	-0.010	86.321	13.151	18.879
8156	222	33	30	C	S	16.102	2.853	3.149	2.052	-2.699	-3.025	-1.971	85.212	18.455	13.465
8157	222	33	30	C	S	25.285	4.262	-3.496	0.700	-3.928	3.448	-0.690	66.883	15.346	17.551
8158	222	33	30	C	S	25.767	0.353	-2.747	4.636	-0.351	2.514	-4.242	66.888	17.539	15.358
8159	222	33	30	C	S	28.055	2.281	-1.853	2.621	-2.182	1.761	-2.490	80.074	16.362	15.364
8160	222	33	30	C	S	30.000	2.934	-4.862	1.972	-2.772	4.678	-1.897	66.773	15.368	17.535

BL number	Atoms	γ-PC unit cells	WS_2 unit cells	γ-PC origin atom	WS_2 origin atom	Twist-angle (°)	γ-PC strain 1 (%)	γ-PC strain 2 (%)	γ-PC strain 3 (%)	WS_2 strain 1 (%)	WS_2 strain 2 (%)	WS_2 strain 3 (%)	γ (°)	**a**(Å)	**b**(Å)
8161	222	33	30	C	S	31.945	2.281	1.853	2.621	-2.182	-1.761	-2.490	80.074	16.362	15.364
8162	222	33	30	C	S	34.233	0.353	2.747	4.636	-0.351	-2.514	-4.242	66.888	17.539	15.358
8163	222	33	30	C	S	34.715	4.262	3.496	0.700	-3.928	-3.448	-0.690	66.883	17.551	15.346
8164	222	33	30	C	S	43.898	2.853	-3.149	2.052	-2.699	3.025	-1.971	85.212	18.455	13.465
8165	222	33	30	C	S	46.827	5.011	-4.593	0.010	-4.554	4.592	-0.010	86.321	13.151	18.879
8166	222	33	30	C	S	47.480	2.326	3.251	2.576	-2.223	-3.092	-2.450	85.196	13.473	18.445
8167	222	33	30	C	S	52.411	0.334	5.412	4.656	-0.332	-4.951	-4.260	86.082	18.904	13.136
8168	222	33	30	C	S	67.589	0.334	-5.412	4.656	-0.332	4.951	-4.260	86.082	18.904	13.136
8169	222	33	30	C	S	72.520	2.326	-3.251	2.576	-2.223	3.092	-2.450	85.196	13.473	18.445
8170	222	33	30	C	S	73.174	5.011	4.593	0.010	-4.554	-4.592	-0.010	86.321	13.151	18.879
8171	222	33	30	C	S	76.102	2.853	3.149	2.052	-2.699	-3.025	-1.971	85.212	18.455	13.465
8172	222	33	30	C	S	85.285	4.262	-3.496	0.700	-3.928	3.448	-0.690	66.883	15.346	17.551
8173	222	33	30	C	S	85.767	0.353	-2.747	4.636	-0.351	2.514	-4.242	66.888	17.539	15.358
8174	222	33	30	C	S	88.055	2.281	-1.853	2.621	-2.182	1.761	-2.490	80.074	16.362	15.364
8175	222	33	30	C	S	90.000	2.934	4.863	1.972	-2.772	-4.678	-1.897	66.773	15.368	17.535
8176	222	33	30	C	S	91.945	2.281	1.853	2.621	-2.182	-1.761	-2.490	80.074	16.362	15.364
8177	222	33	30	C	S	94.233	0.353	2.747	4.636	-0.351	-2.514	-4.242	66.888	17.539	15.358
8178	222	33	30	C	S	94.715	4.262	3.496	0.700	-3.928	-3.448	-0.690	66.883	17.551	15.346
8179	222	33	30	C	S	103.898	2.853	-3.149	2.052	-2.699	3.025	-1.971	85.212	18.455	13.465
8180	222	33	30	C	S	106.827	5.011	-4.593	0.010	-4.554	4.592	-0.010	86.321	13.151	18.879
8181	222	33	30	C	S	107.480	2.326	3.251	2.576	-2.223	-3.092	-2.450	85.196	13.473	18.445
8182	222	33	30	C	S	112.411	0.334	5.412	4.656	-0.332	-4.951	-4.260	86.082	18.904	13.136
8183	222	33	30	C	W	7.589	0.334	-5.412	4.656	-0.332	4.951	-4.260	86.082	18.904	13.136
8184	222	33	30	C	W	12.520	2.326	-3.251	2.576	-2.223	3.092	-2.450	85.196	13.473	18.445
8185	222	33	30	C	W	13.174	5.011	4.593	0.010	-4.554	-4.592	-0.010	86.321	13.151	18.879
8186	222	33	30	C	W	16.102	2.853	3.149	2.052	-2.699	-3.025	-1.971	85.212	18.455	13.465
8187	222	33	30	C	W	25.285	4.262	-3.496	0.700	-3.928	3.448	-0.690	66.883	15.346	17.551
8188	222	33	30	C	W	25.767	0.353	-2.747	4.636	-0.351	2.514	-4.242	66.888	17.539	15.358
8189	222	33	30	C	W	28.055	2.281	-1.853	2.621	-2.182	1.761	-2.490	80.074	16.362	15.364
8190	222	33	30	C	W	30.000	2.934	-4.862	1.972	-2.772	4.678	-1.897	66.773	15.368	17.535
8191	222	33	30	C	W	31.945	2.281	1.853	2.621	-2.182	-1.761	-2.490	80.074	16.362	15.364
8192	222	33	30	C	W	34.233	0.353	2.747	4.636	-0.351	-2.514	-4.242	66.888	17.539	15.358
8193	222	33	30	C	W	34.715	4.262	3.496	0.700	-3.928	-3.448	-0.690	66.883	17.551	15.346
8194	222	33	30	C	W	43.898	2.853	-3.149	2.052	-2.699	3.025	-1.971	85.212	18.455	13.465
8195	222	33	30	C	W	46.827	5.011	-4.593	0.010	-4.554	4.592	-0.010	86.321	13.151	18.879
8196	222	33	30	C	W	47.480	2.326	3.251	2.576	-2.223	-3.092	-2.450	85.196	13.473	18.445
8197	222	33	30	C	W	52.411	0.334	5.412	4.656	-0.332	-4.951	-4.260	86.082	18.904	13.136
8198	222	33	30	C	W	67.589	0.334	-5.412	4.656	-0.332	4.951	-4.260	86.082	18.904	13.136
8199	222	33	30	C	W	72.520	2.326	-3.251	2.576	-2.223	3.092	-2.450	85.196	13.473	18.445
8200	222	33	30	C	W	73.174	5.011	4.593	0.010	-4.554	-4.592	-0.010	86.321	13.151	18.879
8201	222	33	30	C	W	76.102	2.853	3.149	2.052	-2.699	-3.025	-1.971	85.212	18.455	13.465
8202	222	33	30	C	W	85.285	4.262	-3.496	0.700	-3.928	3.448	-0.690	66.883	15.346	17.551
8203	222	33	30	C	W	85.767	0.353	-2.747	4.636	-0.351	2.514	-4.242	66.888	17.539	15.358
8204	222	33	30	C	W	88.055	2.281	-1.853	2.621	-2.182	1.761	-2.490	80.074	16.362	15.364
8205	222	33	30	C	W	90.000	2.934	4.863	1.972	-2.772	-4.678	-1.897	66.773	15.368	17.535
8206	222	33	30	C	W	91.945	2.281	1.853	2.621	-2.182	-1.761	-2.490	80.074	16.362	15.364
8207	222	33	30	C	W	94.233	0.353	2.747	4.636	-0.351	-2.514	-4.242	66.888	17.539	15.358
8208	222	33	30	C	W	94.715	4.262	3.496	0.700	-3.928	-3.448	-0.690	66.883	17.551	15.346
8209	222	33	30	C	W	103.898	2.853	-3.149	2.052	-2.699	3.025	-1.971	85.212	18.455	13.465
8210	222	33	30	C	W	106.827	5.011	-4.593	0.010	-4.554	4.592	-0.010	86.321	13.151	18.879
8211	222	33	30	C	W	107.480	2.326	3.251	2.576	-2.223	-3.092	-2.450	85.196	13.473	18.445

BL number	Atoms	γ-PC unit cells	WS_2 unit cells	γ-PC origin atom	WS_2 origin atom	Twist-angle (°)	γ-PC strain 1 (%)	γ-PC strain 2 (%)	γ-PC strain 3 (%)	WS_2 strain 1 (%)	WS_2 strain 2 (%)	WS_2 strain 3 (%)	γ (°)	$\lvert\mathbf{a}\rvert$(Å)	$\lvert\mathbf{b}\rvert$(Å)
8212	222	33	30	C	W	112.411	0.334	5.412	4.656	-0.332	-4.951	-4.260	86.082	18.904	13.136
8213	222	33	30	P	S	7.589	0.334	-5.412	4.656	-0.332	4.951	-4.260	86.082	18.904	13.136
8214	222	33	30	P	S	13.174	5.011	4.593	0.010	-4.554	-4.592	-0.010	86.321	13.151	18.879
8215	222	33	30	P	S	25.285	4.262	-3.496	0.700	-3.928	3.448	-0.690	66.883	15.346	17.551
8216	222	33	30	P	S	25.767	0.353	-2.747	4.636	-0.351	2.514	-4.242	66.888	17.539	15.358
8217	222	33	30	P	S	28.055	2.281	-1.853	2.621	-2.182	1.761	-2.490	80.074	16.362	15.364
8218	222	33	30	P	S	30.000	2.934	-4.862	1.972	-2.772	4.678	-1.897	66.773	15.368	17.535
8219	222	33	30	P	S	31.945	2.281	1.853	2.621	-2.182	-1.761	-2.490	80.074	16.362	15.364
8220	222	33	30	P	S	34.233	0.353	2.747	4.636	-0.351	-2.514	-4.242	66.888	17.539	15.358
8221	222	33	30	P	S	34.715	4.262	3.496	0.700	-3.928	-3.448	-0.690	66.883	17.551	15.346
8222	222	33	30	P	S	46.827	5.011	-4.593	0.010	-4.554	4.592	-0.010	86.321	13.151	18.879
8223	222	33	30	P	S	52.411	0.334	5.412	4.656	-0.332	-4.951	-4.260	86.082	18.904	13.136
8224	222	33	30	P	S	67.589	0.334	-5.412	4.656	-0.332	4.951	-4.260	86.082	18.904	13.136
8225	222	33	30	P	S	73.174	5.011	4.593	0.010	-4.554	-4.592	-0.010	86.321	13.151	18.879
8226	222	33	30	P	S	85.285	4.262	-3.496	0.700	-3.928	3.448	-0.690	66.883	15.346	17.551
8227	222	33	30	P	S	85.767	0.353	-2.747	4.636	-0.351	2.514	-4.242	66.888	17.539	15.358
8228	222	33	30	P	S	88.055	2.281	-1.853	2.621	-2.182	1.761	-2.490	80.074	16.362	15.364
8229	222	33	30	P	S	90.000	2.934	4.863	1.972	-2.772	-4.678	-1.897	66.773	15.368	17.535
8230	222	33	30	P	S	91.945	2.281	1.853	2.621	-2.182	-1.761	-2.490	80.074	16.362	15.364
8231	222	33	30	P	S	94.233	0.353	2.747	4.636	-0.351	-2.514	-4.242	66.888	17.539	15.358
8232	222	33	30	P	S	94.715	4.262	3.496	0.700	-3.928	-3.448	-0.690	66.883	17.551	15.346
8233	222	33	30	P	S	106.827	5.011	-4.593	0.010	-4.554	4.592	-0.010	86.321	13.151	18.879
8234	222	33	30	P	S	112.411	0.334	5.412	4.656	-0.332	-4.951	-4.260	86.082	18.904	13.136
8235	222	33	30	P	W	7.589	0.334	-5.412	4.656	-0.332	4.951	-4.260	86.082	18.904	13.136
8236	222	33	30	P	W	13.174	5.011	4.593	0.010	-4.554	-4.592	-0.010	86.321	13.151	18.879
8237	222	33	30	P	W	25.285	4.262	-3.496	0.700	-3.928	3.448	-0.690	66.883	15.346	17.551
8238	222	33	30	P	W	25.767	0.353	-2.747	4.636	-0.351	2.514	-4.242	66.888	17.539	15.358
8239	222	33	30	P	W	28.055	2.281	-1.853	2.621	-2.182	1.761	-2.490	80.074	16.362	15.364
8240	222	33	30	P	W	30.000	2.934	-4.862	1.972	-2.772	4.678	-1.897	66.773	15.368	17.535
8241	222	33	30	P	W	31.945	2.281	1.853	2.621	-2.182	-1.761	-2.490	80.074	16.362	15.364
8242	222	33	30	P	W	34.233	0.353	2.747	4.636	-0.351	-2.514	-4.242	66.888	17.539	15.358
8243	222	33	30	P	W	34.715	4.262	3.496	0.700	-3.928	-3.448	-0.690	66.883	17.551	15.346
8244	222	33	30	P	W	46.827	5.011	-4.593	0.010	-4.554	4.592	-0.010	86.321	13.151	18.879
8245	222	33	30	P	W	52.411	0.334	5.412	4.656	-0.332	-4.951	-4.260	86.082	18.904	13.136
8246	222	33	30	P	W	67.589	0.334	-5.412	4.656	-0.332	4.951	-4.260	86.082	18.904	13.136
8247	222	33	30	P	W	73.174	5.011	4.593	0.010	-4.554	-4.592	-0.010	86.321	13.151	18.879
8248	222	33	30	P	W	85.285	4.262	-3.496	0.700	-3.928	3.448	-0.690	66.883	15.346	17.551
8249	222	33	30	P	W	85.767	0.353	-2.747	4.636	-0.351	2.514	-4.242	66.888	17.539	15.358
8250	222	33	30	P	W	88.055	2.281	-1.853	2.621	-2.182	1.761	-2.490	80.074	16.362	15.364
8251	222	33	30	P	W	90.000	2.934	4.863	1.972	-2.772	-4.678	-1.897	66.773	15.368	17.535
8252	222	33	30	P	W	91.945	2.281	1.853	2.621	-2.182	-1.761	-2.490	80.074	16.362	15.364
8253	222	33	30	P	W	94.233	0.353	2.747	4.636	-0.351	-2.514	-4.242	66.888	17.539	15.358
8254	222	33	30	P	W	94.715	4.262	3.496	0.700	-3.928	-3.448	-0.690	66.883	17.551	15.346
8255	222	33	30	P	W	106.827	5.011	-4.593	0.010	-4.554	4.592	-0.010	86.321	13.151	18.879
8256	222	33	30	P	W	112.411	0.334	5.412	4.656	-0.332	-4.951	-4.260	86.082	18.904	13.136
8257	222	36	26	C	S	3.004	-3.388	-3.154	-3.111	3.635	3.363	3.317	75.679	17.335	14.343
8258	222	36	26	C	S	4.715	-1.702	-0.256	-4.748	1.762	0.282	5.246	75.622	14.353	17.333
8259	222	36	26	C	S	6.587	-3.853	2.922	-2.638	4.175	-3.084	2.786	75.691	14.354	17.322
8260	222	36	26	C	S	19.107	-1.485	-4.063	-4.950	1.530	4.509	5.494	71.122	14.708	17.317
8261	222	36	26	C	S	40.893	-1.485	4.063	-4.950	1.530	-4.509	5.494	71.122	14.708	17.317
8262	222	36	26	C	S	53.413	-3.853	-2.922	-2.638	4.175	3.084	2.786	75.691	14.354	17.322

BL number	Atoms	γ-PC unit cells	WS_2 unit cells	γ-PC origin atom	WS_2 origin atom	Twist-angle (°)	γ-PC strain 1 (%)	γ-PC strain 2 (%)	γ-PC strain 3 (%)	WS_2 strain 1 (%)	WS_2 strain 2 (%)	WS_2 strain 3 (%)	γ (°)	|**a**|(Å)	|**b**|(Å)
8263	222	36	26	C	S	53.822	-2.145	-2.186	-4.329	2.241	2.393	4.739	76.525	17.102	14.488
8264	222	36	26	C	S	55.285	-1.702	0.256	-4.748	1.762	-0.282	5.246	75.622	17.333	14.353
8265	222	36	26	C	S	55.693	-4.685	0.966	-1.769	5.169	-1.001	1.834	76.550	14.491	17.098
8266	222	36	26	C	S	56.996	-3.388	3.154	-3.111	3.635	-3.363	3.317	75.679	17.335	14.343
8267	222	36	26	C	S	63.005	-3.388	-3.154	-3.111	3.635	3.363	3.317	75.679	17.335	14.343
8268	222	36	26	C	S	64.307	-4.685	-0.965	-1.769	5.169	1.001	1.834	76.550	14.491	17.098
8269	222	36	26	C	S	64.715	-1.702	-0.256	-4.748	1.762	0.282	5.246	75.622	14.353	17.333
8270	222	36	26	C	S	66.178	-2.145	2.186	-4.329	2.241	-2.393	4.739	76.525	17.102	14.488
8271	222	36	26	C	S	66.587	-3.853	2.922	-2.638	4.175	-3.084	2.786	75.691	14.354	17.322
8272	222	36	26	C	S	79.107	-1.485	-4.063	-4.950	1.530	4.509	5.494	71.122	14.708	17.317
8273	222	36	26	C	S	100.893	-1.485	4.063	-4.950	1.530	-4.509	5.494	71.122	14.708	17.317
8274	222	36	26	C	S	113.413	-3.853	-2.922	-2.638	4.175	3.084	2.786	75.691	14.354	17.322
8275	222	36	26	C	S	113.822	-2.145	-2.186	-4.329	2.241	2.393	4.739	76.525	17.102	14.488
8276	222	36	26	C	S	115.285	-1.702	0.256	-4.748	1.762	-0.282	5.246	75.622	17.333	14.353
8277	222	36	26	C	S	116.996	-3.388	3.154	-3.111	3.635	-3.363	3.317	75.679	17.335	14.343
8278	222	36	26	C	W	3.004	-3.388	-3.154	-3.111	3.635	3.363	3.317	75.679	17.335	14.343
8279	222	36	26	C	W	4.307	-4.685	-0.965	-1.769	5.169	1.001	1.834	76.550	14.491	17.098
8280	222	36	26	C	W	4.715	-1.702	-0.256	-4.748	1.762	0.282	5.246	75.622	14.353	17.333
8281	222	36	26	C	W	6.178	-2.145	2.186	-4.329	2.241	-2.393	4.739	76.525	17.102	14.488
8282	222	36	26	C	W	6.587	-3.853	2.922	-2.638	4.175	-3.084	2.786	75.691	14.354	17.322
8283	222	36	26	C	W	53.413	-3.853	-2.922	-2.638	4.175	3.084	2.786	75.691	14.354	17.322
8284	222	36	26	C	W	55.285	-1.702	0.256	-4.748	1.762	-0.282	5.246	75.622	17.333	14.353
8285	222	36	26	C	W	56.996	-3.388	3.154	-3.111	3.635	-3.363	3.317	75.679	17.335	14.343
8286	222	36	26	C	W	63.005	-3.388	-3.154	-3.111	3.635	3.363	3.317	75.679	17.335	14.343
8287	222	36	26	C	W	64.715	-1.702	-0.256	-4.748	1.762	0.282	5.246	75.622	14.353	17.333
8288	222	36	26	C	W	66.587	-3.853	2.922	-2.638	4.175	-3.084	2.786	75.691	14.354	17.322
8289	222	36	26	C	W	113.413	-3.853	-2.922	-2.638	4.175	3.084	2.786	75.691	14.354	17.322
8290	222	36	26	C	W	115.285	-1.702	0.256	-4.748	1.762	-0.282	5.246	75.622	17.333	14.353
8291	222	36	26	C	W	115.693	-4.685	0.966	-1.769	5.169	-1.001	1.834	76.550	14.491	17.098
8292	222	36	26	C	W	116.996	-3.388	3.154	-3.111	3.635	-3.363	3.317	75.679	17.335	14.343
8293	222	36	26	P	S	6.178	-2.145	2.186	-4.329	2.241	-2.393	4.739	76.525	17.102	14.488
8294	222	36	26	P	S	19.107	-1.485	-4.063	-4.950	1.530	4.509	5.494	71.122	14.708	17.317
8295	222	36	26	P	S	40.893	-1.485	4.063	-4.950	1.530	-4.509	5.494	71.122	14.708	17.317
8296	222	36	26	P	S	53.413	-3.853	-2.922	-2.638	4.175	3.084	2.786	75.691	14.354	17.322
8297	222	36	26	P	S	53.822	-2.145	-2.186	-4.329	2.241	2.393	4.739	76.525	17.102	14.488
8298	222	36	26	P	S	55.285	-1.702	0.256	-4.748	1.762	-0.282	5.246	75.622	17.333	14.353
8299	222	36	26	P	S	56.996	-3.388	3.154	-3.111	3.635	-3.363	3.317	75.679	17.335	14.343
8300	222	36	26	P	S	63.005	-3.388	-3.154	-3.111	3.635	3.363	3.317	75.679	17.335	14.343
8301	222	36	26	P	S	64.307	-4.685	-0.965	-1.769	5.169	1.001	1.834	76.550	14.491	17.098
8302	222	36	26	P	S	64.715	-1.702	-0.256	-4.748	1.762	0.282	5.246	75.622	14.353	17.333
8303	222	36	26	P	S	66.587	-3.853	2.922	-2.638	4.175	-3.084	2.786	75.691	14.354	17.322
8304	222	36	26	P	S	79.107	-1.485	-4.063	-4.950	1.530	4.509	5.494	71.122	14.708	17.317
8305	222	36	26	P	S	100.893	-1.485	4.063	-4.950	1.530	-4.509	5.494	71.122	14.708	17.317
8306	222	36	26	P	S	113.822	-2.145	-2.186	-4.329	2.241	2.393	4.739	76.525	17.102	14.488
8307	222	36	26	P	S	115.693	-4.685	0.966	-1.769	5.169	-1.001	1.834	76.550	14.491	17.098
8308	222	36	26	P	S	116.996	-3.388	3.154	-3.111	3.635	-3.363	3.317	75.679	17.335	14.343
8309	222	36	26	P	W	3.004	-3.388	-3.154	-3.111	3.635	3.363	3.317	75.679	17.335	14.343
8310	222	36	26	P	W	4.307	-4.685	-0.965	-1.769	5.169	1.001	1.834	76.550	14.491	17.098
8311	222	36	26	P	W	4.715	-1.702	-0.256	-4.748	1.762	0.282	5.246	75.622	14.353	17.333
8312	222	36	26	P	W	6.587	-3.853	2.922	-2.638	4.175	-3.084	2.786	75.691	14.354	17.322
8313	222	36	26	P	W	55.693	-4.685	0.966	-1.769	5.169	-1.001	1.834	76.550	14.491	17.098

BL number	Atoms	γ-PC unit cells	WS_2 unit cells	γ-PC origin atom	WS_2 origin atom	Twist-angle (°)	γ-PC strain 1 (%)	γ-PC strain 2 (%)	γ-PC strain 3 (%)	WS_2 strain 1 (%)	WS_2 strain 2 (%)	WS_2 strain 3 (%)	γ (°)	$\lvert\mathbf{a}\rvert$(Å)	$\lvert\mathbf{b}\rvert$(Å)
8314	222	36	26	P	W	56.996	-3.388	3.154	-3.111	3.635	-3.363	3.317	75.679	17.335	14.343
8315	222	36	26	P	W	63.005	-3.388	-3.154	-3.111	3.635	3.363	3.317	75.679	17.335	14.343
8316	222	36	26	P	W	66.178	-2.145	2.186	-4.329	2.241	-2.393	4.739	76.525	17.102	14.488
8317	222	36	26	P	W	113.413	-3.853	-2.922	-2.638	4.175	3.084	2.786	75.691	14.354	17.322
8318	222	36	26	P	W	115.285	-1.702	0.256	-4.748	1.762	-0.282	5.246	75.622	17.333	14.353
8319	222	36	26	P	W	116.996	-3.388	3.154	-3.111	3.635	-3.363	3.317	75.679	17.335	14.343
8320	223	34	29	C	S	0.000	5.011	-2.675	-3.079	-4.554	2.850	3.281	79.066	13.826	18.224
8321	223	34	29	C	S	1.359	-3.292	-0.397	5.261	3.523	0.360	-4.760	79.187	18.221	13.826
8322	223	34	29	C	S	58.641	-3.292	0.397	5.261	3.523	-0.360	-4.760	79.187	18.221	13.826
8323	223	34	29	C	S	60.000	5.011	2.675	-3.079	-4.555	-2.850	3.281	79.066	13.826	18.224
8324	223	34	29	C	S	61.359	-3.292	-0.397	5.261	3.523	0.360	-4.760	79.187	18.221	13.826
8325	223	34	29	C	S	118.641	-3.292	0.397	5.261	3.523	-0.360	-4.760	79.187	18.221	13.826
8326	223	34	29	C	W	0.000	5.011	-2.675	-3.079	-4.554	2.850	3.281	79.066	13.826	18.224
8327	223	34	29	C	W	1.359	-3.292	-0.397	5.261	3.523	0.360	-4.760	79.187	18.221	13.826
8328	223	34	29	C	W	58.641	-3.292	0.397	5.261	3.523	-0.360	-4.760	79.187	18.221	13.826
8329	223	34	29	C	W	60.000	5.011	2.675	-3.079	-4.555	-2.850	3.281	79.066	13.826	18.224
8330	223	34	29	C	W	61.359	-3.292	-0.397	5.261	3.523	0.360	-4.760	79.187	18.221	13.826
8331	223	34	29	C	W	118.641	-3.292	0.397	5.261	3.523	-0.360	-4.760	79.187	18.221	13.826
8332	223	34	29	P	S	0.000	5.011	-2.675	-3.079	-4.554	2.850	3.281	79.066	13.826	18.224
8333	223	34	29	P	S	1.359	-3.292	-0.397	5.261	3.523	0.360	-4.760	79.187	18.221	13.826
8334	223	34	29	P	S	118.641	-3.292	0.397	5.261	3.523	-0.360	-4.760	79.187	18.221	13.826
8335	223	34	29	P	W	58.641	-3.292	0.397	5.261	3.523	-0.360	-4.760	79.187	18.221	13.826
8336	223	34	29	P	W	60.000	5.011	2.675	-3.079	-4.555	-2.850	3.281	79.066	13.826	18.224
8337	223	34	29	P	W	61.359	-3.292	-0.397	5.261	3.523	0.360	-4.760	79.187	18.221	13.826
8338	223	37	25	C	S	25.285	-4.781	0.000	-4.781	5.287	0.000	5.287	60.000	16.641	16.641
8339	223	37	25	C	S	34.715	-4.781	0.000	-4.781	5.287	0.000	5.287	60.000	16.641	16.641
8340	223	37	25	C	S	85.285	-4.781	0.000	-4.781	5.287	0.000	5.287	60.000	16.641	16.641
8341	223	37	25	C	S	94.715	-4.781	0.000	-4.781	5.287	0.000	5.287	60.000	16.641	16.641
8342	223	37	25	C	W	25.285	-4.781	0.000	-4.781	5.287	0.000	5.287	60.000	16.641	16.641
8343	223	37	25	C	W	34.715	-4.781	0.000	-4.781	5.287	0.000	5.287	60.000	16.641	16.641
8344	223	37	25	C	W	85.285	-4.781	0.000	-4.781	5.287	0.000	5.287	60.000	16.641	16.641
8345	223	37	25	C	W	94.715	-4.781	0.000	-4.781	5.287	0.000	5.287	60.000	16.641	16.641
8346	223	37	25	P	S	34.715	-4.781	0.000	-4.781	5.287	0.000	5.287	60.000	16.641	16.641
8347	223	37	25	P	S	85.285	-4.781	0.000	-4.781	5.287	0.000	5.287	60.000	16.641	16.641
8348	223	37	25	P	W	25.285	-4.781	0.000	-4.781	5.287	0.000	5.287	60.000	16.641	16.641
8349	223	37	25	P	W	94.715	-4.781	0.000	-4.781	5.287	0.000	5.287	60.000	16.641	16.641
8350	223	37	25	C	S	19.842	-4.723	3.611	-4.840	5.216	-3.998	5.358	68.999	15.242	16.855
8351	223	37	25	C	S	40.158	-4.723	-3.611	-4.840	5.216	3.997	5.358	68.999	15.242	16.855
8352	223	37	25	C	S	79.842	-4.723	3.611	-4.840	5.216	-3.998	5.358	68.999	15.242	16.855
8353	223	37	25	C	S	100.158	-4.723	-3.611	-4.840	5.216	3.997	5.358	68.999	15.242	16.855
8354	223	37	25	C	W	19.842	-4.723	3.611	-4.840	5.216	-3.998	5.358	68.999	15.242	16.855
8355	223	37	25	C	W	40.158	-4.723	-3.611	-4.840	5.216	3.997	5.358	68.999	15.242	16.855
8356	223	37	25	C	W	79.842	-4.723	3.611	-4.840	5.216	-3.998	5.358	68.999	15.242	16.855
8357	223	37	25	C	W	100.158	-4.723	-3.611	-4.840	5.216	3.997	5.358	68.999	15.242	16.855
8358	223	37	25	P	S	19.842	-4.723	3.611	-4.840	5.216	-3.998	5.358	68.999	15.242	16.855
8359	223	37	25	P	S	40.158	-4.723	-3.611	-4.840	5.216	3.997	5.358	68.999	15.242	16.855
8360	223	37	25	P	S	79.842	-4.723	3.611	-4.840	5.216	-3.998	5.358	68.999	15.242	16.855
8361	223	37	25	P	S	100.158	-4.723	-3.611	-4.840	5.216	3.997	5.358	68.999	15.242	16.855
8362	223	37	25	P	W	19.842	-4.723	3.611	-4.840	5.216	-3.998	5.358	68.999	15.242	16.855
8363	223	37	25	P	W	40.158	-4.723	-3.611	-4.840	5.216	3.997	5.358	68.999	15.242	16.855
8364	223	37	25	P	W	79.842	-4.723	3.611	-4.840	5.216	-3.998	5.358	68.999	15.242	16.855

BL number	Atoms	γ-PC unit cells	WS_2 unit cells	γ-PC origin atom	WS_2 origin atom	Twist-angle (°)	γ-PC strain 1 (%)	γ-PC strain 2 (%)	γ-PC strain 3 (%)	WS_2 strain 1 (%)	WS_2 strain 2 (%)	WS_2 strain 3 (%)	γ (°)	|**a**|(Å)	|**b**|(Å)
8365	223	37	25	P	W	100.158	-4.723	-3.611	-4.840	5.216	3.997	5.358	68.999	15.242	16.855
8366	224	35	28	C	S	6.178	-2.145	-4.036	0.590	2.241	3.988	-0.583	87.703	17.102	14.413
8367	224	35	28	C	S	10.893	0.418	-4.141	-1.982	-0.415	4.312	2.064	87.581	14.426	17.088
8368	224	35	28	C	S	15.608	-2.145	4.035	0.590	2.241	-3.988	-0.583	87.703	17.102	14.413
8369	224	35	28	C	S	23.413	-2.043	-1.913	0.481	2.130	1.895	-0.477	86.489	14.072	17.535
8370	224	35	28	C	S	25.767	0.353	2.163	-1.921	-0.351	-2.249	1.997	86.433	17.539	14.070
8371	224	35	28	C	S	34.233	0.353	-2.163	-1.921	-0.351	2.249	1.997	86.433	17.539	14.070
8372	224	35	28	C	S	36.587	-2.043	1.914	0.481	2.130	-1.895	-0.477	86.489	14.072	17.534
8373	224	35	28	C	S	44.392	-2.145	-4.036	0.590	2.241	3.988	-0.583	87.703	17.102	14.413
8374	224	35	28	C	S	49.107	0.418	-4.141	-1.982	-0.415	4.312	2.064	87.581	14.426	17.088
8375	224	35	28	C	S	53.822	-2.145	4.035	0.590	2.241	-3.988	-0.583	87.703	17.102	14.413
8376	224	35	28	C	S	66.178	-2.145	-4.036	0.590	2.241	3.988	-0.583	87.703	17.102	14.413
8377	224	35	28	C	S	70.893	0.418	-4.141	-1.982	-0.415	4.312	2.064	87.581	14.426	17.088
8378	224	35	28	C	S	75.609	-2.145	4.035	0.590	2.241	-3.988	-0.583	87.703	17.102	14.413
8379	224	35	28	C	S	83.413	-2.043	-1.913	0.481	2.130	1.895	-0.477	86.489	14.072	17.534
8380	224	35	28	C	S	85.767	0.353	2.163	-1.921	-0.351	-2.249	1.997	86.433	17.539	14.070
8381	224	35	28	C	S	94.233	0.353	-2.163	-1.921	-0.351	2.249	1.997	86.433	17.539	14.070
8382	224	35	28	C	S	96.587	-2.043	1.914	0.481	2.130	-1.895	-0.477	86.489	14.072	17.534
8383	224	35	28	C	S	104.392	-2.145	-4.036	0.590	2.241	3.988	-0.583	87.703	17.102	14.413
8384	224	35	28	C	S	109.107	0.418	-4.141	-1.982	-0.415	4.312	2.064	87.581	14.426	17.088
8385	224	35	28	C	S	113.822	-2.145	4.035	0.590	2.241	-3.988	-0.583	87.703	17.102	14.413
8386	224	35	28	C	W	6.178	-2.145	-4.036	0.590	2.241	3.988	-0.583	87.703	17.102	14.413
8387	224	35	28	C	W	10.893	0.418	-4.141	-1.982	-0.415	4.312	2.064	87.581	14.426	17.088
8388	224	35	28	C	W	15.608	-2.145	4.035	0.590	2.241	-3.988	-0.583	87.703	17.102	14.413
8389	224	35	28	C	W	23.413	-2.043	-1.913	0.481	2.130	1.895	-0.477	86.489	14.072	17.535
8390	224	35	28	C	W	25.767	0.353	2.163	-1.921	-0.351	-2.249	1.997	86.433	17.539	14.070
8391	224	35	28	C	W	34.233	0.353	-2.163	-1.921	-0.351	2.249	1.997	86.433	17.539	14.070
8392	224	35	28	C	W	36.587	-2.043	1.914	0.481	2.130	-1.895	-0.477	86.489	14.072	17.534
8393	224	35	28	C	W	44.392	-2.145	-4.036	0.590	2.241	3.988	-0.583	87.703	17.102	14.413
8394	224	35	28	C	W	49.107	0.418	-4.141	-1.982	-0.415	4.312	2.064	87.581	14.426	17.088
8395	224	35	28	C	W	53.822	-2.145	4.035	0.590	2.241	-3.988	-0.583	87.703	17.102	14.413
8396	224	35	28	C	W	66.178	-2.145	-4.036	0.590	2.241	3.988	-0.583	87.703	17.102	14.413
8397	224	35	28	C	W	70.893	0.418	-4.141	-1.982	-0.415	4.312	2.064	87.581	14.426	17.088
8398	224	35	28	C	W	75.609	-2.145	4.035	0.590	2.241	-3.988	-0.583	87.703	17.102	14.413
8399	224	35	28	C	W	83.413	-2.043	-1.913	0.481	2.130	1.895	-0.477	86.489	14.072	17.534
8400	224	35	28	C	W	85.767	0.353	2.163	-1.921	-0.351	-2.249	1.997	86.433	17.539	14.070
8401	224	35	28	C	W	94.233	0.353	-2.163	-1.921	-0.351	2.249	1.997	86.433	17.539	14.070
8402	224	35	28	C	W	96.587	-2.043	1.914	0.481	2.130	-1.895	-0.477	86.489	14.072	17.534
8403	224	35	28	C	W	104.392	-2.145	-4.036	0.590	2.241	3.988	-0.583	87.703	17.102	14.413
8404	224	35	28	C	W	109.107	0.418	-4.141	-1.982	-0.415	4.312	2.064	87.581	14.426	17.088
8405	224	35	28	C	W	113.822	-2.145	4.035	0.590	2.241	-3.988	-0.583	87.703	17.102	14.413
8406	224	35	28	P	S	6.178	-2.145	-4.036	0.590	2.241	3.988	-0.583	87.703	17.102	14.413
8407	224	35	28	P	S	10.893	0.418	-4.141	-1.982	-0.415	4.312	2.064	87.581	14.426	17.088
8408	224	35	28	P	S	15.608	-2.145	4.035	0.590	2.241	-3.988	-0.583	87.703	17.102	14.413
8409	224	35	28	P	S	23.413	-2.043	-1.913	0.481	2.130	1.895	-0.477	86.489	14.072	17.535
8410	224	35	28	P	S	25.767	0.353	2.163	-1.921	-0.351	-2.249	1.997	86.433	17.539	14.070
8411	224	35	28	P	S	44.392	-2.145	-4.036	0.590	2.241	3.988	-0.583	87.703	17.102	14.413
8412	224	35	28	P	S	49.107	0.418	-4.141	-1.982	-0.415	4.312	2.064	87.581	14.426	17.088
8413	224	35	28	P	S	53.822	-2.145	4.035	0.590	2.241	-3.988	-0.583	87.703	17.102	14.413
8414	224	35	28	P	S	66.178	-2.145	-4.036	0.590	2.241	3.988	-0.583	87.703	17.102	14.413
8415	224	35	28	P	S	70.893	0.418	-4.141	-1.982	-0.415	4.312	2.064	87.581	14.426	17.088

BL number	Atoms	γ-PC unit cells	WS_2 unit cells	γ-PC origin atom	WS_2 origin atom	Twist-angle (°)	γ-PC strain 1 (%)	γ-PC strain 2 (%)	γ-PC strain 3 (%)	WS_2 strain 1 (%)	WS_2 strain 2 (%)	WS_2 strain 3 (%)	γ (°)	|**a**|(Å)	|**b**|(Å)
8416	224	35	28	P	S	75.609	-2.145	4.035	0.590	2.241	-3.988	-0.583	87.703	17.102	14.413
8417	224	35	28	P	S	94.233	0.353	-2.163	-1.921	-0.351	2.249	1.997	86.433	17.539	14.070
8418	224	35	28	P	S	96.587	-2.043	1.914	0.481	2.130	-1.895	-0.477	86.489	14.072	17.534
8419	224	35	28	P	S	104.392	-2.145	-4.036	0.590	2.241	3.988	-0.583	87.703	17.102	14.413
8420	224	35	28	P	S	109.107	0.418	-4.141	-1.982	-0.415	4.312	2.064	87.581	14.426	17.088
8421	224	35	28	P	S	113.822	-2.145	4.035	0.590	2.241	-3.988	-0.583	87.703	17.102	14.413
8422	224	35	28	P	W	6.178	-2.145	-4.036	0.590	2.241	3.988	-0.583	87.703	17.102	14.413
8423	224	35	28	P	W	10.893	0.418	-4.141	-1.982	-0.415	4.312	2.064	87.581	14.426	17.088
8424	224	35	28	P	W	15.608	-2.145	4.035	0.590	2.241	-3.988	-0.583	87.703	17.102	14.413
8425	224	35	28	P	W	34.233	0.353	-2.163	-1.921	-0.351	2.249	1.997	86.433	17.539	14.070
8426	224	35	28	P	W	36.587	-2.043	1.914	0.481	2.130	-1.895	-0.477	86.489	14.072	17.534
8427	224	35	28	P	W	44.392	-2.145	-4.036	0.590	2.241	3.988	-0.583	87.703	17.102	14.413
8428	224	35	28	P	W	49.107	0.418	-4.141	-1.982	-0.415	4.312	2.064	87.581	14.426	17.088
8429	224	35	28	P	W	53.822	-2.145	4.035	0.590	2.241	-3.988	-0.583	87.703	17.102	14.413
8430	224	35	28	P	W	66.178	-2.145	-4.036	0.590	2.241	3.988	-0.583	87.703	17.102	14.413
8431	224	35	28	P	W	70.893	0.418	-4.141	-1.982	-0.415	4.312	2.064	87.581	14.426	17.088
8432	224	35	28	P	W	75.609	-2.145	4.035	0.590	2.241	-3.988	-0.583	87.703	17.102	14.413
8433	224	35	28	P	W	83.413	-2.043	-1.913	0.481	2.130	1.895	-0.477	86.489	14.072	17.534
8434	224	35	28	P	W	85.767	0.353	2.163	-1.921	-0.351	-2.249	1.997	86.433	17.539	14.070
8435	224	35	28	P	W	104.392	-2.145	-4.036	0.590	2.241	3.988	-0.583	87.703	17.102	14.413
8436	224	35	28	P	W	109.107	0.418	-4.141	-1.982	-0.415	4.312	2.064	87.581	14.426	17.088
8437	224	35	28	P	W	113.822	-2.145	4.035	0.590	2.241	-3.988	-0.583	87.703	17.102	14.413
8438	225	36	27	C	S	4.307	-4.685	-5.471	0.086	5.169	5.462	-0.085	76.616	14.491	17.416
8439	225	36	27	C	S	4.715	-1.702	-2.812	-3.007	1.762	2.992	3.200	74.199	17.772	14.349
8440	225	36	27	C	S	6.587	-3.853	0.365	-0.817	4.175	-0.371	0.830	74.171	14.354	17.772
8441	225	36	27	C	S	8.213	-2.359	3.132	-2.359	2.476	-3.287	2.476	77.345	14.845	16.941
8442	225	36	27	C	S	13.898	-0.414	-3.686	-4.228	0.417	4.026	4.619	76.465	14.694	17.183
8443	225	36	27	C	S	19.107	-1.485	5.078	-3.218	1.530	-5.428	3.439	76.430	14.708	17.164
8444	225	36	27	C	S	40.893	-1.485	-5.078	-3.218	1.530	5.427	3.439	76.430	14.708	17.164
8445	225	36	27	C	S	46.102	-0.414	3.686	-4.228	0.417	-4.026	4.619	76.465	17.183	14.694
8446	225	36	27	C	S	51.787	-2.359	-3.132	-2.359	2.476	3.287	2.476	77.346	14.845	16.941
8447	225	36	27	C	S	52.846	-0.955	-1.307	-3.723	0.973	1.412	4.023	74.177	17.772	14.354
8448	225	36	27	C	S	53.413	-3.853	-0.365	-0.817	4.175	0.371	0.830	74.171	14.354	17.772
8449	225	36	27	C	S	55.285	-1.702	2.812	-3.007	1.762	-2.992	3.200	74.199	14.349	17.772
8450	225	36	27	C	S	55.693	-4.685	5.471	0.085	5.169	-5.462	-0.085	76.616	14.491	17.416
8451	225	36	27	C	S	64.307	-4.685	-5.471	0.086	5.169	5.462	-0.085	76.616	14.491	17.416
8452	225	36	27	C	S	64.715	-3.007	-2.852	-1.702	3.200	2.952	1.762	77.364	16.951	14.835
8453	225	36	27	C	S	66.587	-3.853	0.365	-0.817	4.175	-0.371	0.830	74.171	14.354	17.772
8454	225	36	27	C	S	67.154	-0.955	1.307	-3.723	0.973	-1.412	4.023	74.177	17.772	14.354
8455	225	36	27	C	S	68.213	-2.359	3.132	-2.359	2.476	-3.287	2.476	77.346	14.845	16.941
8456	225	36	27	C	S	73.898	-4.228	-3.840	-0.414	4.619	3.872	0.417	76.555	17.184	14.687
8457	225	36	27	C	S	79.107	-1.485	5.078	-3.218	1.530	-5.428	3.439	76.430	14.708	17.164
8458	225	36	27	C	S	100.893	-1.485	-5.078	-3.218	1.530	5.427	3.439	76.430	14.708	17.164
8459	225	36	27	C	S	106.102	-0.414	3.686	-4.228	0.417	-4.026	4.619	76.465	17.183	14.694
8460	225	36	27	C	S	111.787	-2.359	-3.132	-2.359	2.476	3.287	2.476	77.345	14.845	16.941
8461	225	36	27	C	S	112.846	-0.955	-1.307	-3.723	0.973	1.412	4.023	74.177	17.772	14.354
8462	225	36	27	C	S	115.285	-1.702	2.812	-3.007	1.762	-2.992	3.200	74.199	14.349	17.772
8463	225	36	27	C	S	115.693	-4.685	5.471	0.085	5.169	-5.462	-0.085	76.616	14.491	17.416
8464	225	36	27	C	W	4.307	-4.685	-5.471	0.086	5.169	5.462	-0.085	76.616	14.491	17.416
8465	225	36	27	C	W	4.715	-3.007	-2.852	-1.702	3.200	2.952	1.762	77.364	16.951	14.835
8466	225	36	27	C	W	6.587	-3.853	0.365	-0.817	4.175	-0.371	0.830	74.171	14.354	17.772

BL number	Atoms	γ-PC unit cells	WS_2 unit cells	γ-PC origin atom	WS_2 origin atom	Twist-angle (°)	γ-PC strain 1 (%)	γ-PC strain 2 (%)	γ-PC strain 3 (%)	WS_2 strain 1 (%)	WS_2 strain 2 (%)	WS_2 strain 3 (%)	γ (°)	**a** (Å)	**b** (Å)
8467	225	36	27	C	W	7.154	-0.955	1.307	-3.723	0.973	-1.412	4.023	74.177	17.772	14.354
8468	225	36	27	C	W	13.898	-4.228	-3.840	-0.414	4.619	3.872	0.417	76.555	17.184	14.687
8469	225	36	27	C	W	52.846	-0.955	-1.307	-3.723	0.973	1.412	4.023	74.177	17.772	14.354
8470	225	36	27	C	W	55.285	-1.702	2.812	-3.007	1.762	-2.992	3.200	74.199	14.349	17.772
8471	225	36	27	C	W	55.693	-4.685	5.471	0.085	5.169	-5.462	-0.085	76.616	14.491	17.416
8472	225	36	27	C	W	64.307	-4.685	-5.471	0.086	5.169	5.462	-0.085	76.616	14.491	17.416
8473	225	36	27	C	W	64.715	-1.702	-2.812	-3.007	1.762	2.992	3.200	74.199	17.772	14.349
8474	225	36	27	C	W	66.587	-3.853	0.365	-0.817	4.175	-0.371	0.830	74.171	14.354	17.772
8475	225	36	27	C	W	106.102	-4.228	3.840	-0.414	4.619	-3.872	0.417	76.555	17.184	14.687
8476	225	36	27	C	W	112.846	-0.955	-1.307	-3.723	0.973	1.412	4.023	74.177	17.772	14.354
8477	225	36	27	C	W	113.413	-3.853	-0.365	-0.817	4.175	0.371	0.830	74.171	14.354	17.772
8478	225	36	27	C	W	115.285	-3.007	2.852	-1.702	3.200	-2.952	1.762	77.364	16.951	14.835
8479	225	36	27	C	W	115.693	-4.685	5.471	0.085	5.169	-5.462	-0.085	76.616	14.491	17.416
8480	225	36	27	P	S	4.307	-4.685	-5.471	0.086	5.169	5.462	-0.085	76.616	14.491	17.416
8481	225	36	27	P	S	7.154	-0.955	1.307	-3.723	0.973	-1.412	4.023	74.177	17.772	14.354
8482	225	36	27	P	S	8.213	-2.359	3.132	-2.359	2.476	-3.287	2.476	77.345	14.845	16.941
8483	225	36	27	P	S	51.787	-2.359	-3.132	-2.359	2.476	3.287	2.476	77.346	14.845	16.941
8484	225	36	27	P	S	55.285	-3.007	2.852	-1.702	3.200	-2.952	1.762	77.364	16.951	14.835
8485	225	36	27	P	S	55.693	-4.685	5.471	0.085	5.169	-5.462	-0.085	76.616	14.491	17.416
8486	225	36	27	P	S	64.307	-4.685	-5.471	0.086	5.169	5.462	-0.085	76.616	14.491	17.416
8487	225	36	27	P	S	64.715	-3.007	-2.852	-1.702	3.200	2.952	1.762	77.364	16.951	14.835
8488	225	36	27	P	S	68.213	-2.359	3.132	-2.359	2.476	-3.287	2.476	77.346	14.845	16.941
8489	225	36	27	P	S	111.787	-2.359	-3.132	-2.359	2.476	3.287	2.476	77.345	14.845	16.941
8490	225	36	27	P	S	113.413	-3.853	-0.365	-0.817	4.175	0.371	0.830	74.171	14.354	17.772
8491	225	36	27	P	S	115.693	-4.685	5.471	0.085	5.169	-5.462	-0.085	76.616	14.491	17.416
8492	225	36	27	P	W	4.307	-4.685	-5.471	0.086	5.169	5.462	-0.085	76.616	14.491	17.416
8493	225	36	27	P	W	4.715	-3.007	-2.852	-1.702	3.200	2.952	1.762	77.364	16.951	14.835
8494	225	36	27	P	W	53.413	-3.853	-0.365	-0.817	4.175	0.371	0.830	74.171	14.354	17.772
8495	225	36	27	P	W	55.693	-4.685	5.471	0.085	5.169	-5.462	-0.085	76.616	14.491	17.416
8496	225	36	27	P	W	64.307	-4.685	-5.471	0.086	5.169	5.462	-0.085	76.616	14.491	17.416
8497	225	36	27	P	W	67.154	-0.955	1.307	-3.723	0.973	-1.412	4.023	74.177	17.772	14.354
8498	225	36	27	P	W	115.285	-3.007	2.852	-1.702	3.200	-2.952	1.762	77.364	16.951	14.835
8499	225	36	27	P	W	115.693	-4.685	5.471	0.085	5.169	-5.462	-0.085	76.616	14.491	17.416
8500	226	34	30	C	S	7.589	0.334	-4.117	3.049	-0.332	3.881	-2.874	80.996	18.904	13.461
8501	226	34	30	C	S	8.948	-0.599	-1.688	4.051	0.606	1.561	-3.747	89.071	15.901	15.812
8502	226	34	30	C	S	10.893	4.020	1.766	-0.570	-3.721	-1.787	0.577	88.982	15.815	15.899
8503	226	34	30	C	S	12.520	2.326	4.664	1.030	-2.223	-4.570	-1.009	80.897	13.473	18.889
8504	226	34	30	C	S	16.102	2.853	-2.183	0.521	-2.699	2.161	-0.516	67.586	15.495	17.544
8505	226	34	30	C	S	16.337	0.353	-1.814	3.029	-0.351	1.710	-2.856	67.587	17.539	15.500
8506	226	34	30	C	S	19.107	1.980	3.117	1.369	-1.905	-3.034	-1.333	67.539	15.504	17.537
8507	226	34	30	C	S	21.052	1.339	1.721	2.010	-1.304	-1.654	-1.933	89.034	16.211	15.502
8508	226	34	30	C	S	25.050	-0.955	4.069	4.443	0.973	-3.737	-4.081	66.052	17.772	15.482
8509	226	34	30	C	S	25.285	4.262	4.279	-0.791	-3.928	-4.347	0.804	66.018	17.787	15.471
8510	226	34	30	C	S	34.715	4.262	-4.279	-0.791	-3.928	4.347	0.804	66.018	15.471	17.787
8511	226	34	30	C	S	34.950	-0.955	-4.069	4.443	0.973	3.737	-4.081	66.052	17.772	15.482
8512	226	34	30	C	S	38.948	1.339	-1.721	2.010	-1.304	1.654	-1.933	89.034	16.211	15.502
8513	226	34	30	C	S	40.893	1.980	-3.117	1.369	-1.905	3.034	-1.333	67.539	15.504	17.537
8514	226	34	30	C	S	43.663	0.353	1.814	3.029	-0.351	-1.710	-2.856	67.587	17.539	15.500
8515	226	34	30	C	S	43.898	2.853	2.183	0.521	-2.699	-2.161	-0.516	67.586	17.544	15.495
8516	226	34	30	C	S	47.480	2.326	-4.664	1.030	-2.223	4.570	-1.009	80.897	13.473	18.889
8517	226	34	30	C	S	49.107	4.020	-1.766	-0.570	-3.721	1.787	0.577	88.982	15.815	15.899

BL number	Atoms	γ-PC unit cells	WS_2 unit cells	γ-PC origin atom	WS_2 origin atom	Twist-angle (°)	γ-PC strain 1 (%)	γ-PC strain 2 (%)	γ-PC strain 3 (%)	WS_2 strain 1 (%)	WS_2 strain 2 (%)	WS_2 strain 3 (%)	γ (°)	|**a**|(Å)	|**b**|(Å)
8518	226	34	30	C	S	51.052	-0.599	1.688	4.051	0.606	-1.561	-3.747	89.071	15.901	15.812
8519	226	34	30	C	S	52.411	0.334	4.118	3.049	-0.332	-3.881	-2.874	80.997	18.904	13.461
8520	226	34	30	C	S	67.589	0.334	-4.117	3.049	-0.332	3.881	-2.874	80.996	18.904	13.461
8521	226	34	30	C	S	68.948	-0.599	-1.688	4.051	0.606	1.561	-3.747	89.071	15.901	15.812
8522	226	34	30	C	S	70.893	4.020	1.766	-0.570	-3.721	-1.787	0.577	88.982	15.815	15.899
8523	226	34	30	C	S	72.520	2.326	4.664	1.030	-2.223	-4.570	-1.009	80.897	13.473	18.889
8524	226	34	30	C	S	76.102	2.853	-2.183	0.521	-2.699	2.161	-0.516	67.586	15.495	17.544
8525	226	34	30	C	S	76.337	0.353	-1.814	3.029	-0.351	1.710	-2.856	67.587	17.539	15.500
8526	226	34	30	C	S	79.107	1.980	-1.732	1.369	-1.905	1.685	-1.332	89.021	15.504	16.209
8527	226	34	30	C	S	81.052	1.339	1.721	2.010	-1.304	-1.654	-1.933	89.034	16.211	15.502
8528	226	34	30	C	S	85.050	-0.955	4.069	4.443	0.973	-3.737	-4.081	66.052	17.772	15.482
8529	226	34	30	C	S	85.285	4.262	4.279	-0.791	-3.928	-4.347	0.804	66.018	17.787	15.471
8530	226	34	30	C	S	94.715	4.262	-4.279	-0.791	-3.928	4.347	0.804	66.018	15.471	17.787
8531	226	34	30	C	S	94.950	-0.955	-4.069	4.443	0.973	3.737	-4.081	66.052	17.772	15.482
8532	226	34	30	C	S	98.948	1.339	-1.721	2.010	-1.304	1.654	-1.933	89.034	16.211	15.502
8533	226	34	30	C	S	100.893	1.980	-3.117	1.369	-1.905	3.034	-1.333	67.539	15.504	17.537
8534	226	34	30	C	S	103.663	0.353	1.814	3.029	-0.351	-1.710	-2.856	67.587	17.539	15.500
8535	226	34	30	C	S	103.898	2.853	2.183	0.521	-2.699	-2.161	-0.516	67.586	17.544	15.495
8536	226	34	30	C	S	107.480	2.326	-4.664	1.030	-2.223	4.570	-1.009	80.897	13.473	18.889
8537	226	34	30	C	S	109.107	4.020	-1.766	-0.570	-3.721	1.787	0.577	88.982	15.815	15.899
8538	226	34	30	C	S	111.052	-0.599	1.688	4.051	0.606	-1.561	-3.747	89.071	15.901	15.812
8539	226	34	30	C	S	112.411	0.334	4.118	3.049	-0.332	-3.881	-2.874	80.997	18.904	13.461
8540	226	34	30	C	W	7.589	0.334	-4.117	3.049	-0.332	3.881	-2.874	80.996	18.904	13.461
8541	226	34	30	C	W	8.948	-0.599	-1.688	4.051	0.606	1.561	-3.747	89.071	15.901	15.812
8542	226	34	30	C	W	10.893	4.020	1.766	-0.570	-3.721	-1.787	0.577	88.982	15.815	15.899
8543	226	34	30	C	W	12.520	2.326	4.664	1.030	-2.223	-4.570	-1.009	80.897	13.473	18.889
8544	226	34	30	C	W	16.102	2.853	-2.183	0.521	-2.699	2.161	-0.516	67.586	15.495	17.544
8545	226	34	30	C	W	16.337	0.353	-1.814	3.029	-0.351	1.710	-2.856	67.587	17.539	15.500
8546	226	34	30	C	W	19.107	1.980	3.117	1.369	-1.905	-3.034	-1.333	67.539	15.504	17.537
8547	226	34	30	C	W	21.052	1.339	1.721	2.010	-1.304	-1.654	-1.933	89.034	16.211	15.502
8548	226	34	30	C	W	25.050	-0.955	4.069	4.443	0.973	-3.737	-4.081	66.052	17.772	15.482
8549	226	34	30	C	W	25.285	4.262	4.279	-0.791	-3.928	-4.347	0.804	66.018	17.787	15.471
8550	226	34	30	C	W	34.715	4.262	-4.279	-0.791	-3.928	4.347	0.804	66.018	15.471	17.787
8551	226	34	30	C	W	34.950	-0.955	-4.069	4.443	0.973	3.737	-4.081	66.052	17.772	15.482
8552	226	34	30	C	W	38.948	1.339	-1.721	2.010	-1.304	1.654	-1.933	89.034	16.211	15.502
8553	226	34	30	C	W	40.893	1.980	-3.117	1.369	-1.905	3.034	-1.333	67.539	15.504	17.537
8554	226	34	30	C	W	43.663	0.353	1.814	3.029	-0.351	-1.710	-2.856	67.587	17.539	15.500
8555	226	34	30	C	W	43.898	2.853	2.183	0.521	-2.699	-2.161	-0.516	67.586	17.544	15.495
8556	226	34	30	C	W	47.480	2.326	-4.664	1.030	-2.223	4.570	-1.009	80.897	13.473	18.889
8557	226	34	30	C	W	49.107	4.020	-1.766	-0.570	-3.721	1.787	0.577	88.982	15.815	15.899
8558	226	34	30	C	W	51.052	-0.599	1.688	4.051	0.606	-1.561	-3.747	89.071	15.901	15.812
8559	226	34	30	C	W	52.411	0.334	4.118	3.049	-0.332	-3.881	-2.874	80.997	18.904	13.461
8560	226	34	30	C	W	67.589	0.334	-4.117	3.049	-0.332	3.881	-2.874	80.996	18.904	13.461
8561	226	34	30	C	W	68.948	-0.599	-1.688	4.051	0.606	1.561	-3.747	89.071	15.901	15.812
8562	226	34	30	C	W	70.893	4.020	1.766	-0.570	-3.721	-1.787	0.577	88.982	15.815	15.899
8563	226	34	30	C	W	72.520	2.326	4.664	1.030	-2.223	-4.570	-1.009	80.897	13.473	18.889
8564	226	34	30	C	W	76.102	2.853	-2.183	0.521	-2.699	2.161	-0.516	67.586	15.495	17.544
8565	226	34	30	C	W	76.337	0.353	-1.814	3.029	-0.351	1.710	-2.856	67.587	17.539	15.500
8566	226	34	30	C	W	79.107	1.980	-1.732	1.369	-1.905	1.685	-1.332	89.021	15.504	16.209
8567	226	34	30	C	W	81.052	1.339	1.721	2.010	-1.304	-1.654	-1.933	89.034	16.211	15.502
8568	226	34	30	C	W	85.050	-0.955	4.069	4.443	0.973	-3.737	-4.081	66.052	17.772	15.482

BL number	Atoms	γ-PC unit cells	WS_2 unit cells	γ-PC origin atom	WS_2 origin atom	Twist-angle (°)	γ-PC strain 1 (%)	γ-PC strain 2 (%)	γ-PC strain 3 (%)	WS_2 strain 1 (%)	WS_2 strain 2 (%)	WS_2 strain 3 (%)	γ (°)	|**a**|(Å)	|**b**|(Å)
8569	226	34	30	C	W	85.285	4.262	4.279	-0.791	-3.928	-4.347	0.804	66.018	17.787	15.471
8570	226	34	30	C	W	94.715	4.262	-4.279	-0.791	-3.928	4.347	0.804	66.018	15.471	17.787
8571	226	34	30	C	W	94.950	-0.955	-4.069	4.443	0.973	3.737	-4.081	66.052	17.772	15.482
8572	226	34	30	C	W	98.948	1.339	-1.721	2.010	-1.304	1.654	-1.933	89.034	16.211	15.502
8573	226	34	30	C	W	100.893	1.980	-3.117	1.369	-1.905	3.034	-1.333	67.539	15.504	17.537
8574	226	34	30	C	W	103.663	0.353	1.814	3.029	-0.351	-1.710	-2.856	67.587	17.539	15.500
8575	226	34	30	C	W	103.898	2.853	2.183	0.521	-2.699	-2.161	-0.516	67.586	17.544	15.495
8576	226	34	30	C	W	107.480	2.326	-4.664	1.030	-2.223	4.570	-1.009	80.897	13.473	18.889
8577	226	34	30	C	W	109.107	4.020	-1.766	-0.570	-3.721	1.787	0.577	88.982	15.815	15.899
8578	226	34	30	C	W	111.052	-0.599	1.688	4.051	0.606	-1.561	-3.747	89.071	15.901	15.812
8579	226	34	30	C	W	112.411	0.334	4.118	3.049	-0.332	-3.881	-2.874	80.997	18.904	13.461
8580	226	34	30	P	S	7.589	0.334	-4.117	3.049	-0.332	3.881	-2.874	80.996	18.904	13.461
8581	226	34	30	P	S	8.948	-0.599	-1.688	4.051	0.606	1.561	-3.747	89.071	15.901	15.812
8582	226	34	30	P	S	10.893	4.020	1.766	-0.570	-3.721	-1.787	0.577	88.982	15.815	15.899
8583	226	34	30	P	S	12.520	2.326	4.664	1.030	-2.223	-4.570	-1.009	80.897	13.473	18.889
8584	226	34	30	P	S	19.107	1.980	-1.732	1.369	-1.905	1.685	-1.332	89.021	15.504	16.209
8585	226	34	30	P	S	21.052	1.339	1.721	2.010	-1.304	-1.654	-1.933	89.034	16.211	15.502
8586	226	34	30	P	S	25.050	-0.955	4.069	4.443	0.973	-3.737	-4.081	66.052	17.772	15.482
8587	226	34	30	P	S	25.285	4.262	4.279	-0.791	-3.928	-4.347	0.804	66.018	17.787	15.471
8588	226	34	30	P	S	34.715	4.262	-4.279	-0.791	-3.928	4.347	0.804	66.018	15.471	17.787
8589	226	34	30	P	S	34.950	-0.955	-4.069	4.443	0.973	3.737	-4.081	66.052	17.772	15.482
8590	226	34	30	P	S	38.948	1.339	-1.721	2.010	-1.304	1.654	-1.933	89.034	16.211	15.502
8591	226	34	30	P	S	40.893	1.980	1.732	1.369	-1.905	-1.686	-1.333	89.021	15.504	16.209
8592	226	34	30	P	S	47.480	2.326	-4.664	1.030	-2.223	4.570	-1.009	80.897	13.473	18.889
8593	226	34	30	P	S	49.107	4.020	-1.766	-0.570	-3.721	1.787	0.577	88.982	15.815	15.899
8594	226	34	30	P	S	51.052	-0.599	1.688	4.051	0.606	-1.561	-3.747	89.071	15.901	15.812
8595	226	34	30	P	S	52.411	0.334	4.118	3.049	-0.332	-3.881	-2.874	80.997	18.904	13.461
8596	226	34	30	P	S	67.589	0.334	-4.117	3.049	-0.332	3.881	-2.874	80.996	18.904	13.461
8597	226	34	30	P	S	68.948	-0.599	-1.688	4.051	0.606	1.561	-3.747	89.071	15.901	15.812
8598	226	34	30	P	S	70.893	4.020	1.766	-0.570	-3.721	-1.787	0.577	88.982	15.815	15.899
8599	226	34	30	P	S	72.520	2.326	4.664	1.030	-2.223	-4.570	-1.009	80.897	13.473	18.889
8600	226	34	30	P	S	79.107	1.980	-1.732	1.369	-1.905	1.685	-1.332	89.021	15.504	16.209
8601	226	34	30	P	S	81.052	1.339	1.721	2.010	-1.304	-1.654	-1.933	89.034	16.211	15.502
8602	226	34	30	P	S	85.050	-0.955	4.069	4.443	0.973	-3.737	-4.081	66.052	17.772	15.482
8603	226	34	30	P	S	85.285	4.262	4.279	-0.791	-3.928	-4.347	0.804	66.018	17.787	15.471
8604	226	34	30	P	S	94.715	4.262	-4.279	-0.791	-3.928	4.347	0.804	66.018	15.471	17.787
8605	226	34	30	P	S	94.950	-0.955	-4.069	4.443	0.973	3.737	-4.081	66.052	17.772	15.482
8606	226	34	30	P	S	98.948	1.339	-1.721	2.010	-1.304	1.654	-1.933	89.034	16.211	15.502
8607	226	34	30	P	S	100.893	1.980	1.732	1.369	-1.905	-1.686	-1.333	89.021	15.504	16.209
8608	226	34	30	P	S	107.480	2.326	-4.664	1.030	-2.223	4.570	-1.009	80.897	13.473	18.889
8609	226	34	30	P	S	109.107	4.020	-1.766	-0.570	-3.721	1.787	0.577	88.982	15.815	15.899
8610	226	34	30	P	S	111.052	-0.599	1.688	4.051	0.606	-1.561	-3.747	89.071	15.901	15.812
8611	226	34	30	P	S	112.411	0.334	4.118	3.049	-0.332	-3.881	-2.874	80.997	18.904	13.461
8612	226	34	30	P	W	7.589	0.334	-4.117	3.049	-0.332	3.881	-2.874	80.996	18.904	13.461
8613	226	34	30	P	W	8.948	-0.599	-1.688	4.051	0.606	1.561	-3.747	89.071	15.901	15.812
8614	226	34	30	P	W	10.893	4.020	1.766	-0.570	-3.721	-1.787	0.577	88.982	15.815	15.899
8615	226	34	30	P	W	12.520	2.326	4.664	1.030	-2.223	-4.570	-1.009	80.897	13.473	18.889
8616	226	34	30	P	W	19.107	1.980	-1.732	1.369	-1.905	1.685	-1.332	89.021	15.504	16.209
8617	226	34	30	P	W	21.052	1.339	1.721	2.010	-1.304	-1.654	-1.933	89.034	16.211	15.502
8618	226	34	30	P	W	25.050	-0.955	4.069	4.443	0.973	-3.737	-4.081	66.052	17.772	15.482
8619	226	34	30	P	W	25.285	4.262	4.279	-0.791	-3.928	-4.347	0.804	66.018	17.787	15.471

BL number	Atoms	γ-PC unit cells	WS_2 unit cells	γ-PC origin atom	WS_2 origin atom	Twist-angle (°)	γ-PC strain 1 (%)	γ-PC strain 2 (%)	γ-PC strain 3 (%)	WS_2 strain 1 (%)	WS_2 strain 2 (%)	WS_2 strain 3 (%)	γ (°)	|**a**|(Å)	|**b**|(Å)
8620	226	34	30	P	W	34.715	4.262	-4.279	-0.791	-3.928	4.347	0.804	66.018	15.471	17.787
8621	226	34	30	P	W	34.950	-0.955	-4.069	4.443	0.973	3.737	-4.081	66.052	17.772	15.482
8622	226	34	30	P	W	38.948	1.339	-1.721	2.010	-1.304	1.654	-1.933	89.034	16.211	15.502
8623	226	34	30	P	W	40.893	1.980	1.732	1.369	-1.905	-1.686	-1.333	89.021	15.504	16.209
8624	226	34	30	P	W	47.480	2.326	-4.664	1.030	-2.223	4.570	-1.009	80.897	13.473	18.889
8625	226	34	30	P	W	49.107	4.020	-1.766	-0.570	-3.721	1.787	0.577	88.982	15.815	15.899
8626	226	34	30	P	W	51.052	-0.599	1.688	4.051	0.606	-1.561	-3.747	89.071	15.901	15.812
8627	226	34	30	P	W	52.411	0.334	4.118	3.049	-0.332	-3.881	-2.874	80.997	18.904	13.461
8628	226	34	30	P	W	67.589	0.334	-4.117	3.049	-0.332	3.881	-2.874	80.996	18.904	13.461
8629	226	34	30	P	W	68.948	-0.599	-1.688	4.051	0.606	1.561	-3.747	89.071	15.901	15.812
8630	226	34	30	P	W	70.893	4.020	1.766	-0.570	-3.721	-1.787	0.577	88.982	15.815	15.899
8631	226	34	30	P	W	72.520	2.326	4.664	1.030	-2.223	-4.570	-1.009	80.897	13.473	18.889
8632	226	34	30	P	W	79.107	1.980	-1.732	1.369	-1.905	1.685	-1.332	89.021	15.504	16.209
8633	226	34	30	P	W	81.052	1.339	1.721	2.010	-1.304	-1.654	-1.933	89.034	16.211	15.502
8634	226	34	30	P	W	85.050	-0.955	4.069	4.443	0.973	-3.737	-4.081	66.052	17.772	15.482
8635	226	34	30	P	W	85.285	4.262	4.279	-0.791	-3.928	-4.347	0.804	66.018	17.787	15.471
8636	226	34	30	P	W	94.715	4.262	-4.279	-0.791	-3.928	4.347	0.804	66.018	15.471	17.787
8637	226	34	30	P	W	94.950	-0.955	-4.069	4.443	0.973	3.737	-4.081	66.052	17.772	15.482
8638	226	34	30	P	W	98.948	1.339	-1.721	2.010	-1.304	1.654	-1.933	89.034	16.211	15.502
8639	226	34	30	P	W	100.893	1.980	1.732	1.369	-1.905	-1.686	-1.333	89.021	15.504	16.209
8640	226	34	30	P	W	107.480	2.326	-4.664	1.030	-2.223	4.570	-1.009	80.897	13.473	18.889
8641	226	34	30	P	W	109.107	4.020	-1.766	-0.570	-3.721	1.787	0.577	88.982	15.815	15.899
8642	226	34	30	P	W	111.052	-0.599	1.688	4.051	0.606	-1.561	-3.747	89.071	15.901	15.812
8643	226	34	30	P	W	112.411	0.334	4.118	3.049	-0.332	-3.881	-2.874	80.997	18.904	13.461
8644	226	37	26	C	S	1.359	-3.292	-1.290	-4.473	3.523	1.417	4.912	67.665	17.337	15.238
8645	226	37	26	C	S	1.945	-4.723	-0.319	-3.033	5.216	0.339	3.229	67.673	15.242	17.333
8646	226	37	26	C	S	56.996	-3.388	-1.454	-4.378	3.635	1.593	4.798	67.683	17.335	15.238
8647	226	37	26	C	S	61.359	-3.292	-1.290	-4.473	3.523	1.417	4.912	67.664	17.337	15.239
8648	226	37	26	C	S	116.996	-3.388	-1.454	-4.378	3.635	1.593	4.798	67.683	17.335	15.238
8649	226	37	26	C	S	118.641	-3.292	1.290	-4.473	3.523	-1.417	4.912	67.665	15.239	17.337
8650	226	37	26	C	W	1.359	-3.292	-1.290	-4.473	3.523	1.417	4.912	67.665	17.337	15.238
8651	226	37	26	C	W	56.996	-3.388	-1.454	-4.378	3.635	1.593	4.798	67.683	17.335	15.238
8652	226	37	26	C	W	58.641	-3.292	1.290	-4.473	3.523	-1.417	4.912	67.664	15.238	17.337
8653	226	37	26	C	W	61.359	-3.292	-1.290	-4.473	3.523	1.417	4.912	67.664	17.337	15.239
8654	226	37	26	C	W	61.945	-4.723	-0.319	-3.033	5.216	0.339	3.229	67.673	15.242	17.333
8655	226	37	26	C	W	116.996	-3.388	-1.454	-4.378	3.635	1.593	4.798	67.683	17.335	15.238
8656	226	37	26	P	S	1.359	-3.292	-1.290	-4.473	3.523	1.417	4.912	67.665	17.337	15.238
8657	226	37	26	P	S	56.996	-3.388	-1.454	-4.378	3.635	1.593	4.798	67.683	17.335	15.238
8658	226	37	26	P	S	58.641	-3.292	1.290	-4.473	3.523	-1.417	4.912	67.664	15.238	17.337
8659	226	37	26	P	S	61.359	-3.292	-1.290	-4.473	3.523	1.417	4.912	67.664	17.337	15.239
8660	226	37	26	P	S	61.945	-4.723	-0.319	-3.033	5.216	0.339	3.229	67.673	15.242	17.333
8661	226	37	26	P	S	116.996	-3.388	-1.454	-4.378	3.635	1.593	4.798	67.683	17.335	15.238
8662	226	37	26	P	W	1.359	-3.292	-1.290	-4.473	3.523	1.417	4.912	67.665	17.337	15.238
8663	226	37	26	P	W	1.945	-4.723	-0.319	-3.033	5.216	0.339	3.229	67.673	15.242	17.333
8664	226	37	26	P	W	56.996	-3.388	-1.454	-4.378	3.635	1.593	4.798	67.683	17.335	15.238
8665	226	37	26	P	W	61.359	-3.292	-1.290	-4.473	3.523	1.417	4.912	67.664	17.337	15.239
8666	226	37	26	P	W	116.996	-3.388	-1.454	-4.378	3.635	1.593	4.798	67.683	17.335	15.238
8667	226	37	26	P	W	118.641	-3.292	1.290	-4.473	3.523	-1.417	4.912	67.665	15.239	17.337
8668	227	35	29	C	S	0.000	5.011	-3.465	-4.419	-4.554	3.800	4.848	78.726	16.799	15.244
8669	227	35	29	C	S	1.945	-4.723	-0.225	5.380	5.216	0.203	-4.857	78.910	15.242	16.797
8670	227	35	29	C	S	7.154	-0.955	-3.004	1.125	0.973	2.938	-1.100	77.995	17.772	14.417

BL number	Atoms	γ-PC unit cells	WS_2 unit cells	γ-PC origin atom	WS_2 origin atom	Twist-angle (°)	γ-PC strain 1 (%)	γ-PC strain 2 (%)	γ-PC strain 3 (%)	WS_2 strain 1 (%)	WS_2 strain 2 (%)	WS_2 strain 3 (%)	γ (°)	|**a**|(Å)	|**b**|(Å)
8671	227	35	29	C	S	10.893	0.418	-4.733	-0.268	-0.415	4.758	0.269	82.565	14.426	17.518
8672	227	35	29	C	S	16.337	0.353	4.753	-0.203	-0.351	-4.772	0.204	82.561	17.539	14.409
8673	227	35	29	C	S	18.613	3.582	-2.494	-3.204	-3.342	2.664	3.423	73.799	18.586	14.057
8674	227	35	29	C	S	23.691	2.390	-1.559	-2.139	-2.281	1.629	2.235	69.468	19.022	14.074
8675	227	35	29	C	S	27.126	1.029	4.369	-0.863	-1.008	-4.446	0.878	69.384	19.034	14.067
8676	227	35	29	C	S	32.874	1.029	-4.369	-0.863	-1.008	4.446	0.878	69.384	19.034	14.067
8677	227	35	29	C	S	36.309	2.390	1.559	-2.139	-2.281	-1.629	2.235	69.468	14.074	19.022
8678	227	35	29	C	S	41.387	3.582	2.494	-3.204	-3.342	-2.664	3.423	73.799	18.586	14.057
8679	227	35	29	C	S	43.663	0.353	-4.752	-0.203	-0.351	4.772	0.204	82.561	17.539	14.409
8680	227	35	29	C	S	49.107	0.418	-3.549	-0.268	-0.415	3.569	0.269	77.946	14.426	17.763
8681	227	35	29	C	S	52.846	-0.955	3.004	1.125	0.973	-2.938	-1.100	77.995	17.772	14.417
8682	227	35	29	C	S	58.055	-4.723	0.225	5.380	5.216	-0.203	-4.857	78.910	15.242	16.797
8683	227	35	29	C	S	60.000	5.011	3.465	-4.419	-4.554	-3.800	4.848	78.726	16.799	15.244
8684	227	35	29	C	S	61.945	-4.723	-0.225	5.380	5.216	0.203	-4.857	78.910	15.242	16.797
8685	227	35	29	C	S	67.154	-0.955	-3.004	1.125	0.973	2.938	-1.100	77.995	17.772	14.417
8686	227	35	29	C	S	70.893	0.418	-4.733	-0.268	-0.415	4.758	0.269	82.565	14.426	17.518
8687	227	35	29	C	S	76.337	0.353	4.753	-0.203	-0.351	-4.772	0.204	82.561	17.539	14.409
8688	227	35	29	C	S	78.613	3.582	-2.494	-3.204	-3.342	2.664	3.423	73.799	18.586	14.057
8689	227	35	29	C	S	83.691	2.390	-1.559	-2.139	-2.281	1.629	2.235	69.468	19.022	14.074
8690	227	35	29	C	S	87.126	1.029	4.369	-0.863	-1.008	-4.446	0.878	69.384	19.034	14.067
8691	227	35	29	C	S	92.874	1.029	-4.369	-0.863	-1.008	4.446	0.878	69.384	19.034	14.067
8692	227	35	29	C	S	96.309	2.390	1.559	-2.139	-2.281	-1.629	2.235	69.468	14.074	19.022
8693	227	35	29	C	S	101.387	3.582	2.494	-3.204	-3.342	-2.664	3.423	73.799	18.586	14.057
8694	227	35	29	C	S	103.663	0.353	-4.752	-0.203	-0.351	4.772	0.204	82.561	17.539	14.409
8695	227	35	29	C	S	109.107	0.418	-3.549	-0.268	-0.415	3.569	0.269	77.946	14.426	17.763
8696	227	35	29	C	S	112.846	-0.955	3.004	1.125	0.973	-2.938	-1.100	77.995	17.772	14.417
8697	227	35	29	C	S	118.055	-4.723	0.225	5.380	5.216	-0.203	-4.857	78.910	15.242	16.797
8698	227	35	29	C	W	0.000	5.011	-3.465	-4.419	-4.554	3.800	4.848	78.726	16.799	15.244
8699	227	35	29	C	W	1.945	-4.723	-0.225	5.380	5.216	0.203	-4.857	78.910	15.242	16.797
8700	227	35	29	C	W	7.154	-0.955	-3.004	1.125	0.973	2.938	-1.100	77.995	17.772	14.417
8701	227	35	29	C	W	10.893	0.418	-4.733	-0.268	-0.415	4.758	0.269	82.565	14.426	17.518
8702	227	35	29	C	W	16.337	0.353	4.753	-0.203	-0.351	-4.772	0.204	82.561	17.539	14.409
8703	227	35	29	C	W	18.613	3.582	-2.494	-3.204	-3.342	2.664	3.423	73.799	18.586	14.057
8704	227	35	29	C	W	23.691	2.390	-1.559	-2.139	-2.281	1.629	2.235	69.468	19.022	14.074
8705	227	35	29	C	W	27.126	1.029	4.369	-0.863	-1.008	-4.446	0.878	69.384	19.034	14.067
8706	227	35	29	C	W	32.874	1.029	-4.369	-0.863	-1.008	4.446	0.878	69.384	19.034	14.067
8707	227	35	29	C	W	36.309	2.390	1.559	-2.139	-2.281	-1.629	2.235	69.468	14.074	19.022
8708	227	35	29	C	W	41.387	3.582	2.494	-3.204	-3.342	-2.664	3.423	73.799	18.586	14.057
8709	227	35	29	C	W	43.663	0.353	-4.752	-0.203	-0.351	4.772	0.204	82.561	17.539	14.409
8710	227	35	29	C	W	49.107	0.418	-3.549	-0.268	-0.415	3.569	0.269	77.946	14.426	17.763
8711	227	35	29	C	W	52.846	-0.955	3.004	1.125	0.973	-2.938	-1.100	77.995	17.772	14.417
8712	227	35	29	C	W	58.055	-4.723	0.225	5.380	5.216	-0.203	-4.857	78.910	15.242	16.797
8713	227	35	29	C	W	60.000	5.011	3.465	-4.419	-4.554	-3.800	4.848	78.726	16.799	15.244
8714	227	35	29	C	W	61.945	-4.723	-0.225	5.380	5.216	0.203	-4.857	78.910	15.242	16.797
8715	227	35	29	C	W	67.154	-0.955	-3.004	1.125	0.973	2.938	-1.100	77.995	17.772	14.417
8716	227	35	29	C	W	70.893	0.418	-4.733	-0.268	-0.415	4.758	0.269	82.565	14.426	17.518
8717	227	35	29	C	W	76.337	0.353	4.753	-0.203	-0.351	-4.772	0.204	82.561	17.539	14.409
8718	227	35	29	C	W	78.613	3.582	-2.494	-3.204	-3.342	2.664	3.423	73.799	18.586	14.057
8719	227	35	29	C	W	83.691	2.390	-1.559	-2.139	-2.281	1.629	2.235	69.468	19.022	14.074
8720	227	35	29	C	W	87.126	1.029	4.369	-0.863	-1.008	-4.446	0.878	69.384	19.034	14.067
8721	227	35	29	C	W	92.874	1.029	-4.369	-0.863	-1.008	4.446	0.878	69.384	19.034	14.067

BL number	Atoms	γ-PC unit cells	WS_2 unit cells	γ-PC origin atom	WS_2 origin atom	Twist-angle (°)	γ-PC strain 1 (%)	γ-PC strain 2 (%)	γ-PC strain 3 (%)	WS_2 strain 1 (%)	WS_2 strain 2 (%)	WS_2 strain 3 (%)	γ (°)	|**a**|(Å)	|**b**|(Å)
8722	227	35	29	C	W	96.309	2.390	1.559	-2.139	-2.281	-1.629	2.235	69.468	14.074	19.022
8723	227	35	29	C	W	101.387	3.582	2.494	-3.204	-3.342	-2.664	3.423	73.799	18.586	14.057
8724	227	35	29	C	W	103.663	0.353	-4.752	-0.203	-0.351	4.772	0.204	82.561	17.539	14.409
8725	227	35	29	C	W	109.107	0.418	-3.549	-0.268	-0.415	3.569	0.269	77.946	14.426	17.763
8726	227	35	29	C	W	112.846	-0.955	3.004	1.125	0.973	-2.938	-1.100	77.995	17.772	14.417
8727	227	35	29	C	W	118.055	-4.723	0.225	5.380	5.216	-0.203	-4.857	78.910	15.242	16.797
8728	227	35	29	P	S	0.000	5.011	-3.465	-4.419	-4.554	3.800	4.848	78.726	16.799	15.244
8729	227	35	29	P	S	1.945	-4.723	-0.225	5.380	5.216	0.203	-4.857	78.910	15.242	16.797
8730	227	35	29	P	S	7.154	-0.955	-3.004	1.125	0.973	2.938	-1.100	77.995	17.772	14.417
8731	227	35	29	P	S	10.893	0.418	-4.733	-0.268	-0.415	4.758	0.269	82.565	14.426	17.518
8732	227	35	29	P	S	16.337	0.353	4.753	-0.203	-0.351	-4.772	0.204	82.561	17.539	14.409
8733	227	35	29	P	S	18.613	3.582	-2.494	-3.204	-3.342	2.664	3.423	73.799	18.586	14.057
8734	227	35	29	P	S	23.691	2.390	-1.559	-2.139	-2.281	1.629	2.235	69.468	19.022	14.074
8735	227	35	29	P	S	27.126	1.029	4.369	-0.863	-1.008	-4.446	0.878	69.384	19.034	14.067
8736	227	35	29	P	S	32.874	1.029	-4.369	-0.863	-1.008	4.446	0.878	69.384	19.034	14.067
8737	227	35	29	P	S	36.309	2.390	1.559	-2.139	-2.281	-1.629	2.235	69.468	14.074	19.022
8738	227	35	29	P	S	41.387	3.582	2.494	-3.204	-3.342	-2.664	3.423	73.799	18.586	14.057
8739	227	35	29	P	S	43.663	0.353	-4.752	-0.203	-0.351	4.772	0.204	82.561	17.539	14.409
8740	227	35	29	P	S	49.107	0.418	-3.549	-0.268	-0.415	3.569	0.269	77.946	14.426	17.763
8741	227	35	29	P	S	52.846	-0.955	3.004	1.125	0.973	-2.938	-1.100	77.995	17.772	14.417
8742	227	35	29	P	S	58.055	-4.723	0.225	5.380	5.216	-0.203	-4.857	78.910	15.242	16.797
8743	227	35	29	P	S	60.000	5.011	3.465	-4.419	-4.554	-3.800	4.848	78.726	16.799	15.244
8744	227	35	29	P	S	61.945	-4.723	-0.225	5.380	5.216	0.203	-4.857	78.910	15.242	16.797
8745	227	35	29	P	S	67.154	-0.955	-3.004	1.125	0.973	2.938	-1.100	77.995	17.772	14.417
8746	227	35	29	P	S	70.893	0.418	-4.733	-0.268	-0.415	4.758	0.269	82.565	14.426	17.518
8747	227	35	29	P	S	76.337	0.353	4.753	-0.203	-0.351	-4.772	0.204	82.561	17.539	14.409
8748	227	35	29	P	S	78.613	3.582	-2.494	-3.204	-3.342	2.664	3.423	73.799	18.586	14.057
8749	227	35	29	P	S	83.691	2.390	-1.559	-2.139	-2.281	1.629	2.235	69.468	19.022	14.074
8750	227	35	29	P	S	87.126	1.029	4.369	-0.863	-1.008	-4.446	0.878	69.384	19.034	14.067
8751	227	35	29	P	S	92.874	1.029	-4.369	-0.863	-1.008	4.446	0.878	69.384	19.034	14.067
8752	227	35	29	P	S	96.309	2.390	1.559	-2.139	-2.281	-1.629	2.235	69.468	14.074	19.022
8753	227	35	29	P	S	101.387	3.582	2.494	-3.204	-3.342	-2.664	3.423	73.799	18.586	14.057
8754	227	35	29	P	S	103.663	0.353	-4.752	-0.203	-0.351	4.772	0.204	82.561	17.539	14.409
8755	227	35	29	P	S	109.107	0.418	-3.549	-0.268	-0.415	3.569	0.269	77.946	14.426	17.763
8756	227	35	29	P	S	112.846	-0.955	3.004	1.125	0.973	-2.938	-1.100	77.995	17.772	14.417
8757	227	35	29	P	S	118.055	-4.723	0.225	5.380	5.216	-0.203	-4.857	78.910	15.242	16.797
8758	227	35	29	P	W	0.000	5.011	-3.465	-4.419	-4.554	3.800	4.848	78.726	16.799	15.244
8759	227	35	29	P	W	1.945	-4.723	-0.225	5.380	5.216	0.203	-4.857	78.910	15.242	16.797
8760	227	35	29	P	W	7.154	-0.955	-3.004	1.125	0.973	2.938	-1.100	77.995	17.772	14.417
8761	227	35	29	P	W	10.893	0.418	-4.733	-0.268	-0.415	4.758	0.269	82.565	14.426	17.518
8762	227	35	29	P	W	16.337	0.353	4.753	-0.203	-0.351	-4.772	0.204	82.561	17.539	14.409
8763	227	35	29	P	W	18.613	3.582	-2.494	-3.204	-3.342	2.664	3.423	73.799	18.586	14.057
8764	227	35	29	P	W	23.691	2.390	-1.559	-2.139	-2.281	1.629	2.235	69.468	19.022	14.074
8765	227	35	29	P	W	27.126	1.029	4.369	-0.863	-1.008	-4.446	0.878	69.384	19.034	14.067
8766	227	35	29	P	W	32.874	1.029	-4.369	-0.863	-1.008	4.446	0.878	69.384	19.034	14.067
8767	227	35	29	P	W	36.309	2.390	1.559	-2.139	-2.281	-1.629	2.235	69.468	14.074	19.022
8768	227	35	29	P	W	41.387	3.582	2.494	-3.204	-3.342	-2.664	3.423	73.799	18.586	14.057
8769	227	35	29	P	W	43.663	0.353	-4.752	-0.203	-0.351	4.772	0.204	82.561	17.539	14.409
8770	227	35	29	P	W	49.107	0.418	-3.549	-0.268	-0.415	3.569	0.269	77.946	14.426	17.763
8771	227	35	29	P	W	52.846	-0.955	3.004	1.125	0.973	-2.938	-1.100	77.995	17.772	14.417
8772	227	35	29	P	W	58.055	-4.723	0.225	5.380	5.216	-0.203	-4.857	78.910	15.242	16.797

BL number	Atoms	γ-PC unit cells	WS_2 unit cells	γ-PC origin atom	WS_2 origin atom	Twist-angle (°)	γ-PC strain 1 (%)	γ-PC strain 2 (%)	γ-PC strain 3 (%)	WS_2 strain 1 (%)	WS_2 strain 2 (%)	WS_2 strain 3 (%)	γ (°)	$\lvert \mathbf{a} \rvert$(Å)	$\lvert \mathbf{b} \rvert$(Å)
8773	227	35	29	P	W	60.000	5.011	3.465	-4.419	-4.554	-3.800	4.848	78.726	16.799	15.244
8774	227	35	29	P	W	61.945	-4.723	-0.225	5.380	5.216	0.203	-4.857	78.910	15.242	16.797
8775	227	35	29	P	W	67.154	-0.955	-3.004	1.125	0.973	2.938	-1.100	77.995	17.772	14.417
8776	227	35	29	P	W	70.893	0.418	-4.733	-0.268	-0.415	4.758	0.269	82.565	14.426	17.518
8777	227	35	29	P	W	76.337	0.353	4.753	-0.203	-0.351	-4.772	0.204	82.561	17.539	14.409
8778	227	35	29	P	W	78.613	3.582	-2.494	-3.204	-3.342	2.664	3.423	73.799	18.586	14.057
8779	227	35	29	P	W	83.691	2.390	-1.559	-2.139	-2.281	1.629	2.235	69.468	19.022	14.074
8780	227	35	29	P	W	87.126	1.029	4.369	-0.863	-1.008	-4.446	0.878	69.384	19.034	14.067
8781	227	35	29	P	W	92.874	1.029	-4.369	-0.863	-1.008	4.446	0.878	69.384	19.034	14.067
8782	227	35	29	P	W	96.309	2.390	1.559	-2.139	-2.281	-1.629	2.235	69.468	14.074	19.022
8783	227	35	29	P	W	101.387	3.582	2.494	-3.204	-3.342	-2.664	3.423	73.799	18.586	14.057
8784	227	35	29	P	W	103.663	0.353	-4.752	-0.203	-0.351	4.772	0.204	82.561	17.539	14.409
8785	227	35	29	P	W	109.107	0.418	-3.549	-0.268	-0.415	3.569	0.269	77.946	14.426	17.763
8786	227	35	29	P	W	112.846	-0.955	3.004	1.125	0.973	-2.938	-1.100	77.995	17.772	14.417
8787	227	35	29	P	W	118.055	-4.723	0.225	5.380	5.216	-0.203	-4.857	78.910	15.242	16.797
8788	228	33	32	C	S	18.613	3.582	-3.012	4.767	-3.342	2.750	-4.352	85.389	18.586	13.820
8789	228	33	32	C	S	21.787	5.011	2.756	3.344	-4.554	-2.583	-3.134	85.426	13.826	18.577
8790	228	33	32	C	S	38.213	5.011	-2.756	3.344	-4.554	2.583	-3.134	85.426	13.826	18.577
8791	228	33	32	C	S	41.387	3.582	3.012	4.767	-3.342	-2.750	-4.352	85.389	18.586	13.820
8792	228	33	32	C	S	78.613	3.582	-3.012	4.767	-3.342	2.750	-4.352	85.389	18.586	13.820
8793	228	33	32	C	S	81.787	5.011	2.756	3.344	-4.554	-2.583	-3.134	85.426	13.826	18.577
8794	228	33	32	C	S	98.213	5.011	-2.756	3.344	-4.554	2.583	-3.134	85.426	13.826	18.577
8795	228	33	32	C	S	101.387	3.582	3.012	4.767	-3.342	-2.750	-4.352	85.389	18.586	13.820
8796	228	33	32	C	W	18.613	3.582	-3.012	4.767	-3.342	2.750	-4.352	85.389	18.586	13.820
8797	228	33	32	C	W	21.787	5.011	2.756	3.344	-4.554	-2.583	-3.134	85.426	13.826	18.577
8798	228	33	32	C	W	38.213	5.011	-2.756	3.344	-4.554	2.583	-3.134	85.426	13.826	18.577
8799	228	33	32	C	W	41.387	3.582	3.012	4.767	-3.342	-2.750	-4.352	85.389	18.586	13.820
8800	228	33	32	C	W	78.613	3.582	-3.012	4.767	-3.342	2.750	-4.352	85.389	18.586	13.820
8801	228	33	32	C	W	81.787	5.011	2.756	3.344	-4.554	-2.583	-3.134	85.426	13.826	18.577
8802	228	33	32	C	W	98.213	5.011	-2.756	3.344	-4.554	2.583	-3.134	85.426	13.826	18.577
8803	228	33	32	C	W	101.387	3.582	3.012	4.767	-3.342	-2.750	-4.352	85.389	18.586	13.820
8804	228	33	32	P	S	38.213	5.011	-2.756	3.344	-4.554	2.583	-3.134	85.426	13.826	18.577
8805	228	33	32	P	S	41.387	3.582	3.012	4.767	-3.342	-2.750	-4.352	85.389	18.586	13.820
8806	228	33	32	P	S	78.613	3.582	-3.012	4.767	-3.342	2.750	-4.352	85.389	18.586	13.820
8807	228	33	32	P	S	81.787	5.011	2.756	3.344	-4.554	-2.583	-3.134	85.426	13.826	18.577
8808	228	33	32	P	W	18.613	3.582	-3.012	4.767	-3.342	2.750	-4.352	85.389	18.586	13.820
8809	228	33	32	P	W	21.787	5.011	2.756	3.344	-4.554	-2.583	-3.134	85.426	13.826	18.577
8810	228	33	32	P	W	98.213	5.011	-2.756	3.344	-4.554	2.583	-3.134	85.426	13.826	18.577
8811	228	33	32	P	W	101.387	3.582	3.012	4.767	-3.342	-2.750	-4.352	85.389	18.586	13.820
8812	228	36	28	C	S	8.948	1.048	1.882	-3.892	-1.026	-2.041	4.221	88.878	17.420	14.351
8813	228	36	28	C	S	10.893	-1.983	-2.947	-0.982	2.064	3.007	1.002	77.578	12.906	19.819
8814	228	36	28	C	S	13.898	-0.414	2.211	-2.533	0.417	-2.329	2.668	77.630	19.824	12.901
8815	228	36	28	C	S	46.102	-0.414	-2.211	-2.533	0.417	2.329	2.668	77.630	19.824	12.901
8816	228	36	28	C	S	49.107	-1.983	-5.305	-0.982	2.064	5.412	1.002	82.203	12.906	19.536
8817	228	36	28	C	S	51.052	1.048	-1.882	-3.892	-1.026	2.041	4.221	88.878	17.420	14.351
8818	228	36	28	C	S	53.413	-3.853	2.191	1.005	4.175	-2.148	-0.985	88.757	14.354	17.416
8819	228	36	28	C	S	66.587	-3.853	-2.191	1.005	4.175	2.148	-0.985	88.757	14.354	17.416
8820	228	36	28	C	S	68.948	1.048	1.882	-3.892	-1.026	-2.041	4.221	88.878	17.420	14.351
8821	228	36	28	C	S	70.893	-1.983	-2.947	-0.982	2.064	3.007	1.002	77.578	12.906	19.819
8822	228	36	28	C	S	73.898	-0.414	2.211	-2.533	0.417	-2.329	2.668	77.630	19.824	12.901
8823	228	36	28	C	S	106.102	-0.414	-2.211	-2.533	0.417	2.329	2.668	77.630	19.824	12.901

BL number	Atoms	γ-PC unit cells	WS_2 unit cells	γ-PC origin atom	WS_2 origin atom	Twist-angle (°)	γ-PC strain 1 (%)	γ-PC strain 2 (%)	γ-PC strain 3 (%)	WS_2 strain 1 (%)	WS_2 strain 2 (%)	WS_2 strain 3 (%)	γ (°)	|**a**|(Å)	|**b**|(Å)
8824	228	36	28	C	S	109.107	-1.983	-5.305	-0.982	2.064	5.412	1.002	82.203	12.906	19.536
8825	228	36	28	C	S	111.052	1.048	-1.882	-3.892	-1.026	2.041	4.221	88.878	17.420	14.351
8826	228	36	28	C	S	113.413	-3.853	2.191	1.005	4.175	-2.148	-0.985	88.757	14.354	17.416
8827	228	36	28	C	W	6.587	-3.853	-2.191	1.005	4.175	2.148	-0.985	88.757	14.354	17.416
8828	228	36	28	C	W	10.893	-1.983	-2.947	-0.982	2.064	3.007	1.002	77.578	12.906	19.819
8829	228	36	28	C	W	13.898	-0.414	2.211	-2.533	0.417	-2.329	2.668	77.630	19.824	12.901
8830	228	36	28	C	W	46.102	-0.414	-2.211	-2.533	0.417	2.329	2.668	77.630	19.824	12.901
8831	228	36	28	C	W	49.107	-1.983	2.947	-0.982	2.064	-3.007	1.002	77.578	12.906	19.819
8832	228	36	28	C	W	70.893	-1.983	-2.947	-0.982	2.064	3.007	1.002	77.578	12.906	19.819
8833	228	36	28	C	W	73.898	-0.414	2.211	-2.533	0.417	-2.329	2.668	77.630	19.824	12.901
8834	228	36	28	C	W	106.102	-0.414	-2.211	-2.533	0.417	2.329	2.668	77.630	19.824	12.901
8835	228	36	28	C	W	109.107	-1.983	2.947	-0.982	2.064	-3.007	1.002	77.578	12.906	19.819
8836	228	36	28	P	S	8.948	1.048	1.882	-3.892	-1.026	-2.041	4.221	88.878	17.420	14.351
8837	228	36	28	P	S	10.893	-1.983	5.305	-0.982	2.064	-5.412	1.002	82.203	12.906	19.536
8838	228	36	28	P	S	46.102	-0.414	-2.211	-2.533	0.417	2.329	2.668	77.630	19.824	12.901
8839	228	36	28	P	S	49.107	-1.983	-5.305	-0.982	2.064	5.412	1.002	82.203	12.906	19.536
8840	228	36	28	P	S	51.052	1.048	-1.882	-3.892	-1.026	2.041	4.221	88.878	17.420	14.351
8841	228	36	28	P	S	53.413	-3.853	2.191	1.005	4.175	-2.148	-0.985	88.757	14.354	17.416
8842	228	36	28	P	S	66.587	-3.853	-2.191	1.005	4.175	2.148	-0.985	88.757	14.354	17.416
8843	228	36	28	P	S	68.948	1.048	1.882	-3.892	-1.026	-2.041	4.221	88.878	17.420	14.351
8844	228	36	28	P	S	70.893	-1.983	-2.947	-0.982	2.064	3.007	1.002	77.578	12.906	19.819
8845	228	36	28	P	S	73.898	-0.414	2.211	-2.533	0.417	-2.329	2.668	77.630	19.824	12.901
8846	228	36	28	P	S	109.107	-1.983	-5.305	-0.982	2.064	5.412	1.002	82.203	12.906	19.536
8847	228	36	28	P	S	111.052	1.048	-1.882	-3.892	-1.026	2.041	4.221	88.878	17.420	14.351
8848	228	36	28	P	S	113.413	-3.853	2.191	1.005	4.175	-2.148	-0.985	88.757	14.354	17.416
8849	228	36	28	P	W	6.587	-3.853	-2.191	1.005	4.175	2.148	-0.985	88.757	14.354	17.416
8850	228	36	28	P	W	10.893	-1.983	-2.947	-0.982	2.064	3.007	1.002	77.578	12.906	19.819
8851	228	36	28	P	W	13.898	-0.414	2.211	-2.533	0.417	-2.329	2.668	77.630	19.824	12.901
8852	228	36	28	P	W	106.102	-0.414	-2.211	-2.533	0.417	2.329	2.668	77.630	19.824	12.901
8853	228	36	28	P	W	109.107	-1.983	2.947	-0.982	2.064	-3.007	1.002	77.578	12.906	19.819
8854	229	34	31	C	S	17.696	1.029	-4.660	4.071	-1.008	4.309	-3.765	76.364	19.034	13.816
8855	229	34	31	C	S	21.787	5.011	2.675	0.157	-4.554	-2.666	-0.157	76.485	13.826	19.017
8856	229	34	31	C	S	25.693	2.326	1.783	2.731	-2.223	-1.691	-2.589	79.485	13.473	19.289
8857	229	34	31	C	S	36.309	2.390	1.806	2.666	-2.281	-1.714	-2.531	79.484	19.291	13.472
8858	229	34	31	C	S	38.213	5.011	-2.675	0.157	-4.554	2.666	-0.157	76.485	13.826	19.017
8859	229	34	31	C	S	42.304	1.029	4.660	4.071	-1.008	-4.309	-3.765	76.364	19.034	13.816
8860	229	34	31	C	S	77.696	1.029	-4.660	4.071	-1.008	4.309	-3.765	76.364	19.034	13.816
8861	229	34	31	C	S	81.787	5.011	2.675	0.157	-4.554	-2.666	-0.157	76.485	13.826	19.017
8862	229	34	31	C	S	85.694	2.326	1.783	2.731	-2.223	-1.691	-2.589	79.485	13.473	19.289
8863	229	34	31	C	S	96.309	2.390	1.806	2.666	-2.281	-1.714	-2.531	79.484	19.291	13.472
8864	229	34	31	C	S	98.213	5.011	-2.675	0.157	-4.554	2.666	-0.157	76.485	13.826	19.017
8865	229	34	31	C	S	102.304	1.029	4.660	4.071	-1.008	-4.309	-3.765	76.364	19.034	13.816
8866	229	34	31	C	W	17.696	1.029	-4.660	4.071	-1.008	4.309	-3.765	76.364	19.034	13.816
8867	229	34	31	C	W	21.787	5.011	2.675	0.157	-4.554	-2.666	-0.157	76.485	13.826	19.017
8868	229	34	31	C	W	25.693	2.326	1.783	2.731	-2.223	-1.691	-2.589	79.485	13.473	19.289
8869	229	34	31	C	W	36.309	2.390	1.806	2.666	-2.281	-1.714	-2.531	79.484	19.291	13.472
8870	229	34	31	C	W	38.213	5.011	-2.675	0.157	-4.554	2.666	-0.157	76.485	13.826	19.017
8871	229	34	31	C	W	42.304	1.029	4.660	4.071	-1.008	-4.309	-3.765	76.364	19.034	13.816
8872	229	34	31	C	W	77.696	1.029	-4.660	4.071	-1.008	4.309	-3.765	76.364	19.034	13.816
8873	229	34	31	C	W	81.787	5.011	2.675	0.157	-4.554	-2.666	-0.157	76.485	13.826	19.017
8874	229	34	31	C	W	85.694	2.326	1.783	2.731	-2.223	-1.691	-2.589	79.485	13.473	19.289

BL number	Atoms	γ-PC unit cells	WS_2 unit cells	γ-PC origin atom	WS_2 origin atom	Twist-angle (°)	γ-PC strain 1 (%)	γ-PC strain 2 (%)	γ-PC strain 3 (%)	WS_2 strain 1 (%)	WS_2 strain 2 (%)	WS_2 strain 3 (%)	γ (°)	|**a**|(Å)	|**b**|(Å)
8875	229	34	31	C	W	96.309	2.390	1.806	2.666	-2.281	-1.714	-2.531	79.484	19.291	13.472
8876	229	34	31	C	W	98.213	5.011	-2.675	0.157	-4.554	2.666	-0.157	76.485	13.826	19.017
8877	229	34	31	C	W	102.304	1.029	4.660	4.071	-1.008	-4.309	-3.765	76.364	19.034	13.816
8878	229	34	31	P	S	25.693	2.326	1.783	2.731	-2.223	-1.691	-2.589	79.485	13.473	19.289
8879	229	34	31	P	S	36.309	2.390	1.806	2.666	-2.281	-1.714	-2.531	79.484	19.291	13.472
8880	229	34	31	P	S	38.213	5.011	-2.675	0.157	-4.554	2.666	-0.157	76.485	13.826	19.017
8881	229	34	31	P	S	42.304	1.029	4.660	4.071	-1.008	-4.309	-3.765	76.364	19.034	13.816
8882	229	34	31	P	S	77.696	1.029	-4.660	4.071	-1.008	4.309	-3.765	76.364	19.034	13.816
8883	229	34	31	P	S	81.787	5.011	2.675	0.157	-4.554	-2.666	-0.157	76.485	13.826	19.017
8884	229	34	31	P	S	85.694	2.326	1.783	2.731	-2.223	-1.691	-2.589	79.485	13.473	19.289
8885	229	34	31	P	S	96.309	2.390	1.806	2.666	-2.281	-1.714	-2.531	79.484	19.291	13.472
8886	229	34	31	P	W	17.696	1.029	-4.660	4.071	-1.008	4.309	-3.765	76.364	19.034	13.816
8887	229	34	31	P	W	21.787	5.011	2.675	0.157	-4.554	-2.666	-0.157	76.485	13.826	19.017
8888	229	34	31	P	W	25.693	2.326	1.783	2.731	-2.223	-1.691	-2.589	79.485	13.473	19.289
8889	229	34	31	P	W	36.309	2.390	1.806	2.666	-2.281	-1.714	-2.531	79.484	19.291	13.472
8890	229	34	31	P	W	85.694	2.326	1.783	2.731	-2.223	-1.691	-2.589	79.485	13.473	19.289
8891	229	34	31	P	W	96.309	2.390	1.806	2.666	-2.281	-1.714	-2.531	79.484	19.291	13.472
8892	229	34	31	P	W	98.213	5.011	-2.675	0.157	-4.554	2.666	-0.157	76.485	13.826	19.017
8893	229	34	31	P	W	102.304	1.029	4.660	4.071	-1.008	-4.309	-3.765	76.364	19.034	13.816
8894	229	37	27	C	S	4.715	-3.007	0.000	-3.007	3.200	0.000	3.200	60.000	16.951	16.951
8895	229	37	27	C	S	55.285	-3.007	0.000	-3.007	3.200	0.000	3.200	60.000	16.951	16.951
8896	229	37	27	C	S	64.715	-3.007	0.000	-3.007	3.200	0.000	3.200	60.000	16.951	16.951
8897	229	37	27	C	S	115.285	-3.007	0.000	-3.007	3.200	0.000	3.200	60.000	16.951	16.951
8898	229	37	27	C	W	4.715	-3.007	0.000	-3.007	3.200	0.000	3.200	60.000	16.951	16.951
8899	229	37	27	C	W	55.285	-3.007	0.000	-3.007	3.200	0.000	3.200	60.000	16.951	16.951
8900	229	37	27	C	W	64.715	-3.007	0.000	-3.007	3.200	0.000	3.200	60.000	16.951	16.951
8901	229	37	27	C	W	115.285	-3.007	0.000	-3.007	3.200	0.000	3.200	60.000	16.951	16.951
8902	230	35	30	C	S	8.513	2.390	-1.234	-0.489	-2.281	1.247	0.494	69.242	14.421	18.906
8903	230	35	30	C	S	10.158	2.281	1.627	-0.386	-2.182	-1.639	0.389	78.478	16.362	15.902
8904	230	35	30	C	S	10.893	0.418	2.958	1.447	-0.415	-2.875	-1.407	69.241	14.426	18.897
8905	230	35	30	C	S	16.102	2.853	3.817	-0.922	-2.699	-3.889	0.940	73.543	18.455	14.407
8906	230	35	30	C	S	23.413	-2.043	-2.339	4.087	2.130	2.162	-3.778	84.284	14.072	18.220
8907	230	35	30	C	S	25.285	4.262	1.003	-2.197	-3.928	-1.049	2.298	84.392	18.222	14.070
8908	230	35	30	C	S	34.715	4.262	-1.003	-2.197	-3.928	1.049	2.298	84.392	18.222	14.070
8909	230	35	30	C	S	36.587	-2.043	2.339	4.087	2.130	-2.162	-3.778	84.284	14.072	18.220
8910	230	35	30	C	S	43.898	2.853	-3.817	-0.922	-2.699	3.889	0.940	73.543	18.455	14.407
8911	230	35	30	C	S	49.107	0.418	-2.958	1.447	-0.415	2.875	-1.406	69.241	14.426	18.897
8912	230	35	30	C	S	49.842	2.281	-1.627	-0.386	-2.182	1.639	0.389	78.478	16.362	15.902
8913	230	35	30	C	S	51.052	-0.599	0.492	2.507	0.606	-0.468	-2.387	78.517	15.901	16.361
8914	230	35	30	C	S	52.411	0.334	2.896	1.533	-0.332	-2.810	-1.487	69.243	18.904	14.420
8915	230	35	30	C	S	68.513	2.390	-1.234	-0.489	-2.281	1.247	0.494	69.242	14.421	18.906
8916	230	35	30	C	S	68.948	-0.599	-0.492	2.506	0.606	0.468	-2.387	78.517	15.901	16.361
8917	230	35	30	C	S	70.158	2.281	1.627	-0.386	-2.182	-1.639	0.389	78.478	16.362	15.902
8918	230	35	30	C	S	70.893	0.418	-5.324	1.447	-0.415	5.174	-1.406	73.437	14.426	18.435
8919	230	35	30	C	S	76.102	2.853	3.817	-0.922	-2.699	-3.889	0.940	73.543	18.455	14.407
8920	230	35	30	C	S	83.413	-2.043	-2.339	4.087	2.130	2.162	-3.778	84.284	14.072	18.220
8921	230	35	30	C	S	85.285	4.262	1.003	-2.197	-3.928	-1.049	2.298	84.392	18.222	14.070
8922	230	35	30	C	S	94.715	4.262	-1.003	-2.197	-3.928	1.049	2.298	84.392	18.222	14.070
8923	230	35	30	C	S	96.587	-2.043	2.339	4.087	2.130	-2.162	-3.778	84.284	14.072	18.220
8924	230	35	30	C	S	103.898	2.853	-3.817	-0.922	-2.699	3.889	0.940	73.543	18.455	14.407
8925	230	35	30	C	S	109.107	0.418	-2.958	1.447	-0.415	2.875	-1.406	69.241	14.426	18.897

BL number	Atoms	γ-PC unit cells	WS_2 unit cells	γ-PC origin atom	WS_2 origin atom	Twist-angle (°)	γ-PC strain 1 (%)	γ-PC strain 2 (%)	γ-PC strain 3 (%)	WS_2 strain 1 (%)	WS_2 strain 2 (%)	WS_2 strain 3 (%)	γ (°)	|**a**|(Å)	|**b**|(Å)
8926	230	35	30	C	S	111.052	-0.599	0.492	2.507	0.606	-0.468	-2.387	78.517	15.901	16.361
8927	230	35	30	C	S	112.411	0.334	2.896	1.533	-0.332	-2.810	-1.487	69.243	18.904	14.420
8928	230	35	30	C	W	8.513	2.390	-1.234	-0.489	-2.281	1.247	0.494	69.242	14.421	18.906
8929	230	35	30	C	W	8.948	-0.599	-0.492	2.506	0.606	0.468	-2.387	78.517	15.901	16.361
8930	230	35	30	C	W	10.158	2.281	1.627	-0.386	-2.182	-1.639	0.389	78.478	16.362	15.902
8931	230	35	30	C	W	10.893	0.418	2.958	1.447	-0.415	-2.875	-1.407	69.241	14.426	18.897
8932	230	35	30	C	W	16.102	2.853	3.817	-0.922	-2.699	-3.889	0.940	73.543	18.455	14.407
8933	230	35	30	C	W	23.413	-2.043	-2.339	4.087	2.130	2.162	-3.778	84.284	14.072	18.220
8934	230	35	30	C	W	25.285	4.262	1.003	-2.197	-3.928	-1.049	2.298	84.392	18.222	14.070
8935	230	35	30	C	W	34.715	4.262	-1.003	-2.197	-3.928	1.049	2.298	84.392	18.222	14.070
8936	230	35	30	C	W	36.587	-2.043	2.339	4.087	2.130	-2.162	-3.778	84.284	14.072	18.220
8937	230	35	30	C	W	43.898	2.853	-3.817	-0.922	-2.699	3.889	0.940	73.543	18.455	14.407
8938	230	35	30	C	W	49.107	0.418	-2.958	1.447	-0.415	2.875	-1.406	69.241	14.426	18.897
8939	230	35	30	C	W	51.052	-0.599	0.492	2.507	0.606	-0.468	-2.387	78.517	15.901	16.361
8940	230	35	30	C	W	52.411	0.334	2.896	1.533	-0.332	-2.810	-1.487	69.243	18.904	14.420
8941	230	35	30	C	W	68.513	2.390	-1.234	-0.489	-2.281	1.247	0.494	69.242	14.421	18.906
8942	230	35	30	C	W	70.158	2.281	1.627	-0.386	-2.182	-1.639	0.389	78.478	16.362	15.902
8943	230	35	30	C	W	70.893	0.418	-5.324	1.447	-0.415	5.174	-1.406	73.437	14.426	18.435
8944	230	35	30	C	W	76.102	2.853	3.817	-0.922	-2.699	-3.889	0.940	73.543	18.455	14.407
8945	230	35	30	C	W	83.413	-2.043	-2.339	4.087	2.130	2.162	-3.778	84.284	14.072	18.220
8946	230	35	30	C	W	85.285	4.262	1.003	-2.197	-3.928	-1.049	2.298	84.392	18.222	14.070
8947	230	35	30	C	W	94.715	4.262	-1.003	-2.197	-3.928	1.049	2.298	84.392	18.222	14.070
8948	230	35	30	C	W	96.587	-2.043	2.339	4.087	2.130	-2.162	-3.778	84.284	14.072	18.220
8949	230	35	30	C	W	103.898	2.853	-3.817	-0.922	-2.699	3.889	0.940	73.543	18.455	14.407
8950	230	35	30	C	W	109.107	0.418	-2.958	1.447	-0.415	2.875	-1.406	69.241	14.426	18.897
8951	230	35	30	C	W	109.842	2.281	-1.627	-0.386	-2.182	1.639	0.389	78.478	16.362	15.902
8952	230	35	30	C	W	111.052	-0.599	0.492	2.507	0.606	-0.468	-2.387	78.517	15.901	16.361
8953	230	35	30	C	W	112.411	0.334	2.896	1.533	-0.332	-2.810	-1.487	69.243	18.904	14.420
8954	230	35	30	P	S	8.948	-0.599	-0.492	2.506	0.606	0.468	-2.387	78.517	15.901	16.361
8955	230	35	30	P	S	16.102	2.853	3.817	-0.922	-2.699	-3.889	0.940	73.543	18.455	14.407
8956	230	35	30	P	S	23.413	-2.043	-2.339	4.087	2.130	2.162	-3.778	84.284	14.072	18.220
8957	230	35	30	P	S	25.285	4.262	1.003	-2.197	-3.928	-1.049	2.298	84.392	18.222	14.070
8958	230	35	30	P	S	34.715	4.262	-1.003	-2.197	-3.928	1.049	2.298	84.392	18.222	14.070
8959	230	35	30	P	S	36.587	-2.043	2.339	4.087	2.130	-2.162	-3.778	84.284	14.072	18.220
8960	230	35	30	P	S	49.107	0.418	-2.958	1.447	-0.415	2.875	-1.406	69.241	14.426	18.897
8961	230	35	30	P	S	52.411	0.334	2.896	1.533	-0.332	-2.810	-1.487	69.243	18.904	14.420
8962	230	35	30	P	S	68.513	2.390	-1.234	-0.489	-2.281	1.247	0.494	69.242	14.421	18.906
8963	230	35	30	P	S	70.893	0.418	2.958	1.447	-0.415	-2.875	-1.407	69.241	14.426	18.897
8964	230	35	30	P	S	83.413	-2.043	-2.339	4.087	2.130	2.162	-3.778	84.284	14.072	18.220
8965	230	35	30	P	S	85.285	4.262	1.003	-2.197	-3.928	-1.049	2.298	84.392	18.222	14.070
8966	230	35	30	P	S	94.715	4.262	-1.003	-2.197	-3.928	1.049	2.298	84.392	18.222	14.070
8967	230	35	30	P	S	96.587	-2.043	2.339	4.087	2.130	-2.162	-3.778	84.284	14.072	18.220
8968	230	35	30	P	S	109.842	2.281	-1.627	-0.386	-2.182	1.639	0.389	78.478	16.362	15.902
8969	230	35	30	P	W	8.513	2.390	-1.234	-0.489	-2.281	1.247	0.494	69.242	14.421	18.906
8970	230	35	30	P	W	10.893	0.418	2.958	1.447	-0.415	-2.875	-1.407	69.241	14.426	18.897
8971	230	35	30	P	W	23.413	-2.043	-2.339	4.087	2.130	2.162	-3.778	84.284	14.072	18.220
8972	230	35	30	P	W	25.285	4.262	1.003	-2.197	-3.928	-1.049	2.298	84.392	18.222	14.070
8973	230	35	30	P	W	34.715	4.262	-1.003	-2.197	-3.928	1.049	2.298	84.392	18.222	14.070
8974	230	35	30	P	W	36.587	-2.043	2.339	4.087	2.130	-2.162	-3.778	84.284	14.072	18.220
8975	230	35	30	P	W	49.842	2.281	-1.627	-0.386	-2.182	1.639	0.389	78.478	16.362	15.902
8976	230	35	30	P	W	68.948	-0.599	-0.492	2.506	0.606	0.468	-2.387	78.517	15.901	16.361

BL number	Atoms	γ-PC unit cells	WS_2 unit cells	γ-PC origin atom	WS_2 origin atom	Twist-angle (°)	γ-PC strain 1 (%)	γ-PC strain 2 (%)	γ-PC strain 3 (%)	WS_2 strain 1 (%)	WS_2 strain 2 (%)	WS_2 strain 3 (%)	γ (°)	$\lvert\mathbf{a}\rvert$(Å)	$\lvert\mathbf{b}\rvert$(Å)
8977	230	35	30	P	W	76.102	2.853	3.817	-0.922	-2.699	-3.889	0.940	73.543	18.455	14.407
8978	230	35	30	P	W	83.413	-2.043	-2.339	4.087	2.130	2.162	-3.778	84.284	14.072	18.220
8979	230	35	30	P	W	85.285	4.262	1.003	-2.197	-3.928	-1.049	2.298	84.392	18.222	14.070
8980	230	35	30	P	W	94.715	4.262	-1.003	-2.197	-3.928	1.049	2.298	84.392	18.222	14.070
8981	230	35	30	P	W	96.587	-2.043	2.339	4.087	2.130	-2.162	-3.778	84.284	14.072	18.220
8982	230	35	30	P	W	109.107	0.418	-2.958	1.447	-0.415	2.875	-1.406	69.241	14.426	18.897
8983	230	35	30	P	W	112.411	0.334	2.896	1.533	-0.332	-2.810	-1.487	69.243	18.904	14.420
8984	231	36	29	C	S	6.587	-3.853	-4.748	2.826	4.175	4.494	-2.675	72.459	14.354	18.591
8985	231	36	29	C	S	8.948	1.048	1.255	-2.245	-1.026	-1.314	2.351	79.458	17.420	14.845
8986	231	36	29	C	S	9.183	3.582	-0.067	-4.504	-3.342	0.074	4.949	72.651	18.586	14.351
8987	231	36	29	C	S	19.107	-1.485	-5.078	0.248	1.530	5.053	-0.247	76.883	14.708	17.745
8988	231	36	29	C	S	25.050	-0.955	5.303	-0.295	0.973	-5.335	0.297	76.853	17.772	14.686
8989	231	36	29	C	S	25.767	0.353	-4.231	-1.587	-0.351	4.370	1.639	77.666	17.539	14.835
8990	231	36	29	C	S	30.000	-2.359	-3.132	1.170	2.476	3.060	-1.143	77.773	14.845	17.524
8991	231	36	29	C	S	34.233	0.353	4.231	-1.587	-0.351	-4.370	1.639	77.666	17.539	14.835
8992	231	36	29	C	S	34.950	-0.955	-5.303	-0.295	0.973	5.335	0.297	76.853	17.772	14.686
8993	231	36	29	C	S	40.893	-1.485	5.078	0.248	1.530	-5.053	-0.247	76.883	14.708	17.745
8994	231	36	29	C	S	50.817	3.582	0.067	-4.504	-3.342	-0.074	4.949	72.651	18.586	14.351
8995	231	36	29	C	S	53.413	-3.853	4.748	2.826	4.175	-4.494	-2.675	72.459	14.354	18.591
8996	231	36	29	C	S	66.587	-3.853	-4.748	2.826	4.175	4.494	-2.675	72.459	14.354	18.591
8997	231	36	29	C	S	69.183	3.582	-0.067	-4.504	-3.342	0.074	4.949	72.651	18.586	14.351
8998	231	36	29	C	S	79.107	-1.485	-5.078	0.248	1.530	5.053	-0.247	76.883	14.708	17.745
8999	231	36	29	C	S	85.050	-0.955	5.303	-0.295	0.973	-5.335	0.297	76.853	17.772	14.686
9000	231	36	29	C	S	85.767	0.353	-4.231	-1.587	-0.351	4.370	1.639	77.666	17.539	14.835
9001	231	36	29	C	S	90.000	-2.359	3.132	1.170	2.476	-3.060	-1.143	77.773	14.845	17.524
9002	231	36	29	C	S	94.233	0.353	4.231	-1.587	-0.351	-4.370	1.639	77.666	17.539	14.835
9003	231	36	29	C	S	94.950	-0.955	-5.303	-0.295	0.973	5.335	0.297	76.853	17.772	14.686
9004	231	36	29	C	S	100.893	-1.485	5.078	0.248	1.530	-5.053	-0.247	76.883	14.708	17.745
9005	231	36	29	C	S	110.817	3.582	0.067	-4.504	-3.342	-0.074	4.949	72.651	18.586	14.351
9006	231	36	29	C	S	111.052	1.048	-1.255	-2.245	-1.027	1.314	2.351	79.458	17.420	14.845
9007	231	36	29	C	S	113.413	-3.853	4.748	2.826	4.175	-4.494	-2.675	72.459	14.354	18.591
9008	231	36	29	C	W	6.587	-3.853	-4.748	2.826	4.175	4.494	-2.675	72.459	14.354	18.591
9009	231	36	29	C	W	9.183	3.582	-0.067	-4.504	-3.342	0.074	4.949	72.651	18.586	14.351
9010	231	36	29	C	W	25.767	0.353	-4.231	-1.587	-0.351	4.370	1.639	77.666	17.539	14.835
9011	231	36	29	C	W	30.000	-2.359	-3.132	1.170	2.476	3.060	-1.143	77.773	14.845	17.524
9012	231	36	29	C	W	34.233	0.353	4.231	-1.587	-0.351	-4.370	1.639	77.666	17.539	14.835
9013	231	36	29	C	W	50.817	3.582	0.067	-4.504	-3.342	-0.074	4.949	72.651	18.586	14.351
9014	231	36	29	C	W	51.052	1.048	-1.255	-2.245	-1.027	1.314	2.351	79.458	17.420	14.845
9015	231	36	29	C	W	53.413	-3.853	4.748	2.826	4.175	-4.494	-2.675	72.459	14.354	18.591
9016	231	36	29	C	W	66.587	-3.853	-4.748	2.826	4.175	4.494	-2.675	72.459	14.354	18.591
9017	231	36	29	C	W	68.948	1.048	1.255	-2.245	-1.026	-1.314	2.351	79.458	17.420	14.845
9018	231	36	29	C	W	69.183	3.582	-0.067	-4.504	-3.342	0.074	4.949	72.651	18.586	14.351
9019	231	36	29	C	W	85.767	0.353	-4.231	-1.587	-0.351	4.370	1.639	77.666	17.539	14.835
9020	231	36	29	C	W	90.000	-2.359	3.132	1.170	2.476	-3.060	-1.143	77.773	14.845	17.524
9021	231	36	29	C	W	94.233	0.353	4.231	-1.587	-0.351	-4.370	1.639	77.666	17.539	14.835
9022	231	36	29	C	W	110.817	3.582	0.067	-4.504	-3.342	-0.074	4.949	72.651	18.586	14.351
9023	231	36	29	C	W	113.413	-3.853	4.748	2.826	4.175	-4.494	-2.675	72.459	14.354	18.591
9024	231	36	29	P	S	19.107	-1.485	-5.078	0.248	1.530	5.053	-0.247	76.883	14.708	17.745
9025	231	36	29	P	S	25.050	-0.955	5.303	-0.295	0.973	-5.335	0.297	76.853	17.772	14.686
9026	231	36	29	P	S	25.767	0.353	-4.231	-1.587	-0.351	4.370	1.639	77.666	17.539	14.835
9027	231	36	29	P	S	30.000	-2.359	-3.132	1.170	2.476	3.060	-1.143	77.773	14.845	17.524

BL number	Atoms	γ-PC unit cells	WS_2 unit cells	γ-PC origin atom	WS_2 origin atom	Twist-angle (°)	γ-PC strain 1 (%)	γ-PC strain 2 (%)	γ-PC strain 3 (%)	WS_2 strain 1 (%)	WS_2 strain 2 (%)	WS_2 strain 3 (%)	γ (°)	|**a**|(Å)	|**b**|(Å)
9028	231	36	29	P	S	34.233	0.353	4.231	-1.587	-0.351	-4.370	1.639	77.666	17.539	14.835
9029	231	36	29	P	S	34.950	-0.955	-5.303	-0.295	0.973	5.335	0.297	76.853	17.772	14.686
9030	231	36	29	P	S	40.893	-1.485	5.078	0.248	1.530	-5.053	-0.247	76.883	14.708	17.745
9031	231	36	29	P	S	50.817	3.582	0.067	-4.504	-3.342	-0.074	4.949	72.651	18.586	14.351
9032	231	36	29	P	S	51.052	1.048	-1.255	-2.245	-1.027	1.314	2.351	79.458	17.420	14.845
9033	231	36	29	P	S	53.413	-3.853	4.748	2.826	4.175	-4.494	-2.675	72.459	14.354	18.591
9034	231	36	29	P	S	66.587	-3.853	-4.748	2.826	4.175	4.494	-2.675	72.459	14.354	18.591
9035	231	36	29	P	S	68.948	1.048	1.255	-2.245	-1.026	-1.314	2.351	79.458	17.420	14.845
9036	231	36	29	P	S	69.183	3.582	-0.067	-4.504	-3.342	0.074	4.949	72.651	18.586	14.351
9037	231	36	29	P	S	79.107	-1.485	-5.078	0.248	1.530	5.053	-0.247	76.883	14.708	17.745
9038	231	36	29	P	S	85.050	-0.955	5.303	-0.295	0.973	-5.335	0.297	76.853	17.772	14.686
9039	231	36	29	P	S	85.767	0.353	-4.231	-1.587	-0.351	4.370	1.639	77.666	17.539	14.835
9040	231	36	29	P	S	90.000	-2.359	3.132	1.170	2.476	-3.060	-1.143	77.773	14.845	17.524
9041	231	36	29	P	S	94.233	0.353	4.231	-1.587	-0.351	-4.370	1.639	77.666	17.539	14.835
9042	231	36	29	P	S	94.950	-0.955	-5.303	-0.295	0.973	5.335	0.297	76.853	17.772	14.686
9043	231	36	29	P	S	100.893	-1.485	5.078	0.248	1.530	-5.053	-0.247	76.883	14.708	17.745
9044	231	36	29	P	W	6.587	-3.853	-4.748	2.826	4.175	4.494	-2.675	72.459	14.354	18.591
9045	231	36	29	P	W	8.948	1.048	1.255	-2.245	-1.026	-1.314	2.351	79.458	17.420	14.845
9046	231	36	29	P	W	9.183	3.582	-0.067	-4.504	-3.342	0.074	4.949	72.651	18.586	14.351
9047	231	36	29	P	W	25.767	0.353	-4.231	-1.587	-0.351	4.370	1.639	77.666	17.539	14.835
9048	231	36	29	P	W	30.000	-2.359	-3.132	1.170	2.476	3.060	-1.143	77.773	14.845	17.524
9049	231	36	29	P	W	34.233	0.353	4.231	-1.587	-0.351	-4.370	1.639	77.666	17.539	14.835
9050	231	36	29	P	W	85.767	0.353	-4.231	-1.587	-0.351	4.370	1.639	77.666	17.539	14.835
9051	231	36	29	P	W	90.000	-2.359	3.132	1.170	2.476	-3.060	-1.143	77.773	14.845	17.524
9052	231	36	29	P	W	94.233	0.353	4.231	-1.587	-0.351	-4.370	1.639	77.666	17.539	14.835
9053	231	36	29	P	W	110.817	3.582	0.067	-4.504	-3.342	-0.074	4.949	72.651	18.586	14.351
9054	231	36	29	P	W	111.052	1.048	-1.255	-2.245	-1.027	1.314	2.351	79.458	17.420	14.845
9055	231	36	29	P	W	113.413	-3.853	4.748	2.826	4.175	-4.494	-2.675	72.459	14.354	18.591
9056	232	37	28	C	S	6.178	-2.145	0.000	-2.145	2.241	0.000	2.241	60.000	17.102	17.102
9057	232	37	28	C	S	15.608	-2.145	0.000	-2.145	2.241	0.000	2.241	60.000	17.102	17.102
9058	232	37	28	C	S	44.392	-2.145	0.000	-2.145	2.241	0.000	2.241	60.000	17.102	17.102
9059	232	37	28	C	S	53.822	-2.145	0.000	-2.145	2.241	0.000	2.241	60.000	17.102	17.102
9060	232	37	28	C	S	66.178	-2.145	0.000	-2.145	2.241	0.000	2.241	60.000	17.102	17.102
9061	232	37	28	C	S	75.609	-2.145	0.000	-2.145	2.241	0.000	2.241	60.000	17.102	17.102
9062	232	37	28	C	S	104.392	-2.145	0.000	-2.145	2.241	0.000	2.241	60.000	17.102	17.102
9063	232	37	28	C	S	113.822	-2.145	0.000	-2.145	2.241	0.000	2.241	60.000	17.102	17.102
9064	232	37	28	C	W	6.178	-2.145	0.000	-2.145	2.241	0.000	2.241	60.000	17.102	17.102
9065	232	37	28	C	W	15.608	-2.145	0.000	-2.145	2.241	0.000	2.241	60.000	17.102	17.102
9066	232	37	28	C	W	44.392	-2.145	0.000	-2.145	2.241	0.000	2.241	60.000	17.102	17.102
9067	232	37	28	C	W	53.822	-2.145	0.000	-2.145	2.241	0.000	2.241	60.000	17.102	17.102
9068	232	37	28	C	W	66.178	-2.145	0.000	-2.145	2.241	0.000	2.241	60.000	17.102	17.102
9069	232	37	28	C	W	75.609	-2.145	0.000	-2.145	2.241	0.000	2.241	60.000	17.102	17.102
9070	232	37	28	C	W	113.822	-2.145	0.000	-2.145	2.241	0.000	2.241	60.000	17.102	17.102
9071	232	37	28	P	S	15.608	-2.145	0.000	-2.145	2.241	0.000	2.241	60.000	17.102	17.102
9072	232	37	28	P	S	53.822	-2.145	0.000	-2.145	2.241	0.000	2.241	60.000	17.102	17.102
9073	232	37	28	P	S	66.178	-2.145	0.000	-2.145	2.241	0.000	2.241	60.000	17.102	17.102
9074	232	37	28	P	S	104.392	-2.145	0.000	-2.145	2.241	0.000	2.241	60.000	17.102	17.102
9075	232	37	28	P	W	6.178	-2.145	0.000	-2.145	2.241	0.000	2.241	60.000	17.102	17.102
9076	232	37	28	P	W	44.392	-2.145	0.000	-2.145	2.241	0.000	2.241	60.000	17.102	17.102
9077	232	37	28	P	W	75.609	-2.145	0.000	-2.145	2.241	0.000	2.241	60.000	17.102	17.102
9078	232	37	28	P	W	113.822	-2.145	0.000	-2.145	2.241	0.000	2.241	60.000	17.102	17.102

BL number	Atoms	γ-PC unit cells	WS_2 unit cells	γ-PC origin atom	WS_2 origin atom	Twist-angle (°)	γ-PC strain 1 (%)	γ-PC strain 2 (%)	γ-PC strain 3 (%)	WS_2 strain 1 (%)	WS_2 strain 2 (%)	WS_2 strain 3 (%)	γ (°)	|**a**|(Å)	|**b**|(Å)
9079	233	35	31	C	S	9.183	3.582	-0.693	0.023	-3.342	0.692	-0.023	75.256	18.586	14.424
9080	233	35	31	C	S	10.893	0.418	2.366	3.162	-0.415	-2.226	-2.974	75.222	14.426	18.583
9081	233	35	31	C	S	27.796	5.011	5.197	-1.276	-4.554	-5.333	1.309	78.110	18.842	14.069
9082	233	35	31	C	S	32.204	5.011	-5.197	-1.276	-4.554	5.333	1.309	78.110	18.842	14.069
9083	233	35	31	C	S	49.107	0.418	-2.366	3.162	-0.415	2.226	-2.974	75.222	14.426	18.583
9084	233	35	31	C	S	50.817	3.582	0.693	0.023	-3.342	-0.692	-0.023	75.256	18.586	14.424
9085	233	35	31	C	S	69.183	3.582	-0.693	0.023	-3.342	0.692	-0.023	75.256	18.586	14.424
9086	233	35	31	C	S	70.893	0.418	2.366	3.162	-0.415	-2.226	-2.974	75.222	14.426	18.583
9087	233	35	31	C	S	87.796	5.011	5.197	-1.276	-4.554	-5.333	1.309	78.110	18.842	14.069
9088	233	35	31	C	S	92.204	5.011	-5.197	-1.276	-4.554	5.333	1.309	78.110	18.842	14.069
9089	233	35	31	C	S	109.107	0.418	-2.366	3.162	-0.415	2.226	-2.974	75.222	14.426	18.583
9090	233	35	31	C	S	110.817	3.582	0.693	0.023	-3.342	-0.692	-0.023	75.256	18.586	14.424
9091	233	35	31	C	W	9.183	3.582	-0.693	0.023	-3.342	0.692	-0.023	75.256	18.586	14.424
9092	233	35	31	C	W	10.893	0.418	2.366	3.162	-0.415	-2.226	-2.974	75.222	14.426	18.583
9093	233	35	31	C	W	27.796	5.011	5.197	-1.276	-4.554	-5.333	1.309	78.110	18.842	14.069
9094	233	35	31	C	W	32.204	5.011	-5.197	-1.276	-4.554	5.333	1.309	78.110	18.842	14.069
9095	233	35	31	C	W	49.107	0.418	-2.366	3.162	-0.415	2.226	-2.974	75.222	14.426	18.583
9096	233	35	31	C	W	50.817	3.582	0.693	0.023	-3.342	-0.692	-0.023	75.256	18.586	14.424
9097	233	35	31	C	W	69.183	3.582	-0.693	0.023	-3.342	0.692	-0.023	75.256	18.586	14.424
9098	233	35	31	C	W	70.893	0.418	2.366	3.162	-0.415	-2.226	-2.974	75.222	14.426	18.583
9099	233	35	31	C	W	87.796	5.011	5.197	-1.276	-4.554	-5.333	1.309	78.110	18.842	14.069
9100	233	35	31	C	W	92.204	5.011	-5.197	-1.276	-4.554	5.333	1.309	78.110	18.842	14.069
9101	233	35	31	C	W	109.107	0.418	-2.366	3.162	-0.415	2.226	-2.974	75.222	14.426	18.583
9102	233	35	31	C	W	110.817	3.582	0.693	0.023	-3.342	-0.692	-0.023	75.256	18.586	14.424
9103	233	35	31	P	S	9.183	3.582	-0.693	0.023	-3.342	0.692	-0.023	75.256	18.586	14.424
9104	233	35	31	P	S	10.893	0.418	2.366	3.162	-0.415	-2.226	-2.974	75.222	14.426	18.583
9105	233	35	31	P	S	27.796	5.011	5.197	-1.276	-4.554	-5.333	1.309	78.110	18.842	14.069
9106	233	35	31	P	S	49.107	0.418	-2.366	3.162	-0.415	2.226	-2.974	75.222	14.426	18.583
9107	233	35	31	P	S	50.817	3.582	0.693	0.023	-3.342	-0.692	-0.023	75.256	18.586	14.424
9108	233	35	31	P	S	69.183	3.582	-0.693	0.023	-3.342	0.692	-0.023	75.256	18.586	14.424
9109	233	35	31	P	S	70.893	0.418	2.366	3.162	-0.415	-2.226	-2.974	75.222	14.426	18.583
9110	233	35	31	P	S	92.204	5.011	-5.197	-1.276	-4.554	5.333	1.309	78.110	18.842	14.069
9111	233	35	31	P	S	109.107	0.418	-2.366	3.162	-0.415	2.226	-2.974	75.222	14.426	18.583
9112	233	35	31	P	S	110.817	3.582	0.693	0.023	-3.342	-0.692	-0.023	75.256	18.586	14.424
9113	233	35	31	P	W	9.183	3.582	-0.693	0.023	-3.342	0.692	-0.023	75.256	18.586	14.424
9114	233	35	31	P	W	10.893	0.418	2.366	3.162	-0.415	-2.226	-2.974	75.222	14.426	18.583
9115	233	35	31	P	W	32.204	5.011	-5.197	-1.276	-4.554	5.333	1.309	78.110	18.842	14.069
9116	233	35	31	P	W	49.107	0.418	-2.366	3.162	-0.415	2.226	-2.974	75.222	14.426	18.583
9117	233	35	31	P	W	50.817	3.582	0.693	0.023	-3.342	-0.692	-0.023	75.256	18.586	14.424
9118	233	35	31	P	W	69.183	3.582	-0.693	0.023	-3.342	0.692	-0.023	75.256	18.586	14.424
9119	233	35	31	P	W	70.893	0.418	2.366	3.162	-0.415	-2.226	-2.974	75.222	14.426	18.583
9120	233	35	31	P	W	87.796	5.011	5.197	-1.276	-4.554	-5.333	1.309	78.110	18.842	14.069
9121	233	35	31	P	W	109.107	0.418	-2.366	3.162	-0.415	2.226	-2.974	75.222	14.426	18.583
9122	233	35	31	P	W	110.817	3.582	0.693	0.023	-3.342	-0.692	-0.023	75.256	18.586	14.424
9123	233	38	27	C	S	1.359	-3.292	-2.915	-3.966	3.523	3.166	4.307	72.973	18.221	14.482
9124	233	38	27	C	S	1.945	-4.723	-1.965	-2.510	5.216	2.068	2.643	77.733	15.242	16.943
9125	233	38	27	C	S	4.307	-4.685	2.058	-2.550	5.169	-2.168	2.688	72.995	14.491	18.209
9126	233	38	27	C	S	4.715	-3.007	2.702	-4.244	3.200	-2.952	4.638	77.701	16.951	15.234
9127	233	38	27	C	S	8.213	-2.359	-4.451	-4.866	2.476	4.930	5.391	82.011	14.845	17.166
9128	233	38	27	C	S	13.898	-4.228	5.093	-3.024	4.619	-5.421	3.218	81.888	17.184	14.831
9129	233	38	27	C	S	46.102	-4.228	-5.093	-3.024	4.619	5.421	3.218	81.887	17.184	14.831

BL number	Atoms	γ-PC unit cells	WS_2 unit cells	γ-PC origin atom	WS_2 origin atom	Twist-angle (°)	γ-PC strain 1 (%)	γ-PC strain 2 (%)	γ-PC strain 3 (%)	WS_2 strain 1 (%)	WS_2 strain 2 (%)	WS_2 strain 3 (%)	γ (°)	|**a**|(Å)	|**b**|(Å)
9130	233	38	27	C	S	51.787	-2.359	4.451	-4.866	2.476	-4.930	5.391	82.011	14.845	17.166
9131	233	38	27	C	S	55.285	-3.007	-2.702	-4.244	3.200	2.952	4.638	77.701	16.951	15.234
9132	233	38	27	C	S	55.693	-4.685	-2.058	-2.550	5.169	2.168	2.688	72.995	14.491	18.209
9133	233	38	27	C	S	58.055	-4.723	1.965	-2.510	5.216	-2.068	2.643	77.733	15.242	16.943
9134	233	38	27	C	S	58.641	-3.292	2.915	-3.966	3.523	-3.166	4.307	72.973	18.221	14.482
9135	233	38	27	C	S	61.359	-3.292	-2.915	-3.966	3.523	3.166	4.307	72.973	18.221	14.482
9136	233	38	27	C	S	61.945	-4.723	-1.965	-2.510	5.216	2.068	2.643	77.733	15.242	16.943
9137	233	38	27	C	S	64.307	-4.685	2.058	-2.550	5.169	-2.168	2.688	72.995	14.491	18.209
9138	233	38	27	C	S	64.715	-3.007	2.702	-4.244	3.200	-2.952	4.638	77.701	16.951	15.234
9139	233	38	27	C	S	68.213	-2.359	-4.451	-4.866	2.476	4.930	5.391	82.011	14.845	17.166
9140	233	38	27	C	S	73.898	-4.228	5.093	-3.024	4.619	-5.421	3.218	81.887	17.184	14.831
9141	233	38	27	C	S	106.102	-4.228	-5.093	-3.024	4.619	5.421	3.218	81.887	17.184	14.831
9142	233	38	27	C	S	111.787	-2.359	4.451	-4.866	2.476	-4.930	5.391	82.011	14.845	17.166
9143	233	38	27	C	S	115.285	-3.007	-2.702	-4.244	3.200	2.952	4.638	77.701	16.951	15.234
9144	233	38	27	C	S	115.693	-4.685	-2.058	-2.550	5.169	2.168	2.688	72.995	14.491	18.209
9145	233	38	27	C	S	118.055	-4.723	1.965	-2.510	5.216	-2.068	2.643	77.733	15.242	16.943
9146	233	38	27	C	S	118.641	-3.292	2.915	-3.966	3.523	-3.166	4.307	72.973	18.221	14.482
9147	233	38	27	C	W	1.359	-3.292	-2.915	-3.966	3.523	3.166	4.307	72.973	18.221	14.482
9148	233	38	27	C	W	1.945	-4.723	-1.965	-2.510	5.216	2.068	2.643	77.733	15.242	16.943
9149	233	38	27	C	W	4.307	-4.685	2.058	-2.550	5.169	-2.168	2.688	72.995	14.491	18.209
9150	233	38	27	C	W	4.715	-3.007	2.702	-4.244	3.200	-2.952	4.638	77.701	16.951	15.234
9151	233	38	27	C	W	8.213	-2.359	-4.451	-4.866	2.476	4.930	5.391	82.011	14.845	17.166
9152	233	38	27	C	W	13.898	-4.228	5.093	-3.024	4.619	-5.421	3.218	81.888	17.184	14.831
9153	233	38	27	C	W	46.102	-4.228	-5.093	-3.024	4.619	5.421	3.218	81.887	17.184	14.831
9154	233	38	27	C	W	51.787	-2.359	4.451	-4.866	2.476	-4.930	5.391	82.011	14.845	17.166
9155	233	38	27	C	W	55.285	-3.007	-2.702	-4.244	3.200	2.952	4.638	77.701	16.951	15.234
9156	233	38	27	C	W	55.693	-4.685	-2.058	-2.550	5.169	2.168	2.688	72.995	14.491	18.209
9157	233	38	27	C	W	58.055	-4.723	1.965	-2.510	5.216	-2.068	2.643	77.733	15.242	16.943
9158	233	38	27	C	W	58.641	-3.292	2.915	-3.966	3.523	-3.166	4.307	72.973	18.221	14.482
9159	233	38	27	C	W	61.359	-3.292	-2.915	-3.966	3.523	3.166	4.307	72.973	18.221	14.482
9160	233	38	27	C	W	61.945	-4.723	-1.965	-2.510	5.216	2.068	2.643	77.733	15.242	16.943
9161	233	38	27	C	W	64.307	-4.685	2.058	-2.550	5.169	-2.168	2.688	72.995	14.491	18.209
9162	233	38	27	C	W	64.715	-3.007	2.702	-4.244	3.200	-2.952	4.638	77.701	16.951	15.234
9163	233	38	27	C	W	68.213	-2.359	-4.451	-4.866	2.476	4.930	5.391	82.011	14.845	17.166
9164	233	38	27	C	W	73.898	-4.228	5.093	-3.024	4.619	-5.421	3.218	81.887	17.184	14.831
9165	233	38	27	C	W	106.102	-4.228	-5.093	-3.024	4.619	5.421	3.218	81.887	17.184	14.831
9166	233	38	27	C	W	111.787	-2.359	4.451	-4.866	2.476	-4.930	5.391	82.011	14.845	17.166
9167	233	38	27	C	W	115.285	-3.007	-2.702	-4.244	3.200	2.952	4.638	77.701	16.951	15.234
9168	233	38	27	C	W	115.693	-4.685	-2.058	-2.550	5.169	2.168	2.688	72.995	14.491	18.209
9169	233	38	27	C	W	118.055	-4.723	1.965	-2.510	5.216	-2.068	2.643	77.733	15.242	16.943
9170	233	38	27	C	W	118.641	-3.292	2.915	-3.966	3.523	-3.166	4.307	72.973	18.221	14.482
9171	233	38	27	P	S	55.285	-3.007	-2.702	-4.244	3.200	2.952	4.638	77.701	16.951	15.234
9172	233	38	27	P	S	58.055	-4.723	1.965	-2.510	5.216	-2.068	2.643	77.733	15.242	16.943
9173	233	38	27	P	S	61.359	-3.292	-2.915	-3.966	3.523	3.166	4.307	72.973	18.221	14.482
9174	233	38	27	P	S	61.945	-4.723	-1.965	-2.510	5.216	2.068	2.643	77.733	15.242	16.943
9175	233	38	27	P	S	64.715	-3.007	2.702	-4.244	3.200	-2.952	4.638	77.701	16.951	15.234
9176	233	38	27	P	W	1.359	-3.292	-2.915	-3.966	3.523	3.166	4.307	72.973	18.221	14.482
9177	233	38	27	P	W	1.945	-4.723	-1.965	-2.510	5.216	2.068	2.643	77.733	15.242	16.943
9178	233	38	27	P	W	4.715	-3.007	2.702	-4.244	3.200	-2.952	4.638	77.701	16.951	15.234
9179	233	38	27	P	W	115.285	-3.007	-2.702	-4.244	3.200	2.952	4.638	77.701	16.951	15.234
9180	233	38	27	P	W	118.055	-4.723	1.965	-2.510	5.216	-2.068	2.643	77.733	15.242	16.943

BL number	Atoms	γ-PC unit cells	WS_2 unit cells	γ-PC origin atom	WS_2 origin atom	Twist-angle (°)	γ-PC strain 1 (%)	γ-PC strain 2 (%)	γ-PC strain 3 (%)	WS_2 strain 1 (%)	WS_2 strain 2 (%)	WS_2 strain 3 (%)	γ (°)	$\lvert\mathbf{a}\rvert$(Å)	$\lvert\mathbf{b}\rvert$(Å)
9181	234	36	30	C	S	30.000	-2.359	0.000	2.934	2.476	0.000	-2.772	90.000	9.718	26.617
9182	234	36	30	C	S	90.000	-2.359	0.000	2.934	2.476	0.000	-2.772	90.000	9.718	26.617
9183	234	36	30	C	S	0.000	5.011	3.368	-4.158	-4.554	-3.674	4.535	68.865	16.799	16.532
9184	234	36	30	C	S	7.589	0.334	-1.743	0.102	-0.332	1.740	-0.101	68.960	17.418	15.901
9185	234	36	30	C	S	8.948	-0.599	0.638	1.048	0.606	-0.625	-1.026	68.946	15.901	17.420
9186	234	36	30	C	S	10.893	4.020	2.224	-3.316	-3.721	-2.382	3.552	73.349	15.815	17.083
9187	234	36	30	C	S	13.898	-0.414	-5.160	0.858	0.417	5.073	-0.843	72.462	18.453	14.691
9188	234	36	30	C	S	16.102	2.853	-1.237	-2.286	-2.699	1.297	2.395	72.437	18.455	14.701
9189	234	36	30	C	S	19.107	-1.485	4.063	1.980	1.530	-3.908	-1.905	72.323	14.708	18.452
9190	234	36	30	C	S	24.791	-3.388	-3.154	4.103	3.635	2.915	-3.792	76.634	17.335	15.348
9191	234	36	30	C	S	25.285	4.262	-2.090	-3.525	-3.928	2.249	3.793	73.354	18.222	14.829
9192	234	36	30	C	S	34.715	4.262	2.090	-3.525	-3.928	-2.249	3.793	73.354	18.222	14.829
9193	234	36	30	C	S	35.209	-3.388	3.154	4.103	3.635	-2.915	-3.792	76.634	17.335	15.348
9194	234	36	30	C	S	40.893	-1.485	-4.063	1.980	1.530	3.908	-1.905	72.323	14.708	18.452
9195	234	36	30	C	S	43.898	2.853	1.237	-2.286	-2.699	-1.297	2.395	72.437	18.455	14.701
9196	234	36	30	C	S	46.102	-0.414	5.160	0.858	0.417	-5.073	-0.843	72.462	14.691	18.453
9197	234	36	30	C	S	49.107	4.020	-2.224	-3.316	-3.721	2.382	3.552	73.349	15.815	17.083
9198	234	36	30	C	S	51.052	1.048	-0.627	-0.599	-1.026	0.635	0.606	68.945	17.420	15.902
9199	234	36	30	C	S	52.411	0.334	1.743	0.102	-0.332	-1.740	-0.101	68.960	15.901	17.418
9200	234	36	30	C	S	60.000	5.011	3.368	-4.158	-4.554	-3.674	4.535	68.865	16.799	16.532
9201	234	36	30	C	S	68.948	1.048	0.627	-0.599	-1.027	-0.635	0.606	68.945	17.420	15.902
9202	234	36	30	C	S	70.893	4.020	2.224	-3.316	-3.721	-2.382	3.552	73.349	15.815	17.083
9203	234	36	30	C	S	73.898	-0.414	-5.160	0.858	0.417	5.073	-0.843	72.462	18.453	14.691
9204	234	36	30	C	S	76.102	2.853	-1.237	-2.286	-2.699	1.297	2.395	72.437	18.455	14.701
9205	234	36	30	C	S	79.107	-1.485	4.063	1.980	1.530	-3.908	-1.905	72.323	14.708	18.452
9206	234	36	30	C	S	84.791	-3.388	-3.154	4.103	3.635	2.915	-3.792	76.634	17.335	15.348
9207	234	36	30	C	S	85.285	4.262	-2.090	-3.525	-3.928	2.249	3.793	73.355	18.222	14.829
9208	234	36	30	C	S	94.715	4.262	2.090	-3.525	-3.928	-2.249	3.793	73.354	18.222	14.829
9209	234	36	30	C	S	95.209	-3.388	3.154	4.103	3.635	-2.915	-3.792	76.634	17.335	15.348
9210	234	36	30	C	S	100.893	-1.485	-4.063	1.980	1.530	3.908	-1.905	72.323	14.708	18.452
9211	234	36	30	C	S	103.898	2.853	1.237	-2.286	-2.699	-1.297	2.395	72.437	18.455	14.701
9212	234	36	30	C	S	106.102	-0.414	5.160	0.858	0.417	-5.073	-0.843	72.462	14.691	18.453
9213	234	36	30	C	S	109.107	4.020	-2.224	-3.316	-3.721	2.382	3.552	73.349	15.815	17.083
9214	234	36	30	C	S	111.052	-0.599	-0.637	1.048	0.606	0.624	-1.026	68.946	15.901	17.420
9215	234	36	30	C	S	112.411	0.334	1.743	0.102	-0.332	-1.740	-0.101	68.960	15.901	17.418
9216	234	36	30	C	W	0.000	5.011	3.368	-4.158	-4.554	-3.674	4.535	68.865	16.799	16.532
9217	234	36	30	C	W	8.948	-0.599	0.638	1.048	0.606	-0.625	-1.026	68.946	15.901	17.420
9218	234	36	30	C	W	10.893	4.020	2.224	-3.316	-3.721	-2.382	3.552	73.349	15.815	17.083
9219	234	36	30	C	W	24.791	-3.388	-3.154	4.103	3.635	2.915	-3.792	76.634	17.335	15.348
9220	234	36	30	C	W	35.209	-3.388	3.154	4.103	3.635	-2.915	-3.792	76.634	17.335	15.348
9221	234	36	30	C	W	49.107	4.020	-2.224	-3.316	-3.721	2.382	3.552	73.349	15.815	17.083
9222	234	36	30	C	W	51.052	-0.599	-0.637	1.048	0.606	0.624	-1.026	68.946	15.901	17.420
9223	234	36	30	C	W	52.411	0.334	1.743	0.102	-0.332	-1.740	-0.101	68.960	15.901	17.418
9224	234	36	30	C	W	60.000	5.011	3.368	-4.158	-4.554	-3.674	4.535	68.865	16.799	16.532
9225	234	36	30	C	W	67.589	0.334	-1.743	0.102	-0.332	1.740	-0.101	68.960	17.418	15.901
9226	234	36	30	C	W	68.948	-0.599	0.638	1.048	0.606	-0.625	-1.026	68.946	15.901	17.420
9227	234	36	30	C	W	70.893	4.020	2.224	-3.316	-3.721	-2.382	3.552	73.349	15.815	17.083
9228	234	36	30	C	W	84.791	-3.388	-3.154	4.103	3.635	2.915	-3.792	76.634	17.335	15.348
9229	234	36	30	C	W	95.209	-3.388	3.154	4.103	3.635	-2.915	-3.792	76.634	17.335	15.348
9230	234	36	30	C	W	109.107	4.020	-2.224	-3.316	-3.721	2.382	3.552	73.349	15.815	17.083
9231	234	36	30	C	W	111.052	1.048	-0.627	-0.599	-1.026	0.635	0.606	68.945	17.420	15.902

BL number	Atoms	γ-PC unit cells	WS_2 unit cells	γ-PC origin atom	WS_2 origin atom	Twist-angle (°)	γ-PC strain 1 (%)	γ-PC strain 2 (%)	γ-PC strain 3 (%)	WS_2 strain 1 (%)	WS_2 strain 2 (%)	WS_2 strain 3 (%)	γ (°)	$\lvert\mathbf{a}\rvert$(Å)	$\lvert\mathbf{b}\rvert$(Å)
9232	234	36	30	C	W	112.411	0.334	1.743	0.102	-0.332	-1.740	-0.101	68.960	15.901	17.418
9233	234	36	30	P	S	0.000	5.011	3.368	-4.158	-4.554	-3.674	4.535	68.865	16.799	16.532
9234	234	36	30	P	S	8.948	-0.599	0.638	1.048	0.606	-0.625	-1.026	68.946	15.901	17.420
9235	234	36	30	P	S	10.893	4.020	2.224	-3.316	-3.721	-2.382	3.552	73.349	15.815	17.083
9236	234	36	30	P	S	25.285	4.262	-2.090	-3.525	-3.928	2.249	3.793	73.354	18.222	14.829
9237	234	36	30	P	S	34.715	4.262	2.090	-3.525	-3.928	-2.249	3.793	73.354	18.222	14.829
9238	234	36	30	P	S	49.107	4.020	-2.224	-3.316	-3.721	2.382	3.552	73.349	15.815	17.083
9239	234	36	30	P	S	51.052	-0.599	-0.637	1.048	0.606	0.624	-1.026	68.946	15.901	17.420
9240	234	36	30	P	S	52.411	0.334	1.743	0.102	-0.332	-1.740	-0.101	68.960	15.901	17.418
9241	234	36	30	P	S	60.000	5.011	3.368	-4.158	-4.554	-3.674	4.535	68.865	16.799	16.532
9242	234	36	30	P	S	67.589	0.334	-1.743	0.102	-0.332	1.740	-0.101	68.960	17.418	15.901
9243	234	36	30	P	S	68.948	-0.599	0.638	1.048	0.606	-0.625	-1.026	68.946	15.901	17.420
9244	234	36	30	P	S	70.893	4.020	2.224	-3.316	-3.721	-2.382	3.552	73.349	15.815	17.083
9245	234	36	30	P	S	85.285	4.262	-2.090	-3.525	-3.928	2.249	3.793	73.355	18.222	14.829
9246	234	36	30	P	S	94.715	4.262	2.090	-3.525	-3.928	-2.249	3.793	73.354	18.222	14.829
9247	234	36	30	P	S	109.107	4.020	-2.224	-3.316	-3.721	2.382	3.552	73.349	15.815	17.083
9248	234	36	30	P	S	111.052	1.048	-0.627	-0.599	-1.026	0.635	0.606	68.945	17.420	15.902
9249	234	36	30	P	S	112.411	0.334	1.743	0.102	-0.332	-1.740	-0.101	68.960	15.901	17.418
9250	234	36	30	P	W	0.000	5.011	3.368	-4.158	-4.554	-3.674	4.535	68.865	16.799	16.532
9251	234	36	30	P	W	7.589	0.334	-1.743	0.102	-0.332	1.740	-0.101	68.960	17.418	15.901
9252	234	36	30	P	W	8.948	-0.599	0.638	1.048	0.606	-0.625	-1.026	68.946	15.901	17.420
9253	234	36	30	P	W	10.893	4.020	2.224	-3.316	-3.721	-2.382	3.552	73.349	15.815	17.083
9254	234	36	30	P	W	49.107	4.020	-2.224	-3.316	-3.721	2.382	3.552	73.349	15.815	17.083
9255	234	36	30	P	W	51.052	1.048	-0.627	-0.599	-1.026	0.635	0.606	68.945	17.420	15.902
9256	234	36	30	P	W	52.411	0.334	1.743	0.102	-0.332	-1.740	-0.101	68.960	15.901	17.418
9257	234	36	30	P	W	60.000	5.011	3.368	-4.158	-4.554	-3.674	4.535	68.865	16.799	16.532
9258	234	36	30	P	W	68.948	1.048	0.627	-0.599	-1.027	-0.635	0.606	68.945	17.420	15.902
9259	234	36	30	P	W	70.893	4.020	2.224	-3.316	-3.721	-2.382	3.552	73.349	15.815	17.083
9260	234	36	30	P	W	109.107	4.020	-2.224	-3.316	-3.721	2.382	3.552	73.349	15.815	17.083
9261	234	36	30	P	W	111.052	-0.599	-0.637	1.048	0.606	0.624	-1.026	68.946	15.901	17.420
9262	234	36	30	P	W	112.411	0.334	1.743	0.102	-0.332	-1.740	-0.101	68.960	15.901	17.418
9263	235	34	33	C	S	9.183	3.582	-1.355	4.817	-3.342	1.236	-4.394	65.425	18.349	15.816
9264	235	34	33	C	S	9.430	5.011	-0.892	3.393	-4.554	0.835	-3.177	65.438	18.353	15.811
9265	235	34	33	C	S	10.893	4.020	1.766	4.373	-3.721	-1.624	-4.021	65.443	15.815	18.347
9266	235	34	33	C	S	50.570	5.011	0.892	3.393	-4.554	-0.835	-3.177	65.438	18.353	15.811
9267	235	34	33	C	S	50.817	3.582	1.355	4.817	-3.342	-1.236	-4.394	65.425	15.816	18.349
9268	235	34	33	C	S	69.183	3.582	-1.355	4.817	-3.342	1.236	-4.394	65.425	18.349	15.816
9269	235	34	33	C	S	69.430	5.011	-0.892	3.393	-4.554	0.835	-3.177	65.438	18.353	15.811
9270	235	34	33	C	S	70.893	4.020	1.766	4.373	-3.721	-1.624	-4.021	65.443	15.815	18.347
9271	235	34	33	C	S	109.107	4.020	-1.766	4.373	-3.721	1.624	-4.021	65.443	15.815	18.347
9272	235	34	33	C	S	110.570	5.011	0.892	3.393	-4.554	-0.835	-3.177	65.438	18.353	15.811
9273	235	34	33	C	S	110.817	3.582	1.355	4.817	-3.342	-1.236	-4.394	65.425	15.816	18.349
9274	235	34	33	C	W	9.183	3.582	-1.355	4.817	-3.342	1.236	-4.394	65.425	18.349	15.816
9275	235	34	33	C	W	9.430	5.011	-0.892	3.393	-4.554	0.835	-3.177	65.438	18.353	15.811
9276	235	34	33	C	W	10.893	4.020	1.766	4.373	-3.721	-1.624	-4.021	65.443	15.815	18.347
9277	235	34	33	C	W	49.107	4.020	-1.766	4.373	-3.721	1.624	-4.021	65.443	15.815	18.347
9278	235	34	33	C	W	50.570	5.011	0.892	3.393	-4.554	-0.835	-3.177	65.438	18.353	15.811
9279	235	34	33	C	W	50.817	3.582	1.355	4.817	-3.342	-1.236	-4.394	65.425	15.816	18.349
9280	235	34	33	C	W	69.183	3.582	-1.355	4.817	-3.342	1.236	-4.394	65.425	18.349	15.816
9281	235	34	33	C	W	69.430	5.011	-0.892	3.393	-4.554	0.835	-3.177	65.438	18.353	15.811
9282	235	34	33	C	W	70.893	4.020	1.766	4.373	-3.721	-1.624	-4.021	65.443	15.815	18.347

BL number	Atoms	γ-PC unit cells	WS_2 unit cells	γ-PC origin atom	WS_2 origin atom	Twist-angle (°)	γ-PC strain 1 (%)	γ-PC strain 2 (%)	γ-PC strain 3 (%)	WS_2 strain 1 (%)	WS_2 strain 2 (%)	WS_2 strain 3 (%)	γ (°)	|**a**|(Å)	|**b**|(Å)
9283	235	34	33	C	W	110.570	5.011	0.892	3.393	-4.554	-0.835	-3.177	65.438	18.353	15.811
9284	235	34	33	C	W	110.817	3.582	1.355	4.817	-3.342	-1.236	-4.394	65.425	15.816	18.349
9285	235	34	33	P	S	9.183	3.582	-1.355	4.817	-3.342	1.236	-4.394	65.425	18.349	15.816
9286	235	34	33	P	S	9.430	5.011	-0.892	3.393	-4.554	0.835	-3.177	65.438	18.353	15.811
9287	235	34	33	P	S	10.893	4.020	1.766	4.373	-3.721	-1.624	-4.021	65.443	15.815	18.347
9288	235	34	33	P	S	49.107	4.020	-1.766	4.373	-3.721	1.624	-4.021	65.443	15.815	18.347
9289	235	34	33	P	S	50.570	5.011	0.892	3.393	-4.554	-0.835	-3.177	65.438	18.353	15.811
9290	235	34	33	P	S	50.817	3.582	1.355	4.817	-3.342	-1.236	-4.394	65.425	15.816	18.349
9291	235	34	33	P	S	69.183	3.582	-1.355	4.817	-3.342	1.236	-4.394	65.425	18.349	15.816
9292	235	34	33	P	S	69.430	5.011	-0.892	3.393	-4.554	0.835	-3.177	65.438	18.353	15.811
9293	235	34	33	P	S	70.893	4.020	1.766	4.373	-3.721	-1.624	-4.021	65.443	15.815	18.347
9294	235	34	33	P	S	110.570	5.011	0.892	3.393	-4.554	-0.835	-3.177	65.438	18.353	15.811
9295	235	34	33	P	S	110.817	3.582	1.355	4.817	-3.342	-1.236	-4.394	65.425	15.816	18.349
9296	235	34	33	P	W	9.183	3.582	-1.355	4.817	-3.342	1.236	-4.394	65.425	18.349	15.816
9297	235	34	33	P	W	9.430	5.011	-0.892	3.393	-4.554	0.835	-3.177	65.438	18.353	15.811
9298	235	34	33	P	W	10.893	4.020	1.766	4.373	-3.721	-1.624	-4.021	65.443	15.815	18.347
9299	235	34	33	P	W	50.570	5.011	0.892	3.393	-4.554	-0.835	-3.177	65.438	18.353	15.811
9300	235	34	33	P	W	50.817	3.582	1.355	4.817	-3.342	-1.236	-4.394	65.425	15.816	18.349
9301	235	34	33	P	W	69.183	3.582	-1.355	4.817	-3.342	1.236	-4.394	65.425	18.349	15.816
9302	235	34	33	P	W	69.430	5.011	-0.892	3.393	-4.554	0.835	-3.177	65.438	18.353	15.811
9303	235	34	33	P	W	70.893	4.020	1.766	4.373	-3.721	-1.624	-4.021	65.443	15.815	18.347
9304	235	34	33	P	W	109.107	4.020	-1.766	4.373	-3.721	1.624	-4.021	65.443	15.815	18.347
9305	235	34	33	P	W	110.570	5.011	0.892	3.393	-4.554	-0.835	-3.177	65.438	18.353	15.811
9306	235	34	33	P	W	110.817	3.582	1.355	4.817	-3.342	-1.236	-4.394	65.425	15.816	18.349
9307	236	35	32	C	S	30.000	0.817	0.000	4.447	-0.804	0.000	-4.084	79.885	16.360	16.360
9308	236	35	32	C	S	90.000	0.817	0.000	4.447	-0.804	0.000	-4.084	79.885	16.360	16.360
9309	236	35	32	C	W	30.000	0.817	0.000	4.447	-0.804	0.000	-4.084	79.885	16.360	16.360
9310	236	35	32	C	W	90.000	0.817	0.000	4.447	-0.804	0.000	-4.084	79.885	16.360	16.360
9311	236	35	32	P	S	30.000	0.817	0.000	4.447	-0.804	0.000	-4.084	79.885	16.360	16.360
9312	236	35	32	P	S	90.000	0.817	0.000	4.447	-0.804	0.000	-4.084	79.885	16.360	16.360
9313	236	35	32	P	W	30.000	0.817	0.000	4.447	-0.804	0.000	-4.084	79.885	16.360	16.360
9314	236	35	32	P	W	90.000	0.817	0.000	4.447	-0.804	0.000	-4.084	79.885	16.360	16.360
9315	236	35	32	C	S	9.430	5.011	-0.866	0.296	-4.554	0.861	-0.294	84.574	18.353	14.424
9316	236	35	32	C	S	10.893	0.418	1.775	4.877	-0.415	-1.617	-4.444	84.522	14.426	18.351
9317	236	35	32	C	S	28.055	2.281	-3.495	2.922	-2.182	3.302	-2.760	79.843	16.362	16.355
9318	236	35	32	C	S	31.945	2.281	3.495	2.922	-2.182	-3.302	-2.760	79.843	16.362	16.355
9319	236	35	32	C	S	49.107	0.418	-1.775	4.877	-0.415	1.617	-4.444	84.522	14.426	18.351
9320	236	35	32	C	S	50.570	5.011	0.866	0.296	-4.554	-0.861	-0.294	84.574	18.353	14.424
9321	236	35	32	C	S	69.430	5.011	-0.866	0.296	-4.554	0.861	-0.294	84.574	18.353	14.424
9322	236	35	32	C	S	70.893	0.418	1.775	4.877	-0.415	-1.617	-4.444	84.522	14.426	18.351
9323	236	35	32	C	S	88.055	2.281	-3.495	2.922	-2.182	3.302	-2.760	79.843	16.362	16.355
9324	236	35	32	C	S	91.945	2.281	3.495	2.922	-2.182	-3.302	-2.760	79.843	16.362	16.355
9325	236	35	32	C	S	109.107	0.418	-1.775	4.877	-0.415	1.617	-4.444	84.522	14.426	18.351
9326	236	35	32	C	S	110.570	5.011	0.866	0.296	-4.554	-0.861	-0.294	84.574	18.353	14.424
9327	236	35	32	C	W	9.430	5.011	-0.866	0.296	-4.554	0.861	-0.294	84.574	18.353	14.424
9328	236	35	32	C	W	10.893	0.418	1.775	4.877	-0.415	-1.617	-4.444	84.522	14.426	18.351
9329	236	35	32	C	W	28.055	2.281	-3.495	2.922	-2.182	3.302	-2.760	79.843	16.362	16.355
9330	236	35	32	C	W	31.945	2.281	3.495	2.922	-2.182	-3.302	-2.760	79.843	16.362	16.355
9331	236	35	32	C	W	49.107	0.418	-1.775	4.877	-0.415	1.617	-4.444	84.522	14.426	18.351
9332	236	35	32	C	W	50.570	5.011	0.866	0.296	-4.554	-0.861	-0.294	84.574	18.353	14.424
9333	236	35	32	C	W	69.430	5.011	-0.866	0.296	-4.554	0.861	-0.294	84.574	18.353	14.424

BL number	Atoms	γ-PC unit cells	WS_2 unit cells	γ-PC origin atom	WS_2 origin atom	Twist-angle (°)	γ-PC strain 1 (%)	γ-PC strain 2 (%)	γ-PC strain 3 (%)	WS_2 strain 1 (%)	WS_2 strain 2 (%)	WS_2 strain 3 (%)	γ (°)	|**a**|(Å)	|**b**|(Å)
9334	236	35	32	C	W	70.893	0.418	1.775	4.877	-0.415	-1.617	-4.444	84.522	14.426	18.351
9335	236	35	32	C	W	88.055	2.281	-3.495	2.922	-2.182	3.302	-2.760	79.843	16.362	16.355
9336	236	35	32	C	W	91.945	2.281	3.495	2.922	-2.182	-3.302	-2.760	79.843	16.362	16.355
9337	236	35	32	C	W	109.107	0.418	-1.775	4.877	-0.415	1.617	-4.444	84.522	14.426	18.351
9338	236	35	32	C	W	110.570	5.011	0.866	0.296	-4.554	-0.861	-0.294	84.574	18.353	14.424
9339	236	35	32	P	S	9.430	5.011	-0.866	0.296	-4.554	0.861	-0.294	84.574	18.353	14.424
9340	236	35	32	P	S	10.893	0.418	1.775	4.877	-0.415	-1.617	-4.444	84.522	14.426	18.351
9341	236	35	32	P	S	28.055	2.281	-3.495	2.922	-2.182	3.302	-2.760	79.843	16.362	16.355
9342	236	35	32	P	S	31.945	2.281	3.495	2.922	-2.182	-3.302	-2.760	79.843	16.362	16.355
9343	236	35	32	P	S	49.107	0.418	-1.775	4.877	-0.415	1.617	-4.444	84.522	14.426	18.351
9344	236	35	32	P	S	50.570	5.011	0.866	0.296	-4.554	-0.861	-0.294	84.574	18.353	14.424
9345	236	35	32	P	S	69.430	5.011	-0.866	0.296	-4.554	0.861	-0.294	84.574	18.353	14.424
9346	236	35	32	P	S	70.893	0.418	1.775	4.877	-0.415	-1.617	-4.444	84.522	14.426	18.351
9347	236	35	32	P	S	88.055	2.281	-3.495	2.922	-2.182	3.302	-2.760	79.843	16.362	16.355
9348	236	35	32	P	S	91.945	2.281	3.495	2.922	-2.182	-3.302	-2.760	79.843	16.362	16.355
9349	236	35	32	P	S	109.107	0.418	-1.775	4.877	-0.415	1.617	-4.444	84.522	14.426	18.351
9350	236	35	32	P	S	110.570	5.011	0.866	0.296	-4.554	-0.861	-0.294	84.574	18.353	14.424
9351	236	35	32	P	W	9.430	5.011	-0.866	0.296	-4.554	0.861	-0.294	84.574	18.353	14.424
9352	236	35	32	P	W	10.893	0.418	1.775	4.877	-0.415	-1.617	-4.444	84.522	14.426	18.351
9353	236	35	32	P	W	28.055	2.281	-3.495	2.922	-2.182	3.302	-2.760	79.843	16.362	16.355
9354	236	35	32	P	W	31.945	2.281	3.495	2.922	-2.182	-3.302	-2.760	79.843	16.362	16.355
9355	236	35	32	P	W	49.107	0.418	-1.775	4.877	-0.415	1.617	-4.444	84.522	14.426	18.351
9356	236	35	32	P	W	50.570	5.011	0.866	0.296	-4.554	-0.861	-0.294	84.574	18.353	14.424
9357	236	35	32	P	W	69.430	5.011	-0.866	0.296	-4.554	0.861	-0.294	84.574	18.353	14.424
9358	236	35	32	P	W	70.893	0.418	1.775	4.877	-0.415	-1.617	-4.444	84.522	14.426	18.351
9359	236	35	32	P	W	88.055	2.281	-3.495	2.922	-2.182	3.302	-2.760	79.843	16.362	16.355
9360	236	35	32	P	W	91.945	2.281	3.495	2.922	-2.182	-3.302	-2.760	79.843	16.362	16.355
9361	236	35	32	P	W	109.107	0.418	-1.775	4.877	-0.415	1.617	-4.444	84.522	14.426	18.351
9362	236	35	32	P	W	110.570	5.011	0.866	0.296	-4.554	-0.861	-0.294	84.574	18.353	14.424
9363	236	38	28	C	S	3.004	-3.388	-4.404	-2.162	3.635	4.603	2.259	87.423	17.335	14.828
9364	236	38	28	C	S	4.307	-4.685	-2.210	-0.793	5.169	2.246	0.806	86.264	14.491	17.765
9365	236	38	28	C	S	7.154	-0.955	2.621	-4.535	0.973	-2.883	4.988	86.160	17.772	14.487
9366	236	38	28	C	S	8.213	-2.359	4.451	-3.195	2.476	-4.754	3.413	41.716	17.316	22.285
9367	236	38	28	C	S	24.791	-3.388	-4.404	-2.162	3.635	4.603	2.259	87.423	17.335	14.828
9368	236	38	28	C	S	30.000	-2.359	-4.451	-3.195	2.476	4.754	3.413	41.716	22.285	17.316
9369	236	38	28	C	S	35.209	-3.388	4.404	-2.162	3.635	-4.603	2.259	87.423	17.335	14.828
9370	236	38	28	C	S	51.787	-2.359	-4.451	-3.195	2.476	4.754	3.413	41.716	22.285	17.316
9371	236	38	28	C	S	52.846	-0.955	-2.621	-4.535	0.973	2.883	4.988	86.160	17.772	14.487
9372	236	38	28	C	S	55.693	-4.685	2.210	-0.793	5.169	-2.246	0.806	86.264	14.491	17.765
9373	236	38	28	C	S	56.996	-3.388	4.404	-2.162	3.635	-4.603	2.259	87.423	17.335	14.828
9374	236	38	28	C	S	63.005	-3.388	-4.404	-2.162	3.635	4.603	2.259	87.423	17.335	14.828
9375	236	38	28	C	S	64.307	-4.685	-2.210	-0.793	5.169	2.246	0.806	86.264	14.491	17.765
9376	236	38	28	C	S	67.154	-0.955	2.621	-4.535	0.973	-2.883	4.988	86.160	17.772	14.487
9377	236	38	28	C	S	68.213	-2.359	4.451	-3.195	2.476	-4.754	3.413	87.368	14.845	17.316
9378	236	38	28	C	S	84.791	-3.388	-4.404	-2.162	3.635	4.603	2.259	87.423	17.335	14.828
9379	236	38	28	C	S	90.000	-2.359	4.451	-3.195	2.476	-4.754	3.413	87.368	14.845	17.316
9380	236	38	28	C	S	95.209	-3.388	4.404	-2.162	3.635	-4.603	2.259	87.423	17.335	14.828
9381	236	38	28	C	S	111.787	-2.359	-4.451	-3.195	2.476	4.754	3.413	87.368	14.845	17.316
9382	236	38	28	C	S	112.846	-0.955	-2.621	-4.535	0.973	2.883	4.988	86.160	17.772	14.486
9383	236	38	28	C	S	115.693	-4.685	2.210	-0.793	5.169	-2.246	0.806	86.264	14.491	17.765
9384	236	38	28	C	S	116.996	-3.388	4.404	-2.162	3.635	-4.603	2.259	87.423	17.335	14.828

BL number	Atoms	γ-PC unit cells	WS_2 unit cells	γ-PC origin atom	WS_2 origin atom	Twist-angle (°)	γ-PC strain 1 (%)	γ-PC strain 2 (%)	γ-PC strain 3 (%)	WS_2 strain 1 (%)	WS_2 strain 2 (%)	WS_2 strain 3 (%)	γ (°)	$\lvert\mathbf{a}\rvert$(Å)	$\lvert\mathbf{b}\rvert$(Å)
9385	236	38	28	C	W	3.004	-3.388	-4.404	-2.162	3.635	4.603	2.259	87.423	17.335	14.828
9386	236	38	28	C	W	4.307	-4.685	-2.210	-0.793	5.169	2.246	0.806	86.264	14.491	17.765
9387	236	38	28	C	W	7.154	-0.955	2.621	-4.535	0.973	-2.883	4.988	86.160	17.772	14.487
9388	236	38	28	C	W	8.213	-2.359	4.451	-3.195	2.476	-4.754	3.413	87.368	14.845	17.316
9389	236	38	28	C	W	24.791	-3.388	-4.404	-2.162	3.635	4.603	2.259	87.423	17.335	14.828
9390	236	38	28	C	W	30.000	-2.359	-4.451	-3.195	2.476	4.754	3.413	87.368	14.845	17.316
9391	236	38	28	C	W	35.209	-3.388	4.404	-2.162	3.635	-4.603	2.259	87.423	17.335	14.828
9392	236	38	28	C	W	51.787	-2.359	-4.451	-3.195	2.476	4.754	3.413	87.368	14.845	17.316
9393	236	38	28	C	W	52.846	-0.955	-2.621	-4.535	0.973	2.883	4.988	86.160	17.772	14.487
9394	236	38	28	C	W	55.693	-4.685	2.210	-0.793	5.169	-2.246	0.806	86.264	14.491	17.765
9395	236	38	28	C	W	56.996	-3.388	4.404	-2.162	3.635	-4.603	2.259	87.423	17.335	14.828
9396	236	38	28	C	W	63.005	-3.388	-4.404	-2.162	3.635	4.603	2.259	87.423	17.335	14.828
9397	236	38	28	C	W	64.307	-4.685	-2.210	-0.793	5.169	2.246	0.806	86.264	14.491	17.765
9398	236	38	28	C	W	67.154	-0.955	2.621	-4.535	0.973	-2.883	4.988	86.160	17.772	14.487
9399	236	38	28	C	W	68.213	-2.359	4.451	-3.195	2.476	-4.754	3.413	87.368	14.845	17.316
9400	236	38	28	C	W	84.791	-3.388	-4.404	-2.162	3.635	4.603	2.259	87.423	17.335	14.828
9401	236	38	28	C	W	90.000	-2.359	4.451	-3.195	2.476	-4.754	3.413	87.368	14.845	17.316
9402	236	38	28	C	W	95.209	-3.388	4.404	-2.162	3.635	-4.603	2.259	87.423	17.335	14.828
9403	236	38	28	C	W	111.787	-2.359	-4.451	-3.195	2.476	4.754	3.413	87.368	14.845	17.316
9404	236	38	28	C	W	112.846	-0.955	-2.621	-4.535	0.973	2.883	4.988	86.160	17.772	14.486
9405	236	38	28	C	W	115.693	-4.685	2.210	-0.793	5.169	-2.246	0.806	86.264	14.491	17.765
9406	236	38	28	C	W	116.996	-3.388	4.404	-2.162	3.635	-4.603	2.259	87.423	17.335	14.828
9407	236	38	28	P	S	3.004	-3.388	-4.404	-2.162	3.635	4.603	2.259	87.423	17.335	14.828
9408	236	38	28	P	S	4.307	-4.685	-2.210	-0.793	5.169	2.246	0.806	86.264	14.491	17.765
9409	236	38	28	P	S	7.154	-0.955	2.621	-4.535	0.973	-2.883	4.988	86.160	17.772	14.487
9410	236	38	28	P	S	8.213	-2.359	4.451	-3.195	2.476	-4.754	3.413	41.716	17.316	22.285
9411	236	38	28	P	S	24.791	-3.388	-4.404	-2.162	3.635	4.603	2.259	87.423	17.335	14.828
9412	236	38	28	P	S	30.000	-2.359	-4.451	-3.195	2.476	4.754	3.413	41.716	22.285	17.316
9413	236	38	28	P	S	35.209	-3.388	4.404	-2.162	3.635	-4.603	2.259	87.423	17.335	14.828
9414	236	38	28	P	S	51.787	-2.359	-4.451	-3.195	2.476	4.754	3.413	41.716	22.285	17.316
9415	236	38	28	P	S	52.846	-0.955	-2.621	-4.535	0.973	2.883	4.988	86.160	17.772	14.487
9416	236	38	28	P	S	55.693	-4.685	2.210	-0.793	5.169	-2.246	0.806	86.264	14.491	17.765
9417	236	38	28	P	S	56.996	-3.388	4.404	-2.162	3.635	-4.603	2.259	87.423	17.335	14.828
9418	236	38	28	P	S	63.005	-3.388	-4.404	-2.162	3.635	4.603	2.259	87.423	17.335	14.828
9419	236	38	28	P	S	64.307	-4.685	-2.210	-0.793	5.169	2.246	0.806	86.264	14.491	17.765
9420	236	38	28	P	S	67.154	-0.955	2.621	-4.535	0.973	-2.883	4.988	86.160	17.772	14.487
9421	236	38	28	P	S	68.213	-2.359	4.451	-3.195	2.476	-4.754	3.413	87.368	14.845	17.316
9422	236	38	28	P	S	84.791	-3.388	-4.404	-2.162	3.635	4.603	2.259	87.423	17.335	14.828
9423	236	38	28	P	S	90.000	-2.359	4.451	-3.195	2.476	-4.754	3.413	87.368	14.845	17.316
9424	236	38	28	P	S	95.209	-3.388	4.404	-2.162	3.635	-4.603	2.259	87.423	17.335	14.828
9425	236	38	28	P	S	111.787	-2.359	-4.451	-3.195	2.476	4.754	3.413	87.368	14.845	17.316
9426	236	38	28	P	S	112.846	-0.955	-2.621	-4.535	0.973	2.883	4.988	86.160	17.772	14.486
9427	236	38	28	P	S	115.693	-4.685	2.210	-0.793	5.169	-2.246	0.806	86.264	14.491	17.765
9428	236	38	28	P	S	116.996	-3.388	4.404	-2.162	3.635	-4.603	2.259	87.423	17.335	14.828
9429	236	38	28	P	W	3.004	-3.388	-4.404	-2.162	3.635	4.603	2.259	87.423	17.335	14.828
9430	236	38	28	P	W	4.307	-4.685	-2.210	-0.793	5.169	2.246	0.806	86.264	14.491	17.765
9431	236	38	28	P	W	7.154	-0.955	2.621	-4.535	0.973	-2.883	4.988	86.160	17.772	14.487
9432	236	38	28	P	W	8.213	-2.359	4.451	-3.195	2.476	-4.754	3.413	87.368	14.845	17.316
9433	236	38	28	P	W	24.791	-3.388	-4.404	-2.162	3.635	4.603	2.259	87.423	17.335	14.828
9434	236	38	28	P	W	30.000	-2.359	-4.451	-3.195	2.476	4.754	3.413	87.368	14.845	17.316
9435	236	38	28	P	W	35.209	-3.388	4.404	-2.162	3.635	-4.603	2.259	87.423	17.335	14.828

BL number	Atoms	γ-PC unit cells	WS_2 unit cells	γ-PC origin atom	WS_2 origin atom	Twist-angle (°)	γ-PC strain 1 (%)	γ-PC strain 2 (%)	γ-PC strain 3 (%)	WS_2 strain 1 (%)	WS_2 strain 2 (%)	WS_2 strain 3 (%)	γ (°)	|**a**|(Å)	|**b**|(Å)
9436	236	38	28	P	W	51.787	-2.359	-4.451	-3.195	2.476	4.754	3.413	87.368	14.845	17.316
9437	236	38	28	P	W	52.846	-0.955	-2.621	-4.535	0.973	2.883	4.988	86.160	17.772	14.487
9438	236	38	28	P	W	55.693	-4.685	2.210	-0.793	5.169	-2.246	0.806	86.264	14.491	17.765
9439	236	38	28	P	W	56.996	-3.388	4.404	-2.162	3.635	-4.603	2.259	87.423	17.335	14.828
9440	236	38	28	P	W	63.005	-3.388	-4.404	-2.162	3.635	4.603	2.259	87.423	17.335	14.828
9441	236	38	28	P	W	64.307	-4.685	-2.210	-0.793	5.169	2.246	0.806	86.264	14.491	17.765
9442	236	38	28	P	W	67.154	-0.955	2.621	-4.535	0.973	-2.883	4.988	86.160	17.772	14.487
9443	236	38	28	P	W	68.213	-2.359	4.451	-3.195	2.476	-4.754	3.413	87.368	14.845	17.316
9444	236	38	28	P	W	84.791	-3.388	-4.404	-2.162	3.635	4.603	2.259	87.423	17.335	14.828
9445	236	38	28	P	W	90.000	-2.359	4.451	-3.195	2.476	-4.754	3.413	87.368	14.845	17.316
9446	236	38	28	P	W	95.209	-3.388	4.404	-2.162	3.635	-4.603	2.259	87.423	17.335	14.828
9447	236	38	28	P	W	111.787	-2.359	-4.451	-3.195	2.476	4.754	3.413	87.368	14.845	17.316
9448	236	38	28	P	W	112.846	-0.955	-2.621	-4.535	0.973	2.883	4.988	86.160	17.772	14.486
9449	236	38	28	P	W	115.693	-4.685	2.210	-0.793	5.169	-2.246	0.806	86.264	14.491	17.765
9450	236	38	28	P	W	116.996	-3.388	4.404	-2.162	3.635	-4.603	2.259	87.423	17.335	14.828
9451	237	36	31	C	S	8.948	1.048	0.000	1.048	-1.026	0.000	-1.026	60.000	17.420	17.420
9452	237	36	31	C	S	51.052	1.048	0.000	1.048	-1.026	0.000	-1.026	60.000	17.420	17.420
9453	237	36	31	C	S	68.948	1.048	0.000	1.048	-1.026	0.000	-1.026	60.000	17.420	17.420
9454	237	36	31	C	S	111.052	1.048	0.000	1.048	-1.026	0.000	-1.026	60.000	17.420	17.420
9455	237	36	31	P	S	8.948	1.048	0.000	1.048	-1.026	0.000	-1.026	60.000	17.420	17.420
9456	237	36	31	P	S	51.052	1.048	0.000	1.048	-1.026	0.000	-1.026	60.000	17.420	17.420
9457	237	36	31	P	S	68.948	1.048	0.000	1.048	-1.026	0.000	-1.026	60.000	17.420	17.420
9458	237	36	31	P	S	111.052	1.048	0.000	1.048	-1.026	0.000	-1.026	60.000	17.420	17.420
9459	237	36	31	C	S	0.000	5.011	-2.526	-2.629	-4.554	2.666	2.775	76.857	13.826	19.545
9460	237	36	31	C	S	8.213	-2.359	-3.132	4.699	2.476	2.863	-4.295	74.932	14.845	18.355
9461	237	36	31	C	S	9.430	5.011	-0.842	-2.629	-4.554	0.889	2.775	74.993	18.353	14.845
9462	237	36	31	C	S	12.520	2.326	-1.684	-0.199	-2.223	1.691	0.199	79.772	13.473	19.823
9463	237	36	31	C	S	13.898	-0.414	0.737	2.553	0.417	-0.701	-2.429	79.813	19.824	13.472
9464	237	36	31	C	S	19.107	-1.485	-1.016	3.713	1.530	0.945	-3.456	74.145	14.708	18.586
9465	237	36	31	C	S	40.893	-1.485	1.016	3.713	1.530	-0.945	-3.456	74.145	14.708	18.586
9466	237	36	31	C	S	41.387	3.582	1.818	-1.366	-3.342	-1.869	1.404	74.107	18.586	14.710
9467	237	36	31	C	S	46.102	-0.414	-0.737	2.553	0.417	0.701	-2.429	79.813	19.824	13.472
9468	237	36	31	C	S	47.480	2.326	1.684	-0.199	-2.223	-1.691	0.199	79.772	13.473	19.823
9469	237	36	31	C	S	50.570	5.011	0.842	-2.629	-4.554	-0.889	2.775	74.993	18.353	14.845
9470	237	36	31	C	S	51.787	-2.359	3.132	4.699	2.476	-2.863	-4.295	74.932	14.845	18.355
9471	237	36	31	C	S	60.000	5.011	2.526	-2.629	-4.554	-2.667	2.775	76.857	13.826	19.545
9472	237	36	31	C	S	68.213	-2.359	-3.132	4.699	2.476	2.863	-4.295	74.932	14.845	18.355
9473	237	36	31	C	S	69.430	5.011	-0.842	-2.629	-4.554	0.889	2.775	74.993	18.353	14.845
9474	237	36	31	C	S	72.520	2.326	-1.684	-0.199	-2.223	1.691	0.199	79.771	13.473	19.823
9475	237	36	31	C	S	73.898	-0.414	0.737	2.553	0.417	-0.701	-2.429	79.813	19.824	13.472
9476	237	36	31	C	S	78.613	3.582	-1.818	-1.366	-3.342	1.869	1.404	74.107	18.586	14.710
9477	237	36	31	C	S	79.107	-1.485	-1.016	3.713	1.530	0.945	-3.456	74.146	14.708	18.586
9478	237	36	31	C	S	100.893	-1.485	1.016	3.713	1.530	-0.945	-3.456	74.145	14.708	18.586
9479	237	36	31	C	S	106.102	-0.414	-0.737	2.553	0.417	0.701	-2.429	79.813	19.824	13.472
9480	237	36	31	C	S	107.480	2.326	1.684	-0.199	-2.223	-1.691	0.199	79.771	13.473	19.823
9481	237	36	31	C	S	110.570	5.011	0.842	-2.629	-4.554	-0.889	2.775	74.993	18.353	14.845
9482	237	36	31	C	S	111.787	-2.359	3.132	4.699	2.476	-2.863	-4.295	74.932	14.845	18.355
9483	237	36	31	C	W	0.000	5.011	-2.526	-2.629	-4.554	2.666	2.775	76.857	13.826	19.545
9484	237	36	31	C	W	8.213	-2.359	-3.132	4.699	2.476	2.863	-4.295	74.932	14.845	18.355
9485	237	36	31	C	W	9.430	5.011	-0.842	-2.629	-4.554	0.889	2.775	74.993	18.353	14.845
9486	237	36	31	C	W	12.520	2.326	-1.684	-0.199	-2.223	1.691	0.199	79.772	13.473	19.823

BL number	Atoms	γ-PC unit cells	WS_2 unit cells	γ-PC origin atom	WS_2 origin atom	Twist-angle (°)	γ-PC strain 1 (%)	γ-PC strain 2 (%)	γ-PC strain 3 (%)	WS_2 strain 1 (%)	WS_2 strain 2 (%)	WS_2 strain 3 (%)	γ (°)	$\lvert\mathbf{a}\rvert$(Å)	$\lvert\mathbf{b}\rvert$(Å)
9487	237	36	31	C	W	13.898	-0.414	0.737	2.553	0.417	-0.701	-2.429	79.813	19.824	13.472
9488	237	36	31	C	W	18.613	3.582	-1.818	-1.366	-3.342	1.869	1.404	74.107	18.586	14.710
9489	237	36	31	C	W	46.102	-0.414	-0.737	2.553	0.417	0.701	-2.429	79.813	19.824	13.472
9490	237	36	31	C	W	47.480	2.326	1.684	-0.199	-2.223	-1.691	0.199	79.772	13.473	19.823
9491	237	36	31	C	W	50.570	5.011	0.842	-2.629	-4.554	-0.889	2.775	74.993	18.353	14.845
9492	237	36	31	C	W	51.787	-2.359	3.132	4.699	2.476	-2.863	-4.295	74.932	14.845	18.355
9493	237	36	31	C	W	60.000	5.011	2.526	-2.629	-4.554	-2.667	2.775	76.857	13.826	19.545
9494	237	36	31	C	W	68.213	-2.359	-3.132	4.699	2.476	2.863	-4.295	74.932	14.845	18.355
9495	237	36	31	C	W	69.430	5.011	-0.842	-2.629	-4.554	0.889	2.775	74.993	18.353	14.845
9496	237	36	31	C	W	72.520	2.326	-1.684	-0.199	-2.223	1.691	0.199	79.771	13.473	19.823
9497	237	36	31	C	W	73.898	-0.414	0.737	2.553	0.417	-0.701	-2.429	79.813	19.824	13.472
9498	237	36	31	C	W	101.387	3.582	1.818	-1.366	-3.342	-1.869	1.404	74.107	18.586	14.710
9499	237	36	31	C	W	106.102	-0.414	-0.737	2.553	0.417	0.701	-2.429	79.813	19.824	13.472
9500	237	36	31	C	W	107.480	2.326	1.684	-0.199	-2.223	-1.691	0.199	79.771	13.473	19.823
9501	237	36	31	C	W	110.570	5.011	0.842	-2.629	-4.554	-0.889	2.775	74.993	18.353	14.845
9502	237	36	31	C	W	111.787	-2.359	3.132	4.699	2.476	-2.863	-4.295	74.932	14.845	18.355
9503	237	36	31	P	S	0.000	5.011	-2.526	-2.629	-4.554	2.666	2.775	76.857	13.826	19.545
9504	237	36	31	P	S	8.213	-2.359	-3.132	4.699	2.476	2.863	-4.295	74.932	14.845	18.355
9505	237	36	31	P	S	9.430	5.011	-0.842	-2.629	-4.554	0.889	2.775	74.993	18.353	14.845
9506	237	36	31	P	S	19.107	-1.485	-1.016	3.713	1.530	0.945	-3.456	74.145	14.708	18.586
9507	237	36	31	P	S	40.893	-1.485	1.016	3.713	1.530	-0.945	-3.456	74.145	14.708	18.586
9508	237	36	31	P	S	41.387	3.582	1.818	-1.366	-3.342	-1.869	1.404	74.107	18.586	14.710
9509	237	36	31	P	S	46.102	-0.414	-0.737	2.553	0.417	0.701	-2.429	79.813	19.824	13.472
9510	237	36	31	P	S	47.480	2.326	1.684	-0.199	-2.223	-1.691	0.199	79.772	13.473	19.823
9511	237	36	31	P	S	50.570	5.011	0.842	-2.629	-4.554	-0.889	2.775	74.993	18.353	14.845
9512	237	36	31	P	S	51.787	-2.359	3.132	4.699	2.476	-2.863	-4.295	74.932	14.845	18.355
9513	237	36	31	P	S	68.213	-2.359	-3.132	4.699	2.476	2.863	-4.295	74.932	14.845	18.355
9514	237	36	31	P	S	69.430	5.011	-0.842	-2.629	-4.554	0.889	2.775	74.993	18.353	14.845
9515	237	36	31	P	S	72.520	2.326	-1.684	-0.199	-2.223	1.691	0.199	79.771	13.473	19.823
9516	237	36	31	P	S	73.898	-0.414	0.737	2.553	0.417	-0.701	-2.429	79.813	19.824	13.472
9517	237	36	31	P	S	78.613	3.582	-1.818	-1.366	-3.342	1.869	1.404	74.107	18.586	14.710
9518	237	36	31	P	S	79.107	-1.485	-1.016	3.713	1.530	0.945	-3.456	74.146	14.708	18.586
9519	237	36	31	P	S	100.893	-1.485	1.016	3.713	1.530	-0.945	-3.456	74.145	14.708	18.586
9520	237	36	31	P	S	110.570	5.011	0.842	-2.629	-4.554	-0.889	2.775	74.993	18.353	14.845
9521	237	36	31	P	S	111.787	-2.359	3.132	4.699	2.476	-2.863	-4.295	74.932	14.845	18.355
9522	237	36	31	P	W	8.213	-2.359	-3.132	4.699	2.476	2.863	-4.295	74.932	14.845	18.355
9523	237	36	31	P	W	9.430	5.011	-0.842	-2.629	-4.554	0.889	2.775	74.993	18.353	14.845
9524	237	36	31	P	W	12.520	2.326	-1.684	-0.199	-2.223	1.691	0.199	79.772	13.473	19.823
9525	237	36	31	P	W	13.898	-0.414	0.737	2.553	0.417	-0.701	-2.429	79.813	19.824	13.472
9526	237	36	31	P	W	18.613	3.582	-1.818	-1.366	-3.342	1.869	1.404	74.107	18.586	14.710
9527	237	36	31	P	W	50.570	5.011	0.842	-2.629	-4.554	-0.889	2.775	74.993	18.353	14.845
9528	237	36	31	P	W	51.787	-2.359	3.132	4.699	2.476	-2.863	-4.295	74.932	14.845	18.355
9529	237	36	31	P	W	60.000	5.011	2.526	-2.629	-4.554	-2.667	2.775	76.857	13.826	19.545
9530	237	36	31	P	W	68.213	-2.359	-3.132	4.699	2.476	2.863	-4.295	74.932	14.845	18.355
9531	237	36	31	P	W	69.430	5.011	-0.842	-2.629	-4.554	0.889	2.775	74.993	18.353	14.845
9532	237	36	31	P	W	101.387	3.582	1.818	-1.366	-3.342	-1.869	1.404	74.107	18.586	14.710
9533	237	36	31	P	W	106.102	-0.414	-0.737	2.553	0.417	0.701	-2.429	79.813	19.824	13.472
9534	237	36	31	P	W	107.480	2.326	1.684	-0.199	-2.223	-1.691	0.199	79.771	13.473	19.823
9535	237	36	31	P	W	110.570	5.011	0.842	-2.629	-4.554	-0.889	2.775	74.993	18.353	14.845
9536	237	36	31	P	W	111.787	-2.359	3.132	4.699	2.476	-2.863	-4.295	74.932	14.845	18.355
9537	238	37	30	C	S	7.154	-0.955	-1.421	0.029	0.973	1.420	-0.029	68.052	17.772	15.899

BL number	Atoms	γ-PC unit cells	WS_2 unit cells	γ-PC origin atom	WS_2 origin atom	Twist-angle (°)	γ-PC strain 1 (%)	γ-PC strain 2 (%)	γ-PC strain 3 (%)	WS_2 strain 1 (%)	WS_2 strain 2 (%)	WS_2 strain 3 (%)	γ (°)	|**a**|(Å)	|**b**|(Å)
9538	238	37	30	C	S	13.898	-4.228	0.747	3.607	4.619	-0.697	-3.364	69.081	17.184	16.352
9539	238	37	30	C	S	16.102	2.853	4.414	-3.575	-2.699	-4.753	3.851	65.111	18.232	15.862
9540	238	37	30	C	S	16.537	-3.292	5.476	2.532	3.523	-5.212	-2.410	65.217	18.221	15.856
9541	238	37	30	C	S	19.842	-4.723	0.319	4.193	5.216	-0.294	-3.868	69.083	15.242	18.444
9542	238	37	30	C	S	21.052	1.339	-5.008	-2.207	-1.304	5.239	2.309	68.924	16.211	17.330
9543	238	37	30	C	S	24.791	-3.388	1.454	2.641	3.635	-1.381	-2.508	69.073	17.335	16.200
9544	238	37	30	C	S	25.285	-4.781	-5.497	4.263	5.287	5.066	-3.928	64.614	16.641	17.467
9545	238	37	30	C	S	34.715	-4.781	-5.497	4.263	5.287	5.066	-3.928	64.614	16.641	17.467
9546	238	37	30	C	S	35.209	-3.388	-1.454	2.641	3.635	1.381	-2.508	69.073	17.335	16.200
9547	238	37	30	C	S	38.948	1.339	5.008	-2.207	-1.304	-5.239	2.309	68.924	16.211	17.330
9548	238	37	30	C	S	40.158	-4.723	-0.319	4.193	5.216	0.294	-3.868	69.083	15.242	18.444
9549	238	37	30	C	S	43.463	-3.292	-5.476	2.532	3.523	5.212	-2.410	65.217	18.221	15.856
9550	238	37	30	C	S	43.898	2.853	-4.414	-3.575	-2.699	4.753	3.850	65.111	15.862	18.232
9551	238	37	30	C	S	46.102	-4.228	-0.747	3.607	4.619	0.697	-3.364	69.081	17.184	16.352
9552	238	37	30	C	S	52.411	0.334	0.652	-1.252	-0.332	-0.669	1.285	68.045	17.772	15.900
9553	238	37	30	C	S	67.154	-0.955	-1.421	0.029	0.973	1.420	-0.029	68.052	17.772	15.899
9554	238	37	30	C	S	73.898	-4.228	0.747	3.607	4.619	-0.697	-3.364	69.081	17.184	16.352
9555	238	37	30	C	S	76.102	2.853	4.414	-3.575	-2.699	-4.753	3.851	65.111	18.232	15.862
9556	238	37	30	C	S	76.537	-3.292	5.476	2.532	3.523	-5.212	-2.410	65.217	18.221	15.856
9557	238	37	30	C	S	79.842	-4.723	0.319	4.193	5.216	-0.294	-3.868	69.083	15.242	18.444
9558	238	37	30	C	S	81.052	1.339	-5.008	-2.207	-1.304	5.239	2.309	68.924	16.211	17.330
9559	238	37	30	C	S	84.791	-3.388	1.454	2.641	3.635	-1.381	-2.508	69.073	17.335	16.200
9560	238	37	30	C	S	85.285	-4.781	-5.497	4.263	5.287	5.066	-3.928	64.614	16.641	17.467
9561	238	37	30	C	S	94.715	-4.781	-5.497	4.263	5.287	5.066	-3.928	64.614	16.641	17.467
9562	238	37	30	C	S	95.209	-3.388	-1.454	2.641	3.635	1.381	-2.508	69.073	17.335	16.200
9563	238	37	30	C	S	98.948	1.339	5.008	-2.207	-1.304	-5.239	2.309	68.924	16.211	17.330
9564	238	37	30	C	S	100.158	-4.723	-0.319	4.193	5.216	0.294	-3.868	69.083	15.242	18.444
9565	238	37	30	C	S	103.463	-3.292	-5.476	2.532	3.523	5.212	-2.410	65.217	18.221	15.856
9566	238	37	30	C	S	103.898	2.853	-4.414	-3.575	-2.699	4.753	3.850	65.111	15.862	18.232
9567	238	37	30	C	S	106.102	-4.228	-0.747	3.607	4.619	0.697	-3.364	69.081	17.184	16.352
9568	238	37	30	C	S	112.411	0.334	0.652	-1.252	-0.332	-0.669	1.285	68.045	17.772	15.900
9569	238	37	30	C	W	7.154	-0.955	-1.421	0.029	0.973	1.420	-0.029	68.052	17.772	15.899
9570	238	37	30	C	W	13.898	-4.228	0.747	3.607	4.619	-0.697	-3.364	69.081	17.184	16.352
9571	238	37	30	C	W	16.102	2.853	4.414	-3.575	-2.699	-4.753	3.851	65.111	18.232	15.862
9572	238	37	30	C	W	16.537	-3.292	5.476	2.532	3.523	-5.212	-2.410	65.217	18.221	15.856
9573	238	37	30	C	W	19.842	-4.723	0.319	4.193	5.216	-0.294	-3.868	69.083	15.242	18.444
9574	238	37	30	C	W	21.052	1.339	-5.008	-2.207	-1.304	5.239	2.309	68.924	16.211	17.330
9575	238	37	30	C	W	24.791	-3.388	1.454	2.641	3.635	-1.381	-2.508	69.073	17.335	16.200
9576	238	37	30	C	W	25.285	-4.781	-5.497	4.263	5.287	5.066	-3.928	64.614	16.641	17.467
9577	238	37	30	C	W	34.715	-4.781	-5.497	4.263	5.287	5.066	-3.928	64.614	16.641	17.467
9578	238	37	30	C	W	35.209	-3.388	-1.454	2.641	3.635	1.381	-2.508	69.073	17.335	16.200
9579	238	37	30	C	W	38.948	1.339	5.008	-2.207	-1.304	-5.239	2.309	68.924	16.211	17.330
9580	238	37	30	C	W	40.158	-4.723	-0.319	4.193	5.216	0.294	-3.868	69.083	15.242	18.444
9581	238	37	30	C	W	43.463	-3.292	-5.476	2.532	3.523	5.212	-2.410	65.217	18.221	15.856
9582	238	37	30	C	W	43.898	2.853	-4.414	-3.575	-2.699	4.753	3.850	65.111	15.862	18.232
9583	238	37	30	C	W	46.102	-4.228	-0.747	3.607	4.619	0.697	-3.364	69.081	17.184	16.352
9584	238	37	30	C	W	52.411	0.334	0.652	-1.252	-0.332	-0.669	1.285	68.045	17.772	15.900
9585	238	37	30	C	W	67.154	-0.955	-1.421	0.029	0.973	1.420	-0.029	68.052	17.772	15.899
9586	238	37	30	C	W	73.898	-4.228	0.747	3.607	4.619	-0.697	-3.364	69.081	17.184	16.352
9587	238	37	30	C	W	76.102	2.853	4.414	-3.575	-2.699	-4.753	3.851	65.111	18.232	15.862
9588	238	37	30	C	W	76.537	-3.292	5.476	2.532	3.523	-5.212	-2.410	65.217	18.221	15.856

BL number	Atoms	γ-PC unit cells	WS_2 unit cells	γ-PC origin atom	WS_2 origin atom	Twist-angle (°)	γ-PC strain 1 (%)	γ-PC strain 2 (%)	γ-PC strain 3 (%)	WS_2 strain 1 (%)	WS_2 strain 2 (%)	WS_2 strain 3 (%)	γ (°)	$\vert\mathbf{a}\vert$(Å)	$\vert\mathbf{b}\vert$(Å)
9589	238	37	30	C	W	79.842	-4.723	0.319	4.193	5.216	-0.294	-3.868	69.083	15.242	18.444
9590	238	37	30	C	W	81.052	1.339	-5.008	-2.207	-1.304	5.239	2.309	68.924	16.211	17.330
9591	238	37	30	C	W	84.791	-3.388	1.454	2.641	3.635	-1.381	-2.508	69.073	17.335	16.200
9592	238	37	30	C	W	85.285	-4.781	-5.497	4.263	5.287	5.066	-3.928	64.614	16.641	17.467
9593	238	37	30	C	W	94.715	-4.781	-5.497	4.263	5.287	5.066	-3.928	64.614	16.641	17.467
9594	238	37	30	C	W	95.209	-3.388	-1.454	2.641	3.635	1.381	-2.508	69.073	17.335	16.200
9595	238	37	30	C	W	98.948	1.339	5.008	-2.207	-1.304	-5.239	2.309	68.924	16.211	17.330
9596	238	37	30	C	W	100.158	-4.723	-0.319	4.193	5.216	0.294	-3.868	69.083	15.242	18.444
9597	238	37	30	C	W	103.463	-3.292	-5.476	2.532	3.523	5.212	-2.410	65.217	18.221	15.856
9598	238	37	30	C	W	103.898	2.853	-4.414	-3.575	-2.699	4.753	3.850	65.111	15.862	18.232
9599	238	37	30	C	W	106.102	-4.228	-0.747	3.607	4.619	0.697	-3.364	69.081	17.184	16.352
9600	238	37	30	C	W	112.411	0.334	0.652	-1.252	-0.332	-0.669	1.285	68.045	17.772	15.900
9601	238	37	30	P	S	7.154	-0.955	-1.421	0.029	0.973	1.420	-0.029	68.052	17.772	15.899
9602	238	37	30	P	S	21.052	1.339	-5.008	-2.207	-1.304	5.239	2.309	68.924	16.211	17.330
9603	238	37	30	P	S	24.791	-3.388	1.454	2.641	3.635	-1.381	-2.508	69.073	17.335	16.200
9604	238	37	30	P	S	25.285	-4.781	-5.497	4.263	5.287	5.066	-3.928	64.614	16.641	17.467
9605	238	37	30	P	S	34.715	-4.781	-5.497	4.263	5.287	5.066	-3.928	64.614	16.641	17.467
9606	238	37	30	P	S	35.209	-3.388	-1.454	2.641	3.635	1.381	-2.508	69.073	17.335	16.200
9607	238	37	30	P	S	38.948	1.339	5.008	-2.207	-1.304	-5.239	2.309	68.924	16.211	17.330
9608	238	37	30	P	S	52.411	0.334	0.652	-1.252	-0.332	-0.669	1.285	68.045	17.772	15.900
9609	238	37	30	P	S	67.154	-0.955	-1.421	0.029	0.973	1.420	-0.029	68.052	17.772	15.899
9610	238	37	30	P	S	81.052	1.339	-5.008	-2.207	-1.304	5.239	2.309	68.924	16.211	17.330
9611	238	37	30	P	S	84.791	-3.388	1.454	2.641	3.635	-1.381	-2.508	69.073	17.335	16.200
9612	238	37	30	P	S	85.285	-4.781	-5.497	4.263	5.287	5.066	-3.928	64.614	16.641	17.467
9613	238	37	30	P	S	94.715	-4.781	-5.497	4.263	5.287	5.066	-3.928	64.614	16.641	17.467
9614	238	37	30	P	S	95.209	-3.388	-1.454	2.641	3.635	1.381	-2.508	69.073	17.335	16.200
9615	238	37	30	P	S	98.948	1.339	5.008	-2.207	-1.304	-5.239	2.309	68.924	16.211	17.330
9616	238	37	30	P	S	103.463	-3.292	-5.476	2.532	3.523	5.212	-2.410	65.217	18.221	15.856
9617	238	37	30	P	S	112.411	0.334	0.652	-1.252	-0.332	-0.669	1.285	68.045	17.772	15.900
9618	238	37	30	P	W	7.154	-0.955	-1.421	0.029	0.973	1.420	-0.029	68.052	17.772	15.899
9619	238	37	30	P	W	21.052	1.339	-5.008	-2.207	-1.304	5.239	2.309	68.924	16.211	17.330
9620	238	37	30	P	W	24.791	-3.388	1.454	2.641	3.635	-1.381	-2.508	69.073	17.335	16.200
9621	238	37	30	P	W	25.285	-4.781	-5.497	4.263	5.287	5.066	-3.928	64.614	16.641	17.467
9622	238	37	30	P	W	34.715	-4.781	-5.497	4.263	5.287	5.066	-3.928	64.614	16.641	17.467
9623	238	37	30	P	W	35.209	-3.388	-1.454	2.641	3.635	1.381	-2.508	69.073	17.335	16.200
9624	238	37	30	P	W	38.948	1.339	5.008	-2.207	-1.304	-5.239	2.309	68.924	16.211	17.330
9625	238	37	30	P	W	43.463	-3.292	-5.476	2.532	3.523	5.212	-2.410	65.217	18.221	15.856
9626	238	37	30	P	W	52.411	0.334	0.652	-1.252	-0.332	-0.669	1.285	68.045	17.772	15.900
9627	238	37	30	P	W	67.154	-0.955	-1.421	0.029	0.973	1.420	-0.029	68.052	17.772	15.899
9628	238	37	30	P	W	81.052	1.339	-5.008	-2.207	-1.304	5.239	2.309	68.924	16.211	17.330
9629	238	37	30	P	W	84.791	-3.388	1.454	2.641	3.635	-1.381	-2.508	69.073	17.335	16.200
9630	238	37	30	P	W	85.285	-4.781	-5.497	4.263	5.287	5.066	-3.928	64.614	16.641	17.467
9631	238	37	30	P	W	94.715	-4.781	-5.497	4.263	5.287	5.066	-3.928	64.614	16.641	17.467
9632	238	37	30	P	W	95.209	-3.388	-1.454	2.641	3.635	1.381	-2.508	69.073	17.335	16.200
9633	238	37	30	P	W	98.948	1.339	5.008	-2.207	-1.304	-5.239	2.309	68.924	16.211	17.330
9634	238	37	30	P	W	112.411	0.334	0.652	-1.252	-0.332	-0.669	1.285	68.045	17.772	15.900
9635	239	35	33	C	S	17.897	5.011	-3.464	1.868	-4.554	3.340	-1.800	79.405	16.799	16.210
9636	239	35	33	C	S	42.103	5.011	3.465	1.868	-4.554	-3.340	-1.800	79.405	16.799	16.210
9637	239	35	33	C	S	77.897	5.011	-3.464	1.868	-4.554	3.340	-1.800	79.405	16.799	16.210
9638	239	35	33	C	S	102.104	5.011	3.465	1.868	-4.554	-3.340	-1.800	79.405	16.799	16.210
9639	239	35	33	C	W	17.897	5.011	-3.464	1.868	-4.554	3.340	-1.800	79.405	16.799	16.210

BL number	Atoms	γ-PC unit cells	WS_2 unit cells	γ-PC origin atom	WS_2 origin atom	Twist-angle (°)	γ-PC strain 1 (%)	γ-PC strain 2 (%)	γ-PC strain 3 (%)	WS_2 strain 1 (%)	WS_2 strain 2 (%)	WS_2 strain 3 (%)	γ (°)	|**a**|(Å)	|**b**|(Å)
9640	239	35	33	C	W	42.103	5.011	3.465	1.868	-4.554	-3.340	-1.800	79.405	16.799	16.210
9641	239	35	33	C	W	77.897	5.011	-3.464	1.868	-4.554	3.340	-1.800	79.405	16.799	16.210
9642	239	35	33	C	W	102.104	5.011	3.465	1.868	-4.554	-3.340	-1.800	79.405	16.799	16.210
9643	239	35	33	P	S	17.897	5.011	-3.464	1.868	-4.554	3.340	-1.800	79.405	16.799	16.210
9644	239	35	33	P	S	42.103	5.011	3.465	1.868	-4.554	-3.340	-1.800	79.405	16.799	16.210
9645	239	35	33	P	S	77.897	5.011	-3.464	1.868	-4.554	3.340	-1.800	79.405	16.799	16.210
9646	239	35	33	P	S	102.104	5.011	3.465	1.868	-4.554	-3.340	-1.800	79.405	16.799	16.210
9647	239	35	33	P	W	17.897	5.011	-3.464	1.868	-4.554	3.340	-1.800	79.405	16.799	16.210
9648	239	35	33	P	W	42.103	5.011	3.465	1.868	-4.554	-3.340	-1.800	79.405	16.799	16.210
9649	239	35	33	P	W	77.897	5.011	-3.464	1.868	-4.554	3.340	-1.800	79.405	16.799	16.210
9650	239	35	33	P	W	102.104	5.011	3.465	1.868	-4.554	-3.340	-1.800	79.405	16.799	16.210
9651	239	38	29	C	S	16.337	0.353	-4.009	-4.135	-0.351	4.370	4.507	77.994	17.539	15.235
9652	239	38	29	C	S	17.480	-4.685	-2.210	0.964	5.169	2.169	-0.946	71.353	14.491	19.041
9653	239	38	29	C	S	17.696	1.029	-1.722	-4.742	-1.008	1.902	5.239	71.406	19.034	14.492
9654	239	38	29	C	S	19.842	-4.723	1.965	1.008	5.216	-1.926	-0.988	78.148	15.242	17.527
9655	239	38	29	C	S	25.050	-0.955	-4.005	-2.911	0.973	4.252	3.092	82.354	17.772	14.832
9656	239	38	29	C	S	30.000	-2.359	4.451	-1.523	2.476	-4.590	1.571	82.279	14.845	17.758
9657	239	38	29	C	S	34.950	-0.955	4.005	-2.911	0.973	-4.252	3.092	82.354	17.772	14.832
9658	239	38	29	C	S	40.158	-4.723	-1.965	1.008	5.216	1.926	-0.988	78.148	15.242	17.527
9659	239	38	29	C	S	42.304	1.029	1.722	-4.742	-1.008	-1.902	5.239	71.406	19.034	14.492
9660	239	38	29	C	S	42.520	-4.685	2.210	0.964	5.169	-2.169	-0.946	71.353	14.491	19.041
9661	239	38	29	C	S	43.663	0.353	4.009	-4.135	-0.351	-4.370	4.507	77.994	17.539	15.235
9662	239	38	29	C	S	76.337	0.353	-4.009	-4.135	-0.351	4.370	4.507	77.994	17.539	15.235
9663	239	38	29	C	S	77.480	-4.685	-2.210	0.964	5.169	2.169	-0.946	71.353	14.491	19.041
9664	239	38	29	C	S	77.696	1.029	-1.722	-4.742	-1.008	1.902	5.239	71.406	19.034	14.492
9665	239	38	29	C	S	79.842	-4.723	1.965	1.008	5.216	-1.926	-0.988	78.148	15.242	17.527
9666	239	38	29	C	S	85.050	-0.955	-4.005	-2.911	0.973	4.252	3.092	82.354	17.772	14.832
9667	239	38	29	C	S	90.000	-2.359	-4.450	-1.523	2.476	4.590	1.571	82.279	14.845	17.758
9668	239	38	29	C	S	94.950	-0.955	4.005	-2.911	0.973	-4.252	3.092	82.354	17.772	14.832
9669	239	38	29	C	S	100.158	-4.723	-1.965	1.008	5.216	1.926	-0.988	78.148	15.242	17.527
9670	239	38	29	C	S	102.304	1.029	1.722	-4.742	-1.008	-1.902	5.239	71.405	19.034	14.492
9671	239	38	29	C	S	102.520	-4.685	2.210	0.964	5.169	-2.169	-0.946	71.353	14.491	19.041
9672	239	38	29	C	S	103.663	0.353	4.009	-4.135	-0.351	-4.370	4.507	77.994	17.539	15.235
9673	239	38	29	C	W	16.337	0.353	-4.009	-4.135	-0.351	4.370	4.507	77.994	17.539	15.235
9674	239	38	29	C	W	17.480	-4.685	-2.210	0.964	5.169	2.169	-0.946	71.353	14.491	19.041
9675	239	38	29	C	W	17.696	1.029	-1.722	-4.742	-1.008	1.902	5.239	71.406	19.034	14.492
9676	239	38	29	C	W	19.842	-4.723	1.965	1.008	5.216	-1.926	-0.988	78.148	15.242	17.527
9677	239	38	29	C	W	25.050	-0.955	-4.005	-2.911	0.973	4.252	3.092	82.354	17.772	14.832
9678	239	38	29	C	W	30.000	-2.359	4.451	-1.523	2.476	-4.590	1.571	82.279	14.845	17.758
9679	239	38	29	C	W	34.950	-0.955	4.005	-2.911	0.973	-4.252	3.092	82.354	17.772	14.832
9680	239	38	29	C	W	40.158	-4.723	-1.965	1.008	5.216	1.926	-0.988	78.148	15.242	17.527
9681	239	38	29	C	W	42.304	1.029	1.722	-4.742	-1.008	-1.902	5.239	71.406	19.034	14.492
9682	239	38	29	C	W	42.520	-4.685	2.210	0.964	5.169	-2.169	-0.946	71.353	14.491	19.041
9683	239	38	29	C	W	43.663	0.353	4.009	-4.135	-0.351	-4.370	4.507	77.994	17.539	15.235
9684	239	38	29	C	W	76.337	0.353	-4.009	-4.135	-0.351	4.370	4.507	77.994	17.539	15.235
9685	239	38	29	C	W	77.480	-4.685	-2.210	0.964	5.169	2.169	-0.946	71.353	14.491	19.041
9686	239	38	29	C	W	77.696	1.029	-1.722	-4.742	-1.008	1.902	5.239	71.406	19.034	14.492
9687	239	38	29	C	W	79.842	-4.723	1.965	1.008	5.216	-1.926	-0.988	78.148	15.242	17.527
9688	239	38	29	C	W	85.050	-0.955	-4.005	-2.911	0.973	4.252	3.092	82.354	17.772	14.832
9689	239	38	29	C	W	90.000	-2.359	-4.450	-1.523	2.476	4.590	1.571	82.279	14.845	17.758
9690	239	38	29	C	W	94.950	-0.955	4.005	-2.911	0.973	-4.252	3.092	82.354	17.772	14.832

BL number	Atoms	γ-PC unit cells	WS_2 unit cells	γ-PC origin atom	WS_2 origin atom	Twist-angle (°)	γ-PC strain 1 (%)	γ-PC strain 2 (%)	γ-PC strain 3 (%)	WS_2 strain 1 (%)	WS_2 strain 2 (%)	WS_2 strain 3 (%)	γ (°)	|**a**|(Å)	|**b**|(Å)
9691	239	38	29	C	W	100.158	-4.723	-1.965	1.008	5.216	1.926	-0.988	78.148	15.242	17.527
9692	239	38	29	C	W	102.304	1.029	1.722	-4.742	-1.008	-1.902	5.239	71.405	19.034	14.492
9693	239	38	29	C	W	102.520	-4.685	2.210	0.964	5.169	-2.169	-0.946	71.353	14.491	19.041
9694	239	38	29	C	W	103.663	0.353	4.009	-4.135	-0.351	-4.370	4.507	77.994	17.539	15.235
9695	239	38	29	P	S	16.337	0.353	-4.009	-4.135	-0.351	4.370	4.507	77.994	17.539	15.235
9696	239	38	29	P	S	17.480	-4.685	-2.210	0.964	5.169	2.169	-0.946	71.353	14.491	19.041
9697	239	38	29	P	S	17.696	1.029	-1.722	-4.742	-1.008	1.902	5.239	71.406	19.034	14.492
9698	239	38	29	P	S	19.842	-4.723	1.965	1.008	5.216	-1.926	-0.988	78.148	15.242	17.527
9699	239	38	29	P	S	25.050	-0.955	-4.005	-2.911	0.973	4.252	3.092	82.354	17.772	14.832
9700	239	38	29	P	S	30.000	-2.359	4.451	-1.523	2.476	-4.590	1.571	82.279	14.845	17.758
9701	239	38	29	P	S	34.950	-0.955	4.005	-2.911	0.973	-4.252	3.092	82.354	17.772	14.832
9702	239	38	29	P	S	40.158	-4.723	-1.965	1.008	5.216	1.926	-0.988	78.148	15.242	17.527
9703	239	38	29	P	S	42.304	1.029	1.722	-4.742	-1.008	-1.902	5.239	71.406	19.034	14.492
9704	239	38	29	P	S	42.520	-4.685	2.210	0.964	5.169	-2.169	-0.946	71.353	14.491	19.041
9705	239	38	29	P	S	43.663	0.353	4.009	-4.135	-0.351	-4.370	4.507	77.994	17.539	15.235
9706	239	38	29	P	S	76.337	0.353	-4.009	-4.135	-0.351	4.370	4.507	77.994	17.539	15.235
9707	239	38	29	P	S	77.480	-4.685	-2.210	0.964	5.169	2.169	-0.946	71.353	14.491	19.041
9708	239	38	29	P	S	77.696	1.029	-1.722	-4.742	-1.008	1.902	5.239	71.406	19.034	14.492
9709	239	38	29	P	S	79.842	-4.723	1.965	1.008	5.216	-1.926	-0.988	78.148	15.242	17.527
9710	239	38	29	P	S	85.050	-0.955	-4.005	-2.911	0.973	4.252	3.092	82.354	17.772	14.832
9711	239	38	29	P	S	90.000	-2.359	-4.450	-1.523	2.476	4.590	1.571	82.279	14.845	17.758
9712	239	38	29	P	S	94.950	-0.955	4.005	-2.911	0.973	-4.252	3.092	82.354	17.772	14.832
9713	239	38	29	P	S	100.158	-4.723	-1.965	1.008	5.216	1.926	-0.988	78.148	15.242	17.527
9714	239	38	29	P	S	102.304	1.029	1.722	-4.742	-1.008	-1.902	5.239	71.405	19.034	14.492
9715	239	38	29	P	S	102.520	-4.685	2.210	0.964	5.169	-2.169	-0.946	71.353	14.491	19.041
9716	239	38	29	P	S	103.663	0.353	4.009	-4.135	-0.351	-4.370	4.507	77.994	17.539	15.235
9717	239	38	29	P	W	16.337	0.353	-4.009	-4.135	-0.351	4.370	4.507	77.994	17.539	15.235
9718	239	38	29	P	W	17.480	-4.685	-2.210	0.964	5.169	2.169	-0.946	71.353	14.491	19.041
9719	239	38	29	P	W	17.696	1.029	-1.722	-4.742	-1.008	1.902	5.239	71.406	19.034	14.492
9720	239	38	29	P	W	19.842	-4.723	1.965	1.008	5.216	-1.926	-0.988	78.148	15.242	17.527
9721	239	38	29	P	W	25.050	-0.955	-4.005	-2.911	0.973	4.252	3.092	82.354	17.772	14.832
9722	239	38	29	P	W	30.000	-2.359	4.451	-1.523	2.476	-4.590	1.571	82.279	14.845	17.758
9723	239	38	29	P	W	34.950	-0.955	4.005	-2.911	0.973	-4.252	3.092	82.354	17.772	14.832
9724	239	38	29	P	W	40.158	-4.723	-1.965	1.008	5.216	1.926	-0.988	78.148	15.242	17.527
9725	239	38	29	P	W	42.304	1.029	1.722	-4.742	-1.008	-1.902	5.239	71.406	19.034	14.492
9726	239	38	29	P	W	42.520	-4.685	2.210	0.964	5.169	-2.169	-0.946	71.353	14.491	19.041
9727	239	38	29	P	W	43.663	0.353	4.009	-4.135	-0.351	-4.370	4.507	77.994	17.539	15.235
9728	239	38	29	P	W	76.337	0.353	-4.009	-4.135	-0.351	4.370	4.507	77.994	17.539	15.235
9729	239	38	29	P	W	77.480	-4.685	-2.210	0.964	5.169	2.169	-0.946	71.353	14.491	19.041
9730	239	38	29	P	W	77.696	1.029	-1.722	-4.742	-1.008	1.902	5.239	71.406	19.034	14.492
9731	239	38	29	P	W	79.842	-4.723	1.965	1.008	5.216	-1.926	-0.988	78.148	15.242	17.527
9732	239	38	29	P	W	85.050	-0.955	-4.005	-2.911	0.973	4.252	3.092	82.354	17.772	14.832
9733	239	38	29	P	W	90.000	-2.359	-4.450	-1.523	2.476	4.590	1.571	82.279	14.845	17.758
9734	239	38	29	P	W	94.950	-0.955	4.005	-2.911	0.973	-4.252	3.092	82.354	17.772	14.832
9735	239	38	29	P	W	100.158	-4.723	-1.965	1.008	5.216	1.926	-0.988	78.148	15.242	17.527
9736	239	38	29	P	W	102.304	1.029	1.722	-4.742	-1.008	-1.902	5.239	71.405	19.034	14.492
9737	239	38	29	P	W	102.520	-4.685	2.210	0.964	5.169	-2.169	-0.946	71.353	14.491	19.041
9738	239	38	29	P	W	103.663	0.353	4.009	-4.135	-0.351	-4.370	4.507	77.994	17.539	15.235
9739	240	36	32	C	S	0.000	5.011	-5.052	-1.101	-4.554	5.166	1.126	81.287	13.826	19.558
9740	240	36	32	C	S	8.513	2.390	-1.389	1.345	-2.281	1.353	-1.310	69.568	16.358	17.422
9741	240	36	32	C	S	8.948	1.048	-0.627	2.695	-1.026	0.595	-2.557	69.564	17.420	16.362

BL number	Atoms	γ-PC unit cells	WS_2 unit cells	γ-PC origin atom	WS_2 origin atom	Twist-angle (°)	γ-PC strain 1 (%)	γ-PC strain 2 (%)	γ-PC strain 3 (%)	WS_2 strain 1 (%)	WS_2 strain 2 (%)	WS_2 strain 3 (%)	γ (°)	|**a**|(Å)	|**b**|(Å)
9742	240	36	32	C	S	10.158	2.281	1.523	1.452	-2.182	-1.480	-1.411	69.543	16.362	17.421
9743	240	36	32	C	S	49.842	2.281	-1.523	1.452	-2.182	1.480	-1.411	69.543	16.362	17.421
9744	240	36	32	C	S	51.487	2.390	1.389	1.345	-2.281	-1.353	-1.310	69.568	17.422	16.358
9745	240	36	32	C	S	60.000	5.011	-5.052	-1.101	-4.554	5.166	1.126	81.287	13.826	19.558
9746	240	36	32	C	S	68.513	2.390	-1.389	1.345	-2.281	1.353	-1.310	69.568	16.358	17.422
9747	240	36	32	C	S	70.158	2.281	1.523	1.452	-2.182	-1.480	-1.411	69.543	16.362	17.421
9748	240	36	32	C	S	109.842	2.281	-1.523	1.452	-2.182	1.480	-1.411	69.543	16.362	17.421
9749	240	36	32	C	S	111.052	1.048	0.627	2.695	-1.027	-0.595	-2.557	69.564	17.420	16.362
9750	240	36	32	C	S	111.487	2.390	1.389	1.345	-2.281	-1.353	-1.310	69.568	17.422	16.358
9751	240	36	32	C	W	0.000	5.011	-5.052	-1.101	-4.554	5.166	1.126	81.287	13.826	19.558
9752	240	36	32	C	W	8.513	2.390	-1.389	1.345	-2.281	1.353	-1.310	69.568	16.358	17.422
9753	240	36	32	C	W	10.158	2.281	1.523	1.452	-2.182	-1.480	-1.411	69.543	16.362	17.421
9754	240	36	32	C	W	49.842	2.281	-1.523	1.452	-2.182	1.480	-1.411	69.543	16.362	17.421
9755	240	36	32	C	W	51.052	1.048	0.627	2.695	-1.027	-0.595	-2.557	69.564	17.420	16.362
9756	240	36	32	C	W	51.487	2.390	1.389	1.345	-2.281	-1.353	-1.310	69.568	17.422	16.358
9757	240	36	32	C	W	60.000	5.011	-5.052	-1.101	-4.554	5.166	1.126	81.287	13.826	19.558
9758	240	36	32	C	W	68.513	2.390	-1.389	1.345	-2.281	1.353	-1.310	69.568	16.358	17.422
9759	240	36	32	C	W	68.948	1.048	-0.627	2.695	-1.026	0.595	-2.557	69.564	17.420	16.362
9760	240	36	32	C	W	70.158	2.281	1.523	1.452	-2.182	-1.480	-1.411	69.543	16.362	17.421
9761	240	36	32	C	W	109.842	2.281	-1.523	1.452	-2.182	1.480	-1.411	69.543	16.362	17.421
9762	240	36	32	C	W	111.487	2.390	1.389	1.345	-2.281	-1.353	-1.310	69.568	17.422	16.358
9763	240	36	32	P	S	0.000	5.011	-5.052	-1.101	-4.554	5.166	1.126	81.287	13.826	19.558
9764	240	36	32	P	S	8.513	2.390	-1.389	1.345	-2.281	1.353	-1.310	69.568	16.358	17.422
9765	240	36	32	P	S	10.158	2.281	1.523	1.452	-2.182	-1.480	-1.411	69.543	16.362	17.421
9766	240	36	32	P	S	49.842	2.281	-1.523	1.452	-2.182	1.480	-1.411	69.543	16.362	17.421
9767	240	36	32	P	S	51.052	1.048	0.627	2.695	-1.027	-0.595	-2.557	69.564	17.420	16.362
9768	240	36	32	P	S	51.487	2.390	1.389	1.345	-2.281	-1.353	-1.310	69.568	17.422	16.358
9769	240	36	32	P	S	68.513	2.390	-1.389	1.345	-2.281	1.353	-1.310	69.568	16.358	17.422
9770	240	36	32	P	S	68.948	1.048	-0.627	2.695	-1.026	0.595	-2.557	69.564	17.420	16.362
9771	240	36	32	P	S	70.158	2.281	1.523	1.452	-2.182	-1.480	-1.411	69.543	16.362	17.421
9772	240	36	32	P	S	109.842	2.281	-1.523	1.452	-2.182	1.480	-1.411	69.543	16.362	17.421
9773	240	36	32	P	S	111.487	2.390	1.389	1.345	-2.281	-1.353	-1.310	69.568	17.422	16.358
9774	240	36	32	P	W	8.513	2.390	-1.389	1.345	-2.281	1.353	-1.310	69.568	16.358	17.422
9775	240	36	32	P	W	8.948	1.048	-0.627	2.695	-1.026	0.595	-2.557	69.564	17.420	16.362
9776	240	36	32	P	W	10.158	2.281	1.523	1.452	-2.182	-1.480	-1.411	69.543	16.362	17.421
9777	240	36	32	P	W	49.842	2.281	-1.523	1.452	-2.182	1.480	-1.411	69.543	16.362	17.421
9778	240	36	32	P	W	51.487	2.390	1.389	1.345	-2.281	-1.353	-1.310	69.568	17.422	16.358
9779	240	36	32	P	W	60.000	5.011	-5.052	-1.101	-4.554	5.166	1.126	81.287	13.826	19.558
9780	240	36	32	P	W	68.513	2.390	-1.389	1.345	-2.281	1.353	-1.310	69.568	16.358	17.422
9781	240	36	32	P	W	70.158	2.281	1.523	1.452	-2.182	-1.480	-1.411	69.543	16.362	17.421
9782	240	36	32	P	W	109.842	2.281	-1.523	1.452	-2.182	1.480	-1.411	69.543	16.362	17.421
9783	240	36	32	P	W	111.052	1.048	0.627	2.695	-1.027	-0.595	-2.557	69.564	17.420	16.362
9784	240	36	32	P	W	111.487	2.390	1.389	1.345	-2.281	-1.353	-1.310	69.568	17.422	16.358
9785	240	39	28	C	S	3.004	-3.388	0.000	-3.388	3.635	0.000	3.635	60.000	17.335	17.335
9786	240	39	28	C	S	24.791	-3.388	0.000	-3.388	3.635	0.000	3.635	60.000	17.335	17.335
9787	240	39	28	C	S	35.209	-3.388	0.000	-3.388	3.635	0.000	3.635	60.000	17.335	17.335
9788	240	39	28	C	S	56.996	-3.388	0.000	-3.388	3.635	0.000	3.635	60.000	17.335	17.335
9789	240	39	28	C	S	63.005	-3.388	0.000	-3.388	3.635	0.000	3.635	60.000	17.335	17.335
9790	240	39	28	C	S	84.791	-3.388	0.000	-3.388	3.635	0.000	3.635	60.000	17.335	17.335
9791	240	39	28	C	S	95.209	-3.388	0.000	-3.388	3.635	0.000	3.635	60.000	17.335	17.335
9792	240	39	28	C	S	116.996	-3.388	0.000	-3.388	3.635	0.000	3.635	60.000	17.335	17.335

BL number	Atoms	γ-PC unit cells	WS_2 unit cells	γ-PC origin atom	WS_2 origin atom	Twist-angle (°)	γ-PC strain 1 (%)	γ-PC strain 2 (%)	γ-PC strain 3 (%)	WS_2 strain 1 (%)	WS_2 strain 2 (%)	WS_2 strain 3 (%)	γ (°)	|**a**|(Å)	|**b**|(Å)
9793	240	39	28	P	S	3.004	-3.388	0.000	-3.388	3.635	0.000	3.635	60.000	17.335	17.335
9794	240	39	28	P	S	24.791	-3.388	0.000	-3.388	3.635	0.000	3.635	60.000	17.335	17.335
9795	240	39	28	P	S	35.209	-3.388	0.000	-3.388	3.635	0.000	3.635	60.000	17.335	17.335
9796	240	39	28	P	S	56.996	-3.388	0.000	-3.388	3.635	0.000	3.635	60.000	17.335	17.335
9797	240	39	28	P	S	63.005	-3.388	0.000	-3.388	3.635	0.000	3.635	60.000	17.335	17.335
9798	240	39	28	P	S	84.791	-3.388	0.000	-3.388	3.635	0.000	3.635	60.000	17.335	17.335
9799	240	39	28	P	S	95.209	-3.388	0.000	-3.388	3.635	0.000	3.635	60.000	17.335	17.335
9800	240	39	28	P	S	116.996	-3.388	0.000	-3.388	3.635	0.000	3.635	60.000	17.335	17.335
9801	240	39	28	C	S	1.359	-3.292	-4.457	-3.485	3.523	4.791	3.746	85.009	18.221	14.337
9802	240	39	28	C	S	1.945	-4.723	-3.526	-2.014	5.216	3.674	2.099	87.939	15.242	17.089
9803	240	39	28	C	S	6.178	-2.145	3.621	-4.599	2.241	-3.988	5.065	87.826	17.102	15.231
9804	240	39	28	C	S	6.587	-3.853	4.382	-2.919	4.175	-4.654	3.100	85.039	14.354	18.199
9805	240	39	28	C	S	15.608	-2.145	-3.622	-4.599	2.241	3.988	5.065	87.826	17.102	15.231
9806	240	39	28	C	S	19.842	-4.723	3.526	-2.014	5.216	-3.674	2.099	87.939	15.242	17.089
9807	240	39	28	C	S	40.158	-4.723	-3.526	-2.014	5.216	3.674	2.099	87.939	15.242	17.089
9808	240	39	28	C	S	44.392	-2.145	3.621	-4.599	2.241	-3.988	5.065	87.826	17.102	15.231
9809	240	39	28	C	S	53.413	-3.853	-4.382	-2.919	4.175	4.654	3.100	85.039	14.354	18.199
9810	240	39	28	C	S	53.822	-2.145	-3.622	-4.599	2.241	3.988	5.065	87.826	17.102	15.231
9811	240	39	28	C	S	58.055	-4.723	3.526	-2.014	5.216	-3.674	2.099	87.939	15.242	17.089
9812	240	39	28	C	S	58.641	-3.292	4.457	-3.485	3.523	-4.791	3.746	85.009	18.221	14.337
9813	240	39	28	C	S	61.359	-3.292	-4.457	-3.485	3.523	4.791	3.746	85.009	18.221	14.337
9814	240	39	28	C	S	61.945	-4.723	-3.526	-2.014	5.216	3.674	2.099	87.939	15.242	17.089
9815	240	39	28	C	S	66.178	-2.145	3.621	-4.599	2.241	-3.988	5.065	87.826	17.102	15.231
9816	240	39	28	C	S	66.587	-3.853	4.382	-2.919	4.175	-4.654	3.100	85.039	14.354	18.199
9817	240	39	28	C	S	75.609	-2.145	-3.622	-4.599	2.241	3.988	5.065	87.826	17.102	15.231
9818	240	39	28	C	S	79.842	-4.723	3.526	-2.014	5.216	-3.674	2.099	87.939	15.242	17.089
9819	240	39	28	C	S	100.158	-4.723	-3.526	-2.014	5.216	3.674	2.099	87.939	15.242	17.089
9820	240	39	28	C	S	104.392	-2.145	3.621	-4.599	2.241	-3.988	5.065	87.826	17.102	15.231
9821	240	39	28	C	S	113.413	-3.853	-4.382	-2.919	4.175	4.654	3.100	85.039	14.354	18.199
9822	240	39	28	C	S	113.822	-2.145	-3.622	-4.599	2.241	3.988	5.065	87.826	17.102	15.231
9823	240	39	28	C	S	118.055	-4.723	3.526	-2.014	5.216	-3.674	2.099	87.939	15.242	17.089
9824	240	39	28	C	S	118.641	-3.292	4.457	-3.485	3.523	-4.791	3.746	85.009	18.221	14.337
9825	240	39	28	C	W	1.359	-3.292	-4.457	-3.485	3.523	4.791	3.746	85.009	18.221	14.337
9826	240	39	28	C	W	1.945	-4.723	-3.526	-2.014	5.216	3.674	2.099	87.939	15.242	17.089
9827	240	39	28	C	W	6.178	-2.145	3.621	-4.599	2.241	-3.988	5.065	87.826	17.102	15.231
9828	240	39	28	C	W	6.587	-3.853	4.382	-2.919	4.175	-4.654	3.100	85.039	14.354	18.199
9829	240	39	28	C	W	15.608	-2.145	-3.622	-4.599	2.241	3.988	5.065	87.826	17.102	15.231
9830	240	39	28	C	W	19.842	-4.723	3.526	-2.014	5.216	-3.674	2.099	87.939	15.242	17.089
9831	240	39	28	C	W	40.158	-4.723	-3.526	-2.014	5.216	3.674	2.099	87.939	15.242	17.089
9832	240	39	28	C	W	44.392	-2.145	3.621	-4.599	2.241	-3.988	5.065	87.826	17.102	15.231
9833	240	39	28	C	W	53.413	-3.853	-4.382	-2.919	4.175	4.654	3.100	85.039	14.354	18.199
9834	240	39	28	C	W	53.822	-2.145	-3.622	-4.599	2.241	3.988	5.065	87.826	17.102	15.231
9835	240	39	28	C	W	58.055	-4.723	3.526	-2.014	5.216	-3.674	2.099	87.939	15.242	17.089
9836	240	39	28	C	W	58.641	-3.292	4.457	-3.485	3.523	-4.791	3.746	85.009	18.221	14.337
9837	240	39	28	C	W	61.359	-3.292	-4.457	-3.485	3.523	4.791	3.746	85.009	18.221	14.337
9838	240	39	28	C	W	61.945	-4.723	-3.526	-2.014	5.216	3.674	2.099	87.939	15.242	17.089
9839	240	39	28	C	W	66.178	-2.145	3.621	-4.599	2.241	-3.988	5.065	87.826	17.102	15.231
9840	240	39	28	C	W	66.587	-3.853	4.382	-2.919	4.175	-4.654	3.100	85.039	14.354	18.199
9841	240	39	28	C	W	75.609	-2.145	-3.622	-4.599	2.241	3.988	5.065	87.826	17.102	15.231
9842	240	39	28	C	W	79.842	-4.723	3.526	-2.014	5.216	-3.674	2.099	87.939	15.242	17.089
9843	240	39	28	C	W	100.158	-4.723	-3.526	-2.014	5.216	3.674	2.099	87.939	15.242	17.089

BL number	Atoms	γ-PC unit cells	WS_2 unit cells	γ-PC origin atom	WS_2 origin atom	Twist-angle (°)	γ-PC strain 1 (%)	γ-PC strain 2 (%)	γ-PC strain 3 (%)	WS_2 strain 1 (%)	WS_2 strain 2 (%)	WS_2 strain 3 (%)	γ (°)	|**a**|(Å)	|**b**|(Å)
9844	240	39	28	C	W	104.392	-2.145	3.621	-4.599	2.241	-3.988	5.065	87.826	17.102	15.231
9845	240	39	28	C	W	113.413	-3.853	-4.382	-2.919	4.175	4.654	3.100	85.039	14.354	18.199
9846	240	39	28	C	W	113.822	-2.145	-3.622	-4.599	2.241	3.988	5.065	87.826	17.102	15.231
9847	240	39	28	C	W	118.055	-4.723	3.526	-2.014	5.216	-3.674	2.099	87.939	15.242	17.089
9848	240	39	28	C	W	118.641	-3.292	4.457	-3.485	3.523	-4.791	3.746	85.009	18.221	14.337
9849	240	39	28	P	S	1.359	-3.292	-4.457	-3.485	3.523	4.791	3.746	85.009	18.221	14.337
9850	240	39	28	P	S	1.945	-4.723	-3.526	-2.014	5.216	3.674	2.099	87.939	15.242	17.089
9851	240	39	28	P	S	6.178	-2.145	3.621	-4.599	2.241	-3.988	5.065	87.826	17.102	15.231
9852	240	39	28	P	S	6.587	-3.853	4.382	-2.919	4.175	-4.654	3.100	85.039	14.354	18.199
9853	240	39	28	P	S	15.608	-2.145	-3.622	-4.599	2.241	3.988	5.065	87.826	17.102	15.231
9854	240	39	28	P	S	19.842	-4.723	3.526	-2.014	5.216	-3.674	2.099	87.939	15.242	17.089
9855	240	39	28	P	S	40.158	-4.723	-3.526	-2.014	5.216	3.674	2.099	87.939	15.242	17.089
9856	240	39	28	P	S	44.392	-2.145	3.621	-4.599	2.241	-3.988	5.065	87.826	17.102	15.231
9857	240	39	28	P	S	53.413	-3.853	-4.382	-2.919	4.175	4.654	3.100	85.039	14.354	18.199
9858	240	39	28	P	S	53.822	-2.145	-3.622	-4.599	2.241	3.988	5.065	87.826	17.102	15.231
9859	240	39	28	P	S	58.055	-4.723	3.526	-2.014	5.216	-3.674	2.099	87.939	15.242	17.089
9860	240	39	28	P	S	58.641	-3.292	4.457	-3.485	3.523	-4.791	3.746	85.009	18.221	14.337
9861	240	39	28	P	S	61.359	-3.292	-4.457	-3.485	3.523	4.791	3.746	85.009	18.221	14.337
9862	240	39	28	P	S	61.945	-4.723	-3.526	-2.014	5.216	3.674	2.099	87.939	15.242	17.089
9863	240	39	28	P	S	66.178	-2.145	3.621	-4.599	2.241	-3.988	5.065	87.826	17.102	15.231
9864	240	39	28	P	S	66.587	-3.853	4.382	-2.919	4.175	-4.654	3.100	85.039	14.354	18.199
9865	240	39	28	P	S	75.609	-2.145	-3.622	-4.599	2.241	3.988	5.065	87.826	17.102	15.231
9866	240	39	28	P	S	79.842	-4.723	3.526	-2.014	5.216	-3.674	2.099	87.939	15.242	17.089
9867	240	39	28	P	S	100.158	-4.723	-3.526	-2.014	5.216	3.674	2.099	87.939	15.242	17.089
9868	240	39	28	P	S	104.392	-2.145	3.621	-4.599	2.241	-3.988	5.065	87.826	17.102	15.231
9869	240	39	28	P	S	113.413	-3.853	-4.382	-2.919	4.175	4.654	3.100	85.039	14.354	18.199
9870	240	39	28	P	S	113.822	-2.145	-3.622	-4.599	2.241	3.988	5.065	87.826	17.102	15.231
9871	240	39	28	P	S	118.055	-4.723	3.526	-2.014	5.216	-3.674	2.099	87.939	15.242	17.089
9872	240	39	28	P	S	118.641	-3.292	4.457	-3.485	3.523	-4.791	3.746	85.009	18.221	14.337
9873	240	39	28	P	W	1.359	-3.292	-4.457	-3.485	3.523	4.791	3.746	85.009	18.221	14.337
9874	240	39	28	P	W	1.945	-4.723	-3.526	-2.014	5.216	3.674	2.099	87.939	15.242	17.089
9875	240	39	28	P	W	6.178	-2.145	3.621	-4.599	2.241	-3.988	5.065	87.826	17.102	15.231
9876	240	39	28	P	W	6.587	-3.853	4.382	-2.919	4.175	-4.654	3.100	85.039	14.354	18.199
9877	240	39	28	P	W	15.608	-2.145	-3.622	-4.599	2.241	3.988	5.065	87.826	17.102	15.231
9878	240	39	28	P	W	19.842	-4.723	3.526	-2.014	5.216	-3.674	2.099	87.939	15.242	17.089
9879	240	39	28	P	W	40.158	-4.723	-3.526	-2.014	5.216	3.674	2.099	87.939	15.242	17.089
9880	240	39	28	P	W	44.392	-2.145	3.621	-4.599	2.241	-3.988	5.065	87.826	17.102	15.231
9881	240	39	28	P	W	53.413	-3.853	-4.382	-2.919	4.175	4.654	3.100	85.039	14.354	18.199
9882	240	39	28	P	W	53.822	-2.145	-3.622	-4.599	2.241	3.988	5.065	87.826	17.102	15.231
9883	240	39	28	P	W	58.055	-4.723	3.526	-2.014	5.216	-3.674	2.099	87.939	15.242	17.089
9884	240	39	28	P	W	58.641	-3.292	4.457	-3.485	3.523	-4.791	3.746	85.009	18.221	14.337
9885	240	39	28	P	W	61.359	-3.292	-4.457	-3.485	3.523	4.791	3.746	85.009	18.221	14.337
9886	240	39	28	P	W	61.945	-4.723	-3.526	-2.014	5.216	3.674	2.099	87.939	15.242	17.089
9887	240	39	28	P	W	66.178	-2.145	3.621	-4.599	2.241	-3.988	5.065	87.826	17.102	15.231
9888	240	39	28	P	W	66.587	-3.853	4.382	-2.919	4.175	-4.654	3.100	85.039	14.354	18.199
9889	240	39	28	P	W	75.609	-2.145	-3.622	-4.599	2.241	3.988	5.065	87.826	17.102	15.231
9890	240	39	28	P	W	79.842	-4.723	3.526	-2.014	5.216	-3.674	2.099	87.939	15.242	17.089
9891	240	39	28	P	W	100.158	-4.723	-3.526	-2.014	5.216	3.674	2.099	87.939	15.242	17.089
9892	240	39	28	P	W	104.392	-2.145	3.621	-4.599	2.241	-3.988	5.065	87.826	17.102	15.231
9893	240	39	28	P	W	113.413	-3.853	-4.382	-2.919	4.175	4.654	3.100	85.039	14.354	18.199
9894	240	39	28	P	W	113.822	-2.145	-3.622	-4.599	2.241	3.988	5.065	87.826	17.102	15.231

BL number	Atoms	γ-PC unit cells	WS_2 unit cells	γ-PC origin atom	WS_2 origin atom	Twist-angle (°)	γ-PC strain 1 (%)	γ-PC strain 2 (%)	γ-PC strain 3 (%)	WS_2 strain 1 (%)	WS_2 strain 2 (%)	WS_2 strain 3 (%)	γ (°)	|**a**|(Å)	|**b**|(Å)
9895	240	39	28	P	W	118.055	-4.723	3.526	-2.014	5.216	-3.674	2.099	87.939	15.242	17.089
9896	240	39	28	P	W	118.641	-3.292	4.457	-3.485	3.523	-4.791	3.746	85.009	18.221	14.337
9897	241	37	31	C	S	16.337	0.353	0.000	0.353	-0.351	0.000	-0.351	60.000	17.539	17.539
9898	241	37	31	C	S	25.767	0.353	0.000	0.353	-0.351	0.000	-0.351	60.000	17.539	17.539
9899	241	37	31	C	S	34.233	0.353	0.000	0.353	-0.351	0.000	-0.351	60.000	17.539	17.539
9900	241	37	31	C	S	43.663	0.353	0.000	0.353	-0.351	0.000	-0.351	60.000	17.539	17.539
9901	241	37	31	C	S	76.337	0.353	0.000	0.353	-0.351	0.000	-0.351	60.000	17.539	17.539
9902	241	37	31	C	S	85.767	0.353	0.000	0.353	-0.351	0.000	-0.351	60.000	17.539	17.539
9903	241	37	31	C	S	94.233	0.353	0.000	0.353	-0.351	0.000	-0.351	60.000	17.539	17.539
9904	241	37	31	C	S	103.663	0.353	0.000	0.353	-0.351	0.000	-0.351	60.000	17.539	17.539
9905	241	37	31	C	W	16.337	0.353	0.000	0.353	-0.351	0.000	-0.351	60.000	17.539	17.539
9906	241	37	31	C	W	25.767	0.353	0.000	0.353	-0.351	0.000	-0.351	60.000	17.539	17.539
9907	241	37	31	C	W	34.233	0.353	0.000	0.353	-0.351	0.000	-0.351	60.000	17.539	17.539
9908	241	37	31	C	W	43.663	0.353	0.000	0.353	-0.351	0.000	-0.351	60.000	17.539	17.539
9909	241	37	31	C	W	76.337	0.353	0.000	0.353	-0.351	0.000	-0.351	60.000	17.539	17.539
9910	241	37	31	C	W	85.767	0.353	0.000	0.353	-0.351	0.000	-0.351	60.000	17.539	17.539
9911	241	37	31	C	W	94.233	0.353	0.000	0.353	-0.351	0.000	-0.351	60.000	17.539	17.539
9912	241	37	31	C	W	103.663	0.353	0.000	0.353	-0.351	0.000	-0.351	60.000	17.539	17.539
9913	241	37	31	P	S	16.337	0.353	0.000	0.353	-0.351	0.000	-0.351	60.000	17.539	17.539
9914	241	37	31	P	S	34.233	0.353	0.000	0.353	-0.351	0.000	-0.351	60.000	17.539	17.539
9915	241	37	31	P	S	85.767	0.353	0.000	0.353	-0.351	0.000	-0.351	60.000	17.539	17.539
9916	241	37	31	P	S	103.663	0.353	0.000	0.353	-0.351	0.000	-0.351	60.000	17.539	17.539
9917	241	37	31	P	W	25.767	0.353	0.000	0.353	-0.351	0.000	-0.351	60.000	17.539	17.539
9918	241	37	31	P	W	43.663	0.353	0.000	0.353	-0.351	0.000	-0.351	60.000	17.539	17.539
9919	241	37	31	P	W	76.337	0.353	0.000	0.353	-0.351	0.000	-0.351	60.000	17.539	17.539
9920	241	37	31	P	W	94.233	0.353	0.000	0.353	-0.351	0.000	-0.351	60.000	17.539	17.539
9921	241	37	31	C	S	0.000	5.011	3.277	-3.910	-4.554	-3.555	4.241	63.686	16.799	17.726
9922	241	37	31	C	S	17.897	5.011	-3.277	-3.910	-4.554	3.555	4.241	63.686	16.799	17.726
9923	241	37	31	C	S	42.103	5.011	3.277	-3.910	-4.554	-3.555	4.241	63.686	16.799	17.726
9924	241	37	31	C	S	60.000	5.011	3.277	-3.910	-4.554	-3.555	4.241	63.686	16.799	17.726
9925	241	37	31	C	S	77.897	5.011	-3.277	-3.910	-4.554	3.555	4.241	63.686	16.799	17.726
9926	241	37	31	C	S	102.104	5.011	3.277	-3.910	-4.554	-3.555	4.241	63.686	16.799	17.726
9927	241	37	31	C	W	0.000	5.011	3.277	-3.910	-4.554	-3.555	4.241	63.686	16.799	17.726
9928	241	37	31	C	W	17.897	5.011	-3.277	-3.910	-4.554	3.555	4.241	63.686	16.799	17.726
9929	241	37	31	C	W	42.103	5.011	3.277	-3.910	-4.554	-3.555	4.241	63.686	16.799	17.726
9930	241	37	31	C	W	60.000	5.011	3.277	-3.910	-4.554	-3.555	4.241	63.686	16.799	17.726
9931	241	37	31	C	W	77.897	5.011	-3.277	-3.910	-4.554	3.555	4.241	63.686	16.799	17.726
9932	241	37	31	C	W	102.104	5.011	3.277	-3.910	-4.554	-3.555	4.241	63.686	16.799	17.726
9933	241	37	31	P	S	0.000	5.011	3.277	-3.910	-4.554	-3.555	4.241	63.686	16.799	17.726
9934	241	37	31	P	S	17.897	5.011	-3.277	-3.910	-4.554	3.555	4.241	63.686	16.799	17.726
9935	241	37	31	P	S	42.103	5.011	3.277	-3.910	-4.554	-3.555	4.241	63.686	16.799	17.726
9936	241	37	31	P	S	60.000	5.011	3.277	-3.910	-4.554	-3.555	4.241	63.686	16.799	17.726
9937	241	37	31	P	S	77.897	5.011	-3.277	-3.910	-4.554	3.555	4.241	63.686	16.799	17.726
9938	241	37	31	P	S	102.104	5.011	3.277	-3.910	-4.554	-3.555	4.241	63.686	16.799	17.726
9939	241	37	31	P	W	0.000	5.011	3.277	-3.910	-4.554	-3.555	4.241	63.686	16.799	17.726
9940	241	37	31	P	W	17.897	5.011	-3.277	-3.910	-4.554	3.555	4.241	63.686	16.799	17.726
9941	241	37	31	P	W	42.103	5.011	3.277	-3.910	-4.554	-3.555	4.241	63.686	16.799	17.726
9942	241	37	31	P	W	60.000	5.011	3.277	-3.910	-4.554	-3.555	4.241	63.686	16.799	17.726
9943	241	37	31	P	W	77.897	5.011	-3.277	-3.910	-4.554	3.555	4.241	63.686	16.799	17.726
9944	241	37	31	P	W	102.104	5.011	3.277	-3.910	-4.554	-3.555	4.241	63.686	16.799	17.726
9945	242	38	30	C	S	6.178	-2.145	-2.071	-0.076	2.241	2.074	0.076	77.738	17.102	15.895

BL number	Atoms	γ-PC unit cells	WS_2 unit cells	γ-PC origin atom	WS_2 origin atom	Twist-angle (°)	γ-PC strain 1 (%)	γ-PC strain 2 (%)	γ-PC strain 3 (%)	WS_2 strain 1 (%)	WS_2 strain 2 (%)	WS_2 strain 3 (%)	γ (°)	$\lvert\mathbf{a}\rvert$(Å)	$\lvert\mathbf{b}\rvert$(Å)
9946	242	38	30	C	S	7.589	0.334	0.381	-2.535	-0.332	-0.402	2.671	71.212	18.904	14.845
9947	242	38	30	C	S	8.213	-2.359	1.483	0.148	2.476	-1.479	-0.148	71.186	14.845	18.905
9948	242	38	30	C	S	8.948	-0.599	2.718	-1.639	0.606	-2.811	1.694	77.700	15.901	17.096
9949	242	38	30	C	S	10.158	2.281	3.052	-4.303	-2.182	-3.340	4.708	73.737	16.362	16.929
9950	242	38	30	C	S	13.898	-4.228	-4.365	2.196	4.619	4.182	-2.104	87.554	17.184	15.487
9951	242	38	30	C	S	16.102	2.853	-0.391	-4.797	-2.699	0.432	5.306	84.143	18.455	14.490
9952	242	38	30	C	S	16.537	-3.292	-4.218	1.149	3.523	4.124	-1.124	70.136	18.221	15.508
9953	242	38	30	C	S	17.480	-4.685	2.058	2.722	5.169	-1.952	-2.581	84.027	14.491	18.455
9954	242	38	30	C	S	19.107	1.980	-4.029	-4.038	-1.905	4.383	4.393	82.095	15.504	17.312
9955	242	38	30	C	S	21.052	1.339	3.593	-3.464	-1.304	-3.860	3.722	73.701	16.211	17.080
9956	242	38	30	C	S	25.285	-4.781	-0.868	2.834	5.287	0.821	-2.683	77.776	16.641	16.356
9957	242	38	30	C	S	28.055	2.281	3.830	-4.303	-2.182	-4.190	4.708	77.603	16.362	16.640
9958	242	38	30	C	S	31.945	2.281	-3.830	-4.303	-2.182	4.190	4.708	77.603	16.362	16.639
9959	242	38	30	C	S	34.715	-4.781	0.868	2.834	5.287	-0.822	-2.683	77.776	16.641	16.356
9960	242	38	30	C	S	38.948	1.339	-3.592	-3.464	-1.304	3.860	3.722	73.701	16.211	17.080
9961	242	38	30	C	S	40.893	1.980	-4.648	-4.038	-1.905	5.057	4.393	87.227	15.504	17.167
9962	242	38	30	C	S	42.520	-4.685	-2.058	2.722	5.169	1.952	-2.581	84.027	14.491	18.455
9963	242	38	30	C	S	43.463	-3.292	4.218	1.149	3.523	-4.124	-1.124	70.136	18.221	15.508
9964	242	38	30	C	S	43.898	2.853	0.391	-4.797	-2.699	-0.432	5.306	84.143	18.455	14.490
9965	242	38	30	C	S	46.102	-4.228	4.365	2.196	4.619	-4.182	-2.104	87.554	17.184	15.487
9966	242	38	30	C	S	49.842	2.281	-3.052	-4.303	-2.182	3.340	4.708	73.737	16.362	16.929
9967	242	38	30	C	S	51.052	-0.599	-2.718	-1.639	0.606	2.811	1.694	77.700	15.901	17.096
9968	242	38	30	C	S	51.787	-2.359	-1.483	0.148	2.476	1.479	-0.148	71.186	14.845	18.905
9969	242	38	30	C	S	52.411	0.334	-0.381	-2.535	-0.332	0.401	2.671	71.212	18.904	14.845
9970	242	38	30	C	S	53.822	-2.145	2.071	-0.076	2.241	-2.074	0.076	77.738	17.102	15.895
9971	242	38	30	C	S	66.178	-2.145	-2.071	-0.076	2.241	2.074	0.076	77.738	17.102	15.895
9972	242	38	30	C	S	67.589	0.334	0.381	-2.535	-0.332	-0.402	2.671	71.212	18.904	14.845
9973	242	38	30	C	S	68.213	-2.359	1.483	0.148	2.476	-1.479	-0.148	71.186	14.845	18.905
9974	242	38	30	C	S	68.948	-0.599	2.718	-1.639	0.606	-2.811	1.694	77.700	15.901	17.096
9975	242	38	30	C	S	70.158	2.281	3.052	-4.303	-2.182	-3.340	4.708	73.737	16.362	16.929
9976	242	38	30	C	S	73.898	-4.228	-4.365	2.196	4.619	4.182	-2.104	87.554	17.184	15.487
9977	242	38	30	C	S	76.102	2.853	-0.391	-4.797	-2.699	0.432	5.306	84.143	18.455	14.490
9978	242	38	30	C	S	76.537	-3.292	-4.218	1.149	3.523	4.124	-1.124	70.136	18.221	15.508
9979	242	38	30	C	S	77.480	-4.685	2.058	2.722	5.169	-1.952	-2.581	84.027	14.491	18.455
9980	242	38	30	C	S	79.107	1.980	-4.029	-4.038	-1.905	4.383	4.393	82.095	15.504	17.312
9981	242	38	30	C	S	81.052	1.339	3.593	-3.464	-1.304	-3.860	3.722	73.701	16.211	17.080
9982	242	38	30	C	S	85.285	-4.781	-0.868	2.834	5.287	0.821	-2.683	77.776	16.641	16.356
9983	242	38	30	C	S	88.055	2.281	3.830	-4.303	-2.182	-4.190	4.708	77.603	16.362	16.639
9984	242	38	30	C	S	91.945	2.281	-3.830	-4.303	-2.182	4.190	4.708	77.603	16.362	16.639
9985	242	38	30	C	S	94.715	-4.781	0.868	2.834	5.287	-0.822	-2.683	77.776	16.641	16.356
9986	242	38	30	C	S	98.948	1.339	-3.592	-3.464	-1.304	3.860	3.722	73.701	16.211	17.080
9987	242	38	30	C	S	100.893	1.980	-4.648	-4.038	-1.905	5.057	4.393	87.227	15.504	17.167
9988	242	38	30	C	S	102.520	-4.685	-2.058	2.722	5.169	1.952	-2.581	84.027	14.491	18.455
9989	242	38	30	C	S	103.463	-3.292	4.218	1.149	3.523	-4.124	-1.124	70.136	18.221	15.508
9990	242	38	30	C	S	103.898	2.853	0.391	-4.797	-2.699	-0.432	5.306	84.143	18.455	14.490
9991	242	38	30	C	S	106.102	-4.228	4.365	2.196	4.619	-4.182	-2.104	87.554	17.184	15.487
9992	242	38	30	C	S	109.842	2.281	-3.052	-4.303	-2.182	3.340	4.708	73.737	16.362	16.929
9993	242	38	30	C	S	111.052	-0.599	-2.718	-1.639	0.606	2.811	1.694	77.700	15.901	17.096
9994	242	38	30	C	S	111.787	-2.359	-1.483	0.148	2.476	1.479	-0.148	71.186	14.845	18.905
9995	242	38	30	C	S	112.411	0.334	-0.381	-2.535	-0.332	0.401	2.671	71.212	18.904	14.845
9996	242	38	30	C	S	113.822	-2.145	2.071	-0.076	2.241	-2.074	0.076	77.738	17.102	15.895

BL number	Atoms	γ-PC unit cells	WS_2 unit cells	γ-PC origin atom	WS_2 origin atom	Twist-angle (°)	γ-PC strain 1 (%)	γ-PC strain 2 (%)	γ-PC strain 3 (%)	WS_2 strain 1 (%)	WS_2 strain 2 (%)	WS_2 strain 3 (%)	γ (°)	|**a**|(Å)	|**b**|(Å)
9997	242	38	30	C	W	6.178	-2.145	-2.071	-0.076	2.241	2.074	0.076	77.738	17.102	15.895
9998	242	38	30	C	W	7.589	0.334	0.381	-2.535	-0.332	-0.402	2.671	71.212	18.904	14.845
9999	242	38	30	C	W	8.213	-2.359	1.483	0.148	2.476	-1.479	-0.148	71.186	14.845	18.905
10000	242	38	30	C	W	8.948	-0.599	2.718	-1.639	0.606	-2.811	1.694	77.700	15.901	17.096
10001	242	38	30	C	W	10.158	2.281	3.052	-4.303	-2.182	-3.340	4.708	73.737	16.362	16.929
10002	242	38	30	C	W	13.898	-4.228	-4.365	2.196	4.619	4.182	-2.104	87.554	17.184	15.487
10003	242	38	30	C	W	16.102	2.853	-0.391	-4.797	-2.699	0.432	5.306	84.143	18.455	14.490
10004	242	38	30	C	W	16.537	-3.292	-4.218	1.149	3.523	4.124	-1.124	70.136	18.221	15.508
10005	242	38	30	C	W	17.480	-4.685	2.058	2.722	5.169	-1.952	-2.581	84.027	14.491	18.455
10006	242	38	30	C	W	19.107	1.980	-4.029	-4.038	-1.905	4.383	4.393	82.095	15.504	17.312
10007	242	38	30	C	W	21.052	1.339	3.593	-3.464	-1.304	-3.860	3.722	73.701	16.211	17.080
10008	242	38	30	C	W	25.285	-4.781	-0.868	2.834	5.287	0.821	-2.683	77.776	16.641	16.356
10009	242	38	30	C	W	28.055	2.281	3.830	-4.303	-2.182	-4.190	4.708	77.603	16.362	16.640
10010	242	38	30	C	W	31.945	2.281	-3.830	-4.303	-2.182	4.190	4.708	77.603	16.362	16.639
10011	242	38	30	C	W	34.715	-4.781	0.868	2.834	5.287	-0.822	-2.683	77.776	16.641	16.356
10012	242	38	30	C	W	38.948	1.339	-3.592	-3.464	-1.304	3.860	3.722	73.701	16.211	17.080
10013	242	38	30	C	W	40.893	1.980	-4.648	-4.038	-1.905	5.057	4.393	87.227	15.504	17.167
10014	242	38	30	C	W	42.520	-4.685	-2.058	2.722	5.169	1.952	-2.581	84.027	14.491	18.455
10015	242	38	30	C	W	43.463	-3.292	4.218	1.149	3.523	-4.124	-1.124	70.136	18.221	15.508
10016	242	38	30	C	W	43.898	2.853	0.391	-4.797	-2.699	-0.432	5.306	84.143	18.455	14.490
10017	242	38	30	C	W	46.102	-4.228	4.365	2.196	4.619	-4.182	-2.104	87.554	17.184	15.487
10018	242	38	30	C	W	49.842	2.281	-3.052	-4.303	-2.182	3.340	4.708	73.737	16.362	16.929
10019	242	38	30	C	W	51.052	-0.599	-2.718	-1.639	0.606	2.811	1.694	77.700	15.901	17.096
10020	242	38	30	C	W	51.787	-2.359	-1.483	0.148	2.476	1.479	-0.148	71.186	14.845	18.905
10021	242	38	30	C	W	52.411	0.334	-0.381	-2.535	-0.332	0.401	2.671	71.212	18.904	14.845
10022	242	38	30	C	W	53.822	-2.145	2.071	-0.076	2.241	-2.074	0.076	77.738	17.102	15.895
10023	242	38	30	C	W	66.178	-2.145	-2.071	-0.076	2.241	2.074	0.076	77.738	17.102	15.895
10024	242	38	30	C	W	67.589	0.334	0.381	-2.535	-0.332	-0.402	2.671	71.212	18.904	14.845
10025	242	38	30	C	W	68.213	-2.359	1.483	0.148	2.476	-1.479	-0.148	71.186	14.845	18.905
10026	242	38	30	C	W	68.948	-0.599	2.718	-1.639	0.606	-2.811	1.694	77.700	15.901	17.096
10027	242	38	30	C	W	70.158	2.281	3.052	-4.303	-2.182	-3.340	4.708	73.737	16.362	16.929
10028	242	38	30	C	W	73.898	-4.228	-4.365	2.196	4.619	4.182	-2.104	87.554	17.184	15.487
10029	242	38	30	C	W	76.102	2.853	-0.391	-4.797	-2.699	0.432	5.306	84.143	18.455	14.490
10030	242	38	30	C	W	76.537	-3.292	-4.218	1.149	3.523	4.124	-1.124	70.136	18.221	15.508
10031	242	38	30	C	W	77.480	-4.685	2.058	2.722	5.169	-1.952	-2.581	84.027	14.491	18.455
10032	242	38	30	C	W	79.107	1.980	-4.029	-4.038	-1.905	4.383	4.393	82.095	15.504	17.312
10033	242	38	30	C	W	81.052	1.339	3.593	-3.464	-1.304	-3.860	3.722	73.701	16.211	17.080
10034	242	38	30	C	W	85.285	-4.781	-0.868	2.834	5.287	0.821	-2.683	77.776	16.641	16.356
10035	242	38	30	C	W	88.055	2.281	3.830	-4.303	-2.182	-4.190	4.708	77.603	16.362	16.639
10036	242	38	30	C	W	91.945	2.281	-3.830	-4.303	-2.182	4.190	4.708	77.603	16.362	16.639
10037	242	38	30	C	W	94.715	-4.781	0.868	2.834	5.287	-0.822	-2.683	77.776	16.641	16.356
10038	242	38	30	C	W	98.948	1.339	-3.592	-3.464	-1.304	3.860	3.722	73.701	16.211	17.080
10039	242	38	30	C	W	100.893	1.980	-4.648	-4.038	-1.905	5.057	4.393	87.227	15.504	17.167
10040	242	38	30	C	W	102.520	-4.685	-2.058	2.722	5.169	1.952	-2.581	84.027	14.491	18.455
10041	242	38	30	C	W	103.463	-3.292	4.218	1.149	3.523	-4.124	-1.124	70.136	18.221	15.508
10042	242	38	30	C	W	103.898	2.853	0.391	-4.797	-2.699	-0.432	5.306	84.143	18.455	14.490
10043	242	38	30	C	W	106.102	-4.228	4.365	2.196	4.619	-4.182	-2.104	87.554	17.184	15.487
10044	242	38	30	C	W	109.842	2.281	-3.052	-4.303	-2.182	3.340	4.708	73.737	16.362	16.929
10045	242	38	30	C	W	111.052	-0.599	-2.718	-1.639	0.606	2.811	1.694	77.700	15.901	17.096
10046	242	38	30	C	W	111.787	-2.359	-1.483	0.148	2.476	1.479	-0.148	71.186	14.845	18.905
10047	242	38	30	C	W	112.411	0.334	-0.381	-2.535	-0.332	0.401	2.671	71.212	18.904	14.845

BL number	Atoms	γ-PC unit cells	WS_2 unit cells	γ-PC origin atom	WS_2 origin atom	Twist-angle (°)	γ-PC strain 1 (%)	γ-PC strain 2 (%)	γ-PC strain 3 (%)	WS_2 strain 1 (%)	WS_2 strain 2 (%)	WS_2 strain 3 (%)	γ (°)	|**a**|(Å)	|**b**|(Å)
10048	242	38	30	C	W	113.822	-2.145	2.071	-0.076	2.241	-2.074	0.076	77.738	17.102	15.895
10049	242	38	30	P	S	10.158	2.281	3.052	-4.303	-2.182	-3.340	4.708	73.737	16.362	16.929
10050	242	38	30	P	S	16.537	-3.292	-4.218	1.149	3.523	4.124	-1.124	70.136	18.221	15.508
10051	242	38	30	P	S	19.107	1.980	-4.029	-4.038	-1.905	4.383	4.393	82.095	15.504	17.312
10052	242	38	30	P	S	21.052	1.339	3.593	-3.464	-1.304	-3.860	3.722	73.701	16.211	17.080
10053	242	38	30	P	S	38.948	1.339	-3.592	-3.464	-1.304	3.860	3.722	73.701	16.211	17.080
10054	242	38	30	P	S	40.893	1.980	-0.310	-4.038	-1.905	0.337	4.393	70.280	15.504	18.216
10055	242	38	30	P	S	42.520	-4.685	-2.058	2.722	5.169	1.952	-2.581	84.027	14.491	18.455
10056	242	38	30	P	S	43.463	-3.292	4.218	1.149	3.523	-4.124	-1.124	70.136	18.221	15.508
10057	242	38	30	P	S	49.842	2.281	-3.052	-4.303	-2.182	3.340	4.708	73.737	16.362	16.929
10058	242	38	30	P	S	51.787	-2.359	-1.483	0.148	2.476	1.479	-0.148	71.186	14.845	18.905
10059	242	38	30	P	S	52.411	0.334	-0.381	-2.535	-0.332	0.401	2.671	71.212	18.904	14.845
10060	242	38	30	P	S	67.589	0.334	0.381	-2.535	-0.332	-0.402	2.671	71.212	18.904	14.845
10061	242	38	30	P	S	68.213	-2.359	1.483	0.148	2.476	-1.479	-0.148	71.186	14.845	18.905
10062	242	38	30	P	S	70.158	2.281	3.052	-4.303	-2.182	-3.340	4.708	73.737	16.362	16.929
10063	242	38	30	P	S	76.537	-3.292	-4.218	1.149	3.523	4.124	-1.124	70.136	18.221	15.508
10064	242	38	30	P	S	77.480	-4.685	2.058	2.722	5.169	-1.952	-2.581	84.027	14.491	18.455
10065	242	38	30	P	S	79.107	1.980	-4.029	-4.038	-1.905	4.383	4.393	82.095	15.504	17.312
10066	242	38	30	P	S	81.052	1.339	3.593	-3.464	-1.304	-3.860	3.722	73.701	16.211	17.080
10067	242	38	30	P	S	98.948	1.339	-3.592	-3.464	-1.304	3.860	3.722	73.701	16.211	17.080
10068	242	38	30	P	S	100.893	1.980	-0.310	-4.038	-1.905	0.337	4.393	70.280	15.504	18.216
10069	242	38	30	P	S	103.463	-3.292	4.218	1.149	3.523	-4.124	-1.124	70.136	18.221	15.508
10070	242	38	30	P	S	109.842	2.281	-3.052	-4.303	-2.182	3.340	4.708	73.737	16.362	16.929
10071	242	38	30	P	S	111.787	-2.359	-1.483	0.148	2.476	1.479	-0.148	71.186	14.845	18.905
10072	242	38	30	P	W	7.589	0.334	0.381	-2.535	-0.332	-0.402	2.671	71.212	18.904	14.845
10073	242	38	30	P	W	8.213	-2.359	1.483	0.148	2.476	-1.479	-0.148	71.186	14.845	18.905
10074	242	38	30	P	W	10.158	2.281	3.052	-4.303	-2.182	-3.340	4.708	73.737	16.362	16.929
10075	242	38	30	P	W	16.537	-3.292	-4.218	1.149	3.523	4.124	-1.124	70.136	18.221	15.508
10076	242	38	30	P	W	19.107	1.980	-4.029	-4.038	-1.905	4.383	4.393	82.095	15.504	17.312
10077	242	38	30	P	W	21.052	1.339	3.593	-3.464	-1.304	-3.860	3.722	73.701	16.211	17.080
10078	242	38	30	P	W	38.948	1.339	-3.592	-3.464	-1.304	3.860	3.722	73.701	16.211	17.080
10079	242	38	30	P	W	40.893	1.980	-0.310	-4.038	-1.905	0.337	4.393	70.280	15.504	18.216
10080	242	38	30	P	W	43.463	-3.292	4.218	1.149	3.523	-4.124	-1.124	70.136	18.221	15.508
10081	242	38	30	P	W	49.842	2.281	-3.052	-4.303	-2.182	3.340	4.708	73.737	16.362	16.929
10082	242	38	30	P	W	51.052	-0.599	-2.718	-1.639	0.606	2.811	1.694	77.700	15.901	17.096
10083	242	38	30	P	W	51.787	-2.359	-1.483	0.148	2.476	1.479	-0.148	71.186	14.845	18.905
10084	242	38	30	P	W	68.213	-2.359	1.483	0.148	2.476	-1.479	-0.148	71.186	14.845	18.905
10085	242	38	30	P	W	70.158	2.281	3.052	-4.303	-2.182	-3.340	4.708	73.737	16.362	16.929
10086	242	38	30	P	W	76.537	-3.292	-4.218	1.149	3.523	4.124	-1.124	70.136	18.221	15.508
10087	242	38	30	P	W	79.107	1.980	-4.029	-4.038	-1.905	4.383	4.393	82.095	15.504	17.312
10088	242	38	30	P	W	81.052	1.339	3.593	-3.464	-1.304	-3.860	3.722	73.701	16.211	17.080
10089	242	38	30	P	W	98.948	1.339	-3.592	-3.464	-1.304	3.860	3.722	73.701	16.211	17.080
10090	242	38	30	P	W	100.893	1.980	-0.310	-4.038	-1.905	0.337	4.393	70.280	15.504	18.216
10091	242	38	30	P	W	103.463	-3.292	4.218	1.149	3.523	-4.124	-1.124	70.136	18.221	15.508
10092	242	38	30	P	W	109.842	2.281	-3.052	-4.303	-2.182	3.340	4.708	73.737	16.362	16.929
10093	242	38	30	P	W	111.787	-2.359	-1.483	0.148	2.476	1.479	-0.148	71.186	14.845	18.905
10094	242	38	30	P	W	112.411	0.334	-0.381	-2.535	-0.332	0.401	2.671	71.212	18.904	14.845
10095	243	36	33	C	S	8.948	1.048	-1.255	4.341	-1.026	1.154	-3.995	80.110	17.420	15.813
10096	243	36	33	C	S	10.893	4.020	2.224	1.352	-3.721	-2.166	-1.316	80.042	15.815	17.420
10097	243	36	33	C	S	16.337	0.353	4.231	5.091	-0.351	-3.840	-4.621	78.428	17.539	15.797
10098	243	36	33	C	S	27.126	1.029	5.364	4.362	-1.008	-4.934	-4.012	66.846	16.192	18.227

BL number	Atoms	γ-PC unit cells	WS_2 unit cells	γ-PC origin atom	WS_2 origin atom	Twist-angle (°)	γ-PC strain 1 (%)	γ-PC strain 2 (%)	γ-PC strain 3 (%)	WS_2 strain 1 (%)	WS_2 strain 2 (%)	WS_2 strain 3 (%)	γ (°)	|**a**|(Å)	|**b**|(Å)
10099	243	36	33	C	S	32.874	1.029	-5.364	4.362	-1.008	4.934	-4.012	66.846	18.227	16.192
10100	243	36	33	C	S	43.663	0.353	-4.231	5.091	-0.351	3.840	-4.621	78.428	17.539	15.797
10101	243	36	33	C	S	49.107	4.020	-2.224	1.352	-3.721	2.166	-1.316	80.042	15.815	17.420
10102	243	36	33	C	S	51.052	1.048	1.255	4.341	-1.026	-1.154	-3.995	80.110	17.420	15.813
10103	243	36	33	C	S	68.948	1.048	-1.255	4.341	-1.026	1.154	-3.995	80.110	17.420	15.813
10104	243	36	33	C	S	70.893	4.020	2.224	1.352	-3.721	-2.166	-1.316	80.042	15.815	17.420
10105	243	36	33	C	S	76.337	0.353	4.231	5.091	-0.351	-3.840	-4.621	78.428	17.539	15.797
10106	243	36	33	C	S	87.126	1.029	5.364	4.362	-1.008	-4.934	-4.012	66.847	16.192	18.227
10107	243	36	33	C	S	92.874	1.029	-5.364	4.362	-1.008	4.934	-4.012	66.847	18.227	16.192
10108	243	36	33	C	S	103.663	0.353	-4.231	5.091	-0.351	3.840	-4.621	78.427	17.539	15.797
10109	243	36	33	C	S	109.107	4.020	-2.224	1.352	-3.721	2.166	-1.316	80.042	15.815	17.420
10110	243	36	33	C	S	111.052	1.048	1.255	4.341	-1.026	-1.154	-3.995	80.110	17.420	15.813
10111	243	36	33	C	W	8.948	1.048	-1.255	4.341	-1.026	1.154	-3.995	80.110	17.420	15.813
10112	243	36	33	C	W	10.893	4.020	2.224	1.352	-3.721	-2.166	-1.316	80.042	15.815	17.420
10113	243	36	33	C	W	16.337	0.353	4.231	5.091	-0.351	-3.840	-4.621	78.428	17.539	15.797
10114	243	36	33	C	W	27.126	1.029	5.364	4.362	-1.008	-4.934	-4.012	66.846	16.192	18.227
10115	243	36	33	C	W	32.874	1.029	-5.364	4.362	-1.008	4.934	-4.012	66.846	18.227	16.192
10116	243	36	33	C	W	43.663	0.353	-4.231	5.091	-0.351	3.840	-4.621	78.428	17.539	15.797
10117	243	36	33	C	W	49.107	4.020	-2.224	1.352	-3.721	2.166	-1.316	80.042	15.815	17.420
10118	243	36	33	C	W	51.052	1.048	1.255	4.341	-1.026	-1.154	-3.995	80.110	17.420	15.813
10119	243	36	33	C	W	68.948	1.048	-1.255	4.341	-1.026	1.154	-3.995	80.110	17.420	15.813
10120	243	36	33	C	W	70.893	4.020	2.224	1.352	-3.721	-2.166	-1.316	80.042	15.815	17.420
10121	243	36	33	C	W	76.337	0.353	4.231	5.091	-0.351	-3.840	-4.621	78.428	17.539	15.797
10122	243	36	33	C	W	87.126	1.029	5.364	4.362	-1.008	-4.934	-4.012	66.847	16.192	18.227
10123	243	36	33	C	W	92.874	1.029	-5.364	4.362	-1.008	4.934	-4.012	66.847	18.227	16.192
10124	243	36	33	C	W	103.663	0.353	-4.231	5.091	-0.351	3.840	-4.621	78.427	17.539	15.797
10125	243	36	33	C	W	109.107	4.020	-2.224	1.352	-3.721	2.166	-1.316	80.042	15.815	17.420
10126	243	36	33	C	W	111.052	1.048	1.255	4.341	-1.026	-1.154	-3.995	80.110	17.420	15.813
10127	243	36	33	P	S	8.948	1.048	-1.255	4.341	-1.026	1.154	-3.995	80.110	17.420	15.813
10128	243	36	33	P	S	10.893	4.020	2.224	1.352	-3.721	-2.166	-1.316	80.042	15.815	17.420
10129	243	36	33	P	S	16.337	0.353	4.231	5.091	-0.351	-3.840	-4.621	78.428	17.539	15.797
10130	243	36	33	P	S	27.126	1.029	5.364	4.362	-1.008	-4.934	-4.012	66.846	16.192	18.227
10131	243	36	33	P	S	32.874	1.029	-5.364	4.362	-1.008	4.934	-4.012	66.846	18.227	16.192
10132	243	36	33	P	S	43.663	0.353	-4.231	5.091	-0.351	3.840	-4.621	78.428	17.539	15.797
10133	243	36	33	P	S	49.107	4.020	-2.224	1.352	-3.721	2.166	-1.316	80.042	15.815	17.420
10134	243	36	33	P	S	51.052	1.048	1.255	4.341	-1.026	-1.154	-3.995	80.110	17.420	15.813
10135	243	36	33	P	S	68.948	1.048	-1.255	4.341	-1.026	1.154	-3.995	80.110	17.420	15.813
10136	243	36	33	P	S	70.893	4.020	2.224	1.352	-3.721	-2.166	-1.316	80.042	15.815	17.420
10137	243	36	33	P	S	76.337	0.353	4.231	5.091	-0.351	-3.840	-4.621	78.428	17.539	15.797
10138	243	36	33	P	S	87.126	1.029	5.364	4.362	-1.008	-4.934	-4.012	66.847	16.192	18.227
10139	243	36	33	P	S	92.874	1.029	-5.364	4.362	-1.008	4.934	-4.012	66.847	18.227	16.192
10140	243	36	33	P	S	103.663	0.353	-4.231	5.091	-0.351	3.840	-4.621	78.427	17.539	15.797
10141	243	36	33	P	S	109.107	4.020	-2.224	1.352	-3.721	2.166	-1.316	80.042	15.815	17.420
10142	243	36	33	P	S	111.052	1.048	1.255	4.341	-1.026	-1.154	-3.995	80.110	17.420	15.813
10143	243	36	33	P	W	8.948	1.048	-1.255	4.341	-1.026	1.154	-3.995	80.110	17.420	15.813
10144	243	36	33	P	W	10.893	4.020	2.224	1.352	-3.721	-2.166	-1.316	80.042	15.815	17.420
10145	243	36	33	P	W	16.337	0.353	4.231	5.091	-0.351	-3.840	-4.621	78.428	17.539	15.797
10146	243	36	33	P	W	27.126	1.029	5.364	4.362	-1.008	-4.934	-4.012	66.846	16.192	18.227
10147	243	36	33	P	W	32.874	1.029	-5.364	4.362	-1.008	4.934	-4.012	66.846	18.227	16.192
10148	243	36	33	P	W	43.663	0.353	-4.231	5.091	-0.351	3.840	-4.621	78.428	17.539	15.797
10149	243	36	33	P	W	49.107	4.020	-2.224	1.352	-3.721	2.166	-1.316	80.042	15.815	17.420

BL number	Atoms	γ-PC unit cells	WS_2 unit cells	γ-PC origin atom	WS_2 origin atom	Twist-angle (°)	γ-PC strain 1 (%)	γ-PC strain 2 (%)	γ-PC strain 3 (%)	WS_2 strain 1 (%)	WS_2 strain 2 (%)	WS_2 strain 3 (%)	γ (°)	$\lvert\mathbf{a}\rvert$(Å)	$\lvert\mathbf{b}\rvert$(Å)
10150	243	36	33	P	W	51.052	1.048	1.255	4.341	-1.026	-1.154	-3.995	80.110	17.420	15.813
10151	243	36	33	P	W	68.948	1.048	-1.255	4.341	-1.026	1.154	-3.995	80.110	17.420	15.813
10152	243	36	33	P	W	70.893	4.020	2.224	1.352	-3.721	-2.166	-1.316	80.042	15.815	17.420
10153	243	36	33	P	W	76.337	0.353	4.231	5.091	-0.351	-3.840	-4.621	78.428	17.539	15.797
10154	243	36	33	P	W	87.126	1.029	5.364	4.362	-1.008	-4.934	-4.012	66.847	16.192	18.227
10155	243	36	33	P	W	92.874	1.029	-5.364	4.362	-1.008	4.934	-4.012	66.847	18.227	16.192
10156	243	36	33	P	W	103.663	0.353	-4.231	5.091	-0.351	3.840	-4.621	78.427	17.539	15.797
10157	243	36	33	P	W	109.107	4.020	-2.224	1.352	-3.721	2.166	-1.316	80.042	15.815	17.420
10158	243	36	33	P	W	111.052	1.048	1.255	4.341	-1.026	-1.154	-3.995	80.110	17.420	15.813
10159	243	39	29	C	S	16.537	-3.292	-2.494	-1.823	3.523	2.588	1.892	81.097	18.221	14.706
10160	243	39	29	C	S	19.107	-1.485	1.875	-3.617	1.530	-2.021	3.900	81.154	14.708	18.217
10161	243	39	29	C	S	40.893	-1.485	-1.875	-3.617	1.530	2.021	3.900	81.154	14.708	18.217
10162	243	39	29	C	S	43.463	-3.292	2.494	-1.823	3.523	-2.588	1.892	81.097	18.221	14.706
10163	243	39	29	C	S	79.107	-1.485	1.875	-3.617	1.530	-2.021	3.900	81.154	14.708	18.217
10164	243	39	29	C	S	100.893	-1.485	-1.875	-3.617	1.530	2.021	3.900	81.154	14.708	18.217
10165	243	39	29	C	S	103.463	-3.292	2.494	-1.823	3.523	-2.588	1.892	81.097	18.221	14.706
10166	243	39	29	C	W	76.537	-3.292	-2.494	-1.823	3.523	2.588	1.892	81.097	18.221	14.706
10167	243	39	29	P	S	16.537	-3.292	-2.494	-1.823	3.523	2.588	1.892	81.097	18.221	14.706
10168	243	39	29	P	S	19.107	-1.485	1.875	-3.617	1.530	-2.021	3.900	81.154	14.708	18.217
10169	243	39	29	P	S	40.893	-1.485	-1.875	-3.617	1.530	2.021	3.900	81.154	14.708	18.217
10170	243	39	29	P	S	43.463	-3.292	2.494	-1.823	3.523	-2.588	1.892	81.097	18.221	14.706
10171	243	39	29	P	S	79.107	-1.485	1.875	-3.617	1.530	-2.021	3.900	81.154	14.708	18.217
10172	243	39	29	P	S	100.893	-1.485	-1.875	-3.617	1.530	2.021	3.900	81.154	14.708	18.217
10173	243	39	29	P	S	103.463	-3.292	2.494	-1.823	3.523	-2.588	1.892	81.097	18.221	14.706
10174	243	39	29	P	W	76.537	-3.292	-2.494	-1.823	3.523	2.588	1.892	81.097	18.221	14.706
10175	244	37	32	C	S	23.691	2.390	-3.748	-0.043	-2.281	3.752	0.043	68.706	16.344	17.778
10176	244	37	32	C	S	25.050	-0.955	-1.421	3.364	0.973	1.331	-3.152	68.696	17.772	16.356
10177	244	37	32	C	S	28.055	2.281	3.876	0.061	-2.182	-3.871	-0.061	68.599	16.362	17.771
10178	244	37	32	C	S	31.945	2.281	-3.876	0.061	-2.182	3.871	-0.061	68.599	16.362	17.771
10179	244	37	32	C	S	34.950	-0.955	1.421	3.364	0.973	-1.331	-3.152	68.696	17.772	16.356
10180	244	37	32	C	S	36.309	2.390	3.748	-0.043	-2.281	-3.752	0.043	68.706	17.778	16.344
10181	244	37	32	C	S	83.691	2.390	-3.748	-0.043	-2.281	3.752	0.043	68.706	16.344	17.778
10182	244	37	32	C	S	85.050	-0.955	-1.421	3.364	0.973	1.331	-3.152	68.696	17.772	16.356
10183	244	37	32	C	S	88.055	2.281	3.876	0.061	-2.182	-3.871	-0.061	68.599	16.362	17.771
10184	244	37	32	C	S	91.945	2.281	-3.876	0.061	-2.182	3.871	-0.061	68.599	16.362	17.771
10185	244	37	32	C	S	94.950	-0.955	1.421	3.364	0.973	-1.331	-3.152	68.696	17.772	16.356
10186	244	37	32	C	S	96.309	2.390	3.748	-0.043	-2.281	-3.752	0.043	68.706	17.778	16.344
10187	244	37	32	C	W	23.691	2.390	-3.748	-0.043	-2.281	3.752	0.043	68.706	16.344	17.778
10188	244	37	32	C	W	25.050	-0.955	-1.421	3.364	0.973	1.331	-3.152	68.696	17.772	16.356
10189	244	37	32	C	W	28.055	2.281	3.876	0.061	-2.182	-3.871	-0.061	68.599	16.362	17.771
10190	244	37	32	C	W	31.945	2.281	-3.876	0.061	-2.182	3.871	-0.061	68.599	16.362	17.771
10191	244	37	32	C	W	34.950	-0.955	1.421	3.364	0.973	-1.331	-3.152	68.696	17.772	16.356
10192	244	37	32	C	W	36.309	2.390	3.748	-0.043	-2.281	-3.752	0.043	68.706	17.778	16.344
10193	244	37	32	C	W	83.691	2.390	-3.748	-0.043	-2.281	3.752	0.043	68.706	16.344	17.778
10194	244	37	32	C	W	85.050	-0.955	-1.421	3.364	0.973	1.331	-3.152	68.696	17.772	16.356
10195	244	37	32	C	W	88.055	2.281	3.876	0.061	-2.182	-3.871	-0.061	68.599	16.362	17.771
10196	244	37	32	C	W	91.945	2.281	-3.876	0.061	-2.182	3.871	-0.061	68.599	16.362	17.771
10197	244	37	32	C	W	94.950	-0.955	1.421	3.364	0.973	-1.331	-3.152	68.696	17.772	16.356
10198	244	37	32	C	W	96.309	2.390	3.748	-0.043	-2.281	-3.752	0.043	68.706	17.778	16.344
10199	244	37	32	P	S	23.691	2.390	-3.748	-0.043	-2.281	3.752	0.043	68.706	16.344	17.778
10200	244	37	32	P	S	25.050	-0.955	-1.421	3.364	0.973	1.331	-3.152	68.696	17.772	16.356

BL number	Atoms	γ-PC unit cells	WS_2 unit cells	γ-PC origin atom	WS_2 origin atom	Twist-angle (°)	γ-PC strain 1 (%)	γ-PC strain 2 (%)	γ-PC strain 3 (%)	WS_2 strain 1 (%)	WS_2 strain 2 (%)	WS_2 strain 3 (%)	γ (°)	$\lvert\mathbf{a}\rvert$(Å)	$\lvert\mathbf{b}\rvert$(Å)
10201	244	37	32	P	S	28.055	2.281	3.876	0.061	-2.182	-3.871	-0.061	68.599	16.362	17.771
10202	244	37	32	P	S	31.945	2.281	-3.876	0.061	-2.182	3.871	-0.061	68.599	16.362	17.771
10203	244	37	32	P	S	34.950	-0.955	1.421	3.364	0.973	-1.331	-3.152	68.696	17.772	16.356
10204	244	37	32	P	S	36.309	2.390	3.748	-0.043	-2.281	-3.752	0.043	68.706	17.778	16.344
10205	244	37	32	P	S	83.691	2.390	-3.748	-0.043	-2.281	3.752	0.043	68.706	16.344	17.778
10206	244	37	32	P	S	85.050	-0.955	-1.421	3.364	0.973	1.331	-3.152	68.696	17.772	16.356
10207	244	37	32	P	S	88.055	2.281	3.876	0.061	-2.182	-3.871	-0.061	68.599	16.362	17.771
10208	244	37	32	P	S	91.945	2.281	-3.876	0.061	-2.182	3.871	-0.061	68.599	16.362	17.771
10209	244	37	32	P	S	94.950	-0.955	1.421	3.364	0.973	-1.331	-3.152	68.696	17.772	16.356
10210	244	37	32	P	S	96.309	2.390	3.748	-0.043	-2.281	-3.752	0.043	68.706	17.778	16.344
10211	244	37	32	P	W	23.691	2.390	-3.748	-0.043	-2.281	3.752	0.043	68.706	16.344	17.778
10212	244	37	32	P	W	25.050	-0.955	-1.421	3.364	0.973	1.331	-3.152	68.696	17.772	16.356
10213	244	37	32	P	W	28.055	2.281	3.876	0.061	-2.182	-3.871	-0.061	68.599	16.362	17.771
10214	244	37	32	P	W	31.945	2.281	-3.876	0.061	-2.182	3.871	-0.061	68.599	16.362	17.771
10215	244	37	32	P	W	34.950	-0.955	1.421	3.364	0.973	-1.331	-3.152	68.696	17.772	16.356
10216	244	37	32	P	W	36.309	2.390	3.748	-0.043	-2.281	-3.752	0.043	68.706	17.778	16.344
10217	244	37	32	P	W	83.691	2.390	-3.748	-0.043	-2.281	3.752	0.043	68.706	16.344	17.778
10218	244	37	32	P	W	85.050	-0.955	-1.421	3.364	0.973	1.331	-3.152	68.696	17.772	16.356
10219	244	37	32	P	W	88.055	2.281	3.876	0.061	-2.182	-3.871	-0.061	68.599	16.362	17.771
10220	244	37	32	P	W	91.945	2.281	-3.876	0.061	-2.182	3.871	-0.061	68.599	16.362	17.771
10221	244	37	32	P	W	94.950	-0.955	1.421	3.364	0.973	-1.331	-3.152	68.696	17.772	16.356
10222	244	37	32	P	W	96.309	2.390	3.748	-0.043	-2.281	-3.752	0.043	68.706	17.778	16.344
10223	244	40	28	C	S	17.480	-4.685	0.724	-3.254	5.169	-0.774	3.480	87.172	14.491	18.220
10224	244	40	28	C	S	19.842	-4.723	-4.126	-3.214	5.216	4.409	3.435	87.559	15.242	17.317
10225	244	40	28	C	S	24.791	-3.388	4.183	-4.553	3.635	-4.603	5.010	87.490	17.335	15.226
10226	244	40	28	C	S	35.209	-3.388	-4.183	-4.553	3.635	4.603	5.010	87.490	17.335	15.226
10227	244	40	28	C	S	40.158	-4.723	4.126	-3.214	5.216	-4.409	3.435	87.559	15.242	17.317
10228	244	40	28	C	S	43.463	-3.292	0.856	-4.648	3.523	-0.943	5.124	87.161	18.221	14.491
10229	244	40	28	C	S	55.693	-4.685	-4.779	-3.254	5.169	5.111	3.480	89.571	14.491	18.198
10230	244	40	28	C	S	56.996	-3.388	-4.183	-4.553	3.635	4.603	5.010	87.490	17.335	15.226
10231	244	40	28	C	S	58.055	-4.723	-4.126	-3.214	5.216	4.409	3.435	87.559	15.242	17.317
10232	244	40	28	C	S	58.641	-3.292	-4.728	-4.648	3.523	5.212	5.124	89.486	18.221	14.473
10233	244	40	28	C	S	61.359	-3.292	4.728	-4.648	3.523	-5.212	5.124	89.486	18.221	14.473
10234	244	40	28	C	S	61.945	-4.723	4.126	-3.214	5.216	-4.409	3.435	87.559	15.242	17.317
10235	244	40	28	C	S	63.005	-3.388	4.183	-4.553	3.635	-4.603	5.010	87.490	17.335	15.226
10236	244	40	28	C	S	64.307	-4.685	4.779	-3.254	5.169	-5.112	3.480	89.571	14.491	18.198
10237	244	40	28	C	S	77.480	-4.685	0.724	-3.254	5.169	-0.774	3.480	87.172	14.491	18.220
10238	244	40	28	C	S	79.842	-4.723	-4.126	-3.214	5.216	4.409	3.435	87.559	15.242	17.317
10239	244	40	28	C	S	84.791	-3.388	4.183	-4.553	3.635	-4.603	5.010	87.490	17.335	15.226
10240	244	40	28	C	S	95.209	-3.388	-4.183	-4.553	3.635	4.603	5.010	87.490	17.335	15.226
10241	244	40	28	C	S	100.158	-4.723	4.126	-3.214	5.216	-4.409	3.435	87.559	15.242	17.317
10242	244	40	28	C	S	103.463	-3.292	0.856	-4.648	3.523	-0.943	5.124	87.161	18.221	14.491
10243	244	40	28	C	S	115.693	-4.685	-4.779	-3.254	5.169	5.111	3.480	89.571	14.491	18.198
10244	244	40	28	C	S	116.996	-3.388	-4.183	-4.553	3.635	4.603	5.010	87.490	17.335	15.226
10245	244	40	28	C	S	118.055	-4.723	-4.126	-3.214	5.216	4.409	3.435	87.559	15.242	17.317
10246	244	40	28	C	S	118.641	-3.292	-4.728	-4.648	3.523	5.212	5.124	89.486	18.221	14.473
10247	244	40	28	C	W	17.480	-4.685	0.724	-3.254	5.169	-0.774	3.480	87.172	14.491	18.220
10248	244	40	28	C	W	19.842	-4.723	-4.126	-3.214	5.216	4.409	3.435	87.559	15.242	17.317
10249	244	40	28	C	W	24.791	-3.388	4.183	-4.553	3.635	-4.603	5.010	87.490	17.335	15.226
10250	244	40	28	C	W	35.209	-3.388	-4.183	-4.553	3.635	4.603	5.010	87.490	17.335	15.226
10251	244	40	28	C	W	40.158	-4.723	4.126	-3.214	5.216	-4.409	3.435	87.559	15.242	17.317

BL number	Atoms	γ-PC unit cells	WS_2 unit cells	γ-PC origin atom	WS_2 origin atom	Twist-angle (°)	γ-PC strain 1 (%)	γ-PC strain 2 (%)	γ-PC strain 3 (%)	WS_2 strain 1 (%)	WS_2 strain 2 (%)	WS_2 strain 3 (%)	γ (°)	|**a**|(Å)	|**b**|(Å)
10252	244	40	28	C	W	43.463	-3.292	0.856	-4.648	3.523	-0.943	5.124	87.161	18.221	14.491
10253	244	40	28	C	W	55.693	-4.685	-4.779	-3.254	5.169	5.111	3.480	89.571	14.491	18.198
10254	244	40	28	C	W	56.996	-3.388	-4.183	-4.553	3.635	4.603	5.010	87.490	17.335	15.226
10255	244	40	28	C	W	58.055	-4.723	-4.126	-3.214	5.216	4.409	3.435	87.559	15.242	17.317
10256	244	40	28	C	W	58.641	-3.292	-4.728	-4.648	3.523	5.212	5.124	89.486	18.221	14.473
10257	244	40	28	C	W	61.359	-3.292	4.728	-4.648	3.523	-5.212	5.124	89.486	18.221	14.473
10258	244	40	28	C	W	61.945	-4.723	4.126	-3.214	5.216	-4.409	3.435	87.559	15.242	17.317
10259	244	40	28	C	W	63.005	-3.388	4.183	-4.553	3.635	-4.603	5.010	87.490	17.335	15.226
10260	244	40	28	C	W	64.307	-4.685	4.779	-3.254	5.169	-5.112	3.480	89.571	14.491	18.198
10261	244	40	28	C	W	77.480	-4.685	0.724	-3.254	5.169	-0.774	3.480	87.172	14.491	18.220
10262	244	40	28	C	W	79.842	-4.723	-4.126	-3.214	5.216	4.409	3.435	87.559	15.242	17.317
10263	244	40	28	C	W	84.791	-3.388	4.183	-4.553	3.635	-4.603	5.010	87.490	17.335	15.226
10264	244	40	28	C	W	95.209	-3.388	-4.183	-4.553	3.635	4.603	5.010	87.490	17.335	15.226
10265	244	40	28	C	W	100.158	-4.723	4.126	-3.214	5.216	-4.409	3.435	87.559	15.242	17.317
10266	244	40	28	C	W	103.463	-3.292	0.856	-4.648	3.523	-0.943	5.124	87.161	18.221	14.491
10267	244	40	28	C	W	115.693	-4.685	-4.779	-3.254	5.169	5.111	3.480	89.571	14.491	18.198
10268	244	40	28	C	W	116.996	-3.388	-4.183	-4.553	3.635	4.603	5.010	87.490	17.335	15.226
10269	244	40	28	C	W	118.055	-4.723	-4.126	-3.214	5.216	4.409	3.435	87.559	15.242	17.317
10270	244	40	28	C	W	118.641	-3.292	-4.728	-4.648	3.523	5.212	5.124	89.486	18.221	14.473
10271	244	40	28	P	S	17.480	-4.685	0.724	-3.254	5.169	-0.774	3.480	87.172	14.491	18.220
10272	244	40	28	P	S	19.842	-4.723	-4.126	-3.214	5.216	4.409	3.435	87.559	15.242	17.317
10273	244	40	28	P	S	24.791	-3.388	4.183	-4.553	3.635	-4.603	5.010	87.490	17.335	15.226
10274	244	40	28	P	S	35.209	-3.388	-4.183	-4.553	3.635	4.603	5.010	87.490	17.335	15.226
10275	244	40	28	P	S	40.158	-4.723	4.126	-3.214	5.216	-4.409	3.435	87.559	15.242	17.317
10276	244	40	28	P	S	43.463	-3.292	0.856	-4.648	3.523	-0.943	5.124	87.161	18.221	14.491
10277	244	40	28	P	S	55.693	-4.685	-4.779	-3.254	5.169	5.111	3.480	89.571	14.491	18.198
10278	244	40	28	P	S	56.996	-3.388	-4.183	-4.553	3.635	4.603	5.010	87.490	17.335	15.226
10279	244	40	28	P	S	58.055	-4.723	-4.126	-3.214	5.216	4.409	3.435	87.559	15.242	17.317
10280	244	40	28	P	S	58.641	-3.292	-4.728	-4.648	3.523	5.212	5.124	89.486	18.221	14.473
10281	244	40	28	P	S	61.359	-3.292	4.728	-4.648	3.523	-5.212	5.124	89.486	18.221	14.473
10282	244	40	28	P	S	61.945	-4.723	4.126	-3.214	5.216	-4.409	3.435	87.559	15.242	17.317
10283	244	40	28	P	S	63.005	-3.388	4.183	-4.553	3.635	-4.603	5.010	87.490	17.335	15.226
10284	244	40	28	P	S	64.307	-4.685	4.779	-3.254	5.169	-5.112	3.480	89.571	14.491	18.198
10285	244	40	28	P	S	77.480	-4.685	0.724	-3.254	5.169	-0.774	3.480	87.172	14.491	18.220
10286	244	40	28	P	S	79.842	-4.723	-4.126	-3.214	5.216	4.409	3.435	87.559	15.242	17.317
10287	244	40	28	P	S	84.791	-3.388	4.183	-4.553	3.635	-4.603	5.010	87.490	17.335	15.226
10288	244	40	28	P	S	95.209	-3.388	-4.183	-4.553	3.635	4.603	5.010	87.490	17.335	15.226
10289	244	40	28	P	S	100.158	-4.723	4.126	-3.214	5.216	-4.409	3.435	87.559	15.242	17.317
10290	244	40	28	P	S	103.463	-3.292	0.856	-4.648	3.523	-0.943	5.124	87.161	18.221	14.491
10291	244	40	28	P	S	116.996	-3.388	-4.183	-4.553	3.635	4.603	5.010	87.490	17.335	15.226
10292	244	40	28	P	S	118.055	-4.723	-4.126	-3.214	5.216	4.409	3.435	87.559	15.242	17.317
10293	244	40	28	P	S	118.641	-3.292	-4.728	-4.648	3.523	5.212	5.124	89.486	18.221	14.473
10294	244	40	28	P	W	17.480	-4.685	0.724	-3.254	5.169	-0.774	3.480	87.172	14.491	18.220
10295	244	40	28	P	W	19.842	-4.723	-4.126	-3.214	5.216	4.409	3.435	87.559	15.242	17.317
10296	244	40	28	P	W	24.791	-3.388	4.183	-4.553	3.635	-4.603	5.010	87.490	17.335	15.226
10297	244	40	28	P	W	35.209	-3.388	-4.183	-4.553	3.635	4.603	5.010	87.490	17.335	15.226
10298	244	40	28	P	W	40.158	-4.723	4.126	-3.214	5.216	-4.409	3.435	87.559	15.242	17.317
10299	244	40	28	P	W	43.463	-3.292	0.856	-4.648	3.523	-0.943	5.124	87.161	18.221	14.491
10300	244	40	28	P	W	56.996	-3.388	-4.183	-4.553	3.635	4.603	5.010	87.490	17.335	15.226
10301	244	40	28	P	W	58.055	-4.723	-4.126	-3.214	5.216	4.409	3.435	87.559	15.242	17.317
10302	244	40	28	P	W	58.641	-3.292	-4.728	-4.648	3.523	5.212	5.124	89.486	18.221	14.473

BL number	Atoms	γ-PC unit cells	WS_2 unit cells	γ-PC origin atom	WS_2 origin atom	Twist-angle (°)	γ-PC strain 1 (%)	γ-PC strain 2 (%)	γ-PC strain 3 (%)	WS_2 strain 1 (%)	WS_2 strain 2 (%)	WS_2 strain 3 (%)	γ (°)	|**a**|(Å)	|**b**|(Å)
10303	244	40	28	P	W	61.945	-4.723	4.126	-3.214	5.216	-4.409	3.435	87.559	15.242	17.317
10304	244	40	28	P	W	63.005	-3.388	4.183	-4.553	3.635	-4.603	5.010	87.490	17.335	15.226
10305	244	40	28	P	W	77.480	-4.685	0.724	-3.254	5.169	-0.774	3.480	87.172	14.491	18.220
10306	244	40	28	P	W	79.842	-4.723	-4.126	-3.214	5.216	4.409	3.435	87.559	15.242	17.317
10307	244	40	28	P	W	84.791	-3.388	4.183	-4.553	3.635	-4.603	5.010	87.490	17.335	15.226
10308	244	40	28	P	W	95.209	-3.388	-4.183	-4.553	3.635	4.603	5.010	87.490	17.335	15.226
10309	244	40	28	P	W	100.158	-4.723	4.126	-3.214	5.216	-4.409	3.435	87.559	15.242	17.317
10310	244	40	28	P	W	103.463	-3.292	0.856	-4.648	3.523	-0.943	5.124	87.161	18.221	14.491
10311	244	40	28	P	W	115.693	-4.685	-4.779	-3.254	5.169	5.111	3.480	89.571	14.491	18.198
10312	244	40	28	P	W	116.996	-3.388	-4.183	-4.553	3.635	4.603	5.010	87.490	17.335	15.226
10313	244	40	28	P	W	118.055	-4.723	-4.126	-3.214	5.216	4.409	3.435	87.559	15.242	17.317
10314	244	40	28	P	W	118.641	-3.292	-4.728	-4.648	3.523	5.212	5.124	89.486	18.221	14.473
10315	245	38	31	C	S	1.945	-4.723	4.446	4.525	5.216	-4.077	-4.150	75.205	15.242	18.359
10316	245	38	31	C	S	4.307	-4.685	5.259	4.479	5.169	-4.827	-4.111	82.396	14.491	18.835
10317	245	38	31	C	S	27.126	1.029	-3.506	-1.621	-1.008	3.623	1.675	72.901	19.034	14.842
10318	245	38	31	C	S	30.000	-2.359	1.483	1.820	2.476	-1.431	-1.756	72.988	14.845	19.027
10319	245	38	31	C	S	32.874	1.029	3.506	-1.621	-1.008	-3.623	1.675	72.901	19.034	14.842
10320	245	38	31	C	S	55.693	-4.685	-5.259	4.479	5.169	4.827	-4.111	82.396	14.491	18.835
10321	245	38	31	C	S	58.055	-4.723	-4.446	4.525	5.216	4.077	-4.150	75.205	15.242	18.359
10322	245	38	31	C	S	61.945	-4.723	4.446	4.525	5.216	-4.077	-4.150	75.205	15.242	18.360
10323	245	38	31	C	S	64.307	-4.685	5.259	4.479	5.169	-4.827	-4.111	82.396	14.491	18.835
10324	245	38	31	C	S	87.126	1.029	-3.506	-1.621	-1.008	3.623	1.675	72.901	19.034	14.842
10325	245	38	31	C	S	90.000	-2.359	-1.483	1.820	2.476	1.431	-1.756	72.988	14.845	19.027
10326	245	38	31	C	S	92.874	1.029	3.506	-1.621	-1.008	-3.623	1.675	72.901	19.034	14.842
10327	245	38	31	C	S	115.693	-4.685	-5.259	4.479	5.169	4.827	-4.111	82.396	14.491	18.835
10328	245	38	31	C	S	118.055	-4.723	-4.446	4.525	5.216	4.077	-4.150	75.205	15.242	18.359
10329	245	38	31	C	W	1.945	-4.723	4.446	4.525	5.216	-4.077	-4.150	75.205	15.242	18.359
10330	245	38	31	C	W	4.307	-4.685	5.259	4.479	5.169	-4.827	-4.111	82.396	14.491	18.835
10331	245	38	31	C	W	27.126	1.029	-3.506	-1.621	-1.008	3.623	1.675	72.901	19.034	14.842
10332	245	38	31	C	W	30.000	-2.359	1.483	1.820	2.476	-1.431	-1.756	72.988	14.845	19.027
10333	245	38	31	C	W	32.874	1.029	3.506	-1.621	-1.008	-3.623	1.675	72.901	19.034	14.842
10334	245	38	31	C	W	55.693	-4.685	-5.259	4.479	5.169	4.827	-4.111	82.396	14.491	18.835
10335	245	38	31	C	W	58.055	-4.723	-4.446	4.525	5.216	4.077	-4.150	75.205	15.242	18.359
10336	245	38	31	C	W	61.945	-4.723	4.446	4.525	5.216	-4.077	-4.150	75.205	15.242	18.360
10337	245	38	31	C	W	64.307	-4.685	5.259	4.479	5.169	-4.827	-4.111	82.396	14.491	18.835
10338	245	38	31	C	W	87.126	1.029	-3.506	-1.621	-1.008	3.623	1.675	72.901	19.034	14.842
10339	245	38	31	C	W	90.000	-2.359	-1.483	1.820	2.476	1.431	-1.756	72.988	14.845	19.027
10340	245	38	31	C	W	92.874	1.029	3.506	-1.621	-1.008	-3.623	1.675	72.901	19.034	14.842
10341	245	38	31	C	W	115.693	-4.685	-5.259	4.479	5.169	4.827	-4.111	82.396	14.491	18.835
10342	245	38	31	C	W	118.055	-4.723	-4.446	4.525	5.216	4.077	-4.150	75.205	15.242	18.359
10343	245	38	31	P	S	1.945	-4.723	4.446	4.525	5.216	-4.077	-4.150	75.205	15.242	18.359
10344	245	38	31	P	S	4.307	-4.685	5.259	4.479	5.169	-4.827	-4.111	82.396	14.491	18.835
10345	245	38	31	P	S	27.126	1.029	-3.506	-1.621	-1.008	3.623	1.675	72.901	19.034	14.842
10346	245	38	31	P	S	30.000	-2.359	1.483	1.820	2.476	-1.431	-1.756	72.988	14.845	19.027
10347	245	38	31	P	S	32.874	1.029	3.506	-1.621	-1.008	-3.623	1.675	72.901	19.034	14.842
10348	245	38	31	P	S	58.055	-4.723	-4.446	4.525	5.216	4.077	-4.150	75.205	15.242	18.359
10349	245	38	31	P	S	61.945	-4.723	4.446	4.525	5.216	-4.077	-4.150	75.205	15.242	18.360
10350	245	38	31	P	S	87.126	1.029	-3.506	-1.621	-1.008	3.623	1.675	72.901	19.034	14.842
10351	245	38	31	P	S	90.000	-2.359	-1.483	1.820	2.476	1.431	-1.756	72.988	14.845	19.027
10352	245	38	31	P	S	92.874	1.029	3.506	-1.621	-1.008	-3.623	1.675	72.901	19.034	14.842
10353	245	38	31	P	S	115.693	-4.685	-5.259	4.479	5.169	4.827	-4.111	82.396	14.491	18.835

BL number	Atoms	γ-PC unit cells	WS_2 unit cells	γ-PC origin atom	WS_2 origin atom	Twist-angle (°)	γ-PC strain 1 (%)	γ-PC strain 2 (%)	γ-PC strain 3 (%)	WS_2 strain 1 (%)	WS_2 strain 2 (%)	WS_2 strain 3 (%)	γ (°)	|**a**|(Å)	|**b**|(Å)
10354	245	38	31	P	S	118.055	-4.723	-4.446	4.525	5.216	4.077	-4.150	75.205	15.242	18.359
10355	245	38	31	P	W	1.945	-4.723	4.446	4.525	5.216	-4.077	-4.150	75.205	15.242	18.359
10356	245	38	31	P	W	27.126	1.029	-3.506	-1.621	-1.008	3.623	1.675	72.901	19.034	14.842
10357	245	38	31	P	W	30.000	-2.359	1.483	1.820	2.476	-1.431	-1.756	72.988	14.845	19.027
10358	245	38	31	P	W	32.874	1.029	3.506	-1.621	-1.008	-3.623	1.675	72.901	19.034	14.842
10359	245	38	31	P	W	55.693	-4.685	-5.259	4.479	5.169	4.827	-4.111	82.396	14.491	18.835
10360	245	38	31	P	W	58.055	-4.723	-4.446	4.525	5.216	4.077	-4.150	75.205	15.242	18.359
10361	245	38	31	P	W	61.945	-4.723	4.446	4.525	5.216	-4.077	-4.150	75.205	15.242	18.360
10362	245	38	31	P	W	64.307	-4.685	5.259	4.479	5.169	-4.827	-4.111	82.396	14.491	18.835
10363	245	38	31	P	W	87.126	1.029	-3.506	-1.621	-1.008	3.623	1.675	72.901	19.034	14.842
10364	245	38	31	P	W	90.000	-2.359	-1.483	1.820	2.476	1.431	-1.756	72.988	14.845	19.027
10365	245	38	31	P	W	92.874	1.029	3.506	-1.621	-1.008	-3.623	1.675	72.901	19.034	14.842
10366	245	38	31	P	W	118.055	-4.723	-4.446	4.525	5.216	4.077	-4.150	75.205	15.242	18.359
10367	246	36	34	C	S	23.691	2.390	-4.042	4.554	-2.281	3.705	-4.174	67.566	18.224	16.357
10368	246	36	34	C	S	28.055	2.281	3.925	4.667	-2.182	-3.590	-4.269	67.529	16.362	18.223
10369	246	36	34	C	S	31.945	2.281	-3.925	4.667	-2.182	3.590	-4.269	67.529	16.362	18.223
10370	246	36	34	C	S	36.309	2.390	4.042	4.554	-2.281	-3.705	-4.174	67.566	16.357	18.224
10371	246	36	34	C	S	83.691	2.390	-4.042	4.554	-2.281	3.705	-4.174	67.566	18.224	16.357
10372	246	36	34	C	S	88.055	2.281	3.925	4.667	-2.182	-3.590	-4.269	67.529	16.362	18.223
10373	246	36	34	C	S	91.945	2.281	-3.925	4.667	-2.182	3.590	-4.269	67.529	16.362	18.223
10374	246	36	34	C	S	96.309	2.390	4.042	4.554	-2.281	-3.705	-4.174	67.566	16.357	18.224
10375	246	36	34	C	W	23.691	2.390	-4.042	4.554	-2.281	3.705	-4.174	67.566	18.224	16.357
10376	246	36	34	C	W	28.055	2.281	3.925	4.667	-2.182	-3.590	-4.269	67.529	16.362	18.223
10377	246	36	34	C	W	31.945	2.281	-3.925	4.667	-2.182	3.590	-4.269	67.529	16.362	18.223
10378	246	36	34	C	W	36.309	2.390	4.042	4.554	-2.281	-3.705	-4.174	67.566	16.357	18.224
10379	246	36	34	C	W	83.691	2.390	-4.042	4.554	-2.281	3.705	-4.174	67.566	18.224	16.357
10380	246	36	34	C	W	88.055	2.281	3.925	4.667	-2.182	-3.590	-4.269	67.529	16.362	18.223
10381	246	36	34	C	W	91.945	2.281	-3.925	4.667	-2.182	3.590	-4.269	67.529	16.362	18.223
10382	246	36	34	C	W	96.309	2.390	4.042	4.554	-2.281	-3.705	-4.174	67.566	16.357	18.224
10383	246	36	34	P	S	23.691	2.390	-4.042	4.554	-2.281	3.705	-4.174	67.566	18.224	16.357
10384	246	36	34	P	S	28.055	2.281	3.925	4.667	-2.182	-3.590	-4.269	67.529	16.362	18.223
10385	246	36	34	P	S	31.945	2.281	-3.925	4.667	-2.182	3.590	-4.269	67.529	16.362	18.223
10386	246	36	34	P	S	36.309	2.390	4.042	4.554	-2.281	-3.705	-4.174	67.566	16.357	18.224
10387	246	36	34	P	S	83.691	2.390	-4.042	4.554	-2.281	3.705	-4.174	67.566	18.224	16.357
10388	246	36	34	P	S	88.055	2.281	3.925	4.667	-2.182	-3.590	-4.269	67.529	16.362	18.223
10389	246	36	34	P	S	91.945	2.281	-3.925	4.667	-2.182	3.590	-4.269	67.529	16.362	18.223
10390	246	36	34	P	S	96.309	2.390	4.042	4.554	-2.281	-3.705	-4.174	67.566	16.357	18.224
10391	246	36	34	P	W	23.691	2.390	-4.042	4.554	-2.281	3.705	-4.174	67.566	18.224	16.357
10392	246	36	34	P	W	28.055	2.281	3.925	4.667	-2.182	-3.590	-4.269	67.529	16.362	18.223
10393	246	36	34	P	W	31.945	2.281	-3.925	4.667	-2.182	3.590	-4.269	67.529	16.362	18.223
10394	246	36	34	P	W	36.309	2.390	4.042	4.554	-2.281	-3.705	-4.174	67.566	16.357	18.224
10395	246	36	34	P	W	83.691	2.390	-4.042	4.554	-2.281	3.705	-4.174	67.566	18.224	16.357
10396	246	36	34	P	W	88.055	2.281	3.925	4.667	-2.182	-3.590	-4.269	67.529	16.362	18.223
10397	246	36	34	P	W	91.945	2.281	-3.925	4.667	-2.182	3.590	-4.269	67.529	16.362	18.223
10398	246	36	34	P	W	96.309	2.390	4.042	4.554	-2.281	-3.705	-4.174	67.566	16.357	18.224
10399	246	39	30	C	S	4.715	-3.007	-3.590	-0.464	3.200	3.623	0.468	87.935	16.951	15.890
10400	246	39	30	C	S	6.587	-3.853	-0.337	0.444	4.175	0.334	-0.440	82.921	14.354	18.903
10401	246	39	30	C	S	7.589	0.334	1.362	-3.752	-0.332	-1.472	4.057	82.889	18.904	14.354
10402	246	39	30	C	S	8.948	-0.599	3.679	-2.879	0.606	-3.904	3.055	87.831	15.901	16.940
10403	246	39	30	C	S	21.052	1.339	-3.751	-4.658	-1.304	4.136	5.136	87.747	16.211	16.630
10404	246	39	30	C	S	25.285	-4.781	3.524	1.480	5.287	-3.423	-1.437	88.011	16.641	16.199

BL number	Atoms	γ-PC unit cells	WS_2 unit cells	γ-PC origin atom	WS_2 origin atom	Twist-angle (°)	γ-PC strain 1 (%)	γ-PC strain 2 (%)	γ-PC strain 3 (%)	WS_2 strain 1 (%)	WS_2 strain 2 (%)	WS_2 strain 3 (%)	γ (°)	|**a**|(Å)	|**b**|(Å)
10405	246	39	30	C	S	34.715	-4.781	-3.524	1.480	5.287	3.423	-1.437	88.011	16.641	16.199
10406	246	39	30	C	S	38.948	1.339	3.751	-4.658	-1.304	-4.136	5.136	87.747	16.211	16.630
10407	246	39	30	C	S	51.052	-0.599	-3.679	-2.879	0.606	3.904	3.055	87.831	15.901	16.940
10408	246	39	30	C	S	52.411	0.334	-1.362	-3.752	-0.332	1.472	4.057	82.889	18.904	14.354
10409	246	39	30	C	S	53.413	-3.853	0.337	0.444	4.175	-0.334	-0.440	82.921	14.354	18.903
10410	246	39	30	C	S	55.285	-3.007	3.590	-0.464	3.200	-3.623	0.468	87.935	16.951	15.890
10411	246	39	30	C	S	64.715	-3.007	-3.590	-0.464	3.200	3.623	0.468	87.935	16.951	15.890
10412	246	39	30	C	S	66.587	-3.853	-0.337	0.444	4.175	0.334	-0.440	82.921	14.354	18.903
10413	246	39	30	C	S	67.589	0.334	1.362	-3.752	-0.332	-1.472	4.057	82.889	18.904	14.354
10414	246	39	30	C	S	68.948	-0.599	3.679	-2.879	0.606	-3.904	3.055	87.831	15.901	16.940
10415	246	39	30	C	S	81.052	1.339	-3.751	-4.658	-1.304	4.136	5.136	87.747	16.211	16.630
10416	246	39	30	C	S	85.285	-4.781	3.524	1.480	5.287	-3.423	-1.437	88.011	16.641	16.199
10417	246	39	30	C	S	94.715	-4.781	-3.524	1.480	5.287	3.423	-1.437	88.011	16.641	16.199
10418	246	39	30	C	S	98.948	1.339	3.751	-4.658	-1.304	-4.136	5.136	87.747	16.211	16.630
10419	246	39	30	C	S	111.052	-0.599	-3.679	-2.879	0.606	3.904	3.055	87.831	15.901	16.940
10420	246	39	30	C	S	112.411	0.334	-1.362	-3.752	-0.332	1.472	4.057	82.889	18.904	14.354
10421	246	39	30	C	S	113.413	-3.853	0.337	0.444	4.175	-0.334	-0.440	82.921	14.354	18.903
10422	246	39	30	C	S	115.285	-3.007	3.590	-0.464	3.200	-3.623	0.468	87.935	16.951	15.890
10423	246	39	30	C	W	4.715	-3.007	-3.590	-0.464	3.200	3.623	0.468	87.935	16.951	15.890
10424	246	39	30	C	W	6.587	-3.853	-0.337	0.444	4.175	0.334	-0.440	82.921	14.354	18.903
10425	246	39	30	C	W	7.589	0.334	1.362	-3.752	-0.332	-1.472	4.057	82.889	18.904	14.354
10426	246	39	30	C	W	8.948	-0.599	3.679	-2.879	0.606	-3.904	3.055	87.831	15.901	16.940
10427	246	39	30	C	W	21.052	1.339	-3.751	-4.658	-1.304	4.136	5.136	87.747	16.211	16.630
10428	246	39	30	C	W	25.285	-4.781	3.524	1.480	5.287	-3.423	-1.437	88.011	16.641	16.199
10429	246	39	30	C	W	34.715	-4.781	-3.524	1.480	5.287	3.423	-1.437	88.011	16.641	16.199
10430	246	39	30	C	W	38.948	1.339	3.751	-4.658	-1.304	-4.136	5.136	87.747	16.211	16.630
10431	246	39	30	C	W	51.052	-0.599	-3.679	-2.879	0.606	3.904	3.055	87.831	15.901	16.940
10432	246	39	30	C	W	52.411	0.334	-1.362	-3.752	-0.332	1.472	4.057	82.889	18.904	14.354
10433	246	39	30	C	W	53.413	-3.853	0.337	0.444	4.175	-0.334	-0.440	82.921	14.354	18.903
10434	246	39	30	C	W	55.285	-3.007	3.590	-0.464	3.200	-3.623	0.468	87.935	16.951	15.890
10435	246	39	30	C	W	64.715	-3.007	-3.590	-0.464	3.200	3.623	0.468	87.935	16.951	15.890
10436	246	39	30	C	W	66.587	-3.853	-0.337	0.444	4.175	0.334	-0.440	82.921	14.354	18.903
10437	246	39	30	C	W	67.589	0.334	1.362	-3.752	-0.332	-1.472	4.057	82.889	18.904	14.354
10438	246	39	30	C	W	68.948	-0.599	3.679	-2.879	0.606	-3.904	3.055	87.831	15.901	16.940
10439	246	39	30	C	W	81.052	1.339	-3.751	-4.658	-1.304	4.136	5.136	87.747	16.211	16.630
10440	246	39	30	C	W	85.285	-4.781	3.524	1.480	5.287	-3.423	-1.437	88.011	16.641	16.199
10441	246	39	30	C	W	94.715	-4.781	-3.524	1.480	5.287	3.423	-1.437	88.011	16.641	16.199
10442	246	39	30	C	W	98.948	1.339	3.751	-4.658	-1.304	-4.136	5.136	87.747	16.211	16.630
10443	246	39	30	C	W	111.052	-0.599	-3.679	-2.879	0.606	3.904	3.055	87.831	15.901	16.940
10444	246	39	30	C	W	112.411	0.334	-1.362	-3.752	-0.332	1.472	4.057	82.889	18.904	14.354
10445	246	39	30	C	W	113.413	-3.853	0.337	0.444	4.175	-0.334	-0.440	82.921	14.354	18.903
10446	246	39	30	C	W	115.285	-3.007	3.590	-0.464	3.200	-3.623	0.468	87.935	16.951	15.890
10447	246	39	30	P	S	4.715	-3.007	-3.590	-0.464	3.200	3.623	0.468	87.935	16.951	15.890
10448	246	39	30	P	S	6.587	-3.853	-0.337	0.444	4.175	0.334	-0.440	82.921	14.354	18.903
10449	246	39	30	P	S	7.589	0.334	1.362	-3.752	-0.332	-1.472	4.057	82.889	18.904	14.354
10450	246	39	30	P	S	8.948	-0.599	3.679	-2.879	0.606	-3.904	3.055	87.831	15.901	16.940
10451	246	39	30	P	S	21.052	1.339	-3.751	-4.658	-1.304	4.136	5.136	87.747	16.211	16.630
10452	246	39	30	P	S	25.285	-4.781	3.524	1.480	5.287	-3.423	-1.437	88.011	16.641	16.199
10453	246	39	30	P	S	34.715	-4.781	-3.524	1.480	5.287	3.423	-1.437	88.011	16.641	16.199
10454	246	39	30	P	S	38.948	1.339	3.751	-4.658	-1.304	-4.136	5.136	87.747	16.211	16.630
10455	246	39	30	P	S	51.052	-0.599	-3.679	-2.879	0.606	3.904	3.055	87.831	15.901	16.940

BL number	Atoms	γ-PC unit cells	WS_2 unit cells	γ-PC origin atom	WS_2 origin atom	Twist-angle (°)	γ-PC strain 1 (%)	γ-PC strain 2 (%)	γ-PC strain 3 (%)	WS_2 strain 1 (%)	WS_2 strain 2 (%)	WS_2 strain 3 (%)	γ (°)	$\lvert\mathbf{a}\rvert$(Å)	$\lvert\mathbf{b}\rvert$(Å)
10456	246	39	30	P	S	52.411	0.334	-1.362	-3.752	-0.332	1.472	4.057	82.889	18.904	14.354
10457	246	39	30	P	S	53.413	-3.853	0.337	0.444	4.175	-0.334	-0.440	82.921	14.354	18.903
10458	246	39	30	P	S	55.285	-3.007	3.590	-0.464	3.200	-3.623	0.468	87.935	16.951	15.890
10459	246	39	30	P	S	64.715	-3.007	-3.590	-0.464	3.200	3.623	0.468	87.935	16.951	15.890
10460	246	39	30	P	S	66.587	-3.853	-0.337	0.444	4.175	0.334	-0.440	82.921	14.354	18.903
10461	246	39	30	P	S	67.589	0.334	1.362	-3.752	-0.332	-1.472	4.057	82.889	18.904	14.354
10462	246	39	30	P	S	68.948	-0.599	3.679	-2.879	0.606	-3.904	3.055	87.831	15.901	16.940
10463	246	39	30	P	S	81.052	1.339	-3.751	-4.658	-1.304	4.136	5.136	87.747	16.211	16.630
10464	246	39	30	P	S	85.285	-4.781	3.524	1.480	5.287	-3.423	-1.437	88.011	16.641	16.199
10465	246	39	30	P	S	94.715	-4.781	-3.524	1.480	5.287	3.423	-1.437	88.011	16.641	16.199
10466	246	39	30	P	S	98.948	1.339	3.751	-4.658	-1.304	-4.136	5.136	87.747	16.211	16.630
10467	246	39	30	P	S	111.052	-0.599	-3.679	-2.879	0.606	3.904	3.055	87.831	15.901	16.940
10468	246	39	30	P	S	112.411	0.334	-1.362	-3.752	-0.332	1.472	4.057	82.889	18.904	14.354
10469	246	39	30	P	S	113.413	-3.853	0.337	0.444	4.175	-0.334	-0.440	82.921	14.354	18.903
10470	246	39	30	P	S	115.285	-3.007	3.590	-0.464	3.200	-3.623	0.468	87.935	16.951	15.890
10471	246	39	30	P	W	4.715	-3.007	-3.590	-0.464	3.200	3.623	0.468	87.935	16.951	15.890
10472	246	39	30	P	W	6.587	-3.853	-0.337	0.444	4.175	0.334	-0.440	82.921	14.354	18.903
10473	246	39	30	P	W	7.589	0.334	1.362	-3.752	-0.332	-1.472	4.057	82.889	18.904	14.354
10474	246	39	30	P	W	8.948	-0.599	3.679	-2.879	0.606	-3.904	3.055	87.831	15.901	16.940
10475	246	39	30	P	W	21.052	1.339	-3.751	-4.658	-1.304	4.136	5.136	87.747	16.211	16.630
10476	246	39	30	P	W	25.285	-4.781	3.524	1.480	5.287	-3.423	-1.437	88.011	16.641	16.199
10477	246	39	30	P	W	34.715	-4.781	-3.524	1.480	5.287	3.423	-1.437	88.011	16.641	16.199
10478	246	39	30	P	W	38.948	1.339	3.751	-4.658	-1.304	-4.136	5.136	87.747	16.211	16.630
10479	246	39	30	P	W	51.052	-0.599	-3.679	-2.879	0.606	3.904	3.055	87.831	15.901	16.940
10480	246	39	30	P	W	52.411	0.334	-1.362	-3.752	-0.332	1.472	4.057	82.889	18.904	14.354
10481	246	39	30	P	W	53.413	-3.853	0.337	0.444	4.175	-0.334	-0.440	82.921	14.354	18.903
10482	246	39	30	P	W	55.285	-3.007	3.590	-0.464	3.200	-3.623	0.468	87.935	16.951	15.890
10483	246	39	30	P	W	64.715	-3.007	-3.590	-0.464	3.200	3.623	0.468	87.935	16.951	15.890
10484	246	39	30	P	W	66.587	-3.853	-0.337	0.444	4.175	0.334	-0.440	82.921	14.354	18.903
10485	246	39	30	P	W	67.589	0.334	1.362	-3.752	-0.332	-1.472	4.057	82.889	18.904	14.354
10486	246	39	30	P	W	68.948	-0.599	3.679	-2.879	0.606	-3.904	3.055	87.831	15.901	16.940
10487	246	39	30	P	W	81.052	1.339	-3.751	-4.658	-1.304	4.136	5.136	87.747	16.211	16.630
10488	246	39	30	P	W	85.285	-4.781	3.524	1.480	5.287	-3.423	-1.437	88.011	16.641	16.199
10489	246	39	30	P	W	94.715	-4.781	-3.524	1.480	5.287	3.423	-1.437	88.011	16.641	16.199
10490	246	39	30	P	W	98.948	1.339	3.751	-4.658	-1.304	-4.136	5.136	87.747	16.211	16.630
10491	246	39	30	P	W	111.052	-0.599	-3.679	-2.879	0.606	3.904	3.055	87.831	15.901	16.940
10492	246	39	30	P	W	112.411	0.334	-1.362	-3.752	-0.332	1.472	4.057	82.889	18.904	14.354
10493	246	39	30	P	W	113.413	-3.853	0.337	0.444	4.175	-0.334	-0.440	82.921	14.354	18.903
10494	246	39	30	P	W	115.285	-3.007	3.590	-0.464	3.200	-3.623	0.468	87.935	16.951	15.890
10495	247	37	33	C	S	17.696	1.029	-2.855	2.893	-1.008	2.699	-2.734	65.931	18.583	16.206
10496	247	37	33	C	S	21.052	1.339	3.163	2.573	-1.304	-3.008	-2.447	65.912	16.211	18.579
10497	247	37	33	C	S	38.948	1.339	-3.163	2.573	-1.304	3.008	-2.447	65.912	16.211	18.579
10498	247	37	33	C	S	41.387	3.582	1.179	0.372	-3.342	-1.171	-0.370	65.938	18.586	16.205
10499	247	37	33	C	S	77.696	1.029	-2.855	2.893	-1.008	2.699	-2.734	65.931	18.583	16.206
10500	247	37	33	C	S	78.613	3.582	-1.179	0.372	-3.342	1.171	-0.370	65.938	18.586	16.205
10501	247	37	33	C	S	81.052	1.339	3.163	2.573	-1.304	-3.008	-2.447	65.912	16.211	18.579
10502	247	37	33	C	S	98.948	1.339	-3.163	2.573	-1.304	3.008	-2.447	65.912	16.211	18.579
10503	247	37	33	C	S	101.387	3.582	1.179	0.372	-3.342	-1.171	-0.370	65.938	18.586	16.205
10504	247	37	33	C	S	102.304	1.029	2.855	2.892	-1.008	-2.698	-2.734	65.931	16.206	18.582
10505	247	37	33	C	W	17.696	1.029	-2.855	2.893	-1.008	2.699	-2.734	65.931	18.583	16.206
10506	247	37	33	C	W	21.052	1.339	3.163	2.573	-1.304	-3.008	-2.447	65.912	16.211	18.579

BL number	Atoms	γ-PC unit cells	WS_2 unit cells	γ-PC origin atom	WS_2 origin atom	Twist-angle (°)	γ-PC strain 1 (%)	γ-PC strain 2 (%)	γ-PC strain 3 (%)	WS_2 strain 1 (%)	WS_2 strain 2 (%)	WS_2 strain 3 (%)	γ (°)	|**a**|(Å)	|**b**|(Å)
10507	247	37	33	C	W	38.948	1.339	-3.163	2.573	-1.304	3.008	-2.447	65.912	16.211	18.579
10508	247	37	33	C	W	41.387	3.582	1.179	0.372	-3.342	-1.171	-0.370	65.938	18.586	16.205
10509	247	37	33	C	W	42.304	1.029	2.855	2.892	-1.008	-2.698	-2.734	65.931	16.206	18.583
10510	247	37	33	C	W	77.696	1.029	-2.855	2.893	-1.008	2.699	-2.734	65.931	18.583	16.206
10511	247	37	33	C	W	81.052	1.339	3.163	2.573	-1.304	-3.008	-2.447	65.912	16.211	18.579
10512	247	37	33	C	W	98.948	1.339	-3.163	2.573	-1.304	3.008	-2.447	65.912	16.211	18.579
10513	247	37	33	C	W	101.387	3.582	1.179	0.372	-3.342	-1.171	-0.370	65.938	18.586	16.205
10514	247	37	33	C	W	102.304	1.029	2.855	2.892	-1.008	-2.698	-2.734	65.931	16.206	18.582
10515	247	37	33	P	S	17.696	1.029	-2.855	2.893	-1.008	2.699	-2.734	65.931	18.583	16.206
10516	247	37	33	P	S	21.052	1.339	3.163	2.573	-1.304	-3.008	-2.447	65.912	16.211	18.579
10517	247	37	33	P	S	38.948	1.339	-3.163	2.573	-1.304	3.008	-2.447	65.912	16.211	18.579
10518	247	37	33	P	S	41.387	3.582	1.179	0.372	-3.342	-1.171	-0.370	65.938	18.586	16.205
10519	247	37	33	P	S	42.304	1.029	2.855	2.892	-1.008	-2.698	-2.734	65.931	16.206	18.583
10520	247	37	33	P	S	77.696	1.029	-2.855	2.893	-1.008	2.699	-2.734	65.931	18.583	16.206
10521	247	37	33	P	S	81.052	1.339	3.163	2.573	-1.304	-3.008	-2.447	65.912	16.211	18.579
10522	247	37	33	P	S	98.948	1.339	-3.163	2.573	-1.304	3.008	-2.447	65.912	16.211	18.579
10523	247	37	33	P	S	101.387	3.582	1.179	0.372	-3.342	-1.171	-0.370	65.938	18.586	16.205
10524	247	37	33	P	S	102.304	1.029	2.855	2.892	-1.008	-2.698	-2.734	65.931	16.206	18.582
10525	247	37	33	P	W	17.696	1.029	-2.855	2.893	-1.008	2.699	-2.734	65.931	18.583	16.206
10526	247	37	33	P	W	21.052	1.339	3.163	2.573	-1.304	-3.008	-2.447	65.912	16.211	18.579
10527	247	37	33	P	W	38.948	1.339	-3.163	2.573	-1.304	3.008	-2.447	65.912	16.211	18.579
10528	247	37	33	P	W	41.387	3.582	1.179	0.372	-3.342	-1.171	-0.370	65.938	18.586	16.205
10529	247	37	33	P	W	77.696	1.029	-2.855	2.893	-1.008	2.699	-2.734	65.931	18.583	16.206
10530	247	37	33	P	W	78.613	3.582	-1.179	0.372	-3.342	1.171	-0.370	65.938	18.586	16.205
10531	247	37	33	P	W	81.052	1.339	3.163	2.573	-1.304	-3.008	-2.447	65.912	16.211	18.579
10532	247	37	33	P	W	98.948	1.339	-3.163	2.573	-1.304	3.008	-2.447	65.912	16.211	18.579
10533	247	37	33	P	W	101.387	3.582	1.179	0.372	-3.342	-1.171	-0.370	65.938	18.586	16.205
10534	247	37	33	P	W	102.304	1.029	2.855	2.892	-1.008	-2.698	-2.734	65.931	16.206	18.582
10535	247	40	29	C	S	1.945	-4.723	-5.010	-1.543	5.216	5.169	1.592	82.329	15.242	17.759
10536	247	40	29	C	S	4.307	-4.685	0.724	-1.584	5.169	-0.748	1.636	71.076	14.491	19.569
10537	247	40	29	C	S	4.715	-1.702	1.380	-4.574	1.762	-1.519	5.034	71.100	19.567	14.490
10538	247	40	29	C	S	17.480	-4.685	4.779	-1.584	5.169	-4.935	1.636	68.991	14.491	19.830
10539	247	40	29	C	S	42.520	-4.685	-4.779	-1.584	5.169	4.935	1.636	68.991	14.491	19.830
10540	247	40	29	C	S	55.285	-1.702	-1.380	-4.574	1.762	1.519	5.034	71.100	19.567	14.490
10541	247	40	29	C	S	55.693	-4.685	-0.724	-1.584	5.169	0.748	1.636	71.076	14.491	19.569
10542	247	40	29	C	S	58.055	-4.723	5.010	-1.543	5.216	-5.169	1.592	82.329	15.242	17.759
10543	247	40	29	C	S	61.945	-4.723	-5.010	-1.543	5.216	5.169	1.592	82.329	15.242	17.759
10544	247	40	29	C	S	64.307	-4.685	0.724	-1.584	5.169	-0.748	1.636	71.076	14.491	19.569
10545	247	40	29	C	S	64.715	-1.702	1.380	-4.574	1.762	-1.519	5.034	71.100	19.567	14.490
10546	247	40	29	C	S	77.480	-4.685	4.779	-1.584	5.169	-4.935	1.636	68.990	14.491	19.830
10547	247	40	29	C	S	102.520	-4.685	-4.779	-1.584	5.169	4.935	1.636	68.990	14.491	19.830
10548	247	40	29	C	S	115.285	-1.702	-1.380	-4.574	1.762	1.519	5.034	71.100	19.567	14.490
10549	247	40	29	C	S	115.693	-4.685	-0.724	-1.584	5.169	0.748	1.636	71.076	14.491	19.569
10550	247	40	29	C	S	118.055	-4.723	5.010	-1.543	5.216	-5.169	1.592	82.329	15.242	17.759
10551	247	40	29	C	W	1.945	-4.723	-5.010	-1.543	5.216	5.169	1.592	82.329	15.242	17.759
10552	247	40	29	C	W	4.307	-4.685	0.724	-1.584	5.169	-0.748	1.636	71.076	14.491	19.569
10553	247	40	29	C	W	4.715	-1.702	1.380	-4.574	1.762	-1.519	5.034	71.100	19.567	14.490
10554	247	40	29	C	W	17.480	-4.685	4.779	-1.584	5.169	-4.935	1.636	68.991	14.491	19.830
10555	247	40	29	C	W	42.520	-4.685	-4.779	-1.584	5.169	4.935	1.636	68.991	14.491	19.830
10556	247	40	29	C	W	55.285	-1.702	-1.380	-4.574	1.762	1.519	5.034	71.100	19.567	14.490
10557	247	40	29	C	W	55.693	-4.685	-0.724	-1.584	5.169	0.748	1.636	71.076	14.491	19.569

BL number	Atoms	γ-PC unit cells	WS_2 unit cells	γ-PC origin atom	WS_2 origin atom	Twist-angle (°)	γ-PC strain 1 (%)	γ-PC strain 2 (%)	γ-PC strain 3 (%)	WS_2 strain 1 (%)	WS_2 strain 2 (%)	WS_2 strain 3 (%)	γ (°)	|**a**|(Å)	|**b**|(Å)
10558	247	40	29	C	W	58.055	-4.723	5.010	-1.543	5.216	-5.169	1.592	82.329	15.242	17.759
10559	247	40	29	C	W	61.945	-4.723	-5.010	-1.543	5.216	5.169	1.592	82.329	15.242	17.759
10560	247	40	29	C	W	64.307	-4.685	0.724	-1.584	5.169	-0.748	1.636	71.076	14.491	19.569
10561	247	40	29	C	W	64.715	-1.702	1.380	-4.574	1.762	-1.519	5.034	71.100	19.567	14.490
10562	247	40	29	C	W	77.480	-4.685	4.779	-1.584	5.169	-4.935	1.636	68.990	14.491	19.830
10563	247	40	29	C	W	102.520	-4.685	-4.779	-1.584	5.169	4.935	1.636	68.990	14.491	19.830
10564	247	40	29	C	W	115.285	-1.702	-1.380	-4.574	1.762	1.519	5.034	71.100	19.567	14.490
10565	247	40	29	C	W	115.693	-4.685	-0.724	-1.584	5.169	0.748	1.636	71.076	14.491	19.569
10566	247	40	29	C	W	118.055	-4.723	5.010	-1.543	5.216	-5.169	1.592	82.329	15.242	17.759
10567	247	40	29	P	S	1.945	-4.723	-5.010	-1.543	5.216	5.169	1.592	82.329	15.242	17.759
10568	247	40	29	P	S	4.307	-4.685	0.724	-1.584	5.169	-0.748	1.636	71.076	14.491	19.569
10569	247	40	29	P	S	4.715	-1.702	1.380	-4.574	1.762	-1.519	5.034	71.100	19.567	14.490
10570	247	40	29	P	S	42.520	-4.685	-4.779	-1.584	5.169	4.935	1.636	68.991	14.491	19.830
10571	247	40	29	P	S	55.285	-1.702	-1.380	-4.574	1.762	1.519	5.034	71.100	19.567	14.490
10572	247	40	29	P	S	55.693	-4.685	-0.724	-1.584	5.169	0.748	1.636	71.076	14.491	19.569
10573	247	40	29	P	S	58.055	-4.723	5.010	-1.543	5.216	-5.169	1.592	82.329	15.242	17.759
10574	247	40	29	P	S	61.945	-4.723	-5.010	-1.543	5.216	5.169	1.592	82.329	15.242	17.759
10575	247	40	29	P	S	64.307	-4.685	0.724	-1.584	5.169	-0.748	1.636	71.076	14.491	19.569
10576	247	40	29	P	S	64.715	-1.702	1.380	-4.574	1.762	-1.519	5.034	71.100	19.567	14.490
10577	247	40	29	P	S	77.480	-4.685	4.779	-1.584	5.169	-4.935	1.636	68.990	14.491	19.830
10578	247	40	29	P	S	115.285	-1.702	-1.380	-4.574	1.762	1.519	5.034	71.100	19.567	14.490
10579	247	40	29	P	S	115.693	-4.685	-0.724	-1.584	5.169	0.748	1.636	71.076	14.491	19.569
10580	247	40	29	P	S	118.055	-4.723	5.010	-1.543	5.216	-5.169	1.592	82.329	15.242	17.759
10581	247	40	29	P	W	1.945	-4.723	-5.010	-1.543	5.216	5.169	1.592	82.329	15.242	17.759
10582	247	40	29	P	W	4.307	-4.685	0.724	-1.584	5.169	-0.748	1.636	71.076	14.491	19.569
10583	247	40	29	P	W	4.715	-1.702	1.380	-4.574	1.762	-1.519	5.034	71.100	19.567	14.490
10584	247	40	29	P	W	17.480	-4.685	4.779	-1.584	5.169	-4.935	1.636	68.991	14.491	19.830
10585	247	40	29	P	W	55.285	-1.702	-1.380	-4.574	1.762	1.519	5.034	71.100	19.567	14.490
10586	247	40	29	P	W	55.693	-4.685	-0.724	-1.584	5.169	0.748	1.636	71.076	14.491	19.569
10587	247	40	29	P	W	58.055	-4.723	5.010	-1.543	5.216	-5.169	1.592	82.329	15.242	17.759
10588	247	40	29	P	W	61.945	-4.723	-5.010	-1.543	5.216	5.169	1.592	82.329	15.242	17.759
10589	247	40	29	P	W	64.307	-4.685	0.724	-1.584	5.169	-0.748	1.636	71.076	14.491	19.569
10590	247	40	29	P	W	64.715	-1.702	1.380	-4.574	1.762	-1.519	5.034	71.100	19.567	14.490
10591	247	40	29	P	W	102.520	-4.685	-4.779	-1.584	5.169	4.935	1.636	68.990	14.491	19.830
10592	247	40	29	P	W	115.285	-1.702	-1.380	-4.574	1.762	1.519	5.034	71.100	19.567	14.490
10593	247	40	29	P	W	115.693	-4.685	-0.724	-1.584	5.169	0.748	1.636	71.076	14.491	19.569
10594	247	40	29	P	W	118.055	-4.723	5.010	-1.543	5.216	-5.169	1.592	82.329	15.242	17.759
10595	248	38	32	C	S	0.000	5.011	-3.191	-3.675	-4.554	3.444	3.966	73.398	16.799	17.070
10596	248	38	32	C	S	8.213	-2.359	-1.483	3.492	2.476	1.387	-3.264	84.270	14.845	18.586
10597	248	38	32	C	S	9.183	3.582	0.255	-2.439	-3.342	-0.268	2.565	84.323	18.586	14.844
10598	248	38	32	C	S	28.055	2.281	-4.773	-1.256	-2.182	4.896	1.289	73.293	16.362	17.508
10599	248	38	32	C	S	31.945	2.281	4.773	-1.256	-2.182	-4.896	1.289	73.293	16.362	17.508
10600	248	38	32	C	S	50.817	3.582	-0.255	-2.440	-3.342	0.268	2.565	84.323	18.586	14.844
10601	248	38	32	C	S	51.787	-2.359	1.484	3.492	2.476	-1.387	-3.264	84.270	14.845	18.586
10602	248	38	32	C	S	60.000	5.011	3.191	-3.675	-4.554	-3.444	3.966	73.398	16.799	17.070
10603	248	38	32	C	S	68.213	-2.359	-1.483	3.492	2.476	1.387	-3.264	84.270	14.845	18.586
10604	248	38	32	C	S	69.183	3.582	0.255	-2.439	-3.342	-0.268	2.565	84.323	18.586	14.844
10605	248	38	32	C	S	88.055	2.281	-4.773	-1.256	-2.182	4.896	1.289	73.293	16.362	17.508
10606	248	38	32	C	S	91.945	2.281	4.773	-1.256	-2.182	-4.896	1.289	73.293	16.362	17.508
10607	248	38	32	C	S	110.817	3.582	-0.255	-2.440	-3.342	0.268	2.565	84.323	18.586	14.844
10608	248	38	32	C	S	111.787	-2.359	1.484	3.492	2.476	-1.387	-3.264	84.270	14.845	18.586

BL number	Atoms	γ-PC unit cells	WS_2 unit cells	γ-PC origin atom	WS_2 origin atom	Twist-angle (°)	γ-PC strain 1 (%)	γ-PC strain 2 (%)	γ-PC strain 3 (%)	WS_2 strain 1 (%)	WS_2 strain 2 (%)	WS_2 strain 3 (%)	γ (°)	|**a**|(Å)	|**b**|(Å)
10609	248	38	32	C	W	0.000	5.011	-3.191	-3.675	-4.554	3.444	3.966	73.398	16.799	17.070
10610	248	38	32	C	W	8.213	-2.359	-1.483	3.492	2.476	1.387	-3.264	84.270	14.845	18.586
10611	248	38	32	C	W	9.183	3.582	0.255	-2.439	-3.342	-0.268	2.565	84.323	18.586	14.844
10612	248	38	32	C	W	28.055	2.281	-4.773	-1.256	-2.182	4.896	1.289	73.293	16.362	17.508
10613	248	38	32	C	W	31.945	2.281	4.773	-1.256	-2.182	-4.896	1.289	73.293	16.362	17.508
10614	248	38	32	C	W	50.817	3.582	-0.255	-2.440	-3.342	0.268	2.565	84.323	18.586	14.844
10615	248	38	32	C	W	51.787	-2.359	1.484	3.492	2.476	-1.387	-3.264	84.270	14.845	18.586
10616	248	38	32	C	W	60.000	5.011	3.191	-3.675	-4.554	-3.444	3.966	73.398	16.799	17.070
10617	248	38	32	C	W	68.213	-2.359	-1.483	3.492	2.476	1.387	-3.264	84.270	14.845	18.586
10618	248	38	32	C	W	69.183	3.582	0.255	-2.439	-3.342	-0.268	2.565	84.323	18.586	14.844
10619	248	38	32	C	W	88.055	2.281	-4.773	-1.256	-2.182	4.896	1.289	73.293	16.362	17.508
10620	248	38	32	C	W	91.945	2.281	4.773	-1.256	-2.182	-4.896	1.289	73.293	16.362	17.508
10621	248	38	32	C	W	110.817	3.582	-0.255	-2.440	-3.342	0.268	2.565	84.323	18.586	14.844
10622	248	38	32	C	W	111.787	-2.359	1.484	3.492	2.476	-1.387	-3.264	84.270	14.845	18.586
10623	248	38	32	P	S	0.000	5.011	-3.191	-3.675	-4.554	3.444	3.966	73.398	16.799	17.070
10624	248	38	32	P	S	8.213	-2.359	-1.483	3.492	2.476	1.387	-3.264	84.270	14.845	18.586
10625	248	38	32	P	S	9.183	3.582	0.255	-2.439	-3.342	-0.268	2.565	84.323	18.586	14.844
10626	248	38	32	P	S	28.055	2.281	-4.773	-1.256	-2.182	4.896	1.289	73.293	16.362	17.508
10627	248	38	32	P	S	31.945	2.281	4.773	-1.256	-2.182	-4.896	1.289	73.293	16.362	17.508
10628	248	38	32	P	S	50.817	3.582	-0.255	-2.440	-3.342	0.268	2.565	84.323	18.586	14.844
10629	248	38	32	P	S	51.787	-2.359	1.484	3.492	2.476	-1.387	-3.264	84.270	14.845	18.586
10630	248	38	32	P	S	60.000	5.011	3.191	-3.675	-4.554	-3.444	3.966	73.398	16.799	17.070
10631	248	38	32	P	S	68.213	-2.359	-1.483	3.492	2.476	1.387	-3.264	84.270	14.845	18.586
10632	248	38	32	P	S	69.183	3.582	0.255	-2.439	-3.342	-0.268	2.565	84.323	18.586	14.844
10633	248	38	32	P	S	88.055	2.281	-4.773	-1.256	-2.182	4.896	1.289	73.293	16.362	17.508
10634	248	38	32	P	S	91.945	2.281	4.773	-1.256	-2.182	-4.896	1.289	73.293	16.362	17.508
10635	248	38	32	P	S	110.817	3.582	-0.255	-2.440	-3.342	0.268	2.565	84.323	18.586	14.844
10636	248	38	32	P	S	111.787	-2.359	1.484	3.492	2.476	-1.387	-3.264	84.270	14.845	18.586
10637	248	38	32	P	W	0.000	5.011	-3.191	-3.675	-4.554	3.444	3.966	73.398	16.799	17.070
10638	248	38	32	P	W	8.213	-2.359	-1.483	3.492	2.476	1.387	-3.264	84.270	14.845	18.586
10639	248	38	32	P	W	9.183	3.582	0.255	-2.439	-3.342	-0.268	2.565	84.323	18.586	14.844
10640	248	38	32	P	W	28.055	2.281	-4.773	-1.256	-2.182	4.896	1.289	73.293	16.362	17.508
10641	248	38	32	P	W	31.945	2.281	4.773	-1.256	-2.182	-4.896	1.289	73.293	16.362	17.508
10642	248	38	32	P	W	50.817	3.582	-0.255	-2.440	-3.342	0.268	2.565	84.323	18.586	14.844
10643	248	38	32	P	W	51.787	-2.359	1.484	3.492	2.476	-1.387	-3.264	84.270	14.845	18.586
10644	248	38	32	P	W	60.000	5.011	3.191	-3.675	-4.554	-3.444	3.966	73.398	16.799	17.070
10645	248	38	32	P	W	68.213	-2.359	-1.483	3.492	2.476	1.387	-3.264	84.270	14.845	18.586
10646	248	38	32	P	W	69.183	3.582	0.255	-2.439	-3.342	-0.268	2.565	84.323	18.586	14.844
10647	248	38	32	P	W	88.055	2.281	-4.773	-1.256	-2.182	4.896	1.289	73.293	16.362	17.508
10648	248	38	32	P	W	91.945	2.281	4.773	-1.256	-2.182	-4.896	1.289	73.293	16.362	17.508
10649	248	38	32	P	W	110.817	3.582	-0.255	-2.440	-3.342	0.268	2.565	84.323	18.586	14.844
10650	248	38	32	P	W	111.787	-2.359	1.484	3.492	2.476	-1.387	-3.264	84.270	14.845	18.586
10651	249	36	35	C	S	27.796	5.011	-2.526	3.483	-4.554	2.362	-3.256	74.969	18.842	15.369
10652	249	36	35	C	S	32.204	5.011	2.526	3.483	-4.554	-2.362	-3.256	74.969	18.842	15.369
10653	249	36	35	C	S	87.796	5.011	-2.526	3.483	-4.554	2.362	-3.256	74.969	18.842	15.369
10654	249	36	35	C	S	92.204	5.011	2.526	3.483	-4.554	-2.362	-3.256	74.969	18.842	15.369
10655	249	36	35	C	W	27.796	5.011	-2.526	3.483	-4.554	2.362	-3.256	74.969	18.842	15.369
10656	249	36	35	C	W	32.204	5.011	2.526	3.483	-4.554	-2.362	-3.256	74.969	18.842	15.369
10657	249	36	35	C	W	87.796	5.011	-2.526	3.483	-4.554	2.362	-3.256	74.969	18.842	15.369
10658	249	36	35	C	W	92.204	5.011	2.526	3.483	-4.554	-2.362	-3.256	74.969	18.842	15.369
10659	249	36	35	P	S	27.796	5.011	-2.526	3.483	-4.554	2.362	-3.256	74.969	18.842	15.369

BL number	Atoms	γ-PC unit cells	WS_2 unit cells	γ-PC origin atom	WS_2 origin atom	Twist-angle (°)	γ-PC strain 1 (%)	γ-PC strain 2 (%)	γ-PC strain 3 (%)	WS_2 strain 1 (%)	WS_2 strain 2 (%)	WS_2 strain 3 (%)	γ (°)	|**a**|(Å)	|**b**|(Å)
10660	249	36	35	P	S	92.204	5.011	2.526	3.483	-4.554	-2.362	-3.256	74.969	18.842	15.369
10661	249	36	35	P	W	32.204	5.011	2.526	3.483	-4.554	-2.362	-3.256	74.969	18.842	15.369
10662	249	36	35	P	W	87.796	5.011	-2.526	3.483	-4.554	2.362	-3.256	74.969	18.842	15.369
10663	249	39	31	C	S	7.154	-0.955	0.000	-0.955	0.973	0.000	0.973	60.000	17.772	17.772
10664	249	39	31	C	S	25.050	-0.955	0.000	-0.955	0.973	0.000	0.973	60.000	17.772	17.772
10665	249	39	31	C	S	34.950	-0.955	0.000	-0.955	0.973	0.000	0.973	60.000	17.772	17.772
10666	249	39	31	C	S	52.846	-0.955	0.000	-0.955	0.973	0.000	0.973	60.000	17.772	17.772
10667	249	39	31	C	S	67.154	-0.955	0.000	-0.955	0.973	0.000	0.973	60.000	17.772	17.772
10668	249	39	31	C	S	85.050	-0.955	0.000	-0.955	0.973	0.000	0.973	60.000	17.772	17.772
10669	249	39	31	C	S	94.950	-0.955	0.000	-0.955	0.973	0.000	0.973	60.000	17.772	17.772
10670	249	39	31	C	S	112.846	-0.955	0.000	-0.955	0.973	0.000	0.973	60.000	17.772	17.772
10671	249	39	31	P	S	7.154	-0.955	0.000	-0.955	0.973	0.000	0.973	60.000	17.772	17.772
10672	249	39	31	P	S	25.050	-0.955	0.000	-0.955	0.973	0.000	0.973	60.000	17.772	17.772
10673	249	39	31	P	S	34.950	-0.955	0.000	-0.955	0.973	0.000	0.973	60.000	17.772	17.772
10674	249	39	31	P	S	52.846	-0.955	0.000	-0.955	0.973	0.000	0.973	60.000	17.772	17.772
10675	249	39	31	P	S	67.154	-0.955	0.000	-0.955	0.973	0.000	0.973	60.000	17.772	17.772
10676	249	39	31	P	S	85.050	-0.955	0.000	-0.955	0.973	0.000	0.973	60.000	17.772	17.772
10677	249	39	31	P	S	94.950	-0.955	0.000	-0.955	0.973	0.000	0.973	60.000	17.772	17.772
10678	249	39	31	P	S	112.846	-0.955	0.000	-0.955	0.973	0.000	0.973	60.000	17.772	17.772
10679	249	39	31	C	S	6.587	-3.853	-2.697	2.126	4.175	2.587	-2.039	81.366	14.354	19.291
10680	249	39	31	C	S	8.513	2.390	0.700	-4.086	-2.281	-0.762	4.449	81.496	19.291	14.352
10681	249	39	31	C	S	51.487	2.390	-0.700	-4.086	-2.281	0.762	4.449	81.496	19.291	14.352
10682	249	39	31	C	S	53.413	-3.853	2.697	2.126	4.175	-2.587	-2.039	81.366	14.354	19.291
10683	249	39	31	C	S	66.587	-3.853	-2.697	2.126	4.175	2.587	-2.039	81.366	14.354	19.291
10684	249	39	31	C	S	68.513	2.390	0.700	-4.086	-2.281	-0.762	4.449	81.496	19.291	14.352
10685	249	39	31	C	S	111.487	2.390	-0.700	-4.086	-2.281	0.762	4.449	81.496	19.291	14.352
10686	249	39	31	C	S	113.413	-3.853	2.697	2.126	4.175	-2.587	-2.039	81.366	14.354	19.291
10687	249	39	31	C	W	6.587	-3.853	-2.697	2.126	4.175	2.587	-2.039	81.366	14.354	19.291
10688	249	39	31	C	W	8.513	2.390	0.700	-4.086	-2.281	-0.762	4.449	81.496	19.291	14.352
10689	249	39	31	C	W	51.487	2.390	-0.700	-4.086	-2.281	0.762	4.449	81.496	19.291	14.352
10690	249	39	31	C	W	53.413	-3.853	2.697	2.126	4.175	-2.587	-2.039	81.366	14.354	19.291
10691	249	39	31	C	W	66.587	-3.853	-2.697	2.126	4.175	2.587	-2.039	81.366	14.354	19.291
10692	249	39	31	C	W	68.513	2.390	0.700	-4.086	-2.281	-0.762	4.449	81.496	19.291	14.352
10693	249	39	31	C	W	111.487	2.390	-0.700	-4.086	-2.281	0.762	4.449	81.496	19.291	14.352
10694	249	39	31	C	W	113.413	-3.853	2.697	2.126	4.175	-2.587	-2.039	81.366	14.354	19.291
10695	249	39	31	P	S	6.587	-3.853	-2.697	2.126	4.175	2.587	-2.039	81.366	14.354	19.291
10696	249	39	31	P	S	8.513	2.390	0.700	-4.086	-2.281	-0.762	4.449	81.496	19.291	14.352
10697	249	39	31	P	S	51.487	2.390	-0.700	-4.086	-2.281	0.762	4.449	81.496	19.291	14.352
10698	249	39	31	P	S	53.413	-3.853	2.697	2.126	4.175	-2.587	-2.039	81.366	14.354	19.291
10699	249	39	31	P	S	66.587	-3.853	-2.697	2.126	4.175	2.587	-2.039	81.366	14.354	19.291
10700	249	39	31	P	S	68.513	2.390	0.700	-4.086	-2.281	-0.762	4.449	81.496	19.291	14.352
10701	249	39	31	P	S	111.487	2.390	-0.700	-4.086	-2.281	0.762	4.449	81.496	19.291	14.352
10702	249	39	31	P	S	113.413	-3.853	2.697	2.126	4.175	-2.587	-2.039	81.366	14.354	19.291
10703	249	39	31	P	W	6.587	-3.853	-2.697	2.126	4.175	2.587	-2.039	81.366	14.354	19.291
10704	249	39	31	P	W	8.513	2.390	0.700	-4.086	-2.281	-0.762	4.449	81.496	19.291	14.352
10705	249	39	31	P	W	51.487	2.390	-0.700	-4.086	-2.281	0.762	4.449	81.496	19.291	14.352
10706	249	39	31	P	W	53.413	-3.853	2.697	2.126	4.175	-2.587	-2.039	81.366	14.354	19.291
10707	249	39	31	P	W	66.587	-3.853	-2.697	2.126	4.175	2.587	-2.039	81.366	14.354	19.291
10708	249	39	31	P	W	68.513	2.390	0.700	-4.086	-2.281	-0.762	4.449	81.496	19.291	14.352
10709	249	39	31	P	W	111.487	2.390	-0.700	-4.086	-2.281	0.762	4.449	81.496	19.291	14.352
10710	249	39	31	P	W	113.413	-3.853	2.697	2.126	4.175	-2.587	-2.039	81.366	14.354	19.291

BL number	Atoms	γ-PC unit cells	WS_2 unit cells	γ-PC origin atom	WS_2 origin atom	Twist-angle (°)	γ-PC strain 1 (%)	γ-PC strain 2 (%)	γ-PC strain 3 (%)	WS_2 strain 1 (%)	WS_2 strain 2 (%)	WS_2 strain 3 (%)	γ (°)	|**a**|(Å)	|**b**|(Å)
10711	250	37	34	C	S	8.513	2.390	-1.536	3.080	-2.281	1.447	-2.901	66.663	18.582	16.363
10712	250	37	34	C	S	9.183	3.582	-0.328	1.899	-3.342	0.316	-1.829	66.661	18.586	16.361
10713	250	37	34	C	S	27.126	1.029	-3.771	4.495	-1.008	3.460	-4.125	65.045	18.838	16.350
10714	250	37	34	C	S	27.796	5.011	-2.458	0.551	-4.554	2.431	-0.545	65.088	18.842	16.344
10715	250	37	34	C	S	28.055	2.281	-4.959	3.190	-2.182	4.662	-2.999	65.031	16.362	18.822
10716	250	37	34	C	S	31.945	2.281	4.959	3.190	-2.182	-4.662	-2.999	65.031	16.362	18.822
10717	250	37	34	C	S	32.204	5.011	2.458	0.551	-4.554	-2.431	-0.545	65.088	18.842	16.344
10718	250	37	34	C	S	32.874	1.029	3.771	4.495	-1.008	-3.460	-4.124	65.045	16.350	18.838
10719	250	37	34	C	S	49.842	2.281	-1.425	3.190	-2.182	1.339	-2.999	66.664	16.362	18.583
10720	250	37	34	C	S	50.817	3.582	0.328	1.899	-3.342	-0.316	-1.829	66.661	18.586	16.361
10721	250	37	34	C	S	68.513	2.390	-1.536	3.080	-2.281	1.447	-2.901	66.663	18.582	16.363
10722	250	37	34	C	S	69.183	3.582	-0.328	1.899	-3.342	0.316	-1.829	66.661	18.586	16.361
10723	250	37	34	C	S	87.126	1.029	-3.771	4.495	-1.008	3.460	-4.125	65.045	18.838	16.350
10724	250	37	34	C	S	87.796	5.011	-2.458	0.551	-4.554	2.431	-0.545	65.088	18.842	16.344
10725	250	37	34	C	S	88.055	2.281	-4.959	3.190	-2.182	4.662	-2.999	65.031	16.362	18.822
10726	250	37	34	C	S	91.945	2.281	4.959	3.190	-2.182	-4.662	-2.999	65.031	16.362	18.822
10727	250	37	34	C	S	92.204	5.011	2.458	0.551	-4.554	-2.431	-0.545	65.088	18.842	16.344
10728	250	37	34	C	S	92.874	1.029	3.771	4.495	-1.008	-3.460	-4.124	65.045	16.350	18.838
10729	250	37	34	C	S	109.842	2.281	-1.425	3.190	-2.182	1.339	-2.999	66.664	16.362	18.583
10730	250	37	34	C	S	110.817	3.582	0.328	1.899	-3.342	-0.316	-1.829	66.661	18.586	16.361
10731	250	37	34	C	W	8.513	2.390	-1.536	3.080	-2.281	1.447	-2.901	66.663	18.582	16.363
10732	250	37	34	C	W	9.183	3.582	-0.328	1.899	-3.342	0.316	-1.829	66.661	18.586	16.361
10733	250	37	34	C	W	27.126	1.029	-3.771	4.495	-1.008	3.460	-4.125	65.045	18.838	16.350
10734	250	37	34	C	W	27.796	5.011	-2.458	0.551	-4.554	2.431	-0.545	65.088	18.842	16.344
10735	250	37	34	C	W	28.055	2.281	-4.959	3.190	-2.182	4.662	-2.999	65.031	16.362	18.822
10736	250	37	34	C	W	31.945	2.281	4.959	3.190	-2.182	-4.662	-2.999	65.031	16.362	18.822
10737	250	37	34	C	W	32.204	5.011	2.458	0.551	-4.554	-2.431	-0.545	65.088	18.842	16.344
10738	250	37	34	C	W	32.874	1.029	3.771	4.495	-1.008	-3.460	-4.124	65.045	16.350	18.838
10739	250	37	34	C	W	49.842	2.281	-1.425	3.190	-2.182	1.339	-2.999	66.664	16.362	18.583
10740	250	37	34	C	W	50.817	3.582	0.328	1.899	-3.342	-0.316	-1.829	66.661	18.586	16.361
10741	250	37	34	C	W	68.513	2.390	-1.536	3.080	-2.281	1.447	-2.901	66.663	18.582	16.363
10742	250	37	34	C	W	69.183	3.582	-0.328	1.899	-3.342	0.316	-1.829	66.661	18.586	16.361
10743	250	37	34	C	W	87.126	1.029	-3.771	4.495	-1.008	3.460	-4.125	65.045	18.838	16.350
10744	250	37	34	C	W	87.796	5.011	-2.458	0.551	-4.554	2.431	-0.545	65.088	18.842	16.344
10745	250	37	34	C	W	88.055	2.281	-4.959	3.190	-2.182	4.662	-2.999	65.031	16.362	18.822
10746	250	37	34	C	W	91.945	2.281	4.959	3.190	-2.182	-4.662	-2.999	65.031	16.362	18.822
10747	250	37	34	C	W	92.204	5.011	2.458	0.551	-4.554	-2.431	-0.545	65.088	18.842	16.344
10748	250	37	34	C	W	92.874	1.029	3.771	4.495	-1.008	-3.460	-4.124	65.045	16.350	18.838
10749	250	37	34	C	W	109.842	2.281	-1.425	3.190	-2.182	1.339	-2.999	66.664	16.362	18.583
10750	250	37	34	C	W	110.817	3.582	0.328	1.899	-3.342	-0.316	-1.829	66.661	18.586	16.361
10751	250	37	34	P	S	8.513	2.390	-1.536	3.080	-2.281	1.447	-2.901	66.663	18.582	16.363
10752	250	37	34	P	S	9.183	3.582	-0.328	1.899	-3.342	0.316	-1.829	66.661	18.586	16.361
10753	250	37	34	P	S	27.126	1.029	-3.771	4.495	-1.008	3.460	-4.125	65.045	18.838	16.350
10754	250	37	34	P	S	27.796	5.011	-2.458	0.551	-4.554	2.431	-0.545	65.088	18.842	16.344
10755	250	37	34	P	S	31.945	2.281	4.959	3.190	-2.182	-4.662	-2.999	65.031	16.362	18.822
10756	250	37	34	P	S	49.842	2.281	-1.425	3.190	-2.182	1.339	-2.999	66.664	16.362	18.583
10757	250	37	34	P	S	50.817	3.582	0.328	1.899	-3.342	-0.316	-1.829	66.661	18.586	16.361
10758	250	37	34	P	S	68.513	2.390	-1.536	3.080	-2.281	1.447	-2.901	66.663	18.582	16.363
10759	250	37	34	P	S	69.183	3.582	-0.328	1.899	-3.342	0.316	-1.829	66.661	18.586	16.361
10760	250	37	34	P	S	88.055	2.281	-4.959	3.190	-2.182	4.662	-2.999	65.031	16.362	18.822
10761	250	37	34	P	S	92.204	5.011	2.458	0.551	-4.554	-2.431	-0.545	65.088	18.842	16.344

BL number	Atoms	γ-PC unit cells	WS_2 unit cells	γ-PC origin atom	WS_2 origin atom	Twist-angle (°)	γ-PC strain 1 (%)	γ-PC strain 2 (%)	γ-PC strain 3 (%)	WS_2 strain 1 (%)	WS_2 strain 2 (%)	WS_2 strain 3 (%)	γ (°)	|**a**|(Å)	|**b**|(Å)
10762	250	37	34	P	S	92.874	1.029	3.771	4.495	-1.008	-3.460	-4.124	65.045	16.350	18.838
10763	250	37	34	P	S	109.842	2.281	-1.425	3.190	-2.182	1.339	-2.999	66.664	16.362	18.583
10764	250	37	34	P	S	110.817	3.582	0.328	1.899	-3.342	-0.316	-1.829	66.661	18.586	16.361
10765	250	37	34	P	W	8.513	2.390	-1.536	3.080	-2.281	1.447	-2.901	66.663	18.582	16.363
10766	250	37	34	P	W	9.183	3.582	-0.328	1.899	-3.342	0.316	-1.829	66.661	18.586	16.361
10767	250	37	34	P	W	28.055	2.281	-4.959	3.190	-2.182	4.662	-2.999	65.031	16.362	18.822
10768	250	37	34	P	W	32.204	5.011	2.458	0.551	-4.554	-2.431	-0.545	65.088	18.842	16.344
10769	250	37	34	P	W	32.874	1.029	3.771	4.495	-1.008	-3.460	-4.124	65.045	16.350	18.838
10770	250	37	34	P	W	49.842	2.281	-1.425	3.190	-2.182	1.339	-2.999	66.664	16.362	18.583
10771	250	37	34	P	W	50.817	3.582	0.328	1.899	-3.342	-0.316	-1.829	66.661	18.586	16.361
10772	250	37	34	P	W	68.513	2.390	-1.536	3.080	-2.281	1.447	-2.901	66.663	18.582	16.363
10773	250	37	34	P	W	69.183	3.582	-0.328	1.899	-3.342	0.316	-1.829	66.661	18.586	16.361
10774	250	37	34	P	W	87.126	1.029	-3.771	4.495	-1.008	3.460	-4.125	65.045	18.838	16.350
10775	250	37	34	P	W	87.796	5.011	-2.458	0.551	-4.554	2.431	-0.545	65.088	18.842	16.344
10776	250	37	34	P	W	91.945	2.281	4.959	3.190	-2.182	-4.662	-2.999	65.031	16.362	18.822
10777	250	37	34	P	W	109.842	2.281	-1.425	3.190	-2.182	1.339	-2.999	66.664	16.362	18.583
10778	250	37	34	P	W	110.817	3.582	0.328	1.899	-3.342	-0.316	-1.829	66.661	18.586	16.361
10779	250	40	30	C	S	4.307	-4.685	-3.331	0.086	5.169	3.325	-0.085	84.956	14.491	18.899
10780	250	40	30	C	S	4.715	-3.007	-0.933	-1.702	3.200	0.966	1.762	70.161	16.951	17.099
10781	250	40	30	C	S	6.178	-2.145	1.564	-2.572	2.241	-1.648	2.712	70.157	17.102	16.948
10782	250	40	30	C	S	7.589	0.334	2.293	-4.909	-0.332	-2.543	5.443	85.140	18.904	14.485
10783	250	40	30	C	S	8.948	-0.599	4.591	-4.057	0.606	-4.997	4.415	82.167	15.901	17.312
10784	250	40	30	C	S	51.052	-0.599	-4.591	-4.057	0.606	4.997	4.415	82.167	15.901	17.312
10785	250	40	30	C	S	52.411	0.334	-2.293	-4.909	-0.332	2.543	5.443	85.140	18.904	14.485
10786	250	40	30	C	S	53.822	-2.145	-1.564	-2.572	2.241	1.648	2.712	70.157	17.102	16.948
10787	250	40	30	C	S	55.285	-1.702	0.920	-3.007	1.762	-0.979	3.200	70.145	16.950	17.102
10788	250	40	30	C	S	55.693	-4.685	3.331	0.085	5.169	-3.325	-0.085	84.956	14.491	18.899
10789	250	40	30	C	S	64.307	-4.685	-3.331	0.086	5.169	3.325	-0.085	84.956	14.491	18.899
10790	250	40	30	C	S	64.715	-3.007	-0.933	-1.702	3.200	0.966	1.762	70.161	16.951	17.099
10791	250	40	30	C	S	66.178	-2.145	1.564	-2.572	2.241	-1.648	2.712	70.157	17.102	16.948
10792	250	40	30	C	S	67.589	0.334	2.293	-4.909	-0.332	-2.543	5.443	85.140	18.904	14.485
10793	250	40	30	C	S	68.948	-0.599	4.591	-4.057	0.606	-4.997	4.415	82.167	15.901	17.312
10794	250	40	30	C	S	111.052	-0.599	-4.591	-4.057	0.606	4.997	4.415	82.167	15.901	17.312
10795	250	40	30	C	S	112.411	0.334	-2.293	-4.909	-0.332	2.543	5.443	85.140	18.904	14.485
10796	250	40	30	C	S	113.822	-2.145	-1.564	-2.572	2.241	1.648	2.712	70.157	17.102	16.948
10797	250	40	30	C	S	115.285	-3.007	0.933	-1.702	3.200	-0.966	1.762	70.161	16.951	17.099
10798	250	40	30	C	S	115.693	-4.685	3.331	0.085	5.169	-3.325	-0.085	84.956	14.491	18.899
10799	250	40	30	C	W	4.307	-4.685	-3.331	0.086	5.169	3.325	-0.085	84.956	14.491	18.899
10800	250	40	30	C	W	4.715	-3.007	-0.933	-1.702	3.200	0.966	1.762	70.161	16.951	17.099
10801	250	40	30	C	W	6.178	-2.145	1.564	-2.572	2.241	-1.648	2.712	70.157	17.102	16.948
10802	250	40	30	C	W	7.589	0.334	2.293	-4.909	-0.332	-2.543	5.443	85.140	18.904	14.485
10803	250	40	30	C	W	8.948	-0.599	4.591	-4.057	0.606	-4.997	4.415	82.167	15.901	17.312
10804	250	40	30	C	W	51.052	-0.599	-4.591	-4.057	0.606	4.997	4.415	82.167	15.901	17.312
10805	250	40	30	C	W	52.411	0.334	-2.293	-4.909	-0.332	2.543	5.443	85.140	18.904	14.485
10806	250	40	30	C	W	53.822	-2.145	-1.564	-2.572	2.241	1.648	2.712	70.157	17.102	16.948
10807	250	40	30	C	W	55.285	-1.702	0.920	-3.007	1.762	-0.979	3.200	70.145	16.950	17.102
10808	250	40	30	C	W	55.693	-4.685	3.331	0.085	5.169	-3.325	-0.085	84.956	14.491	18.899
10809	250	40	30	C	W	64.307	-4.685	-3.331	0.086	5.169	3.325	-0.085	84.956	14.491	18.899
10810	250	40	30	C	W	64.715	-3.007	-0.933	-1.702	3.200	0.966	1.762	70.161	16.951	17.099
10811	250	40	30	C	W	66.178	-2.145	1.564	-2.572	2.241	-1.648	2.712	70.157	17.102	16.948
10812	250	40	30	C	W	67.589	0.334	2.293	-4.909	-0.332	-2.543	5.443	85.140	18.904	14.485

BL number	Atoms	γ-PC unit cells	WS_2 unit cells	γ-PC origin atom	WS_2 origin atom	Twist-angle (°)	γ-PC strain 1 (%)	γ-PC strain 2 (%)	γ-PC strain 3 (%)	WS_2 strain 1 (%)	WS_2 strain 2 (%)	WS_2 strain 3 (%)	γ (°)	|**a**|(Å)	|**b**|(Å)
10813	250	40	30	C	W	68.948	-0.599	4.591	-4.057	0.606	-4.997	4.415	82.167	15.901	17.312
10814	250	40	30	C	W	111.052	-0.599	-4.591	-4.057	0.606	4.997	4.415	82.167	15.901	17.312
10815	250	40	30	C	W	112.411	0.334	-2.293	-4.909	-0.332	2.543	5.443	85.140	18.904	14.485
10816	250	40	30	C	W	113.822	-2.145	-1.564	-2.572	2.241	1.648	2.712	70.157	17.102	16.948
10817	250	40	30	C	W	115.285	-3.007	0.933	-1.702	3.200	-0.966	1.762	70.161	16.951	17.099
10818	250	40	30	C	W	115.693	-4.685	3.331	0.085	5.169	-3.325	-0.085	84.956	14.491	18.899
10819	250	40	30	P	S	4.307	-4.685	-3.331	0.086	5.169	3.325	-0.085	84.956	14.491	18.899
10820	250	40	30	P	S	4.715	-3.007	-0.933	-1.702	3.200	0.966	1.762	70.161	16.951	17.099
10821	250	40	30	P	S	6.178	-2.145	1.564	-2.572	2.241	-1.648	2.712	70.157	17.102	16.948
10822	250	40	30	P	S	7.589	0.334	2.293	-4.909	-0.332	-2.543	5.443	85.140	18.904	14.485
10823	250	40	30	P	S	8.948	-0.599	4.591	-4.057	0.606	-4.997	4.415	82.167	15.901	17.312
10824	250	40	30	P	S	51.052	-0.599	-4.591	-4.057	0.606	4.997	4.415	82.167	15.901	17.312
10825	250	40	30	P	S	52.411	0.334	-2.293	-4.909	-0.332	2.543	5.443	85.140	18.904	14.485
10826	250	40	30	P	S	53.822	-2.145	-1.564	-2.572	2.241	1.648	2.712	70.157	17.102	16.948
10827	250	40	30	P	S	55.285	-1.702	0.920	-3.007	1.762	-0.979	3.200	70.145	16.950	17.102
10828	250	40	30	P	S	55.693	-4.685	3.331	0.085	5.169	-3.325	-0.085	84.956	14.491	18.899
10829	250	40	30	P	S	64.307	-4.685	-3.331	0.086	5.169	3.325	-0.085	84.956	14.491	18.899
10830	250	40	30	P	S	64.715	-3.007	-0.933	-1.702	3.200	0.966	1.762	70.161	16.951	17.099
10831	250	40	30	P	S	66.178	-2.145	1.564	-2.572	2.241	-1.648	2.712	70.157	17.102	16.948
10832	250	40	30	P	S	67.589	0.334	2.293	-4.909	-0.332	-2.543	5.443	85.140	18.904	14.485
10833	250	40	30	P	S	68.948	-0.599	4.591	-4.057	0.606	-4.997	4.415	82.167	15.901	17.312
10834	250	40	30	P	S	111.052	-0.599	-4.591	-4.057	0.606	4.997	4.415	82.167	15.901	17.312
10835	250	40	30	P	S	112.411	0.334	-2.293	-4.909	-0.332	2.543	5.443	85.140	18.904	14.485
10836	250	40	30	P	S	113.822	-2.145	-1.564	-2.572	2.241	1.648	2.712	70.157	17.102	16.948
10837	250	40	30	P	S	115.285	-3.007	0.933	-1.702	3.200	-0.966	1.762	70.161	16.951	17.099
10838	250	40	30	P	S	115.693	-4.685	3.331	0.085	5.169	-3.325	-0.085	84.956	14.491	18.899
10839	250	40	30	P	W	4.307	-4.685	-3.331	0.086	5.169	3.325	-0.085	84.956	14.491	18.899
10840	250	40	30	P	W	4.715	-3.007	-0.933	-1.702	3.200	0.966	1.762	70.161	16.951	17.099
10841	250	40	30	P	W	6.178	-2.145	1.564	-2.572	2.241	-1.648	2.712	70.157	17.102	16.948
10842	250	40	30	P	W	7.589	0.334	2.293	-4.909	-0.332	-2.543	5.443	85.140	18.904	14.485
10843	250	40	30	P	W	8.948	-0.599	4.591	-4.057	0.606	-4.997	4.415	82.167	15.901	17.312
10844	250	40	30	P	W	51.052	-0.599	-4.591	-4.057	0.606	4.997	4.415	82.167	15.901	17.312
10845	250	40	30	P	W	52.411	0.334	-2.293	-4.909	-0.332	2.543	5.443	85.140	18.904	14.485
10846	250	40	30	P	W	53.822	-2.145	-1.564	-2.572	2.241	1.648	2.712	70.157	17.102	16.948
10847	250	40	30	P	W	55.285	-1.702	0.920	-3.007	1.762	-0.979	3.200	70.145	16.950	17.102
10848	250	40	30	P	W	55.693	-4.685	3.331	0.085	5.169	-3.325	-0.085	84.956	14.491	18.899
10849	250	40	30	P	W	64.307	-4.685	-3.331	0.086	5.169	3.325	-0.085	84.956	14.491	18.899
10850	250	40	30	P	W	64.715	-3.007	-0.933	-1.702	3.200	0.966	1.762	70.161	16.951	17.099
10851	250	40	30	P	W	66.178	-2.145	1.564	-2.572	2.241	-1.648	2.712	70.157	17.102	16.948
10852	250	40	30	P	W	67.589	0.334	2.293	-4.909	-0.332	-2.543	5.443	85.140	18.904	14.485
10853	250	40	30	P	W	68.948	-0.599	4.591	-4.057	0.606	-4.997	4.415	82.167	15.901	17.312
10854	250	40	30	P	W	111.052	-0.599	-4.591	-4.057	0.606	4.997	4.415	82.167	15.901	17.312
10855	250	40	30	P	W	112.411	0.334	-2.293	-4.909	-0.332	2.543	5.443	85.140	18.904	14.485
10856	250	40	30	P	W	113.822	-2.145	-1.564	-2.572	2.241	1.648	2.712	70.157	17.102	16.948
10857	250	40	30	P	W	115.285	-3.007	0.933	-1.702	3.200	-0.966	1.762	70.161	16.951	17.099
10858	250	40	30	P	W	115.693	-4.685	3.331	0.085	5.169	-3.325	-0.085	84.956	14.491	18.899
10859	251	38	33	C	S	7.154	-0.955	-4.005	3.583	0.973	3.737	-3.344	82.828	17.772	15.807
10860	251	38	33	C	S	10.893	4.020	2.634	-1.351	-3.721	-2.707	1.388	82.998	15.815	17.760
10861	251	38	33	C	S	21.052	1.339	-4.362	1.189	-1.304	4.261	-1.162	78.671	16.211	17.526
10862	251	38	33	C	S	25.767	0.353	4.009	2.192	-0.351	-3.840	-2.099	78.718	17.539	16.198
10863	251	38	33	C	S	27.796	5.011	-2.393	-2.227	-4.554	2.505	2.331	85.800	18.842	14.843

BL number	Atoms	γ-PC unit cells	WS_2 unit cells	γ-PC origin atom	WS_2 origin atom	Twist-angle (°)	γ-PC strain 1 (%)	γ-PC strain 2 (%)	γ-PC strain 3 (%)	WS_2 strain 1 (%)	WS_2 strain 2 (%)	WS_2 strain 3 (%)	γ (°)	|**a**|(Å)	|**b**|(Å)
10864	251	38	33	C	S	30.000	-2.359	-1.483	5.163	2.476	1.345	-4.680	85.965	14.845	18.838
10865	251	38	33	C	S	32.204	5.011	2.393	-2.227	-4.554	-2.505	2.331	85.800	18.842	14.843
10866	251	38	33	C	S	34.233	0.353	-4.009	2.192	-0.351	3.840	-2.099	78.718	17.539	16.198
10867	251	38	33	C	S	38.948	1.339	4.363	1.189	-1.304	-4.261	-1.162	78.671	16.211	17.526
10868	251	38	33	C	S	49.107	4.020	-2.634	-1.351	-3.721	2.707	1.388	82.998	15.815	17.760
10869	251	38	33	C	S	52.846	-0.955	4.005	3.583	0.973	-3.737	-3.344	82.828	17.772	15.807
10870	251	38	33	C	S	67.154	-0.955	-4.005	3.583	0.973	3.737	-3.344	82.828	17.772	15.807
10871	251	38	33	C	S	70.893	4.020	2.634	-1.351	-3.721	-2.707	1.388	82.998	15.815	17.760
10872	251	38	33	C	S	81.052	1.339	-4.362	1.189	-1.304	4.261	-1.162	78.671	16.211	17.526
10873	251	38	33	C	S	85.767	0.353	4.009	2.192	-0.351	-3.840	-2.099	78.718	17.539	16.198
10874	251	38	33	C	S	87.796	5.011	-2.393	-2.227	-4.554	2.505	2.331	85.800	18.842	14.843
10875	251	38	33	C	S	90.000	-2.359	1.484	5.163	2.476	-1.345	-4.680	85.965	14.845	18.838
10876	251	38	33	C	S	92.204	5.011	2.393	-2.227	-4.554	-2.505	2.331	85.800	18.842	14.843
10877	251	38	33	C	S	94.233	0.353	-4.009	2.192	-0.351	3.840	-2.099	78.718	17.539	16.198
10878	251	38	33	C	S	98.948	1.339	4.363	1.189	-1.304	-4.261	-1.162	78.671	16.211	17.526
10879	251	38	33	C	S	109.107	4.020	-2.634	-1.351	-3.721	2.707	1.388	82.998	15.815	17.760
10880	251	38	33	C	S	112.846	-0.955	4.005	3.583	0.973	-3.737	-3.344	82.828	17.772	15.807
10881	251	38	33	C	W	7.154	-0.955	-4.005	3.583	0.973	3.737	-3.344	82.828	17.772	15.807
10882	251	38	33	C	W	10.893	4.020	2.634	-1.351	-3.721	-2.707	1.388	82.998	15.815	17.760
10883	251	38	33	C	W	21.052	1.339	-4.362	1.189	-1.304	4.261	-1.162	78.671	16.211	17.526
10884	251	38	33	C	W	25.767	0.353	4.009	2.192	-0.351	-3.840	-2.099	78.718	17.539	16.198
10885	251	38	33	C	W	27.796	5.011	-2.393	-2.227	-4.554	2.505	2.331	85.800	18.842	14.843
10886	251	38	33	C	W	30.000	-2.359	-1.483	5.163	2.476	1.345	-4.680	85.965	14.845	18.838
10887	251	38	33	C	W	32.204	5.011	2.393	-2.227	-4.554	-2.505	2.331	85.800	18.842	14.843
10888	251	38	33	C	W	34.233	0.353	-4.009	2.192	-0.351	3.840	-2.099	78.718	17.539	16.198
10889	251	38	33	C	W	38.948	1.339	4.363	1.189	-1.304	-4.261	-1.162	78.671	16.211	17.526
10890	251	38	33	C	W	49.107	4.020	-2.634	-1.351	-3.721	2.707	1.388	82.998	15.815	17.760
10891	251	38	33	C	W	52.846	-0.955	4.005	3.583	0.973	-3.737	-3.344	82.828	17.772	15.807
10892	251	38	33	C	W	67.154	-0.955	-4.005	3.583	0.973	3.737	-3.344	82.828	17.772	15.807
10893	251	38	33	C	W	70.893	4.020	2.634	-1.351	-3.721	-2.707	1.388	82.998	15.815	17.760
10894	251	38	33	C	W	81.052	1.339	-4.362	1.189	-1.304	4.261	-1.162	78.671	16.211	17.526
10895	251	38	33	C	W	85.767	0.353	4.009	2.192	-0.351	-3.840	-2.099	78.718	17.539	16.198
10896	251	38	33	C	W	87.796	5.011	-2.393	-2.227	-4.554	2.505	2.331	85.800	18.842	14.843
10897	251	38	33	C	W	90.000	-2.359	1.484	5.163	2.476	-1.345	-4.680	85.965	14.845	18.838
10898	251	38	33	C	W	92.204	5.011	2.393	-2.227	-4.554	-2.505	2.331	85.800	18.842	14.843
10899	251	38	33	C	W	94.233	0.353	-4.009	2.192	-0.351	3.840	-2.099	78.718	17.539	16.198
10900	251	38	33	C	W	98.948	1.339	4.363	1.189	-1.304	-4.261	-1.162	78.671	16.211	17.526
10901	251	38	33	C	W	109.107	4.020	-2.634	-1.351	-3.721	2.707	1.388	82.998	15.815	17.760
10902	251	38	33	C	W	112.846	-0.955	4.005	3.583	0.973	-3.737	-3.344	82.828	17.772	15.807
10903	251	38	33	P	S	7.154	-0.955	-4.005	3.583	0.973	3.737	-3.344	82.828	17.772	15.807
10904	251	38	33	P	S	10.893	4.020	2.634	-1.351	-3.721	-2.707	1.388	82.998	15.815	17.760
10905	251	38	33	P	S	21.052	1.339	-4.362	1.189	-1.304	4.261	-1.162	78.671	16.211	17.526
10906	251	38	33	P	S	25.767	0.353	4.009	2.192	-0.351	-3.840	-2.099	78.718	17.539	16.198
10907	251	38	33	P	S	27.796	5.011	-2.393	-2.227	-4.554	2.505	2.331	85.800	18.842	14.843
10908	251	38	33	P	S	30.000	-2.359	1.484	5.163	2.476	-1.345	-4.680	85.965	14.845	18.838
10909	251	38	33	P	S	34.233	0.353	-4.009	2.192	-0.351	3.840	-2.099	78.718	17.539	16.198
10910	251	38	33	P	S	38.948	1.339	4.363	1.189	-1.304	-4.261	-1.162	78.671	16.211	17.526
10911	251	38	33	P	S	49.107	4.020	-2.634	-1.351	-3.721	2.707	1.388	82.998	15.815	17.760
10912	251	38	33	P	S	52.846	-0.955	4.005	3.583	0.973	-3.737	-3.344	82.828	17.772	15.807
10913	251	38	33	P	S	67.154	-0.955	-4.005	3.583	0.973	3.737	-3.344	82.828	17.772	15.807
10914	251	38	33	P	S	70.893	4.020	2.634	-1.351	-3.721	-2.707	1.388	82.998	15.815	17.760

BL number	Atoms	γ-PC unit cells	WS_2 unit cells	γ-PC origin atom	WS_2 origin atom	Twist-angle (°)	γ-PC strain 1 (%)	γ-PC strain 2 (%)	γ-PC strain 3 (%)	WS_2 strain 1 (%)	WS_2 strain 2 (%)	WS_2 strain 3 (%)	γ (°)	$\lvert\mathbf{a}\rvert$(Å)	$\lvert\mathbf{b}\rvert$(Å)
10915	251	38	33	P	S	81.052	1.339	-4.362	1.189	-1.304	4.261	-1.162	78.671	16.211	17.526
10916	251	38	33	P	S	85.767	0.353	4.009	2.192	-0.351	-3.840	-2.099	78.718	17.539	16.198
10917	251	38	33	P	S	90.000	-2.359	-1.483	5.163	2.476	1.345	-4.680	85.965	14.845	18.838
10918	251	38	33	P	S	92.204	5.011	2.393	-2.227	-4.554	-2.505	2.331	85.800	18.842	14.843
10919	251	38	33	P	S	94.233	0.353	-4.009	2.192	-0.351	3.840	-2.099	78.718	17.539	16.198
10920	251	38	33	P	S	98.948	1.339	4.363	1.189	-1.304	-4.261	-1.162	78.671	16.211	17.526
10921	251	38	33	P	S	109.107	4.020	-2.634	-1.351	-3.721	2.707	1.388	82.998	15.815	17.760
10922	251	38	33	P	S	112.846	-0.955	4.005	3.583	0.973	-3.737	-3.344	82.828	17.772	15.807
10923	251	38	33	P	W	7.154	-0.955	-4.005	3.583	0.973	3.737	-3.344	82.828	17.772	15.807
10924	251	38	33	P	W	10.893	4.020	2.634	-1.351	-3.721	-2.707	1.388	82.998	15.815	17.760
10925	251	38	33	P	W	21.052	1.339	-4.362	1.189	-1.304	4.261	-1.162	78.671	16.211	17.526
10926	251	38	33	P	W	25.767	0.353	4.009	2.192	-0.351	-3.840	-2.099	78.718	17.539	16.198
10927	251	38	33	P	W	30.000	-2.359	-1.483	5.163	2.476	1.345	-4.680	85.965	14.845	18.838
10928	251	38	33	P	W	32.204	5.011	2.393	-2.227	-4.554	-2.505	2.331	85.800	18.842	14.843
10929	251	38	33	P	W	34.233	0.353	-4.009	2.192	-0.351	3.840	-2.099	78.718	17.539	16.198
10930	251	38	33	P	W	38.948	1.339	4.363	1.189	-1.304	-4.261	-1.162	78.671	16.211	17.526
10931	251	38	33	P	W	49.107	4.020	-2.634	-1.351	-3.721	2.707	1.388	82.998	15.815	17.760
10932	251	38	33	P	W	52.846	-0.955	4.005	3.583	0.973	-3.737	-3.344	82.828	17.772	15.807
10933	251	38	33	P	W	67.154	-0.955	-4.005	3.583	0.973	3.737	-3.344	82.828	17.772	15.807
10934	251	38	33	P	W	70.893	4.020	2.634	-1.351	-3.721	-2.707	1.388	82.998	15.815	17.760
10935	251	38	33	P	W	81.052	1.339	-4.362	1.189	-1.304	4.261	-1.162	78.671	16.211	17.526
10936	251	38	33	P	W	85.767	0.353	4.009	2.192	-0.351	-3.840	-2.099	78.718	17.539	16.198
10937	251	38	33	P	W	87.796	5.011	-2.393	-2.227	-4.554	2.505	2.331	85.800	18.842	14.843
10938	251	38	33	P	W	90.000	-2.359	1.484	5.163	2.476	-1.345	-4.680	85.965	14.845	18.838
10939	251	38	33	P	W	94.233	0.353	-4.009	2.192	-0.351	3.840	-2.099	78.718	17.539	16.198
10940	251	38	33	P	W	98.948	1.339	4.363	1.189	-1.304	-4.261	-1.162	78.671	16.211	17.526
10941	251	38	33	P	W	109.107	4.020	-2.634	-1.351	-3.721	2.707	1.388	82.998	15.815	17.760
10942	251	38	33	P	W	112.846	-0.955	4.005	3.583	0.973	-3.737	-3.344	82.828	17.772	15.807
10943	251	41	29	C	S	58.055	-4.723	-2.491	-2.725	5.216	2.635	2.882	78.203	15.242	18.208
10944	251	41	29	C	S	58.641	-3.292	-3.075	-4.174	3.523	3.355	4.554	78.167	18.221	15.232
10945	251	41	29	C	S	61.359	-3.292	3.075	-4.174	3.523	-3.355	4.554	78.168	18.221	15.232
10946	251	41	29	C	S	61.945	-4.723	2.492	-2.725	5.216	-2.635	2.882	78.203	15.242	18.208
10947	251	41	29	C	S	118.055	-4.723	-2.491	-2.725	5.216	2.635	2.882	78.203	15.242	18.208
10948	251	41	29	C	S	118.641	-3.292	-3.075	-4.174	3.523	3.355	4.554	78.168	18.221	15.232
10949	251	41	29	C	W	58.055	-4.723	-2.491	-2.725	5.216	2.635	2.882	78.203	15.242	18.208
10950	251	41	29	C	W	58.641	-3.292	-3.075	-4.174	3.523	3.355	4.554	78.167	18.221	15.232
10951	251	41	29	C	W	61.359	-3.292	3.075	-4.174	3.523	-3.355	4.554	78.168	18.221	15.232
10952	251	41	29	C	W	61.945	-4.723	2.492	-2.725	5.216	-2.635	2.882	78.203	15.242	18.208
10953	251	41	29	C	W	118.055	-4.723	-2.491	-2.725	5.216	2.635	2.882	78.203	15.242	18.208
10954	251	41	29	C	W	118.641	-3.292	-3.075	-4.174	3.523	3.355	4.554	78.168	18.221	15.232
10955	251	41	29	P	S	58.055	-4.723	-2.491	-2.725	5.216	2.635	2.882	78.203	15.242	18.208
10956	251	41	29	P	S	58.641	-3.292	-3.075	-4.174	3.523	3.355	4.554	78.167	18.221	15.232
10957	251	41	29	P	S	61.359	-3.292	3.075	-4.174	3.523	-3.355	4.554	78.168	18.221	15.232
10958	251	41	29	P	S	61.945	-4.723	2.492	-2.725	5.216	-2.635	2.882	78.203	15.242	18.208
10959	251	41	29	P	S	118.055	-4.723	-2.491	-2.725	5.216	2.635	2.882	78.203	15.242	18.208
10960	251	41	29	P	S	118.641	-3.292	-3.075	-4.174	3.523	3.355	4.554	78.168	18.221	15.232
10961	251	41	29	P	W	58.055	-4.723	-2.491	-2.725	5.216	2.635	2.882	78.203	15.242	18.208
10962	251	41	29	P	W	58.641	-3.292	-3.075	-4.174	3.523	3.355	4.554	78.167	18.221	15.232
10963	251	41	29	P	W	61.359	-3.292	3.075	-4.174	3.523	-3.355	4.554	78.168	18.221	15.232
10964	251	41	29	P	W	61.945	-4.723	2.492	-2.725	5.216	-2.635	2.882	78.203	15.242	18.208
10965	251	41	29	P	W	118.055	-4.723	-2.491	-2.725	5.216	2.635	2.882	78.203	15.242	18.208

BL number	Atoms	γ-PC unit cells	WS_2 unit cells	γ-PC origin atom	WS_2 origin atom	Twist-angle (°)	γ-PC strain 1 (%)	γ-PC strain 2 (%)	γ-PC strain 3 (%)	WS_2 strain 1 (%)	WS_2 strain 2 (%)	WS_2 strain 3 (%)	γ (°)	|**a**|(Å)	|**b**|(Å)
10966	251	41	29	P	W	118.641	-3.292	-3.075	-4.174	3.523	3.355	4.554	78.168	18.221	15.232
10967	252	39	32	C	S	0.000	5.011	2.332	-4.863	-4.554	-2.583	5.387	64.994	17.338	17.728
10968	252	39	32	C	S	1.945	-4.723	2.720	4.841	5.216	-2.480	-4.414	84.675	15.242	18.352
10969	252	39	32	C	S	19.107	-1.485	0.937	1.181	1.530	-0.916	-1.154	83.131	14.708	19.033
10970	252	39	32	C	S	27.796	5.011	-2.332	-4.863	-4.554	2.583	5.387	64.994	17.728	17.338
10971	252	39	32	C	S	32.204	5.011	2.332	-4.863	-4.554	-2.583	5.387	64.994	17.338	17.728
10972	252	39	32	C	S	40.893	-1.485	-0.937	1.181	1.530	0.916	-1.154	83.131	14.708	19.033
10973	252	39	32	C	S	42.304	1.029	1.516	-1.341	-1.008	-1.558	1.377	83.097	19.034	14.707
10974	252	39	32	C	S	49.842	2.281	-2.920	-2.506	-2.182	3.074	2.639	83.858	16.362	17.090
10975	252	39	32	C	S	53.822	-2.145	4.036	1.887	2.241	-3.889	-1.818	83.683	17.102	16.354
10976	252	39	32	C	S	58.055	-4.723	-2.720	4.841	5.216	2.480	-4.414	84.675	15.242	18.352
10977	252	39	32	C	S	60.000	5.011	2.332	-4.863	-4.554	-2.583	5.387	64.994	17.338	17.728
10978	252	39	32	C	S	61.945	-4.723	2.720	4.841	5.216	-2.480	-4.414	84.675	15.242	18.352
10979	252	39	32	C	S	66.178	-2.145	-4.035	1.887	2.241	3.889	-1.818	83.683	17.102	16.354
10980	252	39	32	C	S	70.158	2.281	2.920	-2.506	-2.182	-3.074	2.638	83.858	16.362	17.090
10981	252	39	32	C	S	77.696	1.029	-1.516	-1.341	-1.008	1.558	1.377	83.097	19.034	14.707
10982	252	39	32	C	S	79.107	-1.485	0.937	1.181	1.530	-0.916	-1.154	83.131	14.708	19.033
10983	252	39	32	C	S	87.796	5.011	-2.332	-4.863	-4.554	2.583	5.387	64.994	17.728	17.338
10984	252	39	32	C	S	92.204	5.011	2.332	-4.863	-4.554	-2.583	5.387	64.994	17.338	17.728
10985	252	39	32	C	S	100.893	-1.485	-0.937	1.181	1.530	0.916	-1.154	83.131	14.708	19.033
10986	252	39	32	C	S	113.822	-2.145	4.036	1.887	2.241	-3.889	-1.818	83.683	17.102	16.354
10987	252	39	32	C	S	118.055	-4.723	-2.720	4.841	5.216	2.480	-4.414	84.675	15.242	18.352
10988	252	39	32	C	W	0.000	5.011	2.332	-4.863	-4.554	-2.583	5.387	64.994	17.338	17.728
10989	252	39	32	C	W	1.945	-4.723	2.720	4.841	5.216	-2.480	-4.414	84.675	15.242	18.352
10990	252	39	32	C	W	6.178	-2.145	-4.035	1.887	2.241	3.889	-1.818	83.683	17.102	16.354
10991	252	39	32	C	W	10.158	2.281	2.920	-2.506	-2.182	-3.074	2.638	83.858	16.362	17.090
10992	252	39	32	C	W	17.696	1.029	-1.516	-1.341	-1.008	1.558	1.377	83.097	19.034	14.707
10993	252	39	32	C	W	27.796	5.011	-2.332	-4.863	-4.554	2.583	5.387	64.994	17.728	17.338
10994	252	39	32	C	W	32.204	5.011	2.332	-4.863	-4.554	-2.583	5.387	64.994	17.338	17.728
10995	252	39	32	C	W	58.055	-4.723	-2.720	4.841	5.216	2.480	-4.414	84.675	15.242	18.352
10996	252	39	32	C	W	60.000	5.011	2.332	-4.863	-4.554	-2.583	5.387	64.994	17.338	17.728
10997	252	39	32	C	W	61.945	-4.723	2.720	4.841	5.216	-2.480	-4.414	84.675	15.242	18.352
10998	252	39	32	C	W	87.796	5.011	-2.332	-4.863	-4.554	2.583	5.387	64.994	17.728	17.338
10999	252	39	32	C	W	92.204	5.011	2.332	-4.863	-4.554	-2.583	5.387	64.994	17.338	17.728
11000	252	39	32	C	W	102.304	1.029	1.516	-1.341	-1.008	-1.558	1.377	83.097	19.034	14.707
11001	252	39	32	C	W	109.842	2.281	-2.920	-2.506	-2.182	3.074	2.639	83.858	16.362	17.090
11002	252	39	32	C	W	118.055	-4.723	-2.720	4.841	5.216	2.480	-4.414	84.675	15.242	18.352
11003	252	39	32	P	S	0.000	5.011	2.332	-4.863	-4.554	-2.583	5.387	64.994	17.338	17.728
11004	252	39	32	P	S	1.945	-4.723	2.720	4.841	5.216	-2.480	-4.414	84.675	15.242	18.352
11005	252	39	32	P	S	19.107	-1.485	0.937	1.181	1.530	-0.916	-1.154	83.131	14.708	19.033
11006	252	39	32	P	S	27.796	5.011	-2.332	-4.863	-4.554	2.583	5.387	64.994	17.728	17.338
11007	252	39	32	P	S	40.893	-1.485	-0.937	1.181	1.530	0.916	-1.154	83.131	14.708	19.033
11008	252	39	32	P	S	42.304	1.029	1.516	-1.341	-1.008	-1.558	1.377	83.097	19.034	14.707
11009	252	39	32	P	S	49.842	2.281	-2.920	-2.506	-2.182	3.074	2.639	83.858	16.362	17.090
11010	252	39	32	P	S	53.822	-2.145	4.036	1.887	2.241	-3.889	-1.818	83.683	17.102	16.354
11011	252	39	32	P	S	58.055	-4.723	-2.720	4.841	5.216	2.480	-4.414	84.675	15.242	18.352
11012	252	39	32	P	S	60.000	5.011	0.777	-4.863	-4.554	-0.861	5.387	84.865	18.353	15.239
11013	252	39	32	P	S	61.945	-4.723	2.720	4.841	5.216	-2.480	-4.414	84.675	15.242	18.352
11014	252	39	32	P	S	66.178	-2.145	-4.035	1.887	2.241	3.889	-1.818	83.683	17.102	16.354
11015	252	39	32	P	S	70.158	2.281	2.920	-2.506	-2.182	-3.074	2.638	83.858	16.362	17.090
11016	252	39	32	P	S	77.696	1.029	-1.516	-1.341	-1.008	1.558	1.377	83.097	19.034	14.707

BL number	Atoms	γ-PC unit cells	WS_2 unit cells	γ-PC origin atom	WS_2 origin atom	Twist-angle (°)	γ-PC strain 1 (%)	γ-PC strain 2 (%)	γ-PC strain 3 (%)	WS_2 strain 1 (%)	WS_2 strain 2 (%)	WS_2 strain 3 (%)	γ (°)	$\lvert\mathbf{a}\rvert$(Å)	$\lvert\mathbf{b}\rvert$(Å)
11017	252	39	32	P	S	79.107	-1.485	0.937	1.181	1.530	-0.916	-1.154	83.131	14.708	19.033
11018	252	39	32	P	S	92.204	5.011	2.332	-4.863	-4.554	-2.583	5.387	64.994	17.338	17.728
11019	252	39	32	P	S	100.893	-1.485	-0.937	1.181	1.530	0.916	-1.154	83.131	14.708	19.033
11020	252	39	32	P	S	113.822	-2.145	4.036	1.887	2.241	-3.889	-1.818	83.683	17.102	16.354
11021	252	39	32	P	S	118.055	-4.723	-2.720	4.841	5.216	2.480	-4.414	84.675	15.242	18.352
11022	252	39	32	P	W	0.000	5.011	-0.777	-4.863	-4.554	0.861	5.387	84.865	18.353	15.239
11023	252	39	32	P	W	1.945	-4.723	2.720	4.841	5.216	-2.480	-4.414	84.675	15.242	18.352
11024	252	39	32	P	W	6.178	-2.145	-4.035	1.887	2.241	3.889	-1.818	83.683	17.102	16.354
11025	252	39	32	P	W	10.158	2.281	2.920	-2.506	-2.182	-3.074	2.638	83.858	16.362	17.090
11026	252	39	32	P	W	17.696	1.029	-1.516	-1.341	-1.008	1.558	1.377	83.097	19.034	14.707
11027	252	39	32	P	W	27.796	5.011	-2.332	-4.863	-4.554	2.583	5.387	64.994	17.728	17.338
11028	252	39	32	P	W	32.204	5.011	2.332	-4.863	-4.554	-2.583	5.387	64.994	17.338	17.728
11029	252	39	32	P	W	58.055	-4.723	-2.720	4.841	5.216	2.480	-4.414	84.675	15.242	18.352
11030	252	39	32	P	W	60.000	5.011	2.332	-4.863	-4.554	-2.583	5.387	64.994	17.338	17.728
11031	252	39	32	P	W	61.945	-4.723	2.720	4.841	5.216	-2.480	-4.414	84.675	15.242	18.352
11032	252	39	32	P	W	87.796	5.011	-2.332	-4.863	-4.554	2.583	5.387	64.994	17.728	17.338
11033	252	39	32	P	W	102.304	1.029	1.516	-1.341	-1.008	-1.558	1.377	83.097	19.034	14.707
11034	252	39	32	P	W	109.842	2.281	-2.920	-2.506	-2.182	3.074	2.639	83.858	16.362	17.090
11035	252	39	32	P	W	118.055	-4.723	-2.720	4.841	5.216	2.480	-4.414	84.675	15.242	18.352
11036	253	40	31	C	S	4.715	-1.702	-3.221	-1.441	1.762	3.317	1.484	72.630	19.567	14.835
11037	253	40	31	C	S	8.213	-2.359	2.819	-0.771	2.476	-2.863	0.783	72.648	14.845	19.554
11038	253	40	31	C	S	51.787	-2.359	-2.819	-0.771	2.476	2.863	0.783	72.648	14.845	19.554
11039	253	40	31	C	S	55.285	-1.702	3.221	-1.441	1.762	-3.317	1.484	72.630	19.567	14.835
11040	253	40	31	C	S	64.715	-1.702	-3.221	-1.441	1.762	3.317	1.484	72.630	19.567	14.835
11041	253	40	31	C	S	68.213	-2.359	2.819	-0.771	2.476	-2.863	0.783	72.648	14.845	19.554
11042	253	40	31	C	S	111.787	-2.359	-2.819	-0.771	2.476	2.863	0.783	72.648	14.845	19.554
11043	253	40	31	C	S	115.285	-1.702	3.221	-1.441	1.762	-3.317	1.484	72.630	19.567	14.835
11044	253	40	31	C	W	4.715	-1.702	-3.221	-1.441	1.762	3.317	1.484	72.630	19.567	14.835
11045	253	40	31	C	W	8.213	-2.359	2.819	-0.771	2.476	-2.863	0.783	72.648	14.845	19.554
11046	253	40	31	C	W	51.787	-2.359	-2.819	-0.771	2.476	2.863	0.783	72.648	14.845	19.554
11047	253	40	31	C	W	55.285	-1.702	3.221	-1.441	1.762	-3.317	1.484	72.630	19.567	14.835
11048	253	40	31	C	W	64.715	-1.702	-3.221	-1.441	1.762	3.317	1.484	72.630	19.567	14.835
11049	253	40	31	C	W	68.213	-2.359	2.819	-0.771	2.476	-2.863	0.783	72.648	14.845	19.554
11050	253	40	31	C	W	111.787	-2.359	-2.819	-0.771	2.476	2.863	0.783	72.648	14.845	19.554
11051	253	40	31	C	W	115.285	-1.702	3.221	-1.441	1.762	-3.317	1.484	72.630	19.567	14.835
11052	253	40	31	P	S	4.715	-1.702	-3.221	-1.441	1.762	3.317	1.484	72.630	19.567	14.835
11053	253	40	31	P	S	8.213	-2.359	2.819	-0.771	2.476	-2.863	0.783	72.648	14.845	19.554
11054	253	40	31	P	S	51.787	-2.359	-2.819	-0.771	2.476	2.863	0.783	72.648	14.845	19.554
11055	253	40	31	P	S	55.285	-1.702	3.221	-1.441	1.762	-3.317	1.484	72.630	19.567	14.835
11056	253	40	31	P	S	64.715	-1.702	-3.221	-1.441	1.762	3.317	1.484	72.630	19.567	14.835
11057	253	40	31	P	S	68.213	-2.359	2.819	-0.771	2.476	-2.863	0.783	72.648	14.845	19.554
11058	253	40	31	P	S	111.787	-2.359	-2.819	-0.771	2.476	2.863	0.783	72.648	14.845	19.554
11059	253	40	31	P	S	115.285	-1.702	3.221	-1.441	1.762	-3.317	1.484	72.630	19.567	14.835
11060	253	40	31	P	W	4.715	-1.702	-3.221	-1.441	1.762	3.317	1.484	72.630	19.567	14.835
11061	253	40	31	P	W	8.213	-2.359	2.819	-0.771	2.476	-2.863	0.783	72.648	14.845	19.554
11062	253	40	31	P	W	51.787	-2.359	-2.819	-0.771	2.476	2.863	0.783	72.648	14.845	19.554
11063	253	40	31	P	W	55.285	-1.702	3.221	-1.441	1.762	-3.317	1.484	72.630	19.567	14.835
11064	253	40	31	P	W	64.715	-1.702	-3.221	-1.441	1.762	3.317	1.484	72.630	19.567	14.835
11065	253	40	31	P	W	68.213	-2.359	2.819	-0.771	2.476	-2.863	0.783	72.648	14.845	19.554
11066	253	40	31	P	W	111.787	-2.359	-2.819	-0.771	2.476	2.863	0.783	72.648	14.845	19.554
11067	253	40	31	P	W	115.285	-1.702	3.221	-1.441	1.762	-3.317	1.484	72.630	19.567	14.835

BL number	Atoms	γ-PC unit cells	WS_2 unit cells	γ-PC origin atom	WS_2 origin atom	Twist-angle (°)	γ-PC strain 1 (%)	γ-PC strain 2 (%)	γ-PC strain 3 (%)	WS_2 strain 1 (%)	WS_2 strain 2 (%)	WS_2 strain 3 (%)	γ (°)	|**a**|(Å)	|**b**|(Å)
11068	254	41	30	C	S	3.004	-3.388	-1.312	-2.495	3.635	1.381	2.626	70.036	17.335	16.947
11069	254	41	30	C	S	4.715	-3.007	1.593	-2.880	3.200	-1.691	3.056	70.034	16.951	17.330
11070	254	41	30	C	S	13.898	-4.228	-0.674	-1.623	4.619	0.697	1.678	70.035	17.184	17.098
11071	254	41	30	C	S	15.608	-2.145	2.215	-3.729	2.241	-2.393	4.030	70.026	17.102	17.180
11072	254	41	30	C	S	25.050	-0.955	1.282	-4.852	0.973	-1.420	5.373	69.078	17.772	16.640
11073	254	41	30	C	S	25.285	-4.781	1.743	-1.031	5.287	-1.780	1.053	69.023	16.641	17.777
11074	254	41	30	C	S	34.715	-4.781	-1.743	-1.031	5.287	1.780	1.053	69.023	16.641	17.777
11075	254	41	30	C	S	34.950	-0.955	-1.282	-4.852	0.973	1.420	5.373	69.078	17.772	16.640
11076	254	41	30	C	S	44.392	-2.145	-2.215	-3.729	2.241	2.393	4.030	70.026	17.102	17.180
11077	254	41	30	C	S	46.102	-4.228	0.674	-1.623	4.619	-0.697	1.678	70.035	17.184	17.098
11078	254	41	30	C	S	55.285	-3.007	-1.593	-2.880	3.200	1.691	3.056	70.034	16.951	17.330
11079	254	41	30	C	S	56.996	-3.388	1.312	-2.495	3.635	-1.381	2.626	70.036	17.335	16.947
11080	254	41	30	C	S	63.005	-3.388	-1.312	-2.495	3.635	1.381	2.626	70.036	17.335	16.947
11081	254	41	30	C	S	64.715	-3.007	1.593	-2.880	3.200	-1.691	3.056	70.034	16.951	17.330
11082	254	41	30	C	S	73.898	-4.228	-0.674	-1.623	4.619	0.697	1.678	70.035	17.184	17.098
11083	254	41	30	C	S	75.609	-2.145	2.215	-3.729	2.241	-2.393	4.030	70.026	17.102	17.180
11084	254	41	30	C	S	85.050	-0.955	1.282	-4.852	0.973	-1.420	5.373	69.078	17.772	16.640
11085	254	41	30	C	S	85.285	-4.781	1.743	-1.031	5.287	-1.780	1.053	69.023	16.641	17.777
11086	254	41	30	C	S	94.715	-4.781	-1.743	-1.031	5.287	1.780	1.053	69.023	16.641	17.777
11087	254	41	30	C	S	94.950	-0.955	-1.282	-4.852	0.973	1.420	5.373	69.078	17.772	16.640
11088	254	41	30	C	S	104.392	-2.145	-2.215	-3.729	2.241	2.393	4.030	70.026	17.102	17.180
11089	254	41	30	C	S	106.102	-4.228	0.674	-1.623	4.619	-0.697	1.678	70.035	17.184	17.098
11090	254	41	30	C	S	115.285	-3.007	-1.593	-2.880	3.200	1.691	3.056	70.034	16.951	17.330
11091	254	41	30	C	S	116.996	-3.388	1.312	-2.495	3.635	-1.381	2.626	70.035	17.335	16.947
11092	254	41	30	C	W	3.004	-3.388	-1.312	-2.495	3.635	1.381	2.626	70.036	17.335	16.947
11093	254	41	30	C	W	4.715	-3.007	1.593	-2.880	3.200	-1.691	3.056	70.034	16.951	17.330
11094	254	41	30	C	W	13.898	-4.228	-0.674	-1.623	4.619	0.697	1.678	70.035	17.184	17.098
11095	254	41	30	C	W	15.608	-2.145	2.215	-3.729	2.241	-2.393	4.030	70.026	17.102	17.180
11096	254	41	30	C	W	25.050	-0.955	1.282	-4.852	0.973	-1.420	5.373	69.078	17.772	16.640
11097	254	41	30	C	W	25.285	-4.781	1.743	-1.031	5.287	-1.780	1.053	69.023	16.641	17.777
11098	254	41	30	C	W	34.715	-4.781	-1.743	-1.031	5.287	1.780	1.053	69.023	16.641	17.777
11099	254	41	30	C	W	34.950	-0.955	-1.282	-4.852	0.973	1.420	5.373	69.078	17.772	16.640
11100	254	41	30	C	W	44.392	-2.145	-2.215	-3.729	2.241	2.393	4.030	70.026	17.102	17.180
11101	254	41	30	C	W	46.102	-4.228	0.674	-1.623	4.619	-0.697	1.678	70.035	17.184	17.098
11102	254	41	30	C	W	55.285	-3.007	-1.593	-2.880	3.200	1.691	3.056	70.034	16.951	17.330
11103	254	41	30	C	W	56.996	-3.388	1.312	-2.495	3.635	-1.381	2.626	70.036	17.335	16.947
11104	254	41	30	C	W	63.005	-3.388	-1.312	-2.495	3.635	1.381	2.626	70.036	17.335	16.947
11105	254	41	30	C	W	64.715	-3.007	1.593	-2.880	3.200	-1.691	3.056	70.034	16.951	17.330
11106	254	41	30	C	W	73.898	-4.228	-0.674	-1.623	4.619	0.697	1.678	70.035	17.184	17.098
11107	254	41	30	C	W	75.609	-2.145	2.215	-3.729	2.241	-2.393	4.030	70.026	17.102	17.180
11108	254	41	30	C	W	85.050	-0.955	1.282	-4.852	0.973	-1.420	5.373	69.078	17.772	16.640
11109	254	41	30	C	W	85.285	-4.781	1.743	-1.031	5.287	-1.780	1.053	69.023	16.641	17.777
11110	254	41	30	C	W	94.715	-4.781	-1.743	-1.031	5.287	1.780	1.053	69.023	16.641	17.777
11111	254	41	30	C	W	94.950	-0.955	-1.282	-4.852	0.973	1.420	5.373	69.078	17.772	16.640
11112	254	41	30	C	W	104.392	-2.145	-2.215	-3.729	2.241	2.393	4.030	70.026	17.102	17.180
11113	254	41	30	C	W	106.102	-4.228	0.674	-1.623	4.619	-0.697	1.678	70.035	17.184	17.098
11114	254	41	30	C	W	115.285	-3.007	-1.593	-2.880	3.200	1.691	3.056	70.034	16.951	17.330
11115	254	41	30	C	W	116.996	-3.388	1.312	-2.495	3.635	-1.381	2.626	70.035	17.335	16.947
11116	254	41	30	P	S	3.004	-3.388	-1.312	-2.495	3.635	1.381	2.626	70.036	17.335	16.947
11117	254	41	30	P	S	4.715	-3.007	1.593	-2.880	3.200	-1.691	3.056	70.034	16.951	17.330
11118	254	41	30	P	S	25.050	-0.955	1.282	-4.852	0.973	-1.420	5.373	69.078	17.772	16.640

BL number	Atoms	γ-PC unit cells	WS_2 unit cells	γ-PC origin atom	WS_2 origin atom	Twist-angle (°)	γ-PC strain 1 (%)	γ-PC strain 2 (%)	γ-PC strain 3 (%)	WS_2 strain 1 (%)	WS_2 strain 2 (%)	WS_2 strain 3 (%)	γ (°)	|**a**|(Å)	|**b**|(Å)
11119	254	41	30	P	S	25.285	-4.781	1.743	-1.031	5.287	-1.780	1.053	69.023	16.641	17.777
11120	254	41	30	P	S	34.715	-4.781	-1.743	-1.031	5.287	1.780	1.053	69.023	16.641	17.777
11121	254	41	30	P	S	34.950	-0.955	-1.282	-4.852	0.973	1.420	5.373	69.078	17.772	16.640
11122	254	41	30	P	S	46.102	-4.228	0.674	-1.623	4.619	-0.697	1.678	70.035	17.184	17.098
11123	254	41	30	P	S	55.285	-3.007	-1.593	-2.880	3.200	1.691	3.056	70.034	16.951	17.330
11124	254	41	30	P	S	56.996	-3.388	1.312	-2.495	3.635	-1.381	2.626	70.036	17.335	16.947
11125	254	41	30	P	S	63.005	-3.388	-1.312	-2.495	3.635	1.381	2.626	70.036	17.335	16.947
11126	254	41	30	P	S	64.715	-3.007	1.593	-2.880	3.200	-1.691	3.056	70.034	16.951	17.330
11127	254	41	30	P	S	73.898	-4.228	-0.674	-1.623	4.619	0.697	1.678	70.035	17.184	17.098
11128	254	41	30	P	S	85.050	-0.955	1.282	-4.852	0.973	-1.420	5.373	69.078	17.772	16.640
11129	254	41	30	P	S	85.285	-4.781	1.743	-1.031	5.287	-1.780	1.053	69.023	16.641	17.777
11130	254	41	30	P	S	94.715	-4.781	-1.743	-1.031	5.287	1.780	1.053	69.023	16.641	17.777
11131	254	41	30	P	S	94.950	-0.955	-1.282	-4.852	0.973	1.420	5.373	69.078	17.772	16.640
11132	254	41	30	P	S	115.285	-3.007	-1.593	-2.880	3.200	1.691	3.056	70.034	16.951	17.330
11133	254	41	30	P	S	116.996	-3.388	1.312	-2.495	3.635	-1.381	2.626	70.035	17.335	16.947
11134	254	41	30	P	W	3.004	-3.388	-1.312	-2.495	3.635	1.381	2.626	70.036	17.335	16.947
11135	254	41	30	P	W	4.715	-3.007	1.593	-2.880	3.200	-1.691	3.056	70.034	16.951	17.330
11136	254	41	30	P	W	25.050	-0.955	1.282	-4.852	0.973	-1.420	5.373	69.078	17.772	16.640
11137	254	41	30	P	W	25.285	-4.781	1.743	-1.031	5.287	-1.780	1.053	69.023	16.641	17.777
11138	254	41	30	P	W	34.715	-4.781	-1.743	-1.031	5.287	1.780	1.053	69.023	16.641	17.777
11139	254	41	30	P	W	34.950	-0.955	-1.282	-4.852	0.973	1.420	5.373	69.078	17.772	16.640
11140	254	41	30	P	W	55.285	-3.007	-1.593	-2.880	3.200	1.691	3.056	70.034	16.951	17.330
11141	254	41	30	P	W	56.996	-3.388	1.312	-2.495	3.635	-1.381	2.626	70.036	17.335	16.947
11142	254	41	30	P	W	63.005	-3.388	-1.312	-2.495	3.635	1.381	2.626	70.036	17.335	16.947
11143	254	41	30	P	W	64.715	-3.007	1.593	-2.880	3.200	-1.691	3.056	70.034	16.951	17.330
11144	254	41	30	P	W	85.050	-0.955	1.282	-4.852	0.973	-1.420	5.373	69.078	17.772	16.640
11145	254	41	30	P	W	85.285	-4.781	1.743	-1.031	5.287	-1.780	1.053	69.023	16.641	17.777
11146	254	41	30	P	W	94.715	-4.781	-1.743	-1.031	5.287	1.780	1.053	69.023	16.641	17.777
11147	254	41	30	P	W	94.950	-0.955	-1.282	-4.852	0.973	1.420	5.373	69.078	17.772	16.640
11148	254	41	30	P	W	115.285	-3.007	-1.593	-2.880	3.200	1.691	3.056	70.034	16.951	17.330
11149	254	41	30	P	W	116.996	-3.388	1.312	-2.495	3.635	-1.381	2.626	70.035	17.335	16.947
11150	255	39	33	C	S	0.000	5.011	-3.109	-3.452	-4.554	3.340	3.708	83.569	16.799	16.934
11151	255	39	33	C	S	4.715	-3.007	5.265	4.490	3.200	-4.831	-4.120	83.208	16.951	16.788
11152	255	39	33	C	S	16.337	0.353	-4.265	0.853	-0.351	4.194	-0.839	83.386	17.539	16.197
11153	255	39	33	C	S	19.107	-1.485	-3.750	2.780	1.530	3.553	-2.634	84.788	14.708	19.275
11154	255	39	33	C	S	21.052	1.339	4.000	-0.123	-1.304	-4.010	0.124	83.431	16.211	17.523
11155	255	39	33	C	S	23.691	2.390	4.314	-1.124	-2.281	-4.413	1.149	84.611	19.291	14.697
11156	255	39	33	C	S	36.309	2.390	-4.314	-1.123	-2.281	4.413	1.149	84.611	19.291	14.697
11157	255	39	33	C	S	38.948	1.339	-4.000	-0.123	-1.304	4.010	0.124	83.431	16.211	17.523
11158	255	39	33	C	S	40.893	-1.485	3.750	2.780	1.530	-3.553	-2.634	84.788	14.708	19.275
11159	255	39	33	C	S	43.663	0.353	4.265	0.853	-0.351	-4.194	-0.839	83.387	17.539	16.197
11160	255	39	33	C	S	55.285	-3.007	-5.265	4.490	3.200	4.831	-4.120	83.208	16.951	16.788
11161	255	39	33	C	S	60.000	5.011	3.109	-3.452	-4.554	-3.340	3.708	83.569	16.799	16.934
11162	255	39	33	C	S	64.715	-3.007	5.265	4.490	3.200	-4.831	-4.120	83.208	16.951	16.788
11163	255	39	33	C	S	76.337	0.353	-4.265	0.853	-0.351	4.194	-0.839	83.387	17.539	16.197
11164	255	39	33	C	S	79.107	-1.485	-3.750	2.780	1.530	3.553	-2.634	84.788	14.708	19.275
11165	255	39	33	C	S	81.052	1.339	4.000	-0.123	-1.304	-4.010	0.124	83.431	16.211	17.523
11166	255	39	33	C	S	96.309	2.390	-4.314	-1.123	-2.281	4.413	1.149	84.611	19.291	14.697
11167	255	39	33	C	S	98.948	1.339	-4.000	-0.123	-1.304	4.010	0.124	83.431	16.211	17.523
11168	255	39	33	C	S	100.893	-1.485	3.750	2.780	1.530	-3.553	-2.634	84.788	14.708	19.275
11169	255	39	33	C	S	103.663	0.353	4.265	0.853	-0.351	-4.194	-0.839	83.386	17.539	16.197

BL number	Atoms	γ-PC unit cells	WS_2 unit cells	γ-PC origin atom	WS_2 origin atom	Twist-angle (°)	γ-PC strain 1 (%)	γ-PC strain 2 (%)	γ-PC strain 3 (%)	WS_2 strain 1 (%)	WS_2 strain 2 (%)	WS_2 strain 3 (%)	γ (°)	|**a**|(Å)	|**b**|(Å)
11170	255	39	33	C	S	115.285	-3.007	-5.265	4.490	3.200	4.831	-4.120	83.208	16.951	16.788
11171	255	39	33	C	W	0.000	5.011	-3.109	-3.452	-4.554	3.340	3.708	83.569	16.799	16.934
11172	255	39	33	C	W	4.715	-3.007	5.265	4.490	3.200	-4.831	-4.120	83.208	16.951	16.788
11173	255	39	33	C	W	16.337	0.353	-4.265	0.853	-0.351	4.194	-0.839	83.386	17.539	16.197
11174	255	39	33	C	W	21.052	1.339	4.000	-0.123	-1.304	-4.010	0.124	83.431	16.211	17.523
11175	255	39	33	C	W	38.948	1.339	-4.000	-0.123	-1.304	4.010	0.124	83.431	16.211	17.523
11176	255	39	33	C	W	43.663	0.353	4.265	0.853	-0.351	-4.194	-0.839	83.387	17.539	16.197
11177	255	39	33	C	W	55.285	-3.007	-5.265	4.490	3.200	4.831	-4.120	83.208	16.951	16.788
11178	255	39	33	C	W	60.000	5.011	3.109	-3.452	-4.554	-3.340	3.708	83.569	16.799	16.934
11179	255	39	33	C	W	64.715	-3.007	5.265	4.490	3.200	-4.831	-4.120	83.208	16.951	16.788
11180	255	39	33	C	W	76.337	0.353	-4.265	0.853	-0.351	4.194	-0.839	83.387	17.539	16.197
11181	255	39	33	C	W	81.052	1.339	4.000	-0.123	-1.304	-4.010	0.124	83.431	16.211	17.523
11182	255	39	33	C	W	83.691	2.390	4.314	-1.124	-2.281	-4.413	1.149	84.611	19.291	14.697
11183	255	39	33	C	W	98.948	1.339	-4.000	-0.123	-1.304	4.010	0.124	83.431	16.211	17.523
11184	255	39	33	C	W	103.663	0.353	4.265	0.853	-0.351	-4.194	-0.839	83.386	17.539	16.197
11185	255	39	33	C	W	115.285	-3.007	-5.265	4.490	3.200	4.831	-4.120	83.208	16.951	16.788
11186	255	39	33	P	S	0.000	5.011	-3.109	-3.452	-4.554	3.340	3.708	83.569	16.799	16.934
11187	255	39	33	P	S	4.715	-3.007	5.265	4.490	3.200	-4.831	-4.120	83.208	16.951	16.788
11188	255	39	33	P	S	16.337	0.353	-4.265	0.853	-0.351	4.194	-0.839	83.386	17.539	16.197
11189	255	39	33	P	S	19.107	-1.485	-3.750	2.780	1.530	3.553	-2.634	84.788	14.708	19.275
11190	255	39	33	P	S	21.052	1.339	4.000	-0.123	-1.304	-4.010	0.124	83.431	16.211	17.523
11191	255	39	33	P	S	23.691	2.390	4.314	-1.124	-2.281	-4.413	1.149	84.611	19.291	14.697
11192	255	39	33	P	S	36.309	2.390	-4.314	-1.123	-2.281	4.413	1.149	84.611	19.291	14.697
11193	255	39	33	P	S	38.948	1.339	-4.000	-0.123	-1.304	4.010	0.124	83.431	16.211	17.523
11194	255	39	33	P	S	40.893	-1.485	3.750	2.780	1.530	-3.553	-2.634	84.788	14.708	19.275
11195	255	39	33	P	S	43.663	0.353	4.265	0.853	-0.351	-4.194	-0.839	83.387	17.539	16.197
11196	255	39	33	P	S	55.285	-3.007	-5.265	4.490	3.200	4.831	-4.120	83.208	16.951	16.788
11197	255	39	33	P	S	60.000	5.011	3.109	-3.452	-4.554	-3.340	3.708	83.569	16.799	16.934
11198	255	39	33	P	S	64.715	-3.007	5.265	4.490	3.200	-4.831	-4.120	83.208	16.951	16.788
11199	255	39	33	P	S	76.337	0.353	-4.265	0.853	-0.351	4.194	-0.839	83.387	17.539	16.197
11200	255	39	33	P	S	79.107	-1.485	-3.750	2.780	1.530	3.553	-2.634	84.788	14.708	19.275
11201	255	39	33	P	S	81.052	1.339	4.000	-0.123	-1.304	-4.010	0.124	83.431	16.211	17.523
11202	255	39	33	P	S	96.309	2.390	-4.314	-1.123	-2.281	4.413	1.149	84.611	19.291	14.697
11203	255	39	33	P	S	98.948	1.339	-4.000	-0.123	-1.304	4.010	0.124	83.431	16.211	17.523
11204	255	39	33	P	S	100.893	-1.485	3.750	2.780	1.530	-3.553	-2.634	84.788	14.708	19.275
11205	255	39	33	P	S	103.663	0.353	4.265	0.853	-0.351	-4.194	-0.839	83.386	17.539	16.197
11206	255	39	33	P	S	115.285	-3.007	-5.265	4.490	3.200	4.831	-4.120	83.208	16.951	16.788
11207	255	39	33	P	W	0.000	5.011	-3.109	-3.452	-4.554	3.340	3.708	83.569	16.799	16.934
11208	255	39	33	P	W	4.715	-3.007	5.265	4.490	3.200	-4.831	-4.120	83.208	16.951	16.788
11209	255	39	33	P	W	16.337	0.353	-4.265	0.853	-0.351	4.194	-0.839	83.386	17.539	16.197
11210	255	39	33	P	W	21.052	1.339	4.000	-0.123	-1.304	-4.010	0.124	83.431	16.211	17.523
11211	255	39	33	P	W	38.948	1.339	-4.000	-0.123	-1.304	4.010	0.124	83.431	16.211	17.523
11212	255	39	33	P	W	43.663	0.353	4.265	0.853	-0.351	-4.194	-0.839	83.387	17.539	16.197
11213	255	39	33	P	W	55.285	-3.007	-5.265	4.490	3.200	4.831	-4.120	83.208	16.951	16.788
11214	255	39	33	P	W	60.000	5.011	3.109	-3.452	-4.554	-3.340	3.708	83.569	16.799	16.934
11215	255	39	33	P	W	64.715	-3.007	5.265	4.490	3.200	-4.831	-4.120	83.208	16.951	16.788
11216	255	39	33	P	W	76.337	0.353	-4.265	0.853	-0.351	4.194	-0.839	83.387	17.539	16.197
11217	255	39	33	P	W	81.052	1.339	4.000	-0.123	-1.304	-4.010	0.124	83.431	16.211	17.523
11218	255	39	33	P	W	83.691	2.390	4.314	-1.124	-2.281	-4.413	1.149	84.611	19.291	14.697
11219	255	39	33	P	W	98.948	1.339	-4.000	-0.123	-1.304	4.010	0.124	83.431	16.211	17.523
11220	255	39	33	P	W	103.663	0.353	4.265	0.853	-0.351	-4.194	-0.839	83.386	17.539	16.197

BL number	Atoms	γ-PC unit cells	WS_2 unit cells	γ-PC origin atom	WS_2 origin atom	Twist-angle (°)	γ-PC strain 1 (%)	γ-PC strain 2 (%)	γ-PC strain 3 (%)	WS_2 strain 1 (%)	WS_2 strain 2 (%)	WS_2 strain 3 (%)	γ (°)	|**a**|(Å)	|**b**|(Å)
11221	255	39	33	P	W	115.285	-3.007	-5.265	4.490	3.200	4.831	-4.120	83.208	16.951	16.788
11222	256	37	36	C	S	25.285	4.262	0.000	4.263	-3.928	0.000	-3.928	60.000	18.222	18.222
11223	256	37	36	C	S	34.715	4.262	0.000	4.263	-3.928	0.000	-3.928	60.000	18.222	18.222
11224	256	37	36	C	S	85.285	4.262	0.000	4.263	-3.928	0.000	-3.928	60.000	18.222	18.222
11225	256	37	36	C	S	94.715	4.262	0.000	4.263	-3.928	0.000	-3.928	60.000	18.222	18.222
11226	256	37	36	C	W	25.285	4.262	0.000	4.263	-3.928	0.000	-3.928	60.000	18.222	18.222
11227	256	37	36	C	W	34.715	4.262	0.000	4.263	-3.928	0.000	-3.928	60.000	18.222	18.222
11228	256	37	36	C	W	85.285	4.262	0.000	4.263	-3.928	0.000	-3.928	60.000	18.222	18.222
11229	256	37	36	C	W	94.715	4.262	0.000	4.263	-3.928	0.000	-3.928	60.000	18.222	18.222
11230	256	37	36	C	S	17.897	5.011	2.458	3.524	-4.554	-2.296	-3.292	68.020	16.799	18.460
11231	256	37	36	C	S	42.103	5.011	-2.458	3.524	-4.554	2.296	-3.292	68.020	16.799	18.460
11232	256	37	36	C	S	77.897	5.011	2.458	3.524	-4.554	-2.296	-3.292	68.020	16.799	18.460
11233	256	37	36	C	S	102.104	5.011	-2.458	3.524	-4.554	2.296	-3.292	68.020	16.799	18.460
11234	256	37	36	C	W	17.897	5.011	2.458	3.524	-4.554	-2.296	-3.292	68.020	16.799	18.460
11235	256	37	36	C	W	42.103	5.011	-2.458	3.524	-4.554	2.296	-3.292	68.020	16.799	18.460
11236	256	37	36	C	W	77.897	5.011	2.458	3.524	-4.554	-2.296	-3.292	68.020	16.799	18.460
11237	256	37	36	C	W	102.104	5.011	-2.458	3.524	-4.554	2.296	-3.292	68.020	16.799	18.460
11238	256	40	32	C	S	10.893	0.418	5.176	-1.982	-0.415	-5.390	2.064	86.977	14.426	19.539
11239	256	40	32	C	S	13.898	-0.414	-2.654	-1.177	0.417	2.718	1.205	69.817	17.536	17.099
11240	256	40	32	C	S	15.608	-2.145	0.303	0.590	2.241	-0.299	-0.583	69.792	17.102	17.539
11241	256	40	32	C	S	16.337	0.353	1.542	-1.921	-0.351	-1.604	1.997	69.783	17.539	17.102
11242	256	40	32	C	S	27.126	1.029	-2.168	-2.557	-1.008	2.285	2.695	85.306	19.034	14.840
11243	256	40	32	C	S	30.000	-2.359	-2.819	0.817	2.476	2.773	-0.804	85.206	14.845	19.030
11244	256	40	32	C	S	32.874	1.029	2.168	-2.557	-1.008	-2.285	2.695	85.306	19.034	14.840
11245	256	40	32	C	S	43.663	0.353	-1.542	-1.921	-0.351	1.604	1.997	69.783	17.539	17.102
11246	256	40	32	C	S	46.102	-0.414	2.654	-1.177	0.417	-2.718	1.205	69.817	17.099	17.536
11247	256	40	32	C	S	49.107	0.418	-5.176	-1.983	-0.415	5.390	2.064	86.977	14.426	19.539
11248	256	40	32	C	S	70.893	0.418	5.176	-1.982	-0.415	-5.390	2.064	86.977	14.426	19.538
11249	256	40	32	C	S	75.609	-2.145	0.303	0.590	2.241	-0.299	-0.583	69.792	17.102	17.539
11250	256	40	32	C	S	76.337	0.353	1.542	-1.921	-0.351	-1.604	1.997	69.783	17.539	17.102
11251	256	40	32	C	S	87.126	1.029	-2.168	-2.557	-1.008	2.285	2.695	85.306	19.034	14.840
11252	256	40	32	C	S	90.000	-2.359	2.819	0.817	2.476	-2.773	-0.804	85.206	14.845	19.030
11253	256	40	32	C	S	92.874	1.029	2.168	-2.557	-1.008	-2.285	2.695	85.306	19.034	14.840
11254	256	40	32	C	S	103.663	0.353	-1.542	-1.921	-0.351	1.604	1.997	69.783	17.539	17.102
11255	256	40	32	C	S	104.392	-2.145	-0.303	0.590	2.241	0.299	-0.583	69.792	17.102	17.539
11256	256	40	32	C	S	106.102	-0.414	2.654	-1.177	0.417	-2.718	1.205	69.817	17.099	17.536
11257	256	40	32	C	S	109.107	0.418	-5.176	-1.983	-0.415	5.390	2.064	86.977	14.426	19.538
11258	256	40	32	C	W	10.893	0.418	5.176	-1.982	-0.415	-5.390	2.064	86.977	14.426	19.539
11259	256	40	32	C	W	15.608	-2.145	0.303	0.590	2.241	-0.299	-0.583	69.792	17.102	17.539
11260	256	40	32	C	W	16.337	0.353	1.542	-1.921	-0.351	-1.604	1.997	69.783	17.539	17.102
11261	256	40	32	C	W	27.126	1.029	-2.168	-2.557	-1.008	2.285	2.695	85.306	19.034	14.840
11262	256	40	32	C	W	30.000	-2.359	-2.819	0.817	2.476	2.773	-0.804	85.206	14.845	19.030
11263	256	40	32	C	W	32.874	1.029	2.168	-2.557	-1.008	-2.285	2.695	85.306	19.034	14.840
11264	256	40	32	C	W	43.663	0.353	-1.542	-1.921	-0.351	1.604	1.997	69.783	17.539	17.102
11265	256	40	32	C	W	44.392	-2.145	-0.303	0.590	2.241	0.299	-0.583	69.792	17.102	17.539
11266	256	40	32	C	W	46.102	-0.414	2.654	-1.177	0.417	-2.718	1.205	69.817	17.099	17.536
11267	256	40	32	C	W	49.107	0.418	-5.176	-1.983	-0.415	5.390	2.064	86.977	14.426	19.539
11268	256	40	32	C	W	70.893	0.418	5.176	-1.982	-0.415	-5.390	2.064	86.977	14.426	19.538
11269	256	40	32	C	W	73.898	-0.414	-2.654	-1.177	0.417	2.718	1.205	69.817	17.536	17.099
11270	256	40	32	C	W	75.609	-2.145	0.303	0.590	2.241	-0.299	-0.583	69.792	17.102	17.539
11271	256	40	32	C	W	76.337	0.353	1.542	-1.921	-0.351	-1.604	1.997	69.783	17.539	17.102

BL number	Atoms	γ-PC unit cells	WS_2 unit cells	γ-PC origin atom	WS_2 origin atom	Twist-angle (°)	γ-PC strain 1 (%)	γ-PC strain 2 (%)	γ-PC strain 3 (%)	WS_2 strain 1 (%)	WS_2 strain 2 (%)	WS_2 strain 3 (%)	γ (°)	|**a**|(Å)	|**b**|(Å)
11272	256	40	32	C	W	87.126	1.029	-2.168	-2.557	-1.008	2.285	2.695	85.306	19.034	14.840
11273	256	40	32	C	W	90.000	-2.359	2.819	0.817	2.476	-2.773	-0.804	85.206	14.845	19.030
11274	256	40	32	C	W	92.874	1.029	2.168	-2.557	-1.008	-2.285	2.695	85.306	19.034	14.840
11275	256	40	32	C	W	103.663	0.353	-1.542	-1.921	-0.351	1.604	1.997	69.783	17.539	17.102
11276	256	40	32	C	W	106.102	-0.414	2.654	-1.177	0.417	-2.718	1.205	69.817	17.099	17.536
11277	256	40	32	C	W	109.107	0.418	-5.176	-1.983	-0.415	5.390	2.064	86.977	14.426	19.538
11278	256	40	32	P	S	10.893	0.418	5.176	-1.982	-0.415	-5.390	2.064	86.977	14.426	19.539
11279	256	40	32	P	S	27.126	1.029	-2.168	-2.557	-1.008	2.285	2.695	85.306	19.034	14.840
11280	256	40	32	P	S	30.000	-2.359	-2.819	0.817	2.476	2.773	-0.804	85.206	14.845	19.030
11281	256	40	32	P	S	32.874	1.029	2.168	-2.557	-1.008	-2.285	2.695	85.306	19.034	14.840
11282	256	40	32	P	S	43.663	0.353	-1.542	-1.921	-0.351	1.604	1.997	69.783	17.539	17.102
11283	256	40	32	P	S	44.392	-2.145	-0.303	0.590	2.241	0.299	-0.583	69.792	17.102	17.539
11284	256	40	32	P	S	46.102	-0.414	2.654	-1.177	0.417	-2.718	1.205	69.817	17.099	17.536
11285	256	40	32	P	S	49.107	0.418	-5.176	-1.983	-0.415	5.390	2.064	86.977	14.426	19.539
11286	256	40	32	P	S	70.893	0.418	5.176	-1.982	-0.415	-5.390	2.064	86.977	14.426	19.538
11287	256	40	32	P	S	73.898	-0.414	-2.654	-1.177	0.417	2.718	1.205	69.817	17.536	17.099
11288	256	40	32	P	S	75.609	-2.145	0.303	0.590	2.241	-0.299	-0.583	69.792	17.102	17.539
11289	256	40	32	P	S	76.337	0.353	1.542	-1.921	-0.351	-1.604	1.997	69.783	17.539	17.102
11290	256	40	32	P	S	87.126	1.029	-2.168	-2.557	-1.008	2.285	2.695	85.306	19.034	14.840
11291	256	40	32	P	S	90.000	-2.359	2.819	0.817	2.476	-2.773	-0.804	85.206	14.845	19.030
11292	256	40	32	P	S	92.874	1.029	2.168	-2.557	-1.008	-2.285	2.695	85.306	19.034	14.840
11293	256	40	32	P	S	103.663	0.353	-1.542	-1.921	-0.351	1.604	1.997	69.783	17.539	17.102
11294	256	40	32	P	S	109.107	0.418	-5.176	-1.983	-0.415	5.390	2.064	86.977	14.426	19.538
11295	256	40	32	P	W	10.893	0.418	5.176	-1.982	-0.415	-5.390	2.064	86.977	14.426	19.539
11296	256	40	32	P	W	13.898	-0.414	-2.654	-1.177	0.417	2.718	1.205	69.817	17.536	17.099
11297	256	40	32	P	W	15.608	-2.145	0.303	0.590	2.241	-0.299	-0.583	69.792	17.102	17.539
11298	256	40	32	P	W	16.337	0.353	1.542	-1.921	-0.351	-1.604	1.997	69.783	17.539	17.102
11299	256	40	32	P	W	27.126	1.029	-2.168	-2.557	-1.008	2.285	2.695	85.306	19.034	14.840
11300	256	40	32	P	W	30.000	-2.359	-2.819	0.817	2.476	2.773	-0.804	85.206	14.845	19.030
11301	256	40	32	P	W	32.874	1.029	2.168	-2.557	-1.008	-2.285	2.695	85.306	19.034	14.840
11302	256	40	32	P	W	43.663	0.353	-1.542	-1.921	-0.351	1.604	1.997	69.783	17.539	17.102
11303	256	40	32	P	W	49.107	0.418	-5.176	-1.983	-0.415	5.390	2.064	86.977	14.426	19.539
11304	256	40	32	P	W	70.893	0.418	5.176	-1.982	-0.415	-5.390	2.064	86.977	14.426	19.538
11305	256	40	32	P	W	87.126	1.029	-2.168	-2.557	-1.008	2.285	2.695	85.306	19.034	14.840
11306	256	40	32	P	W	90.000	-2.359	2.819	0.817	2.476	-2.773	-0.804	85.206	14.845	19.030
11307	256	40	32	P	W	92.874	1.029	2.168	-2.557	-1.008	-2.285	2.695	85.306	19.034	14.840
11308	256	40	32	P	W	103.663	0.353	-1.542	-1.921	-0.351	1.604	1.997	69.783	17.539	17.102
11309	256	40	32	P	W	104.392	-2.145	-0.303	0.590	2.241	0.299	-0.583	69.792	17.102	17.539
11310	256	40	32	P	W	106.102	-0.414	2.654	-1.177	0.417	-2.718	1.205	69.817	17.099	17.536
11311	256	40	32	P	W	109.107	0.418	-5.176	-1.983	-0.415	5.390	2.064	86.977	14.426	19.538
11312	257	38	35	C	S	16.337	0.353	-0.369	5.355	-0.351	0.333	-4.837	77.153	17.539	16.797
11313	257	38	35	C	S	17.897	5.011	2.393	0.668	-4.554	-2.362	-0.659	77.058	16.799	17.541
11314	257	38	35	C	S	18.613	3.582	-0.574	2.019	-3.342	0.552	-1.941	85.032	18.586	15.505
11315	257	38	35	C	S	19.107	1.980	-4.029	3.622	-1.905	3.756	-3.377	73.823	15.504	19.279
11316	257	38	35	C	S	23.691	2.390	4.248	3.203	-2.281	-3.992	-3.010	73.804	19.291	15.496
11317	257	38	35	C	S	36.309	2.390	-4.248	3.203	-2.281	3.992	-3.010	73.804	19.291	15.496
11318	257	38	35	C	S	40.893	1.980	-0.310	3.622	-1.905	0.289	-3.377	85.043	15.504	18.586
11319	257	38	35	C	S	42.103	5.011	-2.393	0.668	-4.554	2.362	-0.659	77.058	16.799	17.541
11320	257	38	35	C	S	43.663	0.353	0.369	5.355	-0.351	-0.333	-4.837	77.153	17.539	16.797
11321	257	38	35	C	S	76.337	0.353	-0.369	5.355	-0.351	0.333	-4.837	77.153	17.539	16.797
11322	257	38	35	C	S	77.897	5.011	2.393	0.668	-4.554	-2.362	-0.659	77.058	16.799	17.541

BL number	Atoms	γ-PC unit cells	WS_2 unit cells	γ-PC origin atom	WS_2 origin atom	Twist-angle (°)	γ-PC strain 1 (%)	γ-PC strain 2 (%)	γ-PC strain 3 (%)	WS_2 strain 1 (%)	WS_2 strain 2 (%)	WS_2 strain 3 (%)	γ (°)	**|a|**(Å)	**|b|**(Å)
11323	257	38	35	C	S	78.613	3.582	-0.574	2.019	-3.342	0.552	-1.941	85.032	18.586	15.505
11324	257	38	35	C	S	79.107	1.980	-4.029	3.622	-1.905	3.756	-3.377	73.823	15.504	19.279
11325	257	38	35	C	S	83.691	2.390	4.248	3.203	-2.281	-3.992	-3.010	73.804	19.291	15.496
11326	257	38	35	C	S	96.309	2.390	-4.248	3.203	-2.281	3.992	-3.010	73.804	19.291	15.496
11327	257	38	35	C	S	100.893	1.980	-0.310	3.622	-1.905	0.289	-3.377	85.043	15.504	18.586
11328	257	38	35	C	S	102.104	5.011	-2.393	0.668	-4.554	2.362	-0.659	77.058	16.799	17.541
11329	257	38	35	C	S	103.663	0.353	0.369	5.355	-0.351	-0.333	-4.837	77.153	17.539	16.797
11330	257	38	35	C	W	16.337	0.353	-0.369	5.355	-0.351	0.333	-4.837	77.153	17.539	16.797
11331	257	38	35	C	W	17.897	5.011	2.393	0.668	-4.554	-2.362	-0.659	77.058	16.799	17.541
11332	257	38	35	C	W	18.613	3.582	-0.574	2.019	-3.342	0.552	-1.941	85.032	18.586	15.505
11333	257	38	35	C	W	19.107	1.980	-4.029	3.622	-1.905	3.756	-3.377	73.823	15.504	19.279
11334	257	38	35	C	W	23.691	2.390	4.248	3.203	-2.281	-3.992	-3.010	73.804	19.291	15.496
11335	257	38	35	C	W	36.309	2.390	-4.248	3.203	-2.281	3.992	-3.010	73.804	19.291	15.496
11336	257	38	35	C	W	40.893	1.980	-0.310	3.622	-1.905	0.289	-3.377	85.043	15.504	18.586
11337	257	38	35	C	W	42.103	5.011	-2.393	0.668	-4.554	2.362	-0.659	77.058	16.799	17.541
11338	257	38	35	C	W	43.663	0.353	0.369	5.355	-0.351	-0.333	-4.837	77.153	17.539	16.797
11339	257	38	35	C	W	76.337	0.353	-0.369	5.355	-0.351	0.333	-4.837	77.153	17.539	16.797
11340	257	38	35	C	W	77.897	5.011	2.393	0.668	-4.554	-2.362	-0.659	77.058	16.799	17.541
11341	257	38	35	C	W	78.613	3.582	-0.574	2.019	-3.342	0.552	-1.941	85.032	18.586	15.505
11342	257	38	35	C	W	79.107	1.980	-4.029	3.622	-1.905	3.756	-3.377	73.823	15.504	19.279
11343	257	38	35	C	W	83.691	2.390	4.248	3.203	-2.281	-3.992	-3.010	73.804	19.291	15.496
11344	257	38	35	C	W	96.309	2.390	-4.248	3.203	-2.281	3.992	-3.010	73.804	19.291	15.496
11345	257	38	35	C	W	100.893	1.980	-0.310	3.622	-1.905	0.289	-3.377	85.043	15.504	18.586
11346	257	38	35	C	W	102.104	5.011	-2.393	0.668	-4.554	2.362	-0.659	77.058	16.799	17.541
11347	257	38	35	C	W	103.663	0.353	0.369	5.355	-0.351	-0.333	-4.837	77.153	17.539	16.797
11348	257	38	35	P	S	16.337	0.353	-0.369	5.355	-0.351	0.333	-4.837	77.153	17.539	16.797
11349	257	38	35	P	S	17.897	5.011	2.393	0.668	-4.554	-2.362	-0.659	77.058	16.799	17.541
11350	257	38	35	P	S	18.613	3.582	-0.574	2.019	-3.342	0.552	-1.941	85.032	18.586	15.505
11351	257	38	35	P	S	19.107	1.980	-4.029	3.622	-1.905	3.756	-3.377	73.823	15.504	19.279
11352	257	38	35	P	S	23.691	2.390	4.248	3.203	-2.281	-3.992	-3.010	73.804	19.291	15.496
11353	257	38	35	P	S	36.309	2.390	-4.248	3.203	-2.281	3.992	-3.010	73.804	19.291	15.496
11354	257	38	35	P	S	40.893	1.980	-0.310	3.622	-1.905	0.289	-3.377	85.043	15.504	18.586
11355	257	38	35	P	S	78.613	3.582	-0.574	2.019	-3.342	0.552	-1.941	85.032	18.586	15.505
11356	257	38	35	P	S	79.107	1.980	-4.029	3.622	-1.905	3.756	-3.377	73.823	15.504	19.279
11357	257	38	35	P	S	83.691	2.390	4.248	3.203	-2.281	-3.992	-3.010	73.804	19.291	15.496
11358	257	38	35	P	S	96.309	2.390	-4.248	3.203	-2.281	3.992	-3.010	73.804	19.291	15.496
11359	257	38	35	P	S	100.893	1.980	-0.310	3.622	-1.905	0.289	-3.377	85.043	15.504	18.586
11360	257	38	35	P	S	102.104	5.011	-2.393	0.668	-4.554	2.362	-0.659	77.058	16.799	17.541
11361	257	38	35	P	S	103.663	0.353	0.369	5.355	-0.351	-0.333	-4.837	77.153	17.539	16.797
11362	257	38	35	P	W	18.613	3.582	-0.574	2.019	-3.342	0.552	-1.941	85.032	18.586	15.505
11363	257	38	35	P	W	19.107	1.980	-4.029	3.622	-1.905	3.756	-3.377	73.823	15.504	19.279
11364	257	38	35	P	W	23.691	2.390	4.248	3.203	-2.281	-3.992	-3.010	73.804	19.291	15.496
11365	257	38	35	P	W	36.309	2.390	-4.248	3.203	-2.281	3.992	-3.010	73.804	19.291	15.496
11366	257	38	35	P	W	40.893	1.980	-0.310	3.622	-1.905	0.289	-3.377	85.043	15.504	18.586
11367	257	38	35	P	W	42.103	5.011	-2.393	0.668	-4.554	2.362	-0.659	77.058	16.799	17.541
11368	257	38	35	P	W	43.663	0.353	0.369	5.355	-0.351	-0.333	-4.837	77.153	17.539	16.797
11369	257	38	35	P	W	76.337	0.353	-0.369	5.355	-0.351	0.333	-4.837	77.153	17.539	16.797
11370	257	38	35	P	W	77.897	5.011	2.393	0.668	-4.554	-2.362	-0.659	77.058	16.799	17.541
11371	257	38	35	P	W	78.613	3.582	-0.574	2.019	-3.342	0.552	-1.941	85.032	18.586	15.505
11372	257	38	35	P	W	79.107	1.980	-4.029	3.622	-1.905	3.756	-3.377	73.823	15.504	19.279
11373	257	38	35	P	W	83.691	2.390	4.248	3.203	-2.281	-3.992	-3.010	73.804	19.291	15.496

BL number	Atoms	γ-PC unit cells	WS_2 unit cells	γ-PC origin atom	WS_2 origin atom	Twist-angle (°)	γ-PC strain 1 (%)	γ-PC strain 2 (%)	γ-PC strain 3 (%)	WS_2 strain 1 (%)	WS_2 strain 2 (%)	WS_2 strain 3 (%)	γ (°)	$\lvert\mathbf{a}\rvert$(Å)	$\lvert\mathbf{b}\rvert$(Å)
11374	257	38	35	P	W	96.309	2.390	-4.248	3.203	-2.281	3.992	-3.010	73.804	19.291	15.496
11375	257	38	35	P	W	100.893	1.980	-0.310	3.622	-1.905	0.289	-3.377	85.043	15.504	18.586
11376	257	41	31	C	S	19.842	-4.723	3.450	0.536	5.216	-3.413	-0.530	75.362	15.242	19.039
11377	257	41	31	C	S	40.158	-4.723	-3.450	0.536	5.216	3.413	-0.530	75.362	15.242	19.039
11378	257	41	31	C	S	79.842	-4.723	3.450	0.536	5.216	-3.413	-0.530	75.362	15.242	19.039
11379	257	41	31	C	S	100.158	-4.723	-3.450	0.536	5.216	3.413	-0.530	75.362	15.242	19.039
11380	257	41	31	C	W	19.842	-4.723	3.450	0.536	5.216	-3.413	-0.530	75.362	15.242	19.039
11381	257	41	31	C	W	40.158	-4.723	-3.450	0.536	5.216	3.413	-0.530	75.362	15.242	19.039
11382	257	41	31	C	W	79.842	-4.723	3.450	0.536	5.216	-3.413	-0.530	75.362	15.242	19.039
11383	257	41	31	C	W	100.158	-4.723	-3.450	0.536	5.216	3.413	-0.530	75.362	15.242	19.039
11384	257	41	31	P	S	19.842	-4.723	3.450	0.536	5.216	-3.413	-0.530	75.362	15.242	19.039
11385	257	41	31	P	S	40.158	-4.723	-3.450	0.536	5.216	3.413	-0.530	75.362	15.242	19.039
11386	257	41	31	P	S	79.842	-4.723	3.450	0.536	5.216	-3.413	-0.530	75.362	15.242	19.039
11387	257	41	31	P	S	100.158	-4.723	-3.450	0.536	5.216	3.413	-0.530	75.362	15.242	19.039
11388	257	41	31	P	W	19.842	-4.723	3.450	0.536	5.216	-3.413	-0.530	75.362	15.242	19.039
11389	257	41	31	P	W	40.158	-4.723	-3.450	0.536	5.216	3.413	-0.530	75.362	15.242	19.039
11390	257	41	31	P	W	79.842	-4.723	3.450	0.536	5.216	-3.413	-0.530	75.362	15.242	19.039
11391	257	41	31	P	W	100.158	-4.723	-3.450	0.536	5.216	3.413	-0.530	75.362	15.242	19.039
11392	258	39	34	C	S	10.158	2.281	-5.462	0.462	-2.182	5.412	-0.458	88.797	16.362	17.514
11393	258	39	34	C	S	16.337	0.353	5.487	2.394	-0.351	-5.236	-2.285	88.914	17.539	16.338
11394	258	39	34	C	S	17.897	5.011	2.332	-2.042	-4.554	-2.431	2.129	86.821	16.799	17.100
11395	258	39	34	C	S	25.767	0.353	-1.941	2.394	-0.351	1.852	-2.285	86.933	17.539	16.359
11396	258	39	34	C	S	28.055	2.281	2.109	0.462	-2.182	-2.090	-0.458	86.888	16.362	17.536
11397	258	39	34	C	S	34.233	0.353	1.941	2.394	-0.351	-1.852	-2.285	86.933	17.539	16.359
11398	258	39	34	C	S	42.103	5.011	-2.332	-2.042	-4.554	2.431	2.129	86.821	16.799	17.100
11399	258	39	34	C	S	43.663	0.353	-5.487	2.394	-0.351	5.236	-2.285	88.914	17.539	16.338
11400	258	39	34	C	S	44.392	-2.145	1.707	5.130	2.241	-1.549	-4.652	86.986	17.102	16.795
11401	258	39	34	C	S	49.842	2.281	5.462	0.462	-2.182	-5.412	-0.458	88.797	16.362	17.514
11402	258	39	34	C	S	70.158	2.281	-5.462	0.462	-2.182	5.412	-0.458	88.797	16.362	17.514
11403	258	39	34	C	S	75.609	-2.145	-1.707	5.130	2.241	1.548	-4.652	86.986	17.102	16.795
11404	258	39	34	C	S	76.337	0.353	5.487	2.394	-0.351	-5.236	-2.285	88.914	17.539	16.338
11405	258	39	34	C	S	85.767	0.353	-1.941	2.394	-0.351	1.852	-2.285	86.932	17.539	16.359
11406	258	39	34	C	S	91.945	2.281	-2.109	0.462	-2.182	2.090	-0.458	86.888	16.362	17.536
11407	258	39	34	C	S	94.233	0.353	1.941	2.394	-0.351	-1.852	-2.285	86.933	17.539	16.359
11408	258	39	34	C	S	102.104	5.011	-2.332	-2.042	-4.554	2.431	2.129	86.821	16.799	17.100
11409	258	39	34	C	S	103.663	0.353	-5.487	2.394	-0.351	5.236	-2.285	88.914	17.539	16.338
11410	258	39	34	C	S	104.392	-2.145	1.707	5.130	2.241	-1.549	-4.652	86.986	17.102	16.795
11411	258	39	34	C	S	109.842	2.281	5.462	0.462	-2.182	-5.412	-0.458	88.797	16.362	17.514
11412	258	39	34	C	W	10.158	2.281	-5.462	0.462	-2.182	5.412	-0.458	88.797	16.362	17.514
11413	258	39	34	C	W	15.608	-2.145	-1.707	5.130	2.241	1.548	-4.652	86.986	17.102	16.795
11414	258	39	34	C	W	16.337	0.353	5.487	2.394	-0.351	-5.236	-2.285	88.914	17.539	16.338
11415	258	39	34	C	W	31.945	2.281	-2.109	0.462	-2.182	2.090	-0.458	86.888	16.362	17.536
11416	258	39	34	C	W	43.663	0.353	-5.487	2.394	-0.351	5.236	-2.285	88.914	17.539	16.338
11417	258	39	34	C	W	49.842	2.281	5.462	0.462	-2.182	-5.412	-0.458	88.797	16.362	17.514
11418	258	39	34	C	W	70.158	2.281	-5.462	0.462	-2.182	5.412	-0.458	88.797	16.362	17.514
11419	258	39	34	C	W	76.337	0.353	5.487	2.394	-0.351	-5.236	-2.285	88.914	17.539	16.338
11420	258	39	34	C	W	77.897	5.011	2.332	-2.042	-4.554	-2.431	2.129	86.821	16.799	17.100
11421	258	39	34	C	W	88.055	2.281	2.109	0.462	-2.182	-2.090	-0.458	86.888	16.362	17.536
11422	258	39	34	C	W	103.663	0.353	-5.487	2.394	-0.351	5.236	-2.285	88.914	17.539	16.338
11423	258	39	34	C	W	109.842	2.281	5.462	0.462	-2.182	-5.412	-0.458	88.797	16.362	17.514
11424	258	39	34	P	S	10.158	2.281	-5.462	0.462	-2.182	5.412	-0.458	88.797	16.362	17.514

BL number	Atoms	γ-PC unit cells	WS_2 unit cells	γ-PC origin atom	WS_2 origin atom	Twist-angle (°)	γ-PC strain 1 (%)	γ-PC strain 2 (%)	γ-PC strain 3 (%)	WS_2 strain 1 (%)	WS_2 strain 2 (%)	WS_2 strain 3 (%)	γ (°)	|**a**|(Å)	|**b**|(Å)
11425	258	39	34	P	S	16.337	0.353	5.487	2.394	-0.351	-5.236	-2.285	88.914	17.539	16.338
11426	258	39	34	P	S	17.897	5.011	2.332	-2.042	-4.554	-2.431	2.129	86.821	16.799	17.100
11427	258	39	34	P	S	25.767	0.353	-1.941	2.394	-0.351	1.852	-2.285	86.933	17.539	16.359
11428	258	39	34	P	S	28.055	2.281	2.109	0.462	-2.182	-2.090	-0.458	86.888	16.362	17.536
11429	258	39	34	P	S	34.233	0.353	1.941	2.394	-0.351	-1.852	-2.285	86.933	17.539	16.359
11430	258	39	34	P	S	42.103	5.011	-2.332	-2.042	-4.554	2.431	2.129	86.821	16.799	17.100
11431	258	39	34	P	S	43.663	0.353	-5.487	2.394	-0.351	5.236	-2.285	88.914	17.539	16.338
11432	258	39	34	P	S	44.392	-2.145	1.707	5.130	2.241	-1.549	-4.652	86.986	17.102	16.795
11433	258	39	34	P	S	49.842	2.281	5.462	0.462	-2.182	-5.412	-0.458	88.797	16.362	17.514
11434	258	39	34	P	S	70.158	2.281	-5.462	0.462	-2.182	5.412	-0.458	88.797	16.362	17.514
11435	258	39	34	P	S	75.609	-2.145	-1.707	5.130	2.241	1.548	-4.652	86.986	17.102	16.795
11436	258	39	34	P	S	76.337	0.353	5.487	2.394	-0.351	-5.236	-2.285	88.914	17.539	16.338
11437	258	39	34	P	S	85.767	0.353	-1.941	2.394	-0.351	1.852	-2.285	86.932	17.539	16.359
11438	258	39	34	P	S	91.945	2.281	-2.109	0.462	-2.182	2.090	-0.458	86.888	16.362	17.536
11439	258	39	34	P	S	94.233	0.353	1.941	2.394	-0.351	-1.852	-2.285	86.933	17.539	16.359
11440	258	39	34	P	S	102.104	5.011	-2.332	-2.042	-4.554	2.431	2.129	86.821	16.799	17.100
11441	258	39	34	P	S	103.663	0.353	-5.487	2.394	-0.351	5.236	-2.285	88.914	17.539	16.338
11442	258	39	34	P	S	104.392	-2.145	1.707	5.130	2.241	-1.549	-4.652	86.986	17.102	16.795
11443	258	39	34	P	S	109.842	2.281	5.462	0.462	-2.182	-5.412	-0.458	88.797	16.362	17.514
11444	258	39	34	P	W	10.158	2.281	-5.462	0.462	-2.182	5.412	-0.458	88.797	16.362	17.514
11445	258	39	34	P	W	15.608	-2.145	-1.707	5.130	2.241	1.548	-4.652	86.986	17.102	16.795
11446	258	39	34	P	W	16.337	0.353	5.487	2.394	-0.351	-5.236	-2.285	88.914	17.539	16.338
11447	258	39	34	P	W	31.945	2.281	-2.109	0.462	-2.182	2.090	-0.458	86.888	16.362	17.536
11448	258	39	34	P	W	43.663	0.353	-5.487	2.394	-0.351	5.236	-2.285	88.914	17.539	16.338
11449	258	39	34	P	W	49.842	2.281	5.462	0.462	-2.182	-5.412	-0.458	88.797	16.362	17.514
11450	258	39	34	P	W	70.158	2.281	-5.462	0.462	-2.182	5.412	-0.458	88.797	16.362	17.514
11451	258	39	34	P	W	76.337	0.353	5.487	2.394	-0.351	-5.236	-2.285	88.914	17.539	16.338
11452	258	39	34	P	W	77.897	5.011	2.332	-2.042	-4.554	-2.431	2.129	86.821	16.799	17.100
11453	258	39	34	P	W	88.055	2.281	2.109	0.462	-2.182	-2.090	-0.458	86.888	16.362	17.536
11454	258	39	34	P	W	103.663	0.353	-5.487	2.394	-0.351	5.236	-2.285	88.914	17.539	16.338
11455	258	39	34	P	W	109.842	2.281	5.462	0.462	-2.182	-5.412	-0.458	88.797	16.362	17.514
11456	258	42	30	C	S	0.000	-4.158	-3.952	-2.848	4.535	4.191	3.020	87.670	16.522	16.936
11457	258	42	30	C	S	1.359	-3.292	1.501	-3.722	3.523	-1.622	4.021	68.265	18.221	16.518
11458	258	42	30	C	S	3.004	-3.388	2.704	-3.626	3.635	-2.915	3.910	77.593	17.335	16.513
11459	258	42	30	C	S	4.715	-3.007	4.000	-4.002	3.200	-4.348	4.350	87.614	16.951	16.507
11460	258	42	30	C	S	6.178	-2.145	4.852	-4.831	2.241	-5.371	5.347	82.267	17.102	16.501
11461	258	42	30	C	S	53.822	-2.145	-4.852	-4.831	2.241	5.371	5.347	82.267	17.102	16.501
11462	258	42	30	C	S	55.285	-3.007	-4.000	-4.002	3.200	4.348	4.350	87.614	16.951	16.507
11463	258	42	30	C	S	56.996	-3.388	-2.703	-3.626	3.635	2.915	3.910	77.593	17.335	16.513
11464	258	42	30	C	S	58.641	-3.292	-1.501	-3.722	3.523	1.622	4.021	68.265	18.221	16.518
11465	258	42	30	C	S	60.000	-4.158	-3.952	-2.848	4.535	4.191	3.020	87.670	16.522	16.936
11466	258	42	30	C	S	61.359	-3.292	1.501	-3.722	3.523	-1.622	4.021	68.265	18.221	16.518
11467	258	42	30	C	S	63.005	-3.388	2.704	-3.626	3.635	-2.915	3.910	77.593	17.335	16.513
11468	258	42	30	C	S	64.715	-3.007	4.000	-4.002	3.200	-4.348	4.350	87.614	16.951	16.507
11469	258	42	30	C	S	66.178	-2.145	4.852	-4.831	2.241	-5.371	5.347	82.267	17.102	16.501
11470	258	42	30	C	S	113.822	-2.145	-4.852	-4.831	2.241	5.371	5.347	82.267	17.102	16.501
11471	258	42	30	C	S	115.285	-3.007	-4.000	-4.002	3.200	4.348	4.350	87.614	16.951	16.507
11472	258	42	30	C	S	116.996	-3.388	-2.703	-3.626	3.635	2.915	3.910	77.593	17.335	16.513
11473	258	42	30	C	S	118.641	-3.292	-1.501	-3.722	3.523	1.622	4.021	68.265	18.221	16.518
11474	258	42	30	C	W	0.000	-4.158	-3.952	-2.848	4.535	4.191	3.020	87.670	16.522	16.936
11475	258	42	30	C	W	1.359	-3.292	1.501	-3.722	3.523	-1.622	4.021	68.265	18.221	16.518

BL number	Atoms	γ-PC unit cells	WS_2 unit cells	γ-PC origin atom	WS_2 origin atom	Twist-angle (°)	γ-PC strain 1 (%)	γ-PC strain 2 (%)	γ-PC strain 3 (%)	WS_2 strain 1 (%)	WS_2 strain 2 (%)	WS_2 strain 3 (%)	γ (°)	|**a**|(Å)	|**b**|(Å)
11476	258	42	30	C	W	3.004	-3.388	2.704	-3.626	3.635	-2.915	3.910	77.593	17.335	16.513
11477	258	42	30	C	W	4.715	-3.007	4.000	-4.002	3.200	-4.348	4.350	87.614	16.951	16.507
11478	258	42	30	C	W	6.178	-2.145	4.852	-4.831	2.241	-5.371	5.347	82.267	17.102	16.501
11479	258	42	30	C	W	53.822	-2.145	-4.852	-4.831	2.241	5.371	5.347	82.267	17.102	16.501
11480	258	42	30	C	W	55.285	-3.007	-4.000	-4.002	3.200	4.348	4.350	87.614	16.951	16.507
11481	258	42	30	C	W	56.996	-3.388	-2.703	-3.626	3.635	2.915	3.910	77.593	17.335	16.513
11482	258	42	30	C	W	58.641	-3.292	-1.501	-3.722	3.523	1.622	4.021	68.265	18.221	16.518
11483	258	42	30	C	W	60.000	-4.158	-3.952	-2.848	4.535	4.191	3.020	87.670	16.522	16.936
11484	258	42	30	C	W	61.359	-3.292	1.501	-3.722	3.523	-1.622	4.021	68.265	18.221	16.518
11485	258	42	30	C	W	63.005	-3.388	2.704	-3.626	3.635	-2.915	3.910	77.593	17.335	16.513
11486	258	42	30	C	W	64.715	-3.007	4.000	-4.002	3.200	-4.348	4.350	87.614	16.951	16.507
11487	258	42	30	C	W	66.178	-2.145	4.852	-4.831	2.241	-5.371	5.347	82.267	17.102	16.501
11488	258	42	30	C	W	113.822	-2.145	-4.852	-4.831	2.241	5.371	5.347	82.267	17.102	16.501
11489	258	42	30	C	W	115.285	-3.007	-4.000	-4.002	3.200	4.348	4.350	87.614	16.951	16.507
11490	258	42	30	C	W	116.996	-3.388	-2.703	-3.626	3.635	2.915	3.910	77.593	17.335	16.513
11491	258	42	30	C	W	118.641	-3.292	-1.501	-3.722	3.523	1.622	4.021	68.265	18.221	16.518
11492	258	42	30	P	S	0.000	-4.158	-3.952	-2.848	4.535	4.191	3.020	87.670	16.522	16.936
11493	258	42	30	P	S	1.359	-3.292	1.501	-3.722	3.523	-1.622	4.021	68.265	18.221	16.518
11494	258	42	30	P	S	4.715	-3.007	4.000	-4.002	3.200	-4.348	4.350	87.614	16.951	16.507
11495	258	42	30	P	S	6.178	-2.145	4.852	-4.831	2.241	-5.371	5.347	82.267	17.102	16.501
11496	258	42	30	P	S	53.822	-2.145	-4.852	-4.831	2.241	5.371	5.347	82.267	17.102	16.501
11497	258	42	30	P	S	55.285	-3.007	-4.000	-4.002	3.200	4.348	4.350	87.614	16.951	16.507
11498	258	42	30	P	S	58.641	-3.292	-1.501	-3.722	3.523	1.622	4.021	68.265	18.221	16.518
11499	258	42	30	P	S	60.000	-4.158	-3.952	-2.848	4.535	4.191	3.020	87.670	16.522	16.936
11500	258	42	30	P	S	61.359	-3.292	1.501	-3.722	3.523	-1.622	4.021	68.265	18.221	16.518
11501	258	42	30	P	S	64.715	-3.007	4.000	-4.002	3.200	-4.348	4.350	87.614	16.951	16.507
11502	258	42	30	P	S	66.178	-2.145	4.852	-4.831	2.241	-5.371	5.347	82.267	17.102	16.501
11503	258	42	30	P	S	113.822	-2.145	-4.852	-4.831	2.241	5.371	5.347	82.267	17.102	16.501
11504	258	42	30	P	S	115.285	-3.007	-4.000	-4.002	3.200	4.348	4.350	87.614	16.951	16.507
11505	258	42	30	P	S	118.641	-3.292	-1.501	-3.722	3.523	1.622	4.021	68.265	18.221	16.518
11506	258	42	30	P	W	0.000	-4.158	-3.952	-2.848	4.535	4.191	3.020	87.670	16.522	16.936
11507	258	42	30	P	W	1.359	-3.292	1.501	-3.722	3.523	-1.622	4.021	68.265	18.221	16.518
11508	258	42	30	P	W	4.715	-3.007	4.000	-4.002	3.200	-4.348	4.350	87.614	16.951	16.507
11509	258	42	30	P	W	6.178	-2.145	4.852	-4.831	2.241	-5.371	5.347	82.267	17.102	16.501
11510	258	42	30	P	W	53.822	-2.145	-4.852	-4.831	2.241	5.371	5.347	82.267	17.102	16.501
11511	258	42	30	P	W	55.285	-3.007	-4.000	-4.002	3.200	4.348	4.350	87.614	16.951	16.507
11512	258	42	30	P	W	58.641	-3.292	-1.501	-3.722	3.523	1.622	4.021	68.265	18.221	16.518
11513	258	42	30	P	W	60.000	-4.158	-3.952	-2.848	4.535	4.191	3.020	87.670	16.522	16.936
11514	258	42	30	P	W	61.359	-3.292	1.501	-3.722	3.523	-1.622	4.021	68.265	18.221	16.518
11515	258	42	30	P	W	64.715	-3.007	4.000	-4.002	3.200	-4.348	4.350	87.614	16.951	16.507
11516	258	42	30	P	W	66.178	-2.145	4.852	-4.831	2.241	-5.371	5.347	82.267	17.102	16.501
11517	258	42	30	P	W	113.822	-2.145	-4.852	-4.831	2.241	5.371	5.347	82.267	17.102	16.501
11518	258	42	30	P	W	115.285	-3.007	-4.000	-4.002	3.200	4.348	4.350	87.614	16.951	16.507
11519	258	42	30	P	W	118.641	-3.292	-1.501	-3.722	3.523	1.622	4.021	68.265	18.221	16.518
11520	259	37	37	C	S	9.430	5.011	0.000	5.011	-4.554	0.000	-4.555	60.000	18.353	18.353
11521	259	37	37	C	S	50.570	5.011	0.000	5.011	-4.554	0.000	-4.555	60.000	18.353	18.353
11522	259	37	37	C	S	69.430	5.011	0.000	5.011	-4.554	0.000	-4.555	60.000	18.353	18.353
11523	259	37	37	C	S	110.570	5.011	0.000	5.011	-4.554	0.000	-4.555	60.000	18.353	18.353
11524	259	37	37	C	W	9.430	5.011	0.000	5.011	-4.554	0.000	-4.555	60.000	18.353	18.353
11525	259	37	37	C	W	50.570	5.011	0.000	5.011	-4.554	0.000	-4.555	60.000	18.353	18.353
11526	259	37	37	C	W	69.430	5.011	0.000	5.011	-4.554	0.000	-4.555	60.000	18.353	18.353

BL number	Atoms	γ-PC unit cells	WS_2 unit cells	γ-PC origin atom	WS_2 origin atom	Twist-angle (°)	γ-PC strain 1 (%)	γ-PC strain 2 (%)	γ-PC strain 3 (%)	WS_2 strain 1 (%)	WS_2 strain 2 (%)	WS_2 strain 3 (%)	γ (°)	|**a**|(Å)	|**b**|(Å)
11527	259	37	37	C	W	110.570	5.011	0.000	5.011	-4.554	0.000	-4.555	60.000	18.353	18.353
11528	259	37	37	P	S	50.570	5.011	0.000	5.011	-4.554	0.000	-4.555	60.000	18.353	18.353
11529	259	37	37	P	S	69.430	5.011	0.000	5.011	-4.554	0.000	-4.555	60.000	18.353	18.353
11530	259	37	37	P	W	9.430	5.011	0.000	5.011	-4.554	0.000	-4.555	60.000	18.353	18.353
11531	259	37	37	P	W	110.570	5.011	0.000	5.011	-4.554	0.000	-4.555	60.000	18.353	18.353
11532	259	40	33	C	S	0.000	5.011	0.758	-4.616	-4.554	-0.835	5.085	67.164	18.353	16.935
11533	259	40	33	C	S	1.945	-4.723	1.080	5.141	5.216	-0.980	-4.662	86.098	15.242	18.839
11534	259	40	33	C	S	8.513	2.390	0.511	-2.345	-2.281	-0.537	2.461	86.835	19.291	14.845
11535	259	40	33	C	S	10.893	4.020	-4.004	-3.783	-3.721	4.331	4.093	84.061	15.815	18.195
11536	259	40	33	C	S	13.898	-4.228	-4.838	4.545	4.619	4.435	-4.166	82.840	17.184	16.793
11537	259	40	33	C	S	17.897	5.011	2.274	-4.616	-4.554	-2.505	5.085	83.204	16.799	17.172
11538	259	40	33	C	S	21.052	1.339	-3.169	-1.370	-1.304	3.259	1.409	83.094	16.211	17.760
11539	259	40	33	C	S	25.050	-0.955	3.805	0.904	0.973	-3.737	-0.888	83.004	17.772	16.202
11540	259	40	33	C	S	34.950	-0.955	-3.805	0.904	0.973	3.737	-0.888	83.004	17.772	16.202
11541	259	40	33	C	S	38.948	1.339	3.169	-1.370	-1.304	-3.259	1.409	83.094	16.211	17.760
11542	259	40	33	C	S	42.103	5.011	-2.273	-4.616	-4.554	2.505	5.085	83.204	16.799	17.172
11543	259	40	33	C	S	46.102	-0.414	-2.654	0.349	0.417	2.635	-0.347	88.485	19.824	14.421
11544	259	40	33	C	S	49.107	4.020	-3.003	-3.783	-3.721	3.248	4.093	67.917	15.815	19.531
11545	259	40	33	C	S	51.487	2.390	-0.511	-2.345	-2.281	0.537	2.461	86.835	19.291	14.845
11546	259	40	33	C	S	58.055	-4.723	-1.080	5.141	5.216	0.980	-4.662	86.098	15.242	18.839
11547	259	40	33	C	S	60.000	5.011	2.274	-4.616	-4.554	-2.505	5.085	85.910	18.842	15.241
11548	259	40	33	C	S	61.945	-4.723	1.080	5.141	5.216	-0.980	-4.662	86.098	15.242	18.839
11549	259	40	33	C	S	68.513	2.390	0.511	-2.345	-2.281	-0.537	2.461	86.835	19.291	14.845
11550	259	40	33	C	S	70.893	4.020	-4.004	-3.783	-3.721	4.331	4.093	84.061	15.815	18.195
11551	259	40	33	C	S	73.898	-4.228	-4.838	4.545	4.619	4.435	-4.166	82.840	17.184	16.793
11552	259	40	33	C	S	77.897	5.011	2.274	-4.616	-4.554	-2.505	5.085	83.204	16.799	17.172
11553	259	40	33	C	S	81.052	1.339	-3.169	-1.370	-1.304	3.259	1.409	83.094	16.211	17.760
11554	259	40	33	C	S	85.050	-0.955	3.805	0.904	0.973	-3.737	-0.888	83.004	17.772	16.202
11555	259	40	33	C	S	94.950	-0.955	-3.805	0.904	0.973	3.737	-0.888	83.004	17.772	16.202
11556	259	40	33	C	S	98.948	1.339	3.169	-1.370	-1.304	-3.259	1.409	83.094	16.211	17.760
11557	259	40	33	C	S	102.104	5.011	-2.273	-4.616	-4.554	2.505	5.085	83.204	16.799	17.172
11558	259	40	33	C	S	106.102	-4.228	4.838	4.545	4.619	-4.435	-4.166	82.840	17.184	16.793
11559	259	40	33	C	S	109.107	0.418	2.588	-0.482	-0.415	-2.613	0.487	88.510	14.426	19.817
11560	259	40	33	C	S	118.055	-4.723	-1.080	5.141	5.216	0.980	-4.662	86.098	15.242	18.839
11561	259	40	33	C	W	0.000	5.011	0.758	-4.616	-4.554	-0.835	5.085	67.164	18.353	16.935
11562	259	40	33	C	W	1.945	-4.723	1.080	5.141	5.216	-0.980	-4.662	86.098	15.242	18.839
11563	259	40	33	C	W	8.513	2.390	0.511	-2.345	-2.281	-0.537	2.461	86.835	19.291	14.845
11564	259	40	33	C	W	10.893	4.020	-4.004	-3.783	-3.721	4.331	4.093	84.061	15.815	18.195
11565	259	40	33	C	W	13.898	-4.228	-4.838	4.545	4.619	4.435	-4.166	82.840	17.184	16.793
11566	259	40	33	C	W	17.897	5.011	2.274	-4.616	-4.554	-2.505	5.085	83.204	16.799	17.172
11567	259	40	33	C	W	21.052	1.339	-3.169	-1.370	-1.304	3.259	1.409	83.094	16.211	17.760
11568	259	40	33	C	W	25.050	-0.955	3.805	0.904	0.973	-3.737	-0.888	83.004	17.772	16.202
11569	259	40	33	C	W	34.950	-0.955	-3.805	0.904	0.973	3.737	-0.888	83.004	17.772	16.202
11570	259	40	33	C	W	38.948	1.339	3.169	-1.370	-1.304	-3.259	1.409	83.094	16.211	17.760
11571	259	40	33	C	W	42.103	5.011	-2.273	-4.616	-4.554	2.505	5.085	83.204	16.799	17.172
11572	259	40	33	C	W	46.102	-0.414	-2.654	0.349	0.417	2.635	-0.347	88.485	19.824	14.421
11573	259	40	33	C	W	49.107	0.418	2.588	-0.482	-0.415	-2.613	0.487	88.510	14.426	19.817
11574	259	40	33	C	W	58.055	-4.723	-1.080	5.141	5.216	0.980	-4.662	86.098	15.242	18.839
11575	259	40	33	C	W	60.000	5.011	2.274	-4.616	-4.554	-2.505	5.085	85.910	18.842	15.241
11576	259	40	33	C	W	61.945	-4.723	1.080	5.141	5.216	-0.980	-4.662	86.098	15.242	18.839
11577	259	40	33	C	W	68.513	2.390	0.511	-2.345	-2.281	-0.537	2.461	86.835	19.291	14.845

BL number	Atoms	γ-PC unit cells	WS_2 unit cells	γ-PC origin atom	WS_2 origin atom	Twist-angle (°)	γ-PC strain 1 (%)	γ-PC strain 2 (%)	γ-PC strain 3 (%)	WS_2 strain 1 (%)	WS_2 strain 2 (%)	WS_2 strain 3 (%)	γ (°)	|**a**|(Å)	|**b**|(Å)
11578	259	40	33	C	W	70.893	0.418	-2.588	-0.482	-0.415	2.613	0.487	88.510	14.426	19.817
11579	259	40	33	C	W	73.898	-4.228	-4.838	4.545	4.619	4.435	-4.166	82.840	17.184	16.793
11580	259	40	33	C	W	77.897	5.011	2.274	-4.616	-4.554	-2.505	5.085	83.204	16.799	17.172
11581	259	40	33	C	W	81.052	1.339	-3.169	-1.370	-1.304	3.259	1.409	83.094	16.211	17.760
11582	259	40	33	C	W	85.050	-0.955	3.805	0.904	0.973	-3.737	-0.888	83.004	17.772	16.202
11583	259	40	33	C	W	94.950	-0.955	-3.805	0.904	0.973	3.737	-0.888	83.004	17.772	16.202
11584	259	40	33	C	W	98.948	1.339	3.169	-1.370	-1.304	-3.259	1.409	83.094	16.211	17.760
11585	259	40	33	C	W	102.104	5.011	-2.273	-4.616	-4.554	2.505	5.085	83.204	16.799	17.172
11586	259	40	33	C	W	106.102	-4.228	4.838	4.545	4.619	-4.435	-4.166	82.840	17.184	16.793
11587	259	40	33	C	W	109.107	4.020	-3.003	-3.783	-3.721	3.248	4.093	67.917	15.815	19.530
11588	259	40	33	C	W	111.487	2.390	-0.511	-2.345	-2.281	0.537	2.461	86.835	19.291	14.845
11589	259	40	33	C	W	118.055	-4.723	-1.080	5.141	5.216	0.980	-4.662	86.098	15.242	18.839
11590	259	40	33	P	S	0.000	5.011	0.758	-4.616	-4.554	-0.835	5.085	67.164	18.353	16.935
11591	259	40	33	P	S	1.945	-4.723	1.080	5.141	5.216	-0.980	-4.662	86.098	15.242	18.839
11592	259	40	33	P	S	10.893	4.020	-4.004	-3.783	-3.721	4.331	4.093	84.061	15.815	18.195
11593	259	40	33	P	S	21.052	1.339	-3.169	-1.370	-1.304	3.259	1.409	83.094	16.211	17.760
11594	259	40	33	P	S	25.050	-0.955	3.805	0.904	0.973	-3.737	-0.888	83.004	17.772	16.202
11595	259	40	33	P	S	34.950	-0.955	-3.805	0.904	0.973	3.737	-0.888	83.004	17.772	16.202
11596	259	40	33	P	S	38.948	1.339	3.169	-1.370	-1.304	-3.259	1.409	83.094	16.211	17.760
11597	259	40	33	P	S	46.102	-0.414	-2.654	0.349	0.417	2.635	-0.347	88.485	19.824	14.421
11598	259	40	33	P	S	49.107	0.418	2.588	-0.482	-0.415	-2.613	0.487	88.510	14.426	19.817
11599	259	40	33	P	S	60.000	5.011	0.758	-4.616	-4.554	-0.835	5.085	67.164	18.353	16.935
11600	259	40	33	P	S	70.893	0.418	-2.588	-0.482	-0.415	2.613	0.487	88.510	14.426	19.817
11601	259	40	33	P	S	73.898	-0.414	2.654	0.349	0.417	-2.635	-0.347	88.485	19.824	14.421
11602	259	40	33	P	S	81.052	1.339	-3.169	-1.370	-1.304	3.259	1.409	83.094	16.211	17.760
11603	259	40	33	P	S	85.050	-0.955	3.805	0.904	0.973	-3.737	-0.888	83.004	17.772	16.202
11604	259	40	33	P	S	94.950	-0.955	-3.805	0.904	0.973	3.737	-0.888	83.004	17.772	16.202
11605	259	40	33	P	S	98.948	1.339	3.169	-1.370	-1.304	-3.259	1.409	83.094	16.211	17.760
11606	259	40	33	P	S	109.107	4.020	-3.003	-3.783	-3.721	3.248	4.093	67.917	15.815	19.530
11607	259	40	33	P	S	111.487	2.390	-0.511	-2.345	-2.281	0.537	2.461	86.835	19.291	14.845
11608	259	40	33	P	S	118.055	-4.723	-1.080	5.141	5.216	0.980	-4.662	86.098	15.242	18.839
11609	259	40	33	P	W	0.000	5.011	0.758	-4.616	-4.554	-0.835	5.085	67.164	18.353	16.935
11610	259	40	33	P	W	10.893	4.020	-4.004	-3.783	-3.721	4.331	4.093	84.061	15.815	18.195
11611	259	40	33	P	W	13.898	-0.414	2.654	0.349	0.417	-2.635	-0.347	88.485	19.824	14.421
11612	259	40	33	P	W	21.052	1.339	-3.169	-1.370	-1.304	3.259	1.409	83.094	16.211	17.760
11613	259	40	33	P	W	25.050	-0.955	3.805	0.904	0.973	-3.737	-0.888	83.004	17.772	16.202
11614	259	40	33	P	W	34.950	-0.955	-3.805	0.904	0.973	3.737	-0.888	83.004	17.772	16.202
11615	259	40	33	P	W	38.948	1.339	3.169	-1.370	-1.304	-3.259	1.409	83.094	16.211	17.760
11616	259	40	33	P	W	49.107	4.020	-3.003	-3.783	-3.721	3.248	4.093	67.917	15.815	19.531
11617	259	40	33	P	W	51.487	2.390	-0.511	-2.345	-2.281	0.537	2.461	86.835	19.291	14.845
11618	259	40	33	P	W	58.055	-4.723	-1.080	5.141	5.216	0.980	-4.662	86.098	15.242	18.839
11619	259	40	33	P	W	60.000	5.011	2.274	-4.616	-4.554	-2.505	5.085	85.910	18.842	15.241
11620	259	40	33	P	W	61.945	-4.723	1.080	5.141	5.216	-0.980	-4.662	86.098	15.242	18.839
11621	259	40	33	P	W	70.893	4.020	-4.004	-3.783	-3.721	4.331	4.093	84.061	15.815	18.195
11622	259	40	33	P	W	81.052	1.339	-3.169	-1.370	-1.304	3.259	1.409	83.094	16.211	17.760
11623	259	40	33	P	W	85.050	-0.955	3.805	0.904	0.973	-3.737	-0.888	83.004	17.772	16.202
11624	259	40	33	P	W	94.950	-0.955	-3.805	0.904	0.973	3.737	-0.888	83.004	17.772	16.202
11625	259	40	33	P	W	98.948	1.339	3.169	-1.370	-1.304	-3.259	1.409	83.094	16.211	17.760
11626	259	40	33	P	W	106.102	-0.414	-2.654	0.349	0.417	2.635	-0.347	88.485	19.824	14.421
11627	259	40	33	P	W	109.107	0.418	2.588	-0.482	-0.415	-2.613	0.487	88.510	14.426	19.817
11628	260	38	36	C	S	8.513	2.390	-1.675	4.723	-2.281	1.531	-4.315	72.734	19.291	15.813

BL number	Atoms	γ-PC unit cells	WS_2 unit cells	γ-PC origin atom	WS_2 origin atom	Twist-angle (°)	γ-PC strain 1 (%)	γ-PC strain 2 (%)	γ-PC strain 3 (%)	WS_2 strain 1 (%)	WS_2 strain 2 (%)	WS_2 strain 3 (%)	γ (°)	|**a**|(Å)	|**b**|(Å)
11629	260	38	36	C	S	10.158	2.281	1.332	4.836	-2.182	-1.214	-4.410	75.971	16.362	18.351
11630	260	38	36	C	S	10.893	4.020	-4.741	3.072	-3.721	4.467	-2.894	87.366	15.815	18.437
11631	260	38	36	C	S	16.102	2.853	4.688	4.244	-2.699	-4.321	-3.912	87.425	18.455	15.799
11632	260	38	36	C	S	43.898	2.853	-4.688	4.244	-2.699	4.321	-3.912	87.425	18.455	15.799
11633	260	38	36	C	S	49.107	4.020	-2.634	3.072	-3.721	2.482	-2.894	72.684	15.815	19.292
11634	260	38	36	C	S	49.842	2.281	-1.332	4.836	-2.182	1.214	-4.410	75.971	16.362	18.351
11635	260	38	36	C	S	51.487	2.390	1.675	4.723	-2.281	-1.531	-4.315	72.734	19.291	15.813
11636	260	38	36	C	S	68.513	2.390	-1.675	4.723	-2.281	1.531	-4.315	72.734	19.291	15.813
11637	260	38	36	C	S	70.158	2.281	1.332	4.836	-2.182	-1.214	-4.410	75.970	16.362	18.351
11638	260	38	36	C	S	70.893	4.020	-4.741	3.072	-3.721	4.467	-2.894	87.366	15.815	18.437
11639	260	38	36	C	S	76.102	2.853	4.688	4.244	-2.699	-4.321	-3.912	87.425	18.455	15.799
11640	260	38	36	C	S	103.898	2.853	-4.688	4.244	-2.699	4.321	-3.912	87.425	18.455	15.799
11641	260	38	36	C	S	109.107	4.020	-2.634	3.072	-3.721	2.482	-2.894	72.684	15.815	19.292
11642	260	38	36	C	S	111.487	2.390	1.675	4.723	-2.281	-1.531	-4.315	72.734	19.291	15.813
11643	260	38	36	C	W	8.513	2.390	-1.675	4.723	-2.281	1.531	-4.315	72.734	19.291	15.813
11644	260	38	36	C	W	10.158	2.281	1.332	4.836	-2.182	-1.214	-4.410	75.971	16.362	18.351
11645	260	38	36	C	W	10.893	4.020	-4.741	3.072	-3.721	4.467	-2.894	87.366	15.815	18.437
11646	260	38	36	C	W	16.102	2.853	4.688	4.244	-2.699	-4.321	-3.912	87.425	18.455	15.799
11647	260	38	36	C	W	43.898	2.853	-4.688	4.244	-2.699	4.321	-3.912	87.425	18.455	15.799
11648	260	38	36	C	W	49.107	4.020	-2.634	3.072	-3.721	2.482	-2.894	72.684	15.815	19.292
11649	260	38	36	C	W	51.487	2.390	1.675	4.723	-2.281	-1.531	-4.315	72.734	19.291	15.813
11650	260	38	36	C	W	68.513	2.390	-1.675	4.723	-2.281	1.531	-4.315	72.734	19.291	15.813
11651	260	38	36	C	W	70.158	2.281	1.332	4.836	-2.182	-1.214	-4.410	75.970	16.362	18.351
11652	260	38	36	C	W	70.893	4.020	-4.741	3.072	-3.721	4.467	-2.894	87.366	15.815	18.437
11653	260	38	36	C	W	76.102	2.853	4.688	4.244	-2.699	-4.321	-3.912	87.425	18.455	15.799
11654	260	38	36	C	W	103.898	2.853	-4.688	4.244	-2.699	4.321	-3.912	87.425	18.455	15.799
11655	260	38	36	C	W	109.107	4.020	-2.634	3.072	-3.721	2.482	-2.894	72.684	15.815	19.292
11656	260	38	36	C	W	109.842	2.281	-1.332	4.836	-2.182	1.214	-4.410	75.970	16.362	18.351
11657	260	38	36	C	W	111.487	2.390	1.675	4.723	-2.281	-1.531	-4.315	72.734	19.291	15.813
11658	260	38	36	P	S	49.107	4.020	-2.634	3.072	-3.721	2.482	-2.894	72.684	15.815	19.292
11659	260	38	36	P	S	51.487	2.390	1.675	4.723	-2.281	-1.531	-4.315	72.734	19.291	15.813
11660	260	38	36	P	S	68.513	2.390	-1.675	4.723	-2.281	1.531	-4.315	72.734	19.291	15.813
11661	260	38	36	P	S	70.893	4.020	2.634	3.072	-3.721	-2.482	-2.894	72.684	15.815	19.292
11662	260	38	36	P	S	109.842	2.281	-1.332	4.836	-2.182	1.214	-4.410	75.970	16.362	18.351
11663	260	38	36	P	W	8.513	2.390	-1.675	4.723	-2.281	1.531	-4.315	72.734	19.291	15.813
11664	260	38	36	P	W	10.893	4.020	2.634	3.072	-3.721	-2.482	-2.894	72.684	15.815	19.292
11665	260	38	36	P	W	49.842	2.281	-1.332	4.836	-2.182	1.214	-4.410	75.971	16.362	18.351
11666	260	38	36	P	W	109.107	4.020	-2.634	3.072	-3.721	2.482	-2.894	72.684	15.815	19.292
11667	260	38	36	P	W	111.487	2.390	1.675	4.723	-2.281	-1.531	-4.315	72.734	19.291	15.813
11668	260	41	32	C	S	6.178	-2.145	-0.394	-0.644	2.241	0.399	0.653	69.670	17.102	17.771
11669	260	41	32	C	S	7.154	-0.955	1.282	-1.842	0.973	-1.331	1.913	69.666	17.772	17.101
11670	260	41	32	C	S	24.791	-3.388	0.583	0.672	3.635	-0.575	-0.663	69.662	17.335	17.539
11671	260	41	32	C	S	34.233	0.353	-2.211	-3.093	-0.351	2.357	3.297	69.649	17.539	17.335
11672	260	41	32	C	S	52.846	-0.955	-1.282	-1.842	0.973	1.331	1.913	69.666	17.772	17.101
11673	260	41	32	C	S	53.822	-2.145	0.394	-0.644	2.241	-0.399	0.653	69.670	17.102	17.771
11674	260	41	32	C	S	66.178	-2.145	-0.394	-0.644	2.241	0.399	0.653	69.670	17.102	17.771
11675	260	41	32	C	S	67.154	-0.955	1.282	-1.842	0.973	-1.331	1.913	69.666	17.772	17.101
11676	260	41	32	C	S	84.791	-3.388	0.583	0.672	3.635	-0.575	-0.663	69.662	17.335	17.539
11677	260	41	32	C	S	94.233	0.353	-2.211	-3.093	-0.351	2.357	3.297	69.649	17.539	17.335
11678	260	41	32	C	S	112.846	-0.955	-1.282	-1.842	0.973	1.331	1.913	69.666	17.772	17.101
11679	260	41	32	C	S	113.822	-2.145	0.394	-0.644	2.241	-0.399	0.653	69.670	17.102	17.771

BL number	Atoms	γ-PC unit cells	WS_2 unit cells	γ-PC origin atom	WS_2 origin atom	Twist-angle (°)	γ-PC strain 1 (%)	γ-PC strain 2 (%)	γ-PC strain 3 (%)	WS_2 strain 1 (%)	WS_2 strain 2 (%)	WS_2 strain 3 (%)	γ (°)	|**a**|(Å)	|**b**|(Å)
11680	260	41	32	C	W	6.178	-2.145	-0.394	-0.644	2.241	0.399	0.653	69.670	17.102	17.771
11681	260	41	32	C	W	7.154	-0.955	1.282	-1.842	0.973	-1.331	1.913	69.666	17.772	17.101
11682	260	41	32	C	W	24.791	-3.388	0.583	0.672	3.635	-0.575	-0.663	69.662	17.335	17.539
11683	260	41	32	C	W	34.233	0.353	-2.211	-3.093	-0.351	2.357	3.297	69.649	17.539	17.335
11684	260	41	32	C	W	52.846	-0.955	-1.282	-1.842	0.973	1.331	1.913	69.666	17.772	17.101
11685	260	41	32	C	W	53.822	-2.145	0.394	-0.644	2.241	-0.399	0.653	69.670	17.102	17.771
11686	260	41	32	C	W	66.178	-2.145	-0.394	-0.644	2.241	0.399	0.653	69.670	17.102	17.771
11687	260	41	32	C	W	67.154	-0.955	1.282	-1.842	0.973	-1.331	1.913	69.666	17.772	17.101
11688	260	41	32	C	W	84.791	-3.388	0.583	0.672	3.635	-0.575	-0.663	69.662	17.335	17.539
11689	260	41	32	C	W	94.233	0.353	-2.211	-3.093	-0.351	2.357	3.297	69.649	17.539	17.335
11690	260	41	32	C	W	112.846	-0.955	-1.282	-1.842	0.973	1.331	1.913	69.666	17.772	17.101
11691	260	41	32	C	W	113.822	-2.145	0.394	-0.644	2.241	-0.399	0.653	69.670	17.102	17.771
11692	260	41	32	P	S	6.178	-2.145	-0.394	-0.644	2.241	0.399	0.653	69.670	17.102	17.771
11693	260	41	32	P	S	7.154	-0.955	1.282	-1.842	0.973	-1.331	1.913	69.666	17.772	17.101
11694	260	41	32	P	S	24.791	-3.388	0.583	0.672	3.635	-0.575	-0.663	69.662	17.335	17.539
11695	260	41	32	P	S	34.233	0.353	-2.211	-3.093	-0.351	2.357	3.297	69.649	17.539	17.335
11696	260	41	32	P	S	52.846	-0.955	-1.282	-1.842	0.973	1.331	1.913	69.666	17.772	17.101
11697	260	41	32	P	S	53.822	-2.145	0.394	-0.644	2.241	-0.399	0.653	69.670	17.102	17.771
11698	260	41	32	P	S	66.178	-2.145	-0.394	-0.644	2.241	0.399	0.653	69.670	17.102	17.771
11699	260	41	32	P	S	67.154	-0.955	1.282	-1.842	0.973	-1.331	1.913	69.666	17.772	17.101
11700	260	41	32	P	S	84.791	-3.388	0.583	0.672	3.635	-0.575	-0.663	69.662	17.335	17.539
11701	260	41	32	P	S	94.233	0.353	-2.211	-3.093	-0.351	2.357	3.297	69.649	17.539	17.335
11702	260	41	32	P	S	112.846	-0.955	-1.282	-1.842	0.973	1.331	1.913	69.666	17.772	17.101
11703	260	41	32	P	S	113.822	-2.145	0.394	-0.644	2.241	-0.399	0.653	69.670	17.102	17.771
11704	260	41	32	P	W	6.178	-2.145	-0.394	-0.644	2.241	0.399	0.653	69.670	17.102	17.771
11705	260	41	32	P	W	7.154	-0.955	1.282	-1.842	0.973	-1.331	1.913	69.666	17.772	17.101
11706	260	41	32	P	W	24.791	-3.388	0.583	0.672	3.635	-0.575	-0.663	69.662	17.335	17.539
11707	260	41	32	P	W	34.233	0.353	-2.211	-3.093	-0.351	2.357	3.297	69.649	17.539	17.335
11708	260	41	32	P	W	52.846	-0.955	-1.282	-1.842	0.973	1.331	1.913	69.666	17.772	17.101
11709	260	41	32	P	W	53.822	-2.145	0.394	-0.644	2.241	-0.399	0.653	69.670	17.102	17.771
11710	260	41	32	P	W	66.178	-2.145	-0.394	-0.644	2.241	0.399	0.653	69.670	17.102	17.771
11711	260	41	32	P	W	67.154	-0.955	1.282	-1.842	0.973	-1.331	1.913	69.666	17.772	17.101
11712	260	41	32	P	W	84.791	-3.388	0.583	0.672	3.635	-0.575	-0.663	69.662	17.335	17.539
11713	260	41	32	P	W	94.233	0.353	-2.211	-3.093	-0.351	2.357	3.297	69.649	17.539	17.335
11714	260	41	32	P	W	112.846	-0.955	-1.282	-1.842	0.973	1.331	1.913	69.666	17.772	17.101
11715	260	41	32	P	W	113.822	-2.145	0.394	-0.644	2.241	-0.399	0.653	69.670	17.102	17.771
11716	261	39	35	C	S	8.948	-0.599	-0.883	4.975	0.606	0.803	-4.525	85.511	15.901	18.353
11717	261	39	35	C	S	27.126	1.029	-2.385	3.221	-1.008	2.241	-3.026	83.876	19.034	15.363
11718	261	39	35	C	S	30.000	2.934	-2.743	1.306	-2.772	2.673	-1.272	83.819	15.368	19.030
11719	261	39	35	C	S	32.874	1.029	2.385	3.221	-1.008	-2.241	-3.026	83.876	19.034	15.363
11720	261	39	35	C	S	51.052	-0.599	0.883	4.975	0.606	-0.803	-4.525	85.512	15.901	18.353
11721	261	39	35	C	S	87.126	1.029	-2.385	3.221	-1.008	2.241	-3.026	83.876	19.034	15.363
11722	261	39	35	C	S	90.000	2.934	-2.743	1.306	-2.772	2.673	-1.272	83.819	15.368	19.030
11723	261	39	35	C	S	92.874	1.029	2.385	3.221	-1.008	-2.241	-3.026	83.876	19.034	15.363
11724	261	39	35	C	S	111.052	-0.599	0.883	4.975	0.606	-0.803	-4.525	85.511	15.901	18.353
11725	261	39	35	C	W	27.126	1.029	-2.385	3.221	-1.008	2.241	-3.026	83.876	19.034	15.363
11726	261	39	35	C	W	30.000	2.934	-2.743	1.306	-2.772	2.673	-1.272	83.819	15.368	19.030
11727	261	39	35	C	W	32.874	1.029	2.385	3.221	-1.008	-2.241	-3.026	83.876	19.034	15.363
11728	261	39	35	C	W	68.948	-0.599	-0.883	4.975	0.606	0.803	-4.525	85.512	15.901	18.353
11729	261	39	35	C	W	87.126	1.029	-2.385	3.221	-1.008	2.241	-3.026	83.876	19.034	15.363
11730	261	39	35	C	W	90.000	2.934	-2.743	1.306	-2.772	2.673	-1.272	83.819	15.368	19.030

BL number	Atoms	γ-PC unit cells	WS_2 unit cells	γ-PC origin atom	WS_2 origin atom	Twist-angle (°)	γ-PC strain 1 (%)	γ-PC strain 2 (%)	γ-PC strain 3 (%)	WS_2 strain 1 (%)	WS_2 strain 2 (%)	WS_2 strain 3 (%)	γ (°)	|**a**|(Å)	|**b**|(Å)
11731	261	39	35	C	W	92.874	1.029	2.385	3.221	-1.008	-2.241	-3.026	83.876	19.034	15.363
11732	261	39	35	P	S	27.126	1.029	-2.385	3.221	-1.008	2.241	-3.026	83.876	19.034	15.363
11733	261	39	35	P	S	30.000	2.934	-2.743	1.306	-2.772	2.673	-1.272	83.819	15.368	19.030
11734	261	39	35	P	S	32.874	1.029	2.385	3.221	-1.008	-2.241	-3.026	83.876	19.034	15.363
11735	261	39	35	P	S	51.052	-0.599	0.883	4.975	0.606	-0.803	-4.525	85.512	15.901	18.353
11736	261	39	35	P	S	68.948	-0.599	-0.883	4.975	0.606	0.803	-4.525	85.512	15.901	18.353
11737	261	39	35	P	S	87.126	1.029	-2.385	3.221	-1.008	2.241	-3.026	83.876	19.034	15.363
11738	261	39	35	P	S	90.000	2.934	-2.743	1.306	-2.772	2.673	-1.272	83.819	15.368	19.030
11739	261	39	35	P	S	92.874	1.029	2.385	3.221	-1.008	-2.241	-3.026	83.876	19.034	15.363
11740	261	39	35	P	S	111.052	-0.599	0.883	4.975	0.606	-0.803	-4.525	85.511	15.901	18.353
11741	261	39	35	P	W	8.948	-0.599	-0.883	4.975	0.606	0.803	-4.525	85.511	15.901	18.353
11742	261	39	35	P	W	27.126	1.029	-2.385	3.221	-1.008	2.241	-3.026	83.876	19.034	15.363
11743	261	39	35	P	W	30.000	2.934	-2.743	1.306	-2.772	2.673	-1.272	83.819	15.368	19.030
11744	261	39	35	P	W	32.874	1.029	2.385	3.221	-1.008	-2.241	-3.026	83.876	19.034	15.363
11745	261	39	35	P	W	87.126	1.029	-2.385	3.221	-1.008	2.241	-3.026	83.876	19.034	15.363
11746	261	39	35	P	W	90.000	2.934	-2.743	1.306	-2.772	2.673	-1.272	83.819	15.368	19.030
11747	261	39	35	P	W	92.874	1.029	2.385	3.221	-1.008	-2.241	-3.026	83.876	19.034	15.363
11748	262	40	34	C	S	0.000	5.011	-3.031	-3.241	-4.554	3.241	3.465	86.415	16.799	17.331
11749	262	40	34	C	S	3.004	-3.388	2.241	5.185	3.635	-2.031	-4.698	86.667	17.335	16.792
11750	262	40	34	C	S	7.154	-0.955	-2.490	2.447	0.973	2.374	-2.333	86.600	17.772	16.356
11751	262	40	34	C	S	10.158	2.281	2.794	-0.800	-2.182	-2.840	0.813	86.502	16.362	17.766
11752	262	40	34	C	S	49.842	2.281	-2.794	-0.800	-2.182	2.840	0.813	86.502	16.362	17.766
11753	262	40	34	C	S	52.846	-0.955	2.490	2.447	0.973	-2.374	-2.333	86.600	17.772	16.356
11754	262	40	34	C	S	56.996	-3.388	-2.241	5.185	3.635	2.030	-4.698	86.667	17.335	16.792
11755	262	40	34	C	S	60.000	5.011	3.032	-3.241	-4.554	-3.242	3.465	86.415	16.799	17.331
11756	262	40	34	C	S	63.005	-3.388	2.241	5.185	3.635	-2.031	-4.698	86.667	17.335	16.792
11757	262	40	34	C	S	67.154	-0.955	-2.490	2.447	0.973	2.374	-2.333	86.599	17.772	16.356
11758	262	40	34	C	S	70.158	2.281	2.794	-0.800	-2.182	-2.840	0.813	86.502	16.362	17.766
11759	262	40	34	C	S	109.842	2.281	-2.794	-0.800	-2.182	2.840	0.813	86.502	16.362	17.766
11760	262	40	34	C	S	112.846	-0.955	2.490	2.447	0.973	-2.374	-2.333	86.600	17.772	16.356
11761	262	40	34	C	S	116.996	-3.388	-2.241	5.185	3.635	2.030	-4.698	86.667	17.335	16.792
11762	262	40	34	C	W	0.000	5.011	-3.031	-3.241	-4.554	3.241	3.465	86.415	16.799	17.331
11763	262	40	34	C	W	3.004	-3.388	2.241	5.185	3.635	-2.031	-4.698	86.667	17.335	16.792
11764	262	40	34	C	W	7.154	-0.955	-2.490	2.447	0.973	2.374	-2.333	86.600	17.772	16.356
11765	262	40	34	C	W	10.158	2.281	2.794	-0.800	-2.182	-2.840	0.813	86.502	16.362	17.766
11766	262	40	34	C	W	49.842	2.281	-2.794	-0.800	-2.182	2.840	0.813	86.502	16.362	17.766
11767	262	40	34	C	W	52.846	-0.955	2.490	2.447	0.973	-2.374	-2.333	86.600	17.772	16.356
11768	262	40	34	C	W	56.996	-3.388	-2.241	5.185	3.635	2.030	-4.698	86.667	17.335	16.792
11769	262	40	34	C	W	60.000	5.011	3.032	-3.241	-4.554	-3.242	3.465	86.415	16.799	17.331
11770	262	40	34	C	W	63.005	-3.388	2.241	5.185	3.635	-2.031	-4.698	86.667	17.335	16.792
11771	262	40	34	C	W	67.154	-0.955	-2.490	2.447	0.973	2.374	-2.333	86.599	17.772	16.356
11772	262	40	34	C	W	70.158	2.281	2.794	-0.800	-2.182	-2.840	0.813	86.502	16.362	17.766
11773	262	40	34	C	W	109.842	2.281	-2.794	-0.800	-2.182	2.840	0.813	86.502	16.362	17.766
11774	262	40	34	C	W	112.846	-0.955	2.490	2.447	0.973	-2.374	-2.333	86.600	17.772	16.356
11775	262	40	34	C	W	116.996	-3.388	-2.241	5.185	3.635	2.030	-4.698	86.667	17.335	16.792
11776	262	40	34	P	S	0.000	5.011	-3.031	-3.241	-4.554	3.241	3.465	86.415	16.799	17.331
11777	262	40	34	P	S	3.004	-3.388	2.241	5.185	3.635	-2.031	-4.698	86.667	17.335	16.792
11778	262	40	34	P	S	7.154	-0.955	-2.490	2.447	0.973	2.374	-2.333	86.600	17.772	16.356
11779	262	40	34	P	S	10.158	2.281	2.794	-0.800	-2.182	-2.840	0.813	86.502	16.362	17.766
11780	262	40	34	P	S	49.842	2.281	-2.794	-0.800	-2.182	2.840	0.813	86.502	16.362	17.766
11781	262	40	34	P	S	52.846	-0.955	2.490	2.447	0.973	-2.374	-2.333	86.600	17.772	16.356

BL number	Atoms	γ-PC unit cells	WS_2 unit cells	γ-PC origin atom	WS_2 origin atom	Twist-angle (°)	γ-PC strain 1 (%)	γ-PC strain 2 (%)	γ-PC strain 3 (%)	WS_2 strain 1 (%)	WS_2 strain 2 (%)	WS_2 strain 3 (%)	γ (°)	$\lvert\mathbf{a}\rvert$(Å)	$\lvert\mathbf{b}\rvert$(Å)
11782	262	40	34	P	S	56.996	-3.388	-2.241	5.185	3.635	2.030	-4.698	86.667	17.335	16.792
11783	262	40	34	P	S	60.000	5.011	3.032	-3.241	-4.554	-3.242	3.465	86.415	16.799	17.331
11784	262	40	34	P	S	63.005	-3.388	2.241	5.185	3.635	-2.031	-4.698	86.667	17.335	16.792
11785	262	40	34	P	S	67.154	-0.955	-2.490	2.447	0.973	2.374	-2.333	86.599	17.772	16.356
11786	262	40	34	P	S	70.158	2.281	2.794	-0.800	-2.182	-2.840	0.813	86.502	16.362	17.766
11787	262	40	34	P	S	109.842	2.281	-2.794	-0.800	-2.182	2.840	0.813	86.502	16.362	17.766
11788	262	40	34	P	S	112.846	-0.955	2.490	2.447	0.973	-2.374	-2.333	86.600	17.772	16.356
11789	262	40	34	P	S	116.996	-3.388	-2.241	5.185	3.635	2.030	-4.698	86.667	17.335	16.792
11790	262	40	34	P	W	0.000	5.011	-3.031	-3.241	-4.554	3.241	3.465	86.415	16.799	17.331
11791	262	40	34	P	W	3.004	-3.388	2.241	5.185	3.635	-2.031	-4.698	86.667	17.335	16.792
11792	262	40	34	P	W	7.154	-0.955	-2.490	2.447	0.973	2.374	-2.333	86.600	17.772	16.356
11793	262	40	34	P	W	10.158	2.281	2.794	-0.800	-2.182	-2.840	0.813	86.502	16.362	17.766
11794	262	40	34	P	W	49.842	2.281	-2.794	-0.800	-2.182	2.840	0.813	86.502	16.362	17.766
11795	262	40	34	P	W	52.846	-0.955	2.490	2.447	0.973	-2.374	-2.333	86.600	17.772	16.356
11796	262	40	34	P	W	56.996	-3.388	-2.241	5.185	3.635	2.030	-4.698	86.667	17.335	16.792
11797	262	40	34	P	W	60.000	5.011	3.032	-3.241	-4.554	-3.242	3.465	86.415	16.799	17.331
11798	262	40	34	P	W	63.005	-3.388	2.241	5.185	3.635	-2.031	-4.698	86.667	17.335	16.792
11799	262	40	34	P	W	67.154	-0.955	-2.490	2.447	0.973	2.374	-2.333	86.599	17.772	16.356
11800	262	40	34	P	W	70.158	2.281	2.794	-0.800	-2.182	-2.840	0.813	86.502	16.362	17.766
11801	262	40	34	P	W	109.842	2.281	-2.794	-0.800	-2.182	2.840	0.813	86.502	16.362	17.766
11802	262	40	34	P	W	112.846	-0.955	2.490	2.447	0.973	-2.374	-2.333	86.600	17.772	16.356
11803	262	40	34	P	W	116.996	-3.388	-2.241	5.185	3.635	2.030	-4.698	86.667	17.335	16.792
11804	262	43	30	C	S	24.791	-3.388	-0.695	-4.704	3.635	0.767	5.193	78.862	17.335	16.640
11805	262	43	30	C	S	34.715	-4.781	-0.128	-3.309	5.287	0.137	3.544	78.855	16.641	17.335
11806	262	43	30	C	S	84.791	-3.388	-0.695	-4.704	3.635	0.767	5.193	78.861	17.335	16.640
11807	262	43	30	C	S	94.715	-4.781	-0.128	-3.309	5.287	0.137	3.544	78.855	16.641	17.335
11808	262	43	30	C	W	24.791	-3.388	-0.695	-4.704	3.635	0.767	5.193	78.862	17.335	16.640
11809	262	43	30	C	W	34.715	-4.781	-0.128	-3.309	5.287	0.137	3.544	78.855	16.641	17.335
11810	262	43	30	C	W	84.791	-3.388	-0.695	-4.704	3.635	0.767	5.193	78.861	17.335	16.640
11811	262	43	30	C	W	94.715	-4.781	-0.128	-3.309	5.287	0.137	3.544	78.855	16.641	17.335
11812	262	43	30	P	S	24.791	-3.388	-0.695	-4.704	3.635	0.767	5.193	78.862	17.335	16.640
11813	262	43	30	P	S	34.715	-4.781	-0.128	-3.309	5.287	0.137	3.544	78.855	16.641	17.335
11814	262	43	30	P	S	84.791	-3.388	-0.695	-4.704	3.635	0.767	5.193	78.861	17.335	16.640
11815	262	43	30	P	S	94.715	-4.781	-0.128	-3.309	5.287	0.137	3.544	78.855	16.641	17.335
11816	262	43	30	P	W	24.791	-3.388	-0.695	-4.704	3.635	0.767	5.193	78.862	17.335	16.640
11817	262	43	30	P	W	34.715	-4.781	-0.128	-3.309	5.287	0.137	3.544	78.855	16.641	17.335
11818	262	43	30	P	W	84.791	-3.388	-0.695	-4.704	3.635	0.767	5.193	78.861	17.335	16.640
11819	262	43	30	P	W	94.715	-4.781	-0.128	-3.309	5.287	0.137	3.544	78.855	16.641	17.335
11820	263	41	33	C	S	9.183	3.582	1.064	-4.542	-3.342	-1.171	4.996	67.037	18.586	16.936
11821	263	41	33	C	S	16.537	-3.292	-3.075	2.147	3.523	2.948	-2.059	78.881	18.221	16.196
11822	263	41	33	C	S	21.052	1.339	4.757	-2.556	-1.304	-5.013	2.694	78.682	16.211	18.209
11823	263	41	33	C	S	38.948	1.339	-4.757	-2.556	-1.304	5.013	2.694	78.682	16.211	18.209
11824	263	41	33	C	S	43.463	-3.292	3.075	2.147	3.523	-2.948	-2.059	78.881	18.221	16.196
11825	263	41	33	C	S	50.817	3.582	-1.064	-4.542	-3.342	1.171	4.996	67.037	18.586	16.936
11826	263	41	33	C	S	69.183	3.582	1.064	-4.542	-3.342	-1.171	4.996	67.037	18.586	16.936
11827	263	41	33	C	S	76.537	-3.292	-3.075	2.147	3.523	2.948	-2.059	78.881	18.221	16.196
11828	263	41	33	C	S	81.052	1.339	4.757	-2.556	-1.304	-5.013	2.694	78.682	16.211	18.209
11829	263	41	33	C	S	98.948	1.339	-4.757	-2.556	-1.304	5.013	2.694	78.682	16.211	18.209
11830	263	41	33	C	S	103.463	-3.292	3.075	2.147	3.523	-2.948	-2.059	78.881	18.221	16.196
11831	263	41	33	C	S	110.817	3.582	-1.064	-4.542	-3.342	1.171	4.996	67.037	18.586	16.936
11832	263	41	33	C	W	9.183	3.582	1.064	-4.542	-3.342	-1.171	4.996	67.037	18.586	16.936

BL number	Atoms	γ-PC unit cells	WS_2 unit cells	γ-PC origin atom	WS_2 origin atom	Twist-angle (°)	γ-PC strain 1 (%)	γ-PC strain 2 (%)	γ-PC strain 3 (%)	WS_2 strain 1 (%)	WS_2 strain 2 (%)	WS_2 strain 3 (%)	γ (°)	|**a**|(Å)	|**b**|(Å)
11833	263	41	33	C	W	16.537	-3.292	-3.075	2.147	3.523	2.948	-2.059	78.881	18.221	16.196
11834	263	41	33	C	W	21.052	1.339	4.757	-2.556	-1.304	-5.013	2.694	78.682	16.211	18.209
11835	263	41	33	C	W	38.948	1.339	-4.757	-2.556	-1.304	5.013	2.694	78.682	16.211	18.209
11836	263	41	33	C	W	43.463	-3.292	3.075	2.147	3.523	-2.948	-2.059	78.881	18.221	16.196
11837	263	41	33	C	W	50.817	3.582	-1.064	-4.542	-3.342	1.171	4.996	67.037	18.586	16.936
11838	263	41	33	C	W	69.183	3.582	1.064	-4.542	-3.342	-1.171	4.996	67.037	18.586	16.936
11839	263	41	33	C	W	76.537	-3.292	-3.075	2.147	3.523	2.948	-2.059	78.881	18.221	16.196
11840	263	41	33	C	W	81.052	1.339	4.757	-2.556	-1.304	-5.013	2.694	78.682	16.211	18.209
11841	263	41	33	C	W	98.948	1.339	-4.757	-2.556	-1.304	5.013	2.694	78.682	16.211	18.209
11842	263	41	33	C	W	103.463	-3.292	3.075	2.147	3.523	-2.948	-2.059	78.881	18.221	16.196
11843	263	41	33	C	W	110.817	3.582	-1.064	-4.542	-3.342	1.171	4.996	67.037	18.586	16.936
11844	263	41	33	P	S	9.183	3.582	1.064	-4.542	-3.342	-1.171	4.996	67.037	18.586	16.936
11845	263	41	33	P	S	16.537	-3.292	-3.075	2.147	3.523	2.948	-2.059	78.881	18.221	16.196
11846	263	41	33	P	S	21.052	1.339	4.757	-2.556	-1.304	-5.013	2.694	78.682	16.211	18.209
11847	263	41	33	P	S	38.948	1.339	-4.757	-2.556	-1.304	5.013	2.694	78.682	16.211	18.209
11848	263	41	33	P	S	43.463	-3.292	3.075	2.147	3.523	-2.948	-2.059	78.881	18.221	16.196
11849	263	41	33	P	S	50.817	3.582	-1.064	-4.542	-3.342	1.171	4.996	67.037	18.586	16.936
11850	263	41	33	P	S	69.183	3.582	1.064	-4.542	-3.342	-1.171	4.996	67.037	18.586	16.936
11851	263	41	33	P	S	76.537	-3.292	-3.075	2.147	3.523	2.948	-2.059	78.881	18.221	16.196
11852	263	41	33	P	S	81.052	1.339	4.757	-2.556	-1.304	-5.013	2.694	78.682	16.211	18.209
11853	263	41	33	P	S	98.948	1.339	-4.757	-2.556	-1.304	5.013	2.694	78.682	16.211	18.209
11854	263	41	33	P	S	103.463	-3.292	3.075	2.147	3.523	-2.948	-2.059	78.881	18.221	16.196
11855	263	41	33	P	S	110.817	3.582	-1.064	-4.542	-3.342	1.171	4.996	67.037	18.586	16.936
11856	263	41	33	P	W	9.183	3.582	1.064	-4.542	-3.342	-1.171	4.996	67.037	18.586	16.936
11857	263	41	33	P	W	16.537	-3.292	-3.075	2.147	3.523	2.948	-2.059	78.881	18.221	16.196
11858	263	41	33	P	W	21.052	1.339	4.757	-2.556	-1.304	-5.013	2.694	78.682	16.211	18.209
11859	263	41	33	P	W	38.948	1.339	-4.757	-2.556	-1.304	5.013	2.694	78.682	16.211	18.209
11860	263	41	33	P	W	43.463	-3.292	3.075	2.147	3.523	-2.948	-2.059	78.881	18.221	16.196
11861	263	41	33	P	W	50.817	3.582	-1.064	-4.542	-3.342	1.171	4.996	67.037	18.586	16.936
11862	263	41	33	P	W	69.183	3.582	1.064	-4.542	-3.342	-1.171	4.996	67.037	18.586	16.936
11863	263	41	33	P	W	76.537	-3.292	-3.075	2.147	3.523	2.948	-2.059	78.881	18.221	16.196
11864	263	41	33	P	W	81.052	1.339	4.757	-2.556	-1.304	-5.013	2.694	78.682	16.211	18.209
11865	263	41	33	P	W	98.948	1.339	-4.757	-2.556	-1.304	5.013	2.694	78.682	16.211	18.209
11866	263	41	33	P	W	103.463	-3.292	3.075	2.147	3.523	-2.948	-2.059	78.881	18.221	16.196
11867	263	41	33	P	W	110.817	3.582	-1.064	-4.542	-3.342	1.171	4.996	67.037	18.586	16.936
11868	264	42	32	C	S	8.948	1.048	3.630	-4.833	-1.026	-4.018	5.350	73.255	17.420	17.310
11869	264	42	32	C	S	51.052	1.048	-3.629	-4.833	-1.026	4.018	5.350	73.255	17.420	17.310
11870	264	42	32	C	S	68.948	1.048	3.630	-4.833	-1.026	-4.018	5.350	73.255	17.420	17.310
11871	264	42	32	C	S	111.052	1.048	-3.629	-4.833	-1.026	4.018	5.350	73.255	17.420	17.310
11872	264	42	32	C	W	8.948	1.048	3.630	-4.833	-1.026	-4.018	5.350	73.255	17.420	17.310
11873	264	42	32	C	W	51.052	1.048	-3.629	-4.833	-1.026	4.018	5.350	73.255	17.420	17.310
11874	264	42	32	C	W	68.948	1.048	3.630	-4.833	-1.026	-4.018	5.350	73.255	17.420	17.310
11875	264	42	32	C	W	111.052	1.048	-3.629	-4.833	-1.026	4.018	5.350	73.255	17.420	17.310
11876	264	42	32	P	S	51.052	1.048	-3.629	-4.833	-1.026	4.018	5.350	73.255	17.420	17.310
11877	264	42	32	P	S	68.948	1.048	3.630	-4.833	-1.026	-4.018	5.350	73.255	17.420	17.310
11878	264	42	32	P	W	8.948	1.048	3.630	-4.833	-1.026	-4.018	5.350	73.255	17.420	17.310
11879	264	42	32	P	W	111.052	1.048	-3.629	-4.833	-1.026	4.018	5.350	73.255	17.420	17.310
11880	265	40	35	C	S	8.948	-0.599	0.144	3.600	0.606	-0.134	-3.359	85.171	15.901	18.586
11881	265	40	35	C	S	9.183	3.582	0.545	-0.582	-3.342	-0.552	0.588	85.156	18.586	15.902
11882	265	40	35	C	S	13.898	-0.414	-3.981	3.401	0.417	3.727	-3.184	73.557	19.824	15.488
11883	265	40	35	C	S	17.696	1.029	-1.320	1.891	-1.008	1.272	-1.822	85.984	19.034	15.503

BL number	Atoms	γ-PC unit cells	WS_2 unit cells	γ-PC origin atom	WS_2 origin atom	Twist-angle (°)	γ-PC strain 1 (%)	γ-PC strain 2 (%)	γ-PC strain 3 (%)	WS_2 strain 1 (%)	WS_2 strain 2 (%)	WS_2 strain 3 (%)	γ (°)	|**a**|(Å)	|**b**|(Å)
11884	265	40	35	C	S	19.107	1.980	1.178	0.941	-1.905	-1.156	-0.924	85.996	15.504	19.033
11885	265	40	35	C	S	40.893	1.980	-5.299	0.941	-1.905	5.201	-0.924	73.433	15.504	19.809
11886	265	40	35	C	S	46.102	-0.414	3.981	3.401	0.417	-3.727	-3.184	73.557	19.824	15.488
11887	265	40	35	C	S	50.817	3.582	-0.545	-0.582	-3.342	0.552	0.588	85.156	18.586	15.902
11888	265	40	35	C	S	51.052	-0.599	-0.143	3.600	0.606	0.134	-3.359	85.171	15.901	18.586
11889	265	40	35	C	S	68.948	-0.599	0.144	3.600	0.606	-0.134	-3.359	85.171	15.901	18.586
11890	265	40	35	C	S	69.183	3.582	0.545	-0.582	-3.342	-0.552	0.588	85.156	18.586	15.902
11891	265	40	35	C	S	73.898	-0.414	-3.981	3.401	0.417	3.727	-3.184	73.557	19.824	15.488
11892	265	40	35	C	S	77.696	1.029	-1.320	1.891	-1.008	1.272	-1.822	85.984	19.034	15.503
11893	265	40	35	C	S	79.107	1.980	5.299	0.941	-1.905	-5.201	-0.923	73.433	15.504	19.809
11894	265	40	35	C	S	100.893	1.980	-5.299	0.941	-1.905	5.201	-0.924	73.433	15.504	19.809
11895	265	40	35	C	S	102.304	1.029	1.320	1.891	-1.008	-1.272	-1.822	85.984	19.034	15.503
11896	265	40	35	C	S	106.102	-0.414	3.981	3.401	0.417	-3.727	-3.184	73.557	19.824	15.488
11897	265	40	35	C	S	110.817	3.582	-0.545	-0.582	-3.342	0.552	0.588	85.156	18.586	15.902
11898	265	40	35	C	S	111.052	-0.599	-0.143	3.600	0.606	0.134	-3.359	85.171	15.901	18.586
11899	265	40	35	C	W	8.948	-0.599	0.144	3.600	0.606	-0.134	-3.359	85.171	15.901	18.586
11900	265	40	35	C	W	9.183	3.582	0.545	-0.582	-3.342	-0.552	0.588	85.156	18.586	15.902
11901	265	40	35	C	W	13.898	-0.414	-3.981	3.401	0.417	3.727	-3.184	73.557	19.824	15.488
11902	265	40	35	C	W	17.696	1.029	-1.320	1.891	-1.008	1.272	-1.822	85.984	19.034	15.503
11903	265	40	35	C	W	19.107	1.980	5.299	0.941	-1.905	-5.201	-0.923	73.433	15.504	19.809
11904	265	40	35	C	W	40.893	1.980	-5.299	0.941	-1.905	5.201	-0.924	73.433	15.504	19.809
11905	265	40	35	C	W	42.304	1.029	1.320	1.891	-1.008	-1.272	-1.822	85.984	19.034	15.503
11906	265	40	35	C	W	46.102	-0.414	3.981	3.401	0.417	-3.727	-3.184	73.557	19.824	15.488
11907	265	40	35	C	W	50.817	3.582	-0.545	-0.582	-3.342	0.552	0.588	85.156	18.586	15.902
11908	265	40	35	C	W	51.052	-0.599	-0.143	3.600	0.606	0.134	-3.359	85.171	15.901	18.586
11909	265	40	35	C	W	68.948	-0.599	0.144	3.600	0.606	-0.134	-3.359	85.171	15.901	18.586
11910	265	40	35	C	W	69.183	3.582	0.545	-0.582	-3.342	-0.552	0.588	85.156	18.586	15.902
11911	265	40	35	C	W	73.898	-0.414	-3.981	3.401	0.417	3.727	-3.184	73.557	19.824	15.488
11912	265	40	35	C	W	77.696	1.029	-1.320	1.891	-1.008	1.272	-1.822	85.984	19.034	15.503
11913	265	40	35	C	W	79.107	1.980	1.178	0.941	-1.905	-1.156	-0.924	85.996	15.504	19.033
11914	265	40	35	C	W	100.893	1.980	-5.299	0.941	-1.905	5.201	-0.924	73.433	15.504	19.809
11915	265	40	35	C	W	106.102	-0.414	3.981	3.401	0.417	-3.727	-3.184	73.557	19.824	15.488
11916	265	40	35	C	W	110.817	3.582	-0.545	-0.582	-3.342	0.552	0.588	85.156	18.586	15.902
11917	265	40	35	C	W	111.052	-0.599	-0.143	3.600	0.606	0.134	-3.359	85.171	15.901	18.586
11918	265	40	35	P	S	8.948	-0.599	0.144	3.600	0.606	-0.134	-3.359	85.171	15.901	18.586
11919	265	40	35	P	S	9.183	3.582	0.545	-0.582	-3.342	-0.552	0.588	85.156	18.586	15.902
11920	265	40	35	P	S	17.696	1.029	-1.320	1.891	-1.008	1.272	-1.822	85.984	19.034	15.503
11921	265	40	35	P	S	40.893	1.980	-5.299	0.941	-1.905	5.201	-0.924	73.433	15.504	19.809
11922	265	40	35	P	S	42.304	1.029	1.320	1.891	-1.008	-1.272	-1.822	85.984	19.034	15.503
11923	265	40	35	P	S	46.102	-0.414	3.981	3.401	0.417	-3.727	-3.184	73.557	19.824	15.488
11924	265	40	35	P	S	50.817	3.582	-0.545	-0.582	-3.342	0.552	0.588	85.156	18.586	15.902
11925	265	40	35	P	S	51.052	-0.599	-0.143	3.600	0.606	0.134	-3.359	85.171	15.901	18.586
11926	265	40	35	P	S	68.948	-0.599	0.144	3.600	0.606	-0.134	-3.359	85.171	15.901	18.586
11927	265	40	35	P	S	69.183	3.582	0.545	-0.582	-3.342	-0.552	0.588	85.156	18.586	15.902
11928	265	40	35	P	S	73.898	-0.414	-3.981	3.401	0.417	3.727	-3.184	73.557	19.824	15.488
11929	265	40	35	P	S	77.696	1.029	-1.320	1.891	-1.008	1.272	-1.822	85.984	19.034	15.503
11930	265	40	35	P	S	79.107	1.980	1.178	0.941	-1.905	-1.156	-0.924	85.996	15.504	19.033
11931	265	40	35	P	S	100.893	1.980	-1.177	0.941	-1.905	1.156	-0.923	85.996	15.504	19.033
11932	265	40	35	P	S	110.817	3.582	-0.545	-0.582	-3.342	0.552	0.588	85.156	18.586	15.902
11933	265	40	35	P	S	111.052	-0.599	-0.143	3.600	0.606	0.134	-3.359	85.171	15.901	18.586
11934	265	40	35	P	W	8.948	-0.599	0.144	3.600	0.606	-0.134	-3.359	85.171	15.901	18.586

BL number	Atoms	γ-PC unit cells	WS_2 unit cells	γ-PC origin atom	WS_2 origin atom	Twist-angle (°)	γ-PC strain 1 (%)	γ-PC strain 2 (%)	γ-PC strain 3 (%)	WS_2 strain 1 (%)	WS_2 strain 2 (%)	WS_2 strain 3 (%)	γ (°)	|**a**|(Å)	|**b**|(Å)
11935	265	40	35	P	W	9.183	3.582	0.545	-0.582	-3.342	-0.552	0.588	85.156	18.586	15.902
11936	265	40	35	P	W	13.898	-0.414	-3.981	3.401	0.417	3.727	-3.184	73.557	19.824	15.488
11937	265	40	35	P	W	17.696	1.029	-1.320	1.891	-1.008	1.272	-1.822	85.984	19.034	15.503
11938	265	40	35	P	W	19.107	1.980	1.178	0.941	-1.905	-1.156	-0.924	85.996	15.504	19.033
11939	265	40	35	P	W	40.893	1.980	-1.177	0.941	-1.905	1.156	-0.923	85.996	15.504	19.033
11940	265	40	35	P	W	50.817	3.582	-0.545	-0.582	-3.342	0.552	0.588	85.156	18.586	15.902
11941	265	40	35	P	W	51.052	-0.599	-0.143	3.600	0.606	0.134	-3.359	85.171	15.901	18.586
11942	265	40	35	P	W	68.948	-0.599	0.144	3.600	0.606	-0.134	-3.359	85.171	15.901	18.586
11943	265	40	35	P	W	69.183	3.582	0.545	-0.582	-3.342	-0.552	0.588	85.156	18.586	15.902
11944	265	40	35	P	W	77.696	1.029	-1.320	1.891	-1.008	1.272	-1.822	85.984	19.034	15.503
11945	265	40	35	P	W	100.893	1.980	-5.299	0.941	-1.905	5.201	-0.924	73.433	15.504	19.809
11946	265	40	35	P	W	102.304	1.029	1.320	1.891	-1.008	-1.272	-1.822	85.984	19.034	15.503
11947	265	40	35	P	W	106.102	-0.414	3.981	3.401	0.417	-3.727	-3.184	73.557	19.824	15.488
11948	265	40	35	P	W	110.817	3.582	-0.545	-0.582	-3.342	0.552	0.588	85.156	18.586	15.902
11949	265	40	35	P	W	111.052	-0.599	-0.143	3.600	0.606	0.134	-3.359	85.171	15.901	18.586
11950	265	43	31	C	S	1.359	-3.292	0.000	-3.292	3.523	0.000	3.524	60.000	18.221	18.221
11951	265	43	31	C	S	16.537	-3.292	0.000	-3.292	3.523	0.000	3.524	60.000	18.221	18.221
11952	265	43	31	C	S	43.463	-3.292	0.000	-3.292	3.523	0.000	3.524	60.000	18.221	18.221
11953	265	43	31	C	S	58.641	-3.292	0.000	-3.292	3.523	0.000	3.524	60.000	18.221	18.221
11954	265	43	31	C	S	61.359	-3.292	0.000	-3.292	3.523	0.000	3.524	60.000	18.221	18.221
11955	265	43	31	C	S	76.537	-3.292	0.000	-3.292	3.523	0.000	3.524	60.000	18.221	18.221
11956	265	43	31	C	S	103.463	-3.292	0.000	-3.292	3.523	0.000	3.524	60.000	18.221	18.221
11957	265	43	31	C	S	118.641	-3.292	0.000	-3.292	3.523	0.000	3.524	60.000	18.221	18.221
11958	265	43	31	C	W	1.359	-3.292	0.000	-3.292	3.523	0.000	3.524	60.000	18.221	18.221
11959	265	43	31	C	W	16.537	-3.292	0.000	-3.292	3.523	0.000	3.524	60.000	18.221	18.221
11960	265	43	31	C	W	43.463	-3.292	0.000	-3.292	3.523	0.000	3.524	60.000	18.221	18.221
11961	265	43	31	C	W	58.641	-3.292	0.000	-3.292	3.523	0.000	3.524	60.000	18.221	18.221
11962	265	43	31	C	W	61.359	-3.292	0.000	-3.292	3.523	0.000	3.524	60.000	18.221	18.221
11963	265	43	31	C	W	76.537	-3.292	0.000	-3.292	3.523	0.000	3.524	60.000	18.221	18.221
11964	265	43	31	C	W	103.463	-3.292	0.000	-3.292	3.523	0.000	3.524	60.000	18.221	18.221
11965	265	43	31	C	W	118.641	-3.292	0.000	-3.292	3.523	0.000	3.524	60.000	18.221	18.221
11966	265	43	31	P	S	43.463	-3.292	0.000	-3.292	3.523	0.000	3.524	60.000	18.221	18.221
11967	265	43	31	P	S	58.641	-3.292	0.000	-3.292	3.523	0.000	3.524	60.000	18.221	18.221
11968	265	43	31	P	S	61.359	-3.292	0.000	-3.292	3.523	0.000	3.524	60.000	18.221	18.221
11969	265	43	31	P	S	76.537	-3.292	0.000	-3.292	3.523	0.000	3.524	60.000	18.221	18.221
11970	265	43	31	P	W	1.359	-3.292	0.000	-3.292	3.523	0.000	3.524	60.000	18.221	18.221
11971	265	43	31	P	W	16.537	-3.292	0.000	-3.292	3.523	0.000	3.524	60.000	18.221	18.221
11972	265	43	31	P	W	103.463	-3.292	0.000	-3.292	3.523	0.000	3.524	60.000	18.221	18.221
11973	265	43	31	P	W	118.641	-3.292	0.000	-3.292	3.523	0.000	3.524	60.000	18.221	18.221
11974	266	41	34	C	S	0.000	5.011	0.739	-4.381	-4.554	-0.810	4.802	67.715	18.353	17.331
11975	266	41	34	C	S	15.608	-2.145	-5.069	2.440	2.241	4.833	-2.327	67.576	17.102	18.586
11976	266	41	34	C	S	18.613	3.582	0.296	-3.164	-3.342	-0.316	3.378	67.719	18.586	17.095
11977	266	41	34	C	S	41.387	3.582	-0.296	-3.164	-3.342	0.316	3.378	67.719	18.586	17.095
11978	266	41	34	C	S	44.392	-2.145	5.069	2.440	2.241	-4.833	-2.327	67.576	17.102	18.586
11979	266	41	34	C	S	60.000	5.011	2.218	-4.381	-4.554	-2.431	4.802	66.215	18.842	17.070
11980	266	41	34	C	S	75.609	-2.145	-5.069	2.440	2.241	4.833	-2.327	67.576	17.102	18.586
11981	266	41	34	C	S	78.613	3.582	0.296	-3.164	-3.342	-0.316	3.378	67.719	18.586	17.095
11982	266	41	34	C	S	101.387	3.582	-0.296	-3.164	-3.342	0.316	3.378	67.719	18.586	17.095
11983	266	41	34	C	S	104.392	-2.145	5.069	2.440	2.241	-4.833	-2.327	67.576	17.102	18.586
11984	266	41	34	C	W	0.000	5.011	0.739	-4.381	-4.554	-0.810	4.802	67.715	18.353	17.331
11985	266	41	34	C	W	15.608	-2.145	-5.069	2.440	2.241	4.833	-2.327	67.576	17.102	18.586

BL number	Atoms	γ-PC unit cells	WS_2 unit cells	γ-PC origin atom	WS_2 origin atom	Twist-angle (°)	γ-PC strain 1 (%)	γ-PC strain 2 (%)	γ-PC strain 3 (%)	WS_2 strain 1 (%)	WS_2 strain 2 (%)	WS_2 strain 3 (%)	γ (°)	|**a**|(Å)	|**b**|(Å)
11986	266	41	34	C	W	18.613	3.582	0.296	-3.164	-3.342	-0.316	3.378	67.719	18.586	17.095
11987	266	41	34	C	W	41.387	3.582	-0.296	-3.164	-3.342	0.316	3.378	67.719	18.586	17.095
11988	266	41	34	C	W	44.392	-2.145	5.069	2.440	2.241	-4.833	-2.327	67.576	17.102	18.586
11989	266	41	34	C	W	60.000	5.011	2.218	-4.381	-4.554	-2.431	4.802	66.215	18.842	17.070
11990	266	41	34	C	W	75.609	-2.145	-5.069	2.440	2.241	4.833	-2.327	67.576	17.102	18.586
11991	266	41	34	C	W	78.613	3.582	0.296	-3.164	-3.342	-0.316	3.378	67.719	18.586	17.095
11992	266	41	34	C	W	101.387	3.582	-0.296	-3.164	-3.342	0.316	3.378	67.719	18.586	17.095
11993	266	41	34	C	W	104.392	-2.145	5.069	2.440	2.241	-4.833	-2.327	67.576	17.102	18.586
11994	266	41	34	P	S	0.000	5.011	0.739	-4.381	-4.554	-0.810	4.802	67.715	18.353	17.331
11995	266	41	34	P	S	15.608	-2.145	-5.069	2.440	2.241	4.833	-2.327	67.576	17.102	18.586
11996	266	41	34	P	S	18.613	3.582	0.296	-3.164	-3.342	-0.316	3.378	67.719	18.586	17.095
11997	266	41	34	P	S	41.387	3.582	-0.296	-3.164	-3.342	0.316	3.378	67.719	18.586	17.095
11998	266	41	34	P	S	44.392	-2.145	5.069	2.440	2.241	-4.833	-2.327	67.576	17.102	18.586
11999	266	41	34	P	S	60.000	5.011	0.739	-4.381	-4.554	-0.810	4.802	67.715	18.353	17.331
12000	266	41	34	P	S	75.609	-2.145	-5.069	2.440	2.241	4.833	-2.327	67.576	17.102	18.586
12001	266	41	34	P	S	78.613	3.582	0.296	-3.164	-3.342	-0.316	3.378	67.719	18.586	17.095
12002	266	41	34	P	S	101.387	3.582	-0.296	-3.164	-3.342	0.316	3.378	67.719	18.586	17.095
12003	266	41	34	P	S	104.392	-2.145	5.069	2.440	2.241	-4.833	-2.327	67.576	17.102	18.586
12004	266	41	34	P	W	0.000	5.011	0.739	-4.381	-4.554	-0.810	4.802	67.715	18.353	17.331
12005	266	41	34	P	W	15.608	-2.145	-5.069	2.440	2.241	4.833	-2.327	67.576	17.102	18.586
12006	266	41	34	P	W	18.613	3.582	0.296	-3.164	-3.342	-0.316	3.378	67.719	18.586	17.095
12007	266	41	34	P	W	41.387	3.582	-0.296	-3.164	-3.342	0.316	3.378	67.719	18.586	17.095
12008	266	41	34	P	W	44.392	-2.145	5.069	2.440	2.241	-4.833	-2.327	67.576	17.102	18.586
12009	266	41	34	P	W	60.000	5.011	2.218	-4.381	-4.554	-2.431	4.802	66.215	18.842	17.070
12010	266	41	34	P	W	75.609	-2.145	-5.069	2.440	2.241	4.833	-2.327	67.576	17.102	18.586
12011	266	41	34	P	W	78.613	3.582	0.296	-3.164	-3.342	-0.316	3.378	67.719	18.586	17.095
12012	266	41	34	P	W	101.387	3.582	-0.296	-3.164	-3.342	0.316	3.378	67.719	18.586	17.095
12013	266	41	34	P	W	104.392	-2.145	5.069	2.440	2.241	-4.833	-2.327	67.576	17.102	18.586
12014	267	39	37	C	S	9.183	3.582	0.000	3.582	-3.342	0.000	-3.342	60.000	18.586	18.586
12015	267	39	37	C	S	18.613	3.582	0.000	3.582	-3.342	0.000	-3.342	60.000	18.586	18.586
12016	267	39	37	C	S	41.387	3.582	0.000	3.582	-3.342	0.000	-3.342	60.000	18.586	18.586
12017	267	39	37	C	S	50.817	3.582	0.000	3.582	-3.342	0.000	-3.342	60.000	18.586	18.586
12018	267	39	37	C	S	69.183	3.582	0.000	3.582	-3.342	0.000	-3.342	60.000	18.586	18.586
12019	267	39	37	C	S	78.613	3.582	0.000	3.582	-3.342	0.000	-3.342	60.000	18.586	18.586
12020	267	39	37	C	S	101.387	3.582	0.000	3.582	-3.342	0.000	-3.342	60.000	18.586	18.586
12021	267	39	37	C	S	110.817	3.582	0.000	3.582	-3.342	0.000	-3.342	60.000	18.586	18.586
12022	267	39	37	P	S	9.183	3.582	0.000	3.582	-3.342	0.000	-3.342	60.000	18.586	18.586
12023	267	39	37	P	S	18.613	3.582	0.000	3.582	-3.342	0.000	-3.342	60.000	18.586	18.586
12024	267	39	37	P	S	41.387	3.582	0.000	3.582	-3.342	0.000	-3.342	60.000	18.586	18.586
12025	267	39	37	P	S	50.817	3.582	0.000	3.582	-3.342	0.000	-3.342	60.000	18.586	18.586
12026	267	39	37	P	S	69.183	3.582	0.000	3.582	-3.342	0.000	-3.342	60.000	18.586	18.586
12027	267	39	37	P	S	78.613	3.582	0.000	3.582	-3.342	0.000	-3.342	60.000	18.586	18.586
12028	267	39	37	P	S	101.387	3.582	0.000	3.582	-3.342	0.000	-3.342	60.000	18.586	18.586
12029	267	39	37	P	S	110.817	3.582	0.000	3.582	-3.342	0.000	-3.342	60.000	18.586	18.586
12030	267	42	33	C	S	4.715	-3.007	-4.222	0.598	3.200	4.172	-0.591	83.073	16.951	17.411
12031	267	42	33	C	S	8.948	1.048	3.092	-3.422	-1.026	-3.319	3.673	83.253	17.420	16.939
12032	267	42	33	C	S	13.898	-4.228	3.291	1.947	4.619	-3.168	-1.874	78.681	17.184	17.399
12033	267	42	33	C	S	46.102	-4.228	-3.291	1.947	4.619	3.168	-1.874	78.681	17.184	17.399
12034	267	42	33	C	S	51.052	1.048	-3.092	-3.422	-1.026	3.319	3.673	83.253	17.420	16.939
12035	267	42	33	C	S	55.285	-3.007	4.222	0.598	3.200	-4.172	-0.591	83.073	16.951	17.411
12036	267	42	33	C	S	64.715	-3.007	-4.222	0.598	3.200	4.172	-0.591	83.073	16.951	17.411

BL number	Atoms	γ-PC unit cells	WS_2 unit cells	γ-PC origin atom	WS_2 origin atom	Twist-angle (°)	γ-PC strain 1 (%)	γ-PC strain 2 (%)	γ-PC strain 3 (%)	WS_2 strain 1 (%)	WS_2 strain 2 (%)	WS_2 strain 3 (%)	γ (°)	|**a**|(Å)	|**b**|(Å)
12037	267	42	33	C	S	68.948	1.048	3.092	-3.422	-1.026	-3.319	3.673	83.253	17.420	16.939
12038	267	42	33	C	S	73.898	-4.228	3.291	1.947	4.619	-3.168	-1.874	78.681	17.184	17.399
12039	267	42	33	C	S	106.102	-4.228	-3.291	1.947	4.619	3.168	-1.874	78.681	17.184	17.399
12040	267	42	33	C	S	111.052	1.048	-3.092	-3.422	-1.026	3.319	3.673	83.253	17.420	16.939
12041	267	42	33	C	S	115.285	-3.007	4.222	0.598	3.200	-4.172	-0.591	83.073	16.951	17.411
12042	267	42	33	C	W	4.715	-3.007	-4.222	0.598	3.200	4.172	-0.591	83.073	16.951	17.411
12043	267	42	33	C	W	8.948	1.048	3.092	-3.422	-1.026	-3.319	3.673	83.253	17.420	16.939
12044	267	42	33	C	W	13.898	-4.228	3.291	1.947	4.619	-3.168	-1.874	78.681	17.184	17.399
12045	267	42	33	C	W	46.102	-4.228	-3.291	1.947	4.619	3.168	-1.874	78.681	17.184	17.399
12046	267	42	33	C	W	51.052	1.048	-3.092	-3.422	-1.026	3.319	3.673	83.253	17.420	16.939
12047	267	42	33	C	W	55.285	-3.007	4.222	0.598	3.200	-4.172	-0.591	83.073	16.951	17.411
12048	267	42	33	C	W	64.715	-3.007	-4.222	0.598	3.200	4.172	-0.591	83.073	16.951	17.411
12049	267	42	33	C	W	68.948	1.048	3.092	-3.422	-1.026	-3.319	3.673	83.253	17.420	16.939
12050	267	42	33	C	W	73.898	-4.228	3.291	1.947	4.619	-3.168	-1.874	78.681	17.184	17.399
12051	267	42	33	C	W	106.102	-4.228	-3.291	1.947	4.619	3.168	-1.874	78.681	17.184	17.399
12052	267	42	33	C	W	111.052	1.048	-3.092	-3.422	-1.026	3.319	3.673	83.253	17.420	16.939
12053	267	42	33	C	W	115.285	-3.007	4.222	0.598	3.200	-4.172	-0.591	83.073	16.951	17.411
12054	267	42	33	P	S	4.715	-3.007	-4.222	0.598	3.200	4.172	-0.591	83.073	16.951	17.411
12055	267	42	33	P	S	8.948	1.048	3.092	-3.422	-1.026	-3.319	3.673	83.253	17.420	16.939
12056	267	42	33	P	S	51.052	1.048	-3.092	-3.422	-1.026	3.319	3.673	83.253	17.420	16.939
12057	267	42	33	P	S	55.285	-3.007	4.222	0.598	3.200	-4.172	-0.591	83.073	16.951	17.411
12058	267	42	33	P	S	64.715	-3.007	-4.222	0.598	3.200	4.172	-0.591	83.073	16.951	17.411
12059	267	42	33	P	S	68.948	1.048	3.092	-3.422	-1.026	-3.319	3.673	83.253	17.420	16.939
12060	267	42	33	P	S	111.052	1.048	-3.092	-3.422	-1.026	3.319	3.673	83.253	17.420	16.939
12061	267	42	33	P	S	115.285	-3.007	4.222	0.598	3.200	-4.172	-0.591	83.073	16.951	17.411
12062	267	42	33	P	W	4.715	-3.007	-4.222	0.598	3.200	4.172	-0.591	83.073	16.951	17.411
12063	267	42	33	P	W	8.948	1.048	3.092	-3.422	-1.026	-3.319	3.673	83.253	17.420	16.939
12064	267	42	33	P	W	51.052	1.048	-3.092	-3.422	-1.026	3.319	3.673	83.253	17.420	16.939
12065	267	42	33	P	W	55.285	-3.007	4.222	0.598	3.200	-4.172	-0.591	83.073	16.951	17.411
12066	267	42	33	P	W	64.715	-3.007	-4.222	0.598	3.200	4.172	-0.591	83.073	16.951	17.411
12067	267	42	33	P	W	68.948	1.048	3.092	-3.422	-1.026	-3.319	3.673	83.253	17.420	16.939
12068	267	42	33	P	W	111.052	1.048	-3.092	-3.422	-1.026	3.319	3.673	83.253	17.420	16.939
12069	267	42	33	P	W	115.285	-3.007	4.222	0.598	3.200	-4.172	-0.591	83.073	16.951	17.411
12070	268	40	36	C	S	7.589	0.334	-2.896	4.110	-0.332	2.676	-3.798	88.406	18.904	15.808
12071	268	40	36	C	S	10.893	4.020	3.003	0.418	-3.721	-2.978	-0.415	88.287	15.815	18.896
12072	268	40	36	C	S	16.102	2.853	-4.454	1.532	-2.699	4.321	-1.486	87.488	18.455	16.197
12073	268	40	36	C	S	21.052	1.339	4.388	3.051	-1.304	-4.136	-2.875	87.562	16.211	18.438
12074	268	40	36	C	S	25.285	4.262	1.003	0.193	-3.928	-0.999	-0.192	69.168	18.222	17.541
12075	268	40	36	C	S	25.767	0.353	1.916	4.089	-0.351	-1.771	-3.780	69.183	17.539	18.221
12076	268	40	36	C	S	34.233	0.353	-1.916	4.089	-0.351	1.771	-3.780	69.183	17.539	18.221
12077	268	40	36	C	S	34.715	4.262	-1.003	0.193	-3.928	0.999	-0.192	69.168	18.222	17.541
12078	268	40	36	C	S	38.948	1.339	-4.388	3.051	-1.304	4.136	-2.875	87.562	16.211	18.438
12079	268	40	36	C	S	43.898	2.853	4.454	1.532	-2.699	-4.321	-1.486	87.488	18.455	16.197
12080	268	40	36	C	S	49.107	4.020	-3.003	0.418	-3.721	2.978	-0.415	88.287	15.815	18.897
12081	268	40	36	C	S	52.411	0.334	2.896	4.110	-0.332	-2.676	-3.798	88.406	18.904	15.808
12082	268	40	36	C	S	67.589	0.334	-2.896	4.110	-0.332	2.676	-3.798	88.406	18.904	15.808
12083	268	40	36	C	S	70.893	4.020	3.003	0.418	-3.721	-2.978	-0.415	88.287	15.815	18.897
12084	268	40	36	C	S	76.102	2.853	-4.454	1.532	-2.699	4.321	-1.486	87.488	18.455	16.197
12085	268	40	36	C	S	81.052	1.339	4.388	3.051	-1.304	-4.136	-2.875	87.562	16.211	18.438
12086	268	40	36	C	S	85.285	4.262	1.003	0.193	-3.928	-0.999	-0.192	69.168	18.222	17.541
12087	268	40	36	C	S	85.767	0.353	1.916	4.089	-0.351	-1.771	-3.780	69.183	17.539	18.221

BL number	Atoms	γ-PC unit cells	WS_2 unit cells	γ-PC origin atom	WS_2 origin atom	Twist-angle (°)	γ-PC strain 1 (%)	γ-PC strain 2 (%)	γ-PC strain 3 (%)	WS_2 strain 1 (%)	WS_2 strain 2 (%)	WS_2 strain 3 (%)	γ (°)	$\lvert\mathbf{a}\rvert$(Å)	$\lvert\mathbf{b}\rvert$(Å)
12088	268	40	36	C	S	94.233	0.353	-1.916	4.089	-0.351	1.771	-3.780	69.183	17.539	18.221
12089	268	40	36	C	S	94.715	4.262	-1.003	0.193	-3.928	0.999	-0.192	69.168	18.222	17.541
12090	268	40	36	C	S	98.948	1.339	-4.388	3.051	-1.304	4.136	-2.875	87.562	16.211	18.438
12091	268	40	36	C	S	103.898	2.853	4.454	1.532	-2.699	-4.321	-1.486	87.488	18.455	16.197
12092	268	40	36	C	S	109.107	4.020	-3.003	0.418	-3.721	2.978	-0.415	88.287	15.815	18.896
12093	268	40	36	C	S	112.411	0.334	2.896	4.110	-0.332	-2.676	-3.798	88.406	18.904	15.808
12094	268	40	36	C	W	7.589	0.334	-2.896	4.110	-0.332	2.676	-3.798	88.406	18.904	15.808
12095	268	40	36	C	W	10.893	4.020	3.003	0.418	-3.721	-2.978	-0.415	88.287	15.815	18.896
12096	268	40	36	C	W	16.102	2.853	-4.454	1.532	-2.699	4.321	-1.486	87.488	18.455	16.197
12097	268	40	36	C	W	21.052	1.339	4.388	3.051	-1.304	-4.136	-2.875	87.562	16.211	18.438
12098	268	40	36	C	W	25.285	4.262	1.003	0.193	-3.928	-0.999	-0.192	69.168	18.222	17.541
12099	268	40	36	C	W	25.767	0.353	1.916	4.089	-0.351	-1.771	-3.780	69.183	17.539	18.221
12100	268	40	36	C	W	34.233	0.353	-1.916	4.089	-0.351	1.771	-3.780	69.183	17.539	18.221
12101	268	40	36	C	W	34.715	4.262	-1.003	0.193	-3.928	0.999	-0.192	69.168	18.222	17.541
12102	268	40	36	C	W	38.948	1.339	-4.388	3.051	-1.304	4.136	-2.875	87.562	16.211	18.438
12103	268	40	36	C	W	43.898	2.853	4.454	1.532	-2.699	-4.321	-1.486	87.488	18.455	16.197
12104	268	40	36	C	W	49.107	4.020	-3.003	0.418	-3.721	2.978	-0.415	88.287	15.815	18.897
12105	268	40	36	C	W	52.411	0.334	2.896	4.110	-0.332	-2.676	-3.798	88.406	18.904	15.808
12106	268	40	36	C	W	67.589	0.334	-2.896	4.110	-0.332	2.676	-3.798	88.406	18.904	15.808
12107	268	40	36	C	W	70.893	4.020	3.003	0.418	-3.721	-2.978	-0.415	88.287	15.815	18.897
12108	268	40	36	C	W	76.102	2.853	-4.454	1.532	-2.699	4.321	-1.486	87.488	18.455	16.197
12109	268	40	36	C	W	81.052	1.339	4.388	3.051	-1.304	-4.136	-2.875	87.562	16.211	18.438
12110	268	40	36	C	W	85.285	4.262	1.003	0.193	-3.928	-0.999	-0.192	69.168	18.222	17.541
12111	268	40	36	C	W	85.767	0.353	1.916	4.089	-0.351	-1.771	-3.780	69.183	17.539	18.221
12112	268	40	36	C	W	94.233	0.353	-1.916	4.089	-0.351	1.771	-3.780	69.183	17.539	18.221
12113	268	40	36	C	W	94.715	4.262	-1.003	0.193	-3.928	0.999	-0.192	69.168	18.222	17.541
12114	268	40	36	C	W	98.948	1.339	-4.388	3.051	-1.304	4.136	-2.875	87.562	16.211	18.438
12115	268	40	36	C	W	103.898	2.853	4.454	1.532	-2.699	-4.321	-1.486	87.488	18.455	16.197
12116	268	40	36	C	W	109.107	4.020	-3.003	0.418	-3.721	2.978	-0.415	88.287	15.815	18.896
12117	268	40	36	C	W	112.411	0.334	2.896	4.110	-0.332	-2.676	-3.798	88.406	18.904	15.808
12118	268	40	36	P	S	25.285	4.262	1.003	0.193	-3.928	-0.999	-0.192	69.168	18.222	17.541
12119	268	40	36	P	S	25.767	0.353	1.916	4.089	-0.351	-1.771	-3.780	69.183	17.539	18.221
12120	268	40	36	P	S	34.233	0.353	-1.916	4.089	-0.351	1.771	-3.780	69.183	17.539	18.221
12121	268	40	36	P	S	34.715	4.262	-1.003	0.193	-3.928	0.999	-0.192	69.168	18.222	17.541
12122	268	40	36	P	S	85.285	4.262	1.003	0.193	-3.928	-0.999	-0.192	69.168	18.222	17.541
12123	268	40	36	P	S	85.767	0.353	1.916	4.089	-0.351	-1.771	-3.780	69.183	17.539	18.221
12124	268	40	36	P	S	94.233	0.353	-1.916	4.089	-0.351	1.771	-3.780	69.183	17.539	18.221
12125	268	40	36	P	S	94.715	4.262	-1.003	0.193	-3.928	0.999	-0.192	69.168	18.222	17.541
12126	268	40	36	P	S	112.411	0.334	2.896	4.110	-0.332	-2.676	-3.798	88.406	18.904	15.808
12127	268	40	36	P	W	25.285	4.262	1.003	0.193	-3.928	-0.999	-0.192	69.168	18.222	17.541
12128	268	40	36	P	W	25.767	0.353	1.916	4.089	-0.351	-1.771	-3.780	69.183	17.539	18.221
12129	268	40	36	P	W	34.233	0.353	-1.916	4.089	-0.351	1.771	-3.780	69.183	17.539	18.221
12130	268	40	36	P	W	34.715	4.262	-1.003	0.193	-3.928	0.999	-0.192	69.168	18.222	17.541
12131	268	40	36	P	W	85.285	4.262	1.003	0.193	-3.928	-0.999	-0.192	69.168	18.222	17.541
12132	268	40	36	P	W	85.767	0.353	1.916	4.089	-0.351	-1.771	-3.780	69.183	17.539	18.221
12133	268	40	36	P	W	94.233	0.353	-1.916	4.089	-0.351	1.771	-3.780	69.183	17.539	18.221
12134	268	40	36	P	W	94.715	4.262	-1.003	0.193	-3.928	0.999	-0.192	69.168	18.222	17.541
12135	268	43	32	C	S	3.004	-3.388	-2.502	-1.685	3.635	2.589	1.744	80.215	17.335	17.093
12136	268	43	32	C	S	6.178	-2.145	2.909	-2.940	2.241	-3.091	3.124	80.181	17.102	17.326
12137	268	43	32	C	S	53.822	-2.145	-2.909	-2.940	2.241	3.091	3.124	80.181	17.102	17.326
12138	268	43	32	C	S	56.996	-3.388	2.502	-1.685	3.635	-2.589	1.744	80.215	17.335	17.093

BL number	Atoms	γ-PC unit cells	WS_2 unit cells	γ-PC origin atom	WS_2 origin atom	Twist-angle (°)	γ-PC strain 1 (%)	γ-PC strain 2 (%)	γ-PC strain 3 (%)	WS_2 strain 1 (%)	WS_2 strain 2 (%)	WS_2 strain 3 (%)	γ (°)	|**a**|(Å)	|**b**|(Å)
12139	268	43	32	C	S	63.005	-3.388	-2.502	-1.685	3.635	2.589	1.744	80.215	17.335	17.093
12140	268	43	32	C	S	66.178	-2.145	2.909	-2.940	2.241	-3.091	3.124	80.181	17.102	17.326
12141	268	43	32	C	S	113.822	-2.145	-2.909	-2.940	2.241	3.091	3.124	80.181	17.102	17.326
12142	268	43	32	C	S	116.996	-3.388	2.502	-1.685	3.635	-2.589	1.744	80.215	17.335	17.093
12143	268	43	32	C	W	3.004	-3.388	-2.502	-1.685	3.635	2.589	1.744	80.215	17.335	17.093
12144	268	43	32	C	W	6.178	-2.145	2.909	-2.940	2.241	-3.091	3.124	80.181	17.102	17.326
12145	268	43	32	C	W	53.822	-2.145	-2.909	-2.940	2.241	3.091	3.124	80.181	17.102	17.326
12146	268	43	32	C	W	56.996	-3.388	2.502	-1.685	3.635	-2.589	1.744	80.215	17.335	17.093
12147	268	43	32	C	W	63.005	-3.388	-2.502	-1.685	3.635	2.589	1.744	80.215	17.335	17.093
12148	268	43	32	C	W	66.178	-2.145	2.909	-2.940	2.241	-3.091	3.124	80.181	17.102	17.326
12149	268	43	32	C	W	113.822	-2.145	-2.909	-2.940	2.241	3.091	3.124	80.181	17.102	17.326
12150	268	43	32	C	W	116.996	-3.388	2.502	-1.685	3.635	-2.589	1.744	80.215	17.335	17.093
12151	268	43	32	P	S	3.004	-3.388	-2.502	-1.685	3.635	2.589	1.744	80.215	17.335	17.093
12152	268	43	32	P	S	6.178	-2.145	2.909	-2.940	2.241	-3.091	3.124	80.181	17.102	17.326
12153	268	43	32	P	S	53.822	-2.145	-2.909	-2.940	2.241	3.091	3.124	80.181	17.102	17.326
12154	268	43	32	P	S	56.996	-3.388	2.502	-1.685	3.635	-2.589	1.744	80.215	17.335	17.093
12155	268	43	32	P	S	63.005	-3.388	-2.502	-1.685	3.635	2.589	1.744	80.215	17.335	17.093
12156	268	43	32	P	S	66.178	-2.145	2.909	-2.940	2.241	-3.091	3.124	80.181	17.102	17.326
12157	268	43	32	P	S	113.822	-2.145	-2.909	-2.940	2.241	3.091	3.124	80.181	17.102	17.326
12158	268	43	32	P	S	116.996	-3.388	2.502	-1.685	3.635	-2.589	1.744	80.215	17.335	17.093
12159	268	43	32	P	W	3.004	-3.388	-2.502	-1.685	3.635	2.589	1.744	80.215	17.335	17.093
12160	268	43	32	P	W	6.178	-2.145	2.909	-2.940	2.241	-3.091	3.124	80.181	17.102	17.326
12161	268	43	32	P	W	53.822	-2.145	-2.909	-2.940	2.241	3.091	3.124	80.181	17.102	17.326
12162	268	43	32	P	W	56.996	-3.388	2.502	-1.685	3.635	-2.589	1.744	80.215	17.335	17.093
12163	268	43	32	P	W	63.005	-3.388	-2.502	-1.685	3.635	2.589	1.744	80.215	17.335	17.093
12164	268	43	32	P	W	66.178	-2.145	2.909	-2.940	2.241	-3.091	3.124	80.181	17.102	17.326
12165	268	43	32	P	W	113.822	-2.145	-2.909	-2.940	2.241	3.091	3.124	80.181	17.102	17.326
12166	268	43	32	P	W	116.996	-3.388	2.502	-1.685	3.635	-2.589	1.744	80.215	17.335	17.093
12167	269	41	35	C	S	0.000	5.011	-2.958	-3.039	-4.554	3.149	3.236	77.115	16.799	18.225
12168	269	41	35	C	S	1.359	-3.292	-0.703	5.308	3.523	0.636	-4.798	77.237	18.221	16.798
12169	269	41	35	C	S	8.513	2.390	0.333	-0.690	-2.281	-0.337	0.700	76.317	19.291	15.902
12170	269	41	35	C	S	51.052	-0.599	-1.120	2.293	0.606	1.071	-2.193	76.311	15.901	19.291
12171	269	41	35	C	S	58.641	-3.292	0.703	5.308	3.523	-0.636	-4.798	77.237	18.221	16.798
12172	269	41	35	C	S	60.000	5.011	2.958	-3.039	-4.554	-3.149	3.236	77.115	16.799	18.225
12173	269	41	35	C	S	61.359	-3.292	-0.703	5.308	3.523	0.636	-4.798	77.237	18.221	16.798
12174	269	41	35	C	S	68.513	2.390	0.333	-0.690	-2.281	-0.337	0.700	76.317	19.291	15.902
12175	269	41	35	C	S	111.052	-0.599	-1.120	2.293	0.606	1.071	-2.193	76.311	15.901	19.291
12176	269	41	35	C	S	118.641	-3.292	0.703	5.308	3.523	-0.636	-4.798	77.237	18.221	16.798
12177	269	41	35	C	W	0.000	5.011	-2.958	-3.039	-4.554	3.149	3.236	77.115	16.799	18.225
12178	269	41	35	C	W	1.359	-3.292	-0.703	5.308	3.523	0.636	-4.798	77.237	18.221	16.798
12179	269	41	35	C	W	8.513	2.390	0.333	-0.690	-2.281	-0.337	0.700	76.317	19.291	15.902
12180	269	41	35	C	W	51.052	-0.599	-1.120	2.293	0.606	1.071	-2.193	76.311	15.901	19.291
12181	269	41	35	C	W	58.641	-3.292	0.703	5.308	3.523	-0.636	-4.798	77.237	18.221	16.798
12182	269	41	35	C	W	60.000	5.011	2.958	-3.039	-4.554	-3.149	3.236	77.115	16.799	18.225
12183	269	41	35	C	W	61.359	-3.292	-0.703	5.308	3.523	0.636	-4.798	77.237	18.221	16.798
12184	269	41	35	C	W	68.513	2.390	0.333	-0.690	-2.281	-0.337	0.700	76.317	19.291	15.902
12185	269	41	35	C	W	111.052	-0.599	-1.120	2.293	0.606	1.071	-2.193	76.311	15.901	19.291
12186	269	41	35	C	W	118.641	-3.292	0.703	5.308	3.523	-0.636	-4.798	77.237	18.221	16.798
12187	269	41	35	P	S	0.000	5.011	-2.958	-3.039	-4.554	3.149	3.236	77.115	16.799	18.225
12188	269	41	35	P	S	1.359	-3.292	-0.703	5.308	3.523	0.636	-4.798	77.237	18.221	16.798
12189	269	41	35	P	S	8.513	2.390	0.333	-0.690	-2.281	-0.337	0.700	76.317	19.291	15.902

BL number	Atoms	γ-PC unit cells	WS_2 unit cells	γ-PC origin atom	WS_2 origin atom	Twist-angle (°)	γ-PC strain 1 (%)	γ-PC strain 2 (%)	γ-PC strain 3 (%)	WS_2 strain 1 (%)	WS_2 strain 2 (%)	WS_2 strain 3 (%)	γ (°)	|**a**|(Å)	|**b**|(Å)
12190	269	41	35	P	S	51.052	-0.599	-1.120	2.293	0.606	1.071	-2.193	76.311	15.901	19.291
12191	269	41	35	P	S	58.641	-3.292	0.703	5.308	3.523	-0.636	-4.798	77.237	18.221	16.798
12192	269	41	35	P	S	60.000	5.011	2.958	-3.039	-4.554	-3.149	3.236	77.115	16.799	18.225
12193	269	41	35	P	S	61.359	-3.292	-0.703	5.308	3.523	0.636	-4.798	77.237	18.221	16.798
12194	269	41	35	P	S	68.513	2.390	0.333	-0.690	-2.281	-0.337	0.700	76.317	19.291	15.902
12195	269	41	35	P	S	111.052	-0.599	-1.120	2.293	0.606	1.071	-2.193	76.311	15.901	19.291
12196	269	41	35	P	S	118.641	-3.292	0.703	5.308	3.523	-0.636	-4.798	77.237	18.221	16.798
12197	269	41	35	P	W	0.000	5.011	-2.958	-3.039	-4.554	3.149	3.236	77.115	16.799	18.225
12198	269	41	35	P	W	1.359	-3.292	-0.703	5.308	3.523	0.636	-4.798	77.237	18.221	16.798
12199	269	41	35	P	W	8.513	2.390	0.333	-0.690	-2.281	-0.337	0.700	76.317	19.291	15.902
12200	269	41	35	P	W	51.052	-0.599	-1.120	2.293	0.606	1.071	-2.193	76.311	15.901	19.291
12201	269	41	35	P	W	58.641	-3.292	0.703	5.308	3.523	-0.636	-4.798	77.237	18.221	16.798
12202	269	41	35	P	W	60.000	5.011	2.958	-3.039	-4.554	-3.149	3.236	77.115	16.799	18.225
12203	269	41	35	P	W	61.359	-3.292	-0.703	5.308	3.523	0.636	-4.798	77.237	18.221	16.798
12204	269	41	35	P	W	68.513	2.390	0.333	-0.690	-2.281	-0.337	0.700	76.317	19.291	15.902
12205	269	41	35	P	W	111.052	-0.599	-1.120	2.293	0.606	1.071	-2.193	76.311	15.901	19.291
12206	269	41	35	P	W	118.641	-3.292	0.703	5.308	3.523	-0.636	-4.798	77.237	18.221	16.798
12207	270	42	34	C	S	6.178	-2.145	-2.258	1.192	2.241	2.205	-1.164	86.712	17.102	17.414
12208	270	42	34	C	S	8.948	1.048	2.554	-2.010	-1.026	-2.661	2.094	86.624	17.420	17.097
12209	270	42	34	C	S	51.052	1.048	-2.554	-2.010	-1.026	2.661	2.094	86.624	17.420	17.097
12210	270	42	34	C	S	53.822	-2.145	2.258	1.192	2.241	-2.206	-1.164	86.712	17.102	17.414
12211	270	42	34	C	S	66.178	-2.145	-2.258	1.192	2.241	2.205	-1.164	86.712	17.102	17.414
12212	270	42	34	C	S	68.948	1.048	2.554	-2.010	-1.026	-2.661	2.094	86.624	17.420	17.097
12213	270	42	34	C	S	111.052	1.048	-2.554	-2.010	-1.026	2.661	2.094	86.624	17.420	17.097
12214	270	42	34	C	S	113.822	-2.145	2.258	1.192	2.241	-2.206	-1.164	86.712	17.102	17.414
12215	270	42	34	C	W	6.178	-2.145	-2.258	1.192	2.241	2.205	-1.164	86.712	17.102	17.414
12216	270	42	34	C	W	8.948	1.048	2.554	-2.010	-1.026	-2.661	2.094	86.624	17.420	17.097
12217	270	42	34	C	W	51.052	1.048	-2.554	-2.010	-1.026	2.661	2.094	86.624	17.420	17.097
12218	270	42	34	C	W	53.822	-2.145	2.258	1.192	2.241	-2.206	-1.164	86.712	17.102	17.414
12219	270	42	34	C	W	66.178	-2.145	-2.258	1.192	2.241	2.205	-1.164	86.712	17.102	17.414
12220	270	42	34	C	W	68.948	1.048	2.554	-2.010	-1.026	-2.661	2.094	86.624	17.420	17.097
12221	270	42	34	C	W	111.052	1.048	-2.554	-2.010	-1.026	2.661	2.094	86.624	17.420	17.097
12222	270	42	34	C	W	113.822	-2.145	2.258	1.192	2.241	-2.206	-1.164	86.712	17.102	17.414
12223	270	42	34	P	S	6.178	-2.145	-2.258	1.192	2.241	2.205	-1.164	86.712	17.102	17.414
12224	270	42	34	P	S	8.948	1.048	2.554	-2.010	-1.026	-2.661	2.094	86.624	17.420	17.097
12225	270	42	34	P	S	51.052	1.048	-2.554	-2.010	-1.026	2.661	2.094	86.624	17.420	17.097
12226	270	42	34	P	S	53.822	-2.145	2.258	1.192	2.241	-2.206	-1.164	86.712	17.102	17.414
12227	270	42	34	P	S	66.178	-2.145	-2.258	1.192	2.241	2.205	-1.164	86.712	17.102	17.414
12228	270	42	34	P	S	68.948	1.048	2.554	-2.010	-1.026	-2.661	2.094	86.624	17.420	17.097
12229	270	42	34	P	S	111.052	1.048	-2.554	-2.010	-1.026	2.661	2.094	86.624	17.420	17.097
12230	270	42	34	P	S	113.822	-2.145	2.258	1.192	2.241	-2.206	-1.164	86.712	17.102	17.414
12231	270	42	34	P	W	6.178	-2.145	-2.258	1.192	2.241	2.205	-1.164	86.712	17.102	17.414
12232	270	42	34	P	W	8.948	1.048	2.554	-2.010	-1.026	-2.661	2.094	86.624	17.420	17.097
12233	270	42	34	P	W	51.052	1.048	-2.554	-2.010	-1.026	2.661	2.094	86.624	17.420	17.097
12234	270	42	34	P	W	53.822	-2.145	2.258	1.192	2.241	-2.206	-1.164	86.712	17.102	17.414
12235	270	42	34	P	W	66.178	-2.145	-2.258	1.192	2.241	2.205	-1.164	86.712	17.102	17.414
12236	270	42	34	P	W	68.948	1.048	2.554	-2.010	-1.026	-2.661	2.094	86.624	17.420	17.097
12237	270	42	34	P	W	111.052	1.048	-2.554	-2.010	-1.026	2.661	2.094	86.624	17.420	17.097
12238	270	42	34	P	W	113.822	-2.145	2.258	1.192	2.241	-2.206	-1.164	86.712	17.102	17.414
12239	271	43	33	C	S	4.715	-3.007	-1.736	-0.579	3.200	1.757	0.585	79.851	16.951	17.766
12240	271	43	33	C	S	7.154	-0.955	2.445	-2.647	0.973	-2.582	2.795	79.808	17.772	16.947

BL number	Atoms	γ-PC unit cells	WS_2 unit cells	γ-PC origin atom	WS_2 origin atom	Twist-angle (°)	γ-PC strain 1 (%)	γ-PC strain 2 (%)	γ-PC strain 3 (%)	WS_2 strain 1 (%)	WS_2 strain 2 (%)	WS_2 strain 3 (%)	γ (°)	|**a**|(Å)	|**b**|(Å)
12241	271	43	33	C	S	13.898	-4.228	-1.286	0.739	4.619	1.267	-0.729	79.868	17.184	17.533
12242	271	43	33	C	S	16.337	0.353	2.869	-3.877	-0.351	-3.110	4.203	79.773	17.539	17.181
12243	271	43	33	C	S	43.663	0.353	-2.869	-3.877	-0.351	3.110	4.203	79.773	17.539	17.181
12244	271	43	33	C	S	46.102	-4.228	1.286	0.739	4.619	-1.267	-0.729	79.868	17.184	17.533
12245	271	43	33	C	S	52.846	-0.955	-2.445	-2.647	0.973	2.582	2.795	79.808	17.772	16.947
12246	271	43	33	C	S	55.285	-3.007	1.736	-0.579	3.200	-1.757	0.586	79.851	16.951	17.766
12247	271	43	33	C	S	64.715	-3.007	-1.736	-0.579	3.200	1.757	0.585	79.851	16.951	17.766
12248	271	43	33	C	S	67.154	-0.955	2.445	-2.647	0.973	-2.582	2.795	79.808	17.772	16.947
12249	271	43	33	C	S	73.898	-4.228	-1.286	0.739	4.619	1.267	-0.729	79.868	17.184	17.533
12250	271	43	33	C	S	76.337	0.353	2.869	-3.877	-0.351	-3.110	4.203	79.773	17.539	17.181
12251	271	43	33	C	S	103.663	0.353	-2.869	-3.877	-0.351	3.110	4.203	79.773	17.539	17.181
12252	271	43	33	C	S	106.102	-4.228	1.286	0.739	4.619	-1.267	-0.729	79.868	17.184	17.533
12253	271	43	33	C	S	112.846	-0.955	-2.445	-2.647	0.973	2.582	2.795	79.808	17.772	16.947
12254	271	43	33	C	S	115.285	-3.007	1.736	-0.579	3.200	-1.757	0.586	79.851	16.951	17.766
12255	271	43	33	C	W	4.715	-3.007	-1.736	-0.579	3.200	1.757	0.585	79.851	16.951	17.766
12256	271	43	33	C	W	7.154	-0.955	2.445	-2.647	0.973	-2.582	2.795	79.808	17.772	16.947
12257	271	43	33	C	W	13.898	-4.228	-1.286	0.739	4.619	1.267	-0.729	79.868	17.184	17.533
12258	271	43	33	C	W	16.337	0.353	2.869	-3.877	-0.351	-3.110	4.203	79.773	17.539	17.181
12259	271	43	33	C	W	43.663	0.353	-2.869	-3.877	-0.351	3.110	4.203	79.773	17.539	17.181
12260	271	43	33	C	W	46.102	-4.228	1.286	0.739	4.619	-1.267	-0.729	79.868	17.184	17.533
12261	271	43	33	C	W	52.846	-0.955	-2.445	-2.647	0.973	2.582	2.795	79.808	17.772	16.947
12262	271	43	33	C	W	55.285	-3.007	1.736	-0.579	3.200	-1.757	0.586	79.851	16.951	17.766
12263	271	43	33	C	W	64.715	-3.007	-1.736	-0.579	3.200	1.757	0.585	79.851	16.951	17.766
12264	271	43	33	C	W	67.154	-0.955	2.445	-2.647	0.973	-2.582	2.795	79.808	17.772	16.947
12265	271	43	33	C	W	73.898	-4.228	-1.286	0.739	4.619	1.267	-0.729	79.868	17.184	17.533
12266	271	43	33	C	W	76.337	0.353	2.869	-3.877	-0.351	-3.110	4.203	79.773	17.539	17.181
12267	271	43	33	C	W	103.663	0.353	-2.869	-3.877	-0.351	3.110	4.203	79.773	17.539	17.181
12268	271	43	33	C	W	106.102	-4.228	1.286	0.739	4.619	-1.267	-0.729	79.868	17.184	17.533
12269	271	43	33	C	W	112.846	-0.955	-2.445	-2.647	0.973	2.582	2.795	79.808	17.772	16.947
12270	271	43	33	C	W	115.285	-3.007	1.736	-0.579	3.200	-1.757	0.586	79.851	16.951	17.766
12271	271	43	33	P	S	4.715	-3.007	-1.736	-0.579	3.200	1.757	0.585	79.851	16.951	17.766
12272	271	43	33	P	S	7.154	-0.955	2.445	-2.647	0.973	-2.582	2.795	79.808	17.772	16.947
12273	271	43	33	P	S	52.846	-0.955	-2.445	-2.647	0.973	2.582	2.795	79.808	17.772	16.947
12274	271	43	33	P	S	55.285	-3.007	1.736	-0.579	3.200	-1.757	0.586	79.851	16.951	17.766
12275	271	43	33	P	S	64.715	-3.007	-1.736	-0.579	3.200	1.757	0.585	79.851	16.951	17.766
12276	271	43	33	P	S	67.154	-0.955	2.445	-2.647	0.973	-2.582	2.795	79.808	17.772	16.947
12277	271	43	33	P	S	112.846	-0.955	-2.445	-2.647	0.973	2.582	2.795	79.808	17.772	16.947
12278	271	43	33	P	S	115.285	-3.007	1.736	-0.579	3.200	-1.757	0.586	79.851	16.951	17.766
12279	271	43	33	P	W	4.715	-3.007	-1.736	-0.579	3.200	1.757	0.585	79.851	16.951	17.766
12280	271	43	33	P	W	7.154	-0.955	2.445	-2.647	0.973	-2.582	2.795	79.808	17.772	16.947
12281	271	43	33	P	W	52.846	-0.955	-2.445	-2.647	0.973	2.582	2.795	79.808	17.772	16.947
12282	271	43	33	P	W	55.285	-3.007	1.736	-0.579	3.200	-1.757	0.586	79.851	16.951	17.766
12283	271	43	33	P	W	64.715	-3.007	-1.736	-0.579	3.200	1.757	0.585	79.851	16.951	17.766
12284	271	43	33	P	W	67.154	-0.955	2.445	-2.647	0.973	-2.582	2.795	79.808	17.772	16.947
12285	271	43	33	P	W	112.846	-0.955	-2.445	-2.647	0.973	2.582	2.795	79.808	17.772	16.947
12286	271	43	33	P	W	115.285	-3.007	1.736	-0.579	3.200	-1.757	0.586	79.851	16.951	17.766
12287	272	41	36	C	S	7.589	0.334	-1.884	2.790	-0.332	1.784	-2.643	77.847	18.904	16.358
12288	272	41	36	C	S	10.158	2.281	2.675	0.824	-2.182	-2.631	-0.811	77.794	16.362	18.901
12289	272	41	36	C	S	16.102	2.853	0.724	0.275	-2.699	-0.720	-0.273	69.055	18.455	17.540
12290	272	41	36	C	S	16.337	0.353	1.162	2.770	-0.351	-1.101	-2.625	69.064	17.539	18.455
12291	272	41	36	C	S	21.052	1.339	-2.616	1.757	-1.304	2.527	-1.697	75.205	16.211	19.284

BL number	Atoms	γ-PC unit cells	WS_2 unit cells	γ-PC origin atom	WS_2 origin atom	Twist-angle (°)	γ-PC strain 1 (%)	γ-PC strain 2 (%)	γ-PC strain 3 (%)	WS_2 strain 1 (%)	WS_2 strain 2 (%)	WS_2 strain 3 (%)	γ (°)	|**a**|(Å)	|**b**|(Å)
12292	272	41	36	C	S	23.691	2.390	2.052	0.719	-2.281	-2.023	-0.709	75.226	19.291	16.205
12293	272	41	36	C	S	25.050	-0.955	-2.227	4.177	0.973	2.055	-3.855	69.051	17.772	18.223
12294	272	41	36	C	S	25.285	4.262	-1.713	-1.031	-3.928	1.749	1.053	69.031	18.222	17.776
12295	272	41	36	C	S	27.126	1.029	-1.134	2.072	-1.008	1.089	-1.989	76.069	19.034	16.361
12296	272	41	36	C	S	28.055	2.281	0.515	0.824	-2.182	-0.506	-0.811	76.074	16.362	19.033
12297	272	41	36	C	S	31.945	2.281	-0.514	0.824	-2.182	0.506	-0.811	76.074	16.362	19.033
12298	272	41	36	C	S	34.715	4.262	1.713	-1.031	-3.928	-1.749	1.053	69.031	18.222	17.776
12299	272	41	36	C	S	34.950	-0.955	2.227	4.178	0.973	-2.055	-3.855	69.051	17.772	18.223
12300	272	41	36	C	S	36.309	2.390	-2.052	0.719	-2.281	2.023	-0.709	75.226	19.291	16.205
12301	272	41	36	C	S	38.948	1.339	2.616	1.757	-1.304	-2.527	-1.697	75.205	16.211	19.284
12302	272	41	36	C	S	43.663	0.353	-1.162	2.770	-0.351	1.101	-2.625	69.064	17.539	18.455
12303	272	41	36	C	S	43.898	2.853	-0.724	0.275	-2.699	0.720	-0.273	69.055	18.455	17.540
12304	272	41	36	C	S	49.842	2.281	-2.675	0.824	-2.182	2.631	-0.811	77.794	16.362	18.901
12305	272	41	36	C	S	52.411	0.334	1.884	2.790	-0.332	-1.784	-2.643	77.847	18.904	16.358
12306	272	41	36	C	S	67.589	0.334	-1.884	2.790	-0.332	1.784	-2.643	77.847	18.904	16.358
12307	272	41	36	C	S	70.158	2.281	2.675	0.824	-2.182	-2.631	-0.811	77.794	16.362	18.901
12308	272	41	36	C	S	76.102	2.853	0.724	0.275	-2.699	-0.720	-0.273	69.055	18.455	17.540
12309	272	41	36	C	S	76.337	0.353	1.162	2.770	-0.351	-1.101	-2.625	69.064	17.539	18.455
12310	272	41	36	C	S	81.052	1.339	-2.616	1.757	-1.304	2.527	-1.697	75.205	16.211	19.284
12311	272	41	36	C	S	83.691	2.390	2.052	0.719	-2.281	-2.023	-0.709	75.226	19.291	16.205
12312	272	41	36	C	S	85.050	-0.955	-2.227	4.177	0.973	2.055	-3.855	69.051	17.772	18.223
12313	272	41	36	C	S	85.285	4.262	-1.713	-1.031	-3.928	1.749	1.053	69.031	18.222	17.776
12314	272	41	36	C	S	87.126	1.029	-1.134	2.072	-1.008	1.089	-1.989	76.069	19.034	16.361
12315	272	41	36	C	S	91.945	2.281	-0.514	0.824	-2.182	0.506	-0.811	76.074	16.362	19.033
12316	272	41	36	C	S	92.874	1.029	1.134	2.072	-1.008	-1.089	-1.989	76.069	19.034	16.361
12317	272	41	36	C	S	94.715	4.262	1.713	-1.031	-3.928	-1.749	1.053	69.031	18.222	17.776
12318	272	41	36	C	S	94.950	-0.955	2.227	4.178	0.973	-2.055	-3.855	69.050	17.772	18.223
12319	272	41	36	C	S	96.309	2.390	-2.052	0.719	-2.281	2.023	-0.709	75.226	19.291	16.205
12320	272	41	36	C	S	98.948	1.339	2.616	1.757	-1.304	-2.527	-1.697	75.205	16.211	19.284
12321	272	41	36	C	S	103.663	0.353	-1.162	2.770	-0.351	1.101	-2.625	69.064	17.539	18.455
12322	272	41	36	C	S	103.898	2.853	-0.724	0.275	-2.699	0.720	-0.273	69.055	18.455	17.540
12323	272	41	36	C	S	109.842	2.281	-2.675	0.824	-2.182	2.631	-0.811	77.794	16.362	18.901
12324	272	41	36	C	S	112.411	0.334	1.884	2.790	-0.332	-1.784	-2.643	77.847	18.904	16.358
12325	272	41	36	C	W	7.589	0.334	-1.884	2.790	-0.332	1.784	-2.643	77.847	18.904	16.358
12326	272	41	36	C	W	10.158	2.281	2.675	0.824	-2.182	-2.631	-0.811	77.794	16.362	18.901
12327	272	41	36	C	W	16.102	2.853	0.724	0.275	-2.699	-0.720	-0.273	69.055	18.455	17.540
12328	272	41	36	C	W	16.337	0.353	1.162	2.770	-0.351	-1.101	-2.625	69.064	17.539	18.455
12329	272	41	36	C	W	21.052	1.339	-2.616	1.757	-1.304	2.527	-1.697	75.205	16.211	19.284
12330	272	41	36	C	W	23.691	2.390	2.052	0.719	-2.281	-2.023	-0.709	75.226	19.291	16.205
12331	272	41	36	C	W	25.050	-0.955	-2.227	4.177	0.973	2.055	-3.855	69.051	17.772	18.223
12332	272	41	36	C	W	25.285	4.262	-1.713	-1.031	-3.928	1.749	1.053	69.031	18.222	17.776
12333	272	41	36	C	W	27.126	1.029	-1.134	2.072	-1.008	1.089	-1.989	76.069	19.034	16.361
12334	272	41	36	C	W	31.945	2.281	-0.514	0.824	-2.182	0.506	-0.811	76.074	16.362	19.033
12335	272	41	36	C	W	32.874	1.029	1.134	2.072	-1.008	-1.089	-1.989	76.069	19.034	16.361
12336	272	41	36	C	W	34.715	4.262	1.713	-1.031	-3.928	-1.749	1.053	69.031	18.222	17.776
12337	272	41	36	C	W	34.950	-0.955	2.227	4.178	0.973	-2.055	-3.855	69.051	17.772	18.223
12338	272	41	36	C	W	36.309	2.390	-2.052	0.719	-2.281	2.023	-0.709	75.226	19.291	16.205
12339	272	41	36	C	W	38.948	1.339	2.616	1.757	-1.304	-2.527	-1.697	75.205	16.211	19.284
12340	272	41	36	C	W	43.663	0.353	-1.162	2.770	-0.351	1.101	-2.625	69.064	17.539	18.455
12341	272	41	36	C	W	43.898	2.853	-0.724	0.275	-2.699	0.720	-0.273	69.055	18.455	17.540
12342	272	41	36	C	W	49.842	2.281	-2.675	0.824	-2.182	2.631	-0.811	77.794	16.362	18.901

BL number	Atoms	γ-PC unit cells	WS_2 unit cells	γ-PC origin atom	WS_2 origin atom	Twist-angle (°)	γ-PC strain 1 (%)	γ-PC strain 2 (%)	γ-PC strain 3 (%)	WS_2 strain 1 (%)	WS_2 strain 2 (%)	WS_2 strain 3 (%)	γ (°)	|**a**|(Å)	|**b**|(Å)
12343	272	41	36	C	W	52.411	0.334	1.884	2.790	-0.332	-1.784	-2.643	77.847	18.904	16.358
12344	272	41	36	C	W	67.589	0.334	-1.884	2.790	-0.332	1.784	-2.643	77.847	18.904	16.358
12345	272	41	36	C	W	70.158	2.281	2.675	0.824	-2.182	-2.631	-0.811	77.794	16.362	18.901
12346	272	41	36	C	W	76.337	0.353	1.162	2.770	-0.351	-1.101	-2.625	69.064	17.539	18.455
12347	272	41	36	C	W	81.052	1.339	-2.616	1.757	-1.304	2.527	-1.697	75.205	16.211	19.284
12348	272	41	36	C	W	83.691	2.390	2.052	0.719	-2.281	-2.023	-0.709	75.226	19.291	16.205
12349	272	41	36	C	W	85.050	-0.955	-2.227	4.177	0.973	2.055	-3.855	69.051	17.772	18.223
12350	272	41	36	C	W	85.285	4.262	-1.713	-1.031	-3.928	1.749	1.053	69.031	18.222	17.776
12351	272	41	36	C	W	87.126	1.029	-1.134	2.072	-1.008	1.089	-1.989	76.069	19.034	16.361
12352	272	41	36	C	W	88.055	2.281	0.515	0.824	-2.182	-0.506	-0.811	76.074	16.362	19.033
12353	272	41	36	C	W	91.945	2.281	-0.514	0.824	-2.182	0.506	-0.811	76.074	16.362	19.033
12354	272	41	36	C	W	94.715	4.262	1.713	-1.031	-3.928	-1.749	1.053	69.031	18.222	17.776
12355	272	41	36	C	W	94.950	-0.955	2.227	4.178	0.973	-2.055	-3.855	69.050	17.772	18.223
12356	272	41	36	C	W	96.309	2.390	-2.052	0.719	-2.281	2.023	-0.709	75.226	19.291	16.205
12357	272	41	36	C	W	98.948	1.339	2.616	1.757	-1.304	-2.527	-1.697	75.205	16.211	19.284
12358	272	41	36	C	W	103.663	0.353	-1.162	2.770	-0.351	1.101	-2.625	69.064	17.539	18.455
12359	272	41	36	C	W	103.898	2.853	-0.724	0.275	-2.699	0.720	-0.273	69.055	18.455	17.540
12360	272	41	36	C	W	109.842	2.281	-2.675	0.824	-2.182	2.631	-0.811	77.794	16.362	18.901
12361	272	41	36	C	W	112.411	0.334	1.884	2.790	-0.332	-1.784	-2.643	77.847	18.904	16.358
12362	272	41	36	P	S	7.589	0.334	-1.884	2.790	-0.332	1.784	-2.643	77.847	18.904	16.358
12363	272	41	36	P	S	10.158	2.281	2.675	0.824	-2.182	-2.631	-0.811	77.794	16.362	18.901
12364	272	41	36	P	S	21.052	1.339	-2.616	1.757	-1.304	2.527	-1.697	75.205	16.211	19.284
12365	272	41	36	P	S	23.691	2.390	2.052	0.719	-2.281	-2.023	-0.709	75.226	19.291	16.205
12366	272	41	36	P	S	25.050	-0.955	-2.227	4.177	0.973	2.055	-3.855	69.051	17.772	18.223
12367	272	41	36	P	S	25.285	4.262	-1.713	-1.031	-3.928	1.749	1.053	69.031	18.222	17.776
12368	272	41	36	P	S	27.126	1.029	-1.134	2.072	-1.008	1.089	-1.989	76.069	19.034	16.361
12369	272	41	36	P	S	31.945	2.281	-0.514	0.824	-2.182	0.506	-0.811	76.074	16.362	19.033
12370	272	41	36	P	S	32.874	1.029	1.134	2.072	-1.008	-1.089	-1.989	76.069	19.034	16.361
12371	272	41	36	P	S	34.715	4.262	1.713	-1.031	-3.928	-1.749	1.053	69.031	18.222	17.776
12372	272	41	36	P	S	34.950	-0.955	2.227	4.178	0.973	-2.055	-3.855	69.051	17.772	18.223
12373	272	41	36	P	S	43.898	2.853	-0.724	0.275	-2.699	0.720	-0.273	69.055	18.455	17.540
12374	272	41	36	P	S	49.842	2.281	-2.675	0.824	-2.182	2.631	-0.811	77.794	16.362	18.901
12375	272	41	36	P	S	52.411	0.334	1.884	2.790	-0.332	-1.784	-2.643	77.847	18.904	16.358
12376	272	41	36	P	S	67.589	0.334	-1.884	2.790	-0.332	1.784	-2.643	77.847	18.904	16.358
12377	272	41	36	P	S	70.158	2.281	2.675	0.824	-2.182	-2.631	-0.811	77.794	16.362	18.901
12378	272	41	36	P	S	85.050	-0.955	-2.227	4.177	0.973	2.055	-3.855	69.051	17.772	18.223
12379	272	41	36	P	S	85.285	4.262	-1.713	-1.031	-3.928	1.749	1.053	69.031	18.222	17.776
12380	272	41	36	P	S	87.126	1.029	-1.134	2.072	-1.008	1.089	-1.989	76.069	19.034	16.361
12381	272	41	36	P	S	88.055	2.281	0.515	0.824	-2.182	-0.506	-0.811	76.074	16.362	19.033
12382	272	41	36	P	S	91.945	2.281	-0.514	0.824	-2.182	0.506	-0.811	76.074	16.362	19.033
12383	272	41	36	P	S	94.715	4.262	1.713	-1.031	-3.928	-1.749	1.053	69.031	18.222	17.776
12384	272	41	36	P	S	94.950	-0.955	2.227	4.178	0.973	-2.055	-3.855	69.050	17.772	18.223
12385	272	41	36	P	S	96.309	2.390	-2.052	0.719	-2.281	2.023	-0.709	75.226	19.291	16.205
12386	272	41	36	P	S	98.948	1.339	2.616	1.757	-1.304	-2.527	-1.697	75.205	16.211	19.284
12387	272	41	36	P	S	109.842	2.281	-2.675	0.824	-2.182	2.631	-0.811	77.794	16.362	18.901
12388	272	41	36	P	S	112.411	0.334	1.884	2.790	-0.332	-1.784	-2.643	77.847	18.904	16.358
12389	272	41	36	P	W	7.589	0.334	-1.884	2.790	-0.332	1.784	-2.643	77.847	18.904	16.358
12390	272	41	36	P	W	10.158	2.281	2.675	0.824	-2.182	-2.631	-0.811	77.794	16.362	18.901
12391	272	41	36	P	W	16.102	2.853	0.724	0.275	-2.699	-0.720	-0.273	69.055	18.455	17.540
12392	272	41	36	P	W	25.050	-0.955	-2.227	4.177	0.973	2.055	-3.855	69.051	17.772	18.223
12393	272	41	36	P	W	25.285	4.262	-1.713	-1.031	-3.928	1.749	1.053	69.031	18.222	17.776

BL number	Atoms	γ-PC unit cells	WS_2 unit cells	γ-PC origin atom	WS_2 origin atom	Twist-angle (°)	γ-PC strain 1 (%)	γ-PC strain 2 (%)	γ-PC strain 3 (%)	WS_2 strain 1 (%)	WS_2 strain 2 (%)	WS_2 strain 3 (%)	γ (°)	$\lvert\mathbf{a}\rvert$(Å)	$\lvert\mathbf{b}\rvert$(Å)
12394	272	41	36	P	W	27.126	1.029	-1.134	2.072	-1.008	1.089	-1.989	76.069	19.034	16.361
12395	272	41	36	P	W	28.055	2.281	0.515	0.824	-2.182	-0.506	-0.811	76.074	16.362	19.033
12396	272	41	36	P	W	31.945	2.281	-0.514	0.824	-2.182	0.506	-0.811	76.074	16.362	19.033
12397	272	41	36	P	W	34.715	4.262	1.713	-1.031	-3.928	-1.749	1.053	69.031	18.222	17.776
12398	272	41	36	P	W	34.950	-0.955	2.227	4.178	0.973	-2.055	-3.855	69.051	17.772	18.223
12399	272	41	36	P	W	36.309	2.390	-2.052	0.719	-2.281	2.023	-0.709	75.226	19.291	16.205
12400	272	41	36	P	W	38.948	1.339	2.616	1.757	-1.304	-2.527	-1.697	75.205	16.211	19.284
12401	272	41	36	P	W	49.842	2.281	-2.675	0.824	-2.182	2.631	-0.811	77.794	16.362	18.901
12402	272	41	36	P	W	52.411	0.334	1.884	2.790	-0.332	-1.784	-2.643	77.847	18.904	16.358
12403	272	41	36	P	W	67.589	0.334	-1.884	2.790	-0.332	1.784	-2.643	77.847	18.904	16.358
12404	272	41	36	P	W	70.158	2.281	2.675	0.824	-2.182	-2.631	-0.811	77.794	16.362	18.901
12405	272	41	36	P	W	76.102	2.853	0.724	0.275	-2.699	-0.720	-0.273	69.055	18.455	17.540
12406	272	41	36	P	W	81.052	1.339	-2.616	1.757	-1.304	2.527	-1.697	75.205	16.211	19.284
12407	272	41	36	P	W	83.691	2.390	2.052	0.719	-2.281	-2.023	-0.709	75.226	19.291	16.205
12408	272	41	36	P	W	85.050	-0.955	-2.227	4.177	0.973	2.055	-3.855	69.051	17.772	18.223
12409	272	41	36	P	W	85.285	4.262	-1.713	-1.031	-3.928	1.749	1.053	69.031	18.222	17.776
12410	272	41	36	P	W	87.126	1.029	-1.134	2.072	-1.008	1.089	-1.989	76.069	19.034	16.361
12411	272	41	36	P	W	91.945	2.281	-0.514	0.824	-2.182	0.506	-0.811	76.074	16.362	19.033
12412	272	41	36	P	W	92.874	1.029	1.134	2.072	-1.008	-1.089	-1.989	76.069	19.034	16.361
12413	272	41	36	P	W	94.715	4.262	1.713	-1.031	-3.928	-1.749	1.053	69.031	18.222	17.776
12414	272	41	36	P	W	94.950	-0.955	2.227	4.178	0.973	-2.055	-3.855	69.050	17.772	18.223
12415	272	41	36	P	W	103.898	2.853	-0.724	0.275	-2.699	0.720	-0.273	69.055	18.455	17.540
12416	272	41	36	P	W	109.842	2.281	-2.675	0.824	-2.182	2.631	-0.811	77.794	16.362	18.901
12417	272	41	36	P	W	112.411	0.334	1.884	2.790	-0.332	-1.784	-2.643	77.847	18.904	16.358
12418	272	44	32	C	S	1.359	-3.292	-1.433	-2.881	3.523	1.520	3.057	69.332	18.221	17.330
12419	272	44	32	C	S	1.945	-4.723	-1.965	-1.391	5.216	2.021	1.431	82.468	15.242	19.559
12420	272	44	32	C	S	3.004	-3.388	1.358	-2.783	3.635	-1.438	2.947	69.332	17.335	18.216
12421	272	44	32	C	S	4.715	-1.702	2.719	-4.431	1.762	-2.983	4.862	82.396	19.567	15.236
12422	272	44	32	C	S	55.285	-1.702	-2.719	-4.431	1.762	2.983	4.862	82.396	19.567	15.236
12423	272	44	32	C	S	56.996	-3.388	-1.358	-2.783	3.635	1.438	2.947	69.332	17.335	18.216
12424	272	44	32	C	S	58.055	-4.723	1.965	-1.391	5.216	-2.021	1.431	82.468	15.242	19.559
12425	272	44	32	C	S	58.641	-3.292	1.433	-2.881	3.523	-1.520	3.057	69.332	18.221	17.330
12426	272	44	32	C	S	61.359	-3.292	-1.433	-2.881	3.523	1.520	3.057	69.332	18.221	17.330
12427	272	44	32	C	S	61.945	-4.723	-1.965	-1.391	5.216	2.021	1.431	82.468	15.242	19.559
12428	272	44	32	C	S	63.005	-3.388	1.358	-2.783	3.635	-1.438	2.947	69.332	17.335	18.216
12429	272	44	32	C	S	64.715	-1.702	2.719	-4.431	1.762	-2.983	4.862	82.396	19.567	15.236
12430	272	44	32	C	S	115.285	-1.702	-2.719	-4.431	1.762	2.983	4.862	82.396	19.567	15.236
12431	272	44	32	C	S	116.996	-3.388	-1.358	-2.783	3.635	1.438	2.947	69.332	17.335	18.216
12432	272	44	32	C	S	118.055	-4.723	1.965	-1.391	5.216	-2.021	1.431	82.468	15.242	19.559
12433	272	44	32	C	S	118.641	-3.292	1.433	-2.881	3.523	-1.520	3.057	69.332	18.221	17.330
12434	272	44	32	C	W	1.359	-3.292	-1.433	-2.881	3.523	1.520	3.057	69.332	18.221	17.330
12435	272	44	32	C	W	1.945	-4.723	-1.965	-1.391	5.216	2.021	1.431	82.468	15.242	19.559
12436	272	44	32	C	W	3.004	-3.388	1.358	-2.783	3.635	-1.438	2.947	69.332	17.335	18.216
12437	272	44	32	C	W	4.715	-1.702	2.719	-4.431	1.762	-2.983	4.862	82.396	19.567	15.236
12438	272	44	32	C	W	55.285	-1.702	-2.719	-4.431	1.762	2.983	4.862	82.396	19.567	15.236
12439	272	44	32	C	W	56.996	-3.388	-1.358	-2.783	3.635	1.438	2.947	69.332	17.335	18.216
12440	272	44	32	C	W	58.055	-4.723	1.965	-1.391	5.216	-2.021	1.431	82.468	15.242	19.559
12441	272	44	32	C	W	58.641	-3.292	1.433	-2.881	3.523	-1.520	3.057	69.332	18.221	17.330
12442	272	44	32	C	W	61.359	-3.292	-1.433	-2.881	3.523	1.520	3.057	69.332	18.221	17.330
12443	272	44	32	C	W	61.945	-4.723	-1.965	-1.391	5.216	2.021	1.431	82.468	15.242	19.559
12444	272	44	32	C	W	63.005	-3.388	1.358	-2.783	3.635	-1.438	2.947	69.332	17.335	18.216

BL number	Atoms	γ-PC unit cells	WS_2 unit cells	γ-PC origin atom	WS_2 origin atom	Twist-angle (°)	γ-PC strain 1 (%)	γ-PC strain 2 (%)	γ-PC strain 3 (%)	WS_2 strain 1 (%)	WS_2 strain 2 (%)	WS_2 strain 3 (%)	γ (°)	|**a**|(Å)	|**b**|(Å)
12445	272	44	32	C	W	64.715	-1.702	2.719	-4.431	1.762	-2.983	4.862	82.396	19.567	15.236
12446	272	44	32	C	W	115.285	-1.702	-2.719	-4.431	1.762	2.983	4.862	82.396	19.567	15.236
12447	272	44	32	C	W	116.996	-3.388	-1.358	-2.783	3.635	1.438	2.947	69.332	17.335	18.216
12448	272	44	32	C	W	118.055	-4.723	1.965	-1.391	5.216	-2.021	1.431	82.468	15.242	19.559
12449	272	44	32	C	W	118.641	-3.292	1.433	-2.881	3.523	-1.520	3.057	69.332	18.221	17.330
12450	272	44	32	P	S	1.359	-3.292	-1.433	-2.881	3.523	1.520	3.057	69.332	18.221	17.330
12451	272	44	32	P	S	1.945	-4.723	-1.965	-1.391	5.216	2.021	1.431	82.468	15.242	19.559
12452	272	44	32	P	S	3.004	-3.388	1.358	-2.783	3.635	-1.438	2.947	69.332	17.335	18.216
12453	272	44	32	P	S	4.715	-1.702	2.719	-4.431	1.762	-2.983	4.862	82.396	19.567	15.236
12454	272	44	32	P	S	55.285	-1.702	-2.719	-4.431	1.762	2.983	4.862	82.396	19.567	15.236
12455	272	44	32	P	S	56.996	-3.388	-1.358	-2.783	3.635	1.438	2.947	69.332	17.335	18.216
12456	272	44	32	P	S	58.055	-4.723	1.965	-1.391	5.216	-2.021	1.431	82.468	15.242	19.559
12457	272	44	32	P	S	58.641	-3.292	1.433	-2.881	3.523	-1.520	3.057	69.332	18.221	17.330
12458	272	44	32	P	S	61.359	-3.292	-1.433	-2.881	3.523	1.520	3.057	69.332	18.221	17.330
12459	272	44	32	P	S	61.945	-4.723	-1.965	-1.391	5.216	2.021	1.431	82.468	15.242	19.559
12460	272	44	32	P	S	63.005	-3.388	1.358	-2.783	3.635	-1.438	2.947	69.332	17.335	18.216
12461	272	44	32	P	S	64.715	-1.702	2.719	-4.431	1.762	-2.983	4.862	82.396	19.567	15.236
12462	272	44	32	P	S	115.285	-1.702	-2.719	-4.431	1.762	2.983	4.862	82.396	19.567	15.236
12463	272	44	32	P	S	116.996	-3.388	-1.358	-2.783	3.635	1.438	2.947	69.332	17.335	18.216
12464	272	44	32	P	S	118.055	-4.723	1.965	-1.391	5.216	-2.021	1.431	82.468	15.242	19.559
12465	272	44	32	P	S	118.641	-3.292	1.433	-2.881	3.523	-1.520	3.057	69.332	18.221	17.330
12466	272	44	32	P	W	1.359	-3.292	-1.433	-2.881	3.523	1.520	3.057	69.332	18.221	17.330
12467	272	44	32	P	W	1.945	-4.723	-1.965	-1.391	5.216	2.021	1.431	82.468	15.242	19.559
12468	272	44	32	P	W	3.004	-3.388	1.358	-2.783	3.635	-1.438	2.947	69.332	17.335	18.216
12469	272	44	32	P	W	4.715	-1.702	2.719	-4.431	1.762	-2.983	4.862	82.396	19.567	15.236
12470	272	44	32	P	W	55.285	-1.702	-2.719	-4.431	1.762	2.983	4.862	82.396	19.567	15.236
12471	272	44	32	P	W	56.996	-3.388	-1.358	-2.783	3.635	1.438	2.947	69.332	17.335	18.216
12472	272	44	32	P	W	58.055	-4.723	1.965	-1.391	5.216	-2.021	1.431	82.468	15.242	19.559
12473	272	44	32	P	W	58.641	-3.292	1.433	-2.881	3.523	-1.520	3.057	69.332	18.221	17.330
12474	272	44	32	P	W	61.359	-3.292	-1.433	-2.881	3.523	1.520	3.057	69.332	18.221	17.330
12475	272	44	32	P	W	61.945	-4.723	-1.965	-1.391	5.216	2.021	1.431	82.468	15.242	19.559
12476	272	44	32	P	W	63.005	-3.388	1.358	-2.783	3.635	-1.438	2.947	69.332	17.335	18.216
12477	272	44	32	P	W	64.715	-1.702	2.719	-4.431	1.762	-2.983	4.862	82.396	19.567	15.236
12478	272	44	32	P	W	115.285	-1.702	-2.719	-4.431	1.762	2.983	4.862	82.396	19.567	15.236
12479	272	44	32	P	W	116.996	-3.388	-1.358	-2.783	3.635	1.438	2.947	69.332	17.335	18.216
12480	272	44	32	P	W	118.055	-4.723	1.965	-1.391	5.216	-2.021	1.431	82.468	15.242	19.559
12481	272	44	32	P	W	118.641	-3.292	1.433	-2.881	3.523	-1.520	3.057	69.332	18.221	17.330
12482	273	42	35	C	S	0.000	5.011	2.165	-4.158	-4.554	-2.362	4.535	76.044	18.842	16.526
12483	273	42	35	C	S	7.154	-0.955	-1.120	1.418	0.973	1.089	-1.379	77.014	17.772	17.417
12484	273	42	35	C	S	8.948	1.048	2.016	-0.599	-1.026	-2.041	0.606	76.979	17.420	17.770
12485	273	42	35	C	S	51.052	1.048	-2.016	-0.599	-1.026	2.041	0.606	76.979	17.420	17.770
12486	273	42	35	C	S	52.846	-0.955	1.120	1.418	0.973	-1.089	-1.379	77.014	17.772	17.417
12487	273	42	35	C	S	60.000	5.011	2.165	-4.158	-4.554	-2.362	4.535	76.044	18.842	16.527
12488	273	42	35	C	S	67.154	-0.955	-1.120	1.418	0.973	1.089	-1.379	77.014	17.772	17.417
12489	273	42	35	C	S	68.948	1.048	2.016	-0.599	-1.026	-2.041	0.606	76.979	17.420	17.770
12490	273	42	35	C	S	111.052	1.048	-2.016	-0.599	-1.026	2.041	0.606	76.979	17.420	17.770
12491	273	42	35	C	S	112.846	-0.955	1.120	1.418	0.973	-1.089	-1.379	77.014	17.772	17.417
12492	273	42	35	C	W	0.000	5.011	2.165	-4.158	-4.554	-2.362	4.535	76.044	18.842	16.526
12493	273	42	35	C	W	7.154	-0.955	-1.120	1.418	0.973	1.089	-1.379	77.014	17.772	17.417
12494	273	42	35	C	W	8.948	1.048	2.016	-0.599	-1.026	-2.041	0.606	76.979	17.420	17.770
12495	273	42	35	C	W	51.052	1.048	-2.016	-0.599	-1.026	2.041	0.606	76.979	17.420	17.770

BL number	Atoms	γ-PC unit cells	WS_2 unit cells	γ-PC origin atom	WS_2 origin atom	Twist-angle (°)	γ-PC strain 1 (%)	γ-PC strain 2 (%)	γ-PC strain 3 (%)	WS_2 strain 1 (%)	WS_2 strain 2 (%)	WS_2 strain 3 (%)	γ (°)	$\lvert\mathbf{a}\rvert$(Å)	$\lvert\mathbf{b}\rvert$(Å)
12496	273	42	35	C	W	52.846	-0.955	1.120	1.418	0.973	-1.089	-1.379	77.014	17.772	17.417
12497	273	42	35	C	W	60.000	5.011	2.165	-4.158	-4.554	-2.362	4.535	76.044	18.842	16.527
12498	273	42	35	C	W	67.154	-0.955	-1.120	1.418	0.973	1.089	-1.379	77.014	17.772	17.417
12499	273	42	35	C	W	68.948	1.048	2.016	-0.599	-1.026	-2.041	0.606	76.979	17.420	17.770
12500	273	42	35	C	W	111.052	1.048	-2.016	-0.599	-1.026	2.041	0.606	76.979	17.420	17.770
12501	273	42	35	C	W	112.846	-0.955	1.120	1.418	0.973	-1.089	-1.379	77.014	17.772	17.417
12502	273	42	35	P	S	0.000	5.011	2.165	-4.158	-4.554	-2.362	4.535	76.044	18.842	16.526
12503	273	42	35	P	S	51.052	1.048	-2.016	-0.599	-1.026	2.041	0.606	76.979	17.420	17.770
12504	273	42	35	P	S	52.846	-0.955	1.120	1.418	0.973	-1.089	-1.379	77.014	17.772	17.417
12505	273	42	35	P	S	60.000	-4.158	-0.790	5.011	4.534	0.718	-4.554	85.266	16.522	18.353
12506	273	42	35	P	S	67.154	-0.955	-1.120	1.418	0.973	1.089	-1.379	77.014	17.772	17.417
12507	273	42	35	P	S	68.948	1.048	2.016	-0.599	-1.026	-2.041	0.606	76.979	17.420	17.770
12508	273	42	35	P	W	0.000	5.011	0.722	-4.158	-4.554	-0.787	4.535	85.265	18.353	16.523
12509	273	42	35	P	W	7.154	-0.955	-1.120	1.418	0.973	1.089	-1.379	77.014	17.772	17.417
12510	273	42	35	P	W	8.948	1.048	2.016	-0.599	-1.026	-2.041	0.606	76.979	17.420	17.770
12511	273	42	35	P	W	60.000	5.011	2.165	-4.158	-4.554	-2.362	4.535	76.044	18.842	16.527
12512	273	42	35	P	W	111.052	1.048	-2.016	-0.599	-1.026	2.041	0.606	76.979	17.420	17.770
12513	273	42	35	P	W	112.846	-0.955	1.120	1.418	0.973	-1.089	-1.379	77.014	17.772	17.417
12514	274	40	38	C	S	31.945	2.281	-0.475	4.989	-2.182	0.432	-4.536	84.920	16.362	18.842
12515	274	40	38	C	S	91.945	2.281	-0.475	4.989	-2.182	0.432	-4.536	84.920	16.362	18.842
12516	274	40	38	C	W	31.945	2.281	-0.475	4.989	-2.182	0.432	-4.536	84.920	16.362	18.842
12517	274	40	38	C	W	91.945	2.281	-0.475	4.989	-2.182	0.432	-4.536	84.920	16.362	18.842
12518	274	40	38	P	S	91.945	2.281	-0.475	4.989	-2.182	0.432	-4.536	84.920	16.362	18.842
12519	274	40	38	P	W	31.945	2.281	-0.475	4.989	-2.182	0.432	-4.536	84.920	16.362	18.842
12520	275	41	37	C	S	25.767	0.353	-3.692	4.236	-0.351	3.404	-3.905	68.126	17.539	18.838
12521	275	41	37	C	S	34.233	0.353	3.692	4.236	-0.351	-3.404	-3.905	68.126	17.539	18.838
12522	275	41	37	C	S	85.767	0.353	-3.692	4.236	-0.351	3.404	-3.905	68.126	17.539	18.838
12523	275	41	37	C	S	94.233	0.353	3.692	4.236	-0.351	-3.404	-3.905	68.126	17.539	18.838
12524	275	41	37	C	W	25.767	0.353	-3.692	4.236	-0.351	3.404	-3.905	68.126	17.539	18.838
12525	275	41	37	C	W	34.233	0.353	3.692	4.236	-0.351	-3.404	-3.905	68.126	17.539	18.838
12526	275	41	37	C	W	85.767	0.353	-3.692	4.236	-0.351	3.404	-3.905	68.126	17.539	18.838
12527	275	41	37	C	W	94.233	0.353	3.692	4.236	-0.351	-3.404	-3.905	68.126	17.539	18.838
12528	275	41	37	P	S	25.767	0.353	-3.692	4.236	-0.351	3.404	-3.905	68.126	17.539	18.838
12529	275	41	37	P	S	94.233	0.353	3.692	4.236	-0.351	-3.404	-3.905	68.126	17.539	18.838
12530	275	41	37	P	W	34.233	0.353	3.692	4.236	-0.351	-3.404	-3.905	68.126	17.539	18.838
12531	275	41	37	P	W	85.767	0.353	-3.692	4.236	-0.351	3.404	-3.905	68.126	17.539	18.838
12532	275	44	33	C	S	4.715	-3.007	0.636	-1.702	3.200	-0.659	1.762	64.706	16.951	19.569
12533	275	44	33	C	S	19.842	-4.723	4.733	0.128	5.216	-4.721	-0.128	84.144	15.242	19.792
12534	275	44	33	C	S	40.158	-4.723	-4.733	0.128	5.216	4.721	-0.128	84.144	15.242	19.792
12535	275	44	33	C	S	55.285	-1.702	-0.627	-3.007	1.762	0.668	3.200	64.720	19.567	16.951
12536	275	44	33	C	S	64.715	-3.007	0.636	-1.702	3.200	-0.659	1.762	64.706	16.951	19.569
12537	275	44	33	C	S	79.842	-4.723	4.733	0.128	5.216	-4.721	-0.128	84.144	15.242	19.792
12538	275	44	33	C	S	100.158	-4.723	-4.733	0.128	5.216	4.721	-0.128	84.144	15.242	19.792
12539	275	44	33	C	S	115.285	-1.702	-0.627	-3.007	1.762	0.668	3.200	64.720	19.567	16.951
12540	275	44	33	C	W	4.715	-3.007	0.636	-1.702	3.200	-0.659	1.762	64.706	16.951	19.569
12541	275	44	33	C	W	19.842	-4.723	4.733	0.128	5.216	-4.721	-0.128	84.144	15.242	19.792
12542	275	44	33	C	W	40.158	-4.723	-4.733	0.128	5.216	4.721	-0.128	84.144	15.242	19.792
12543	275	44	33	C	W	55.285	-1.702	-0.627	-3.007	1.762	0.668	3.200	64.720	19.567	16.951
12544	275	44	33	C	W	64.715	-3.007	0.636	-1.702	3.200	-0.659	1.762	64.706	16.951	19.569
12545	275	44	33	C	W	79.842	-4.723	4.733	0.128	5.216	-4.721	-0.128	84.144	15.242	19.792
12546	275	44	33	C	W	100.158	-4.723	-4.733	0.128	5.216	4.721	-0.128	84.144	15.242	19.792

BL number	Atoms	γ-PC unit cells	WS_2 unit cells	γ-PC origin atom	WS_2 origin atom	Twist-angle (°)	γ-PC strain 1 (%)	γ-PC strain 2 (%)	γ-PC strain 3 (%)	WS_2 strain 1 (%)	WS_2 strain 2 (%)	WS_2 strain 3 (%)	γ (°)	$\lvert\mathbf{a}\rvert$(Å)	$\lvert\mathbf{b}\rvert$(Å)
12547	275	44	33	C	W	115.285	-3.007	-0.636	-1.702	3.200	0.659	1.762	64.706	16.951	19.569
12548	275	44	33	P	S	4.715	-3.007	0.636	-1.702	3.200	-0.659	1.762	64.706	16.951	19.569
12549	275	44	33	P	S	40.158	-4.723	-4.733	0.128	5.216	4.721	-0.128	84.144	15.242	19.792
12550	275	44	33	P	S	55.285	-1.702	-0.627	-3.007	1.762	0.668	3.200	64.720	19.567	16.951
12551	275	44	33	P	S	64.715	-3.007	0.636	-1.702	3.200	-0.659	1.762	64.706	16.951	19.569
12552	275	44	33	P	S	79.842	-4.723	4.733	0.128	5.216	-4.721	-0.128	84.144	15.242	19.792
12553	275	44	33	P	S	115.285	-3.007	-0.636	-1.702	3.200	0.659	1.762	64.706	16.951	19.569
12554	275	44	33	P	W	4.715	-3.007	0.636	-1.702	3.200	-0.659	1.762	64.706	16.951	19.569
12555	275	44	33	P	W	19.842	-4.723	4.733	0.128	5.216	-4.721	-0.128	84.144	15.242	19.792
12556	275	44	33	P	W	55.285	-1.702	-0.627	-3.007	1.762	0.668	3.200	64.720	19.567	16.951
12557	275	44	33	P	W	64.715	-3.007	0.636	-1.702	3.200	-0.659	1.762	64.706	16.951	19.569
12558	275	44	33	P	W	79.842	-4.723	4.733	0.128	5.216	-4.721	-0.128	84.144	15.242	19.792
12559	275	44	33	P	W	100.158	-4.723	-4.733	0.128	5.216	4.721	-0.128	84.144	15.242	19.792
12560	275	44	33	P	W	115.285	-1.702	-0.627	-3.007	1.762	0.668	3.200	64.720	19.567	16.951
12561	276	42	36	C	S	7.589	0.334	-0.920	1.533	-0.332	0.892	-1.487	68.274	18.904	17.419
12562	276	42	36	C	S	8.948	1.048	1.479	0.813	-1.026	-1.455	-0.800	68.263	17.420	18.903
12563	276	42	36	C	S	51.052	1.048	-1.478	0.813	-1.026	1.455	-0.800	68.263	17.420	18.903
12564	276	42	36	C	S	52.411	0.334	0.920	1.533	-0.332	-0.892	-1.487	68.274	18.904	17.419
12565	276	42	36	C	S	67.589	0.334	-0.920	1.533	-0.332	0.892	-1.487	68.274	18.904	17.419
12566	276	42	36	C	S	68.948	1.048	1.479	0.813	-1.026	-1.455	-0.800	68.263	17.420	18.903
12567	276	42	36	C	S	111.052	1.048	-1.478	0.813	-1.026	1.455	-0.800	68.263	17.420	18.903
12568	276	42	36	C	S	112.411	0.334	0.920	1.533	-0.332	-0.892	-1.487	68.274	18.904	17.419
12569	276	42	36	C	W	7.589	0.334	-0.920	1.533	-0.332	0.892	-1.487	68.274	18.904	17.419
12570	276	42	36	C	W	8.948	1.048	1.479	0.813	-1.026	-1.455	-0.800	68.263	17.420	18.903
12571	276	42	36	C	W	51.052	1.048	-1.478	0.813	-1.026	1.455	-0.800	68.263	17.420	18.903
12572	276	42	36	C	W	52.411	0.334	0.920	1.533	-0.332	-0.892	-1.487	68.274	18.904	17.419
12573	276	42	36	C	W	67.589	0.334	-0.920	1.533	-0.332	0.892	-1.487	68.274	18.904	17.419
12574	276	42	36	C	W	68.948	1.048	1.479	0.813	-1.026	-1.455	-0.800	68.263	17.420	18.903
12575	276	42	36	C	W	111.052	1.048	-1.478	0.813	-1.026	1.455	-0.800	68.263	17.420	18.903
12576	276	42	36	C	W	112.411	0.334	0.920	1.533	-0.332	-0.892	-1.487	68.274	18.904	17.419
12577	276	42	36	P	S	7.589	0.334	-0.920	1.533	-0.332	0.892	-1.487	68.274	18.904	17.419
12578	276	42	36	P	S	8.948	1.048	1.479	0.813	-1.026	-1.455	-0.800	68.263	17.420	18.903
12579	276	42	36	P	S	51.052	1.048	-1.478	0.813	-1.026	1.455	-0.800	68.263	17.420	18.903
12580	276	42	36	P	S	52.411	0.334	0.920	1.533	-0.332	-0.892	-1.487	68.274	18.904	17.419
12581	276	42	36	P	S	67.589	0.334	-0.920	1.533	-0.332	0.892	-1.487	68.274	18.904	17.419
12582	276	42	36	P	S	68.948	1.048	1.479	0.813	-1.026	-1.455	-0.800	68.263	17.420	18.903
12583	276	42	36	P	S	111.052	1.048	-1.478	0.813	-1.026	1.455	-0.800	68.263	17.420	18.903
12584	276	42	36	P	S	112.411	0.334	0.920	1.533	-0.332	-0.892	-1.487	68.274	18.904	17.419
12585	276	42	36	P	W	7.589	0.334	-0.920	1.533	-0.332	0.892	-1.487	68.274	18.904	17.419
12586	276	42	36	P	W	8.948	1.048	1.479	0.813	-1.026	-1.455	-0.800	68.263	17.420	18.903
12587	276	42	36	P	W	51.052	1.048	-1.478	0.813	-1.026	1.455	-0.800	68.263	17.420	18.903
12588	276	42	36	P	W	52.411	0.334	0.920	1.533	-0.332	-0.892	-1.487	68.274	18.904	17.419
12589	276	42	36	P	W	67.589	0.334	-0.920	1.533	-0.332	0.892	-1.487	68.274	18.904	17.419
12590	276	42	36	P	W	68.948	1.048	1.479	0.813	-1.026	-1.455	-0.800	68.263	17.420	18.903
12591	276	42	36	P	W	111.052	1.048	-1.478	0.813	-1.026	1.455	-0.800	68.263	17.420	18.903
12592	276	42	36	P	W	112.411	0.334	0.920	1.533	-0.332	-0.892	-1.487	68.274	18.904	17.419
12593	276	45	32	C	S	56.996	-3.388	-5.047	-3.832	3.635	5.465	4.150	84.792	17.335	17.314
12594	276	45	32	C	S	63.005	-3.388	5.047	-3.832	3.635	-5.466	4.150	84.792	17.335	17.314
12595	276	45	32	C	S	116.996	-3.388	-5.047	-3.832	3.635	5.465	4.150	84.792	17.335	17.314
12596	276	45	32	C	W	56.996	-3.388	-5.047	-3.832	3.635	5.465	4.150	84.792	17.335	17.314
12597	276	45	32	C	W	63.005	-3.388	5.047	-3.832	3.635	-5.466	4.150	84.792	17.335	17.314

BL number	Atoms	γ-PC unit cells	WS_2 unit cells	γ-PC origin atom	WS_2 origin atom	Twist-angle (°)	γ-PC strain 1 (%)	γ-PC strain 2 (%)	γ-PC strain 3 (%)	WS_2 strain 1 (%)	WS_2 strain 2 (%)	WS_2 strain 3 (%)	γ (°)	$\lvert\mathbf{a}\rvert$(Å)	$\lvert\mathbf{b}\rvert$(Å)
12598	276	45	32	C	W	116.996	-3.388	-5.047	-3.832	3.635	5.465	4.150	84.792	17.335	17.314
12599	276	45	32	P	S	56.996	-3.388	-5.047	-3.832	3.635	5.465	4.150	84.792	17.335	17.314
12600	276	45	32	P	S	63.005	-3.388	5.047	-3.832	3.635	-5.466	4.150	84.792	17.335	17.314
12601	276	45	32	P	W	116.996	-3.388	-5.047	-3.832	3.635	5.465	4.150	84.792	17.335	17.314
12602	277	40	39	C	S	18.613	3.582	0.545	5.066	-3.342	-0.495	-4.600	85.415	18.586	16.799
12603	277	40	39	C	S	41.387	3.582	-0.545	5.066	-3.342	0.495	-4.600	85.415	18.586	16.799
12604	277	40	39	C	S	42.103	5.011	0.758	3.636	-4.554	-0.707	-3.389	85.401	16.799	18.586
12605	277	40	39	C	S	77.897	5.011	-0.758	3.636	-4.555	0.706	-3.389	85.401	16.799	18.586
12606	277	40	39	C	S	78.613	3.582	0.545	5.066	-3.342	-0.495	-4.600	85.416	18.586	16.799
12607	277	40	39	C	S	102.104	5.011	0.758	3.636	-4.554	-0.707	-3.389	85.401	16.799	18.586
12608	277	40	39	C	W	17.897	5.011	-0.758	3.636	-4.555	0.706	-3.389	85.401	16.799	18.586
12609	277	40	39	C	W	18.613	3.582	0.545	5.066	-3.342	-0.495	-4.600	85.415	18.586	16.799
12610	277	40	39	C	W	42.103	5.011	0.758	3.636	-4.554	-0.707	-3.389	85.401	16.799	18.586
12611	277	40	39	C	W	78.613	3.582	0.545	5.066	-3.342	-0.495	-4.600	85.416	18.586	16.799
12612	277	40	39	C	W	101.387	3.582	-0.545	5.066	-3.342	0.495	-4.600	85.415	18.586	16.799
12613	277	40	39	C	W	102.104	5.011	0.758	3.636	-4.554	-0.707	-3.389	85.401	16.799	18.586
12614	277	40	39	P	S	17.897	5.011	-0.758	3.636	-4.555	0.706	-3.389	85.401	16.799	18.586
12615	277	40	39	P	S	18.613	3.582	0.545	5.066	-3.342	-0.495	-4.600	85.415	18.586	16.799
12616	277	40	39	P	S	42.103	5.011	0.758	3.636	-4.554	-0.707	-3.389	85.401	16.799	18.586
12617	277	40	39	P	S	78.613	3.582	0.545	5.066	-3.342	-0.495	-4.600	85.416	18.586	16.799
12618	277	40	39	P	S	101.387	3.582	-0.545	5.066	-3.342	0.495	-4.600	85.415	18.586	16.799
12619	277	40	39	P	S	102.104	5.011	0.758	3.636	-4.554	-0.707	-3.389	85.401	16.799	18.586
12620	277	40	39	P	W	18.613	3.582	0.545	5.066	-3.342	-0.495	-4.600	85.415	18.586	16.799
12621	277	40	39	P	W	41.387	3.582	-0.545	5.066	-3.342	0.495	-4.600	85.415	18.586	16.799
12622	277	40	39	P	W	42.103	5.011	0.758	3.636	-4.554	-0.707	-3.389	85.401	16.799	18.586
12623	277	40	39	P	W	77.897	5.011	-0.758	3.636	-4.555	0.706	-3.389	85.401	16.799	18.586
12624	277	40	39	P	W	78.613	3.582	0.545	5.066	-3.342	-0.495	-4.600	85.416	18.586	16.799
12625	277	40	39	P	W	102.104	5.011	0.758	3.636	-4.554	-0.707	-3.389	85.401	16.799	18.586
12626	277	43	35	C	S	25.050	-0.955	-0.901	0.223	0.973	0.897	-0.222	78.249	17.772	17.539
12627	277	43	35	C	S	25.285	-4.781	-4.603	4.473	5.287	4.225	-4.106	77.177	16.641	18.847
12628	277	43	35	C	S	25.767	0.353	0.348	-1.082	-0.351	-0.355	1.106	78.259	17.539	17.771
12629	277	43	35	C	S	34.715	-4.781	4.603	4.473	5.287	-4.225	-4.106	77.177	16.641	18.847
12630	277	43	35	C	S	34.950	-0.955	0.901	0.223	0.973	-0.897	-0.222	78.249	17.772	17.539
12631	277	43	35	C	S	85.285	-4.781	-4.603	4.473	5.287	4.225	-4.106	77.177	16.641	18.847
12632	277	43	35	C	S	85.767	0.353	0.348	-1.082	-0.351	-0.355	1.106	78.259	17.539	17.771
12633	277	43	35	C	S	94.233	0.353	-0.348	-1.082	-0.351	0.355	1.106	78.259	17.539	17.771
12634	277	43	35	C	S	94.715	-4.781	4.603	4.473	5.287	-4.225	-4.106	77.177	16.641	18.847
12635	277	43	35	C	S	94.950	-0.955	0.901	0.223	0.973	-0.897	-0.222	78.249	17.772	17.539
12636	277	43	35	C	W	25.285	-4.781	-4.603	4.473	5.287	4.225	-4.106	77.177	16.641	18.847
12637	277	43	35	C	W	25.767	0.353	0.348	-1.082	-0.351	-0.355	1.106	78.259	17.539	17.771
12638	277	43	35	C	W	34.233	0.353	-0.348	-1.082	-0.351	0.355	1.106	78.259	17.539	17.771
12639	277	43	35	C	W	34.715	-4.781	4.603	4.473	5.287	-4.225	-4.106	77.177	16.641	18.847
12640	277	43	35	C	W	34.950	-0.955	0.901	0.223	0.973	-0.897	-0.222	78.249	17.772	17.539
12641	277	43	35	C	W	85.050	-0.955	-0.901	0.223	0.973	0.897	-0.222	78.249	17.772	17.539
12642	277	43	35	C	W	85.285	-4.781	-4.603	4.473	5.287	4.225	-4.106	77.177	16.641	18.847
12643	277	43	35	C	W	85.767	0.353	0.348	-1.082	-0.351	-0.355	1.106	78.259	17.539	17.771
12644	277	43	35	C	W	94.715	-4.781	4.603	4.473	5.287	-4.225	-4.106	77.177	16.641	18.847
12645	277	43	35	C	W	94.950	-0.955	0.901	0.223	0.973	-0.897	-0.222	78.249	17.772	17.539
12646	277	43	35	P	S	25.285	-4.781	-4.603	4.473	5.287	4.225	-4.106	77.177	16.641	18.847
12647	277	43	35	P	S	25.767	0.353	0.348	-1.082	-0.351	-0.355	1.106	78.259	17.539	17.771
12648	277	43	35	P	S	34.233	0.353	-0.348	-1.082	-0.351	0.355	1.106	78.259	17.539	17.771

BL number	Atoms	γ-PC unit cells	WS_2 unit cells	γ-PC origin atom	WS_2 origin atom	Twist-angle (°)	γ-PC strain 1 (%)	γ-PC strain 2 (%)	γ-PC strain 3 (%)	WS_2 strain 1 (%)	WS_2 strain 2 (%)	WS_2 strain 3 (%)	γ (°)	|**a**|(Å)	|**b**|(Å)
12649	277	43	35	P	S	34.950	-0.955	0.901	0.223	0.973	-0.897	-0.222	78.249	17.772	17.539
12650	277	43	35	P	S	85.050	-0.955	-0.901	0.223	0.973	0.897	-0.222	78.249	17.772	17.539
12651	277	43	35	P	S	85.767	0.353	0.348	-1.082	-0.351	-0.355	1.106	78.259	17.539	17.771
12652	277	43	35	P	S	94.715	-4.781	4.603	4.473	5.287	-4.225	-4.106	77.177	16.641	18.847
12653	277	43	35	P	S	94.950	-0.955	0.901	0.223	0.973	-0.897	-0.222	78.249	17.772	17.539
12654	277	43	35	P	W	25.050	-0.955	-0.901	0.223	0.973	0.897	-0.222	78.249	17.772	17.539
12655	277	43	35	P	W	25.767	0.353	0.348	-1.082	-0.351	-0.355	1.106	78.259	17.539	17.771
12656	277	43	35	P	W	34.715	-4.781	4.603	4.473	5.287	-4.225	-4.106	77.177	16.641	18.847
12657	277	43	35	P	W	34.950	-0.955	0.901	0.223	0.973	-0.897	-0.222	78.249	17.772	17.539
12658	277	43	35	P	W	85.285	-4.781	-4.603	4.473	5.287	4.225	-4.106	77.177	16.641	18.847
12659	277	43	35	P	W	85.767	0.353	0.348	-1.082	-0.351	-0.355	1.106	78.259	17.539	17.771
12660	277	43	35	P	W	94.233	0.353	-0.348	-1.082	-0.351	0.355	1.106	78.259	17.539	17.771
12661	277	43	35	P	W	94.950	-0.955	0.901	0.223	0.973	-0.897	-0.222	78.249	17.772	17.539
12662	278	41	38	C	S	17.696	1.029	-1.134	4.965	-1.008	1.032	-4.516	76.438	19.034	16.798
12663	278	41	38	C	S	17.897	5.011	-0.739	0.986	-4.554	0.725	-0.967	76.417	16.799	19.036
12664	278	41	38	C	S	42.103	5.011	0.739	0.986	-4.554	-0.725	-0.967	76.417	16.799	19.036
12665	278	41	38	C	S	77.696	1.029	-1.134	4.965	-1.008	1.032	-4.516	76.438	19.034	16.798
12666	278	41	38	C	S	77.897	5.011	-0.739	0.986	-4.554	0.725	-0.967	76.417	16.799	19.036
12667	278	41	38	C	S	102.104	5.011	0.739	0.986	-4.554	-0.725	-0.967	76.417	16.799	19.036
12668	278	41	38	C	S	102.304	1.029	1.134	4.964	-1.008	-1.032	-4.516	76.438	19.034	16.798
12669	278	41	38	C	W	17.696	1.029	-1.134	4.965	-1.008	1.032	-4.516	76.438	19.034	16.798
12670	278	41	38	C	W	17.897	5.011	-0.739	0.986	-4.554	0.725	-0.967	76.417	16.799	19.036
12671	278	41	38	C	W	42.103	5.011	0.739	0.986	-4.554	-0.725	-0.967	76.417	16.799	19.036
12672	278	41	38	C	W	42.304	1.029	1.134	4.964	-1.008	-1.032	-4.516	76.438	19.034	16.798
12673	278	41	38	C	W	77.696	1.029	-1.134	4.965	-1.008	1.032	-4.516	76.438	19.034	16.798
12674	278	41	38	C	W	77.897	5.011	-0.739	0.986	-4.554	0.725	-0.967	76.417	16.799	19.036
12675	278	41	38	C	W	102.104	5.011	0.739	0.986	-4.554	-0.725	-0.967	76.417	16.799	19.036
12676	278	41	38	C	W	102.304	1.029	1.134	4.964	-1.008	-1.032	-4.516	76.438	19.034	16.798
12677	278	41	38	P	S	17.696	1.029	-1.134	4.965	-1.008	1.032	-4.516	76.438	19.034	16.798
12678	278	41	38	P	S	17.897	5.011	-0.739	0.986	-4.554	0.725	-0.967	76.417	16.799	19.036
12679	278	41	38	P	S	42.103	5.011	0.739	0.986	-4.554	-0.725	-0.967	76.417	16.799	19.036
12680	278	41	38	P	S	42.304	1.029	1.134	4.964	-1.008	-1.032	-4.516	76.438	19.034	16.798
12681	278	41	38	P	S	77.696	1.029	-1.134	4.965	-1.008	1.032	-4.516	76.438	19.034	16.798
12682	278	41	38	P	S	77.897	5.011	-0.739	0.986	-4.554	0.725	-0.967	76.417	16.799	19.036
12683	278	41	38	P	S	102.104	5.011	0.739	0.986	-4.554	-0.725	-0.967	76.417	16.799	19.036
12684	278	41	38	P	S	102.304	1.029	1.134	4.964	-1.008	-1.032	-4.516	76.438	19.034	16.798
12685	278	41	38	P	W	17.696	1.029	-1.134	4.965	-1.008	1.032	-4.516	76.438	19.034	16.798
12686	278	41	38	P	W	17.897	5.011	-0.739	0.986	-4.554	0.725	-0.967	76.417	16.799	19.036
12687	278	41	38	P	W	42.103	5.011	0.739	0.986	-4.554	-0.725	-0.967	76.417	16.799	19.036
12688	278	41	38	P	W	77.696	1.029	-1.134	4.965	-1.008	1.032	-4.516	76.438	19.034	16.798
12689	278	41	38	P	W	77.897	5.011	-0.739	0.986	-4.554	0.725	-0.967	76.417	16.799	19.036
12690	278	41	38	P	W	102.104	5.011	0.739	0.986	-4.554	-0.725	-0.967	76.417	16.799	19.036
12691	278	41	38	P	W	102.304	1.029	1.134	4.964	-1.008	-1.032	-4.516	76.438	19.034	16.798
12692	278	44	34	C	S	4.715	-1.702	-1.464	-1.583	1.762	1.512	1.635	65.443	19.567	17.099
12693	278	44	34	C	S	6.178	-2.145	1.055	-1.135	2.241	-1.079	1.161	65.445	17.102	19.564
12694	278	44	34	C	S	13.898	-0.414	-0.603	-2.841	0.417	0.639	3.012	64.809	17.100	19.669
12695	278	44	34	C	S	15.608	-2.145	2.339	-1.135	2.241	-2.393	1.161	64.833	17.102	19.661
12696	278	44	34	C	S	24.791	-3.388	-3.939	0.168	3.635	3.926	-0.168	67.267	17.335	19.040
12697	278	44	34	C	S	27.126	1.029	0.143	-4.174	-1.008	-0.156	4.555	67.387	19.034	17.331
12698	278	44	34	C	S	32.874	1.029	-0.143	-4.174	-1.008	0.156	4.555	67.387	19.034	17.331
12699	278	44	34	C	S	35.209	-3.388	3.939	0.168	3.635	-3.926	-0.168	67.267	17.335	19.040

BL number	Atoms	γ-PC unit cells	WS_2 unit cells	γ-PC origin atom	WS_2 origin atom	Twist-angle (°)	γ-PC strain 1 (%)	γ-PC strain 2 (%)	γ-PC strain 3 (%)	WS_2 strain 1 (%)	WS_2 strain 2 (%)	WS_2 strain 3 (%)	γ (°)	|**a**|(Å)	|**b**|(Å)
12700	278	44	34	C	S	44.392	-2.145	-2.339	-1.135	2.241	2.393	1.161	64.833	17.102	19.661
12701	278	44	34	C	S	46.102	-0.414	0.603	-2.841	0.417	-0.639	3.012	64.809	19.669	17.100
12702	278	44	34	C	S	53.822	-2.145	-1.055	-1.135	2.241	1.079	1.161	65.445	17.102	19.564
12703	278	44	34	C	S	55.285	-1.702	1.464	-1.583	1.762	-1.512	1.635	65.443	19.567	17.099
12704	278	44	34	C	S	64.715	-1.702	-1.464	-1.583	1.762	1.512	1.635	65.443	19.567	17.099
12705	278	44	34	C	S	66.178	-2.145	1.055	-1.135	2.241	-1.079	1.161	65.445	17.102	19.564
12706	278	44	34	C	S	73.898	-0.414	-0.603	-2.841	0.417	0.639	3.012	64.809	17.100	19.669
12707	278	44	34	C	S	75.609	-2.145	2.339	-1.135	2.241	-2.393	1.161	64.833	17.102	19.661
12708	278	44	34	C	S	84.791	-3.388	-3.939	0.168	3.635	3.926	-0.168	67.267	17.335	19.040
12709	278	44	34	C	S	87.126	1.029	0.143	-4.174	-1.008	-0.156	4.555	67.387	19.034	17.331
12710	278	44	34	C	S	92.874	1.029	-0.143	-4.174	-1.008	0.156	4.555	67.387	19.034	17.331
12711	278	44	34	C	S	95.209	-3.388	3.939	0.168	3.635	-3.926	-0.168	67.267	17.335	19.040
12712	278	44	34	C	S	104.392	-2.145	-2.339	-1.135	2.241	2.393	1.161	64.833	17.102	19.661
12713	278	44	34	C	S	106.102	-0.414	0.603	-2.841	0.417	-0.639	3.012	64.809	19.669	17.100
12714	278	44	34	C	S	113.822	-2.145	-1.055	-1.135	2.241	1.079	1.161	65.445	17.102	19.564
12715	278	44	34	C	W	4.715	-1.702	-1.464	-1.583	1.762	1.512	1.635	65.443	19.567	17.099
12716	278	44	34	C	W	6.178	-2.145	1.055	-1.135	2.241	-1.079	1.161	65.445	17.102	19.564
12717	278	44	34	C	W	13.898	-0.414	-0.603	-2.841	0.417	0.639	3.012	64.809	17.100	19.669
12718	278	44	34	C	W	15.608	-2.145	2.339	-1.135	2.241	-2.393	1.161	64.833	17.102	19.661
12719	278	44	34	C	W	24.791	-3.388	-3.939	0.168	3.635	3.926	-0.168	67.267	17.335	19.040
12720	278	44	34	C	W	27.126	1.029	0.143	-4.174	-1.008	-0.156	4.555	67.387	19.034	17.331
12721	278	44	34	C	W	32.874	1.029	-0.143	-4.174	-1.008	0.156	4.555	67.387	19.034	17.331
12722	278	44	34	C	W	35.209	-3.388	3.939	0.168	3.635	-3.926	-0.168	67.267	17.335	19.040
12723	278	44	34	C	W	44.392	-2.145	-2.339	-1.135	2.241	2.393	1.161	64.833	17.102	19.661
12724	278	44	34	C	W	46.102	-0.414	0.603	-2.841	0.417	-0.639	3.012	64.809	19.669	17.100
12725	278	44	34	C	W	53.822	-2.145	-1.055	-1.135	2.241	1.079	1.161	65.445	17.102	19.564
12726	278	44	34	C	W	64.715	-1.702	-1.464	-1.583	1.762	1.512	1.635	65.443	19.567	17.099
12727	278	44	34	C	W	66.178	-2.145	1.055	-1.135	2.241	-1.079	1.161	65.445	17.102	19.564
12728	278	44	34	C	W	73.898	-0.414	-0.603	-2.841	0.417	0.639	3.012	64.809	17.100	19.669
12729	278	44	34	C	W	75.609	-2.145	2.339	-1.135	2.241	-2.393	1.161	64.833	17.102	19.661
12730	278	44	34	C	W	84.791	-3.388	-3.939	0.168	3.635	3.926	-0.168	67.267	17.335	19.040
12731	278	44	34	C	W	87.126	1.029	0.143	-4.174	-1.008	-0.156	4.555	67.387	19.034	17.331
12732	278	44	34	C	W	92.874	1.029	-0.143	-4.174	-1.008	0.156	4.555	67.387	19.034	17.331
12733	278	44	34	C	W	95.209	-3.388	3.939	0.168	3.635	-3.926	-0.168	67.267	17.335	19.040
12734	278	44	34	C	W	104.392	-2.145	-2.339	-1.135	2.241	2.393	1.161	64.833	17.102	19.661
12735	278	44	34	C	W	106.102	-0.414	0.603	-2.841	0.417	-0.639	3.012	64.809	19.669	17.100
12736	278	44	34	C	W	113.822	-2.145	-1.055	-1.135	2.241	1.079	1.161	65.445	17.102	19.564
12737	278	44	34	C	W	115.285	-1.702	1.464	-1.583	1.762	-1.512	1.635	65.443	19.567	17.099
12738	278	44	34	P	S	4.715	-1.702	-1.464	-1.583	1.762	1.512	1.635	65.443	19.567	17.099
12739	278	44	34	P	S	6.178	-2.145	1.055	-1.135	2.241	-1.079	1.161	65.445	17.102	19.564
12740	278	44	34	P	S	24.791	-3.388	-3.939	0.168	3.635	3.926	-0.168	67.267	17.335	19.040
12741	278	44	34	P	S	27.126	1.029	0.143	-4.174	-1.008	-0.156	4.555	67.387	19.034	17.331
12742	278	44	34	P	S	32.874	1.029	-0.143	-4.174	-1.008	0.156	4.555	67.387	19.034	17.331
12743	278	44	34	P	S	35.209	-3.388	3.939	0.168	3.635	-3.926	-0.168	67.267	17.335	19.040
12744	278	44	34	P	S	44.392	-2.145	-2.339	-1.135	2.241	2.393	1.161	64.833	17.102	19.661
12745	278	44	34	P	S	46.102	-0.414	0.603	-2.841	0.417	-0.639	3.012	64.809	19.669	17.100
12746	278	44	34	P	S	53.822	-2.145	-1.055	-1.135	2.241	1.079	1.161	65.445	17.102	19.564
12747	278	44	34	P	S	64.715	-1.702	-1.464	-1.583	1.762	1.512	1.635	65.443	19.567	17.099
12748	278	44	34	P	S	66.178	-2.145	1.055	-1.135	2.241	-1.079	1.161	65.445	17.102	19.564
12749	278	44	34	P	S	73.898	-0.414	-0.603	-2.841	0.417	0.639	3.012	64.809	17.100	19.669
12750	278	44	34	P	S	75.609	-2.145	2.339	-1.135	2.241	-2.393	1.161	64.833	17.102	19.661

BL number	Atoms	γ-PC unit cells	WS_2 unit cells	γ-PC origin atom	WS_2 origin atom	Twist-angle (°)	γ-PC strain 1 (%)	γ-PC strain 2 (%)	γ-PC strain 3 (%)	WS_2 strain 1 (%)	WS_2 strain 2 (%)	WS_2 strain 3 (%)	γ (°)	|**a**|(Å)	|**b**|(Å)
12751	278	44	34	P	S	84.791	-3.388	-3.939	0.168	3.635	3.926	-0.168	67.267	17.335	19.040
12752	278	44	34	P	S	87.126	1.029	0.143	-4.174	-1.008	-0.156	4.555	67.387	19.034	17.331
12753	278	44	34	P	S	92.874	1.029	-0.143	-4.174	-1.008	0.156	4.555	67.387	19.034	17.331
12754	278	44	34	P	S	95.209	-3.388	3.939	0.168	3.635	-3.926	-0.168	67.267	17.335	19.040
12755	278	44	34	P	S	106.102	-0.414	0.603	-2.841	0.417	-0.639	3.012	64.809	19.669	17.100
12756	278	44	34	P	S	113.822	-2.145	-1.055	-1.135	2.241	1.079	1.161	65.445	17.102	19.564
12757	278	44	34	P	S	115.285	-1.702	1.464	-1.583	1.762	-1.512	1.635	65.443	19.567	17.099
12758	278	44	34	P	W	4.715	-1.702	-1.464	-1.583	1.762	1.512	1.635	65.443	19.567	17.099
12759	278	44	34	P	W	6.178	-2.145	1.055	-1.135	2.241	-1.079	1.161	65.445	17.102	19.564
12760	278	44	34	P	W	13.898	-0.414	-0.603	-2.841	0.417	0.639	3.012	64.809	17.100	19.669
12761	278	44	34	P	W	15.608	-2.145	2.339	-1.135	2.241	-2.393	1.161	64.833	17.102	19.661
12762	278	44	34	P	W	24.791	-3.388	-3.939	0.168	3.635	3.926	-0.168	67.267	17.335	19.040
12763	278	44	34	P	W	27.126	1.029	0.143	-4.174	-1.008	-0.156	4.555	67.387	19.034	17.331
12764	278	44	34	P	W	32.874	1.029	-0.143	-4.174	-1.008	0.156	4.555	67.387	19.034	17.331
12765	278	44	34	P	W	35.209	-3.388	3.939	0.168	3.635	-3.926	-0.168	67.267	17.335	19.040
12766	278	44	34	P	W	53.822	-2.145	-1.055	-1.135	2.241	1.079	1.161	65.445	17.102	19.564
12767	278	44	34	P	W	55.285	-1.702	1.464	-1.583	1.762	-1.512	1.635	65.443	19.567	17.099
12768	278	44	34	P	W	64.715	-1.702	-1.464	-1.583	1.762	1.512	1.635	65.443	19.567	17.099
12769	278	44	34	P	W	66.178	-2.145	1.055	-1.135	2.241	-1.079	1.161	65.445	17.102	19.564
12770	278	44	34	P	W	84.791	-3.388	-3.939	0.168	3.635	3.926	-0.168	67.267	17.335	19.040
12771	278	44	34	P	W	87.126	1.029	0.143	-4.174	-1.008	-0.156	4.555	67.387	19.034	17.331
12772	278	44	34	P	W	92.874	1.029	-0.143	-4.174	-1.008	0.156	4.555	67.387	19.034	17.331
12773	278	44	34	P	W	95.209	-3.388	3.939	0.168	3.635	-3.926	-0.168	67.267	17.335	19.040
12774	278	44	34	P	W	104.392	-2.145	-2.339	-1.135	2.241	2.393	1.161	64.833	17.102	19.661
12775	278	44	34	P	W	106.102	-0.414	0.603	-2.841	0.417	-0.639	3.012	64.809	19.669	17.100
12776	278	44	34	P	W	113.822	-2.145	-1.055	-1.135	2.241	1.079	1.161	65.445	17.102	19.564
12777	279	42	37	C	S	8.513	2.390	0.162	0.887	-2.281	-0.160	-0.871	67.367	19.291	17.420
12778	279	42	37	C	S	51.487	2.390	-0.162	0.887	-2.281	0.159	-0.871	67.367	19.291	17.420
12779	279	42	37	C	S	68.513	2.390	0.162	0.887	-2.281	-0.160	-0.871	67.367	19.291	17.420
12780	279	42	37	C	S	111.487	2.390	-0.162	0.887	-2.281	0.159	-0.871	67.367	19.291	17.420
12781	279	42	37	C	W	8.513	2.390	0.162	0.887	-2.281	-0.160	-0.871	67.367	19.291	17.420
12782	279	42	37	C	W	51.487	2.390	-0.162	0.887	-2.281	0.159	-0.871	67.367	19.291	17.420
12783	279	42	37	C	W	68.513	2.390	0.162	0.887	-2.281	-0.160	-0.871	67.367	19.291	17.420
12784	279	42	37	C	W	111.487	2.390	-0.162	0.887	-2.281	0.159	-0.871	67.367	19.291	17.420
12785	279	42	37	P	S	8.513	2.390	0.162	0.887	-2.281	-0.160	-0.871	67.367	19.291	17.420
12786	279	42	37	P	S	51.487	2.390	-0.162	0.887	-2.281	0.159	-0.871	67.367	19.291	17.420
12787	279	42	37	P	S	68.513	2.390	0.162	0.887	-2.281	-0.160	-0.871	67.367	19.291	17.420
12788	279	42	37	P	S	111.487	2.390	-0.162	0.887	-2.281	0.159	-0.871	67.367	19.291	17.420
12789	279	42	37	P	W	8.513	2.390	0.162	0.887	-2.281	-0.160	-0.871	67.367	19.291	17.420
12790	279	42	37	P	W	51.487	2.390	-0.162	0.887	-2.281	0.159	-0.871	67.367	19.291	17.420
12791	279	42	37	P	W	68.513	2.390	0.162	0.887	-2.281	-0.160	-0.871	67.367	19.291	17.420
12792	279	42	37	P	W	111.487	2.390	-0.162	0.887	-2.281	0.159	-0.871	67.367	19.291	17.420
12793	279	45	33	C	S	1.359	-3.292	-2.802	-2.488	3.523	2.949	2.618	79.376	18.221	16.941
12794	279	45	33	C	S	4.715	-3.007	2.904	-2.775	3.200	-3.074	2.938	79.368	16.951	18.210
12795	279	45	33	C	S	55.285	-3.007	-2.903	-2.775	3.200	3.074	2.938	79.368	16.951	18.210
12796	279	45	33	C	S	58.641	-3.292	2.802	-2.488	3.523	-2.949	2.618	79.376	18.221	16.941
12797	279	45	33	C	S	61.359	-3.292	-2.802	-2.488	3.523	2.949	2.618	79.376	18.221	16.941
12798	279	45	33	C	S	64.715	-3.007	2.904	-2.775	3.200	-3.074	2.938	79.368	16.951	18.210
12799	279	45	33	C	S	115.285	-3.007	-2.903	-2.775	3.200	3.074	2.938	79.368	16.951	18.210
12800	279	45	33	C	S	118.641	-3.292	2.802	-2.488	3.523	-2.949	2.618	79.376	18.221	16.941
12801	279	45	33	C	W	1.359	-3.292	-2.802	-2.488	3.523	2.949	2.618	79.376	18.221	16.941

BL number	Atoms	γ-PC unit cells	WS_2 unit cells	γ-PC origin atom	WS_2 origin atom	Twist-angle (°)	γ-PC strain 1 (%)	γ-PC strain 2 (%)	γ-PC strain 3 (%)	WS_2 strain 1 (%)	WS_2 strain 2 (%)	WS_2 strain 3 (%)	γ (°)	|**a**|(Å)	|**b**|(Å)
12802	279	45	33	C	W	4.715	-3.007	2.904	-2.775	3.200	-3.074	2.938	79.368	16.951	18.210
12803	279	45	33	C	W	55.285	-3.007	-2.903	-2.775	3.200	3.074	2.938	79.368	16.951	18.210
12804	279	45	33	C	W	58.641	-3.292	2.802	-2.488	3.523	-2.949	2.618	79.376	18.221	16.941
12805	279	45	33	C	W	61.359	-3.292	-2.802	-2.488	3.523	2.949	2.618	79.376	18.221	16.941
12806	279	45	33	C	W	64.715	-3.007	2.904	-2.775	3.200	-3.074	2.938	79.368	16.951	18.210
12807	279	45	33	C	W	115.285	-3.007	-2.903	-2.775	3.200	3.074	2.938	79.368	16.951	18.210
12808	279	45	33	C	W	118.641	-3.292	2.802	-2.488	3.523	-2.949	2.618	79.376	18.221	16.941
12809	279	45	33	P	S	1.359	-3.292	-2.802	-2.488	3.523	2.949	2.618	79.376	18.221	16.941
12810	279	45	33	P	S	4.715	-3.007	2.904	-2.775	3.200	-3.074	2.938	79.368	16.951	18.210
12811	279	45	33	P	S	55.285	-3.007	-2.903	-2.775	3.200	3.074	2.938	79.368	16.951	18.210
12812	279	45	33	P	S	58.641	-3.292	2.802	-2.488	3.523	-2.949	2.618	79.376	18.221	16.941
12813	279	45	33	P	S	61.359	-3.292	-2.802	-2.488	3.523	2.949	2.618	79.376	18.221	16.941
12814	279	45	33	P	S	64.715	-3.007	2.904	-2.775	3.200	-3.074	2.938	79.368	16.951	18.210
12815	279	45	33	P	S	115.285	-3.007	-2.903	-2.775	3.200	3.074	2.938	79.368	16.951	18.210
12816	279	45	33	P	S	118.641	-3.292	2.802	-2.488	3.523	-2.949	2.618	79.376	18.221	16.941
12817	279	45	33	P	W	1.359	-3.292	-2.802	-2.488	3.523	2.949	2.618	79.376	18.221	16.941
12818	279	45	33	P	W	4.715	-3.007	2.904	-2.775	3.200	-3.074	2.938	79.368	16.951	18.210
12819	279	45	33	P	W	55.285	-3.007	-2.903	-2.775	3.200	3.074	2.938	79.368	16.951	18.210
12820	279	45	33	P	W	58.641	-3.292	2.802	-2.488	3.523	-2.949	2.618	79.376	18.221	16.941
12821	279	45	33	P	W	61.359	-3.292	-2.802	-2.488	3.523	2.949	2.618	79.376	18.221	16.941
12822	279	45	33	P	W	64.715	-3.007	2.904	-2.775	3.200	-3.074	2.938	79.368	16.951	18.210
12823	279	45	33	P	W	115.285	-3.007	-2.903	-2.775	3.200	3.074	2.938	79.368	16.951	18.210
12824	279	45	33	P	W	118.641	-3.292	2.802	-2.488	3.523	-2.949	2.618	79.376	18.221	16.941
12825	280	43	36	C	S	7.589	0.334	0.000	0.334	-0.332	0.000	-0.332	60.000	18.904	18.904
12826	280	43	36	C	S	52.411	0.334	0.000	0.334	-0.332	0.000	-0.332	60.000	18.904	18.904
12827	280	43	36	C	S	67.589	0.334	0.000	0.334	-0.332	0.000	-0.332	60.000	18.904	18.904
12828	280	43	36	C	S	112.411	0.334	0.000	0.334	-0.332	0.000	-0.332	60.000	18.904	18.904
12829	280	43	36	C	W	7.589	0.334	0.000	0.334	-0.332	0.000	-0.332	60.000	18.904	18.904
12830	280	43	36	C	W	52.411	0.334	0.000	0.334	-0.332	0.000	-0.332	60.000	18.904	18.904
12831	280	43	36	C	W	67.589	0.334	0.000	0.334	-0.332	0.000	-0.332	60.000	18.904	18.904
12832	280	43	36	C	W	112.411	0.334	0.000	0.334	-0.332	0.000	-0.332	60.000	18.904	18.904
12833	280	43	36	P	S	7.589	0.334	0.000	0.334	-0.332	0.000	-0.332	60.000	18.904	18.904
12834	280	43	36	P	S	67.589	0.334	0.000	0.334	-0.332	0.000	-0.332	60.000	18.904	18.904
12835	280	43	36	C	S	0.000	5.011	-0.705	-3.944	-4.554	0.765	4.282	77.182	18.353	17.328
12836	280	43	36	C	S	3.004	-3.388	4.725	4.355	3.635	-4.347	-4.006	76.953	17.335	18.353
12837	280	43	36	C	S	6.178	-2.145	-4.036	2.943	2.241	3.811	-2.779	77.012	17.102	18.583
12838	280	43	36	C	S	9.183	3.582	1.297	-2.716	-3.342	-1.371	2.872	77.169	18.586	17.094
12839	280	43	36	C	S	15.608	-2.145	0.563	2.943	2.241	-0.532	-2.779	78.860	17.102	18.455
12840	280	43	36	C	S	16.102	2.853	1.381	-2.064	-2.699	-1.440	2.153	78.833	18.455	17.103
12841	280	43	36	C	S	24.791	-3.388	1.112	4.355	3.635	-1.023	-4.006	78.849	17.335	18.223
12842	280	43	36	C	S	25.285	4.262	1.867	-3.309	-3.928	-1.999	3.544	78.808	18.222	17.337
12843	280	43	36	C	S	34.715	4.262	-1.867	-3.309	-3.928	1.999	3.544	78.808	18.222	17.337
12844	280	43	36	C	S	35.209	-3.388	-1.112	4.355	3.635	1.023	-4.006	78.849	17.335	18.223
12845	280	43	36	C	S	43.898	2.853	-1.381	-2.064	-2.699	1.440	2.152	78.833	18.455	17.103
12846	280	43	36	C	S	44.392	-2.145	-0.563	2.943	2.241	0.532	-2.779	78.860	17.102	18.455
12847	280	43	36	C	S	50.817	3.582	-1.297	-2.716	-3.342	1.371	2.872	77.169	18.586	17.094
12848	280	43	36	C	S	53.822	-2.145	4.036	2.943	2.241	-3.811	-2.779	77.012	17.102	18.583
12849	280	43	36	C	S	56.996	-3.388	-4.725	4.355	3.635	4.347	-4.006	76.953	17.335	18.353
12850	280	43	36	C	S	60.000	5.011	2.115	-3.944	-4.554	-2.296	4.282	76.385	18.842	16.933
12851	280	43	36	C	S	63.005	-3.388	4.725	4.355	3.635	-4.347	-4.006	76.953	17.335	18.353
12852	280	43	36	C	S	66.178	-2.145	-4.036	2.943	2.241	3.811	-2.779	77.012	17.102	18.583

BL number	Atoms	γ-PC unit cells	WS_2 unit cells	γ-PC origin atom	WS_2 origin atom	Twist-angle (°)	γ-PC strain 1 (%)	γ-PC strain 2 (%)	γ-PC strain 3 (%)	WS_2 strain 1 (%)	WS_2 strain 2 (%)	WS_2 strain 3 (%)	γ (°)	$\lvert\mathbf{a}\rvert$(Å)	$\lvert\mathbf{b}\rvert$(Å)
12853	280	43	36	C	S	69.183	3.582	1.297	-2.716	-3.342	-1.371	2.872	77.169	18.586	17.094
12854	280	43	36	C	S	75.609	-2.145	0.563	2.943	2.241	-0.532	-2.779	78.860	17.102	18.455
12855	280	43	36	C	S	76.102	2.853	1.381	-2.064	-2.699	-1.440	2.153	78.833	18.455	17.103
12856	280	43	36	C	S	84.791	-3.388	1.112	4.355	3.635	-1.023	-4.006	78.849	17.335	18.223
12857	280	43	36	C	S	85.285	4.262	1.867	-3.309	-3.928	-1.999	3.544	78.808	18.222	17.337
12858	280	43	36	C	S	94.715	4.262	-1.867	-3.309	-3.928	1.999	3.544	78.808	18.222	17.337
12859	280	43	36	C	S	95.209	-3.388	-1.112	4.355	3.635	1.023	-4.006	78.849	17.335	18.223
12860	280	43	36	C	S	103.898	2.853	-1.381	-2.064	-2.699	1.440	2.152	78.833	18.455	17.103
12861	280	43	36	C	S	104.392	-2.145	-0.563	2.943	2.241	0.532	-2.779	78.860	17.102	18.455
12862	280	43	36	C	S	110.817	3.582	-1.297	-2.716	-3.342	1.371	2.872	77.169	18.586	17.094
12863	280	43	36	C	S	113.822	-2.145	4.036	2.943	2.241	-3.811	-2.779	77.012	17.102	18.583
12864	280	43	36	C	S	116.996	-3.388	-4.725	4.355	3.635	4.347	-4.006	76.953	17.335	18.353
12865	280	43	36	C	W	0.000	5.011	-0.705	-3.944	-4.554	0.765	4.282	77.182	18.353	17.328
12866	280	43	36	C	W	3.004	-3.388	4.725	4.355	3.635	-4.347	-4.006	76.953	17.335	18.353
12867	280	43	36	C	W	6.178	-2.145	-4.036	2.943	2.241	3.811	-2.779	77.012	17.102	18.583
12868	280	43	36	C	W	9.183	3.582	1.297	-2.716	-3.342	-1.371	2.872	77.169	18.586	17.094
12869	280	43	36	C	W	15.608	-2.145	0.563	2.943	2.241	-0.532	-2.779	78.860	17.102	18.455
12870	280	43	36	C	W	16.102	2.853	1.381	-2.064	-2.699	-1.440	2.153	78.833	18.455	17.103
12871	280	43	36	C	W	24.791	-3.388	1.112	4.355	3.635	-1.023	-4.006	78.849	17.335	18.223
12872	280	43	36	C	W	25.285	4.262	1.867	-3.309	-3.928	-1.999	3.544	78.808	18.222	17.337
12873	280	43	36	C	W	34.715	4.262	-1.867	-3.309	-3.928	1.999	3.544	78.808	18.222	17.337
12874	280	43	36	C	W	35.209	-3.388	-1.112	4.355	3.635	1.023	-4.006	78.849	17.335	18.223
12875	280	43	36	C	W	43.898	2.853	-1.381	-2.064	-2.699	1.440	2.152	78.833	18.455	17.103
12876	280	43	36	C	W	44.392	-2.145	-0.563	2.943	2.241	0.532	-2.779	78.860	17.102	18.455
12877	280	43	36	C	W	50.817	3.582	-1.297	-2.716	-3.342	1.371	2.872	77.169	18.586	17.094
12878	280	43	36	C	W	53.822	-2.145	4.036	2.943	2.241	-3.811	-2.779	77.012	17.102	18.583
12879	280	43	36	C	W	56.996	-3.388	-4.725	4.355	3.635	4.347	-4.006	76.953	17.335	18.353
12880	280	43	36	C	W	60.000	5.011	2.115	-3.944	-4.554	-2.296	4.282	76.385	18.842	16.933
12881	280	43	36	C	W	63.005	-3.388	4.725	4.355	3.635	-4.347	-4.006	76.953	17.335	18.353
12882	280	43	36	C	W	66.178	-2.145	-4.036	2.943	2.241	3.811	-2.779	77.012	17.102	18.583
12883	280	43	36	C	W	69.183	3.582	1.297	-2.716	-3.342	-1.371	2.872	77.169	18.586	17.094
12884	280	43	36	C	W	75.609	-2.145	0.563	2.943	2.241	-0.532	-2.779	78.860	17.102	18.455
12885	280	43	36	C	W	76.102	2.853	1.381	-2.064	-2.699	-1.440	2.153	78.833	18.455	17.103
12886	280	43	36	C	W	84.791	-3.388	1.112	4.355	3.635	-1.023	-4.006	78.849	17.335	18.223
12887	280	43	36	C	W	85.285	4.262	1.867	-3.309	-3.928	-1.999	3.544	78.808	18.222	17.337
12888	280	43	36	C	W	94.715	4.262	-1.867	-3.309	-3.928	1.999	3.544	78.808	18.222	17.337
12889	280	43	36	C	W	95.209	-3.388	-1.112	4.355	3.635	1.023	-4.006	78.849	17.335	18.223
12890	280	43	36	C	W	103.898	2.853	-1.381	-2.064	-2.699	1.440	2.152	78.833	18.455	17.103
12891	280	43	36	C	W	104.392	-2.145	-0.563	2.943	2.241	0.532	-2.779	78.860	17.102	18.455
12892	280	43	36	C	W	110.817	3.582	-1.297	-2.716	-3.342	1.371	2.872	77.169	18.586	17.094
12893	280	43	36	C	W	113.822	-2.145	4.036	2.943	2.241	-3.811	-2.779	77.012	17.102	18.583
12894	280	43	36	C	W	116.996	-3.388	-4.725	4.355	3.635	4.347	-4.006	76.953	17.335	18.353
12895	280	43	36	P	S	0.000	5.011	-0.705	-3.944	-4.554	0.765	4.282	77.182	18.353	17.328
12896	280	43	36	P	S	3.004	-3.388	4.725	4.355	3.635	-4.347	-4.006	76.953	17.335	18.353
12897	280	43	36	P	S	6.178	-2.145	-4.036	2.943	2.241	3.811	-2.779	77.012	17.102	18.583
12898	280	43	36	P	S	9.183	3.582	1.297	-2.716	-3.342	-1.371	2.872	77.169	18.586	17.094
12899	280	43	36	P	S	24.791	-3.388	1.112	4.355	3.635	-1.023	-4.006	78.849	17.335	18.223
12900	280	43	36	P	S	25.285	4.262	1.867	-3.309	-3.928	-1.999	3.544	78.808	18.222	17.337
12901	280	43	36	P	S	34.715	4.262	-1.867	-3.309	-3.928	1.999	3.544	78.808	18.222	17.337
12902	280	43	36	P	S	35.209	-3.388	-1.112	4.355	3.635	1.023	-4.006	78.849	17.335	18.223
12903	280	43	36	P	S	44.392	-2.145	-0.563	2.943	2.241	0.532	-2.779	78.860	17.102	18.455

BL number	Atoms	γ-PC unit cells	WS_2 unit cells	γ-PC origin atom	WS_2 origin atom	Twist-angle (°)	γ-PC strain 1 (%)	γ-PC strain 2 (%)	γ-PC strain 3 (%)	WS_2 strain 1 (%)	WS_2 strain 2 (%)	WS_2 strain 3 (%)	γ (°)	|**a**|(Å)	|**b**|(Å)
12904	280	43	36	P	S	50.817	3.582	-1.297	-2.716	-3.342	1.371	2.872	77.169	18.586	17.094
12905	280	43	36	P	S	53.822	-2.145	4.036	2.943	2.241	-3.811	-2.779	77.012	17.102	18.583
12906	280	43	36	P	S	56.996	-3.388	-4.725	4.355	3.635	4.347	-4.006	76.953	17.335	18.353
12907	280	43	36	P	S	60.000	5.011	0.705	-3.944	-4.554	-0.766	4.282	77.182	18.353	17.328
12908	280	43	36	P	S	63.005	-3.388	4.725	4.355	3.635	-4.347	-4.006	76.953	17.335	18.353
12909	280	43	36	P	S	66.178	-2.145	-4.036	2.943	2.241	3.811	-2.779	77.012	17.102	18.583
12910	280	43	36	P	S	69.183	3.582	1.297	-2.716	-3.342	-1.371	2.872	77.169	18.586	17.094
12911	280	43	36	P	S	84.791	-3.388	1.112	4.355	3.635	-1.023	-4.006	78.849	17.335	18.223
12912	280	43	36	P	S	85.285	4.262	1.867	-3.309	-3.928	-1.999	3.544	78.808	18.222	17.337
12913	280	43	36	P	S	94.715	4.262	-1.867	-3.309	-3.928	1.999	3.544	78.808	18.222	17.337
12914	280	43	36	P	S	95.209	-3.388	-1.112	4.355	3.635	1.023	-4.006	78.849	17.335	18.223
12915	280	43	36	P	S	110.817	3.582	-1.297	-2.716	-3.342	1.371	2.872	77.169	18.586	17.094
12916	280	43	36	P	S	113.822	-2.145	4.036	2.943	2.241	-3.811	-2.779	77.012	17.102	18.583
12917	280	43	36	P	S	116.996	-3.388	-4.725	4.355	3.635	4.347	-4.006	76.953	17.335	18.353
12918	280	43	36	P	W	0.000	5.011	-0.705	-3.944	-4.554	0.765	4.282	77.182	18.353	17.328
12919	280	43	36	P	W	3.004	-3.388	4.725	4.355	3.635	-4.347	-4.006	76.953	17.335	18.353
12920	280	43	36	P	W	6.178	-2.145	-4.036	2.943	2.241	3.811	-2.779	77.012	17.102	18.583
12921	280	43	36	P	W	9.183	3.582	1.297	-2.716	-3.342	-1.371	2.872	77.169	18.586	17.094
12922	280	43	36	P	W	24.791	-3.388	1.112	4.355	3.635	-1.023	-4.006	78.849	17.335	18.223
12923	280	43	36	P	W	25.285	4.262	1.867	-3.309	-3.928	-1.999	3.544	78.808	18.222	17.337
12924	280	43	36	P	W	34.715	4.262	-1.867	-3.309	-3.928	1.999	3.544	78.808	18.222	17.337
12925	280	43	36	P	W	35.209	-3.388	-1.112	4.355	3.635	1.023	-4.006	78.849	17.335	18.223
12926	280	43	36	P	W	50.817	3.582	-1.297	-2.716	-3.342	1.371	2.872	77.169	18.586	17.094
12927	280	43	36	P	W	53.822	-2.145	4.036	2.943	2.241	-3.811	-2.779	77.012	17.102	18.583
12928	280	43	36	P	W	56.996	-3.388	-4.725	4.355	3.635	4.347	-4.006	76.953	17.335	18.353
12929	280	43	36	P	W	60.000	5.011	2.115	-3.944	-4.554	-2.296	4.282	76.385	18.842	16.933
12930	280	43	36	P	W	63.005	-3.388	4.725	4.355	3.635	-4.347	-4.006	76.953	17.335	18.353
12931	280	43	36	P	W	66.178	-2.145	-4.036	2.943	2.241	3.811	-2.779	77.012	17.102	18.583
12932	280	43	36	P	W	69.183	3.582	1.297	-2.716	-3.342	-1.371	2.872	77.169	18.586	17.094
12933	280	43	36	P	W	84.791	-3.388	1.112	4.355	3.635	-1.023	-4.006	78.849	17.335	18.223
12934	280	43	36	P	W	85.285	4.262	1.867	-3.309	-3.928	-1.999	3.544	78.808	18.222	17.337
12935	280	43	36	P	W	94.715	4.262	-1.867	-3.309	-3.928	1.999	3.544	78.808	18.222	17.337
12936	280	43	36	P	W	95.209	-3.388	-1.112	4.355	3.635	1.023	-4.006	78.849	17.335	18.223
12937	280	43	36	P	W	104.392	-2.145	-0.563	2.943	2.241	0.532	-2.779	78.860	17.102	18.455
12938	280	43	36	P	W	110.817	3.582	-1.297	-2.716	-3.342	1.371	2.872	77.169	18.586	17.094
12939	280	43	36	P	W	113.822	-2.145	4.036	2.943	2.241	-3.811	-2.779	77.012	17.102	18.583
12940	280	43	36	P	W	116.996	-3.388	-4.725	4.355	3.635	4.347	-4.006	76.953	17.335	18.353
12941	281	44	35	C	S	4.715	-1.702	-3.556	-0.159	1.762	3.567	0.160	83.191	19.567	15.889
12942	281	44	35	C	S	8.948	-0.599	-4.304	-1.272	0.606	4.417	1.306	78.573	15.901	19.806
12943	281	44	35	C	S	13.898	-0.414	4.222	-1.454	0.417	-4.348	1.498	78.582	19.824	15.887
12944	281	44	35	C	S	46.102	-0.414	-4.222	-1.454	0.417	4.348	1.498	78.582	19.824	15.887
12945	281	44	35	C	S	51.052	-0.599	-3.782	-1.272	0.606	3.881	1.306	83.145	15.901	19.553
12946	281	44	35	C	S	55.285	-1.702	3.556	-0.159	1.762	-3.567	0.160	83.190	19.567	15.889
12947	281	44	35	C	S	64.715	-1.702	-3.556	-0.159	1.762	3.567	0.160	83.190	19.567	15.889
12948	281	44	35	C	S	68.948	-0.599	-4.304	-1.272	0.606	4.417	1.306	78.573	15.901	19.806
12949	281	44	35	C	S	73.898	-0.414	4.222	-1.454	0.417	-4.348	1.498	78.582	19.824	15.887
12950	281	44	35	C	S	106.102	-0.414	-4.222	-1.454	0.417	4.348	1.498	78.582	19.824	15.887
12951	281	44	35	C	S	111.052	-0.599	-3.782	-1.272	0.606	3.881	1.306	83.145	15.901	19.553
12952	281	44	35	C	S	115.285	-1.702	3.556	-0.159	1.762	-3.567	0.160	83.190	19.567	15.889
12953	281	44	35	C	W	4.715	-1.702	-3.556	-0.159	1.762	3.567	0.160	83.191	19.567	15.889
12954	281	44	35	C	W	8.948	-0.599	-4.304	-1.272	0.606	4.417	1.306	78.573	15.901	19.806

BL number	Atoms	γ-PC unit cells	WS_2 unit cells	γ-PC origin atom	WS_2 origin atom	Twist-angle (°)	γ-PC strain 1 (%)	γ-PC strain 2 (%)	γ-PC strain 3 (%)	WS_2 strain 1 (%)	WS_2 strain 2 (%)	WS_2 strain 3 (%)	γ (°)	$\lvert\mathbf{a}\rvert$(Å)	$\lvert\mathbf{b}\rvert$(Å)
12955	281	44	35	C	W	13.898	-0.414	4.222	-1.454	0.417	-4.348	1.498	78.582	19.824	15.887
12956	281	44	35	C	W	46.102	-0.414	-4.222	-1.454	0.417	4.348	1.498	78.582	19.824	15.887
12957	281	44	35	C	W	51.052	-0.599	-3.782	-1.272	0.606	3.881	1.306	83.145	15.901	19.553
12958	281	44	35	C	W	55.285	-1.702	3.556	-0.159	1.762	-3.567	0.160	83.190	19.567	15.889
12959	281	44	35	C	W	64.715	-1.702	-3.556	-0.159	1.762	3.567	0.160	83.190	19.567	15.889
12960	281	44	35	C	W	68.948	-0.599	-4.304	-1.272	0.606	4.417	1.306	78.573	15.901	19.806
12961	281	44	35	C	W	73.898	-0.414	4.222	-1.454	0.417	-4.348	1.498	78.582	19.824	15.887
12962	281	44	35	C	W	106.102	-0.414	-4.222	-1.454	0.417	4.348	1.498	78.582	19.824	15.887
12963	281	44	35	C	W	111.052	-0.599	-3.782	-1.272	0.606	3.881	1.306	83.145	15.901	19.553
12964	281	44	35	C	W	115.285	-1.702	3.556	-0.159	1.762	-3.567	0.160	83.190	19.567	15.889
12965	281	44	35	P	S	4.715	-1.702	-3.556	-0.159	1.762	3.567	0.160	83.191	19.567	15.889
12966	281	44	35	P	S	8.948	-0.599	3.782	-1.272	0.606	-3.881	1.306	83.145	15.901	19.553
12967	281	44	35	P	S	46.102	-0.414	-4.222	-1.454	0.417	4.348	1.498	78.582	19.824	15.887
12968	281	44	35	P	S	51.052	-0.599	-3.782	-1.272	0.606	3.881	1.306	83.145	15.901	19.553
12969	281	44	35	P	S	55.285	-1.702	3.556	-0.159	1.762	-3.567	0.160	83.190	19.567	15.889
12970	281	44	35	P	S	64.715	-1.702	-3.556	-0.159	1.762	3.567	0.160	83.190	19.567	15.889
12971	281	44	35	P	S	68.948	-0.599	-4.304	-1.272	0.606	4.417	1.306	78.573	15.901	19.806
12972	281	44	35	P	S	73.898	-0.414	4.222	-1.454	0.417	-4.348	1.498	78.582	19.824	15.887
12973	281	44	35	P	S	106.102	-0.414	-4.222	-1.454	0.417	4.348	1.498	78.582	19.824	15.887
12974	281	44	35	P	S	111.052	-0.599	-3.782	-1.272	0.606	3.881	1.306	83.145	15.901	19.553
12975	281	44	35	P	S	115.285	-1.702	3.556	-0.159	1.762	-3.567	0.160	83.190	19.567	15.889
12976	281	44	35	P	W	4.715	-1.702	-3.556	-0.159	1.762	3.567	0.160	83.191	19.567	15.889
12977	281	44	35	P	W	8.948	-0.599	-4.304	-1.272	0.606	4.417	1.306	78.573	15.901	19.806
12978	281	44	35	P	W	13.898	-0.414	4.222	-1.454	0.417	-4.348	1.498	78.582	19.824	15.887
12979	281	44	35	P	W	46.102	-0.414	-4.222	-1.454	0.417	4.348	1.498	78.582	19.824	15.887
12980	281	44	35	P	W	51.052	-0.599	-3.782	-1.272	0.606	3.881	1.306	83.145	15.901	19.553
12981	281	44	35	P	W	55.285	-1.702	3.556	-0.159	1.762	-3.567	0.160	83.190	19.567	15.889
12982	281	44	35	P	W	64.715	-1.702	-3.556	-0.159	1.762	3.567	0.160	83.190	19.567	15.889
12983	281	44	35	P	W	68.948	-0.599	3.782	-1.272	0.606	-3.881	1.306	83.145	15.901	19.553
12984	281	44	35	P	W	73.898	-0.414	4.222	-1.454	0.417	-4.348	1.498	78.582	19.824	15.887
12985	281	44	35	P	W	106.102	-0.414	-4.222	-1.454	0.417	4.348	1.498	78.582	19.824	15.887
12986	281	44	35	P	W	111.052	-0.599	-3.782	-1.272	0.606	3.881	1.306	83.145	15.901	19.553
12987	281	44	35	P	W	115.285	-1.702	3.556	-0.159	1.762	-3.567	0.160	83.190	19.567	15.889
12988	282	42	38	C	S	8.948	1.048	0.403	3.636	-1.026	-0.376	-3.389	76.226	17.420	18.585
12989	282	42	38	C	S	9.183	3.582	0.808	1.099	-3.342	-0.791	-1.076	76.206	18.586	17.421
12990	282	42	38	C	S	50.817	3.582	-0.808	1.099	-3.342	0.791	-1.076	76.206	18.586	17.421
12991	282	42	38	C	S	51.052	1.048	-0.403	3.636	-1.026	0.376	-3.389	76.226	17.420	18.585
12992	282	42	38	C	S	68.948	1.048	0.403	3.636	-1.026	-0.376	-3.389	76.226	17.420	18.585
12993	282	42	38	C	S	69.183	3.582	0.808	1.099	-3.342	-0.791	-1.076	76.206	18.586	17.421
12994	282	42	38	C	S	110.817	3.582	-0.808	1.099	-3.342	0.791	-1.076	76.206	18.586	17.421
12995	282	42	38	C	S	111.052	1.048	-0.403	3.636	-1.026	0.376	-3.389	76.226	17.420	18.585
12996	282	42	38	C	W	8.948	1.048	0.403	3.636	-1.026	-0.376	-3.389	76.226	17.420	18.585
12997	282	42	38	C	W	9.183	3.582	0.808	1.099	-3.342	-0.791	-1.076	76.206	18.586	17.421
12998	282	42	38	C	W	50.817	3.582	-0.808	1.099	-3.342	0.791	-1.076	76.206	18.586	17.421
12999	282	42	38	C	W	51.052	1.048	-0.403	3.636	-1.026	0.376	-3.389	76.226	17.420	18.585
13000	282	42	38	C	W	68.948	1.048	0.403	3.636	-1.026	-0.376	-3.389	76.226	17.420	18.585
13001	282	42	38	C	W	69.183	3.582	0.808	1.099	-3.342	-0.791	-1.076	76.206	18.586	17.421
13002	282	42	38	C	W	110.817	3.582	-0.808	1.099	-3.342	0.791	-1.076	76.206	18.586	17.421
13003	282	42	38	C	W	111.052	1.048	-0.403	3.636	-1.026	0.376	-3.389	76.226	17.420	18.585
13004	282	42	38	P	S	50.817	3.582	-0.808	1.099	-3.342	0.791	-1.076	76.206	18.586	17.421
13005	282	42	38	P	S	51.052	1.048	-0.403	3.636	-1.026	0.376	-3.389	76.226	17.420	18.585

BL number	Atoms	γ-PC unit cells	WS_2 unit cells	γ-PC origin atom	WS_2 origin atom	Twist-angle (°)	γ-PC strain 1 (%)	γ-PC strain 2 (%)	γ-PC strain 3 (%)	WS_2 strain 1 (%)	WS_2 strain 2 (%)	WS_2 strain 3 (%)	γ (°)	|**a**|(Å)	|**b**|(Å)
13006	282	42	38	P	S	68.948	1.048	0.403	3.636	-1.026	-0.376	-3.389	76.226	17.420	18.585
13007	282	42	38	P	S	69.183	3.582	0.808	1.099	-3.342	-0.791	-1.076	76.206	18.586	17.421
13008	282	42	38	P	W	8.948	1.048	0.403	3.636	-1.026	-0.376	-3.389	76.226	17.420	18.585
13009	282	42	38	P	W	9.183	3.582	0.808	1.099	-3.342	-0.791	-1.076	76.206	18.586	17.421
13010	282	42	38	P	W	110.817	3.582	-0.808	1.099	-3.342	0.791	-1.076	76.206	18.586	17.421
13011	282	42	38	P	W	111.052	1.048	-0.403	3.636	-1.026	0.376	-3.389	76.226	17.420	18.585
13012	282	45	34	C	S	7.154	-0.955	3.505	-3.381	0.973	-3.759	3.626	89.818	17.772	17.324
13013	282	45	34	C	S	24.791	-3.388	-0.133	-0.947	3.635	0.135	0.965	87.927	17.335	17.772
13014	282	45	34	C	S	34.950	-0.955	-0.307	-3.381	0.973	0.330	3.626	87.923	17.772	17.335
13015	282	45	34	C	S	52.846	-0.955	-3.505	-3.381	0.973	3.759	3.626	89.818	17.772	17.324
13016	282	45	34	C	S	56.996	-3.388	3.586	-0.947	3.635	-3.655	0.965	89.923	17.335	17.760
13017	282	45	34	C	S	63.005	-3.388	-3.586	-0.947	3.635	3.655	0.965	89.923	17.335	17.760
13018	282	45	34	C	S	67.154	-0.955	3.505	-3.381	0.973	-3.759	3.626	89.818	17.772	17.324
13019	282	45	34	C	S	84.791	-3.388	-0.133	-0.947	3.635	0.135	0.965	87.927	17.335	17.772
13020	282	45	34	C	S	94.950	-0.955	-0.307	-3.381	0.973	0.330	3.626	87.922	17.772	17.335
13021	282	45	34	C	S	95.209	-3.388	0.133	-0.947	3.634	-0.135	0.965	87.927	17.335	17.772
13022	282	45	34	C	S	112.846	-0.955	-3.505	-3.381	0.973	3.759	3.626	89.818	17.772	17.324
13023	282	45	34	C	S	116.996	-3.388	3.586	-0.947	3.635	-3.655	0.965	89.923	17.335	17.760
13024	282	45	34	C	W	3.004	-3.388	-3.586	-0.947	3.635	3.655	0.965	89.923	17.335	17.760
13025	282	45	34	C	W	24.791	-3.388	-0.133	-0.947	3.635	0.135	0.965	87.927	17.335	17.772
13026	282	45	34	C	W	34.950	-0.955	-0.307	-3.381	0.973	0.330	3.626	87.923	17.772	17.335
13027	282	45	34	C	W	35.209	-3.388	0.133	-0.947	3.634	-0.135	0.965	87.927	17.335	17.772
13028	282	45	34	C	W	84.791	-3.388	-0.133	-0.947	3.635	0.135	0.965	87.927	17.335	17.772
13029	282	45	34	C	W	85.050	-0.955	0.308	-3.381	0.973	-0.330	3.626	87.923	17.772	17.335
13030	282	45	34	C	W	94.950	-0.955	-0.307	-3.381	0.973	0.330	3.626	87.922	17.772	17.335
13031	282	45	34	P	S	7.154	-0.955	3.505	-3.381	0.973	-3.759	3.626	89.818	17.772	17.324
13032	282	45	34	P	S	34.950	-0.955	-0.307	-3.381	0.973	0.330	3.626	87.923	17.772	17.335
13033	282	45	34	P	S	35.209	-3.388	0.133	-0.947	3.634	-0.135	0.965	87.927	17.335	17.772
13034	282	45	34	P	S	52.846	-0.955	-3.505	-3.381	0.973	3.759	3.626	89.818	17.772	17.324
13035	282	45	34	P	S	56.996	-3.388	3.586	-0.947	3.635	-3.655	0.965	89.923	17.335	17.760
13036	282	45	34	P	S	63.005	-3.388	-3.586	-0.947	3.635	3.655	0.965	89.923	17.335	17.760
13037	282	45	34	P	S	67.154	-0.955	3.505	-3.381	0.973	-3.759	3.626	89.818	17.772	17.324
13038	282	45	34	P	S	84.791	-3.388	-0.133	-0.947	3.635	0.135	0.965	87.927	17.335	17.772
13039	282	45	34	P	S	85.050	-0.955	0.308	-3.381	0.973	-0.330	3.626	87.923	17.772	17.335
13040	282	45	34	P	S	112.846	-0.955	-3.505	-3.381	0.973	3.759	3.626	89.818	17.772	17.324
13041	282	45	34	P	S	116.996	-3.388	3.586	-0.947	3.635	-3.655	0.965	89.923	17.335	17.760
13042	282	45	34	P	W	3.004	-3.388	-3.586	-0.947	3.635	3.655	0.965	89.923	17.335	17.760
13043	282	45	34	P	W	24.791	-3.388	-0.133	-0.947	3.635	0.135	0.965	87.927	17.335	17.772
13044	282	45	34	P	W	94.950	-0.955	-0.307	-3.381	0.973	0.330	3.626	87.922	17.772	17.335
13045	282	45	34	P	W	95.209	-3.388	0.133	-0.947	3.634	-0.135	0.965	87.927	17.335	17.772
13046	283	43	37	C	S	17.696	1.029	0.000	1.029	-1.008	0.000	-1.008	60.000	19.034	19.034
13047	283	43	37	C	S	27.126	1.029	0.000	1.029	-1.008	0.000	-1.008	60.000	19.034	19.035
13048	283	43	37	C	S	32.874	1.029	0.000	1.029	-1.008	0.000	-1.008	60.000	19.034	19.035
13049	283	43	37	C	S	42.304	1.029	0.000	1.029	-1.008	0.000	-1.008	60.000	19.034	19.035
13050	283	43	37	C	S	77.696	1.029	0.000	1.029	-1.008	0.000	-1.008	60.000	19.034	19.035
13051	283	43	37	C	S	87.126	1.029	0.000	1.029	-1.008	0.000	-1.008	60.000	19.034	19.035
13052	283	43	37	C	S	92.874	1.029	0.000	1.029	-1.008	0.000	-1.008	60.000	19.034	19.035
13053	283	43	37	C	S	102.304	1.029	0.000	1.029	-1.008	0.000	-1.008	60.000	19.034	19.035
13054	283	43	37	C	W	17.696	1.029	0.000	1.029	-1.008	0.000	-1.008	60.000	19.034	19.034
13055	283	43	37	C	W	27.126	1.029	0.000	1.029	-1.008	0.000	-1.008	60.000	19.034	19.035
13056	283	43	37	C	W	32.874	1.029	0.000	1.029	-1.008	0.000	-1.008	60.000	19.034	19.035

BL number	Atoms	γ-PC unit cells	WS_2 unit cells	γ-PC origin atom	WS_2 origin atom	Twist-angle (°)	γ-PC strain 1 (%)	γ-PC strain 2 (%)	γ-PC strain 3 (%)	WS_2 strain 1 (%)	WS_2 strain 2 (%)	WS_2 strain 3 (%)	γ (°)	|**a**|(Å)	|**b**|(Å)
13057	283	43	37	C	W	42.304	1.029	0.000	1.029	-1.008	0.000	-1.008	60.000	19.034	19.035
13058	283	43	37	C	W	77.696	1.029	0.000	1.029	-1.008	0.000	-1.008	60.000	19.034	19.035
13059	283	43	37	C	W	87.126	1.029	0.000	1.029	-1.008	0.000	-1.008	60.000	19.034	19.035
13060	283	43	37	C	W	92.874	1.029	0.000	1.029	-1.008	0.000	-1.008	60.000	19.034	19.035
13061	283	43	37	C	W	102.304	1.029	0.000	1.029	-1.008	0.000	-1.008	60.000	19.034	19.035
13062	283	43	37	P	S	27.126	1.029	0.000	1.029	-1.008	0.000	-1.008	60.000	19.034	19.035
13063	283	43	37	P	S	42.304	1.029	0.000	1.029	-1.008	0.000	-1.008	60.000	19.034	19.035
13064	283	43	37	P	S	77.696	1.029	0.000	1.029	-1.008	0.000	-1.008	60.000	19.034	19.035
13065	283	43	37	P	S	92.874	1.029	0.000	1.029	-1.008	0.000	-1.008	60.000	19.034	19.035
13066	283	43	37	P	W	17.696	1.029	0.000	1.029	-1.008	0.000	-1.008	60.000	19.034	19.034
13067	283	43	37	P	W	32.874	1.029	0.000	1.029	-1.008	0.000	-1.008	60.000	19.034	19.035
13068	283	43	37	P	W	87.126	1.029	0.000	1.029	-1.008	0.000	-1.008	60.000	19.034	19.035
13069	283	43	37	P	W	102.304	1.029	0.000	1.029	-1.008	0.000	-1.008	60.000	19.034	19.035
13070	283	46	33	C	S	55.285	-3.007	-5.072	-3.802	3.200	5.490	4.115	84.100	16.951	18.198
13071	283	46	33	C	S	64.715	-3.007	5.072	-3.802	3.200	-5.490	4.115	84.100	16.951	18.198
13072	283	46	33	C	S	115.285	-3.007	-5.072	-3.802	3.200	5.490	4.115	84.100	16.951	18.198
13073	283	46	33	C	W	55.285	-3.007	-5.072	-3.802	3.200	5.490	4.115	84.100	16.951	18.198
13074	283	46	33	C	W	64.715	-3.007	5.072	-3.802	3.200	-5.490	4.115	84.100	16.951	18.198
13075	283	46	33	C	W	115.285	-3.007	-5.072	-3.802	3.200	5.490	4.115	84.100	16.951	18.198
13076	283	46	33	P	S	55.285	-3.007	-5.072	-3.802	3.200	5.490	4.115	84.100	16.951	18.198
13077	283	46	33	P	S	64.715	-3.007	5.072	-3.802	3.200	-5.490	4.115	84.100	16.951	18.198
13078	283	46	33	P	S	115.285	-3.007	-5.072	-3.802	3.200	5.490	4.115	84.100	16.951	18.198
13079	283	46	33	P	W	55.285	-3.007	-5.072	-3.802	3.200	5.490	4.115	84.100	16.951	18.198
13080	283	46	33	P	W	64.715	-3.007	5.072	-3.802	3.200	-5.490	4.115	84.100	16.951	18.198
13081	283	46	33	P	W	115.285	-3.007	-5.072	-3.802	3.200	5.490	4.115	84.100	16.951	18.198
13082	284	41	40	C	S	9.183	3.582	0.296	5.101	-3.342	-0.268	-4.628	69.317	18.586	18.352
13083	284	41	40	C	S	9.430	5.011	0.739	3.669	-4.554	-0.689	-3.418	69.294	18.353	18.588
13084	284	41	40	C	S	50.570	5.011	-0.739	3.669	-4.554	0.689	-3.418	69.294	18.353	18.588
13085	284	41	40	C	S	50.817	3.582	-0.296	5.101	-3.342	0.268	-4.628	69.317	18.586	18.352
13086	284	41	40	C	S	69.183	3.582	0.296	5.101	-3.342	-0.268	-4.628	69.317	18.586	18.352
13087	284	41	40	C	S	69.430	5.011	0.739	3.669	-4.554	-0.689	-3.418	69.294	18.353	18.588
13088	284	41	40	C	S	110.570	5.011	-0.739	3.669	-4.554	0.689	-3.418	69.294	18.353	18.588
13089	284	41	40	C	S	110.817	3.582	-0.296	5.101	-3.342	0.268	-4.628	69.317	18.586	18.352
13090	284	41	40	C	W	9.183	3.582	0.296	5.101	-3.342	-0.268	-4.628	69.317	18.586	18.352
13091	284	41	40	C	W	9.430	5.011	0.739	3.669	-4.554	-0.689	-3.418	69.294	18.353	18.588
13092	284	41	40	C	W	50.570	5.011	-0.739	3.669	-4.554	0.689	-3.418	69.294	18.353	18.588
13093	284	41	40	C	W	50.817	3.582	-0.296	5.101	-3.342	0.268	-4.628	69.317	18.586	18.352
13094	284	41	40	C	W	69.183	3.582	0.296	5.101	-3.342	-0.268	-4.628	69.317	18.586	18.352
13095	284	41	40	C	W	69.430	5.011	0.739	3.669	-4.554	-0.689	-3.418	69.294	18.353	18.588
13096	284	41	40	C	W	110.570	5.011	-0.739	3.669	-4.554	0.689	-3.418	69.294	18.353	18.588
13097	284	41	40	C	W	110.817	3.582	-0.296	5.101	-3.342	0.268	-4.628	69.317	18.586	18.352
13098	284	41	40	P	S	9.183	3.582	0.296	5.101	-3.342	-0.268	-4.628	69.317	18.586	18.352
13099	284	41	40	P	S	9.430	5.011	0.739	3.669	-4.554	-0.689	-3.418	69.294	18.353	18.588
13100	284	41	40	P	S	50.570	5.011	-0.739	3.669	-4.554	0.689	-3.418	69.294	18.353	18.588
13101	284	41	40	P	S	50.817	3.582	-0.296	5.101	-3.342	0.268	-4.628	69.317	18.586	18.352
13102	284	41	40	P	S	69.183	3.582	0.296	5.101	-3.342	-0.268	-4.628	69.317	18.586	18.352
13103	284	41	40	P	S	69.430	5.011	0.739	3.669	-4.554	-0.689	-3.418	69.294	18.353	18.588
13104	284	41	40	P	S	110.570	5.011	-0.739	3.669	-4.554	0.689	-3.418	69.294	18.353	18.588
13105	284	41	40	P	S	110.817	3.582	-0.296	5.101	-3.342	0.268	-4.628	69.317	18.586	18.352
13106	284	41	40	P	W	9.183	3.582	0.296	5.101	-3.342	-0.268	-4.628	69.317	18.586	18.352
13107	284	41	40	P	W	9.430	5.011	0.739	3.669	-4.554	-0.689	-3.418	69.294	18.353	18.588

BL number	Atoms	γ-PC unit cells	WS_2 unit cells	γ-PC origin atom	WS_2 origin atom	Twist-angle (°)	γ-PC strain 1 (%)	γ-PC strain 2 (%)	γ-PC strain 3 (%)	WS_2 strain 1 (%)	WS_2 strain 2 (%)	WS_2 strain 3 (%)	γ (°)	$\lvert\mathbf{a}\rvert$(Å)	$\lvert\mathbf{b}\rvert$(Å)
13108	284	41	40	P	W	50.570	5.011	-0.739	3.669	-4.554	0.689	-3.418	69.294	18.353	18.588
13109	284	41	40	P	W	50.817	3.582	-0.296	5.101	-3.342	0.268	-4.628	69.317	18.586	18.352
13110	284	41	40	P	W	69.183	3.582	0.296	5.101	-3.342	-0.268	-4.628	69.317	18.586	18.352
13111	284	41	40	P	W	69.430	5.011	0.739	3.669	-4.554	-0.689	-3.418	69.294	18.353	18.588
13112	284	41	40	P	W	110.570	5.011	-0.739	3.669	-4.554	0.689	-3.418	69.294	18.353	18.588
13113	284	41	40	P	W	110.817	3.582	-0.296	5.101	-3.342	0.268	-4.628	69.317	18.586	18.352
13114	284	44	36	C	S	30.000	-2.359	0.000	1.972	2.476	0.000	-1.897	90.000	9.718	32.228
13115	284	44	36	C	S	90.000	-2.359	0.000	1.972	2.476	0.000	-1.897	90.000	9.718	32.228
13116	284	44	36	C	W	30.000	-2.359	0.000	1.972	2.476	0.000	-1.897	90.000	9.718	32.228
13117	284	44	36	C	W	90.000	-2.359	0.000	1.972	2.476	0.000	-1.897	90.000	9.718	32.228
13118	284	44	36	C	S	7.154	-0.955	0.126	0.484	0.973	-0.125	-0.479	68.731	17.772	18.904
13119	284	44	36	C	S	10.158	2.281	3.834	-2.641	-2.182	-4.048	2.789	78.426	16.362	19.542
13120	284	44	36	C	S	16.102	2.853	-2.699	-3.153	-2.699	2.881	3.365	68.685	18.455	18.225
13121	284	44	36	C	S	16.537	-3.292	-2.088	3.009	3.523	1.969	-2.839	68.702	18.221	18.459
13122	284	44	36	C	S	43.463	-3.292	2.088	3.009	3.523	-1.969	-2.839	68.702	18.221	18.459
13123	284	44	36	C	S	43.898	2.853	2.699	-3.153	-2.699	-2.881	3.365	68.685	18.455	18.225
13124	284	44	36	C	S	49.842	2.281	-3.834	-2.641	-2.182	4.048	2.789	78.426	16.362	19.542
13125	284	44	36	C	S	52.411	0.334	-0.878	-0.809	-0.332	0.892	0.823	68.727	18.904	17.772
13126	284	44	36	C	S	67.154	-0.955	0.126	0.484	0.973	-0.125	-0.479	68.731	17.772	18.904
13127	284	44	36	C	S	70.158	2.281	3.834	-2.641	-2.182	-4.048	2.789	78.426	16.362	19.542
13128	284	44	36	C	S	76.102	2.853	-2.699	-3.153	-2.699	2.881	3.365	68.685	18.455	18.225
13129	284	44	36	C	S	76.537	-3.292	-2.088	3.009	3.523	1.969	-2.839	68.702	18.221	18.459
13130	284	44	36	C	S	103.463	-3.292	2.088	3.009	3.523	-1.969	-2.839	68.702	18.221	18.459
13131	284	44	36	C	S	103.898	2.853	2.699	-3.153	-2.699	-2.881	3.365	68.685	18.455	18.225
13132	284	44	36	C	S	109.842	2.281	-3.834	-2.641	-2.182	4.048	2.789	78.425	16.362	19.542
13133	284	44	36	C	S	112.411	0.334	-0.878	-0.809	-0.332	0.892	0.823	68.727	18.904	17.772
13134	284	44	36	C	W	7.154	-0.955	0.126	0.484	0.973	-0.125	-0.479	68.731	17.772	18.904
13135	284	44	36	C	W	10.158	2.281	3.834	-2.641	-2.182	-4.048	2.789	78.426	16.362	19.542
13136	284	44	36	C	W	16.102	2.853	-2.699	-3.153	-2.699	2.881	3.365	68.685	18.455	18.225
13137	284	44	36	C	W	16.537	-3.292	-2.088	3.009	3.523	1.969	-2.839	68.702	18.221	18.459
13138	284	44	36	C	W	43.463	-3.292	2.088	3.009	3.523	-1.969	-2.839	68.702	18.221	18.459
13139	284	44	36	C	W	43.898	2.853	2.699	-3.153	-2.699	-2.881	3.365	68.685	18.455	18.225
13140	284	44	36	C	W	49.842	2.281	-3.834	-2.641	-2.182	4.048	2.789	78.426	16.362	19.542
13141	284	44	36	C	W	52.411	0.334	-0.878	-0.809	-0.332	0.892	0.823	68.727	18.904	17.772
13142	284	44	36	C	W	67.154	-0.955	0.126	0.484	0.973	-0.125	-0.479	68.731	17.772	18.904
13143	284	44	36	C	W	70.158	2.281	3.834	-2.641	-2.182	-4.048	2.789	78.426	16.362	19.542
13144	284	44	36	C	W	76.102	2.853	-2.699	-3.153	-2.699	2.881	3.365	68.685	18.455	18.225
13145	284	44	36	C	W	76.537	-3.292	-2.088	3.009	3.523	1.969	-2.839	68.702	18.221	18.459
13146	284	44	36	C	W	103.463	-3.292	2.088	3.009	3.523	-1.969	-2.839	68.702	18.221	18.459
13147	284	44	36	C	W	103.898	2.853	2.699	-3.153	-2.699	-2.881	3.365	68.685	18.455	18.225
13148	284	44	36	C	W	109.842	2.281	-3.834	-2.641	-2.182	4.048	2.789	78.425	16.362	19.542
13149	284	44	36	C	W	112.411	0.334	-0.878	-0.809	-0.332	0.892	0.823	68.727	18.904	17.772
13150	284	44	36	P	S	7.154	-0.955	0.126	0.484	0.973	-0.125	-0.479	68.731	17.772	18.904
13151	284	44	36	P	S	10.158	2.281	3.834	-2.641	-2.182	-4.048	2.789	78.426	16.362	19.542
13152	284	44	36	P	S	49.842	2.281	-3.834	-2.641	-2.182	4.048	2.789	78.426	16.362	19.542
13153	284	44	36	P	S	52.411	0.334	-0.878	-0.809	-0.332	0.892	0.823	68.727	18.904	17.772
13154	284	44	36	P	S	67.154	-0.955	0.126	0.484	0.973	-0.125	-0.479	68.731	17.772	18.904
13155	284	44	36	P	S	70.158	2.281	3.834	-2.641	-2.182	-4.048	2.789	78.426	16.362	19.542
13156	284	44	36	P	S	109.842	2.281	-3.834	-2.641	-2.182	4.048	2.789	78.425	16.362	19.542
13157	284	44	36	P	S	112.411	0.334	-0.878	-0.809	-0.332	0.892	0.823	68.727	18.904	17.772
13158	284	44	36	P	W	7.154	-0.955	0.126	0.484	0.973	-0.125	-0.479	68.731	17.772	18.904

BL number	Atoms	γ-PC unit cells	WS_2 unit cells	γ-PC origin atom	WS_2 origin atom	Twist-angle (°)	γ-PC strain 1 (%)	γ-PC strain 2 (%)	γ-PC strain 3 (%)	WS_2 strain 1 (%)	WS_2 strain 2 (%)	WS_2 strain 3 (%)	γ (°)	|**a**|(Å)	|**b**|(Å)
13159	284	44	36	P	W	10.158	2.281	3.834	-2.641	-2.182	-4.048	2.789	78.426	16.362	19.542
13160	284	44	36	P	W	49.842	2.281	-3.834	-2.641	-2.182	4.048	2.789	78.426	16.362	19.542
13161	284	44	36	P	W	52.411	0.334	-0.878	-0.809	-0.332	0.892	0.823	68.727	18.904	17.772
13162	284	44	36	P	W	67.154	-0.955	0.126	0.484	0.973	-0.125	-0.479	68.731	17.772	18.904
13163	284	44	36	P	W	70.158	2.281	3.834	-2.641	-2.182	-4.048	2.789	78.426	16.362	19.542
13164	284	44	36	P	W	109.842	2.281	-3.834	-2.641	-2.182	4.048	2.789	78.425	16.362	19.542
13165	284	44	36	P	W	112.411	0.334	-0.878	-0.809	-0.332	0.892	0.823	68.727	18.904	17.772
13166	285	42	39	C	S	8.948	1.048	-0.134	5.047	-1.026	0.122	-4.584	85.537	17.420	18.352
13167	285	42	39	C	S	9.430	5.011	0.722	1.082	-4.554	-0.706	-1.059	85.509	18.353	17.420
13168	285	42	39	C	S	50.570	5.011	-0.722	1.082	-4.554	0.706	-1.059	85.509	18.353	17.420
13169	285	42	39	C	S	51.052	1.048	0.135	5.047	-1.026	-0.122	-4.584	85.537	17.420	18.352
13170	285	42	39	C	S	68.948	1.048	-0.134	5.047	-1.026	0.122	-4.584	85.537	17.420	18.352
13171	285	42	39	C	S	69.430	5.011	0.722	1.082	-4.554	-0.706	-1.059	85.509	18.353	17.420
13172	285	42	39	C	S	110.570	5.011	-0.722	1.082	-4.554	0.706	-1.059	85.509	18.353	17.420
13173	285	42	39	C	S	111.052	1.048	0.135	5.047	-1.026	-0.122	-4.584	85.537	17.420	18.352
13174	285	42	39	C	W	8.948	1.048	-0.134	5.047	-1.026	0.122	-4.584	85.537	17.420	18.352
13175	285	42	39	C	W	9.430	5.011	0.722	1.082	-4.554	-0.706	-1.059	85.509	18.353	17.420
13176	285	42	39	C	W	50.570	5.011	-0.722	1.082	-4.554	0.706	-1.059	85.509	18.353	17.420
13177	285	42	39	C	W	51.052	1.048	0.135	5.047	-1.026	-0.122	-4.584	85.537	17.420	18.352
13178	285	42	39	C	W	68.948	1.048	-0.134	5.047	-1.026	0.122	-4.584	85.537	17.420	18.352
13179	285	42	39	C	W	69.430	5.011	0.722	1.082	-4.554	-0.706	-1.059	85.509	18.353	17.420
13180	285	42	39	C	W	110.570	5.011	-0.722	1.082	-4.554	0.706	-1.059	85.509	18.353	17.420
13181	285	42	39	C	W	111.052	1.048	0.135	5.047	-1.026	-0.122	-4.584	85.537	17.420	18.352
13182	285	42	39	P	S	8.948	1.048	-0.134	5.047	-1.026	0.122	-4.584	85.537	17.420	18.352
13183	285	42	39	P	S	9.430	5.011	0.722	1.082	-4.554	-0.706	-1.059	85.509	18.353	17.420
13184	285	42	39	P	S	50.570	5.011	-0.722	1.082	-4.554	0.706	-1.059	85.509	18.353	17.420
13185	285	42	39	P	S	51.052	1.048	0.135	5.047	-1.026	-0.122	-4.584	85.537	17.420	18.352
13186	285	42	39	P	S	68.948	1.048	-0.134	5.047	-1.026	0.122	-4.584	85.537	17.420	18.352
13187	285	42	39	P	S	69.430	5.011	0.722	1.082	-4.554	-0.706	-1.059	85.509	18.353	17.420
13188	285	42	39	P	S	110.570	5.011	-0.722	1.082	-4.554	0.706	-1.059	85.509	18.353	17.420
13189	285	42	39	P	S	111.052	1.048	0.135	5.047	-1.026	-0.122	-4.584	85.537	17.420	18.352
13190	285	42	39	P	W	8.948	1.048	-0.134	5.047	-1.026	0.122	-4.584	85.537	17.420	18.352
13191	285	42	39	P	W	9.430	5.011	0.722	1.082	-4.554	-0.706	-1.059	85.509	18.353	17.420
13192	285	42	39	P	W	50.570	5.011	-0.722	1.082	-4.554	0.706	-1.059	85.509	18.353	17.420
13193	285	42	39	P	W	51.052	1.048	0.135	5.047	-1.026	-0.122	-4.584	85.537	17.420	18.352
13194	285	42	39	P	W	68.948	1.048	-0.134	5.047	-1.026	0.122	-4.584	85.537	17.420	18.352
13195	285	42	39	P	W	69.430	5.011	0.722	1.082	-4.554	-0.706	-1.059	85.509	18.353	17.420
13196	285	42	39	P	W	110.570	5.011	-0.722	1.082	-4.554	0.706	-1.059	85.509	18.353	17.420
13197	285	42	39	P	W	111.052	1.048	0.135	5.047	-1.026	-0.122	-4.584	85.537	17.420	18.352
13198	285	45	35	C	S	16.337	0.353	-0.955	-3.256	-0.351	1.022	3.483	77.791	17.539	18.221
13199	285	45	35	C	S	25.285	-4.781	3.176	2.052	5.287	-3.051	-1.971	76.796	16.641	19.296
13200	285	45	35	C	S	34.715	-4.781	-3.176	2.052	5.287	3.051	-1.971	76.796	16.641	19.296
13201	285	45	35	C	S	43.463	-3.292	0.640	0.392	3.523	-0.635	-0.389	77.785	18.221	17.539
13202	285	45	35	C	S	76.337	0.353	-0.955	-3.256	-0.351	1.022	3.483	77.791	17.539	18.221
13203	285	45	35	C	S	85.285	-4.781	3.176	2.052	5.287	-3.051	-1.971	76.795	16.641	19.296
13204	285	45	35	C	S	94.715	-4.781	-3.176	2.052	5.287	3.051	-1.971	76.796	16.641	19.296
13205	285	45	35	C	S	103.463	-3.292	0.640	0.392	3.523	-0.635	-0.389	77.785	18.221	17.539
13206	285	45	35	C	W	16.337	0.353	-0.955	-3.256	-0.351	1.022	3.483	77.791	17.539	18.221
13207	285	45	35	C	W	25.285	-4.781	3.176	2.052	5.287	-3.051	-1.971	76.796	16.641	19.296
13208	285	45	35	C	W	34.715	-4.781	-3.176	2.052	5.287	3.051	-1.971	76.796	16.641	19.296
13209	285	45	35	C	W	43.463	-3.292	0.640	0.392	3.523	-0.635	-0.389	77.785	18.221	17.539

BL number	Atoms	γ-PC unit cells	WS_2 unit cells	γ-PC origin atom	WS_2 origin atom	Twist-angle (°)	γ-PC strain 1 (%)	γ-PC strain 2 (%)	γ-PC strain 3 (%)	WS_2 strain 1 (%)	WS_2 strain 2 (%)	WS_2 strain 3 (%)	γ (°)	|**a**|(Å)	|**b**|(Å)
13210	285	45	35	C	W	76.337	0.353	-0.955	-3.256	-0.351	1.022	3.483	77.791	17.539	18.221
13211	285	45	35	C	W	85.285	-4.781	3.176	2.052	5.287	-3.051	-1.971	76.795	16.641	19.296
13212	285	45	35	C	W	94.715	-4.781	-3.176	2.052	5.287	3.051	-1.971	76.796	16.641	19.296
13213	285	45	35	C	W	103.463	-3.292	0.640	0.392	3.523	-0.635	-0.389	77.785	18.221	17.539
13214	285	45	35	P	S	16.337	0.353	-0.955	-3.256	-0.351	1.022	3.483	77.791	17.539	18.221
13215	285	45	35	P	S	25.285	-4.781	3.176	2.052	5.287	-3.051	-1.971	76.796	16.641	19.296
13216	285	45	35	P	S	34.715	-4.781	-3.176	2.052	5.287	3.051	-1.971	76.796	16.641	19.296
13217	285	45	35	P	S	43.463	-3.292	0.640	0.392	3.523	-0.635	-0.389	77.785	18.221	17.539
13218	285	45	35	P	S	76.337	0.353	-0.955	-3.256	-0.351	1.022	3.483	77.791	17.539	18.221
13219	285	45	35	P	S	85.285	-4.781	3.176	2.052	5.287	-3.051	-1.971	76.795	16.641	19.296
13220	285	45	35	P	S	94.715	-4.781	-3.176	2.052	5.287	3.051	-1.971	76.796	16.641	19.296
13221	285	45	35	P	S	103.463	-3.292	0.640	0.392	3.523	-0.635	-0.389	77.785	18.221	17.539
13222	285	45	35	P	W	16.337	0.353	-0.955	-3.256	-0.351	1.022	3.483	77.791	17.539	18.221
13223	285	45	35	P	W	25.285	-4.781	3.176	2.052	5.287	-3.051	-1.971	76.796	16.641	19.296
13224	285	45	35	P	W	34.715	-4.781	-3.176	2.052	5.287	3.051	-1.971	76.796	16.641	19.296
13225	285	45	35	P	W	43.463	-3.292	0.640	0.392	3.523	-0.635	-0.389	77.785	18.221	17.539
13226	285	45	35	P	W	76.337	0.353	-0.955	-3.256	-0.351	1.022	3.483	77.791	17.539	18.221
13227	285	45	35	P	W	85.285	-4.781	3.176	2.052	5.287	-3.051	-1.971	76.795	16.641	19.296
13228	285	45	35	P	W	94.715	-4.781	-3.176	2.052	5.287	3.051	-1.971	76.796	16.641	19.296
13229	285	45	35	P	W	103.463	-3.292	0.640	0.392	3.523	-0.635	-0.389	77.785	18.221	17.539
13230	286	43	38	C	S	7.154	-0.955	-3.411	4.528	0.973	3.127	-4.152	77.403	17.772	18.350
13231	286	43	38	C	S	9.430	5.011	0.705	-1.386	-4.554	-0.725	1.425	77.504	18.353	17.767
13232	286	43	38	C	S	16.337	0.353	-2.760	3.111	-0.351	2.598	-2.929	77.441	17.539	18.582
13233	286	43	38	C	S	18.613	3.582	1.297	-0.089	-3.342	-1.299	0.089	77.495	18.586	17.533
13234	286	43	38	C	S	41.387	3.582	-1.297	-0.089	-3.342	1.299	0.089	77.495	18.586	17.533
13235	286	43	38	C	S	43.663	0.353	2.760	3.111	-0.351	-2.598	-2.929	77.441	17.539	18.582
13236	286	43	38	C	S	50.570	5.011	-0.705	-1.386	-4.554	0.725	1.425	77.504	18.353	17.767
13237	286	43	38	C	S	52.846	-0.955	3.411	4.528	0.973	-3.127	-4.152	77.403	17.772	18.350
13238	286	43	38	C	S	67.154	-0.955	-3.411	4.528	0.973	3.127	-4.152	77.403	17.772	18.350
13239	286	43	38	C	S	69.430	5.011	0.705	-1.386	-4.554	-0.725	1.425	77.504	18.353	17.767
13240	286	43	38	C	S	76.337	0.353	-2.760	3.111	-0.351	2.598	-2.929	77.441	17.539	18.582
13241	286	43	38	C	S	78.613	3.582	1.297	-0.089	-3.342	-1.299	0.089	77.495	18.586	17.533
13242	286	43	38	C	S	101.387	3.582	-1.297	-0.089	-3.342	1.299	0.089	77.495	18.586	17.533
13243	286	43	38	C	S	103.663	0.353	2.760	3.111	-0.351	-2.598	-2.929	77.441	17.539	18.582
13244	286	43	38	C	S	110.570	5.011	-0.705	-1.386	-4.554	0.725	1.425	77.504	18.353	17.767
13245	286	43	38	C	S	112.846	-0.955	3.411	4.528	0.973	-3.127	-4.152	77.403	17.772	18.350
13246	286	43	38	C	W	7.154	-0.955	-3.411	4.528	0.973	3.127	-4.152	77.403	17.772	18.350
13247	286	43	38	C	W	9.430	5.011	0.705	-1.386	-4.554	-0.725	1.425	77.504	18.353	17.767
13248	286	43	38	C	W	16.337	0.353	-2.760	3.111	-0.351	2.598	-2.929	77.441	17.539	18.582
13249	286	43	38	C	W	18.613	3.582	1.297	-0.089	-3.342	-1.299	0.089	77.495	18.586	17.533
13250	286	43	38	C	W	41.387	3.582	-1.297	-0.089	-3.342	1.299	0.089	77.495	18.586	17.533
13251	286	43	38	C	W	43.663	0.353	2.760	3.111	-0.351	-2.598	-2.929	77.441	17.539	18.582
13252	286	43	38	C	W	50.570	5.011	-0.705	-1.386	-4.554	0.725	1.425	77.504	18.353	17.767
13253	286	43	38	C	W	52.846	-0.955	3.411	4.528	0.973	-3.127	-4.152	77.403	17.772	18.350
13254	286	43	38	C	W	67.154	-0.955	-3.411	4.528	0.973	3.127	-4.152	77.403	17.772	18.350
13255	286	43	38	C	W	69.430	5.011	0.705	-1.386	-4.554	-0.725	1.425	77.504	18.353	17.767
13256	286	43	38	C	W	76.337	0.353	-2.760	3.111	-0.351	2.598	-2.929	77.441	17.539	18.582
13257	286	43	38	C	W	78.613	3.582	1.297	-0.089	-3.342	-1.299	0.089	77.495	18.586	17.533
13258	286	43	38	C	W	101.387	3.582	-1.297	-0.089	-3.342	1.299	0.089	77.495	18.586	17.533
13259	286	43	38	C	W	103.663	0.353	2.760	3.111	-0.351	-2.598	-2.929	77.441	17.539	18.582
13260	286	43	38	C	W	110.570	5.011	-0.705	-1.386	-4.554	0.725	1.425	77.504	18.353	17.767

BL number	Atoms	γ-PC unit cells	WS_2 unit cells	γ-PC origin atom	WS_2 origin atom	Twist-angle (°)	γ-PC strain 1 (%)	γ-PC strain 2 (%)	γ-PC strain 3 (%)	WS_2 strain 1 (%)	WS_2 strain 2 (%)	WS_2 strain 3 (%)	γ (°)	$\lvert \mathbf{a} \rvert$(Å)	$\lvert \mathbf{b} \rvert$(Å)
13261	286	43	38	C	W	112.846	-0.955	3.411	4.528	0.973	-3.127	-4.152	77.403	17.772	18.350
13262	286	43	38	P	S	7.154	-0.955	-3.411	4.528	0.973	3.127	-4.152	77.403	17.772	18.350
13263	286	43	38	P	S	9.430	5.011	0.705	-1.386	-4.554	-0.725	1.425	77.504	18.353	17.767
13264	286	43	38	P	S	16.337	0.353	-2.760	3.111	-0.351	2.598	-2.929	77.441	17.539	18.582
13265	286	43	38	P	S	18.613	3.582	1.297	-0.089	-3.342	-1.299	0.089	77.495	18.586	17.533
13266	286	43	38	P	S	41.387	3.582	-1.297	-0.089	-3.342	1.299	0.089	77.495	18.586	17.533
13267	286	43	38	P	S	43.663	0.353	2.760	3.111	-0.351	-2.598	-2.929	77.441	17.539	18.582
13268	286	43	38	P	S	50.570	5.011	-0.705	-1.386	-4.554	0.725	1.425	77.504	18.353	17.767
13269	286	43	38	P	S	52.846	-0.955	3.411	4.528	0.973	-3.127	-4.152	77.403	17.772	18.350
13270	286	43	38	P	S	67.154	-0.955	-3.411	4.528	0.973	3.127	-4.152	77.403	17.772	18.350
13271	286	43	38	P	S	69.430	5.011	0.705	-1.386	-4.554	-0.725	1.425	77.504	18.353	17.767
13272	286	43	38	P	S	76.337	0.353	-2.760	3.111	-0.351	2.598	-2.929	77.441	17.539	18.582
13273	286	43	38	P	S	78.613	3.582	1.297	-0.089	-3.342	-1.299	0.089	77.495	18.586	17.533
13274	286	43	38	P	S	101.387	3.582	-1.297	-0.089	-3.342	1.299	0.089	77.495	18.586	17.533
13275	286	43	38	P	S	103.663	0.353	2.760	3.111	-0.351	-2.598	-2.929	77.441	17.539	18.582
13276	286	43	38	P	S	110.570	5.011	-0.705	-1.386	-4.554	0.725	1.425	77.504	18.353	17.767
13277	286	43	38	P	S	112.846	-0.955	3.411	4.528	0.973	-3.127	-4.152	77.403	17.772	18.350
13278	286	43	38	P	W	7.154	-0.955	-3.411	4.528	0.973	3.127	-4.152	77.403	17.772	18.350
13279	286	43	38	P	W	9.430	5.011	0.705	-1.386	-4.554	-0.725	1.425	77.504	18.353	17.767
13280	286	43	38	P	W	16.337	0.353	-2.760	3.111	-0.351	2.598	-2.929	77.441	17.539	18.582
13281	286	43	38	P	W	18.613	3.582	1.297	-0.089	-3.342	-1.299	0.089	77.495	18.586	17.533
13282	286	43	38	P	W	41.387	3.582	-1.297	-0.089	-3.342	1.299	0.089	77.495	18.586	17.533
13283	286	43	38	P	W	43.663	0.353	2.760	3.111	-0.351	-2.598	-2.929	77.441	17.539	18.582
13284	286	43	38	P	W	50.570	5.011	-0.705	-1.386	-4.554	0.725	1.425	77.504	18.353	17.767
13285	286	43	38	P	W	52.846	-0.955	3.411	4.528	0.973	-3.127	-4.152	77.403	17.772	18.350
13286	286	43	38	P	W	67.154	-0.955	-3.411	4.528	0.973	3.127	-4.152	77.403	17.772	18.350
13287	286	43	38	P	W	69.430	5.011	0.705	-1.386	-4.554	-0.725	1.425	77.504	18.353	17.767
13288	286	43	38	P	W	76.337	0.353	-2.760	3.111	-0.351	2.598	-2.929	77.441	17.539	18.582
13289	286	43	38	P	W	78.613	3.582	1.297	-0.089	-3.342	-1.299	0.089	77.495	18.586	17.533
13290	286	43	38	P	W	101.387	3.582	-1.297	-0.089	-3.342	1.299	0.089	77.495	18.586	17.533
13291	286	43	38	P	W	103.663	0.353	2.760	3.111	-0.351	-2.598	-2.929	77.441	17.539	18.582
13292	286	43	38	P	W	110.570	5.011	-0.705	-1.386	-4.554	0.725	1.425	77.504	18.353	17.767
13293	286	43	38	P	W	112.846	-0.955	3.411	4.528	0.973	-3.127	-4.152	77.403	17.772	18.350
13294	286	46	34	C	S	1.359	-3.292	-4.111	-2.112	3.523	4.293	2.206	89.565	18.221	17.087
13295	286	46	34	C	S	6.178	-2.145	4.079	-3.260	2.241	-4.364	3.487	89.506	17.102	18.204
13296	286	46	34	C	S	15.608	-2.145	-0.833	-3.260	2.241	0.892	3.487	87.585	17.102	18.220
13297	286	46	34	C	S	43.463	-3.292	-0.744	-2.112	3.523	0.777	2.206	87.595	18.221	17.101
13298	286	46	34	C	S	53.822	-2.145	-4.079	-3.260	2.241	4.364	3.487	89.506	17.102	18.204
13299	286	46	34	C	S	58.641	-3.292	4.111	-2.112	3.523	-4.293	2.206	89.565	18.221	17.087
13300	286	46	34	C	S	61.359	-3.292	-4.111	-2.112	3.523	4.293	2.206	89.565	18.221	17.087
13301	286	46	34	C	S	66.178	-2.145	4.079	-3.260	2.241	-4.364	3.487	89.506	17.102	18.204
13302	286	46	34	C	S	75.609	-2.145	-0.833	-3.260	2.241	0.892	3.487	87.585	17.102	18.220
13303	286	46	34	C	S	103.463	-3.292	-0.744	-2.112	3.523	0.777	2.206	87.595	18.221	17.101
13304	286	46	34	C	S	113.822	-2.145	-4.079	-3.260	2.241	4.364	3.487	89.506	17.102	18.204
13305	286	46	34	C	S	118.641	-3.292	4.111	-2.112	3.523	-4.293	2.206	89.565	18.221	17.087
13306	286	46	34	C	W	1.359	-3.292	-4.111	-2.112	3.523	4.293	2.206	89.565	18.221	17.087
13307	286	46	34	C	W	6.178	-2.145	4.079	-3.260	2.241	-4.364	3.487	89.506	17.102	18.204
13308	286	46	34	C	W	15.608	-2.145	-0.833	-3.260	2.241	0.892	3.487	87.585	17.102	18.220
13309	286	46	34	C	W	43.463	-3.292	-0.744	-2.112	3.523	0.777	2.206	87.595	18.221	17.101
13310	286	46	34	C	W	53.822	-2.145	-4.079	-3.260	2.241	4.364	3.487	89.506	17.102	18.204
13311	286	46	34	C	W	58.641	-3.292	4.111	-2.112	3.523	-4.293	2.206	89.565	18.221	17.087

BL number	Atoms	γ-PC unit cells	WS_2 unit cells	γ-PC origin atom	WS_2 origin atom	Twist-angle (°)	γ-PC strain 1 (%)	γ-PC strain 2 (%)	γ-PC strain 3 (%)	WS_2 strain 1 (%)	WS_2 strain 2 (%)	WS_2 strain 3 (%)	γ (°)	|**a**|(Å)	|**b**|(Å)
13312	286	46	34	C	W	61.359	-3.292	-4.111	-2.112	3.523	4.293	2.206	89.565	18.221	17.087
13313	286	46	34	C	W	66.178	-2.145	4.079	-3.260	2.241	-4.364	3.487	89.506	17.102	18.204
13314	286	46	34	C	W	75.609	-2.145	-0.833	-3.260	2.241	0.892	3.487	87.585	17.102	18.220
13315	286	46	34	C	W	103.463	-3.292	-0.744	-2.112	3.523	0.777	2.206	87.595	18.221	17.101
13316	286	46	34	C	W	113.822	-2.145	-4.079	-3.260	2.241	4.364	3.487	89.506	17.102	18.204
13317	286	46	34	C	W	118.641	-3.292	4.111	-2.112	3.523	-4.293	2.206	89.565	18.221	17.087
13318	286	46	34	P	S	15.608	-2.145	-0.833	-3.260	2.241	0.892	3.487	87.585	17.102	18.220
13319	286	46	34	P	S	43.463	-3.292	-0.744	-2.112	3.523	0.777	2.206	87.595	18.221	17.101
13320	286	46	34	P	S	53.822	-2.145	-4.079	-3.260	2.241	4.364	3.487	89.506	17.102	18.204
13321	286	46	34	P	S	58.641	-3.292	4.111	-2.112	3.523	-4.293	2.206	89.565	18.221	17.087
13322	286	46	34	P	S	61.359	-3.292	-4.111	-2.112	3.523	4.293	2.206	89.565	18.221	17.087
13323	286	46	34	P	S	66.178	-2.145	4.079	-3.260	2.241	-4.364	3.487	89.506	17.102	18.204
13324	286	46	34	P	S	75.609	-2.145	-0.833	-3.260	2.241	0.892	3.487	87.585	17.102	18.220
13325	286	46	34	P	S	103.463	-3.292	-0.744	-2.112	3.523	0.777	2.206	87.595	18.221	17.101
13326	286	46	34	P	W	1.359	-3.292	-4.111	-2.112	3.523	4.293	2.206	89.565	18.221	17.087
13327	286	46	34	P	W	6.178	-2.145	4.079	-3.260	2.241	-4.364	3.487	89.506	17.102	18.204
13328	286	46	34	P	W	15.608	-2.145	-0.833	-3.260	2.241	0.892	3.487	87.585	17.102	18.220
13329	286	46	34	P	W	43.463	-3.292	-0.744	-2.112	3.523	0.777	2.206	87.595	18.221	17.101
13330	286	46	34	P	W	75.609	-2.145	-0.833	-3.260	2.241	0.892	3.487	87.585	17.102	18.220
13331	286	46	34	P	W	103.463	-3.292	-0.744	-2.112	3.523	0.777	2.206	87.595	18.221	17.101
13332	286	46	34	P	W	113.822	-2.145	-4.079	-3.260	2.241	4.364	3.487	89.506	17.102	18.204
13333	286	46	34	P	W	118.641	-3.292	4.111	-2.112	3.523	-4.293	2.206	89.565	18.221	17.087
13334	287	44	37	C	S	0.000	5.011	2.067	-3.741	-4.554	-2.234	4.043	67.806	18.842	18.226
13335	287	44	37	C	S	1.359	-3.292	4.728	4.482	3.523	-4.339	-4.113	67.725	18.221	18.851
13336	287	44	37	C	S	9.430	5.011	0.689	-3.741	-4.554	-0.745	4.043	62.414	18.353	19.548
13337	287	44	37	C	S	13.898	-0.414	-1.809	1.320	0.417	1.763	-1.286	65.942	19.824	17.534
13338	287	44	37	C	S	16.337	0.353	2.485	0.538	-0.351	-2.458	-0.532	65.925	17.539	19.820
13339	287	44	37	C	S	23.691	2.390	0.155	-1.426	-2.281	-0.160	1.468	67.838	19.291	17.771
13340	287	44	37	C	S	25.050	-0.955	2.578	1.886	0.973	-2.485	-1.817	67.806	17.772	19.291
13341	287	44	37	C	S	36.309	2.390	-0.155	-1.426	-2.281	0.160	1.468	67.838	19.291	17.771
13342	287	44	37	C	S	43.663	0.353	-2.485	0.538	-0.351	2.458	-0.532	65.925	17.539	19.820
13343	287	44	37	C	S	46.102	-0.414	1.809	1.320	0.417	-1.763	-1.286	65.942	19.824	17.534
13344	287	44	37	C	S	50.570	5.011	-0.689	-3.741	-4.554	0.745	4.043	62.414	18.353	19.548
13345	287	44	37	C	S	58.641	-3.292	-4.728	4.482	3.523	4.339	-4.113	67.725	18.221	18.851
13346	287	44	37	C	S	60.000	5.011	2.067	-3.741	-4.554	-2.234	4.043	67.806	18.842	18.226
13347	287	44	37	C	S	61.359	-3.292	4.728	4.482	3.523	-4.339	-4.113	67.725	18.221	18.851
13348	287	44	37	C	S	69.430	5.011	0.689	-3.741	-4.554	-0.745	4.043	62.414	18.353	19.548
13349	287	44	37	C	S	73.898	-0.414	-1.809	1.320	0.417	1.763	-1.286	65.942	19.824	17.534
13350	287	44	37	C	S	76.337	0.353	2.485	0.538	-0.351	-2.458	-0.532	65.925	17.539	19.820
13351	287	44	37	C	S	85.050	-0.955	2.578	1.886	0.973	-2.485	-1.817	67.806	17.772	19.291
13352	287	44	37	C	S	94.950	-0.955	-2.578	1.886	0.973	2.484	-1.817	67.806	17.772	19.291
13353	287	44	37	C	S	96.309	2.390	-0.155	-1.426	-2.281	0.160	1.468	67.838	19.291	17.771
13354	287	44	37	C	S	103.663	0.353	-2.485	0.538	-0.351	2.458	-0.532	65.925	17.539	19.820
13355	287	44	37	C	S	106.102	-0.414	1.809	1.320	0.417	-1.763	-1.286	65.942	19.824	17.534
13356	287	44	37	C	S	110.570	5.011	-0.689	-3.741	-4.554	0.745	4.043	62.414	18.353	19.548
13357	287	44	37	C	S	118.641	-3.292	-4.728	4.482	3.523	4.339	-4.113	67.725	18.221	18.851
13358	287	44	37	C	W	0.000	5.011	2.067	-3.741	-4.554	-2.234	4.043	67.806	18.842	18.226
13359	287	44	37	C	W	1.359	-3.292	4.728	4.482	3.523	-4.339	-4.113	67.725	18.221	18.851
13360	287	44	37	C	W	9.430	5.011	0.689	-3.741	-4.554	-0.745	4.043	62.414	18.353	19.548
13361	287	44	37	C	W	13.898	-0.414	-1.809	1.320	0.417	1.763	-1.286	65.942	19.824	17.534
13362	287	44	37	C	W	16.337	0.353	2.485	0.538	-0.351	-2.458	-0.532	65.925	17.539	19.820

BL number	Atoms	γ-PC unit cells	WS_2 unit cells	γ-PC origin atom	WS_2 origin atom	Twist-angle (°)	γ-PC strain 1 (%)	γ-PC strain 2 (%)	γ-PC strain 3 (%)	WS_2 strain 1 (%)	WS_2 strain 2 (%)	WS_2 strain 3 (%)	γ (°)	$\lvert\mathbf{a}\rvert$(Å)	$\lvert\mathbf{b}\rvert$(Å)
13363	287	44	37	C	W	25.050	-0.955	2.578	1.886	0.973	-2.485	-1.817	67.806	17.772	19.291
13364	287	44	37	C	W	34.950	-0.955	-2.578	1.886	0.973	2.484	-1.817	67.806	17.772	19.291
13365	287	44	37	C	W	36.309	2.390	-0.155	-1.426	-2.281	0.160	1.468	67.838	19.291	17.771
13366	287	44	37	C	W	43.663	0.353	-2.485	0.538	-0.351	2.458	-0.532	65.925	17.539	19.820
13367	287	44	37	C	W	46.102	-0.414	1.809	1.320	0.417	-1.763	-1.286	65.942	19.824	17.534
13368	287	44	37	C	W	50.570	5.011	-0.689	-3.741	-4.554	0.745	4.043	62.414	18.353	19.548
13369	287	44	37	C	W	58.641	-3.292	-4.728	4.482	3.523	4.339	-4.113	67.725	18.221	18.851
13370	287	44	37	C	W	60.000	5.011	2.067	-3.741	-4.554	-2.234	4.043	67.806	18.842	18.226
13371	287	44	37	C	W	61.359	-3.292	4.728	4.482	3.523	-4.339	-4.113	67.725	18.221	18.851
13372	287	44	37	C	W	69.430	5.011	0.689	-3.741	-4.554	-0.745	4.043	62.414	18.353	19.548
13373	287	44	37	C	W	73.898	-0.414	-1.809	1.320	0.417	1.763	-1.286	65.942	19.824	17.534
13374	287	44	37	C	W	76.337	0.353	2.485	0.538	-0.351	-2.458	-0.532	65.925	17.539	19.820
13375	287	44	37	C	W	83.691	2.390	0.155	-1.426	-2.281	-0.160	1.468	67.838	19.291	17.771
13376	287	44	37	C	W	85.050	-0.955	2.578	1.886	0.973	-2.485	-1.817	67.806	17.772	19.291
13377	287	44	37	C	W	96.309	2.390	-0.155	-1.426	-2.281	0.160	1.468	67.838	19.291	17.771
13378	287	44	37	C	W	103.663	0.353	-2.485	0.538	-0.351	2.458	-0.532	65.925	17.539	19.820
13379	287	44	37	C	W	106.102	-0.414	1.809	1.320	0.417	-1.763	-1.286	65.942	19.824	17.534
13380	287	44	37	C	W	110.570	5.011	-0.689	-3.741	-4.554	0.745	4.043	62.414	18.353	19.548
13381	287	44	37	C	W	118.641	-3.292	-4.728	4.482	3.523	4.339	-4.113	67.725	18.221	18.851
13382	287	44	37	P	S	0.000	5.011	2.067	-3.741	-4.554	-2.234	4.043	67.806	18.842	18.226
13383	287	44	37	P	S	1.359	-3.292	4.728	4.482	3.523	-4.339	-4.113	67.725	18.221	18.851
13384	287	44	37	P	S	9.430	5.011	0.689	-3.741	-4.554	-0.745	4.043	62.414	18.353	19.548
13385	287	44	37	P	S	25.050	-0.955	2.578	1.886	0.973	-2.485	-1.817	67.806	17.772	19.291
13386	287	44	37	P	S	34.950	-0.955	-2.578	1.886	0.973	2.484	-1.817	67.806	17.772	19.291
13387	287	44	37	P	S	36.309	2.390	-0.155	-1.426	-2.281	0.160	1.468	67.838	19.291	17.771
13388	287	44	37	P	S	43.663	0.353	-2.485	0.538	-0.351	2.458	-0.532	65.925	17.539	19.820
13389	287	44	37	P	S	46.102	-0.414	1.809	1.320	0.417	-1.763	-1.286	65.942	19.824	17.534
13390	287	44	37	P	S	50.570	5.011	-0.689	-3.741	-4.554	0.745	4.043	62.414	18.353	19.548
13391	287	44	37	P	S	60.000	5.011	0.689	-3.741	-4.554	-0.745	4.043	62.414	18.353	19.548
13392	287	44	37	P	S	69.430	5.011	0.689	-3.741	-4.554	-0.745	4.043	62.414	18.353	19.548
13393	287	44	37	P	S	73.898	-0.414	-1.809	1.320	0.417	1.763	-1.286	65.942	19.824	17.534
13394	287	44	37	P	S	76.337	0.353	2.485	0.538	-0.351	-2.458	-0.532	65.925	17.539	19.820
13395	287	44	37	P	S	83.691	2.390	0.155	-1.426	-2.281	-0.160	1.468	67.838	19.291	17.771
13396	287	44	37	P	S	85.050	-0.955	2.578	1.886	0.973	-2.485	-1.817	67.806	17.772	19.291
13397	287	44	37	P	S	96.309	2.390	-0.155	-1.426	-2.281	0.160	1.468	67.838	19.291	17.771
13398	287	44	37	P	S	110.570	5.011	-0.689	-3.741	-4.554	0.745	4.043	62.414	18.353	19.548
13399	287	44	37	P	S	118.641	-3.292	-4.728	4.482	3.523	4.339	-4.113	67.725	18.221	18.851
13400	287	44	37	P	W	0.000	5.011	-0.689	-3.741	-4.554	0.745	4.043	62.414	18.353	19.548
13401	287	44	37	P	W	9.430	5.011	0.689	-3.741	-4.554	-0.745	4.043	62.414	18.353	19.548
13402	287	44	37	P	W	13.898	-0.414	-1.809	1.320	0.417	1.763	-1.286	65.942	19.824	17.534
13403	287	44	37	P	W	16.337	0.353	2.485	0.538	-0.351	-2.458	-0.532	65.925	17.539	19.820
13404	287	44	37	P	W	23.691	2.390	0.155	-1.426	-2.281	-0.160	1.468	67.838	19.291	17.771
13405	287	44	37	P	W	25.050	-0.955	2.578	1.886	0.973	-2.485	-1.817	67.806	17.772	19.291
13406	287	44	37	P	W	36.309	2.390	-0.155	-1.426	-2.281	0.160	1.468	67.838	19.291	17.771
13407	287	44	37	P	W	50.570	5.011	-0.689	-3.741	-4.554	0.745	4.043	62.414	18.353	19.548
13408	287	44	37	P	W	58.641	-3.292	-4.728	4.482	3.523	4.339	-4.113	67.725	18.221	18.851
13409	287	44	37	P	W	60.000	5.011	2.067	-3.741	-4.554	-2.234	4.043	67.806	18.842	18.226
13410	287	44	37	P	W	61.359	-3.292	4.728	4.482	3.523	-4.339	-4.113	67.725	18.221	18.851
13411	287	44	37	P	W	69.430	5.011	0.689	-3.741	-4.554	-0.745	4.043	62.414	18.353	19.548
13412	287	44	37	P	W	85.050	-0.955	2.578	1.886	0.973	-2.485	-1.817	67.806	17.772	19.291
13413	287	44	37	P	W	94.950	-0.955	-2.578	1.886	0.973	2.484	-1.817	67.806	17.772	19.291

BL number	Atoms	γ-PC unit cells	WS_2 unit cells	γ-PC origin atom	WS_2 origin atom	Twist-angle (°)	γ-PC strain 1 (%)	γ-PC strain 2 (%)	γ-PC strain 3 (%)	WS_2 strain 1 (%)	WS_2 strain 2 (%)	WS_2 strain 3 (%)	γ (°)	|**a**|(Å)	|**b**|(Å)
13414	287	44	37	P	W	96.309	2.390	-0.155	-1.426	-2.281	0.160	1.468	67.838	19.291	17.771
13415	287	44	37	P	W	103.663	0.353	-2.485	0.538	-0.351	2.458	-0.532	65.925	17.539	19.820
13416	287	44	37	P	W	106.102	-0.414	1.809	1.320	0.417	-1.763	-1.286	65.942	19.824	17.534
13417	287	44	37	P	W	110.570	5.011	-0.689	-3.741	-4.554	0.745	4.043	62.414	18.353	19.548
13418	287	47	33	C	S	13.898	-4.228	-2.353	-3.579	4.619	2.534	3.855	82.356	17.184	18.218
13419	287	47	33	C	S	16.537	-3.292	2.069	-4.510	3.523	-2.275	4.957	82.388	18.221	17.181
13420	287	47	33	C	S	43.463	-3.292	-2.069	-4.510	3.523	2.275	4.957	82.388	18.221	17.181
13421	287	47	33	C	S	46.102	-4.228	2.353	-3.579	4.619	-2.534	3.855	82.356	17.184	18.218
13422	287	47	33	C	S	73.898	-4.228	-2.353	-3.579	4.619	2.534	3.855	82.356	17.184	18.218
13423	287	47	33	C	S	76.537	-3.292	2.069	-4.510	3.523	-2.275	4.957	82.388	18.221	17.181
13424	287	47	33	C	S	103.463	-3.292	-2.069	-4.510	3.523	2.275	4.957	82.388	18.221	17.181
13425	287	47	33	C	S	106.102	-4.228	2.353	-3.579	4.619	-2.534	3.855	82.356	17.184	18.218
13426	287	47	33	C	W	13.898	-4.228	-2.353	-3.579	4.619	2.534	3.855	82.356	17.184	18.218
13427	287	47	33	C	W	16.537	-3.292	2.069	-4.510	3.523	-2.275	4.957	82.388	18.221	17.181
13428	287	47	33	C	W	43.463	-3.292	-2.069	-4.510	3.523	2.275	4.957	82.388	18.221	17.181
13429	287	47	33	C	W	46.102	-4.228	2.353	-3.579	4.619	-2.534	3.855	82.356	17.184	18.218
13430	287	47	33	C	W	73.898	-4.228	-2.353	-3.579	4.619	2.534	3.855	82.356	17.184	18.218
13431	287	47	33	C	W	76.537	-3.292	2.069	-4.510	3.523	-2.275	4.957	82.388	18.221	17.181
13432	287	47	33	C	W	103.463	-3.292	-2.069	-4.510	3.523	2.275	4.957	82.388	18.221	17.181
13433	287	47	33	C	W	106.102	-4.228	2.353	-3.579	4.619	-2.534	3.855	82.356	17.184	18.218
13434	287	47	33	P	S	46.102	-4.228	2.353	-3.579	4.619	-2.534	3.855	82.356	17.184	18.218
13435	287	47	33	P	W	13.898	-4.228	-2.353	-3.579	4.619	2.534	3.855	82.356	17.184	18.218
13436	287	47	33	P	W	106.102	-4.228	2.353	-3.579	4.619	-2.534	3.855	82.356	17.184	18.218
13437	288	42	40	C	S	30.000	2.934	0.000	4.447	-2.772	0.000	-4.084	90.000	15.368	21.007
13438	288	42	40	C	S	90.000	2.934	0.000	4.447	-2.772	0.000	-4.084	90.000	15.368	21.007
13439	288	42	40	C	W	30.000	2.934	0.000	4.447	-2.772	0.000	-4.084	90.000	15.368	21.007
13440	288	42	40	C	W	90.000	2.934	0.000	4.447	-2.772	0.000	-4.084	90.000	15.368	21.007
13441	288	42	40	P	S	30.000	2.934	0.000	4.447	-2.772	0.000	-4.084	90.000	15.368	21.007
13442	288	42	40	P	S	90.000	2.934	0.000	4.447	-2.772	0.000	-4.084	90.000	15.368	21.007
13443	288	42	40	P	W	30.000	2.934	0.000	4.447	-2.772	0.000	-4.084	90.000	15.368	21.007
13444	288	42	40	P	W	90.000	2.934	0.000	4.447	-2.772	0.000	-4.084	90.000	15.368	21.007
13445	288	45	36	C	S	6.178	-2.145	-0.717	0.590	2.241	0.709	-0.583	78.412	17.102	18.902
13446	288	45	36	C	S	7.589	0.334	1.716	-1.903	-0.332	-1.784	1.978	78.383	18.904	17.101
13447	288	45	36	C	S	15.608	-2.145	-2.601	0.590	2.241	2.570	-0.583	76.611	17.102	19.034
13448	288	45	36	C	S	17.696	1.029	1.033	-2.557	-1.008	-1.089	2.695	76.681	19.034	17.099
13449	288	45	36	C	S	42.304	1.029	-1.033	-2.557	-1.008	1.089	2.695	76.681	19.034	17.099
13450	288	45	36	C	S	44.392	-2.145	2.601	0.590	2.241	-2.570	-0.583	76.611	17.102	19.034
13451	288	45	36	C	S	51.487	2.390	-1.869	-3.789	-2.281	2.023	4.100	75.880	19.291	16.940
13452	288	45	36	C	S	52.411	0.334	-1.716	-1.903	-0.332	1.784	1.978	78.383	18.904	17.101
13453	288	45	36	C	S	53.822	-2.145	0.717	0.590	2.241	-0.709	-0.583	78.412	17.102	18.902
13454	288	45	36	C	S	55.285	-3.007	4.770	1.518	3.200	-4.630	-1.473	75.674	16.951	19.287
13455	288	45	36	C	S	64.715	-3.007	-4.770	1.518	3.200	4.630	-1.473	75.674	16.951	19.287
13456	288	45	36	C	S	66.178	-2.145	-0.717	0.590	2.241	0.709	-0.583	78.412	17.102	18.902
13457	288	45	36	C	S	67.589	0.334	1.716	-1.903	-0.332	-1.784	1.978	78.383	18.904	17.101
13458	288	45	36	C	S	68.513	2.390	1.869	-3.789	-2.281	-2.023	4.100	75.880	19.291	16.940
13459	288	45	36	C	S	75.609	-2.145	-2.601	0.590	2.241	2.570	-0.583	76.611	17.102	19.034
13460	288	45	36	C	S	77.696	1.029	1.033	-2.557	-1.008	-1.089	2.695	76.681	19.034	17.099
13461	288	45	36	C	S	102.304	1.029	-1.033	-2.557	-1.008	1.089	2.695	76.681	19.034	17.099
13462	288	45	36	C	S	104.392	-2.145	2.601	0.590	2.241	-2.570	-0.583	76.611	17.102	19.034
13463	288	45	36	C	S	111.487	2.390	-1.869	-3.789	-2.281	2.023	4.100	75.880	19.291	16.940
13464	288	45	36	C	S	112.411	0.334	-1.716	-1.903	-0.332	1.784	1.978	78.383	18.904	17.101

BL number	Atoms	γ-PC unit cells	WS_2 unit cells	γ-PC origin atom	WS_2 origin atom	Twist-angle (°)	γ-PC strain 1 (%)	γ-PC strain 2 (%)	γ-PC strain 3 (%)	WS_2 strain 1 (%)	WS_2 strain 2 (%)	WS_2 strain 3 (%)	γ (°)	|**a**|(Å)	|**b**|(Å)
13465	288	45	36	C	S	113.822	-2.145	0.717	0.590	2.241	-0.709	-0.583	78.412	17.102	18.902
13466	288	45	36	C	W	4.715	-3.007	-4.770	1.518	3.200	4.630	-1.473	75.674	16.951	19.287
13467	288	45	36	C	W	6.178	-2.145	-0.717	0.590	2.241	0.709	-0.583	78.412	17.102	18.902
13468	288	45	36	C	W	7.589	0.334	1.716	-1.903	-0.332	-1.784	1.978	78.383	18.904	17.101
13469	288	45	36	C	W	8.513	2.390	1.869	-3.789	-2.281	-2.023	4.100	75.880	19.291	16.940
13470	288	45	36	C	W	15.608	-2.145	-2.601	0.590	2.241	2.570	-0.583	76.611	17.102	19.034
13471	288	45	36	C	W	17.696	1.029	1.033	-2.557	-1.008	-1.089	2.695	76.681	19.034	17.099
13472	288	45	36	C	W	42.304	1.029	-1.033	-2.557	-1.008	1.089	2.695	76.681	19.034	17.099
13473	288	45	36	C	W	44.392	-2.145	2.601	0.590	2.241	-2.570	-0.583	76.611	17.102	19.034
13474	288	45	36	C	W	52.411	0.334	-1.716	-1.903	-0.332	1.784	1.978	78.383	18.904	17.101
13475	288	45	36	C	W	53.822	-2.145	0.717	0.590	2.241	-0.709	-0.583	78.412	17.102	18.902
13476	288	45	36	C	W	66.178	-2.145	-0.717	0.590	2.241	0.709	-0.583	78.412	17.102	18.902
13477	288	45	36	C	W	67.589	0.334	1.716	-1.903	-0.332	-1.784	1.978	78.383	18.904	17.101
13478	288	45	36	C	W	75.609	-2.145	-2.601	0.590	2.241	2.570	-0.583	76.611	17.102	19.034
13479	288	45	36	C	W	77.696	1.029	1.033	-2.557	-1.008	-1.089	2.695	76.681	19.034	17.099
13480	288	45	36	C	W	102.304	1.029	-1.033	-2.557	-1.008	1.089	2.695	76.681	19.034	17.099
13481	288	45	36	C	W	104.392	-2.145	2.601	0.590	2.241	-2.570	-0.583	76.611	17.102	19.034
13482	288	45	36	C	W	112.411	0.334	-1.716	-1.903	-0.332	1.784	1.978	78.383	18.904	17.101
13483	288	45	36	C	W	113.822	-2.145	0.717	0.590	2.241	-0.709	-0.583	78.412	17.102	18.902
13484	288	45	36	C	W	115.285	-3.007	4.770	1.518	3.200	-4.630	-1.473	75.674	16.951	19.287
13485	288	45	36	P	S	6.178	-2.145	-0.717	0.590	2.241	0.709	-0.583	78.412	17.102	18.902
13486	288	45	36	P	S	7.589	0.334	1.716	-1.903	-0.332	-1.784	1.978	78.383	18.904	17.101
13487	288	45	36	P	S	15.608	-2.145	-2.601	0.590	2.241	2.570	-0.583	76.611	17.102	19.034
13488	288	45	36	P	S	17.696	1.029	1.033	-2.557	-1.008	-1.089	2.695	76.681	19.034	17.099
13489	288	45	36	P	S	42.304	1.029	-1.033	-2.557	-1.008	1.089	2.695	76.681	19.034	17.099
13490	288	45	36	P	S	44.392	-2.145	2.601	0.590	2.241	-2.570	-0.583	76.611	17.102	19.034
13491	288	45	36	P	S	51.487	2.390	-1.869	-3.789	-2.281	2.023	4.100	75.880	19.291	16.940
13492	288	45	36	P	S	52.411	0.334	-1.716	-1.903	-0.332	1.784	1.978	78.383	18.904	17.101
13493	288	45	36	P	S	53.822	-2.145	0.717	0.590	2.241	-0.709	-0.583	78.412	17.102	18.902
13494	288	45	36	P	S	55.285	-3.007	4.770	1.518	3.200	-4.630	-1.473	75.674	16.951	19.287
13495	288	45	36	P	S	64.715	-3.007	-4.770	1.518	3.200	4.630	-1.473	75.674	16.951	19.287
13496	288	45	36	P	S	66.178	-2.145	-0.717	0.590	2.241	0.709	-0.583	78.412	17.102	18.902
13497	288	45	36	P	S	67.589	0.334	1.716	-1.903	-0.332	-1.784	1.978	78.383	18.904	17.101
13498	288	45	36	P	S	68.513	2.390	1.869	-3.789	-2.281	-2.023	4.100	75.880	19.291	16.940
13499	288	45	36	P	S	75.609	-2.145	-2.601	0.590	2.241	2.570	-0.583	76.611	17.102	19.034
13500	288	45	36	P	S	77.696	1.029	1.033	-2.557	-1.008	-1.089	2.695	76.681	19.034	17.099
13501	288	45	36	P	S	102.304	1.029	-1.033	-2.557	-1.008	1.089	2.695	76.681	19.034	17.099
13502	288	45	36	P	S	104.392	-2.145	2.601	0.590	2.241	-2.570	-0.583	76.611	17.102	19.034
13503	288	45	36	P	S	111.487	2.390	-1.869	-3.789	-2.281	2.023	4.100	75.880	19.291	16.940
13504	288	45	36	P	S	112.411	0.334	-1.716	-1.903	-0.332	1.784	1.978	78.383	18.904	17.101
13505	288	45	36	P	S	113.822	-2.145	0.717	0.590	2.241	-0.709	-0.583	78.412	17.102	18.902
13506	288	45	36	P	W	4.715	-3.007	-4.770	1.518	3.200	4.630	-1.473	75.674	16.951	19.287
13507	288	45	36	P	W	6.178	-2.145	-0.717	0.590	2.241	0.709	-0.583	78.412	17.102	18.902
13508	288	45	36	P	W	7.589	0.334	1.716	-1.903	-0.332	-1.784	1.978	78.383	18.904	17.101
13509	288	45	36	P	W	8.513	2.390	1.869	-3.789	-2.281	-2.023	4.100	75.880	19.291	16.940
13510	288	45	36	P	W	15.608	-2.145	-2.601	0.590	2.241	2.570	-0.583	76.611	17.102	19.034
13511	288	45	36	P	W	17.696	1.029	1.033	-2.557	-1.008	-1.089	2.695	76.681	19.034	17.099
13512	288	45	36	P	W	42.304	1.029	-1.033	-2.557	-1.008	1.089	2.695	76.681	19.034	17.099
13513	288	45	36	P	W	44.392	-2.145	2.601	0.590	2.241	-2.570	-0.583	76.611	17.102	19.034
13514	288	45	36	P	W	52.411	0.334	-1.716	-1.903	-0.332	1.784	1.978	78.383	18.904	17.101
13515	288	45	36	P	W	53.822	-2.145	0.717	0.590	2.241	-0.709	-0.583	78.412	17.102	18.902

BL number	Atoms	γ-PC unit cells	WS_2 unit cells	γ-PC origin atom	WS_2 origin atom	Twist-angle (°)	γ-PC strain 1 (%)	γ-PC strain 2 (%)	γ-PC strain 3 (%)	WS_2 strain 1 (%)	WS_2 strain 2 (%)	WS_2 strain 3 (%)	γ (°)	|**a**|(Å)	|**b**|(Å)
13516	288	45	36	P	W	66.178	-2.145	-0.717	0.590	2.241	0.709	-0.583	78.412	17.102	18.902
13517	288	45	36	P	W	67.589	0.334	1.716	-1.903	-0.332	-1.784	1.978	78.383	18.904	17.101
13518	288	45	36	P	W	75.609	-2.145	-2.601	0.590	2.241	2.570	-0.583	76.611	17.102	19.034
13519	288	45	36	P	W	77.696	1.029	1.033	-2.557	-1.008	-1.089	2.695	76.681	19.034	17.099
13520	288	45	36	P	W	102.304	1.029	-1.033	-2.557	-1.008	1.089	2.695	76.681	19.034	17.099
13521	288	45	36	P	W	104.392	-2.145	2.601	0.590	2.241	-2.570	-0.583	76.611	17.102	19.034
13522	288	45	36	P	W	112.411	0.334	-1.716	-1.903	-0.332	1.784	1.978	78.383	18.904	17.101
13523	288	45	36	P	W	113.822	-2.145	0.717	0.590	2.241	-0.709	-0.583	78.412	17.102	18.902
13524	288	45	36	P	W	115.285	-3.007	4.770	1.518	3.200	-4.630	-1.473	75.674	16.951	19.287
13525	289	43	39	C	S	8.513	2.390	0.000	2.390	-2.281	0.000	-2.281	60.000	19.291	19.291
13526	289	43	39	C	S	23.691	2.390	0.000	2.390	-2.281	0.000	-2.281	60.000	19.291	19.291
13527	289	43	39	C	S	36.309	2.390	0.000	2.390	-2.281	0.000	-2.281	60.000	19.291	19.291
13528	289	43	39	C	S	51.487	2.390	0.000	2.390	-2.281	0.000	-2.281	60.000	19.291	19.291
13529	289	43	39	C	S	68.513	2.390	0.000	2.390	-2.281	0.000	-2.281	60.000	19.291	19.291
13530	289	43	39	C	S	83.691	2.390	0.000	2.390	-2.281	0.000	-2.281	60.000	19.291	19.291
13531	289	43	39	C	S	96.309	2.390	0.000	2.390	-2.281	0.000	-2.281	60.000	19.291	19.291
13532	289	43	39	C	S	111.487	2.390	0.000	2.390	-2.281	0.000	-2.281	60.000	19.291	19.291
13533	289	43	39	C	W	8.513	2.390	0.000	2.390	-2.281	0.000	-2.281	60.000	19.291	19.291
13534	289	43	39	C	W	23.691	2.390	0.000	2.390	-2.281	0.000	-2.281	60.000	19.291	19.291
13535	289	43	39	C	W	36.309	2.390	0.000	2.390	-2.281	0.000	-2.281	60.000	19.291	19.291
13536	289	43	39	C	W	51.487	2.390	0.000	2.390	-2.281	0.000	-2.281	60.000	19.291	19.291
13537	289	43	39	C	W	68.513	2.390	0.000	2.390	-2.281	0.000	-2.281	60.000	19.291	19.291
13538	289	43	39	C	W	83.691	2.390	0.000	2.390	-2.281	0.000	-2.281	60.000	19.291	19.291
13539	289	43	39	C	W	96.309	2.390	0.000	2.390	-2.281	0.000	-2.281	60.000	19.291	19.291
13540	289	43	39	C	W	111.487	2.390	0.000	2.390	-2.281	0.000	-2.281	60.000	19.291	19.291
13541	289	46	35	C	S	25.285	-4.781	-2.032	0.920	5.287	1.995	-0.904	86.138	16.641	19.033
13542	289	46	35	C	S	27.126	1.029	1.148	-4.877	-1.008	-1.272	5.405	86.259	19.034	16.639
13543	289	46	35	C	S	32.874	1.029	-1.148	-4.878	-1.008	1.272	5.405	86.259	19.034	16.639
13544	289	46	35	C	S	34.715	-4.781	2.032	0.920	5.287	-1.995	-0.904	86.138	16.641	19.033
13545	289	46	35	C	S	85.285	-4.781	-2.032	0.920	5.287	1.995	-0.904	86.138	16.641	19.033
13546	289	46	35	C	S	87.126	1.029	1.148	-4.877	-1.008	-1.272	5.405	86.259	19.034	16.639
13547	289	46	35	C	S	92.874	1.029	-1.148	-4.878	-1.008	1.272	5.405	86.259	19.034	16.639
13548	289	46	35	C	S	94.715	-4.781	2.032	0.920	5.287	-1.995	-0.904	86.138	16.641	19.033
13549	289	46	35	C	W	25.285	-4.781	-2.032	0.920	5.287	1.995	-0.904	86.138	16.641	19.033
13550	289	46	35	C	W	27.126	1.029	1.148	-4.877	-1.008	-1.272	5.405	86.259	19.034	16.639
13551	289	46	35	C	W	32.874	1.029	-1.148	-4.878	-1.008	1.272	5.405	86.259	19.034	16.639
13552	289	46	35	C	W	34.715	-4.781	2.032	0.920	5.287	-1.995	-0.904	86.138	16.641	19.033
13553	289	46	35	C	W	85.285	-4.781	-2.032	0.920	5.287	1.995	-0.904	86.138	16.641	19.033
13554	289	46	35	C	W	87.126	1.029	1.148	-4.877	-1.008	-1.272	5.405	86.259	19.034	16.639
13555	289	46	35	C	W	92.874	1.029	-1.148	-4.878	-1.008	1.272	5.405	86.259	19.034	16.639
13556	289	46	35	C	W	94.715	-4.781	2.032	0.920	5.287	-1.995	-0.904	86.138	16.641	19.033
13557	289	46	35	P	S	25.285	-4.781	-2.032	0.920	5.287	1.995	-0.904	86.138	16.641	19.033
13558	289	46	35	P	S	27.126	1.029	1.148	-4.877	-1.008	-1.272	5.405	86.259	19.034	16.639
13559	289	46	35	P	S	32.874	1.029	-1.148	-4.878	-1.008	1.272	5.405	86.259	19.034	16.639
13560	289	46	35	P	S	34.715	-4.781	2.032	0.920	5.287	-1.995	-0.904	86.138	16.641	19.033
13561	289	46	35	P	S	85.285	-4.781	-2.032	0.920	5.287	1.995	-0.904	86.138	16.641	19.033
13562	289	46	35	P	S	87.126	1.029	1.148	-4.877	-1.008	-1.272	5.405	86.259	19.034	16.639
13563	289	46	35	P	S	92.874	1.029	-1.148	-4.878	-1.008	1.272	5.405	86.259	19.034	16.639
13564	289	46	35	P	S	94.715	-4.781	2.032	0.920	5.287	-1.995	-0.904	86.138	16.641	19.033
13565	289	46	35	P	W	25.285	-4.781	-2.032	0.920	5.287	1.995	-0.904	86.138	16.641	19.033
13566	289	46	35	P	W	27.126	1.029	1.148	-4.877	-1.008	-1.272	5.405	86.259	19.034	16.639

BL number	Atoms	γ-PC unit cells	WS_2 unit cells	γ-PC origin atom	WS_2 origin atom	Twist-angle (°)	γ-PC strain 1 (%)	γ-PC strain 2 (%)	γ-PC strain 3 (%)	WS_2 strain 1 (%)	WS_2 strain 2 (%)	WS_2 strain 3 (%)	γ (°)	|**a**|(Å)	|**b**|(Å)
13567	289	46	35	P	W	32.874	1.029	-1.148	-4.878	-1.008	1.272	5.405	86.259	19.034	16.639
13568	289	46	35	P	W	34.715	-4.781	2.032	0.920	5.287	-1.995	-0.904	86.138	16.641	19.033
13569	289	46	35	P	W	85.285	-4.781	-2.032	0.920	5.287	1.995	-0.904	86.138	16.641	19.033
13570	289	46	35	P	W	87.126	1.029	1.148	-4.877	-1.008	-1.272	5.405	86.259	19.034	16.639
13571	289	46	35	P	W	92.874	1.029	-1.148	-4.878	-1.008	1.272	5.405	86.259	19.034	16.639
13572	289	46	35	P	W	94.715	-4.781	2.032	0.920	5.287	-1.995	-0.904	86.138	16.641	19.033
13573	290	44	38	C	S	0.000	5.011	-2.756	-2.491	-4.554	2.900	2.621	78.809	16.799	19.546
13574	290	44	38	C	S	10.158	2.281	-3.595	-0.010	-2.182	3.596	0.010	82.869	16.362	19.815
13575	290	44	38	C	S	13.898	-0.414	3.016	2.707	0.417	-2.861	-2.568	82.970	19.824	16.353
13576	290	44	38	C	S	46.102	-0.414	-3.015	2.707	0.417	2.861	-2.568	82.970	19.824	16.353
13577	290	44	38	C	S	49.842	2.281	3.595	-0.010	-2.182	-3.596	0.010	82.869	16.362	19.815
13578	290	44	38	C	S	60.000	5.011	2.756	-2.491	-4.554	-2.900	2.621	78.809	16.799	19.546
13579	290	44	38	C	S	70.158	2.281	-3.595	-0.010	-2.182	3.596	0.010	82.868	16.362	19.815
13580	290	44	38	C	S	73.898	-0.414	3.016	2.707	0.417	-2.861	-2.568	82.970	19.824	16.353
13581	290	44	38	C	S	106.102	-0.414	-3.015	2.707	0.417	2.861	-2.568	82.970	19.824	16.353
13582	290	44	38	C	S	109.842	2.281	3.595	-0.010	-2.182	-3.596	0.010	82.868	16.362	19.815
13583	290	44	38	C	W	0.000	5.011	-2.756	-2.491	-4.554	2.900	2.621	78.809	16.799	19.546
13584	290	44	38	C	W	10.158	2.281	-3.595	-0.010	-2.182	3.596	0.010	82.869	16.362	19.815
13585	290	44	38	C	W	13.898	-0.414	3.016	2.707	0.417	-2.861	-2.568	82.970	19.824	16.353
13586	290	44	38	C	W	46.102	-0.414	-3.015	2.707	0.417	2.861	-2.568	82.970	19.824	16.353
13587	290	44	38	C	W	49.842	2.281	3.595	-0.010	-2.182	-3.596	0.010	82.869	16.362	19.815
13588	290	44	38	C	W	60.000	5.011	2.756	-2.491	-4.554	-2.900	2.621	78.809	16.799	19.546
13589	290	44	38	C	W	70.158	2.281	-3.595	-0.010	-2.182	3.596	0.010	82.868	16.362	19.815
13590	290	44	38	C	W	73.898	-0.414	3.016	2.707	0.417	-2.861	-2.568	82.970	19.824	16.353
13591	290	44	38	C	W	106.102	-0.414	-3.015	2.707	0.417	2.861	-2.568	82.970	19.824	16.353
13592	290	44	38	C	W	109.842	2.281	3.595	-0.010	-2.182	-3.596	0.010	82.868	16.362	19.815
13593	290	44	38	P	S	0.000	5.011	-2.756	-2.491	-4.554	2.900	2.621	78.809	16.799	19.546
13594	290	44	38	P	S	46.102	-0.414	-3.015	2.707	0.417	2.861	-2.568	82.970	19.824	16.353
13595	290	44	38	P	S	49.842	2.281	3.595	-0.010	-2.182	-3.596	0.010	82.869	16.362	19.815
13596	290	44	38	P	S	60.000	5.011	2.756	-2.491	-4.554	-2.900	2.621	78.809	16.799	19.546
13597	290	44	38	P	S	70.158	2.281	-3.595	-0.010	-2.182	3.596	0.010	82.868	16.362	19.815
13598	290	44	38	P	S	73.898	-0.414	3.016	2.707	0.417	-2.861	-2.568	82.970	19.824	16.353
13599	290	44	38	P	W	0.000	5.011	-2.756	-2.491	-4.554	2.900	2.621	78.809	16.799	19.546
13600	290	44	38	P	W	10.158	2.281	-3.595	-0.010	-2.182	3.596	0.010	82.869	16.362	19.815
13601	290	44	38	P	W	13.898	-0.414	3.016	2.707	0.417	-2.861	-2.568	82.970	19.824	16.353
13602	290	44	38	P	W	60.000	5.011	2.756	-2.491	-4.554	-2.900	2.621	78.809	16.799	19.546
13603	290	44	38	P	W	106.102	-0.414	-3.015	2.707	0.417	2.861	-2.568	82.970	19.824	16.353
13604	290	44	38	P	W	109.842	2.281	3.595	-0.010	-2.182	-3.596	0.010	82.868	16.362	19.815
13605	290	47	34	C	S	56.996	-3.388	-3.687	-3.034	3.635	3.926	3.230	85.875	17.335	18.206
13606	290	47	34	C	S	58.641	-3.292	-3.698	-3.131	3.523	3.945	3.340	85.870	18.221	17.321
13607	290	47	34	C	S	61.359	-3.292	3.698	-3.131	3.523	-3.945	3.340	85.871	18.221	17.321
13608	290	47	34	C	S	63.005	-3.388	3.688	-3.034	3.635	-3.926	3.230	85.875	17.335	18.206
13609	290	47	34	C	S	116.996	-3.388	-3.687	-3.034	3.635	3.926	3.230	85.875	17.335	18.206
13610	290	47	34	C	S	118.641	-3.292	-3.698	-3.131	3.523	3.945	3.340	85.871	18.221	17.321
13611	290	47	34	C	W	56.996	-3.388	-3.687	-3.034	3.635	3.926	3.230	85.875	17.335	18.206
13612	290	47	34	C	W	58.641	-3.292	-3.698	-3.131	3.523	3.945	3.340	85.870	18.221	17.321
13613	290	47	34	C	W	61.359	-3.292	3.698	-3.131	3.523	-3.945	3.340	85.871	18.221	17.321
13614	290	47	34	C	W	63.005	-3.388	3.688	-3.034	3.635	-3.926	3.230	85.875	17.335	18.206
13615	290	47	34	C	W	116.996	-3.388	-3.687	-3.034	3.635	3.926	3.230	85.875	17.335	18.206
13616	290	47	34	C	W	118.641	-3.292	-3.698	-3.131	3.523	3.945	3.340	85.871	18.221	17.321
13617	290	47	34	P	S	56.996	-3.388	-3.687	-3.034	3.635	3.926	3.230	85.875	17.335	18.206

BL number	Atoms	γ-PC unit cells	WS_2 unit cells	γ-PC origin atom	WS_2 origin atom	Twist-angle (°)	γ-PC strain 1 (%)	γ-PC strain 2 (%)	γ-PC strain 3 (%)	WS_2 strain 1 (%)	WS_2 strain 2 (%)	WS_2 strain 3 (%)	γ (°)	|**a**|(Å)	|**b**|(Å)
13618	290	47	34	P	S	58.641	-3.292	-3.698	-3.131	3.523	3.945	3.340	85.870	18.221	17.321
13619	290	47	34	P	S	61.359	-3.292	3.698	-3.131	3.523	-3.945	3.340	85.871	18.221	17.321
13620	290	47	34	P	S	63.005	-3.388	3.688	-3.034	3.635	-3.926	3.230	85.875	17.335	18.206
13621	290	47	34	P	S	116.996	-3.388	-3.687	-3.034	3.635	3.926	3.230	85.875	17.335	18.206
13622	290	47	34	P	S	118.641	-3.292	-3.698	-3.131	3.523	3.945	3.340	85.871	18.221	17.321
13623	290	47	34	P	W	56.996	-3.388	-3.687	-3.034	3.635	3.926	3.230	85.875	17.335	18.206
13624	290	47	34	P	W	58.641	-3.292	-3.698	-3.131	3.523	3.945	3.340	85.870	18.221	17.321
13625	290	47	34	P	W	61.359	-3.292	3.698	-3.131	3.523	-3.945	3.340	85.871	18.221	17.321
13626	290	47	34	P	W	63.005	-3.388	3.688	-3.034	3.635	-3.926	3.230	85.875	17.335	18.206
13627	290	47	34	P	W	116.996	-3.388	-3.687	-3.034	3.635	3.926	3.230	85.875	17.335	18.206
13628	290	47	34	P	W	118.641	-3.292	-3.698	-3.131	3.523	3.945	3.340	85.871	18.221	17.321
13629	292	46	36	C	S	4.715	-3.007	-2.435	0.398	3.200	2.416	-0.395	88.611	16.951	18.897
13630	292	46	36	C	S	7.589	0.334	2.519	-2.948	-0.332	-2.676	3.133	88.513	18.904	16.946
13631	292	46	36	C	S	52.411	0.334	-2.519	-2.948	-0.332	2.676	3.133	88.513	18.904	16.946
13632	292	46	36	C	S	55.285	-3.007	2.435	0.398	3.200	-2.416	-0.395	88.611	16.951	18.897
13633	292	46	36	C	S	64.715	-3.007	-2.435	0.398	3.200	2.416	-0.395	88.611	16.951	18.897
13634	292	46	36	C	S	67.589	0.334	2.519	-2.948	-0.332	-2.676	3.133	88.514	18.904	16.946
13635	292	46	36	C	S	112.411	0.334	-2.519	-2.948	-0.332	2.676	3.133	88.513	18.904	16.946
13636	292	46	36	C	S	115.285	-3.007	2.435	0.398	3.200	-2.416	-0.395	88.611	16.951	18.897
13637	292	46	36	C	W	4.715	-3.007	-2.435	0.398	3.200	2.416	-0.395	88.611	16.951	18.897
13638	292	46	36	C	W	7.589	0.334	2.519	-2.948	-0.332	-2.676	3.133	88.513	18.904	16.946
13639	292	46	36	C	W	52.411	0.334	-2.519	-2.948	-0.332	2.676	3.133	88.513	18.904	16.946
13640	292	46	36	C	W	55.285	-3.007	2.435	0.398	3.200	-2.416	-0.395	88.611	16.951	18.897
13641	292	46	36	C	W	64.715	-3.007	-2.435	0.398	3.200	2.416	-0.395	88.611	16.951	18.897
13642	292	46	36	C	W	67.589	0.334	2.519	-2.948	-0.332	-2.676	3.133	88.514	18.904	16.946
13643	292	46	36	C	W	112.411	0.334	-2.519	-2.948	-0.332	2.676	3.133	88.513	18.904	16.946
13644	292	46	36	C	W	115.285	-3.007	2.435	0.398	3.200	-2.416	-0.395	88.611	16.951	18.897
13645	292	46	36	P	S	55.285	-3.007	2.435	0.398	3.200	-2.416	-0.395	88.611	16.951	18.897
13646	292	46	36	P	W	115.285	-3.007	2.435	0.398	3.200	-2.416	-0.395	88.611	16.951	18.897
13647	293	44	39	C	S	0.000	5.011	-4.823	-1.240	-4.554	4.945	1.272	83.237	16.799	19.556
13648	293	44	39	C	S	60.000	5.011	-4.823	-1.240	-4.554	4.945	1.272	83.237	16.799	19.556
13649	293	44	39	C	W	0.000	5.011	-4.823	-1.240	-4.554	4.945	1.272	83.237	16.799	19.556
13650	293	44	39	C	W	60.000	5.011	-4.823	-1.240	-4.554	4.945	1.272	83.237	16.799	19.556
13651	293	44	39	P	S	0.000	5.011	-4.823	-1.240	-4.554	4.945	1.272	83.237	16.799	19.556
13652	293	44	39	P	S	60.000	5.011	-4.823	-1.240	-4.554	4.945	1.272	83.237	16.799	19.556
13653	293	44	39	P	W	0.000	5.011	-4.823	-1.240	-4.554	4.945	1.272	83.237	16.799	19.556
13654	293	44	39	P	W	60.000	5.011	-4.823	-1.240	-4.554	4.945	1.272	83.237	16.799	19.556
13655	293	47	35	C	S	7.154	-0.955	4.474	-4.052	0.973	-4.869	4.409	80.868	17.772	18.199
13656	293	47	35	C	S	52.846	-0.955	-4.474	-4.052	0.973	4.869	4.409	80.867	17.772	18.199
13657	293	47	35	C	S	67.154	-0.955	4.474	-4.052	0.973	-4.869	4.409	80.867	17.772	18.199
13658	293	47	35	C	S	112.846	-0.955	-4.474	-4.052	0.973	4.869	4.409	80.868	17.772	18.199
13659	293	47	35	C	W	7.154	-0.955	4.474	-4.052	0.973	-4.869	4.409	80.868	17.772	18.199
13660	293	47	35	C	W	52.846	-0.955	-4.474	-4.052	0.973	4.869	4.409	80.867	17.772	18.199
13661	293	47	35	C	W	67.154	-0.955	4.474	-4.052	0.973	-4.869	4.409	80.867	17.772	18.199
13662	293	47	35	C	W	112.846	-0.955	-4.474	-4.052	0.973	4.869	4.409	80.868	17.772	18.199
13663	293	47	35	P	S	7.154	-0.955	4.474	-4.052	0.973	-4.869	4.409	80.868	17.772	18.199
13664	293	47	35	P	S	52.846	-0.955	-4.474	-4.052	0.973	4.869	4.409	80.867	17.772	18.199
13665	293	47	35	P	S	67.154	-0.955	4.474	-4.052	0.973	-4.869	4.409	80.867	17.772	18.199
13666	293	47	35	P	S	112.846	-0.955	-4.474	-4.052	0.973	4.869	4.409	80.868	17.772	18.199
13667	293	47	35	P	W	7.154	-0.955	4.474	-4.052	0.973	-4.869	4.409	80.868	17.772	18.199
13668	293	47	35	P	W	52.846	-0.955	-4.474	-4.052	0.973	4.869	4.409	80.867	17.772	18.199

BL number	Atoms	γ-PC unit cells	WS_2 unit cells	γ-PC origin atom	WS_2 origin atom	Twist-angle (°)	γ-PC strain 1 (%)	γ-PC strain 2 (%)	γ-PC strain 3 (%)	WS_2 strain 1 (%)	WS_2 strain 2 (%)	WS_2 strain 3 (%)	γ (°)	|**a**|(Å)	|**b**|(Å)
13669	293	47	35	P	W	67.154	-0.955	4.474	-4.052	0.973	-4.869	4.409	80.867	17.772	18.199
13670	293	47	35	P	W	112.846	-0.955	-4.474	-4.052	0.973	4.869	4.409	80.868	17.772	18.199
13671	294	45	38	C	S	0.000	5.011	0.674	-3.546	-4.554	-0.725	3.817	77.021	18.353	18.221
13672	294	45	38	C	S	1.359	-3.292	3.222	4.711	3.523	-2.944	-4.305	76.927	18.221	18.356
13673	294	45	38	C	S	3.004	-3.388	3.320	4.824	3.635	-3.028	-4.400	86.159	17.335	18.837
13674	294	45	38	C	S	27.126	1.029	1.033	0.079	-1.008	-1.032	-0.079	77.020	19.034	17.537
13675	294	45	38	C	S	32.874	1.029	-1.033	0.079	-1.008	1.032	-0.079	77.020	19.034	17.537
13676	294	45	38	C	S	34.233	0.353	1.350	0.751	-0.351	-1.330	-0.739	77.012	17.539	19.033
13677	294	45	38	C	S	56.996	-3.388	-3.320	4.824	3.635	3.028	-4.400	86.159	17.335	18.837
13678	294	45	38	C	S	58.641	-3.292	-3.222	4.711	3.523	2.944	-4.305	76.927	18.221	18.356
13679	294	45	38	C	S	60.000	5.011	2.021	-3.546	-4.554	-2.175	3.817	86.403	18.842	17.328
13680	294	45	38	C	S	61.359	-3.292	3.222	4.711	3.523	-2.944	-4.305	76.927	18.221	18.356
13681	294	45	38	C	S	63.005	-3.388	3.320	4.824	3.635	-3.028	-4.400	86.158	17.335	18.837
13682	294	45	38	C	S	85.767	0.353	-1.350	0.750	-0.351	1.330	-0.739	77.012	17.539	19.033
13683	294	45	38	C	S	87.126	1.029	1.033	0.079	-1.008	-1.032	-0.079	77.020	19.034	17.537
13684	294	45	38	C	S	94.233	0.353	1.350	0.751	-0.351	-1.330	-0.739	77.012	17.539	19.033
13685	294	45	38	C	S	116.996	-3.388	-3.320	4.824	3.635	3.028	-4.400	86.158	17.335	18.837
13686	294	45	38	C	S	118.641	-3.292	-3.222	4.711	3.523	2.944	-4.305	76.927	18.221	18.356
13687	294	45	38	C	W	0.000	5.011	0.674	-3.546	-4.554	-0.725	3.817	77.021	18.353	18.221
13688	294	45	38	C	W	1.359	-3.292	3.222	4.711	3.523	-2.944	-4.305	76.927	18.221	18.356
13689	294	45	38	C	W	3.004	-3.388	3.320	4.824	3.635	-3.028	-4.400	86.159	17.335	18.837
13690	294	45	38	C	W	25.767	0.353	-1.350	0.750	-0.351	1.330	-0.739	77.012	17.539	19.033
13691	294	45	38	C	W	27.126	1.029	1.033	0.079	-1.008	-1.032	-0.079	77.020	19.034	17.537
13692	294	45	38	C	W	34.233	0.353	1.350	0.751	-0.351	-1.330	-0.739	77.012	17.539	19.033
13693	294	45	38	C	W	56.996	-3.388	-3.320	4.824	3.635	3.028	-4.400	86.159	17.335	18.837
13694	294	45	38	C	W	58.641	-3.292	-3.222	4.711	3.523	2.944	-4.305	76.927	18.221	18.356
13695	294	45	38	C	W	60.000	5.011	2.021	-3.546	-4.554	-2.175	3.817	86.403	18.842	17.328
13696	294	45	38	C	W	61.359	-3.292	3.222	4.711	3.523	-2.944	-4.305	76.927	18.221	18.356
13697	294	45	38	C	W	63.005	-3.388	3.320	4.824	3.635	-3.028	-4.400	86.158	17.335	18.837
13698	294	45	38	C	W	87.126	1.029	1.033	0.079	-1.008	-1.032	-0.079	77.020	19.034	17.537
13699	294	45	38	C	W	92.874	1.029	-1.033	0.079	-1.008	1.032	-0.079	77.020	19.034	17.537
13700	294	45	38	C	W	94.233	0.353	1.350	0.751	-0.351	-1.330	-0.739	77.012	17.539	19.033
13701	294	45	38	C	W	116.996	-3.388	-3.320	4.824	3.635	3.028	-4.400	86.158	17.335	18.837
13702	294	45	38	C	W	118.641	-3.292	-3.222	4.711	3.523	2.944	-4.305	76.927	18.221	18.356
13703	294	45	38	P	S	0.000	5.011	0.674	-3.546	-4.554	-0.725	3.817	77.021	18.353	18.221
13704	294	45	38	P	S	1.359	-3.292	3.222	4.711	3.523	-2.944	-4.305	76.927	18.221	18.356
13705	294	45	38	P	S	3.004	-3.388	3.320	4.824	3.635	-3.028	-4.400	86.159	17.335	18.837
13706	294	45	38	P	S	25.767	0.353	-1.350	0.750	-0.351	1.330	-0.739	77.012	17.539	19.033
13707	294	45	38	P	S	27.126	1.029	1.033	0.079	-1.008	-1.032	-0.079	77.020	19.034	17.537
13708	294	45	38	P	S	34.233	0.353	1.350	0.751	-0.351	-1.330	-0.739	77.012	17.539	19.033
13709	294	45	38	P	S	58.641	-3.292	-3.222	4.711	3.523	2.944	-4.305	76.927	18.221	18.356
13710	294	45	38	P	S	60.000	5.011	0.674	-3.546	-4.554	-0.725	3.817	77.021	18.353	18.221
13711	294	45	38	P	S	61.359	-3.292	3.222	4.711	3.523	-2.944	-4.305	76.927	18.221	18.356
13712	294	45	38	P	S	87.126	1.029	1.033	0.079	-1.008	-1.032	-0.079	77.020	19.034	17.537
13713	294	45	38	P	S	92.874	1.029	-1.033	0.079	-1.008	1.032	-0.079	77.020	19.034	17.537
13714	294	45	38	P	S	94.233	0.353	1.350	0.751	-0.351	-1.330	-0.739	77.012	17.539	19.033
13715	294	45	38	P	S	116.996	-3.388	-3.320	4.824	3.635	3.028	-4.400	86.158	17.335	18.837
13716	294	45	38	P	S	118.641	-3.292	-3.222	4.711	3.523	2.944	-4.305	76.927	18.221	18.356
13717	294	45	38	P	W	0.000	5.011	0.674	-3.546	-4.554	-0.725	3.817	77.021	18.353	18.221
13718	294	45	38	P	W	1.359	-3.292	3.222	4.711	3.523	-2.944	-4.305	76.927	18.221	18.356
13719	294	45	38	P	W	27.126	1.029	1.033	0.079	-1.008	-1.032	-0.079	77.020	19.034	17.537

BL number	Atoms	γ-PC unit cells	WS_2 unit cells	γ-PC origin atom	WS_2 origin atom	Twist-angle (°)	γ-PC strain 1 (%)	γ-PC strain 2 (%)	γ-PC strain 3 (%)	WS_2 strain 1 (%)	WS_2 strain 2 (%)	WS_2 strain 3 (%)	γ (°)	**a**(Å)	**b**(Å)
13720	294	45	38	P	W	32.874	1.029	-1.033	0.079	-1.008	1.032	-0.079	77.020	19.034	17.537
13721	294	45	38	P	W	34.233	0.353	1.350	0.751	-0.351	-1.330	-0.739	77.012	17.539	19.033
13722	294	45	38	P	W	56.996	-3.388	-3.320	4.824	3.635	3.028	-4.400	86.159	17.335	18.837
13723	294	45	38	P	W	58.641	-3.292	-3.222	4.711	3.523	2.944	-4.305	76.927	18.221	18.356
13724	294	45	38	P	W	60.000	5.011	2.021	-3.546	-4.554	-2.175	3.817	86.403	18.842	17.328
13725	294	45	38	P	W	61.359	-3.292	3.222	4.711	3.523	-2.944	-4.305	76.927	18.221	18.356
13726	294	45	38	P	W	63.005	-3.388	3.320	4.824	3.635	-3.028	-4.400	86.158	17.335	18.837
13727	294	45	38	P	W	85.767	0.353	-1.350	0.750	-0.351	1.330	-0.739	77.012	17.539	19.033
13728	294	45	38	P	W	87.126	1.029	1.033	0.079	-1.008	-1.032	-0.079	77.020	19.034	17.537
13729	294	45	38	P	W	94.233	0.353	1.350	0.751	-0.351	-1.330	-0.739	77.012	17.539	19.033
13730	294	45	38	P	W	118.641	-3.292	-3.222	4.711	3.523	2.944	-4.305	76.927	18.221	18.356
13731	296	44	40	C	S	13.898	-0.414	-3.016	5.481	0.417	2.718	-4.940	83.153	19.824	16.788
13732	296	44	40	C	S	17.897	5.011	4.134	0.010	-4.554	-4.133	-0.010	82.931	16.799	19.816
13733	296	44	40	C	S	42.103	5.011	-4.134	0.010	-4.554	4.133	-0.010	82.931	16.799	19.816
13734	296	44	40	C	S	46.102	-0.414	3.016	5.481	0.417	-2.718	-4.940	83.153	19.824	16.788
13735	296	44	40	C	S	73.898	-0.414	-3.016	5.481	0.417	2.718	-4.940	83.153	19.824	16.788
13736	296	44	40	C	S	77.897	5.011	4.134	0.010	-4.554	-4.133	-0.010	82.931	16.799	19.816
13737	296	44	40	C	S	102.104	5.011	-4.134	0.010	-4.554	4.133	-0.010	82.931	16.799	19.816
13738	296	44	40	C	S	106.102	-0.414	3.016	5.481	0.417	-2.718	-4.940	83.153	19.824	16.788
13739	296	44	40	C	W	13.898	-0.414	-3.016	5.481	0.417	2.718	-4.940	83.153	19.824	16.788
13740	296	44	40	C	W	17.897	5.011	4.134	0.010	-4.554	-4.133	-0.010	82.931	16.799	19.816
13741	296	44	40	C	W	42.103	5.011	-4.134	0.010	-4.554	4.133	-0.010	82.931	16.799	19.816
13742	296	44	40	C	W	46.102	-0.414	3.016	5.481	0.417	-2.718	-4.940	83.153	19.824	16.788
13743	296	44	40	C	W	73.898	-0.414	-3.016	5.481	0.417	2.718	-4.940	83.153	19.824	16.788
13744	296	44	40	C	W	77.897	5.011	4.134	0.010	-4.554	-4.133	-0.010	82.931	16.799	19.816
13745	296	44	40	C	W	102.104	5.011	-4.134	0.010	-4.554	4.133	-0.010	82.931	16.799	19.816
13746	296	44	40	C	W	106.102	-0.414	3.016	5.481	0.417	-2.718	-4.940	83.153	19.824	16.788
13747	296	44	40	P	S	42.103	5.011	-4.134	0.010	-4.554	4.133	-0.010	82.931	16.799	19.816
13748	296	44	40	P	S	46.102	-0.414	3.016	5.481	0.417	-2.718	-4.940	83.153	19.824	16.788
13749	296	44	40	P	S	73.898	-0.414	-3.016	5.481	0.417	2.718	-4.940	83.153	19.824	16.788
13750	296	44	40	P	S	77.897	5.011	4.134	0.010	-4.554	-4.133	-0.010	82.931	16.799	19.816
13751	296	44	40	P	W	13.898	-0.414	-3.016	5.481	0.417	2.718	-4.940	83.153	19.824	16.788
13752	296	44	40	P	W	17.897	5.011	4.134	0.010	-4.554	-4.133	-0.010	82.931	16.799	19.816
13753	296	44	40	P	W	102.104	5.011	-4.134	0.010	-4.554	4.133	-0.010	82.931	16.799	19.816
13754	296	44	40	P	W	106.102	-0.414	3.016	5.481	0.417	-2.718	-4.940	83.153	19.824	16.788
13755	296	47	36	C	S	3.004	-3.388	-4.578	-0.271	3.635	4.603	0.273	81.301	17.335	18.893
13756	296	47	36	C	S	7.589	0.334	3.287	-3.949	-0.332	-3.569	4.288	81.486	18.904	17.321
13757	296	47	36	C	S	52.411	0.334	-3.287	-3.949	-0.332	3.569	4.288	81.486	18.904	17.321
13758	296	47	36	C	S	56.996	-3.388	4.578	-0.271	3.635	-4.603	0.273	81.301	17.335	18.893
13759	296	47	36	C	S	63.005	-3.388	-4.578	-0.271	3.635	4.603	0.273	81.301	17.335	18.893
13760	296	47	36	C	S	67.589	0.334	3.287	-3.949	-0.332	-3.569	4.288	81.486	18.904	17.321
13761	296	47	36	C	S	112.411	0.334	-3.287	-3.949	-0.332	3.569	4.288	81.486	18.904	17.321
13762	296	47	36	C	S	116.996	-3.388	4.578	-0.271	3.635	-4.603	0.273	81.301	17.335	18.893
13763	296	47	36	C	W	3.004	-3.388	-4.578	-0.271	3.635	4.603	0.273	81.301	17.335	18.893
13764	296	47	36	C	W	7.589	0.334	3.287	-3.949	-0.332	-3.569	4.288	81.486	18.904	17.321
13765	296	47	36	C	W	52.411	0.334	-3.287	-3.949	-0.332	3.569	4.288	81.486	18.904	17.321
13766	296	47	36	C	W	56.996	-3.388	4.578	-0.271	3.635	-4.603	0.273	81.301	17.335	18.893
13767	296	47	36	C	W	63.005	-3.388	-4.578	-0.271	3.635	4.603	0.273	81.301	17.335	18.893
13768	296	47	36	C	W	67.589	0.334	3.287	-3.949	-0.332	-3.569	4.288	81.486	18.904	17.321
13769	296	47	36	C	W	112.411	0.334	-3.287	-3.949	-0.332	3.569	4.288	81.486	18.904	17.321
13770	296	47	36	C	W	116.996	-3.388	4.578	-0.271	3.635	-4.603	0.273	81.301	17.335	18.893

BL number	Atoms	γ-PC unit cells	WS_2 unit cells	γ-PC origin atom	WS_2 origin atom	Twist-angle (°)	γ-PC strain 1 (%)	γ-PC strain 2 (%)	γ-PC strain 3 (%)	WS_2 strain 1 (%)	WS_2 strain 2 (%)	WS_2 strain 3 (%)	γ (°)	|**a**|(Å)	|**b**|(Å)
13771	296	47	36	P	S	3.004	-3.388	-4.578	-0.271	3.635	4.603	0.273	81.301	17.335	18.893
13772	296	47	36	P	S	7.589	0.334	3.287	-3.949	-0.332	-3.569	4.288	81.486	18.904	17.321
13773	296	47	36	P	S	52.411	0.334	-3.287	-3.949	-0.332	3.569	4.288	81.486	18.904	17.321
13774	296	47	36	P	S	56.996	-3.388	4.578	-0.271	3.635	-4.603	0.273	81.301	17.335	18.893
13775	296	47	36	P	S	63.005	-3.388	-4.578	-0.271	3.635	4.603	0.273	81.301	17.335	18.893
13776	296	47	36	P	S	67.589	0.334	3.287	-3.949	-0.332	-3.569	4.288	81.486	18.904	17.321
13777	296	47	36	P	S	112.411	0.334	-3.287	-3.949	-0.332	3.569	4.288	81.486	18.904	17.321
13778	296	47	36	P	S	116.996	-3.388	4.578	-0.271	3.635	-4.603	0.273	81.301	17.335	18.893
13779	296	47	36	P	W	3.004	-3.388	-4.578	-0.271	3.635	4.603	0.273	81.301	17.335	18.893
13780	296	47	36	P	W	7.589	0.334	3.287	-3.949	-0.332	-3.569	4.288	81.486	18.904	17.321
13781	296	47	36	P	W	52.411	0.334	-3.287	-3.949	-0.332	3.569	4.288	81.486	18.904	17.321
13782	296	47	36	P	W	56.996	-3.388	4.578	-0.271	3.635	-4.603	0.273	81.301	17.335	18.893
13783	296	47	36	P	W	63.005	-3.388	-4.578	-0.271	3.635	4.603	0.273	81.301	17.335	18.893
13784	296	47	36	P	W	67.589	0.334	3.287	-3.949	-0.332	-3.569	4.288	81.486	18.904	17.321
13785	296	47	36	P	W	112.411	0.334	-3.287	-3.949	-0.332	3.569	4.288	81.486	18.904	17.321
13786	296	47	36	P	W	116.996	-3.388	4.578	-0.271	3.635	-4.603	0.273	81.301	17.335	18.893
13787	297	45	39	C	S	7.154	-0.955	-2.091	3.475	0.973	1.955	-3.249	86.735	17.772	18.583
13788	297	45	39	C	S	9.183	3.582	1.508	-1.052	-3.342	-1.541	1.075	86.821	18.586	17.768
13789	297	45	39	C	S	50.817	3.582	-1.508	-1.052	-3.342	1.541	1.075	86.821	18.586	17.768
13790	297	45	39	C	S	52.846	-0.955	2.091	3.475	0.973	-1.955	-3.249	86.735	17.772	18.583
13791	297	45	39	C	S	67.154	-0.955	-2.091	3.475	0.973	1.955	-3.249	86.735	17.772	18.583
13792	297	45	39	C	S	69.183	3.582	1.508	-1.052	-3.342	-1.541	1.075	86.821	18.586	17.768
13793	297	45	39	C	S	110.817	3.582	-1.508	-1.052	-3.342	1.541	1.075	86.821	18.586	17.768
13794	297	45	39	C	S	112.846	-0.955	2.091	3.475	0.973	-1.955	-3.249	86.735	17.772	18.583
13795	297	45	39	C	W	7.154	-0.955	-2.091	3.475	0.973	1.955	-3.249	86.735	17.772	18.583
13796	297	45	39	C	W	9.183	3.582	1.508	-1.052	-3.342	-1.541	1.075	86.821	18.586	17.768
13797	297	45	39	C	W	50.817	3.582	-1.508	-1.052	-3.342	1.541	1.075	86.821	18.586	17.768
13798	297	45	39	C	W	52.846	-0.955	2.091	3.475	0.973	-1.955	-3.249	86.735	17.772	18.583
13799	297	45	39	C	W	67.154	-0.955	-2.091	3.475	0.973	1.955	-3.249	86.735	17.772	18.583
13800	297	45	39	C	W	69.183	3.582	1.508	-1.052	-3.342	-1.541	1.075	86.821	18.586	17.768
13801	297	45	39	C	W	110.817	3.582	-1.508	-1.052	-3.342	1.541	1.075	86.821	18.586	17.768
13802	297	45	39	C	W	112.846	-0.955	2.091	3.475	0.973	-1.955	-3.249	86.735	17.772	18.583
13803	297	48	35	C	S	60.000	-1.865	0.000	-4.158	1.938	0.000	4.535	76.491	18.219	18.219
13804	297	48	35	P	S	0.000	-1.865	0.000	-4.158	1.938	0.000	4.535	76.491	18.219	18.219
13805	297	48	35	P	W	60.000	-1.865	0.000	-4.158	1.938	0.000	4.535	76.491	18.219	18.219
13806	297	48	35	C	S	0.000	-4.158	-4.150	-1.865	4.535	4.311	1.938	87.578	16.522	19.552
13807	297	48	35	C	S	4.715	-1.702	3.835	-4.313	1.762	-4.197	4.720	87.705	19.567	16.509
13808	297	48	35	C	S	55.285	-1.702	-3.834	-4.313	1.762	4.197	4.720	87.705	19.567	16.509
13809	297	48	35	C	S	58.641	-3.292	-2.308	-2.758	3.523	2.442	2.919	76.535	18.221	18.212
13810	297	48	35	C	S	60.000	-4.158	-4.150	-1.865	4.535	4.311	1.938	87.578	16.522	19.552
13811	297	48	35	C	S	61.359	-3.292	2.308	-2.758	3.523	-2.442	2.919	76.535	18.221	18.212
13812	297	48	35	C	S	64.715	-1.702	3.835	-4.313	1.762	-4.197	4.720	87.705	19.567	16.509
13813	297	48	35	C	S	115.285	-1.702	-3.834	-4.313	1.762	4.197	4.720	87.705	19.567	16.509
13814	297	48	35	C	S	118.641	-3.292	-2.308	-2.758	3.523	2.442	2.919	76.535	18.221	18.212
13815	297	48	35	C	W	0.000	-4.158	-4.150	-1.865	4.535	4.311	1.938	87.578	16.522	19.552
13816	297	48	35	C	W	4.715	-1.702	3.835	-4.313	1.762	-4.197	4.720	87.705	19.567	16.509
13817	297	48	35	C	W	55.285	-1.702	-3.834	-4.313	1.762	4.197	4.720	87.705	19.567	16.509
13818	297	48	35	C	W	60.000	-4.158	-4.150	-1.865	4.535	4.311	1.938	87.578	16.522	19.552
13819	297	48	35	C	W	64.715	-1.702	3.835	-4.313	1.762	-4.197	4.720	87.705	19.567	16.509
13820	297	48	35	C	W	115.285	-1.702	-3.834	-4.313	1.762	4.197	4.720	87.705	19.567	16.509
13821	297	48	35	P	S	55.285	-1.702	-3.834	-4.313	1.762	4.197	4.720	87.705	19.567	16.509

BL number	Atoms	γ-PC unit cells	WS_2 unit cells	γ-PC origin atom	WS_2 origin atom	Twist-angle (°)	γ-PC strain 1 (%)	γ-PC strain 2 (%)	γ-PC strain 3 (%)	WS_2 strain 1 (%)	WS_2 strain 2 (%)	WS_2 strain 3 (%)	γ (°)	|**a**|(Å)	|**b**|(Å)
13822	297	48	35	P	S	58.641	-3.292	-2.308	-2.758	3.523	2.442	2.919	76.535	18.221	18.212
13823	297	48	35	P	S	60.000	-4.158	-4.150	-1.865	4.535	4.311	1.938	87.578	16.522	19.552
13824	297	48	35	P	S	61.359	-3.292	2.308	-2.758	3.523	-2.442	2.919	76.535	18.221	18.212
13825	297	48	35	P	S	64.715	-1.702	3.835	-4.313	1.762	-4.197	4.720	87.705	19.567	16.509
13826	297	48	35	P	S	118.641	-3.292	-2.308	-2.758	3.523	2.442	2.919	76.535	18.221	18.212
13827	297	48	35	P	W	0.000	-4.158	-4.150	-1.865	4.535	4.311	1.938	87.578	16.522	19.552
13828	297	48	35	P	W	4.715	-1.702	3.835	-4.313	1.762	-4.197	4.720	87.705	19.567	16.509
13829	297	48	35	P	W	115.285	-1.702	-3.834	-4.313	1.762	4.197	4.720	87.705	19.567	16.509
13830	298	43	42	C	S	25.285	4.262	-2.450	4.473	-3.928	2.249	-4.106	77.350	18.222	18.834
13831	298	43	42	C	S	27.796	5.011	2.115	3.732	-4.554	-1.968	-3.472	77.359	18.842	18.214
13832	298	43	42	C	S	32.204	5.011	-2.115	3.732	-4.554	1.968	-3.472	77.359	18.842	18.214
13833	298	43	42	C	S	34.715	4.262	2.450	4.473	-3.928	-2.249	-4.106	77.350	18.222	18.834
13834	298	43	42	C	S	85.285	4.262	-2.450	4.473	-3.928	2.249	-4.106	77.350	18.222	18.834
13835	298	43	42	C	S	87.796	5.011	2.115	3.732	-4.554	-1.968	-3.472	77.359	18.842	18.214
13836	298	43	42	C	S	92.204	5.011	-2.115	3.732	-4.554	1.968	-3.472	77.359	18.842	18.214
13837	298	43	42	C	S	94.715	4.262	2.450	4.473	-3.928	-2.249	-4.106	77.350	18.222	18.834
13838	298	43	42	C	W	25.285	4.262	-2.450	4.473	-3.928	2.249	-4.106	77.350	18.222	18.834
13839	298	43	42	C	W	27.796	5.011	2.115	3.732	-4.554	-1.968	-3.472	77.359	18.842	18.214
13840	298	43	42	C	W	32.204	5.011	-2.115	3.732	-4.554	1.968	-3.472	77.359	18.842	18.214
13841	298	43	42	C	W	34.715	4.262	2.450	4.473	-3.928	-2.249	-4.106	77.350	18.222	18.834
13842	298	43	42	C	W	85.285	4.262	-2.450	4.473	-3.928	2.249	-4.106	77.350	18.222	18.834
13843	298	43	42	C	W	87.796	5.011	2.115	3.732	-4.554	-1.968	-3.472	77.359	18.842	18.214
13844	298	43	42	C	W	92.204	5.011	-2.115	3.732	-4.554	1.968	-3.472	77.359	18.842	18.214
13845	298	43	42	C	W	94.715	4.262	2.450	4.473	-3.928	-2.249	-4.106	77.350	18.222	18.834
13846	298	43	42	P	S	25.285	4.262	-2.450	4.473	-3.928	2.249	-4.106	77.350	18.222	18.834
13847	298	43	42	P	S	27.796	5.011	2.115	3.732	-4.554	-1.968	-3.472	77.359	18.842	18.214
13848	298	43	42	P	S	92.204	5.011	-2.115	3.732	-4.554	1.968	-3.472	77.359	18.842	18.214
13849	298	43	42	P	S	94.715	4.262	2.450	4.473	-3.928	-2.249	-4.106	77.350	18.222	18.834
13850	298	43	42	P	W	32.204	5.011	-2.115	3.732	-4.554	1.968	-3.472	77.359	18.842	18.214
13851	298	43	42	P	W	34.715	4.262	2.450	4.473	-3.928	-2.249	-4.106	77.350	18.222	18.834
13852	298	43	42	P	W	85.285	4.262	-2.450	4.473	-3.928	2.249	-4.106	77.350	18.222	18.834
13853	298	43	42	P	W	87.796	5.011	2.115	3.732	-4.554	-1.968	-3.472	77.359	18.842	18.214
13854	298	43	42	P	W	94.715	4.262	2.450	4.473	-3.928	-2.249	-4.106	77.350	18.222	18.834
13855	298	46	38	C	S	6.178	-2.145	-2.413	2.239	2.241	2.309	-2.143	85.902	17.102	19.288
13856	298	46	38	C	S	8.513	2.390	1.680	-2.283	-2.281	-1.761	2.392	86.007	19.291	17.098
13857	298	46	38	C	S	51.487	2.390	-1.680	-2.283	-2.281	1.761	2.392	86.007	19.291	17.098
13858	298	46	38	C	S	53.822	-2.145	2.413	2.239	2.241	-2.309	-2.143	85.902	17.102	19.288
13859	298	46	38	C	S	66.178	-2.145	-2.413	2.239	2.241	2.309	-2.143	85.902	17.102	19.288
13860	298	46	38	C	S	68.513	2.390	1.680	-2.283	-2.281	-1.761	2.392	86.007	19.291	17.098
13861	298	46	38	C	S	111.487	2.390	-1.680	-2.283	-2.281	1.761	2.392	86.007	19.291	17.098
13862	298	46	38	C	S	113.822	-2.145	2.413	2.239	2.241	-2.309	-2.143	85.902	17.102	19.288
13863	298	46	38	C	W	6.178	-2.145	-2.413	2.239	2.241	2.309	-2.143	85.902	17.102	19.288
13864	298	46	38	C	W	8.513	2.390	1.680	-2.283	-2.281	-1.761	2.392	86.007	19.291	17.098
13865	298	46	38	C	W	51.487	2.390	-1.680	-2.283	-2.281	1.761	2.392	86.007	19.291	17.098
13866	298	46	38	C	W	53.822	-2.145	2.413	2.239	2.241	-2.309	-2.143	85.902	17.102	19.288
13867	298	46	38	C	W	66.178	-2.145	-2.413	2.239	2.241	2.309	-2.143	85.902	17.102	19.288
13868	298	46	38	C	W	68.513	2.390	1.680	-2.283	-2.281	-1.761	2.392	86.007	19.291	17.098
13869	298	46	38	C	W	111.487	2.390	-1.680	-2.283	-2.281	1.761	2.392	86.007	19.291	17.098
13870	298	46	38	C	W	113.822	-2.145	2.413	2.239	2.241	-2.309	-2.143	85.902	17.102	19.288
13871	298	46	38	P	S	6.178	-2.145	-2.413	2.239	2.241	2.309	-2.143	85.902	17.102	19.288
13872	298	46	38	P	S	8.513	2.390	1.680	-2.283	-2.281	-1.761	2.392	86.007	19.291	17.098

BL number	Atoms	γ-PC unit cells	WS_2 unit cells	γ-PC origin atom	WS_2 origin atom	Twist-angle (°)	γ-PC strain 1 (%)	γ-PC strain 2 (%)	γ-PC strain 3 (%)	WS_2 strain 1 (%)	WS_2 strain 2 (%)	WS_2 strain 3 (%)	γ (°)	|**a**|(Å)	|**b**|(Å)
13873	298	46	38	P	S	51.487	2.390	-1.680	-2.283	-2.281	1.761	2.392	86.007	19.291	17.098
13874	298	46	38	P	S	53.822	-2.145	2.413	2.239	2.241	-2.309	-2.143	85.902	17.102	19.288
13875	298	46	38	P	S	66.178	-2.145	-2.413	2.239	2.241	2.309	-2.143	85.902	17.102	19.288
13876	298	46	38	P	S	68.513	2.390	1.680	-2.283	-2.281	-1.761	2.392	86.007	19.291	17.098
13877	298	46	38	P	S	111.487	2.390	-1.680	-2.283	-2.281	1.761	2.392	86.007	19.291	17.098
13878	298	46	38	P	S	113.822	-2.145	2.413	2.239	2.241	-2.309	-2.143	85.902	17.102	19.288
13879	298	46	38	P	W	6.178	-2.145	-2.413	2.239	2.241	2.309	-2.143	85.902	17.102	19.288
13880	298	46	38	P	W	8.513	2.390	1.680	-2.283	-2.281	-1.761	2.392	86.007	19.291	17.098
13881	298	46	38	P	W	51.487	2.390	-1.680	-2.283	-2.281	1.761	2.392	86.007	19.291	17.098
13882	298	46	38	P	W	53.822	-2.145	2.413	2.239	2.241	-2.309	-2.143	85.902	17.102	19.288
13883	298	46	38	P	W	66.178	-2.145	-2.413	2.239	2.241	2.309	-2.143	85.902	17.102	19.288
13884	298	46	38	P	W	68.513	2.390	1.680	-2.283	-2.281	-1.761	2.392	86.007	19.291	17.098
13885	298	46	38	P	W	111.487	2.390	-1.680	-2.283	-2.281	1.761	2.392	86.007	19.291	17.098
13886	298	46	38	P	W	113.822	-2.145	2.413	2.239	2.241	-2.309	-2.143	85.902	17.102	19.288
13887	299	44	41	C	S	8.513	2.390	-0.155	3.825	-2.281	0.144	-3.553	68.858	19.291	18.586
13888	299	44	41	C	S	27.126	1.029	0.914	5.260	-1.008	-0.827	-4.760	68.868	19.034	18.841
13889	299	44	41	C	S	27.796	5.011	2.067	1.260	-4.554	-2.016	-1.229	68.803	18.842	19.040
13890	299	44	41	C	S	32.204	5.011	-2.067	1.260	-4.554	2.016	-1.229	68.803	18.842	19.040
13891	299	44	41	C	S	32.874	1.029	-0.914	5.260	-1.008	0.827	-4.760	68.868	19.034	18.841
13892	299	44	41	C	S	50.817	3.582	-1.047	2.627	-3.342	0.995	-2.496	68.838	18.586	19.293
13893	299	44	41	C	S	68.513	2.390	-0.155	3.825	-2.281	0.144	-3.553	68.858	19.291	18.586
13894	299	44	41	C	S	87.126	1.029	0.914	5.260	-1.008	-0.827	-4.760	68.868	19.034	18.841
13895	299	44	41	C	S	87.796	5.011	2.067	1.260	-4.554	-2.016	-1.229	68.803	18.842	19.040
13896	299	44	41	C	S	92.204	5.011	-2.067	1.260	-4.554	2.016	-1.229	68.803	18.842	19.040
13897	299	44	41	C	S	92.874	1.029	-0.914	5.260	-1.008	0.827	-4.760	68.868	19.034	18.841
13898	299	44	41	C	S	110.817	3.582	-1.047	2.627	-3.342	0.995	-2.496	68.838	18.586	19.293
13899	299	44	41	C	W	8.513	2.390	-0.155	3.825	-2.281	0.144	-3.553	68.858	19.291	18.586
13900	299	44	41	C	W	27.126	1.029	0.914	5.260	-1.008	-0.827	-4.760	68.868	19.034	18.841
13901	299	44	41	C	W	27.796	5.011	2.067	1.260	-4.554	-2.016	-1.229	68.803	18.842	19.040
13902	299	44	41	C	W	32.204	5.011	-2.067	1.260	-4.554	2.016	-1.229	68.803	18.842	19.040
13903	299	44	41	C	W	32.874	1.029	-0.914	5.260	-1.008	0.827	-4.760	68.868	19.034	18.841
13904	299	44	41	C	W	50.817	3.582	-1.047	2.627	-3.342	0.995	-2.496	68.838	18.586	19.293
13905	299	44	41	C	W	68.513	2.390	-0.155	3.825	-2.281	0.144	-3.553	68.858	19.291	18.586
13906	299	44	41	C	W	87.126	1.029	0.914	5.260	-1.008	-0.827	-4.760	68.868	19.034	18.841
13907	299	44	41	C	W	87.796	5.011	2.067	1.260	-4.554	-2.016	-1.229	68.803	18.842	19.040
13908	299	44	41	C	W	92.204	5.011	-2.067	1.260	-4.554	2.016	-1.229	68.803	18.842	19.040
13909	299	44	41	C	W	92.874	1.029	-0.914	5.260	-1.008	0.827	-4.760	68.868	19.034	18.841
13910	299	44	41	C	W	110.817	3.582	-1.047	2.627	-3.342	0.995	-2.496	68.838	18.586	19.293
13911	299	44	41	P	S	8.513	2.390	-0.155	3.825	-2.281	0.144	-3.553	68.858	19.291	18.586
13912	299	44	41	P	S	27.126	1.029	0.914	5.260	-1.008	-0.827	-4.760	68.868	19.034	18.841
13913	299	44	41	P	S	27.796	5.011	2.067	1.260	-4.554	-2.016	-1.229	68.803	18.842	19.040
13914	299	44	41	P	S	50.817	3.582	-1.047	2.627	-3.342	0.995	-2.496	68.838	18.586	19.293
13915	299	44	41	P	S	68.513	2.390	-0.155	3.825	-2.281	0.144	-3.553	68.858	19.291	18.586
13916	299	44	41	P	S	87.796	5.011	2.067	1.260	-4.554	-2.016	-1.229	68.803	18.842	19.040
13917	299	44	41	P	S	92.204	5.011	-2.067	1.260	-4.554	2.016	-1.229	68.803	18.842	19.040
13918	299	44	41	P	S	92.874	1.029	-0.914	5.260	-1.008	0.827	-4.760	68.868	19.034	18.841
13919	299	44	41	P	S	110.817	3.582	-1.047	2.627	-3.342	0.995	-2.496	68.838	18.586	19.293
13920	299	44	41	P	W	8.513	2.390	-0.155	3.825	-2.281	0.144	-3.553	68.858	19.291	18.586
13921	299	44	41	P	W	27.796	5.011	2.067	1.260	-4.554	-2.016	-1.229	68.803	18.842	19.040
13922	299	44	41	P	W	32.204	5.011	-2.067	1.260	-4.554	2.016	-1.229	68.803	18.842	19.040
13923	299	44	41	P	W	32.874	1.029	-0.914	5.260	-1.008	0.827	-4.760	68.868	19.034	18.841

BL number	Atoms	γ-PC unit cells	WS_2 unit cells	γ-PC origin atom	WS_2 origin atom	Twist-angle (°)	γ-PC strain 1 (%)	γ-PC strain 2 (%)	γ-PC strain 3 (%)	WS_2 strain 1 (%)	WS_2 strain 2 (%)	WS_2 strain 3 (%)	γ (°)	|**a**|(Å)	|**b**|(Å)
13924	299	44	41	P	W	50.817	3.582	-1.047	2.627	-3.342	0.995	-2.496	68.838	18.586	19.293
13925	299	44	41	P	W	68.513	2.390	-0.155	3.825	-2.281	0.144	-3.553	68.858	19.291	18.586
13926	299	44	41	P	W	87.126	1.029	0.914	5.260	-1.008	-0.827	-4.760	68.868	19.034	18.841
13927	299	44	41	P	W	87.796	5.011	2.067	1.260	-4.554	-2.016	-1.229	68.803	18.842	19.040
13928	299	44	41	P	W	110.817	3.582	-1.047	2.627	-3.342	0.995	-2.496	68.838	18.586	19.293
13929	300	45	40	C	S	25.050	-0.955	-2.890	4.846	0.973	2.635	-4.418	86.342	17.772	18.837
13930	300	45	40	C	S	27.796	5.011	2.021	-1.101	-4.554	-2.066	1.126	86.492	18.842	17.766
13931	300	45	40	C	S	32.204	5.011	-2.021	-1.101	-4.554	2.066	1.126	86.492	18.842	17.766
13932	300	45	40	C	S	34.950	-0.955	2.890	4.846	0.973	-2.635	-4.418	86.342	17.772	18.837
13933	300	45	40	C	S	85.050	-0.955	-2.890	4.846	0.973	2.635	-4.418	86.342	17.772	18.837
13934	300	45	40	C	S	87.796	5.011	2.021	-1.101	-4.554	-2.066	1.126	86.492	18.842	17.766
13935	300	45	40	C	S	92.204	5.011	-2.021	-1.101	-4.554	2.066	1.126	86.492	18.842	17.766
13936	300	45	40	C	S	94.950	-0.955	2.890	4.846	0.973	-2.635	-4.418	86.342	17.772	18.837
13937	300	45	40	C	W	25.050	-0.955	-2.890	4.846	0.973	2.635	-4.418	86.342	17.772	18.837
13938	300	45	40	C	W	27.796	5.011	2.021	-1.101	-4.554	-2.066	1.126	86.492	18.842	17.766
13939	300	45	40	C	W	32.204	5.011	-2.021	-1.101	-4.554	2.066	1.126	86.492	18.842	17.766
13940	300	45	40	C	W	34.950	-0.955	2.890	4.846	0.973	-2.635	-4.418	86.342	17.772	18.837
13941	300	45	40	C	W	85.050	-0.955	-2.890	4.846	0.973	2.635	-4.418	86.342	17.772	18.837
13942	300	45	40	C	W	87.796	5.011	2.021	-1.101	-4.554	-2.066	1.126	86.492	18.842	17.766
13943	300	45	40	C	W	92.204	5.011	-2.021	-1.101	-4.554	2.066	1.126	86.492	18.842	17.766
13944	300	45	40	C	W	94.950	-0.955	2.890	4.846	0.973	-2.635	-4.418	86.342	17.772	18.837
13945	300	45	40	P	S	25.050	-0.955	-2.890	4.846	0.973	2.635	-4.418	86.342	17.772	18.837
13946	300	45	40	P	S	27.796	5.011	2.021	-1.101	-4.554	-2.066	1.126	86.492	18.842	17.766
13947	300	45	40	P	S	92.204	5.011	-2.021	-1.101	-4.554	2.066	1.126	86.492	18.842	17.766
13948	300	45	40	P	S	94.950	-0.955	2.890	4.846	0.973	-2.635	-4.418	86.342	17.772	18.837
13949	300	45	40	P	W	32.204	5.011	-2.021	-1.101	-4.554	2.066	1.126	86.492	18.842	17.766
13950	300	45	40	P	W	34.950	-0.955	2.890	4.846	0.973	-2.635	-4.418	86.342	17.772	18.837
13951	300	45	40	P	W	85.050	-0.955	-2.890	4.846	0.973	2.635	-4.418	86.342	17.772	18.837
13952	300	45	40	P	W	87.796	5.011	2.021	-1.101	-4.554	-2.066	1.126	86.492	18.842	17.766
13953	300	48	36	C	S	3.004	-3.388	-0.996	-1.307	3.635	1.023	1.342	74.757	17.335	19.563
13954	300	48	36	C	S	4.715	-1.702	1.917	-3.007	1.762	-2.040	3.200	74.741	19.567	17.332
13955	300	48	36	C	S	7.589	0.334	4.023	-4.909	-0.332	-4.461	5.443	72.177	18.904	18.193
13956	300	48	36	C	S	13.898	-0.414	1.106	-4.228	0.417	-1.208	4.619	73.900	19.824	17.184
13957	300	48	36	C	S	46.102	-0.414	-1.106	-4.228	0.417	1.208	4.619	73.900	19.824	17.184
13958	300	48	36	C	S	52.411	0.334	-4.023	-4.909	-0.332	4.461	5.443	72.177	18.904	18.193
13959	300	48	36	C	S	55.285	-1.702	-1.917	-3.007	1.762	2.040	3.200	74.741	19.567	17.332
13960	300	48	36	C	S	56.996	-3.388	0.996	-1.307	3.635	-1.023	1.342	74.757	17.335	19.563
13961	300	48	36	C	S	63.005	-3.388	-0.996	-1.307	3.635	1.023	1.342	74.757	17.335	19.563
13962	300	48	36	C	S	64.715	-1.702	1.917	-3.007	1.762	-2.040	3.200	74.741	19.567	17.332
13963	300	48	36	C	S	67.589	0.334	4.023	-4.909	-0.332	-4.461	5.443	72.177	18.904	18.193
13964	300	48	36	C	S	73.898	-4.228	1.152	-0.414	4.619	-1.162	0.417	73.871	17.184	19.826
13965	300	48	36	C	S	106.102	-0.414	-1.106	-4.228	0.417	1.208	4.619	73.900	19.824	17.184
13966	300	48	36	C	S	112.411	0.334	-4.023	-4.909	-0.332	4.461	5.443	72.177	18.904	18.193
13967	300	48	36	C	S	115.285	-1.702	-1.917	-3.007	1.762	2.040	3.200	74.741	19.567	17.332
13968	300	48	36	C	S	116.996	-3.388	0.996	-1.307	3.635	-1.023	1.342	74.757	17.335	19.563
13969	300	48	36	C	W	3.004	-3.388	-0.996	-1.307	3.635	1.023	1.342	74.757	17.335	19.563
13970	300	48	36	C	W	4.715	-1.702	1.917	-3.007	1.762	-2.040	3.200	74.741	19.567	17.332
13971	300	48	36	C	W	7.589	0.334	4.023	-4.909	-0.332	-4.461	5.443	72.177	18.904	18.193
13972	300	48	36	C	W	13.898	-4.228	1.152	-0.414	4.619	-1.162	0.417	73.871	17.184	19.826
13973	300	48	36	C	W	52.411	0.334	-4.023	-4.909	-0.332	4.461	5.443	72.177	18.904	18.193
13974	300	48	36	C	W	55.285	-1.702	-1.917	-3.007	1.762	2.040	3.200	74.741	19.567	17.332

BL number	Atoms	γ-PC unit cells	WS_2 unit cells	γ-PC origin atom	WS_2 origin atom	Twist-angle (°)	γ-PC strain 1 (%)	γ-PC strain 2 (%)	γ-PC strain 3 (%)	WS_2 strain 1 (%)	WS_2 strain 2 (%)	WS_2 strain 3 (%)	γ (°)	|**a**|(Å)	|**b**|(Å)
13975	300	48	36	C	W	56.996	-3.388	0.996	-1.307	3.635	-1.023	1.342	74.757	17.335	19.563
13976	300	48	36	C	W	63.005	-3.388	-0.996	-1.307	3.635	1.023	1.342	74.757	17.335	19.563
13977	300	48	36	C	W	64.715	-1.702	1.917	-3.007	1.762	-2.040	3.200	74.741	19.567	17.332
13978	300	48	36	C	W	67.589	0.334	4.023	-4.909	-0.332	-4.461	5.443	72.177	18.904	18.193
13979	300	48	36	C	W	106.102	-4.228	-1.152	-0.414	4.619	1.162	0.417	73.871	17.184	19.826
13980	300	48	36	C	W	112.411	0.334	-4.023	-4.909	-0.332	4.461	5.443	72.177	18.904	18.193
13981	300	48	36	C	W	115.285	-1.702	-1.917	-3.007	1.762	2.040	3.200	74.741	19.567	17.332
13982	300	48	36	C	W	116.996	-3.388	0.996	-1.307	3.635	-1.023	1.342	74.757	17.335	19.563
13983	300	48	36	P	S	7.589	0.334	4.023	-4.909	-0.332	-4.461	5.443	72.177	18.904	18.193
13984	300	48	36	P	S	52.411	0.334	-4.023	-4.909	-0.332	4.461	5.443	72.177	18.904	18.193
13985	300	48	36	P	S	67.589	0.334	4.023	-4.909	-0.332	-4.461	5.443	72.177	18.904	18.193
13986	300	48	36	P	S	112.411	0.334	-4.023	-4.909	-0.332	4.461	5.443	72.177	18.904	18.193
13987	300	48	36	P	W	7.589	0.334	4.023	-4.909	-0.332	-4.461	5.443	72.177	18.904	18.193
13988	300	48	36	P	W	52.411	0.334	-4.023	-4.909	-0.332	4.461	5.443	72.177	18.904	18.193
13989	300	48	36	P	W	67.589	0.334	4.023	-4.909	-0.332	-4.461	5.443	72.177	18.904	18.193
13990	300	48	36	P	W	112.411	0.334	-4.023	-4.909	-0.332	4.461	5.443	72.177	18.904	18.193
13991	301	43	43	C	S	15.178	5.011	0.000	5.011	-4.554	0.000	-4.555	60.000	19.785	19.785
13992	301	43	43	C	S	44.822	5.011	0.000	5.011	-4.554	0.000	-4.555	60.000	19.785	19.785
13993	301	43	43	C	S	75.178	5.011	0.000	5.011	-4.554	0.000	-4.555	60.000	19.785	19.785
13994	301	43	43	C	S	104.822	5.011	0.000	5.011	-4.554	0.000	-4.555	60.000	19.785	19.785
13995	301	43	43	C	W	15.178	5.011	0.000	5.011	-4.554	0.000	-4.555	60.000	19.785	19.785
13996	301	43	43	C	W	44.822	5.011	0.000	5.011	-4.554	0.000	-4.555	60.000	19.785	19.785
13997	301	43	43	C	W	75.178	5.011	0.000	5.011	-4.554	0.000	-4.555	60.000	19.785	19.785
13998	301	43	43	C	W	104.822	5.011	0.000	5.011	-4.554	0.000	-4.555	60.000	19.785	19.785
13999	301	43	43	P	S	15.178	5.011	0.000	5.011	-4.554	0.000	-4.555	60.000	19.785	19.785
14000	301	43	43	P	S	104.822	5.011	0.000	5.011	-4.554	0.000	-4.555	60.000	19.785	19.785
14001	301	43	43	P	W	44.822	5.011	0.000	5.011	-4.554	0.000	-4.555	60.000	19.785	19.785
14002	301	43	43	P	W	75.178	5.011	0.000	5.011	-4.554	0.000	-4.555	60.000	19.785	19.785
14003	301	46	39	C	S	0.000	5.011	-0.659	-3.360	-4.554	0.707	3.602	86.488	18.353	18.219
14004	301	46	39	C	S	1.359	-3.292	1.781	4.930	3.523	-1.622	-4.487	86.381	18.221	18.352
14005	301	46	39	C	S	16.337	0.353	-1.239	0.954	-0.351	1.216	-0.936	86.442	17.539	19.033
14006	301	46	39	C	S	17.696	1.029	1.148	0.279	-1.008	-1.142	-0.278	86.451	19.034	17.537
14007	301	46	39	C	S	42.304	1.029	-1.148	0.279	-1.008	1.142	-0.278	86.451	19.034	17.537
14008	301	46	39	C	S	43.663	0.353	1.239	0.954	-0.351	-1.216	-0.936	86.442	17.539	19.033
14009	301	46	39	C	S	58.641	-3.292	-1.781	4.930	3.523	1.622	-4.487	86.381	18.221	18.352
14010	301	46	39	C	S	60.000	5.011	0.659	-3.360	-4.554	-0.707	3.602	86.488	18.353	18.219
14011	301	46	39	C	S	61.359	-3.292	1.781	4.930	3.523	-1.622	-4.487	86.381	18.221	18.352
14012	301	46	39	C	S	76.337	0.353	-1.239	0.954	-0.351	1.216	-0.936	86.442	17.539	19.033
14013	301	46	39	C	S	77.696	1.029	1.148	0.279	-1.008	-1.142	-0.278	86.451	19.034	17.537
14014	301	46	39	C	S	102.304	1.029	-1.148	0.279	-1.008	1.142	-0.278	86.451	19.034	17.537
14015	301	46	39	C	S	103.663	0.353	1.239	0.954	-0.351	-1.216	-0.936	86.442	17.539	19.033
14016	301	46	39	C	S	118.641	-3.292	-1.781	4.930	3.523	1.622	-4.487	86.381	18.221	18.352
14017	301	46	39	C	W	0.000	5.011	-0.659	-3.360	-4.554	0.707	3.602	86.488	18.353	18.219
14018	301	46	39	C	W	1.359	-3.292	1.781	4.930	3.523	-1.622	-4.487	86.381	18.221	18.352
14019	301	46	39	C	W	16.337	0.353	-1.239	0.954	-0.351	1.216	-0.936	86.442	17.539	19.033
14020	301	46	39	C	W	17.696	1.029	1.148	0.279	-1.008	-1.142	-0.278	86.451	19.034	17.537
14021	301	46	39	C	W	42.304	1.029	-1.148	0.279	-1.008	1.142	-0.278	86.451	19.034	17.537
14022	301	46	39	C	W	43.663	0.353	1.239	0.954	-0.351	-1.216	-0.936	86.442	17.539	19.033
14023	301	46	39	C	W	58.641	-3.292	-1.781	4.930	3.523	1.622	-4.487	86.381	18.221	18.352
14024	301	46	39	C	W	60.000	5.011	0.659	-3.360	-4.554	-0.707	3.602	86.488	18.353	18.219
14025	301	46	39	C	W	61.359	-3.292	1.781	4.930	3.523	-1.622	-4.487	86.381	18.221	18.352

BL number	Atoms	γ-PC unit cells	WS_2 unit cells	γ-PC origin atom	WS_2 origin atom	Twist-angle (°)	γ-PC strain 1 (%)	γ-PC strain 2 (%)	γ-PC strain 3 (%)	WS_2 strain 1 (%)	WS_2 strain 2 (%)	WS_2 strain 3 (%)	γ (°)	|**a**|(Å)	|**b**|(Å)
14026	301	46	39	C	W	76.337	0.353	-1.239	0.954	-0.351	1.216	-0.936	86.442	17.539	19.033
14027	301	46	39	C	W	77.696	1.029	1.148	0.279	-1.008	-1.142	-0.278	86.451	19.034	17.537
14028	301	46	39	C	W	102.304	1.029	-1.148	0.279	-1.008	1.142	-0.278	86.451	19.034	17.537
14029	301	46	39	C	W	103.663	0.353	1.239	0.954	-0.351	-1.216	-0.936	86.442	17.539	19.033
14030	301	46	39	C	W	118.641	-3.292	-1.781	4.930	3.523	1.622	-4.487	86.381	18.221	18.352
14031	301	46	39	P	S	0.000	5.011	-0.659	-3.360	-4.554	0.707	3.602	86.488	18.353	18.219
14032	301	46	39	P	S	1.359	-3.292	1.781	4.930	3.523	-1.622	-4.487	86.381	18.221	18.352
14033	301	46	39	P	S	16.337	0.353	-1.239	0.954	-0.351	1.216	-0.936	86.442	17.539	19.033
14034	301	46	39	P	S	17.696	1.029	1.148	0.279	-1.008	-1.142	-0.278	86.451	19.034	17.537
14035	301	46	39	P	S	42.304	1.029	-1.148	0.279	-1.008	1.142	-0.278	86.451	19.034	17.537
14036	301	46	39	P	S	43.663	0.353	1.239	0.954	-0.351	-1.216	-0.936	86.442	17.539	19.033
14037	301	46	39	P	S	58.641	-3.292	-1.781	4.930	3.523	1.622	-4.487	86.381	18.221	18.352
14038	301	46	39	P	S	60.000	5.011	0.659	-3.360	-4.554	-0.707	3.602	86.488	18.353	18.219
14039	301	46	39	P	S	61.359	-3.292	1.781	4.930	3.523	-1.622	-4.487	86.381	18.221	18.352
14040	301	46	39	P	S	76.337	0.353	-1.239	0.954	-0.351	1.216	-0.936	86.442	17.539	19.033
14041	301	46	39	P	S	77.696	1.029	1.148	0.279	-1.008	-1.142	-0.278	86.451	19.034	17.537
14042	301	46	39	P	S	102.304	1.029	-1.148	0.279	-1.008	1.142	-0.278	86.451	19.034	17.537
14043	301	46	39	P	S	103.663	0.353	1.239	0.954	-0.351	-1.216	-0.936	86.442	17.539	19.033
14044	301	46	39	P	S	118.641	-3.292	-1.781	4.930	3.523	1.622	-4.487	86.381	18.221	18.352
14045	301	46	39	P	W	0.000	5.011	-0.659	-3.360	-4.554	0.707	3.602	86.488	18.353	18.219
14046	301	46	39	P	W	1.359	-3.292	1.781	4.930	3.523	-1.622	-4.487	86.381	18.221	18.352
14047	301	46	39	P	W	16.337	0.353	-1.239	0.954	-0.351	1.216	-0.936	86.442	17.539	19.033
14048	301	46	39	P	W	17.696	1.029	1.148	0.279	-1.008	-1.142	-0.278	86.451	19.034	17.537
14049	301	46	39	P	W	42.304	1.029	-1.148	0.279	-1.008	1.142	-0.278	86.451	19.034	17.537
14050	301	46	39	P	W	43.663	0.353	1.239	0.954	-0.351	-1.216	-0.936	86.442	17.539	19.033
14051	301	46	39	P	W	58.641	-3.292	-1.781	4.930	3.523	1.622	-4.487	86.381	18.221	18.352
14052	301	46	39	P	W	60.000	5.011	0.659	-3.360	-4.554	-0.707	3.602	86.488	18.353	18.219
14053	301	46	39	P	W	61.359	-3.292	1.781	4.930	3.523	-1.622	-4.487	86.381	18.221	18.352
14054	301	46	39	P	W	76.337	0.353	-1.239	0.954	-0.351	1.216	-0.936	86.442	17.539	19.033
14055	301	46	39	P	W	77.696	1.029	1.148	0.279	-1.008	-1.142	-0.278	86.451	19.034	17.537
14056	301	46	39	P	W	102.304	1.029	-1.148	0.279	-1.008	1.142	-0.278	86.451	19.034	17.537
14057	301	46	39	P	W	103.663	0.353	1.239	0.954	-0.351	-1.216	-0.936	86.442	17.539	19.033
14058	301	46	39	P	W	118.641	-3.292	-1.781	4.930	3.523	1.622	-4.487	86.381	18.221	18.352
14059	302	47	38	C	S	23.691	2.390	-1.500	-3.298	-2.281	1.605	3.531	84.201	19.291	17.335
14060	302	47	38	C	S	24.791	-3.388	0.381	2.491	3.635	-0.363	-2.373	84.261	17.335	19.290
14061	302	47	38	C	S	35.209	-3.388	-0.381	2.491	3.635	0.363	-2.373	84.261	17.335	19.290
14062	302	47	38	C	S	36.309	2.390	1.500	-3.298	-2.281	-1.605	3.531	84.201	19.291	17.335
14063	302	47	38	C	S	83.691	2.390	-1.500	-3.298	-2.281	1.605	3.531	84.201	19.291	17.335
14064	302	47	38	C	S	84.791	-3.388	0.381	2.491	3.635	-0.363	-2.373	84.262	17.335	19.290
14065	302	47	38	C	S	95.209	-3.388	-0.381	2.491	3.635	0.363	-2.373	84.261	17.335	19.290
14066	302	47	38	C	S	96.309	2.390	1.500	-3.298	-2.281	-1.605	3.531	84.201	19.291	17.335
14067	302	47	38	C	W	23.691	2.390	-1.500	-3.298	-2.281	1.605	3.531	84.201	19.291	17.335
14068	302	47	38	C	W	24.791	-3.388	0.381	2.491	3.635	-0.363	-2.373	84.261	17.335	19.290
14069	302	47	38	C	W	35.209	-3.388	-0.381	2.491	3.635	0.363	-2.373	84.261	17.335	19.290
14070	302	47	38	C	W	36.309	2.390	1.500	-3.298	-2.281	-1.605	3.531	84.201	19.291	17.335
14071	302	47	38	C	W	83.691	2.390	-1.500	-3.298	-2.281	1.605	3.531	84.201	19.291	17.335
14072	302	47	38	C	W	84.791	-3.388	0.381	2.491	3.635	-0.363	-2.373	84.262	17.335	19.290
14073	302	47	38	C	W	95.209	-3.388	-0.381	2.491	3.635	0.363	-2.373	84.261	17.335	19.290
14074	302	47	38	C	W	96.309	2.390	1.500	-3.298	-2.281	-1.605	3.531	84.201	19.291	17.335
14075	302	47	38	P	S	23.691	2.390	-1.500	-3.298	-2.281	1.605	3.531	84.201	19.291	17.335
14076	302	47	38	P	S	24.791	-3.388	0.381	2.491	3.635	-0.363	-2.373	84.261	17.335	19.290

BL number	Atoms	γ-PC unit cells	WS_2 unit cells	γ-PC origin atom	WS_2 origin atom	Twist-angle (°)	γ-PC strain 1 (%)	γ-PC strain 2 (%)	γ-PC strain 3 (%)	WS_2 strain 1 (%)	WS_2 strain 2 (%)	WS_2 strain 3 (%)	γ (°)	$\lvert\mathbf{a}\rvert$(Å)	$\lvert\mathbf{b}\rvert$(Å)
14077	302	47	38	P	S	35.209	-3.388	-0.381	2.491	3.635	0.363	-2.373	84.261	17.335	19.290
14078	302	47	38	P	S	36.309	2.390	1.500	-3.298	-2.281	-1.605	3.531	84.201	19.291	17.335
14079	302	47	38	P	S	83.691	2.390	-1.500	-3.298	-2.281	1.605	3.531	84.201	19.291	17.335
14080	302	47	38	P	S	84.791	-3.388	0.381	2.491	3.635	-0.363	-2.373	84.262	17.335	19.290
14081	302	47	38	P	S	95.209	-3.388	-0.381	2.491	3.635	0.363	-2.373	84.261	17.335	19.290
14082	302	47	38	P	S	96.309	2.390	1.500	-3.298	-2.281	-1.605	3.531	84.201	19.291	17.335
14083	302	47	38	P	W	23.691	2.390	-1.500	-3.298	-2.281	1.605	3.531	84.201	19.291	17.335
14084	302	47	38	P	W	24.791	-3.388	0.381	2.491	3.635	-0.363	-2.373	84.261	17.335	19.290
14085	302	47	38	P	W	35.209	-3.388	-0.381	2.491	3.635	0.363	-2.373	84.261	17.335	19.290
14086	302	47	38	P	W	36.309	2.390	1.500	-3.298	-2.281	-1.605	3.531	84.201	19.291	17.335
14087	302	47	38	P	W	83.691	2.390	-1.500	-3.298	-2.281	1.605	3.531	84.201	19.291	17.335
14088	302	47	38	P	W	84.791	-3.388	0.381	2.491	3.635	-0.363	-2.373	84.262	17.335	19.290
14089	302	47	38	P	W	95.209	-3.388	-0.381	2.491	3.635	0.363	-2.373	84.261	17.335	19.290
14090	302	47	38	P	W	96.309	2.390	1.500	-3.298	-2.281	-1.605	3.531	84.201	19.291	17.335
14091	303	48	37	C	S	4.715	-1.702	0.000	-1.702	1.762	0.000	1.762	60.000	19.567	19.567
14092	303	48	37	C	S	55.285	-1.702	0.000	-1.702	1.762	0.000	1.762	60.000	19.567	19.567
14093	303	48	37	C	S	64.715	-1.702	0.000	-1.702	1.762	0.000	1.762	60.000	19.567	19.567
14094	303	48	37	C	S	115.285	-1.702	0.000	-1.702	1.762	0.000	1.762	60.000	19.567	19.567
14095	303	48	37	P	S	4.715	-1.702	0.000	-1.702	1.762	0.000	1.762	60.000	19.567	19.567
14096	303	48	37	P	S	55.285	-1.702	0.000	-1.702	1.762	0.000	1.762	60.000	19.567	19.567
14097	303	48	37	P	S	64.715	-1.702	0.000	-1.702	1.762	0.000	1.762	60.000	19.567	19.567
14098	303	48	37	P	S	115.285	-1.702	0.000	-1.702	1.762	0.000	1.762	60.000	19.567	19.567
14099	304	49	36	C	S	0.000	-2.848	0.000	-2.848	3.020	0.000	3.020	60.000	19.539	19.539
14100	304	49	36	C	S	21.787	-2.848	0.000	-2.848	3.020	0.000	3.020	60.000	19.539	19.539
14101	304	49	36	C	S	38.213	-2.848	0.000	-2.848	3.020	0.000	3.020	60.000	19.539	19.539
14102	304	49	36	C	S	60.000	-2.848	0.000	-2.848	3.020	0.000	3.020	60.000	19.539	19.539
14103	304	49	36	C	S	81.787	-2.848	0.000	-2.848	3.020	0.000	3.020	60.000	19.539	19.539
14104	304	49	36	C	S	98.213	-2.848	0.000	-2.848	3.020	0.000	3.020	60.000	19.539	19.539
14105	304	49	36	C	W	0.000	-2.848	0.000	-2.848	3.020	0.000	3.020	60.000	19.539	19.539
14106	304	49	36	C	W	21.787	-2.848	0.000	-2.848	3.020	0.000	3.020	60.000	19.539	19.539
14107	304	49	36	C	W	38.213	-2.848	0.000	-2.848	3.020	0.000	3.020	60.000	19.539	19.539
14108	304	49	36	C	W	60.000	-2.848	0.000	-2.848	3.020	0.000	3.020	60.000	19.539	19.539
14109	304	49	36	C	W	81.787	-2.848	0.000	-2.848	3.020	0.000	3.020	60.000	19.539	19.539
14110	304	49	36	C	W	98.213	-2.848	0.000	-2.848	3.020	0.000	3.020	60.000	19.539	19.539
14111	305	47	39	C	S	16.537	-3.292	-1.226	3.761	3.523	1.141	-3.498	84.788	18.221	18.582
14112	305	47	39	C	S	18.613	3.582	2.373	-3.135	-3.342	-2.532	3.345	84.645	18.586	18.219
14113	305	47	39	C	S	25.050	-0.955	-1.648	1.200	0.973	1.610	-1.172	84.748	17.772	19.031
14114	305	47	39	C	S	27.126	1.029	1.979	-0.791	-1.008	-2.011	0.803	84.707	19.034	17.769
14115	305	47	39	C	S	32.874	1.029	-1.979	-0.791	-1.008	2.011	0.803	84.707	19.034	17.769
14116	305	47	39	C	S	34.950	-0.955	1.648	1.200	0.973	-1.610	-1.172	84.748	17.772	19.031
14117	305	47	39	C	S	41.387	3.582	-2.373	-3.135	-3.342	2.532	3.345	84.646	18.586	18.219
14118	305	47	39	C	S	43.463	-3.292	1.226	3.761	3.523	-1.141	-3.498	84.788	18.221	18.582
14119	305	47	39	C	S	76.537	-3.292	-1.226	3.761	3.523	1.141	-3.498	84.788	18.221	18.582
14120	305	47	39	C	S	78.613	3.582	2.373	-3.135	-3.342	-2.532	3.345	84.646	18.586	18.219
14121	305	47	39	C	S	85.050	-0.955	-1.648	1.200	0.973	1.610	-1.172	84.748	17.772	19.031
14122	305	47	39	C	S	87.126	1.029	1.979	-0.791	-1.008	-2.011	0.803	84.707	19.034	17.769
14123	305	47	39	C	S	92.874	1.029	-1.979	-0.791	-1.008	2.011	0.803	84.707	19.034	17.769
14124	305	47	39	C	S	94.950	-0.955	1.648	1.200	0.973	-1.610	-1.172	84.748	17.772	19.031
14125	305	47	39	C	S	101.387	3.582	-2.373	-3.135	-3.342	2.532	3.345	84.646	18.586	18.219
14126	305	47	39	C	S	103.463	-3.292	1.226	3.761	3.523	-1.141	-3.498	84.788	18.221	18.582
14127	305	47	39	C	W	16.537	-3.292	-1.226	3.761	3.523	1.141	-3.498	84.788	18.221	18.582

BL number	Atoms	γ-PC unit cells	WS_2 unit cells	γ-PC origin atom	WS_2 origin atom	Twist-angle (°)	γ-PC strain 1 (%)	γ-PC strain 2 (%)	γ-PC strain 3 (%)	WS_2 strain 1 (%)	WS_2 strain 2 (%)	WS_2 strain 3 (%)	γ (°)	|**a**|(Å)	|**b**|(Å)
14128	305	47	39	C	W	18.613	3.582	2.373	-3.135	-3.342	-2.532	3.345	84.645	18.586	18.219
14129	305	47	39	C	W	25.050	-0.955	-1.648	1.200	0.973	1.610	-1.172	84.748	17.772	19.031
14130	305	47	39	C	W	27.126	1.029	1.979	-0.791	-1.008	-2.011	0.803	84.707	19.034	17.769
14131	305	47	39	C	W	32.874	1.029	-1.979	-0.791	-1.008	2.011	0.803	84.707	19.034	17.769
14132	305	47	39	C	W	34.950	-0.955	1.648	1.200	0.973	-1.610	-1.172	84.748	17.772	19.031
14133	305	47	39	C	W	41.387	3.582	-2.373	-3.135	-3.342	2.532	3.345	84.646	18.586	18.219
14134	305	47	39	C	W	43.463	-3.292	1.226	3.761	3.523	-1.141	-3.498	84.788	18.221	18.582
14135	305	47	39	C	W	76.537	-3.292	-1.226	3.761	3.523	1.141	-3.498	84.788	18.221	18.582
14136	305	47	39	C	W	78.613	3.582	2.373	-3.135	-3.342	-2.532	3.345	84.646	18.586	18.219
14137	305	47	39	C	W	85.050	-0.955	-1.648	1.200	0.973	1.610	-1.172	84.748	17.772	19.031
14138	305	47	39	C	W	87.126	1.029	1.979	-0.791	-1.008	-2.011	0.803	84.707	19.034	17.769
14139	305	47	39	C	W	92.874	1.029	-1.979	-0.791	-1.008	2.011	0.803	84.707	19.034	17.769
14140	305	47	39	C	W	94.950	-0.955	1.648	1.200	0.973	-1.610	-1.172	84.748	17.772	19.031
14141	305	47	39	C	W	101.387	3.582	-2.373	-3.135	-3.342	2.532	3.345	84.646	18.586	18.219
14142	305	47	39	C	W	103.463	-3.292	1.226	3.761	3.523	-1.141	-3.498	84.788	18.221	18.582
14143	305	47	39	P	S	16.537	-3.292	-1.226	3.761	3.523	1.141	-3.498	84.788	18.221	18.582
14144	305	47	39	P	S	18.613	3.582	2.373	-3.135	-3.342	-2.532	3.345	84.645	18.586	18.219
14145	305	47	39	P	S	25.050	-0.955	-1.648	1.200	0.973	1.610	-1.172	84.748	17.772	19.031
14146	305	47	39	P	S	27.126	1.029	1.979	-0.791	-1.008	-2.011	0.803	84.707	19.034	17.769
14147	305	47	39	P	S	32.874	1.029	-1.979	-0.791	-1.008	2.011	0.803	84.707	19.034	17.769
14148	305	47	39	P	S	34.950	-0.955	1.648	1.200	0.973	-1.610	-1.172	84.748	17.772	19.031
14149	305	47	39	P	S	41.387	3.582	-2.373	-3.135	-3.342	2.532	3.345	84.646	18.586	18.219
14150	305	47	39	P	S	43.463	-3.292	1.226	3.761	3.523	-1.141	-3.498	84.788	18.221	18.582
14151	305	47	39	P	S	76.537	-3.292	-1.226	3.761	3.523	1.141	-3.498	84.788	18.221	18.582
14152	305	47	39	P	S	78.613	3.582	2.373	-3.135	-3.342	-2.532	3.345	84.646	18.586	18.219
14153	305	47	39	P	S	85.050	-0.955	-1.648	1.200	0.973	1.610	-1.172	84.748	17.772	19.031
14154	305	47	39	P	S	87.126	1.029	1.979	-0.791	-1.008	-2.011	0.803	84.707	19.034	17.769
14155	305	47	39	P	S	92.874	1.029	-1.979	-0.791	-1.008	2.011	0.803	84.707	19.034	17.769
14156	305	47	39	P	S	94.950	-0.955	1.648	1.200	0.973	-1.610	-1.172	84.748	17.772	19.031
14157	305	47	39	P	S	101.387	3.582	-2.373	-3.135	-3.342	2.532	3.345	84.646	18.586	18.219
14158	305	47	39	P	S	103.463	-3.292	1.226	3.761	3.523	-1.141	-3.498	84.788	18.221	18.582
14159	305	47	39	P	W	16.537	-3.292	-1.226	3.761	3.523	1.141	-3.498	84.788	18.221	18.582
14160	305	47	39	P	W	18.613	3.582	2.373	-3.135	-3.342	-2.532	3.345	84.645	18.586	18.219
14161	305	47	39	P	W	25.050	-0.955	-1.648	1.200	0.973	1.610	-1.172	84.748	17.772	19.031
14162	305	47	39	P	W	27.126	1.029	1.979	-0.791	-1.008	-2.011	0.803	84.707	19.034	17.769
14163	305	47	39	P	W	32.874	1.029	-1.979	-0.791	-1.008	2.011	0.803	84.707	19.034	17.769
14164	305	47	39	P	W	34.950	-0.955	1.648	1.200	0.973	-1.610	-1.172	84.748	17.772	19.031
14165	305	47	39	P	W	41.387	3.582	-2.373	-3.135	-3.342	2.532	3.345	84.646	18.586	18.219
14166	305	47	39	P	W	43.463	-3.292	1.226	3.761	3.523	-1.141	-3.498	84.788	18.221	18.582
14167	305	47	39	P	W	76.537	-3.292	-1.226	3.761	3.523	1.141	-3.498	84.788	18.221	18.582
14168	305	47	39	P	W	78.613	3.582	2.373	-3.135	-3.342	-2.532	3.345	84.646	18.586	18.219
14169	305	47	39	P	W	85.050	-0.955	-1.648	1.200	0.973	1.610	-1.172	84.748	17.772	19.031
14170	305	47	39	P	W	87.126	1.029	1.979	-0.791	-1.008	-2.011	0.803	84.707	19.034	17.769
14171	305	47	39	P	W	92.874	1.029	-1.979	-0.791	-1.008	2.011	0.803	84.707	19.034	17.769
14172	305	47	39	P	W	94.950	-0.955	1.648	1.200	0.973	-1.610	-1.172	84.748	17.772	19.031
14173	305	47	39	P	W	101.387	3.582	-2.373	-3.135	-3.342	2.532	3.345	84.646	18.586	18.219
14174	305	47	39	P	W	103.463	-3.292	1.226	3.761	3.523	-1.141	-3.498	84.788	18.221	18.582
14175	306	45	42	C	S	16.102	2.853	-2.309	3.440	-2.699	2.161	-3.219	86.532	18.455	18.581
14176	306	45	42	C	S	18.613	3.582	2.209	2.713	-3.342	-2.095	-2.573	86.548	18.586	18.450
14177	306	45	42	C	S	23.691	2.390	-1.869	3.912	-2.281	1.734	-3.628	76.889	19.291	18.218
14178	306	45	42	C	S	25.285	4.262	1.003	2.052	-3.928	-0.964	-1.971	76.902	18.222	19.287

BL number	Atoms	γ-PC unit cells	WS_2 unit cells	γ-PC origin atom	WS_2 origin atom	Twist-angle (°)	γ-PC strain 1 (%)	γ-PC strain 2 (%)	γ-PC strain 3 (%)	WS_2 strain 1 (%)	WS_2 strain 2 (%)	WS_2 strain 3 (%)	γ (°)	|**a**|(Å)	|**b**|(Å)
14179	306	45	42	C	S	34.715	4.262	-1.003	2.052	-3.928	0.964	-1.971	76.902	18.222	19.287
14180	306	45	42	C	S	36.309	2.390	1.869	3.912	-2.281	-1.734	-3.628	76.889	19.291	18.218
14181	306	45	42	C	S	41.387	3.582	-2.209	2.713	-3.342	2.095	-2.573	86.548	18.586	18.450
14182	306	45	42	C	S	43.898	2.853	2.309	3.440	-2.699	-2.161	-3.219	86.532	18.455	18.581
14183	306	45	42	C	S	76.102	2.853	-2.309	3.440	-2.699	2.161	-3.219	86.532	18.455	18.581
14184	306	45	42	C	S	78.613	3.582	2.209	2.713	-3.342	-2.095	-2.573	86.548	18.586	18.450
14185	306	45	42	C	S	85.285	4.262	1.003	2.052	-3.928	-0.964	-1.971	76.902	18.222	19.287
14186	306	45	42	C	S	94.715	4.262	-1.003	2.052	-3.928	0.964	-1.971	76.902	18.222	19.287
14187	306	45	42	C	S	96.309	2.390	1.869	3.912	-2.281	-1.734	-3.628	76.889	19.291	18.218
14188	306	45	42	C	S	101.387	3.582	-2.209	2.713	-3.342	2.095	-2.573	86.548	18.586	18.450
14189	306	45	42	C	S	103.898	2.853	2.309	3.440	-2.699	-2.161	-3.219	86.532	18.455	18.581
14190	306	45	42	C	W	16.102	2.853	-2.309	3.440	-2.699	2.161	-3.219	86.532	18.455	18.581
14191	306	45	42	C	W	18.613	3.582	2.209	2.713	-3.342	-2.095	-2.573	86.548	18.586	18.450
14192	306	45	42	C	W	41.387	3.582	-2.209	2.713	-3.342	2.095	-2.573	86.548	18.586	18.450
14193	306	45	42	C	W	43.898	2.853	2.309	3.440	-2.699	-2.161	-3.219	86.532	18.455	18.581
14194	306	45	42	C	W	76.102	2.853	-2.309	3.440	-2.699	2.161	-3.219	86.532	18.455	18.581
14195	306	45	42	C	W	78.613	3.582	2.209	2.713	-3.342	-2.095	-2.573	86.548	18.586	18.450
14196	306	45	42	C	W	83.691	2.390	-1.869	3.912	-2.281	1.734	-3.628	76.889	19.291	18.218
14197	306	45	42	C	W	101.387	3.582	-2.209	2.713	-3.342	2.095	-2.573	86.548	18.586	18.450
14198	306	45	42	C	W	103.898	2.853	2.309	3.440	-2.699	-2.161	-3.219	86.532	18.455	18.581
14199	306	45	42	P	S	23.691	2.390	-1.869	3.912	-2.281	1.734	-3.628	76.889	19.291	18.218
14200	306	45	42	P	S	25.285	4.262	1.003	2.052	-3.928	-0.964	-1.971	76.902	18.222	19.287
14201	306	45	42	P	S	34.715	4.262	-1.003	2.052	-3.928	0.964	-1.971	76.902	18.222	19.287
14202	306	45	42	P	S	36.309	2.390	1.869	3.912	-2.281	-1.734	-3.628	76.889	19.291	18.218
14203	306	45	42	P	S	76.102	2.853	-2.309	3.440	-2.699	2.161	-3.219	86.532	18.455	18.581
14204	306	45	42	P	S	85.285	4.262	1.003	2.052	-3.928	-0.964	-1.971	76.902	18.222	19.287
14205	306	45	42	P	S	94.715	4.262	-1.003	2.052	-3.928	0.964	-1.971	76.902	18.222	19.287
14206	306	45	42	P	S	96.309	2.390	1.869	3.912	-2.281	-1.734	-3.628	76.889	19.291	18.218
14207	306	45	42	P	W	83.691	2.390	-1.869	3.912	-2.281	1.734	-3.628	76.889	19.291	18.218
14208	306	45	42	P	W	85.285	4.262	1.003	2.052	-3.928	-0.964	-1.971	76.902	18.222	19.287
14209	306	48	38	C	S	6.587	-3.853	0.000	1.915	4.175	0.000	-1.845	90.000	14.354	23.426
14210	306	48	38	C	S	53.413	-3.853	0.000	1.915	4.175	0.000	-1.845	90.000	14.354	23.426
14211	306	48	38	C	S	66.587	-3.853	0.000	1.915	4.175	0.000	-1.845	90.000	14.354	23.426
14212	306	48	38	C	S	113.413	-3.853	0.000	1.915	4.175	0.000	-1.845	90.000	14.354	23.426
14213	306	48	38	C	W	6.587	-3.853	0.000	1.915	4.175	0.000	-1.845	90.000	14.354	23.426
14214	306	48	38	C	W	53.413	-3.853	0.000	1.915	4.175	0.000	-1.845	90.000	14.354	23.426
14215	306	48	38	C	W	66.587	-3.853	0.000	1.915	4.175	0.000	-1.845	90.000	14.354	23.426
14216	306	48	38	C	W	113.413	1.915	0.000	-3.853	-1.845	0.000	4.175	90.000	23.426	14.354
14217	306	48	38	P	S	6.587	-3.853	0.000	1.915	4.175	0.000	-1.845	90.000	14.354	23.426
14218	306	48	38	P	S	53.413	-3.853	0.000	1.915	4.175	0.000	-1.845	90.000	14.354	23.426
14219	306	48	38	P	S	66.587	-3.853	0.000	1.915	4.175	0.000	-1.845	90.000	14.354	23.426
14220	306	48	38	P	S	113.413	1.915	0.000	-3.853	-1.845	0.000	4.175	90.000	23.426	14.354
14221	306	48	38	P	W	6.587	-3.853	0.000	1.915	4.175	0.000	-1.845	90.000	14.354	23.426
14222	306	48	38	P	W	53.413	-3.853	0.000	1.915	4.175	0.000	-1.845	90.000	14.354	23.426
14223	306	48	38	P	W	66.587	-3.853	0.000	1.915	4.175	0.000	-1.845	90.000	14.354	23.426
14224	306	48	38	P	W	113.413	-3.853	0.000	1.915	4.175	0.000	-1.845	90.000	14.354	23.426
14225	306	48	38	C	S	4.715	-1.702	-1.917	-0.397	1.762	1.933	0.400	75.123	19.567	17.766
14226	306	48	38	C	S	7.154	-0.955	2.306	-1.152	0.973	-2.360	1.180	75.108	17.772	19.562
14227	306	48	38	C	S	52.846	-0.955	-2.306	-1.152	0.973	2.360	1.180	75.108	17.772	19.562
14228	306	48	38	C	S	55.285	-1.702	1.917	-0.397	1.762	-1.933	0.400	75.123	19.567	17.766
14229	306	48	38	C	S	64.715	-1.702	-1.917	-0.397	1.762	1.933	0.400	75.123	19.567	17.766

BL number	Atoms	γ-PC unit cells	WS_2 unit cells	γ-PC origin atom	WS_2 origin atom	Twist-angle (°)	γ-PC strain 1 (%)	γ-PC strain 2 (%)	γ-PC strain 3 (%)	WS_2 strain 1 (%)	WS_2 strain 2 (%)	WS_2 strain 3 (%)	γ (°)	|**a**|(Å)	|**b**|(Å)
14230	306	48	38	C	S	67.154	-0.955	2.306	-1.152	0.973	-2.360	1.180	75.108	17.772	19.562
14231	306	48	38	C	S	112.846	-0.955	-2.306	-1.152	0.973	2.360	1.180	75.108	17.772	19.562
14232	306	48	38	C	S	115.285	-1.702	1.917	-0.397	1.762	-1.933	0.400	75.123	19.567	17.766
14233	306	48	38	C	W	4.715	-1.702	-1.917	-0.397	1.762	1.933	0.400	75.123	19.567	17.766
14234	306	48	38	C	W	7.154	-0.955	2.306	-1.152	0.973	-2.360	1.180	75.108	17.772	19.562
14235	306	48	38	C	W	52.846	-0.955	-2.306	-1.152	0.973	2.360	1.180	75.108	17.772	19.562
14236	306	48	38	C	W	55.285	-1.702	1.917	-0.397	1.762	-1.933	0.400	75.123	19.567	17.766
14237	306	48	38	C	W	64.715	-1.702	-1.917	-0.397	1.762	1.933	0.400	75.123	19.567	17.766
14238	306	48	38	C	W	67.154	-0.955	2.306	-1.152	0.973	-2.360	1.180	75.108	17.772	19.562
14239	306	48	38	C	W	112.846	-0.955	-2.306	-1.152	0.973	2.360	1.180	75.108	17.772	19.562
14240	306	48	38	C	W	115.285	-1.702	1.917	-0.397	1.762	-1.933	0.400	75.123	19.567	17.766
14241	306	48	38	P	S	52.846	-0.955	-2.306	-1.152	0.973	2.360	1.180	75.108	17.772	19.562
14242	306	48	38	P	S	55.285	-1.702	1.917	-0.397	1.762	-1.933	0.400	75.123	19.567	17.766
14243	306	48	38	P	S	64.715	-1.702	-1.917	-0.397	1.762	1.933	0.400	75.123	19.567	17.766
14244	306	48	38	P	S	67.154	-0.955	2.306	-1.152	0.973	-2.360	1.180	75.108	17.772	19.562
14245	306	48	38	P	W	4.715	-1.702	-1.917	-0.397	1.762	1.933	0.400	75.123	19.567	17.766
14246	306	48	38	P	W	7.154	-0.955	2.306	-1.152	0.973	-2.360	1.180	75.108	17.772	19.562
14247	306	48	38	P	W	112.846	-0.955	-2.306	-1.152	0.973	2.360	1.180	75.108	17.772	19.562
14248	306	48	38	P	W	115.285	-1.702	1.917	-0.397	1.762	-1.933	0.400	75.123	19.567	17.766
14249	307	49	37	C	S	3.498	-2.197	0.000	-2.197	2.298	0.000	2.298	60.000	19.670	19.670
14250	307	49	37	C	S	12.928	-2.197	0.000	-2.197	2.298	0.000	2.298	60.000	19.670	19.670
14251	307	49	37	C	S	25.285	-2.197	0.000	-2.197	2.298	0.000	2.298	60.000	19.670	19.670
14252	307	49	37	C	S	34.715	-2.197	0.000	-2.197	2.298	0.000	2.298	60.000	19.670	19.670
14253	307	49	37	C	S	47.072	-2.197	0.000	-2.197	2.298	0.000	2.298	60.000	19.670	19.670
14254	307	49	37	C	S	56.502	-2.197	0.000	-2.197	2.298	0.000	2.298	60.000	19.670	19.670
14255	307	49	37	C	S	63.498	-2.197	0.000	-2.197	2.298	0.000	2.298	60.000	19.670	19.670
14256	307	49	37	C	S	72.928	-2.197	0.000	-2.197	2.298	0.000	2.298	60.000	19.670	19.670
14257	307	49	37	C	S	85.285	-2.197	0.000	-2.197	2.298	0.000	2.298	60.000	19.670	19.670
14258	307	49	37	C	S	94.715	-2.197	0.000	-2.197	2.298	0.000	2.298	60.000	19.670	19.670
14259	307	49	37	C	S	107.072	-2.197	0.000	-2.197	2.298	0.000	2.298	60.000	19.670	19.670
14260	307	49	37	C	S	116.502	-2.197	0.000	-2.197	2.298	0.000	2.298	60.000	19.670	19.670
14261	307	49	37	C	W	3.498	-2.197	0.000	-2.197	2.298	0.000	2.298	60.000	19.670	19.670
14262	307	49	37	C	W	12.928	-2.197	0.000	-2.197	2.298	0.000	2.298	60.000	19.670	19.670
14263	307	49	37	C	W	25.285	-2.197	0.000	-2.197	2.298	0.000	2.298	60.000	19.670	19.670
14264	307	49	37	C	W	34.715	-2.197	0.000	-2.197	2.298	0.000	2.298	60.000	19.670	19.670
14265	307	49	37	C	W	47.072	-2.197	0.000	-2.197	2.298	0.000	2.298	60.000	19.670	19.670
14266	307	49	37	C	W	56.502	-2.197	0.000	-2.197	2.298	0.000	2.298	60.000	19.670	19.670
14267	307	49	37	C	W	63.498	-2.197	0.000	-2.197	2.298	0.000	2.298	60.000	19.670	19.670
14268	307	49	37	C	W	72.928	-2.197	0.000	-2.197	2.298	0.000	2.298	60.000	19.670	19.670
14269	307	49	37	C	W	85.285	-2.197	0.000	-2.197	2.298	0.000	2.298	60.000	19.670	19.670
14270	307	49	37	C	W	94.715	-2.197	0.000	-2.197	2.298	0.000	2.298	60.000	19.670	19.670
14271	307	49	37	C	W	107.072	-2.197	0.000	-2.197	2.298	0.000	2.298	60.000	19.670	19.670
14272	307	49	37	C	W	116.502	-2.197	0.000	-2.197	2.298	0.000	2.298	60.000	19.670	19.670
14273	307	49	37	P	S	12.928	-2.197	0.000	-2.197	2.298	0.000	2.298	60.000	19.670	19.670
14274	307	49	37	P	S	25.285	-2.197	0.000	-2.197	2.298	0.000	2.298	60.000	19.670	19.670
14275	307	49	37	P	S	56.502	-2.197	0.000	-2.197	2.298	0.000	2.298	60.000	19.670	19.670
14276	307	49	37	P	S	63.498	-2.197	0.000	-2.197	2.298	0.000	2.298	60.000	19.670	19.670
14277	307	49	37	P	S	94.715	-2.197	0.000	-2.197	2.298	0.000	2.298	60.000	19.670	19.670
14278	307	49	37	P	S	107.072	-2.197	0.000	-2.197	2.298	0.000	2.298	60.000	19.670	19.670
14279	307	49	37	P	W	3.498	-2.197	0.000	-2.197	2.298	0.000	2.298	60.000	19.670	19.670
14280	307	49	37	P	W	34.715	-2.197	0.000	-2.197	2.298	0.000	2.298	60.000	19.670	19.670

BL number	Atoms	γ-PC unit cells	WS_2 unit cells	γ-PC origin atom	WS_2 origin atom	Twist-angle (°)	γ-PC strain 1 (%)	γ-PC strain 2 (%)	γ-PC strain 3 (%)	WS_2 strain 1 (%)	WS_2 strain 2 (%)	WS_2 strain 3 (%)	γ (°)	|**a**|(Å)	|**b**|(Å)
14281	307	49	37	P	W	47.072	-2.197	0.000	-2.197	2.298	0.000	2.298	60.000	19.670	19.670
14282	307	49	37	P	W	72.928	-2.197	0.000	-2.197	2.298	0.000	2.298	60.000	19.670	19.670
14283	307	49	37	P	W	85.285	-2.197	0.000	-2.197	2.298	0.000	2.298	60.000	19.670	19.670
14284	307	49	37	P	W	116.502	-2.197	0.000	-2.197	2.298	0.000	2.298	60.000	19.670	19.670
14285	308	47	40	C	S	0.000	5.011	-1.935	-3.182	-4.554	2.066	3.399	84.294	18.842	18.221
14286	308	47	40	C	S	1.359	-3.292	0.402	5.140	3.523	-0.365	-4.661	84.411	18.221	18.841
14287	308	47	40	C	S	7.154	-0.955	-0.883	2.513	0.973	0.841	-2.392	84.391	17.772	19.289
14288	308	47	40	C	S	8.513	2.390	1.500	-0.840	-2.281	-1.525	0.854	84.344	19.291	17.771
14289	308	47	40	C	S	51.487	2.390	-1.499	-0.840	-2.281	1.525	0.854	84.344	19.291	17.771
14290	308	47	40	C	S	52.846	-0.955	0.883	2.513	0.973	-0.841	-2.392	84.391	17.772	19.289
14291	308	47	40	C	S	58.641	-3.292	-0.402	5.140	3.523	0.365	-4.661	84.411	18.221	18.841
14292	308	47	40	C	S	60.000	5.011	1.935	-3.182	-4.554	-2.066	3.398	84.294	18.842	18.221
14293	308	47	40	C	S	61.359	-3.292	0.402	5.140	3.523	-0.365	-4.661	84.411	18.221	18.841
14294	308	47	40	C	S	67.154	-0.955	-0.883	2.513	0.973	0.841	-2.392	84.391	17.772	19.289
14295	308	47	40	C	S	68.513	2.390	1.500	-0.840	-2.281	-1.525	0.854	84.344	19.291	17.771
14296	308	47	40	C	S	111.487	2.390	-1.499	-0.840	-2.281	1.525	0.854	84.344	19.291	17.771
14297	308	47	40	C	S	112.846	-0.955	0.883	2.513	0.973	-0.841	-2.392	84.391	17.772	19.289
14298	308	47	40	C	S	118.641	-3.292	-0.402	5.140	3.523	0.365	-4.661	84.411	18.221	18.841
14299	308	47	40	C	W	0.000	5.011	-1.935	-3.182	-4.554	2.066	3.399	84.294	18.842	18.221
14300	308	47	40	C	W	1.359	-3.292	0.402	5.140	3.523	-0.365	-4.661	84.411	18.221	18.841
14301	308	47	40	C	W	7.154	-0.955	-0.883	2.513	0.973	0.841	-2.392	84.391	17.772	19.289
14302	308	47	40	C	W	8.513	2.390	1.500	-0.840	-2.281	-1.525	0.854	84.344	19.291	17.771
14303	308	47	40	C	W	51.487	2.390	-1.499	-0.840	-2.281	1.525	0.854	84.344	19.291	17.771
14304	308	47	40	C	W	52.846	-0.955	0.883	2.513	0.973	-0.841	-2.392	84.391	17.772	19.289
14305	308	47	40	C	W	58.641	-3.292	-0.402	5.140	3.523	0.365	-4.661	84.411	18.221	18.841
14306	308	47	40	C	W	60.000	5.011	1.935	-3.182	-4.554	-2.066	3.398	84.294	18.842	18.221
14307	308	47	40	C	W	61.359	-3.292	0.402	5.140	3.523	-0.365	-4.661	84.411	18.221	18.841
14308	308	47	40	C	W	67.154	-0.955	-0.883	2.513	0.973	0.841	-2.392	84.391	17.772	19.289
14309	308	47	40	C	W	68.513	2.390	1.500	-0.840	-2.281	-1.525	0.854	84.344	19.291	17.771
14310	308	47	40	C	W	111.487	2.390	-1.499	-0.840	-2.281	1.525	0.854	84.344	19.291	17.771
14311	308	47	40	C	W	112.846	-0.955	0.883	2.513	0.973	-0.841	-2.392	84.391	17.772	19.289
14312	308	47	40	C	W	118.641	-3.292	-0.402	5.140	3.523	0.365	-4.661	84.411	18.221	18.841
14313	308	47	40	P	S	0.000	5.011	-1.935	-3.182	-4.554	2.066	3.399	84.294	18.842	18.221
14314	308	47	40	P	S	1.359	-3.292	0.402	5.140	3.523	-0.365	-4.661	84.411	18.221	18.841
14315	308	47	40	P	S	7.154	-0.955	-0.883	2.513	0.973	0.841	-2.392	84.391	17.772	19.289
14316	308	47	40	P	S	8.513	2.390	1.500	-0.840	-2.281	-1.525	0.854	84.344	19.291	17.771
14317	308	47	40	P	S	51.487	2.390	-1.499	-0.840	-2.281	1.525	0.854	84.344	19.291	17.771
14318	308	47	40	P	S	52.846	-0.955	0.883	2.513	0.973	-0.841	-2.392	84.391	17.772	19.289
14319	308	47	40	P	S	67.154	-0.955	-0.883	2.513	0.973	0.841	-2.392	84.391	17.772	19.289
14320	308	47	40	P	S	68.513	2.390	1.500	-0.840	-2.281	-1.525	0.854	84.344	19.291	17.771
14321	308	47	40	P	S	111.487	2.390	-1.499	-0.840	-2.281	1.525	0.854	84.344	19.291	17.771
14322	308	47	40	P	S	112.846	-0.955	0.883	2.513	0.973	-0.841	-2.392	84.391	17.772	19.289
14323	308	47	40	P	S	118.641	-3.292	-0.402	5.140	3.523	0.365	-4.661	84.411	18.221	18.841
14324	308	47	40	P	W	7.154	-0.955	-0.883	2.513	0.973	0.841	-2.392	84.391	17.772	19.289
14325	308	47	40	P	W	8.513	2.390	1.500	-0.840	-2.281	-1.525	0.854	84.344	19.291	17.771
14326	308	47	40	P	W	51.487	2.390	-1.499	-0.840	-2.281	1.525	0.854	84.344	19.291	17.771
14327	308	47	40	P	W	52.846	-0.955	0.883	2.513	0.973	-0.841	-2.392	84.391	17.772	19.289
14328	308	47	40	P	W	58.641	-3.292	-0.402	5.140	3.523	0.365	-4.661	84.411	18.221	18.841
14329	308	47	40	P	W	60.000	5.011	1.935	-3.182	-4.554	-2.066	3.398	84.294	18.842	18.221
14330	308	47	40	P	W	61.359	-3.292	0.402	5.140	3.523	-0.365	-4.661	84.411	18.221	18.841
14331	308	47	40	P	W	67.154	-0.955	-0.883	2.513	0.973	0.841	-2.392	84.391	17.772	19.289

BL number	Atoms	γ-PC unit cells	WS_2 unit cells	γ-PC origin atom	WS_2 origin atom	Twist-angle (°)	γ-PC strain 1 (%)	γ-PC strain 2 (%)	γ-PC strain 3 (%)	WS_2 strain 1 (%)	WS_2 strain 2 (%)	WS_2 strain 3 (%)	γ (°)	$\lvert\mathbf{a}\rvert$(Å)	$\lvert\mathbf{b}\rvert$(Å)
14332	308	47	40	P	W	68.513	2.390	1.500	-0.840	-2.281	-1.525	0.854	84.344	19.291	17.771
14333	308	47	40	P	W	111.487	2.390	-1.499	-0.840	-2.281	1.525	0.854	84.344	19.291	17.771
14334	308	47	40	P	W	112.846	-0.955	0.883	2.513	0.973	-0.841	-2.392	84.391	17.772	19.289
14335	309	45	43	C	S	8.513	2.390	-0.303	5.196	-2.281	0.275	-4.707	78.173	19.291	18.352
14336	309	45	43	C	S	9.430	5.011	1.347	2.566	-4.554	-1.282	-2.441	78.131	18.353	19.293
14337	309	45	43	C	S	50.570	5.011	-1.347	2.566	-4.554	1.281	-2.441	78.131	18.353	19.293
14338	309	45	43	C	S	51.487	2.390	0.303	5.196	-2.281	-0.275	-4.707	78.173	19.291	18.352
14339	309	45	43	C	S	68.513	2.390	-0.303	5.196	-2.281	0.275	-4.707	78.173	19.291	18.352
14340	309	45	43	C	S	69.430	5.011	1.347	2.566	-4.554	-1.282	-2.441	78.131	18.353	19.293
14341	309	45	43	C	S	110.570	5.011	-1.347	2.566	-4.554	1.281	-2.441	78.131	18.353	19.293
14342	309	45	43	C	S	111.487	2.390	0.303	5.196	-2.281	-0.275	-4.707	78.173	19.291	18.352
14343	309	45	43	C	W	8.513	2.390	-0.303	5.196	-2.281	0.275	-4.707	78.173	19.291	18.352
14344	309	45	43	C	W	9.430	5.011	1.347	2.566	-4.554	-1.282	-2.441	78.131	18.353	19.293
14345	309	45	43	C	W	50.570	5.011	-1.347	2.566	-4.554	1.281	-2.441	78.131	18.353	19.293
14346	309	45	43	C	W	51.487	2.390	0.303	5.196	-2.281	-0.275	-4.707	78.173	19.291	18.352
14347	309	45	43	C	W	68.513	2.390	-0.303	5.196	-2.281	0.275	-4.707	78.173	19.291	18.352
14348	309	45	43	C	W	69.430	5.011	1.347	2.566	-4.554	-1.282	-2.441	78.131	18.353	19.293
14349	309	45	43	C	W	110.570	5.011	-1.347	2.566	-4.554	1.281	-2.441	78.131	18.353	19.293
14350	309	45	43	C	W	111.487	2.390	0.303	5.196	-2.281	-0.275	-4.707	78.173	19.291	18.352
14351	309	45	43	P	S	8.513	2.390	-0.303	5.196	-2.281	0.275	-4.707	78.173	19.291	18.352
14352	309	45	43	P	S	9.430	5.011	1.347	2.566	-4.554	-1.282	-2.441	78.131	18.353	19.293
14353	309	45	43	P	S	50.570	5.011	-1.347	2.566	-4.554	1.281	-2.441	78.131	18.353	19.293
14354	309	45	43	P	S	51.487	2.390	0.303	5.196	-2.281	-0.275	-4.707	78.173	19.291	18.352
14355	309	45	43	P	S	68.513	2.390	-0.303	5.196	-2.281	0.275	-4.707	78.173	19.291	18.352
14356	309	45	43	P	S	69.430	5.011	1.347	2.566	-4.554	-1.282	-2.441	78.131	18.353	19.293
14357	309	45	43	P	S	110.570	5.011	-1.347	2.566	-4.554	1.281	-2.441	78.131	18.353	19.293
14358	309	45	43	P	S	111.487	2.390	0.303	5.196	-2.281	-0.275	-4.707	78.173	19.291	18.352
14359	309	45	43	P	W	8.513	2.390	-0.303	5.196	-2.281	0.275	-4.707	78.173	19.291	18.352
14360	309	45	43	P	W	9.430	5.011	1.347	2.566	-4.554	-1.282	-2.441	78.131	18.353	19.293
14361	309	45	43	P	W	50.570	5.011	-1.347	2.566	-4.554	1.281	-2.441	78.131	18.353	19.293
14362	309	45	43	P	W	51.487	2.390	0.303	5.196	-2.281	-0.275	-4.707	78.173	19.291	18.352
14363	309	45	43	P	W	68.513	2.390	-0.303	5.196	-2.281	0.275	-4.707	78.173	19.291	18.352
14364	309	45	43	P	W	69.430	5.011	1.347	2.566	-4.554	-1.282	-2.441	78.131	18.353	19.293
14365	309	45	43	P	W	110.570	5.011	-1.347	2.566	-4.554	1.281	-2.441	78.131	18.353	19.293
14366	309	45	43	P	W	111.487	2.390	0.303	5.196	-2.281	-0.275	-4.707	78.173	19.291	18.352
14367	309	48	39	C	S	4.715	-1.702	-3.835	0.909	1.762	3.766	-0.893	87.824	19.567	17.408
14368	309	48	39	C	S	8.948	1.048	3.529	-1.834	-1.026	-3.663	1.903	87.941	17.420	19.554
14369	309	48	39	C	S	51.052	1.048	-3.528	-1.834	-1.026	3.663	1.904	87.941	17.420	19.554
14370	309	48	39	C	S	55.285	-1.702	3.835	0.909	1.762	-3.766	-0.893	87.824	19.567	17.408
14371	309	48	39	C	S	64.715	-1.702	-3.835	0.909	1.762	3.766	-0.893	87.824	19.567	17.408
14372	309	48	39	C	S	68.948	1.048	3.529	-1.834	-1.026	-3.663	1.903	87.941	17.420	19.554
14373	309	48	39	C	S	111.052	1.048	-3.528	-1.834	-1.026	3.663	1.904	87.941	17.420	19.554
14374	309	48	39	C	S	115.285	-1.702	3.835	0.909	1.762	-3.766	-0.893	87.824	19.567	17.408
14375	309	48	39	C	W	4.715	-1.702	-3.835	0.909	1.762	3.766	-0.893	87.824	19.567	17.408
14376	309	48	39	C	W	8.948	1.048	3.529	-1.834	-1.026	-3.663	1.903	87.941	17.420	19.554
14377	309	48	39	C	W	51.052	1.048	-3.528	-1.834	-1.026	3.663	1.904	87.941	17.420	19.554
14378	309	48	39	C	W	55.285	-1.702	3.835	0.909	1.762	-3.766	-0.893	87.824	19.567	17.408
14379	309	48	39	C	W	64.715	-1.702	-3.835	0.909	1.762	3.766	-0.893	87.824	19.567	17.408
14380	309	48	39	C	W	68.948	1.048	3.529	-1.834	-1.026	-3.663	1.903	87.941	17.420	19.554
14381	309	48	39	C	W	111.052	1.048	-3.528	-1.834	-1.026	3.663	1.904	87.941	17.420	19.554
14382	309	48	39	C	W	115.285	-1.702	3.835	0.909	1.762	-3.766	-0.893	87.824	19.567	17.408

BL number	Atoms	γ-PC unit cells	WS_2 unit cells	γ-PC origin atom	WS_2 origin atom	Twist-angle (°)	γ-PC strain 1 (%)	γ-PC strain 2 (%)	γ-PC strain 3 (%)	WS_2 strain 1 (%)	WS_2 strain 2 (%)	WS_2 strain 3 (%)	γ (°)	|**a**|(Å)	|**b**|(Å)
14383	310	46	42	C	S	7.589	0.334	-1.994	4.894	-0.332	1.816	-4.458	86.165	18.904	18.350
14384	310	46	42	C	S	9.430	5.011	1.318	0.227	-4.554	-1.312	-0.226	86.238	18.353	18.900
14385	310	46	42	C	S	25.285	4.262	-1.418	0.920	-3.928	1.392	-0.904	86.229	18.222	19.031
14386	310	46	42	C	S	27.126	1.029	1.885	4.147	-1.008	-1.741	-3.829	86.178	19.034	18.219
14387	310	46	42	C	S	32.874	1.029	-1.885	4.147	-1.008	1.740	-3.829	86.178	19.034	18.219
14388	310	46	42	C	S	34.715	4.262	1.418	0.920	-3.928	-1.392	-0.904	86.229	18.222	19.031
14389	310	46	42	C	S	50.570	5.011	-1.318	0.227	-4.554	1.312	-0.226	86.238	18.353	18.900
14390	310	46	42	C	S	52.411	0.334	1.994	4.894	-0.332	-1.816	-4.458	86.165	18.904	18.350
14391	310	46	42	C	S	67.589	0.334	-1.994	4.894	-0.332	1.816	-4.458	86.165	18.904	18.350
14392	310	46	42	C	S	69.430	5.011	1.318	0.227	-4.554	-1.312	-0.226	86.238	18.353	18.901
14393	310	46	42	C	S	85.285	4.262	-1.418	0.920	-3.928	1.392	-0.904	86.229	18.222	19.031
14394	310	46	42	C	S	87.126	1.029	1.885	4.147	-1.008	-1.741	-3.829	86.178	19.034	18.219
14395	310	46	42	C	S	92.874	1.029	-1.885	4.147	-1.008	1.740	-3.829	86.178	19.034	18.219
14396	310	46	42	C	S	94.715	4.262	1.418	0.920	-3.928	-1.392	-0.904	86.229	18.222	19.031
14397	310	46	42	C	S	110.570	5.011	-1.318	0.227	-4.554	1.312	-0.226	86.238	18.353	18.900
14398	310	46	42	C	S	112.411	0.334	1.994	4.894	-0.332	-1.816	-4.458	86.165	18.904	18.350
14399	310	46	42	C	W	7.589	0.334	-1.994	4.894	-0.332	1.816	-4.458	86.165	18.904	18.350
14400	310	46	42	C	W	9.430	5.011	1.318	0.227	-4.554	-1.312	-0.226	86.238	18.353	18.900
14401	310	46	42	C	W	25.285	4.262	-1.418	0.920	-3.928	1.392	-0.904	86.229	18.222	19.031
14402	310	46	42	C	W	27.126	1.029	1.885	4.147	-1.008	-1.741	-3.829	86.178	19.034	18.219
14403	310	46	42	C	W	32.874	1.029	-1.885	4.147	-1.008	1.740	-3.829	86.178	19.034	18.219
14404	310	46	42	C	W	34.715	4.262	1.418	0.920	-3.928	-1.392	-0.904	86.229	18.222	19.031
14405	310	46	42	C	W	50.570	5.011	-1.318	0.227	-4.554	1.312	-0.226	86.238	18.353	18.900
14406	310	46	42	C	W	52.411	0.334	1.994	4.894	-0.332	-1.816	-4.458	86.165	18.904	18.350
14407	310	46	42	C	W	67.589	0.334	-1.994	4.894	-0.332	1.816	-4.458	86.165	18.904	18.350
14408	310	46	42	C	W	69.430	5.011	1.318	0.227	-4.554	-1.312	-0.226	86.238	18.353	18.901
14409	310	46	42	C	W	85.285	4.262	-1.418	0.920	-3.928	1.392	-0.904	86.229	18.222	19.031
14410	310	46	42	C	W	87.126	1.029	1.885	4.147	-1.008	-1.741	-3.829	86.178	19.034	18.219
14411	310	46	42	C	W	92.874	1.029	-1.885	4.147	-1.008	1.740	-3.829	86.178	19.034	18.219
14412	310	46	42	C	W	94.715	4.262	1.418	0.920	-3.928	-1.392	-0.904	86.229	18.222	19.031
14413	310	46	42	C	W	110.570	5.011	-1.318	0.227	-4.554	1.312	-0.226	86.238	18.353	18.900
14414	310	46	42	C	W	112.411	0.334	1.994	4.894	-0.332	-1.816	-4.458	86.165	18.904	18.350
14415	310	46	42	P	S	7.589	0.334	-1.994	4.894	-0.332	1.816	-4.458	86.165	18.904	18.350
14416	310	46	42	P	S	9.430	5.011	1.318	0.227	-4.554	-1.312	-0.226	86.238	18.353	18.900
14417	310	46	42	P	S	25.285	4.262	-1.418	0.920	-3.928	1.392	-0.904	86.229	18.222	19.031
14418	310	46	42	P	S	27.126	1.029	1.885	4.147	-1.008	-1.741	-3.829	86.178	19.034	18.219
14419	310	46	42	P	S	32.874	1.029	-1.885	4.147	-1.008	1.740	-3.829	86.178	19.034	18.219
14420	310	46	42	P	S	34.715	4.262	1.418	0.920	-3.928	-1.392	-0.904	86.229	18.222	19.031
14421	310	46	42	P	S	50.570	5.011	-1.318	0.227	-4.554	1.312	-0.226	86.238	18.353	18.900
14422	310	46	42	P	S	52.411	0.334	1.994	4.894	-0.332	-1.816	-4.458	86.165	18.904	18.350
14423	310	46	42	P	S	67.589	0.334	-1.994	4.894	-0.332	1.816	-4.458	86.165	18.904	18.350
14424	310	46	42	P	S	69.430	5.011	1.318	0.227	-4.554	-1.312	-0.226	86.238	18.353	18.901
14425	310	46	42	P	S	85.285	4.262	-1.418	0.920	-3.928	1.392	-0.904	86.229	18.222	19.031
14426	310	46	42	P	S	87.126	1.029	1.885	4.147	-1.008	-1.741	-3.829	86.178	19.034	18.219
14427	310	46	42	P	S	92.874	1.029	-1.885	4.147	-1.008	1.740	-3.829	86.178	19.034	18.219
14428	310	46	42	P	S	94.715	4.262	1.418	0.920	-3.928	-1.392	-0.904	86.229	18.222	19.031
14429	310	46	42	P	S	110.570	5.011	-1.318	0.227	-4.554	1.312	-0.226	86.238	18.353	18.900
14430	310	46	42	P	S	112.411	0.334	1.994	4.894	-0.332	-1.816	-4.458	86.165	18.904	18.350
14431	310	46	42	P	W	7.589	0.334	-1.994	4.894	-0.332	1.816	-4.458	86.165	18.904	18.350
14432	310	46	42	P	W	9.430	5.011	1.318	0.227	-4.554	-1.312	-0.226	86.238	18.353	18.900
14433	310	46	42	P	W	25.285	4.262	-1.418	0.920	-3.928	1.392	-0.904	86.229	18.222	19.031

BL number	Atoms	γ-PC unit cells	WS_2 unit cells	γ-PC origin atom	WS_2 origin atom	Twist-angle (°)	γ-PC strain 1 (%)	γ-PC strain 2 (%)	γ-PC strain 3 (%)	WS_2 strain 1 (%)	WS_2 strain 2 (%)	WS_2 strain 3 (%)	γ (°)	|**a**|(Å)	|**b**|(Å)
14434	310	46	42	P	W	27.126	1.029	1.885	4.147	-1.008	-1.741	-3.829	86.178	19.034	18.219
14435	310	46	42	P	W	32.874	1.029	-1.885	4.147	-1.008	1.740	-3.829	86.178	19.034	18.219
14436	310	46	42	P	W	34.715	4.262	1.418	0.920	-3.928	-1.392	-0.904	86.229	18.222	19.031
14437	310	46	42	P	W	50.570	5.011	-1.318	0.227	-4.554	1.312	-0.226	86.238	18.353	18.900
14438	310	46	42	P	W	52.411	0.334	1.994	4.894	-0.332	-1.816	-4.458	86.165	18.904	18.350
14439	310	46	42	P	W	67.589	0.334	-1.994	4.894	-0.332	1.816	-4.458	86.165	18.904	18.350
14440	310	46	42	P	W	69.430	5.011	1.318	0.227	-4.554	-1.312	-0.226	86.238	18.353	18.901
14441	310	46	42	P	W	85.285	4.262	-1.418	0.920	-3.928	1.392	-0.904	86.229	18.222	19.031
14442	310	46	42	P	W	87.126	1.029	1.885	4.147	-1.008	-1.741	-3.829	86.178	19.034	18.219
14443	310	46	42	P	W	92.874	1.029	-1.885	4.147	-1.008	1.740	-3.829	86.178	19.034	18.219
14444	310	46	42	P	W	94.715	4.262	1.418	0.920	-3.928	-1.392	-0.904	86.229	18.222	19.031
14445	310	46	42	P	W	110.570	5.011	-1.318	0.227	-4.554	1.312	-0.226	86.238	18.353	18.900
14446	310	46	42	P	W	112.411	0.334	1.994	4.894	-0.332	-1.816	-4.458	86.165	18.904	18.350
14447	312	48	40	C	S	50.817	3.582	-2.121	-2.935	-3.342	2.254	3.118	71.742	18.586	19.548
14448	312	48	40	C	S	69.183	3.582	2.122	-2.935	-3.342	-2.254	3.118	71.742	18.586	19.548
14449	312	48	40	C	W	9.183	3.582	2.122	-2.935	-3.342	-2.254	3.118	71.742	18.586	19.548
14450	312	48	40	C	W	110.817	3.582	-2.121	-2.935	-3.342	2.254	3.118	71.742	18.586	19.548
14451	312	48	40	P	S	50.817	3.582	-2.121	-2.935	-3.342	2.254	3.118	71.742	18.586	19.548
14452	312	48	40	P	S	69.183	3.582	2.122	-2.935	-3.342	-2.254	3.118	71.742	18.586	19.548
14453	312	48	40	P	W	9.183	3.582	2.122	-2.935	-3.342	-2.254	3.118	71.742	18.586	19.548
14454	312	48	40	P	W	110.817	3.582	-2.121	-2.935	-3.342	2.254	3.118	71.742	18.586	19.548
14455	313	49	39	C	S	5.685	-0.923	0.000	-0.922	0.940	0.000	0.940	60.000	19.927	19.927
14456	313	49	39	C	S	16.102	-0.923	0.000	-0.922	0.940	0.000	0.940	60.000	19.927	19.927
14457	313	49	39	C	S	22.111	-0.923	0.000	-0.922	0.940	0.000	0.940	60.000	19.927	19.927
14458	313	49	39	C	S	37.889	-0.923	0.000	-0.922	0.940	0.000	0.940	60.000	19.927	19.927
14459	313	49	39	C	S	43.898	-0.923	0.000	-0.922	0.940	0.000	0.940	60.000	19.927	19.927
14460	313	49	39	C	S	54.315	-0.923	0.000	-0.922	0.940	0.000	0.940	60.000	19.927	19.927
14461	313	49	39	C	S	65.685	-0.923	0.000	-0.922	0.940	0.000	0.940	60.000	19.927	19.927
14462	313	49	39	C	S	76.102	-0.923	0.000	-0.922	0.940	0.000	0.940	60.000	19.927	19.927
14463	313	49	39	C	S	82.111	-0.923	0.000	-0.922	0.940	0.000	0.940	60.000	19.927	19.927
14464	313	49	39	C	S	97.889	-0.923	0.000	-0.922	0.940	0.000	0.940	60.000	19.927	19.927
14465	313	49	39	C	S	103.898	-0.923	0.000	-0.922	0.940	0.000	0.940	60.000	19.927	19.927
14466	313	49	39	C	S	114.315	-0.923	0.000	-0.922	0.940	0.000	0.940	60.000	19.927	19.927
14467	313	49	39	C	W	5.685	-0.923	0.000	-0.922	0.940	0.000	0.940	60.000	19.927	19.927
14468	313	49	39	C	W	16.102	-0.923	0.000	-0.922	0.940	0.000	0.940	60.000	19.927	19.927
14469	313	49	39	C	W	22.111	-0.923	0.000	-0.922	0.940	0.000	0.940	60.000	19.927	19.927
14470	313	49	39	C	W	37.889	-0.923	0.000	-0.922	0.940	0.000	0.940	60.000	19.927	19.927
14471	313	49	39	C	W	43.898	-0.923	0.000	-0.922	0.940	0.000	0.940	60.000	19.927	19.927
14472	313	49	39	C	W	54.315	-0.923	0.000	-0.922	0.940	0.000	0.940	60.000	19.927	19.927
14473	313	49	39	C	W	65.685	-0.923	0.000	-0.922	0.940	0.000	0.940	60.000	19.927	19.927
14474	313	49	39	C	W	76.102	-0.923	0.000	-0.922	0.940	0.000	0.940	60.000	19.927	19.927
14475	313	49	39	C	W	82.111	-0.923	0.000	-0.922	0.940	0.000	0.940	60.000	19.927	19.927
14476	313	49	39	C	W	97.889	-0.923	0.000	-0.922	0.940	0.000	0.940	60.000	19.927	19.927
14477	313	49	39	C	W	103.898	-0.923	0.000	-0.922	0.940	0.000	0.940	60.000	19.927	19.927
14478	313	49	39	C	W	114.315	-0.923	0.000	-0.922	0.940	0.000	0.940	60.000	19.927	19.927
14479	314	47	42	C	S	7.589	0.334	-1.130	3.726	-0.332	1.051	-3.467	84.514	18.904	18.584
14480	314	47	42	C	S	9.183	3.582	1.702	0.470	-3.342	-1.686	-0.466	84.457	18.586	18.903
14481	314	47	42	C	S	16.102	2.853	-1.579	1.166	-2.699	1.543	-1.140	84.472	18.455	19.033
14482	314	47	42	C	S	17.696	1.029	1.258	2.995	-1.008	-1.187	-2.826	84.503	19.034	18.453
14483	314	47	42	C	S	42.304	1.029	-1.258	2.995	-1.008	1.187	-2.826	84.503	19.034	18.453
14484	314	47	42	C	S	43.898	2.853	1.579	1.166	-2.699	-1.543	-1.140	84.472	18.455	19.033

BL number	Atoms	γ-PC unit cells	WS_2 unit cells	γ-PC origin atom	WS_2 origin atom	Twist-angle (°)	γ-PC strain 1 (%)	γ-PC strain 2 (%)	γ-PC strain 3 (%)	WS_2 strain 1 (%)	WS_2 strain 2 (%)	WS_2 strain 3 (%)	γ (°)	$\lvert\mathbf{a}\rvert$(Å)	$\lvert\mathbf{b}\rvert$(Å)
14485	314	47	42	C	S	50.817	3.582	-1.702	0.470	-3.342	1.686	-0.466	84.457	18.586	18.903
14486	314	47	42	C	S	52.411	0.334	1.130	3.726	-0.332	-1.052	-3.467	84.514	18.904	18.584
14487	314	47	42	C	S	67.589	0.334	-1.130	3.726	-0.332	1.051	-3.467	84.514	18.904	18.584
14488	314	47	42	C	S	69.183	3.582	1.702	0.470	-3.342	-1.686	-0.466	84.458	18.586	18.903
14489	314	47	42	C	S	76.102	2.853	-1.579	1.166	-2.699	1.543	-1.140	84.472	18.455	19.033
14490	314	47	42	C	S	77.696	1.029	1.258	2.995	-1.008	-1.187	-2.826	84.503	19.034	18.453
14491	314	47	42	C	S	102.304	1.029	-1.258	2.995	-1.008	1.187	-2.826	84.503	19.034	18.453
14492	314	47	42	C	S	103.898	2.853	1.579	1.166	-2.699	-1.543	-1.140	84.472	18.455	19.033
14493	314	47	42	C	S	110.817	3.582	-1.702	0.470	-3.342	1.686	-0.466	84.458	18.586	18.903
14494	314	47	42	C	S	112.411	0.334	1.130	3.726	-0.332	-1.052	-3.467	84.514	18.904	18.584
14495	314	47	42	C	W	7.589	0.334	-1.130	3.726	-0.332	1.051	-3.467	84.514	18.904	18.584
14496	314	47	42	C	W	9.183	3.582	1.702	0.470	-3.342	-1.686	-0.466	84.457	18.586	18.903
14497	314	47	42	C	W	16.102	2.853	-1.579	1.166	-2.699	1.543	-1.140	84.472	18.455	19.033
14498	314	47	42	C	W	17.696	1.029	1.258	2.995	-1.008	-1.187	-2.826	84.503	19.034	18.453
14499	314	47	42	C	W	42.304	1.029	-1.258	2.995	-1.008	1.187	-2.826	84.503	19.034	18.453
14500	314	47	42	C	W	43.898	2.853	1.579	1.166	-2.699	-1.543	-1.140	84.472	18.455	19.033
14501	314	47	42	C	W	50.817	3.582	-1.702	0.470	-3.342	1.686	-0.466	84.457	18.586	18.903
14502	314	47	42	C	W	52.411	0.334	1.130	3.726	-0.332	-1.052	-3.467	84.514	18.904	18.584
14503	314	47	42	C	W	67.589	0.334	-1.130	3.726	-0.332	1.051	-3.467	84.514	18.904	18.584
14504	314	47	42	C	W	69.183	3.582	1.702	0.470	-3.342	-1.686	-0.466	84.458	18.586	18.903
14505	314	47	42	C	W	76.102	2.853	-1.579	1.166	-2.699	1.543	-1.140	84.472	18.455	19.033
14506	314	47	42	C	W	77.696	1.029	1.258	2.995	-1.008	-1.187	-2.826	84.503	19.034	18.453
14507	314	47	42	C	W	102.304	1.029	-1.258	2.995	-1.008	1.187	-2.826	84.503	19.034	18.453
14508	314	47	42	C	W	103.898	2.853	1.579	1.166	-2.699	-1.543	-1.140	84.472	18.455	19.033
14509	314	47	42	C	W	110.817	3.582	-1.702	0.470	-3.342	1.686	-0.466	84.458	18.586	18.903
14510	314	47	42	C	W	112.411	0.334	1.130	3.726	-0.332	-1.052	-3.467	84.514	18.904	18.584
14511	314	47	42	P	S	7.589	0.334	-1.130	3.726	-0.332	1.051	-3.467	84.514	18.904	18.584
14512	314	47	42	P	S	9.183	3.582	1.702	0.470	-3.342	-1.686	-0.466	84.457	18.586	18.903
14513	314	47	42	P	S	50.817	3.582	-1.702	0.470	-3.342	1.686	-0.466	84.457	18.586	18.903
14514	314	47	42	P	S	52.411	0.334	1.130	3.726	-0.332	-1.052	-3.467	84.514	18.904	18.584
14515	314	47	42	P	S	67.589	0.334	-1.130	3.726	-0.332	1.051	-3.467	84.514	18.904	18.584
14516	314	47	42	P	S	69.183	3.582	1.702	0.470	-3.342	-1.686	-0.466	84.458	18.586	18.903
14517	314	47	42	P	S	110.817	3.582	-1.702	0.470	-3.342	1.686	-0.466	84.458	18.586	18.903
14518	314	47	42	P	S	112.411	0.334	1.130	3.726	-0.332	-1.052	-3.467	84.514	18.904	18.584
14519	314	47	42	P	W	7.589	0.334	-1.130	3.726	-0.332	1.051	-3.467	84.514	18.904	18.584
14520	314	47	42	P	W	9.183	3.582	1.702	0.470	-3.342	-1.686	-0.466	84.457	18.586	18.903
14521	314	47	42	P	W	50.817	3.582	-1.702	0.470	-3.342	1.686	-0.466	84.457	18.586	18.903
14522	314	47	42	P	W	52.411	0.334	1.130	3.726	-0.332	-1.052	-3.467	84.514	18.904	18.584
14523	314	47	42	P	W	67.589	0.334	-1.130	3.726	-0.332	1.051	-3.467	84.514	18.904	18.584
14524	314	47	42	P	W	69.183	3.582	1.702	0.470	-3.342	-1.686	-0.466	84.458	18.586	18.903
14525	314	47	42	P	W	110.817	3.582	-1.702	0.470	-3.342	1.686	-0.466	84.458	18.586	18.903
14526	314	47	42	P	W	112.411	0.334	1.130	3.726	-0.332	-1.052	-3.467	84.514	18.904	18.584
14527	315	45	45	C	S	27.796	5.011	-4.042	5.011	-4.554	3.674	-4.555	90.000	18.842	18.828
14528	315	45	45	C	S	32.204	5.011	4.042	5.011	-4.554	-3.674	-4.555	90.000	18.842	18.828
14529	315	45	45	C	S	87.796	5.011	-4.042	5.011	-4.554	3.674	-4.555	90.000	18.842	18.828
14530	315	45	45	C	W	92.204	5.011	4.042	5.011	-4.554	-3.674	-4.555	90.000	18.842	18.828
14531	315	48	41	C	S	0.000	5.011	-1.895	-3.011	-4.554	2.016	3.204	71.629	18.842	19.545
14532	315	48	41	C	S	18.613	3.582	2.778	-1.758	-3.342	-2.879	1.822	71.601	18.586	19.801
14533	315	48	41	C	S	23.691	2.390	4.784	-0.661	-2.281	-4.848	0.670	72.208	19.291	19.002
14534	315	48	41	C	S	36.309	2.390	-4.784	-0.661	-2.281	4.848	0.669	72.208	19.291	19.002
14535	315	48	41	C	S	41.387	3.582	-2.778	-1.758	-3.342	2.879	1.822	71.601	18.586	19.801

BL number	Atoms	γ-PC unit cells	WS_2 unit cells	γ-PC origin atom	WS_2 origin atom	Twist-angle (°)	γ-PC strain 1 (%)	γ-PC strain 2 (%)	γ-PC strain 3 (%)	WS_2 strain 1 (%)	WS_2 strain 2 (%)	WS_2 strain 3 (%)	γ (°)	|**a**|(Å)	|**b**|(Å)
14536	315	48	41	C	S	60.000	5.011	1.895	-3.011	-4.554	-2.016	3.204	71.629	18.842	19.545
14537	315	48	41	C	S	78.613	3.582	2.778	-1.758	-3.342	-2.879	1.822	71.601	18.586	19.801
14538	315	48	41	C	S	83.691	2.390	4.784	-0.661	-2.281	-4.848	0.670	72.208	19.291	19.002
14539	315	48	41	C	S	96.309	2.390	-4.784	-0.661	-2.281	4.848	0.669	72.208	19.291	19.002
14540	315	48	41	C	S	101.387	3.582	-2.778	-1.758	-3.342	2.879	1.822	71.601	18.586	19.801
14541	315	48	41	C	W	0.000	5.011	-1.895	-3.011	-4.554	2.016	3.204	71.629	18.842	19.545
14542	315	48	41	C	W	18.613	3.582	2.778	-1.758	-3.342	-2.879	1.822	71.601	18.586	19.801
14543	315	48	41	C	W	23.691	2.390	4.784	-0.661	-2.281	-4.848	0.670	72.208	19.291	19.002
14544	315	48	41	C	W	36.309	2.390	-4.784	-0.661	-2.281	4.848	0.669	72.208	19.291	19.002
14545	315	48	41	C	W	41.387	3.582	-2.778	-1.758	-3.342	2.879	1.822	71.601	18.586	19.801
14546	315	48	41	C	W	60.000	5.011	1.895	-3.011	-4.554	-2.016	3.204	71.629	18.842	19.545
14547	315	48	41	C	W	78.613	3.582	2.778	-1.758	-3.342	-2.879	1.822	71.601	18.586	19.801
14548	315	48	41	C	W	83.691	2.390	4.784	-0.661	-2.281	-4.848	0.670	72.208	19.291	19.002
14549	315	48	41	C	W	96.309	2.390	-4.784	-0.661	-2.281	4.848	0.669	72.208	19.291	19.002
14550	315	48	41	C	W	101.387	3.582	-2.778	-1.758	-3.342	2.879	1.822	71.601	18.586	19.801
14551	315	48	41	P	S	0.000	5.011	-1.895	-3.011	-4.554	2.016	3.204	71.629	18.842	19.545
14552	315	48	41	P	S	23.691	2.390	4.784	-0.661	-2.281	-4.848	0.670	72.208	19.291	19.002
14553	315	48	41	P	S	36.309	2.390	-4.784	-0.661	-2.281	4.848	0.669	72.208	19.291	19.002
14554	315	48	41	P	S	41.387	3.582	-2.778	-1.758	-3.342	2.879	1.822	71.601	18.586	19.801
14555	315	48	41	P	S	78.613	3.582	2.778	-1.758	-3.342	-2.879	1.822	71.601	18.586	19.801
14556	315	48	41	P	S	83.691	2.390	4.784	-0.661	-2.281	-4.848	0.670	72.208	19.291	19.002
14557	315	48	41	P	S	96.309	2.390	-4.784	-0.661	-2.281	4.848	0.669	72.208	19.291	19.002
14558	315	48	41	P	W	18.613	3.582	2.778	-1.758	-3.342	-2.879	1.822	71.601	18.586	19.801
14559	315	48	41	P	W	23.691	2.390	4.784	-0.661	-2.281	-4.848	0.670	72.208	19.291	19.002
14560	315	48	41	P	W	36.309	2.390	-4.784	-0.661	-2.281	4.848	0.669	72.208	19.291	19.002
14561	315	48	41	P	W	60.000	5.011	1.895	-3.011	-4.554	-2.016	3.204	71.629	18.842	19.545
14562	315	48	41	P	W	83.691	2.390	4.784	-0.661	-2.281	-4.848	0.670	72.208	19.291	19.002
14563	315	48	41	P	W	96.309	2.390	-4.784	-0.661	-2.281	4.848	0.669	72.208	19.291	19.002
14564	315	48	41	P	W	101.387	3.582	-2.778	-1.758	-3.342	2.879	1.822	71.601	18.586	19.801
14565	317	47	43	C	S	18.613	3.582	-4.333	1.672	-3.342	4.193	-1.617	81.175	18.586	19.268
14566	317	47	43	C	S	23.691	2.390	4.741	2.847	-2.281	-4.485	-2.694	81.124	19.291	18.564
14567	317	47	43	C	S	27.126	1.029	-5.233	4.257	-1.008	4.822	-3.923	81.059	19.034	18.822
14568	317	47	43	C	S	27.796	5.011	-3.870	0.329	-4.554	3.845	-0.327	81.229	18.842	19.011
14569	317	47	43	C	S	32.204	5.011	3.870	0.329	-4.554	-3.845	-0.327	81.229	18.842	19.011
14570	317	47	43	C	S	32.874	1.029	5.233	4.257	-1.008	-4.822	-3.923	81.059	19.034	18.822
14571	317	47	43	C	S	36.309	2.390	-4.741	2.847	-2.281	4.485	-2.694	81.124	19.291	18.564
14572	317	47	43	C	S	41.387	3.582	4.333	1.672	-3.342	-4.193	-1.617	81.175	18.586	19.268
14573	317	47	43	C	S	78.613	3.582	-4.333	1.672	-3.342	4.193	-1.617	81.175	18.586	19.268
14574	317	47	43	C	S	83.691	2.390	4.741	2.847	-2.281	-4.485	-2.694	81.124	19.291	18.564
14575	317	47	43	C	S	87.126	1.029	-5.233	4.257	-1.008	4.822	-3.923	81.059	19.034	18.822
14576	317	47	43	C	S	87.796	5.011	-3.870	0.329	-4.554	3.845	-0.327	81.229	18.842	19.011
14577	317	47	43	C	S	92.204	5.011	3.870	0.329	-4.554	-3.845	-0.327	81.229	18.842	19.011
14578	317	47	43	C	S	92.874	1.029	5.233	4.257	-1.008	-4.822	-3.923	81.059	19.034	18.822
14579	317	47	43	C	S	96.309	2.390	-4.741	2.847	-2.281	4.485	-2.694	81.124	19.291	18.564
14580	317	47	43	C	S	101.387	3.582	4.333	1.672	-3.342	-4.193	-1.617	81.175	18.586	19.268
14581	317	47	43	C	W	18.613	3.582	-4.333	1.672	-3.342	4.193	-1.617	81.175	18.586	19.268
14582	317	47	43	C	W	23.691	2.390	4.741	2.847	-2.281	-4.485	-2.694	81.124	19.291	18.564
14583	317	47	43	C	W	27.126	1.029	-5.233	4.257	-1.008	4.822	-3.923	81.059	19.034	18.822
14584	317	47	43	C	W	27.796	5.011	-3.870	0.329	-4.554	3.845	-0.327	81.229	18.842	19.011
14585	317	47	43	C	W	32.204	5.011	3.870	0.329	-4.554	-3.845	-0.327	81.229	18.842	19.011
14586	317	47	43	C	W	32.874	1.029	5.233	4.257	-1.008	-4.822	-3.923	81.059	19.034	18.822

BL number	Atoms	γ-PC unit cells	WS_2 unit cells	γ-PC origin atom	WS_2 origin atom	Twist-angle (°)	γ-PC strain 1 (%)	γ-PC strain 2 (%)	γ-PC strain 3 (%)	WS_2 strain 1 (%)	WS_2 strain 2 (%)	WS_2 strain 3 (%)	γ (°)	|**a**|(Å)	|**b**|(Å)
14587	317	47	43	C	W	36.309	2.390	-4.741	2.847	-2.281	4.485	-2.694	81.124	19.291	18.564
14588	317	47	43	C	W	41.387	3.582	4.333	1.672	-3.342	-4.193	-1.617	81.175	18.586	19.268
14589	317	47	43	C	W	78.613	3.582	-4.333	1.672	-3.342	4.193	-1.617	81.175	18.586	19.268
14590	317	47	43	C	W	83.691	2.390	4.741	2.847	-2.281	-4.485	-2.694	81.124	19.291	18.564
14591	317	47	43	C	W	87.126	1.029	-5.233	4.257	-1.008	4.822	-3.923	81.059	19.034	18.822
14592	317	47	43	C	W	87.796	5.011	-3.870	0.329	-4.554	3.845	-0.327	81.229	18.842	19.011
14593	317	47	43	C	W	92.204	5.011	3.870	0.329	-4.554	-3.845	-0.327	81.229	18.842	19.011
14594	317	47	43	C	W	92.874	1.029	5.233	4.257	-1.008	-4.822	-3.923	81.059	19.034	18.822
14595	317	47	43	C	W	96.309	2.390	-4.741	2.847	-2.281	4.485	-2.694	81.124	19.291	18.564
14596	317	47	43	C	W	101.387	3.582	4.333	1.672	-3.342	-4.193	-1.617	81.175	18.586	19.268
14597	317	47	43	P	S	18.613	3.582	-4.333	1.672	-3.342	4.193	-1.617	81.175	18.586	19.268
14598	317	47	43	P	S	23.691	2.390	4.741	2.847	-2.281	-4.485	-2.694	81.124	19.291	18.564
14599	317	47	43	P	S	27.796	5.011	-3.870	0.329	-4.554	3.845	-0.327	81.229	18.842	19.011
14600	317	47	43	P	S	32.874	1.029	5.233	4.257	-1.008	-4.822	-3.923	81.059	19.034	18.822
14601	317	47	43	P	S	36.309	2.390	-4.741	2.847	-2.281	4.485	-2.694	81.124	19.291	18.564
14602	317	47	43	P	S	41.387	3.582	4.333	1.672	-3.342	-4.193	-1.617	81.175	18.586	19.268
14603	317	47	43	P	S	78.613	3.582	-4.333	1.672	-3.342	4.193	-1.617	81.175	18.586	19.268
14604	317	47	43	P	S	83.691	2.390	4.741	2.847	-2.281	-4.485	-2.694	81.124	19.291	18.564
14605	317	47	43	P	S	87.126	1.029	-5.233	4.257	-1.008	4.822	-3.923	81.059	19.034	18.822
14606	317	47	43	P	S	92.204	5.011	3.870	0.329	-4.554	-3.845	-0.327	81.229	18.842	19.011
14607	317	47	43	P	S	96.309	2.390	-4.741	2.847	-2.281	4.485	-2.694	81.124	19.291	18.564
14608	317	47	43	P	S	101.387	3.582	4.333	1.672	-3.342	-4.193	-1.617	81.175	18.586	19.268
14609	317	47	43	P	W	18.613	3.582	-4.333	1.672	-3.342	4.193	-1.617	81.175	18.586	19.268
14610	317	47	43	P	W	23.691	2.390	4.741	2.847	-2.281	-4.485	-2.694	81.124	19.291	18.564
14611	317	47	43	P	W	27.126	1.029	-5.233	4.257	-1.008	4.822	-3.923	81.059	19.034	18.822
14612	317	47	43	P	W	32.204	5.011	3.870	0.329	-4.554	-3.845	-0.327	81.229	18.842	19.011
14613	317	47	43	P	W	36.309	2.390	-4.741	2.847	-2.281	4.485	-2.694	81.124	19.291	18.564
14614	317	47	43	P	W	41.387	3.582	4.333	1.672	-3.342	-4.193	-1.617	81.175	18.586	19.268
14615	317	47	43	P	W	78.613	3.582	-4.333	1.672	-3.342	4.193	-1.617	81.175	18.586	19.268
14616	317	47	43	P	W	83.691	2.390	4.741	2.847	-2.281	-4.485	-2.694	81.124	19.291	18.564
14617	317	47	43	P	W	87.796	5.011	-3.870	0.329	-4.554	3.845	-0.327	81.229	18.842	19.011
14618	317	47	43	P	W	92.874	1.029	5.233	4.257	-1.008	-4.822	-3.923	81.059	19.034	18.822
14619	317	47	43	P	W	96.309	2.390	-4.741	2.847	-2.281	4.485	-2.694	81.124	19.291	18.564
14620	317	47	43	P	W	101.387	3.582	4.333	1.672	-3.342	-4.193	-1.617	81.175	18.586	19.268
14621	318	48	42	C	S	40.893	-1.485	0.000	4.580	1.530	0.000	-4.195	90.000	14.708	24.038
14622	318	48	42	C	S	100.893	-1.485	0.000	4.580	1.530	0.000	-4.195	90.000	14.708	24.038
14623	318	48	42	P	S	19.107	-1.485	0.000	4.580	1.530	0.000	-4.195	90.000	14.708	24.038
14624	318	48	42	P	S	40.893	-1.485	0.000	4.580	1.530	0.000	-4.195	90.000	14.708	24.038
14625	318	48	42	P	S	79.107	-1.485	0.000	4.580	1.530	0.000	-4.195	90.000	14.708	24.038
14626	318	48	42	P	S	100.893	-1.485	0.000	4.580	1.530	0.000	-4.195	90.000	14.708	24.038
14627	318	48	42	C	S	7.589	0.334	-0.302	2.607	-0.332	0.287	-2.477	75.650	18.904	19.290
14628	318	48	42	C	S	13.898	-0.414	-1.106	3.401	0.417	1.035	-3.184	75.033	19.824	18.451
14629	318	48	42	C	S	16.102	2.853	2.783	0.100	-2.699	-2.778	-0.100	74.954	18.455	19.824
14630	318	48	42	C	S	43.898	2.853	-2.783	0.100	-2.699	2.778	-0.100	74.954	18.455	19.824
14631	318	48	42	C	S	46.102	-0.414	1.106	3.401	0.417	-1.035	-3.184	75.033	19.824	18.451
14632	318	48	42	C	S	51.487	2.390	-1.326	0.543	-2.281	1.312	-0.537	75.625	19.291	18.904
14633	318	48	42	C	S	52.411	0.334	0.302	2.607	-0.332	-0.287	-2.477	75.650	18.904	19.290
14634	318	48	42	C	S	67.589	0.334	-0.302	2.607	-0.332	0.287	-2.477	75.650	18.904	19.290
14635	318	48	42	C	S	68.513	2.390	1.326	0.543	-2.281	-1.312	-0.537	75.625	19.291	18.904
14636	318	48	42	C	S	73.898	-0.414	-1.106	3.401	0.417	1.035	-3.184	75.033	19.824	18.451
14637	318	48	42	C	S	76.102	2.853	2.783	0.100	-2.699	-2.778	-0.100	74.954	18.455	19.824

BL number	Atoms	γ-PC unit cells	WS_2 unit cells	γ-PC origin atom	WS_2 origin atom	Twist-angle (°)	γ-PC strain 1 (%)	γ-PC strain 2 (%)	γ-PC strain 3 (%)	WS_2 strain 1 (%)	WS_2 strain 2 (%)	WS_2 strain 3 (%)	γ (°)	|**a**|(Å)	|**b**|(Å)
14638	318	48	42	C	S	103.898	2.853	-2.783	0.100	-2.699	2.778	-0.100	74.954	18.455	19.824
14639	318	48	42	C	S	106.102	-0.414	1.106	3.401	0.417	-1.035	-3.184	75.033	19.824	18.451
14640	318	48	42	C	S	111.487	2.390	-1.326	0.543	-2.281	1.312	-0.537	75.625	19.291	18.904
14641	318	48	42	C	S	112.411	0.334	0.302	2.607	-0.332	-0.287	-2.477	75.650	18.904	19.290
14642	318	48	42	C	W	8.513	2.390	1.326	0.543	-2.281	-1.312	-0.537	75.625	19.291	18.905
14643	318	48	42	C	W	13.898	-0.414	-1.106	3.401	0.417	1.035	-3.184	75.033	19.824	18.451
14644	318	48	42	P	S	7.589	0.334	-0.302	2.607	-0.332	0.287	-2.477	75.650	18.904	19.290
14645	318	48	42	P	S	51.487	2.390	-1.326	0.543	-2.281	1.312	-0.537	75.625	19.291	18.904
14646	318	48	42	P	S	52.411	0.334	0.302	2.607	-0.332	-0.287	-2.477	75.650	18.904	19.290
14647	318	48	42	P	S	67.589	0.334	-0.302	2.607	-0.332	0.287	-2.477	75.650	18.904	19.290
14648	318	48	42	P	S	68.513	2.390	1.326	0.543	-2.281	-1.312	-0.537	75.625	19.291	18.904
14649	318	48	42	P	S	73.898	-0.414	-1.106	3.401	0.417	1.035	-3.184	75.033	19.824	18.451
14650	318	48	42	P	S	111.487	2.390	-1.326	0.543	-2.281	1.312	-0.537	75.625	19.291	18.904
14651	318	48	42	P	S	112.411	0.334	0.302	2.607	-0.332	-0.287	-2.477	75.650	18.904	19.290
14652	318	48	42	P	W	8.513	2.390	1.326	0.543	-2.281	-1.312	-0.537	75.625	19.291	18.905
14653	318	48	42	P	W	112.411	0.334	0.302	2.607	-0.332	-0.287	-2.477	75.650	18.904	19.290
14654	319	52	37	C	S	11.387	-3.597	0.000	-3.597	3.876	0.000	3.876	60.000	19.974	19.974
14655	319	52	37	C	S	20.817	-3.597	0.000	-3.597	3.876	0.000	3.876	60.000	19.974	19.974
14656	319	52	37	C	S	39.183	-3.597	0.000	-3.597	3.876	0.000	3.876	60.000	19.974	19.974
14657	319	52	37	C	S	48.613	-3.597	0.000	-3.597	3.876	0.000	3.876	60.000	19.974	19.974
14658	319	52	37	C	S	71.387	-3.597	0.000	-3.597	3.876	0.000	3.876	60.000	19.974	19.974
14659	319	52	37	C	S	80.817	-3.597	0.000	-3.597	3.876	0.000	3.876	60.000	19.974	19.974
14660	319	52	37	C	S	99.183	-3.597	0.000	-3.597	3.876	0.000	3.876	60.000	19.974	19.974
14661	319	52	37	C	S	108.613	-3.597	0.000	-3.597	3.876	0.000	3.876	60.000	19.974	19.974
14662	319	52	37	C	W	11.387	-3.597	0.000	-3.597	3.876	0.000	3.876	60.000	19.974	19.974
14663	319	52	37	C	W	39.183	-3.597	0.000	-3.597	3.876	0.000	3.876	60.000	19.974	19.974
14664	319	52	37	C	W	48.613	-3.597	0.000	-3.597	3.876	0.000	3.876	60.000	19.974	19.974
14665	319	52	37	C	W	71.387	-3.597	0.000	-3.597	3.876	0.000	3.876	60.000	19.974	19.974
14666	319	52	37	C	W	80.817	-3.597	0.000	-3.597	3.876	0.000	3.876	60.000	19.974	19.974
14667	319	52	37	C	W	99.183	-3.597	0.000	-3.597	3.876	0.000	3.876	60.000	19.974	19.974
14668	319	52	37	C	W	108.613	-3.597	0.000	-3.597	3.876	0.000	3.876	60.000	19.974	19.974
14669	319	52	37	P	S	11.387	-3.597	0.000	-3.597	3.876	0.000	3.876	60.000	19.974	19.974
14670	319	52	37	P	S	39.183	-3.597	0.000	-3.597	3.876	0.000	3.876	60.000	19.974	19.974
14671	319	52	37	P	S	80.817	-3.597	0.000	-3.597	3.876	0.000	3.876	60.000	19.974	19.974
14672	319	52	37	P	S	108.613	-3.597	0.000	-3.597	3.876	0.000	3.876	60.000	19.974	19.974
14673	319	52	37	P	W	20.817	-3.597	0.000	-3.597	3.876	0.000	3.876	60.000	19.974	19.974
14674	319	52	37	P	W	48.613	-3.597	0.000	-3.597	3.876	0.000	3.876	60.000	19.974	19.974
14675	319	52	37	P	W	71.387	-3.597	0.000	-3.597	3.876	0.000	3.876	60.000	19.974	19.974
14676	319	52	37	P	W	99.183	-3.597	0.000	-3.597	3.876	0.000	3.876	60.000	19.974	19.974
14677	321	48	43	C	S	22.411	2.067	0.000	2.067	-1.985	0.000	-1.985	60.000	20.317	20.317
14678	321	48	43	C	S	37.589	2.067	0.000	2.067	-1.985	0.000	-1.985	60.000	20.317	20.317
14679	321	48	43	C	S	82.411	2.067	0.000	2.067	-1.985	0.000	-1.985	60.000	20.317	20.317
14680	321	48	43	C	S	97.589	2.067	0.000	2.067	-1.985	0.000	-1.985	60.000	20.317	20.317
14681	321	48	43	P	S	22.411	2.067	0.000	2.067	-1.985	0.000	-1.985	60.000	20.317	20.317
14682	321	48	43	P	S	37.589	2.067	0.000	2.067	-1.985	0.000	-1.985	60.000	20.317	20.317
14683	321	48	43	P	S	82.411	2.067	0.000	2.067	-1.985	0.000	-1.985	60.000	20.317	20.317
14684	321	48	43	P	S	97.589	2.067	0.000	2.067	-1.985	0.000	-1.985	60.000	20.317	20.317
14685	321	48	43	C	S	9.183	3.582	-5.101	0.595	-3.342	5.042	-0.588	76.151	18.586	19.815
14686	321	48	43	C	S	13.898	-0.414	3.317	4.672	0.417	-3.034	-4.273	76.344	19.824	18.569
14687	321	48	43	C	S	23.691	2.390	-3.363	1.746	-2.281	3.250	-1.687	76.871	19.291	19.029
14688	321	48	43	C	S	27.126	1.029	2.775	3.126	-1.008	-2.612	-2.942	76.921	19.034	19.283

BL number	Atoms	γ-PC unit cells	WS_2 unit cells	γ-PC origin atom	WS_2 origin atom	Twist-angle (°)	γ-PC strain 1 (%)	γ-PC strain 2 (%)	γ-PC strain 3 (%)	WS_2 strain 1 (%)	WS_2 strain 2 (%)	WS_2 strain 3 (%)	γ (°)	|**a**|(Å)	|**b**|(Å)
14689	321	48	43	C	S	32.874	1.029	-2.775	3.126	-1.008	2.612	-2.942	76.921	19.034	19.283
14690	321	48	43	C	S	36.309	2.390	3.363	1.746	-2.281	-3.250	-1.687	76.871	19.291	19.029
14691	321	48	43	C	S	46.102	-0.414	-3.317	4.672	0.417	3.034	-4.273	76.344	19.824	18.569
14692	321	48	43	C	S	50.817	3.582	5.101	0.595	-3.342	-5.042	-0.588	76.151	18.586	19.815
14693	321	48	43	C	S	69.183	3.582	-5.101	0.595	-3.342	5.042	-0.588	76.151	18.586	19.815
14694	321	48	43	C	S	73.898	-0.414	3.317	4.672	0.417	-3.034	-4.273	76.344	19.824	18.569
14695	321	48	43	C	S	83.691	2.390	-3.363	1.746	-2.281	3.250	-1.687	76.871	19.291	19.029
14696	321	48	43	C	S	87.126	1.029	2.775	3.126	-1.008	-2.612	-2.942	76.921	19.034	19.283
14697	321	48	43	C	S	92.874	1.029	-2.775	3.126	-1.008	2.612	-2.942	76.921	19.034	19.283
14698	321	48	43	C	S	96.309	2.390	3.363	1.746	-2.281	-3.250	-1.687	76.871	19.291	19.029
14699	321	48	43	C	S	106.102	-0.414	-3.317	4.672	0.417	3.034	-4.273	76.344	19.824	18.569
14700	321	48	43	C	S	110.817	3.582	5.101	0.595	-3.342	-5.042	-0.588	76.151	18.586	19.815
14701	321	48	43	C	W	9.183	3.582	-5.101	0.595	-3.342	5.042	-0.588	76.151	18.586	19.815
14702	321	48	43	C	W	13.898	-0.414	3.317	4.672	0.417	-3.034	-4.273	76.344	19.824	18.569
14703	321	48	43	C	W	23.691	2.390	-3.363	1.746	-2.281	3.250	-1.687	76.871	19.291	19.029
14704	321	48	43	C	W	27.126	1.029	2.775	3.126	-1.008	-2.612	-2.942	76.921	19.034	19.283
14705	321	48	43	C	W	32.874	1.029	-2.775	3.126	-1.008	2.612	-2.942	76.921	19.034	19.283
14706	321	48	43	C	W	36.309	2.390	3.363	1.746	-2.281	-3.250	-1.687	76.871	19.291	19.029
14707	321	48	43	C	W	46.102	-0.414	-3.317	4.672	0.417	3.034	-4.273	76.344	19.824	18.569
14708	321	48	43	C	W	50.817	3.582	5.101	0.595	-3.342	-5.042	-0.588	76.151	18.586	19.815
14709	321	48	43	C	W	69.183	3.582	-5.101	0.595	-3.342	5.042	-0.588	76.151	18.586	19.815
14710	321	48	43	C	W	73.898	-0.414	3.317	4.672	0.417	-3.034	-4.273	76.344	19.824	18.569
14711	321	48	43	C	W	83.691	2.390	-3.363	1.746	-2.281	3.250	-1.687	76.871	19.291	19.029
14712	321	48	43	C	W	87.126	1.029	2.775	3.126	-1.008	-2.612	-2.942	76.921	19.034	19.283
14713	321	48	43	C	W	92.874	1.029	-2.775	3.126	-1.008	2.612	-2.942	76.921	19.034	19.283
14714	321	48	43	C	W	96.309	2.390	3.363	1.746	-2.281	-3.250	-1.687	76.871	19.291	19.029
14715	321	48	43	C	W	106.102	-0.414	-3.317	4.672	0.417	3.034	-4.273	76.344	19.824	18.569
14716	321	48	43	C	W	110.817	3.582	5.101	0.595	-3.342	-5.042	-0.588	76.151	18.586	19.815
14717	321	48	43	P	S	23.691	2.390	-3.363	1.746	-2.281	3.250	-1.687	76.871	19.291	19.029
14718	321	48	43	P	S	27.126	1.029	2.775	3.126	-1.008	-2.612	-2.942	76.921	19.034	19.283
14719	321	48	43	P	S	32.874	1.029	-2.775	3.126	-1.008	2.612	-2.942	76.921	19.034	19.283
14720	321	48	43	P	S	36.309	2.390	3.363	1.746	-2.281	-3.250	-1.687	76.871	19.291	19.029
14721	321	48	43	P	S	46.102	-0.414	-3.317	4.672	0.417	3.034	-4.273	76.344	19.824	18.569
14722	321	48	43	P	S	50.817	3.582	5.101	0.595	-3.342	-5.042	-0.588	76.151	18.586	19.815
14723	321	48	43	P	S	69.183	3.582	-5.101	0.595	-3.342	5.042	-0.588	76.151	18.586	19.815
14724	321	48	43	P	S	73.898	-0.414	3.317	4.672	0.417	-3.034	-4.273	76.344	19.824	18.569
14725	321	48	43	P	S	83.691	2.390	-3.363	1.746	-2.281	3.250	-1.687	76.871	19.291	19.029
14726	321	48	43	P	S	87.126	1.029	2.775	3.126	-1.008	-2.612	-2.942	76.921	19.034	19.283
14727	321	48	43	P	S	92.874	1.029	-2.775	3.126	-1.008	2.612	-2.942	76.921	19.034	19.283
14728	321	48	43	P	S	96.309	2.390	3.363	1.746	-2.281	-3.250	-1.687	76.871	19.291	19.029
14729	321	48	43	P	W	9.183	3.582	-5.101	0.595	-3.342	5.042	-0.588	76.151	18.586	19.815
14730	321	48	43	P	W	13.898	-0.414	3.317	4.672	0.417	-3.034	-4.273	76.344	19.824	18.569
14731	321	48	43	P	W	23.691	2.390	-3.363	1.746	-2.281	3.250	-1.687	76.871	19.291	19.029
14732	321	48	43	P	W	27.126	1.029	2.775	3.126	-1.008	-2.612	-2.942	76.921	19.034	19.283
14733	321	48	43	P	W	32.874	1.029	-2.775	3.126	-1.008	2.612	-2.942	76.921	19.034	19.283
14734	321	48	43	P	W	36.309	2.390	3.363	1.746	-2.281	-3.250	-1.687	76.871	19.291	19.029
14735	321	48	43	P	W	83.691	2.390	-3.363	1.746	-2.281	3.250	-1.687	76.871	19.291	19.029
14736	321	48	43	P	W	87.126	1.029	2.775	3.126	-1.008	-2.612	-2.942	76.921	19.034	19.283
14737	321	48	43	P	W	92.874	1.029	-2.775	3.126	-1.008	2.612	-2.942	76.921	19.034	19.283
14738	321	48	43	P	W	96.309	2.390	3.363	1.746	-2.281	-3.250	-1.687	76.871	19.291	19.029
14739	321	48	43	P	W	106.102	-0.414	-3.317	4.672	0.417	3.034	-4.273	76.344	19.824	18.569

BL number	Atoms	γ-PC unit cells	WS_2 unit cells	γ-PC origin atom	WS_2 origin atom	Twist-angle (°)	γ-PC strain 1 (%)	γ-PC strain 2 (%)	γ-PC strain 3 (%)	WS_2 strain 1 (%)	WS_2 strain 2 (%)	WS_2 strain 3 (%)	γ (°)	|**a**|(Å)	|**b**|(Å)
14740	321	48	43	P	W	110.817	3.582	5.101	0.595	-3.342	-5.042	-0.588	76.151	18.586	19.815
14741	322	52	38	C	S	55.285	-1.702	-4.778	-4.212	1.762	5.218	4.600	79.430	19.567	18.198
14742	322	52	38	C	S	64.715	-1.702	4.779	-4.212	1.762	-5.218	4.600	79.429	19.567	18.198
14743	322	52	38	C	S	115.285	-1.702	-4.778	-4.212	1.762	5.218	4.600	79.429	19.567	18.198
14744	322	52	38	C	W	55.285	-1.702	-4.778	-4.212	1.762	5.218	4.600	79.430	19.567	18.198
14745	322	52	38	C	W	64.715	-1.702	4.779	-4.212	1.762	-5.218	4.600	79.429	19.567	18.198
14746	322	52	38	C	W	115.285	-1.702	-4.778	-4.212	1.762	5.218	4.600	79.429	19.567	18.198
14747	322	52	38	P	S	55.285	-1.702	-4.778	-4.212	1.762	5.218	4.600	79.430	19.567	18.198
14748	322	52	38	P	S	64.715	-1.702	4.779	-4.212	1.762	-5.218	4.600	79.429	19.567	18.198
14749	322	52	38	P	S	115.285	-1.702	-4.778	-4.212	1.762	5.218	4.600	79.429	19.567	18.198
14750	322	52	38	P	W	55.285	-1.702	-4.778	-4.212	1.762	5.218	4.600	79.430	19.567	18.198
14751	322	52	38	P	W	64.715	-1.702	4.779	-4.212	1.762	-5.218	4.600	79.429	19.567	18.198
14752	322	52	38	P	W	115.285	-1.702	-4.778	-4.212	1.762	5.218	4.600	79.429	19.567	18.198
14753	323	47	45	C	S	23.691	2.390	-3.580	5.305	-2.281	3.236	-4.796	85.796	19.291	18.831
14754	323	47	45	C	S	27.796	5.011	3.870	2.670	-4.554	-3.674	-2.535	85.689	18.842	19.281
14755	323	47	45	C	S	32.204	5.011	-3.870	2.670	-4.554	3.674	-2.535	85.689	18.842	19.281
14756	323	47	45	C	S	36.309	2.390	3.580	5.305	-2.281	-3.236	-4.796	85.796	19.291	18.831
14757	323	47	45	C	S	83.691	2.390	-3.580	5.305	-2.281	3.236	-4.796	85.796	19.291	18.831
14758	323	47	45	C	S	87.796	5.011	3.870	2.670	-4.554	-3.674	-2.535	85.689	18.842	19.281
14759	323	47	45	C	S	92.204	5.011	-3.870	2.670	-4.554	3.674	-2.535	85.689	18.842	19.281
14760	323	47	45	C	S	96.309	2.390	3.580	5.305	-2.281	-3.236	-4.796	85.796	19.291	18.831
14761	323	47	45	C	W	23.691	2.390	-3.580	5.305	-2.281	3.236	-4.796	85.796	19.291	18.831
14762	323	47	45	C	W	27.796	5.011	3.870	2.670	-4.554	-3.674	-2.535	85.689	18.842	19.281
14763	323	47	45	C	W	32.204	5.011	-3.870	2.670	-4.554	3.674	-2.535	85.689	18.842	19.281
14764	323	47	45	C	W	36.309	2.390	3.580	5.305	-2.281	-3.236	-4.796	85.796	19.291	18.831
14765	323	47	45	C	W	83.691	2.390	-3.580	5.305	-2.281	3.236	-4.796	85.796	19.291	18.831
14766	323	47	45	C	W	87.796	5.011	3.870	2.670	-4.554	-3.674	-2.535	85.689	18.842	19.281
14767	323	47	45	C	W	92.204	5.011	-3.870	2.670	-4.554	3.674	-2.535	85.689	18.842	19.281
14768	323	47	45	C	W	96.309	2.390	3.580	5.305	-2.281	-3.236	-4.796	85.796	19.291	18.831
14769	323	47	45	P	S	23.691	2.390	-3.580	5.305	-2.281	3.236	-4.796	85.796	19.291	18.831
14770	323	47	45	P	S	27.796	5.011	3.870	2.670	-4.554	-3.674	-2.535	85.689	18.842	19.281
14771	323	47	45	P	S	92.204	5.011	-3.870	2.670	-4.554	3.674	-2.535	85.689	18.842	19.281
14772	323	47	45	P	S	96.309	2.390	3.580	5.305	-2.281	-3.236	-4.796	85.796	19.291	18.831
14773	323	47	45	P	W	32.204	5.011	-3.870	2.670	-4.554	3.674	-2.535	85.689	18.842	19.281
14774	323	47	45	P	W	36.309	2.390	3.580	5.305	-2.281	-3.236	-4.796	85.796	19.291	18.831
14775	323	47	45	P	W	83.691	2.390	-3.580	5.305	-2.281	3.236	-4.796	85.796	19.291	18.831
14776	323	47	45	P	W	87.796	5.011	3.870	2.670	-4.554	-3.674	-2.535	85.689	18.842	19.281
14777	325	49	43	C	S	7.589	1.533	0.000	1.533	-1.487	0.000	-1.487	60.000	20.421	20.421
14778	325	49	43	C	S	14.198	1.533	0.000	1.533	-1.487	0.000	-1.487	60.000	20.421	20.421
14779	325	49	43	C	S	29.376	1.533	0.000	1.533	-1.487	0.000	-1.487	60.000	20.421	20.421
14780	325	49	43	C	S	30.624	1.533	0.000	1.533	-1.487	0.000	-1.487	60.000	20.421	20.421
14781	325	49	43	C	S	45.802	1.533	0.000	1.533	-1.487	0.000	-1.487	60.000	20.421	20.421
14782	325	49	43	C	S	52.411	1.533	0.000	1.533	-1.487	0.000	-1.487	60.000	20.421	20.421
14783	325	49	43	C	S	67.589	1.533	0.000	1.533	-1.487	0.000	-1.487	60.000	20.421	20.421
14784	325	49	43	C	S	74.198	1.533	0.000	1.533	-1.487	0.000	-1.487	60.000	20.421	20.421
14785	325	49	43	C	S	89.376	1.533	0.000	1.533	-1.487	0.000	-1.487	60.000	20.421	20.421
14786	325	49	43	C	S	90.624	1.533	0.000	1.533	-1.487	0.000	-1.487	60.000	20.421	20.421
14787	325	49	43	C	S	105.802	1.533	0.000	1.533	-1.487	0.000	-1.487	60.000	20.421	20.421
14788	325	49	43	C	S	112.411	1.533	0.000	1.533	-1.487	0.000	-1.487	60.000	20.421	20.421
14789	325	49	43	C	W	7.589	1.533	0.000	1.533	-1.487	0.000	-1.487	60.000	20.421	20.421
14790	325	49	43	C	W	14.198	1.533	0.000	1.533	-1.487	0.000	-1.487	60.000	20.421	20.421

BL number	Atoms	γ-PC unit cells	WS_2 unit cells	γ-PC origin atom	WS_2 origin atom	Twist-angle (°)	γ-PC strain 1 (%)	γ-PC strain 2 (%)	γ-PC strain 3 (%)	WS_2 strain 1 (%)	WS_2 strain 2 (%)	WS_2 strain 3 (%)	γ (°)	|**a**|(Å)	|**b**|(Å)
14791	325	49	43	C	W	29.376	1.533	0.000	1.533	-1.487	0.000	-1.487	60.000	20.421	20.421
14792	325	49	43	C	W	30.624	1.533	0.000	1.533	-1.487	0.000	-1.487	60.000	20.421	20.421
14793	325	49	43	C	W	45.802	1.533	0.000	1.533	-1.487	0.000	-1.487	60.000	20.421	20.421
14794	325	49	43	C	W	52.411	1.533	0.000	1.533	-1.487	0.000	-1.487	60.000	20.421	20.421
14795	325	49	43	C	W	67.589	1.533	0.000	1.533	-1.487	0.000	-1.487	60.000	20.421	20.421
14796	325	49	43	C	W	74.198	1.533	0.000	1.533	-1.487	0.000	-1.487	60.000	20.421	20.421
14797	325	49	43	C	W	89.376	1.533	0.000	1.533	-1.487	0.000	-1.487	60.000	20.421	20.421
14798	325	49	43	C	W	90.624	1.533	0.000	1.533	-1.487	0.000	-1.487	60.000	20.421	20.421
14799	325	49	43	C	W	105.802	1.533	0.000	1.533	-1.487	0.000	-1.487	60.000	20.421	20.421
14800	325	49	43	C	W	112.411	1.533	0.000	1.533	-1.487	0.000	-1.487	60.000	20.421	20.421
14801	325	49	43	P	S	14.198	1.533	0.000	1.533	-1.487	0.000	-1.487	60.000	20.421	20.421
14802	325	49	43	P	S	29.376	1.533	0.000	1.533	-1.487	0.000	-1.487	60.000	20.421	20.421
14803	325	49	43	P	S	52.411	1.533	0.000	1.533	-1.487	0.000	-1.487	60.000	20.421	20.421
14804	325	49	43	P	S	67.589	1.533	0.000	1.533	-1.487	0.000	-1.487	60.000	20.421	20.421
14805	325	49	43	P	S	90.624	1.533	0.000	1.533	-1.487	0.000	-1.487	60.000	20.421	20.421
14806	325	49	43	P	S	105.802	1.533	0.000	1.533	-1.487	0.000	-1.487	60.000	20.421	20.421
14807	325	49	43	P	W	7.589	1.533	0.000	1.533	-1.487	0.000	-1.487	60.000	20.421	20.421
14808	325	49	43	P	W	30.624	1.533	0.000	1.533	-1.487	0.000	-1.487	60.000	20.421	20.421
14809	325	49	43	P	W	45.802	1.533	0.000	1.533	-1.487	0.000	-1.487	60.000	20.421	20.421
14810	325	49	43	P	W	74.198	1.533	0.000	1.533	-1.487	0.000	-1.487	60.000	20.421	20.421
14811	325	49	43	P	W	89.376	1.533	0.000	1.533	-1.487	0.000	-1.487	60.000	20.421	20.421
14812	325	49	43	P	W	112.411	1.533	0.000	1.533	-1.487	0.000	-1.487	60.000	20.421	20.421
14813	325	52	39	C	S	2.204	-2.359	0.000	-2.359	2.476	0.000	2.476	60.000	20.230	20.230
14814	325	52	39	C	S	57.796	-2.359	0.000	-2.359	2.476	0.000	2.476	60.000	20.230	20.230
14815	325	52	39	C	S	62.204	-2.359	0.000	-2.359	2.476	0.000	2.476	60.000	20.230	20.230
14816	325	52	39	C	S	117.796	-2.359	0.000	-2.359	2.476	0.000	2.476	60.000	20.230	20.230
14817	325	52	39	C	W	2.204	-2.359	0.000	-2.359	2.476	0.000	2.476	60.000	20.230	20.230
14818	325	52	39	C	W	57.796	-2.359	0.000	-2.359	2.476	0.000	2.476	60.000	20.230	20.230
14819	325	52	39	C	W	62.204	-2.359	0.000	-2.359	2.476	0.000	2.476	60.000	20.230	20.230
14820	325	52	39	C	W	117.796	-2.359	0.000	-2.359	2.476	0.000	2.476	60.000	20.230	20.230
14821	325	52	39	C	S	1.359	-3.292	-2.719	-1.408	3.523	2.798	1.449	84.113	18.221	19.557
14822	325	52	39	C	S	4.715	-1.702	3.009	-3.007	1.762	-3.201	3.200	84.062	19.567	18.212
14823	325	52	39	C	S	55.285	-1.702	-3.009	-3.007	1.762	3.201	3.200	84.062	19.567	18.212
14824	325	52	39	C	S	58.641	-3.292	2.719	-1.408	3.523	-2.798	1.449	84.113	18.221	19.557
14825	325	52	39	C	S	61.359	-3.292	-2.719	-1.408	3.523	2.798	1.449	84.113	18.221	19.557
14826	325	52	39	C	S	64.715	-1.702	3.009	-3.007	1.762	-3.201	3.200	84.062	19.567	18.212
14827	325	52	39	C	S	115.285	-1.702	-3.009	-3.007	1.762	3.201	3.200	84.062	19.567	18.212
14828	325	52	39	C	S	118.641	-3.292	2.719	-1.408	3.523	-2.798	1.449	84.113	18.221	19.557
14829	325	52	39	C	W	1.359	-3.292	-2.719	-1.408	3.523	2.798	1.449	84.113	18.221	19.557
14830	325	52	39	C	W	4.715	-1.702	3.009	-3.007	1.762	-3.201	3.200	84.062	19.567	18.212
14831	325	52	39	C	W	55.285	-1.702	-3.009	-3.007	1.762	3.201	3.200	84.062	19.567	18.212
14832	325	52	39	C	W	58.641	-3.292	2.719	-1.408	3.523	-2.798	1.449	84.113	18.221	19.557
14833	325	52	39	C	W	61.359	-3.292	-2.719	-1.408	3.523	2.798	1.449	84.113	18.221	19.557
14834	325	52	39	C	W	64.715	-1.702	3.009	-3.007	1.762	-3.201	3.200	84.062	19.567	18.212
14835	325	52	39	C	W	115.285	-1.702	-3.009	-3.007	1.762	3.201	3.200	84.062	19.567	18.212
14836	325	52	39	C	W	118.641	-3.292	2.719	-1.408	3.523	-2.798	1.449	84.113	18.221	19.557
14837	325	52	39	P	S	1.359	-3.292	-2.719	-1.408	3.523	2.798	1.449	84.113	18.221	19.557
14838	325	52	39	P	S	4.715	-1.702	3.009	-3.007	1.762	-3.201	3.200	84.062	19.567	18.212
14839	325	52	39	P	S	55.285	-1.702	-3.009	-3.007	1.762	3.201	3.200	84.062	19.567	18.212
14840	325	52	39	P	S	58.641	-3.292	2.719	-1.408	3.523	-2.798	1.449	84.113	18.221	19.557
14841	325	52	39	P	S	61.359	-3.292	-2.719	-1.408	3.523	2.798	1.449	84.113	18.221	19.557

BL number	Atoms	γ-PC unit cells	WS_2 unit cells	γ-PC origin atom	WS_2 origin atom	Twist-angle (°)	γ-PC strain 1 (%)	γ-PC strain 2 (%)	γ-PC strain 3 (%)	WS_2 strain 1 (%)	WS_2 strain 2 (%)	WS_2 strain 3 (%)	γ (°)	|**a**|(Å)	|**b**|(Å)
14842	325	52	39	P	S	64.715	-1.702	3.009	-3.007	1.762	-3.201	3.200	84.062	19.567	18.212
14843	325	52	39	P	S	115.285	-1.702	-3.009	-3.007	1.762	3.201	3.200	84.062	19.567	18.212
14844	325	52	39	P	S	118.641	-3.292	2.719	-1.408	3.523	-2.798	1.449	84.113	18.221	19.557
14845	325	52	39	P	W	1.359	-3.292	-2.719	-1.408	3.523	2.798	1.449	84.113	18.221	19.557
14846	325	52	39	P	W	4.715	-1.702	3.009	-3.007	1.762	-3.201	3.200	84.062	19.567	18.212
14847	325	52	39	P	W	55.285	-1.702	-3.009	-3.007	1.762	3.201	3.200	84.062	19.567	18.212
14848	325	52	39	P	W	58.641	-3.292	2.719	-1.408	3.523	-2.798	1.449	84.113	18.221	19.557
14849	325	52	39	P	W	61.359	-3.292	-2.719	-1.408	3.523	2.798	1.449	84.113	18.221	19.557
14850	325	52	39	P	W	64.715	-1.702	3.009	-3.007	1.762	-3.201	3.200	84.062	19.567	18.212
14851	325	52	39	P	W	115.285	-1.702	-3.009	-3.007	1.762	3.201	3.200	84.062	19.567	18.212
14852	325	52	39	P	W	118.641	-3.292	2.719	-1.408	3.523	-2.798	1.449	84.113	18.221	19.557
14853	328	52	40	C	S	13.898	-0.414	-2.552	-3.054	0.417	2.718	3.253	83.692	19.824	18.215
14854	328	52	40	C	S	16.537	-3.292	1.974	-0.162	3.523	-1.981	0.163	83.763	18.221	19.817
14855	328	52	40	C	S	43.463	-3.292	-1.974	-0.162	3.523	1.981	0.163	83.763	18.221	19.817
14856	328	52	40	C	S	46.102	-0.414	2.552	-3.054	0.417	-2.718	3.253	83.692	19.824	18.215
14857	328	52	40	C	S	73.898	-0.414	-2.552	-3.054	0.417	2.718	3.253	83.692	19.824	18.215
14858	328	52	40	C	S	76.537	-3.292	1.974	-0.162	3.523	-1.981	0.163	83.763	18.221	19.817
14859	328	52	40	C	S	103.463	-3.292	-1.974	-0.162	3.523	1.981	0.163	83.763	18.221	19.817
14860	328	52	40	C	S	106.102	-0.414	2.552	-3.054	0.417	-2.718	3.253	83.692	19.824	18.215
14861	328	52	40	C	W	13.898	-0.414	-2.552	-3.054	0.417	2.718	3.253	83.692	19.824	18.215
14862	328	52	40	C	W	16.537	-3.292	1.974	-0.162	3.523	-1.981	0.163	83.763	18.221	19.817
14863	328	52	40	C	W	43.463	-3.292	-1.974	-0.162	3.523	1.981	0.163	83.763	18.221	19.817
14864	328	52	40	C	W	46.102	-0.414	2.552	-3.054	0.417	-2.718	3.253	83.692	19.824	18.215
14865	328	52	40	C	W	73.898	-0.414	-2.552	-3.054	0.417	2.718	3.253	83.692	19.824	18.215
14866	328	52	40	C	W	76.537	-3.292	1.974	-0.162	3.523	-1.981	0.163	83.763	18.221	19.817
14867	328	52	40	C	W	103.463	-3.292	-1.974	-0.162	3.523	1.981	0.163	83.763	18.221	19.817
14868	328	52	40	C	W	106.102	-0.414	2.552	-3.054	0.417	-2.718	3.253	83.692	19.824	18.215
14869	328	52	40	P	S	43.463	-3.292	-1.974	-0.162	3.523	1.981	0.163	83.763	18.221	19.817
14870	328	52	40	P	S	46.102	-0.414	2.552	-3.054	0.417	-2.718	3.253	83.692	19.824	18.215
14871	328	52	40	P	S	73.898	-0.414	-2.552	-3.054	0.417	2.718	3.253	83.692	19.824	18.215
14872	328	52	40	P	S	76.537	-3.292	1.974	-0.162	3.523	-1.981	0.163	83.763	18.221	19.817
14873	328	52	40	P	W	13.898	-0.414	-2.552	-3.054	0.417	2.718	3.253	83.692	19.824	18.215
14874	328	52	40	P	W	16.537	-3.292	1.974	-0.162	3.523	-1.981	0.163	83.763	18.221	19.817
14875	328	52	40	P	W	103.463	-3.292	-1.974	-0.162	3.523	1.981	0.163	83.763	18.221	19.817
14876	328	52	40	P	W	106.102	-0.414	2.552	-3.054	0.417	-2.718	3.253	83.692	19.824	18.215
14877	334	52	42	C	S	13.898	-4.228	0.000	3.401	4.619	0.000	-3.184	90.000	17.184	21.423
14878	334	52	42	C	S	46.102	3.400	0.000	-4.228	-3.184	0.000	4.619	90.000	21.423	17.184
14879	334	52	42	C	S	73.898	-4.228	0.000	3.401	4.619	0.000	-3.184	90.000	17.184	21.423
14880	334	52	42	C	S	106.102	3.400	0.000	-4.228	-3.184	0.000	4.619	90.000	21.423	17.184
14881	334	52	42	C	W	13.898	-4.228	0.000	3.401	4.619	0.000	-3.184	90.000	17.184	21.423
14882	334	52	42	C	W	46.102	3.400	0.000	-4.228	-3.184	0.000	4.619	90.000	21.423	17.184
14883	334	52	42	C	W	73.898	-4.228	0.000	3.401	4.619	0.000	-3.184	90.000	17.184	21.423
14884	334	52	42	C	W	106.102	3.400	0.000	-4.228	-3.184	0.000	4.619	90.000	21.423	17.184
14885	334	52	42	C	S	4.715	-1.702	-2.301	0.607	1.762	2.273	-0.600	83.875	19.567	18.897
14886	334	52	42	C	S	7.589	0.334	2.692	-1.440	-0.332	-2.772	1.483	83.818	18.904	19.561
14887	334	52	42	C	S	52.411	0.334	-2.692	-1.440	-0.332	2.772	1.483	83.818	18.904	19.561
14888	334	52	42	C	S	55.285	-1.702	2.301	0.607	1.762	-2.273	-0.600	83.875	19.567	18.897
14889	334	52	42	C	S	64.715	-1.702	-2.301	0.607	1.762	2.273	-0.600	83.875	19.567	18.897
14890	334	52	42	C	S	67.589	0.334	2.692	-1.440	-0.332	-2.772	1.483	83.818	18.904	19.561
14891	334	52	42	C	S	112.411	0.334	-2.692	-1.440	-0.332	2.772	1.483	83.818	18.904	19.561
14892	334	52	42	C	S	115.285	-1.702	2.301	0.607	1.762	-2.273	-0.600	83.875	19.567	18.897

BL number	Atoms	γ-PC unit cells	WS_2 unit cells	γ-PC origin atom	WS_2 origin atom	Twist-angle (°)	γ-PC strain 1 (%)	γ-PC strain 2 (%)	γ-PC strain 3 (%)	WS_2 strain 1 (%)	WS_2 strain 2 (%)	WS_2 strain 3 (%)	γ (°)	|**a**|(Å)	|**b**|(Å)
14893	334	52	42	C	W	4.715	-1.702	-2.301	0.607	1.762	2.273	-0.600	83.875	19.567	18.897
14894	334	52	42	C	W	7.589	0.334	2.692	-1.440	-0.332	-2.772	1.483	83.818	18.904	19.561
14895	334	52	42	C	W	52.411	0.334	-2.692	-1.440	-0.332	2.772	1.483	83.818	18.904	19.561
14896	334	52	42	C	W	55.285	-1.702	2.301	0.607	1.762	-2.273	-0.600	83.875	19.567	18.897
14897	334	52	42	C	W	64.715	-1.702	-2.301	0.607	1.762	2.273	-0.600	83.875	19.567	18.897
14898	334	52	42	C	W	67.589	0.334	2.692	-1.440	-0.332	-2.772	1.483	83.818	18.904	19.561
14899	334	52	42	C	W	112.411	0.334	-2.692	-1.440	-0.332	2.772	1.483	83.818	18.904	19.561
14900	334	52	42	C	W	115.285	-1.702	2.301	0.607	1.762	-2.273	-0.600	83.875	19.567	18.897
14901	334	52	42	P	S	4.715	-1.702	-2.301	0.607	1.762	2.273	-0.600	83.875	19.567	18.897
14902	334	52	42	P	S	7.589	0.334	2.692	-1.440	-0.332	-2.772	1.483	83.818	18.904	19.561
14903	334	52	42	P	S	52.411	0.334	-2.692	-1.440	-0.332	2.772	1.483	83.818	18.904	19.561
14904	334	52	42	P	S	55.285	-1.702	2.301	0.607	1.762	-2.273	-0.600	83.875	19.567	18.897
14905	334	52	42	P	S	64.715	-1.702	-2.301	0.607	1.762	2.273	-0.600	83.875	19.567	18.897
14906	334	52	42	P	S	67.589	0.334	2.692	-1.440	-0.332	-2.772	1.483	83.818	18.904	19.561
14907	334	52	42	P	S	112.411	0.334	-2.692	-1.440	-0.332	2.772	1.483	83.818	18.904	19.561
14908	334	52	42	P	S	115.285	-1.702	2.301	0.607	1.762	-2.273	-0.600	83.875	19.567	18.897
14909	334	52	42	P	W	4.715	-1.702	-2.301	0.607	1.762	2.273	-0.600	83.875	19.567	18.897
14910	334	52	42	P	W	7.589	0.334	2.692	-1.440	-0.332	-2.772	1.483	83.818	18.904	19.561
14911	334	52	42	P	W	52.411	0.334	-2.692	-1.440	-0.332	2.772	1.483	83.818	18.904	19.561
14912	334	52	42	P	W	55.285	-1.702	2.301	0.607	1.762	-2.273	-0.600	83.875	19.567	18.897
14913	334	52	42	P	W	64.715	-1.702	-2.301	0.607	1.762	2.273	-0.600	83.875	19.567	18.897
14914	334	52	42	P	W	67.589	0.334	2.692	-1.440	-0.332	-2.772	1.483	83.818	18.904	19.561
14915	334	52	42	P	W	112.411	0.334	-2.692	-1.440	-0.332	2.772	1.483	83.818	18.904	19.561
14916	334	52	42	P	W	115.285	-1.702	2.301	0.607	1.762	-2.273	-0.600	83.875	19.567	18.897
14917	337	52	43	C	S	6.309	0.024	0.000	0.024	-0.024	0.000	-0.024	60.000	20.724	20.724
14918	337	52	43	C	S	21.487	0.024	0.000	0.024	-0.024	0.000	-0.024	60.000	20.724	20.724
14919	337	52	43	C	S	38.513	0.024	0.000	0.024	-0.024	0.000	-0.024	60.000	20.724	20.724
14920	337	52	43	C	S	53.691	0.024	0.000	0.024	-0.024	0.000	-0.024	60.000	20.724	20.724
14921	337	52	43	C	S	66.309	0.024	0.000	0.024	-0.024	0.000	-0.024	60.000	20.724	20.724
14922	337	52	43	C	S	81.487	0.024	0.000	0.024	-0.024	0.000	-0.024	60.000	20.724	20.724
14923	337	52	43	C	S	98.513	0.024	0.000	0.024	-0.024	0.000	-0.024	60.000	20.724	20.724
14924	337	52	43	C	S	113.691	0.024	0.000	0.024	-0.024	0.000	-0.024	60.000	20.724	20.724
14925	337	52	43	C	W	6.309	0.024	0.000	0.024	-0.024	0.000	-0.024	60.000	20.724	20.724
14926	337	52	43	C	W	21.487	0.024	0.000	0.024	-0.024	0.000	-0.024	60.000	20.724	20.724
14927	337	52	43	C	W	38.513	0.024	0.000	0.024	-0.024	0.000	-0.024	60.000	20.724	20.724
14928	337	52	43	C	W	53.691	0.024	0.000	0.024	-0.024	0.000	-0.024	60.000	20.724	20.724
14929	337	52	43	C	W	66.309	0.024	0.000	0.024	-0.024	0.000	-0.024	60.000	20.724	20.724
14930	337	52	43	C	W	81.487	0.024	0.000	0.024	-0.024	0.000	-0.024	60.000	20.724	20.724
14931	337	52	43	C	W	98.513	0.024	0.000	0.024	-0.024	0.000	-0.024	60.000	20.724	20.724
14932	337	52	43	C	W	113.691	0.024	0.000	0.024	-0.024	0.000	-0.024	60.000	20.724	20.724
14933	337	52	43	P	S	38.513	0.024	0.000	0.024	-0.024	0.000	-0.024	60.000	20.724	20.724
14934	337	52	43	P	S	53.691	0.024	0.000	0.024	-0.024	0.000	-0.024	60.000	20.724	20.724
14935	337	52	43	P	S	66.309	0.024	0.000	0.024	-0.024	0.000	-0.024	60.000	20.724	20.724
14936	337	52	43	P	S	81.487	0.024	0.000	0.024	-0.024	0.000	-0.024	60.000	20.724	20.724
14937	337	52	43	P	W	6.309	0.024	0.000	0.024	-0.024	0.000	-0.024	60.000	20.724	20.724
14938	337	52	43	P	W	21.487	0.024	0.000	0.024	-0.024	0.000	-0.024	60.000	20.724	20.724
14939	337	52	43	P	W	98.513	0.024	0.000	0.024	-0.024	0.000	-0.024	60.000	20.724	20.724
14940	337	52	43	P	W	113.691	0.024	0.000	0.024	-0.024	0.000	-0.024	60.000	20.724	20.724
14941	337	52	43	C	S	4.715	-1.702	-4.071	1.812	1.762	3.928	-1.749	80.429	19.567	19.283
14942	337	52	43	C	S	8.513	2.390	2.580	-2.234	-2.281	-2.700	2.339	80.579	19.291	19.554
14943	337	52	43	C	S	13.898	-0.414	-3.572	0.466	0.417	3.539	-0.462	80.484	19.824	19.025

BL number	Atoms	γ-PC unit cells	WS_2 unit cells	γ-PC origin atom	WS_2 origin atom	Twist-angle (°)	γ-PC strain 1 (%)	γ-PC strain 2 (%)	γ-PC strain 3 (%)	WS_2 strain 1 (%)	WS_2 strain 2 (%)	WS_2 strain 3 (%)	γ (°)	|**a**|(Å)	|**b**|(Å)
14944	337	52	43	C	S	17.696	1.029	3.047	-0.960	-1.008	-3.106	0.979	80.537	19.034	19.812
14945	337	52	43	C	S	42.304	1.029	-3.047	-0.960	-1.008	3.106	0.979	80.537	19.034	19.812
14946	337	52	43	C	S	46.102	-0.414	3.572	0.466	0.417	-3.539	-0.462	80.484	19.824	19.025
14947	337	52	43	C	S	51.487	2.390	-2.580	-2.234	-2.281	2.700	2.339	80.579	19.291	19.554
14948	337	52	43	C	S	55.285	-1.702	4.071	1.812	1.762	-3.928	-1.749	80.429	19.567	19.283
14949	337	52	43	C	S	64.715	-1.702	-4.071	1.812	1.762	3.928	-1.749	80.429	19.567	19.283
14950	337	52	43	C	S	68.513	2.390	2.580	-2.234	-2.281	-2.700	2.339	80.579	19.291	19.554
14951	337	52	43	C	S	73.898	-0.414	-3.572	0.466	0.417	3.539	-0.462	80.484	19.824	19.025
14952	337	52	43	C	S	77.696	1.029	3.047	-0.960	-1.008	-3.106	0.979	80.537	19.034	19.812
14953	337	52	43	C	S	102.304	1.029	-3.047	-0.960	-1.008	3.106	0.979	80.537	19.034	19.812
14954	337	52	43	C	S	106.102	-0.414	3.572	0.466	0.417	-3.539	-0.462	80.484	19.824	19.025
14955	337	52	43	C	S	111.487	2.390	-2.580	-2.234	-2.281	2.700	2.339	80.579	19.291	19.554
14956	337	52	43	C	S	115.285	-1.702	4.071	1.812	1.762	-3.928	-1.749	80.429	19.567	19.283
14957	337	52	43	C	W	4.715	-1.702	-4.071	1.812	1.762	3.928	-1.749	80.429	19.567	19.283
14958	337	52	43	C	W	8.513	2.390	2.580	-2.234	-2.281	-2.700	2.339	80.579	19.291	19.554
14959	337	52	43	C	W	13.898	-0.414	-3.572	0.466	0.417	3.539	-0.462	80.484	19.824	19.025
14960	337	52	43	C	W	17.696	1.029	3.047	-0.960	-1.008	-3.106	0.979	80.537	19.034	19.812
14961	337	52	43	C	W	42.304	1.029	-3.047	-0.960	-1.008	3.106	0.979	80.537	19.034	19.812
14962	337	52	43	C	W	46.102	-0.414	3.572	0.466	0.417	-3.539	-0.462	80.484	19.824	19.025
14963	337	52	43	C	W	51.487	2.390	-2.580	-2.234	-2.281	2.700	2.339	80.579	19.291	19.554
14964	337	52	43	C	W	55.285	-1.702	4.071	1.812	1.762	-3.928	-1.749	80.429	19.567	19.283
14965	337	52	43	C	W	64.715	-1.702	-4.071	1.812	1.762	3.928	-1.749	80.429	19.567	19.283
14966	337	52	43	C	W	68.513	2.390	2.580	-2.234	-2.281	-2.700	2.339	80.579	19.291	19.554
14967	337	52	43	C	W	73.898	-0.414	-3.572	0.466	0.417	3.539	-0.462	80.484	19.824	19.025
14968	337	52	43	C	W	77.696	1.029	3.047	-0.960	-1.008	-3.106	0.979	80.537	19.034	19.812
14969	337	52	43	C	W	102.304	1.029	-3.047	-0.960	-1.008	3.106	0.979	80.537	19.034	19.812
14970	337	52	43	C	W	106.102	-0.414	3.572	0.466	0.417	-3.539	-0.462	80.484	19.824	19.025
14971	337	52	43	C	W	111.487	2.390	-2.580	-2.234	-2.281	2.700	2.339	80.579	19.291	19.554
14972	337	52	43	C	W	115.285	-1.702	4.071	1.812	1.762	-3.928	-1.749	80.429	19.567	19.283
14973	337	52	43	P	S	4.715	-1.702	-4.071	1.812	1.762	3.928	-1.749	80.429	19.567	19.283
14974	337	52	43	P	S	8.513	2.390	2.580	-2.234	-2.281	-2.700	2.339	80.579	19.291	19.554
14975	337	52	43	P	S	42.304	1.029	-3.047	-0.960	-1.008	3.106	0.979	80.537	19.034	19.812
14976	337	52	43	P	S	46.102	-0.414	3.572	0.466	0.417	-3.539	-0.462	80.484	19.824	19.025
14977	337	52	43	P	S	51.487	2.390	-2.580	-2.234	-2.281	2.700	2.339	80.579	19.291	19.554
14978	337	52	43	P	S	55.285	-1.702	4.071	1.812	1.762	-3.928	-1.749	80.429	19.567	19.283
14979	337	52	43	P	S	64.715	-1.702	-4.071	1.812	1.762	3.928	-1.749	80.429	19.567	19.283
14980	337	52	43	P	S	68.513	2.390	2.580	-2.234	-2.281	-2.700	2.339	80.579	19.291	19.554
14981	337	52	43	P	S	73.898	-0.414	-3.572	0.466	0.417	3.539	-0.462	80.484	19.824	19.025
14982	337	52	43	P	S	77.696	1.029	3.047	-0.960	-1.008	-3.106	0.979	80.537	19.034	19.812
14983	337	52	43	P	S	111.487	2.390	-2.580	-2.234	-2.281	2.700	2.339	80.579	19.291	19.554
14984	337	52	43	P	S	115.285	-1.702	4.071	1.812	1.762	-3.928	-1.749	80.429	19.567	19.283
14985	337	52	43	P	W	4.715	-1.702	-4.071	1.812	1.762	3.928	-1.749	80.429	19.567	19.283
14986	337	52	43	P	W	8.513	2.390	2.580	-2.234	-2.281	-2.700	2.339	80.579	19.291	19.554
14987	337	52	43	P	W	13.898	-0.414	-3.572	0.466	0.417	3.539	-0.462	80.484	19.824	19.025
14988	337	52	43	P	W	17.696	1.029	3.047	-0.960	-1.008	-3.106	0.979	80.537	19.034	19.812
14989	337	52	43	P	W	51.487	2.390	-2.580	-2.234	-2.281	2.700	2.339	80.579	19.291	19.554
14990	337	52	43	P	W	55.285	-1.702	4.071	1.812	1.762	-3.928	-1.749	80.429	19.567	19.283
14991	337	52	43	P	W	64.715	-1.702	-4.071	1.812	1.762	3.928	-1.749	80.429	19.567	19.283
14992	337	52	43	P	W	68.513	2.390	2.580	-2.234	-2.281	-2.700	2.339	80.579	19.291	19.554
14993	337	52	43	P	W	102.304	1.029	-3.047	-0.960	-1.008	3.106	0.979	80.537	19.034	19.812
14994	337	52	43	P	W	106.102	-0.414	3.572	0.466	0.417	-3.539	-0.462	80.484	19.824	19.025

BL number	Atoms	γ-PC unit cells	WS_2 unit cells	γ-PC origin atom	WS_2 origin atom	Twist-angle (°)	γ-PC strain 1 (%)	γ-PC strain 2 (%)	γ-PC strain 3 (%)	WS_2 strain 1 (%)	WS_2 strain 2 (%)	WS_2 strain 3 (%)	γ (°)	|**a**|(Å)	|**b**|(Å)
14995	337	52	43	P	W	111.487	2.390	-2.580	-2.234	-2.281	2.700	2.339	80.579	19.291	19.554
14996	337	52	43	P	W	115.285	-1.702	4.071	1.812	1.762	-3.928	-1.749	80.429	19.567	19.283
14997	340	49	48	C	S	8.213	4.447	0.000	4.447	-4.084	0.000	-4.084	60.000	21.007	21.007
14998	340	49	48	C	S	30.000	4.447	0.000	4.447	-4.084	0.000	-4.084	60.000	21.007	21.007
14999	340	49	48	C	S	51.787	4.447	0.000	4.447	-4.084	0.000	-4.084	60.000	21.007	21.007
15000	340	49	48	C	S	68.213	4.447	0.000	4.447	-4.084	0.000	-4.084	60.000	21.007	21.007
15001	340	49	48	C	S	90.000	4.447	0.000	4.447	-4.084	0.000	-4.084	60.000	21.007	21.007
15002	340	49	48	C	S	111.787	4.447	0.000	4.447	-4.084	0.000	-4.084	60.000	21.007	21.007
15003	340	49	48	C	W	8.213	4.447	0.000	4.447	-4.084	0.000	-4.084	60.000	21.007	21.007
15004	340	49	48	C	W	30.000	4.447	0.000	4.447	-4.084	0.000	-4.084	60.000	21.007	21.007
15005	340	49	48	C	W	51.787	4.447	0.000	4.447	-4.084	0.000	-4.084	60.000	21.007	21.007
15006	340	49	48	C	W	68.213	4.447	0.000	4.447	-4.084	0.000	-4.084	60.000	21.007	21.007
15007	340	49	48	C	W	90.000	4.447	0.000	4.447	-4.084	0.000	-4.084	60.000	21.007	21.007
15008	340	49	48	C	W	111.787	4.447	0.000	4.447	-4.084	0.000	-4.084	60.000	21.007	21.007
15009	343	49	49	C	S	16.426	5.011	0.000	5.011	-4.554	0.000	-4.555	60.000	21.120	21.120
15010	343	49	49	C	S	43.574	5.011	0.000	5.011	-4.554	0.000	-4.555	60.000	21.120	21.120
15011	343	49	49	C	S	76.427	5.011	0.000	5.011	-4.554	0.000	-4.555	60.000	21.120	21.120
15012	343	49	49	C	S	103.574	5.011	0.000	5.011	-4.554	0.000	-4.555	60.000	21.120	21.120
15013	343	49	49	C	W	16.426	5.011	0.000	5.011	-4.554	0.000	-4.555	60.000	21.120	21.120
15014	343	49	49	C	W	43.574	5.011	0.000	5.011	-4.554	0.000	-4.555	60.000	21.120	21.120
15015	343	49	49	C	W	76.427	5.011	0.000	5.011	-4.554	0.000	-4.555	60.000	21.120	21.120
15016	343	49	49	C	W	103.574	5.011	0.000	5.011	-4.554	0.000	-4.555	60.000	21.120	21.120
15017	343	49	49	P	S	43.574	5.011	0.000	5.011	-4.554	0.000	-4.555	60.000	21.120	21.120
15018	343	49	49	P	S	76.427	5.011	0.000	5.011	-4.554	0.000	-4.555	60.000	21.120	21.120
15019	343	49	49	P	W	16.426	5.011	0.000	5.011	-4.554	0.000	-4.555	60.000	21.120	21.120
15020	343	49	49	P	W	103.574	5.011	0.000	5.011	-4.554	0.000	-4.555	60.000	21.120	21.120
15021	343	52	45	C	S	8.513	2.390	-4.941	-0.013	-2.281	4.942	0.013	85.027	19.291	19.804
15022	343	52	45	C	S	13.898	-0.414	4.593	2.814	0.417	-4.348	-2.664	85.177	19.824	19.269
15023	343	52	45	C	S	46.102	-0.414	-4.593	2.814	0.417	4.348	-2.664	85.177	19.824	19.269
15024	343	52	45	C	S	51.487	2.390	4.941	-0.013	-2.281	-4.942	0.013	85.027	19.291	19.804
15025	343	52	45	C	S	68.513	2.390	-4.941	-0.013	-2.281	4.942	0.013	85.027	19.291	19.804
15026	343	52	45	C	S	73.898	-0.414	4.593	2.814	0.417	-4.348	-2.664	85.177	19.824	19.269
15027	343	52	45	C	S	106.102	-0.414	-4.593	2.814	0.417	4.348	-2.664	85.177	19.824	19.269
15028	343	52	45	C	S	111.487	2.390	4.941	-0.013	-2.281	-4.942	0.013	85.027	19.291	19.804
15029	343	52	45	C	W	8.513	2.390	-4.941	-0.013	-2.281	4.942	0.013	85.027	19.291	19.804
15030	343	52	45	C	W	13.898	-0.414	4.593	2.814	0.417	-4.348	-2.664	85.177	19.824	19.269
15031	343	52	45	C	W	46.102	-0.414	-4.593	2.814	0.417	4.348	-2.664	85.177	19.824	19.269
15032	343	52	45	C	W	51.487	2.390	4.941	-0.013	-2.281	-4.942	0.013	85.027	19.291	19.804
15033	343	52	45	C	W	68.513	2.390	-4.941	-0.013	-2.281	4.942	0.013	85.027	19.291	19.804
15034	343	52	45	C	W	73.898	-0.414	4.593	2.814	0.417	-4.348	-2.664	85.177	19.824	19.269
15035	343	52	45	C	W	106.102	-0.414	-4.593	2.814	0.417	4.348	-2.664	85.177	19.824	19.269
15036	343	52	45	C	W	111.487	2.390	4.941	-0.013	-2.281	-4.942	0.013	85.027	19.291	19.804
15037	343	52	45	P	S	46.102	-0.414	-4.593	2.814	0.417	4.348	-2.664	85.177	19.824	19.269
15038	343	52	45	P	S	51.487	2.390	4.941	-0.013	-2.281	-4.942	0.013	85.027	19.291	19.804
15039	343	52	45	P	S	68.513	2.390	-4.941	-0.013	-2.281	4.942	0.013	85.027	19.291	19.804
15040	343	52	45	P	S	73.898	-0.414	4.593	2.814	0.417	-4.348	-2.664	85.177	19.824	19.269
15041	343	52	45	P	W	8.513	2.390	-4.941	-0.013	-2.281	4.942	0.013	85.027	19.291	19.804
15042	343	52	45	P	W	13.898	-0.414	4.593	2.814	0.417	-4.348	-2.664	85.177	19.824	19.269
15043	343	52	45	P	W	46.102	-0.414	-4.593	2.814	0.417	4.348	-2.664	85.177	19.824	19.269
15044	343	52	45	P	W	73.898	-0.414	4.593	2.814	0.417	-4.348	-2.664	85.177	19.824	19.269
15045	343	52	45	P	W	106.102	-0.414	-4.593	2.814	0.417	4.348	-2.664	85.177	19.824	19.269

BL number	Atoms	γ-PC unit cells	WS_2 unit cells	γ-PC origin atom	WS_2 origin atom	Twist-angle (°)	γ-PC strain 1 (%)	γ-PC strain 2 (%)	γ-PC strain 3 (%)	WS_2 strain 1 (%)	WS_2 strain 2 (%)	WS_2 strain 3 (%)	γ (°)	$\lvert\mathbf{a}\rvert$(Å)	$\lvert\mathbf{b}\rvert$(Å)
15046	343	52	45	P	W	111.487	2.390	4.941	-0.013	-2.281	-4.942	0.013	85.027	19.291	19.804
15047	355	52	49	C	S	7.889	3.400	0.000	3.401	-3.184	0.000	-3.184	60.000	21.423	21.423
15048	355	52	49	C	S	13.898	3.400	0.000	3.401	-3.184	0.000	-3.184	60.000	21.423	21.423
15049	355	52	49	C	S	24.315	3.400	0.000	3.401	-3.184	0.000	-3.184	60.000	21.423	21.423
15050	355	52	49	C	S	35.685	3.400	0.000	3.401	-3.184	0.000	-3.184	60.000	21.423	21.423
15051	355	52	49	C	S	46.102	3.400	0.000	3.401	-3.184	0.000	-3.184	60.000	21.423	21.423
15052	355	52	49	C	S	52.111	3.400	0.000	3.401	-3.184	0.000	-3.184	60.000	21.423	21.423
15053	355	52	49	C	S	67.889	3.400	0.000	3.401	-3.184	0.000	-3.184	60.000	21.423	21.423
15054	355	52	49	C	S	73.898	3.400	0.000	3.401	-3.184	0.000	-3.184	60.000	21.423	21.423
15055	355	52	49	C	S	84.315	3.400	0.000	3.401	-3.184	0.000	-3.184	60.000	21.423	21.423
15056	355	52	49	C	S	95.685	3.400	0.000	3.401	-3.184	0.000	-3.184	60.000	21.423	21.423
15057	355	52	49	C	S	106.102	3.400	0.000	3.401	-3.184	0.000	-3.184	60.000	21.423	21.423
15058	355	52	49	C	S	112.111	3.400	0.000	3.401	-3.184	0.000	-3.184	60.000	21.423	21.423
15059	355	52	49	C	W	7.889	3.400	0.000	3.401	-3.184	0.000	-3.184	60.000	21.423	21.423
15060	355	52	49	C	W	13.898	3.400	0.000	3.401	-3.184	0.000	-3.184	60.000	21.423	21.423
15061	355	52	49	C	W	24.315	3.400	0.000	3.401	-3.184	0.000	-3.184	60.000	21.423	21.423
15062	355	52	49	C	W	35.685	3.400	0.000	3.401	-3.184	0.000	-3.184	60.000	21.423	21.423
15063	355	52	49	C	W	46.102	3.400	0.000	3.401	-3.184	0.000	-3.184	60.000	21.423	21.423
15064	355	52	49	C	W	52.111	3.400	0.000	3.401	-3.184	0.000	-3.184	60.000	21.423	21.423
15065	355	52	49	C	W	67.889	3.400	0.000	3.401	-3.184	0.000	-3.184	60.000	21.423	21.423
15066	355	52	49	C	W	73.898	3.400	0.000	3.401	-3.184	0.000	-3.184	60.000	21.423	21.423
15067	355	52	49	C	W	84.315	3.400	0.000	3.401	-3.184	0.000	-3.184	60.000	21.423	21.423
15068	355	52	49	C	W	95.685	3.400	0.000	3.401	-3.184	0.000	-3.184	60.000	21.423	21.423
15069	355	52	49	C	W	106.102	3.400	0.000	3.401	-3.184	0.000	-3.184	60.000	21.423	21.423
15070	355	52	49	C	W	112.111	3.400	0.000	3.401	-3.184	0.000	-3.184	60.000	21.423	21.423
15071	355	52	49	P	S	13.898	3.400	0.000	3.401	-3.184	0.000	-3.184	60.000	21.423	21.423
15072	355	52	49	P	S	24.315	3.400	0.000	3.401	-3.184	0.000	-3.184	60.000	21.423	21.423
15073	355	52	49	P	S	52.111	3.400	0.000	3.401	-3.184	0.000	-3.184	60.000	21.423	21.423
15074	355	52	49	P	S	67.889	3.400	0.000	3.401	-3.184	0.000	-3.184	60.000	21.423	21.423
15075	355	52	49	P	S	95.685	3.400	0.000	3.401	-3.184	0.000	-3.184	60.000	21.423	21.423
15076	355	52	49	P	S	106.102	3.400	0.000	3.401	-3.184	0.000	-3.184	60.000	21.423	21.423
15077	355	52	49	P	W	7.889	3.400	0.000	3.401	-3.184	0.000	-3.184	60.000	21.423	21.423
15078	355	52	49	P	W	35.685	3.400	0.000	3.401	-3.184	0.000	-3.184	60.000	21.423	21.423
15079	355	52	49	P	W	46.102	3.400	0.000	3.401	-3.184	0.000	-3.184	60.000	21.423	21.423
15080	355	52	49	P	W	73.898	3.400	0.000	3.401	-3.184	0.000	-3.184	60.000	21.423	21.423
15081	355	52	49	P	W	84.315	3.400	0.000	3.401	-3.184	0.000	-3.184	60.000	21.423	21.423
15082	355	52	49	P	W	112.111	3.400	0.000	3.401	-3.184	0.000	-3.184	60.000	21.423	21.423
15083	357	57	43	C	S	1.002	-2.220	0.000	-2.220	2.323	0.000	2.323	60.000	21.210	21.210
15084	357	57	43	C	S	14.176	-2.220	0.000	-2.220	2.323	0.000	2.323	60.000	21.210	21.210
15085	357	57	43	C	S	45.824	-2.220	0.000	-2.220	2.323	0.000	2.323	60.000	21.210	21.210
15086	357	57	43	C	S	58.998	-2.220	0.000	-2.220	2.323	0.000	2.323	60.000	21.210	21.210
15087	357	57	43	C	S	61.002	-2.220	0.000	-2.220	2.323	0.000	2.323	60.000	21.210	21.210
15088	357	57	43	C	S	74.176	-2.220	0.000	-2.220	2.323	0.000	2.323	60.000	21.210	21.210
15089	357	57	43	C	S	105.824	-2.220	0.000	-2.220	2.323	0.000	2.323	60.000	21.210	21.210
15090	357	57	43	C	S	118.998	-2.220	0.000	-2.220	2.323	0.000	2.323	60.000	21.210	21.210
15091	357	57	43	P	S	1.002	-2.220	0.000	-2.220	2.323	0.000	2.323	60.000	21.210	21.210
15092	357	57	43	P	S	14.176	-2.220	0.000	-2.220	2.323	0.000	2.323	60.000	21.210	21.210
15093	357	57	43	P	S	45.824	-2.220	0.000	-2.220	2.323	0.000	2.323	60.000	21.210	21.210
15094	357	57	43	P	S	58.998	-2.220	0.000	-2.220	2.323	0.000	2.323	60.000	21.210	21.210
15095	357	57	43	P	S	61.002	-2.220	0.000	-2.220	2.323	0.000	2.323	60.000	21.210	21.210
15096	357	57	43	P	S	74.176	-2.220	0.000	-2.220	2.323	0.000	2.323	60.000	21.210	21.210

BL number	Atoms	γ-PC unit cells	WS_2 unit cells	γ-PC origin atom	WS_2 origin atom	Twist-angle (°)	γ-PC strain 1 (%)	γ-PC strain 2 (%)	γ-PC strain 3 (%)	WS_2 strain 1 (%)	WS_2 strain 2 (%)	WS_2 strain 3 (%)	γ (°)	|**a**|(Å)	|**b**|(Å)
15097	357	57	43	P	S	105.824	-2.220	0.000	-2.220	2.323	0.000	2.323	60.000	21.210	21.210
15098	357	57	43	P	S	118.998	-2.220	0.000	-2.220	2.323	0.000	2.323	60.000	21.210	21.210
15099	359	56	45	C	S	19.107	1.980	0.000	-3.218	-1.905	0.000	3.439	84.763	19.920	19.920
15100	359	56	45	C	S	40.893	-3.218	0.000	1.980	3.439	0.000	-1.905	84.763	19.920	19.920
15101	359	56	45	C	S	79.107	1.980	0.000	-3.218	-1.905	0.000	3.439	84.763	19.920	19.920
15102	359	56	45	C	S	100.893	-3.218	0.000	1.980	3.439	0.000	-1.905	84.763	19.920	19.920
15103	359	56	45	C	W	19.107	1.980	0.000	-3.218	-1.905	0.000	3.439	84.763	19.920	19.920
15104	359	56	45	C	W	40.893	-3.218	0.000	1.980	3.439	0.000	-1.905	84.763	19.920	19.920
15105	359	56	45	C	W	79.107	1.980	0.000	-3.218	-1.905	0.000	3.439	84.763	19.920	19.920
15106	359	56	45	C	W	100.893	-3.218	0.000	1.980	3.439	0.000	-1.905	84.763	19.920	19.920
15107	359	56	45	P	S	19.107	1.980	0.000	-3.218	-1.905	0.000	3.439	84.763	19.920	19.920
15108	359	56	45	P	S	100.893	-3.218	0.000	1.980	3.439	0.000	-1.905	84.763	19.920	19.920
15109	359	56	45	P	W	40.893	-3.218	0.000	1.980	3.439	0.000	-1.905	84.763	19.920	19.920
15110	359	56	45	P	W	79.107	1.980	0.000	-3.218	-1.905	0.000	3.439	84.763	19.920	19.920
15111	364	55	48	C	S	30.000	0.817	0.000	1.972	-0.804	0.000	-1.897	75.793	20.420	20.420
15112	364	55	48	C	S	90.000	0.817	0.000	1.972	-0.804	0.000	-1.897	75.793	20.420	20.420
15113	364	55	48	C	W	30.000	0.817	0.000	1.972	-0.804	0.000	-1.897	75.793	20.420	20.420
15114	364	55	48	C	W	90.000	0.817	0.000	1.972	-0.804	0.000	-1.897	75.793	20.420	20.420
15115	364	55	48	P	S	30.000	0.817	0.000	1.972	-0.804	0.000	-1.897	75.793	20.420	20.420
15116	364	55	48	P	S	90.000	0.817	0.000	1.972	-0.804	0.000	-1.897	75.793	20.420	20.420
15117	364	55	48	P	W	30.000	0.817	0.000	1.972	-0.804	0.000	-1.897	75.793	20.420	20.420
15118	364	55	48	P	W	90.000	0.817	0.000	1.972	-0.804	0.000	-1.897	75.793	20.420	20.420
15119	373	61	43	C	S	18.740	-3.813	0.000	-3.813	4.128	0.000	4.128	60.000	21.585	21.585
15120	373	61	43	C	S	26.081	-3.813	0.000	-3.813	4.128	0.000	4.128	60.000	21.585	21.585
15121	373	61	43	C	S	33.919	-3.813	0.000	-3.813	4.128	0.000	4.128	60.000	21.585	21.585
15122	373	61	43	C	S	41.260	-3.813	0.000	-3.813	4.128	0.000	4.128	60.000	21.585	21.585
15123	373	61	43	C	S	78.741	-3.813	0.000	-3.813	4.128	0.000	4.128	60.000	21.585	21.585
15124	373	61	43	C	S	86.082	-3.813	0.000	-3.813	4.128	0.000	4.128	60.000	21.585	21.585
15125	373	61	43	C	S	93.919	-3.813	0.000	-3.813	4.128	0.000	4.128	60.000	21.585	21.585
15126	373	61	43	C	S	101.260	-3.813	0.000	-3.813	4.128	0.000	4.128	60.000	21.585	21.585
15127	373	61	43	C	W	18.740	-3.813	0.000	-3.813	4.128	0.000	4.128	60.000	21.585	21.585
15128	373	61	43	C	W	26.081	-3.813	0.000	-3.813	4.128	0.000	4.128	60.000	21.585	21.585
15129	373	61	43	C	W	33.919	-3.813	0.000	-3.813	4.128	0.000	4.128	60.000	21.585	21.585
15130	373	61	43	C	W	41.260	-3.813	0.000	-3.813	4.128	0.000	4.128	60.000	21.585	21.585
15131	373	61	43	C	W	78.741	-3.813	0.000	-3.813	4.128	0.000	4.128	60.000	21.585	21.585
15132	373	61	43	C	W	86.082	-3.813	0.000	-3.813	4.128	0.000	4.128	60.000	21.585	21.585
15133	373	61	43	C	W	93.919	-3.813	0.000	-3.813	4.128	0.000	4.128	60.000	21.585	21.585
15134	373	61	43	C	W	101.260	-3.813	0.000	-3.813	4.128	0.000	4.128	60.000	21.585	21.585
15135	373	61	43	P	S	26.081	-3.813	0.000	-3.813	4.128	0.000	4.128	60.000	21.585	21.585
15136	373	61	43	P	S	41.260	-3.813	0.000	-3.813	4.128	0.000	4.128	60.000	21.585	21.585
15137	373	61	43	P	S	78.741	-3.813	0.000	-3.813	4.128	0.000	4.128	60.000	21.585	21.585
15138	373	61	43	P	S	93.919	-3.813	0.000	-3.813	4.128	0.000	4.128	60.000	21.585	21.585
15139	373	61	43	P	W	18.740	-3.813	0.000	-3.813	4.128	0.000	4.128	60.000	21.585	21.585
15140	373	61	43	P	W	33.919	-3.813	0.000	-3.813	4.128	0.000	4.128	60.000	21.585	21.585
15141	373	61	43	P	W	86.082	-3.813	0.000	-3.813	4.128	0.000	4.128	60.000	21.585	21.585
15142	373	61	43	P	W	101.260	-3.813	0.000	-3.813	4.128	0.000	4.128	60.000	21.585	21.585
15143	375	57	49	C	S	6.587	1.005	0.000	1.005	-0.985	0.000	-0.985	60.000	21.910	21.910
15144	375	57	49	C	S	15.200	1.005	0.000	1.005	-0.985	0.000	-0.985	60.000	21.910	21.910
15145	375	57	49	C	S	28.374	1.005	0.000	1.005	-0.985	0.000	-0.985	60.000	21.910	21.910
15146	375	57	49	C	S	31.626	1.005	0.000	1.005	-0.985	0.000	-0.985	60.000	21.910	21.910
15147	375	57	49	C	S	44.800	1.005	0.000	1.005	-0.985	0.000	-0.985	60.000	21.910	21.910

BL number	Atoms	γ-PC unit cells	WS_2 unit cells	γ-PC origin atom	WS_2 origin atom	Twist-angle (°)	γ-PC strain 1 (%)	γ-PC strain 2 (%)	γ-PC strain 3 (%)	WS_2 strain 1 (%)	WS_2 strain 2 (%)	WS_2 strain 3 (%)	γ (°)	|**a**|(Å)	|**b**|(Å)
15148	375	57	49	C	S	53.413	1.005	0.000	1.005	-0.985	0.000	-0.985	60.000	21.910	21.910
15149	375	57	49	C	S	66.587	1.005	0.000	1.005	-0.985	0.000	-0.985	60.000	21.910	21.910
15150	375	57	49	C	S	75.200	1.005	0.000	1.005	-0.985	0.000	-0.985	60.000	21.910	21.910
15151	375	57	49	C	S	88.374	1.005	0.000	1.005	-0.985	0.000	-0.985	60.000	21.910	21.910
15152	375	57	49	C	S	91.627	1.005	0.000	1.005	-0.985	0.000	-0.985	60.000	21.910	21.910
15153	375	57	49	C	S	104.800	1.005	0.000	1.005	-0.985	0.000	-0.985	60.000	21.910	21.910
15154	375	57	49	C	S	113.413	1.005	0.000	1.005	-0.985	0.000	-0.985	60.000	21.910	21.910
15155	375	57	49	P	S	6.587	1.005	0.000	1.005	-0.985	0.000	-0.985	60.000	21.910	21.910
15156	375	57	49	P	S	15.200	1.005	0.000	1.005	-0.985	0.000	-0.985	60.000	21.910	21.910
15157	375	57	49	P	S	28.374	1.005	0.000	1.005	-0.985	0.000	-0.985	60.000	21.910	21.910
15158	375	57	49	P	S	31.626	1.005	0.000	1.005	-0.985	0.000	-0.985	60.000	21.910	21.910
15159	375	57	49	P	S	44.800	1.005	0.000	1.005	-0.985	0.000	-0.985	60.000	21.910	21.910
15160	375	57	49	P	S	53.413	1.005	0.000	1.005	-0.985	0.000	-0.985	60.000	21.910	21.910
15161	375	57	49	P	S	66.587	1.005	0.000	1.005	-0.985	0.000	-0.985	60.000	21.910	21.910
15162	375	57	49	P	S	75.200	1.005	0.000	1.005	-0.985	0.000	-0.985	60.000	21.910	21.910
15163	375	57	49	P	S	88.374	1.005	0.000	1.005	-0.985	0.000	-0.985	60.000	21.910	21.910
15164	375	57	49	P	S	104.800	1.005	0.000	1.005	-0.985	0.000	-0.985	60.000	21.910	21.910
15165	375	57	49	P	S	113.413	1.005	0.000	1.005	-0.985	0.000	-0.985	60.000	21.910	21.910
15166	375	57	49	P	W	91.627	1.005	0.000	1.005	-0.985	0.000	-0.985	60.000	21.910	21.910
15167	375	60	45	C	S	60.000	-4.158	0.000	-0.490	4.534	0.000	0.495	89.947	20.226	20.226
15168	375	60	45	C	W	0.000	-4.158	0.000	-0.490	4.534	0.000	0.495	89.947	20.226	20.226
15169	381	63	43	C	S	11.518	-4.552	0.000	-4.552	5.008	0.000	5.008	60.000	21.767	21.767
15170	381	63	43	C	S	26.696	-4.552	0.000	-4.552	5.008	0.000	5.008	60.000	21.767	21.767
15171	381	63	43	C	S	33.304	-4.552	0.000	-4.552	5.008	0.000	5.008	60.000	21.767	21.767
15172	381	63	43	C	S	48.483	-4.552	0.000	-4.552	5.008	0.000	5.008	60.000	21.767	21.767
15173	381	63	43	C	S	71.518	-4.552	0.000	-4.552	5.008	0.000	5.008	60.000	21.767	21.767
15174	381	63	43	C	S	86.696	-4.552	0.000	-4.552	5.008	0.000	5.008	60.000	21.767	21.767
15175	381	63	43	C	S	93.304	-4.552	0.000	-4.552	5.008	0.000	5.008	60.000	21.767	21.767
15176	381	63	43	C	S	108.483	-4.552	0.000	-4.552	5.008	0.000	5.008	60.000	21.767	21.767
15177	381	63	43	P	S	11.518	-4.552	0.000	-4.552	5.008	0.000	5.008	60.000	21.767	21.767
15178	381	63	43	P	S	26.696	-4.552	0.000	-4.552	5.008	0.000	5.008	60.000	21.767	21.767
15179	381	63	43	P	S	33.304	-4.552	0.000	-4.552	5.008	0.000	5.008	60.000	21.767	21.767
15180	381	63	43	P	S	48.483	-4.552	0.000	-4.552	5.008	0.000	5.008	60.000	21.767	21.767
15181	381	63	43	P	S	71.518	-4.552	0.000	-4.552	5.008	0.000	5.008	60.000	21.767	21.767
15182	381	63	43	P	S	86.696	-4.552	0.000	-4.552	5.008	0.000	5.008	60.000	21.767	21.767
15183	381	63	43	P	S	93.304	-4.552	0.000	-4.552	5.008	0.000	5.008	60.000	21.767	21.767
15184	381	63	43	P	S	108.483	-4.552	0.000	-4.552	5.008	0.000	5.008	60.000	21.767	21.767
15185	384	57	52	C	S	7.311	2.543	0.000	2.543	-2.420	0.000	-2.420	60.000	22.244	22.244
15186	384	57	52	C	S	20.485	2.543	0.000	2.543	-2.420	0.000	-2.420	60.000	22.244	22.244
15187	384	57	52	C	S	39.515	2.543	0.000	2.543	-2.420	0.000	-2.420	60.000	22.244	22.244
15188	384	57	52	C	S	52.689	2.543	0.000	2.543	-2.420	0.000	-2.420	60.000	22.244	22.244
15189	384	57	52	C	S	67.311	2.543	0.000	2.543	-2.420	0.000	-2.420	60.000	22.244	22.244
15190	384	57	52	C	S	80.485	2.543	0.000	2.543	-2.420	0.000	-2.420	60.000	22.244	22.244
15191	384	57	52	C	S	99.515	2.543	0.000	2.543	-2.420	0.000	-2.420	60.000	22.244	22.244
15192	384	57	52	C	S	112.689	2.543	0.000	2.543	-2.420	0.000	-2.420	60.000	22.244	22.244
15193	384	57	52	P	S	7.311	2.543	0.000	2.543	-2.420	0.000	-2.420	60.000	22.244	22.244
15194	384	57	52	P	S	20.485	2.543	0.000	2.543	-2.420	0.000	-2.420	60.000	22.244	22.244
15195	384	57	52	P	S	39.515	2.543	0.000	2.543	-2.420	0.000	-2.420	60.000	22.244	22.244
15196	384	57	52	P	S	52.689	2.543	0.000	2.543	-2.420	0.000	-2.420	60.000	22.244	22.244
15197	384	57	52	P	S	67.311	2.543	0.000	2.543	-2.420	0.000	-2.420	60.000	22.244	22.244
15198	384	57	52	P	S	80.485	2.543	0.000	2.543	-2.420	0.000	-2.420	60.000	22.244	22.244

BL number	Atoms	γ-PC unit cells	WS_2 unit cells	γ-PC origin atom	WS_2 origin atom	Twist-angle (°)	γ-PC strain 1 (%)	γ-PC strain 2 (%)	γ-PC strain 3 (%)	WS_2 strain 1 (%)	WS_2 strain 2 (%)	WS_2 strain 3 (%)	γ (°)	|**a**|(Å)	|**b**|(Å)
15199	384	57	52	P	S	99.515	2.543	0.000	2.543	-2.420	0.000	-2.420	60.000	22.244	22.244
15200	384	57	52	P	S	112.689	2.543	0.000	2.543	-2.420	0.000	-2.420	60.000	22.244	22.244
15201	385	64	43	C	S	7.589	-4.909	0.000	-4.909	5.443	0.000	5.443	60.000	21.857	21.857
15202	385	64	43	C	S	52.411	-4.909	0.000	-4.909	5.443	0.000	5.443	60.000	21.857	21.857
15203	385	64	43	C	S	67.589	-4.909	0.000	-4.909	5.443	0.000	5.443	60.000	21.857	21.857
15204	385	64	43	C	S	112.411	-4.909	0.000	-4.909	5.443	0.000	5.443	60.000	21.857	21.857
15205	385	64	43	C	W	7.589	-4.909	0.000	-4.909	5.443	0.000	5.443	60.000	21.857	21.857
15206	385	64	43	C	W	52.411	-4.909	0.000	-4.909	5.443	0.000	5.443	60.000	21.857	21.857
15207	385	64	43	C	W	67.589	-4.909	0.000	-4.909	5.443	0.000	5.443	60.000	21.857	21.857
15208	385	64	43	C	W	112.411	-4.909	0.000	-4.909	5.443	0.000	5.443	60.000	21.857	21.857
15209	385	64	43	P	S	7.589	-4.909	0.000	-4.909	5.443	0.000	5.443	60.000	21.857	21.857
15210	385	64	43	P	S	112.411	-4.909	0.000	-4.909	5.443	0.000	5.443	60.000	21.857	21.857
15211	385	64	43	P	W	52.411	-4.909	0.000	-4.909	5.443	0.000	5.443	60.000	21.857	21.857
15212	385	64	43	P	W	67.589	-4.909	0.000	-4.909	5.443	0.000	5.443	60.000	21.857	21.857
15213	388	61	48	C	S	3.671	-1.202	0.000	-1.202	1.231	0.000	1.231	60.000	22.171	22.171
15214	388	61	48	C	S	56.330	-1.202	0.000	-1.202	1.231	0.000	1.231	60.000	22.171	22.171
15215	388	61	48	C	S	63.671	-1.202	0.000	-1.202	1.231	0.000	1.231	60.000	22.171	22.171
15216	388	61	48	C	S	116.330	-1.202	0.000	-1.202	1.231	0.000	1.231	60.000	22.171	22.171
15217	388	61	48	C	W	3.671	-1.202	0.000	-1.202	1.231	0.000	1.231	60.000	22.171	22.171
15218	388	61	48	C	W	56.330	-1.202	0.000	-1.202	1.231	0.000	1.231	60.000	22.171	22.171
15219	388	61	48	C	W	63.671	-1.202	0.000	-1.202	1.231	0.000	1.231	60.000	22.171	22.171
15220	388	61	48	C	W	116.330	-1.202	0.000	-1.202	1.231	0.000	1.231	60.000	22.171	22.171
15221	391	61	49	C	S	4.543	-0.696	0.000	-0.696	0.706	0.000	0.706	60.000	22.284	22.284
15222	391	61	49	C	S	11.884	-0.696	0.000	-0.696	0.706	0.000	0.706	60.000	22.284	22.284
15223	391	61	49	C	S	26.330	-0.696	0.000	-0.696	0.706	0.000	0.706	60.000	22.284	22.284
15224	391	61	49	C	S	33.671	-0.696	0.000	-0.696	0.706	0.000	0.706	60.000	22.284	22.284
15225	391	61	49	C	S	48.116	-0.696	0.000	-0.696	0.706	0.000	0.706	60.000	22.284	22.284
15226	391	61	49	C	S	55.457	-0.696	0.000	-0.696	0.706	0.000	0.706	60.000	22.284	22.284
15227	391	61	49	C	S	64.543	-0.696	0.000	-0.696	0.706	0.000	0.706	60.000	22.284	22.284
15228	391	61	49	C	S	71.884	-0.696	0.000	-0.696	0.706	0.000	0.706	60.000	22.284	22.284
15229	391	61	49	C	S	86.329	-0.696	0.000	-0.696	0.706	0.000	0.706	60.000	22.284	22.284
15230	391	61	49	C	S	93.670	-0.696	0.000	-0.696	0.706	0.000	0.706	60.000	22.284	22.284
15231	391	61	49	C	S	108.116	-0.696	0.000	-0.696	0.706	0.000	0.706	60.000	22.284	22.284
15232	391	61	49	C	S	115.457	-0.696	0.000	-0.696	0.706	0.000	0.706	60.000	22.284	22.284
15233	391	61	49	C	W	4.543	-0.696	0.000	-0.696	0.706	0.000	0.706	60.000	22.284	22.284
15234	391	61	49	C	W	11.884	-0.696	0.000	-0.696	0.706	0.000	0.706	60.000	22.284	22.284
15235	391	61	49	C	W	26.330	-0.696	0.000	-0.696	0.706	0.000	0.706	60.000	22.284	22.284
15236	391	61	49	C	W	33.671	-0.696	0.000	-0.696	0.706	0.000	0.706	60.000	22.284	22.284
15237	391	61	49	C	W	48.116	-0.696	0.000	-0.696	0.706	0.000	0.706	60.000	22.284	22.284
15238	391	61	49	C	W	55.457	-0.696	0.000	-0.696	0.706	0.000	0.706	60.000	22.284	22.284
15239	391	61	49	C	W	64.543	-0.696	0.000	-0.696	0.706	0.000	0.706	60.000	22.284	22.284
15240	391	61	49	C	W	71.884	-0.696	0.000	-0.696	0.706	0.000	0.706	60.000	22.284	22.284
15241	391	61	49	C	W	86.329	-0.696	0.000	-0.696	0.706	0.000	0.706	60.000	22.284	22.284
15242	391	61	49	C	W	93.670	-0.696	0.000	-0.696	0.706	0.000	0.706	60.000	22.284	22.284
15243	391	61	49	C	W	108.116	-0.696	0.000	-0.696	0.706	0.000	0.706	60.000	22.284	22.284
15244	391	61	49	C	W	115.457	-0.696	0.000	-0.696	0.706	0.000	0.706	60.000	22.284	22.284
15245	391	61	49	P	S	11.884	-0.696	0.000	-0.696	0.706	0.000	0.706	60.000	22.284	22.284
15246	391	61	49	P	S	26.330	-0.696	0.000	-0.696	0.706	0.000	0.706	60.000	22.284	22.284
15247	391	61	49	P	S	55.457	-0.696	0.000	-0.696	0.706	0.000	0.706	60.000	22.284	22.284
15248	391	61	49	P	S	64.543	-0.696	0.000	-0.696	0.706	0.000	0.706	60.000	22.284	22.284
15249	391	61	49	P	S	93.670	-0.696	0.000	-0.696	0.706	0.000	0.706	60.000	22.284	22.284

BL number	Atoms	γ-PC unit cells	WS_2 unit cells	γ-PC origin atom	WS_2 origin atom	Twist-angle (°)	γ-PC strain 1 (%)	γ-PC strain 2 (%)	γ-PC strain 3 (%)	WS_2 strain 1 (%)	WS_2 strain 2 (%)	WS_2 strain 3 (%)	γ (°)	|**a**|(Å)	|**b**|(Å)
15250	391	61	49	P	S	108.116	-0.696	0.000	-0.696	0.706	0.000	0.706	60.000	22.284	22.284
15251	391	61	49	P	W	4.543	-0.696	0.000	-0.696	0.706	0.000	0.706	60.000	22.284	22.284
15252	391	61	49	P	W	33.671	-0.696	0.000	-0.696	0.706	0.000	0.706	60.000	22.284	22.284
15253	391	61	49	P	W	48.116	-0.696	0.000	-0.696	0.706	0.000	0.706	60.000	22.284	22.284
15254	391	61	49	P	W	71.884	-0.696	0.000	-0.696	0.706	0.000	0.706	60.000	22.284	22.284
15255	391	61	49	P	W	86.329	-0.696	0.000	-0.696	0.706	0.000	0.706	60.000	22.284	22.284
15256	391	61	49	P	W	115.457	-0.696	0.000	-0.696	0.706	0.000	0.706	60.000	22.284	22.284
15257	392	62	48	C	S	38.948	-4.365	0.000	1.339	4.783	0.000	-1.304	90.000	26.498	16.211
15258	392	62	48	C	S	81.052	1.339	0.000	-4.365	-1.304	0.000	4.783	90.000	16.211	26.498
15259	392	62	48	C	S	98.948	-4.365	0.000	1.339	4.783	0.000	-1.304	90.000	26.498	16.211
15260	392	62	48	C	W	21.052	1.339	0.000	-4.365	-1.304	0.000	4.783	90.000	16.211	26.498
15261	392	62	48	C	W	38.948	-4.365	0.000	1.339	4.783	0.000	-1.304	90.000	26.498	16.211
15262	392	62	48	C	W	81.052	1.339	0.000	-4.365	-1.304	0.000	4.783	90.000	16.211	26.498
15263	392	62	48	C	W	98.948	-4.365	0.000	1.339	4.783	0.000	-1.304	90.000	26.498	16.211
15264	392	62	48	P	S	21.052	1.339	0.000	-4.365	-1.304	0.000	4.783	90.000	16.211	26.498
15265	392	62	48	P	S	38.948	-4.365	0.000	1.339	4.783	0.000	-1.304	90.000	26.498	16.211
15266	392	62	48	P	S	81.052	1.339	0.000	-4.365	-1.304	0.000	4.783	90.000	16.211	26.498
15267	392	62	48	P	S	98.948	-4.365	0.000	1.339	4.783	0.000	-1.304	90.000	26.498	16.211
15268	392	62	48	P	W	21.052	1.339	0.000	-4.365	-1.304	0.000	4.783	90.000	16.211	26.498
15269	392	62	48	P	W	38.948	-4.365	0.000	1.339	4.783	0.000	-1.304	90.000	26.498	16.211
15270	392	62	48	P	W	81.052	1.339	0.000	-4.365	-1.304	0.000	4.783	90.000	16.211	26.498
15271	392	62	48	P	W	98.948	-4.365	0.000	1.339	4.783	0.000	-1.304	90.000	26.498	16.211
15272	399	63	49	C	S	2.680	-1.485	0.000	-1.485	1.530	0.000	1.530	60.000	22.467	22.466
15273	399	63	49	C	S	57.320	-1.485	0.000	-1.485	1.530	0.000	1.530	60.000	22.466	22.466
15274	399	63	49	C	S	62.680	-1.485	0.000	-1.485	1.530	0.000	1.530	60.000	22.466	22.466
15275	399	63	49	C	S	117.320	-1.485	0.000	-1.485	1.530	0.000	1.530	60.000	22.466	22.466
15276	399	63	49	C	W	117.320	-1.485	0.000	-1.485	1.530	0.000	1.530	60.000	22.466	22.466
15277	399	63	49	P	S	2.680	-1.485	0.000	-1.485	1.530	0.000	1.530	60.000	22.467	22.466
15278	399	63	49	P	S	57.320	-1.485	0.000	-1.485	1.530	0.000	1.530	60.000	22.466	22.466
15279	399	63	49	P	S	62.680	-1.485	0.000	-1.485	1.530	0.000	1.530	60.000	22.466	22.466
15280	399	63	49	P	S	117.320	-1.485	0.000	-1.485	1.530	0.000	1.530	60.000	22.466	22.466
15281	399	63	49	P	W	117.320	-1.485	0.000	-1.485	1.530	0.000	1.530	60.000	22.466	22.466
15282	400	61	52	C	S	12.432	0.791	0.000	0.791	-0.779	0.000	-0.779	60.000	22.618	22.618
15283	400	61	52	C	S	19.773	0.791	0.000	0.791	-0.779	0.000	-0.779	60.000	22.618	22.618
15284	400	61	52	C	S	40.227	0.791	0.000	0.791	-0.779	0.000	-0.779	60.000	22.618	22.618
15285	400	61	52	C	S	47.568	0.791	0.000	0.791	-0.779	0.000	-0.779	60.000	22.618	22.618
15286	400	61	52	C	S	72.432	0.791	0.000	0.791	-0.779	0.000	-0.779	60.000	22.618	22.618
15287	400	61	52	C	S	79.773	0.791	0.000	0.791	-0.779	0.000	-0.779	60.000	22.618	22.618
15288	400	61	52	C	S	100.227	0.791	0.000	0.791	-0.779	0.000	-0.779	60.000	22.618	22.618
15289	400	61	52	C	S	107.568	0.791	0.000	0.791	-0.779	0.000	-0.779	60.000	22.618	22.618
15290	400	61	52	C	W	12.432	0.791	0.000	0.791	-0.779	0.000	-0.779	60.000	22.618	22.618
15291	400	61	52	C	W	19.773	0.791	0.000	0.791	-0.779	0.000	-0.779	60.000	22.618	22.618
15292	400	61	52	C	W	40.227	0.791	0.000	0.791	-0.779	0.000	-0.779	60.000	22.618	22.618
15293	400	61	52	C	W	47.568	0.791	0.000	0.791	-0.779	0.000	-0.779	60.000	22.618	22.618
15294	400	61	52	C	W	72.432	0.791	0.000	0.791	-0.779	0.000	-0.779	60.000	22.618	22.618
15295	400	61	52	C	W	79.773	0.791	0.000	0.791	-0.779	0.000	-0.779	60.000	22.618	22.618
15296	400	61	52	C	W	100.227	0.791	0.000	0.791	-0.779	0.000	-0.779	60.000	22.618	22.618
15297	400	61	52	C	W	107.568	0.791	0.000	0.791	-0.779	0.000	-0.779	60.000	22.618	22.618
15298	400	61	52	P	S	12.432	0.791	0.000	0.791	-0.779	0.000	-0.779	60.000	22.618	22.618
15299	400	61	52	P	S	40.227	0.791	0.000	0.791	-0.779	0.000	-0.779	60.000	22.618	22.618
15300	400	61	52	P	S	79.773	0.791	0.000	0.791	-0.779	0.000	-0.779	60.000	22.618	22.618

BL number	Atoms	γ-PC unit cells	WS_2 unit cells	γ-PC origin atom	WS_2 origin atom	Twist-angle (°)	γ-PC strain 1 (%)	γ-PC strain 2 (%)	γ-PC strain 3 (%)	WS_2 strain 1 (%)	WS_2 strain 2 (%)	WS_2 strain 3 (%)	γ (°)	|**a**|(Å)	|**b**|(Å)
15301	400	61	52	P	S	107.568	0.791	0.000	0.791	-0.779	0.000	-0.779	60.000	22.618	22.618
15302	400	61	52	P	W	19.773	0.791	0.000	0.791	-0.779	0.000	-0.779	60.000	22.618	22.618
15303	400	61	52	P	W	47.568	0.791	0.000	0.791	-0.779	0.000	-0.779	60.000	22.618	22.618
15304	400	61	52	P	W	72.432	0.791	0.000	0.791	-0.779	0.000	-0.779	60.000	22.618	22.618
15305	400	61	52	P	W	100.227	0.791	0.000	0.791	-0.779	0.000	-0.779	60.000	22.618	22.618
15306	403	64	49	C	S	0.000	-1.865	0.000	-1.865	1.938	0.000	1.938	60.000	22.557	22.557
15307	403	64	49	C	S	21.787	-1.865	0.000	-1.865	1.938	0.000	1.938	60.000	22.557	22.557
15308	403	64	49	C	S	38.213	-1.865	0.000	-1.865	1.938	0.000	1.938	60.000	22.557	22.557
15309	403	64	49	C	S	60.000	-1.865	0.000	-1.865	1.938	0.000	1.938	60.000	22.557	22.557
15310	403	64	49	C	S	81.787	-1.865	0.000	-1.865	1.938	0.000	1.938	60.000	22.557	22.557
15311	403	64	49	C	S	98.213	-1.865	0.000	-1.865	1.938	0.000	1.938	60.000	22.557	22.557
15312	403	64	49	C	W	0.000	-1.865	0.000	-1.865	1.938	0.000	1.938	60.000	22.557	22.557
15313	403	64	49	C	W	21.787	-1.865	0.000	-1.865	1.938	0.000	1.938	60.000	22.557	22.557
15314	403	64	49	C	W	38.213	-1.865	0.000	-1.865	1.938	0.000	1.938	60.000	22.557	22.557
15315	403	64	49	C	W	60.000	-1.865	0.000	-1.865	1.938	0.000	1.938	60.000	22.557	22.557
15316	403	64	49	C	W	81.787	-1.865	0.000	-1.865	1.938	0.000	1.938	60.000	22.557	22.557
15317	403	64	49	C	W	98.213	-1.865	0.000	-1.865	1.938	0.000	1.938	60.000	22.557	22.557
15318	403	64	49	P	S	21.787	-1.865	0.000	-1.865	1.938	0.000	1.938	60.000	22.557	22.557
15319	403	64	49	P	S	60.000	-1.865	0.000	-1.865	1.938	0.000	1.938	60.000	22.557	22.557
15320	403	64	49	P	S	98.213	-1.865	0.000	-1.865	1.938	0.000	1.938	60.000	22.557	22.557
15321	403	64	49	P	W	0.000	-1.865	0.000	-1.865	1.938	0.000	1.938	60.000	22.557	22.557
15322	403	64	49	P	W	38.213	-1.865	0.000	-1.865	1.938	0.000	1.938	60.000	22.557	22.557
15323	403	64	49	P	W	81.787	-1.865	0.000	-1.865	1.938	0.000	1.938	60.000	22.557	22.557
15324	408	63	52	C	S	5.209	-0.022	0.000	-0.022	0.022	0.000	0.022	60.000	22.800	22.800
15325	408	63	52	C	S	26.996	-0.022	0.000	-0.022	0.022	0.000	0.022	60.000	22.800	22.800
15326	408	63	52	C	S	33.005	-0.022	0.000	-0.022	0.022	0.000	0.022	60.000	22.800	22.800
15327	408	63	52	C	S	54.791	-0.022	0.000	-0.022	0.022	0.000	0.022	60.000	22.800	22.800
15328	408	63	52	C	S	65.209	-0.022	0.000	-0.022	0.022	0.000	0.022	60.000	22.800	22.800
15329	408	63	52	C	S	86.996	-0.022	0.000	-0.022	0.022	0.000	0.022	60.000	22.800	22.800
15330	408	63	52	C	S	93.005	-0.022	0.000	-0.022	0.022	0.000	0.022	60.000	22.800	22.800
15331	408	63	52	C	S	114.791	-0.022	0.000	-0.022	0.022	0.000	0.022	60.000	22.800	22.800
15332	408	63	52	P	S	5.209	-0.022	0.000	-0.022	0.022	0.000	0.022	60.000	22.800	22.800
15333	408	63	52	P	S	26.996	-0.022	0.000	-0.022	0.022	0.000	0.022	60.000	22.800	22.800
15334	408	63	52	P	S	33.005	-0.022	0.000	-0.022	0.022	0.000	0.022	60.000	22.800	22.800
15335	408	63	52	P	S	54.791	-0.022	0.000	-0.022	0.022	0.000	0.022	60.000	22.800	22.800
15336	408	63	52	P	S	65.209	-0.022	0.000	-0.022	0.022	0.000	0.022	60.000	22.800	22.800
15337	408	63	52	P	S	86.996	-0.022	0.000	-0.022	0.022	0.000	0.022	60.000	22.800	22.800
15338	408	63	52	P	S	93.005	-0.022	0.000	-0.022	0.022	0.000	0.022	60.000	22.800	22.800
15339	408	63	52	P	S	114.791	-0.022	0.000	-0.022	0.022	0.000	0.022	60.000	22.800	22.800
15340	412	67	48	C	S	17.784	-3.438	0.000	-3.438	3.692	0.000	3.692	60.000	22.709	22.709
15341	412	67	48	C	S	42.216	-3.438	0.000	-3.438	3.692	0.000	3.692	60.000	22.709	22.709
15342	412	67	48	C	S	77.784	-3.438	0.000	-3.438	3.692	0.000	3.692	60.000	22.709	22.709
15343	412	67	48	C	S	102.216	-3.438	0.000	-3.438	3.692	0.000	3.692	60.000	22.709	22.709
15344	412	67	48	C	W	17.784	-3.438	0.000	-3.438	3.692	0.000	3.692	60.000	22.709	22.709
15345	412	67	48	C	W	42.216	-3.438	0.000	-3.438	3.692	0.000	3.692	60.000	22.709	22.709
15346	412	67	48	C	W	77.784	-3.438	0.000	-3.438	3.692	0.000	3.692	60.000	22.709	22.709
15347	412	67	48	C	W	102.216	-3.438	0.000	-3.438	3.692	0.000	3.692	60.000	22.709	22.709
15348	415	61	57	C	S	19.743	3.177	0.000	3.177	-2.987	0.000	-2.987	60.000	23.153	23.153
15349	415	61	57	C	S	27.084	3.177	0.000	3.177	-2.987	0.000	-2.987	60.000	23.153	23.153
15350	415	61	57	C	S	32.916	3.177	0.000	3.177	-2.987	0.000	-2.987	60.000	23.153	23.153
15351	415	61	57	C	S	40.257	3.177	0.000	3.177	-2.987	0.000	-2.987	60.000	23.153	23.153

BL number	Atoms	γ-PC unit cells	WS_2 unit cells	γ-PC origin atom	WS_2 origin atom	Twist-angle (°)	γ-PC strain 1 (%)	γ-PC strain 2 (%)	γ-PC strain 3 (%)	WS_2 strain 1 (%)	WS_2 strain 2 (%)	WS_2 strain 3 (%)	γ (°)	|**a**|(Å)	|**b**|(Å)
15352	415	61	57	C	S	79.743	3.177	0.000	3.177	-2.987	0.000	-2.987	60.000	23.153	23.153
15353	415	61	57	C	S	87.084	3.177	0.000	3.177	-2.987	0.000	-2.987	60.000	23.153	23.153
15354	415	61	57	C	S	92.916	3.177	0.000	3.177	-2.987	0.000	-2.987	60.000	23.153	23.153
15355	415	61	57	C	S	100.257	3.177	0.000	3.177	-2.987	0.000	-2.987	60.000	23.153	23.153
15356	415	61	57	C	W	19.743	3.177	0.000	3.177	-2.987	0.000	-2.987	60.000	23.153	23.153
15357	415	61	57	C	W	27.084	3.177	0.000	3.177	-2.987	0.000	-2.987	60.000	23.153	23.153
15358	415	61	57	C	W	32.916	3.177	0.000	3.177	-2.987	0.000	-2.987	60.000	23.153	23.153
15359	415	61	57	C	W	40.257	3.177	0.000	3.177	-2.987	0.000	-2.987	60.000	23.153	23.153
15360	415	61	57	C	W	79.743	3.177	0.000	3.177	-2.987	0.000	-2.987	60.000	23.153	23.153
15361	415	61	57	C	W	87.084	3.177	0.000	3.177	-2.987	0.000	-2.987	60.000	23.153	23.153
15362	415	61	57	C	W	92.916	3.177	0.000	3.177	-2.987	0.000	-2.987	60.000	23.153	23.153
15363	415	61	57	C	W	100.257	3.177	0.000	3.177	-2.987	0.000	-2.987	60.000	23.153	23.153
15364	415	67	49	C	S	9.570	-2.955	0.000	-2.955	3.141	0.000	3.141	60.000	22.823	22.823
15365	415	67	49	C	S	12.216	-2.955	0.000	-2.955	3.141	0.000	3.141	60.000	22.823	22.823
15366	415	67	49	C	S	25.997	-2.955	0.000	-2.955	3.141	0.000	3.141	60.000	22.823	22.823
15367	415	67	49	C	S	34.003	-2.955	0.000	-2.955	3.141	0.000	3.141	60.000	22.823	22.823
15368	415	67	49	C	S	47.784	-2.955	0.000	-2.955	3.141	0.000	3.141	60.000	22.823	22.823
15369	415	67	49	C	S	50.430	-2.955	0.000	-2.955	3.141	0.000	3.141	60.000	22.823	22.823
15370	415	67	49	C	S	69.571	-2.955	0.000	-2.955	3.141	0.000	3.141	60.000	22.823	22.823
15371	415	67	49	C	S	72.216	-2.955	0.000	-2.955	3.141	0.000	3.141	60.000	22.823	22.823
15372	415	67	49	C	S	85.997	-2.955	0.000	-2.955	3.141	0.000	3.141	60.000	22.823	22.823
15373	415	67	49	C	S	94.003	-2.955	0.000	-2.955	3.141	0.000	3.141	60.000	22.823	22.823
15374	415	67	49	C	S	107.784	-2.955	0.000	-2.955	3.141	0.000	3.141	60.000	22.823	22.823
15375	415	67	49	C	S	110.430	-2.955	0.000	-2.955	3.141	0.000	3.141	60.000	22.823	22.823
15376	415	67	49	C	W	9.570	-2.955	0.000	-2.955	3.141	0.000	3.141	60.000	22.823	22.823
15377	415	67	49	C	W	12.216	-2.955	0.000	-2.955	3.141	0.000	3.141	60.000	22.823	22.823
15378	415	67	49	C	W	25.997	-2.955	0.000	-2.955	3.141	0.000	3.141	60.000	22.823	22.823
15379	415	67	49	C	W	34.003	-2.955	0.000	-2.955	3.141	0.000	3.141	60.000	22.823	22.823
15380	415	67	49	C	W	47.784	-2.955	0.000	-2.955	3.141	0.000	3.141	60.000	22.823	22.823
15381	415	67	49	C	W	50.430	-2.955	0.000	-2.955	3.141	0.000	3.141	60.000	22.823	22.823
15382	415	67	49	C	W	69.571	-2.955	0.000	-2.955	3.141	0.000	3.141	60.000	22.823	22.823
15383	415	67	49	C	W	72.216	-2.955	0.000	-2.955	3.141	0.000	3.141	60.000	22.823	22.823
15384	415	67	49	C	W	85.997	-2.955	0.000	-2.955	3.141	0.000	3.141	60.000	22.823	22.823
15385	415	67	49	C	W	94.003	-2.955	0.000	-2.955	3.141	0.000	3.141	60.000	22.823	22.823
15386	415	67	49	C	W	107.784	-2.955	0.000	-2.955	3.141	0.000	3.141	60.000	22.823	22.823
15387	415	67	49	C	W	110.430	-2.955	0.000	-2.955	3.141	0.000	3.141	60.000	22.823	22.823
15388	415	67	49	P	S	9.570	-2.955	0.000	-2.955	3.141	0.000	3.141	60.000	22.823	22.823
15389	415	67	49	P	S	34.003	-2.955	0.000	-2.955	3.141	0.000	3.141	60.000	22.823	22.823
15390	415	67	49	P	S	47.784	-2.955	0.000	-2.955	3.141	0.000	3.141	60.000	22.823	22.823
15391	415	67	49	P	S	72.216	-2.955	0.000	-2.955	3.141	0.000	3.141	60.000	22.823	22.823
15392	415	67	49	P	S	85.997	-2.955	0.000	-2.955	3.141	0.000	3.141	60.000	22.823	22.823
15393	415	67	49	P	S	110.430	-2.955	0.000	-2.955	3.141	0.000	3.141	60.000	22.823	22.823
15394	415	67	49	P	W	12.216	-2.955	0.000	-2.955	3.141	0.000	3.141	60.000	22.823	22.823
15395	415	67	49	P	W	25.997	-2.955	0.000	-2.955	3.141	0.000	3.141	60.000	22.823	22.823
15396	415	67	49	P	W	50.430	-2.955	0.000	-2.955	3.141	0.000	3.141	60.000	22.823	22.823
15397	415	67	49	P	W	69.571	-2.955	0.000	-2.955	3.141	0.000	3.141	60.000	22.823	22.823
15398	415	67	49	P	W	94.003	-2.955	0.000	-2.955	3.141	0.000	3.141	60.000	22.823	22.823
15399	415	67	49	P	W	107.784	-2.955	0.000	-2.955	3.141	0.000	3.141	60.000	22.823	22.823
15400	424	67	52	C	S	1.682	-1.537	0.000	-1.537	1.585	0.000	1.585	60.000	23.157	23.157
15401	424	67	52	C	S	26.114	-1.537	0.000	-1.537	1.585	0.000	1.585	60.000	23.157	23.157
15402	424	67	52	C	S	33.886	-1.537	0.000	-1.537	1.585	0.000	1.585	60.000	23.157	23.157

BL number	Atoms	γ-PC unit cells	WS_2 unit cells	γ-PC origin atom	WS_2 origin atom	Twist-angle (°)	γ-PC strain 1 (%)	γ-PC strain 2 (%)	γ-PC strain 3 (%)	WS_2 strain 1 (%)	WS_2 strain 2 (%)	WS_2 strain 3 (%)	γ (°)	|**a**|(Å)	|**b**|(Å)
15403	424	67	52	C	S	58.319	-1.537	0.000	-1.537	1.585	0.000	1.585	60.000	23.157	23.157
15404	424	67	52	C	S	61.682	-1.537	0.000	-1.537	1.585	0.000	1.585	60.000	23.157	23.157
15405	424	67	52	C	S	86.114	-1.537	0.000	-1.537	1.585	0.000	1.585	60.000	23.157	23.157
15406	424	67	52	C	S	93.886	-1.537	0.000	-1.537	1.585	0.000	1.585	60.000	23.157	23.157
15407	424	67	52	C	S	118.318	-1.537	0.000	-1.537	1.585	0.000	1.585	60.000	23.157	23.157
15408	424	67	52	C	W	1.682	-1.537	0.000	-1.537	1.585	0.000	1.585	60.000	23.157	23.157
15409	424	67	52	C	W	26.114	-1.537	0.000	-1.537	1.585	0.000	1.585	60.000	23.157	23.157
15410	424	67	52	C	W	33.886	-1.537	0.000	-1.537	1.585	0.000	1.585	60.000	23.157	23.157
15411	424	67	52	C	W	58.319	-1.537	0.000	-1.537	1.585	0.000	1.585	60.000	23.157	23.157
15412	424	67	52	C	W	61.682	-1.537	0.000	-1.537	1.585	0.000	1.585	60.000	23.157	23.157
15413	424	67	52	C	W	86.114	-1.537	0.000	-1.537	1.585	0.000	1.585	60.000	23.157	23.157
15414	424	67	52	C	W	93.886	-1.537	0.000	-1.537	1.585	0.000	1.585	60.000	23.157	23.157
15415	424	67	52	C	W	118.318	-1.537	0.000	-1.537	1.585	0.000	1.585	60.000	23.157	23.157
15416	424	67	52	P	S	26.114	-1.537	0.000	-1.537	1.585	0.000	1.585	60.000	23.157	23.157
15417	424	67	52	P	S	33.886	-1.537	0.000	-1.537	1.585	0.000	1.585	60.000	23.157	23.157
15418	424	67	52	P	S	58.319	-1.537	0.000	-1.537	1.585	0.000	1.585	60.000	23.157	23.157
15419	424	67	52	P	S	61.682	-1.537	0.000	-1.537	1.585	0.000	1.585	60.000	23.157	23.157
15420	424	67	52	P	S	86.114	-1.537	0.000	-1.537	1.585	0.000	1.585	60.000	23.157	23.157
15421	424	67	52	P	S	118.318	-1.537	0.000	-1.537	1.585	0.000	1.585	60.000	23.157	23.157
15422	424	67	52	P	W	1.682	-1.537	0.000	-1.537	1.585	0.000	1.585	60.000	23.157	23.157
15423	424	67	52	P	W	26.114	-1.537	0.000	-1.537	1.585	0.000	1.585	60.000	23.157	23.157
15424	424	67	52	P	W	33.886	-1.537	0.000	-1.537	1.585	0.000	1.585	60.000	23.157	23.157
15425	424	67	52	P	W	58.319	-1.537	0.000	-1.537	1.585	0.000	1.585	60.000	23.157	23.157
15426	424	67	52	P	W	61.682	-1.537	0.000	-1.537	1.585	0.000	1.585	60.000	23.157	23.157
15427	424	67	52	P	W	86.114	-1.537	0.000	-1.537	1.585	0.000	1.585	60.000	23.157	23.157
15428	424	67	52	P	W	93.886	-1.537	0.000	-1.537	1.585	0.000	1.585	60.000	23.157	23.157
15429	424	67	52	P	W	118.318	-1.537	0.000	-1.537	1.585	0.000	1.585	60.000	23.157	23.157
15430	427	61	61	C	S	7.341	5.011	0.000	5.011	-4.554	0.000	-4.555	60.000	23.565	23.565
15431	427	61	61	C	S	52.659	5.011	0.000	5.011	-4.554	0.000	-4.555	60.000	23.565	23.565
15432	427	61	61	C	S	67.341	5.011	0.000	5.011	-4.554	0.000	-4.555	60.000	23.565	23.565
15433	427	61	61	C	S	112.659	5.011	0.000	5.011	-4.554	0.000	-4.555	60.000	23.565	23.565
15434	427	61	61	C	W	7.341	5.011	0.000	5.011	-4.554	0.000	-4.555	60.000	23.565	23.565
15435	427	61	61	C	W	52.659	5.011	0.000	5.011	-4.554	0.000	-4.555	60.000	23.565	23.565
15436	427	61	61	C	W	67.341	5.011	0.000	5.011	-4.554	0.000	-4.555	60.000	23.565	23.565
15437	427	61	61	C	W	112.659	5.011	0.000	5.011	-4.554	0.000	-4.555	60.000	23.565	23.565
15438	427	61	61	P	S	52.659	5.011	0.000	5.011	-4.554	0.000	-4.555	60.000	23.565	23.565
15439	427	61	61	P	S	67.341	5.011	0.000	5.011	-4.554	0.000	-4.555	60.000	23.565	23.565
15440	427	61	61	P	W	7.341	5.011	0.000	5.011	-4.554	0.000	-4.555	60.000	23.565	23.565
15441	427	61	61	P	W	112.659	5.011	0.000	5.011	-4.554	0.000	-4.555	60.000	23.565	23.565
15442	427	64	57	C	S	6.587	1.915	0.000	1.915	-1.845	0.000	-1.845	60.000	23.426	23.426
15443	427	64	57	C	S	53.413	1.915	0.000	1.915	-1.845	0.000	-1.845	60.000	23.426	23.426
15444	427	64	57	C	S	66.587	1.915	0.000	1.915	-1.845	0.000	-1.845	60.000	23.426	23.426
15445	427	64	57	C	S	113.413	1.915	0.000	1.915	-1.845	0.000	-1.845	60.000	23.426	23.426
15446	427	64	57	C	W	6.587	1.915	0.000	1.915	-1.845	0.000	-1.845	60.000	23.426	23.426
15447	427	64	57	C	W	53.413	1.915	0.000	1.915	-1.845	0.000	-1.845	60.000	23.426	23.426
15448	427	64	57	C	W	66.587	1.915	0.000	1.915	-1.845	0.000	-1.845	60.000	23.426	23.426
15449	427	64	57	C	W	113.413	1.915	0.000	1.915	-1.845	0.000	-1.845	60.000	23.426	23.426
15450	435	63	61	C	S	7.223	4.131	0.000	4.131	-3.816	0.000	-3.816	60.000	23.747	23.747
15451	435	63	61	C	S	14.564	4.131	0.000	4.131	-3.816	0.000	-3.816	60.000	23.747	23.747
15452	435	63	61	C	S	45.436	4.131	0.000	4.131	-3.816	0.000	-3.816	60.000	23.747	23.747
15453	435	63	61	C	S	52.777	4.131	0.000	4.131	-3.816	0.000	-3.816	60.000	23.747	23.747

BL number	Atoms	γ-PC unit cells	WS_2 unit cells	γ-PC origin atom	WS_2 origin atom	Twist-angle (°)	γ-PC strain 1 (%)	γ-PC strain 2 (%)	γ-PC strain 3 (%)	WS_2 strain 1 (%)	WS_2 strain 2 (%)	WS_2 strain 3 (%)	γ (°)	|**a**|(Å)	|**b**|(Å)
15454	435	63	61	C	S	67.223	4.131	0.000	4.131	-3.816	0.000	-3.816	60.000	23.747	23.747
15455	435	63	61	C	S	74.564	4.131	0.000	4.131	-3.816	0.000	-3.816	60.000	23.747	23.747
15456	435	63	61	C	S	105.436	4.131	0.000	4.131	-3.816	0.000	-3.816	60.000	23.747	23.747
15457	435	63	61	C	S	112.777	4.131	0.000	4.131	-3.816	0.000	-3.816	60.000	23.747	23.747
15458	435	63	61	P	S	7.223	4.131	0.000	4.131	-3.816	0.000	-3.816	60.000	23.747	23.747
15459	435	63	61	P	S	14.564	4.131	0.000	4.131	-3.816	0.000	-3.816	60.000	23.747	23.747
15460	435	63	61	P	S	45.436	4.131	0.000	4.131	-3.816	0.000	-3.816	60.000	23.747	23.747
15461	435	63	61	P	S	52.777	4.131	0.000	4.131	-3.816	0.000	-3.816	60.000	23.747	23.747
15462	435	63	61	P	S	67.223	4.131	0.000	4.131	-3.816	0.000	-3.816	60.000	23.747	23.747
15463	435	63	61	P	S	74.564	4.131	0.000	4.131	-3.816	0.000	-3.816	60.000	23.747	23.747
15464	435	63	61	P	S	105.436	4.131	0.000	4.131	-3.816	0.000	-3.816	60.000	23.747	23.747
15465	435	63	61	P	S	112.777	4.131	0.000	4.131	-3.816	0.000	-3.816	60.000	23.747	23.747
15466	436	70	52	C	S	16.102	-4.194	0.000	-0.922	4.578	0.000	0.940	90.000	23.839	19.927
15467	436	70	52	C	S	43.898	-4.194	0.000	-0.922	4.578	0.000	0.940	90.000	23.839	19.927
15468	436	70	52	C	S	76.102	-4.194	0.000	-0.922	4.578	0.000	0.940	90.000	23.839	19.927
15469	436	70	52	C	S	103.898	-4.194	0.000	-0.922	4.578	0.000	0.940	90.000	23.839	19.927
15470	436	70	52	C	W	16.102	-4.194	0.000	-0.922	4.578	0.000	0.940	90.000	23.839	19.927
15471	436	70	52	C	W	43.898	-4.194	0.000	-0.922	4.578	0.000	0.940	90.000	23.839	19.927
15472	436	70	52	C	W	76.102	-4.194	0.000	-0.922	4.578	0.000	0.940	90.000	23.839	19.927
15473	436	70	52	C	W	103.898	-4.194	0.000	-0.922	4.578	0.000	0.940	90.000	23.839	19.927
15474	436	70	52	P	S	16.102	-4.194	0.000	-0.922	4.578	0.000	0.940	90.000	23.839	19.927
15475	436	70	52	P	S	43.898	-4.194	0.000	-0.922	4.578	0.000	0.940	90.000	23.839	19.927
15476	436	70	52	P	S	76.102	-4.194	0.000	-0.922	4.578	0.000	0.940	90.000	23.839	19.927
15477	436	70	52	P	S	103.898	-4.194	0.000	-0.922	4.578	0.000	0.940	90.000	23.839	19.927
15478	436	70	52	P	W	16.102	-4.194	0.000	-0.922	4.578	0.000	0.940	90.000	23.839	19.927
15479	436	70	52	P	W	43.898	-4.194	0.000	-0.922	4.578	0.000	0.940	90.000	23.839	19.927
15480	436	70	52	P	W	76.102	-4.194	0.000	-0.922	4.578	0.000	0.940	90.000	23.839	19.927
15481	436	70	52	P	W	103.898	-4.194	0.000	-0.922	4.578	0.000	0.940	90.000	23.839	19.927
15482	439	64	61	C	S	26.330	3.706	0.000	3.706	-3.450	0.000	-3.450	60.000	23.837	23.837
15483	439	64	61	C	S	33.671	3.706	0.000	3.706	-3.450	0.000	-3.450	60.000	23.837	23.837
15484	439	64	61	C	S	86.329	3.706	0.000	3.706	-3.450	0.000	-3.450	60.000	23.837	23.837
15485	439	64	61	C	S	93.670	3.706	0.000	3.706	-3.450	0.000	-3.450	60.000	23.837	23.837
15486	439	64	61	C	W	26.330	3.706	0.000	3.706	-3.450	0.000	-3.450	60.000	23.837	23.837
15487	439	64	61	C	W	33.671	3.706	0.000	3.706	-3.450	0.000	-3.450	60.000	23.837	23.837
15488	439	64	61	C	W	86.329	3.706	0.000	3.706	-3.450	0.000	-3.450	60.000	23.837	23.837
15489	439	64	61	C	W	93.670	3.706	0.000	3.706	-3.450	0.000	-3.450	60.000	23.837	23.837
15490	439	64	61	P	S	33.671	3.706	0.000	3.706	-3.450	0.000	-3.450	60.000	23.837	23.837
15491	439	64	61	P	S	86.329	3.706	0.000	3.706	-3.450	0.000	-3.450	60.000	23.837	23.837
15492	439	64	61	P	W	26.330	3.706	0.000	3.706	-3.450	0.000	-3.450	60.000	23.837	23.837
15493	439	64	61	P	W	93.670	3.706	0.000	3.706	-3.450	0.000	-3.450	60.000	23.837	23.837
15494	439	67	57	C	S	5.630	0.740	0.000	0.740	-0.729	0.000	-0.729	60.000	23.692	23.692
15495	439	67	57	C	S	18.803	0.740	0.000	0.740	-0.729	0.000	-0.729	60.000	23.692	23.692
15496	439	67	57	C	S	41.197	0.740	0.000	0.740	-0.729	0.000	-0.729	60.000	23.692	23.692
15497	439	67	57	C	S	54.371	0.740	0.000	0.740	-0.729	0.000	-0.729	60.000	23.692	23.692
15498	439	67	57	C	S	65.630	0.740	0.000	0.740	-0.729	0.000	-0.729	60.000	23.692	23.692
15499	439	67	57	C	S	78.803	0.740	0.000	0.740	-0.729	0.000	-0.729	60.000	23.692	23.692
15500	439	67	57	C	S	101.197	0.740	0.000	0.740	-0.729	0.000	-0.729	60.000	23.692	23.692
15501	439	67	57	C	S	114.371	0.740	0.000	0.740	-0.729	0.000	-0.729	60.000	23.692	23.692
15502	439	67	57	C	W	5.630	0.740	0.000	0.740	-0.729	0.000	-0.729	60.000	23.692	23.692
15503	439	67	57	C	W	18.803	0.740	0.000	0.740	-0.729	0.000	-0.729	60.000	23.692	23.692
15504	439	67	57	C	W	41.197	0.740	0.000	0.740	-0.729	0.000	-0.729	60.000	23.692	23.692

BL number	Atoms	γ-PC unit cells	WS_2 unit cells	γ-PC origin atom	WS_2 origin atom	Twist-angle (°)	γ-PC strain 1 (%)	γ-PC strain 2 (%)	γ-PC strain 3 (%)	WS_2 strain 1 (%)	WS_2 strain 2 (%)	WS_2 strain 3 (%)	γ (°)	|**a**|(Å)	|**b**|(Å)
15505	439	67	57	C	W	54.371	0.740	0.000	0.740	-0.729	0.000	-0.729	60.000	23.692	23.692
15506	439	67	57	C	W	65.630	0.740	0.000	0.740	-0.729	0.000	-0.729	60.000	23.692	23.692
15507	439	67	57	C	W	78.803	0.740	0.000	0.740	-0.729	0.000	-0.729	60.000	23.692	23.692
15508	439	67	57	C	W	101.197	0.740	0.000	0.740	-0.729	0.000	-0.729	60.000	23.692	23.692
15509	439	67	57	C	W	114.371	0.740	0.000	0.740	-0.729	0.000	-0.729	60.000	23.692	23.692
15510	439	73	49	C	S	5.818	-4.930	0.000	-4.930	5.469	0.000	5.470	60.000	23.338	23.338
15511	439	73	49	C	S	15.969	-4.930	0.000	-4.930	5.469	0.000	5.470	60.000	23.338	23.338
15512	439	73	49	C	S	27.604	-4.930	0.000	-4.930	5.469	0.000	5.470	60.000	23.338	23.338
15513	439	73	49	C	S	32.396	-4.930	0.000	-4.930	5.469	0.000	5.470	60.000	23.338	23.338
15514	439	73	49	C	S	44.031	-4.930	0.000	-4.930	5.469	0.000	5.470	60.000	23.338	23.338
15515	439	73	49	C	S	54.183	-4.930	0.000	-4.930	5.469	0.000	5.470	60.000	23.338	23.338
15516	439	73	49	C	S	65.818	-4.930	0.000	-4.930	5.469	0.000	5.470	60.000	23.338	23.338
15517	439	73	49	C	S	75.969	-4.930	0.000	-4.930	5.469	0.000	5.470	60.000	23.338	23.338
15518	439	73	49	C	S	87.604	-4.930	0.000	-4.930	5.469	0.000	5.470	60.000	23.338	23.338
15519	439	73	49	C	S	92.396	-4.930	0.000	-4.930	5.469	0.000	5.470	60.000	23.338	23.338
15520	439	73	49	C	S	104.031	-4.930	0.000	-4.930	5.469	0.000	5.470	60.000	23.338	23.338
15521	439	73	49	C	S	114.182	-4.930	0.000	-4.930	5.469	0.000	5.470	60.000	23.338	23.338
15522	439	73	49	C	W	5.818	-4.930	0.000	-4.930	5.469	0.000	5.470	60.000	23.338	23.338
15523	439	73	49	C	W	15.969	-4.930	0.000	-4.930	5.469	0.000	5.470	60.000	23.338	23.338
15524	439	73	49	C	W	27.604	-4.930	0.000	-4.930	5.469	0.000	5.470	60.000	23.338	23.338
15525	439	73	49	C	W	32.396	-4.930	0.000	-4.930	5.469	0.000	5.470	60.000	23.338	23.338
15526	439	73	49	C	W	44.031	-4.930	0.000	-4.930	5.469	0.000	5.470	60.000	23.338	23.338
15527	439	73	49	C	W	54.183	-4.930	0.000	-4.930	5.469	0.000	5.470	60.000	23.338	23.338
15528	439	73	49	C	W	65.818	-4.930	0.000	-4.930	5.469	0.000	5.470	60.000	23.338	23.338
15529	439	73	49	C	W	87.604	-4.930	0.000	-4.930	5.469	0.000	5.470	60.000	23.338	23.338
15530	439	73	49	C	W	92.396	-4.930	0.000	-4.930	5.469	0.000	5.470	60.000	23.338	23.338
15531	439	73	49	C	W	104.031	-4.930	0.000	-4.930	5.469	0.000	5.470	60.000	23.338	23.338
15532	439	73	49	C	W	114.182	-4.930	0.000	-4.930	5.469	0.000	5.470	60.000	23.338	23.338
15533	439	73	49	P	S	5.818	-4.930	0.000	-4.930	5.469	0.000	5.470	60.000	23.338	23.338
15534	439	73	49	P	S	32.396	-4.930	0.000	-4.930	5.469	0.000	5.470	60.000	23.338	23.338
15535	439	73	49	P	S	44.031	-4.930	0.000	-4.930	5.469	0.000	5.470	60.000	23.338	23.338
15536	439	73	49	P	S	75.969	-4.930	0.000	-4.930	5.469	0.000	5.470	60.000	23.338	23.338
15537	439	73	49	P	S	87.604	-4.930	0.000	-4.930	5.469	0.000	5.470	60.000	23.338	23.338
15538	439	73	49	P	S	114.182	-4.930	0.000	-4.930	5.469	0.000	5.470	60.000	23.338	23.338
15539	439	73	49	P	W	15.969	-4.930	0.000	-4.930	5.469	0.000	5.470	60.000	23.338	23.338
15540	439	73	49	P	W	27.604	-4.930	0.000	-4.930	5.469	0.000	5.470	60.000	23.338	23.338
15541	439	73	49	P	W	54.183	-4.930	0.000	-4.930	5.469	0.000	5.470	60.000	23.338	23.338
15542	439	73	49	P	W	65.818	-4.930	0.000	-4.930	5.469	0.000	5.470	60.000	23.338	23.338
15543	439	73	49	P	W	92.396	-4.930	0.000	-4.930	5.469	0.000	5.470	60.000	23.338	23.338
15544	439	73	49	P	W	104.031	-4.930	0.000	-4.930	5.469	0.000	5.470	60.000	23.338	23.338
15545	444	63	64	C	S	19.107	5.446	0.000	5.446	-4.911	0.000	-4.911	60.000	24.047	24.047
15546	444	63	64	C	S	40.893	5.446	0.000	5.446	-4.911	0.000	-4.911	60.000	24.047	24.047
15547	444	63	64	C	S	79.107	5.446	0.000	5.446	-4.911	0.000	-4.911	60.000	24.047	24.047
15548	444	63	64	C	S	100.893	5.446	0.000	5.446	-4.911	0.000	-4.911	60.000	24.047	24.047
15549	444	63	64	P	S	19.107	5.446	0.000	5.446	-4.911	0.000	-4.911	60.000	24.047	24.047
15550	444	63	64	P	S	40.893	5.446	0.000	5.446	-4.911	0.000	-4.911	60.000	24.047	24.047
15551	444	63	64	P	S	79.107	5.446	0.000	5.446	-4.911	0.000	-4.911	60.000	24.047	24.047
15552	444	63	64	P	S	100.893	5.446	0.000	5.446	-4.911	0.000	-4.911	60.000	24.047	24.047
15553	444	66	60	C	S	30.000	2.934	0.000	1.972	-2.772	0.000	-1.897	90.000	15.368	32.228
15554	444	66	60	C	S	90.000	2.934	0.000	1.972	-2.772	0.000	-1.897	90.000	15.368	32.228
15555	444	66	60	C	W	30.000	2.934	0.000	1.972	-2.772	0.000	-1.897	90.000	15.368	32.228

BL number	Atoms	γ-PC unit cells	WS_2 unit cells	γ-PC origin atom	WS_2 origin atom	Twist-angle (°)	γ-PC strain 1 (%)	γ-PC strain 2 (%)	γ-PC strain 3 (%)	WS_2 strain 1 (%)	WS_2 strain 2 (%)	WS_2 strain 3 (%)	γ (°)	|**a**|(Å)	|**b**|(Å)
15556	444	66	60	C	W	90.000	2.934	0.000	1.972	-2.772	0.000	-1.897	90.000	15.368	32.228
15557	444	66	60	P	S	30.000	2.934	0.000	1.972	-2.772	0.000	-1.897	90.000	15.368	32.228
15558	444	66	60	P	S	90.000	1.972	0.000	2.934	-1.897	0.000	-2.772	90.000	32.228	15.368
15559	444	66	60	P	W	30.000	2.934	0.000	1.972	-2.772	0.000	-1.897	90.000	15.368	32.228
15560	444	66	60	P	W	90.000	1.972	0.000	2.934	-1.897	0.000	-2.772	90.000	32.228	15.368
15561	445	64	63	C	S	19.107	4.579	0.000	4.580	-4.195	0.000	-4.195	60.000	24.038	24.038
15562	445	64	63	C	S	40.893	4.579	0.000	4.580	-4.195	0.000	-4.195	60.000	24.038	24.038
15563	445	64	63	C	S	79.107	4.579	0.000	4.580	-4.195	0.000	-4.195	60.000	24.038	24.038
15564	445	64	63	C	S	100.893	4.579	0.000	4.580	-4.195	0.000	-4.195	60.000	24.038	24.038
15565	445	64	63	C	W	19.107	4.579	0.000	4.580	-4.195	0.000	-4.195	60.000	24.038	24.038
15566	445	64	63	C	W	40.893	4.579	0.000	4.580	-4.195	0.000	-4.195	60.000	24.038	24.038
15567	445	64	63	C	W	79.107	4.579	0.000	4.580	-4.195	0.000	-4.195	60.000	24.038	24.038
15568	445	64	63	C	W	100.893	4.579	0.000	4.580	-4.195	0.000	-4.195	60.000	24.038	24.038
15569	448	73	52	C	S	8.080	-3.571	0.000	-3.571	3.846	0.000	3.846	60.000	23.672	23.672
15570	448	73	52	C	S	19.715	-3.571	0.000	-3.571	3.846	0.000	3.846	60.000	23.672	23.672
15571	448	73	52	C	S	40.285	-3.571	0.000	-3.571	3.846	0.000	3.846	60.000	23.672	23.672
15572	448	73	52	C	S	51.920	-3.571	0.000	-3.571	3.846	0.000	3.846	60.000	23.672	23.672
15573	448	73	52	C	S	68.080	-3.571	0.000	-3.571	3.846	0.000	3.846	60.000	23.672	23.672
15574	448	73	52	C	S	79.716	-3.571	0.000	-3.571	3.846	0.000	3.846	60.000	23.672	23.672
15575	448	73	52	C	S	100.285	-3.571	0.000	-3.571	3.846	0.000	3.846	60.000	23.672	23.672
15576	448	73	52	C	S	111.920	-3.571	0.000	-3.571	3.846	0.000	3.846	60.000	23.672	23.672
15577	448	73	52	C	W	8.080	-3.571	0.000	-3.571	3.846	0.000	3.846	60.000	23.672	23.672
15578	448	73	52	C	W	19.715	-3.571	0.000	-3.571	3.846	0.000	3.846	60.000	23.672	23.672
15579	448	73	52	C	W	40.285	-3.571	0.000	-3.571	3.846	0.000	3.846	60.000	23.672	23.672
15580	448	73	52	C	W	51.920	-3.571	0.000	-3.571	3.846	0.000	3.846	60.000	23.672	23.672
15581	448	73	52	C	W	68.080	-3.571	0.000	-3.571	3.846	0.000	3.846	60.000	23.672	23.672
15582	448	73	52	C	W	79.716	-3.571	0.000	-3.571	3.846	0.000	3.846	60.000	23.672	23.672
15583	448	73	52	C	W	100.285	-3.571	0.000	-3.571	3.846	0.000	3.846	60.000	23.672	23.672
15584	448	73	52	C	W	111.920	-3.571	0.000	-3.571	3.846	0.000	3.846	60.000	23.672	23.672
15585	448	73	52	P	S	8.080	-3.571	0.000	-3.571	3.846	0.000	3.846	60.000	23.672	23.672
15586	448	73	52	P	S	19.715	-3.571	0.000	-3.571	3.846	0.000	3.846	60.000	23.672	23.672
15587	448	73	52	P	S	100.285	-3.571	0.000	-3.571	3.846	0.000	3.846	60.000	23.672	23.672
15588	448	73	52	P	S	111.920	-3.571	0.000	-3.571	3.846	0.000	3.846	60.000	23.672	23.672
15589	448	73	52	P	W	40.285	-3.571	0.000	-3.571	3.846	0.000	3.846	60.000	23.672	23.672
15590	448	73	52	P	W	51.920	-3.571	0.000	-3.571	3.846	0.000	3.846	60.000	23.672	23.672
15591	448	73	52	P	W	68.080	-3.571	0.000	-3.571	3.846	0.000	3.846	60.000	23.672	23.672
15592	448	73	52	P	W	79.716	-3.571	0.000	-3.571	3.846	0.000	3.846	60.000	23.672	23.672
15593	451	67	61	C	S	14.113	2.490	0.000	2.490	-2.372	0.000	-2.372	60.000	24.104	24.104
15594	451	67	61	C	S	21.454	2.490	0.000	2.490	-2.372	0.000	-2.372	60.000	24.104	24.104
15595	451	67	61	C	S	38.546	2.490	0.000	2.490	-2.372	0.000	-2.372	60.000	24.104	24.104
15596	451	67	61	C	S	45.887	2.490	0.000	2.490	-2.372	0.000	-2.372	60.000	24.104	24.104
15597	451	67	61	C	S	74.113	2.490	0.000	2.490	-2.372	0.000	-2.372	60.000	24.104	24.104
15598	451	67	61	C	S	81.454	2.490	0.000	2.490	-2.372	0.000	-2.372	60.000	24.104	24.104
15599	451	67	61	C	S	98.546	2.490	0.000	2.490	-2.372	0.000	-2.372	60.000	24.104	24.104
15600	451	67	61	C	S	105.887	2.490	0.000	2.490	-2.372	0.000	-2.372	60.000	24.104	24.104
15601	451	67	61	C	W	14.113	2.490	0.000	2.490	-2.372	0.000	-2.372	60.000	24.104	24.104
15602	451	67	61	C	W	21.454	2.490	0.000	2.490	-2.372	0.000	-2.372	60.000	24.104	24.104
15603	451	67	61	C	W	38.546	2.490	0.000	2.490	-2.372	0.000	-2.372	60.000	24.104	24.104
15604	451	67	61	C	W	45.887	2.490	0.000	2.490	-2.372	0.000	-2.372	60.000	24.104	24.104
15605	451	67	61	C	W	74.113	2.490	0.000	2.490	-2.372	0.000	-2.372	60.000	24.104	24.104
15606	451	67	61	C	W	81.454	2.490	0.000	2.490	-2.372	0.000	-2.372	60.000	24.104	24.104

BL number	Atoms	γ-PC unit cells	WS_2 unit cells	γ-PC origin atom	WS_2 origin atom	Twist-angle (°)	γ-PC strain 1 (%)	γ-PC strain 2 (%)	γ-PC strain 3 (%)	WS_2 strain 1 (%)	WS_2 strain 2 (%)	WS_2 strain 3 (%)	γ (°)	|**a**|(Å)	|**b**|(Å)
15607	451	67	61	C	W	98.546	2.490	0.000	2.490	-2.372	0.000	-2.372	60.000	24.104	24.104
15608	451	67	61	C	W	105.887	2.490	0.000	2.490	-2.372	0.000	-2.372	60.000	24.104	24.104
15609	451	67	61	P	S	21.454	2.490	0.000	2.490	-2.372	0.000	-2.372	60.000	24.104	24.104
15610	451	67	61	P	S	45.887	2.490	0.000	2.490	-2.372	0.000	-2.372	60.000	24.104	24.104
15611	451	67	61	P	S	74.113	2.490	0.000	2.490	-2.372	0.000	-2.372	60.000	24.104	24.104
15612	451	67	61	P	S	98.546	2.490	0.000	2.490	-2.372	0.000	-2.372	60.000	24.104	24.104
15613	451	67	61	P	W	14.113	2.490	0.000	2.490	-2.372	0.000	-2.372	60.000	24.104	24.104
15614	451	67	61	P	W	38.546	2.490	0.000	2.490	-2.372	0.000	-2.372	60.000	24.104	24.104
15615	451	67	61	P	W	81.454	2.490	0.000	2.490	-2.372	0.000	-2.372	60.000	24.104	24.104
15616	451	67	61	P	W	105.887	2.490	0.000	2.490	-2.372	0.000	-2.372	60.000	24.104	24.104
15617	456	75	52	C	S	16.102	-4.194	0.000	-4.194	4.578	0.000	4.578	60.000	23.839	23.839
15618	456	75	52	C	S	43.898	-4.194	0.000	-4.194	4.578	0.000	4.578	60.000	23.839	23.839
15619	456	75	52	C	S	76.102	-4.194	0.000	-4.194	4.578	0.000	4.578	60.000	23.839	23.839
15620	456	75	52	C	S	103.898	-4.194	0.000	-4.194	4.578	0.000	4.578	60.000	23.839	23.839
15621	456	75	52	P	S	16.102	-4.194	0.000	-4.194	4.578	0.000	4.578	60.000	23.839	23.839
15622	456	75	52	P	S	43.898	-4.194	0.000	-4.194	4.578	0.000	4.578	60.000	23.839	23.839
15623	456	75	52	P	S	76.102	-4.194	0.000	-4.194	4.578	0.000	4.578	60.000	23.839	23.839
15624	456	75	52	P	S	103.898	-4.194	0.000	-4.194	4.578	0.000	4.578	60.000	23.839	23.839
15625	457	67	63	C	S	6.890	3.343	0.000	3.343	-3.134	0.000	-3.134	60.000	24.304	24.304
15626	457	67	63	C	S	28.677	3.343	0.000	3.343	-3.134	0.000	-3.134	60.000	24.304	24.304
15627	457	67	63	C	S	31.323	3.343	0.000	3.343	-3.134	0.000	-3.134	60.000	24.304	24.304
15628	457	67	63	C	S	53.110	3.343	0.000	3.343	-3.134	0.000	-3.134	60.000	24.304	24.304
15629	457	67	63	C	S	66.890	3.343	0.000	3.343	-3.134	0.000	-3.134	60.000	24.304	24.304
15630	457	67	63	C	S	88.677	3.343	0.000	3.343	-3.134	0.000	-3.134	60.000	24.304	24.304
15631	457	67	63	C	S	91.323	3.343	0.000	3.343	-3.134	0.000	-3.134	60.000	24.304	24.304
15632	457	67	63	C	S	113.110	3.343	0.000	3.343	-3.134	0.000	-3.134	60.000	24.304	24.304
15633	457	67	63	C	W	6.890	3.343	0.000	3.343	-3.134	0.000	-3.134	60.000	24.304	24.304
15634	457	67	63	C	W	28.677	3.343	0.000	3.343	-3.134	0.000	-3.134	60.000	24.304	24.304
15635	457	67	63	C	W	31.323	3.343	0.000	3.343	-3.134	0.000	-3.134	60.000	24.304	24.304
15636	457	67	63	C	W	53.110	3.343	0.000	3.343	-3.134	0.000	-3.134	60.000	24.304	24.304
15637	457	67	63	C	W	66.890	3.343	0.000	3.343	-3.134	0.000	-3.134	60.000	24.304	24.304
15638	457	67	63	C	W	88.677	3.343	0.000	3.343	-3.134	0.000	-3.134	60.000	24.304	24.304
15639	457	67	63	C	W	91.323	3.343	0.000	3.343	-3.134	0.000	-3.134	60.000	24.304	24.304
15640	457	67	63	C	W	113.110	3.343	0.000	3.343	-3.134	0.000	-3.134	60.000	24.304	24.304
15641	460	67	64	C	S	12.216	3.765	0.000	3.765	-3.502	0.000	-3.502	60.000	24.403	24.403
15642	460	67	64	C	S	47.784	3.765	0.000	3.765	-3.502	0.000	-3.502	60.000	24.403	24.403
15643	460	67	64	C	S	72.216	3.765	0.000	3.765	-3.502	0.000	-3.502	60.000	24.403	24.403
15644	460	67	64	C	S	107.784	3.765	0.000	3.765	-3.502	0.000	-3.502	60.000	24.403	24.403
15645	460	67	64	C	W	12.216	3.765	0.000	3.765	-3.502	0.000	-3.502	60.000	24.403	24.403
15646	460	67	64	C	W	47.784	3.765	0.000	3.765	-3.502	0.000	-3.502	60.000	24.403	24.403
15647	460	67	64	C	W	72.216	3.765	0.000	3.765	-3.502	0.000	-3.502	60.000	24.403	24.403
15648	460	67	64	C	W	107.784	3.765	0.000	3.765	-3.502	0.000	-3.502	60.000	24.403	24.403
15649	460	67	64	P	S	12.216	3.765	0.000	3.765	-3.502	0.000	-3.502	60.000	24.403	24.403
15650	460	67	64	P	S	107.784	3.765	0.000	3.765	-3.502	0.000	-3.502	60.000	24.403	24.403
15651	460	67	64	P	W	47.784	3.765	0.000	3.765	-3.502	0.000	-3.502	60.000	24.403	24.403
15652	460	67	64	P	W	72.216	3.765	0.000	3.765	-3.502	0.000	-3.502	60.000	24.403	24.403
15653	463	73	57	C	S	0.769	-1.390	0.000	-1.390	1.430	0.000	1.430	60.000	24.207	24.207
15654	463	73	57	C	S	12.404	-1.390	0.000	-1.390	1.430	0.000	1.430	60.000	24.207	24.207
15655	463	73	57	C	S	47.596	-1.390	0.000	-1.390	1.430	0.000	1.430	60.000	24.207	24.207
15656	463	73	57	C	S	59.231	-1.390	0.000	-1.390	1.430	0.000	1.430	60.000	24.207	24.207
15657	463	73	57	C	S	60.769	-1.390	0.000	-1.390	1.430	0.000	1.430	60.000	24.207	24.207

BL number	Atoms	γ-PC unit cells	WS_2 unit cells	γ-PC origin atom	WS_2 origin atom	Twist-angle (°)	γ-PC strain 1 (%)	γ-PC strain 2 (%)	γ-PC strain 3 (%)	WS_2 strain 1 (%)	WS_2 strain 2 (%)	WS_2 strain 3 (%)	γ (°)	|**a**|(Å)	|**b**|(Å)
15658	463	73	57	C	S	72.404	-1.390	0.000	-1.390	1.430	0.000	1.430	60.000	24.207	24.207
15659	463	73	57	C	S	107.596	-1.390	0.000	-1.390	1.430	0.000	1.430	60.000	24.207	24.207
15660	463	73	57	C	S	119.231	-1.390	0.000	-1.390	1.430	0.000	1.430	60.000	24.207	24.207
15661	463	73	57	C	W	0.769	-1.390	0.000	-1.390	1.430	0.000	1.430	60.000	24.207	24.207
15662	463	73	57	C	W	12.404	-1.390	0.000	-1.390	1.430	0.000	1.430	60.000	24.207	24.207
15663	463	73	57	C	W	47.596	-1.390	0.000	-1.390	1.430	0.000	1.430	60.000	24.207	24.207
15664	463	73	57	C	W	59.231	-1.390	0.000	-1.390	1.430	0.000	1.430	60.000	24.207	24.207
15665	463	73	57	C	W	60.769	-1.390	0.000	-1.390	1.430	0.000	1.430	60.000	24.207	24.207
15666	463	73	57	C	W	72.404	-1.390	0.000	-1.390	1.430	0.000	1.430	60.000	24.207	24.207
15667	463	73	57	C	W	107.596	-1.390	0.000	-1.390	1.430	0.000	1.430	60.000	24.207	24.207
15668	463	73	57	C	W	119.231	-1.390	0.000	-1.390	1.430	0.000	1.430	60.000	24.207	24.207
15669	469	67	67	C	S	24.433	5.011	0.000	5.011	-4.554	0.000	-4.555	60.000	24.696	24.696
15670	469	67	67	C	S	35.567	5.011	0.000	5.011	-4.554	0.000	-4.555	60.000	24.696	24.696
15671	469	67	67	C	S	84.433	5.011	0.000	5.011	-4.554	0.000	-4.555	60.000	24.696	24.696
15672	469	67	67	C	S	95.567	5.011	0.000	5.011	-4.554	0.000	-4.555	60.000	24.696	24.696
15673	469	67	67	C	W	24.433	5.011	0.000	5.011	-4.554	0.000	-4.555	60.000	24.696	24.696
15674	469	67	67	C	W	35.567	5.011	0.000	5.011	-4.554	0.000	-4.555	60.000	24.696	24.696
15675	469	67	67	C	W	84.433	5.011	0.000	5.011	-4.554	0.000	-4.555	60.000	24.696	24.696
15676	469	67	67	C	W	95.567	5.011	0.000	5.011	-4.554	0.000	-4.555	60.000	24.696	24.696
15677	469	67	67	P	S	24.433	5.011	0.000	5.011	-4.554	0.000	-4.555	60.000	24.696	24.696
15678	469	67	67	P	S	95.567	5.011	0.000	5.011	-4.554	0.000	-4.555	60.000	24.696	24.696
15679	469	67	67	P	W	35.567	5.011	0.000	5.011	-4.554	0.000	-4.555	60.000	24.696	24.696
15680	469	67	67	P	W	84.433	5.011	0.000	5.011	-4.554	0.000	-4.555	60.000	24.696	24.696
15681	475	73	61	C	S	20.512	0.286	0.000	0.287	-0.285	0.000	-0.285	60.000	24.619	24.619
15682	475	73	61	C	S	27.853	0.286	0.000	0.287	-0.285	0.000	-0.285	60.000	24.619	24.619
15683	475	73	61	C	S	32.147	0.286	0.000	0.287	-0.285	0.000	-0.285	60.000	24.619	24.619
15684	475	73	61	C	S	39.488	0.286	0.000	0.287	-0.285	0.000	-0.285	60.000	24.619	24.619
15685	475	73	61	C	S	80.512	0.286	0.000	0.287	-0.285	0.000	-0.285	60.000	24.619	24.619
15686	475	73	61	C	S	87.853	0.286	0.000	0.287	-0.285	0.000	-0.285	60.000	24.619	24.619
15687	475	73	61	C	S	92.147	0.286	0.000	0.287	-0.285	0.000	-0.285	60.000	24.619	24.619
15688	475	73	61	C	S	99.488	0.286	0.000	0.287	-0.285	0.000	-0.285	60.000	24.619	24.619
15689	475	73	61	C	W	20.512	0.286	0.000	0.287	-0.285	0.000	-0.285	60.000	24.619	24.619
15690	475	73	61	C	W	27.853	0.286	0.000	0.287	-0.285	0.000	-0.285	60.000	24.619	24.619
15691	475	73	61	C	W	32.147	0.286	0.000	0.287	-0.285	0.000	-0.285	60.000	24.619	24.619
15692	475	73	61	C	W	39.488	0.286	0.000	0.287	-0.285	0.000	-0.285	60.000	24.619	24.619
15693	475	73	61	C	W	80.512	0.286	0.000	0.287	-0.285	0.000	-0.285	60.000	24.619	24.619
15694	475	73	61	C	W	87.853	0.286	0.000	0.287	-0.285	0.000	-0.285	60.000	24.619	24.619
15695	475	73	61	C	W	92.147	0.286	0.000	0.287	-0.285	0.000	-0.285	60.000	24.619	24.619
15696	475	73	61	C	W	99.488	0.286	0.000	0.287	-0.285	0.000	-0.285	60.000	24.619	24.619
15697	475	73	61	P	S	20.512	0.286	0.000	0.287	-0.285	0.000	-0.285	60.000	24.619	24.619
15698	475	73	61	P	S	32.147	0.286	0.000	0.287	-0.285	0.000	-0.285	60.000	24.619	24.619
15699	475	73	61	P	S	87.853	0.286	0.000	0.287	-0.285	0.000	-0.285	60.000	24.619	24.619
15700	475	73	61	P	S	99.488	0.286	0.000	0.287	-0.285	0.000	-0.285	60.000	24.619	24.619
15701	475	73	61	P	W	27.853	0.286	0.000	0.287	-0.285	0.000	-0.285	60.000	24.619	24.619
15702	475	73	61	P	W	39.488	0.286	0.000	0.287	-0.285	0.000	-0.285	60.000	24.619	24.619
15703	475	73	61	P	W	80.512	0.286	0.000	0.287	-0.285	0.000	-0.285	60.000	24.619	24.619
15704	475	73	61	P	W	92.147	0.286	0.000	0.287	-0.285	0.000	-0.285	60.000	24.619	24.619
15705	475	76	57	C	S	16.826	-2.359	0.000	-2.359	2.476	0.000	2.476	60.000	24.457	24.457
15706	475	76	57	C	S	43.174	-2.359	0.000	-2.359	2.476	0.000	2.476	60.000	24.457	24.457
15707	475	76	57	C	S	76.827	-2.359	0.000	-2.359	2.476	0.000	2.476	60.000	24.457	24.457
15708	475	76	57	C	S	103.174	-2.359	0.000	-2.359	2.476	0.000	2.476	60.000	24.457	24.457

BL number	Atoms	γ-PC unit cells	WS_2 unit cells	γ-PC origin atom	WS_2 origin atom	Twist-angle (°)	γ-PC strain 1 (%)	γ-PC strain 2 (%)	γ-PC strain 3 (%)	WS_2 strain 1 (%)	WS_2 strain 2 (%)	WS_2 strain 3 (%)	γ (°)	|**a**|(Å)	|**b**|(Å)
15709	475	76	57	C	W	16.826	-2.359	0.000	-2.359	2.476	0.000	2.476	60.000	24.457	24.457
15710	475	76	57	C	W	43.174	-2.359	0.000	-2.359	2.476	0.000	2.476	60.000	24.457	24.457
15711	475	76	57	C	W	76.827	-2.359	0.000	-2.359	2.476	0.000	2.476	60.000	24.457	24.457
15712	475	76	57	C	W	103.174	-2.359	0.000	-2.359	2.476	0.000	2.476	60.000	24.457	24.457
15713	476	74	60	C	S	4.715	-3.007	0.000	2.214	3.200	0.000	-2.120	90.000	16.951	30.941
15714	476	74	60	C	S	25.285	-4.781	0.000	4.263	5.287	0.000	-3.928	90.000	28.823	18.222
15715	476	74	60	C	S	34.715	-4.781	0.000	4.263	5.287	0.000	-3.928	90.000	16.641	31.561
15716	476	74	60	C	S	55.285	2.214	0.000	-3.007	-2.120	0.000	3.200	90.000	30.941	16.951
15717	476	74	60	C	S	64.715	-3.007	0.000	2.214	3.200	0.000	-2.120	90.000	16.951	30.941
15718	476	74	60	C	S	85.285	-4.781	0.000	4.263	5.287	0.000	-3.928	90.000	28.823	18.222
15719	476	74	60	C	S	94.715	-4.781	0.000	4.263	5.287	0.000	-3.928	90.000	16.641	31.561
15720	476	74	60	C	S	115.285	2.214	0.000	-3.007	-2.120	0.000	3.200	90.000	30.941	16.951
15721	476	74	60	C	W	4.715	-3.007	0.000	2.214	3.200	0.000	-2.120	90.000	16.951	30.941
15722	476	74	60	C	W	25.285	4.262	0.000	-4.781	-3.928	0.000	5.287	90.000	31.561	16.641
15723	476	74	60	C	W	34.715	-4.781	0.000	4.263	5.287	0.000	-3.928	90.000	16.641	31.561
15724	476	74	60	C	W	55.285	2.214	0.000	-3.007	-2.120	0.000	3.200	90.000	30.941	16.951
15725	476	74	60	C	W	64.715	-3.007	0.000	2.214	3.200	0.000	-2.120	90.000	16.951	30.941
15726	476	74	60	C	W	85.285	4.262	0.000	-4.781	-3.928	0.000	5.287	90.000	31.561	16.641
15727	476	74	60	C	W	94.715	-4.781	0.000	4.263	5.287	0.000	-3.928	90.000	16.641	31.561
15728	476	74	60	C	W	115.285	2.214	0.000	-3.007	-2.120	0.000	3.200	90.000	30.941	16.951
15729	476	74	60	P	S	4.715	-3.007	0.000	2.214	3.200	0.000	-2.120	90.000	16.951	30.941
15730	476	74	60	P	S	34.715	4.262	0.000	-4.781	-3.928	0.000	5.287	90.000	18.222	28.823
15731	476	74	60	P	S	55.285	2.214	0.000	-3.007	-2.120	0.000	3.200	90.000	30.941	16.951
15732	476	74	60	P	S	64.715	-3.007	0.000	2.214	3.200	0.000	-2.120	90.000	16.951	30.941
15733	476	74	60	P	S	85.285	-4.781	0.000	4.263	5.287	0.000	-3.928	90.000	28.823	18.222
15734	476	74	60	P	S	94.715	4.262	0.000	-4.781	-3.928	0.000	5.287	90.000	31.561	16.641
15735	476	74	60	P	S	115.285	2.214	0.000	-3.007	-2.120	0.000	3.200	90.000	30.941	16.951
15736	476	74	60	P	W	4.715	-3.007	0.000	2.214	3.200	0.000	-2.120	90.000	16.951	30.941
15737	476	74	60	P	W	25.285	-4.781	0.000	4.263	5.287	0.000	-3.928	90.000	28.823	18.222
15738	476	74	60	P	W	55.285	2.214	0.000	-3.007	-2.120	0.000	3.200	90.000	30.941	16.951
15739	476	74	60	P	W	64.715	-3.007	0.000	2.214	3.200	0.000	-2.120	90.000	16.951	30.941
[truncated: 304,267 more chars]
