# Supplementary material for: Systematic DFT Modeling van der Waals Heterostructures from a Complete Configurational Basis Applied to γ-PC/WS2
Source: J Chem Theory Comput. 2024 Mar 6;20(6):2377–89. doi: 10.1021/acs.jctc.3c00932 (PMC10976641; doi:10.1021/acs.jctc.3c00932)
Supplement: Supplementary file 3 — ct3c00932_si_003.pdf [file ct3c00932_si_003.pdf]

BL number	Atoms	h-BN cells	graphene cells	h-BN origin	graphene origin	Twist-angle (°)	h-BN Strain 1 (%)	h-BN Strain 2 (%)	h-BN Strain 3 (%)	graphene Strain 1 (%)	graphene Strain 2 (%)	graphene Strain 3 (%)	γ (°)	|**a**|(Å)	|**b**|(Å)
1	4	1	1	B	C	0	-0.889	0	-0.889	0.906	0	0.906	60	2.49	2.49
2	4	1	1	B	C	60	-0.889	0	-0.889	0.906	0	0.906	60	2.49	2.49
3	28	7	7	B	C	38.213	-0.889	0	-0.889	0.906	0	0.906	60	6.587	6.587
4	28	7	7	B	C	98.213	-0.889	0	-0.889	0.906	0	0.906	60	6.587	6.587
5	28	7	7	B	C	21.787	-0.889	0	-0.889	0.906	0	0.906	60	6.587	6.587
6	28	7	7	B	C	81.787	-0.889	0	-0.889	0.906	0	0.906	60	6.587	6.587
7	34	8	9	B	C	42.103	-0.889	3.576	5.249	0.906	-3.236	-4.751	51.943	6.582	8.799
8	34	8	9	B	C	102.103	-0.889	3.576	5.249	0.906	-3.236	-4.751	51.943	6.582	8.799
9	34	8	9	B	C	21.787	-0.889	3.576	5.249	0.906	-3.236	-4.751	80.358	6.587	7.022
10	34	8	9	B	C	81.787	-0.889	3.576	5.249	0.906	-3.236	-4.751	80.358	6.587	7.022
11	34	8	9	B	C	100.893	5.686	1.271	-1.275	-5.106	-1.304	1.308	80.497	7.024	6.584
12	40	10	10	B	C	16.102	-2.816	-4.208	1.116	2.984	4.116	-1.091	68.085	8.802	6.576
13	40	10	10	B	C	76.102	-2.816	-4.208	1.116	2.984	4.116	-1.091	68.085	8.802	6.576
14	40	10	10	B	C	16.102	1.116	-4.042	-2.816	-1.092	4.283	2.984	68.099	8.799	6.578
15	40	10	10	B	C	43.898	1.116	4.042	-2.816	-1.092	-4.283	2.984	68.099	8.799	6.578
16	40	10	10	B	C	76.102	1.116	-4.042	-2.816	-1.092	4.283	2.984	68.099	8.799	6.578
17	40	10	10	B	C	103.898	1.116	4.042	-2.816	-1.092	-4.283	2.984	68.099	8.799	6.578
18	40	10	10	B	C	40.893	1.974	-1.051	-3.595	-1.899	1.133	3.873	68.044	8.795	6.586
19	40	10	10	B	C	79.107	1.974	1.051	-3.595	-1.899	-1.133	3.873	68.044	6.586	8.795
20	40	10	10	B	C	100.893	1.974	-1.051	-3.595	-1.899	1.133	3.873	68.044	8.795	6.586
21	40	10	10	B	C	79.107	1.974	1.051	-3.595	-1.899	-1.133	3.873	68.044	6.586	8.795
22	40	10	10	N	C	81.052	1.974	1.051	-3.595	-1.899	-1.133	3.873	68.044	6.586	8.795
23	40	10	10	N	C	98.948	1.974	-1.051	-3.595	-1.899	1.133	3.873	68.044	8.795	6.586
24	44	11	11	B	C	25.693	-3.287	1.603	1.631	3.518	-1.552	-1.579	89.982	6.581	8.977
25	44	11	11	B	C	85.693	-3.287	1.603	1.631	3.518	-1.552	-1.579	89.982	6.581	8.977
26	44	11	11	B	C	32.204	-0.889	-5.202	-0.889	0.906	5.296	0.906	90	8.976	6.578
27	44	11	11	B	C	92.204	-0.889	-5.202	-0.889	0.906	5.296	0.906	90	8.976	6.578
28	44	10	12	B	C	46.102	4.483	5.278	3.121	-4.114	-4.968	-2.938	72.487	8.807	7.01
29	44	10	12	B	C	106.102	4.483	5.278	3.121	-4.114	-4.968	-2.938	72.487	8.807	7.01
30	44	11	11	B	C	21.787	-0.889	-5.202	-0.889	0.906	5.296	0.906	90	6.587	8.964
31	44	11	11	B	C	81.787	-0.889	-5.202	-0.889	0.906	5.296	0.906	90	6.587	8.964
32	44	10	12	B	C	40.893	5.686	-4.068	1.974	-5.106	3.913	-1.899	72.301	7.024	8.801
33	44	10	12	B	C	100.893	5.686	-4.068	1.974	-5.106	3.913	-1.899	72.301	7.024	8.801
34	44	11	11	B	C	25.693	1.631	1.524	-3.286	-1.579	-1.631	3.518	89.969	8.976	6.582
35	44	11	11	B	C	85.693	1.631	1.524	-3.286	-1.579	-1.631	3.518	89.969	8.976	6.582
36	44	10	12	B	C	13.898	4.483	-5.278	3.121	-4.114	4.968	-2.938	72.487	7.01	8.807
37	44	10	12	B	C	73.898	4.483	-5.278	3.121	-4.114	4.968	-2.938	72.487	7.01	8.807
38	46	11	12	B	C	19.107	5.686	2.773	-2.751	-5.106	-2.935	2.911	82.924	6.59	9.447
39	46	11	12	B	C	79.107	5.686	2.773	-2.751	-5.106	-2.935	2.911	82.924	6.59	9.447
40	46	11	12	B	C	40.893	5.686	-2.773	-2.751	-5.106	2.935	2.911	88.366	7.024	8.799
41	46	11	12	B	C	100.893	5.686	-2.773	-2.751	-5.106	2.935	2.911	88.366	7.024	8.799
42	46	11	12	N	C	109.842	5.686	-2.773	-2.751	-5.106	2.935	2.911	82.924	9.447	6.59

43	50	13	12	B	C	21.052	-4.167	-3.547	-1.425	4.546	3.651	1.467	78.204	6.592	10.4
44	50	13	12	B	C	81.052	-4.167	-3.547	-1.425	4.546	3.651	1.467	78.204	6.592	10.4
45	50	13	12	B	C	25.693	-3.287	4.408	-2.341	3.518	-4.625	2.456	78.379	10.4	6.587
46	50	13	12	B	C	85.693	-3.287	4.408	-2.341	3.518	-4.625	2.456	78.379	10.4	6.587
47	50	13	12	B	C	16.102	-2.816	0	-2.816	2.984	0	2.984	60	8.802	8.802
48	50	13	12	B	C	76.102	-2.816	0	-2.816	2.984	0	2.984	60	8.802	8.802
49	50	12	13	B	C	21.787	-0.889	-2.384	3.203	0.906	2.241	-3.01	78.254	6.587	10.401
50	50	13	12	B	C	21.787	-0.889	-2.201	-4.667	0.906	2.427	5.148	78.263	6.587	10.408
51	50	12	13	B	C	81.787	-0.889	-2.384	3.203	0.906	2.241	-3.01	78.254	6.587	10.401
52	50	13	12	B	C	81.787	-0.889	-2.201	-4.667	0.906	2.427	5.148	78.263	6.587	10.408
53	50	13	12	B	C	36.587	-4.933	-0.528	-0.6	5.473	0.534	0.607	78.301	10.409	6.586
54	50	13	12	B	C	96.587	-4.933	-0.528	-0.6	5.473	0.534	0.607	78.301	10.409	6.586
55	50	12	13	B	C	25.693	1.631	4.54	0.606	-1.579	-4.486	-0.599	78.37	6.585	10.396
56	50	12	13	B	C	85.693	1.631	4.54	0.606	-1.579	-4.486	-0.599	78.37	6.585	10.396
57	50	12	13	B	C	21.052	2.623	-3.584	-0.348	-2.492	3.609	0.35	78.232	10.395	6.59
58	50	12	13	B	C	38.948	2.623	3.584	-0.348	-2.492	-3.609	0.35	78.232	6.59	10.395
59	50	12	13	B	C	81.052	2.623	-3.584	-0.348	-2.492	3.609	0.35	78.232	10.395	6.59
60	50	12	13	B	C	98.948	2.623	3.584	-0.348	-2.492	-3.609	0.35	78.232	6.59	10.395
61	50	13	12	B	C	23.413	-4.933	0.528	-0.6	5.473	-0.534	0.607	78.301	10.409	6.586
62	50	13	12	B	C	83.413	-4.933	0.528	-0.6	5.473	-0.534	0.607	78.301	10.409	6.586
63	50	12	13	B	C	23.413	3.517	0.524	-1.178	-3.286	-0.537	1.206	78.311	10.401	6.586
64	50	12	13	B	C	36.587	3.517	-0.524	-1.178	-3.286	0.537	1.206	78.311	10.401	6.586
65	50	12	13	B	C	83.413	3.517	0.524	-1.178	-3.286	-0.537	1.206	78.311	10.401	6.586
66	50	12	13	B	C	96.587	3.517	-0.524	-1.178	-3.286	0.537	1.206	78.311	10.401	6.586
67	50	13	12	N	C	31.626	-4.933	0.528	-0.6	5.473	-0.534	0.607	78.301	10.409	6.586
68	50	13	12	N	C	75.2	-4.933	0.528	-0.6	5.473	-0.534	0.607	78.301	10.409	6.586
69	50	13	12	N	C	93.74	-4.167	3.547	-1.425	4.546	-3.651	1.467	78.204	10.4	6.592
70	52	13	13	B	C	32.204	-0.889	0	-0.889	0.906	0	0.906	60	8.976	8.977
71	52	13	13	B	C	92.204	-0.889	0	-0.889	0.906	0	0.906	60	8.976	8.977
72	54	12	15	B	C	40.893	5.686	4.237	4.139	-5.106	-3.913	-3.823	81.211	7.024	10.397
73	54	12	15	B	C	100.893	5.686	4.237	4.139	-5.106	-3.913	-3.823	81.211	7.024	10.397
74	56	14	14	B	C	42.103	-0.889	-4.087	-0.889	0.906	4.161	0.906	76.066	8.799	8.799
75	56	14	14	B	C	102.103	-0.889	-4.087	-0.889	0.906	4.161	0.906	76.066	8.799	8.799
76	56	13	15	B	C	19.107	5.686	-4.694	-0.025	-5.106	4.696	0.025	78.52	11.622	6.591
77	56	13	15	B	C	79.107	5.686	-4.694	-0.025	-5.106	4.696	0.025	78.52	11.622	6.591
78	56	14	14	B	C	16.102	-2.816	1.002	1.116	2.984	-0.98	-1.091	76.087	8.802	8.799
79	56	14	14	B	C	76.102	-2.816	1.002	1.116	2.984	-0.98	-1.091	76.087	8.802	8.799
80	56	13	15	B	C	40.893	5.686	4.694	-0.025	-5.106	-4.696	0.025	74.268	7.024	11.102
81	56	13	15	B	C	100.893	5.686	4.694	-0.025	-5.106	-4.696	0.025	74.268	7.024	11.102
82	56	14	14	B	C	13.174	-0.889	-4.087	-0.889	0.906	4.161	0.906	76.161	8.798	8.798
83	56	14	14	B	C	73.174	-0.889	-4.087	-0.889	0.906	4.161	0.906	76.161	8.798	8.798
84	56	14	14	B	C	43.898	1.116	-0.962	-2.816	-1.092	1.02	2.984	76.096	8.799	8.802
85	56	14	14	B	C	76.102	1.116	0.962	-2.816	-1.092	-1.02	2.984	76.096	8.799	8.802
86	56	14	14	B	C	103.898	1.116	-0.962	-2.816	-1.092	1.02	2.984	76.096	8.799	8.802

87	56	14	14	B	C	106.826	-0.889	4.087	-0.889	0.906	-4.161	0.906	76.161	8.798	8.798
88	56	14	14	B	C	46.826	-0.889	4.087	-0.889	0.906	-4.161	0.906	76.161	8.798	8.798
89	58	13	16	B	C	46.102	4.483	0	4.483	-4.114	0	-4.114	60	9.463	9.463
90	58	13	16	B	C	106.102	4.483	0	4.483	-4.114	0	-4.114	60	9.463	9.463
91	58	14	15	B	C	40.893	5.686	-5.085	-3.595	-5.106	5.479	3.873	86.981	7.024	11.113
92	58	14	15	B	C	40.893	5.686	5.085	-3.595	-5.106	-5.479	3.873	86.981	7.024	11.113
93	58	14	15	B	C	100.893	5.686	-5.085	-3.595	-5.106	5.479	3.873	86.981	7.024	11.113
94	58	14	15	B	C	100.893	5.686	5.085	-3.595	-5.106	-5.479	3.873	86.981	7.024	11.113
95	58	13	16	B	C	13.898	4.483	0	4.483	-4.114	0	-4.114	60	9.463	9.463
96	58	13	16	B	C	73.898	4.483	0	4.483	-4.114	0	-4.114	60	9.463	9.463
97	60	16	14	B	C	12.52	-3.287	-3.306	-4.823	3.518	3.659	5.338	74.996	8.803	9.466
98	60	16	14	B	C	72.52	-3.287	-3.306	-4.823	3.518	3.659	5.338	74.996	8.803	9.466
99	60	15	15	B	C	32.204	-0.889	3.815	-0.889	0.906	-3.884	0.906	90	8.976	8.97
100	60	15	15	B	C	92.204	-0.889	3.815	-0.889	0.906	-3.884	0.906	90	8.976	8.97
101	60	14	16	B	C	46.102	4.483	1.077	0.592	-4.114	-1.065	-0.585	75.012	9.463	8.796
102	60	14	16	B	C	106.102	4.483	1.077	0.592	-4.114	-1.065	-0.585	75.012	9.463	8.796
103	60	14	16	B	C	47.48	1.631	3.592	3.387	-1.579	-3.365	-3.172	75.013	8.797	9.46
104	60	14	16	B	C	107.48	1.631	3.592	3.387	-1.579	-3.365	-3.172	75.013	8.797	9.46
105	60	14	16	B	C	16.102	1.116	2.887	3.925	-1.092	-2.677	-3.639	74.969	8.799	9.461
106	60	14	16	B	C	43.898	1.116	-2.887	3.925	-1.092	2.677	-3.639	74.969	8.799	9.461
107	60	14	16	B	C	76.102	1.116	2.887	3.925	-1.092	-2.677	-3.639	74.969	8.799	9.461
108	60	14	16	B	C	103.898	1.116	-2.887	3.925	-1.092	2.677	-3.639	74.969	8.799	9.461
109	60	14	16	B	C	13.898	4.483	-1.077	0.592	-4.114	1.065	-0.585	75.012	9.463	8.796
110	60	14	16	B	C	73.898	4.483	-1.077	0.592	-4.114	1.065	-0.585	75.012	9.463	8.796
111	60	15	15	B	C	30	1.037	0	-2.743	-1.016	0	2.903	89.98	8.975	8.975
112	60	15	15	B	C	90	1.037	0	-2.743	-1.016	0	2.903	89.98	8.975	8.975
113	62	16	15	B	C	21.052	-4.167	0.576	-0.666	4.546	-0.584	0.675	80.496	12.811	6.587
114	62	16	15	B	C	81.052	-4.167	0.576	-0.666	4.546	-0.584	0.675	80.496	12.811	6.587
115	62	15	16	B	C	16.102	-2.816	1.87	4.524	2.984	-1.715	-4.148	88.975	8.802	9.461
116	62	15	16	B	C	76.102	-2.816	1.87	4.524	2.984	-1.715	-4.148	88.975	8.802	9.461
117	62	15	16	B	C	46.102	4.483	2.011	-2.781	-4.114	-2.129	2.945	88.815	9.463	8.801
118	62	15	16	B	C	106.102	4.483	2.011	-2.781	-4.114	-2.129	2.945	88.815	9.463	8.801
119	62	16	15	B	C	40.893	-3.595	2.783	-1.275	3.873	-2.856	1.308	80.4	12.814	6.586
120	62	16	15	B	C	100.893	-3.595	2.783	-1.275	3.873	-2.856	1.308	80.4	12.814	6.586
121	62	15	16	B	C	21.787	-0.889	1.907	2.385	0.906	-1.821	-2.276	80.482	6.587	12.804
122	62	16	15	B	C	81.787	-0.889	1.788	-3.959	0.906	-1.942	4.299	80.487	6.587	12.811
123	62	15	16	B	C	81.787	-0.889	1.907	2.385	0.906	-1.821	-2.276	80.482	6.587	12.804
124	62	15	16	B	C	38.948	2.623	-0.573	-1.112	-2.492	0.587	1.137	80.468	12.805	6.587
125	62	15	16	B	C	81.052	2.623	0.573	-1.112	-2.492	-0.586	1.137	80.468	6.587	12.805
126	62	15	16	B	C	98.948	2.623	-0.573	-1.112	-2.492	0.587	1.137	80.468	12.805	6.587
127	62	15	16	B	C	13.898	4.483	-2.011	-2.781	-4.114	2.129	2.945	88.815	9.463	8.801
128	62	15	16	B	C	73.898	4.483	-2.011	-2.781	-4.114	2.129	2.945	88.815	9.463	8.801
129	62	15	16	B	C	40.893	1.974	2.804	-0.501	-1.899	-2.832	0.506	80.404	12.808	6.586
130	62	15	16	B	C	79.107	1.974	-2.804	-0.501	-1.899	2.832	0.506	80.404	12.808	6.586

131	62	15	16	B	C	100.893	1.974	2.804	-0.501	-1.899	-2.832	0.506	80.404	12.808	6.586
132	62	15	16	B	C	79.107	1.974	-2.804	-0.501	-1.899	2.832	0.506	80.404	12.808	6.586
133	62	15	16	N	C	81.052	1.974	-2.804	-0.501	-1.899	2.832	0.506	80.404	12.808	6.586
134	62	15	16	N	C	98.948	1.974	2.804	-0.501	-1.899	-2.832	0.506	80.404	12.808	6.586
135	62	16	15	N	C	93.74	-4.167	-0.576	-0.666	4.546	0.584	0.675	80.496	6.587	12.811
136	64	16	16	B	C	36.587	-4.933	-4.288	3.517	5.473	4.006	-3.286	70.392	10.409	8.775
137	64	16	16	B	C	96.587	-4.933	-4.288	3.517	5.473	4.006	-3.286	70.392	10.409	8.775
138	64	16	16	B	C	23.413	-4.933	4.288	3.517	5.473	-4.006	-3.286	70.392	10.409	8.775
139	64	16	16	B	C	83.413	-4.933	4.288	3.517	5.473	-4.006	-3.286	70.392	10.409	8.775
140	64	16	16	B	C	23.413	3.517	3.932	-4.933	-3.286	-4.362	5.473	70.42	10.401	8.78
141	64	16	16	B	C	36.587	3.517	-3.932	-4.933	-3.286	4.362	5.473	70.42	10.401	8.78
142	64	16	16	B	C	83.413	3.517	3.932	-4.933	-3.286	-4.362	5.473	70.42	10.401	8.78
143	64	16	16	B	C	96.587	3.517	-3.932	-4.933	-3.286	4.362	5.473	70.42	10.401	8.78
144	64	16	16	N	C	31.626	-4.933	4.288	3.517	5.473	-4.006	-3.286	70.392	10.409	8.775
145	64	16	16	N	C	75.2	-4.933	4.288	3.517	5.473	-4.006	-3.286	70.392	10.409	8.775
146	66	17	16	B	C	12.52	-3.287	-2.852	-1.406	3.518	2.935	1.447	68.084	10.857	8.794
147	66	17	16	B	C	72.52	-3.287	-2.852	-1.406	3.518	2.935	1.447	68.084	10.857	8.794
148	66	17	16	B	C	38.213	-0.889	5.049	-3.778	0.906	-5.462	4.087	71.727	10.406	8.966
149	66	16	17	B	C	38.213	-0.889	5.364	2.18	0.906	-5.14	-2.089	71.756	8.962	10.403
150	66	16	17	B	C	98.213	-0.889	5.364	2.18	0.906	-5.14	-2.089	71.756	8.962	10.403
151	66	17	16	B	C	98.213	-0.889	5.049	-3.778	0.906	-5.462	4.087	71.727	10.406	8.966
152	66	17	16	B	C	16.102	-2.816	3.3	-1.891	2.984	-3.43	1.965	68.016	8.802	10.852
153	66	17	16	B	C	76.102	-2.816	3.3	-1.891	2.984	-3.43	1.965	68.016	8.802	10.852
154	66	16	17	B	C	32.204	-0.889	-5.364	2.18	0.906	5.14	-2.089	71.608	8.976	10.395
155	66	17	16	B	C	32.204	-0.889	-5.049	-3.778	0.906	5.462	4.087	71.617	8.976	10.4
156	66	16	17	B	C	92.204	-0.889	-5.364	2.18	0.906	5.14	-2.089	71.608	8.976	10.395
157	66	17	16	B	C	92.204	-0.889	-5.049	-3.778	0.906	5.462	4.087	71.617	8.976	10.4
158	66	16	17	B	C	21.787	-0.889	-5.364	2.18	0.906	5.14	-2.089	84.999	6.587	13.494
159	66	17	16	B	C	21.787	-0.889	-5.049	-3.778	0.906	5.462	4.087	85.002	6.587	13.501
160	66	16	17	B	C	81.787	-0.889	-5.364	2.18	0.906	5.14	-2.089	84.999	6.587	13.494
161	66	17	16	B	C	81.787	-0.889	-5.049	-3.778	0.906	5.462	4.087	85.002	6.587	13.501
162	66	15	18	B	C	40.893	5.686	0.678	1.974	-5.106	-0.652	-1.899	79.087	7.024	12.809
163	66	15	18	B	C	100.893	5.686	0.678	1.974	-5.106	-0.652	-1.899	79.087	7.024	12.809
164	66	17	16	B	C	36.587	-4.933	2.422	0.369	5.473	-2.404	-0.366	71.752	10.409	8.967
165	66	17	16	B	C	96.587	-4.933	2.422	0.369	5.473	-2.404	-0.366	71.752	10.409	8.967
166	66	16	17	B	C	13.174	-0.889	-1.788	2.18	0.906	1.714	-2.089	68.075	10.852	8.795
167	66	17	16	B	C	13.174	-0.889	-1.683	-3.778	0.906	1.821	4.087	68.086	10.852	8.8
168	66	16	17	B	C	73.174	-0.889	-1.788	2.18	0.906	1.714	-2.089	68.075	10.852	8.795
169	66	16	17	B	C	47.48	1.631	2.881	-0.367	-1.579	-2.903	0.37	68.066	10.854	8.793
170	66	16	17	B	C	107.48	1.631	2.881	-0.367	-1.579	-2.903	0.37	68.066	10.854	8.793
171	66	16	17	B	C	16.102	1.116	3.368	0.133	-1.092	-3.359	-0.133	68.02	8.799	10.85
172	66	16	17	B	C	43.898	1.116	-3.368	0.133	-1.092	3.359	-0.133	68.02	8.799	10.85
173	66	16	17	B	C	76.102	1.116	3.368	0.133	-1.092	-3.359	-0.133	68.02	8.799	10.85
174	66	16	17	B	C	103.898	1.116	-3.368	0.133	-1.092	3.359	-0.133	68.02	8.799	10.85

175	66	15	18	B	C	21.052	2.623	2.867	4.999	-2.492	-2.606	-4.545	79.115	12.81	7.021
176	66	15	18	B	C	38.948	2.623	-2.867	4.999	-2.492	2.606	-4.545	79.115	7.021	12.81
177	66	15	18	B	C	81.052	2.623	2.867	4.999	-2.492	-2.606	-4.545	79.115	12.81	7.021
178	66	15	18	B	C	98.948	2.623	-2.867	4.999	-2.492	2.606	-4.545	79.115	7.021	12.81
179	66	17	16	B	C	23.413	-4.933	-2.422	0.369	5.473	2.404	-0.366	71.752	10.409	8.967
180	66	17	16	B	C	83.413	-4.933	-2.422	0.369	5.473	2.404	-0.366	71.752	10.409	8.967
181	66	17	16	B	C	106.826	-0.889	1.683	-3.778	0.906	-1.82	4.087	68.086	10.852	8.8
182	66	17	16	B	C	46.826	-0.889	1.683	-3.778	0.906	-1.821	4.087	68.086	10.852	8.8
183	66	16	17	B	C	46.826	-0.889	1.788	2.18	0.906	-1.714	-2.089	68.075	10.852	8.795
184	66	16	17	B	C	21.787	-0.889	-5.364	2.18	0.906	5.14	-2.089	71.756	10.403	8.962
185	66	17	16	B	C	21.787	-0.889	-5.049	-3.778	0.906	5.462	4.087	71.727	8.966	10.406
186	66	16	17	B	C	23.413	3.517	-2.359	-2.116	-3.286	2.463	2.21	71.769	10.401	8.968
187	66	16	17	B	C	36.587	3.517	2.359	-2.116	-3.286	-2.463	2.21	71.769	10.401	8.968
188	66	16	17	B	C	83.413	3.517	-2.359	-2.116	-3.286	2.463	2.21	71.769	10.401	8.968
189	66	16	17	B	C	96.587	3.517	2.359	-2.116	-3.286	-2.463	2.21	71.769	10.401	8.968
190	68	17	17	B	C	42.103	-0.889	3.366	-0.889	0.906	-3.427	0.906	90	13.862	6.583
191	68	17	17	B	C	102.103	-0.889	3.366	-0.889	0.906	-3.427	0.906	90	13.862	6.583
192	68	17	17	B	C	25.693	-3.287	-4.668	1.631	3.518	4.52	-1.579	66.306	11.133	8.957
193	68	17	17	B	C	85.693	-3.287	-4.668	1.631	3.518	4.52	-1.579	66.306	11.133	8.957
194	68	17	17	B	C	21.787	-0.889	3.366	-0.889	0.906	-3.427	0.906	90	6.587	13.854
195	68	17	17	B	C	81.787	-0.889	3.366	-0.889	0.906	-3.427	0.906	90	6.587	13.854
196	68	17	17	B	C	25.693	1.631	-4.438	-3.286	-1.579	4.75	3.518	66.325	11.128	8.96
197	68	17	17	B	C	85.693	1.631	-4.438	-3.286	-1.579	4.75	3.518	66.325	11.128	8.96
198	70	17	18	B	C	19.107	5.686	-1.795	-4.141	-5.106	1.957	4.515	74.337	11.104	8.803
199	70	17	18	B	C	79.107	5.686	-1.795	-4.141	-5.106	1.957	4.515	74.337	11.104	8.803
200	70	17	18	B	C	40.893	5.686	1.795	-4.141	-5.106	-1.957	4.515	88.927	7.024	13.402
201	70	17	18	B	C	100.893	5.686	1.795	-4.141	-5.106	-1.957	4.515	88.927	7.024	13.402
202	70	19	16	B	C	36.587	-4.933	0	-4.933	5.473	0	5.473	60	10.409	10.409
203	70	19	16	B	C	96.587	-4.933	0	-4.933	5.473	0	5.473	60	10.409	10.409
204	70	19	16	B	C	23.413	-4.933	0	-4.933	5.473	0	5.473	60	10.409	10.409
205	70	19	16	B	C	83.413	-4.933	0	-4.933	5.473	0	5.473	60	10.409	10.409
206	70	16	19	B	C	23.413	3.517	0	3.517	-3.286	0	-3.286	60	10.401	10.401
207	70	16	19	B	C	36.587	3.517	0	3.517	-3.286	0	-3.286	60	10.401	10.401
208	70	16	19	B	C	83.413	3.517	0	3.517	-3.286	0	-3.286	60	10.401	10.401
209	70	16	19	B	C	96.587	3.517	0	3.517	-3.286	0	-3.286	60	10.401	10.401
210	70	17	18	N	C	109.842	5.686	1.795	-4.141	-5.106	-1.957	4.515	74.337	8.803	11.104
211	70	17	18	N	C	28.055	5.686	1.795	-4.141	-5.106	-1.957	4.515	74.337	8.803	11.104
212	72	18	18	B	C	12.52	-3.287	-2.449	1.631	3.518	2.372	-1.579	80.963	11.133	8.793
213	72	18	18	B	C	72.52	-3.287	-2.449	1.631	3.518	2.372	-1.579	80.963	11.133	8.793
214	72	18	18	B	C	16.102	-2.816	3.896	1.116	2.984	-3.811	-1.091	80.807	8.802	11.125
215	72	16	20	B	C	46.102	4.483	-3.299	5.335	-4.114	2.981	-4.82	72.377	9.463	10.669
216	72	16	20	B	C	106.102	4.483	-3.299	5.335	-4.114	2.981	-4.82	72.377	9.463	10.669
217	72	18	18	B	C	10.893	-1.774	-5.251	0.012	1.84	5.25	-0.012	80.972	11.132	8.789
218	72	18	18	B	C	49.107	0.012	5.155	-1.774	-0.012	-5.345	1.84	80.954	11.132	8.79

219	72	18	18	B	C	70.893	0.012	-5.155	-1.774	-0.012	5.345	1.84	80.954	8.79	11.132
220	72	18	18	B	C	109.107	0.012	5.155	-1.774	-0.012	-5.345	1.84	80.954	11.132	8.79
221	72	18	18	B	C	16.102	1.116	3.742	-2.816	-1.092	-3.966	2.984	80.813	8.799	11.128
222	72	18	18	B	C	43.898	1.116	-3.742	-2.816	-1.092	3.966	2.984	80.813	8.799	11.128
223	72	18	18	B	C	76.102	1.116	3.742	-2.816	-1.092	-3.966	2.984	80.813	8.799	11.128
224	72	18	18	B	C	103.898	1.116	-3.742	-2.816	-1.092	3.966	2.984	80.813	8.799	11.128
225	72	18	18	B	C	38.213	-1.774	5.251	0.012	1.84	-5.25	-0.012	80.972	8.789	11.132
226	72	16	20	B	C	13.898	4.483	3.299	5.335	-4.114	-2.981	-4.82	72.377	9.463	10.669
227	72	16	20	B	C	73.898	4.483	3.299	5.335	-4.114	-2.981	-4.82	72.377	9.463	10.669
228	72	18	18	N	C	98.213	-1.774	5.251	0.012	1.84	-5.25	-0.012	80.972	8.789	11.132
229	74	19	18	B	C	21.052	-4.167	3.397	-0.147	4.546	-3.407	0.147	81.999	8.788	11.417
230	74	19	18	B	C	81.052	-4.167	3.397	-0.147	4.546	-3.407	0.147	81.999	8.788	11.417
231	74	19	18	B	C	31.945	-3.326	-1.993	-1.045	3.563	2.036	1.068	83.788	8.979	11.127
232	74	19	18	B	C	91.945	-3.326	-1.993	-1.045	3.563	2.036	1.068	83.788	8.979	11.127
233	74	19	18	B	C	25.693	-3.287	-2.088	-1.087	3.518	2.134	1.111	83.709	11.133	8.976
234	74	19	18	B	C	85.693	-3.287	-2.088	-1.087	3.518	2.134	1.111	83.709	11.133	8.976
235	74	19	18	B	C	38.213	-0.889	-4.517	-3.474	0.906	4.855	3.734	82.008	11.409	8.792
236	74	18	19	B	C	38.213	-0.889	-4.768	1.839	0.906	4.599	-1.774	82.004	11.409	8.788
237	74	19	18	B	C	98.213	-0.889	-4.517	-3.474	0.906	4.855	3.734	82.008	11.409	8.792
238	74	18	19	B	C	98.213	-0.889	-4.768	1.839	0.906	4.599	-1.774	82.004	11.409	8.788
239	74	20	17	B	C	109.107	-4.99	2.939	-4.454	5.542	-3.226	4.889	70.248	9.462	11.141
240	74	17	20	B	C	49.107	3.584	3.166	2.954	-3.344	-2.99	-2.789	70.243	11.133	9.456
241	74	17	20	B	C	109.107	3.584	3.166	2.954	-3.344	-2.99	-2.789	70.243	11.133	9.456
242	74	19	18	B	C	16.102	-2.816	-5.168	-1.574	2.984	5.336	1.626	81.889	8.802	11.397
243	74	19	18	B	C	76.102	-2.816	-5.168	-1.574	2.984	5.336	1.626	81.889	8.802	11.397
244	74	18	19	B	C	32.204	-0.889	-1.589	1.839	0.906	1.533	-1.774	83.765	8.976	11.126
245	74	19	18	B	C	32.204	-0.889	-1.506	-3.474	0.906	1.618	3.734	83.768	8.976	11.131
246	74	18	19	B	C	92.204	-0.889	-1.589	1.839	0.906	1.533	-1.774	83.765	8.976	11.126
247	74	19	18	B	C	92.204	-0.889	-1.506	-3.474	0.906	1.618	3.734	83.768	8.976	11.131
248	74	17	20	B	C	46.102	4.483	-2.218	2.08	-4.114	2.129	-1.997	70.168	9.463	11.13
249	74	17	20	B	C	106.102	4.483	-2.218	2.08	-4.114	2.129	-1.997	70.168	9.463	11.13
250	74	20	17	B	C	10.893	-4.99	-2.939	-4.454	5.543	3.226	4.889	70.248	11.141	9.462
251	74	18	19	B	C	25.693	1.631	-2.096	-0.691	-1.579	2.125	0.701	83.711	11.128	8.975
252	74	17	20	B	C	47.48	1.631	0.247	4.957	-1.579	-0.224	-4.51	70.232	11.128	9.463
253	74	18	19	B	C	85.693	1.631	-2.096	-0.691	-1.579	2.125	0.701	83.711	11.128	8.975
254	74	17	20	B	C	107.48	1.631	0.247	4.957	-1.579	-0.224	-4.51	70.232	11.128	9.463
255	74	18	19	B	C	16.102	1.116	-5.239	-0.195	-1.092	5.26	0.196	81.891	8.799	11.396
256	74	18	19	B	C	43.898	1.116	5.239	-0.195	-1.092	-5.26	0.196	81.891	8.799	11.396
257	74	18	19	B	C	76.102	1.116	-5.239	-0.195	-1.092	5.26	0.196	81.891	8.799	11.396
258	74	18	19	B	C	103.898	1.116	5.239	-0.195	-1.092	-5.26	0.196	81.891	8.799	11.396
259	74	18	19	B	C	21.052	2.623	3.345	-1.621	-2.492	-3.457	1.675	81.959	11.414	8.787
260	74	18	19	B	C	38.948	2.623	-3.345	-1.621	-2.492	3.457	1.675	81.959	8.787	11.414
261	74	18	19	B	C	81.052	2.623	3.345	-1.621	-2.492	-3.457	1.675	81.959	11.414	8.787
262	74	18	19	B	C	98.948	2.623	-3.345	-1.621	-2.492	3.457	1.675	81.959	8.787	11.414

263	74	17	20	B	C	13.898	4.483	2.218	2.08	-4.114	-2.129	-1.997	70.168	9.463	11.13
264	74	17	20	B	C	73.898	4.483	2.218	2.08	-4.114	-2.129	-1.997	70.168	9.463	11.13
265	74	19	18	B	C	21.787	-0.889	4.517	-3.474	0.906	-4.855	3.734	82.008	11.409	8.792
266	74	18	19	B	C	21.787	-0.889	4.768	1.839	0.906	-4.599	-1.774	82.004	11.409	8.788
267	74	17	20	B	C	10.893	3.584	-3.166	2.954	-3.344	2.99	-2.789	70.243	9.456	11.133
268	74	17	20	B	C	70.893	3.584	-3.166	2.954	-3.344	2.99	-2.789	70.243	9.456	11.133
269	74	19	18	B	C	49.842	-4.167	-3.397	-0.147	4.546	3.407	0.147	81.999	11.417	8.788
270	74	20	17	B	C	70.893	-4.99	-2.939	-4.454	5.543	3.226	4.889	70.248	11.141	9.462
271	74	20	17	B	C	109.107	-4.99	2.939	-4.454	5.542	-3.226	4.889	70.248	9.462	11.141
272	74	18	19	N	C	33.811	1.675	1.999	-0.733	-1.62	-2.029	0.744	83.765	8.978	11.124
273	76	20	18	B	C	70.158	-3.326	-5.083	-3.493	3.563	5.465	3.755	83.135	10.858	9.459
274	76	20	18	B	C	70.158	-3.326	-5.083	-3.493	3.563	5.465	3.755	83.135	10.858	9.459
275	76	18	20	B	C	49.107	3.584	3.987	0.012	-3.344	-3.986	-0.012	76.548	11.924	8.787
276	76	18	20	B	C	109.107	3.584	3.987	0.012	-3.344	-3.986	-0.012	76.548	11.924	8.787
277	76	20	18	B	C	16.102	-2.816	4.909	-3.996	2.984	-5.336	4.343	76.446	8.802	11.917
278	76	20	18	B	C	76.102	-2.816	4.91	-3.996	2.984	-5.336	4.343	76.446	8.802	11.917
279	76	18	20	B	C	46.102	4.483	-1.257	-0.814	-4.114	1.277	0.827	83.017	9.463	10.853
280	76	18	20	B	C	49.842	1.675	5.365	1.86	-1.62	-5.173	-1.793	83.137	10.853	9.454
281	76	20	18	B	C	10.893	-4.99	-3.918	-1.774	5.543	4.062	1.84	76.54	11.933	8.789
282	76	18	20	B	C	13.174	-0.889	0	4.567	0.906	0	-4.185	83.067	10.852	9.463
283	76	19	19	B	C	13.174	-0.889	0	-0.889	0.906	0	0.906	60	10.852	10.852
284	76	19	19	B	C	73.174	-0.889	0	-0.889	0.906	0	0.906	60	10.852	10.852
285	76	18	20	B	C	10.893	0.012	-4.124	3.584	-0.012	3.848	-3.344	76.474	11.924	8.79
286	76	18	20	B	C	49.107	0.012	4.124	3.584	-0.012	-3.849	-3.344	76.474	8.79	11.924
287	76	18	20	B	C	70.893	0.012	-4.124	3.584	-0.012	3.848	-3.344	76.474	11.924	8.79
288	76	18	20	B	C	109.107	0.012	4.124	3.584	-0.012	-3.848	-3.344	76.474	8.79	11.924
289	76	18	20	B	C	16.102	1.116	5.239	2.427	-1.092	-4.997	-2.314	76.443	8.799	11.91
290	76	18	20	B	C	43.898	1.116	-5.239	2.427	-1.092	4.997	-2.314	76.443	8.799	11.91
291	76	18	20	B	C	76.102	1.116	5.239	2.427	-1.092	-4.997	-2.314	76.443	8.799	11.91
292	76	18	20	B	C	103.898	1.116	-5.239	2.427	-1.092	4.997	-2.314	76.443	8.799	11.91
293	76	18	20	B	C	106.826	-0.889	0	4.567	0.906	0	-4.185	83.067	10.852	9.463
294	76	19	19	B	C	106.826	-0.889	0	-0.889	0.906	0	0.906	60	10.852	10.852
295	76	19	19	B	C	46.826	-0.889	0	-0.889	0.906	0	0.906	60	10.852	10.852
296	76	18	20	B	C	13.898	4.483	1.257	-0.814	-4.114	-1.278	0.827	83.017	9.463	10.853
297	76	18	20	B	C	10.893	3.584	-3.987	0.012	-3.344	3.986	-0.012	76.548	11.924	8.787
298	76	18	20	B	C	70.893	3.584	-3.987	0.012	-3.344	3.986	-0.012	76.548	11.924	8.787
299	76	20	18	B	C	70.893	-4.99	-3.918	-1.774	5.543	4.062	1.84	76.54	11.933	8.789
300	76	20	18	B	C	109.107	-4.99	3.918	-1.774	5.542	-4.062	1.84	76.54	11.933	8.789
301	76	20	18	B	C	109.107	-4.99	3.918	-1.774	5.542	-4.062	1.84	76.54	11.933	8.789
302	78	21	18	B	C	21.052	-4.167	4.83	-4.895	4.546	-5.355	5.426	77.65	12.165	8.802
303	78	19	20	B	C	12.52	-3.287	-2.088	4.348	3.518	1.921	-4	84.034	11.133	9.463
304	78	19	20	B	C	72.52	-3.287	-2.088	4.348	3.518	1.921	-4	84.034	11.133	9.463
305	78	19	20	B	C	49.107	3.584	4.721	-2.62	-3.344	-4.983	2.765	87.224	11.924	8.794
306	78	19	20	B	C	109.107	3.584	4.721	-2.62	-3.344	-4.983	2.765	87.224	11.924	8.794

307	78	18	21	B	C	19.107	5.686	1.695	0.53	-5.106	-1.677	-0.524	77.637	12.166	8.794
308	78	18	21	B	C	79.107	5.686	1.695	0.53	-5.106	-1.677	-0.524	77.637	12.166	8.794
309	78	19	20	B	C	16.102	-2.816	4.43	3.806	2.984	-4.116	-3.537	87.557	8.802	11.911
310	78	19	20	B	C	76.102	-2.816	4.43	3.806	2.984	-4.116	-3.537	87.557	8.802	11.911
311	78	19	20	B	C	46.102	4.483	-0.397	-3.402	-4.114	0.426	3.651	84.136	9.463	11.132
312	78	19	20	B	C	106.102	4.483	-0.397	-3.402	-4.114	0.426	3.651	84.136	9.463	11.132
313	78	20	19	B	C	109.107	-4.99	4.898	0.905	5.542	-4.811	-0.889	87.221	11.933	8.791
314	78	20	19	B	C	10.893	-4.99	-4.898	0.905	5.543	4.811	-0.889	87.221	11.933	8.791
315	78	18	21	B	C	100.893	5.686	-1.695	0.53	-5.106	1.677	-0.524	77.637	7.024	15.232
316	78	18	21	B	C	16.102	1.116	-3.742	5.048	-1.092	3.399	-4.585	77.545	8.799	12.161
317	78	18	21	B	C	43.898	1.116	3.742	5.048	-1.092	-3.399	-4.585	77.545	8.799	12.161
318	78	18	21	B	C	76.102	1.116	-3.742	5.048	-1.092	3.399	-4.585	77.545	8.799	12.161
319	78	18	21	B	C	103.898	1.116	3.742	5.048	-1.092	-3.399	-4.585	77.545	8.799	12.161
320	78	18	21	B	C	21.052	2.623	5.256	3.472	-2.492	-4.915	-3.246	77.657	8.795	12.156
321	78	18	21	B	C	38.948	2.623	-5.256	3.472	-2.492	4.915	-3.246	77.657	12.156	8.795
322	78	18	21	B	C	81.052	2.623	5.256	3.472	-2.492	-4.915	-3.246	77.657	8.795	12.156
323	78	18	21	B	C	98.948	2.623	-5.256	3.472	-2.492	4.915	-3.246	77.657	12.156	8.795
324	78	19	20	B	C	13.898	4.483	0.397	-3.402	-4.114	-0.426	3.651	84.136	9.463	11.132
325	78	19	20	B	C	73.898	4.483	0.397	-3.402	-4.114	-0.426	3.651	84.136	9.463	11.132
326	78	19	20	B	C	10.893	3.584	-4.721	-2.62	-3.344	4.983	2.765	87.224	11.924	8.794
327	78	19	20	B	C	70.893	3.584	-4.721	-2.62	-3.344	4.983	2.765	87.224	11.924	8.794
328	78	21	18	B	C	49.842	-4.167	-4.83	-4.895	4.546	5.355	5.426	77.65	8.802	12.165
329	78	20	19	B	C	70.893	-4.99	-4.898	0.905	5.543	4.811	-0.889	87.221	11.933	8.791
330	78	20	19	B	C	109.107	-4.99	4.898	0.905	5.542	-4.811	-0.889	87.221	11.933	8.791
331	78	21	18	N	C	10.158	-4.167	4.83	-4.895	4.546	-5.355	5.426	77.65	12.165	8.802
332	78	18	21	N	C	109.842	5.686	-1.695	0.53	-5.106	1.677	-0.524	77.637	12.166	8.794
333	80	21	19	B	C	12.52	-3.287	0	-3.286	3.518	0	3.518	60	11.133	11.133
334	80	21	19	B	C	25.693	-3.287	0	-3.286	3.518	0	3.518	60	11.133	11.133
335	80	20	20	B	C	36.587	-4.933	-1.029	3.517	5.473	0.962	-3.286	83.384	10.409	10.402
336	80	20	20	B	C	96.587	-4.933	-1.029	3.517	5.473	0.962	-3.286	83.384	10.409	10.402
337	80	19	21	B	C	25.693	1.631	0	1.631	-1.579	0	-1.579	60	11.128	11.128
338	80	19	21	B	C	47.48	1.631	0	1.631	-1.579	0	-1.579	60	11.128	11.128
339	80	19	21	B	C	85.693	1.631	0	1.631	-1.579	0	-1.579	60	11.128	11.128
340	80	19	21	B	C	107.48	1.631	0	1.631	-1.579	0	-1.579	60	11.128	11.128
341	80	20	20	B	C	23.413	-4.933	1.029	3.517	5.473	-0.962	-3.286	83.384	10.409	10.402
342	80	20	20	B	C	83.413	-4.933	1.029	3.517	5.473	-0.962	-3.286	83.384	10.409	10.402
343	80	20	20	B	C	23.413	3.517	0.944	-4.933	-3.286	-1.047	5.473	83.394	10.401	10.41
344	80	20	20	B	C	36.587	3.517	-0.944	-4.933	-3.286	1.047	5.473	83.394	10.401	10.41
345	80	20	20	B	C	83.413	3.517	0.944	-4.933	-3.286	-1.047	5.473	83.394	10.401	10.41
346	80	20	20	B	C	96.587	3.517	-0.944	-4.933	-3.286	1.047	5.473	83.394	10.401	10.41
347	82	20	21	B	C	21.052	-4.167	4.15	5.254	4.546	-3.755	-4.754	78.293	10.817	10.408
348	82	20	21	B	C	81.052	-4.167	4.15	5.254	4.546	-3.755	-4.754	78.293	10.817	10.408
349	82	21	20	B	C	31.945	-3.326	0.949	-0.786	3.563	-0.964	0.799	75.036	12.691	8.978
350	82	21	20	B	C	70.158	-3.326	-4.936	-0.786	3.563	5.015	0.799	73.538	13.065	8.785

351	82	21	20	B	C	91.945	-3.326	0.949	-0.786	3.563	-0.964	0.799	75.036	12.691	8.978
352	82	21	20	B	C	70.158	-3.326	-4.936	-0.786	3.563	5.015	0.799	73.538	13.065	8.785
353	82	20	21	B	C	25.693	-3.287	-0.22	4.212	3.518	0.203	-3.885	72.069	11.133	10.399
354	82	20	21	B	C	85.693	-3.287	-0.22	4.212	3.518	0.203	-3.885	72.069	11.133	10.399
355	82	21	20	B	C	16.102	-2.816	5.344	-1.318	2.984	-5.488	1.354	73.415	8.802	13.046
356	82	21	20	B	C	76.102	-2.816	5.344	-1.318	2.984	-5.488	1.354	73.415	8.802	13.046
357	82	21	20	B	C	32.204	-0.889	1.362	-3.228	0.906	-1.456	3.451	75.024	8.976	12.693
358	82	20	21	B	C	32.204	-0.889	1.431	1.566	0.906	-1.387	-1.519	75.018	8.976	12.687
359	82	20	21	B	C	92.204	-0.889	1.431	1.566	0.906	-1.387	-1.519	75.018	8.976	12.687
360	82	20	21	B	C	10.893	-1.774	-3.781	2.512	1.84	3.6	-2.392	73.562	13.056	8.788
361	82	20	21	B	C	70.893	-1.774	-3.781	2.512	1.84	3.6	-2.392	73.562	13.056	8.788
362	82	20	21	B	C	49.842	1.675	4.923	-0.992	-1.62	-5.023	1.013	73.515	13.061	8.785
363	82	20	21	B	C	91.945	1.675	0.947	-0.992	-1.62	-0.966	1.013	75.012	8.977	12.687
364	82	20	21	B	C	109.842	1.675	4.923	-0.992	-1.62	-5.023	1.013	73.515	13.061	8.785
365	82	21	20	B	C	36.587	-4.933	4.247	0.969	5.473	-4.167	-0.95	71.902	10.409	11.133
366	82	21	20	B	C	96.587	-4.933	4.247	0.969	5.473	-4.167	-0.95	71.902	10.409	11.133
367	82	21	20	B	C	30	-2.743	2.406	-1.393	2.903	-2.475	1.433	74.973	12.695	8.976
368	82	21	20	B	C	30	-2.743	-2.406	-1.393	2.903	2.475	1.433	74.973	12.695	8.976
369	82	21	20	B	C	90	-2.743	-2.406	-1.393	2.903	2.475	1.433	74.973	12.695	8.976
370	82	21	20	B	C	90	-2.743	2.406	-1.393	2.903	-2.475	1.433	74.973	12.695	8.976
371	82	21	20	B	C	10.893	0.012	-3.535	-4.071	-0.012	3.848	4.432	73.573	13.054	8.793
372	82	21	20	B	C	49.107	0.012	3.535	-4.071	-0.012	-3.849	4.432	73.574	13.054	8.793
373	82	21	20	B	C	70.893	0.012	-3.535	-4.071	-0.012	3.848	4.432	73.574	13.054	8.793
374	82	21	20	B	C	109.107	0.012	3.535	-4.071	-0.012	-3.848	4.432	73.574	13.054	8.793
375	82	20	21	B	C	16.102	1.116	5.389	-0.457	-1.092	-5.439	0.461	73.419	8.799	13.045
376	82	20	21	B	C	43.898	1.116	-5.389	-0.457	-1.092	5.439	0.461	73.419	8.799	13.045
377	82	20	21	B	C	76.102	1.116	5.389	-0.457	-1.092	-5.439	0.461	73.419	8.799	13.045
378	82	20	21	B	C	103.898	1.116	-5.389	-0.457	-1.092	5.439	0.461	73.419	8.799	13.045
379	82	21	20	B	C	23.413	-4.933	-4.247	0.969	5.473	4.167	-0.95	71.902	10.409	11.133
380	82	21	20	B	C	83.413	-4.933	-4.247	0.969	5.473	4.167	-0.95	71.902	10.409	11.133
381	82	20	21	B	C	38.213	-1.774	3.781	2.512	1.84	-3.6	-2.392	73.562	13.056	8.788
382	82	20	21	B	C	49.842	-4.167	-4.15	5.254	4.546	3.755	-4.754	78.293	10.408	10.817
383	82	20	21	B	C	30	1.037	-2.431	-0.38	-1.016	2.449	0.383	74.977	12.69	8.975
384	82	20	21	B	C	30	1.037	2.431	-0.38	-1.016	-2.449	0.383	74.977	12.69	8.975
385	82	20	21	B	C	90	1.037	-2.431	-0.38	-1.016	2.449	0.383	74.977	12.69	8.975
386	82	20	21	B	C	90	1.037	2.431	-0.38	-1.016	-2.449	0.383	74.977	12.69	8.975
387	82	20	21	B	C	23.413	3.517	-4.089	-2.68	-3.286	4.321	2.831	71.921	10.401	11.135
388	82	20	21	B	C	36.587	3.517	4.089	-2.68	-3.286	-4.321	2.831	71.921	10.401	11.135
389	82	20	21	B	C	83.413	3.517	-4.089	-2.68	-3.286	4.321	2.831	71.921	10.401	11.135
390	82	20	21	B	C	96.587	3.517	4.089	-2.68	-3.286	-4.321	2.831	71.921	10.401	11.135
391	82	20	21	N	C	24.791	3.517	4.089	-2.68	-3.286	-4.321	2.831	71.921	10.401	11.135
392	82	20	21	N	C	10.158	-4.167	4.15	5.254	4.546	-3.755	-4.754	78.293	10.817	10.408
393	82	21	20	N	C	31.626	-4.933	-4.247	0.969	5.473	4.167	-0.95	71.902	10.409	11.133
394	82	20	21	N	C	98.213	-1.774	3.781	2.512	1.84	-3.6	-2.392	73.562	13.056	8.788

395	82	20	21	N	C	33.811	1.675	-0.947	-0.992	-1.62	0.966	1.013	75.012	12.687	8.977
396	82	20	21	N	C	93.74	-4.167	-4.15	5.254	4.546	3.755	-4.754	78.293	10.408	10.817
397	84	20	22	B	C	38.213	-0.889	0	4.022	0.906	0	-3.722	71.734	11.409	10.4
398	84	20	22	B	C	98.213	-0.889	0	4.022	0.906	0	-3.722	71.734	11.409	10.4
399	84	22	20	B	C	16.102	-2.816	-2.55	-3.531	2.984	2.744	3.799	88.486	8.802	12.81
400	84	22	20	B	C	76.102	-2.816	-2.55	-3.531	2.984	2.744	3.799	88.486	8.802	12.81
401	84	22	20	B	C	40.893	-3.595	-2.53	-2.751	3.873	2.677	2.911	88.51	12.814	8.799
402	84	22	20	B	C	40.893	-3.595	4.554	-2.751	3.873	-4.819	2.911	71.794	10.394	11.416
403	84	22	20	B	C	100.893	-3.595	-2.53	-2.751	3.873	2.677	2.911	88.51	12.814	8.799
404	84	22	20	B	C	100.893	-3.595	4.554	-2.751	3.873	-4.819	2.911	71.794	10.394	11.416
405	84	22	20	B	C	36.587	-4.933	-2.807	-1.348	5.473	2.884	1.385	71.638	10.409	11.413
406	84	22	20	B	C	96.587	-4.933	-2.807	-1.348	5.473	2.884	1.386	71.638	10.409	11.413
407	84	20	22	B	C	16.102	1.116	-2.694	1.902	-1.092	2.596	-1.833	88.485	8.799	12.803
408	84	20	22	B	C	43.898	1.116	2.694	1.902	-1.092	-2.596	-1.833	88.485	8.799	12.803
409	84	20	22	B	C	76.102	1.116	-2.694	1.902	-1.092	2.596	-1.833	88.485	8.799	12.803
410	84	20	22	B	C	103.898	1.116	2.694	1.902	-1.092	-2.596	-1.833	88.485	8.799	12.803
411	84	22	20	B	C	23.413	-4.933	2.807	-1.348	5.473	-2.884	1.385	71.638	10.409	11.413
412	84	22	20	B	C	83.413	-4.933	2.807	-1.348	5.473	-2.884	1.386	71.638	10.409	11.413
413	84	22	20	B	C	12.52	-3.595	2.53	-2.751	3.873	-2.677	2.911	88.51	12.814	8.799
414	84	20	22	B	C	21.787	-0.889	0	4.022	0.906	0	-3.722	71.734	11.409	10.4
415	84	20	22	B	C	40.893	1.974	4.731	1.046	-1.899	-4.634	-1.024	71.785	11.411	10.39
416	84	20	22	B	C	40.893	1.974	-2.628	1.046	-1.899	2.575	-1.024	88.51	12.808	8.796
417	84	20	22	B	C	79.107	1.974	-4.731	1.046	-1.899	4.634	-1.024	71.786	10.39	11.411
418	84	20	22	B	C	79.107	1.974	2.628	1.046	-1.899	-2.575	-1.024	88.51	12.808	8.796
419	84	20	22	B	C	100.893	1.974	4.731	1.046	-1.899	-4.634	-1.024	71.786	11.411	10.39
420	84	20	22	B	C	100.893	1.974	-2.628	1.046	-1.899	2.575	-1.024	88.51	12.808	8.796
421	84	20	22	B	C	79.107	1.974	-4.731	1.046	-1.899	4.634	-1.024	71.786	10.39	11.411
422	84	20	22	B	C	79.107	1.974	2.628	1.046	-1.899	-2.575	-1.024	88.51	12.808	8.796
423	84	20	22	B	C	23.413	3.517	2.831	-0.426	-3.286	-2.855	0.43	71.649	10.401	11.411
424	84	20	22	B	C	36.587	3.517	-2.831	-0.426	-3.286	2.855	0.43	71.649	10.401	11.411
425	84	20	22	B	C	83.413	3.517	2.831	-0.426	-3.286	-2.855	0.43	71.649	10.401	11.411
426	84	20	22	B	C	96.587	3.517	-2.831	-0.426	-3.286	2.855	0.43	71.649	10.401	11.411
427	84	20	22	N	C	81.052	1.974	-4.731	1.046	-1.899	4.634	-1.024	71.786	10.39	11.411
428	84	20	22	N	C	81.052	1.974	2.628	1.046	-1.899	-2.575	-1.024	88.51	12.808	8.796
429	84	20	22	N	C	98.948	1.974	-2.628	1.046	-1.899	2.575	-1.024	88.51	12.808	8.796
430	84	22	20	N	C	75.2	-4.933	2.807	-1.348	5.473	-2.884	1.386	71.638	10.409	11.413
431	86	22	21	B	C	21.052	-4.167	5.449	0.231	4.546	-5.424	-0.23	78.42	13.403	8.795
432	86	22	21	B	C	81.052	-4.167	5.449	0.231	4.546	-5.424	-0.23	78.42	13.403	8.795
433	86	22	21	B	C	12.52	-3.287	0.2	-0.716	3.518	-0.203	0.727	72.84	11.133	10.852
434	86	22	21	B	C	72.52	-3.287	0.2	-0.716	3.518	-0.203	0.727	72.84	11.133	10.852
435	86	19	24	B	C	19.107	5.686	4.817	4.709	-5.106	-4.403	-4.304	87.366	12.166	9.454
436	86	19	24	B	C	79.107	5.686	4.817	4.709	-5.106	-4.403	-4.304	87.366	12.166	9.454
437	86	21	22	B	C	16.102	-2.816	-3.34	3.55	2.984	3.118	-3.315	78.692	8.802	13.38
438	86	21	22	B	C	76.102	-2.816	-3.34	3.55	2.984	3.118	-3.315	78.692	8.802	13.38

439	86	22	21	B	C	10.893	-1.774	-2.578	-2.261	1.84	2.7	2.369	72.865	10.852	11.129
440	86	22	21	B	C	70.893	-1.774	-2.578	-2.261	1.84	2.7	2.369	72.865	10.852	11.129
441	86	23	20	B	C	36.587	-4.933	2.088	-3.463	5.473	-2.244	3.721	84.528	10.409	11.132
442	86	23	20	B	C	96.587	-4.933	2.088	-3.463	5.473	-2.244	3.721	84.528	10.409	11.132
443	86	21	22	B	C	13.174	-0.889	1.362	1.449	0.906	-1.324	-1.408	72.835	10.852	11.128
444	86	21	22	B	C	73.174	-0.889	1.362	1.449	0.906	-1.324	-1.408	72.835	10.852	11.128
445	86	20	23	B	C	25.693	1.631	1.886	3.721	-1.579	-1.755	-3.463	84.575	11.128	10.4
446	86	21	22	B	C	47.48	1.631	-0.2	-1.062	-1.579	0.204	1.085	72.848	11.128	10.852
447	86	20	23	B	C	85.693	1.631	1.886	3.721	-1.579	-1.755	-3.463	84.575	11.128	10.4
448	86	21	22	B	C	107.48	1.631	-0.2	-1.062	-1.579	0.204	1.085	72.848	11.128	10.852
449	86	21	22	B	C	10.893	0.012	-2.651	0.522	-0.012	2.624	-0.517	72.87	11.127	10.85
450	86	21	22	B	C	49.107	0.012	2.651	0.522	-0.012	-2.624	-0.517	72.87	10.85	11.127
451	86	21	22	B	C	70.893	0.012	-2.651	0.522	-0.012	2.624	-0.517	72.87	11.127	10.85
452	86	21	22	B	C	109.107	0.012	2.651	0.522	-0.012	-2.624	-0.517	72.87	10.85	11.127
453	86	23	20	B	C	23.413	-4.933	-2.088	-3.463	5.473	2.244	3.721	84.528	10.409	11.132
454	86	23	20	B	C	83.413	-4.933	-2.088	-3.463	5.473	2.244	3.721	84.528	10.409	11.132
455	86	22	21	B	C	106.826	-0.889	-1.3	-3.122	0.906	1.387	3.33	72.842	10.852	11.132
456	86	22	21	B	C	46.826	-0.889	-1.3	-3.122	0.906	1.387	3.33	72.842	10.852	11.132
457	86	22	21	B	C	49.842	-4.167	-5.449	0.231	4.546	5.424	-0.23	78.42	13.403	8.795
458	86	20	23	B	C	23.413	3.517	-2.202	1.827	-3.286	2.124	-1.763	84.53	10.401	11.127
459	86	20	23	B	C	36.587	3.517	2.202	1.827	-3.286	-2.124	-1.763	84.53	10.401	11.127
460	86	20	23	B	C	83.413	3.517	-2.202	1.827	-3.286	2.124	-1.763	84.53	10.401	11.127
461	86	20	23	B	C	96.587	3.517	2.202	1.827	-3.286	-2.124	-1.763	84.53	10.401	11.127
462	86	22	21	N	C	10.158	-4.167	5.449	0.231	4.546	-5.424	-0.23	78.42	13.403	8.795
463	86	23	20	N	C	31.626	-4.933	-2.088	-3.463	5.473	2.244	3.721	84.528	10.409	11.132
464	86	19	24	N	C	109.842	5.686	-4.817	4.709	-5.106	4.403	-4.304	87.366	12.166	9.454
465	88	20	24	B	C	51.052	4.687	4.387	2.923	-4.285	-4.145	-2.762	71.415	13.149	9.449
466	88	20	24	B	C	68.948	4.687	-4.387	2.923	-4.285	4.145	-2.762	71.415	13.149	9.449
467	88	20	24	B	C	111.052	4.687	4.387	2.923	-4.285	-4.145	-2.762	71.415	13.149	9.449
468	88	20	24	B	C	68.948	4.687	-4.387	2.923	-4.285	4.145	-2.762	71.415	13.149	9.449
469	88	22	22	B	C	70.158	-3.326	-4.802	1.675	3.563	4.647	-1.62	84.149	13.521	8.786
470	88	22	22	B	C	70.158	-3.326	-4.802	1.675	3.563	4.647	-1.62	84.149	13.521	8.786
471	88	20	24	B	C	51.052	2.923	4.457	4.687	-2.762	-4.075	-4.285	82.625	11.129	10.669
472	88	20	24	B	C	111.052	2.923	4.457	4.687	-2.762	-4.075	-4.285	82.625	11.129	10.669
473	88	20	24	B	C	19.107	5.686	-3.051	1.974	-5.106	2.935	-1.899	68.747	12.166	10.388
474	88	20	24	B	C	79.107	5.686	-3.051	1.974	-5.106	2.935	-1.899	68.747	12.166	10.388
475	88	20	24	B	C	46.102	4.483	-4.524	3.121	-4.114	4.258	-2.938	71.409	9.463	13.129
476	88	20	24	B	C	106.102	4.483	-4.524	3.121	-4.114	4.258	-2.938	71.409	9.463	13.129
477	88	22	22	B	C	49.842	1.675	4.562	-3.326	-1.62	-4.887	3.563	84.154	13.515	8.789
478	88	22	22	B	C	109.842	1.675	4.562	-3.326	-1.62	-4.887	3.563	84.154	13.515	8.789
479	88	20	24	B	C	13.898	4.483	4.524	3.121	-4.114	-4.258	-2.938	71.409	9.463	13.129
480	88	20	24	B	C	73.898	4.483	4.524	3.121	-4.114	-4.258	-2.938	71.409	9.463	13.129
481	88	20	24	B	C	23.413	3.517	4.718	4.08	-3.286	-4.362	-3.773	68.701	10.401	12.151
482	88	20	24	B	C	36.587	3.517	-4.718	4.08	-3.286	4.362	-3.773	68.701	10.401	12.151

483	88	20	24	B	C	83.413	3.517	4.718	4.08	-3.286	-4.362	-3.773	68.701	10.401	12.151
484	88	20	24	B	C	83.413	3.517	4.718	4.08	-3.286	-4.362	-3.773	68.701	10.401	12.151
485	88	20	24	B	C	96.587	3.517	-4.718	4.08	-3.286	4.362	-3.773	68.701	10.401	12.151
486	88	20	24	N	C	10.893	2.923	-4.457	4.687	-2.762	4.075	-4.285	82.625	10.669	11.129
487	90	20	25	B	C	51.052	4.687	2.681	5.128	-4.285	-2.431	-4.651	70.907	11.929	10.67
488	90	20	25	B	C	68.948	4.687	-2.681	5.128	-4.285	2.431	-4.651	70.908	10.67	11.929
489	90	20	25	B	C	111.052	4.687	2.681	5.128	-4.285	-2.431	-4.651	70.908	11.929	10.67
490	90	20	25	B	C	68.948	4.687	-2.681	5.128	-4.285	2.431	-4.651	70.908	10.67	11.929
491	90	23	22	B	C	31.945	-3.326	3.38	-0.572	3.563	-3.419	0.579	84.969	13.521	8.97
492	90	23	22	B	C	91.945	-3.326	3.38	-0.572	3.563	-3.419	0.579	84.969	13.521	8.97
493	90	22	23	B	C	42.103	-0.889	-1.3	1.343	0.906	1.266	-1.308	81.965	13.862	8.798
494	90	23	22	B	C	42.103	-0.889	-1.244	-3.025	0.906	1.324	3.219	81.969	13.862	8.801
495	90	22	23	B	C	102.103	-0.889	-1.3	1.343	0.906	1.266	-1.308	81.965	13.862	8.798
496	90	23	22	B	C	102.103	-0.889	-1.244	-3.025	0.906	1.324	3.219	81.969	13.862	8.801
497	90	21	24	B	C	51.052	2.923	5.188	2.083	-2.762	-4.98	-2	77.68	13.062	9.449
498	90	21	24	B	C	111.052	2.923	5.188	2.083	-2.762	-4.98	-2	77.68	13.062	9.449
499	90	23	22	B	C	16.102	-2.816	-1.83	-1.107	2.984	1.871	1.132	81.927	8.802	13.861
500	90	23	22	B	C	76.102	-2.816	-1.83	-1.107	2.984	1.871	1.132	81.927	8.802	13.861
501	90	23	22	B	C	32.204	-0.889	3.732	-3.025	0.906	-3.972	3.219	84.869	8.976	13.512
502	90	22	23	B	C	32.204	-0.889	3.902	1.343	0.906	-3.799	-1.308	84.867	8.976	13.506
503	90	23	22	B	C	92.204	-0.889	3.732	-3.025	0.906	-3.972	3.219	84.869	8.976	13.512
504	90	22	23	B	C	92.204	-0.889	3.902	1.343	0.906	-3.799	-1.308	84.867	8.976	13.506
505	90	21	24	B	C	46.102	4.483	-3.591	0.592	-4.114	3.549	-0.585	77.521	9.463	13.054
506	90	21	24	B	C	106.102	4.483	-3.591	0.592	-4.114	3.549	-0.585	77.521	9.463	13.054
507	90	22	23	B	C	31.945	1.675	3.357	-1.205	-1.62	-3.44	1.234	84.971	13.515	8.97
508	90	22	23	B	C	91.945	1.675	3.357	-1.205	-1.62	-3.44	1.234	84.971	13.515	8.97
509	90	21	24	B	C	10.893	0.012	-1.768	5.115	-0.012	1.604	-4.64	77.669	13.054	9.46
510	90	21	24	B	C	49.107	0.012	1.768	5.115	-0.012	-1.604	-4.64	77.669	13.054	9.46
511	90	21	24	B	C	70.893	0.012	-1.768	5.115	-0.012	1.604	-4.64	77.669	13.054	9.46
512	90	21	24	B	C	109.107	0.012	1.768	5.115	-0.012	-1.604	-4.64	77.669	13.054	9.46
513	90	22	23	B	C	16.102	1.116	-1.837	-0.671	-1.092	1.862	0.681	81.929	8.799	13.86
514	90	22	23	B	C	43.898	1.116	1.837	-0.671	-1.092	-1.862	0.681	81.929	8.799	13.86
515	90	22	23	B	C	76.102	1.116	-1.837	-0.671	-1.092	1.862	0.681	81.929	8.799	13.86
516	90	22	23	B	C	103.898	1.116	1.837	-0.671	-1.092	-1.862	0.681	81.929	8.799	13.86
517	90	21	24	B	C	13.898	4.483	3.591	0.592	-4.114	-3.549	-0.585	77.521	9.463	13.054
518	90	21	24	B	C	73.898	4.483	3.591	0.592	-4.114	-3.549	-0.585	77.521	9.463	13.054
519	90	22	23	N	C	33.811	1.675	-3.357	-1.205	-1.62	3.44	1.234	84.971	13.515	8.97
520	90	21	24	N	C	10.893	2.923	-5.188	2.083	-2.762	4.98	-2	77.68	9.449	13.062
521	92	23	23	B	C	12.52	-3.287	0.383	1.631	3.518	-0.371	-1.579	85.686	11.133	11.128
522	92	23	23	B	C	72.52	-3.287	0.383	1.631	3.518	-0.371	-1.579	85.686	11.133	11.128
523	92	21	25	B	C	49.107	3.584	0	3.584	-3.344	0	-3.344	60	11.924	11.924
524	92	22	24	B	C	46.102	4.483	-2.742	-1.708	-4.114	2.839	1.768	88.402	9.463	13.052
525	92	22	24	B	C	106.102	4.483	-2.742	-1.708	-4.114	2.839	1.768	88.402	9.463	13.052
526	92	22	24	B	C	10.893	-1.774	-2.578	4.558	1.84	2.362	-4.177	88.588	13.056	9.46

527	92	22	24	B	C	70.893	-1.774	-2.578	4.558	1.84	2.362	-4.177	88.588	13.056	9.46
528	92	23	23	B	C	47.48	1.631	-0.364	-3.286	-1.579	0.39	3.518	85.69	11.128	11.133
529	92	23	23	B	C	107.48	1.631	-0.364	-3.286	-1.579	0.39	3.518	85.69	11.128	11.133
530	92	21	25	B	C	21.052	2.623	-4.915	4.563	-2.492	4.504	-4.181	72.071	11.634	11.125
531	92	21	25	B	C	38.948	2.623	4.915	4.563	-2.492	-4.504	-4.181	72.071	11.125	11.634
532	92	21	25	B	C	81.052	2.623	-4.915	4.563	-2.492	4.504	-4.181	72.071	11.634	11.125
533	92	21	25	B	C	98.948	2.623	4.915	4.563	-2.492	-4.504	-4.181	72.071	11.125	11.634
534	92	22	24	B	C	13.898	4.483	2.742	-1.708	-4.114	-2.839	1.768	88.402	9.463	13.052
535	92	22	24	B	C	73.898	4.483	2.742	-1.708	-4.114	-2.839	1.768	88.402	9.463	13.052
536	94	23	24	B	C	70.158	-3.326	-4.68	3.921	3.563	4.34	-3.636	80.81	13.521	9.46
537	94	25	22	B	C	70.158	-3.326	-1.993	-4.526	3.563	2.192	4.977	71.689	11.134	11.929
538	94	23	24	B	C	70.158	-3.326	-4.68	3.921	3.563	4.34	-3.636	80.81	13.521	9.46
539	94	25	22	B	C	70.158	-3.326	-1.993	-4.526	3.563	2.192	4.977	71.689	11.134	11.929
540	94	24	23	B	C	25.693	-3.287	3.122	-0.521	3.518	-3.155	0.526	83.783	11.133	11.401
541	94	24	23	B	C	85.693	-3.287	3.122	-0.521	3.518	-3.155	0.526	83.783	11.133	11.401
542	94	23	24	B	C	38.213	-0.889	3.732	1.246	0.906	-3.641	-1.215	83.69	11.409	11.122
543	94	24	23	B	C	98.213	-0.889	3.576	-2.936	0.906	-3.799	3.119	83.692	11.409	11.126
544	94	23	24	B	C	98.213	-0.889	3.732	1.246	0.906	-3.641	-1.215	83.69	11.409	11.122
545	94	22	25	B	C	49.107	3.584	0.816	1.148	-3.344	-0.797	-1.123	71.708	11.924	11.126
546	94	22	25	B	C	109.107	3.584	0.816	1.148	-3.344	-0.797	-1.123	71.708	11.924	11.126
547	94	23	24	B	C	19.107	5.686	-3.979	-4.805	-5.106	4.403	5.316	87.606	12.166	10.403
548	94	23	24	B	C	79.107	5.686	-3.979	-4.805	-5.106	4.403	5.316	87.606	12.166	10.403
549	94	23	24	B	C	46.102	4.483	-1.967	-3.808	-4.114	2.129	4.122	81.09	9.463	13.512
550	94	23	24	B	C	106.102	4.483	-1.967	-3.808	-4.114	2.129	4.122	81.09	9.463	13.512
551	94	22	25	B	C	109.842	1.675	2.152	3.038	-1.62	-2.029	-2.864	71.702	11.127	11.922
552	94	23	24	B	C	25.693	1.631	3.098	-1.256	-1.579	-3.178	1.288	83.786	11.128	11.402
553	94	23	24	B	C	85.693	1.631	3.098	-1.256	-1.579	-3.178	1.288	83.786	11.128	11.402
554	94	22	25	B	C	107.48	1.631	-2.096	3.084	-1.579	1.974	-2.904	71.695	11.128	11.922
555	94	25	22	B	C	10.893	-4.99	-0.784	-2.846	5.543	0.831	3.018	71.701	11.933	11.13
556	94	23	24	B	C	13.898	4.483	1.967	-3.808	-4.114	-2.129	4.122	81.09	9.463	13.512
557	94	23	24	B	C	73.898	4.483	1.967	-3.808	-4.114	-2.129	4.122	81.09	9.463	13.512
558	94	23	24	B	C	21.787	-0.889	-3.732	1.246	0.906	3.641	-1.215	83.69	11.409	11.122
559	94	24	23	B	C	21.787	-0.889	-3.576	-2.936	0.906	3.799	3.119	83.692	11.409	11.126
560	94	25	22	B	C	70.893	-4.99	-0.784	-2.846	5.543	0.831	3.018	71.701	11.933	11.13
561	94	22	25	B	C	10.893	3.584	-0.816	1.148	-3.344	0.797	-1.123	71.708	11.924	11.126
562	94	22	25	B	C	70.893	3.584	-0.816	1.148	-3.344	0.797	-1.123	71.708	11.924	11.126
563	94	25	22	B	C	109.107	-4.99	0.784	-2.846	5.542	-0.831	3.018	71.701	11.933	11.13
564	94	25	22	B	C	109.107	-4.99	0.784	-2.846	5.542	-0.831	3.018	71.701	11.933	11.13
565	96	24	24	B	C	21.052	-4.167	-5.379	2.623	4.546	5.111	-2.492	67.616	13.403	10.407
566	96	24	24	B	C	81.052	-4.167	-5.379	2.623	4.546	5.111	-2.492	67.616	13.403	10.407
567	96	25	23	B	C	109.107	-4.99	1.567	-0.703	5.542	-1.59	0.713	84.59	11.933	10.849
568	96	23	25	B	C	49.107	3.584	1.56	-1.076	-3.344	-1.594	1.099	84.595	11.924	10.849
569	96	23	25	B	C	109.107	3.584	1.56	-1.076	-3.344	-1.594	1.099	84.595	11.924	10.849
570	96	24	24	B	C	10.893	-1.774	-1.575	0.012	1.84	1.575	-0.012	65.453	13.056	10.849

571	96	24	24	B	C	70.893	-1.774	-1.575	0.012	1.84	1.575	-0.012	65.453	13.056	10.849
572	96	24	24	B	C	36.587	-4.933	1.144	3.517	5.473	-1.068	-3.286	67.77	10.409	13.395
573	96	24	24	B	C	96.587	-4.933	1.144	3.517	5.473	-1.068	-3.286	67.77	10.409	13.395
574	96	24	24	B	C	13.174	-0.889	2.384	-0.889	0.906	-2.427	0.906	65.436	10.852	13.053
575	96	25	23	B	C	13.174	-0.889	2.289	-4.818	0.906	-2.533	5.332	84.506	10.852	11.931
576	96	23	25	B	C	13.174	-0.889	2.488	3.381	0.906	-2.33	-3.167	84.502	10.852	11.922
577	96	24	24	B	C	73.174	-0.889	2.384	-0.889	0.906	-2.427	0.906	65.436	10.852	13.053
578	96	25	23	B	C	73.174	-0.889	2.289	-4.818	0.906	-2.533	5.332	84.506	10.852	11.931
579	96	23	25	B	C	73.174	-0.889	2.488	3.381	0.906	-2.33	-3.167	84.502	10.852	11.922
580	96	25	23	B	C	10.893	-4.99	-1.567	-0.703	5.543	1.59	0.713	84.59	11.933	10.849
581	96	24	24	B	C	10.893	0.012	-1.547	-1.774	-0.012	1.604	1.84	65.457	13.054	10.85
582	96	24	24	B	C	49.107	0.012	1.547	-1.774	-0.012	-1.604	1.84	65.457	13.054	10.85
583	96	24	24	B	C	109.107	0.012	1.547	-1.774	-0.012	-1.604	1.84	65.457	13.054	10.85
584	96	24	24	B	C	21.052	2.623	-5.017	-4.167	-2.492	5.473	4.546	67.641	13.395	10.412
585	96	24	24	B	C	38.948	2.623	5.017	-4.167	-2.492	-5.473	4.546	67.641	13.395	10.412
586	96	24	24	B	C	81.052	2.623	-5.017	-4.167	-2.492	5.473	4.546	67.641	13.395	10.412
587	96	24	24	B	C	98.948	2.623	5.017	-4.167	-2.492	-5.473	4.546	67.641	13.395	10.412
588	96	24	24	B	C	23.413	-4.933	-1.144	3.517	5.473	1.068	-3.286	67.77	10.409	13.395
589	96	24	24	B	C	83.413	-4.933	-1.144	3.517	5.473	1.068	-3.286	67.77	10.409	13.395
590	96	24	24	B	C	106.826	-0.889	-2.384	-0.889	0.906	2.427	0.906	65.436	10.852	13.053
591	96	23	25	B	C	106.826	-0.889	-2.488	3.381	0.906	2.33	-3.167	84.502	10.852	11.922
592	96	25	23	B	C	106.826	-0.889	-2.289	-4.818	0.906	2.533	5.332	84.506	10.852	11.931
593	96	24	24	B	C	46.826	-0.889	-2.384	-0.889	0.906	2.427	0.906	65.436	10.852	13.053
594	96	23	25	B	C	46.826	-0.889	-2.488	3.381	0.906	2.33	-3.167	84.502	10.852	11.922
595	96	25	23	B	C	46.826	-0.889	-2.289	-4.818	0.906	2.533	5.332	84.506	10.852	11.931
596	96	23	25	B	C	10.893	3.584	-1.56	-1.076	-3.344	1.594	1.099	84.595	11.924	10.849
597	96	23	25	B	C	70.893	3.584	-1.56	-1.076	-3.344	1.594	1.099	84.595	11.924	10.849
598	96	25	23	B	C	70.893	-4.99	-1.567	-0.703	5.543	1.59	0.713	84.59	11.933	10.849
599	96	24	24	B	C	23.413	3.517	-1.048	-4.933	-3.286	1.163	5.473	67.8	10.401	13.402
600	96	24	24	B	C	36.587	3.517	1.048	-4.933	-3.286	-1.163	5.473	67.8	10.401	13.402
601	96	24	24	B	C	83.413	3.517	-1.048	-4.933	-3.286	1.163	5.473	67.8	10.401	13.402
602	96	25	23	B	C	109.107	-4.99	1.567	-0.703	5.542	-1.59	0.713	84.59	11.933	10.849
603	96	24	24	B	C	96.587	3.517	1.048	-4.933	-3.286	-1.163	5.473	67.8	10.401	13.402
604	96	24	24	N	C	31.626	-4.933	-1.144	3.517	5.473	1.068	-3.286	67.77	10.409	13.395
605	96	24	24	N	C	75.2	-4.933	-1.144	3.517	5.473	1.068	-3.286	67.77	10.409	13.395
606	96	24	24	N	C	93.74	-4.167	5.379	2.623	4.546	-5.111	-2.492	67.616	13.403	10.407
607	98	25	24	B	C	31.945	-3.326	5.422	-0.392	3.563	-5.465	0.396	68.837	11.108	12.7
608	98	25	24	B	C	91.945	-3.326	5.422	-0.392	3.563	-5.465	0.396	68.837	11.108	12.7
609	98	24	25	B	C	12.52	-3.287	0.551	3.782	3.518	-0.512	-3.516	82.638	11.133	11.922
610	98	25	24	B	C	109.107	-4.99	2.351	1.441	5.542	-2.285	-1.4	82.518	11.933	11.129
611	98	24	25	B	C	49.107	3.584	2.243	-3.114	-3.344	-2.392	3.321	82.527	11.924	11.133
612	98	24	25	B	C	40.893	-3.595	-3.711	4.139	3.873	3.427	-3.823	67.718	12.814	11.104
613	98	24	25	B	C	40.893	-3.595	2.783	4.139	3.873	-2.57	-3.823	81.389	12.814	10.392
614	98	24	25	B	C	100.893	-3.595	-3.711	4.139	3.873	3.427	-3.823	67.718	12.814	11.104

615	98	25	24	B	C	36.587	-4.933	-4.94	1.376	5.473	4.807	-1.34	81.077	10.409	12.804
616	98	25	24	B	C	96.587	-4.933	-4.94	1.376	5.473	4.807	-1.339	81.077	10.409	12.804
617	98	25	24	B	C	10.893	-4.99	-2.351	1.441	5.543	2.285	-1.4	82.518	11.933	11.129
618	98	24	25	B	C	30	-2.743	-2.106	3.164	2.903	1.98	-2.976	68.803	12.695	11.118
619	98	24	25	B	C	30	-2.743	2.106	3.164	2.903	-1.98	-2.976	68.803	12.695	11.118
620	98	24	25	B	C	90	-2.743	-2.106	3.164	2.903	1.98	-2.976	68.803	12.695	11.118
621	98	24	25	B	C	90	-2.743	2.106	3.164	2.903	-1.98	-2.976	68.803	12.695	11.118
622	98	25	24	B	C	23.413	-4.933	4.94	1.376	5.473	-4.807	-1.34	81.077	10.409	12.804
623	98	25	24	B	C	83.413	-4.933	4.94	1.376	5.473	-4.807	-1.339	81.077	10.409	12.804
624	98	24	25	B	C	12.52	-3.595	-2.783	4.139	3.873	2.57	-3.823	81.389	12.814	10.392
625	98	24	25	B	C	12.52	-3.595	3.711	4.139	3.873	-3.427	-3.823	67.718	12.814	11.104
626	98	24	25	B	C	10.893	3.584	-2.243	-3.114	-3.344	2.392	3.321	82.527	11.924	11.133
627	98	25	24	B	C	30	1.037	-1.945	-4.634	-1.016	2.143	5.107	68.824	12.69	11.124
628	98	25	24	B	C	30	1.037	1.945	-4.634	-1.016	-2.143	5.107	68.824	12.69	11.124
629	98	25	24	B	C	90	1.037	1.945	-4.634	-1.016	-2.143	5.107	68.824	12.69	11.124
630	98	25	24	B	C	90	1.037	-1.945	-4.634	-1.016	2.143	5.107	68.824	12.69	11.124
631	98	25	24	B	C	70.893	-4.99	-2.351	1.441	5.543	2.285	-1.4	82.518	11.933	11.129
632	98	24	25	B	C	23.413	3.517	4.718	-3.055	-3.286	-5.025	3.254	81.087	10.401	12.808
633	98	24	25	B	C	36.587	3.517	-4.718	-3.055	-3.286	5.026	3.254	81.087	10.401	12.808
634	98	25	24	B	C	109.107	-4.99	2.351	1.441	5.542	-2.285	-1.4	82.518	11.933	11.129
635	98	25	24	N	C	31.626	-4.933	-5.489	1.376	5.473	5.342	-1.34	86.901	10.409	12.667
636	100	26	24	B	C	70.158	-3.326	-1.993	-2.3	3.563	2.09	2.411	67.425	13.061	11.127
637	100	26	24	B	C	70.158	-3.326	-1.993	-2.3	3.563	2.09	2.411	67.425	13.061	11.127
638	100	26	24	B	C	12.52	-3.287	2.035	-2.341	3.518	-2.134	2.456	67.38	11.133	13.058
639	100	26	24	B	C	72.52	-3.287	2.035	-2.341	3.518	-2.134	2.456	67.38	11.133	13.058
640	100	25	25	B	C	109.107	-4.99	3.134	3.584	5.542	-2.925	-3.344	70.812	11.933	11.93
641	100	26	24	B	C	10.893	-1.774	-0.727	-3.835	1.84	0.788	4.154	67.421	13.056	11.132
642	100	26	24	B	C	70.893	-1.774	-0.727	-3.835	1.84	0.788	4.154	67.421	13.056	11.132
643	100	24	26	B	C	49.842	1.675	2.051	0.563	-1.62	-2.029	-0.557	67.415	13.056	11.124
644	100	24	26	B	C	109.842	1.675	2.051	0.563	-1.62	-2.029	-0.557	67.415	13.056	11.124
645	100	25	25	B	C	10.893	-4.99	-3.135	3.584	5.543	2.925	-3.344	70.812	11.933	11.93
646	100	24	26	B	C	47.48	1.631	-2.096	0.606	-1.579	2.071	-0.599	67.384	11.128	13.054
647	100	24	26	B	C	107.48	1.631	-2.096	0.606	-1.579	2.071	-0.599	67.384	11.128	13.054
648	100	24	26	B	C	10.893	0.012	-0.773	2.244	-0.012	0.74	-2.148	67.413	13.054	11.127
649	100	24	26	B	C	49.107	0.012	0.773	2.244	-0.012	-0.74	-2.148	67.413	13.054	11.127
650	100	24	26	B	C	70.893	0.012	-0.773	2.244	-0.012	0.74	-2.148	67.413	13.054	11.127
651	100	25	25	B	C	70.893	-4.99	-3.134	3.584	5.543	2.925	-3.344	70.812	11.933	11.93
652	100	25	25	B	C	109.107	-4.99	3.134	3.584	5.542	-2.925	-3.344	70.812	11.933	11.93
653	100	23	27	N	C	109.842	5.686	-5.306	0.844	-5.106	5.218	-0.83	82.506	12.166	11.106
654	102	27	24	B	C	31.945	-3.326	-4.282	-4.067	3.563	4.661	4.427	76.749	13.521	10.397
655	102	27	24	B	C	91.945	-3.326	-4.282	-4.067	3.563	4.661	4.427	76.749	13.521	10.397
656	102	25	26	B	C	42.103	-0.889	3.433	1.075	0.906	-3.361	-1.052	69.58	11.401	12.809
657	102	26	25	B	C	42.103	-0.889	3.301	-2.778	0.906	-3.495	2.942	69.561	12.812	11.404
658	102	25	26	B	C	102.103	-0.889	3.433	1.075	0.906	-3.361	-1.052	69.58	11.401	12.809

659	102	26	25	B	C	102.103	-0.889	3.301	-2.778	0.906	-3.495	2.942	69.561	12.812	11.404
660	102	24	27	B	C	38.213	-0.889	-3.576	5.249	0.906	3.236	-4.751	80.358	11.409	12.163
661	102	25	26	B	C	38.213	-0.889	-3.433	1.075	0.906	3.361	-1.052	69.512	11.409	12.806
662	102	26	25	B	C	38.213	-0.889	-3.301	-2.778	0.906	3.495	2.942	69.519	11.409	12.81
663	102	25	26	B	C	98.213	-0.889	-3.433	1.075	0.906	3.361	-1.052	69.512	11.409	12.806
664	102	26	25	B	C	98.213	-0.889	-3.301	-2.778	0.906	3.495	2.942	69.519	11.409	12.81
665	102	24	27	B	C	19.107	5.686	-1.271	-1.275	-5.106	1.304	1.308	80.497	12.166	11.405
666	102	26	25	B	C	40.893	-3.595	-4.71	-0.025	3.873	4.712	0.025	80.518	12.814	10.834
667	102	26	25	B	C	40.893	-3.595	1.284	-0.025	3.873	-1.285	0.025	69.565	12.814	11.404
668	102	26	25	B	C	100.893	-3.595	-4.71	-0.025	3.873	4.712	0.025	80.518	12.814	10.834
669	102	26	25	B	C	100.893	-3.595	1.284	-0.025	3.873	-1.285	0.025	69.565	12.814	11.404
670	102	24	27	B	C	31.945	1.675	-4.576	2.508	-1.62	4.358	-2.388	76.747	13.515	10.391
671	102	24	27	B	C	91.945	1.675	-4.576	2.508	-1.62	4.358	-2.388	76.747	13.515	10.391
672	102	27	24	B	C	36.587	-4.933	3.558	-2.429	5.473	-3.739	2.553	76.809	10.409	13.504
673	102	27	24	B	C	96.587	-4.933	3.558	-2.429	5.473	-3.739	2.553	76.809	10.409	13.504
674	102	27	24	B	C	23.413	-4.933	-3.558	-2.429	5.473	3.739	2.553	76.809	10.409	13.504
675	102	27	24	B	C	83.413	-4.933	-3.558	-2.429	5.473	3.739	2.553	76.809	10.409	13.504
676	102	26	25	B	C	12.52	-3.595	-1.284	-0.025	3.873	1.285	0.025	69.565	12.814	11.404
677	102	26	25	B	C	12.52	-3.595	4.71	-0.025	3.873	-4.712	0.025	80.518	12.814	10.834
678	102	25	26	B	C	21.787	-0.889	3.433	1.075	0.906	-3.361	-1.052	69.512	11.409	12.806
679	102	24	27	B	C	21.787	-0.889	3.576	5.249	0.906	-3.237	-4.751	80.358	11.409	12.163
680	102	26	25	B	C	21.787	-0.889	3.301	-2.778	0.906	-3.495	2.942	69.519	11.409	12.81
681	102	25	26	B	C	40.893	1.974	1.262	-1.739	-1.899	-1.307	1.801	69.578	12.808	11.405
682	102	25	26	B	C	40.893	1.974	-4.626	-1.739	-1.899	4.793	1.801	80.524	12.808	10.836
683	102	25	26	B	C	79.107	1.974	-1.262	-1.739	-1.899	1.307	1.801	69.578	12.808	11.405
684	102	25	26	B	C	79.107	1.974	4.626	-1.739	-1.899	-4.793	1.801	80.524	12.808	10.836
685	102	25	26	B	C	100.893	1.974	-4.626	-1.739	-1.899	4.793	1.801	80.524	12.808	10.836
686	102	25	26	B	C	100.893	1.974	1.262	-1.739	-1.899	-1.307	1.801	69.578	12.808	11.405
687	102	25	26	B	C	79.107	1.974	-1.262	-1.739	-1.899	1.307	1.801	69.578	12.808	11.405
688	102	25	26	B	C	79.107	1.974	4.626	-1.739	-1.899	-4.793	1.801	80.524	12.808	10.836
689	102	24	27	B	C	23.413	3.517	-3.67	0.7	-3.286	3.619	-0.691	76.815	10.401	13.5
690	102	24	27	B	C	36.587	3.517	3.67	0.7	-3.286	-3.619	-0.691	76.815	10.401	13.5
691	102	24	27	B	C	83.413	3.517	-3.67	0.7	-3.286	3.619	-0.691	76.815	10.401	13.5
692	102	24	27	B	C	96.587	3.517	3.67	0.7	-3.286	-3.619	-0.691	76.815	10.401	13.5
693	102	25	26	N	C	81.052	1.974	-1.262	-1.739	-1.899	1.307	1.801	69.578	12.808	11.405
694	102	27	24	N	C	31.626	-4.933	-3.558	-2.429	5.473	3.739	2.553	76.809	10.409	13.504
695	102	24	27	N	C	109.842	5.686	1.271	-1.275	-5.106	-1.304	1.308	80.497	12.166	11.405
696	104	28	24	B	C	21.052	-4.167	-1.976	-4.895	4.546	2.191	5.426	88.81	13.403	10.407
697	104	28	24	B	C	81.052	-4.167	-1.976	-4.895	4.546	2.191	5.426	88.81	13.403	10.407
698	104	26	26	B	C	31.945	-3.326	-4.37	1.675	3.563	4.229	-1.62	65.121	13.521	11.385
699	104	26	26	B	C	91.945	-3.326	-4.37	1.675	3.563	4.229	-1.62	65.121	13.521	11.385
700	104	27	25	B	C	25.693	-3.287	-3.592	-2.194	3.518	3.757	2.295	81.269	11.133	12.683
701	104	27	25	B	C	85.693	-3.287	-3.592	-2.194	3.518	3.757	2.295	81.269	11.133	12.683
702	104	24	28	B	C	46.102	4.483	2.828	1.646	-4.114	-2.738	-1.593	74.948	9.463	15.242

703	104	26	26	B	C	31.945	1.675	-4.151	-3.326	-1.62	4.447	3.563	65.141	13.515	11.389
704	104	26	26	B	C	91.945	1.675	-4.151	-3.326	-1.62	4.447	3.563	65.141	13.515	11.389
705	104	28	24	B	C	36.587	-4.933	-1.96	-4.128	5.473	2.137	4.5	88.829	10.409	13.4
706	104	28	24	B	C	96.587	-4.933	-1.96	-4.128	5.473	2.137	4.5	88.829	10.409	13.4
707	104	25	27	B	C	25.693	1.631	-3.688	0.451	-1.579	3.655	-0.447	81.271	11.128	12.68
708	104	25	27	B	C	85.693	1.631	-3.688	0.451	-1.579	3.655	-0.447	81.271	11.128	12.68
709	104	27	25	B	C	30	-2.743	-3.743	-2.743	2.903	3.961	2.903	81.247	12.695	11.123
710	104	27	25	B	C	30	-2.743	3.743	-2.743	2.903	-3.961	2.903	81.247	12.695	11.123
711	104	27	25	B	C	90	-2.743	-3.743	-2.743	2.903	3.961	2.903	81.247	12.695	11.123
712	104	27	25	B	C	90	-2.743	3.743	-2.743	2.903	-3.961	2.903	81.247	12.695	11.123
713	104	24	28	B	C	38.948	2.623	2.15	3.472	-2.492	-2.011	-3.246	88.81	13.395	10.399
714	104	24	28	B	C	81.052	2.623	-2.15	3.472	-2.492	2.011	-3.246	88.81	13.395	10.399
715	104	28	24	B	C	83.413	-4.933	1.96	-4.128	5.473	-2.137	4.5	88.829	10.409	13.4
716	104	24	28	B	C	13.898	4.483	-2.828	1.646	-4.114	2.738	-1.593	74.948	9.463	15.242
717	104	25	27	B	C	30	1.037	-3.889	1.037	-1.016	3.81	-1.016	81.247	12.69	11.119
718	104	25	27	B	C	30	1.037	0	1.037	-1.016	0	-1.016	60	12.69	12.69
719	104	25	27	B	C	30	1.037	3.889	1.037	-1.016	-3.81	-1.016	81.247	12.69	11.119
720	104	25	27	B	C	90	1.037	-3.889	1.037	-1.016	3.81	-1.016	81.247	12.69	11.119
721	104	25	27	B	C	90	1.037	0	1.037	-1.016	0	-1.016	60	12.69	12.69
722	104	25	27	B	C	90	1.037	3.889	1.037	-1.016	-3.81	-1.016	81.247	12.69	11.119
723	104	24	28	B	C	23.413	3.517	2.097	2.578	-3.286	-1.994	-2.452	88.829	10.401	13.392
724	104	24	28	B	C	36.587	3.517	-2.097	2.578	-3.286	1.994	-2.452	88.829	10.401	13.392
725	104	24	28	B	C	83.413	3.517	2.097	2.578	-3.286	-1.994	-2.452	88.829	10.401	13.392
726	104	24	28	B	C	96.587	3.517	-2.097	2.578	-3.286	1.994	-2.452	88.829	10.401	13.392
727	104	28	24	N	C	75.2	-4.933	1.96	-4.128	5.473	-2.137	4.5	88.829	10.409	13.4
728	104	26	26	N	C	33.811	1.675	4.151	-3.326	-1.62	-4.447	3.563	65.141	13.515	11.389
729	104	28	24	N	C	93.74	-4.167	1.976	-4.895	4.546	-2.191	5.426	88.81	13.403	10.407
730	106	25	28	B	C	51.052	4.687	4.094	-0.605	-4.285	-4.145	0.613	85.402	13.149	10.846
731	106	25	28	B	C	68.948	4.687	-4.094	-0.605	-4.285	4.145	0.613	85.402	13.149	10.846
732	106	25	28	B	C	111.052	4.687	4.094	-0.605	-4.285	-4.145	0.613	85.402	13.149	10.846
733	106	25	28	B	C	68.948	4.687	-4.094	-0.605	-4.285	4.145	0.613	85.402	13.149	10.846
734	106	27	26	B	C	70.158	-3.326	-1.993	-0.239	3.563	2.003	0.241	75.996	13.521	10.847
735	106	27	26	B	C	12.52	-3.287	2.123	-0.282	3.518	-2.134	0.283	78.254	11.133	13.055
736	106	26	27	B	C	25.693	-3.287	-3.9	3.617	3.518	3.637	-3.372	71.456	11.133	13.489
737	106	27	26	B	C	72.52	-3.287	2.123	-0.282	3.518	-2.134	0.283	78.254	11.133	13.055
738	106	26	27	B	C	85.693	-3.287	-3.9	3.617	3.518	3.637	-3.372	71.456	11.133	13.489
739	106	28	25	B	C	40.893	-3.595	0	-3.595	3.873	0	3.873	60	12.814	12.814
740	106	28	25	B	C	100.893	-3.595	0	-3.595	3.873	0	3.873	60	12.814	12.814
741	106	26	27	B	C	10.893	-1.774	-0.727	1.935	1.84	0.7	-1.863	78.314	13.056	11.127
742	106	26	27	B	C	70.893	-1.774	-0.727	1.935	1.84	0.7	-1.863	78.314	13.056	11.127
743	106	26	27	B	C	49.842	1.675	1.966	-1.531	-1.62	-2.029	1.579	76.004	13.515	10.847
744	106	26	27	B	C	109.842	1.675	1.966	-1.531	-1.62	-2.029	1.579	76.004	13.515	10.847
745	106	27	26	B	C	13.174	-0.889	3.179	-2.708	0.906	-3.361	2.864	75.937	10.852	13.515
746	106	26	27	B	C	13.174	-0.889	3.301	0.999	0.906	-3.236	-0.98	75.932	10.852	13.511

747	106	25	28	B	C	13.174	-0.889	3.433	5.004	0.906	-3.121	-4.549	85.637	10.852	13.139
748	106	26	27	B	C	73.174	-0.889	3.301	0.999	0.906	-3.237	-0.98	75.932	10.852	13.511
749	106	25	28	B	C	73.174	-0.889	3.433	5.004	0.906	-3.121	-4.549	85.637	10.852	13.139
750	106	26	27	B	C	47.48	1.631	-2.096	-1.49	-1.579	2.16	1.536	78.26	11.128	13.056
751	106	26	27	B	C	107.48	1.631	-2.096	-1.49	-1.579	2.16	1.536	78.26	11.128	13.056
752	106	27	26	B	C	10.893	0.012	-0.687	-3.56	-0.012	0.74	3.834	78.321	13.054	11.132
753	106	27	26	B	C	49.107	0.012	0.687	-3.56	-0.012	-0.74	3.834	78.321	13.054	11.132
754	106	27	26	B	C	70.893	0.012	-0.687	-3.56	-0.012	0.74	3.834	78.321	13.054	11.132
755	106	27	26	B	C	109.107	0.012	0.687	-3.56	-0.012	-0.74	3.834	78.321	13.054	11.132
756	106	27	26	B	C	106.826	-0.889	-3.179	-2.708	0.906	3.361	2.864	75.937	10.852	13.515
757	106	25	28	B	C	106.826	-0.889	-3.433	5.004	0.906	3.121	-4.549	85.637	10.852	13.139
758	106	26	27	B	C	106.826	-0.889	-3.301	0.999	0.906	3.237	-0.98	75.932	10.852	13.511
759	106	25	28	B	C	46.826	-0.889	-3.433	5.004	0.906	3.121	-4.549	85.637	10.852	13.139
760	106	26	27	B	C	46.826	-0.889	-3.301	0.999	0.906	3.236	-0.98	75.932	10.852	13.511
761	106	25	28	B	C	40.893	1.974	0	1.974	-1.899	0	-1.899	60	12.808	12.808
762	106	25	28	B	C	79.107	1.974	0	1.974	-1.899	0	-1.899	60	12.808	12.808
763	106	25	28	B	C	100.893	1.974	0	1.974	-1.899	0	-1.899	60	12.808	12.808
764	106	25	28	B	C	79.107	1.974	0	1.974	-1.899	0	-1.899	60	12.808	12.808
765	106	25	28	N	C	98.948	1.974	0	1.974	-1.899	0	-1.899	60	12.808	12.808
766	108	25	29	B	C	51.052	4.687	2.73	1.159	-4.285	-2.668	-1.133	81.667	13.149	11.121
767	108	25	29	B	C	68.948	4.687	-2.73	1.159	-4.285	2.668	-1.133	81.667	13.149	11.121
768	108	25	29	B	C	111.052	4.687	2.73	1.159	-4.285	-2.668	-1.133	81.667	13.149	11.121
769	108	25	29	B	C	68.948	4.687	-2.73	1.159	-4.285	2.668	-1.133	81.667	13.149	11.121
770	108	27	27	B	C	21.052	-4.167	-2.732	2.623	4.546	2.596	-2.492	76.886	13.403	11.116
771	108	25	29	B	C	47.48	1.631	-3.688	4.188	-1.579	3.403	-3.864	81.579	11.128	13.141
772	108	25	29	B	C	107.48	1.631	-3.688	4.188	-1.579	3.403	-3.864	81.579	11.128	13.141
773	108	27	27	B	C	21.052	2.623	-2.548	-4.167	-2.492	2.78	4.546	76.902	13.395	11.122
774	108	27	27	B	C	38.948	2.623	2.548	-4.167	-2.492	-2.78	4.546	76.902	13.395	11.122
775	108	27	27	B	C	81.052	2.623	-2.548	-4.167	-2.492	2.78	4.546	76.902	13.395	11.122
776	108	27	27	B	C	98.948	2.623	2.548	-4.167	-2.492	-2.78	4.546	76.902	13.395	11.122
777	108	27	27	N	C	93.74	-4.167	2.732	2.623	4.546	-2.596	-2.492	76.886	13.403	11.116
778	110	25	30	B	C	51.052	4.687	1.365	2.923	-4.285	-1.289	-2.762	69.917	13.149	11.92
779	110	25	30	B	C	68.948	4.687	-1.365	2.923	-4.285	1.289	-2.762	69.917	13.149	11.92
780	110	25	30	B	C	111.052	4.687	1.365	2.923	-4.285	-1.289	-2.762	69.917	13.149	11.92
781	110	25	30	B	C	68.948	4.687	-1.365	2.923	-4.285	1.289	-2.762	69.917	13.149	11.92
782	110	29	26	B	C	31.945	-3.326	-1.993	-3.671	3.563	2.151	3.962	78.662	13.521	11.132
783	110	29	26	B	C	91.945	-3.326	-1.993	-3.671	3.563	2.151	3.962	78.662	13.521	11.132
784	110	25	30	B	C	51.052	2.923	1.387	4.687	-2.762	-1.268	-4.285	69.89	11.924	13.146
785	110	25	30	B	C	111.052	2.923	1.387	4.687	-2.762	-1.268	-4.285	69.89	11.924	13.146
786	110	29	26	B	C	25.693	-3.287	-1.976	-3.71	3.518	2.134	4.008	78.663	11.133	13.52
787	110	29	26	B	C	85.693	-3.287	-1.976	-3.71	3.518	2.134	4.008	78.663	11.133	13.52
788	110	25	30	B	C	49.107	3.584	-2.153	4.013	-3.344	1.993	-3.715	69.914	11.924	13.144
789	110	25	30	B	C	109.107	3.584	-2.153	4.013	-3.344	1.993	-3.715	69.913	11.924	13.144
790	110	25	30	B	C	19.107	5.686	1.22	1.974	-5.106	-1.174	-1.899	70.86	12.166	12.81

791	110	25	30	B	C	79.107	5.686	1.22	1.974	-5.106	-1.174	-1.899	70.86	12.166	12.81
792	110	28	27	B	C	10.893	-1.774	0	-1.774	1.84	0	1.84	60	13.056	13.056
793	110	28	27	B	C	70.893	-1.774	0	-1.774	1.84	0	1.84	60	13.056	13.056
794	110	26	29	B	C	31.945	1.675	-2.112	2.059	-1.62	2.029	-1.978	78.661	13.515	11.126
795	110	26	29	B	C	91.945	1.675	-2.112	2.059	-1.62	2.029	-1.978	78.661	13.515	11.126
796	110	26	29	B	C	25.693	1.631	-2.096	2.104	-1.579	2.011	-2.019	78.662	11.128	13.513
797	110	26	29	B	C	85.693	1.631	-2.096	2.104	-1.579	2.011	-2.019	78.662	11.128	13.513
798	110	25	30	B	C	10.893	3.584	2.153	4.013	-3.344	-1.993	-3.715	69.913	11.924	13.144
799	110	25	30	B	C	70.893	3.584	2.153	4.013	-3.344	-1.993	-3.715	69.913	11.924	13.144
800	110	25	30	N	C	63.67	4.687	-1.365	2.923	-4.285	1.289	-2.762	69.917	13.149	11.92
801	110	25	30	N	C	116.33	4.687	1.365	2.923	-4.285	-1.289	-2.762	69.917	13.149	11.92
802	110	25	30	N	C	64.056	4.687	-1.365	2.923	-4.285	1.289	-2.762	69.917	13.149	11.92
803	110	25	30	N	C	10.893	2.923	-1.387	4.687	-2.762	1.268	-4.285	69.89	13.146	11.924
804	112	25	31	B	C	51.052	4.687	0	4.687	-4.285	0	-4.285	60	13.149	13.149
805	112	25	31	B	C	68.948	4.687	0	4.687	-4.285	0	-4.285	60	13.149	13.149
806	112	25	31	B	C	111.052	4.687	0	4.687	-4.285	0	-4.285	60	13.149	13.149
807	112	25	31	B	C	68.948	4.687	0	4.687	-4.285	0	-4.285	60	13.149	13.149
808	112	29	27	B	C	21.052	-4.167	-1.272	-1.006	4.546	1.298	1.027	79.447	13.403	11.41
809	112	29	27	B	C	81.052	-4.167	-1.272	-1.006	4.546	1.298	1.027	79.446	13.403	11.41
810	112	28	28	B	C	31.945	-3.326	-1.993	1.675	3.563	1.929	-1.62	88.877	13.521	11.125
811	112	28	28	B	C	70.158	-3.326	-1.993	1.675	3.563	1.929	-1.62	88.877	13.521	11.125
812	112	28	28	B	C	91.945	-3.326	-1.993	1.675	3.563	1.929	-1.62	88.877	13.521	11.125
813	112	28	28	B	C	70.158	-3.326	-1.993	1.675	3.563	1.929	-1.62	88.877	13.521	11.125
814	112	30	26	B	C	109.107	-4.99	-1.306	-3.56	5.542	1.406	3.834	68.558	11.933	13.524
815	112	27	29	B	C	42.103	-0.889	-4.239	2.748	0.906	4.018	-2.605	77.217	13.862	11.115
816	112	29	27	B	C	42.103	-0.889	-3.946	-4.276	0.906	4.315	4.676	77.225	13.862	11.122
817	112	27	29	B	C	102.103	-0.889	-4.239	2.748	0.906	4.018	-2.605	77.217	13.862	11.115
818	112	29	27	B	C	102.103	-0.889	-3.946	-4.276	0.906	4.315	4.676	77.225	13.862	11.122
819	112	26	30	B	C	51.052	2.923	2.095	2.584	-2.762	-1.992	-2.457	68.599	13.518	11.919
820	112	26	30	B	C	111.052	2.923	2.095	2.584	-2.762	-1.992	-2.457	68.599	13.518	11.919
821	112	29	27	B	C	12.52	-3.287	-5.168	-1.93	3.518	5.376	2.008	77.08	11.133	13.852
822	112	28	28	B	C	12.52	-3.287	2.204	1.631	3.518	-2.134	-1.579	88.758	11.133	13.512
823	112	28	28	B	C	25.693	-3.287	-2.204	1.631	3.518	2.134	-1.579	88.758	11.133	13.512
824	112	29	27	B	C	72.52	-3.287	-5.168	-1.93	3.518	5.376	2.008	77.08	11.133	13.852
825	112	28	28	B	C	72.52	-3.287	2.204	1.631	3.518	-2.134	-1.579	88.758	11.133	13.512
826	112	28	28	B	C	85.693	-3.287	-2.204	1.631	3.518	2.134	-1.579	88.758	11.133	13.512
827	112	29	27	B	C	38.213	-0.889	0	-4.276	0.906	0	4.676	79.49	11.409	13.403
828	112	27	29	B	C	38.213	-0.889	0	2.748	0.906	0	-2.605	79.484	11.409	13.395
829	112	29	27	B	C	98.213	-0.889	0	-4.276	0.906	0	4.676	79.49	11.409	13.403
830	112	27	29	B	C	98.213	-0.889	0	2.748	0.906	0	-2.605	79.484	11.409	13.395
831	112	26	30	B	C	49.107	3.584	-1.38	1.935	-3.344	1.329	-1.863	68.564	11.924	13.517
832	112	26	30	B	C	109.107	3.584	-1.38	1.935	-3.344	1.329	-1.863	68.564	11.924	13.517
833	112	28	28	B	C	31.945	1.675	-1.894	-3.326	-1.62	2.029	3.563	88.878	13.515	11.131
834	112	26	30	B	C	49.842	1.675	-0.073	3.854	-1.62	0.068	-3.578	68.594	13.515	11.924

835	112	28	28	B	C	49.842	1.675	1.894	-3.326	-1.62	-2.029	3.563	88.878	13.515	11.131
836	112	28	28	B	C	91.945	1.675	-1.894	-3.326	-1.62	2.029	3.563	88.878	13.515	11.131
837	112	26	30	B	C	109.842	1.675	-0.073	3.854	-1.62	0.068	-3.578	68.594	13.515	11.924
838	112	28	28	B	C	109.842	1.675	1.894	-3.326	-1.62	-2.029	3.563	88.878	13.515	11.131
839	112	28	28	B	C	25.693	1.631	-2.096	-3.286	-1.579	2.243	3.518	88.759	11.128	13.518
840	112	28	28	B	C	47.48	1.631	-2.096	-3.286	-1.579	2.243	3.518	88.759	11.128	13.518
841	112	27	29	B	C	47.48	1.631	5.278	0.174	-1.579	-5.26	-0.173	77.083	11.128	13.849
842	112	28	28	B	C	85.693	1.631	-2.096	-3.286	-1.579	2.243	3.518	88.759	11.128	13.518
843	112	28	28	B	C	107.48	1.631	-2.096	-3.286	-1.579	2.243	3.518	88.759	11.128	13.518
844	112	27	29	B	C	107.48	1.631	5.278	0.174	-1.579	-5.26	-0.173	77.083	11.128	13.849
845	112	30	26	B	C	10.893	-4.99	1.306	-3.56	5.543	-1.406	3.834	68.558	11.933	13.524
846	112	27	29	B	C	21.052	2.623	-1.274	-0.772	-2.492	1.294	0.784	79.453	13.395	11.409
847	112	27	29	B	C	38.948	2.623	1.274	-0.772	-2.492	-1.294	0.784	79.453	13.395	11.409
848	112	27	29	B	C	81.052	2.623	-1.274	-0.772	-2.492	1.294	0.784	79.453	13.395	11.409
849	112	27	29	B	C	98.948	2.623	1.274	-0.772	-2.492	-1.294	0.784	79.453	13.395	11.409
850	112	27	29	B	C	21.787	-0.889	0	2.748	0.906	0	-2.605	79.484	11.409	13.395
851	112	29	27	B	C	21.787	-0.889	0	-4.276	0.906	0	4.676	79.49	11.409	13.403
852	112	26	30	B	C	10.893	3.584	1.38	1.935	-3.344	-1.329	-1.863	68.564	11.924	13.517
853	112	26	30	B	C	70.893	3.584	1.38	1.935	-3.344	-1.329	-1.863	68.564	11.924	13.517
854	112	30	26	B	C	70.893	-4.99	1.306	-3.56	5.543	-1.406	3.834	68.558	11.933	13.524
855	112	30	26	B	C	109.107	-4.99	-1.306	-3.56	5.542	1.406	3.834	68.558	11.933	13.524
856	112	25	31	N	C	63.67	4.687	0	4.687	-4.285	0	-4.285	60	13.149	13.149
857	112	26	30	N	C	64.402	1.675	0.073	3.854	-1.62	-0.068	-3.578	68.594	13.515	11.924
858	112	29	27	N	C	93.74	-4.167	1.272	-1.006	4.546	-1.298	1.027	79.447	13.403	11.41
859	112	26	30	N	C	10.893	2.923	-2.095	2.584	-2.762	1.992	-2.457	68.599	11.919	13.518
860	114	27	30	B	C	51.052	2.923	2.751	0.636	-2.762	-2.717	-0.628	72.691	14.395	11.12
861	114	27	30	B	C	49.107	3.584	-0.664	0.012	-3.344	0.664	-0.012	79.094	11.924	13.055
862	114	27	30	B	C	109.107	3.584	-0.664	0.012	-3.344	0.664	-0.012	79.094	11.924	13.055
863	114	30	27	B	C	109.107	-4.99	-0.653	-1.774	5.542	0.677	1.84	79.087	11.933	13.057
864	114	27	30	B	C	47.48	1.631	-3.57	1.904	-1.579	3.439	-1.834	72.658	11.128	14.386
865	114	30	27	B	C	10.893	-4.99	0.653	-1.774	5.543	-0.677	1.84	79.087	11.933	13.057
866	114	27	30	B	C	10.893	0.012	0.687	3.584	-0.012	-0.641	-3.344	79.107	13.054	11.924
867	114	27	30	B	C	70.893	0.012	0.687	3.584	-0.012	-0.641	-3.344	79.107	13.054	11.924
868	114	27	30	B	C	109.107	0.012	-0.687	3.584	-0.012	0.641	-3.344	79.107	13.054	11.924
869	114	27	30	B	C	10.893	3.584	0.664	0.012	-3.344	-0.664	-0.012	79.094	11.924	13.055
870	114	27	30	N	C	10.893	2.923	-2.751	0.636	-2.762	2.717	-0.628	72.691	14.395	11.12
871	116	31	27	B	C	21.052	-4.167	0	-4.167	4.546	0	4.546	60	13.403	13.403
872	116	31	27	B	C	81.052	-4.167	0	-4.167	4.546	0	4.546	60	13.403	13.403
873	116	30	28	B	C	109.107	-4.99	0	0.012	5.542	0	-0.012	90	11.933	13.054
874	116	28	30	B	C	51.052	2.923	3.36	-1.172	-2.762	-3.441	1.2	85.6	14.395	10.844
875	116	28	30	B	C	111.052	2.923	3.36	-1.172	-2.762	-3.441	1.2	85.6	14.395	10.844
876	116	28	30	B	C	49.107	3.584	0	-1.774	-3.344	0	1.84	90	11.924	13.056
877	116	28	30	B	C	109.107	3.584	0	-1.774	-3.344	0	1.84	90	11.924	13.056
878	116	28	30	B	C	19.107	5.686	0	-3.595	-5.106	0	3.873	90	12.166	12.814

879	116	28	30	B	C	79.107	5.686	0	-3.595	-5.106	0	3.873	90	12.166	12.814
880	116	28	30	B	C	10.893	-1.774	0	3.584	1.84	0	-3.344	90	13.056	11.924
881	116	28	30	B	C	70.893	-1.774	0	3.584	1.84	0	-3.344	90	13.056	11.924
882	116	28	30	B	C	13.174	-0.889	4.087	2.618	0.906	-3.884	-2.488	85.446	10.852	14.386
883	116	28	30	B	C	73.174	-0.889	4.087	2.618	0.906	-3.884	-2.488	85.445	10.852	14.386
884	116	30	28	B	C	10.893	-4.99	0	0.012	5.543	0	-0.012	90	11.933	13.054
885	116	27	31	B	C	21.052	2.623	0	2.623	-2.492	0	-2.492	60	13.395	13.395
886	116	28	30	B	C	106.826	-0.889	-4.087	2.618	0.906	3.884	-2.488	85.445	10.852	14.386
887	116	28	30	B	C	46.826	-0.889	-4.087	2.618	0.906	3.884	-2.488	85.446	10.852	14.386
888	116	30	28	B	C	70.893	-4.99	0	0.012	5.543	0	-0.012	90	11.933	13.054
889	116	30	28	B	C	109.107	-4.99	0	0.012	5.542	0	-0.012	90	11.933	13.054
890	116	31	27	N	C	93.74	-4.167	0	-4.167	4.546	0	4.546	60	13.403	13.403
891	116	28	30	N	C	10.893	2.923	-3.36	-1.172	-2.762	3.441	1.2	85.6	14.395	10.844
892	118	31	28	B	C	31.945	-3.326	0	-3.326	3.563	0	3.563	60	13.521	13.521
893	118	29	30	B	C	70.158	-3.326	-1.993	3.456	3.563	1.864	-3.233	79.425	13.521	11.925
894	118	31	28	B	C	70.158	-3.326	0	-3.326	3.563	0	3.563	60	13.521	13.521
895	118	31	28	B	C	91.945	-3.326	0	-3.326	3.563	0	3.563	60	13.521	13.521
896	118	29	30	B	C	70.158	-3.326	-1.993	3.456	3.563	1.864	-3.233	79.425	13.521	11.925
897	118	31	28	B	C	70.158	-3.326	0	-3.326	3.563	0	3.563	60	13.521	13.521
898	118	29	30	B	C	51.052	2.923	3.927	-2.856	-2.762	-4.165	3.029	81.502	14.395	11.129
899	118	30	29	B	C	109.107	-4.99	0.653	1.798	5.542	-0.63	-1.735	79.462	11.933	13.515
900	118	29	30	B	C	111.052	2.923	3.927	-2.856	-2.762	-4.165	3.029	81.502	14.395	11.129
901	118	30	29	B	C	12.52	-3.287	-4.849	-0.09	3.518	4.858	0.09	69	11.133	15.241
902	118	29	30	B	C	12.52	-3.287	2.28	3.411	3.518	-2.134	-3.193	81.72	11.133	14.386
903	118	29	30	B	C	72.52	-3.287	2.28	3.411	3.518	-2.134	-3.193	81.72	11.133	14.386
904	118	29	30	B	C	49.107	3.584	0.619	-3.437	-3.344	-0.664	3.691	79.475	11.924	13.521
905	118	29	30	B	C	109.107	3.584	0.619	-3.437	-3.344	-0.664	3.691	79.475	11.924	13.521
906	118	28	31	B	C	31.945	1.675	0	1.675	-1.62	0	-1.62	60	13.515	13.515
907	118	28	31	B	C	49.842	1.675	0	1.675	-1.62	0	-1.62	60	13.515	13.515
908	118	30	29	B	C	49.842	1.675	1.83	-4.882	-1.62	-2.029	5.41	79.437	13.515	11.934
909	118	28	31	B	C	91.945	1.675	0	1.675	-1.62	0	-1.62	60	13.515	13.515
910	118	28	31	B	C	109.842	1.675	0	1.675	-1.62	0	-1.62	60	13.515	13.515
911	118	30	29	B	C	109.842	1.675	1.83	-4.882	-1.62	-2.029	5.41	79.437	13.515	11.934
912	118	30	29	B	C	10.893	-4.99	-0.653	1.798	5.543	0.63	-1.735	79.462	11.933	13.515
913	118	29	30	B	C	10.893	3.584	-0.619	-3.437	-3.344	0.664	3.691	79.475	11.924	13.521
914	118	29	30	B	C	70.893	3.584	-0.619	-3.437	-3.344	0.664	3.691	79.475	11.924	13.521
915	118	30	29	B	C	70.893	-4.99	-0.653	1.798	5.543	0.63	-1.735	79.462	11.933	13.515
916	118	30	29	B	C	109.107	-4.99	0.653	1.798	5.542	-0.63	-1.735	79.462	11.933	13.515
917	118	28	31	N	C	33.811	1.675	0	1.675	-1.62	0	-1.62	60	13.515	13.515
918	118	28	31	N	C	64.402	1.675	0	1.674	-1.62	0	-1.62	60	13.515	13.515
919	118	29	30	N	C	10.893	2.923	-3.927	-2.856	-2.762	4.165	3.029	81.502	14.395	11.129
920	120	30	30	B	C	21.052	-4.167	-0.615	2.623	4.546	0.584	-2.492	69.926	13.403	12.807
921	120	30	30	B	C	81.052	-4.167	-0.615	2.623	4.546	0.584	-2.492	69.926	13.403	12.807
922	120	30	30	B	C	31.945	-3.326	0.066	1.675	3.563	-0.064	-1.62	69.932	13.521	12.688

923	120	30	30	B	C	91.945	-3.326	0.066	1.675	3.563	-0.064	-1.62	69.932	13.521	12.688
924	120	30	30	B	C	51.052	2.923	4.457	-4.427	-2.762	-4.89	4.857	69.803	14.395	11.937
925	120	27	33	B	C	19.107	5.686	-2.26	2.936	-5.106	2.135	-2.773	80.038	12.166	13.396
926	120	27	33	B	C	79.107	5.686	-2.26	2.936	-5.106	2.135	-2.773	80.038	12.166	13.396
927	120	30	30	B	C	10.893	-4.99	-1.306	3.584	5.543	1.219	-3.344	69.915	11.933	14.396
928	120	30	30	B	C	40.893	-3.595	4.082	1.974	3.873	-3.927	-1.899	69.821	12.814	13.399
929	120	30	30	B	C	100.893	-3.595	4.082	1.974	3.873	-3.927	-1.899	69.821	12.814	13.399
930	120	30	30	B	C	10.893	-1.774	0.63	0.012	1.84	-0.63	-0.012	70.888	13.056	13.055
931	120	30	30	B	C	70.893	-1.774	0.63	0.012	1.84	-0.63	-0.012	70.888	13.056	13.055
932	120	30	30	B	C	31.945	1.675	0.063	-3.326	-1.62	-0.068	3.563	69.949	13.515	12.693
933	120	30	30	B	C	91.945	1.675	0.063	-3.326	-1.62	-0.068	3.563	69.949	13.515	12.693
934	120	30	30	B	C	30	-2.743	-3.369	1.037	2.903	3.301	-1.016	69.857	12.695	13.517
935	120	30	30	B	C	30	-2.743	3.369	1.037	2.903	-3.301	-1.016	69.857	12.695	13.517
936	120	30	30	B	C	90	-2.743	-3.369	1.037	2.903	3.301	-1.016	69.857	12.695	13.517
937	120	30	30	B	C	90	-2.743	3.369	1.037	2.903	-3.301	-1.016	69.857	12.695	13.517
938	120	30	30	B	C	10.893	0.012	0.619	-1.774	-0.012	-0.641	1.84	70.893	13.054	13.056
939	120	30	30	B	C	49.107	0.012	-0.619	-1.774	-0.012	0.641	1.84	70.893	13.054	13.056
940	120	30	30	B	C	70.893	0.012	0.619	-1.774	-0.012	-0.641	1.84	70.893	13.054	13.056
941	120	30	30	B	C	109.107	0.012	-0.619	-1.774	-0.012	0.641	1.84	70.893	13.054	13.056
942	120	30	30	B	C	21.052	2.623	-0.573	-4.167	-2.492	0.626	4.546	69.949	13.395	12.813
943	120	30	30	B	C	38.948	2.623	0.573	-4.167	-2.492	-0.626	4.546	69.949	13.395	12.813
944	120	30	30	B	C	81.052	2.623	-0.573	-4.167	-2.492	0.626	4.546	69.949	13.395	12.813
945	120	30	30	B	C	98.948	2.623	0.573	-4.167	-2.492	-0.626	4.546	69.949	13.395	12.813
946	120	30	30	B	C	30	1.037	-3.241	-2.743	-1.016	3.429	2.903	69.87	12.69	13.52
947	120	30	30	B	C	30	1.037	3.241	-2.743	-1.016	-3.429	2.903	69.87	12.69	13.52
948	120	30	30	B	C	90	1.037	-3.241	-2.743	-1.016	3.429	2.903	69.87	12.69	13.52
949	120	30	30	B	C	90	1.037	3.241	-2.743	-1.016	-3.429	2.903	69.87	12.69	13.52
950	120	30	30	B	C	40.893	1.974	3.855	-3.595	-1.899	-4.154	3.873	69.839	12.808	13.405
951	120	30	30	B	C	109.107	-4.99	1.306	3.584	5.542	-1.219	-3.344	69.915	11.933	14.396
952	120	30	30	B	C	79.107	1.974	-3.855	-3.595	-1.899	4.154	3.873	69.839	12.808	13.405
953	120	30	30	B	C	100.893	1.974	3.855	-3.595	-1.899	-4.154	3.873	69.839	12.808	13.405
954	120	30	30	B	C	79.107	1.974	-3.855	-3.595	-1.899	4.154	3.873	69.839	12.808	13.405
955	120	30	30	N	C	81.052	1.974	-3.855	-3.595	-1.899	4.154	3.873	69.839	12.808	13.405
956	120	30	30	N	C	98.948	1.974	3.855	-3.595	-1.899	-4.154	3.873	69.839	12.808	13.405
957	120	30	30	N	C	33.811	1.675	-0.063	-3.326	-1.62	0.068	3.563	69.949	13.515	12.693
958	120	30	30	N	C	10.893	2.923	-4.457	-4.427	-2.762	4.89	4.858	69.803	14.395	11.937
959	124	30	32	B	C	51.052	4.687	3.899	-2.957	-4.285	-4.145	3.143	69.442	13.149	13.528
960	124	30	32	B	C	68.948	4.687	-3.899	-2.957	-4.285	4.145	3.143	69.442	13.149	13.528
961	124	30	32	B	C	70.158	-3.326	-1.993	5.119	3.563	1.808	-4.644	69.548	13.521	13.151
962	124	32	30	B	C	70.158	-3.326	-0.062	-1.555	3.563	0.064	1.605	70.52	13.521	13.056
963	124	32	30	B	C	70.158	-3.326	-0.062	-1.555	3.563	0.064	1.605	70.52	13.521	13.056
964	124	31	31	B	C	42.103	-0.889	0	-0.889	0.906	0	0.906	60	13.862	13.862
965	124	30	32	B	C	42.103	-0.889	0	2.385	0.906	0	-2.276	69.565	13.862	12.807
966	124	32	30	B	C	42.103	-0.889	0	-3.959	0.906	0	4.299	69.576	13.862	12.814

967	124	31	31	B	C	102.103	-0.889	0	-0.889	0.906	0	0.906	60	13.862	13.862
968	124	32	30	B	C	102.103	-0.889	0	-3.959	0.906	0	4.299	69.576	13.862	12.814
969	124	29	33	B	C	19.107	5.686	2.104	-0.715	-5.106	-2.135	0.725	80.378	12.166	13.864
970	124	29	33	B	C	79.107	5.686	2.104	-0.715	-5.106	-2.135	0.725	80.378	12.166	13.864
971	124	32	30	B	C	40.893	-3.595	-2.087	-1.275	3.873	2.142	1.308	69.519	12.814	13.865
972	124	32	30	B	C	100.893	-3.595	-2.087	-1.275	3.873	2.142	1.308	69.519	12.814	13.865
973	124	30	32	B	C	49.842	1.675	0.063	-0.215	-1.62	-0.063	0.215	70.526	13.515	13.054
974	124	30	32	B	C	109.842	1.675	0.063	-0.215	-1.62	-0.063	0.216	70.526	13.515	13.054
975	124	30	32	B	C	10.893	0.012	1.237	1.441	-0.012	-1.203	-1.4	70.52	13.054	13.514
976	124	30	32	B	C	70.893	0.012	1.237	1.441	-0.012	-1.203	-1.4	70.52	13.054	13.514
977	124	32	30	B	C	12.52	-3.595	2.087	-1.275	3.873	-2.142	1.308	69.519	12.814	13.865
978	124	30	32	B	C	40.893	1.974	-2.103	-0.501	-1.899	2.124	0.506	69.527	12.808	13.863
979	124	30	32	B	C	79.107	1.974	2.103	-0.501	-1.899	-2.124	0.506	69.527	12.808	13.863
980	124	30	32	B	C	100.893	1.974	-2.103	-0.501	-1.899	2.124	0.506	69.527	12.808	13.863
981	124	30	32	N	C	81.052	1.974	2.103	-0.501	-1.899	-2.124	0.506	69.527	12.808	13.863
982	124	30	32	N	C	98.948	1.974	-2.103	-0.501	-1.899	2.124	0.506	69.527	12.808	13.863
983	124	30	32	N	C	63.67	4.687	-3.899	-2.957	-4.285	4.145	3.143	69.442	13.149	13.528
984	124	30	32	N	C	116.33	4.687	3.899	-2.957	-4.285	-4.145	3.143	69.442	13.149	13.528
985	124	30	32	N	C	64.056	4.687	-3.899	-2.957	-4.285	4.145	3.143	69.442	13.149	13.528
986	126	30	33	B	C	51.052	4.687	2.762	-1.487	-4.285	-2.847	1.533	79.992	13.149	13.057
987	126	30	33	B	C	68.948	4.687	-2.762	-1.487	-4.285	2.847	1.533	79.992	13.149	13.057
988	126	30	33	B	C	111.052	4.687	2.762	-1.487	-4.285	-2.847	1.533	79.992	13.149	13.057
989	126	30	33	B	C	68.948	4.687	-2.762	-1.487	-4.285	2.847	1.533	79.992	13.149	13.057
990	126	33	30	B	C	31.945	-3.326	1.752	-3.023	3.563	-1.864	3.218	80.072	13.521	12.694
991	126	33	30	B	C	91.945	-3.326	1.752	-3.023	3.563	-1.864	3.218	80.072	13.521	12.694
992	126	30	33	B	C	10.893	-1.774	0.63	5.013	1.84	-0.573	-4.556	80.129	13.056	13.146
993	126	30	33	B	C	70.893	-1.774	0.63	5.013	1.84	-0.573	-4.556	80.129	13.056	13.146
994	126	30	33	B	C	31.945	1.675	1.83	1.341	-1.62	-1.783	-1.306	80.073	13.515	12.689
995	126	33	30	B	C	30	-2.743	1.531	-3.603	2.903	-1.65	3.882	80.088	12.695	13.52
996	126	33	30	B	C	90	-2.743	1.531	-3.603	2.903	-1.65	3.882	80.088	12.695	13.52
997	126	30	33	B	C	30	1.037	-1.62	1.982	-1.016	1.559	-1.907	80.086	12.69	13.513
998	126	30	33	B	C	30	1.037	1.62	1.982	-1.016	-1.559	-1.907	80.086	12.69	13.513
999	126	30	33	B	C	90	1.037	-1.62	1.982	-1.016	1.559	-1.907	80.086	12.69	13.513
1000	126	30	33	B	C	90	1.037	1.62	1.982	-1.016	-1.559	-1.907	80.086	12.69	13.513
1001	126	30	33	N	C	33.811	1.675	-1.83	1.341	-1.62	1.783	-1.306	80.073	13.515	12.689
1002	128	30	34	B	C	51.052	4.687	1.625	-0.017	-4.285	-1.625	0.017	89.069	13.149	13.052
1003	128	30	34	B	C	68.948	4.687	-1.625	-0.017	-4.285	1.625	0.017	89.069	13.149	13.052
1004	128	30	34	B	C	111.052	4.687	1.625	-0.017	-4.285	-1.625	0.017	89.069	13.149	13.052
1005	128	30	34	B	C	68.948	4.687	-1.625	-0.017	-4.285	1.625	0.017	89.069	13.149	13.052
1006	128	34	30	B	C	21.052	-4.167	1.627	-3.568	4.546	-1.752	3.842	89.033	13.403	12.812
1007	128	34	30	B	C	81.052	-4.167	1.627	-3.568	4.546	-1.752	3.842	89.033	13.403	12.812
1008	128	32	32	B	C	31.945	-3.326	1.869	1.675	3.563	-1.808	-1.62	70.126	13.521	13.517
1009	128	32	32	B	C	91.945	-3.326	1.869	1.675	3.563	-1.808	-1.62	70.126	13.521	13.517
1010	128	34	30	B	C	40.893	-3.595	1.637	-4.141	3.873	-1.785	4.515	89.022	12.814	13.401

1011	128	34	30	B	C	100.893	-3.595	1.637	-4.141	3.873	-1.785	4.515	89.022	12.814	13.401
1012	128	32	32	B	C	31.945	1.675	1.775	-3.326	-1.62	-1.902	3.563	70.143	13.515	13.522
1013	128	32	32	B	C	91.945	1.675	1.775	-3.326	-1.62	-1.902	3.563	70.143	13.515	13.522
1014	128	30	34	B	C	10.893	0.012	1.856	4.656	-0.012	-1.698	-4.259	88.984	13.054	13.147
1015	128	30	34	B	C	49.107	0.012	-1.856	4.656	-0.012	1.698	-4.259	88.984	13.054	13.147
1016	128	30	34	B	C	70.893	0.012	1.856	4.656	-0.012	-1.698	-4.259	88.984	13.054	13.147
1017	128	30	34	B	C	109.107	0.012	-1.856	4.656	-0.012	1.698	-4.259	88.984	13.054	13.147
1018	128	30	34	B	C	21.052	2.623	1.72	1.944	-2.492	-1.656	-1.871	89.033	13.395	12.806
1019	128	30	34	B	C	98.948	2.623	-1.72	1.944	-2.492	1.656	-1.871	89.033	13.395	12.806
1020	128	30	34	B	C	40.893	1.974	-3.154	2.593	-1.899	2.999	-2.465	67.544	12.808	14.49
1021	128	30	34	B	C	40.893	1.974	1.752	2.593	-1.899	-1.666	-2.465	89.022	12.808	13.393
1022	128	30	34	B	C	79.107	1.974	3.154	2.593	-1.899	-2.999	-2.465	67.544	12.808	14.49
1023	128	30	34	B	C	79.107	1.974	-1.752	2.593	-1.899	1.666	-2.465	89.022	12.808	13.393
1024	128	30	34	B	C	100.893	1.974	1.752	2.593	-1.899	-1.666	-2.465	89.022	12.808	13.393
1025	128	30	34	B	C	79.107	1.974	-1.752	2.593	-1.899	1.666	-2.465	89.022	12.808	13.393
1026	128	30	34	N	C	81.052	1.974	3.154	2.593	-1.899	-2.999	-2.465	67.544	12.808	14.49
1027	128	30	34	N	C	81.052	1.974	-1.752	2.593	-1.899	1.666	-2.465	89.022	12.808	13.393
1028	128	30	34	N	C	98.948	1.974	-3.154	2.592	-1.899	2.999	-2.465	67.544	12.808	14.49
1029	128	30	34	N	C	98.948	1.974	1.752	2.593	-1.899	-1.666	-2.465	89.022	12.808	13.393
1030	128	32	32	N	C	33.811	1.675	-1.775	-3.326	-1.62	1.902	3.563	70.143	13.515	13.522
1031	130	30	35	B	C	51.052	4.687	0.487	1.453	-4.285	-0.474	-1.412	78.503	13.149	13.513
1032	130	30	35	B	C	68.948	4.687	-0.487	1.453	-4.285	0.474	-1.412	78.503	13.149	13.513
1033	130	33	32	B	C	70.158	-3.326	-0.121	0.109	3.563	0.12	-0.108	81.422	13.521	13.054
1034	130	33	32	B	C	70.158	-3.326	-0.121	0.109	3.563	0.12	-0.108	81.422	13.521	13.054
1035	130	30	35	B	C	49.107	3.584	5.382	2.512	-3.344	-5.125	-2.392	73.431	11.924	15.232
1036	130	32	33	B	C	10.893	-1.774	1.182	1.575	1.84	-1.145	-1.527	81.401	13.056	13.515
1037	130	32	33	B	C	70.893	-1.774	1.182	1.575	1.84	-1.145	-1.527	81.401	13.056	13.515
1038	130	30	35	B	C	49.842	1.675	-1.704	4.453	-1.62	1.565	-4.089	78.489	13.515	13.147
1039	130	32	33	B	C	49.842	1.675	0.118	-1.868	-1.62	-0.123	1.94	81.427	13.515	13.056
1040	130	32	33	B	C	109.842	1.675	0.118	-1.868	-1.62	-0.123	1.94	81.427	13.515	13.056
1041	130	33	32	B	C	10.893	0.012	1.125	-3.236	-0.012	-1.203	3.46	81.407	13.054	13.521
1042	130	33	32	B	C	49.107	0.012	-1.125	-3.236	-0.012	1.203	3.46	81.407	13.054	13.521
1043	130	33	32	B	C	70.893	0.012	1.125	-3.236	-0.012	-1.203	3.46	81.407	13.054	13.521
1044	130	33	32	B	C	109.107	0.012	-1.125	-3.236	-0.012	1.203	3.46	81.407	13.054	13.521
1045	130	30	35	B	C	10.893	3.584	-5.382	2.512	-3.344	5.125	-2.392	73.431	11.924	15.232
1046	130	30	35	N	C	63.67	4.687	-0.487	1.453	-4.285	0.474	-1.412	78.503	13.149	13.513
1047	130	30	35	N	C	116.33	4.687	0.487	1.453	-4.285	-0.474	-1.412	78.503	13.149	13.513
1048	130	30	35	N	C	64.402	1.675	1.704	4.453	-1.62	-1.565	-4.089	78.489	13.515	13.147
1049	132	30	36	B	C	51.052	4.687	-0.65	2.923	-4.285	0.614	-2.762	68.934	13.149	14.396
1050	132	30	36	B	C	68.948	4.687	0.65	2.923	-4.285	-0.614	-2.762	68.934	13.149	14.396
1051	132	30	36	B	C	111.052	4.687	-0.65	2.923	-4.285	0.614	-2.762	68.934	13.149	14.396
1052	132	30	36	B	C	68.948	4.687	0.65	2.923	-4.285	-0.614	-2.762	68.934	13.149	14.396
1053	132	33	33	B	C	21.052	-4.167	1.118	2.623	4.546	-1.062	-2.492	81.025	13.403	13.396
1054	132	33	33	B	C	81.052	-4.167	1.118	2.623	4.546	-1.062	-2.492	81.025	13.403	13.396

1055	132	30	36	B	C	111.052	2.923	-0.66	4.687	-2.762	0.604	-4.285	68.957	14.395	13.148
1056	132	30	36	B	C	19.107	5.686	4.068	1.974	-5.106	-3.913	-1.899	72.301	12.166	15.244
1057	132	33	33	B	C	38.948	2.623	-1.043	-4.167	-2.492	1.137	4.546	81.036	13.395	13.404
1058	132	33	33	B	C	98.948	2.623	-1.043	-4.167	-2.492	1.137	4.546	81.036	13.395	13.404
1059	132	30	36	N	C	63.67	4.687	0.65	2.923	-4.285	-0.614	-2.762	68.934	13.149	14.396
1060	132	30	36	N	C	116.33	4.687	-0.65	2.923	-4.285	0.614	-2.762	68.934	13.149	14.396
1061	132	30	36	N	C	64.056	4.687	0.65	2.923	-4.285	-0.614	-2.762	68.934	13.149	14.396
1062	132	30	36	N	C	109.842	5.686	-4.068	1.974	-5.106	3.913	-1.899	72.301	12.166	15.244
1063	132	33	33	N	C	93.74	-4.167	-1.118	2.623	4.546	1.062	-2.492	81.025	13.403	13.396
1064	134	35	32	B	C	31.945	-3.326	3.303	-2.755	3.563	-3.496	2.915	79.84	13.521	13.51
1065	134	35	32	B	C	91.945	-3.326	3.303	-2.755	3.563	-3.496	2.915	79.84	13.521	13.51
1066	134	31	36	B	C	51.052	2.923	0	2.923	-2.762	0	-2.762	60	14.395	14.395
1067	134	31	36	B	C	111.052	2.923	0	2.923	-2.762	0	-2.762	60	14.395	14.395
1068	134	32	35	B	C	31.945	1.675	3.432	1.05	-1.62	-3.362	-1.028	79.841	13.515	13.506
1069	134	32	35	B	C	91.945	1.675	3.432	1.05	-1.62	-3.362	-1.028	79.841	13.515	13.506
1070	134	32	35	N	C	33.811	1.675	-3.432	1.05	-1.62	3.362	-1.028	79.841	13.515	13.506
1071	134	31	36	N	C	10.893	2.923	0	2.923	-2.762	0	-2.762	60	14.395	14.395
1072	136	35	33	B	C	21.052	-4.167	2.108	-0.384	4.546	-2.124	0.387	79.511	13.403	13.854
1073	136	35	33	B	C	81.052	-4.167	2.108	-0.384	4.546	-2.124	0.387	79.511	13.403	13.854
1074	136	34	34	B	C	31.945	-3.326	3.459	1.675	3.563	-3.347	-1.62	89.901	13.521	13.507
1075	136	34	34	B	C	70.158	-3.326	-0.176	1.675	3.563	0.17	-1.62	88.052	13.521	13.515
1076	136	34	34	B	C	91.945	-3.326	3.459	1.675	3.563	-3.347	-1.62	89.901	13.521	13.507
1077	136	34	34	B	C	70.158	-3.326	-0.176	1.675	3.563	0.17	-1.62	88.052	13.521	13.515
1078	136	35	33	B	C	42.103	-0.889	3.27	-3.696	0.906	-3.531	3.991	79.422	13.862	13.397
1079	136	33	35	B	C	42.103	-0.889	3.468	2.087	0.906	-3.329	-2.003	79.417	13.862	13.391
1080	136	35	33	B	C	102.103	-0.889	3.27	-3.696	0.906	-3.531	3.991	79.422	13.862	13.397
1081	136	33	35	B	C	102.103	-0.889	3.468	2.087	0.906	-3.329	-2.003	79.417	13.862	13.391
1082	136	32	36	B	C	51.052	2.923	0.619	1.269	-2.762	-0.604	-1.238	69.552	14.395	13.513
1083	136	32	36	B	C	111.052	2.923	0.619	1.269	-2.762	-0.604	-1.238	69.552	14.395	13.513
1084	136	34	34	B	C	31.945	1.675	3.286	-3.326	-1.62	-3.52	3.563	89.901	13.515	13.513
1085	136	32	36	B	C	49.842	1.675	-1.538	2.508	-1.62	1.465	-2.388	69.549	13.515	14.393
1086	136	34	34	B	C	49.842	1.675	0.167	-3.326	-1.62	-0.179	3.563	88.053	13.515	13.521
1087	136	34	34	B	C	91.945	1.675	3.286	-3.326	-1.62	-3.52	3.563	89.901	13.515	13.513
1088	136	34	34	B	C	109.842	1.675	0.167	-3.326	-1.62	-0.179	3.563	88.053	13.515	13.521
1089	136	33	35	B	C	21.052	2.623	2.085	-1.389	-2.492	-2.145	1.429	79.518	13.395	13.855
1090	136	33	35	B	C	38.948	2.623	-2.085	-1.389	-2.492	2.145	1.429	79.518	13.395	13.855
1091	136	33	35	B	C	81.052	2.623	2.085	-1.389	-2.492	-2.145	1.429	79.518	13.395	13.855
1092	136	33	35	B	C	98.948	2.623	-2.085	-1.389	-2.492	2.145	1.429	79.518	13.395	13.855
1093	136	34	34	N	C	33.811	1.675	-3.286	-3.326	-1.62	3.52	3.563	89.901	13.515	13.513
1094	136	34	34	N	C	64.402	1.675	-0.167	-3.326	-1.62	0.179	3.563	88.053	13.515	13.521
1095	136	32	36	N	C	64.402	1.675	1.539	2.508	-1.62	-1.465	-2.388	69.549	13.515	14.393
1096	136	32	36	N	C	10.893	2.923	-0.619	1.269	-2.762	0.604	-1.238	69.552	14.395	13.513
1097	138	33	36	B	C	51.052	2.923	1.201	-0.284	-2.762	-1.207	0.286	80.098	14.395	13.052
1098	138	33	36	B	C	111.052	2.923	1.201	-0.284	-2.762	-1.207	0.286	80.098	14.395	13.052

1099	138	33	36	B	C	10.893	0.012	2.25	2.61	-0.012	-2.138	-2.48	80.052	13.054	14.393
1100	138	33	36	B	C	49.107	0.012	-2.25	2.61	-0.012	2.138	-2.48	80.052	13.054	14.393
1101	138	33	36	B	C	70.893	0.012	2.25	2.61	-0.012	-2.138	-2.48	80.052	13.054	14.393
1102	138	33	36	B	C	109.107	0.012	-2.25	2.61	-0.012	2.138	-2.48	80.052	13.054	14.393
1103	138	33	36	N	C	10.893	2.923	-1.2	-0.284	-2.762	1.207	0.286	80.098	14.395	13.052
1104	140	34	36	B	C	51.052	2.923	1.748	-1.747	-2.762	-1.811	1.81	88.981	14.395	13.055
1105	140	34	36	B	C	111.052	2.923	1.748	-1.747	-2.762	-1.811	1.81	88.981	14.395	13.055
1106	140	34	36	B	C	10.893	-1.774	1.668	2.954	1.84	-1.575	-2.789	89.072	13.056	14.393
1107	140	34	36	B	C	70.893	-1.774	1.668	2.954	1.84	-1.575	-2.789	89.072	13.056	14.393
1108	140	36	34	B	C	10.893	0.012	1.547	-4.454	-0.012	-1.698	4.889	89.073	13.054	14.403
1109	140	36	34	B	C	49.107	0.012	-1.547	-4.454	-0.012	1.698	4.889	89.073	13.054	14.403
1110	140	34	36	N	C	10.893	2.923	-1.748	-1.747	-2.762	1.811	1.81	88.981	14.395	13.055
1111	142	35	36	B	C	70.158	-3.326	-0.228	3.151	3.563	0.214	-2.964	78.515	13.521	14.394
1112	142	35	36	B	C	70.158	-3.326	-0.228	3.151	3.563	0.214	-2.964	78.515	13.521	14.394
1113	142	35	36	B	C	51.052	2.923	2.264	-3.125	-2.762	-2.415	3.334	78.444	14.395	13.522
1114	142	35	36	B	C	111.052	2.923	2.264	-3.125	-2.762	-2.415	3.334	78.444	14.395	13.522
1115	142	36	35	B	C	40.893	-3.595	-3.711	0.53	3.873	3.672	-0.524	77.513	12.814	15.239
1116	142	36	35	B	C	31.945	1.675	-3.472	-4.623	-1.62	3.825	5.094	76.659	13.515	14.508
1117	142	36	35	B	C	12.52	-3.595	3.711	0.53	3.873	-3.672	-0.524	77.513	12.814	15.239
1118	142	36	35	N	C	33.811	1.675	3.472	-4.623	-1.62	-3.825	5.094	76.659	13.515	14.508
1119	142	35	36	N	C	10.893	2.923	-2.264	-3.125	-2.762	2.415	3.334	78.444	14.395	13.522
1120	144	36	36	B	C	21.052	-4.167	2.561	2.623	4.546	-2.434	-2.492	71.506	13.403	15.221
1121	144	36	36	B	C	10.893	-1.774	2.1	0.012	1.84	-2.1	-0.012	74.729	13.056	15.344
1122	144	36	36	B	C	49.842	-4.167	-2.561	2.623	4.546	2.434	-2.492	71.506	13.403	15.221
1123	144	36	36	N	C	10.158	-4.167	2.561	2.623	4.546	-2.434	-2.492	71.506	13.403	15.221
1124	144	36	36	N	C	93.74	-4.167	-2.561	2.623	4.546	2.434	-2.492	71.506	13.403	15.221
1125	146	39	34	B	C	31.945	-3.326	-1.993	-4.95	3.563	2.212	5.494	86.891	13.521	14.505
1126	148	36	38	B	C	109.842	1.675	-1.263	-0.733	-1.62	1.281	0.744	75.958	13.515	15.144
1127	148	36	38	N	C	64.402	1.675	1.262	-0.733	-1.62	-1.281	0.744	75.958	13.515	15.144
