# Supplementary material for: Systematic DFT Modeling van der Waals Heterostructures from a Complete Configurational Basis Applied to γ-PC/WS2
Source: J Chem Theory Comput. 2024 Mar 6;20(6):2377–89. doi: 10.1021/acs.jctc.3c00932 (PMC10976641; doi:10.1021/acs.jctc.3c00932)
Supplement: Supplementary file 4 — ct3c00932_si_004.pdf [file ct3c00932_si_004.pdf]

BL number	Atoms	$MoSe_2$ cells	WSe_2 cells	$MoSe_2$ origin	WSe_2 origin	Twist-angle (°)	$MoSe_2$ Strain 1 (%)	$MoSe_2$ Strain 2 (%)	$MoSe_2$ Strain 3 (%)	WSe_2 Strain 1 (%)	WSe_2 Strain 2 (%)	WSe_2 Strain 3 (%)	γ (°)	|**a**|(Å)	|**b**|(Å)
1	6	1	1	Mo	W	0	-0.037	0	-0.037	0.037	0	0.037	60	3.335	3.335
2	6	1	1	Mo	W	60	-0.037	0	-0.037	0.037	0	0.037	60	3.335	3.335
3	6	1	1	Mo	Se	0	-0.037	0	-0.037	0.037	0	0.037	60	3.335	3.335
4	6	1	1	Mo	Se	60	-0.037	0	-0.037	0.037	0	0.037	60	3.335	3.335
5	6	1	1	Se	W	0	-0.037	0	-0.037	0.037	0	0.037	60	3.335	3.335
6	6	1	1	Se	Se	60	-0.037	0	-0.037	0.037	0	0.037	60	3.335	3.335
7	42	7	7	Mo	W	21.787	-0.037	0	-0.037	0.037	0	0.037	60	8.824	8.824
8	42	7	7	Mo	W	81.787	-0.037	0	-0.037	0.037	0	0.037	60	8.824	8.824
9	42	7	7	Mo	Se	21.787	-0.037	0	-0.037	0.037	0	0.037	60	8.824	8.824
10	42	7	7	Mo	Se	81.787	-0.037	0	-0.037	0.037	0	0.037	60	8.824	8.824
11	42	7	7	Mo	W	98.213	-0.037	0	-0.037	0.037	0	0.037	60	8.824	8.824
12	42	7	7	Mo	W	38.213	-0.037	0	-0.037	0.037	0	0.037	60	8.824	8.824
13	42	7	7	Mo	Se	98.213	-0.037	0	-0.037	0.037	0	0.037	60	8.824	8.824
14	42	7	7	Mo	Se	38.213	-0.037	0	-0.037	0.037	0	0.037	60	8.824	8.824
15	42	7	7	Se	W	81.787	-0.037	0	-0.037	0.037	0	0.037	60	8.824	8.824
16	42	7	7	Se	Se	21.787	-0.037	0	-0.037	0.037	0	0.037	60	8.824	8.824
17	42	7	7	Se	W	38.213	-0.037	0	-0.037	0.037	0	0.037	60	8.824	8.824
18	42	7	7	Se	Se	98.213	-0.037	0	-0.037	0.037	0	0.037	60	8.824	8.824
19	60	10	10	Mo	W	16.102	2.003	-4.077	-1.997	-1.926	4.247	2.08	68.092	11.789	8.81
20	60	10	10	Mo	W	43.898	2.003	4.077	-1.997	-1.926	-4.247	2.08	68.092	11.789	8.81
21	60	10	10	Mo	W	76.102	2.003	-4.077	-1.997	-1.926	4.247	2.08	68.092	11.789	8.81
22	60	10	10	Mo	W	103.898	2.003	4.077	-1.997	-1.926	-4.247	2.08	68.092	11.789	8.81
23	60	10	10	Mo	Se	16.102	2.003	-4.077	-1.997	-1.926	4.247	2.08	68.092	11.789	8.81
24	60	10	10	Mo	Se	43.898	2.003	4.077	-1.997	-1.926	-4.247	2.08	68.092	11.789	8.81
25	60	10	10	Mo	Se	76.102	2.003	-4.077	-1.997	-1.926	4.247	2.08	68.092	11.789	8.81
26	60	10	10	Mo	Se	103.898	2.003	4.077	-1.997	-1.926	-4.247	2.08	68.092	11.789	8.81
27	60	10	10	Mo	W	103.898	-1.997	4.244	2.003	2.08	-4.08	-1.926	68.092	11.789	8.81
28	60	10	10	Mo	W	43.898	-1.997	4.244	2.003	2.08	-4.08	-1.926	68.092	11.789	8.81
29	60	10	10	Mo	Se	103.898	-1.997	4.244	2.003	2.08	-4.08	-1.926	68.092	11.789	8.81
30	60	10	10	Mo	Se	43.898	-1.997	4.244	2.003	2.08	-4.08	-1.926	68.092	11.789	8.81
31	60	10	10	Mo	W	16.102	-1.997	-4.244	2.003	2.08	4.08	-1.926	68.092	11.789	8.81
32	60	10	10	Mo	W	76.102	-1.997	-4.244	2.003	2.08	4.08	-1.926	68.092	11.789	8.81
33	60	10	10	Mo	Se	16.102	-1.997	-4.244	2.003	2.08	4.08	-1.926	68.092	11.789	8.81
34	60	10	10	Mo	Se	76.102	-1.997	-4.244	2.003	2.08	4.08	-1.926	68.092	11.789	8.81
35	60	10	10	Se	W	16.102	-1.997	-4.244	2.003	2.08	4.08	-1.926	68.092	11.789	8.81
36	60	10	10	Se	W	76.102	-1.997	-4.244	2.003	2.08	4.08	-1.926	68.092	11.789	8.81
37	60	10	10	Se	Se	16.102	-1.997	-4.244	2.003	2.08	4.08	-1.926	68.092	11.789	8.81
38	60	10	10	Se	Se	76.102	-1.997	-4.244	2.003	2.08	4.08	-1.926	68.092	11.789	8.81
39	60	10	10	Se	W	16.102	2.003	-4.077	-1.997	-1.926	4.247	2.08	68.092	11.789	8.81
40	60	10	10	Se	W	43.898	2.003	4.077	-1.997	-1.926	-4.247	2.08	68.092	11.789	8.81
41	60	10	10	Se	W	76.102	2.003	-4.077	-1.997	-1.926	4.247	2.08	68.092	11.789	8.81
42	60	10	10	Se	W	103.898	2.003	4.077	-1.997	-1.926	-4.247	2.08	68.092	11.789	8.81

43	60	10	10	Se	Se	16.102	2.003	-4.077	-1.997	-1.926	4.247	2.08	68.092	11.789	8.81
44	60	10	10	Se	Se	43.898	2.003	4.077	-1.997	-1.926	-4.247	2.08	68.092	11.789	8.81
45	60	10	10	Se	Se	76.102	2.003	-4.077	-1.997	-1.926	4.247	2.08	68.092	11.789	8.81
46	60	10	10	Se	Se	103.898	2.003	4.077	-1.997	-1.926	-4.247	2.08	68.092	11.789	8.81
47	60	10	10	Se	W	103.898	-1.997	4.244	2.003	2.08	-4.08	-1.926	68.092	11.789	8.81
48	60	10	10	Se	W	43.898	-1.997	4.244	2.003	2.08	-4.08	-1.926	68.092	11.789	8.81
49	60	10	10	Se	Se	103.898	-1.997	4.244	2.003	2.08	-4.08	-1.926	68.092	11.789	8.81
50	60	10	10	Se	Se	43.898	-1.997	4.244	2.003	2.08	-4.08	-1.926	68.092	11.789	8.81
51	66	11	11	Mo	Se	32.204	-0.037	-5.247	-0.037	0.037	5.251	0.037	90	12.025	8.812
52	66	12	10	Mo	W	106.102	-4.964	4.924	-3.809	5.511	-5.33	4.123	72.494	9.395	11.802
53	66	11	11	Mo	Se	92.204	-0.037	-5.247	-0.037	0.037	5.251	0.037	90	12.025	8.812
54	66	10	12	Mo	W	46.102	5.429	5.326	4.043	-4.897	-4.928	-3.741	72.493	11.802	9.394
55	66	12	10	Mo	Se	13.898	-4.964	-4.924	-3.809	5.511	5.33	4.123	72.494	11.802	9.395
56	66	10	12	Mo	W	106.102	5.429	5.326	4.043	-4.897	-4.928	-3.741	72.493	11.802	9.394
57	66	10	12	Mo	Se	46.102	5.429	5.326	4.043	-4.897	-4.928	-3.741	72.493	11.802	9.394
58	66	10	12	Mo	Se	106.102	5.429	5.326	4.043	-4.897	-4.928	-3.741	72.493	11.802	9.394
59	66	11	11	Mo	W	21.787	-0.037	-5.247	-0.037	0.037	5.251	0.037	90	8.824	12.008
60	66	11	11	Mo	W	81.787	-0.037	-5.247	-0.037	0.037	5.251	0.037	90	8.824	12.008
61	66	11	11	Mo	Se	21.787	-0.037	-5.247	-0.037	0.037	5.251	0.037	90	8.824	12.008
62	66	11	11	Mo	Se	81.787	-0.037	-5.247	-0.037	0.037	5.251	0.037	90	8.824	12.008
63	66	12	10	Mo	W	73.898	-4.964	-4.924	-3.809	5.511	5.33	4.123	72.494	11.802	9.395
64	66	12	10	Mo	Se	46.102	-4.964	4.924	-3.809	5.511	-5.33	4.123	72.494	9.395	11.802
65	66	12	10	Mo	Se	73.898	-4.964	-4.924	-3.809	5.511	5.33	4.123	72.494	11.802	9.395
66	66	11	11	Mo	W	98.213	-0.037	5.247	-0.037	0.037	-5.251	0.037	90	8.824	12.008
67	66	11	11	Mo	W	38.213	-0.037	5.247	-0.037	0.037	-5.251	0.037	90	8.824	12.008
68	66	11	11	Mo	Se	98.213	-0.037	5.247	-0.037	0.037	-5.251	0.037	90	8.824	12.008
69	66	11	11	Mo	Se	38.213	-0.037	5.247	-0.037	0.037	-5.251	0.037	90	8.824	12.008
70	66	11	11	Mo	W	87.796	-0.037	5.247	-0.037	0.037	-5.251	0.037	90	12.025	8.812
71	66	11	11	Mo	W	27.796	-0.037	5.247	-0.037	0.037	-5.251	0.037	90	12.025	8.812
72	66	11	11	Mo	Se	87.796	-0.037	5.247	-0.037	0.037	-5.251	0.037	90	12.025	8.812
73	66	11	11	Mo	Se	27.796	-0.037	5.247	-0.037	0.037	-5.251	0.037	90	12.025	8.812
74	66	10	12	Mo	W	13.898	5.429	-5.326	4.043	-4.897	4.928	-3.741	72.493	9.394	11.802
75	66	10	12	Mo	W	73.898	5.429	-5.326	4.043	-4.897	4.928	-3.741	72.493	9.394	11.802
76	66	10	12	Mo	Se	13.898	5.429	-5.326	4.043	-4.897	4.928	-3.741	72.493	9.394	11.802
77	66	12	10	Mo	Se	106.102	-4.964	4.924	-3.809	5.511	-5.33	4.123	72.494	9.395	11.802
78	66	10	12	Mo	Se	73.898	5.429	-5.326	4.043	-4.897	4.928	-3.741	72.493	9.394	11.802
79	66	12	10	Mo	W	13.898	-4.964	-4.924	-3.809	5.511	5.33	4.123	72.494	11.802	9.395
80	66	12	10	Mo	W	46.102	-4.964	4.924	-3.809	5.511	-5.33	4.123	72.494	9.395	11.802
81	66	11	11	Mo	W	32.204	-0.037	-5.247	-0.037	0.037	5.251	0.037	90	12.025	8.812
82	66	11	11	Mo	W	92.204	-0.037	-5.247	-0.037	0.037	5.251	0.037	90	12.025	8.812
83	66	12	10	Se	W	13.898	-4.964	-4.924	-3.809	5.511	5.33	4.123	72.494	11.802	9.395
84	66	12	10	Se	W	46.102	-4.964	4.924	-3.809	5.511	-5.33	4.123	72.494	9.395	11.802
85	66	12	10	Se	W	73.898	-4.964	-4.924	-3.809	5.511	5.33	4.123	72.494	11.802	9.395
86	66	12	10	Se	W	106.102	-4.964	4.924	-3.809	5.511	-5.33	4.123	72.494	9.395	11.802

87	66	12	10	Se	Se	13.898	-4.964	-4.924	-3.809	5.511	5.33	4.123	72.494	11.802	9.395
88	66	12	10	Se	Se	46.102	-4.964	4.924	-3.809	5.511	-5.33	4.123	72.494	9.395	11.802
89	66	12	10	Se	Se	73.898	-4.964	-4.924	-3.809	5.511	5.33	4.123	72.494	11.802	9.395
90	66	12	10	Se	Se	106.102	-4.964	4.924	-3.809	5.511	-5.33	4.123	72.494	9.395	11.802
91	66	11	11	Se	W	32.204	-0.037	-5.247	-0.037	0.037	5.251	0.037	90	12.025	8.812
92	66	11	11	Se	W	92.204	-0.037	-5.247	-0.037	0.037	5.251	0.037	90	12.025	8.812
93	66	11	11	Se	Se	32.204	-0.037	-5.247	-0.037	0.037	5.251	0.037	90	12.025	8.812
94	66	11	11	Se	Se	92.204	-0.037	-5.247	-0.037	0.037	5.251	0.037	90	12.025	8.812
95	66	10	12	Se	W	46.102	5.429	5.326	4.043	-4.897	-4.928	-3.741	72.493	11.802	9.394
96	66	10	12	Se	W	106.102	5.429	5.326	4.043	-4.897	-4.928	-3.741	72.493	11.802	9.394
97	66	10	12	Se	Se	46.102	5.429	5.326	4.043	-4.897	-4.928	-3.741	72.493	11.802	9.394
98	66	10	12	Se	Se	106.102	5.429	5.326	4.043	-4.897	-4.928	-3.741	72.493	11.802	9.394
99	66	11	11	Se	W	21.787	-0.037	-5.247	-0.037	0.037	5.251	0.037	90	8.824	12.008
100	66	11	11	Se	W	81.787	-0.037	-5.247	-0.037	0.037	5.251	0.037	90	8.824	12.008
101	66	11	11	Se	Se	21.787	-0.037	-5.247	-0.037	0.037	5.251	0.037	90	8.824	12.008
102	66	11	11	Se	Se	81.787	-0.037	-5.247	-0.037	0.037	5.251	0.037	90	8.824	12.008
103	66	11	11	Se	W	98.213	-0.037	5.247	-0.037	0.037	-5.251	0.037	90	8.824	12.008
104	66	11	11	Se	W	38.213	-0.037	5.247	-0.037	0.037	-5.251	0.037	90	8.824	12.008
105	66	11	11	Se	Se	98.213	-0.037	5.247	-0.037	0.037	-5.251	0.037	90	8.824	12.008
106	66	11	11	Se	Se	38.213	-0.037	5.247	-0.037	0.037	-5.251	0.037	90	8.824	12.008
107	66	11	11	Se	W	87.796	-0.037	5.247	-0.037	0.037	-5.251	0.037	90	12.025	8.812
108	66	11	11	Se	W	27.796	-0.037	5.247	-0.037	0.037	-5.251	0.037	90	12.025	8.812
109	66	11	11	Se	Se	87.796	-0.037	5.247	-0.037	0.037	-5.251	0.037	90	12.025	8.812
110	66	11	11	Se	Se	27.796	-0.037	5.247	-0.037	0.037	-5.251	0.037	90	12.025	8.812
111	66	10	12	Se	W	13.898	5.429	-5.326	4.043	-4.897	4.928	-3.741	72.493	9.394	11.802
112	66	10	12	Se	W	73.898	5.429	-5.326	4.043	-4.897	4.928	-3.741	72.493	9.394	11.802
113	66	10	12	Se	Se	13.898	5.429	-5.326	4.043	-4.897	4.928	-3.741	72.493	9.394	11.802
114	66	10	12	Se	Se	73.898	5.429	-5.326	4.043	-4.897	4.928	-3.741	72.493	9.394	11.802
115	75	12	13	Mo	W	21.787	-0.037	-2.405	4.127	0.037	2.221	-3.812	78.258	8.824	13.937
116	75	13	12	Mo	W	21.787	-0.037	-2.22	-3.88	0.037	2.406	4.206	78.259	8.824	13.937
117	75	12	13	Mo	W	81.787	-0.037	-2.405	4.127	0.037	2.221	-3.812	78.258	8.824	13.937
118	75	13	12	Mo	W	81.787	-0.037	-2.22	-3.88	0.037	2.406	4.206	78.259	8.824	13.937
119	75	12	13	Mo	Se	21.787	-0.037	-2.405	4.127	0.037	2.221	-3.812	78.258	8.824	13.937
120	75	13	12	Mo	Se	21.787	-0.037	-2.22	-3.88	0.037	2.406	4.206	78.259	8.824	13.937
121	75	12	13	Mo	Se	81.787	-0.037	-2.405	4.127	0.037	2.221	-3.812	78.258	8.824	13.937
122	75	13	12	Mo	Se	81.787	-0.037	-2.22	-3.88	0.037	2.406	4.206	78.259	8.824	13.937
123	75	13	12	Mo	W	36.587	-4.151	-0.532	0.258	4.526	0.529	-0.257	78.306	13.939	8.822
124	75	13	12	Mo	W	96.587	-4.151	-0.532	0.258	4.526	0.529	-0.257	78.306	13.939	8.822
125	75	13	12	Mo	Se	36.587	-4.151	-0.532	0.258	4.526	0.529	-0.257	78.306	13.939	8.822
126	75	13	12	Mo	Se	96.587	-4.151	-0.532	0.258	4.526	0.529	-0.257	78.306	13.939	8.822
127	75	12	13	Mo	W	16.102	2.003	0	2.003	-1.926	0	-1.926	60	11.789	11.789
128	75	12	13	Mo	W	43.898	2.003	0	2.003	-1.926	0	-1.926	60	11.789	11.789
129	75	12	13	Mo	W	76.102	2.003	0	2.003	-1.926	0	-1.926	60	11.789	11.789
130	75	12	13	Mo	W	103.898	2.003	0	2.003	-1.926	0	-1.926	60	11.789	11.789

131	75	13	12	Mo	W	23.413	-4.151	0.532	0.258	4.526	-0.529	-0.257	78.306	13.939	8.822
132	75	13	12	Mo	W	83.413	-4.151	0.532	0.258	4.526	-0.529	-0.257	78.306	13.939	8.822
133	75	13	12	Mo	Se	23.413	-4.151	0.532	0.258	4.526	-0.529	-0.257	78.306	13.939	8.822
134	75	13	12	Mo	Se	83.413	-4.151	0.532	0.258	4.526	-0.529	-0.257	78.306	13.939	8.822
135	75	13	12	Mo	W	98.213	-0.037	2.22	-3.88	0.037	-2.406	4.206	78.259	8.824	13.937
136	75	12	13	Mo	W	98.213	-0.037	2.405	4.127	0.037	-2.221	-3.812	78.258	8.824	13.937
137	75	13	12	Mo	W	38.213	-0.037	2.22	-3.88	0.037	-2.406	4.206	78.259	8.824	13.937
138	75	12	13	Mo	W	38.213	-0.037	2.405	4.127	0.037	-2.221	-3.812	78.258	8.824	13.937
139	75	13	12	Mo	Se	98.213	-0.037	2.22	-3.88	0.037	-2.406	4.206	78.259	8.824	13.937
140	75	12	13	Mo	Se	98.213	-0.037	2.405	4.127	0.037	-2.221	-3.812	78.258	8.824	13.937
141	75	13	12	Mo	Se	38.213	-0.037	2.22	-3.88	0.037	-2.406	4.206	78.259	8.824	13.937
142	75	12	13	Mo	Se	38.213	-0.037	2.405	4.127	0.037	-2.221	-3.812	78.258	8.824	13.937
143	75	13	12	Mo	W	103.898	-1.997	0	-1.997	2.08	0	2.08	60	11.789	11.789
144	75	13	12	Mo	W	43.898	-1.997	0	-1.997	2.08	0	2.08	60	11.789	11.789
145	75	13	12	Mo	Se	103.898	-1.997	0	-1.997	2.08	0	2.08	60	11.789	11.789
146	75	13	12	Mo	Se	43.898	-1.997	0	-1.997	2.08	0	2.08	60	11.789	11.789
147	75	12	13	Mo	W	23.413	4.446	0.529	-0.33	-4.083	-0.532	0.332	78.306	13.938	8.822
148	75	12	13	Mo	W	36.587	4.446	-0.529	-0.33	-4.083	0.532	0.332	78.306	13.938	8.822
149	75	12	13	Mo	W	83.413	4.446	0.529	-0.33	-4.083	-0.532	0.332	78.306	13.938	8.822
150	75	12	13	Mo	W	96.587	4.446	-0.529	-0.33	-4.083	0.532	0.332	78.306	13.938	8.822
151	75	12	13	Mo	Se	23.413	4.446	0.529	-0.33	-4.083	-0.532	0.332	78.306	13.938	8.822
152	75	12	13	Mo	Se	36.587	4.446	-0.529	-0.33	-4.083	0.532	0.332	78.306	13.938	8.822
153	75	12	13	Mo	Se	83.413	4.446	0.529	-0.33	-4.083	-0.532	0.332	78.306	13.938	8.822
154	75	12	13	Mo	Se	96.587	4.446	-0.529	-0.33	-4.083	0.532	0.332	78.306	13.938	8.822
155	75	13	12	Mo	W	16.102	-1.997	0	-1.997	2.08	0	2.08	60	11.789	11.789
156	75	13	12	Mo	W	76.102	-1.997	0	-1.997	2.08	0	2.08	60	11.789	11.789
157	75	13	12	Mo	Se	16.102	-1.997	0	-1.997	2.08	0	2.08	60	11.789	11.789
158	75	13	12	Mo	Se	76.102	-1.997	0	-1.997	2.08	0	2.08	60	11.789	11.789
159	75	12	13	Se	W	23.413	4.446	0.529	-0.33	-4.083	-0.532	0.332	78.306	13.938	8.822
160	75	12	13	Se	W	36.587	4.446	-0.529	-0.33	-4.083	0.532	0.332	78.306	13.938	8.822
161	75	12	13	Se	W	83.413	4.446	0.529	-0.33	-4.083	-0.532	0.332	78.306	13.938	8.822
162	75	12	13	Se	W	96.587	4.446	-0.529	-0.33	-4.083	0.532	0.332	78.306	13.938	8.822
163	75	12	13	Se	Se	23.413	4.446	0.529	-0.33	-4.083	-0.532	0.332	78.306	13.938	8.822
164	75	12	13	Se	Se	36.587	4.446	-0.529	-0.33	-4.083	0.532	0.332	78.306	13.938	8.822
165	75	12	13	Se	Se	83.413	4.446	0.529	-0.33	-4.083	-0.532	0.332	78.306	13.938	8.822
166	75	12	13	Se	Se	96.587	4.446	-0.529	-0.33	-4.083	0.532	0.332	78.306	13.938	8.822
167	75	12	13	Se	W	21.787	-0.037	-2.405	4.127	0.037	2.221	-3.812	78.258	8.824	13.937
168	75	13	12	Se	W	21.787	-0.037	-2.22	-3.88	0.037	2.406	4.206	78.259	8.824	13.937
169	75	12	13	Se	W	81.787	-0.037	-2.405	4.127	0.037	2.221	-3.812	78.258	8.824	13.937
170	75	13	12	Se	W	81.787	-0.037	-2.22	-3.88	0.037	2.406	4.206	78.259	8.824	13.937
171	75	12	13	Se	Se	21.787	-0.037	-2.405	4.127	0.037	2.221	-3.812	78.258	8.824	13.937
172	75	13	12	Se	Se	21.787	-0.037	-2.22	-3.88	0.037	2.406	4.206	78.259	8.824	13.937
173	75	12	13	Se	Se	81.787	-0.037	-2.405	4.127	0.037	2.221	-3.812	78.258	8.824	13.937
174	75	13	12	Se	Se	81.787	-0.037	-2.22	-3.88	0.037	2.406	4.206	78.259	8.824	13.937

175	75	13	12	Se	W	36.587	-4.151	-0.532	0.258	4.526	0.529	-0.257	78.306	13.939	8.822
176	75	13	12	Se	W	96.587	-4.151	-0.532	0.258	4.526	0.529	-0.257	78.306	13.939	8.822
177	75	13	12	Se	Se	36.587	-4.151	-0.532	0.258	4.526	0.529	-0.257	78.306	13.939	8.822
178	75	13	12	Se	Se	96.587	-4.151	-0.532	0.258	4.526	0.529	-0.257	78.306	13.939	8.822
179	75	12	13	Se	W	16.102	2.003	0	2.003	-1.926	0	-1.926	60	11.789	11.789
180	75	12	13	Se	W	43.898	2.003	0	2.003	-1.926	0	-1.926	60	11.789	11.789
181	75	12	13	Se	W	76.102	2.003	0	2.003	-1.926	0	-1.926	60	11.789	11.789
182	75	12	13	Se	W	103.898	2.003	0	2.003	-1.926	0	-1.926	60	11.789	11.789
183	75	13	12	Se	W	23.413	-4.151	0.532	0.258	4.526	-0.529	-0.257	78.306	13.939	8.822
184	75	13	12	Se	W	83.413	-4.151	0.532	0.258	4.526	-0.529	-0.257	78.306	13.939	8.822
185	75	13	12	Se	Se	23.413	-4.151	0.532	0.258	4.526	-0.529	-0.257	78.306	13.939	8.822
186	75	13	12	Se	Se	83.413	-4.151	0.532	0.258	4.526	-0.529	-0.257	78.306	13.939	8.822
187	75	13	12	Se	W	98.213	-0.037	2.22	-3.88	0.037	-2.406	4.206	78.259	8.824	13.937
188	75	12	13	Se	W	98.213	-0.037	2.405	4.127	0.037	-2.221	-3.812	78.258	8.824	13.937
189	75	13	12	Se	W	38.213	-0.037	2.22	-3.88	0.037	-2.406	4.206	78.259	8.824	13.937
190	75	12	13	Se	W	38.213	-0.037	2.405	4.127	0.037	-2.221	-3.812	78.258	8.824	13.937
191	75	13	12	Se	Se	98.213	-0.037	2.22	-3.88	0.037	-2.406	4.206	78.259	8.824	13.937
192	75	12	13	Se	Se	98.213	-0.037	2.405	4.127	0.037	-2.221	-3.812	78.258	8.824	13.937
193	75	13	12	Se	Se	38.213	-0.037	2.22	-3.88	0.037	-2.406	4.206	78.259	8.824	13.937
194	75	12	13	Se	Se	38.213	-0.037	2.405	4.127	0.037	-2.221	-3.812	78.258	8.824	13.937
195	78	13	13	Mo	Se	32.204	-0.037	0	-0.037	0.037	0	0.037	60	12.025	12.025
196	78	13	13	Mo	Se	92.204	-0.037	0	-0.037	0.037	0	0.037	60	12.025	12.025
197	78	13	13	Mo	W	87.796	-0.037	0	-0.037	0.037	0	0.037	60	12.025	12.025
198	78	13	13	Mo	W	27.796	-0.037	0	-0.037	0.037	0	0.037	60	12.025	12.025
199	78	13	13	Mo	Se	87.796	-0.037	0	-0.037	0.037	0	0.037	60	12.025	12.025
200	78	13	13	Mo	Se	27.796	-0.037	0	-0.037	0.037	0	0.037	60	12.025	12.025
201	78	13	13	Mo	W	32.204	-0.037	0	-0.037	0.037	0	0.037	60	12.025	12.025
202	78	13	13	Mo	W	92.204	-0.037	0	-0.037	0.037	0	0.037	60	12.025	12.025
203	78	13	13	Se	W	92.204	-0.037	0	-0.037	0.037	0	0.037	60	12.025	12.025
204	78	13	13	Se	Se	32.204	-0.037	0	-0.037	0.037	0	0.037	60	12.025	12.025
205	78	13	13	Se	W	27.796	-0.037	0	-0.037	0.037	0	0.037	60	12.025	12.025
206	78	13	13	Se	Se	87.796	-0.037	0	-0.037	0.037	0	0.037	60	12.025	12.025
207	84	14	14	Mo	W	13.174	-0.037	-4.122	-0.037	0.037	4.125	0.037	76.161	11.785	11.785
208	84	14	14	Mo	W	73.174	-0.037	-4.122	-0.037	0.037	4.125	0.037	76.161	11.785	11.785
209	84	14	14	Mo	Se	13.174	-0.037	-4.122	-0.037	0.037	4.125	0.037	76.161	11.785	11.785
210	84	14	14	Mo	Se	73.174	-0.037	-4.122	-0.037	0.037	4.125	0.037	76.161	11.785	11.785
211	84	14	14	Mo	W	43.898	2.003	-0.971	-1.997	-1.926	1.011	2.08	76.092	11.789	11.789
212	84	14	14	Mo	W	76.102	2.003	0.971	-1.997	-1.926	-1.011	2.08	76.092	11.789	11.789
213	84	14	14	Mo	W	103.898	2.003	-0.971	-1.997	-1.926	1.011	2.08	76.092	11.789	11.789
214	84	14	14	Mo	Se	16.102	2.003	0.971	-1.997	-1.926	-1.011	2.08	76.092	11.789	11.789
215	84	14	14	Mo	Se	43.898	2.003	-0.971	-1.997	-1.926	1.011	2.08	76.092	11.789	11.789
216	84	14	14	Mo	Se	103.898	2.003	-0.971	-1.997	-1.926	1.011	2.08	76.092	11.789	11.789
217	84	14	14	Mo	W	106.826	-0.037	4.122	-0.037	0.037	-4.125	0.037	76.161	11.785	11.785
218	84	14	14	Mo	W	46.826	-0.037	4.122	-0.037	0.037	-4.126	0.037	76.161	11.785	11.785

219	84	14	14	Mo	Se	106.826	-0.037	4.122	-0.037	0.037	-4.125	0.037	76.161	11.785	11.785
220	84	14	14	Mo	Se	46.826	-0.037	4.122	-0.037	0.037	-4.126	0.037	76.161	11.785	11.785
221	84	14	14	Mo	W	43.898	-1.997	-1.01	2.003	2.08	0.971	-1.926	76.091	11.789	11.789
222	84	14	14	Mo	Se	103.898	-1.997	-1.01	2.003	2.08	0.971	-1.926	76.091	11.789	11.789
223	84	14	14	Mo	W	16.102	-1.997	1.01	2.003	2.08	-0.971	-1.926	76.091	11.789	11.789
224	84	14	14	Mo	W	76.102	-1.997	1.01	2.003	2.08	-0.971	-1.926	76.091	11.789	11.789
225	84	14	14	Mo	Se	16.102	-1.997	1.01	2.003	2.08	-0.971	-1.926	76.091	11.789	11.789
226	84	14	14	Mo	Se	76.102	-1.997	1.01	2.003	2.08	-0.971	-1.926	76.091	11.789	11.789
227	84	14	14	Se	W	16.102	-1.997	1.01	2.003	2.08	-0.971	-1.926	76.091	11.789	11.789
228	84	14	14	Se	W	76.102	-1.997	1.01	2.003	2.08	-0.971	-1.926	76.091	11.789	11.789
229	84	14	14	Se	Se	16.102	-1.997	1.01	2.003	2.08	-0.971	-1.926	76.091	11.789	11.789
230	84	14	14	Se	Se	76.102	-1.997	1.01	2.003	2.08	-0.971	-1.926	76.091	11.789	11.789
231	84	14	14	Se	W	13.174	-0.037	-4.122	-0.037	0.037	4.125	0.037	76.161	11.785	11.785
232	84	14	14	Se	W	73.174	-0.037	-4.122	-0.037	0.037	4.125	0.037	76.161	11.785	11.785
233	84	14	14	Se	Se	13.174	-0.037	-4.122	-0.037	0.037	4.125	0.037	76.161	11.785	11.785
234	84	14	14	Se	Se	73.174	-0.037	-4.122	-0.037	0.037	4.125	0.037	76.161	11.785	11.785
235	84	14	14	Se	W	16.102	2.003	0.971	-1.997	-1.926	-1.011	2.08	76.092	11.789	11.789
236	84	14	14	Se	W	43.898	2.003	-0.971	-1.997	-1.926	1.011	2.08	76.092	11.789	11.789
237	84	14	14	Se	W	103.898	2.003	-0.971	-1.997	-1.926	1.011	2.08	76.092	11.789	11.789
238	84	14	14	Se	Se	43.898	2.003	-0.971	-1.997	-1.926	1.011	2.08	76.092	11.789	11.789
239	84	14	14	Se	Se	76.102	2.003	0.971	-1.997	-1.926	-1.011	2.08	76.092	11.789	11.789
240	84	14	14	Se	Se	103.898	2.003	-0.971	-1.997	-1.926	1.011	2.08	76.092	11.789	11.789
241	84	14	14	Se	W	106.826	-0.037	4.122	-0.037	0.037	-4.125	0.037	76.161	11.785	11.785
242	84	14	14	Se	W	46.826	-0.037	4.122	-0.037	0.037	-4.126	0.037	76.161	11.785	11.785
243	84	14	14	Se	Se	106.826	-0.037	4.122	-0.037	0.037	-4.125	0.037	76.161	11.785	11.785
244	84	14	14	Se	Se	46.826	-0.037	4.122	-0.037	0.037	-4.126	0.037	76.161	11.785	11.785
245	84	14	14	Se	W	103.898	-1.997	-1.01	2.003	2.08	0.971	-1.926	76.091	11.789	11.789
246	84	14	14	Se	Se	43.898	-1.997	-1.01	2.003	2.08	0.971	-1.926	76.091	11.789	11.789
247	87	13	16	Mo	W	46.102	5.429	0	5.429	-4.897	0	-4.897	60	12.682	12.682
248	87	13	16	Mo	W	106.102	5.429	0	5.429	-4.897	0	-4.897	60	12.682	12.682
249	87	13	16	Mo	Se	46.102	5.429	0	5.429	-4.897	0	-4.897	60	12.682	12.682
250	87	13	16	Mo	Se	106.102	5.429	0	5.429	-4.897	0	-4.897	60	12.682	12.682
251	87	13	16	Mo	W	13.898	5.429	0	5.429	-4.897	0	-4.897	60	12.682	12.682
252	87	13	16	Mo	W	73.898	5.429	0	5.429	-4.897	0	-4.897	60	12.682	12.682
253	87	13	16	Mo	Se	13.898	5.429	0	5.429	-4.897	0	-4.897	60	12.682	12.682
254	87	13	16	Mo	Se	73.898	5.429	0	5.429	-4.897	0	-4.897	60	12.682	12.682
255	87	13	16	Se	W	46.102	5.429	0	5.429	-4.897	0	-4.897	60	12.682	12.682
256	87	13	16	Se	Se	106.102	5.429	0	5.429	-4.897	0	-4.897	60	12.682	12.682
257	87	13	16	Se	W	73.898	5.429	0	5.429	-4.897	0	-4.897	60	12.682	12.682
258	87	13	16	Se	Se	13.898	5.429	0	5.429	-4.897	0	-4.897	60	12.682	12.682
259	90	15	15	Mo	W	92.204	-0.037	3.848	-0.037	0.037	-3.85	0.037	90	12.025	12.016
260	90	15	15	Mo	Se	32.204	-0.037	3.848	-0.037	0.037	-3.85	0.037	90	12.025	12.016
261	90	15	15	Mo	Se	92.204	-0.037	3.848	-0.037	0.037	-3.85	0.037	90	12.025	12.016
262	90	14	16	Mo	W	46.102	5.429	1.087	1.47	-4.897	-1.056	-1.428	75.008	12.682	11.785

263	90	14	16	Mo	W	106.102	5.429	1.087	1.47	-4.897	-1.056	-1.428	75.008	12.682	11.785
264	90	14	16	Mo	Se	46.102	5.429	1.087	1.47	-4.897	-1.056	-1.428	75.008	12.682	11.785
265	90	14	16	Mo	Se	106.102	5.429	1.087	1.47	-4.897	-1.056	-1.428	75.008	12.682	11.785
266	90	16	14	Mo	Se	13.898	-4.964	-1.055	-1.499	5.511	1.088	1.546	75.007	12.683	11.785
267	90	14	16	Mo	W	16.102	2.003	2.912	4.861	-1.926	-2.654	-4.43	74.972	11.789	12.678
268	90	16	14	Mo	Se	46.102	-4.964	1.055	-1.499	5.511	-1.088	1.546	75.007	12.683	11.785
269	90	14	16	Mo	W	43.898	2.003	-2.912	4.861	-1.926	2.654	-4.43	74.972	11.789	12.678
270	90	14	16	Mo	W	76.102	2.003	2.912	4.861	-1.926	-2.654	-4.43	74.972	11.789	12.678
271	90	14	16	Mo	W	103.898	2.003	-2.912	4.861	-1.926	2.654	-4.43	74.972	11.789	12.678
272	90	14	16	Mo	Se	16.102	2.003	2.912	4.861	-1.926	-2.654	-4.43	74.972	11.789	12.678
273	90	14	16	Mo	Se	43.898	2.003	-2.912	4.861	-1.926	2.654	-4.43	74.972	11.789	12.678
274	90	14	16	Mo	Se	76.102	2.003	2.912	4.861	-1.926	-2.654	-4.43	74.972	11.789	12.678
275	90	14	16	Mo	Se	103.898	2.003	-2.912	4.861	-1.926	2.654	-4.43	74.972	11.789	12.678
276	90	16	14	Mo	Se	73.898	-4.964	-1.055	-1.499	5.511	1.088	1.546	75.007	12.683	11.785
277	90	16	14	Mo	W	103.898	-1.997	-2.652	-4.497	2.08	2.914	4.942	74.973	11.789	12.679
278	90	16	14	Mo	W	43.898	-1.997	-2.652	-4.497	2.08	2.914	4.942	74.973	11.789	12.679
279	90	16	14	Mo	Se	103.898	-1.997	-2.652	-4.497	2.08	2.914	4.942	74.973	11.789	12.679
280	90	16	14	Mo	Se	43.898	-1.997	-2.652	-4.497	2.08	2.914	4.942	74.973	11.789	12.679
281	90	15	15	Mo	W	87.796	-0.037	-3.848	-0.037	0.037	3.85	0.037	90	12.025	12.016
282	90	16	14	Mo	Se	106.102	-4.964	1.055	-1.499	5.511	-1.088	1.546	75.007	12.683	11.785
283	90	15	15	Mo	W	27.796	-0.037	-3.848	-0.037	0.037	3.85	0.037	90	12.025	12.016
284	90	15	15	Mo	Se	87.796	-0.037	-3.848	-0.037	0.037	3.85	0.037	90	12.025	12.016
285	90	15	15	Mo	Se	27.796	-0.037	-3.848	-0.037	0.037	3.85	0.037	90	12.025	12.016
286	90	14	16	Mo	W	13.898	5.429	-1.087	1.47	-4.897	1.056	-1.428	75.008	12.682	11.785
287	90	14	16	Mo	W	73.898	5.429	-1.087	1.47	-4.897	1.056	-1.428	75.008	12.682	11.785
288	90	14	16	Mo	Se	13.898	5.429	-1.087	1.47	-4.897	1.056	-1.428	75.008	12.682	11.785
289	90	14	16	Mo	Se	73.898	5.429	-1.087	1.47	-4.897	1.056	-1.428	75.008	12.682	11.785
290	90	16	14	Mo	W	13.898	-4.964	-1.055	-1.499	5.511	1.088	1.546	75.007	12.683	11.785
291	90	16	14	Mo	W	46.102	-4.964	1.055	-1.499	5.511	-1.088	1.546	75.007	12.683	11.785
292	90	16	14	Mo	W	73.898	-4.964	-1.055	-1.499	5.511	1.088	1.546	75.007	12.683	11.785
293	90	16	14	Mo	W	16.102	-1.997	2.652	-4.497	2.08	-2.914	4.941	74.973	11.789	12.679
294	90	16	14	Mo	W	76.102	-1.997	2.652	-4.497	2.08	-2.914	4.942	74.973	11.789	12.679
295	90	16	14	Mo	W	106.102	-4.964	1.055	-1.499	5.511	-1.088	1.546	75.007	12.683	11.785
296	90	16	14	Mo	Se	16.102	-1.997	2.652	-4.497	2.08	-2.914	4.941	74.973	11.789	12.679
297	90	16	14	Mo	Se	76.102	-1.997	2.652	-4.497	2.08	-2.914	4.942	74.973	11.789	12.679
298	90	15	15	Mo	W	32.204	-0.037	3.848	-0.037	0.037	-3.85	0.037	90	12.025	12.016
299	90	16	14	Se	W	13.898	-4.964	-1.055	-1.499	5.511	1.088	1.546	75.007	12.683	11.785
300	90	16	14	Se	W	46.102	-4.964	1.055	-1.499	5.511	-1.088	1.546	75.007	12.683	11.785
301	90	16	14	Se	W	73.898	-4.964	-1.055	-1.499	5.511	1.088	1.546	75.007	12.683	11.785
302	90	16	14	Se	W	106.102	-4.964	1.055	-1.499	5.511	-1.088	1.546	75.007	12.683	11.785
303	90	16	14	Se	Se	13.898	-4.964	-1.055	-1.499	5.511	1.088	1.546	75.007	12.683	11.785
304	90	16	14	Se	Se	46.102	-4.964	1.055	-1.499	5.511	-1.088	1.546	75.007	12.683	11.785
305	90	16	14	Se	Se	73.898	-4.964	-1.055	-1.499	5.511	1.088	1.546	75.007	12.683	11.785
306	90	16	14	Se	Se	106.102	-4.964	1.055	-1.499	5.511	-1.088	1.546	75.007	12.683	11.785

307	90	16	14	Se	W	16.102	-1.997	2.652	-4.497	2.08	-2.914	4.941	74.973	11.789	12.679
308	90	16	14	Se	W	76.102	-1.997	2.652	-4.497	2.08	-2.914	4.942	74.973	11.789	12.679
309	90	16	14	Se	Se	16.102	-1.997	2.652	-4.497	2.08	-2.914	4.941	74.973	11.789	12.679
310	90	16	14	Se	Se	76.102	-1.997	2.652	-4.497	2.08	-2.914	4.942	74.973	11.789	12.679
311	90	15	15	Se	W	32.204	-0.037	3.848	-0.037	0.037	-3.85	0.037	90	12.025	12.016
312	90	15	15	Se	W	92.204	-0.037	3.848	-0.037	0.037	-3.85	0.037	90	12.025	12.016
313	90	15	15	Se	Se	32.204	-0.037	3.848	-0.037	0.037	-3.85	0.037	90	12.025	12.016
314	90	15	15	Se	Se	92.204	-0.037	3.848	-0.037	0.037	-3.85	0.037	90	12.025	12.016
315	90	14	16	Se	W	46.102	5.429	1.087	1.47	-4.897	-1.056	-1.428	75.008	12.682	11.785
316	90	14	16	Se	W	106.102	5.429	1.087	1.47	-4.897	-1.056	-1.428	75.008	12.682	11.785
317	90	14	16	Se	Se	46.102	5.429	1.087	1.47	-4.897	-1.056	-1.428	75.008	12.682	11.785
318	90	14	16	Se	Se	106.102	5.429	1.087	1.47	-4.897	-1.056	-1.428	75.008	12.682	11.785
319	90	14	16	Se	W	16.102	2.003	2.912	4.861	-1.926	-2.654	-4.43	74.972	11.789	12.678
320	90	14	16	Se	W	43.898	2.003	-2.912	4.861	-1.926	2.654	-4.43	74.972	11.789	12.678
321	90	14	16	Se	W	76.102	2.003	2.912	4.861	-1.926	-2.654	-4.43	74.972	11.789	12.678
322	90	14	16	Se	W	103.898	2.003	-2.912	4.861	-1.926	2.654	-4.43	74.972	11.789	12.678
323	90	14	16	Se	Se	16.102	2.003	2.912	4.861	-1.926	-2.654	-4.43	74.972	11.789	12.678
324	90	14	16	Se	Se	43.898	2.003	-2.912	4.861	-1.926	2.654	-4.43	74.972	11.789	12.678
325	90	14	16	Se	Se	76.102	2.003	2.912	4.861	-1.926	-2.654	-4.43	74.972	11.789	12.678
326	90	14	16	Se	Se	103.898	2.003	-2.912	4.861	-1.926	2.654	-4.43	74.972	11.789	12.678
327	90	16	14	Se	W	103.898	-1.997	-2.652	-4.497	2.08	2.914	4.942	74.973	11.789	12.679
328	90	16	14	Se	W	43.898	-1.997	-2.652	-4.497	2.08	2.914	4.942	74.973	11.789	12.679
329	90	16	14	Se	Se	103.898	-1.997	-2.652	-4.497	2.08	2.914	4.942	74.973	11.789	12.679
330	90	16	14	Se	Se	43.898	-1.997	-2.652	-4.497	2.08	2.914	4.942	74.973	11.789	12.679
331	90	15	15	Se	W	87.796	-0.037	-3.848	-0.037	0.037	3.85	0.037	90	12.025	12.016
332	90	15	15	Se	W	27.796	-0.037	-3.848	-0.037	0.037	3.85	0.037	90	12.025	12.016
333	90	15	15	Se	Se	87.796	-0.037	-3.848	-0.037	0.037	3.85	0.037	90	12.025	12.016
334	90	15	15	Se	Se	27.796	-0.037	-3.848	-0.037	0.037	3.85	0.037	90	12.025	12.016
335	90	14	16	Se	W	13.898	5.429	-1.087	1.47	-4.897	1.056	-1.428	75.008	12.682	11.785
336	90	14	16	Se	W	73.898	5.429	-1.087	1.47	-4.897	1.056	-1.428	75.008	12.682	11.785
337	90	14	16	Se	Se	13.898	5.429	-1.087	1.47	-4.897	1.056	-1.428	75.008	12.682	11.785
338	90	14	16	Se	Se	73.898	5.429	-1.087	1.47	-4.897	1.056	-1.428	75.008	12.682	11.785
339	93	15	16	Mo	W	46.102	5.429	2.029	-1.961	-4.897	-2.112	2.041	88.814	12.682	11.787
340	93	15	16	Mo	W	106.102	5.429	2.029	-1.961	-4.897	-2.112	2.041	88.814	12.682	11.787
341	93	15	16	Mo	Se	46.102	5.429	2.029	-1.961	-4.897	-2.112	2.041	88.814	12.682	11.787
342	93	15	16	Mo	Se	106.102	5.429	2.029	-1.961	-4.897	-2.112	2.041	88.814	12.682	11.787
343	93	16	15	Mo	Se	13.898	-4.964	-2.11	1.965	5.511	2.031	-1.891	88.814	12.683	11.787
344	93	16	15	Mo	Se	46.102	-4.964	2.11	1.965	5.511	-2.031	-1.891	88.814	12.683	11.787
345	93	16	15	Mo	Se	73.898	-4.964	-2.11	1.965	5.511	2.031	-1.891	88.814	12.683	11.787
346	93	15	16	Mo	W	103.898	-1.997	-1.886	5.47	2.08	1.7	-4.931	88.976	11.789	12.68
347	93	15	16	Mo	W	43.898	-1.997	-1.886	5.47	2.08	1.7	-4.931	88.976	11.789	12.68
348	93	15	16	Mo	Se	103.898	-1.997	-1.886	5.47	2.08	1.7	-4.931	88.976	11.789	12.68
349	93	15	16	Mo	Se	43.898	-1.997	-1.886	5.47	2.08	1.7	-4.931	88.976	11.789	12.68
350	93	16	15	Mo	Se	106.102	-4.964	2.11	1.965	5.511	-2.031	-1.891	88.814	12.683	11.787

351	93	15	16	Mo	W	13.898	5.429	-2.029	-1.961	-4.897	2.112	2.041	88.814	12.682	11.787
352	93	15	16	Mo	W	73.898	5.429	-2.029	-1.961	-4.897	2.112	2.041	88.814	12.682	11.787
353	93	15	16	Mo	Se	13.898	5.429	-2.029	-1.961	-4.897	2.112	2.041	88.814	12.682	11.787
354	93	15	16	Mo	Se	73.898	5.429	-2.029	-1.961	-4.897	2.112	2.041	88.814	12.682	11.787
355	93	16	15	Mo	W	13.898	-4.964	-2.11	1.965	5.511	2.031	-1.891	88.814	12.683	11.787
356	93	16	15	Mo	W	46.102	-4.964	2.11	1.965	5.511	-2.031	-1.891	88.814	12.683	11.787
357	93	16	15	Mo	W	73.898	-4.964	-2.11	1.965	5.511	2.031	-1.891	88.814	12.683	11.787
358	93	15	16	Mo	W	16.102	-1.997	1.886	5.47	2.08	-1.7	-4.931	88.976	11.789	12.68
359	93	15	16	Mo	W	76.102	-1.997	1.886	5.47	2.08	-1.7	-4.931	88.976	11.789	12.68
360	93	15	16	Mo	Se	16.102	-1.997	1.886	5.47	2.08	-1.7	-4.931	88.976	11.789	12.68
361	93	15	16	Mo	Se	76.102	-1.997	1.886	5.47	2.08	-1.7	-4.931	88.976	11.789	12.68
362	93	16	15	Mo	W	106.102	-4.964	2.11	1.965	5.511	-2.031	-1.891	88.814	12.683	11.787
363	93	16	15	Se	W	13.898	-4.964	-2.11	1.965	5.511	2.031	-1.891	88.814	12.683	11.787
364	93	16	15	Se	W	46.102	-4.964	2.11	1.965	5.511	-2.031	-1.891	88.814	12.683	11.787
365	93	16	15	Se	W	73.898	-4.964	-2.11	1.965	5.511	2.031	-1.891	88.814	12.683	11.787
366	93	16	15	Se	W	106.102	-4.964	2.11	1.965	5.511	-2.031	-1.891	88.814	12.683	11.787
367	93	16	15	Se	Se	13.898	-4.964	-2.11	1.965	5.511	2.031	-1.891	88.814	12.683	11.787
368	93	16	15	Se	Se	46.102	-4.964	2.11	1.965	5.511	-2.031	-1.891	88.814	12.683	11.787
369	93	16	15	Se	Se	73.898	-4.964	-2.11	1.965	5.511	2.031	-1.891	88.814	12.683	11.787
370	93	16	15	Se	Se	106.102	-4.964	2.11	1.965	5.511	-2.031	-1.891	88.814	12.683	11.787
371	93	15	16	Se	W	16.102	-1.997	1.886	5.47	2.08	-1.7	-4.931	88.976	11.789	12.68
372	93	15	16	Se	W	76.102	-1.997	1.886	5.47	2.08	-1.7	-4.931	88.976	11.789	12.68
373	93	15	16	Se	Se	16.102	-1.997	1.886	5.47	2.08	-1.7	-4.931	88.976	11.789	12.68
374	93	15	16	Se	Se	76.102	-1.997	1.886	5.47	2.08	-1.7	-4.931	88.976	11.789	12.68
375	93	15	16	Se	W	46.102	5.429	2.029	-1.961	-4.897	-2.112	2.041	88.814	12.682	11.787
376	93	15	16	Se	W	106.102	5.429	2.029	-1.961	-4.897	-2.112	2.041	88.814	12.682	11.787
377	93	15	16	Se	Se	46.102	5.429	2.029	-1.961	-4.897	-2.112	2.041	88.814	12.682	11.787
378	93	15	16	Se	Se	106.102	5.429	2.029	-1.961	-4.897	-2.112	2.041	88.814	12.682	11.787
379	93	15	16	Se	W	103.898	-1.997	-1.886	5.47	2.08	1.7	-4.931	88.976	11.789	12.68
380	93	15	16	Se	W	43.898	-1.997	-1.886	5.47	2.08	1.7	-4.931	88.976	11.789	12.68
381	93	15	16	Se	Se	103.898	-1.997	-1.886	5.47	2.08	1.7	-4.931	88.976	11.789	12.68
382	93	15	16	Se	Se	43.898	-1.997	-1.886	5.47	2.08	1.7	-4.931	88.976	11.789	12.68
383	93	15	16	Se	W	13.898	5.429	-2.029	-1.961	-4.897	2.112	2.041	88.814	12.682	11.787
384	93	15	16	Se	W	73.898	5.429	-2.029	-1.961	-4.897	2.112	2.041	88.814	12.682	11.787
385	93	15	16	Se	Se	13.898	5.429	-2.029	-1.961	-4.897	2.112	2.041	88.814	12.682	11.787
386	93	15	16	Se	Se	73.898	5.429	-2.029	-1.961	-4.897	2.112	2.041	88.814	12.682	11.787
387	96	16	16	Mo	Se	13.898	-4.964	-3.166	5.429	5.511	2.856	-4.897	73.815	12.683	12.687
388	96	16	16	Mo	Se	46.102	-4.964	3.166	5.429	5.511	-2.856	-4.897	73.815	12.683	12.687
389	96	16	16	Mo	Se	73.898	-4.964	-3.166	5.429	5.511	2.855	-4.897	73.815	12.683	12.687
390	96	16	16	Mo	W	73.898	-4.964	-3.166	5.429	5.511	2.855	-4.897	73.815	12.683	12.687
391	96	16	16	Mo	Se	106.102	-4.964	3.166	5.429	5.511	-2.856	-4.897	73.815	12.683	12.687
392	96	16	16	Mo	W	13.898	-4.964	-3.166	5.429	5.511	2.856	-4.897	73.815	12.683	12.687
393	96	16	16	Mo	W	46.102	-4.964	3.166	5.429	5.511	-2.856	-4.897	73.815	12.683	12.687
394	96	16	16	Mo	W	106.102	-4.964	3.166	5.429	5.511	-2.856	-4.897	73.815	12.683	12.687

395	96	16	16	Se	W	46.102	-4.964	3.166	5.429	5.511	-2.856	-4.897	73.815	12.683	12.687
396	96	16	16	Se	W	73.898	-4.964	-3.166	5.429	5.511	2.855	-4.897	73.815	12.683	12.687
397	96	16	16	Se	Se	13.898	-4.964	-3.166	5.429	5.511	2.856	-4.897	73.815	12.683	12.687
398	96	16	16	Se	Se	106.102	-4.964	3.166	5.429	5.511	-2.856	-4.897	73.815	12.683	12.687
399	99	16	17	Mo	Se	32.204	-0.037	-5.411	3.086	0.037	5.096	-2.907	71.612	12.025	13.928
400	99	17	16	Mo	Se	32.204	-0.037	-5.092	-2.976	0.037	5.415	3.164	71.613	12.025	13.929
401	99	16	17	Mo	Se	92.204	-0.037	-5.411	3.086	0.037	5.096	-2.907	71.612	12.025	13.928
402	99	17	16	Mo	Se	92.204	-0.037	-5.092	-2.976	0.037	5.415	3.164	71.613	12.025	13.929
403	99	17	16	Mo	W	36.587	-4.151	2.441	1.243	4.526	-2.382	-1.213	71.76	13.939	12.012
404	99	17	16	Mo	W	96.587	-4.151	2.441	1.243	4.526	-2.382	-1.213	71.76	13.939	12.012
405	99	17	16	Mo	Se	36.587	-4.151	2.441	1.243	4.526	-2.382	-1.213	71.76	13.939	12.012
406	99	17	16	Mo	Se	96.587	-4.151	2.441	1.243	4.526	-2.382	-1.213	71.76	13.939	12.012
407	99	16	17	Mo	W	13.174	-0.037	-1.803	3.086	0.037	1.699	-2.907	68.08	14.537	11.785
408	99	17	16	Mo	W	13.174	-0.037	-1.697	-2.976	0.037	1.805	3.164	68.081	14.537	11.785
409	99	16	17	Mo	W	73.174	-0.037	-1.803	3.086	0.037	1.699	-2.907	68.08	14.537	11.785
410	99	17	16	Mo	W	73.174	-0.037	-1.697	-2.976	0.037	1.805	3.164	68.081	14.537	11.785
411	99	16	17	Mo	Se	13.174	-0.037	-1.803	3.086	0.037	1.699	-2.907	68.08	14.537	11.785
412	99	17	16	Mo	Se	13.174	-0.037	-1.697	-2.976	0.037	1.805	3.164	68.081	14.537	11.785
413	99	16	17	Mo	Se	73.174	-0.037	-1.803	3.086	0.037	1.699	-2.907	68.08	14.537	11.785
414	99	17	16	Mo	Se	73.174	-0.037	-1.697	-2.976	0.037	1.805	3.164	68.081	14.537	11.785
415	99	16	17	Mo	W	16.102	2.003	3.398	1.003	-1.926	-3.331	-0.984	68.018	11.789	14.536
416	99	16	17	Mo	W	43.898	2.003	-3.398	1.003	-1.926	3.331	-0.984	68.018	11.789	14.536
417	99	16	17	Mo	W	76.102	2.003	3.398	1.003	-1.926	-3.331	-0.984	68.018	11.789	14.536
418	99	16	17	Mo	W	103.898	2.003	-3.398	1.003	-1.926	3.331	-0.984	68.018	11.789	14.536
419	99	16	17	Mo	Se	16.102	2.003	3.398	1.003	-1.926	-3.331	-0.984	68.018	11.789	14.536
420	99	16	17	Mo	Se	43.898	2.003	-3.398	1.003	-1.926	3.331	-0.984	68.018	11.789	14.536
421	99	16	17	Mo	Se	76.102	2.003	3.398	1.003	-1.926	-3.331	-0.984	68.018	11.789	14.536
422	99	16	17	Mo	Se	103.898	2.003	-3.398	1.003	-1.926	3.331	-0.984	68.018	11.789	14.536
423	99	17	16	Mo	W	23.413	-4.151	-2.441	1.243	4.526	2.382	-1.213	71.76	13.939	12.012
424	99	17	16	Mo	W	83.413	-4.151	-2.441	1.243	4.526	2.382	-1.213	71.76	13.939	12.012
425	99	17	16	Mo	Se	23.413	-4.151	-2.441	1.243	4.526	2.382	-1.213	71.76	13.939	12.012
426	99	17	16	Mo	Se	83.413	-4.151	-2.441	1.243	4.526	2.382	-1.213	71.76	13.939	12.012
427	99	17	16	Mo	W	106.826	-0.037	1.698	-2.976	0.037	-1.805	3.164	68.081	14.537	11.785
428	99	16	17	Mo	W	106.826	-0.037	1.804	3.086	0.037	-1.699	-2.907	68.08	14.537	11.785
429	99	17	16	Mo	W	46.826	-0.037	1.698	-2.976	0.037	-1.805	3.164	68.081	14.537	11.785
430	99	16	17	Mo	W	46.826	-0.037	1.804	3.086	0.037	-1.699	-2.907	68.08	14.537	11.785
431	99	17	16	Mo	Se	106.826	-0.037	1.698	-2.976	0.037	-1.805	3.164	68.081	14.537	11.785
432	99	16	17	Mo	Se	106.826	-0.037	1.804	3.086	0.037	-1.699	-2.907	68.08	14.537	11.785
433	99	17	16	Mo	Se	46.826	-0.037	1.698	-2.976	0.037	-1.805	3.164	68.081	14.537	11.785
434	99	16	17	Mo	Se	46.826	-0.037	1.804	3.086	0.037	-1.699	-2.907	68.08	14.537	11.785
435	99	17	16	Mo	W	103.898	-1.997	-3.328	-1.056	2.08	3.4	1.078	68.018	11.789	14.536
436	99	17	16	Mo	W	43.898	-1.997	-3.328	-1.056	2.08	3.4	1.078	68.018	11.789	14.536
437	99	17	16	Mo	Se	103.898	-1.997	-3.328	-1.056	2.08	3.4	1.078	68.018	11.789	14.536
438	99	17	16	Mo	Se	43.898	-1.997	-3.328	-1.056	2.08	3.4	1.078	68.018	11.789	14.536

439	99	17	16	Mo	W	87.796	-0.037	5.092	-2.976	0.037	-5.415	3.164	71.613	12.025	13.929
440	99	16	17	Mo	W	87.796	-0.037	5.411	3.086	0.037	-5.096	-2.907	71.612	12.025	13.928
441	99	17	16	Mo	W	27.796	-0.037	5.092	-2.976	0.037	-5.415	3.164	71.613	12.025	13.929
442	99	16	17	Mo	W	27.796	-0.037	5.411	3.086	0.037	-5.096	-2.907	71.612	12.025	13.928
443	99	17	16	Mo	Se	87.796	-0.037	5.092	-2.976	0.037	-5.415	3.164	71.613	12.025	13.929
444	99	16	17	Mo	Se	87.796	-0.037	5.411	3.086	0.037	-5.096	-2.907	71.612	12.025	13.928
445	99	17	16	Mo	Se	27.796	-0.037	5.092	-2.976	0.037	-5.415	3.164	71.613	12.025	13.929
446	99	16	17	Mo	Se	27.796	-0.037	5.411	3.086	0.037	-5.096	-2.907	71.612	12.025	13.928
447	99	16	17	Mo	W	23.413	4.446	-2.38	-1.285	-4.083	2.443	1.319	71.76	13.938	12.012
448	99	16	17	Mo	W	36.587	4.446	2.38	-1.285	-4.083	-2.443	1.319	71.76	13.938	12.012
449	99	16	17	Mo	W	83.413	4.446	-2.38	-1.285	-4.083	2.443	1.319	71.76	13.938	12.012
450	99	16	17	Mo	W	96.587	4.446	2.38	-1.285	-4.083	-2.443	1.319	71.76	13.938	12.012
451	99	16	17	Mo	Se	23.413	4.446	-2.38	-1.285	-4.083	2.443	1.319	71.76	13.938	12.012
452	99	16	17	Mo	Se	36.587	4.446	2.38	-1.285	-4.083	-2.443	1.319	71.76	13.938	12.012
453	99	16	17	Mo	Se	83.413	4.446	-2.38	-1.285	-4.083	2.443	1.319	71.76	13.938	12.012
454	99	16	17	Mo	Se	96.587	4.446	2.38	-1.285	-4.083	-2.443	1.319	71.76	13.938	12.012
455	99	17	16	Mo	W	16.102	-1.997	3.328	-1.056	2.08	-3.4	1.078	68.018	11.789	14.536
456	99	17	16	Mo	W	76.102	-1.997	3.328	-1.056	2.08	-3.4	1.078	68.018	11.789	14.536
457	99	17	16	Mo	Se	16.102	-1.997	3.328	-1.056	2.08	-3.4	1.078	68.018	11.789	14.536
458	99	17	16	Mo	Se	76.102	-1.997	3.328	-1.056	2.08	-3.4	1.078	68.018	11.789	14.536
459	99	16	17	Mo	W	32.204	-0.037	-5.411	3.086	0.037	5.096	-2.907	71.612	12.025	13.928
460	99	17	16	Mo	W	32.204	-0.037	-5.092	-2.976	0.037	5.415	3.164	71.613	12.025	13.929
461	99	16	17	Mo	W	92.204	-0.037	-5.411	3.086	0.037	5.096	-2.907	71.612	12.025	13.928
462	99	17	16	Mo	W	92.204	-0.037	-5.092	-2.976	0.037	5.415	3.164	71.613	12.025	13.929
463	99	16	17	Se	W	36.587	4.446	2.38	-1.285	-4.083	-2.443	1.319	71.76	13.938	12.012
464	99	16	17	Se	W	83.413	4.446	-2.38	-1.285	-4.083	2.443	1.319	71.76	13.938	12.012
465	99	16	17	Se	Se	23.413	4.446	-2.38	-1.285	-4.083	2.443	1.319	71.76	13.938	12.012
466	99	16	17	Se	Se	96.587	4.446	2.38	-1.285	-4.083	-2.443	1.319	71.76	13.938	12.012
467	99	17	16	Se	W	16.102	-1.997	3.328	-1.056	2.08	-3.4	1.078	68.018	11.789	14.536
468	99	17	16	Se	W	76.102	-1.997	3.328	-1.056	2.08	-3.4	1.078	68.018	11.789	14.536
469	99	17	16	Se	Se	16.102	-1.997	3.328	-1.056	2.08	-3.4	1.078	68.018	11.789	14.536
470	99	17	16	Se	Se	76.102	-1.997	3.328	-1.056	2.08	-3.4	1.078	68.018	11.789	14.536
471	99	16	17	Se	W	32.204	-0.037	-5.411	3.086	0.037	5.096	-2.907	71.612	12.025	13.928
472	99	17	16	Se	W	32.204	-0.037	-5.092	-2.976	0.037	5.415	3.164	71.613	12.025	13.929
473	99	16	17	Se	Se	92.204	-0.037	-5.411	3.086	0.037	5.096	-2.907	71.612	12.025	13.928
474	99	17	16	Se	Se	92.204	-0.037	-5.092	-2.976	0.037	5.415	3.164	71.613	12.025	13.929
475	99	17	16	Se	W	36.587	-4.151	2.441	1.243	4.526	-2.382	-1.213	71.76	13.939	12.012
476	99	17	16	Se	Se	96.587	-4.151	2.441	1.243	4.526	-2.382	-1.213	71.76	13.939	12.012
477	99	16	17	Se	W	13.174	-0.037	-1.803	3.086	0.037	1.699	-2.907	68.08	14.537	11.785
478	99	17	16	Se	W	13.174	-0.037	-1.697	-2.976	0.037	1.805	3.164	68.081	14.537	11.785
479	99	16	17	Se	W	73.174	-0.037	-1.803	3.086	0.037	1.699	-2.907	68.08	14.537	11.785
480	99	17	16	Se	W	73.174	-0.037	-1.697	-2.976	0.037	1.805	3.164	68.081	14.537	11.785
481	99	16	17	Se	Se	13.174	-0.037	-1.803	3.086	0.037	1.699	-2.907	68.08	14.537	11.785
482	99	17	16	Se	Se	13.174	-0.037	-1.697	-2.976	0.037	1.805	3.164	68.081	14.537	11.785

483	99	16	17	Se	Se	73.174	-0.037	-1.803	3.086	0.037	1.699	-2.907	68.08	14.537	11.785
484	99	17	16	Se	Se	73.174	-0.037	-1.697	-2.976	0.037	1.805	3.164	68.081	14.537	11.785
485	99	16	17	Se	W	16.102	2.003	3.398	1.003	-1.926	-3.331	-0.984	68.018	11.789	14.536
486	99	16	17	Se	W	43.898	2.003	-3.398	1.003	-1.926	3.331	-0.984	68.018	11.789	14.536
487	99	16	17	Se	W	76.102	2.003	3.398	1.003	-1.926	-3.331	-0.984	68.018	11.789	14.536
488	99	16	17	Se	W	103.898	2.003	-3.398	1.003	-1.926	3.331	-0.984	68.018	11.789	14.536
489	99	16	17	Se	Se	16.102	2.003	3.398	1.003	-1.926	-3.331	-0.984	68.018	11.789	14.536
490	99	16	17	Se	Se	43.898	2.003	-3.398	1.003	-1.926	3.331	-0.984	68.018	11.789	14.536
491	99	16	17	Se	Se	76.102	2.003	3.398	1.003	-1.926	-3.331	-0.984	68.018	11.789	14.536
492	99	16	17	Se	Se	103.898	2.003	-3.398	1.003	-1.926	3.331	-0.984	68.018	11.789	14.536
493	99	17	16	Se	W	83.413	-4.151	-2.441	1.243	4.526	2.382	-1.213	71.76	13.939	12.012
494	99	17	16	Se	Se	23.413	-4.151	-2.441	1.243	4.526	2.382	-1.213	71.76	13.939	12.012
495	99	17	16	Se	W	106.826	-0.037	1.698	-2.976	0.037	-1.805	3.164	68.081	14.537	11.785
496	99	16	17	Se	W	106.826	-0.037	1.804	3.086	0.037	-1.699	-2.907	68.08	14.537	11.785
497	99	17	16	Se	W	46.826	-0.037	1.698	-2.976	0.037	-1.805	3.164	68.081	14.537	11.785
498	99	16	17	Se	W	46.826	-0.037	1.804	3.086	0.037	-1.699	-2.907	68.08	14.537	11.785
499	99	17	16	Se	Se	106.826	-0.037	1.698	-2.976	0.037	-1.805	3.164	68.081	14.537	11.785
500	99	16	17	Se	Se	106.826	-0.037	1.804	3.086	0.037	-1.699	-2.907	68.08	14.537	11.785
501	99	17	16	Se	Se	46.826	-0.037	1.698	-2.976	0.037	-1.805	3.164	68.081	14.537	11.785
502	99	16	17	Se	Se	46.826	-0.037	1.804	3.086	0.037	-1.699	-2.907	68.08	14.537	11.785
503	99	17	16	Se	W	103.898	-1.997	-3.328	-1.056	2.08	3.4	1.078	68.018	11.789	14.536
504	99	17	16	Se	W	43.898	-1.997	-3.328	-1.056	2.08	3.4	1.078	68.018	11.789	14.536
505	99	17	16	Se	Se	103.898	-1.997	-3.328	-1.056	2.08	3.4	1.078	68.018	11.789	14.536
506	99	17	16	Se	Se	43.898	-1.997	-3.328	-1.056	2.08	3.4	1.078	68.018	11.789	14.536
507	99	17	16	Se	W	87.796	-0.037	5.092	-2.976	0.037	-5.415	3.164	71.613	12.025	13.929
508	99	16	17	Se	W	87.796	-0.037	5.411	3.086	0.037	-5.096	-2.907	71.612	12.025	13.928
509	99	17	16	Se	Se	27.796	-0.037	5.092	-2.976	0.037	-5.415	3.164	71.613	12.025	13.929
510	99	16	17	Se	Se	27.796	-0.037	5.411	3.086	0.037	-5.096	-2.907	71.612	12.025	13.928
511	105	19	16	Mo	W	36.587	-4.151	0	-4.151	4.526	0	4.526	60	13.939	13.939
512	105	19	16	Mo	W	96.587	-4.151	0	-4.151	4.526	0	4.526	60	13.939	13.939
513	105	19	16	Mo	Se	36.587	-4.151	0	-4.151	4.526	0	4.526	60	13.939	13.939
514	105	19	16	Mo	Se	96.587	-4.151	0	-4.151	4.526	0	4.526	60	13.939	13.939
515	105	19	16	Mo	W	23.413	-4.151	0	-4.151	4.526	0	4.526	60	13.939	13.939
516	105	19	16	Mo	W	83.413	-4.151	0	-4.151	4.526	0	4.526	60	13.939	13.939
517	105	19	16	Mo	Se	23.413	-4.151	0	-4.151	4.526	0	4.526	60	13.939	13.939
518	105	19	16	Mo	Se	83.413	-4.151	0	-4.151	4.526	0	4.526	60	13.939	13.939
519	105	16	19	Mo	W	23.413	4.446	0	4.446	-4.083	0	-4.083	60	13.938	13.938
520	105	16	19	Mo	W	36.587	4.446	0	4.446	-4.083	0	-4.083	60	13.938	13.938
521	105	16	19	Mo	W	83.413	4.446	0	4.446	-4.083	0	-4.083	60	13.938	13.938
522	105	16	19	Mo	W	96.587	4.446	0	4.446	-4.083	0	-4.083	60	13.938	13.938
523	105	16	19	Mo	Se	23.413	4.446	0	4.446	-4.083	0	-4.083	60	13.938	13.938
524	105	16	19	Mo	Se	36.587	4.446	0	4.446	-4.083	0	-4.083	60	13.938	13.938
525	105	16	19	Mo	Se	83.413	4.446	0	4.446	-4.083	0	-4.083	60	13.938	13.938
526	105	16	19	Mo	Se	96.587	4.446	0	4.446	-4.083	0	-4.083	60	13.938	13.938

527	105	16	19	Se	W	23.413	4.446	0	4.446	-4.083	0	-4.083	60	13.938	13.938
528	105	16	19	Se	W	96.587	4.446	0	4.446	-4.083	0	-4.083	60	13.938	13.938
529	105	16	19	Se	Se	36.587	4.446	0	4.446	-4.083	0	-4.083	60	13.938	13.938
530	105	16	19	Se	Se	83.413	4.446	0	4.446	-4.083	0	-4.083	60	13.938	13.938
531	105	19	16	Se	W	96.587	-4.151	0	-4.151	4.526	0	4.526	60	13.939	13.939
532	105	19	16	Se	Se	36.587	-4.151	0	-4.151	4.526	0	4.526	60	13.939	13.939
533	105	19	16	Se	W	23.413	-4.151	0	-4.151	4.526	0	4.526	60	13.939	13.939
534	105	19	16	Se	Se	83.413	-4.151	0	-4.151	4.526	0	4.526	60	13.939	13.939
535	114	18	20	Mo	W	46.102	5.429	-1.268	0.04	-4.897	1.267	-0.04	83.014	12.682	14.538
536	114	18	20	Mo	W	106.102	5.429	-1.268	0.04	-4.897	1.267	-0.04	83.014	12.682	14.538
537	114	20	18	Mo	Se	13.898	-4.964	1.266	-0.114	5.511	-1.269	0.114	83.014	12.683	14.538
538	114	19	19	Mo	W	13.174	-0.037	0	-0.037	0.037	0	0.037	60	14.537	14.537
539	114	20	18	Mo	Se	46.102	-4.964	-1.266	-0.114	5.511	1.269	0.114	83.014	12.683	14.538
540	114	19	19	Mo	W	73.174	-0.037	0	-0.037	0.037	0	0.037	60	14.537	14.537
541	114	19	19	Mo	Se	13.174	-0.037	0	-0.037	0.037	0	0.037	60	14.537	14.537
542	114	19	19	Mo	Se	73.174	-0.037	0	-0.037	0.037	0	0.037	60	14.537	14.537
543	114	19	19	Mo	W	106.826	-0.037	0	-0.037	0.037	0	0.037	60	14.537	14.537
544	114	19	19	Mo	W	46.826	-0.037	0	-0.037	0.037	0	0.037	60	14.537	14.537
545	114	19	19	Mo	Se	106.826	-0.037	0	-0.037	0.037	0	0.037	60	14.537	14.537
546	114	19	19	Mo	Se	46.826	-0.037	0	-0.037	0.037	0	0.037	60	14.537	14.537
547	114	20	18	Mo	Se	73.898	-4.964	1.266	-0.114	5.511	-1.269	0.114	83.014	12.683	14.538
548	114	20	18	Mo	Se	106.102	-4.964	-1.266	-0.114	5.511	1.269	0.114	83.014	12.683	14.538
549	114	18	20	Mo	W	13.898	5.429	1.268	0.04	-4.897	-1.267	-0.04	83.014	12.682	14.538
550	114	18	20	Mo	W	73.898	5.429	1.268	0.04	-4.897	-1.267	-0.04	83.014	12.682	14.538
551	114	20	18	Mo	W	13.898	-4.964	1.266	-0.114	5.511	-1.269	0.114	83.014	12.683	14.538
552	114	20	18	Mo	W	46.102	-4.964	-1.266	-0.114	5.511	1.269	0.114	83.014	12.683	14.538
553	114	20	18	Mo	W	73.898	-4.964	1.266	-0.114	5.511	-1.269	0.114	83.014	12.683	14.538
554	114	20	18	Mo	W	106.102	-4.964	-1.266	-0.114	5.511	1.269	0.114	83.014	12.683	14.538
555	114	18	20	Se	W	46.102	5.429	-1.268	0.04	-4.897	1.267	-0.04	83.014	12.682	14.538
556	114	18	20	Se	W	106.102	5.429	-1.268	0.04	-4.897	1.267	-0.04	83.014	12.682	14.538
557	114	19	19	Se	W	73.174	-0.037	0	-0.037	0.037	0	0.037	60	14.537	14.537
558	114	19	19	Se	Se	13.174	-0.037	0	-0.037	0.037	0	0.037	60	14.537	14.537
559	114	19	19	Se	W	46.826	-0.037	0	-0.037	0.037	0	0.037	60	14.537	14.537
560	114	19	19	Se	Se	106.826	-0.037	0	-0.037	0.037	0	0.037	60	14.537	14.537
561	114	18	20	Se	W	13.898	5.429	1.268	0.04	-4.897	-1.267	-0.04	83.014	12.682	14.538
562	114	18	20	Se	W	73.898	5.429	1.268	0.04	-4.897	-1.267	-0.04	83.014	12.682	14.538
563	120	20	20	Mo	W	23.413	4.446	0.952	-4.151	-4.083	-1.038	4.526	83.389	13.938	13.94
564	120	20	20	Mo	W	36.587	4.446	-0.952	-4.151	-4.083	1.038	4.526	83.389	13.938	13.94
565	120	20	20	Mo	W	83.413	4.446	0.952	-4.151	-4.083	-1.038	4.526	83.389	13.938	13.94
566	120	20	20	Mo	W	96.587	4.446	-0.952	-4.151	-4.083	1.038	4.526	83.389	13.938	13.94
567	120	20	20	Mo	Se	23.413	4.446	0.952	-4.151	-4.083	-1.038	4.526	83.389	13.938	13.94
568	120	20	20	Mo	Se	36.587	4.446	-0.952	-4.151	-4.083	1.038	4.526	83.389	13.938	13.94
569	120	20	20	Mo	Se	83.413	4.446	0.952	-4.151	-4.083	-1.038	4.526	83.389	13.938	13.94
570	120	20	20	Mo	Se	96.587	4.446	-0.952	-4.151	-4.083	1.038	4.526	83.389	13.938	13.94

571	120	20	20	Se	W	23.413	4.446	0.952	-4.151	-4.083	-1.038	4.526	83.389	13.938	13.94
572	120	20	20	Se	W	96.587	4.446	-0.952	-4.151	-4.083	1.038	4.526	83.389	13.938	13.94
573	120	20	20	Se	Se	36.587	4.446	-0.952	-4.151	-4.083	1.038	4.526	83.389	13.938	13.94
574	120	20	20	Se	Se	83.413	4.446	0.952	-4.151	-4.083	-1.038	4.526	83.389	13.938	13.94
