# Supplementary material for: Systematic DFT Modeling van der Waals Heterostructures from a Complete Configurational Basis Applied to γ-PC/WS2
Source: J Chem Theory Comput. 2024 Mar 6;20(6):2377–89. doi: 10.1021/acs.jctc.3c00932 (PMC10976641; doi:10.1021/acs.jctc.3c00932)
Supplement: Supplementary file 5 — ct3c00932_si_005.pdf [file ct3c00932_si_005.pdf]

BL number	Atoms	SnS_2 cells	TiO_2 cells	SnS_2 origin	TiO_2 origin	Twist-angle (°)	SnS_2 Strain 1 (%)	SnS_2 Strain 2 (%)	SnS_2 Strain 3 (%)	TiO_2 Strain 1 (%)	TiO_2 Strain 2 (%)	TiO_2 Strain 3 (%)	γ (°)	$|\mathbf{a}|$(Å)	$|\mathbf{b}|$(Å)
1	24	3	5	S	Ti	13.898	-2.165	-1.881	-2.2	2.263	1.968	2.301	71.223	5.25	8.16
2	24	3	5	S	Ti	46.102	-2.165	1.881	-2.2	2.263	-1.968	2.301	71.223	8.16	5.25
3	24	3	5	S	O	13.898	-2.165	-1.881	-2.2	2.263	1.968	2.301	71.223	5.25	8.16
4	24	3	5	S	O	46.102	-2.165	1.881	-2.2	2.263	-1.968	2.301	71.223	8.16	5.25
5	24	3	5	S	O	13.898	-2.165	-1.881	-2.2	2.263	1.968	2.301	71.223	5.25	8.16
6	24	3	5	S	O	46.102	-2.165	1.881	-2.2	2.263	-1.968	2.301	71.223	8.16	5.25
7	24	3	5	S	Ti	14.328	-2.906	-1.156	-1.447	3.086	1.19	1.49	71.256	5.25	8.159
8	24	3	5	S	O	14.328	-2.906	-1.156	-1.447	3.086	1.19	1.49	71.256	5.25	8.159
9	24	3	5	S	O	14.328	-2.906	-1.156	-1.447	3.086	1.19	1.49	71.256	5.25	8.159
10	24	3	5	S	Ti	45	-1.253	0	-3.094	1.286	0	3.298	71.239	5.249	8.161
11	24	3	5	S	O	45	-1.253	0	-3.094	1.286	0	3.298	71.239	5.249	8.161
12	24	3	5	S	O	45	-1.253	0	-3.094	1.286	0	3.298	71.239	5.249	8.161
13	24	3	5	S	Ti	15.672	-2.906	1.156	-1.447	3.086	-1.19	1.49	71.256	8.159	5.25
14	24	3	5	S	O	15.672	-2.906	1.156	-1.447	3.086	-1.19	1.49	71.256	8.159	5.25
15	24	3	5	S	O	15.672	-2.906	1.156	-1.447	3.086	-1.19	1.49	71.256	8.159	5.25
16	24	3	5	S	Ti	44.328	-2.906	-1.156	-1.447	3.086	1.19	1.49	71.256	5.25	8.159
17	24	3	5	S	O	44.328	-2.906	-1.156	-1.447	3.086	1.19	1.49	71.256	5.25	8.159
18	24	3	5	S	Ti	13.898	-2.165	-1.881	-2.2	2.263	1.968	2.301	71.223	5.25	8.16
19	24	3	5	S	Ti	46.102	-2.165	1.881	-2.2	2.263	-1.968	2.301	71.223	8.16	5.25
20	24	3	5	S	O	13.898	-2.165	-1.881	-2.2	2.263	1.968	2.301	71.223	5.25	8.16
21	24	3	5	S	O	46.102	-2.165	1.881	-2.2	2.263	-1.968	2.301	71.223	8.16	5.25
22	24	3	5	S	O	13.898	-2.165	-1.881	-2.2	2.263	1.968	2.301	71.223	5.25	8.16
23	24	3	5	S	O	46.102	-2.165	1.881	-2.2	2.263	-1.968	2.301	71.223	8.16	5.25
24	24	3	5	S	Ti	14.328	-2.906	-1.156	-1.447	3.086	1.19	1.49	71.256	5.25	8.159
25	24	3	5	S	O	14.328	-2.906	-1.156	-1.447	3.086	1.19	1.49	71.256	5.25	8.159
26	24	3	5	S	O	14.328	-2.906	-1.156	-1.447	3.086	1.19	1.49	71.256	5.25	8.159
27	24	3	5	S	Ti	45	-1.253	0	-3.094	1.286	0	3.298	71.239	5.249	8.161
28	24	3	5	S	O	45	-1.253	0	-3.094	1.286	0	3.298	71.239	5.249	8.161
29	24	3	5	S	O	45	-1.253	0	-3.094	1.286	0	3.298	71.239	5.249	8.161
30	24	3	5	S	Ti	15.672	-2.906	1.156	-1.447	3.086	-1.19	1.49	71.256	8.159	5.25
31	24	3	5	S	O	15.672	-2.906	1.156	-1.447	3.086	-1.19	1.49	71.256	8.159	5.25
32	24	3	5	S	O	15.672	-2.906	1.156	-1.447	3.086	-1.19	1.49	71.256	8.159	5.25
33	24	3	5	S	Ti	45.672	-2.906	1.156	-1.447	3.086	-1.19	1.49	71.256	8.159	5.25
34	24	3	5	S	O	45.672	-2.906	1.156	-1.447	3.086	-1.19	1.49	71.256	8.159	5.25
35	24	3	5	Sn	Ti	46.102	-2.165	1.881	-2.2	2.263	-1.968	2.301	71.223	8.16	5.25
36	24	3	5	Sn	O	13.898	-2.165	-1.881	-2.2	2.263	1.968	2.301	71.223	5.25	8.16
37	24	3	5	Sn	Ti	45	-1.253	0	-3.094	1.286	0	3.298	71.239	5.249	8.161
38	24	3	5	Sn	O	45	-1.253	0	-3.094	1.286	0	3.298	71.239	5.249	8.161
39	24	3	5	Sn	O	45	-1.253	0	-3.094	1.286	0	3.298	71.239	5.249	8.161
40	24	3	5	Sn	O	46.102	-2.165	1.881	-2.2	2.263	-1.968	2.301	71.223	8.16	5.25

41	24	3	5	Sn	O	13.898	-2.165	-1.881	-2.2	2.263	1.968	2.301	71.223	5.25	8.16
42	24	3	5	Sn	O	46.102	-2.165	1.881	-2.2	2.263	-1.968	2.301	71.223	8.16	5.25
43	24	3	5	Sn	Ti	15.672	-2.906	1.156	-1.447	3.086	-1.19	1.49	71.256	8.159	5.25
44	24	3	5	Sn	O	15.672	-2.906	1.156	-1.447	3.086	-1.19	1.49	71.256	8.159	5.25
45	24	3	5	Sn	O	15.672	-2.906	1.156	-1.447	3.086	-1.19	1.49	71.256	8.159	5.25
46	24	3	5	Sn	Ti	44.328	-2.906	-1.156	-1.447	3.086	1.19	1.49	71.256	5.25	8.159
47	24	3	5	Sn	O	44.328	-2.906	-1.156	-1.447	3.086	1.19	1.49	71.256	5.25	8.159
48	24	3	5	Sn	Ti	13.898	-2.165	-1.881	-2.2	2.263	1.968	2.301	71.223	5.25	8.16
49	24	3	5	Sn	Ti	75	-1.253	0	-3.094	1.286	0	3.298	71.239	5.249	8.161
50	24	3	5	Sn	O	75	-1.253	0	-3.094	1.286	0	3.298	71.239	5.249	8.161
51	24	3	5	Sn	Ti	45.672	-2.906	1.156	-1.447	3.086	-1.19	1.49	71.256	8.159	5.25
52	24	3	5	Sn	O	45.672	-2.906	1.156	-1.447	3.086	-1.19	1.49	71.256	8.159	5.25
53	24	3	5	Sn	Ti	14.328	-2.906	-1.156	-1.447	3.086	1.19	1.49	71.256	5.25	8.159
54	24	3	5	Sn	O	14.328	-2.906	-1.156	-1.447	3.086	1.19	1.49	71.256	5.25	8.159
55	24	3	5	Sn	O	14.328	-2.906	-1.156	-1.447	3.086	1.19	1.49	71.256	5.25	8.159
56	33	4	7	S	Ti	19.107	2.652	0	-4.402	-2.519	0	4.827	61.769	7.717	8.157
57	33	4	7	S	Ti	40.893	2.652	0	-4.402	-2.519	0	4.827	61.769	7.717	8.157
58	33	4	7	S	O	19.107	2.652	0	-4.402	-2.519	0	4.827	61.769	7.717	8.157
59	33	4	7	S	O	40.893	2.652	0	-4.402	-2.519	0	4.827	61.769	7.717	8.157
60	33	4	7	S	O	19.107	2.652	0	-4.402	-2.519	0	4.827	61.769	7.717	8.157
61	33	4	7	S	O	40.893	2.652	0	-4.402	-2.519	0	4.827	61.769	7.717	8.157
62	33	4	7	S	Ti	49.107	2.652	0	-4.402	-2.519	0	4.827	61.769	7.717	8.157
63	33	4	7	S	Ti	10.893	2.652	0	-4.402	-2.519	0	4.827	61.769	7.717	8.157
64	33	4	7	S	O	49.107	2.652	0	-4.402	-2.519	0	4.827	61.769	7.717	8.157
65	33	4	7	S	O	10.893	2.652	0	-4.402	-2.519	0	4.827	61.769	7.717	8.157
66	33	4	7	S	Ti	19.107	2.652	0	-4.402	-2.519	0	4.827	61.769	7.717	8.157
67	33	4	7	S	Ti	19.107	2.652	0	-4.402	-2.519	0	4.827	61.769	7.717	8.157
68	33	4	7	S	Ti	40.893	2.652	0	-4.402	-2.519	0	4.827	61.769	7.717	8.157
69	33	4	7	S	Ti	40.893	2.652	0	-4.402	-2.519	0	4.827	61.769	7.717	8.157
70	33	4	7	S	O	19.107	2.652	0	-4.402	-2.519	0	4.827	61.769	7.717	8.157
71	33	4	7	S	O	19.107	2.652	0	-4.402	-2.519	0	4.827	61.769	7.717	8.157
72	33	4	7	S	O	40.893	2.652	0	-4.402	-2.519	0	4.827	61.769	7.717	8.157
73	33	4	7	S	O	40.893	2.652	0	-4.402	-2.519	0	4.827	61.769	7.717	8.157
74	33	4	7	S	O	19.107	2.652	0	-4.402	-2.519	0	4.827	61.769	7.717	8.157
75	33	4	7	S	O	19.107	2.652	0	-4.402	-2.519	0	4.827	61.769	7.717	8.157
76	33	4	7	S	O	40.893	2.652	0	-4.402	-2.519	0	4.827	61.769	7.717	8.157
77	33	4	7	S	O	40.893	2.652	0	-4.402	-2.519	0	4.827	61.769	7.717	8.157
78	33	4	7	S	Ti	49.107	2.652	0	-4.402	-2.519	0	4.827	61.769	7.717	8.157
79	33	4	7	S	Ti	10.893	2.652	0	-4.402	-2.519	0	4.827	61.769	7.717	8.157
80	33	4	7	S	O	49.107	2.652	0	-4.402	-2.519	0	4.827	61.769	7.717	8.157
81	33	4	7	S	O	10.893	2.652	0	-4.402	-2.519	0	4.827	61.769	7.717	8.157
82	33	4	7	Sn	Ti	19.107	2.652	0	-4.402	-2.519	0	4.827	61.769	7.717	8.157

83	33	4	7	Sn	Ti	40.893	2.652	0	-4.402	-2.519	0	4.827	61.769	7.717	8.157
84	33	4	7	Sn	O	19.107	2.652	0	-4.402	-2.519	0	4.827	61.769	7.717	8.157
85	33	4	7	Sn	Ti	49.107	2.652	0	-4.402	-2.519	0	4.827	61.769	7.717	8.157
86	33	4	7	Sn	Ti	10.893	2.652	0	-4.402	-2.519	0	4.827	61.769	7.717	8.157
87	33	4	7	Sn	O	49.107	2.652	0	-4.402	-2.519	0	4.827	61.769	7.717	8.157
88	33	4	7	Sn	O	10.893	2.652	0	-4.402	-2.519	0	4.827	61.769	7.717	8.157
89	33	4	7	Sn	O	40.893	2.652	0	-4.402	-2.519	0	4.827	61.769	7.717	8.157
90	33	4	7	Sn	O	19.107	2.652	0	-4.402	-2.519	0	4.827	61.769	7.717	8.157
91	33	4	7	Sn	O	40.893	2.652	0	-4.402	-2.519	0	4.827	61.769	7.717	8.157
92	42	5	9	S	Ti	15.417	0.587	0.719	-1.184	-0.58	-0.736	1.213	78.893	13.633	5.249
93	42	5	9	S	O	15.417	0.587	0.719	-1.184	-0.58	-0.736	1.213	78.893	13.633	5.249
94	42	5	9	S	O	15.417	0.587	0.719	-1.184	-0.58	-0.736	1.213	78.893	13.633	5.249
95	42	5	9	S	Ti	45	-1.253	0	0.659	1.286	0	-0.65	78.9	5.249	13.633
96	42	5	9	S	O	45	-1.253	0	0.659	1.286	0	-0.65	78.9	5.249	13.633
97	42	5	9	S	O	45	-1.253	0	0.659	1.286	0	-0.65	78.9	5.249	13.633
98	42	5	9	S	Ti	14.583	0.587	-0.719	-1.184	-0.58	0.736	1.213	78.905	13.632	5.249
99	42	5	9	S	O	14.583	0.587	-0.719	-1.184	-0.58	0.736	1.213	78.905	13.632	5.249
100	42	5	9	S	O	14.583	0.587	-0.719	-1.184	-0.58	0.736	1.213	78.905	13.632	5.249
101	42	5	9	S	Ti	45.417	0.587	0.719	-1.184	-0.58	-0.736	1.213	78.893	13.633	5.249
102	42	5	9	S	O	45.417	0.587	0.719	-1.184	-0.58	-0.736	1.213	78.893	13.633	5.249
103	42	5	9	S	Ti	75	-1.253	0	0.659	1.286	0	-0.65	78.9	5.249	13.633
104	42	5	9	S	O	75	-1.253	0	0.659	1.286	0	-0.65	78.9	5.249	13.633
105	42	5	9	S	O	44.583	0.587	-0.719	-1.184	-0.58	0.736	1.213	78.905	13.632	5.249
106	42	5	9	S	Ti	15.417	0.587	0.719	-1.184	-0.58	-0.736	1.213	78.893	13.633	5.249
107	42	5	9	S	O	15.417	0.587	0.719	-1.184	-0.58	-0.736	1.213	78.893	13.633	5.249
108	42	5	9	S	O	15.417	0.587	0.719	-1.184	-0.58	-0.736	1.213	78.893	13.633	5.249
109	42	5	9	S	Ti	45	-1.253	0	0.659	1.286	0	-0.65	78.9	5.249	13.633
110	42	5	9	S	O	45	-1.253	0	0.659	1.286	0	-0.65	78.9	5.249	13.633
111	42	5	9	S	O	45	-1.253	0	0.659	1.286	0	-0.65	78.9	5.249	13.633
112	42	5	9	S	Ti	14.583	0.587	-0.719	-1.184	-0.58	0.736	1.213	78.905	13.632	5.249
113	42	5	9	S	O	14.583	0.587	-0.719	-1.184	-0.58	0.736	1.213	78.905	13.632	5.249
114	42	5	9	S	O	14.583	0.587	-0.719	-1.184	-0.58	0.736	1.213	78.905	13.632	5.249
115	42	5	9	S	Ti	45.417	0.587	0.719	-1.184	-0.58	-0.736	1.213	78.893	13.633	5.249
116	42	5	9	S	O	45.417	0.587	0.719	-1.184	-0.58	-0.736	1.213	78.893	13.633	5.249
117	42	5	9	S	Ti	75	-1.253	0	0.659	1.286	0	-0.65	78.9	5.249	13.633
118	42	5	9	S	O	75	-1.253	0	0.659	1.286	0	-0.65	78.9	5.249	13.633
119	42	5	9	S	Ti	44.583	0.587	-0.719	-1.184	-0.58	0.736	1.213	78.905	13.632	5.249
120	42	5	9	S	O	44.583	0.587	-0.719	-1.184	-0.58	0.736	1.213	78.905	13.632	5.249
121	42	5	9	Sn	Ti	15.417	0.587	0.719	-1.184	-0.58	-0.736	1.213	78.893	13.633	5.249
122	42	5	9	Sn	O	15.417	0.587	0.719	-1.184	-0.58	-0.736	1.213	78.893	13.633	5.249
123	42	5	9	Sn	O	15.417	0.587	0.719	-1.184	-0.58	-0.736	1.213	78.893	13.633	5.249
124	42	5	9	Sn	Ti	45	-1.253	0	0.659	1.286	0	-0.65	78.9	5.249	13.633

125	42	5	9	Sn	O	45	-1.253	0	0.659	1.286	0	-0.65	78.9	5.249	13.633
126	42	5	9	Sn	O	45	-1.253	0	0.659	1.286	0	-0.65	78.9	5.249	13.633
127	42	5	9	Sn	Ti	14.583	0.587	-0.719	-1.184	-0.58	0.736	1.213	78.905	13.632	5.249
128	42	5	9	Sn	O	14.583	0.587	-0.719	-1.184	-0.58	0.736	1.213	78.905	13.632	5.249
129	42	5	9	Sn	O	14.583	0.587	-0.719	-1.184	-0.58	0.736	1.213	78.905	13.632	5.249
130	42	5	9	Sn	Ti	45.417	0.587	0.719	-1.184	-0.58	-0.736	1.213	78.893	13.633	5.249
131	42	5	9	Sn	O	45.417	0.587	0.719	-1.184	-0.58	-0.736	1.213	78.893	13.633	5.249
132	42	5	9	Sn	Ti	75	-1.253	0	0.659	1.286	0	-0.65	78.9	5.249	13.633
133	42	5	9	Sn	O	75	-1.253	0	0.659	1.286	0	-0.65	78.9	5.249	13.633
134	42	5	9	Sn	Ti	44.583	0.587	-0.719	-1.184	-0.58	0.736	1.213	78.905	13.632	5.249
135	42	5	9	Sn	O	44.583	0.587	-0.719	-1.184	-0.58	0.736	1.213	78.905	13.632	5.249
136	48	6	10	S	Ti	28.898	0.737	3.875	-4.934	-0.726	-4.299	5.474	68.497	10.71	8.148
137	48	6	10	S	Ti	31.102	0.737	-3.875	-4.934	-0.726	4.299	5.474	68.497	10.71	8.148
138	48	6	10	S	Ti	1.102	0.737	-3.875	-4.934	-0.726	4.299	5.474	68.497	10.71	8.148
139	48	6	10	S	Ti	58.898	0.737	3.875	-4.934	-0.726	-4.299	5.474	68.497	10.71	8.148
140	48	6	10	Sn	Ti	1.102	0.737	-3.875	-4.934	-0.726	4.299	5.474	68.497	10.71	8.148
141	48	6	10	Sn	Ti	28.898	0.737	3.875	-4.934	-0.726	-4.299	5.474	68.497	10.71	8.148
142	48	6	10	Sn	Ti	31.102	0.737	-3.875	-4.934	-0.726	4.299	5.474	68.497	10.71	8.148
143	48	6	10	Sn	Ti	58.898	0.737	3.875	-4.934	-0.726	-4.299	5.474	68.497	10.71	8.148
144	51	6	11	S	Ti	13.898	-2.165	2.822	2.58	2.263	-2.684	-2.453	88.424	11.033	7.715
145	51	6	11	S	Ti	46.102	-2.165	-2.822	2.58	2.263	2.684	-2.453	88.424	11.033	7.715
146	51	6	11	S	O	13.898	-2.165	2.822	2.58	2.263	-2.684	-2.453	88.424	11.033	7.715
147	51	6	11	S	O	46.102	-2.165	-2.822	2.58	2.263	2.684	-2.453	88.424	11.033	7.715
148	51	6	11	S	O	13.898	-2.165	2.822	2.58	2.263	-2.684	-2.453	88.424	11.033	7.715
149	51	6	11	S	O	46.102	-2.165	-2.822	2.58	2.263	2.684	-2.453	88.424	11.033	7.715
150	51	6	11	S	Ti	19.107	2.652	2.444	-2.23	-2.519	-2.558	2.335	88.568	7.717	11.029
151	51	6	11	S	Ti	40.893	2.652	-2.444	-2.23	-2.519	2.558	2.335	88.568	7.717	11.029
152	51	6	11	S	O	19.107	2.652	2.444	-2.23	-2.519	-2.558	2.335	88.568	7.717	11.029
153	51	6	11	S	O	40.893	2.652	-2.444	-2.23	-2.519	2.558	2.335	88.568	7.717	11.029
154	51	6	11	S	O	19.107	2.652	2.444	-2.23	-2.519	-2.558	2.335	88.568	7.717	11.029
155	51	6	11	S	O	40.893	2.652	-2.444	-2.23	-2.519	2.558	2.335	88.568	7.717	11.029
156	51	6	11	S	Ti	49.723	-1.882	3.658	2.271	1.956	-3.499	-2.173	88.56	11.024	7.72
157	51	6	11	S	O	49.723	-1.882	3.658	2.271	1.956	-3.499	-2.173	88.56	11.024	7.72
158	51	6	11	S	O	49.723	-1.882	3.658	2.271	1.956	-3.499	-2.173	88.56	11.024	7.72
159	51	6	11	S	Ti	15.417	0.587	5.389	-0.28	-0.58	-5.419	0.281	88.429	11.033	7.71
160	51	6	11	S	O	15.417	0.587	5.389	-0.28	-0.58	-5.419	0.281	88.429	11.033	7.71
161	51	6	11	S	O	15.417	0.587	5.389	-0.28	-0.58	-5.419	0.281	88.429	11.033	7.71
162	51	6	11	S	Ti	40.277	-1.882	-3.658	2.271	1.956	3.499	-2.173	88.56	7.72	11.024
163	51	6	11	S	O	40.277	-1.882	-3.658	2.271	1.956	3.499	-2.173	88.56	7.72	11.024
164	51	6	11	S	O	40.277	-1.882	-3.658	2.271	1.956	3.499	-2.173	88.56	7.72	11.024
165	51	6	11	S	Ti	14.583	0.587	-5.389	-0.28	-0.58	5.419	0.281	88.429	7.71	11.033
166	51	6	11	S	O	14.583	0.587	-5.389	-0.28	-0.58	5.419	0.281	88.429	7.71	11.033

167	51	6	11	S	O	14.583	0.587	-5.389	-0.28	-0.58	5.419	0.281	88.429	7.71	11.033
168	51	6	11	S	Ti	43.898	-2.165	2.822	2.58	2.263	-2.684	-2.453	88.424	11.033	7.715
169	51	6	11	S	Ti	16.102	-2.165	-2.822	2.58	2.263	2.684	-2.453	88.424	11.033	7.715
170	51	6	11	S	O	43.898	-2.165	2.822	2.58	2.263	-2.684	-2.453	88.424	11.033	7.715
171	51	6	11	S	O	16.102	-2.165	-2.822	2.58	2.263	2.684	-2.453	88.424	11.033	7.715
172	51	6	11	S	Ti	49.107	2.652	2.444	-2.23	-2.519	-2.558	2.335	88.568	7.717	11.029
173	51	6	11	S	Ti	10.893	2.652	-2.444	-2.23	-2.519	2.558	2.335	88.568	7.717	11.029
174	51	6	11	S	O	49.107	2.652	2.444	-2.23	-2.519	-2.558	2.335	88.568	7.717	11.029
175	51	6	11	S	O	10.893	2.652	-2.444	-2.23	-2.519	2.558	2.335	88.568	7.717	11.029
176	51	6	11	S	Ti	19.723	-1.882	3.658	2.271	1.956	-3.499	-2.173	88.56	11.024	7.72
177	51	6	11	S	O	19.723	-1.882	3.658	2.271	1.956	-3.499	-2.173	88.56	11.024	7.72
178	51	6	11	S	Ti	45.417	0.587	5.389	-0.28	-0.58	-5.419	0.281	88.429	11.033	7.71
179	51	6	11	S	O	45.417	0.587	5.389	-0.28	-0.58	-5.419	0.281	88.429	11.033	7.71
180	51	6	11	S	Ti	10.277	-1.882	-3.658	2.271	1.956	3.499	-2.173	88.56	7.72	11.024
181	51	6	11	S	Ti	44.583	0.587	-5.389	-0.28	-0.58	5.419	0.281	88.429	7.71	11.033
182	51	6	11	S	O	44.583	0.587	-5.389	-0.28	-0.58	5.419	0.281	88.429	7.71	11.033
183	51	6	11	S	Ti	13.898	-2.165	2.822	2.58	2.263	-2.684	-2.453	88.424	11.033	7.715
184	51	6	11	S	Ti	46.102	-2.165	-2.822	2.58	2.263	2.684	-2.453	88.424	11.033	7.715
185	51	6	11	S	O	13.898	-2.165	2.822	2.58	2.263	-2.684	-2.453	88.424	11.033	7.715
186	51	6	11	S	O	46.102	-2.165	-2.822	2.58	2.263	2.684	-2.453	88.424	11.033	7.715
187	51	6	11	S	O	13.898	-2.165	2.822	2.58	2.263	-2.684	-2.453	88.424	11.033	7.715
188	51	6	11	S	O	46.102	-2.165	-2.822	2.58	2.263	2.684	-2.453	88.424	11.033	7.715
189	51	6	11	S	Ti	19.107	2.652	2.444	-2.23	-2.519	-2.558	2.335	88.568	7.717	11.029
190	51	6	11	S	Ti	40.893	2.652	-2.444	-2.23	-2.519	2.558	2.335	88.568	7.717	11.029
191	51	6	11	S	O	19.107	2.652	2.444	-2.23	-2.519	-2.558	2.335	88.568	7.717	11.029
192	51	6	11	S	O	40.893	2.652	-2.444	-2.23	-2.519	2.558	2.335	88.568	7.717	11.029
193	51	6	11	S	O	19.107	2.652	2.444	-2.23	-2.519	-2.558	2.335	88.568	7.717	11.029
194	51	6	11	S	O	40.893	2.652	-2.444	-2.23	-2.519	2.558	2.335	88.568	7.717	11.029
195	51	6	11	S	Ti	49.723	-1.882	3.658	2.271	1.956	-3.499	-2.173	88.56	11.024	7.72
196	51	6	11	S	O	49.723	-1.882	3.658	2.271	1.956	-3.499	-2.173	88.56	11.024	7.72
197	51	6	11	S	O	49.723	-1.882	3.658	2.271	1.956	-3.499	-2.173	88.56	11.024	7.72
198	51	6	11	S	Ti	15.417	0.587	5.389	-0.28	-0.58	-5.419	0.281	88.429	11.033	7.71
199	51	6	11	S	O	15.417	0.587	5.389	-0.28	-0.58	-5.419	0.281	88.429	11.033	7.71
200	51	6	11	S	O	15.417	0.587	5.389	-0.28	-0.58	-5.419	0.281	88.429	11.033	7.71
201	51	6	11	S	Ti	40.277	-1.882	-3.658	2.271	1.956	3.499	-2.173	88.56	7.72	11.024
202	51	6	11	S	O	40.277	-1.882	-3.658	2.271	1.956	3.499	-2.173	88.56	7.72	11.024
203	51	6	11	S	O	40.277	-1.882	-3.658	2.271	1.956	3.499	-2.173	88.56	7.72	11.024
204	51	6	11	S	Ti	14.583	0.587	-5.389	-0.28	-0.58	5.419	0.281	88.429	7.71	11.033
205	51	6	11	S	O	14.583	0.587	-5.389	-0.28	-0.58	5.419	0.281	88.429	7.71	11.033
206	51	6	11	S	O	14.583	0.587	-5.389	-0.28	-0.58	5.419	0.281	88.429	7.71	11.033
207	51	6	11	S	Ti	43.898	-2.165	2.822	2.58	2.263	-2.684	-2.453	88.424	11.033	7.715
208	51	6	11	S	Ti	16.102	-2.165	-2.822	2.58	2.263	2.684	-2.453	88.424	11.033	7.715

209	51	6	11	S	O	43.898	-2.165	2.822	2.58	2.263	-2.684	-2.453	88.424	11.033	7.715
210	51	6	11	S	O	16.102	-2.165	-2.822	2.58	2.263	2.684	-2.453	88.424	11.033	7.715
211	51	6	11	S	Ti	49.107	2.652	2.444	-2.23	-2.519	-2.558	2.335	88.568	7.717	11.029
212	51	6	11	S	Ti	10.893	2.652	-2.444	-2.23	-2.519	2.558	2.335	88.568	7.717	11.029
213	51	6	11	S	O	49.107	2.652	2.444	-2.23	-2.519	-2.558	2.335	88.568	7.717	11.029
214	51	6	11	S	O	10.893	2.652	-2.444	-2.23	-2.519	2.558	2.335	88.568	7.717	11.029
215	51	6	11	S	Ti	19.723	-1.882	3.658	2.271	1.956	-3.499	-2.173	88.56	11.024	7.72
216	51	6	11	S	O	19.723	-1.882	3.658	2.271	1.956	-3.499	-2.173	88.56	11.024	7.72
217	51	6	11	S	Ti	45.417	0.587	5.389	-0.28	-0.58	-5.419	0.281	88.429	11.033	7.71
218	51	6	11	S	O	45.417	0.587	5.389	-0.28	-0.58	-5.419	0.281	88.429	11.033	7.71
219	51	6	11	S	Ti	10.277	-1.882	-3.658	2.271	1.956	3.499	-2.173	88.56	7.72	11.024
220	51	6	11	S	Ti	44.583	0.587	-5.389	-0.28	-0.58	5.419	0.281	88.429	7.71	11.033
221	51	6	11	S	O	44.583	0.587	-5.389	-0.28	-0.58	5.419	0.281	88.429	7.71	11.033
222	51	6	11	Sn	Ti	49.723	-1.882	3.658	2.271	1.956	-3.499	-2.173	88.56	11.024	7.72
223	51	6	11	Sn	O	49.723	-1.882	3.658	2.271	1.956	-3.499	-2.173	88.56	11.024	7.72
224	51	6	11	Sn	O	49.723	-1.882	3.658	2.271	1.956	-3.499	-2.173	88.56	11.024	7.72
225	51	6	11	Sn	Ti	15.417	0.587	5.389	-0.28	-0.58	-5.419	0.281	88.429	11.033	7.71
226	51	6	11	Sn	O	15.417	0.587	5.389	-0.28	-0.58	-5.419	0.281	88.429	11.033	7.71
227	51	6	11	Sn	O	13.898	-2.165	2.822	2.58	2.263	-2.684	-2.453	88.424	11.033	7.715
228	51	6	11	Sn	O	15.417	0.587	5.389	-0.28	-0.58	-5.419	0.281	88.429	11.033	7.71
229	51	6	11	Sn	O	46.102	-2.165	-2.822	2.58	2.263	2.684	-2.453	88.424	11.033	7.715
230	51	6	11	Sn	Ti	40.277	-1.882	-3.658	2.271	1.956	3.499	-2.173	88.56	7.72	11.024
231	51	6	11	Sn	O	40.277	-1.882	-3.658	2.271	1.956	3.499	-2.173	88.56	7.72	11.024
232	51	6	11	Sn	O	40.277	-1.882	-3.658	2.271	1.956	3.499	-2.173	88.56	7.72	11.024
233	51	6	11	Sn	O	13.898	-2.165	2.822	2.58	2.263	-2.684	-2.453	88.424	11.033	7.715
234	51	6	11	Sn	Ti	14.583	0.587	-5.389	-0.28	-0.58	5.419	0.281	88.429	7.71	11.033
235	51	6	11	Sn	O	14.583	0.587	-5.389	-0.28	-0.58	5.419	0.281	88.429	7.71	11.033
236	51	6	11	Sn	O	14.583	0.587	-5.389	-0.28	-0.58	5.419	0.281	88.429	7.71	11.033
237	51	6	11	Sn	O	46.102	-2.165	-2.822	2.58	2.263	2.684	-2.453	88.424	11.033	7.715
238	51	6	11	Sn	Ti	19.107	2.652	2.444	-2.23	-2.519	-2.558	2.335	88.568	7.717	11.029
239	51	6	11	Sn	Ti	40.893	2.652	-2.444	-2.23	-2.519	2.558	2.335	88.568	7.717	11.029
240	51	6	11	Sn	O	19.107	2.652	2.444	-2.23	-2.519	-2.558	2.335	88.568	7.717	11.029
241	51	6	11	Sn	Ti	43.898	-2.165	2.822	2.58	2.263	-2.684	-2.453	88.424	11.033	7.715
242	51	6	11	Sn	Ti	16.102	-2.165	-2.822	2.58	2.263	2.684	-2.453	88.424	11.033	7.715
243	51	6	11	Sn	O	43.898	-2.165	2.822	2.58	2.263	-2.684	-2.453	88.424	11.033	7.715
244	51	6	11	Sn	O	16.102	-2.165	-2.822	2.58	2.263	2.684	-2.453	88.424	11.033	7.715
245	51	6	11	Sn	O	40.893	2.652	-2.444	-2.23	-2.519	2.558	2.335	88.568	7.717	11.029
246	51	6	11	Sn	Ti	49.107	2.652	2.444	-2.23	-2.519	-2.558	2.335	88.568	7.717	11.029
247	51	6	11	Sn	Ti	10.893	2.652	-2.444	-2.23	-2.519	2.558	2.335	88.568	7.717	11.029
248	51	6	11	Sn	O	49.107	2.652	2.444	-2.23	-2.519	-2.558	2.335	88.568	7.717	11.029
249	51	6	11	Sn	O	10.893	2.652	-2.444	-2.23	-2.519	2.558	2.335	88.568	7.717	11.029
250	51	6	11	Sn	O	19.107	2.652	2.444	-2.23	-2.519	-2.558	2.335	88.568	7.717	11.029

251	51	6	11	Sn	O	40.893	2.652	-2.444	-2.23	-2.519	2.558	2.335	88.568	7.717	11.029
252	51	6	11	Sn	Ti	19.723	-1.882	3.658	2.271	1.956	-3.499	-2.173	88.56	11.024	7.72
253	51	6	11	Sn	O	19.723	-1.882	3.658	2.271	1.956	-3.499	-2.173	88.56	11.024	7.72
254	51	6	11	Sn	Ti	45.417	0.587	5.389	-0.28	-0.58	-5.419	0.281	88.429	11.033	7.71
255	51	6	11	Sn	O	45.417	0.587	5.389	-0.28	-0.58	-5.419	0.281	88.429	11.033	7.71
256	51	6	11	Sn	Ti	10.277	-1.882	-3.658	2.271	1.956	3.499	-2.173	88.56	7.72	11.024
257	51	6	11	Sn	Ti	44.583	0.587	-5.389	-0.28	-0.58	5.419	0.281	88.429	7.71	11.033
258	51	6	11	Sn	O	44.583	0.587	-5.389	-0.28	-0.58	5.419	0.281	88.429	7.71	11.033
259	51	6	11	Sn	Ti	13.898	-2.165	2.822	2.58	2.263	-2.684	-2.453	88.424	11.033	7.715
260	51	6	11	Sn	Ti	46.102	-2.165	-2.822	2.58	2.263	2.684	-2.453	88.424	11.033	7.715
261	54	7	11	S	Ti	32.333	-4.619	-4.193	-2.494	5.09	4.413	2.625	84.474	11.338	8.151
262	54	7	11	S	O	32.333	-4.619	-4.193	-2.494	5.09	4.413	2.625	84.474	11.338	8.151
263	54	6	12	S	Ti	0	3.069	-3.221	1.704	-2.891	3.115	-1.647	72.77	8.684	10.715
264	54	6	12	S	O	0	3.069	-3.221	1.704	-2.891	3.115	-1.647	72.77	8.684	10.715
265	54	6	12	S	O	0	3.069	-3.221	1.704	-2.891	3.115	-1.647	72.77	8.684	10.715
266	54	7	11	S	Ti	27.667	-4.619	4.193	-2.494	5.09	-4.413	2.625	84.474	11.338	8.151
267	54	7	11	S	O	27.667	-4.619	4.193	-2.494	5.09	-4.413	2.625	84.474	11.338	8.151
268	54	7	11	S	O	27.667	-4.619	4.193	-2.494	5.09	-4.413	2.625	84.474	11.338	8.151
269	54	7	11	S	Ti	52.542	-2.906	-4.458	-4.222	3.086	4.87	4.611	84.388	8.161	11.325
270	54	7	11	S	O	52.542	-2.906	-4.458	-4.222	3.086	4.87	4.611	84.388	8.161	11.325
271	54	7	11	S	O	52.542	-2.906	-4.458	-4.222	3.086	4.87	4.611	84.388	8.161	11.325
272	54	6	12	S	Ti	1.102	0.737	-1.291	4.079	-0.726	1.194	-3.772	72.765	10.71	8.689
273	54	6	12	S	O	1.102	0.737	-1.291	4.079	-0.726	1.194	-3.772	72.765	10.71	8.689
274	54	7	11	S	Ti	37.458	-2.906	4.458	-4.222	3.086	-4.87	4.611	84.388	8.161	11.325
275	54	7	11	S	O	37.458	-2.906	4.458	-4.222	3.086	-4.87	4.611	84.388	8.161	11.325
276	54	7	11	S	O	37.458	-2.906	4.458	-4.222	3.086	-4.87	4.611	84.388	8.161	11.325
277	54	6	12	S	Ti	56.565	3.399	-2.872	1.383	-3.183	2.795	-1.346	72.691	8.691	10.711
278	54	6	12	S	O	56.565	3.399	-2.872	1.383	-3.183	2.795	-1.346	72.691	8.691	10.711
279	54	6	12	S	O	56.565	3.399	-2.872	1.383	-3.183	2.795	-1.346	72.691	8.691	10.711
280	54	7	11	S	Ti	2.333	-4.619	-4.192	-2.494	5.09	4.413	2.625	84.474	11.338	8.151
281	54	7	11	S	O	2.333	-4.619	-4.192	-2.494	5.09	4.413	2.625	84.474	11.338	8.151
282	54	7	11	S	O	2.333	-4.619	-4.192	-2.494	5.09	4.413	2.625	84.474	11.338	8.151
283	54	7	11	S	Ti	57.667	-4.619	4.192	-2.494	5.09	-4.413	2.625	84.474	11.338	8.151
284	54	7	11	S	O	57.667	-4.619	4.192	-2.494	5.09	-4.413	2.625	84.474	11.338	8.151
285	54	7	11	S	Ti	22.542	-2.906	-4.458	-4.222	3.086	4.87	4.611	84.388	8.161	11.325
286	54	7	11	S	O	22.542	-2.906	-4.458	-4.222	3.086	4.87	4.611	84.388	8.161	11.325
287	54	6	12	S	Ti	3.435	3.399	2.872	1.383	-3.183	-2.795	-1.346	72.691	8.691	10.711
288	54	6	12	S	O	3.435	3.399	2.872	1.383	-3.183	-2.795	-1.346	72.691	8.691	10.711
289	54	6	12	S	O	3.435	3.399	2.872	1.383	-3.183	-2.795	-1.346	72.691	8.691	10.711
290	54	6	12	S	Ti	58.898	0.737	1.291	4.079	-0.726	-1.194	-3.772	72.765	10.71	8.689
291	54	6	12	S	O	58.898	0.737	1.291	4.079	-0.726	-1.194	-3.772	72.765	10.71	8.689
292	54	7	11	S	Ti	7.458	-2.906	4.458	-4.222	3.086	-4.87	4.611	84.388	8.161	11.325

293	54	7	11	S	Ti	27.667	-4.619	4.193	-2.494	5.09	-4.413	2.625	84.474	11.338	8.151
294	54	7	11	S	O	27.667	-4.619	4.193	-2.494	5.09	-4.413	2.625	84.474	11.338	8.151
295	54	7	11	S	O	27.667	-4.619	4.193	-2.494	5.09	-4.413	2.625	84.474	11.338	8.151
296	54	7	11	S	Ti	52.542	-2.906	-4.458	-4.222	3.086	4.87	4.611	84.388	8.161	11.325
297	54	7	11	S	O	52.542	-2.906	-4.458	-4.222	3.086	4.87	4.611	84.388	8.161	11.325
298	54	7	11	S	O	52.542	-2.906	-4.458	-4.222	3.086	4.87	4.611	84.388	8.161	11.325
299	54	6	12	S	Ti	33.435	3.399	2.872	1.383	-3.183	-2.795	-1.346	72.691	8.691	10.711
300	54	6	12	S	O	33.435	3.399	2.872	1.383	-3.183	-2.795	-1.346	72.691	8.691	10.711
301	54	6	12	S	O	33.435	3.399	2.872	1.383	-3.183	-2.795	-1.346	72.691	8.691	10.711
302	54	6	12	S	Ti	28.898	0.737	1.291	4.079	-0.726	-1.194	-3.772	72.765	10.71	8.689
303	54	6	12	S	O	28.898	0.737	1.291	4.079	-0.726	-1.194	-3.772	72.765	10.71	8.689
304	54	6	12	S	O	28.898	0.737	1.291	4.079	-0.726	-1.194	-3.772	72.765	10.71	8.689
305	54	7	11	S	Ti	37.458	-2.906	4.458	-4.222	3.086	-4.87	4.611	84.388	8.161	11.325
306	54	7	11	S	O	37.458	-2.906	4.458	-4.222	3.086	-4.87	4.611	84.388	8.161	11.325
307	54	7	11	S	O	37.458	-2.906	4.458	-4.222	3.086	-4.87	4.611	84.388	8.161	11.325
308	54	7	11	S	Ti	2.333	-4.619	-4.192	-2.494	5.09	4.413	2.625	84.474	11.338	8.151
309	54	7	11	S	O	2.333	-4.619	-4.192	-2.494	5.09	4.413	2.625	84.474	11.338	8.151
310	54	7	11	S	O	2.333	-4.619	-4.192	-2.494	5.09	4.413	2.625	84.474	11.338	8.151
311	54	6	12	S	Ti	30	3.069	-3.221	1.704	-2.891	3.115	-1.647	72.77	8.684	10.715
312	54	6	12	S	Ti	30	3.069	3.221	1.704	-2.891	-3.115	-1.647	72.77	10.715	8.684
313	54	6	12	S	O	30	3.069	-3.221	1.704	-2.891	3.115	-1.647	72.77	8.684	10.715
314	54	6	12	S	O	30	3.069	3.221	1.704	-2.891	-3.115	-1.647	72.77	10.715	8.684
315	54	7	11	S	Ti	57.667	-4.619	4.192	-2.494	5.09	-4.413	2.625	84.474	11.338	8.151
316	54	7	11	S	O	57.667	-4.619	4.192	-2.494	5.09	-4.413	2.625	84.474	11.338	8.151
317	54	7	11	S	Ti	22.542	-2.906	-4.458	-4.222	3.086	4.87	4.611	84.388	8.161	11.325
318	54	7	11	S	O	22.542	-2.906	-4.458	-4.222	3.086	4.87	4.611	84.388	8.161	11.325
319	54	6	12	S	Ti	31.102	0.737	-1.291	4.079	-0.726	1.194	-3.772	72.765	10.71	8.689
320	54	7	11	S	Ti	7.458	-2.906	4.458	-4.222	3.086	-4.87	4.611	84.388	8.161	11.325
321	54	6	12	S	Ti	26.565	3.399	-2.872	1.383	-3.183	2.795	-1.346	72.691	8.691	10.711
322	54	6	12	S	O	26.565	3.399	-2.872	1.383	-3.183	2.795	-1.346	72.691	8.691	10.711
323	54	7	11	S	Ti	32.333	-4.619	-4.193	-2.494	5.09	4.413	2.625	84.474	11.338	8.151
324	54	7	11	S	O	32.333	-4.619	-4.193	-2.494	5.09	4.413	2.625	84.474	11.338	8.151
325	54	6	12	Sn	Ti	33.435	3.399	2.872	1.383	-3.183	-2.795	-1.346	72.691	8.691	10.711
326	54	6	12	Sn	O	33.435	3.399	2.872	1.383	-3.183	-2.795	-1.346	72.691	8.691	10.711
327	54	6	12	Sn	O	33.435	3.399	2.872	1.383	-3.183	-2.795	-1.346	72.691	8.691	10.711
328	54	6	12	Sn	Ti	1.102	0.737	-1.291	4.079	-0.726	1.194	-3.772	72.765	10.71	8.689
329	54	6	12	Sn	Ti	28.898	0.737	1.291	4.079	-0.726	-1.194	-3.772	72.765	10.71	8.689
330	54	6	12	Sn	O	1.102	0.737	-1.291	4.079	-0.726	1.194	-3.772	72.765	10.71	8.689
331	54	6	12	Sn	O	28.898	0.737	1.291	4.079	-0.726	-1.194	-3.772	72.765	10.71	8.689
332	54	6	12	Sn	O	1.102	0.737	-1.291	4.079	-0.726	1.194	-3.772	72.765	10.71	8.689
333	54	6	12	Sn	O	28.898	0.737	1.291	4.079	-0.726	-1.194	-3.772	72.765	10.71	8.689
334	54	7	11	Sn	Ti	37.458	-2.906	4.458	-4.222	3.086	-4.87	4.611	84.388	8.161	11.325

335	54	7	11	Sn	O	37.458	-2.906	4.458	-4.222	3.086	-4.87	4.611	84.388	8.161	11.325
336	54	7	11	Sn	O	37.458	-2.906	4.458	-4.222	3.086	-4.87	4.611	84.388	8.161	11.325
337	54	6	12	Sn	Ti	56.565	3.399	-2.872	1.383	-3.183	2.795	-1.346	72.691	8.691	10.711
338	54	6	12	Sn	O	56.565	3.399	-2.872	1.383	-3.183	2.795	-1.346	72.691	8.691	10.711
339	54	6	12	Sn	O	56.565	3.399	-2.872	1.383	-3.183	2.795	-1.346	72.691	8.691	10.711
340	54	7	11	Sn	Ti	2.333	-4.619	-4.192	-2.494	5.09	4.413	2.625	84.474	11.338	8.151
341	54	7	11	Sn	O	2.333	-4.619	-4.192	-2.494	5.09	4.413	2.625	84.474	11.338	8.151
342	54	7	11	Sn	O	2.333	-4.619	-4.192	-2.494	5.09	4.413	2.625	84.474	11.338	8.151
343	54	6	12	Sn	Ti	30	3.069	-3.221	1.704	-2.891	3.115	-1.647	72.77	8.684	10.715
344	54	6	12	Sn	Ti	30	3.069	3.221	1.704	-2.891	-3.115	-1.647	72.77	10.715	8.684
345	54	6	12	Sn	O	30	3.069	-3.221	1.704	-2.891	3.115	-1.647	72.77	8.684	10.715
346	54	6	12	Sn	O	30	3.069	3.221	1.704	-2.891	-3.115	-1.647	72.77	10.715	8.684
347	54	7	11	Sn	Ti	57.667	-4.619	4.192	-2.494	5.09	-4.413	2.625	84.474	11.338	8.151
348	54	7	11	Sn	O	57.667	-4.619	4.192	-2.494	5.09	-4.413	2.625	84.474	11.338	8.151
349	54	7	11	Sn	Ti	22.542	-2.906	-4.458	-4.222	3.086	4.87	4.611	84.388	8.161	11.325
350	54	7	11	Sn	O	22.542	-2.906	-4.458	-4.222	3.086	4.87	4.611	84.388	8.161	11.325
351	54	6	12	Sn	Ti	3.435	3.399	2.872	1.383	-3.183	-2.795	-1.346	72.691	8.691	10.711
352	54	6	12	Sn	O	3.435	3.399	2.872	1.383	-3.183	-2.795	-1.346	72.691	8.691	10.711
353	54	6	12	Sn	Ti	0	3.069	-3.221	1.704	-2.891	3.115	-1.647	72.77	8.684	10.715
354	54	6	12	Sn	O	3.435	3.399	2.872	1.383	-3.183	-2.795	-1.346	72.691	8.691	10.711
355	54	6	12	Sn	O	0	3.069	-3.221	1.704	-2.891	3.115	-1.647	72.77	8.684	10.715
356	54	6	12	Sn	O	0	3.069	-3.221	1.704	-2.891	3.115	-1.647	72.77	8.684	10.715
357	54	6	12	Sn	Ti	31.102	0.737	-1.291	4.079	-0.726	1.194	-3.772	72.765	10.71	8.689
358	54	6	12	Sn	Ti	58.898	0.737	1.291	4.079	-0.726	-1.194	-3.772	72.765	10.71	8.689
359	54	6	12	Sn	O	58.898	0.737	1.291	4.079	-0.726	-1.194	-3.772	72.765	10.71	8.689
360	54	7	11	Sn	Ti	27.667	-4.619	4.193	-2.494	5.09	-4.413	2.625	84.474	11.338	8.151
361	54	7	11	Sn	Ti	7.458	-2.906	4.458	-4.222	3.086	-4.87	4.611	84.388	8.161	11.325
362	54	6	12	Sn	Ti	26.565	3.399	-2.872	1.383	-3.183	2.795	-1.346	72.691	8.691	10.711
363	54	6	12	Sn	O	26.565	3.399	-2.872	1.383	-3.183	2.795	-1.346	72.691	8.691	10.711
364	54	7	11	Sn	Ti	32.333	-4.619	-4.193	-2.494	5.09	4.413	2.625	84.474	11.338	8.151
365	54	7	11	Sn	O	32.333	-4.619	-4.193	-2.494	5.09	4.413	2.625	84.474	11.338	8.151
366	54	7	11	Sn	O	27.667	-4.619	4.193	-2.494	5.09	-4.413	2.625	84.474	11.338	8.151
367	54	7	11	Sn	O	27.667	-4.619	4.193	-2.494	5.09	-4.413	2.625	84.474	11.338	8.151
368	54	7	11	Sn	Ti	52.542	-2.906	-4.458	-4.222	3.086	4.87	4.611	84.388	8.161	11.325
369	54	7	11	Sn	O	52.542	-2.906	-4.458	-4.222	3.086	4.87	4.611	84.388	8.161	11.325
370	54	7	11	Sn	O	52.542	-2.906	-4.458	-4.222	3.086	4.87	4.611	84.388	8.161	11.325
371	57	7	12	S	Ti	45.672	-2.906	-2.972	-0.06	3.086	2.976	0.06	80.434	8.161	11.926
372	57	7	12	S	O	45.672	-2.906	-2.972	-0.06	3.086	2.976	0.06	80.434	8.161	11.926
373	57	7	12	S	Ti	18.435	0.345	1.792	-3.286	-0.343	-1.918	3.517	80.533	11.928	8.159
374	57	7	12	S	O	18.435	0.345	1.792	-3.286	-0.343	-1.918	3.517	80.533	11.928	8.159
375	57	7	12	S	Ti	41.565	0.345	-1.792	-3.286	-0.343	1.918	3.517	80.533	11.928	8.159
376	57	7	12	S	O	41.565	0.345	-1.792	-3.286	-0.343	1.918	3.517	80.533	11.928	8.159

377	57	7	12	S	O	41.565	0.345	-1.792	-3.286	-0.343	1.918	3.517	80.533	11.928	8.159
378	57	7	12	S	Ti	14.328	-2.906	2.972	-0.06	3.086	-2.976	0.06	80.434	8.161	11.926
379	57	7	12	S	O	14.328	-2.906	2.972	-0.06	3.086	-2.976	0.06	80.434	8.161	11.926
380	57	7	12	S	O	14.328	-2.906	2.972	-0.06	3.086	-2.976	0.06	80.434	8.161	11.926
381	57	7	12	S	Ti	49.723	-1.882	4.057	-1.123	1.956	-4.151	1.149	80.532	8.153	11.932
382	57	7	12	S	O	49.723	-1.882	4.057	-1.123	1.956	-4.151	1.149	80.532	8.153	11.932
383	57	7	12	S	O	49.723	-1.882	4.057	-1.123	1.956	-4.151	1.149	80.532	8.153	11.932
384	57	7	12	S	Ti	40.277	-1.882	-4.057	-1.123	1.956	4.151	1.149	80.532	11.932	8.153
385	57	7	12	S	O	40.277	-1.882	-4.057	-1.123	1.956	4.151	1.149	80.532	11.932	8.153
386	57	7	12	S	O	40.277	-1.882	-4.057	-1.123	1.956	4.151	1.149	80.532	11.932	8.153
387	57	7	12	S	Ti	15.672	-2.906	-2.972	-0.06	3.086	2.976	0.06	80.434	8.161	11.926
388	57	7	12	S	O	15.672	-2.906	-2.972	-0.06	3.086	2.976	0.06	80.434	8.161	11.926
389	57	7	12	S	O	15.672	-2.906	-2.972	-0.06	3.086	2.976	0.06	80.434	8.161	11.926
390	57	7	12	S	Ti	48.435	0.345	1.792	-3.286	-0.343	-1.918	3.517	80.533	11.928	8.159
391	57	7	12	S	O	48.435	0.345	1.792	-3.286	-0.343	-1.918	3.517	80.533	11.928	8.159
392	57	7	12	S	O	48.435	0.345	1.792	-3.286	-0.343	-1.918	3.517	80.533	11.928	8.159
393	57	7	12	S	Ti	11.565	0.345	-1.792	-3.286	-0.343	1.918	3.517	80.533	11.928	8.159
394	57	7	12	S	O	11.565	0.345	-1.792	-3.286	-0.343	1.918	3.517	80.533	11.928	8.159
395	57	7	12	S	O	11.565	0.345	-1.792	-3.286	-0.343	1.918	3.517	80.533	11.928	8.159
396	57	7	12	S	Ti	44.328	-2.906	2.972	-0.06	3.086	-2.976	0.06	80.434	8.161	11.926
397	57	7	12	S	O	44.328	-2.906	2.972	-0.06	3.086	-2.976	0.06	80.434	8.161	11.926
398	57	7	12	S	Ti	19.723	-1.882	4.057	-1.123	1.956	-4.151	1.149	80.532	8.153	11.932
399	57	7	12	S	O	19.723	-1.882	4.057	-1.123	1.956	-4.151	1.149	80.532	8.153	11.932
400	57	7	12	S	Ti	10.277	-1.882	-4.057	-1.123	1.956	4.151	1.149	80.532	11.932	8.153
401	57	7	12	S	Ti	41.565	0.345	-1.792	-3.286	-0.343	1.918	3.517	80.533	11.928	8.159
402	57	7	12	S	O	41.565	0.345	-1.792	-3.286	-0.343	1.918	3.517	80.533	11.928	8.159
403	57	7	12	S	Ti	18.435	0.345	1.792	-3.286	-0.343	-1.918	3.517	80.533	11.928	8.159
404	57	7	12	S	O	18.435	0.345	1.792	-3.286	-0.343	-1.918	3.517	80.533	11.928	8.159
405	57	7	12	Sn	Ti	49.723	-1.882	4.057	-1.123	1.956	-4.151	1.149	80.532	8.153	11.932
406	57	7	12	Sn	O	49.723	-1.882	4.057	-1.123	1.956	-4.151	1.149	80.532	8.153	11.932
407	57	7	12	Sn	O	49.723	-1.882	4.057	-1.123	1.956	-4.151	1.149	80.532	8.153	11.932
408	57	7	12	Sn	Ti	40.277	-1.882	-4.057	-1.123	1.956	4.151	1.149	80.532	11.932	8.153
409	57	7	12	Sn	Ti	15.672	-2.906	-2.972	-0.06	3.086	2.976	0.06	80.434	8.161	11.926
410	57	7	12	Sn	O	15.672	-2.906	-2.972	-0.06	3.086	2.976	0.06	80.434	8.161	11.926
411	57	7	12	Sn	O	15.672	-2.906	-2.972	-0.06	3.086	2.976	0.06	80.434	8.161	11.926
412	57	7	12	Sn	Ti	48.435	0.345	1.792	-3.286	-0.343	-1.918	3.517	80.533	11.928	8.159
413	57	7	12	Sn	O	48.435	0.345	1.792	-3.286	-0.343	-1.918	3.517	80.533	11.928	8.159
414	57	7	12	Sn	O	48.435	0.345	1.792	-3.286	-0.343	-1.918	3.517	80.533	11.928	8.159
415	57	7	12	Sn	Ti	11.565	0.345	-1.792	-3.286	-0.343	1.918	3.517	80.533	11.928	8.159
416	57	7	12	Sn	O	11.565	0.345	-1.792	-3.286	-0.343	1.918	3.517	80.533	11.928	8.159
417	57	7	12	Sn	O	11.565	0.345	-1.792	-3.286	-0.343	1.918	3.517	80.533	11.928	8.159
418	57	7	12	Sn	Ti	44.328	-2.906	2.972	-0.06	3.086	-2.976	0.06	80.434	8.161	11.926

419	57	7	12	Sn	O	44.328	-2.906	2.972	-0.06	3.086	-2.976	0.06	80.434	8.161	11.926
420	57	7	12	Sn	Ti	19.723	-1.882	4.057	-1.123	1.956	-4.151	1.149	80.532	8.153	11.932
421	57	7	12	Sn	O	19.723	-1.882	4.057	-1.123	1.956	-4.151	1.149	80.532	8.153	11.932
422	57	7	12	Sn	Ti	10.277	-1.882	-4.057	-1.123	1.956	4.151	1.149	80.532	11.932	8.153
423	57	7	12	Sn	Ti	45.672	-2.906	-2.972	-0.06	3.086	2.976	0.06	80.434	8.161	11.926
424	57	7	12	Sn	O	45.672	-2.906	-2.972	-0.06	3.086	2.976	0.06	80.434	8.161	11.926
425	57	7	12	Sn	Ti	18.435	0.345	1.792	-3.286	-0.343	-1.918	3.517	80.533	11.928	8.159
426	57	7	12	Sn	O	18.435	0.345	1.792	-3.286	-0.343	-1.918	3.517	80.533	11.928	8.159
427	57	7	12	Sn	Ti	41.565	0.345	-1.792	-3.286	-0.343	1.918	3.517	80.533	11.928	8.159
428	57	7	12	Sn	O	41.565	0.345	-1.792	-3.286	-0.343	1.918	3.517	80.533	11.928	8.159
429	57	7	12	Sn	O	41.565	0.345	-1.792	-3.286	-0.343	1.918	3.517	80.533	11.928	8.159
430	57	7	12	Sn	Ti	14.328	-2.906	2.972	-0.06	3.086	-2.976	0.06	80.434	8.161	11.926
431	57	7	12	Sn	O	14.328	-2.906	2.972	-0.06	3.086	-2.976	0.06	80.434	8.161	11.926
432	57	7	12	Sn	O	14.328	-2.906	2.972	-0.06	3.086	-2.976	0.06	80.434	8.161	11.926
433	60	7	13	S	Ti	41.848	4.862	2.365	-3.559	-4.431	-2.547	3.832	79.804	12.465	8.153
434	60	7	13	S	Ti	18.152	4.862	-2.365	-3.559	-4.431	2.547	3.832	79.804	12.465	8.153
435	60	7	13	S	O	18.152	4.862	-2.365	-3.559	-4.431	2.547	3.832	79.804	12.465	8.153
436	60	7	13	S	O	18.152	4.862	-2.365	-3.559	-4.431	2.547	3.832	79.804	12.465	8.153
437	60	7	13	S	Ti	52.542	-2.906	5.449	4.102	3.086	-5.036	-3.791	79.486	8.161	12.459
438	60	7	13	S	O	52.542	-2.906	5.449	4.102	3.086	-5.036	-3.791	79.486	8.161	12.459
439	60	7	13	S	O	52.542	-2.906	5.449	4.102	3.086	-5.036	-3.791	79.486	8.161	12.459
440	60	7	13	S	Ti	37.458	-2.906	-5.449	4.102	3.086	5.036	-3.791	79.486	8.161	12.459
441	60	7	13	S	O	37.458	-2.906	-5.449	4.102	3.086	5.036	-3.791	79.486	8.161	12.459
442	60	7	13	S	O	37.458	-2.906	-5.449	4.102	3.086	5.036	-3.791	79.486	8.161	12.459
443	60	7	13	S	Ti	11.848	4.862	2.365	-3.559	-4.431	-2.547	3.832	79.804	12.465	8.153
444	60	7	13	S	O	11.848	4.862	2.365	-3.559	-4.431	-2.547	3.832	79.804	12.465	8.153
445	60	7	13	S	O	11.848	4.862	2.365	-3.559	-4.431	-2.547	3.832	79.804	12.465	8.153
446	60	7	13	S	Ti	48.152	4.862	-2.365	-3.559	-4.431	2.547	3.832	79.804	12.465	8.153
447	60	7	13	S	O	48.152	4.862	-2.365	-3.559	-4.431	2.547	3.832	79.804	12.465	8.153
448	60	7	13	S	Ti	22.542	-2.906	5.449	4.102	3.086	-5.036	-3.791	79.486	8.161	12.459
449	60	7	13	S	O	22.542	-2.906	5.449	4.102	3.086	-5.036	-3.791	79.486	8.161	12.459
450	60	7	13	S	Ti	7.458	-2.906	-5.449	4.102	3.086	5.036	-3.791	79.486	8.161	12.459
451	60	7	13	S	Ti	18.152	4.862	-2.365	-3.559	-4.431	2.547	3.832	79.804	12.465	8.153
452	60	7	13	S	O	18.152	4.862	-2.365	-3.559	-4.431	2.547	3.832	79.804	12.465	8.153
453	60	7	13	S	O	18.152	4.862	-2.365	-3.559	-4.431	2.547	3.832	79.804	12.465	8.153
454	60	7	13	S	Ti	52.542	-2.906	5.449	4.102	3.086	-5.036	-3.791	79.486	8.161	12.459
455	60	7	13	S	O	52.542	-2.906	5.449	4.102	3.086	-5.036	-3.791	79.486	8.161	12.459
456	60	7	13	S	O	52.542	-2.906	5.449	4.102	3.086	-5.036	-3.791	79.486	8.161	12.459
457	60	7	13	S	Ti	37.458	-2.906	-5.449	4.102	3.086	5.036	-3.791	79.486	8.161	12.459
458	60	7	13	S	O	37.458	-2.906	-5.449	4.102	3.086	5.036	-3.791	79.486	8.161	12.459
459	60	7	13	S	O	37.458	-2.906	-5.449	4.102	3.086	5.036	-3.791	79.486	8.161	12.459
460	60	7	13	S	Ti	11.848	4.862	2.365	-3.559	-4.431	-2.547	3.832	79.804	12.465	8.153

461	60	7	13	S	O	11.848	4.862	2.365	-3.559	-4.431	-2.547	3.832	79.804	12.465	8.153
462	60	7	13	S	O	11.848	4.862	2.365	-3.559	-4.431	-2.547	3.832	79.804	12.465	8.153
463	60	7	13	S	Ti	48.152	4.862	-2.365	-3.559	-4.431	2.547	3.832	79.804	12.465	8.153
464	60	7	13	S	O	48.152	4.862	-2.365	-3.559	-4.431	2.547	3.832	79.804	12.465	8.153
465	60	7	13	S	Ti	22.542	-2.906	5.449	4.102	3.086	-5.036	-3.791	79.486	8.161	12.459
466	60	7	13	S	O	22.542	-2.906	5.449	4.102	3.086	-5.036	-3.791	79.486	8.161	12.459
467	60	7	13	S	Ti	7.458	-2.906	-5.449	4.102	3.086	5.036	-3.791	79.486	8.161	12.459
468	60	7	13	S	Ti	41.848	4.862	2.365	-3.559	-4.431	-2.547	3.832	79.804	12.465	8.153
469	60	7	13	Sn	O	52.542	-2.906	5.449	4.102	3.086	-5.036	-3.791	79.486	8.161	12.459
470	60	7	13	Sn	Ti	37.458	-2.906	-5.449	4.102	3.086	5.036	-3.791	79.486	8.161	12.459
471	60	7	13	Sn	O	37.458	-2.906	-5.449	4.102	3.086	5.036	-3.791	79.486	8.161	12.459
472	60	7	13	Sn	O	37.458	-2.906	-5.449	4.102	3.086	5.036	-3.791	79.486	8.161	12.459
473	60	7	13	Sn	Ti	11.848	4.862	2.365	-3.559	-4.431	-2.547	3.832	79.804	12.465	8.153
474	60	7	13	Sn	O	11.848	4.862	2.365	-3.559	-4.431	-2.547	3.832	79.804	12.465	8.153
475	60	7	13	Sn	O	11.848	4.862	2.365	-3.559	-4.431	-2.547	3.832	79.804	12.465	8.153
476	60	7	13	Sn	Ti	48.152	4.862	-2.365	-3.559	-4.431	2.547	3.832	79.804	12.465	8.153
477	60	7	13	Sn	O	48.152	4.862	-2.365	-3.559	-4.431	2.547	3.832	79.804	12.465	8.153
478	60	7	13	Sn	Ti	22.542	-2.906	5.449	4.102	3.086	-5.036	-3.791	79.486	8.161	12.459
479	60	7	13	Sn	O	22.542	-2.906	5.449	4.102	3.086	-5.036	-3.791	79.486	8.161	12.459
480	60	7	13	Sn	Ti	7.458	-2.906	-5.449	4.102	3.086	5.036	-3.791	79.486	8.161	12.459
481	60	7	13	Sn	Ti	41.848	4.862	2.365	-3.559	-4.431	-2.547	3.832	79.804	12.465	8.153
482	60	7	13	Sn	Ti	18.152	4.862	-2.365	-3.559	-4.431	2.547	3.832	79.804	12.465	8.153
483	60	7	13	Sn	O	18.152	4.862	-2.365	-3.559	-4.431	2.547	3.832	79.804	12.465	8.153
484	60	7	13	Sn	O	18.152	4.862	-2.365	-3.559	-4.431	2.547	3.832	79.804	12.465	8.153
485	60	7	13	Sn	Ti	52.542	-2.906	5.449	4.102	3.086	-5.036	-3.791	79.486	8.161	12.459
486	60	7	13	Sn	O	52.542	-2.906	5.449	4.102	3.086	-5.036	-3.791	79.486	8.161	12.459
487	63	8	13	S	Ti	45.672	-2.906	4.768	-2.661	3.086	-5.036	2.811	80.148	8.161	13.286
488	63	8	13	S	O	45.672	-2.906	4.768	-2.661	3.086	-5.036	2.811	80.148	8.161	13.286
489	63	8	13	S	Ti	14.328	-2.906	-4.768	-2.661	3.086	5.036	2.811	80.148	8.161	13.286
490	63	8	13	S	O	14.328	-2.906	-4.768	-2.661	3.086	5.036	2.811	80.148	8.161	13.286
491	63	8	13	S	O	14.328	-2.906	-4.768	-2.661	3.086	5.036	2.811	80.148	8.161	13.286
492	63	8	13	S	Ti	49.723	-1.882	4.357	-3.668	1.956	-4.702	3.959	80.21	13.298	8.153
493	63	8	13	S	O	49.723	-1.882	4.357	-3.668	1.956	-4.702	3.959	80.21	13.298	8.153
494	63	8	13	S	O	49.723	-1.882	4.357	-3.668	1.956	-4.702	3.959	80.21	13.298	8.153
495	63	8	13	S	Ti	40.277	-1.882	-4.357	-3.668	1.956	4.702	3.959	80.21	13.298	8.153
496	63	8	13	S	O	40.277	-1.882	-4.357	-3.668	1.956	4.702	3.959	80.21	13.298	8.153
497	63	8	13	S	O	40.277	-1.882	-4.357	-3.668	1.956	4.702	3.959	80.21	13.298	8.153
498	63	8	13	S	Ti	15.672	-2.906	4.768	-2.661	3.086	-5.036	2.811	80.148	8.161	13.286
499	63	8	13	S	O	15.672	-2.906	4.768	-2.661	3.086	-5.036	2.811	80.148	8.161	13.286
500	63	8	13	S	O	15.672	-2.906	4.768	-2.661	3.086	-5.036	2.811	80.148	8.161	13.286
501	63	8	13	S	Ti	44.328	-2.906	-4.768	-2.661	3.086	5.036	2.811	80.148	8.161	13.286
502	63	8	13	S	O	44.328	-2.906	-4.768	-2.661	3.086	5.036	2.811	80.148	8.161	13.286

503	63	8	13	S	Ti	19.723	-1.882	4.357	-3.668	1.956	-4.702	3.959	80.21	13.298	8.153
504	63	8	13	S	O	19.723	-1.882	4.357	-3.668	1.956	-4.702	3.959	80.21	13.298	8.153
505	63	8	13	S	Ti	10.277	-1.882	-4.357	-3.668	1.956	4.702	3.959	80.21	13.298	8.153
506	63	8	13	S	Ti	14.328	-2.906	-4.768	-2.661	3.086	5.036	2.811	80.148	8.161	13.286
507	63	8	13	S	O	14.328	-2.906	-4.768	-2.661	3.086	5.036	2.811	80.148	8.161	13.286
508	63	8	13	S	O	14.328	-2.906	-4.768	-2.661	3.086	5.036	2.811	80.148	8.161	13.286
509	63	8	13	S	Ti	49.723	-1.882	4.357	-3.668	1.956	-4.702	3.959	80.21	13.298	8.153
510	63	8	13	S	O	49.723	-1.882	4.357	-3.668	1.956	-4.702	3.959	80.21	13.298	8.153
511	63	8	13	S	O	49.723	-1.882	4.357	-3.668	1.956	-4.702	3.959	80.21	13.298	8.153
512	63	8	13	S	Ti	40.277	-1.882	-4.357	-3.668	1.956	4.702	3.959	80.21	13.298	8.153
513	63	8	13	S	O	40.277	-1.882	-4.357	-3.668	1.956	4.702	3.959	80.21	13.298	8.153
514	63	8	13	S	O	40.277	-1.882	-4.357	-3.668	1.956	4.702	3.959	80.21	13.298	8.153
515	63	8	13	S	Ti	15.672	-2.906	4.768	-2.661	3.086	-5.036	2.811	80.148	8.161	13.286
516	63	8	13	S	O	15.672	-2.906	4.768	-2.661	3.086	-5.036	2.811	80.148	8.161	13.286
517	63	8	13	S	O	15.672	-2.906	4.768	-2.661	3.086	-5.036	2.811	80.148	8.161	13.286
518	63	8	13	S	Ti	44.328	-2.906	-4.768	-2.661	3.086	5.036	2.811	80.148	8.161	13.286
519	63	8	13	S	O	44.328	-2.906	-4.768	-2.661	3.086	5.036	2.811	80.148	8.161	13.286
520	63	8	13	S	Ti	19.723	-1.882	4.357	-3.668	1.956	-4.702	3.959	80.21	13.298	8.153
521	63	8	13	S	O	19.723	-1.882	4.357	-3.668	1.956	-4.702	3.959	80.21	13.298	8.153
522	63	8	13	S	Ti	10.277	-1.882	-4.357	-3.668	1.956	4.702	3.959	80.21	13.298	8.153
523	63	8	13	S	Ti	45.672	-2.906	4.768	-2.661	3.086	-5.036	2.811	80.148	8.161	13.286
524	63	8	13	S	O	45.672	-2.906	4.768	-2.661	3.086	-5.036	2.811	80.148	8.161	13.286
525	63	8	13	Sn	Ti	49.723	-1.882	4.357	-3.668	1.956	-4.702	3.959	80.21	13.298	8.153
526	63	8	13	Sn	O	49.723	-1.882	4.357	-3.668	1.956	-4.702	3.959	80.21	13.298	8.153
527	63	8	13	Sn	O	49.723	-1.882	4.357	-3.668	1.956	-4.702	3.959	80.21	13.298	8.153
528	63	8	13	Sn	Ti	40.277	-1.882	-4.357	-3.668	1.956	4.702	3.959	80.21	13.298	8.153
529	63	8	13	Sn	O	40.277	-1.882	-4.357	-3.668	1.956	4.702	3.959	80.21	13.298	8.153
530	63	8	13	Sn	O	40.277	-1.882	-4.357	-3.668	1.956	4.702	3.959	80.21	13.298	8.153
531	63	8	13	Sn	Ti	15.672	-2.906	4.768	-2.661	3.086	-5.036	2.811	80.148	8.161	13.286
532	63	8	13	Sn	O	15.672	-2.906	4.768	-2.661	3.086	-5.036	2.811	80.148	8.161	13.286
533	63	8	13	Sn	O	15.672	-2.906	4.768	-2.661	3.086	-5.036	2.811	80.148	8.161	13.286
534	63	8	13	Sn	Ti	44.328	-2.906	-4.768	-2.661	3.086	5.036	2.811	80.148	8.161	13.286
535	63	8	13	Sn	O	44.328	-2.906	-4.768	-2.661	3.086	5.036	2.811	80.148	8.161	13.286
536	63	8	13	Sn	Ti	19.723	-1.882	4.357	-3.668	1.956	-4.702	3.959	80.21	13.298	8.153
537	63	8	13	Sn	O	19.723	-1.882	4.357	-3.668	1.956	-4.702	3.959	80.21	13.298	8.153
538	63	8	13	Sn	Ti	10.277	-1.882	-4.357	-3.668	1.956	4.702	3.959	80.21	13.298	8.153
539	63	8	13	Sn	Ti	45.672	-2.906	4.768	-2.661	3.086	-5.036	2.811	80.148	8.161	13.286
540	63	8	13	Sn	O	45.672	-2.906	4.768	-2.661	3.086	-5.036	2.811	80.148	8.161	13.286
541	63	8	13	Sn	Ti	14.328	-2.906	-4.768	-2.661	3.086	5.036	2.811	80.148	8.161	13.286
542	63	8	13	Sn	O	14.328	-2.906	-4.768	-2.661	3.086	5.036	2.811	80.148	8.161	13.286
543	63	8	13	Sn	O	14.328	-2.906	-4.768	-2.661	3.086	5.036	2.811	80.148	8.161	13.286
544	66	7	15	S	Ti	26.565	3.399	1.231	5.054	-3.183	-1.118	-4.59	82.656	8.691	12.464

545	66	7	15	S	O	26.565	3.399	1.231	5.054	-3.183	-1.118	-4.59	82.656	8.691	12.464
546	66	7	15	S	Ti	55.022	4.862	-1.577	3.585	-4.431	1.471	-3.345	82.627	12.465	8.691
547	66	7	15	S	O	55.022	4.862	-1.577	3.585	-4.431	1.471	-3.345	82.627	12.465	8.691
548	66	7	15	S	Ti	4.978	4.862	1.577	3.585	-4.431	-1.471	-3.345	82.627	12.465	8.691
549	66	7	15	S	O	4.978	4.862	1.577	3.585	-4.431	-1.471	-3.345	82.627	12.465	8.691
550	66	7	15	S	O	4.978	4.862	1.577	3.585	-4.431	-1.471	-3.345	82.627	12.465	8.691
551	66	7	15	S	Ti	33.435	3.399	-1.231	5.054	-3.183	1.118	-4.59	82.656	8.691	12.464
552	66	7	15	S	O	33.435	3.399	-1.231	5.054	-3.183	1.118	-4.59	82.656	8.691	12.464
553	66	7	15	S	O	33.435	3.399	-1.231	5.054	-3.183	1.118	-4.59	82.656	8.691	12.464
554	66	7	15	S	Ti	26.31	5.194	0.751	3.263	-4.706	-0.705	-3.063	82.684	8.692	12.462
555	66	7	15	S	O	26.31	5.194	0.751	3.263	-4.706	-0.705	-3.063	82.684	8.692	12.462
556	66	7	15	S	O	26.31	5.194	0.751	3.263	-4.706	-0.705	-3.063	82.684	8.692	12.462
557	66	7	15	S	Ti	3.69	5.194	-0.751	3.263	-4.706	0.705	-3.063	82.684	12.462	8.692
558	66	7	15	S	O	3.69	5.194	-0.751	3.263	-4.706	0.705	-3.063	82.684	12.462	8.692
559	66	7	15	S	O	3.69	5.194	-0.751	3.263	-4.706	0.705	-3.063	82.684	12.462	8.692
560	66	7	15	S	Ti	56.565	3.399	1.231	5.054	-3.183	-1.118	-4.59	82.656	8.691	12.464
561	66	7	15	S	O	56.565	3.399	1.231	5.054	-3.183	-1.118	-4.59	82.656	8.691	12.464
562	66	7	15	S	O	56.565	3.399	1.231	5.054	-3.183	-1.118	-4.59	82.656	8.691	12.464
563	66	7	15	S	Ti	25.022	4.862	-1.577	3.585	-4.431	1.471	-3.345	82.627	12.465	8.691
564	66	7	15	S	O	25.022	4.862	-1.577	3.585	-4.431	1.471	-3.345	82.627	12.465	8.691
565	66	7	15	S	O	25.022	4.862	-1.577	3.585	-4.431	1.471	-3.345	82.627	12.465	8.691
566	66	7	15	S	Ti	34.978	4.862	1.577	3.585	-4.431	-1.471	-3.345	82.627	12.465	8.691
567	66	7	15	S	Ti	3.435	3.399	-1.231	5.054	-3.183	1.118	-4.59	82.656	8.691	12.464
568	66	7	15	S	O	3.435	3.399	-1.231	5.054	-3.183	1.118	-4.59	82.656	8.691	12.464
569	66	7	15	S	O	3.435	3.399	-1.231	5.054	-3.183	1.118	-4.59	82.656	8.691	12.464
570	66	7	15	S	Ti	56.31	5.194	0.751	3.263	-4.706	-0.705	-3.063	82.684	8.692	12.462
571	66	7	15	S	O	56.31	5.194	0.751	3.263	-4.706	-0.705	-3.063	82.684	8.692	12.462
572	66	7	15	S	Ti	33.69	5.194	-0.751	3.263	-4.706	0.705	-3.063	82.684	12.462	8.692
573	66	7	15	S	O	33.69	5.194	-0.751	3.263	-4.706	0.705	-3.063	82.684	12.462	8.692
574	66	7	15	S	Ti	4.978	4.862	1.577	3.585	-4.431	-1.471	-3.345	82.627	12.465	8.691
575	66	7	15	S	O	4.978	4.862	1.577	3.585	-4.431	-1.471	-3.345	82.627	12.465	8.691
576	66	7	15	S	O	4.978	4.862	1.577	3.585	-4.431	-1.471	-3.345	82.627	12.465	8.691
577	66	7	15	S	Ti	33.435	3.399	-1.231	5.054	-3.183	1.118	-4.59	82.656	8.691	12.464
578	66	7	15	S	O	33.435	3.399	-1.231	5.054	-3.183	1.118	-4.59	82.656	8.691	12.464
579	66	7	15	S	O	33.435	3.399	-1.231	5.054	-3.183	1.118	-4.59	82.656	8.691	12.464
580	66	7	15	S	Ti	26.31	5.194	0.751	3.263	-4.706	-0.705	-3.063	82.684	8.692	12.462
581	66	7	15	S	O	26.31	5.194	0.751	3.263	-4.706	-0.705	-3.063	82.684	8.692	12.462
582	66	7	15	S	O	26.31	5.194	0.751	3.263	-4.706	-0.705	-3.063	82.684	8.692	12.462
583	66	7	15	S	Ti	3.69	5.194	-0.751	3.263	-4.706	0.705	-3.063	82.684	12.462	8.692
584	66	7	15	S	O	3.69	5.194	-0.751	3.263	-4.706	0.705	-3.063	82.684	12.462	8.692
585	66	7	15	S	O	3.69	5.194	-0.751	3.263	-4.706	0.705	-3.063	82.684	12.462	8.692
586	66	7	15	S	Ti	56.565	3.399	1.231	5.054	-3.183	-1.118	-4.59	82.656	8.691	12.464

587	66	7	15	S	O	56.565	3.399	1.231	5.054	-3.183	-1.118	-4.59	82.656	8.691	12.464
588	66	7	15	S	O	56.565	3.399	1.231	5.054	-3.183	-1.118	-4.59	82.656	8.691	12.464
589	66	7	15	S	Ti	25.022	4.862	-1.577	3.585	-4.431	1.471	-3.345	82.627	12.465	8.691
590	66	7	15	S	O	25.022	4.862	-1.577	3.585	-4.431	1.471	-3.345	82.627	12.465	8.691
591	66	7	15	S	O	25.022	4.862	-1.577	3.585	-4.431	1.471	-3.345	82.627	12.465	8.691
592	66	7	15	S	Ti	34.978	4.862	1.577	3.585	-4.431	-1.471	-3.345	82.627	12.465	8.691
593	66	7	15	S	Ti	3.435	3.399	-1.231	5.054	-3.183	1.118	-4.59	82.656	8.691	12.464
594	66	7	15	S	O	3.435	3.399	-1.231	5.054	-3.183	1.118	-4.59	82.656	8.691	12.464
595	66	7	15	S	O	3.435	3.399	-1.231	5.054	-3.183	1.118	-4.59	82.656	8.691	12.464
596	66	7	15	S	Ti	56.31	5.194	0.751	3.263	-4.706	-0.705	-3.063	82.684	8.692	12.462
597	66	7	15	S	O	56.31	5.194	0.751	3.263	-4.706	-0.705	-3.063	82.684	8.692	12.462
598	66	7	15	S	Ti	33.69	5.194	-0.751	3.263	-4.706	0.705	-3.063	82.684	12.462	8.692
599	66	7	15	S	O	33.69	5.194	-0.751	3.263	-4.706	0.705	-3.063	82.684	12.462	8.692
600	66	7	15	S	Ti	26.565	3.399	1.231	5.054	-3.183	-1.118	-4.59	82.656	8.691	12.464
601	66	7	15	S	O	26.565	3.399	1.231	5.054	-3.183	-1.118	-4.59	82.656	8.691	12.464
602	66	7	15	S	Ti	55.022	4.862	-1.577	3.585	-4.431	1.471	-3.345	82.627	12.465	8.691
603	66	7	15	S	O	55.022	4.862	-1.577	3.585	-4.431	1.471	-3.345	82.627	12.465	8.691
604	66	7	15	Sn	Ti	33.435	3.399	-1.231	5.054	-3.183	1.118	-4.59	82.656	8.691	12.464
605	66	7	15	Sn	O	33.435	3.399	-1.231	5.054	-3.183	1.118	-4.59	82.656	8.691	12.464
606	66	7	15	Sn	O	33.435	3.399	-1.231	5.054	-3.183	1.118	-4.59	82.656	8.691	12.464
607	66	7	15	Sn	Ti	26.31	5.194	0.751	3.263	-4.706	-0.705	-3.063	82.684	8.692	12.462
608	66	7	15	Sn	O	26.31	5.194	0.751	3.263	-4.706	-0.705	-3.063	82.684	8.692	12.462
609	66	7	15	Sn	O	26.31	5.194	0.751	3.263	-4.706	-0.705	-3.063	82.684	8.692	12.462
610	66	7	15	Sn	Ti	3.69	5.194	-0.751	3.263	-4.706	0.705	-3.063	82.684	12.462	8.692
611	66	7	15	Sn	O	3.69	5.194	-0.751	3.263	-4.706	0.705	-3.063	82.684	12.462	8.692
612	66	7	15	Sn	O	3.69	5.194	-0.751	3.263	-4.706	0.705	-3.063	82.684	12.462	8.692
613	66	7	15	Sn	Ti	56.565	3.399	1.231	5.054	-3.183	-1.118	-4.59	82.656	8.691	12.464
614	66	7	15	Sn	O	56.565	3.399	1.231	5.054	-3.183	-1.118	-4.59	82.656	8.691	12.464
615	66	7	15	Sn	O	56.565	3.399	1.231	5.054	-3.183	-1.118	-4.59	82.656	8.691	12.464
616	66	7	15	Sn	Ti	25.022	4.862	-1.577	3.585	-4.431	1.471	-3.345	82.627	12.465	8.691
617	66	7	15	Sn	O	25.022	4.862	-1.577	3.585	-4.431	1.471	-3.345	82.627	12.465	8.691
618	66	7	15	Sn	O	25.022	4.862	-1.577	3.585	-4.431	1.471	-3.345	82.627	12.465	8.691
619	66	7	15	Sn	Ti	34.978	4.862	1.577	3.585	-4.431	-1.471	-3.345	82.627	12.465	8.691
620	66	7	15	Sn	Ti	3.435	3.399	-1.231	5.054	-3.183	1.118	-4.59	82.656	8.691	12.464
621	66	7	15	Sn	O	3.435	3.399	-1.231	5.054	-3.183	1.118	-4.59	82.656	8.691	12.464
622	66	7	15	Sn	O	3.435	3.399	-1.231	5.054	-3.183	1.118	-4.59	82.656	8.691	12.464
623	66	7	15	Sn	Ti	56.31	5.194	0.751	3.263	-4.706	-0.705	-3.063	82.684	8.692	12.462
624	66	7	15	Sn	O	56.31	5.194	0.751	3.263	-4.706	-0.705	-3.063	82.684	8.692	12.462
625	66	7	15	Sn	Ti	33.69	5.194	-0.751	3.263	-4.706	0.705	-3.063	82.684	12.462	8.692
626	66	7	15	Sn	O	33.69	5.194	-0.751	3.263	-4.706	0.705	-3.063	82.684	12.462	8.692
627	66	7	15	Sn	Ti	26.565	3.399	1.231	5.054	-3.183	-1.118	-4.59	82.656	8.691	12.464
628	66	7	15	Sn	O	26.565	3.399	1.231	5.054	-3.183	-1.118	-4.59	82.656	8.691	12.464

629	66	7	15	Sn	Ti	55.022	4.862	-1.577	3.585	-4.431	1.471	-3.345	82.627	12.465	8.691
630	66	7	15	Sn	O	55.022	4.862	-1.577	3.585	-4.431	1.471	-3.345	82.627	12.465	8.691
631	66	7	15	Sn	Ti	4.978	4.862	1.577	3.585	-4.431	-1.471	-3.345	82.627	12.465	8.691
632	66	7	15	Sn	O	4.978	4.862	1.577	3.585	-4.431	-1.471	-3.345	82.627	12.465	8.691
633	66	7	15	Sn	O	4.978	4.862	1.577	3.585	-4.431	-1.471	-3.345	82.627	12.465	8.691
634	69	8	15	S	Ti	26.565	3.399	-1.077	-1.828	-3.183	1.118	1.897	83.119	8.691	13.298
635	69	8	15	S	O	26.565	3.399	-1.077	-1.828	-3.183	1.118	1.897	83.119	8.691	13.298
636	69	9	14	S	Ti	30	-4.041	-3.998	-3.565	4.397	4.305	3.839	73.816	10.821	11.324
637	69	9	14	S	Ti	30	-4.041	3.998	-3.565	4.397	-4.305	3.839	73.816	10.821	11.324
638	69	8	15	S	Ti	52.542	-2.906	-4.768	4.622	3.086	4.365	-4.231	80.822	8.161	14.252
639	69	8	15	S	O	52.542	-2.906	-4.768	4.622	3.086	4.365	-4.231	80.822	8.161	14.252
640	69	8	15	S	O	52.542	-2.906	-4.768	4.622	3.086	4.365	-4.231	80.822	8.161	14.252
641	69	8	15	S	Ti	33.435	3.399	1.077	-1.828	-3.183	-1.118	1.897	83.119	8.691	13.298
642	69	8	15	S	O	33.435	3.399	1.077	-1.828	-3.183	-1.118	1.897	83.119	8.691	13.298
643	69	8	15	S	O	33.435	3.399	1.077	-1.828	-3.183	-1.118	1.897	83.119	8.691	13.298
644	69	8	15	S	Ti	2.897	-1.882	0.161	3.46	1.956	-0.151	-3.236	83.151	13.298	8.691
645	69	8	15	S	O	2.897	-1.882	0.161	3.46	1.956	-0.151	-3.236	83.151	13.298	8.691
646	69	8	15	S	O	2.897	-1.882	0.161	3.46	1.956	-0.151	-3.236	83.151	13.298	8.691
647	69	8	15	S	Ti	26.31	5.194	1.972	-3.395	-4.706	-2.116	3.642	81.104	14.257	8.155
648	69	8	15	S	O	26.31	5.194	1.972	-3.395	-4.706	-2.116	3.642	81.104	14.257	8.155
649	69	8	15	S	O	26.31	5.194	1.972	-3.395	-4.706	-2.116	3.642	81.104	14.257	8.155
650	69	8	15	S	Ti	28.898	0.737	-1.937	0.7	-0.726	1.911	-0.69	88.898	10.71	10.708
651	69	8	15	S	O	28.898	0.737	-1.937	0.7	-0.726	1.911	-0.69	88.898	10.71	10.708
652	69	8	15	S	O	28.898	0.737	-1.937	0.7	-0.726	1.911	-0.69	88.898	10.71	10.708
653	69	8	15	S	Ti	27.103	-1.882	-0.161	3.46	1.956	0.151	-3.236	83.151	13.298	8.691
654	69	8	15	S	O	27.103	-1.882	-0.161	3.46	1.956	0.151	-3.236	83.151	13.298	8.691
655	69	8	15	S	O	27.103	-1.882	-0.161	3.46	1.956	0.151	-3.236	83.151	13.298	8.691
656	69	8	15	S	Ti	3.69	5.194	-1.972	-3.395	-4.706	2.116	3.642	81.104	14.257	8.155
657	69	8	15	S	O	3.69	5.194	-1.972	-3.395	-4.706	2.116	3.642	81.104	14.257	8.155
658	69	8	15	S	O	3.69	5.194	-1.972	-3.395	-4.706	2.116	3.642	81.104	14.257	8.155
659	69	8	15	S	Ti	37.458	-2.906	4.768	4.622	3.086	-4.365	-4.231	80.822	8.161	14.252
660	69	8	15	S	O	37.458	-2.906	4.768	4.622	3.086	-4.365	-4.231	80.822	8.161	14.252
661	69	8	15	S	O	37.458	-2.906	4.768	4.622	3.086	-4.365	-4.231	80.822	8.161	14.252
662	69	8	15	S	Ti	56.565	3.399	-1.077	-1.828	-3.183	1.118	1.897	83.119	8.691	13.298
663	69	8	15	S	O	56.565	3.399	-1.077	-1.828	-3.183	1.118	1.897	83.119	8.691	13.298
664	69	8	15	S	O	56.565	3.399	-1.077	-1.828	-3.183	1.118	1.897	83.119	8.691	13.298
665	69	9	14	S	Ti	34.537	-4.619	3.669	-2.974	5.09	-3.9	3.161	73.835	11.338	10.807
666	69	9	14	S	Ti	25.463	-4.619	-3.669	-2.974	5.09	3.9	3.161	73.835	11.338	10.807
667	69	8	15	S	Ti	22.542	-2.906	-4.768	4.622	3.086	4.365	-4.231	80.822	8.161	14.252
668	69	8	15	S	O	22.542	-2.906	-4.768	4.622	3.086	4.365	-4.231	80.822	8.161	14.252
669	69	8	15	S	Ti	3.435	3.399	1.077	-1.828	-3.183	-1.118	1.897	83.119	8.691	13.298
670	69	8	15	S	O	3.435	3.399	1.077	-1.828	-3.183	-1.118	1.897	83.119	8.691	13.298

671	69	8	15	S	O	3.435	3.399	1.077	-1.828	-3.183	-1.118	1.897	83.119	8.691	13.298
672	69	8	15	S	Ti	32.897	-1.882	0.161	3.46	1.956	-0.151	-3.236	83.151	13.298	8.691
673	69	8	15	S	O	32.897	-1.882	0.161	3.46	1.956	-0.151	-3.236	83.151	13.298	8.691
674	69	8	15	S	Ti	56.31	5.194	1.972	-3.395	-4.706	-2.116	3.642	81.104	14.257	8.155
675	69	8	15	S	O	56.31	5.194	1.972	-3.395	-4.706	-2.116	3.642	81.104	14.257	8.155
676	69	8	15	S	Ti	31.102	0.737	1.937	0.7	-0.726	-1.911	-0.69	88.898	10.71	10.708
677	69	8	15	S	Ti	58.898	0.737	-1.937	0.7	-0.726	1.911	-0.69	88.898	10.71	10.708
678	69	8	15	S	O	58.898	0.737	-1.937	0.7	-0.726	1.911	-0.69	88.898	10.71	10.708
679	69	8	15	S	Ti	57.103	-1.882	-0.161	3.46	1.956	0.151	-3.236	83.151	13.298	8.691
680	69	8	15	S	O	57.103	-1.882	-0.161	3.46	1.956	0.151	-3.236	83.151	13.298	8.691
681	69	8	15	S	Ti	33.69	5.194	-1.972	-3.395	-4.706	2.116	3.642	81.104	14.257	8.155
682	69	8	15	S	O	33.69	5.194	-1.972	-3.395	-4.706	2.116	3.642	81.104	14.257	8.155
683	69	8	15	S	Ti	7.458	-2.906	4.768	4.622	3.086	-4.365	-4.231	80.822	8.161	14.252
684	69	9	14	S	Ti	55.463	-4.619	-3.669	-2.974	5.09	3.9	3.161	73.835	11.338	10.807
685	69	8	15	S	Ti	52.542	-2.906	-4.768	4.622	3.086	4.365	-4.231	80.822	8.161	14.252
686	69	8	15	S	O	52.542	-2.906	-4.768	4.622	3.086	4.365	-4.231	80.822	8.161	14.252
687	69	8	15	S	O	52.542	-2.906	-4.768	4.622	3.086	4.365	-4.231	80.822	8.161	14.252
688	69	8	15	S	Ti	33.435	3.399	1.077	-1.828	-3.183	-1.118	1.897	83.119	8.691	13.298
689	69	8	15	S	O	33.435	3.399	1.077	-1.828	-3.183	-1.118	1.897	83.119	8.691	13.298
690	69	8	15	S	O	33.435	3.399	1.077	-1.828	-3.183	-1.118	1.897	83.119	8.691	13.298
691	69	8	15	S	Ti	2.897	-1.882	0.161	3.46	1.956	-0.151	-3.236	83.151	13.298	8.691
692	69	8	15	S	O	2.897	-1.882	0.161	3.46	1.956	-0.151	-3.236	83.151	13.298	8.691
693	69	8	15	S	O	2.897	-1.882	0.161	3.46	1.956	-0.151	-3.236	83.151	13.298	8.691
694	69	8	15	S	Ti	26.31	5.194	1.972	-3.395	-4.706	-2.116	3.642	81.104	14.257	8.155
695	69	8	15	S	O	26.31	5.194	1.972	-3.395	-4.706	-2.116	3.642	81.104	14.257	8.155
696	69	8	15	S	O	26.31	5.194	1.972	-3.395	-4.706	-2.116	3.642	81.104	14.257	8.155
697	69	8	15	S	Ti	28.898	0.737	-1.937	0.7	-0.726	1.911	-0.69	88.898	10.71	10.708
698	69	8	15	S	O	28.898	0.737	-1.937	0.7	-0.726	1.911	-0.69	88.898	10.71	10.708
699	69	8	15	S	O	28.898	0.737	-1.937	0.7	-0.726	1.911	-0.69	88.898	10.71	10.708
700	69	8	15	S	Ti	27.103	-1.882	-0.161	3.46	1.956	0.151	-3.236	83.151	13.298	8.691
701	69	8	15	S	O	27.103	-1.882	-0.161	3.46	1.956	0.151	-3.236	83.151	13.298	8.691
702	69	8	15	S	O	27.103	-1.882	-0.161	3.46	1.956	0.151	-3.236	83.151	13.298	8.691
703	69	8	15	S	Ti	3.69	5.194	-1.972	-3.395	-4.706	2.116	3.642	81.104	14.257	8.155
704	69	8	15	S	O	3.69	5.194	-1.972	-3.395	-4.706	2.116	3.642	81.104	14.257	8.155
705	69	8	15	S	O	3.69	5.194	-1.972	-3.395	-4.706	2.116	3.642	81.104	14.257	8.155
706	69	8	15	S	Ti	37.458	-2.906	4.768	4.622	3.086	-4.365	-4.231	80.822	8.161	14.252
707	69	8	15	S	O	37.458	-2.906	4.768	4.622	3.086	-4.365	-4.231	80.822	8.161	14.252
708	69	8	15	S	O	37.458	-2.906	4.768	4.622	3.086	-4.365	-4.231	80.822	8.161	14.252
709	69	8	15	S	Ti	56.565	3.399	-1.077	-1.828	-3.183	1.118	1.897	83.119	8.691	13.298
710	69	8	15	S	O	56.565	3.399	-1.077	-1.828	-3.183	1.118	1.897	83.119	8.691	13.298
711	69	8	15	S	O	56.565	3.399	-1.077	-1.828	-3.183	1.118	1.897	83.119	8.691	13.298
712	69	9	14	S	Ti	0	-4.041	-3.998	-3.565	4.397	4.305	3.839	73.816	10.821	11.324

713	69	8	15	S	Ti	22.542	-2.906	-4.768	4.622	3.086	4.365	-4.231	80.822	8.161	14.252
714	69	8	15	S	O	22.542	-2.906	-4.768	4.622	3.086	4.365	-4.231	80.822	8.161	14.252
715	69	8	15	S	Ti	3.435	3.399	1.077	-1.828	-3.183	-1.118	1.897	83.119	8.691	13.298
716	69	8	15	S	O	3.435	3.399	1.077	-1.828	-3.183	-1.118	1.897	83.119	8.691	13.298
717	69	8	15	S	O	3.435	3.399	1.077	-1.828	-3.183	-1.118	1.897	83.119	8.691	13.298
718	69	8	15	S	Ti	32.897	-1.882	0.161	3.46	1.956	-0.151	-3.236	83.151	13.298	8.691
719	69	8	15	S	O	32.897	-1.882	0.161	3.46	1.956	-0.151	-3.236	83.151	13.298	8.691
720	69	8	15	S	Ti	56.31	5.194	1.972	-3.395	-4.706	-2.116	3.642	81.104	14.257	8.155
721	69	8	15	S	O	56.31	5.194	1.972	-3.395	-4.706	-2.116	3.642	81.104	14.257	8.155
722	69	8	15	S	Ti	58.898	0.737	-1.937	0.7	-0.726	1.911	-0.69	88.898	10.71	10.708
723	69	8	15	S	O	58.898	0.737	-1.937	0.7	-0.726	1.911	-0.69	88.898	10.71	10.708
724	69	8	15	S	Ti	57.103	-1.882	-0.161	3.46	1.956	0.151	-3.236	83.151	13.298	8.691
725	69	8	15	S	O	57.103	-1.882	-0.161	3.46	1.956	0.151	-3.236	83.151	13.298	8.691
726	69	8	15	S	Ti	33.69	5.194	-1.972	-3.395	-4.706	2.116	3.642	81.104	14.257	8.155
727	69	8	15	S	O	33.69	5.194	-1.972	-3.395	-4.706	2.116	3.642	81.104	14.257	8.155
728	69	8	15	S	Ti	7.458	-2.906	4.768	4.622	3.086	-4.365	-4.231	80.822	8.161	14.252
729	69	8	15	S	Ti	26.565	3.399	-1.077	-1.828	-3.183	1.118	1.897	83.119	8.691	13.298
730	69	8	15	S	O	26.565	3.399	-1.077	-1.828	-3.183	1.118	1.897	83.119	8.691	13.298
731	69	8	15	Sn	Ti	33.435	3.399	1.077	-1.828	-3.183	-1.118	1.897	83.119	8.691	13.298
732	69	8	15	Sn	O	33.435	3.399	1.077	-1.828	-3.183	-1.118	1.897	83.119	8.691	13.298
733	69	8	15	Sn	O	33.435	3.399	1.077	-1.828	-3.183	-1.118	1.897	83.119	8.691	13.298
734	69	8	15	Sn	Ti	2.897	-1.882	0.161	3.46	1.956	-0.151	-3.236	83.151	13.298	8.691
735	69	8	15	Sn	O	2.897	-1.882	0.161	3.46	1.956	-0.151	-3.236	83.151	13.298	8.691
736	69	8	15	Sn	O	2.897	-1.882	0.161	3.46	1.956	-0.151	-3.236	83.151	13.298	8.691
737	69	8	15	Sn	Ti	26.31	5.194	1.972	-3.395	-4.706	-2.116	3.642	81.104	14.257	8.155
738	69	8	15	Sn	O	26.31	5.194	1.972	-3.395	-4.706	-2.116	3.642	81.104	14.257	8.155
739	69	9	14	Sn	O	30	-4.041	-3.998	-3.565	4.397	4.305	3.839	73.816	10.821	11.324
740	69	8	15	Sn	O	26.31	5.194	1.972	-3.395	-4.706	-2.116	3.642	81.104	14.257	8.155
741	69	8	15	Sn	Ti	28.898	0.737	-1.937	0.7	-0.726	1.911	-0.69	88.898	10.71	10.708
742	69	8	15	Sn	O	28.898	0.737	-1.937	0.7	-0.726	1.911	-0.69	88.898	10.71	10.708
743	69	8	15	Sn	O	28.898	0.737	-1.937	0.7	-0.726	1.911	-0.69	88.898	10.71	10.708
744	69	8	15	Sn	Ti	27.103	-1.882	-0.161	3.46	1.956	0.151	-3.236	83.151	13.298	8.691
745	69	8	15	Sn	O	27.103	-1.882	-0.161	3.46	1.956	0.151	-3.236	83.151	13.298	8.691
746	69	8	15	Sn	O	27.103	-1.882	-0.161	3.46	1.956	0.151	-3.236	83.151	13.298	8.691
747	69	8	15	Sn	Ti	3.69	5.194	-1.972	-3.395	-4.706	2.116	3.642	81.104	14.257	8.155
748	69	8	15	Sn	O	3.69	5.194	-1.972	-3.395	-4.706	2.116	3.642	81.104	14.257	8.155
749	69	8	15	Sn	O	3.69	5.194	-1.972	-3.395	-4.706	2.116	3.642	81.104	14.257	8.155
750	69	9	14	Sn	O	30	-4.041	3.998	-3.565	4.397	-4.305	3.839	73.816	10.821	11.324
751	69	8	15	Sn	Ti	37.458	-2.906	4.768	4.622	3.086	-4.365	-4.231	80.822	8.161	14.252
752	69	8	15	Sn	O	37.458	-2.906	4.768	4.622	3.086	-4.365	-4.231	80.822	8.161	14.252
753	69	8	15	Sn	O	37.458	-2.906	4.768	4.622	3.086	-4.365	-4.231	80.822	8.161	14.252
754	69	8	15	Sn	Ti	56.565	3.399	-1.077	-1.828	-3.183	1.118	1.897	83.119	8.691	13.298

755	69	8	15	Sn	O	56.565	3.399	-1.077	-1.828	-3.183	1.118	1.897	83.119	8.691	13.298
756	69	8	15	Sn	O	56.565	3.399	-1.077	-1.828	-3.183	1.118	1.897	83.119	8.691	13.298
757	69	9	14	Sn	Ti	34.537	-4.619	3.669	-2.974	5.09	-3.9	3.161	73.835	11.338	10.807
758	69	9	14	Sn	Ti	0	-4.041	-3.998	-3.565	4.397	4.305	3.839	73.816	10.821	11.324
759	69	8	15	Sn	Ti	22.542	-2.906	-4.768	4.622	3.086	4.365	-4.231	80.822	8.161	14.252
760	69	8	15	Sn	O	22.542	-2.906	-4.768	4.622	3.086	4.365	-4.231	80.822	8.161	14.252
761	69	8	15	Sn	Ti	3.435	3.399	1.077	-1.828	-3.183	-1.118	1.897	83.119	8.691	13.298
762	69	8	15	Sn	O	3.435	3.399	1.077	-1.828	-3.183	-1.118	1.897	83.119	8.691	13.298
763	69	8	15	Sn	O	3.435	3.399	1.077	-1.828	-3.183	-1.118	1.897	83.119	8.691	13.298
764	69	8	15	Sn	Ti	32.897	-1.882	0.161	3.46	1.956	-0.151	-3.236	83.151	13.298	8.691
765	69	8	15	Sn	O	32.897	-1.882	0.161	3.46	1.956	-0.151	-3.236	83.151	13.298	8.691
766	69	8	15	Sn	Ti	56.31	5.194	1.972	-3.395	-4.706	-2.116	3.642	81.104	14.257	8.155
767	69	8	15	Sn	O	56.31	5.194	1.972	-3.395	-4.706	-2.116	3.642	81.104	14.257	8.155
768	69	8	15	Sn	Ti	58.898	0.737	-1.937	0.7	-0.726	1.911	-0.69	88.898	10.71	10.708
769	69	8	15	Sn	O	58.898	0.737	-1.937	0.7	-0.726	1.911	-0.69	88.898	10.71	10.708
770	69	8	15	Sn	Ti	57.103	-1.882	-0.161	3.46	1.956	0.151	-3.236	83.151	13.298	8.691
771	69	8	15	Sn	O	57.103	-1.882	-0.161	3.46	1.956	0.151	-3.236	83.151	13.298	8.691
772	69	8	15	Sn	Ti	33.69	5.194	-1.972	-3.395	-4.706	2.116	3.642	81.104	14.257	8.155
773	69	8	15	Sn	O	33.69	5.194	-1.972	-3.395	-4.706	2.116	3.642	81.104	14.257	8.155
774	69	9	14	Sn	Ti	55.463	-4.619	-3.669	-2.974	5.09	3.9	3.161	73.835	11.338	10.807
775	69	8	15	Sn	Ti	7.458	-2.906	4.768	4.622	3.086	-4.365	-4.231	80.822	8.161	14.252
776	69	8	15	Sn	Ti	26.565	3.399	-1.077	-1.828	-3.183	1.118	1.897	83.119	8.691	13.298
777	69	8	15	Sn	O	26.565	3.399	-1.077	-1.828	-3.183	1.118	1.897	83.119	8.691	13.298
778	69	9	14	Sn	Ti	4.537	-4.619	3.669	-2.974	5.09	-3.9	3.161	73.835	11.338	10.807
779	69	8	15	Sn	Ti	52.542	-2.906	-4.768	4.622	3.086	4.365	-4.231	80.822	8.161	14.252
780	69	8	15	Sn	O	52.542	-2.906	-4.768	4.622	3.086	4.365	-4.231	80.822	8.161	14.252
781	69	8	15	Sn	O	52.542	-2.906	-4.768	4.622	3.086	4.365	-4.231	80.822	8.161	14.252
782	75	10	15	S	Ti	32.333	-4.619	-1.834	-4.653	5.09	2.022	5.13	88.898	11.338	11.336
783	75	10	15	S	O	32.333	-4.619	-1.834	-4.653	5.09	2.022	5.13	88.898	11.338	11.336
784	75	9	16	S	Ti	18.435	0.345	2.787	-1.555	-0.343	-2.877	1.605	72.701	11.928	11.031
785	75	9	16	S	O	18.435	0.345	2.787	-1.555	-0.343	-2.877	1.605	72.701	11.928	11.031
786	75	9	16	S	Ti	13.898	-2.165	1.254	0.987	2.263	-1.23	-0.967	72.75	11.033	11.924
787	75	9	16	S	Ti	46.102	-2.165	-1.254	0.987	2.263	1.23	-0.967	72.75	11.033	11.924
788	75	9	16	S	O	13.898	-2.165	1.254	0.987	2.263	-1.23	-0.967	72.75	11.033	11.924
789	75	9	16	S	O	46.102	-2.165	-1.254	0.987	2.263	1.23	-0.967	72.75	11.033	11.924
790	75	9	16	S	O	13.898	-2.165	1.254	0.987	2.263	-1.23	-0.967	72.75	11.033	11.924
791	75	9	16	S	O	46.102	-2.165	-1.254	0.987	2.263	1.23	-0.967	72.75	11.033	11.924
792	75	9	16	S	Ti	0	3.069	-4.294	-4.041	-2.891	4.672	4.397	72.605	11.623	11.34
793	75	9	16	S	O	0	3.069	-4.294	-4.041	-2.891	4.672	4.397	72.605	11.623	11.34
794	75	9	16	S	O	0	3.069	-4.294	-4.041	-2.891	4.672	4.397	72.605	11.623	11.34
795	75	10	15	S	Ti	27.667	-4.619	1.834	-4.653	5.09	-2.022	5.13	88.898	11.338	11.336
796	75	10	15	S	Ti	55.463	-4.619	-1.834	-4.653	5.09	2.022	5.131	88.898	11.338	11.336

797	75	10	15	S	O	27.667	-4.619	1.834	-4.653	5.09	-2.022	5.13	88.898	11.338	11.336
798	75	10	15	S	O	55.463	-4.619	-1.834	-4.653	5.09	2.022	5.131	88.898	11.338	11.336
799	75	10	15	S	O	27.667	-4.619	1.834	-4.653	5.09	-2.022	5.13	88.898	11.338	11.336
800	75	10	15	S	O	55.463	-4.619	-1.834	-4.653	5.09	2.022	5.131	88.898	11.338	11.336
801	75	9	16	S	Ti	41.565	0.345	-2.787	-1.555	-0.343	2.877	1.605	72.701	11.928	11.031
802	75	9	16	S	O	41.565	0.345	-2.787	-1.555	-0.343	2.877	1.605	72.701	11.928	11.031
803	75	9	16	S	O	41.565	0.345	-2.787	-1.555	-0.343	2.877	1.605	72.701	11.928	11.031
804	75	9	16	S	Ti	15.417	0.587	-2.395	-1.786	-0.58	2.484	1.853	72.745	11.923	11.033
805	75	9	16	S	O	15.417	0.587	-2.395	-1.786	-0.58	2.484	1.853	72.745	11.923	11.033
806	75	9	16	S	O	15.417	0.587	-2.395	-1.786	-0.58	2.484	1.853	72.745	11.923	11.033
807	75	9	16	S	Ti	14.583	0.587	2.395	-1.786	-0.58	-2.484	1.853	72.745	11.033	11.923
808	75	9	16	S	O	14.583	0.587	2.395	-1.786	-0.58	-2.484	1.853	72.745	11.033	11.923
809	75	9	16	S	O	14.583	0.587	2.395	-1.786	-0.58	-2.484	1.853	72.745	11.033	11.923
810	75	9	16	S	Ti	2.333	-4.619	-0.408	3.745	5.09	0.379	-3.484	72.756	11.338	11.621
811	75	10	15	S	Ti	2.333	-4.619	-1.834	-4.653	5.09	2.022	5.131	88.898	11.338	11.336
812	75	10	15	S	Ti	34.537	-4.619	1.834	-4.653	5.09	-2.022	5.131	88.898	11.338	11.336
813	75	10	15	S	O	2.333	-4.619	-1.834	-4.653	5.09	2.022	5.131	88.898	11.338	11.336
814	75	10	15	S	O	34.537	-4.619	1.834	-4.653	5.09	-2.022	5.131	88.898	11.338	11.336
815	75	9	16	S	O	2.333	-4.619	-0.408	3.745	5.09	0.379	-3.484	72.756	11.338	11.621
816	75	10	15	S	O	2.333	-4.619	-1.834	-4.653	5.09	2.022	5.131	88.898	11.338	11.336
817	75	10	15	S	O	34.537	-4.619	1.834	-4.653	5.09	-2.022	5.131	88.898	11.338	11.336
818	75	9	16	S	Ti	48.435	0.345	2.787	-1.555	-0.343	-2.877	1.605	72.701	11.928	11.031
819	75	9	16	S	O	48.435	0.345	2.787	-1.555	-0.343	-2.877	1.605	72.701	11.928	11.031
820	75	9	16	S	O	48.435	0.345	2.787	-1.555	-0.343	-2.877	1.605	72.701	11.928	11.031
821	75	9	16	S	Ti	43.898	-2.165	1.254	0.987	2.263	-1.23	-0.967	72.75	11.033	11.924
822	75	9	16	S	Ti	16.102	-2.165	-1.254	0.987	2.263	1.23	-0.967	72.75	11.033	11.924
823	75	9	16	S	O	43.898	-2.165	1.254	0.987	2.263	-1.23	-0.967	72.75	11.033	11.924
824	75	9	16	S	O	16.102	-2.165	-1.254	0.987	2.263	1.23	-0.967	72.75	11.033	11.924
825	75	9	16	S	Ti	57.667	-4.619	0.408	3.745	5.09	-0.379	-3.484	72.756	11.338	11.621
826	75	9	16	S	O	57.667	-4.619	0.408	3.745	5.09	-0.379	-3.484	72.756	11.338	11.621
827	75	9	16	S	Ti	11.565	0.345	-2.787	-1.555	-0.343	2.877	1.605	72.701	11.928	11.031
828	75	9	16	S	O	11.565	0.345	-2.787	-1.555	-0.343	2.877	1.605	72.701	11.928	11.031
829	75	9	16	S	O	11.565	0.345	-2.787	-1.555	-0.343	2.877	1.605	72.701	11.928	11.031
830	75	9	16	S	Ti	45.417	0.587	-2.395	-1.786	-0.58	2.484	1.853	72.745	11.923	11.033
831	75	9	16	S	O	45.417	0.587	-2.395	-1.786	-0.58	2.484	1.853	72.745	11.923	11.033
832	75	9	16	S	Ti	44.583	0.587	2.395	-1.786	-0.58	-2.484	1.853	72.745	11.033	11.923
833	75	9	16	S	O	44.583	0.587	2.395	-1.786	-0.58	-2.484	1.853	72.745	11.033	11.923
834	75	9	16	S	Ti	27.667	-4.619	0.408	3.745	5.09	-0.379	-3.484	72.756	11.338	11.621
835	75	10	15	S	Ti	27.667	-4.619	1.834	-4.653	5.09	-2.022	5.13	88.898	11.338	11.336
836	75	9	16	S	O	27.667	-4.619	0.408	3.745	5.09	-0.379	-3.484	72.756	11.338	11.621
837	75	10	15	S	O	27.667	-4.619	1.834	-4.653	5.09	-2.022	5.13	88.898	11.338	11.336
838	75	9	16	S	O	27.667	-4.619	0.408	3.745	5.09	-0.379	-3.484	72.756	11.338	11.621

839	75	10	15	S	O	27.667	-4.619	1.834	-4.653	5.09	-2.022	5.13	88.898	11.338	11.336
840	75	10	15	S	Ti	2.333	-4.619	-1.834	-4.653	5.09	2.022	5.131	88.898	11.338	11.336
841	75	10	15	S	Ti	34.537	-4.619	1.834	-4.653	5.09	-2.022	5.131	88.898	11.338	11.336
842	75	10	15	S	O	2.333	-4.619	-1.834	-4.653	5.09	2.022	5.131	88.898	11.338	11.336
843	75	10	15	S	O	34.537	-4.619	1.834	-4.653	5.09	-2.022	5.131	88.898	11.338	11.336
844	75	10	15	S	O	2.333	-4.619	-1.834	-4.653	5.09	2.022	5.131	88.898	11.338	11.336
845	75	10	15	S	O	34.537	-4.619	1.834	-4.653	5.09	-2.022	5.131	88.898	11.338	11.336
846	75	9	16	S	Ti	30	3.069	-4.294	-4.041	-2.891	4.672	4.397	72.605	11.623	11.34
847	75	9	16	S	Ti	30	3.069	4.294	-4.041	-2.891	-4.672	4.397	72.605	11.623	11.34
848	75	9	16	S	O	30	3.069	-4.294	-4.041	-2.891	4.672	4.397	72.605	11.623	11.34
849	75	9	16	S	O	30	3.069	4.294	-4.041	-2.891	-4.672	4.397	72.605	11.623	11.34
850	75	10	15	S	Ti	57.667	-4.619	1.834	-4.653	5.09	-2.022	5.13	88.898	11.338	11.336
851	75	10	15	S	O	57.667	-4.619	1.834	-4.653	5.09	-2.022	5.13	88.898	11.338	11.336
852	75	10	15	S	Ti	4.537	-4.619	1.834	-4.653	5.09	-2.022	5.13	88.898	11.338	11.336
853	75	9	16	S	Ti	32.333	-4.619	-0.408	3.745	5.09	0.379	-3.484	72.756	11.338	11.621
854	75	9	16	S	O	32.333	-4.619	-0.408	3.745	5.09	0.379	-3.484	72.756	11.338	11.621
855	75	9	16	Sn	Ti	15.417	0.587	-2.395	-1.786	-0.58	2.484	1.853	72.745	11.923	11.033
856	75	9	16	Sn	O	13.898	-2.165	1.254	0.987	2.263	-1.23	-0.967	72.75	11.033	11.924
857	75	9	16	Sn	O	15.417	0.587	-2.395	-1.786	-0.58	2.484	1.853	72.745	11.923	11.033
858	75	9	16	Sn	O	15.417	0.587	-2.395	-1.786	-0.58	2.484	1.853	72.745	11.923	11.033
859	75	9	16	Sn	O	46.102	-2.165	-1.254	0.987	2.263	1.23	-0.967	72.75	11.033	11.924
860	75	9	16	Sn	O	13.898	-2.165	1.254	0.987	2.263	-1.23	-0.967	72.75	11.033	11.924
861	75	9	16	Sn	Ti	14.583	0.587	2.395	-1.786	-0.58	-2.484	1.853	72.745	11.033	11.923
862	75	9	16	Sn	O	14.583	0.587	2.395	-1.786	-0.58	-2.484	1.853	72.745	11.033	11.923
863	75	9	16	Sn	O	14.583	0.587	2.395	-1.786	-0.58	-2.484	1.853	72.745	11.033	11.923
864	75	9	16	Sn	O	46.102	-2.165	-1.254	0.987	2.263	1.23	-0.967	72.75	11.033	11.924
865	75	9	16	Sn	Ti	2.333	-4.619	-0.408	3.745	5.09	0.379	-3.484	72.756	11.338	11.621
866	75	10	15	Sn	Ti	2.333	-4.619	-1.834	-4.653	5.09	2.022	5.131	88.898	11.338	11.336
867	75	10	15	Sn	Ti	34.537	-4.619	1.834	-4.653	5.09	-2.022	5.131	88.898	11.338	11.336
868	75	9	16	Sn	O	2.333	-4.619	-0.408	3.745	5.09	0.379	-3.484	72.756	11.338	11.621
869	75	10	15	Sn	O	2.333	-4.619	-1.834	-4.653	5.09	2.022	5.131	88.898	11.338	11.336
870	75	10	15	Sn	O	34.537	-4.619	1.834	-4.653	5.09	-2.022	5.131	88.898	11.338	11.336
871	75	9	16	Sn	O	2.333	-4.619	-0.408	3.745	5.09	0.379	-3.484	72.756	11.338	11.621
872	75	10	15	Sn	O	2.333	-4.619	-1.834	-4.653	5.09	2.022	5.131	88.898	11.338	11.336
873	75	10	15	Sn	O	34.537	-4.619	1.834	-4.653	5.09	-2.022	5.131	88.898	11.338	11.336
874	75	9	16	Sn	Ti	48.435	0.345	2.787	-1.555	-0.343	-2.877	1.605	72.701	11.928	11.031
875	75	9	16	Sn	O	48.435	0.345	2.787	-1.555	-0.343	-2.877	1.605	72.701	11.928	11.031
876	75	9	16	Sn	O	48.435	0.345	2.787	-1.555	-0.343	-2.877	1.605	72.701	11.928	11.031
877	75	9	16	Sn	Ti	43.898	-2.165	1.254	0.987	2.263	-1.23	-0.967	72.75	11.033	11.924
878	75	9	16	Sn	Ti	16.102	-2.165	-1.254	0.987	2.263	1.23	-0.967	72.75	11.033	11.924
879	75	9	16	Sn	O	43.898	-2.165	1.254	0.987	2.263	-1.23	-0.967	72.75	11.033	11.924
880	75	9	16	Sn	O	16.102	-2.165	-1.254	0.987	2.263	1.23	-0.967	72.75	11.033	11.924

881	75	9	16	Sn	Ti	30	3.069	-4.294	-4.041	-2.891	4.672	4.397	72.605	11.623	11.34
882	75	9	16	Sn	Ti	30	3.069	4.294	-4.041	-2.891	-4.672	4.397	72.605	11.623	11.34
883	75	9	16	Sn	O	30	3.069	-4.294	-4.041	-2.891	4.672	4.397	72.605	11.623	11.34
884	75	9	16	Sn	O	30	3.069	4.294	-4.041	-2.891	-4.672	4.397	72.605	11.623	11.34
885	75	9	16	Sn	Ti	57.667	-4.619	0.408	3.745	5.09	-0.379	-3.484	72.756	11.338	11.621
886	75	10	15	Sn	Ti	57.667	-4.619	1.834	-4.653	5.09	-2.022	5.13	88.898	11.338	11.336
887	75	10	15	Sn	Ti	25.463	-4.619	-1.834	-4.653	5.09	2.022	5.131	88.898	11.338	11.336
888	75	9	16	Sn	O	57.667	-4.619	0.408	3.745	5.09	-0.379	-3.484	72.756	11.338	11.621
889	75	10	15	Sn	O	57.667	-4.619	1.834	-4.653	5.09	-2.022	5.13	88.898	11.338	11.336
890	75	10	15	Sn	O	25.463	-4.619	-1.834	-4.653	5.09	2.022	5.131	88.898	11.338	11.336
891	75	9	16	Sn	Ti	11.565	0.345	-2.787	-1.555	-0.343	2.877	1.605	72.701	11.928	11.031
892	75	9	16	Sn	O	11.565	0.345	-2.787	-1.555	-0.343	2.877	1.605	72.701	11.928	11.031
893	75	9	16	Sn	O	30	-4.041	0	3.069	4.397	0	-2.891	90	10.821	11.623
894	75	9	16	Sn	O	11.565	0.345	-2.787	-1.555	-0.343	2.877	1.605	72.701	11.928	11.031
895	75	9	16	Sn	Ti	0	3.069	-4.294	-4.041	-2.891	4.672	4.397	72.605	11.623	11.34
896	75	9	16	Sn	Ti	45.417	0.587	-2.395	-1.786	-0.58	2.484	1.853	72.745	11.923	11.033
897	75	9	16	Sn	Ti	0	3.069	0	-4.041	-2.891	0	4.397	90	11.623	10.821
898	75	9	16	Sn	O	45.417	0.587	-2.395	-1.786	-0.58	2.484	1.853	72.745	11.923	11.033
899	75	9	16	Sn	O	0	3.069	-4.294	-4.041	-2.891	4.672	4.397	72.605	11.623	11.34
900	75	9	16	Sn	O	0	3.069	-4.294	-4.041	-2.891	4.672	4.397	72.605	11.623	11.34
901	75	9	16	Sn	Ti	27.667	-4.619	0.408	3.745	5.09	-0.379	-3.484	72.756	11.338	11.621
902	75	9	16	Sn	Ti	44.583	0.587	2.395	-1.786	-0.58	-2.484	1.853	72.745	11.033	11.923
903	75	10	15	Sn	Ti	27.667	-4.619	1.834	-4.653	5.09	-2.022	5.13	88.898	11.338	11.336
904	75	9	16	Sn	O	44.583	0.587	2.395	-1.786	-0.58	-2.484	1.853	72.745	11.033	11.923
905	75	10	15	Sn	Ti	4.537	-4.619	1.834	-4.653	5.09	-2.022	5.13	88.898	11.338	11.336
906	75	10	15	Sn	Ti	55.463	-4.619	-1.834	-4.653	5.09	2.022	5.131	88.898	11.338	11.336
907	75	9	16	Sn	Ti	32.333	-4.619	-0.408	3.745	5.09	0.379	-3.484	72.756	11.338	11.621
908	75	10	15	Sn	Ti	32.333	-4.619	-1.834	-4.653	5.09	2.022	5.13	88.898	11.338	11.336
909	75	9	16	Sn	O	32.333	-4.619	-0.408	3.745	5.09	0.379	-3.484	72.756	11.338	11.621
910	75	10	15	Sn	O	32.333	-4.619	-1.834	-4.653	5.09	2.022	5.13	88.898	11.338	11.336
911	75	9	16	Sn	Ti	18.435	0.345	2.787	-1.555	-0.343	-2.877	1.605	72.701	11.928	11.031
912	75	9	16	Sn	O	27.667	-4.619	0.408	3.745	5.09	-0.379	-3.484	72.756	11.338	11.621
913	75	9	16	Sn	O	18.435	0.345	2.787	-1.555	-0.343	-2.877	1.605	72.701	11.928	11.031
914	75	10	15	Sn	O	27.667	-4.619	1.834	-4.653	5.09	-2.022	5.13	88.898	11.338	11.336
915	75	9	16	Sn	Ti	13.898	-2.165	1.254	0.987	2.263	-1.23	-0.967	72.75	11.033	11.924
916	75	10	15	Sn	O	55.463	-4.619	-1.834	-4.653	5.09	2.022	5.131	88.898	11.338	11.336
917	75	9	16	Sn	O	27.667	-4.619	0.408	3.745	5.09	-0.379	-3.484	72.756	11.338	11.621
918	75	10	15	Sn	O	27.667	-4.619	1.834	-4.653	5.09	-2.022	5.13	88.898	11.338	11.336
919	75	10	15	Sn	O	55.463	-4.619	-1.834	-4.653	5.09	2.022	5.131	88.898	11.338	11.336
920	75	9	16	Sn	Ti	46.102	-2.165	-1.254	0.987	2.263	1.23	-0.967	72.75	11.033	11.924
921	78	9	17	S	Ti	41.848	4.862	0.613	-2.766	-4.431	-0.649	2.927	70.548	12.465	11.032
922	78	9	17	S	Ti	13.898	-2.165	4.39	4.173	2.263	-4.052	-3.852	70.445	11.033	12.467

923	78	9	17	S	Ti	46.102	-2.165	-4.39	4.173	2.263	4.052	-3.852	70.445	11.033	12.467
924	78	9	17	S	O	13.898	-2.165	4.39	4.173	2.263	-4.052	-3.852	70.445	11.033	12.467
925	78	9	17	S	O	46.102	-2.165	-4.39	4.173	2.263	4.052	-3.852	70.445	11.033	12.467
926	78	9	17	S	O	13.898	-2.165	4.39	4.173	2.263	-4.052	-3.852	70.445	11.033	12.467
927	78	9	17	S	O	46.102	-2.165	-4.39	4.173	2.263	4.052	-3.852	70.445	11.033	12.467
928	78	9	17	S	Ti	18.152	4.862	-0.613	-2.766	-4.431	0.649	2.927	70.548	12.465	11.032
929	78	9	17	S	O	18.152	4.862	-0.613	-2.766	-4.431	0.649	2.927	70.548	12.465	11.032
930	78	9	17	S	O	18.152	4.862	-0.613	-2.766	-4.431	0.649	2.927	70.548	12.465	11.032
931	78	9	17	S	Ti	11.848	4.862	0.613	-2.766	-4.431	-0.649	2.927	70.548	12.465	11.032
932	78	9	17	S	O	11.848	4.862	0.613	-2.766	-4.431	-0.649	2.927	70.548	12.465	11.032
933	78	9	17	S	O	11.848	4.862	0.613	-2.766	-4.431	-0.649	2.927	70.548	12.465	11.032
934	78	9	17	S	Ti	43.898	-2.165	4.39	4.173	2.263	-4.052	-3.852	70.445	11.033	12.466
935	78	9	17	S	Ti	16.102	-2.165	-4.39	4.173	2.263	4.052	-3.852	70.445	11.033	12.466
936	78	9	17	S	O	43.898	-2.165	4.39	4.173	2.263	-4.052	-3.852	70.445	11.033	12.466
937	78	9	17	S	O	16.102	-2.165	-4.39	4.173	2.263	4.052	-3.852	70.445	11.033	12.466
938	78	9	17	S	Ti	48.152	4.862	-0.613	-2.766	-4.431	0.649	2.927	70.548	12.465	11.032
939	78	9	17	S	O	48.152	4.862	-0.613	-2.766	-4.431	0.649	2.927	70.548	12.465	11.032
940	78	9	17	S	Ti	13.898	-2.165	4.39	4.173	2.263	-4.052	-3.852	70.445	11.033	12.467
941	78	9	17	S	Ti	46.102	-2.165	-4.39	4.173	2.263	4.052	-3.852	70.445	11.033	12.467
942	78	9	17	S	O	13.898	-2.165	4.39	4.173	2.263	-4.052	-3.852	70.445	11.033	12.467
943	78	9	17	S	O	46.102	-2.165	-4.39	4.173	2.263	4.052	-3.852	70.445	11.033	12.467
944	78	9	17	S	O	13.898	-2.165	4.39	4.173	2.263	-4.052	-3.852	70.445	11.033	12.467
945	78	9	17	S	O	46.102	-2.165	-4.39	4.173	2.263	4.052	-3.852	70.445	11.033	12.467
946	78	9	17	S	Ti	18.152	4.862	-0.613	-2.766	-4.431	0.649	2.927	70.548	12.465	11.032
947	78	9	17	S	O	18.152	4.862	-0.613	-2.766	-4.431	0.649	2.927	70.548	12.465	11.032
948	78	9	17	S	O	18.152	4.862	-0.613	-2.766	-4.431	0.649	2.927	70.548	12.465	11.032
949	78	9	17	S	Ti	11.848	4.862	0.613	-2.766	-4.431	-0.649	2.927	70.548	12.465	11.032
950	78	9	17	S	O	11.848	4.862	0.613	-2.766	-4.431	-0.649	2.927	70.548	12.465	11.032
951	78	9	17	S	O	11.848	4.862	0.613	-2.766	-4.431	-0.649	2.927	70.548	12.465	11.032
952	78	9	17	S	Ti	43.898	-2.165	4.39	4.173	2.263	-4.052	-3.852	70.445	11.033	12.466
953	78	9	17	S	Ti	16.102	-2.165	-4.39	4.173	2.263	4.052	-3.852	70.445	11.033	12.466
954	78	9	17	S	O	43.898	-2.165	4.39	4.173	2.263	-4.052	-3.852	70.445	11.033	12.466
955	78	9	17	S	O	16.102	-2.165	-4.39	4.173	2.263	4.052	-3.852	70.445	11.033	12.466
956	78	9	17	S	Ti	48.152	4.862	-0.613	-2.766	-4.431	0.649	2.927	70.548	12.465	11.032
957	78	9	17	S	O	48.152	4.862	-0.613	-2.766	-4.431	0.649	2.927	70.548	12.465	11.032
958	78	9	17	S	Ti	41.848	4.862	0.613	-2.766	-4.431	-0.649	2.927	70.548	12.465	11.032
959	78	9	17	Sn	Ti	46.102	-2.165	-4.39	4.173	2.263	4.052	-3.852	70.445	11.033	12.467
960	78	9	17	Sn	O	13.898	-2.165	4.39	4.173	2.263	-4.052	-3.852	70.445	11.033	12.467
961	78	9	17	Sn	O	46.102	-2.165	-4.39	4.173	2.263	4.052	-3.852	70.445	11.033	12.467
962	78	9	17	Sn	O	13.898	-2.165	4.39	4.173	2.263	-4.052	-3.852	70.445	11.033	12.467
963	78	9	17	Sn	O	46.102	-2.165	-4.39	4.173	2.263	4.052	-3.852	70.445	11.033	12.467
964	78	9	17	Sn	Ti	11.848	4.862	0.613	-2.766	-4.431	-0.649	2.927	70.548	12.465	11.032

965	78	9	17	Sn	O	11.848	4.862	0.613	-2.766	-4.431	-0.649	2.927	70.548	12.465	11.032
966	78	9	17	Sn	O	11.848	4.862	0.613	-2.766	-4.431	-0.649	2.927	70.548	12.465	11.032
967	78	9	17	Sn	Ti	43.898	-2.165	4.39	4.173	2.263	-4.052	-3.852	70.445	11.033	12.466
968	78	9	17	Sn	Ti	16.102	-2.165	-4.39	4.173	2.263	4.052	-3.852	70.445	11.033	12.466
969	78	9	17	Sn	O	43.898	-2.165	4.39	4.173	2.263	-4.052	-3.852	70.445	11.033	12.466
970	78	9	17	Sn	O	16.102	-2.165	-4.39	4.173	2.263	4.052	-3.852	70.445	11.033	12.466
971	78	9	17	Sn	Ti	48.152	4.862	-0.613	-2.766	-4.431	0.649	2.927	70.548	12.465	11.032
972	78	9	17	Sn	O	48.152	4.862	-0.613	-2.766	-4.431	0.649	2.927	70.548	12.465	11.032
973	78	9	17	Sn	Ti	41.848	4.862	0.613	-2.766	-4.431	-0.649	2.927	70.548	12.465	11.032
974	78	9	17	Sn	Ti	18.152	4.862	-0.613	-2.766	-4.431	0.649	2.927	70.548	12.465	11.032
975	78	9	17	Sn	O	18.152	4.862	-0.613	-2.766	-4.431	0.649	2.927	70.548	12.465	11.032
976	78	9	17	Sn	Ti	13.898	-2.165	4.39	4.173	2.263	-4.052	-3.852	70.445	11.033	12.467
977	78	9	17	Sn	O	18.152	4.862	-0.613	-2.766	-4.431	0.649	2.927	70.548	12.465	11.032
978	84	10	18	S	Ti	58.898	0.737	-3.875	-1.328	-0.726	3.98	1.365	80.635	10.71	13.29
979	84	10	18	S	O	58.898	0.737	-3.875	-1.328	-0.726	3.98	1.365	80.635	10.71	13.29
980	84	10	18	S	Ti	57.103	-1.882	-3.098	1.321	1.956	3.019	-1.287	80.737	13.298	10.703
981	84	10	18	S	O	57.103	-1.882	-3.098	1.321	1.956	3.019	-1.287	80.737	13.298	10.703
982	84	10	18	S	Ti	28.898	0.737	-3.875	-1.328	-0.726	3.98	1.365	80.635	10.71	13.29
983	84	10	18	S	O	28.898	0.737	-3.875	-1.328	-0.726	3.98	1.365	80.635	10.71	13.29
984	84	10	18	S	O	28.898	0.737	-3.875	-1.328	-0.726	3.98	1.365	80.635	10.71	13.29
985	84	10	18	S	Ti	27.103	-1.882	-3.098	1.321	1.956	3.019	-1.287	80.737	13.298	10.703
986	84	10	18	S	O	27.103	-1.882	-3.098	1.321	1.956	3.019	-1.287	80.737	13.298	10.703
987	84	10	18	S	O	27.103	-1.882	-3.098	1.321	1.956	3.019	-1.287	80.737	13.298	10.703
988	84	10	18	S	Ti	32.897	-1.882	3.098	1.321	1.956	-3.019	-1.287	80.737	13.298	10.703
989	84	10	18	S	O	32.897	-1.882	3.098	1.321	1.956	-3.019	-1.287	80.737	13.298	10.703
990	84	10	18	S	Ti	31.102	0.737	3.875	-1.328	-0.726	-3.98	1.365	80.635	10.71	13.29
991	84	10	18	Sn	Ti	62.897	-1.882	3.098	1.321	1.956	-3.019	-1.287	80.737	13.298	10.703
992	84	10	18	Sn	O	62.897	-1.882	3.098	1.321	1.956	-3.019	-1.287	80.737	13.298	10.703
993	84	10	18	Sn	O	62.897	-1.882	3.098	1.321	1.956	-3.019	-1.287	80.737	13.298	10.703
994	84	10	18	Sn	Ti	28.898	0.737	-3.875	-1.328	-0.726	3.98	1.365	80.635	10.71	13.29
995	84	10	18	Sn	O	28.898	0.737	-3.875	-1.328	-0.726	3.98	1.365	80.635	10.71	13.29
996	84	10	18	Sn	O	28.898	0.737	-3.875	-1.328	-0.726	3.98	1.365	80.635	10.71	13.29
997	84	10	18	Sn	Ti	27.103	-1.882	-3.098	1.321	1.956	3.019	-1.287	80.737	13.298	10.703
998	84	10	18	Sn	O	27.103	-1.882	-3.098	1.321	1.956	3.019	-1.287	80.737	13.298	10.703
999	84	10	18	Sn	O	27.103	-1.882	-3.098	1.321	1.956	3.019	-1.287	80.737	13.298	10.703
1000	84	10	18	Sn	Ti	32.897	-1.882	3.098	1.321	1.956	-3.019	-1.287	80.737	13.298	10.703
1001	84	10	18	Sn	O	32.897	-1.882	3.098	1.321	1.956	-3.019	-1.287	80.737	13.298	10.703
1002	84	10	18	Sn	Ti	31.102	0.737	3.875	-1.328	-0.726	-3.98	1.365	80.635	10.71	13.29
1003	84	10	18	Sn	Ti	58.898	0.737	-3.875	-1.328	-0.726	3.98	1.365	80.635	10.71	13.29
1004	84	10	18	Sn	O	58.898	0.737	-3.875	-1.328	-0.726	3.98	1.365	80.635	10.71	13.29
1005	84	10	18	Sn	Ti	57.103	-1.882	-3.098	1.321	1.956	3.019	-1.287	80.737	13.298	10.703
1006	84	10	18	Sn	O	57.103	-1.882	-3.098	1.321	1.956	3.019	-1.287	80.737	13.298	10.703

1007	87	10	19	S	Ti	37.203	0.587	5.389	1.528	-0.58	-5.229	-1.483	81.746	13.633	10.696
1008	87	10	19	S	O	37.203	0.587	5.389	1.528	-0.58	-5.229	-1.483	81.746	13.633	10.696
1009	87	10	19	S	O	37.203	0.587	5.389	1.528	-0.58	-5.229	-1.483	81.746	13.633	10.696
1010	87	10	19	S	Ti	1.102	0.737	-5.424	1.375	-0.726	5.279	-1.339	81.737	10.71	13.614
1011	87	10	19	S	Ti	28.898	0.737	5.424	1.375	-0.726	-5.279	-1.339	81.737	10.71	13.614
1012	87	10	19	S	O	1.102	0.737	-5.424	1.375	-0.726	5.279	-1.339	81.737	10.71	13.614
1013	87	10	19	S	O	28.898	0.737	5.424	1.375	-0.726	-5.279	-1.339	81.737	10.71	13.614
1014	87	10	19	S	O	1.102	0.737	-5.424	1.375	-0.726	5.279	-1.339	81.737	10.71	13.614
1015	87	10	19	S	O	28.898	0.737	5.424	1.375	-0.726	-5.279	-1.339	81.737	10.71	13.614
1016	87	10	19	S	Ti	52.797	0.587	-5.389	1.528	-0.58	5.229	-1.483	81.746	13.633	10.696
1017	87	10	19	S	O	52.797	0.587	-5.389	1.528	-0.58	5.229	-1.483	81.746	13.633	10.696
1018	87	10	19	S	O	52.797	0.587	-5.389	1.528	-0.58	5.229	-1.483	81.746	13.633	10.696
1019	87	9	20	S	Ti	3.69	5.194	4.675	5.235	-4.706	-4.232	-4.739	71.298	11.628	12.781
1020	87	9	20	S	O	3.69	5.194	4.675	5.235	-4.706	-4.232	-4.739	71.298	11.628	12.781
1021	87	9	20	S	O	3.69	5.194	4.675	5.235	-4.706	-4.232	-4.739	71.298	11.628	12.781
1022	87	10	19	S	Ti	7.203	0.587	5.389	1.528	-0.58	-5.229	-1.483	81.746	13.633	10.696
1023	87	10	19	S	O	7.203	0.587	5.389	1.528	-0.58	-5.229	-1.483	81.746	13.633	10.696
1024	87	9	20	S	Ti	56.31	5.194	-4.675	5.235	-4.706	4.232	-4.739	71.298	12.781	11.628
1025	87	9	20	S	O	56.31	5.194	-4.675	5.235	-4.706	4.232	-4.739	71.298	12.781	11.628
1026	87	10	19	S	Ti	31.102	0.737	-5.424	1.375	-0.726	5.279	-1.339	81.737	10.71	13.614
1027	87	10	19	S	Ti	58.898	0.737	5.424	1.375	-0.726	-5.279	-1.339	81.737	10.71	13.614
1028	87	10	19	S	O	58.898	0.737	5.424	1.375	-0.726	-5.279	-1.339	81.737	10.71	13.614
1029	87	10	19	S	Ti	22.797	0.587	-5.389	1.528	-0.58	5.229	-1.483	81.746	13.633	10.696
1030	87	10	19	S	Ti	37.203	0.587	5.389	1.528	-0.58	-5.229	-1.483	81.746	13.633	10.696
1031	87	10	19	S	O	37.203	0.587	5.389	1.528	-0.58	-5.229	-1.483	81.746	13.633	10.696
1032	87	10	19	S	O	37.203	0.587	5.389	1.528	-0.58	-5.229	-1.483	81.746	13.633	10.696
1033	87	9	20	S	Ti	26.31	5.194	-4.675	5.235	-4.706	4.232	-4.739	71.298	12.781	11.628
1034	87	9	20	S	O	26.31	5.194	-4.675	5.235	-4.706	4.232	-4.739	71.298	12.781	11.628
1035	87	9	20	S	O	26.31	5.194	-4.675	5.235	-4.706	4.232	-4.739	71.298	12.781	11.628
1036	87	10	19	S	Ti	1.102	0.737	-5.424	1.375	-0.726	5.279	-1.339	81.737	10.71	13.614
1037	87	10	19	S	Ti	28.898	0.737	5.424	1.375	-0.726	-5.279	-1.339	81.737	10.71	13.614
1038	87	10	19	S	O	1.102	0.737	-5.424	1.375	-0.726	5.279	-1.339	81.737	10.71	13.614
1039	87	10	19	S	O	28.898	0.737	5.424	1.375	-0.726	-5.279	-1.339	81.737	10.71	13.614
1040	87	10	19	S	O	1.102	0.737	-5.424	1.375	-0.726	5.279	-1.339	81.737	10.71	13.614
1041	87	10	19	S	O	28.898	0.737	5.424	1.375	-0.726	-5.279	-1.339	81.737	10.71	13.614
1042	87	10	19	S	Ti	52.797	0.587	-5.389	1.528	-0.58	5.229	-1.483	81.746	13.633	10.696
1043	87	10	19	S	O	52.797	0.587	-5.389	1.528	-0.58	5.229	-1.483	81.746	13.633	10.696
1044	87	10	19	S	O	52.797	0.587	-5.389	1.528	-0.58	5.229	-1.483	81.746	13.633	10.696
1045	87	10	19	S	Ti	7.203	0.587	5.389	1.528	-0.58	-5.229	-1.483	81.746	13.633	10.696
1046	87	10	19	S	O	7.203	0.587	5.389	1.528	-0.58	-5.229	-1.483	81.746	13.633	10.696
1047	87	10	19	S	Ti	31.102	0.737	-5.424	1.375	-0.726	5.279	-1.339	81.737	10.71	13.614
1048	87	10	19	S	Ti	58.898	0.737	5.424	1.375	-0.726	-5.279	-1.339	81.737	10.71	13.614

1049	87	10	19	S	O	58.898	0.737	5.424	1.375	-0.726	-5.279	-1.339	81.737	10.71	13.614
1050	87	10	19	S	Ti	22.797	0.587	-5.389	1.528	-0.58	5.229	-1.483	81.746	13.633	10.696
1051	87	9	20	S	Ti	33.69	5.194	4.675	5.235	-4.706	-4.232	-4.739	71.298	11.628	12.781
1052	87	9	20	S	O	33.69	5.194	4.675	5.235	-4.706	-4.232	-4.739	71.298	11.628	12.781
1053	87	10	19	Sn	Ti	37.203	0.587	5.389	1.528	-0.58	-5.229	-1.483	81.746	13.633	10.696
1054	87	10	19	Sn	O	37.203	0.587	5.389	1.528	-0.58	-5.229	-1.483	81.746	13.633	10.696
1055	87	10	19	Sn	O	37.203	0.587	5.389	1.528	-0.58	-5.229	-1.483	81.746	13.633	10.696
1056	87	9	20	Sn	Ti	26.31	5.194	-4.675	5.235	-4.706	4.232	-4.739	71.298	12.781	11.628
1057	87	9	20	Sn	O	26.31	5.194	-4.675	5.235	-4.706	4.232	-4.739	71.298	12.781	11.628
1058	87	9	20	Sn	O	26.31	5.194	-4.675	5.235	-4.706	4.232	-4.739	71.298	12.781	11.628
1059	87	10	19	Sn	Ti	1.102	0.737	-5.424	1.375	-0.726	5.279	-1.339	81.737	10.71	13.614
1060	87	10	19	Sn	Ti	28.898	0.737	5.424	1.375	-0.726	-5.279	-1.339	81.737	10.71	13.614
1061	87	10	19	Sn	O	1.102	0.737	-5.424	1.375	-0.726	5.279	-1.339	81.737	10.71	13.614
1062	87	10	19	Sn	O	28.898	0.737	5.424	1.375	-0.726	-5.279	-1.339	81.737	10.71	13.614
1063	87	10	19	Sn	O	1.102	0.737	-5.424	1.375	-0.726	5.279	-1.339	81.737	10.71	13.614
1064	87	10	19	Sn	O	28.898	0.737	5.424	1.375	-0.726	-5.279	-1.339	81.737	10.71	13.614
1065	87	10	19	Sn	Ti	52.797	0.587	-5.389	1.528	-0.58	5.229	-1.483	81.746	13.633	10.696
1066	87	10	19	Sn	O	52.797	0.587	-5.389	1.528	-0.58	5.229	-1.483	81.746	13.633	10.696
1067	87	10	19	Sn	O	52.797	0.587	-5.389	1.528	-0.58	5.229	-1.483	81.746	13.633	10.696
1068	87	9	20	Sn	Ti	3.69	5.194	4.675	5.235	-4.706	-4.232	-4.739	71.298	11.628	12.781
1069	87	9	20	Sn	O	3.69	5.194	4.675	5.235	-4.706	-4.232	-4.739	71.298	11.628	12.781
1070	87	9	20	Sn	O	3.69	5.194	4.675	5.235	-4.706	-4.232	-4.739	71.298	11.628	12.781
1071	87	10	19	Sn	Ti	7.203	0.587	5.389	1.528	-0.58	-5.229	-1.483	81.746	13.633	10.696
1072	87	10	19	Sn	O	7.203	0.587	5.389	1.528	-0.58	-5.229	-1.483	81.746	13.633	10.696
1073	87	9	20	Sn	Ti	56.31	5.194	-4.675	5.235	-4.706	4.232	-4.739	71.298	12.781	11.628
1074	87	9	20	Sn	O	56.31	5.194	-4.675	5.235	-4.706	4.232	-4.739	71.298	12.781	11.628
1075	87	10	19	Sn	Ti	31.102	0.737	-5.424	1.375	-0.726	5.279	-1.339	81.737	10.71	13.614
1076	87	10	19	Sn	Ti	58.898	0.737	5.424	1.375	-0.726	-5.279	-1.339	81.737	10.71	13.614
1077	87	10	19	Sn	O	58.898	0.737	5.424	1.375	-0.726	-5.279	-1.339	81.737	10.71	13.614
1078	87	10	19	Sn	Ti	22.797	0.587	-5.389	1.528	-0.58	5.229	-1.483	81.746	13.633	10.696
1079	87	9	20	Sn	Ti	33.69	5.194	4.675	5.235	-4.706	-4.232	-4.739	71.298	11.628	12.781
1080	87	9	20	Sn	O	33.69	5.194	4.675	5.235	-4.706	-4.232	-4.739	71.298	11.628	12.781
1081	90	10	20	S	Ti	26.31	5.194	-3.156	-0.288	-4.706	3.174	0.29	76.361	14.257	10.697
1082	90	10	20	S	O	26.31	5.194	-3.156	-0.288	-4.706	3.174	0.29	76.361	14.257	10.697
1083	90	10	20	S	O	26.31	5.194	-3.156	-0.288	-4.706	3.174	0.29	76.361	14.257	10.697
1084	90	10	20	S	Ti	28.898	0.737	-5.424	4.079	-0.726	5.015	-3.772	76.196	10.71	14.243
1085	90	10	20	S	O	28.898	0.737	-5.424	4.079	-0.726	5.015	-3.772	76.196	10.71	14.243
1086	90	10	20	S	O	28.898	0.737	-5.424	4.079	-0.726	5.015	-3.772	76.196	10.71	14.243
1087	90	10	20	S	Ti	31.102	0.737	5.424	4.079	-0.726	-5.015	-3.772	76.196	10.71	14.243
1088	90	10	20	S	Ti	33.69	5.194	3.156	-0.288	-4.706	-3.174	0.29	76.361	14.257	10.697
1089	90	10	20	S	O	33.69	5.194	3.156	-0.288	-4.706	-3.174	0.29	76.361	14.257	10.697
1090	90	10	20	S	Ti	56.31	5.194	-3.156	-0.288	-4.706	3.174	0.29	76.361	14.257	10.697

1091	90	10	20	S	O	56.31	5.194	-3.156	-0.288	-4.706	3.174	0.29	76.361	14.257	10.697
1092	90	10	20	S	Ti	58.898	0.737	-5.424	4.079	-0.726	5.015	-3.772	76.196	10.71	14.243
1093	90	10	20	S	O	58.898	0.737	-5.424	4.079	-0.726	5.015	-3.772	76.196	10.71	14.243
1094	90	10	20	Sn	Ti	26.31	5.194	-3.156	-0.288	-4.706	3.174	0.29	76.361	14.257	10.697
1095	90	10	20	Sn	O	26.31	5.194	-3.156	-0.288	-4.706	3.174	0.29	76.361	14.257	10.697
1096	90	10	20	Sn	O	26.31	5.194	-3.156	-0.288	-4.706	3.174	0.29	76.361	14.257	10.697
1097	90	10	20	Sn	Ti	28.898	0.737	-5.424	4.079	-0.726	5.015	-3.772	76.196	10.71	14.243
1098	90	10	20	Sn	O	28.898	0.737	-5.424	4.079	-0.726	5.015	-3.772	76.196	10.71	14.243
1099	90	10	20	Sn	O	28.898	0.737	-5.424	4.079	-0.726	5.015	-3.772	76.196	10.71	14.243
1100	90	10	20	Sn	Ti	56.31	5.194	-3.156	-0.288	-4.706	3.174	0.29	76.361	14.257	10.697
1101	90	10	20	Sn	O	56.31	5.194	-3.156	-0.288	-4.706	3.174	0.29	76.361	14.257	10.697
1102	90	10	20	Sn	Ti	58.898	0.737	-5.424	4.079	-0.726	5.015	-3.772	76.196	10.71	14.243
1103	90	10	20	Sn	O	58.898	0.737	-5.424	4.079	-0.726	5.015	-3.772	76.196	10.71	14.243
1104	93	11	20	S	Ti	4.537	-4.619	3.669	4.966	5.09	-3.337	-4.517	74.211	11.338	14.263
1105	93	11	20	S	Ti	18.435	0.345	3.421	-0.454	-0.343	-3.452	0.458	72.814	11.928	13.624
1106	93	11	20	S	O	18.435	0.345	3.421	-0.454	-0.343	-3.452	0.458	72.814	11.928	13.624
1107	93	11	20	S	Ti	55.463	-4.619	-3.669	4.966	5.09	3.337	-4.517	74.211	11.338	14.263
1108	93	11	20	S	O	55.463	-4.619	-3.669	4.966	5.09	3.337	-4.517	74.211	11.338	14.263
1109	93	11	20	S	O	55.463	-4.619	-3.669	4.966	5.09	3.337	-4.517	74.211	11.338	14.263
1110	93	11	20	S	Ti	41.565	0.345	-3.421	-0.454	-0.343	3.452	0.458	72.814	11.928	13.624
1111	93	11	20	S	O	41.565	0.345	-3.421	-0.454	-0.343	3.452	0.458	72.814	11.928	13.624
1112	93	11	20	S	O	41.565	0.345	-3.421	-0.454	-0.343	3.452	0.458	72.814	11.928	13.624
1113	93	11	20	S	Ti	15.417	0.587	3.266	-0.691	-0.58	-3.312	0.7	72.823	13.633	11.92
1114	93	11	20	S	O	15.417	0.587	3.266	-0.691	-0.58	-3.312	0.7	72.823	13.633	11.92
1115	93	11	20	S	O	15.417	0.587	3.266	-0.691	-0.58	-3.312	0.7	72.823	13.633	11.92
1116	93	11	20	S	Ti	14.583	0.587	-3.266	-0.691	-0.58	3.311	0.7	72.823	13.633	11.92
1117	93	11	20	S	O	14.583	0.587	-3.266	-0.691	-0.58	3.311	0.7	72.823	13.633	11.92
1118	93	11	20	S	O	14.583	0.587	-3.266	-0.691	-0.58	3.311	0.7	72.823	13.633	11.92
1119	93	11	20	S	Ti	3.69	5.194	1.913	-4.807	-4.706	-2.116	5.319	74.289	14.257	11.34
1120	93	11	20	S	O	3.69	5.194	1.913	-4.807	-4.706	-2.116	5.319	74.289	14.257	11.34
1121	93	11	20	S	O	3.69	5.194	1.913	-4.807	-4.706	-2.116	5.319	74.289	14.257	11.34
1122	93	11	20	S	Ti	48.435	0.345	3.421	-0.454	-0.343	-3.452	0.458	72.814	11.928	13.624
1123	93	11	20	S	O	48.435	0.345	3.421	-0.454	-0.343	-3.452	0.458	72.814	11.928	13.624
1124	93	11	20	S	O	48.435	0.345	3.421	-0.454	-0.343	-3.452	0.458	72.814	11.928	13.624
1125	93	11	20	S	Ti	11.565	0.345	-3.421	-0.454	-0.343	3.452	0.458	72.814	11.928	13.624
1126	93	11	20	S	O	11.565	0.345	-3.421	-0.454	-0.343	3.452	0.458	72.814	11.928	13.624
1127	93	11	20	S	O	11.565	0.345	-3.421	-0.454	-0.343	3.452	0.458	72.814	11.928	13.624
1128	93	11	20	S	Ti	45.417	0.587	3.266	-0.691	-0.58	-3.311	0.7	72.823	13.633	11.92
1129	93	11	20	S	O	45.417	0.587	3.266	-0.691	-0.58	-3.311	0.7	72.823	13.633	11.92
1130	93	11	20	S	Ti	56.31	5.194	-1.913	-4.807	-4.706	2.116	5.319	74.289	14.257	11.34
1131	93	11	20	S	O	56.31	5.194	-1.913	-4.807	-4.706	2.116	5.319	74.289	14.257	11.34
1132	93	11	20	S	Ti	44.583	0.587	-3.266	-0.691	-0.58	3.312	0.7	72.823	13.633	11.92

1133	93	11	20	S	O	44.583	0.587	-3.266	-0.691	-0.58	3.312	0.7	72.823	13.633	11.92
1134	93	11	20	S	Ti	26.31	5.194	-1.913	-4.807	-4.706	2.116	5.319	74.289	14.257	11.34
1135	93	11	20	S	O	26.31	5.194	-1.913	-4.807	-4.706	2.116	5.319	74.289	14.257	11.34
1136	93	11	20	S	O	26.31	5.194	-1.913	-4.807	-4.706	2.116	5.319	74.289	14.257	11.34
1137	93	11	20	S	Ti	34.537	-4.619	3.669	4.966	5.09	-3.337	-4.517	74.211	11.338	14.263
1138	93	11	20	S	O	34.537	-4.619	3.669	4.966	5.09	-3.337	-4.517	74.211	11.338	14.263
1139	93	11	20	S	O	34.537	-4.619	3.669	4.966	5.09	-3.337	-4.517	74.211	11.338	14.263
1140	93	11	20	S	Ti	25.463	-4.619	-3.669	4.966	5.09	3.337	-4.517	74.211	11.338	14.263
1141	93	11	20	S	O	25.463	-4.619	-3.669	4.966	5.09	3.337	-4.517	74.211	11.338	14.263
1142	93	11	20	S	Ti	33.69	5.194	1.913	-4.807	-4.706	-2.116	5.319	74.289	14.257	11.34
1143	93	11	20	S	O	33.69	5.194	1.913	-4.807	-4.706	-2.116	5.319	74.289	14.257	11.34
1144	93	11	20	Sn	Ti	15.417	0.587	3.266	-0.691	-0.58	-3.312	0.7	72.823	13.633	11.92
1145	93	11	20	Sn	O	15.417	0.587	3.266	-0.691	-0.58	-3.312	0.7	72.823	13.633	11.92
1146	93	11	20	Sn	O	15.417	0.587	3.266	-0.691	-0.58	-3.312	0.7	72.823	13.633	11.92
1147	93	11	20	Sn	Ti	26.31	5.194	-1.913	-4.807	-4.706	2.116	5.319	74.289	14.257	11.34
1148	93	11	20	Sn	O	26.31	5.194	-1.913	-4.807	-4.706	2.116	5.319	74.289	14.257	11.34
1149	93	11	20	Sn	O	26.31	5.194	-1.913	-4.807	-4.706	2.116	5.319	74.289	14.257	11.34
1150	93	11	20	Sn	Ti	14.583	0.587	-3.266	-0.691	-0.58	3.311	0.7	72.823	13.633	11.92
1151	93	11	20	Sn	O	14.583	0.587	-3.266	-0.691	-0.58	3.311	0.7	72.823	13.633	11.92
1152	93	11	20	Sn	O	14.583	0.587	-3.266	-0.691	-0.58	3.311	0.7	72.823	13.633	11.92
1153	93	11	20	Sn	Ti	3.69	5.194	1.913	-4.807	-4.706	-2.116	5.319	74.289	14.257	11.34
1154	93	11	20	Sn	O	3.69	5.194	1.913	-4.807	-4.706	-2.116	5.319	74.289	14.257	11.34
1155	93	11	20	Sn	O	3.69	5.194	1.913	-4.807	-4.706	-2.116	5.319	74.289	14.257	11.34
1156	93	11	20	Sn	Ti	34.537	-4.619	3.669	4.966	5.09	-3.337	-4.517	74.211	11.338	14.263
1157	93	11	20	Sn	O	34.537	-4.619	3.669	4.966	5.09	-3.337	-4.517	74.211	11.338	14.263
1158	93	11	20	Sn	O	34.537	-4.619	3.669	4.966	5.09	-3.337	-4.517	74.211	11.338	14.263
1159	93	11	20	Sn	Ti	48.435	0.345	3.421	-0.454	-0.343	-3.452	0.458	72.814	11.928	13.624
1160	93	11	20	Sn	O	48.435	0.345	3.421	-0.454	-0.343	-3.452	0.458	72.814	11.928	13.624
1161	93	11	20	Sn	O	48.435	0.345	3.421	-0.454	-0.343	-3.452	0.458	72.814	11.928	13.624
1162	93	11	20	Sn	Ti	25.463	-4.619	-3.669	4.966	5.09	3.337	-4.517	74.211	11.338	14.263
1163	93	11	20	Sn	O	25.463	-4.619	-3.669	4.966	5.09	3.337	-4.517	74.211	11.338	14.263
1164	93	11	20	Sn	Ti	11.565	0.345	-3.421	-0.454	-0.343	3.452	0.458	72.814	11.928	13.624
1165	93	11	20	Sn	O	11.565	0.345	-3.421	-0.454	-0.343	3.452	0.458	72.814	11.928	13.624
1166	93	11	20	Sn	O	11.565	0.345	-3.421	-0.454	-0.343	3.452	0.458	72.814	11.928	13.624
1167	93	11	20	Sn	Ti	45.417	0.587	3.266	-0.691	-0.58	-3.311	0.7	72.823	13.633	11.92
1168	93	11	20	Sn	O	45.417	0.587	3.266	-0.691	-0.58	-3.311	0.7	72.823	13.633	11.92
1169	93	11	20	Sn	Ti	56.31	5.194	-1.913	-4.807	-4.706	2.116	5.319	74.289	14.257	11.34
1170	93	11	20	Sn	O	56.31	5.194	-1.913	-4.807	-4.706	2.116	5.319	74.289	14.257	11.34
1171	93	11	20	Sn	Ti	44.583	0.587	-3.266	-0.691	-0.58	3.312	0.7	72.823	13.633	11.92
1172	93	11	20	Sn	O	44.583	0.587	-3.266	-0.691	-0.58	3.312	0.7	72.823	13.633	11.92
1173	93	11	20	Sn	Ti	33.69	5.194	1.913	-4.807	-4.706	-2.116	5.319	74.289	14.257	11.34
1174	93	11	20	Sn	O	33.69	5.194	1.913	-4.807	-4.706	-2.116	5.319	74.289	14.257	11.34

1175	93	11	20	Sn	Ti	55.463	-4.619	-3.669	4.966	5.09	3.337	-4.517	74.211	11.338	14.263
1176	93	11	20	Sn	Ti	4.537	-4.619	3.669	4.966	5.09	-3.337	-4.517	74.211	11.338	14.263
1177	93	11	20	Sn	Ti	18.435	0.345	3.421	-0.454	-0.343	-3.452	0.458	72.814	11.928	13.624
1178	93	11	20	Sn	O	18.435	0.345	3.421	-0.454	-0.343	-3.452	0.458	72.814	11.928	13.624
1179	93	11	20	Sn	O	55.463	-4.619	-3.669	4.966	5.09	3.337	-4.517	74.211	11.338	14.263
1180	93	11	20	Sn	O	55.463	-4.619	-3.669	4.966	5.09	3.337	-4.517	74.211	11.338	14.263
1181	93	11	20	Sn	Ti	41.565	0.345	-3.421	-0.454	-0.343	3.452	0.458	72.814	11.928	13.624
1182	93	11	20	Sn	O	41.565	0.345	-3.421	-0.454	-0.343	3.452	0.458	72.814	11.928	13.624
1183	93	11	20	Sn	O	41.565	0.345	-3.421	-0.454	-0.343	3.452	0.458	72.814	11.928	13.624
1184	96	11	21	S	Ti	41.848	4.862	-0.502	-2.26	-4.431	0.526	2.367	73.962	12.465	13.298
1185	96	11	21	S	Ti	18.152	4.862	0.502	-2.26	-4.431	-0.526	2.367	73.962	12.465	13.298
1186	96	11	21	S	O	18.152	4.862	0.502	-2.26	-4.431	-0.526	2.367	73.962	12.465	13.298
1187	96	11	21	S	O	18.152	4.862	0.502	-2.26	-4.431	-0.526	2.367	73.962	12.465	13.298
1188	96	11	21	S	Ti	49.723	-1.882	3.404	4.432	1.956	-3.127	-4.071	73.89	13.298	12.466
1189	96	11	21	S	O	49.723	-1.882	3.404	4.432	1.956	-3.127	-4.071	73.89	13.298	12.466
1190	96	11	21	S	O	49.723	-1.882	3.404	4.432	1.956	-3.127	-4.071	73.89	13.298	12.466
1191	96	11	21	S	Ti	40.277	-1.882	-3.404	4.432	1.956	3.127	-4.071	73.89	13.298	12.466
1192	96	11	21	S	O	40.277	-1.882	-3.404	4.432	1.956	3.127	-4.071	73.89	13.298	12.466
1193	96	11	21	S	O	40.277	-1.882	-3.404	4.432	1.956	3.127	-4.071	73.89	13.298	12.466
1194	96	11	21	S	Ti	11.848	4.862	-0.502	-2.26	-4.431	0.526	2.367	73.962	12.465	13.298
1195	96	11	21	S	O	11.848	4.862	-0.502	-2.26	-4.431	0.526	2.367	73.962	12.465	13.298
1196	96	11	21	S	O	11.848	4.862	-0.502	-2.26	-4.431	0.526	2.367	73.962	12.465	13.298
1197	96	11	21	S	Ti	48.152	4.862	0.502	-2.26	-4.431	-0.526	2.367	73.962	12.465	13.298
1198	96	11	21	S	O	48.152	4.862	0.502	-2.26	-4.431	-0.526	2.367	73.962	12.465	13.298
1199	96	11	21	S	Ti	19.723	-1.882	3.404	4.432	1.956	-3.127	-4.071	73.89	13.298	12.466
1200	96	11	21	S	O	19.723	-1.882	3.404	4.432	1.956	-3.127	-4.071	73.89	13.298	12.466
1201	96	11	21	S	Ti	10.277	-1.882	-3.404	4.432	1.956	3.127	-4.071	73.89	13.298	12.466
1202	96	11	21	S	Ti	18.152	4.862	0.502	-2.26	-4.431	-0.526	2.367	73.962	12.465	13.298
1203	96	11	21	S	O	18.152	4.862	0.502	-2.26	-4.431	-0.526	2.367	73.962	12.465	13.298
1204	96	11	21	S	O	18.152	4.862	0.502	-2.26	-4.431	-0.526	2.367	73.962	12.465	13.298
1205	96	11	21	S	Ti	49.723	-1.882	3.404	4.432	1.956	-3.127	-4.071	73.89	13.298	12.466
1206	96	11	21	S	O	49.723	-1.882	3.404	4.432	1.956	-3.127	-4.071	73.89	13.298	12.466
1207	96	11	21	S	O	49.723	-1.882	3.404	4.432	1.956	-3.127	-4.071	73.89	13.298	12.466
1208	96	11	21	S	Ti	40.277	-1.882	-3.404	4.432	1.956	3.127	-4.071	73.89	13.298	12.466
1209	96	11	21	S	O	40.277	-1.882	-3.404	4.432	1.956	3.127	-4.071	73.89	13.298	12.466
1210	96	11	21	S	O	40.277	-1.882	-3.404	4.432	1.956	3.127	-4.071	73.89	13.298	12.466
1211	96	11	21	S	Ti	11.848	4.862	-0.502	-2.26	-4.431	0.526	2.367	73.962	12.465	13.298
1212	96	11	21	S	O	11.848	4.862	-0.502	-2.26	-4.431	0.526	2.367	73.962	12.465	13.298
1213	96	11	21	S	O	11.848	4.862	-0.502	-2.26	-4.431	0.526	2.367	73.962	12.465	13.298
1214	96	11	21	S	Ti	48.152	4.862	0.502	-2.26	-4.431	-0.526	2.367	73.962	12.465	13.298
1215	96	11	21	S	O	48.152	4.862	0.502	-2.26	-4.431	-0.526	2.367	73.962	12.465	13.298
1216	96	11	21	S	Ti	19.723	-1.882	3.404	4.432	1.956	-3.127	-4.071	73.89	13.298	12.466

1217	96	11	21	S	O	19.723	-1.882	3.404	4.432	1.956	-3.127	-4.071	73.89	13.298	12.466
1218	96	11	21	S	Ti	10.277	-1.882	-3.404	4.432	1.956	3.127	-4.071	73.89	13.298	12.466
1219	96	11	21	S	Ti	41.848	4.862	-0.502	-2.26	-4.431	0.526	2.367	73.962	12.465	13.298
1220	96	11	21	Sn	Ti	49.723	-1.882	3.404	4.432	1.956	-3.127	-4.071	73.89	13.298	12.466
1221	96	11	21	Sn	O	49.723	-1.882	3.404	4.432	1.956	-3.127	-4.071	73.89	13.298	12.466
1222	96	11	21	Sn	O	49.723	-1.882	3.404	4.432	1.956	-3.127	-4.071	73.89	13.298	12.466
1223	96	11	21	Sn	Ti	40.277	-1.882	-3.404	4.432	1.956	3.127	-4.071	73.89	13.298	12.466
1224	96	11	21	Sn	O	40.277	-1.882	-3.404	4.432	1.956	3.127	-4.071	73.89	13.298	12.466
1225	96	11	21	Sn	O	40.277	-1.882	-3.404	4.432	1.956	3.127	-4.071	73.89	13.298	12.466
1226	96	11	21	Sn	Ti	11.848	4.862	-0.502	-2.26	-4.431	0.526	2.367	73.962	12.465	13.298
1227	96	11	21	Sn	O	11.848	4.862	-0.502	-2.26	-4.431	0.526	2.367	73.962	12.465	13.298
1228	96	11	21	Sn	O	11.848	4.862	-0.502	-2.26	-4.431	0.526	2.367	73.962	12.465	13.298
1229	96	11	21	Sn	Ti	48.152	4.862	0.502	-2.26	-4.431	-0.526	2.367	73.962	12.465	13.298
1230	96	11	21	Sn	O	48.152	4.862	0.502	-2.26	-4.431	-0.526	2.367	73.962	12.465	13.298
1231	96	11	21	Sn	Ti	19.723	-1.882	3.404	4.432	1.956	-3.127	-4.071	73.89	13.298	12.466
1232	96	11	21	Sn	O	19.723	-1.882	3.404	4.432	1.956	-3.127	-4.071	73.89	13.298	12.466
1233	96	11	21	Sn	Ti	10.277	-1.882	-3.404	4.432	1.956	3.127	-4.071	73.89	13.298	12.466
1234	96	11	21	Sn	Ti	41.848	4.862	-0.502	-2.26	-4.431	0.526	2.367	73.962	12.465	13.298
1235	96	11	21	Sn	Ti	18.152	4.862	0.502	-2.26	-4.431	-0.526	2.367	73.962	12.465	13.298
1236	96	11	21	Sn	O	18.152	4.862	0.502	-2.26	-4.431	-0.526	2.367	73.962	12.465	13.298
1237	96	11	21	Sn	O	18.152	4.862	0.502	-2.26	-4.431	-0.526	2.367	73.962	12.465	13.298
1238	99	11	22	S	Ti	55.022	4.862	0.753	0.013	-4.431	-0.752	-0.013	73.644	12.465	13.629
1239	99	11	22	S	O	55.022	4.862	0.753	0.013	-4.431	-0.752	-0.013	73.644	12.465	13.629
1240	99	11	22	S	Ti	4.978	4.862	-0.753	0.013	-4.431	0.752	-0.013	73.644	12.465	13.629
1241	99	11	22	S	O	4.978	4.862	-0.753	0.013	-4.431	0.752	-0.013	73.644	12.465	13.629
1242	99	11	22	S	O	4.978	4.862	-0.753	0.013	-4.431	0.752	-0.013	73.644	12.465	13.629
1243	99	12	21	S	Ti	27.103	-1.882	-5.056	-0.104	1.956	5.067	0.105	70.271	13.298	13.277
1244	99	12	21	S	O	27.103	-1.882	-5.056	-0.104	1.956	5.067	0.105	70.271	13.298	13.277
1245	99	12	21	S	O	27.103	-1.882	-5.056	-0.104	1.956	5.067	0.105	70.271	13.298	13.277
1246	99	11	22	S	Ti	52.797	0.587	-3.266	4.24	-0.58	3.01	-3.909	73.59	13.633	12.462
1247	99	11	22	S	O	52.797	0.587	-3.266	4.24	-0.58	3.01	-3.909	73.59	13.633	12.462
1248	99	11	22	S	O	52.797	0.587	-3.266	4.24	-0.58	3.01	-3.909	73.59	13.633	12.462
1249	99	12	21	S	Ti	32.897	-1.882	5.056	-0.104	1.956	-5.067	0.105	70.271	13.298	13.277
1250	99	12	21	S	O	32.897	-1.882	5.056	-0.104	1.956	-5.067	0.105	70.271	13.298	13.277
1251	99	11	22	S	Ti	7.203	0.587	3.266	4.24	-0.58	-3.01	-3.909	73.59	13.633	12.462
1252	99	11	22	S	O	7.203	0.587	3.266	4.24	-0.58	-3.01	-3.909	73.59	13.633	12.462
1253	99	12	21	S	Ti	57.103	-1.882	-5.056	-0.104	1.956	5.067	0.105	70.271	13.298	13.277
1254	99	12	21	S	O	57.103	-1.882	-5.056	-0.104	1.956	5.067	0.105	70.271	13.298	13.277
1255	99	11	22	S	Ti	37.203	0.587	3.266	4.24	-0.58	-3.01	-3.909	73.59	13.633	12.462
1256	99	11	22	S	O	37.203	0.587	3.266	4.24	-0.58	-3.01	-3.909	73.59	13.633	12.462
1257	99	12	21	S	Ti	27.103	-1.882	-5.056	-0.104	1.956	5.067	0.105	70.271	13.298	13.277
1258	99	12	21	S	O	27.103	-1.882	-5.056	-0.104	1.956	5.067	0.105	70.271	13.298	13.277

1259	99	12	21	S	O	27.103	-1.882	-5.056	-0.104	1.956	5.067	0.105	70.271	13.298	13.277
1260	99	11	22	S	Ti	25.022	4.862	0.753	0.013	-4.431	-0.752	-0.013	73.644	12.465	13.629
1261	99	11	22	S	O	25.022	4.862	0.753	0.013	-4.431	-0.752	-0.013	73.644	12.465	13.629
1262	99	11	22	S	O	25.022	4.862	0.753	0.013	-4.431	-0.752	-0.013	73.644	12.465	13.629
1263	99	11	22	S	Ti	34.978	4.862	-0.753	0.013	-4.431	0.752	-0.013	73.644	12.465	13.629
1264	99	12	21	S	Ti	57.103	-1.882	-5.056	-0.104	1.956	5.067	0.105	70.271	13.298	13.277
1265	99	12	21	S	O	57.103	-1.882	-5.056	-0.104	1.956	5.067	0.105	70.271	13.298	13.277
1266	99	11	22	S	Ti	22.797	0.587	-3.266	4.24	-0.58	3.01	-3.909	73.59	13.633	12.462
1267	99	11	22	Sn	Ti	37.203	0.587	3.266	4.24	-0.58	-3.01	-3.909	73.59	13.633	12.462
1268	99	12	21	Sn	O	62.897	-1.882	5.056	-0.104	1.956	-5.067	0.105	70.271	13.298	13.277
1269	99	11	22	Sn	O	37.203	0.587	3.266	4.24	-0.58	-3.01	-3.909	73.59	13.633	12.462
1270	99	11	22	Sn	O	37.203	0.587	3.266	4.24	-0.58	-3.01	-3.909	73.59	13.633	12.462
1271	99	12	21	Sn	Ti	27.103	-1.882	-5.056	-0.104	1.956	5.067	0.105	70.271	13.298	13.277
1272	99	12	21	Sn	O	27.103	-1.882	-5.056	-0.104	1.956	5.067	0.105	70.271	13.298	13.277
1273	99	12	21	Sn	O	27.103	-1.882	-5.056	-0.104	1.956	5.067	0.105	70.271	13.298	13.277
1274	99	11	22	Sn	Ti	52.797	0.587	-3.266	4.24	-0.58	3.01	-3.909	73.59	13.633	12.462
1275	99	11	22	Sn	O	52.797	0.587	-3.266	4.24	-0.58	3.01	-3.909	73.59	13.633	12.462
1276	99	11	22	Sn	O	52.797	0.587	-3.266	4.24	-0.58	3.01	-3.909	73.59	13.633	12.462
1277	99	11	22	Sn	Ti	25.022	4.862	0.753	0.013	-4.431	-0.752	-0.013	73.644	12.465	13.629
1278	99	11	22	Sn	O	25.022	4.862	0.753	0.013	-4.431	-0.752	-0.013	73.644	12.465	13.629
1279	99	11	22	Sn	O	25.022	4.862	0.753	0.013	-4.431	-0.752	-0.013	73.644	12.465	13.629
1280	99	11	22	Sn	Ti	34.978	4.862	-0.753	0.013	-4.431	0.752	-0.013	73.644	12.465	13.629
1281	99	11	22	Sn	Ti	7.203	0.587	3.266	4.24	-0.58	-3.01	-3.909	73.59	13.633	12.462
1282	99	11	22	Sn	O	7.203	0.587	3.266	4.24	-0.58	-3.01	-3.909	73.59	13.633	12.462
1283	99	12	21	Sn	Ti	57.103	-1.882	-5.056	-0.104	1.956	5.067	0.105	70.271	13.298	13.277
1284	99	12	21	Sn	O	57.103	-1.882	-5.056	-0.104	1.956	5.067	0.105	70.271	13.298	13.277
1285	99	11	22	Sn	Ti	22.797	0.587	-3.266	4.24	-0.58	3.01	-3.909	73.59	13.633	12.462
1286	99	11	22	Sn	Ti	55.022	4.862	0.753	0.013	-4.431	-0.752	-0.013	73.644	12.465	13.629
1287	99	11	22	Sn	O	55.022	4.862	0.753	0.013	-4.431	-0.752	-0.013	73.644	12.465	13.629
1288	99	11	22	Sn	Ti	4.978	4.862	-0.753	0.013	-4.431	0.752	-0.013	73.644	12.465	13.629
1289	99	11	22	Sn	O	4.978	4.862	-0.753	0.013	-4.431	0.752	-0.013	73.644	12.465	13.629
1290	108	13	23	S	Ti	2.897	-1.882	-3.873	0.444	1.956	3.839	-0.44	87.792	13.298	13.623
1291	108	13	23	S	Ti	49.723	-1.882	3.873	0.444	1.956	-3.839	-0.44	87.792	13.298	13.623
1292	108	13	23	S	O	2.897	-1.882	-3.873	0.444	1.956	3.839	-0.44	87.792	13.298	13.623
1293	108	13	23	S	O	49.723	-1.882	3.873	0.444	1.956	-3.839	-0.44	87.792	13.298	13.623
1294	108	13	23	S	O	2.897	-1.882	-3.873	0.444	1.956	3.839	-0.44	87.792	13.298	13.623
1295	108	13	23	S	O	49.723	-1.882	3.873	0.444	1.956	-3.839	-0.44	87.792	13.298	13.623
1296	108	13	23	S	Ti	15.417	0.587	-3.592	-2.018	-0.58	3.744	2.103	87.9	13.633	13.288
1297	108	13	23	S	Ti	37.203	0.587	3.592	-2.018	-0.58	-3.744	2.103	87.9	13.633	13.288
1298	108	13	23	S	O	15.417	0.587	-3.592	-2.018	-0.58	3.744	2.103	87.9	13.633	13.288
1299	108	13	23	S	O	37.203	0.587	3.592	-2.018	-0.58	-3.744	2.103	87.9	13.633	13.288
1300	108	13	23	S	O	15.417	0.587	-3.592	-2.018	-0.58	3.744	2.103	87.9	13.633	13.288

1301	108	13	23	S	O	37.203	0.587	3.592	-2.018	-0.58	-3.744	2.103	87.9	13.633	13.288
1302	108	13	23	S	Ti	27.103	-1.882	3.873	0.444	1.956	-3.839	-0.44	87.792	13.298	13.623
1303	108	13	23	S	Ti	40.277	-1.882	-3.873	0.444	1.956	3.839	-0.44	87.792	13.298	13.623
1304	108	13	23	S	O	27.103	-1.882	3.873	0.444	1.956	-3.839	-0.44	87.792	13.298	13.623
1305	108	13	23	S	O	40.277	-1.882	-3.873	0.444	1.956	3.839	-0.44	87.792	13.298	13.623
1306	108	13	23	S	O	27.103	-1.882	3.873	0.444	1.956	-3.839	-0.44	87.792	13.298	13.623
1307	108	13	23	S	O	40.277	-1.882	-3.873	0.444	1.956	3.839	-0.44	87.792	13.298	13.623
1308	108	13	23	S	Ti	14.583	0.587	3.592	-2.018	-0.58	-3.744	2.103	87.9	13.633	13.288
1309	108	13	23	S	Ti	52.797	0.587	-3.592	-2.018	-0.58	3.744	2.103	87.9	13.633	13.288
1310	108	13	23	S	O	52.797	0.587	-3.592	-2.018	-0.58	3.744	2.103	87.9	13.633	13.288
1311	108	13	23	S	O	14.583	0.587	3.592	-2.018	-0.58	-3.744	2.103	87.9	13.633	13.288
1312	108	13	23	S	O	52.797	0.587	-3.592	-2.018	-0.58	3.744	2.103	87.9	13.633	13.288
1313	108	13	23	S	Ti	32.897	-1.882	-3.873	0.444	1.956	3.839	-0.44	87.792	13.298	13.623
1314	108	13	23	S	Ti	19.723	-1.882	3.873	0.444	1.956	-3.839	-0.44	87.792	13.298	13.623
1315	108	13	23	S	O	32.897	-1.882	-3.873	0.444	1.956	3.839	-0.44	87.792	13.298	13.623
1316	108	13	23	S	O	19.723	-1.882	3.873	0.444	1.956	-3.839	-0.44	87.792	13.298	13.623
1317	108	13	23	S	Ti	45.417	0.587	-3.592	-2.018	-0.58	3.744	2.103	87.9	13.633	13.288
1318	108	13	23	S	Ti	7.203	0.587	3.592	-2.018	-0.58	-3.744	2.103	87.9	13.633	13.288
1319	108	13	23	S	O	45.417	0.587	-3.592	-2.018	-0.58	3.744	2.103	87.9	13.633	13.288
1320	108	13	23	S	O	7.203	0.587	3.592	-2.018	-0.58	-3.744	2.103	87.9	13.633	13.288
1321	108	13	23	S	Ti	10.277	-1.882	-3.873	0.444	1.956	3.839	-0.44	87.792	13.298	13.623
1322	108	13	23	S	Ti	57.103	-1.882	3.873	0.444	1.956	-3.839	-0.44	87.792	13.298	13.623
1323	108	13	23	S	O	57.103	-1.882	3.873	0.444	1.956	-3.839	-0.44	87.792	13.298	13.623
1324	108	13	23	S	Ti	22.797	0.587	-3.592	-2.018	-0.58	3.744	2.103	87.9	13.633	13.288
1325	108	13	23	S	Ti	44.583	0.587	3.592	-2.018	-0.58	-3.744	2.103	87.9	13.633	13.288
1326	108	13	23	S	O	44.583	0.587	3.592	-2.018	-0.58	-3.744	2.103	87.9	13.633	13.288
1327	108	13	23	S	Ti	2.897	-1.882	-3.873	0.444	1.956	3.839	-0.44	87.792	13.298	13.623
1328	108	13	23	S	Ti	49.723	-1.882	3.873	0.444	1.956	-3.839	-0.44	87.792	13.298	13.623
1329	108	13	23	S	O	2.897	-1.882	-3.873	0.444	1.956	3.839	-0.44	87.792	13.298	13.623
1330	108	13	23	S	O	49.723	-1.882	3.873	0.444	1.956	-3.839	-0.44	87.792	13.298	13.623
1331	108	13	23	S	O	2.897	-1.882	-3.873	0.444	1.956	3.839	-0.44	87.792	13.298	13.623
1332	108	13	23	S	O	49.723	-1.882	3.873	0.444	1.956	-3.839	-0.44	87.792	13.298	13.623
1333	108	13	23	S	Ti	15.417	0.587	-3.592	-2.018	-0.58	3.744	2.103	87.9	13.633	13.288
1334	108	13	23	S	Ti	37.203	0.587	3.592	-2.018	-0.58	-3.744	2.103	87.9	13.633	13.288
1335	108	13	23	S	O	15.417	0.587	-3.592	-2.018	-0.58	3.744	2.103	87.9	13.633	13.288
1336	108	13	23	S	O	37.203	0.587	3.592	-2.018	-0.58	-3.744	2.103	87.9	13.633	13.288
1337	108	13	23	S	O	15.417	0.587	-3.592	-2.018	-0.58	3.744	2.103	87.9	13.633	13.288
1338	108	13	23	S	O	37.203	0.587	3.592	-2.018	-0.58	-3.744	2.103	87.9	13.633	13.288
1339	108	13	23	S	Ti	27.103	-1.882	3.873	0.444	1.956	-3.839	-0.44	87.792	13.298	13.623
1340	108	13	23	S	Ti	40.277	-1.882	-3.873	0.444	1.956	3.839	-0.44	87.792	13.298	13.623
1341	108	13	23	S	O	27.103	-1.882	3.873	0.444	1.956	-3.839	-0.44	87.792	13.298	13.623
1342	108	13	23	S	O	40.277	-1.882	-3.873	0.444	1.956	3.839	-0.44	87.792	13.298	13.623

1343	108	13	23	S	O	27.103	-1.882	3.873	0.444	1.956	-3.839	-0.44	87.792	13.298	13.623
1344	108	13	23	S	O	40.277	-1.882	-3.873	0.444	1.956	3.839	-0.44	87.792	13.298	13.623
1345	108	13	23	S	Ti	14.583	0.587	3.592	-2.018	-0.58	-3.744	2.103	87.9	13.633	13.288
1346	108	13	23	S	Ti	52.797	0.587	-3.592	-2.018	-0.58	3.744	2.103	87.9	13.633	13.288
1347	108	13	23	S	O	14.583	0.587	3.592	-2.018	-0.58	-3.744	2.103	87.9	13.633	13.288
1348	108	13	23	S	O	52.797	0.587	-3.592	-2.018	-0.58	3.744	2.103	87.9	13.633	13.288
1349	108	13	23	S	O	14.583	0.587	3.592	-2.018	-0.58	-3.744	2.103	87.9	13.633	13.288
1350	108	13	23	S	O	52.797	0.587	-3.592	-2.018	-0.58	3.744	2.103	87.9	13.633	13.288
1351	108	13	23	S	Ti	32.897	-1.882	-3.873	0.444	1.956	3.839	-0.44	87.792	13.298	13.623
1352	108	13	23	S	Ti	19.723	-1.882	3.873	0.444	1.956	-3.839	-0.44	87.792	13.298	13.623
1353	108	13	23	S	O	32.897	-1.882	-3.873	0.444	1.956	3.839	-0.44	87.792	13.298	13.623
1354	108	13	23	S	O	19.723	-1.882	3.873	0.444	1.956	-3.839	-0.44	87.792	13.298	13.623
1355	108	13	23	S	Ti	45.417	0.587	-3.592	-2.018	-0.58	3.744	2.103	87.9	13.633	13.288
1356	108	13	23	S	Ti	7.203	0.587	3.592	-2.018	-0.58	-3.744	2.103	87.9	13.633	13.288
1357	108	13	23	S	O	45.417	0.587	-3.592	-2.018	-0.58	3.744	2.103	87.9	13.633	13.288
1358	108	13	23	S	O	7.203	0.587	3.592	-2.018	-0.58	-3.744	2.103	87.9	13.633	13.288
1359	108	13	23	S	Ti	10.277	-1.882	-3.873	0.444	1.956	3.839	-0.44	87.792	13.298	13.623
1360	108	13	23	S	Ti	57.103	-1.882	3.873	0.444	1.956	-3.839	-0.44	87.792	13.298	13.623
1361	108	13	23	S	O	57.103	-1.882	3.873	0.444	1.956	-3.839	-0.44	87.792	13.298	13.623
1362	108	13	23	S	Ti	22.797	0.587	-3.592	-2.018	-0.58	3.744	2.103	87.9	13.633	13.288
1363	108	13	23	S	Ti	44.583	0.587	3.592	-2.018	-0.58	-3.744	2.103	87.9	13.633	13.288
1364	108	13	23	S	O	44.583	0.587	3.592	-2.018	-0.58	-3.744	2.103	87.9	13.633	13.288
1365	108	13	23	Sn	Ti	2.897	-1.882	-3.873	0.444	1.956	3.839	-0.44	87.792	13.298	13.623
1366	108	13	23	Sn	Ti	49.723	-1.882	3.873	0.444	1.956	-3.839	-0.44	87.792	13.298	13.623
1367	108	13	23	Sn	O	2.897	-1.882	-3.873	0.444	1.956	3.839	-0.44	87.792	13.298	13.623
1368	108	13	23	Sn	O	49.723	-1.882	3.873	0.444	1.956	-3.839	-0.44	87.792	13.298	13.623
1369	108	13	23	Sn	O	2.897	-1.882	-3.873	0.444	1.956	3.839	-0.44	87.792	13.298	13.623
1370	108	13	23	Sn	O	49.723	-1.882	3.873	0.444	1.956	-3.839	-0.44	87.792	13.298	13.623
1371	108	13	23	Sn	Ti	15.417	0.587	-3.592	-2.018	-0.58	3.744	2.103	87.9	13.633	13.288
1372	108	13	23	Sn	Ti	37.203	0.587	3.592	-2.018	-0.58	-3.744	2.103	87.9	13.633	13.288
1373	108	13	23	Sn	O	15.417	0.587	-3.592	-2.018	-0.58	3.744	2.103	87.9	13.633	13.288
1374	108	13	23	Sn	O	37.203	0.587	3.592	-2.018	-0.58	-3.744	2.103	87.9	13.633	13.288
1375	108	13	23	Sn	O	15.417	0.587	-3.592	-2.018	-0.58	3.744	2.103	87.9	13.633	13.288
1376	108	13	23	Sn	O	37.203	0.587	3.592	-2.018	-0.58	-3.744	2.103	87.9	13.633	13.288
1377	108	13	23	Sn	Ti	27.103	-1.882	3.873	0.444	1.956	-3.839	-0.44	87.792	13.298	13.623
1378	108	13	23	Sn	Ti	40.277	-1.882	-3.873	0.444	1.956	3.839	-0.44	87.792	13.298	13.623
1379	108	13	23	Sn	O	27.103	-1.882	3.873	0.444	1.956	-3.839	-0.44	87.792	13.298	13.623
1380	108	13	23	Sn	O	40.277	-1.882	-3.873	0.444	1.956	3.839	-0.44	87.792	13.298	13.623
1381	108	13	23	Sn	O	27.103	-1.882	3.873	0.444	1.956	-3.839	-0.44	87.792	13.298	13.623
1382	108	13	23	Sn	O	40.277	-1.882	-3.873	0.444	1.956	3.839	-0.44	87.792	13.298	13.623
1383	108	13	23	Sn	Ti	14.583	0.587	3.592	-2.018	-0.58	-3.744	2.103	87.9	13.633	13.288
1384	108	13	23	Sn	Ti	52.797	0.587	-3.592	-2.018	-0.58	3.744	2.103	87.9	13.633	13.288

1385	108	13	23	Sn	O	14.583	0.587	3.592	-2.018	-0.58	-3.744	2.103	87.9	13.633	13.288
1386	108	13	23	Sn	O	52.797	0.587	-3.592	-2.018	-0.58	3.744	2.103	87.9	13.633	13.288
1387	108	13	23	Sn	O	14.583	0.587	3.592	-2.018	-0.58	-3.744	2.103	87.9	13.633	13.288
1388	108	13	23	Sn	O	52.797	0.587	-3.592	-2.018	-0.58	3.744	2.103	87.9	13.633	13.288
1389	108	13	23	Sn	Ti	32.897	-1.882	-3.873	0.444	1.956	3.839	-0.44	87.792	13.298	13.623
1390	108	13	23	Sn	Ti	19.723	-1.882	3.873	0.444	1.956	-3.839	-0.44	87.792	13.298	13.623
1391	108	13	23	Sn	O	32.897	-1.882	-3.873	0.444	1.956	3.839	-0.44	87.792	13.298	13.623
1392	108	13	23	Sn	O	19.723	-1.882	3.873	0.444	1.956	-3.839	-0.44	87.792	13.298	13.623
1393	108	13	23	Sn	Ti	45.417	0.587	-3.592	-2.018	-0.58	3.744	2.103	87.9	13.633	13.288
1394	108	13	23	Sn	Ti	7.203	0.587	3.592	-2.018	-0.58	-3.744	2.103	87.9	13.633	13.288
1395	108	13	23	Sn	O	45.417	0.587	-3.592	-2.018	-0.58	3.744	2.103	87.9	13.633	13.288
1396	108	13	23	Sn	O	7.203	0.587	3.592	-2.018	-0.58	-3.744	2.103	87.9	13.633	13.288
1397	108	13	23	Sn	Ti	10.277	-1.882	-3.873	0.444	1.956	3.839	-0.44	87.792	13.298	13.623
1398	108	13	23	Sn	Ti	57.103	-1.882	3.873	0.444	1.956	-3.839	-0.44	87.792	13.298	13.623
1399	108	13	23	Sn	O	57.103	-1.882	3.873	0.444	1.956	-3.839	-0.44	87.792	13.298	13.623
1400	108	13	23	Sn	Ti	22.797	0.587	-3.592	-2.018	-0.58	3.744	2.103	87.9	13.633	13.288
1401	108	13	23	Sn	Ti	44.583	0.587	3.592	-2.018	-0.58	-3.744	2.103	87.9	13.633	13.288
1402	108	13	23	Sn	O	44.583	0.587	3.592	-2.018	-0.58	-3.744	2.103	87.9	13.633	13.288
1403	111	12	25	S	Ti	26.31	5.194	4.821	1.783	-4.706	-4.655	-1.722	69.03	14.257	13.637
1404	111	12	25	S	O	26.31	5.194	4.821	1.783	-4.706	-4.655	-1.722	69.03	14.257	13.637
1405	111	12	25	S	O	26.31	5.194	4.821	1.783	-4.706	-4.655	-1.722	69.03	14.257	13.637
1406	111	12	25	S	Ti	3.69	5.194	-4.821	1.783	-4.706	4.655	-1.722	69.03	14.257	13.637
1407	111	12	25	S	O	3.69	5.194	-4.821	1.783	-4.706	4.655	-1.722	69.03	14.257	13.637
1408	111	12	25	S	O	3.69	5.194	-4.821	1.783	-4.706	4.655	-1.722	69.03	14.257	13.637
1409	111	12	25	S	Ti	56.31	5.194	4.821	1.783	-4.706	-4.655	-1.722	69.03	14.257	13.637
1410	111	12	25	S	O	56.31	5.194	4.821	1.783	-4.706	-4.655	-1.722	69.03	14.257	13.637
1411	111	12	25	S	Ti	33.69	5.194	-4.821	1.783	-4.706	4.655	-1.722	69.03	14.257	13.637
1412	111	12	25	S	O	33.69	5.194	-4.821	1.783	-4.706	4.655	-1.722	69.03	14.257	13.637
1413	111	12	25	S	Ti	26.31	5.194	4.821	1.783	-4.706	-4.655	-1.722	69.03	14.257	13.637
1414	111	12	25	S	O	26.31	5.194	4.821	1.783	-4.706	-4.655	-1.722	69.03	14.257	13.637
1415	111	12	25	S	O	26.31	5.194	4.821	1.783	-4.706	-4.655	-1.722	69.03	14.257	13.637
1416	111	12	25	S	Ti	3.69	5.194	-4.821	1.783	-4.706	4.655	-1.722	69.03	14.257	13.637
1417	111	12	25	S	O	3.69	5.194	-4.821	1.783	-4.706	4.655	-1.722	69.03	14.257	13.637
1418	111	12	25	S	O	3.69	5.194	-4.821	1.783	-4.706	4.655	-1.722	69.03	14.257	13.637
1419	111	12	25	S	Ti	56.31	5.194	4.821	1.783	-4.706	-4.655	-1.722	69.03	14.257	13.637
1420	111	12	25	S	O	56.31	5.194	4.821	1.783	-4.706	-4.655	-1.722	69.03	14.257	13.637
1421	111	12	25	S	Ti	33.69	5.194	-4.821	1.783	-4.706	4.655	-1.722	69.03	14.257	13.637
1422	111	12	25	S	O	33.69	5.194	-4.821	1.783	-4.706	4.655	-1.722	69.03	14.257	13.637
1423	111	12	25	Sn	Ti	26.31	5.194	4.821	1.783	-4.706	-4.655	-1.722	69.03	14.257	13.637
1424	111	12	25	Sn	O	26.31	5.194	4.821	1.783	-4.706	-4.655	-1.722	69.03	14.257	13.637
1425	111	12	25	Sn	O	26.31	5.194	4.821	1.783	-4.706	-4.655	-1.722	69.03	14.257	13.637
1426	111	12	25	Sn	Ti	3.69	5.194	-4.821	1.783	-4.706	4.655	-1.722	69.03	14.257	13.637

1427	111	12	25	Sn	O	3.69	5.194	-4.821	1.783	-4.706	4.655	-1.722	69.03	14.257	13.637
1428	111	12	25	Sn	O	3.69	5.194	-4.821	1.783	-4.706	4.655	-1.722	69.03	14.257	13.637
1429	111	12	25	Sn	Ti	56.31	5.194	4.821	1.783	-4.706	-4.655	-1.722	69.03	14.257	13.637
1430	111	12	25	Sn	O	56.31	5.194	4.821	1.783	-4.706	-4.655	-1.722	69.03	14.257	13.637
1431	111	12	25	Sn	Ti	33.69	5.194	-4.821	1.783	-4.706	4.655	-1.722	69.03	14.257	13.637
1432	111	12	25	Sn	O	33.69	5.194	-4.821	1.783	-4.706	4.655	-1.722	69.03	14.257	13.637
